# Prdm1 Regulates Thymic Epithelial Function To Prevent Autoimmunity

**DOI:** 10.4049/jimmunol.1600941

**Published:** 2017-07-12

**Authors:** Natalie A. Roberts, Brian D. Adams, Nicholas I. McCarthy, Reuben M. Tooze, Sonia M. Parnell, Graham Anderson, Susan M. Kaech, Valerie Horsley

**Affiliations:** * Department of Molecular, Cellular and Developmental Biology, Yale University, New Haven, CT 06520;; † The Francis Crick Institute, London NW1 1AT, United Kingdom;; ‡ The RNA Institute, University at Albany, State University of New York, Albany, NY 12222;; § Investigative Medicine Program, Yale University School of Medicine, New Haven, CT 06520;; ¶ School of Immunity and Infection, Medical Research Council Centre for Immune Regulation, University of Birmingham, Birmingham B15 2TT, United Kingdom;; ‖ Section of Experimental Haematology, Leeds Institute of Molecular Medicine, University of Leeds, Leeds LS2 9JT, United Kingdom;; # Department of Immunobiology, Yale University, New Haven, CT 06520; and; ** Department of Dermatology, Yale University, New Haven, CT 06520

## Abstract

Autoimmunity is largely prevented by medullary thymic epithelial cells (TECs) through their expression and presentation of tissue-specific Ags to developing thymocytes, resulting in deletion of self-reactive T cells and supporting regulatory T cell development. The transcription factor *Prdm1* has been implicated in autoimmune diseases in humans through genome-wide association studies and in mice using cell type–specific deletion of *Prdm1* in T and dendritic cells. In this article, we demonstrate that Prdm1 functions in TECs to prevent autoimmunity in mice. Prdm1 is expressed by a subset of mouse TECs, and conditional deletion of *Prdm1* in either *Keratin 14–* or *Foxn1-*expressing cells in mice resulted in multisymptom autoimmune pathology. Notably, the development of Foxp3^+^ regulatory T cells occurs normally in the absence of Blimp1. Importantly, nude mice developed anti-nuclear Abs when transplanted with *Prdm1* null TECs, but not wild-type TECs, indicating that *Prdm1* functions in TECs to regulate autoantibody production. We show that *Prdm1* acts independently of Aire, a crucial transcription factor implicated in medullary TEC function. Collectively, our data highlight a previously unrecognized role for Prdm1 in regulating thymic epithelial function.

## Introduction

The thymus is essential for the prevention of autoimmunity through the induction of T cell tolerance and the generation of FoxP3^+^ regulatory T cells (Tregs). Thymocytes expressing a functional TCR are positively selected by interacting with cortical thymic epithelial cells (TECs) (cTECs), after which they migrate and interact with tissue-specific Ags (TSAs) presented on medullary TECs (mTECs) and dendritic cells. Recognition of TSAs results in negative selection, whereby autoreactive T cells are eliminated (1–4). Although little is known about the precise mechanisms that control TSA expression in TECs, the transcription factor autoimmune regulator (Aire) is central to TSA expression (5). Aire can bind to the repressive MBD1-ATF7ip complex, which methylates CpG dinucleotides to target specific TSA genomic loci. Aire also recruits proteins that promote transcriptional elongation and pre-mRNA processing (6). However, additional molecular players that alter the epigenetic landscape to enable the full function of Aire have yet to be fully elucidated. Furthermore, whereas Aire ensures that self-antigens are expressed within the thymus in mTECs (5, 7), several Aire-independent self-antigens are expressed within the thymus (8, 9), and each self-antigen is expressed by a low percentage of mTECs, (1) suggesting that multiple mechanisms exist to regulate mTEC function.

Prdm1 (Blimp1) is a transcription factor that controls gene expression and chromatin structure in several embryonic and adult tissues. Prdm1 acts as a transcriptional repressor by binding to DNA through its proline-rich zinc finger domain and recruiting transcriptional cofactors such as hGroucho, histone deacetylases (HDACs), and histone methyltransferases (10–14). In differentiating plasma cells, Prdm1 represses genes involved in B cell maturation and proliferation (15, 16), mediating terminal differentiation (15, 17, 18). Prdm1 also controls gene expression patterns in many lymphocytes and myeloid cells, including dendritic cells (19), macrophages (20), T cells (21, 22), and NK cells (23).

Beyond the immune system, Prdm1 has numerous roles in regulating epithelial development. In the intestinal epithelium, Prdm1 controls multiple aspects of the neonatal-to-adult transition (24, 25), namely terminal differentiation of the skin epidermis (26) and sebocyte progenitor cell function (27).

Given the similarities between skin and the thymic epithelium (28–30) and the models used to describe the roles of skin inflammation with age in *Prdm1* conditional knockout (KO) (cKO) mice (31, 32), we sought to determine whether Prdm1 influences thymic epithelial function. In this study, we identified and mapped the expression of Prdm1 to the thymus medulla. In addition, we have shown that *Prdm1* is expressed in TECs and that mice lacking *Prdm1* in either *keratin-14* (*K14*)- or *FoxN1-*expressing epithelial cells generate self-reactive Abs and develop several autoimmune phenotypes. These phenotypes in mice lacking *Prdm1* in epithelium are not due to defects in the development of CD4, CD8, or Foxp3^+^ Tregs. In fact, by performing thymus transplantation experiments into nude mice, we demonstrated that *Prdm1*-deficient mTECs are sufficient to induce autoimmunity. Together, our findings implicate Prdm1 in the regulation of autoimmunity in TECs and are consistent with the identification of polymorphisms in *Prdm1* associated with autoimmune diseases, such as systemic lupus erythematosus (SLE) (33, 34).

## Materials and Methods

### Animal use

All animals were housed and handled according to the institutional guidelines of Yale University. *Keratin14-*Cre (35), *Prdm1-floxed* (27), *Prdm1-eGFP* (36), *Prdm1*-YFP (37), *mTmG* (38), *Aire* KO (39), and *FoxN1-*Cre (40) mice were previously described. MRL/*Fas^lpr^* (MRL/MpJ-*Fas^lpr^*/2J) animals were purchased from The Jackson Laboratory (41).

For *Prdm1^fl/fl^;K14*Cre, *Prdm1^fl/fl^;FoxN1*Cre, *Prdm1eGFP*, and *Prdm1YFP*, mice were maintained in the inbred C57BL/6 background, and age-matched *Prdm1fl/^+^;Cre^+^* littermates were used as controls. For the *K14*Cre*;mTmG* and *FoxN1Cre;mTmG* lineage tracing experiments, mice were in a mixed C57BL/6,129X1/SvJ background. Control mice for all experiments were *Prdm1^fl/+^;K14*Cre*^+^* age-matched littermates because these mice did not develop the initial ventral alopecia and dermatitis.

### Cell isolation

Thymocyte, splenocyte, and lymph node suspensions were obtained from adult organs by mechanical dissociation. Stromal cells from adult thymi were isolated, as previously described (42). In brief, thymus lobes were cut into 1-mm^3^ pieces, washed, and digested with R-5 medium (l-glutamine–supplemented RPMI 1640 [Sigma-Aldrich], 10 mM HEPES [Life Technologies, Invitrogen], 5% FCS) containing 0.32 Wunsch U/ml Liberase/thermolysin (Roche) and 50 Kunitz U/ml^−1^ DNase I (Sigma-Aldrich) at 37°C for 40 min using a gyratory water bath shaker (New Brunswick Scientific). Enzymatic treatment was repeated for an additional 20 min, followed by incubation with 5 mM EDTA on ice for 10 min. Remaining tissue fragments were mechanically dispersed by careful pipetting. Cell suspensions from each digestion were pooled and washed in ice-cold PBS containing 2% FCS and 2 mM EDTA to prevent aggregate formation. Stromal cells were enriched by MACS immunomagnetic depletion of CD45^+^ cells (Miltenyi Biotec), according to the manufacturer’s protocol. Cell surface Ab staining was performed in 2% FCS and 2 mM EDTA to prevent aggregate formation.

### Immunofluorescence of tissues

Wild-type (WT) tissues were embedded in OCT compound (Tissue-Tek), frozen using dry ice, and 6-μm sections were cut and fixed in 100% acetone (4°C, 20 min). Thymus samples from *Prdm1*-GFP mice were fixed in 3.7% formaldehyde in PBS for 1 h at room temperature (RT), washed twice in PBS, and placed consecutively in 10% sucrose overnight at 4°C, 20% sucrose overnight at 4°C, and 30% sucrose overnight at 4°C, before embedding in OCT compound, and sectioned. Tissue sections were incubated with primary Abs for 40 min and washed in PBS before incubating in secondary (and/or tertiary) Abs for 30 min. Slides were washed in PBS three times before staining with DAPI (Pierce) and mounting with Prolong Gold anti-fade reagent (Life Technologies, Grand Island, NY). Images were acquired using Zeiss Imager M.1 fluorescent microscope (Zeiss, Oberkochen, Germany) with AxioCam MR3 camera and AxioVision (release 4.8.2) software.

Abs used were as follows: primary Abs, IgG (poly4053) DyLight 488, IgM (RMM-1) biotinylated (BioLegend), Keratin 5 (gift from Julie Segre), Keratin 8 (Progen) biotinylated, goat anti-Aire (D17) (Santa Cruz Biotechnology), rabbit anti-Involucrin (gift from Julie Segre); secondary Abs, donkey anti-chicken 488 (Invitrogen), chicken anti-GFP 488 (Abcam), anti-mouse IgG FITC (Southern Biotechnology), anti-mouse IgG biotinylated (Vector Laboratories), anti-goat Rhodamine Red-X (Jackson ImmunoResearch), and goat anti-rabbit Alexa Fluor 647 (Life Technologies). Biotinylated Abs were picked up with Alexa Fluor 555–conjugated streptavidin (Invitrogen).

### Prdm1 immunohistochemistry

Normal human thymus (use of human tissue approved by National Research Ethics Service/Research Ethics Committee reference: 07/Q1206/47) stained rabbit anti-PRDM1 polyclonal Ab R23 (14, 27, 43) with heat-mediated Ag retrieval was used, with standard streptavidin Avidin-Biotin Complex immunocytochemistry and 3,3′-diaminobenzidine as the chromogen. We obtained 40× magnification using an Olympus BX50 microscope (Olympus, Tokyo, Japan), a Plan Apo 40×/0.95 objective lens (Tokyo, Japan), a Leica DFC-320 digital camera (Leica, Solms, Germany), and associated Leica IM50 software.

### Anti-nuclear Ab staining

A 1:40 dilution of the serum samples isolated from *Prdm1fl/fl;K14Cre* and *Prdm1fl/^+^;K14Cre* mice or from nude hosts grafted with embryonic thymus isolated from *Prdm1fl/fl;K14Cre* and *Prdm1fl/^+^;K14Cre* mice were incubated for 30 min at RT on 12 spot anti-nuclear Ab (ANA) diagnostic HEp2 Slides (Bio-Rad). Serum isolated from MRL/*Fas^lpr^* mice was used as an ANA-positive control (41). FITC anti-mouse secondary Ab (Southern Biotechnology), diluted 1:50 in 0.1% BSA/PBS, was used for detection. Coverslips were mounted using Fluoromount.

Images were acquired using a Zeiss LSM 510 confocal microscope (Zeiss) equipped with a 40× Plan-Apochromat objective. Image acquisition and processing were performed using ZEN software.

### Flow cytometry and FACS

For flow cytometry single-cell thymocyte, splenocyte, and lymph node, suspensions were stained with the following Abs: anti-CD80 (16-10A1) FITC, CD80 (B7-1) PerCP-eFluor710, anti-CD45 (30F11) PE, TCR β-chain (TCRβ; H57-597) biotinylated, CD8 (53-6.7) allophycocyanin, CD44 (IM7) Alexa Fluor 450, CD62L (MEL-14) allophycocyanin 780, FoxP3 (FJK-16s) Alexa Fluor 647, CD4 (RM4-5) allophycocyanin Cy7, CD25 (PC61.5) Alexa Fluor 488, anti–BP-1/Ly51 (6C3) biotinylated, MHC class II (MHCII; I-A/I-E) clone M5/114.15.2 eFluor450 (all eBioscience), and CD4 (GK1.5) PerCP (BioLegend). Anti-EPCAM1 (G8.8) (BD Pharmingen) was directly conjugated using the Alexa Fluor 647 mAb Labeling Kit (Invitrogen) according to the manufacturer’s instructions. Biotinylated Abs were picked up with either PE-Cy7–conjugated streptavidin or allophycocyanin-conjugated streptavidin (BD Biosciences). For intracellular staining of Foxp3, cells were fixed and permeabilized using the Foxp3 fixation and permeabilization concentrate and diluent set (eBioscience) according to the manufacturer’s protocol and stained with anti-Foxp3 647 (clone FJK-16s; eBioscience). For RNA isolation, the populations were sorted directly into TRIzol LS (Life Technologies). Given the low number of mTECs, mTECs were sorted and pooled from up to 10 mice to analyze mRNA expression in mTEC populations. Cells were analyzed or sorted using a FACSAria III equipped with FACSDiva software (BD Biosciences).

### Fetal thymus organ culture and thymus grafting

Embryonic day (E) 15 fetal thymuses were isolated from *Prdm1^fl/fl^;K14Cre^+^* and *Prdm1^fl/+^;K14Cre^+^* (control) embryos. Fetal thymic organ cultures were prepared as described previously (44), cultured in the presence of 1.35 mM 2-deoxyguanosine monohydrate (Sigma) for 7 d, and used as thymus tissue for transplantation under the kidney capsule of adult (6–8 wk) male *Nude* recipients (5).

### ELISA

Ab isotypes were measured by ELISA using serum isolated from *Prdm1^fl/fl^;K14Cre^+^* and *Prdm1^fl/+^*;*K14Cre^+^* mice or from nude hosts grafted with embryonic (E15) thymus isolated from *Prdm1^fl/fl^;K14Cre^+^* and *Prdm1^fl/+^;K14Cre^+^* mice. Ninety-six-well Polysorp microtiter plates (Nunc) were coated overnight with a 9:1 ratio mixture of anti-κ: anti-λ (3 μg/ml) (Southern Biotechnology) in 0.1 M Na_2_HPO_4_/NaH_2_PO_4_ (pH 9). Subsequently, a 1:200,000 dilution of sera was incubated for 2 h at RT. Alkaline phosphatase–labeled goat anti-mouse IgG1, IgG2a, IgG2b, IgG3, IgE, or IgM secondary Abs were used for detection (Southern Biotechnology). The optical densities were converted to units based on standard curves with Mouse Ig Panel Kit of IgG1, IgG2a, IgG2b, IgG3, IgM, and IgE (Southern Biotechnology) using a four-parameter logistic equation (Softmax Pro 3.1 software; Molecular Devices).

### RNA sequencing data analysis

Bioinformatic analysis and identification of *Prdm1*-related genes were derived from the Brennecke (45) dataset (E-MTAB-3346 and E-MTAB-3624 within ArrayExpress; https://www.ebi.ac.uk/arrayexpress/), which contains single-cell transcriptome analysis of mTECs. From this previously normalized single-cell RNA-sequencing dataset, cells with any detectable *Prdm1* transcript levels were classified as Prdm1^+^ cells. All 36,017 genes from the 105-bp paired-end Illumina HiSEquation 2500 sequencing analysis served as the input for Gene Set Enrichment Analysis (46). Standard Gene Set Enrichment Analysis heat-map test was performed, and the top 50 *Prdm1*-associated features are displayed. Spearman-rank correlations for *Prdm1* were obtained for each gene across all 200 cells, and resultant rho values were plotted as a bar graph. Statistical computations were performed using WinStat 2014 (R. Fitch Software, Bad Krozingen, Germany). The *p* values obtained from this analysis were tabulated and plotted in Plotly. All genes associated with *Prdm1* with an α cutoff of 0.0005 are reported. Additional analysis was performed whereby Aire-dependent and Aire-independent gene sets identified by Sansom et al. (47) (GSE53111 within the National Center for Biotechnology Information Gene Expression Omnibus; http://www.ncbi.nlm.nih.gov/geo/) and Brennecke et al. (45) were analyzed for associations with *Prdm1*. Box and whisker plots were generated to describe the median Spearman-rank rho values for Prdm1 to Aire-dependent, Aire-independent, and all gene sets.

### Statistical analysis

Analyses were performed using GraphPad Prism version for Macintosh (GraphPad Software, La Jolla, CA). Categorical data were analyzed using Fisher exact test, whereas noncategorical data between two groups were analyzed using a Mann–Whitney *U* test and a nonparametric test that does not assume a normal distribution; noncategorical data between multiple groups were compared using one-way ANOVA.

## Results

### Epithelial deletion of *Prdm1* results in autoimmunity

**FIGURE 1.**
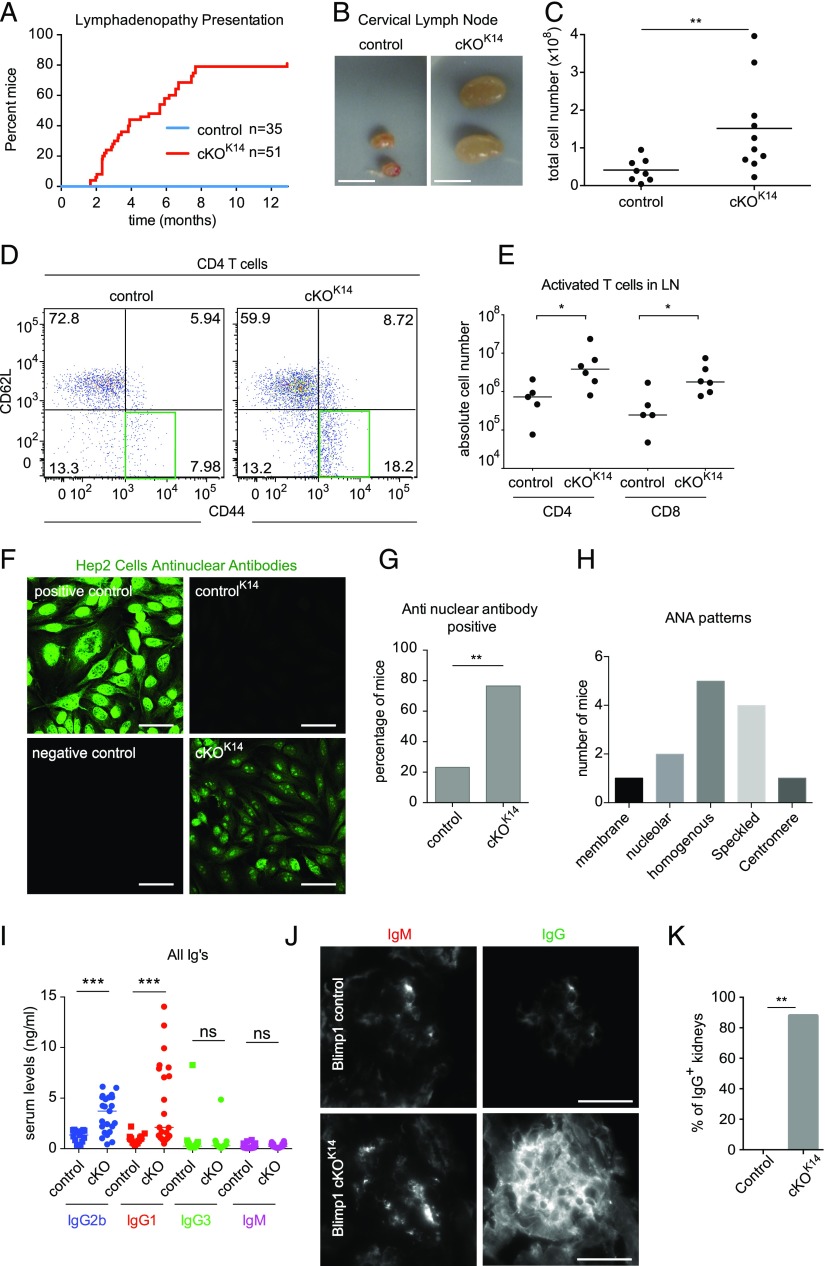
Conditional deletion of Prdm1 in *K14*^+^ epithelium results in spontaneous development of autoimmunity. (**A**) Quantification of cervical lymphadenopathy presentation in control^K14^ (*n* = 35) and *Prdm1 cKO^K14^* (*n* = 51). (**B**) Images of mandibular cervical lymphadenopathy in *Prdm1 cKO^K14^* mice compared with the normal nodes of control mice. Scale bar, 10 mm. (**C**) Total lymphocyte numbers are elevated in *Prdm1 cKO^K14^* and *Prdm1 cKO^FoxN1^* mandibular and accessory mandibular cervical lymph nodes. (**D**) Flow cytometric analysis of CD4 T cells isolated from cervical (mandibular and accessory mandibular) lymph nodes shows an increase in proportion of CD44^+^CD62L^−^ activated CD4 T cells. (**E**) An increase in CD44^+^CD62L^−^ activated CD4 and CD8 T cells from *Prdm1 cKO^K14^* cervical (mandibular and accessory mandibular) lymph nodes compared with control. (**F**) Serum isolated from *Prdm1 cKO^K14^* mice contains ANAs indicated by positive staining of HEp2 cells. Positive control is MRL/*Fas^lpr^* mouse serum; negative control is secondary Ab alone. Scale bar, 50 μm. (**G**) The presence of ANAs is significantly more frequent in *Prdm1 cKO^K14^* than in control. (**H**) Quantification of nuclear patterns identified in sera derived from *Prdm1* cKO^K14^ mice. (**I**) ELISA of serum Ab isotypes. Note IgG2b and IgG1 are significantly increased in *Prdm1* cKO^K14^ serum. (**J**) Representative images of *Prdm1* cKO^K14^ kidney sections exhibit more intense IgG staining than those of control mice. Scale bar, 25 μm. (**K**) The presence of IgG is significantly more frequent in *Prdm1 cKO^K14^* mice than in control. Data are mean ± SEM. *n* ≥ 8 (C), *n* ≥ 5 (D and E), *n* ≥ 13 (F–H), *n* ≥ 15 (I), and *n* ≥ 3 (J) for each genotype. *p < 0.05, **p < 0.01, ***p < 0.001.

Previous studies have established a role for Prdm1 in barrier epithelial tissues, namely the skin and intestine (24, 27, 31, 32), but a role within thymic epithelium remained unexplored. Mice lacking *Prdm1* in squamous epithelial cells were generated by crossing *Prdm1^fl/fl^* mice (27) to mice expressing Cre recombinase driven by the K14 promoter (*Prdm1* cKO*^K14^*) (35) or the forkhead*/winged-helix* box protein N1 (*FoxN1*) promoter (*Prdm1* cKO*^FoxN1^*) (40). Interestingly, in contrast with WT or heterozygous littermates, ∼80% of *Prdm1* cKO*^K14^* mice stochastically developed mandibular and accessory mandibular cervical lymphadenopathy as they aged (≥2 mo) (Fig. 1A–C). Similarly, 100% of *Prdm1* cKO*^FoxN1^* mice analyzed developed lymphadenopathy, albeit at a slightly later time point (≥5 mo) (Supplemental Fig. 1A, 1B). Further investigation of the immunological phenotype in *Prdm1* cKO*^K14^* mice revealed a significant infiltration of activated T lymphocytes in their cervical lymph nodes compared with littermate control mice (Fig. 1D, 1E), with *Prdm1* cKO*^FoxN1^* mice following the same trend (Supplemental Fig. 1C, 1D). Interestingly, the lymphadenopathy observed in these mice was not systemic, as suggested by the absence of significant splenomegaly (Supplemental Fig. 1E–G).

Given the similarities in phenotypes between the deletion of *Prdm1* with K14- and FoxN1-Cre mice, we sought to confirm that Cre recombinase was active in the thymus of *K14*-Cre mice. To this end, we crossed *K14*-Cre mice with the membrane tomato/membrane GFP (*mT/mG)* reporter line, which induces mG expression after Cre-mediated recombination in *K14*-expressing cells (38). Flow cytometric analysis of mTEC and cTEC cell populations (Supplemental Fig. 2A) revealed the expression of *mG* in both mTEC and cTECs (Supplemental Fig. 2B–E), indicating the activity of Cre recombinase within the thymus of *K14*-Cre mice. Although the absolute cell number of GFP^+^ cells tended to be greater in mTECs compared with cTECs, this was not significantly different (Supplemental Fig. 2D), and on average the proportion of GFP^+^ cells in mTECs and cTECs was comparable (Supplemental Fig. 2E).

Because both *K14*-Cre and *FoxN1*-Cre are active in TECs (40), we hypothesized that activation of T cells observed in *Prdm1* cKO*^K14^* and *Prdm1* cKO*^FoxN1^* mice may be because of a breakdown in central tolerance. Consequently, we sought to examine whether *Prdm1* cKO*^K14^* displayed characteristics of autoimmunity, such as the presence of autoantibodies (48, 49). The sera of 80% of *Prdm1* cKO*^K14^* (Fig. 1F, 1G) and 100% of *Prdm1* cKO*^FoxN1^* (Supplemental Fig. 1H) mice positively stained HEp2 cells with a nuclear pattern, whereas only ∼20% of control mice displayed ANA staining (Fig. 1G). The positive staining in *Prdm1* cKO*^K14^* mice exhibited a range of antinuclear patterns (50) with homogenous and speckled patterns being most prevalent (Fig. 1H). Interestingly, this was observed in this particular model only. Analysis of total serum Ig levels by ELISA revealed normal levels of IgM and IgG3 in control versus *Prdm1* cKO*^K14^* mice, whereas IgG1 and IgG2b levels were significantly higher in the blood of *Prdm1* cKO*^K14^* mice (Fig. 1I). Moreover, *Prdm1* cKO*^K14^* mice displayed deposits of IgG in their kidneys (Fig. 1J, 1K). Together, these data demonstrate that the absence of *Prdm1* in epithelial cells leads to the development of spontaneous autoimmune-like disease and T cell activation.

### Prdm1 is expressed in TECs

Because the conditional deletion of *Prdm1* from TECs results in autoimmune-like pathology and TECs are pivotal in establishing central tolerance, we next sought to determine whether *Prdm1* was expressed in TECs. Indeed, published microarray and RNA sequencing experiments demonstrated the presence of *Prdm1* mRNA expression in purified mTECs (GDS1655 and GSE70798) (51, 52).

**FIGURE 2.**
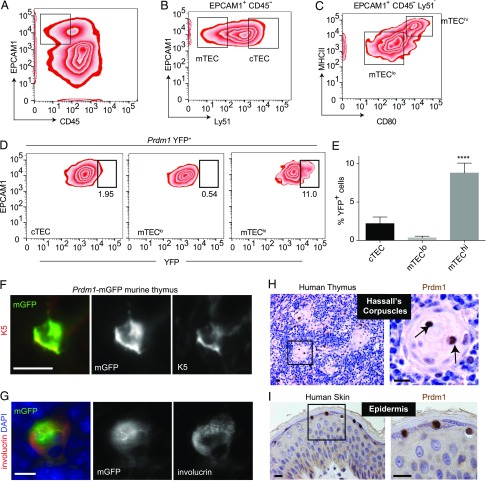
Expression of Prdm1 in the mouse thymus. (**A**) Representative flow cytometry plots indicating the gating strategy for the CD45^−^ EPCAM1^+^ epithelium, (**B**) cTEC (Ly51^+^EPCAM1^+^), mTEC (Ly51^−^EPCAM1^+^), (**C**) mTEC^lo^ (Ly51^−^EPCAM1^+^CD80^−^MHCII^lo^), and mTEC^hi^ (Ly51^−^EPCAM1^+^CD80^+^MHCII^hi^) for *Prdm1*-YFP mice. (**D**) Representative flow cytometry plots indicating the YFP expression within the populations shown in (A). (**E**) Quantification of the percentage of YFP^+^ cells for each of the indicated epithelial cell populations. Data are mean ± SEM. *n* = 5 mice. (**F** and **G**) Sections of the thymus from *Prdm1*-mGFP mice reveal expression of mGFP in K5^+^ and involucrin^+^ cells in the medulla. (**H**) Human thymus sections illustrating Prdm1 expression within mTECs and TECs within Hassles corpuscles. (**I**) Sections from human skin epithelium illustrating positive stain for Prdm1 in the granular layer of the epidermis and negative staining below. Scale bars, 5 μm (G); 10 μm (F); 20 μm (H and I). ****p < 0.0001.

To quantify the percentage of TECs that express *Prdm1*, we used flow cytometry of isolated TECs from a mouse model expressing YFP driven by the *Prdm1* promoter (37). Thymi from *Prdm1-YFP* mice (6–8 wk of age) were enzymatically dissociated, and the resulting single-cell suspensions were stained with Abs against EPCAM1, Ly51, MHCII, and CD80 and analyzed by flow cytometry (42, 53) (Fig. 2A–E). mTECs were identified as CD45^−^, EPCAM1^+^, Ly51^lo^ epithelial cells, whereas cTECs were identified as CD45^−^, EPCAM1^+^, Ly51^hi^ cells (Fig. 2B). The mTEC population was dissected further as mTEC^lo^ and mTEC^hi^ based on MHCII and CD80 expression levels (Fig. 2C). Although control mice displayed no detectable YFP expression (data not shown), a small percentage of cTECs were YFP^+^ (Fig. 2D). Interestingly, YFP was not detected in mTEC^lo^ cells, whereas YFP was detected in a proportion of mTEC^hi^ cells. Quantification of the percentage of YFP^+^ cells in multiple mice revealed a significantly higher percentage of mTEC^hi^ cells (∼9%) were YFP^+^, compared with ∼2% of YFP^+^ cTECs (Fig. 2E).

To further determine the expression of *Prdm1* within TECs, we analyzed thymi from a reporter mouse line that expressed membrane-localized GFP (mGFP) driven by *Prdm1* promoter (36). Immunofluorescence staining of adult thymi from the *Prdm1*-mGFP reporter line with Abs against K5, or involucrin, revealed expression of GFP in a subset of TECs (Fig. 2F, 2G); both K5^+^ and involucrin^+^ cells expressing mGFP were identified (54).

To explore whether Prdm1 was expressed in human TECs, we immunostained sections of human thymus with Abs against PRDM1. PRDM1^+^ nuclei were identified in human Hassall’s corpuscles (Fig. 2H, arrows) (55) with a comparable staining pattern to that seen within human epidermis used as the staining control (Fig. 2I) (27). Together, these data are consistent with the expression of Prdm1 in K5^+^ cells in mouse thymus, within Hassall’s corpuscles in mouse and human thymus, and suggest that Prdm1 is expressed in mature mTECs.

### Prdm1 does not regulate TEC or T cell development

**FIGURE 3.**
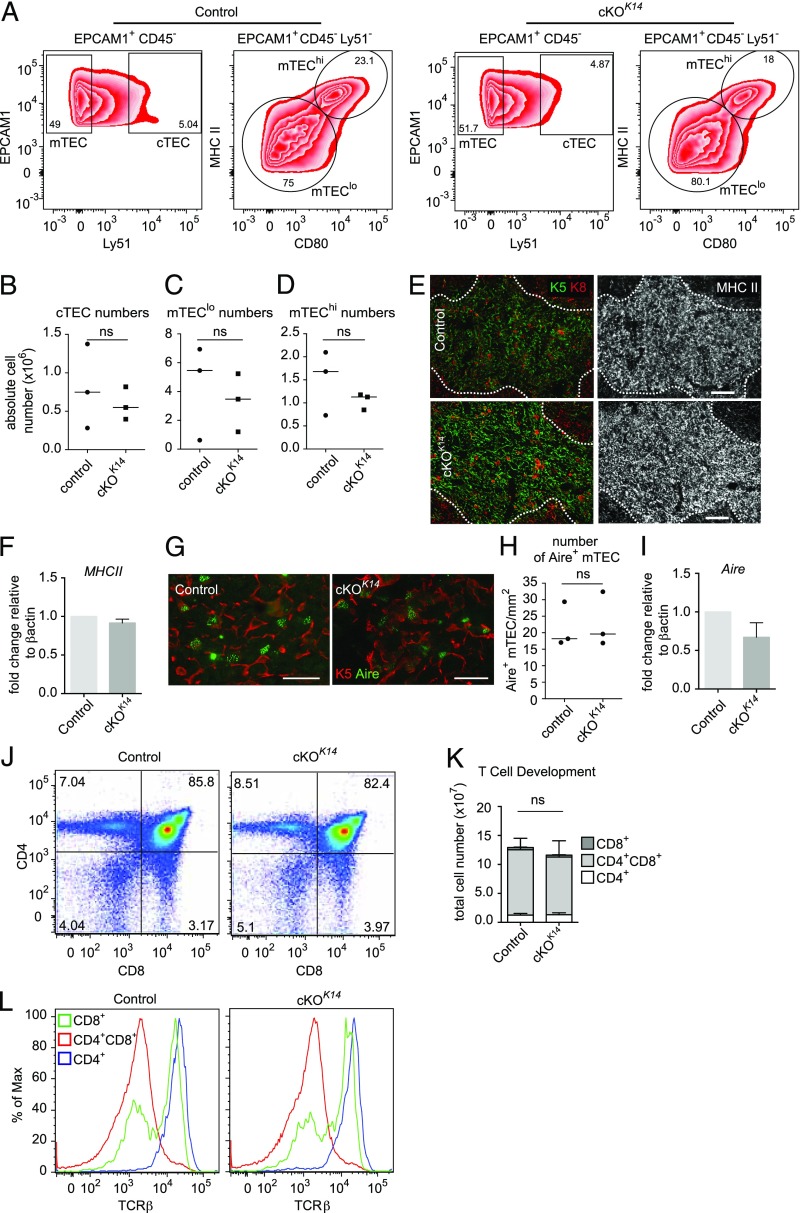
Normal TEC and T cell development in the absence of *Prdm1*. (**A**) Representative flow cytometry plots indicating the gating strategy for CD45^−^ cTECs (Ly51^+^EPCAM1^+^), immature mTECs (Ly51^−^EPCAM1^+^CD80^−^), and mature mTECs (Ly51^−^EPCAM1^+^CD80^+^) for both control and *Prdm1 cKO^K14^* thymus. (**B**–**D**) Summary of the absolute cell numbers for each of the indicated epithelial cell populations. *n* = 3 mice for each genotype. (B) *p* > 0.9999, (C) *p* = 0.7, (D) *p* = 0.7. (**E**) Representative fluorescence images of control and *Prdm1* cKO^K14^ thymus sections stained for (left) K5 (green), K8 (red), and (right) MHCII (gray). Scale bars, 100 μm. (**F**) mRNA expression of MHCII in FACS-isolated control and *Prdm1* cKO^K14^ mTECs. Data are mean (± SD) of at least three independent experiments. (**G**) Representative fluorescence images of control and *Prdm1 cKO^K14^* thymus sections stained for K5 (red) and Aire (green). Scale bars, 25 μm. (**H**) Quantification of the number of Aire-positive mTECs (K5^+^). *n* = 3 mice for each genotype. (**I**) Quantitative real-time PCR analysis of mRNA transcript levels of *Aire* in control and *Prdm1* cKO^K14^ mTECs. (**J**) Representative CD4 and CD8 profiles from control and *Prdm1* cKO^K14^ thymocytes. (**K**) Quantification of the total cell numbers for each thymocyte population of CD4^+^ and CD8^+^ single-positive cells and CD4^+^CD8^+^ double-positive cells. (**L**) Histograms illustrating the expected upregulation of the TCRβ with thymocyte maturation. Data are mean (B–D, H) or mean ± SD (F, I–K). *n* = 3 mice for each genotype. ns, not significant.

Given the autoimmune pathology in *Prdm1* cKO*^K14^* mice, the expression of *Prdm1* in TECs, and the Cre activity in *K14*-Cre mice, we hypothesized that Prdm1 may regulate TEC development or function. To identify whether TECs developed normally in *Prdm1* cKO*^K14^* mice, we analyzed control and *Prdm1* cKO*^K14^* TECs by flow cytometry for the expression of EPCAM1, Ly51, CD80, and MHCII to examine TEC maturation (42, 53) (Fig. 3A). cTECs (EPCAM1^+^ Ly51^hi^), immature mTECs (EPCAM1^+^ Ly51^lo^ MHCII^lo^ CD80^lo^), and mature mTECs (EPCAM1^+^ Ly51^lo^ MHCII^hi^ CD80^hi^) were present in *Prdm1* cKO*^K14^* at similar cell numbers compared with littermate control mice (Fig. 3B–D). Similarly, thymi from *Prdm1* cKO*^FoxN1^* mice and control mice displayed similar numbers of cTEC and mTEC populations (Supplemental Fig. 2F–I). Crucially, the levels of MHCII expression were comparable between control and *Prdm1* cKO*^K14^* mTECs (Fig. 3A, 3E, 3F). Furthermore, the gross thymic architecture was unaltered in the absence of Prdm1 (Fig. 3E).

To further confirm that development of the mTEC compartment was normal in the absence of *Prdm1*, we investigated Aire expression in *Prdm1* cKO*^K14^* mice. Similar numbers of K5^+^, Aire^+^ cells were present in sections of *Prdm1* cKO*^K14^* thymi compared with littermate controls (Fig. 3G, 3H). In addition, the level of *Aire* mRNA expression was comparable between littermate control and *Prdm1* cKO*^K14^* mice mTECs (Fig. 3I).

To investigate whether T cell development was impaired in the absence of *Prdm1*, we isolated thymocytes from control and *Prdm1* cKO*^K14^* mice or *Prdm1* cKO*^FoxN1^* mice, stained them for CD4, CD8, and TCRβ, and analyzed them by flow cytometry. Both control and *Prdm1* cKO*^K14^* mice displayed typical CD4 and CD8 profiles (Fig. 3J) and cell numbers (Fig. 3K), suggesting T cell development was not grossly impaired in these mice. In addition, TCRβ expression in CD4^+^ and CD8^+^ T cells from *Prdm1* cKO*^K14^* mice was as seen in control mice (Fig. 3L), suggesting normal TCR expression. Normal T cell development also occurred in *Prdm1* cKO*^FoxN1^* mice (Supplemental Fig. 2J–L).

**FIGURE 4.**
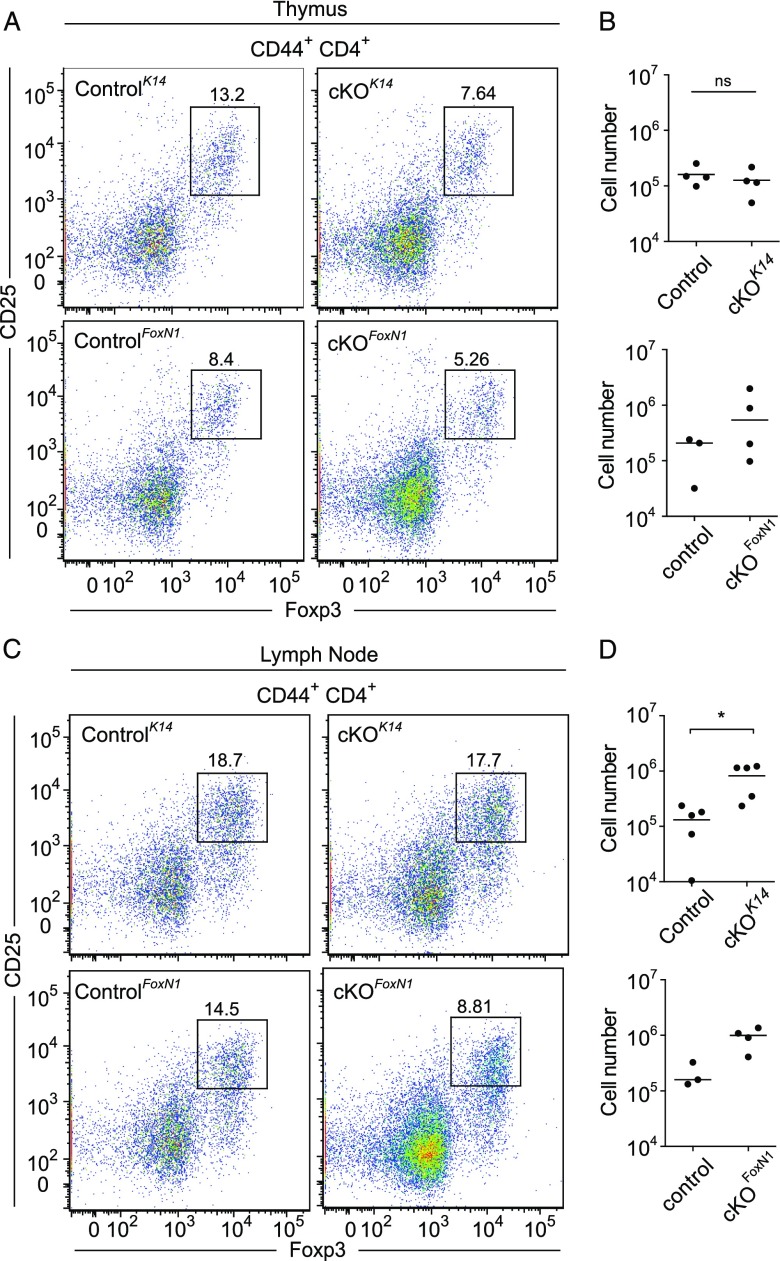
Spontaneous development of autoimmunity is not the result of an absence of Tregs. (**A**–**C**) Representative flow cytometric plots showing CD44^+^CD4^+^CD25^+^Foxp3^+^ Tregs in thymus and cervical (mandibular and accessory mandibular) lymph nodes. Note the levels of Foxp3 and CD25 are similar in control, *Prdm1* cKO*^K14^*, and *Prdm1* cKO*^FoxN1^*. (B) Absolute cell numbers of Tregs found within the thymus are not significantly different between control and cKO mice. (**D**) Absolute cell numbers of Tregs found within the cervical lymph node suggest an increase in cKO mice compared with control. Data are mean. *n* ≥ 3 mice for each genotype; each point represents an individual mouse. *p = 0.0159. ns, not significant.

Tregs develop within the thymus and require mTECs (4, 56–58). To define whether the loss of epithelial *Prdm1* expression resulted in Treg developmental defects, we isolated thymocytes from control, *Prdm1* cKO*^K14^*, and *Prdm1* cKO*^FoxN1^* mice, stained them for CD44, CD25, CD4, and Foxp3, and analyzed them by flow cytometry (Fig. 4). Tregs (CD4^+^, CD44^+^, CD25^+^, Foxp3^+^) were present in similar numbers in thymi from control, *Prdm1* cKO^*K14*^, and *Prdm1* cKO*^FoxN1^* mice (Fig. 4A, 4B). In addition, the levels of CD25 and Foxp3 were comparable among control, *Prdm1* cKO*^K14^*, and *Prdm1* cKO*^FoxN1^* Tregs, suggesting normal development of this population. Importantly, Tregs were also identified within peripheral lymphoid tissues of *Prdm1* cKO*^K14^*, *Prdm1* cKO*^FoxN1^*, and control mice (Fig. 4C, 4D). Taken together, these data suggest that in the absence of *Prdm1*, the thymic epithelium develops normally and is able to support a normal T cell developmental program including Tregs.

### *Prdm1* functions in the thymus to regulate autoimmunity

**FIGURE 5.**
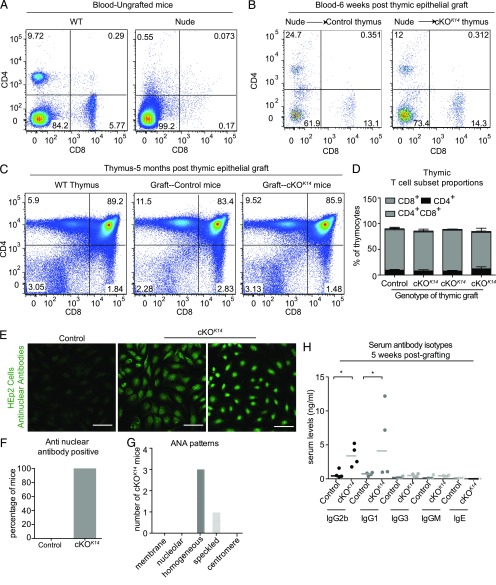
Transplantation of *Prdm1* cKO^*K14*^ thymic stroma leads to autoimmunity. Representative flow cytometry analysis of blood CD4 and CD8 T cells in WT and Nude mice prior to transplantation (**A**) or 6 wk after grafting of control or *Prdm1* cKO*^K14^* embryonic (E15) thymus lobes under the kidney capsule (**B**). (**C**) Analysis of CD4 and CD8 T cell development in the grafted thymic epithelial tissue after 5 mo revealed T cell profiles similar to those in WT thymus. (**D**) Quantification of percentage of T cell subsets between control and *Prdm1* cKO*^K14^* grafts. Each bar represents three or more independently analyzed thymic grafts harvested from a single host. (**E**) Compared with serum isolated from control host mice, *Prdm1* cKO*^K14^* host serum contains ANAs as indicated by positive staining of HEp2 cells. Representative images from sera derived from one host, engrafted with thymi from control, or two hosts engrafted with thymi from *Prdm1* cKO^*K14*^ mice. Scale bars, 50 μm. (**F**) The presence of ANAs is more frequent in *Prdm1* cKO*^K14^* host mice compared with control. (**G**) Homogenous and speckled nuclear staining patterns were observed from sera isolated from mice engrafted with thymi from *Prdm1* cKO*^K14^* mice. (**H**) ELISA of serum Ab isotypes after 5 wk of engraftment. Note the increase in IgG2b and IgG1 in *Prdm1* cKO*^K14^* serum compared with control serum. Data are mean. *n* = 4 grafted mice for each genotype of transplanted thymi. *p < 0.05.

To definitively ascertain whether autoimmunity associated with epithelial deletion of *Prdm1* was due to a role of Prdm1 within TECs, we determined whether transplanted thymic epithelial tissue from *Prdm1* cKO*^K14^* mice could generate autoimmune phenotypes. Thymus lobes from E15 littermate control and *Prdm1* cKO*^K14^* mice were treated with 2-deoxyguanosine and grafted under the kidney capsule of 6- to 8-wk-old, male, Nude mice, which lack T cells because of the absence of a functional thymus (59) (Fig. 5A). Six weeks after engraftment, blood from Nude host mice grafted with control or *Prdm1* cKO*^K14^* thymus under the kidney capsule contained CD4^+^ and CD8^+^ T cells, as seen in WT blood (Fig. 5B), confirming successful and functional engraftment (5). After 5 mo, thymic grafts from both control and *Prdm1* cKO*^K14^* mice contained thymocytes expressing CD4 and CD8 similar to WT thymus (Fig. 5C). Furthermore, the percentage of developing T cell populations, CD4^+^, CD8^+^ and CD4^+^CD8^+^, were comparable between the hosts and WT mice (Fig. 5D), indicating maintenance of the epithelial grafts and confirming successful T cell development.

To determine whether thymi from *Prdm1* cKO*^K14^* mice were sufficient to induce autoimmune phenotypes in Nude host mice, we evaluated the serum of host mice for the presence of ANAs. Although none of the mice engrafted with control thymi had ANAs after 5 wk, 100% of the mice engrafted with thymi from *Prdm1* cKO*^K14^* mice displayed ANAs (Fig. 5E, 5F). The pattern of HEp2 nuclear staining was mostly homogeneous and speckled (Fig. 5G), similar to that observed in the *Prdm1* cKO*^K14^* mice (Fig. 1H). Furthermore, the sera of Nude mice engrafted with control thymi did not contain elevated levels of IgG isotypes, whereas the sera of Nude mice engrafted with *Prdm1* cKO*^K14^* thymi displayed elevated levels of IgG2b and IgG1 (Fig. 5H). Both of these isotypes were also elevated in *Prdm1* cKO*^K14^* mice (Fig. 1I) and are T cell dependent (60), suggesting a T cell–mediated response in the autoimmune phenotype of the mice.

Recent work has suggested that expression of TSAs is coordinated within populations of mTECs (38). To determine whether *Prdm1*^+^ mTECs display unique gene expression patterns, we performed an in silico analytical approach to assess the coexpression of genes within single-cell RNA sequencing data from CD45^–^, Ly51^lo^, MHCII^hi^, Aire-positive mTECs (52) (Supplemental Fig. 3). Of the 200 cells analyzed, 24% of the cells displayed expression of *Prdm1*, supporting the expression of *Prdm1* in a subset of mTECs (Fig. 2B, Supplemental Fig. 3A). Furthermore, this analysis demonstrated that *Aire* and *Prdm1* are coexpressed within a subset of mTECs. *Prdm1* expression was detected in both WT and *Aire* KO mTECs, which is consistent with similar levels of *Prdm1* expression in *Aire* KO mice (Supplemental Fig. 3B).

Global analysis of gene expression in Prdm1^+^ mTECs revealed distinct patterns of genes compared with *Prdm1*^–^ mTECs (Supplemental Fig. 3A). Spearman-rank correlation analysis between *Prdm1* and all expressed genes within the Meredith RNA-sequencing dataset indicated that a predominant number of transcripts were associated with *Prdm1* expression (Supplemental Fig. 3C). We further filtered this gene set using a stringent cutoff of *p* ≤ 0.0005 to identify robust *Prdm1*-associated genes (Supplemental Fig. 3D, Supplemental Table I). To determine whether *Prdm1* expression was correlated with TSA-associated gene expression patterns in Aire^+^ mTECs, we compared *Prdm1* expression with previously defined Aire-dependent or Aire-independent genes derived from three TSA-encoding genes: *Tspan8*, *Ceacam1*, and *Klk5* (38, 39). Interestingly, we found that *Prdm1* was more significantly linked with Aire-independent TSA-associated genes than Aire-dependent TSA-associated genes or all expressed genes (Supplemental Fig. 3E, 3F, Supplemental Table I). In addition, *Prdm1* and *Aire* did not correlate with each other (rho = 0.07, *p* = 0.162). Together, these data suggest that Prdm1^+^ mTECs represent a unique subset of mTECs important in the prevention of autoimmunity.

## Discussion

Our study has revealed a novel mechanism for TEC function via the transcription factor Prdm1. It is intriguing to speculate that Prdm1 regulates TEC function by regulating TSA expression in a subset of TECs either in coordination or independently from the transcription factor Aire. Perhaps, Prdm1 creates some of the epigenetic markers that Aire is thought to recognize to enable Aire’s subsequent activity (61–63). Our in silico analysis of gene expression in single mTECs reveals that Prdm1 may regulate a subset of TSAs and in particular, Aire-independent TSAs. Other studies have indicated distinct functions of Aire in a cell type–dependent manner (6, 64, 65).

Prdm1 and its human ortholog PRDI-BF1 elicit their transcriptional modulation by several different mechanisms, including direct competition for DNA binding (66) and recruitment of corepressor proteins such as the Groucho family that function in part through association with HDACs (12, 67). Prdm1 has been shown to interact with several chromatin-modifying proteins, for example, G9a lysine methyltransferase (10), HDAC1/2 (13), protein arginine methyltransferase 5 (68), and lysine deacetylase 1 (69). Thus, it seems possible that PRDI-BF1 might act as a DNA-binding scaffold protein capable of directly recruiting multiple chromatin-modifying enzymes to targeted promoters (10). Interestingly, Prdm1 is associated with HDAC5 expression in individual mTECs, and although further validation of this interaction remains an opportunity for future study, this may provide an additional epigenetic mechanism for regulation of TSAs and a novel interaction for Prdm1.

Furthermore, although Prdm1 is most commonly known as a transcriptional repressor, it has been identified in transcriptional activation (16, 70). Therefore, it is possible that Prdm1 could be directly promoting TSA expression; the exact mechanism by which Prdm1 controls TEC function will be an interesting area of future study.

Mice lacking *Prdm1* in epithelial cells develop chronic skin inflammation, which is thought to be due to defects in epidermal barrier immunity (31, 32). Until now, these observations have precluded the analysis of Prdm1’s role in the thymic epithelium. We find that Prdm1 is expressed within the medullary thymic epithelium and Hassall’s corpuscles, which are thought to represent the terminally differentiated epithelial compartment within the thymus (55). This is consistent with Prdm1’s expression in the most differentiated layers of the skin epidermis (26). Although the function of Hassall’s corpuscles has not been fully explored, they are thought to be involved in removing dead thymocytes, maturation of medullary thymocytes (71, 72), and education of dendritic cells during Treg development (73). Because thymus tissue from immunodeficient patients lacks Hassall’s corpuscles (74, 75), it is exciting to speculate that these structures play a role in establishing tolerance. It will be interesting to determine whether Prdm1 could be a therapeutic target to maintain thymic function in these patients.

Intriguingly, Prdm1 does not affect mTEC development, as indicated by its expression within the most differentiated cell types within the thymus and by the absence of a defect in the epithelial compartment of the *Prdm1* cKO*^K14^* and *Prdm1* cKO*^FoxN1^* thymus. Together with the absence of an identified association with HDAC3 in the global analysis of gene expression, these data suggest that Prdm1 works independently of HDAC3, which has recently been reported to be a master regulator of mTEC development and important for epigenetic regulation of Prdm1 in B lymphocytes (76, 77).

Slightly different phenotypes are observed between the Prdm1 cKO*^K14^* and cKO*^FoxN1^* mouse models. Although the reasons for these differences remain unclear, it is important to note that the timing and pattern of expression of Cre in the K14Cre mouse is not well defined, despite both strains being frequently used as models to delete genes from total thymic epithelium. Furthermore, *Foxn1* is comprised of eight coding exons and utilizes two alternative first exons in a tissue-specific fashion (78, 79); therefore, it is conceivable that FoxN1cre activity remains absent from the skin after birth (80), whereas it is possible that the K14cre is active within the skin.

Importantly, *Prdm1* cKO*^K14^* thymus transplantation demonstrates that Prdm1 deficiency in thymic epithelium is sufficient to elicit a breakdown in thymic tolerance because this model enabled the role of Prdm1 in thymic epithelium to be separated from its known role within skin epidermis (27, 32). With functional Prdm1 in the epidermis, hosts that received *Prdm1*-deficient thymus lobes developed ANAs. However, it was intriguing that, despite this, other phenotypes observed in the cKO^K14^, such as cervical lymphadenopathy, were not observed. Perhaps this could be attributable to a previously undetermined role for Prdm1 in other barrier epithelial tissues, promoting their functional integrity and homeostasis, such as the tongue and esophagus, where the activity of the K14 promoter has been identified (81); further investigation would be required to clarify this. In addition, impaired containment of the commensal microflora at these sites could explain the local inflammation resulting in enlarged cervical lymph nodes.

Because it is known that Prdm1 negatively regulates the expression of CIITA, a transcription factor that regulates MHCII gene expression (82), and that TSA expression is dependent on MHC expression levels, it was critical to check the protein and mRNA expression levels of MHCII; these did not differ between control and Prdm1 cKO*^K14^* mice, ruling out a dysregulation of MHCII in the autoimmune phenotype presented by these mice. In addition, it was important to decipher whether *Prdm1* was an Aire-regulated gene. To do this, we analyzed mTECs from both WT and *Aire* KO for *Prdm1* expression, which was detected at similar levels in both WT and *Aire* KO mTECs (Supplemental Fig. 3F), implying that *Prdm1* is not an Aire-dependent gene.

Despite the ability of mTECs and Hassall’s corpuscles to direct Treg development (4, 56–58, 73), loss of *Prdm1* in epithelial cells did not alter Treg development or export to the periphery because comparable numbers within both the thymus and the peripheral lymph nodes were identified in control and *Prdm1* cKO*^K14^* and *Prdm1* cKO*^FoxN1^* mice. This suggests that the autoimmune phenotype that presented in *Prdm1* cKO mice was not due to an absence of immune regulation and that *Prdm1* null mTECs are unable to efficiently delete self-reactive T cells.

Interestingly, polymorphisms in *Prdm1* are associated with autoimmune diseases, such as SLE (33, 34). Importantly, dermatitis (83, 84), hypergammaglobulinemia, and kidney immune complex deposition are frequently used as diagnostic markers for SLE. Furthermore, ANAs (50), particularly the homogenous and speckled patterns, are prevalent within patients with SLE (85). Importantly, we show that *Prdm1* cKO*^K14^* mice develop SLE-associated symptoms, including ANAs, hypergammaglobulinemia, and kidney immune complex deposition, suggestive of glomerulonephritis (33, 83, 84, 86), and previous studies reported the dermatitis using the K14-Cre and K5-Cre mouse models (31, 32). Together, these data raise the interesting possibility that SLE may be driven by a breakdown in thymic tolerance, and the data are consistent with a role for Prdm1 in SLE. Of relevance, we show that the autoantibodies produced in *Prdm1* cKO*^K14^* and *Prdm1* cKO*^FoxN1^* mice are the T cell–dependent isotypes, IgG2b and IgG1 (60). This highlights the requirement of T cell help for Ab production in this model, consistent with a T cell component in activating autoreactive B cells in SLE.

Collectively, our findings show that despite Prdm1 being expressed within the medullary thymic epithelium, particularly the Hassall’s corpuscles, it does not regulate mTEC development and does not alter T cell development including intrathymic Treg development. The absence of Prdm1 from the thymic epithelium does, however, result in an autoimmune-like phenotype that manifests as the presentation of ANAs and hypergammaglobulinemia that is transferable by transplantation of *Prdm1* cKO thymus lobes into Nude hosts, uncoupling the role of *Prdm1* in the skin epithelium from being the sole reason for the phenotype presented by cKO mice. Furthermore, our data present the possibility that Prdm1 is involved in the regulation of Aire-independent TSA expression within the thymus, thus adding another dimension to the association of *Prdm1* mutations with autoimmune disorders such as SLE (33), rheumatoid arthritis (87), and Crohn’s disease (88).

## Supplementary Material

Data Supplement
