# Supplementary material for: Prdm1 Regulates Thymic Epithelial Function To Prevent Autoimmunity
Source: J Immunol. 2017 Jul 12;199(4):1250–60. doi: 10.4049/jimmunol.1600941 (PMC5544928; doi:10.4049/jimmunol.1600941)
Supplement: Data Supplement [file JI_1600941.zip › JI_1600941_Supplemental_Material_1.pdf]

SFigure 1. Roberts et al.

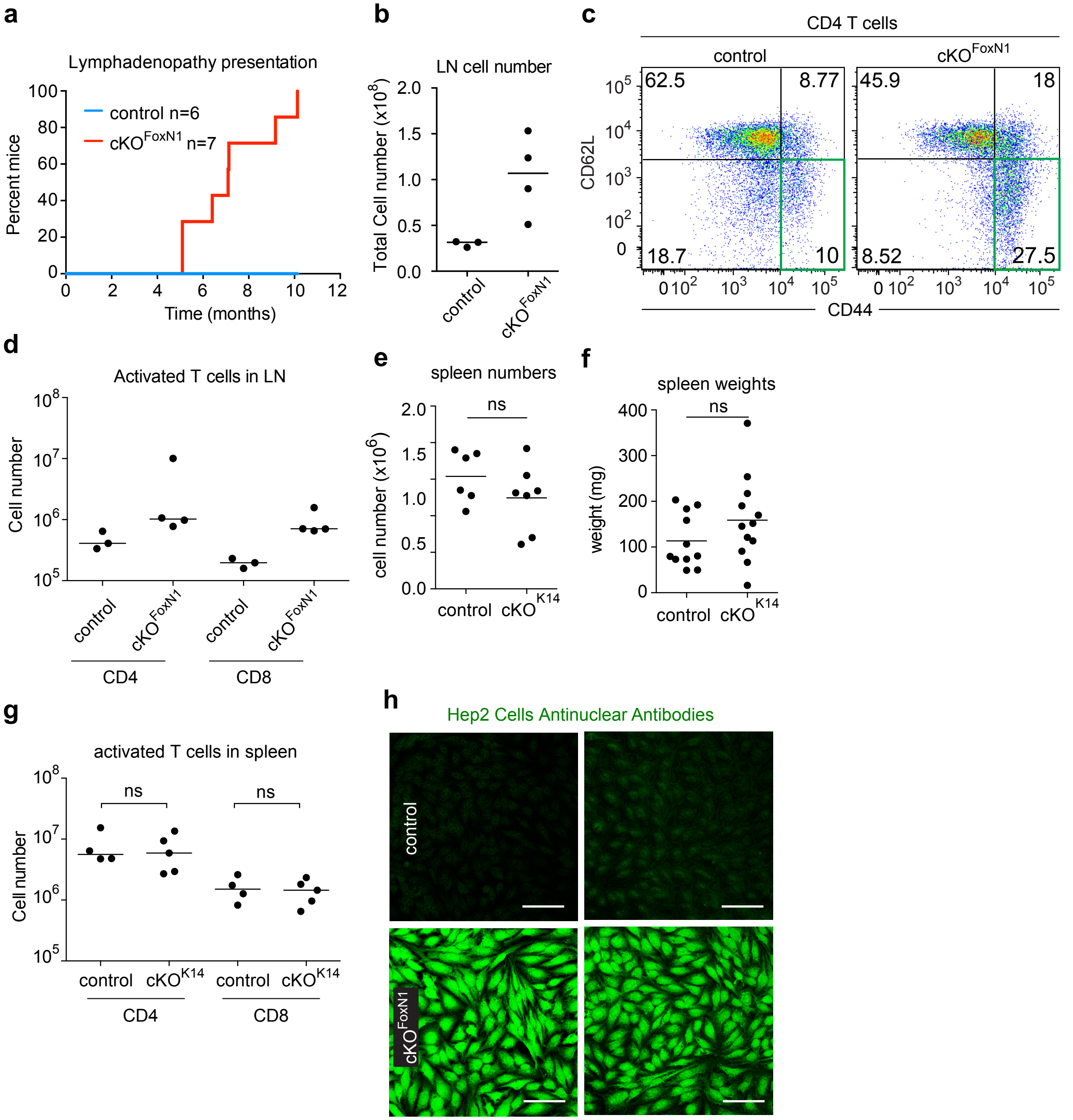

Supplementary Figure 1. Conditional deletion of Prdm1 from epithelium promotes spontaneous development of autoimmunity.
(a) Quantification of cervical lymphadenopathy presentation in control (n=6) and Prdm1 cKO^{FoxN1} (n=7) mice. (b) Total lymphocyte numbers isolated from cervical lymph nodes of Prdm1 cKO^{FoxN1} and control mice. Note cellular infiltration of Prdm1 cKO^{K14} lymph nodes. (c) Flow cytometric analysis of lymphocytes isolated from cervical lymph nodes shows an increase in proportion of $CD44^{+}CD62L^{-}$ activated T cells in Prdm1 cKO^{FoxN1} mice copared to control mice. (d) Absolute cell numbers of activated CD4 and CD8 T cells highlights the increase in the CD4 T cell compartment in Prdm1 cKO^{FoxN1} cervical lymph nodes. (e) Total lymphocyte number isolated from spleen of Prdm1 cKO^{K14} and control mice. Each point represents an individual mouse, n≥6. (f) Spleen weights (mg) of Prdm1 cKO^{K14} and control mice. Each point represents an individual mouse, n≥11. (g) Absolute cell numbers of $CD44^{+}CD62L^{-}$ activated CD4 and CD8 T cells from spleen of Prdm1 cKO^{K14} and control mice. Each point represents an individual mouse, n≥4. ns= not significant; p=0.3869 (e), p=0.2318 (f). (h) Serum isolated from Prdm1 cKO^{FoxN1} mice contains antinuclear antibodies indicated by positive staining of HEp2 cells. Scale bar = 50μm. Data are mean, n=3-4 mice for each genotype.

SFigure 2. Roberts et al.

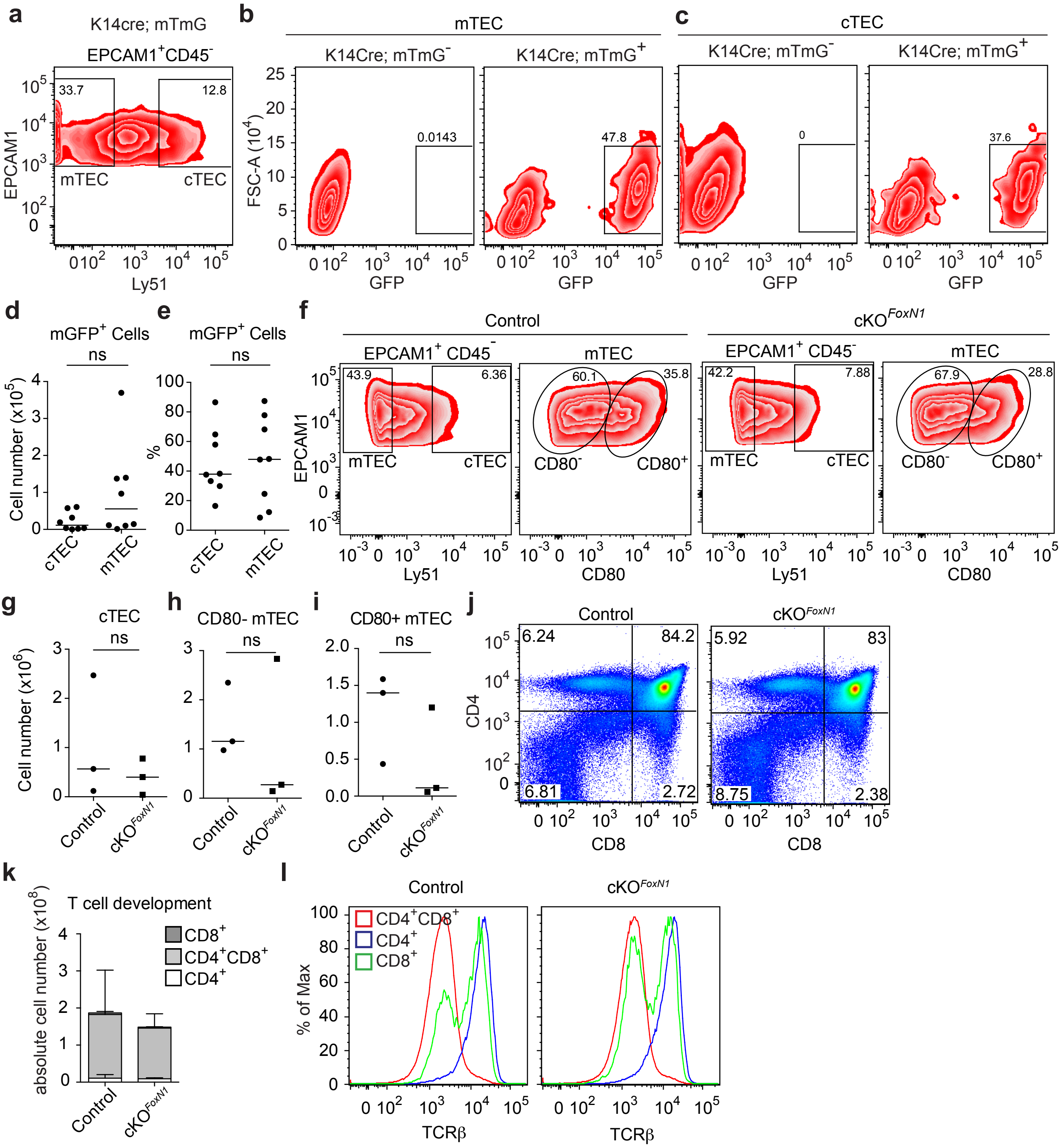

Supplementary Figure 2. Thymic keratin 14 cre recombinase activity and thymic epithelial cell and T cell development in the absence of Prdm1 from FoxN1 expressing cells. (a) Single cell suspensions of thymic epithelium isolated from control and K14-Cre;mTmG mice were immunostained for mTEC (CD45$^-$ EPCAM1$^+$ Ly51$^-$) and cTEC (CD45$^-$EPCAM1$^+$ Ly51$^+$) populations. mTEC (b) and cTEC (c) populations indicated in A, were analysed for GFP expression. GFP positive cells were present in both TEC populations compared to control indicating Cre recombinase activity. Data are representative of 8 mice for each cell population. Absolute cell numbers (d) and proportions (e) of GFP$^+$ cells in cTEC and mTEC populations. (f) Representative flow cytometry plots indicating the gating strategy for the CD45$^-$ cTEC (Ly51$^+$EPCAM1$^+$) and mTEC (Ly51$^-$EPCAM1$^+$CD80$^-$) or (Ly51$^-$EPCAM1$^+$CD80$^+$) populations for both control and Prdm1 cKO^{FoxN1} thymus. (g-i) Summary of the absolute cell numbers for each of the indicated epithelial cell populations. (j) Representative CD4 and CD8 profiles from control and Prdm1 cKO^{FoxN1} thymocytes. (k) Quantification of the total cell numbers for each thymocyte population CD4$^+$ and CD8$^+$ single positive and CD4$^+$CD8$^+$ double positive cells. (l) Histograms illustrating the expected upregulation of the T cell receptor beta chain (TCRβ) with thymocyte maturation in Prdm1 cKO^{FoxN1} mice. Data are mean, n=3 mice for each genotype. ns, not significant; p=0.7 (g), p=0.4 (h), p=0.2 (i).

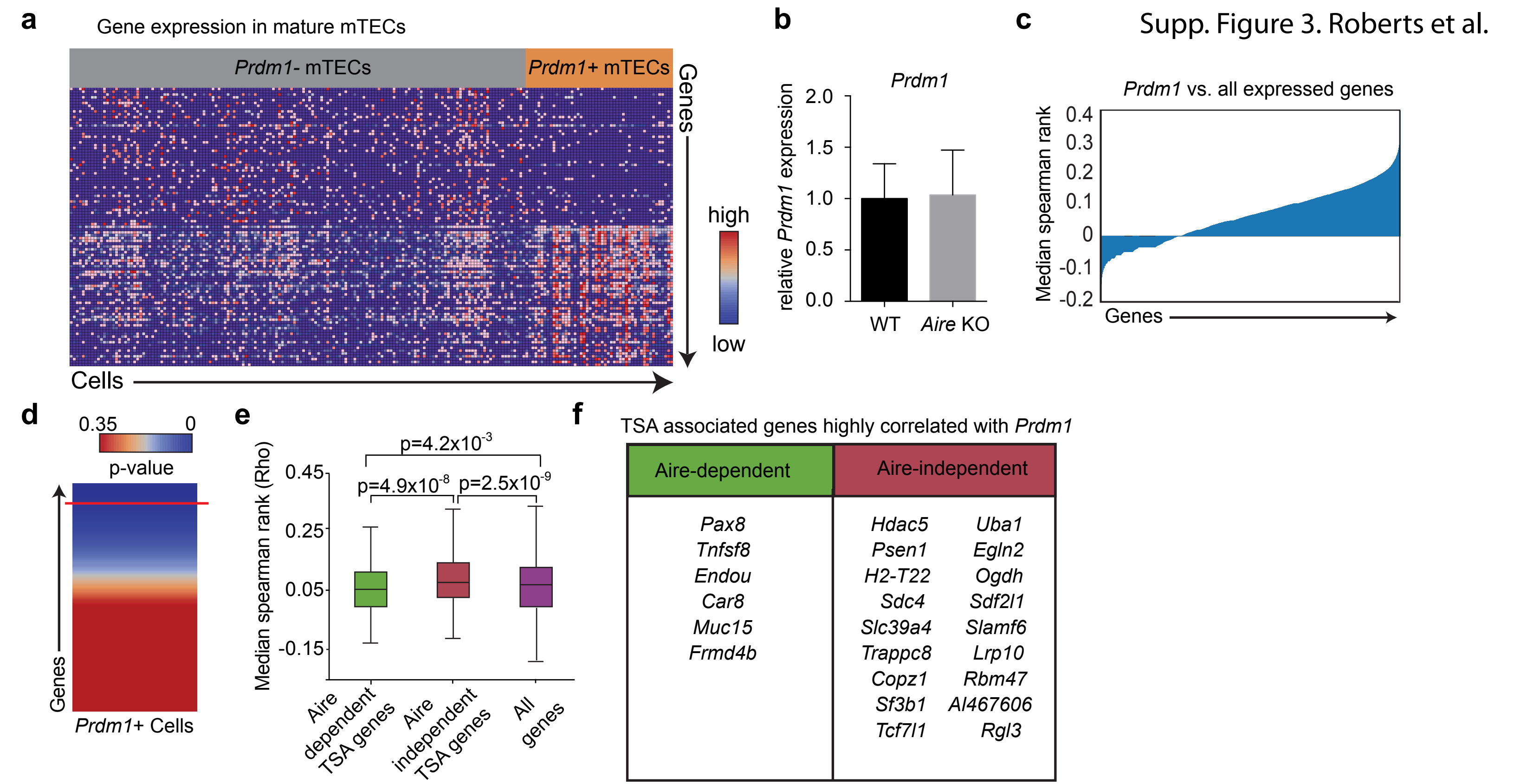

Supplementary Figure 3. Prdm1 associates with Aire-independent tissue specific antigen expression in mTECs. (a) Heatmap from GSEA analysis of the top 50 features associated with Prdm1. Range of colors (red, pink, light blue, dark blue) indicates the range of expression values (high, moderate, low, lowest) respectively. b) mRNA expression of Prdm1 in mTECs from WT and Aire KO mice. Data are mean (±SD) of at least three independent experiments. (c) Bar plot of all Spearman-Rank rho associations associated with Prdm1 in all 200 single cells and all 36,017 genes. (d) Significance plot analysis of the Prdm1 associations described in B. Alpha cut-off of 0.0005 is indicated by the red line. (e) Box-whisker plots of Prdm1 associations in Aire-dependent, Aire independent, and all gene data sets. Student's t tests were performed to generate p values from each comparison noted in figure.
(f) Table highlighting the Aire-dependent and Aire independent tissue specific antigen genes highly correlated with Prdm1 expression. Original data (E-MTAB-3346 and E-MTAB-3624) (45) and (GSE53111) (47).

Table S1, Related to Supplemental Figure 3C. Prdm1 associated genes

GENE NAME	GENE NUMBER	Spearman rho	Rank Order	pvalue
Prdm1	*1*	1	20031.5	#DIV/0!
Aaas	2	0.183636212	17578	9.243807E-03
Aacs	*3*	0.044335547	7885	5.330375E-01
Aadac	4	-0.021524641	4190.5	7.622470E-01
Aadacl2	*5*	0.020875719	6412.5	7.692098E-01
Aadacl3	6	0.052728522	8550.5	4.583686E-01
Aadat	*7*	0.112793664	12907	1.117844E-01
Aaed1	8	0.11470357	13306	1.058074E-01
Aagab	*9*	0.042572659	7780	5.494623E-01
Aak1	10	0.083073284	10840	2.422053E-01
Aamdc	*11*	0.025042813	6822	7.248426E-01
Aamp	12	0.126246308	14061	7.485765E-02
Aanat	*13*	-0.038406171	3065	5.892384E-01
Aar2	14	0.137334675	14780	5.247330E-02
Aard	*15*	0.139960134	14948	4.807908E-02
Aars	16	0.066080078	9596	3.525407E-01
Aars2	*17*	0.003294601	5492	9.630705E-01
Aarsd1	18	0.254766368	19561	2.719868E-04
Aasdh	*19*	0.025969355	6869	7.150952E-01
Aasdhppt	20	0.17547352	17187	1.294314E-02
Aass	*21*	0.077283761	10428	2.767124E-01
Aatf	22	0.058329459	9110	4.119702E-01
Aatk	*23*	-0.013539251	4591	8.490878E-01
AB041803	24	0.087930923	11199	2.156656E-01
AB041806	*25*		#N/A	1.000000E+00
AB099516	26	0.066539708	9623	3.491943E-01
AB124611	*27*	0.001885981	5415	9.788548E-01
Abat	28	0.009696009	5761	8.916110E-01
Abca1	*29*	-0.015801595	4481	8.242505E-01
Abca12	30	-0.040645292	2460	5.676984E-01
Abca13	*31*	-0.047877152	2240	5.008026E-01
Abca14	32	0.063483595	9432	3.718211E-01
Abca15	*33*	0.034384503	7306	6.288364E-01
Abca16	34	-0.066156793	1302	3.519808E-01
Abca17	*35*	0.042734919	7784	5.479403E-01
Abca2	36	0.115538087	13361	1.032762E-01
Abca3	*37*	0.120894126	13718	8.814879E-02
Abca4	38	-0.025642029	4082	7.185336E-01
Abca5	*39*	-0.020801906	4211	7.700029E-01
Abca6	40	0.01433127	6030	8.403743E-01
Abca7	*41*	-0.015510112	4493	8.274413E-01
Abca8a	42	-0.077393517	797	2.760287E-01

Spearman Rank correlation analysis performed between Prdm1 and all-expressed genes within the Meredith RNA-seq dataset. Robust Prdm1–associated genes were identified using a cut-off of $p < 0.0005$.

Table S1, Related to Supplemental Figure 3C. Prdm1 associated genes

Abca8b	*43*	0.046994573	8076	5.087386E-01
Abca9	44	0.039813567	7624	5.756557E-01
Abcb10	*45*	0.099902217	12058	1.592796E-01
Abcb11	46	0.014494232	6042	8.385838E-01
Abcb1a	*47*	-0.083357749	596	2.405910E-01
Abcb1b	48	0.106170988	12437	1.345741E-01
Abcb4	*49*	0.084201007	10934	2.358496E-01
Abcb5	50	-0.06485515	1325.5	3.615570E-01
Abcb6	*51*	0.019881932	6330	7.799086E-01
Abcb7	52	0.140458685	14985	4.728033E-02
Abcb8	*53*	0.147388776	15499	3.727719E-02
Abcb9	54	0.117505337	13494	9.749804E-02
Abcc1	*55*	0.11173327	12834	1.152151E-01
Abcc10	56	0.068038809	9705	3.384195E-01
Abcc12	*57*	0.007210755	5639	9.192825E-01
Abcc2	58	-0.054451513	1703	4.437940E-01
Abcc3	*59*	0.052887254	8587	4.570148E-01
Abcc4	60	-0.054709316	1530	4.416361E-01
Abcc5	*61*	0.169277677	16893	1.656326E-02
Abcc6	62	0.090858474	11420.5	2.007115E-01
Abcc8	*63*	0.001621973	5399	9.818143E-01
Abcc9	64	0.004870768	5556	9.454261E-01
Abcd1	*65*	0.279703672	19794	6.041886E-05
Abcd2	66	0.027578552	6955	6.982760E-01
Abcd3	*67*	0.073962586	10135	2.979410E-01
Abcd4	68	0.128158632	14193	7.052227E-02
Abce1	*69*	0.038630619	7537	5.870624E-01
Abcf1	70	0.099431698	12028	1.612630E-01
Abcf2	*71*	0.08170699	10719	2.500645E-01
Abcf3	72	0.078226963	10481	2.708748E-01
Abcg1	*73*	0.085552196	11024	2.283899E-01
Abcg2	74	-0.008926864	4790	9.001625E-01
Abcg3	*75*	0.028490609	6993	6.888075E-01
Abcg4	76	-0.086746694	532	2.219355E-01
Abcg5	*77*	0.193435608	17968	6.061889E-03
Abcg8	78	0.030320056	7078	6.699616E-01
Abhd1	79	0.233664795	19250	8.683795E-04
Abhd10	*80*	-0.12367809	94	8.101722E-02
Abhd11	81	-0.008754278	4797	9.020829E-01
Abhd12	82	0.061249673	9276	3.889181E-01
Abhd12b	*83*	-0.033075221	3785	6.419675E-01
Abhd13	84	0.194921475	18040	5.676454E-03
Abhd14a	85	-0.1069009	200	1.319000E-01

Spearman Rank correlation analysis performed between Prdm1 and all-expressed genes within the Meredith RNA-seq dataset. Robust Prdm1–associated genes were identified using a cut-off of $p < 0.0005$.

Table S1, Related to Supplemental Figure 3C. Prdm1 associated genes

Abhd14b	*86*	0.129760779	14286	6.704944E-02
Abhd15	87	0.235990603	19288	7.678400E-04
Abhd16a	88	-0.111764195	147	1.151139E-01
Abhd16b	*89*	-0.066855996	1239.5	3.469032E-01
Abhd17a	90	0.03492999	7328	6.233994E-01
Abhd17b	91	0.202706757	18346	3.993381E-03
Abhd17c	*92*	0.174450975	17145	1.348790E-02
Abhd2	93	0.143915668	15307	4.204103E-02
Abhd3	94	0.12125232	13742	8.720400E-02
Abhd4	*95*	0.13203164	14414	6.236767E-02
Abhd5	96	-0.05473846	1529	4.413925E-01
Abhd6	97	0.132192167	14425	6.204714E-02
Abhd8	*98*	0.166535197	16750	1.842902E-02
Abi1	99	0.124705281	13951	7.850633E-02
Abi2	100	0.130983625	14351	6.449387E-02
Abi3	*101*	0.000307056	5327	9.965570E-01
Abi3bp	102	-0.080108555	639	2.594812E-01
Abl1	103	0.064187248	9484	3.665330E-01
Abl2	*104*	0.076233487	10363	2.833120E-01
Ablim1	105	0.18774385	17752	7.763834E-03
Ablim2	106	-0.061546031	1381	3.866230E-01
Ablim3	*107*	0.082484026	10789	2.455734E-01
Abo	108	-0.038406171	3065	5.892384E-01
Abp1	109	0.020078445	6332	7.777896E-01
Abr	*110*	-0.020784296	4212	7.701922E-01
Abra	111		#N/A	1.000000E+00
Abracl	112	0.070996715	9911	3.177884E-01
Abt1	*113*	0.111186706	12802	1.170153E-01
Abtb1	114	0.001163	5370	9.869598E-01
Abtb2	115	-0.001412331	5123	9.841645E-01
Acaa1a	*116*	0.129558529	14276	6.747998E-02
Acaa1b	117	0.033772895	7283	6.349562E-01
Acaa2	118	0.06455496	9507	3.637882E-01
Acaca	*119*	0.076605126	10387	2.809647E-01
Acacb	120	0.053988555	8864	4.476841E-01
Acad10	121	0.143036064	15143	4.332557E-02
Acad11	*122*	0.134610138	14594	5.738054E-02
Acad12	123	0.074574898	10201.5	2.939481E-01
Acad8	124	0.147029326	15480	3.774807E-02
Acad9	*125*	0.173466521	17100	1.403126E-02
Acadl	126	0.182497599	17530	9.695992E-03
Acadm	127	0.117963386	13523	9.619019E-02
Acads	*128*	0.087115402	11136	2.199696E-01

Spearman Rank correlation analysis performed between Prdm1 and all-expressed genes within the Meredith RNA-seq dataset. Robust Prdm1–associated genes were identified using a cut-off of $p < 0.0005$.

Table S1, Related to Supplemental Figure 3C. Prdm1 associated genes

Acadsb	129	0.076573698	10385	2.811627E-01
Acadvl	130	0.102436857	12208	1.489092E-01
Acan	*131*	-0.001224019	5151	9.862757E-01
Acap1	132	0.192672415	17938	6.268818E-03
Acap2	133	0.261103033	19639	1.881643E-04
Acap3	*134*	0.031188612	7139	6.610850E-01
Acat1	135	0.053850012	8856	4.488519E-01
Acat2	136	0.174689162	17150	1.335924E-02
Acat3	*137*	0.083820256	10909	2.379823E-01
Acbd3	138	0.215206853	18752	2.210799E-03
Acbd4	139	0.13745959	14786	5.225693E-02
Acbd5	*140*	0.119991663	13662	9.056552E-02
Acbd6	141	0.136008096	14702	5.481731E-02
Acbd7	142	0.07417953	10147.5	2.965222E-01
Accs	*143*	0.031917497	7182	6.536722E-01
Accsl	144		#N/A	1.000000E+00
Acd	145	0.118774176	13571	9.390936E-02
Ace	*146*	0.10637852	12450	1.338095E-01
Ace2	147	0.121565273	13761	8.638519E-02
Ace3	148		#N/A	1.000000E+00
Acer1	*149*	0.031615721	7165	6.567372E-01
Acer2	150	0.03013249	7072	6.718846E-01
Acer3	151	0.011829001	5868	8.679645E-01
Ache	*152*	-0.029984649	3927	6.734018E-01
Acin1	153	0.264680562	19674	1.521972E-04
Acly	154	0.099306083	12021	1.617956E-01
Acmsd	*155*	0.111327214	12811	1.165504E-01
Acn9	156	0.21677053	18814	2.048383E-03
Acnat1	157	0.175633322	17197	1.285978E-02
Acnat2	*158*	0.200462341	18259	4.425221E-03
Aco1	159	0.056615761	9016	4.258646E-01
Aco2	160	0.076477927	10377	2.817667E-01
Acot1	*161*	-0.008489435	4801	9.050311E-01
Acot10	162	0.018446509	6243	7.954347E-01
Acot11	163	-0.121525097	97	8.648996E-02
Acot12	*164*	0.153032851	15845	3.051003E-02
Acot13	165	0.089001428	11272	2.101076E-01
Acot2	166	0.15881329	16217	2.469403E-02
Acot3	*167*	-0.054451513	1703	4.437940E-01
Acot4	168	0.004603232	5542	9.484194E-01
Acot5	169	-0.066858259	1029.5	3.468868E-01
Acot6	*170*	-0.013540297	4588.5	8.490763E-01
Acot7	171	-0.000388153	5263	9.956476E-01

Spearman Rank correlation analysis performed between Prdm1 and all-expressed genes within the Meredith RNA-seq dataset. Robust Prdm1–associated genes were identified using a cut-off of $p < 0.0005$.

Table S1, Related to Supplemental Figure 3C. Prdm1 associated genes

Acot8	172	0.06912689	9789	3.307331E-01
Acot9	*173*	0.217239472	18825	2.001836E-03
Acox1	174	0.093585411	11619	1.874713E-01
Acox2	175	0.156971564	16104	2.643385E-02
Acox3	*176*	0.011698092	5862	8.694126E-01
Acoxl	177	0.000386421	5335	9.956671E-01
Acp1	178	0.101856578	12170	1.512370E-01
Acp2	*179*	0.225157858	19049	1.348206E-03
Acp5	180	0.125335174	13993	7.699795E-02
Acp6	181	-0.074840896	888	2.922247E-01
Acpl2	*182*	0.175330656	17180	1.301807E-02
Acpp	183	-0.059621967	1403	4.016696E-01
Acpt	184		#N/A	1.000000E+00
Acr	*185*		#N/A	1.000000E+00
Acrbp	186	-0.017814679	4377	8.022948E-01
Acrv1	187	-0.103166823	231.5	1.460195E-01
Acsbg1	*188*	0.064659469	9514	3.630105E-01
Acsbg2	189	-0.054451513	1703	4.437940E-01
Acsf2	190	0.038615889	7536	5.872051E-01
Acsf3	*191*	-0.060876449	1387	3.918201E-01
Acsl1	192	0.220702523	18926	1.686839E-03
Acsl3	193	0.155671036	16015	2.772482E-02
Acsl4	*194*	0.121475782	13751	8.661870E-02
Acsl5	195	0.261827968	19647	1.802906E-04
Acsl6	196	-0.066856562	1139	3.468991E-01
Acsm1	*197*	-0.038406171	3065	5.892384E-01
Acsm2	198	0.204087258	18394	3.747006E-03
Acsm3	199	-0.000841282	5208.5	9.905669E-01
Acsm4	*200*	0.260174631	19626	1.987164E-04
Acsm5	201	0.020434681	6357	7.739526E-01
Acss1	202	0.084100505	10928	2.364113E-01
Acss2	*203*	0.094280302	11659	1.842021E-01
Acss3	204	0.045101237	7948	5.259812E-01
Acta1	205	0.207979148	18521	3.124493E-03
Acta2	*206*	0.165363359	16677	1.928032E-02
Actb	207	0.151368205	15739	3.238745E-02
Actbl2	208		#N/A	1.000000E+00
Actc1	*209*	0.089684867	11320	2.066136E-01
Actg-ps1	210		#N/A	1.000000E+00
Actg1	211	0.12765525	14165	7.164315E-02
Actg2	*212*	0.041421692	7707	5.603179E-01
Actl10	213		#N/A	1.000000E+00
Actl11	214		#N/A	1.000000E+00

Spearman Rank correlation analysis performed between Prdm1 and all-expressed genes within the Meredith RNA-seq dataset. Robust Prdm1–associated genes were identified using a cut-off of $p < 0.0005$.

Table S1, Related to Supplemental Figure 3C. Prdm1 associated genes

Actl6a	*215*	0.097151522	11847	1.711376E-01
Actl6b	216	0.074194755	10154.5	2.964228E-01
Actl7a	217		#N/A	1.000000E+00
ACTL7B	*218*		#N/A	1.000000E+00
Actl7b	219		#N/A	1.000000E+00
Actl9	220		#N/A	1.000000E+00
Actn1	*221*	0.068992293	9775	3.316778E-01
Actn2	222	-0.045026824	2332	5.266649E-01
Actn3	223	0.040707498	7677	5.671053E-01
Actn4	*224*	0.112319549	12880	1.133083E-01
Actr10	225	0.11610963	13401	1.015703E-01
Actr1a	226	0.116347053	13421	1.008682E-01
Actr1b	*227*	0.058376678	9114	4.115912E-01
Actr2	228	0.230795109	19184	1.009087E-03
Actr3	229	0.195136432	18051	5.622550E-03
Actr3b	*230*	0.166184726	16729	1.868014E-02
Actr5	231	0.044957069	7930	5.273062E-01
Actr6	232	0.133500471	14507	5.948489E-02
Actr8	*233*	-0.007873503	4827	9.118924E-01
Actrt1	234		#N/A	1.000000E+00
Actrt2	235		#N/A	1.000000E+00
Actrt3	*236*		#N/A	1.000000E+00
Acvr1	237	0.239649629	19361	6.311626E-04
Acvr1b	238	0.036743098	7419	6.054750E-01
Acvr1c	*239*	0.084765123	10970	2.327147E-01
Acvr2a	240	0.025783121	6860	7.170508E-01
Acvr2b	241	-0.086755146	452	2.218903E-01
Acvrl1	*242*	0.016025705	6126	8.217991E-01
Acy1	243	0.010875243	5820	8.785247E-01
Acy3	244	0.259015365	19617	2.126673E-04
Acyp1	*245*	0.017331992	6191	8.075458E-01
Acyp2	246	0.023412402	6712	7.421019E-01
Ada	247	0.091695071	11502	1.965795E-01
Adad1	*248*	0.115808317	13382	1.024668E-01
Adad2	249		#N/A	1.000000E+00
Adal	250	0.094375689	11667	1.837567E-01
Adam10	*251*	0.075918127	10344	2.853142E-01
Adam11	252	0.055167357	8936	4.378168E-01
Adam12	253	0.028738004	7003	6.862475E-01
Adam15	*254*	-0.086748028	498.5	2.219283E-01
Adam17	255	0.212709937	18683	2.494562E-03
Adam18	256	-0.086750697	472.5	2.219141E-01
Adam19	*257*	0.075678227	10302	2.868436E-01

Spearman Rank correlation analysis performed between Prdm1 and all-expressed genes within the Meredith RNA-seq dataset. Robust Prdm1–associated genes were identified using a cut-off of $p < 0.0005$.

Table S1, Related to Supplemental Figure 3C. Prdm1 associated genes

Adam1a	258		#N/A	1.000000E+00
Adam1b	259		#N/A	1.000000E+00
Adam2	*260*	0.211273316	18622	2.672437E-03
Adam20	261		#N/A	1.000000E+00
Adam21	262	0.213663002	18705	2.382563E-03
Adam22	*263*	0.167266712	16785	1.791432E-02
Adam23	264	0.064356006	9496	3.652718E-01
Adam24	265	0.143605682	15224	4.249002E-02
Adam25	*266*	-0.066855996	1239.5	3.469032E-01
Adam26a	267	-0.054451513	1703	4.437940E-01
Adam26b	268		#N/A	1.000000E+00
Adam28	*269*	0.105527848	12393	1.369645E-01
Adam29	270	0.099271295	12017.5	1.619434E-01
Adam3	271		#N/A	1.000000E+00
Adam30	*272*		#N/A	1.000000E+00
Adam32	273	0.047211432	8097	5.067826E-01
Adam33	274	-0.066855996	1239.5	3.469032E-01
Adam34	*275*	0.121945876	13783	8.539769E-02
Adam39	276		#N/A	1.000000E+00
Adam4	277		#N/A	1.000000E+00
Adam5	*278*	0.046604408	8055.5	5.122676E-01
Adam6a	279		#N/A	1.000000E+00
Adam6b	280		#N/A	1.000000E+00
Adam7	*281*	-0.045438426	2311	5.228889E-01
Adam8	282	0.146792936	15466	3.806047E-02
Adam9	283	0.09370855	11627	1.868889E-01
Adamdec1	*284*	0.01009232	5775	8.872096E-01
Adamts1	285	-0.016731569	4428.5	8.140896E-01
Adamts10	286	-0.130643041	61	6.519749E-02
Adamts12	*287*	0.018237216	6234	7.977054E-01
Adamts13	288	0.006091291	5602	9.317807E-01
Adamts14	289	0.141497652	15051	4.565131E-02
Adamts15	*290*	-0.038406171	3065	5.892384E-01
Adamts16	291	0.115284896	13343	1.040390E-01
Adamts17	292	0.123384827	13871	8.174583E-02
Adamts18	*293*	0.088350842	11230	2.134730E-01
Adamts19	294	0.108219835	12565	1.271719E-01
Adamts2	295	0.135593629	14666	5.556718E-02
Adamts20	*296*	0.011003173	5824	8.771070E-01
Adamts3	297	0.204342692	18402	3.702952E-03
Adamts4	298	0.24174408	19389	5.634139E-04
Adamts5	*299*	0.180043283	17402	1.073778E-02
Adamts6	300	0.199688756	18234	4.583524E-03

Spearman Rank correlation analysis performed between Prdm1 and all-expressed genes within the Meredith RNA-seq dataset. Robust Prdm1–associated genes were identified using a cut-off of $p < 0.0005$.

Table S1, Related to Supplemental Figure 3C. Prdm1 associated genes

Adamts7	301	-0.015030921	4510	8.326931E-01
Adamts8	*302*	-0.044269403	2360.5	5.336493E-01
Adamts9	303	0.022669718	6664	7.500074E-01
Adamtsl1	304	-0.05229822	2156	4.620496E-01
Adamtsl2	*305*	-0.056078575	1484	4.302753E-01
Adamtsl3	306	0.14670973	15463	3.817095E-02
Adamtsl4	307	-0.077393023	832.5	2.760318E-01
Adamtsl5	*308*	0.020875719	6412.5	7.692098E-01
Adap1	309	0.119390807	13617	9.220370E-02
Adap2	310	0.192232974	17921	6.390808E-03
Adar	*311*	0.102238312	12194	1.497026E-01
Adarb1	312	0.12694882	14117	7.324056E-02
Adarb2	313	0.108554419	12586	1.259935E-01
Adat1	*314*	0.02593897	6867	7.154141E-01
Adat2	315	0.167715801	16807.5	1.760456E-02
Adat3	316		#N/A	1.000000E+00
Adc	*317*	-0.082775065	608	2.439058E-01
Adck1	318	0.022879034	6685	7.477766E-01
Adck2	319	0.160167514	16286	2.347838E-02
Adck3	*320*	0.052661259	8518	4.589429E-01
Adck4	321	-0.022449378	4161	7.523578E-01
Adck5	322	-0.040488918	2463	5.691904E-01
Adcy1	*323*	0.053835406	8855	4.489751E-01
Adcy10	324	0.105988958	12420	1.352474E-01
Adcy2	325	0.124826393	13957	7.821447E-02
Adcy3	*326*	0.121032001	13729	8.778416E-02
Adcy4	327	0.246887369	19472.5	4.245310E-04
Adcy5	328	0.119401782	13618	9.217357E-02
Adcy6	*329*	-0.005030928	4933	9.436345E-01
Adcy7	330	0.159409955	16249	2.415191E-02
Adcy8	331	0.093482615	11613	1.879586E-01
Adcy9	*332*	-0.044972925	2335	5.271604E-01
Adcyap1	333	-0.04885054	2219	4.921262E-01
Adcyap1r1	334	0.215109181	18744.5	2.221323E-03
Add1	*335*	0.104201744	12306	1.419959E-01
Add2	336	-0.066858259	1029.5	3.468868E-01
Add3	337	0.191165957	17882	6.695881E-03
Adgb	*338*	0.060930302	9257	3.914006E-01
Adh1	339	0.017316047	6190	8.077194E-01
Adh4	340	-0.043687291	2385	5.390486E-01
Adh5	*341*	0.155785991	16024	2.760858E-02
Adh6-ps1	342	0.0711002	9919	3.170817E-01
Adh6a	343	0.02175313	6571	7.597999E-01

Spearman Rank correlation analysis performed between Prdm1 and all-expressed genes within the Meredith RNA-seq dataset. Robust Prdm1–associated genes were identified using a cut-off of $p < 0.0005$.

Table S1, Related to Supplemental Figure 3C. Prdm1 associated genes

Adh6b	*344*	-0.077393023	832.5	2.760318E-01
Adh7	345	0.026040072	6873	7.143531E-01
Adhfe1	346	0.072449949	10029	3.079584E-01
Adi1	*347*	0.182727137	17538	9.603290E-03
Adig	348	0.0815845	10706	2.507776E-01
Adipoq	349	0.246470512	19461	4.344787E-04
Adipor1	*350*	0.111568608	12823	1.157552E-01
Adipor2	351	0.1829649	17550	9.508095E-03
Adk	352	0.16061878	16372	2.308492E-02
Adm	*353*	0.227339197	19089	1.206217E-03
Adm2	354	0.057208523	9046	4.210282E-01
Adnp	355	0.107130827	12504	1.310661E-01
Adnp2	*356*	0.123879718	13897	8.051932E-02
Ado	357	0.149827707	15639	3.421148E-02
Adora1	358	0.137589224	14796	5.203317E-02
Adora2a	*359*	-0.032445878	3830.5	6.483195E-01
Adora2b	360	-0.066855996	1239.5	3.469032E-01
Adora3	361	0.020413742	6345	7.741780E-01
Adpgk	*362*	0.05459834	8903	4.425642E-01
Adprh	363	0.152729447	15806	3.084513E-02
Adprhl1	364	0.06357024	9441	3.711674E-01
Adprhl2	*365*	-0.018739903	4296	7.922545E-01
Adprm	366	-0.036177932	3680	6.110377E-01
Adra1a	367	-0.085531231	561	2.285044E-01
Adra1b	*368*	0.328561388	19984	2.034458E-06
Adra1d	369	0.197662879	18146	5.022481E-03
Adra2a	370		#N/A	1.000000E+00
Adra2b	*371*	0.107077481	12500	1.312592E-01
Adra2c	372	-0.054450825	1984	4.437997E-01
Adrb1	373	0.083525677	10882	2.396415E-01
Adrb2	*374*	0.031280337	7142	6.601503E-01
Adrb3	375	-0.038406171	3065	5.892384E-01
Adrbk1	376	0.153697328	15874	2.978696E-02
Adrbk2	*377*	0.096506541	11809	1.740106E-01
Adrm1	378	0.203948746	18388	3.771092E-03
Adsl	379	0.115296211	13346	1.040049E-01
Adss	*380*	-0.00028184	5275	9.968397E-01
Adssl1	381	0.185292275	17649	8.619460E-03
Adtrp	382	0.125488968	14003	7.663325E-02
Aebp1	*383*		#N/A	1.000000E+00
Aebp2	384	-0.007726676	4831	9.135290E-01
Aen	385	-0.055072524	1515	4.386060E-01
Aes	*386*	0.043661668	7842	5.392869E-01

Spearman Rank correlation analysis performed between Prdm1 and all-expressed genes within the Meredith RNA-seq dataset. Robust Prdm1–associated genes were identified using a cut-off of $p < 0.0005$.

Table S1, Related to Supplemental Figure 3C. Prdm1 associated genes

AF067061	387	-0.017417495	4389.5	8.066150E-01
AF067063	388		#N/A	1.000000E+00
AF251705	*389*	-0.003357792	5012	9.623627E-01
AF357341	390		#N/A	1.000000E+00
AF357355	391		#N/A	1.000000E+00
AF357399	*392*		#N/A	1.000000E+00
AF357425	393		#N/A	1.000000E+00
AF357428	394		#N/A	1.000000E+00
AF366264	*395*	-0.047062893	2259	5.081220E-01
AF529169	396	0.04876715	8202	4.928664E-01
Afap1	397	0.009312019	5738	8.958788E-01
Afap1l1	*398*	-0.011157951	4698	8.753922E-01
Afap1l2	399	-0.03558135	3699	6.169338E-01
Aff1	400	0.213371635	18695	2.416309E-03
Aff2	*401*	0.130207724	14308	6.610596E-02
Aff3	402	0.121877001	13781	8.557572E-02
Aff4	403	0.135831283	14684	5.513618E-02
Afg3l1	*404*	0.160505863	16366	2.318283E-02
Afg3l2	405	0.137307018	14774	5.252131E-02
Afm	406	-0.077397959	691.5	2.760011E-01
Afmid	*407*	-0.013382316	4602	8.508165E-01
Afp	408	-0.038406171	3065	5.892384E-01
Aftph	409	0.117966359	13524	9.618174E-02
Aga	*410*	0.2041056	18396	3.743827E-03
Agap1	411	0.141822662	15071	4.515145E-02
Agap2	412	0.024734813	6801	7.280928E-01
Agap3	*413*	-0.046255532	2281	5.154339E-01
Agbl1	414	-0.008337476	4808	9.067232E-01
Agbl2	415	-0.000841282	5208.5	9.905669E-01
Agbl3	*416*	-0.002638095	5045	9.704256E-01
Agbl4	417	0.174957697	17163	1.321548E-02
Agbl5	418	0.099998876	12066	1.588744E-01
Ager	*419*		#N/A	1.000000E+00
Agfg1	420	0.065411754	9553	3.574424E-01
Agfg2	421	-0.02544065	4088	7.206518E-01
Aggf1	*422*	0.266507392	19690	1.364139E-04
Agk	423	0.199569993	18230	4.608273E-03
Agl	424	0.065678372	9570	3.554818E-01
Agmat	*425*	0.121010592	13727	8.784070E-02
Agmo	426	0.229843999	19146	1.060157E-03
Ago1	427	0.094894663	11695	1.813468E-01
Ago2	*428*	0.1077177	12536	1.289562E-01
Ago3	429	0.040364894	7654	5.703752E-01

Spearman Rank correlation analysis performed between Prdm1 and all-expressed genes within the Meredith RNA-seq dataset. Robust Prdm1–associated genes were identified using a cut-off of $p < 0.0005$.

Table S1, Related to Supplemental Figure 3C. Prdm1 associated genes

Ago4	430	0.201540223	18311	4.212846E-03
Agpat1	*431*	0.197237655	18131	5.119271E-03
Agpat2	432	-0.011031722	4702	8.767906E-01
Agpat3	433	0.06875002	9756	3.333826E-01
Agpat4	*434*	0.00797176	5678	9.107974E-01
Agpat5	435	0.199367576	18219	4.650731E-03
Agpat6	436	0.100015336	12068	1.588055E-01
Agpat9	*437*	0.139739531	14933	4.843610E-02
Agphd1	438	0.19987834	18240	4.544264E-03
Agps	439	0.145475337	15378	3.984197E-02
Agr2	*440*	-0.038406171	3065	5.892384E-01
Agr3	441	-0.038406171	3065	5.892384E-01
Agrn	442	0.034723161	7323	6.254585E-01
Agrp	*443*	-0.054450825	1984	4.437997E-01
Agt	444	-0.054450825	1984	4.437997E-01
Agtpbp1	445	0.241783575	19392	5.622035E-04
Agtr1a	*446*	-0.038406171	3065	5.892384E-01
Agtr1b	447	0.067056391	9648	3.454565E-01
Agtr2	448	0.199230252	18211	4.679735E-03
Agtrap	*449*	0.087935765	11200	2.156402E-01
Agxt	450	-0.038406171	3065	5.892384E-01
Agxt2	451		#N/A	1.000000E+00
Agxt2l1	*452*	0.064441598	9502	3.646331E-01
Agxt2l2	453	0.08393315	10916	2.373485E-01
Ahctf1	454	0.018243265	6235	7.976397E-01
Ahcy	*455*	0.069797176	9832	3.260544E-01
Ahcyl1	456	0.148551148	15564	3.578832E-02
Ahcyl2	457	0.023599152	6726	7.401183E-01
Ahdc1	*458*	0.133912753	14546	5.869575E-02
Ahi1	459	0.083594498	10886	2.392531E-01
Ahnak	460	-0.026139281	4062	7.133125E-01
Ahnak2	*461*	0.08184645	10733	2.492543E-01
Ahr	462	0.193148651	17954	6.138966E-03
Ahrr	463	0.014915162	6067	8.339629E-01
Ahsa1	*464*	-0.000747094	5231	9.916229E-01
Ahsa2	465	0.013216141	5945	8.526478E-01
Ahsg	466	-0.038406171	3065	5.892384E-01
AI118078	*467*	-0.015647044	4491	8.259420E-01
AI182371	468	0.113548679	13094	1.093908E-01
AI314180	469	0.077570558	10447	2.749284E-01
AI314831	*470*	0.077242587	10424.5	2.769691E-01
AI317395	471	-0.030877746	3889	6.642567E-01
AI413582	472	0.160756532	16378	2.296594E-02

Spearman Rank correlation analysis performed between Prdm1 and all-expressed genes within the Meredith RNA-seq dataset. Robust Prdm1–associated genes were identified using a cut-off of $p < 0.0005$.

Table S1, Related to Supplemental Figure 3C. Prdm1 associated genes

AI427809	*473*	0.03981323	7621.5	5.756589E-01
AI429214	474	0.12732284	14141	7.239125E-02
AI462493	475	0.040294793	7649	5.710453E-01
AI464131	*476*	0.098091152	11920	1.670153E-01
AI467606	477	0.24538405	19446	4.614294E-04
AI480526	478	0.069494984	9816	3.281584E-01
AI481877	*479*	0.072353696	10019	3.086032E-01
AI504432	480	0.256599734	19588	2.447189E-04
AI507597	481	-0.054450825	1984	4.437997E-01
AI593442	*482*	0.05990774	9205	3.994130E-01
AI597468	483	0.261731816	19646	1.813170E-04
AI597479	484	-0.148436306	29	3.593314E-02
AI606181	*485*	0.108608001	12596	1.258056E-01
AI606473	486	0.143605682	15224	4.249002E-02
AI607873	487	-0.077394997	736.5	2.760195E-01
AI646519	*488*	-0.054451513	1703	4.437940E-01
AI661453	489	-0.103159953	253.5	1.460465E-01
AI662270	490	0.232859978	19227	9.058999E-04
AI747448	*491*	-0.086746694	532	2.219355E-01
AI836003	492	0.063402353	9424	3.724346E-01
AI837181	493	0.065817472	9577	3.544617E-01
AI838599	*494*		#N/A	1.000000E+00
AI846148	495	0.004019901	5517	9.549484E-01
AI847159	496	-0.066856562	1139	3.468991E-01
AI848285	*497*		#N/A	1.000000E+00
AI854703	498		#N/A	1.000000E+00
AI987944	499	0.133606359	14518	5.928138E-02
Aicda	*500*	0.222602947	18973	1.533881E-03
Aida	501	0.223258919	18993	1.484101E-03
Aif1	502	0.058503965	9126	4.105705E-01
Aif1l	*503*	0.050265405	8323	4.796588E-01
Aifm1	504	0.144363593	15328	4.139929E-02
Aifm2	505	0.072186195	10008	3.097275E-01
Aifm3	*506*	0.013541198	5972	8.490663E-01
Aig1	507	-0.008438868	4805	9.055941E-01
Aim1	508	0.040000479	7641	5.738629E-01
Aim1l	*509*	0.11651758	13435	1.003663E-01
Aim2	510	0.145011854	15354	4.048511E-02
Aimp1	511	0.121655811	13769	8.614946E-02
Aimp2	*512*	-0.066177309	1299	3.518311E-01
Aip	513	0.284921292	19834	4.328243E-05
AIP	514		#N/A	1.000000E+00
Aipl1	*515*	-0.038406171	3065	5.892384E-01

Spearman Rank correlation analysis performed between Prdm1 and all-expressed genes within the Meredith RNA-seq dataset. Robust Prdm1–associated genes were identified using a cut-off of $p < 0.0005$.

Table S1, Related to Supplemental Figure 3C. Prdm1 associated genes

Aire	516	0.07043672	9869	3.216302E-01
Airn	517	0.196898933	18118	5.197566E-03
Ajap1	*518*	0.019629921	6308	7.826283E-01
Ajuba	519	0.061992947	9330	3.831775E-01
AK010878	520	0.128397277	14208	6.999588E-02
Ak1	*521*	0.009184847	5736	8.972928E-01
AK129341	522	0.047914325	8143	5.004698E-01
AK157302	523		#N/A	1.000000E+00
Ak2	*524*	0.216374786	18797	2.088427E-03
Ak3	525	0.041503142	7711	5.595463E-01
Ak3l2-ps	526		#N/A	1.000000E+00
Ak4	*527*	0.121175947	13738	8.740476E-02
Ak5	528	0.006944849	5628	9.222495E-01
Ak7	529	0.118890592	13577	9.358543E-02
Ak8	*530*	0.098647291	11966	1.646106E-01
Akap1	531	0.19144617	17892	6.614532E-03
Akap10	532	0.143008715	15142	4.336603E-02
Akap11	*533*	0.052533622	8504	4.600338E-01
Akap12	534	0.050628318	8352	4.764887E-01
Akap13	535	0.132130858	14419	6.216940E-02
Akap14	*536*	-0.103163006	242.5	1.460345E-01
Akap17b	537	0.136036501	14705	5.476623E-02
Akap2	538	0.071783182	9982	3.124435E-01
Akap3	*539*	-0.038406171	3065	5.892384E-01
Akap4	540		#N/A	1.000000E+00
Akap5	541	-0.034527868	3726	6.274055E-01
Akap6	*542*	0.220446366	18915	1.708488E-03
Akap7	543	0.06471122	9520	3.626257E-01
Akap8	544	0.18779612	17755	7.746444E-03
Akap8l	*545*	-0.052014189	2161	4.644883E-01
Akap9	546	0.118514719	13558	9.463450E-02
Akd1	547	0.023709146	6733	7.389508E-01
Akip1	*548*	0.151818254	15754	3.187044E-02
Akirin1	549	-0.01162717	4682	8.701973E-01
Akirin2	550	-0.022948406	4150	7.470378E-01
Akna	*551*	0.195767332	18077	5.466975E-03
Aknad1	552	0.055120216	8933	4.382090E-01
Akp3	553		#N/A	1.000000E+00
Akr1a1	*554*	0.105763192	12407	1.360860E-01
Akr1b10	555	0.215587504	18781	2.170218E-03
Akr1b3	556	0.163353707	16563	2.081978E-02
Akr1b7	*557*	0.08865127	11248	2.119142E-01
Akr1b8	558	-0.077394997	736.5	2.760195E-01

Spearman Rank correlation analysis performed between Prdm1 and all-expressed genes within the Meredith RNA-seq dataset. Robust Prdm1–associated genes were identified using a cut-off of $p < 0.0005$.

Table S1, Related to Supplemental Figure 3C. Prdm1 associated genes

Akr1c12	559	-0.101680573	264	1.519484E-01
Akr1c13	*560*	0.017067014	6173	8.104321E-01
Akr1c14	561	-0.038406171	3065	5.892384E-01
Akr1c18	562	0.048457274	8181	4.956220E-01
Akr1c19	*563*	0.209390823	18562	2.922914E-03
Akr1c20	564	0.053267784	8685	4.537786E-01
Akr1c21	565	-0.038406171	3065	5.892384E-01
Akr1c6	*566*	-0.054450825	1984	4.437997E-01
Akr1cl	567	-0.010800165	4710	8.793569E-01
Akr1d1	568	0.135543457	14664	5.565853E-02
Akr1e1	*569*	-0.010323343	4733	8.846454E-01
Akr7a5	570	0.142639242	15129	4.391576E-02
Akt1	571	0.111870973	12841	1.147650E-01
Akt1s1	*572*	-0.066855996	1239.5	3.469032E-01
Akt2	573	0.084231689	10937	2.356784E-01
Akt2-ps	574		#N/A	1.000000E+00
Akt3	*575*	0.081330042	10688	2.522635E-01
Aktip	576	0.078191781	10478	2.710911E-01
AL450399.1	577	0.160985049	16395	2.276975E-02
AL513014.1	*578*	-0.038406171	3065	5.892384E-01
AL589650.1	579		#N/A	1.000000E+00
AL591113.1	580	0.052728522	8550.5	4.583686E-01
AL591143.1	*581*		#N/A	1.000000E+00
AL591207.1	582		#N/A	1.000000E+00
AL592169.1	583		#N/A	1.000000E+00
AL593853.1	*584*	0.021316405	6469	7.644793E-01
AL596215.1	585		#N/A	1.000000E+00
AL596386.1	586		#N/A	1.000000E+00
AL603843.1	*587*	-0.015468584	4494	8.278961E-01
AL606528.1	588	0.122799419	13832	8.321606E-02
AL606962.1	589	0.223345343	18994	1.477654E-03
AL607072.1	*590*		#N/A	1.000000E+00
AL607108.1	591		#N/A	1.000000E+00
AL607123.1	592	0.263077708	19659	1.674345E-04
AL607127.1	*593*	0.12046769	13690.5	8.928425E-02
AL627077.1	594	-0.027278993	4025	7.013962E-01
AL627077.2	595		#N/A	1.000000E+00
AL627104.1	*596*		#N/A	1.000000E+00
AL627184.1	597		#N/A	1.000000E+00
AL627304.1	598	0.003914976	5512	9.561231E-01
AL645470.1	*599*	-0.038406171	3065	5.892384E-01
AL645646.1	600	0.049599398	8261	4.855061E-01
AL645861.1	601		#N/A	1.000000E+00

Spearman Rank correlation analysis performed between Prdm1 and all-expressed genes within the Meredith RNA-seq dataset. Robust Prdm1–associated genes were identified using a cut-off of $p < 0.0005$.

Table S1, Related to Supplemental Figure 3C. Prdm1 associated genes

AL646002.1	*602*	-0.002130185	5069	9.761176E-01
AL662835.1	603	0.09963975	12036	1.603837E-01
AL662835.2	604	0.089528701	11312	2.074082E-01
AL663030.1	*605*	0.021316405	6469	7.644793E-01
AL669855.1	606	-0.066855996	1239.5	3.469032E-01
AL669897.1	607	-0.038406171	3065	5.892384E-01
AL669948.1	*608*	-0.038406171	3065	5.892384E-01
AL670236.1	609		#N/A	1.000000E+00
AL671335.1	610		#N/A	1.000000E+00
AL671873.1	*611*	-0.003389066	5010	9.620124E-01
AL672219.1	612	0.020875719	6412.5	7.692098E-01
AL672246.1	613	-0.030072101	3922	6.725042E-01
AL672268.1	*614*	0.156129434	16056.5	2.726377E-02
AL672276.1	615	0.138851285	14873	4.989603E-02
AL672276.2	616		#N/A	1.000000E+00
AL672278.1	*617*		#N/A	1.000000E+00
AL683799.1	618		#N/A	1.000000E+00
AL691450.1	619	-0.054450825	1984	4.437997E-01
AL691450.2	*620*	0.101593108	12155	1.523029E-01
AL691505.1	621	0.087339186	11152	2.187825E-01
AL713863.1	622	-0.038406171	3065	5.892384E-01
AL714024.1	*623*	0.113548679	13094	1.093908E-01
AL731663.1	624	0.159944123	16275	2.367529E-02
AL731663.2	625	0.222359579	18964	1.552735E-03
AL731670.1	*626*	0.105883671	12413	1.356380E-01
AL731822.1	627	-0.077394997	736.5	2.760195E-01
AL732311.1	628	0.032722952	7220	6.455198E-01
AL732403.1	*629*	0.072800557	10049	3.056171E-01
AL732490.1	630	-0.038406171	3065	5.892384E-01
AL732527.1	631	-0.054451513	1703	4.437940E-01
AL732557.1	*632*	-0.066856562	1139	3.468991E-01
AL732590.1	633		#N/A	1.000000E+00
AL772167.1	634	-0.038406171	3065	5.892384E-01
AL772170.1	*635*	0.02148887	6552	7.626303E-01
AL772190.1	636		#N/A	1.000000E+00
AL772299.1	637	-0.054450825	1984	4.437997E-01
AL772401.1	*638*		#N/A	1.000000E+00
AL805969.1	639	0.160304018	16323	2.335876E-02
AL806526.1	640		#N/A	1.000000E+00
AL807833.1	*641*	0.158268736	16190	2.519790E-02
AL831764.1	642	0.183242283	17560	9.398092E-03
AL833775.1	643		#N/A	1.000000E+00
AL844180.1	*644*		#N/A	1.000000E+00

Spearman Rank correlation analysis performed between Prdm1 and all-expressed genes within the Meredith RNA-seq dataset. Robust Prdm1–associated genes were identified using a cut-off of $p < 0.0005$.

Table S1, Related to Supplemental Figure 3C. Prdm1 associated genes

AL844576.1	645		#N/A	1.000000E+00
AL844581.1	646	0.097397819	11865.5	1.700498E-01
AL844859.1	*647*		#N/A	1.000000E+00
AL845162.1	648		#N/A	1.000000E+00
AL845174.1	649		#N/A	1.000000E+00
AL845483.1	*650*	-0.054450825	1984	4.437997E-01
AL928605.1	651		#N/A	1.000000E+00
AL928605.2	652		#N/A	1.000000E+00
AL928696.1	*653*		#N/A	1.000000E+00
AL928963.1	654	0.201443968	18306	4.231431E-03
AL929411.1	655		#N/A	1.000000E+00
AL929563.1	*656*	0.05880045	9147	4.081988E-01
AL935177.1	657	0.154680387	15962	2.874406E-02
Alad	658	0.054944529	8921	4.396724E-01
Alas1	*659*	0.078692162	10512	2.680267E-01
Alas2	660	-0.099231176	284	1.621139E-01
Alb	661	-0.001606757	5107.5	9.819849E-01
Alcam	*662*	0.106549589	12463	1.331819E-01
Aldh16a1	663	-0.075674164	872	2.868695E-01
Aldh18a1	664	0.248197663	19485	3.946203E-04
Aldh1a1	*665*	0.112251061	12870	1.135298E-01
Aldh1a2	666	0.09917863	12009	1.623374E-01
Aldh1a3	667	0.04141128	7706	5.604165E-01
Aldh1a7	*668*	-0.008285905	4811	9.072976E-01
Aldh1b1	669	0.148464033	15559	3.589813E-02
Aldh1l1	670	-0.051989742	2162	4.646985E-01
Aldh1l2	*671*	0.182093519	17487	9.861109E-03
Aldh2	672	0.101857916	12171	1.512316E-01
Aldh3a1	673	-0.001475162	5119	9.834601E-01
Aldh3a2	*674*	-0.084204691	586	2.358291E-01
Aldh3b1	675	-0.036783137	3666	6.050818E-01
Aldh3b2	676	0.022244754	6647	7.545427E-01
Aldh4a1	*677*		#N/A	1.000000E+00
Aldh5a1	678	0.092934719	11579	1.905710E-01
Aldh6a1	679	0.166612164	16754	1.837427E-02
Aldh7a1	*680*	-0.154115794	18	2.933914E-02
Aldh8a1	681	-0.030875624	3897.5	6.642784E-01
Aldh9a1	682	0.092001675	11518	1.950807E-01
Aldoa	*683*	0.136646104	14739	5.367938E-02
Aldoart1	684	0.250884413	19519	3.393021E-04
Aldoart2	685	0.020434681	6357	7.739526E-01
Aldob	*686*	0.154892479	15973	2.852319E-02
Aldoc	687	0.034466667	7311	6.280161E-01

Spearman Rank correlation analysis performed between Prdm1 and all-expressed genes within the Meredith RNA-seq dataset. Robust Prdm1–associated genes were identified using a cut-off of $p < 0.0005$.

Table S1, Related to Supplemental Figure 3C. Prdm1 associated genes

Alg1	688	0.085991798	11052	2.259993E-01
Alg10b	*689*	-0.05506313	1518.5	4.386842E-01
Alg11	690	0.229278654	19128	1.091627E-03
Alg12	691	0.022816863	6681	7.484390E-01
Alg13	*692*	0.181633947	17467	1.005192E-02
Alg14	693	0.144157669	15319	4.169329E-02
Alg2	694	0.024310107	6774	7.325824E-01
Alg3	*695*	0.23873124	19343	6.631780E-04
Alg5	696	0.129414967	14267	6.778695E-02
Alg6	697	0.130510536	14322	6.547293E-02
Alg8	*698*	0.001068313	5361	9.880214E-01
Alg9	699	0.066562048	9625	3.490322E-01
Alk	700	-0.062702101	1367	3.777488E-01
Alkbh1	*701*	0.217483501	18834	1.977998E-03
Alkbh2	702	0.076157716	10358	2.837922E-01
Alkbh3	703	0.085027133	10991	2.312687E-01
Alkbh4	*704*	0.056368261	9002	4.278935E-01
Alkbh5	705	0.118535144	13559	9.457726E-02
Alkbh6	706	-0.052628544	2149	4.592224E-01
Alkbh7	*707*	-0.003619159	5000	9.594355E-01
Alkbh8	708	-0.127030414	75	7.305459E-02
Allc	709	-0.019133303	4265.5	7.879956E-01
Alms1	*710*	0.084926043	10985	2.318258E-01
Alms1-ps1	711	-0.066856562	1139	3.468991E-01
Alms1-ps2	712	0.097178565	11852	1.710179E-01
Alox12	*713*	0.041545787	7714	5.591425E-01
Alox12b	714	-0.021550603	4187	7.619688E-01
Alox12e	715	0.150779331	15697	3.307470E-02
Alox15	*716*	0.112135843	12863	1.139031E-01
Alox5	717	0.039811625	7620	5.756743E-01
Alox5ap	718	0.062763821	9377	3.772786E-01
Alox8	*719*	0.147807328	15526	3.673515E-02
Aloxe3	720	-0.037368243	3651	5.993484E-01
Alpi	721	-0.077393517	797	2.760287E-01
Alpk1	*722*	0.140217334	14969	4.766562E-02
Alpk2	723	0.210247158	18581	2.806449E-03
Alpk3	724	0.258310928	19603	2.215849E-04
Alpl	*725*	0.009688855	5760	8.916905E-01
Alppl2	726		#N/A	1.000000E+00
Als2	727	0.032235362	7194	6.504500E-01
Als2cl	*728*	0.110696398	12771	1.186487E-01
Als2cr11	729	0.135474972	14662	5.578341E-02
Als2cr12	730	-0.057249275	1455	4.206968E-01

Spearman Rank correlation analysis performed between Prdm1 and all-expressed genes within the Meredith RNA-seq dataset. Robust Prdm1–associated genes were identified using a cut-off of $p < 0.0005$.

Table S1, Related to Supplemental Figure 3C. Prdm1 associated genes

Alx1	*731*	0.123243342	13860	8.209924E-02
Alx3	732		#N/A	1.000000E+00
Alx4	733	0.092490457	11549	1.927087E-01
Alyref	*734*	0.156274543	16069	2.711919E-02
Alyref2	735	0.090555649	11388	2.022226E-01
Amacr	736	0.043944448	7860	5.366600E-01
Ambn	*737*	0.022198129	6607.5	7.550408E-01
Ambp	738	-0.016728755	4450	8.141203E-01
Ambra1	739	0.150011937	15652	3.398885E-02
Amd-ps1	*740*		#N/A	1.000000E+00
Amd-ps3	741		#N/A	1.000000E+00
Amd-ps4	742		#N/A	1.000000E+00
Amd1	*743*	0.192675081	17939	6.268084E-03
Amd2	744	-0.086750697	472.5	2.219141E-01
Amdhd1	745	0.166413449	16745	1.851592E-02
Amdhd2	*746*	-0.087583968	437	2.174893E-01
Amelx	747	-0.038406171	3065	5.892384E-01
Amer1	748	0.143683381	15289	4.237711E-02
Amer2	*749*	0.289234896	19860	3.268655E-05
Amer3	750		#N/A	1.000000E+00
Amfr	751	0.111313001	12809	1.165974E-01
Amh	*752*		#N/A	1.000000E+00
Amhr2	753	0.059166757	9165	4.052797E-01
Amica1	754	0.094357383	11665	1.838421E-01
Amigo1	*755*	0.002381149	5437	9.733050E-01
Amigo2	756	0.131828286	14396	6.277568E-02
Amigo3	757		#N/A	1.000000E+00
Ammecr1	*758*	0.154943389	15974	2.847039E-02
Ammecr1l	759	0.033096553	7245	6.417527E-01
Amn	760	-0.117569446	123	9.731414E-02
Amn1	*761*	0.072317122	10012	3.088485E-01
Amot	762	0.140523274	14988	4.717766E-02
Amotl1	763	0.086012784	11054	2.258856E-01
Amotl2	*764*	0.025861669	6864	7.162258E-01
Ampd1	765	0.063365727	9418	3.727114E-01
Ampd2	766	0.105830267	12410	1.358365E-01
Ampd3	*767*	0.089100985	11278	2.095960E-01
Amph	768	0.103971537	12295	1.428836E-01
Amt	769		#N/A	1.000000E+00
Amtn	*770*	0.052658175	8517	4.589692E-01
Amy1	771	0.149141002	15601	3.505223E-02
Amy2-ps1	772	0.053805701	8820	4.492258E-01
Amy2a1	*773*		#N/A	1.000000E+00

Spearman Rank correlation analysis performed between Prdm1 and all-expressed genes within the Meredith RNA-seq dataset. Robust Prdm1–associated genes were identified using a cut-off of $p < 0.0005$.

Table S1, Related to Supplemental Figure 3C. Prdm1 associated genes

Amy2a2	774		#N/A	1.000000E+00
Amy2a3	775		#N/A	1.000000E+00
Amy2a4	776		#N/A	1.000000E+00
Amy2a5	777		#N/A	1.000000E+00
Amy2b	778	-0.054451513	1703	4.437940E-01
Amz1	779	0.154047744	15892	2.941157E-02
Amz2	780	0.060240903	9224	3.967917E-01
Anapc1	781	0.090082762	11352	2.045987E-01
Anapc10	782	0.080994279	10658	2.542335E-01
Anapc11	783	0.151277045	15732	3.249304E-02
Anapc13	784	0.212128388	18654	2.565230E-03
Anapc15	785	0.04930192	8245	4.881301E-01
Anapc15-ps	786		#N/A	1.000000E+00
Anapc16	787	0.133642041	14521	5.921293E-02
Anapc2	788	0.276803323	19773	7.252019E-05
Anapc4	789	0.163385918	16568	2.079429E-02
Anapc5	790	0.184881662	17629	8.770672E-03
Anapc7	791	-0.02818312	3991	6.919944E-01
Ang	792		#N/A	1.000000E+00
Ang-ps1	793		#N/A	1.000000E+00
Ang-ps2	794		#N/A	1.000000E+00
Ang-ps3	795		#N/A	1.000000E+00
Ang2	796	-0.09526662	364	1.796340E-01
Ang4	797	0.021238051	6453	7.653197E-01
Ang5	798	-0.054451513	1703	4.437940E-01
Ang6	799		#N/A	1.000000E+00
Angel1	800	0.035531809	7352	6.174245E-01
Angel2	801	0.257816889	19599	2.280456E-04
Angpt1	802	0.0698322	9834	3.258111E-01
Angpt2	803		#N/A	1.000000E+00
Angpt4	804	0.216412436	18798	2.084587E-03
Angptl1	805	-0.066855996	1239.5	3.469032E-01
Angptl2	806	0.220350357	18910	1.716668E-03
Angptl3	807		#N/A	1.000000E+00
Angptl4	808	-0.038406171	3065	5.892384E-01
Angptl6	809	-0.084793503	576	2.325578E-01
Angptl7	810	0.021757451	6575	7.597536E-01
Ank	811	0.007497504	5654	9.160841E-01
Ank1	812	0.210716169	18600	2.744460E-03
Ank2	813	0.057491027	9061	4.187345E-01
Ank3	814	0.108373982	12574	1.266279E-01
Ankar	815	-0.020022635	4238	7.783912E-01
Ankdd1a	816		#N/A	1.000000E+00

Spearman Rank correlation analysis performed between Prdm1 and all-expressed genes within the Meredith RNA-seq dataset. Robust Prdm1–associated genes were identified using a cut-off of $p < 0.0005$.

Table S1, Related to Supplemental Figure 3C. Prdm1 associated genes

Ankdd1b	817	0.231869328	19207	9.541294E-04
Ankef1	*818*	-0.054451513	1703	4.437940E-01
Ankfn1	819	0.058116827	9096	4.136796E-01
Ankfy1	820	0.18605507	17684	8.344682E-03
Ankhd1	*821*	0.16235732	16490	2.162170E-02
Ankib1	822	0.052880387	8586	4.570733E-01
Ankk1	823	0.086412184	11087.5	2.237298E-01
Ankle1	*824*	0.154082975	15897	2.937405E-02
Ankle2	825	0.166087741	16709	1.875015E-02
Ankmy1	826		#N/A	1.000000E+00
Ankmy2	*827*	0.099941819	12064	1.591135E-01
Ankra2	828	-0.016964345	4415	8.115511E-01
Ankrd1	829	0.2569728	19590	2.394915E-04
Ankrd10	*830*	0.162032266	16469	2.188903E-02
Ankrd11	831	0.071455685	9948	3.146620E-01
Ankrd12	832	0.007115946	5631	9.203402E-01
Ankrd13a	*833*	0.155820114	16027	2.757416E-02
Ankrd13b	834	0.187788639	17754	7.748931E-03
Ankrd13c	835	0.241672874	19386	5.656022E-04
Ankrd13d	*836*	-0.054451513	1703	4.437940E-01
Ankrd16	837	-0.038413326	2505	5.891689E-01
Ankrd17	838	0.204257219	18399	3.717641E-03
Ankrd2	*839*	0.053805701	8820	4.492258E-01
Ankrd22	840	0.130847034	14341	6.477531E-02
Ankrd23	841	0.053805701	8820	4.492258E-01
Ankrd24	*842*	-0.044268912	2363.5	5.336539E-01
Ankrd26	843	0.07007732	9845	3.241116E-01
Ankrd27	844	-0.002765889	5041	9.689937E-01
Ankrd28	*845*	0.07867151	10509	2.681527E-01
Ankrd29	846	0.184056196	17593	9.081792E-03
Ankrd32	847	0.036040268	7384	6.123960E-01
Ankrd33	*848*		#N/A	1.000000E+00
Ankrd33b	849	0.052775194	8581	4.579703E-01
Ankrd34a	850		#N/A	1.000000E+00
Ankrd34b	*851*	-0.054451513	1703	4.437940E-01
Ankrd34c	852		#N/A	1.000000E+00
Ankrd35	853	0.16123772	16436	2.255450E-02
Ankrd36	*854*	0.236775049	19300	7.364263E-04
Ankrd37	855	-0.09908461	285	1.627379E-01
Ankrd39	856	0.068354733	9729	3.361762E-01
Ankrd40	*857*	0.205753865	18447	3.467886E-03
Ankrd42	858	0.220992936	18932	1.662597E-03
Ankrd44	859	0.142063028	15084	4.478473E-02

Spearman Rank correlation analysis performed between Prdm1 and all-expressed genes within the Meredith RNA-seq dataset. Robust Prdm1–associated genes were identified using a cut-off of $p < 0.0005$.

Table S1, Related to Supplemental Figure 3C. Prdm1 associated genes

Ankrd45	*860*	0.180439681	17426	1.056311E-02
Ankrd46	861	0.019257005	6294	7.866577E-01
Ankrd49	862	-0.051400985	2174	4.697774E-01
Ankrd50	*863*	0.111917132	12846	1.146145E-01
Ankrd52	864	0.180061297	17404	1.072979E-02
Ankrd53	865		#N/A	1.000000E+00
Ankrd54	*866*	0.053753616	8786	4.496655E-01
Ankrd55	867	0.07682649	10405	2.795729E-01
Ankrd6	868	0.105292537	12379	1.378472E-01
Ankrd60	*869*	0.102215783	12193	1.497928E-01
Ankrd61	870		#N/A	1.000000E+00
Ankrd63	871	-0.038406171	3065	5.892384E-01
Ankrd65	*872*	0.113548679	13094	1.093908E-01
Ankrd66	873		#N/A	1.000000E+00
Ankrd7	874	-0.038406171	3065	5.892384E-01
Ankrd9	*875*	0.020632245	6380	7.718270E-01
Anks1	876	0.037779628	7475	5.953319E-01
Anks1b	877	0.073752835	10113	2.993170E-01
Anks3	*878*	0.032464276	7202	6.481334E-01
Anks4b	879	0.05165629	8444	4.675713E-01
Anks6	880	-0.077393517	797	2.760287E-01
Ankzf1	*881*	0.077011552	10414	2.784128E-01
Anln	882	0.246810387	19470	4.263520E-04
Ano1	883	-0.021283276	4199	7.648346E-01
Ano10	*884*	0.298818478	19902	1.723024E-05
Ano2	885	-0.120034261	102	9.045027E-02
Ano3	886	0.069340294	9800	3.292389E-01
Ano4	*887*	0.10900524	12624	1.244192E-01
Ano5	888	-0.032907185	3788	6.436610E-01
Ano6	889	0.117413304	13485	9.776251E-02
Ano7	*890*		#N/A	1.000000E+00
Ano8	891	-0.020779655	4213	7.702421E-01
Ano9	892	-0.038406171	3065	5.892384E-01
Anp32-ps	*893*		#N/A	1.000000E+00
Anp32a	894	0.010262319	5783	8.853226E-01
Anp32b	895	0.16577496	16690	1.897750E-02
Anp32b-ps1	*896*		#N/A	1.000000E+00
Anp32e	897	0.311577775	19943	7.086974E-06
Anpep	898	-0.090575695	411	2.021223E-01
Antxr1	*899*	-0.044886401	2338	5.279563E-01
Antxr2	900	0.045232613	7954	5.247752E-01
Antxrl	901	0.074574898	10201.5	2.939481E-01
Anxa1	*902*	0.11131184	12808	1.166012E-01

Spearman Rank correlation analysis performed between Prdm1 and all-expressed genes within the Meredith RNA-seq dataset. Robust Prdm1–associated genes were identified using a cut-off of $p < 0.0005$.

Table S1, Related to Supplemental Figure 3C. Prdm1 associated genes

Anxa10	903	0.080834844	10652	2.551727E-01
Anxa11	904	0.091989656	11516	1.951393E-01
Anxa13	*905*	-0.086746694	532	2.219355E-01
Anxa2	906	0.093195512	11595	1.893242E-01
Anxa3	907	0.081003179	10660	2.541812E-01
Anxa4	*908*	0.053160841	8602	4.546868E-01
Anxa5	909	0.130540057	14327	6.541148E-02
Anxa6	910	0.217723551	18839	1.954800E-03
Anxa7	*911*	0.247936826	19480	4.004141E-04
Anxa8	912	-0.002842184	5038	9.681388E-01
Anxa9	913	0.050415415	8334	4.783470E-01
Aoah	*914*	0.058626067	9136	4.095927E-01
Aoc2	915	0.054691253	8907	4.417871E-01
Aoc3	916	-0.003977039	4984	9.554282E-01
Aox1	*917*	0.049281459	8244	4.883109E-01
Aox3	918	0.131821455	14395	6.278942E-02
Aox3l1	919	-0.056976985	1459.5	4.229135E-01
Aox4	*920*	-0.002605699	5046	9.707886E-01
Ap1ar	921	0.088674357	11250	2.117947E-01
Ap1b1	922	0.119402733	13619	9.217096E-02
Ap1g1	*923*	0.063462778	9430	3.719782E-01
Ap1g2	924	0.119624391	13633	9.156407E-02
Ap1m1	925	0.00256605	5446	9.712329E-01
Ap1m2	*926*	0.203762911	18379	3.803626E-03
Ap1s1	927	0.157621617	16133	2.580813E-02
Ap1s2	928	0.029475848	7043	6.786334E-01
Ap1s3	*929*	0.173806771	17113	1.384133E-02
Ap2a1	930	0.004315305	5531	9.516416E-01
Ap2a2	931	0.098500675	11955	1.652420E-01
Ap2b1	*932*	0.167282105	16787	1.790362E-02
Ap2m1	933	0.162170702	16478	2.177483E-02
Ap2s1	934	0.212657417	18682	2.500871E-03
Ap3b1	*935*	0.160287324	16294	2.337336E-02
Ap3b2	936	-0.018139249	4333	7.987688E-01
Ap3d1	937	0.164406325	16613	2.000067E-02
Ap3m1	*938*	0.14097819	15019	4.645982E-02
Ap3m1-ps	939		#N/A	1.000000E+00
Ap3m2	940	0.011235458	5839	8.745337E-01
Ap3s1	*941*	0.075212186	10258	2.898303E-01
Ap3s1-ps1	942		#N/A	1.000000E+00
Ap3s1-ps2	943		#N/A	1.000000E+00
Ap3s2	*944*	0.29359944	19878	2.448830E-05
Ap4b1	945	0.207387071	18510	3.212720E-03

Spearman Rank correlation analysis performed between Prdm1 and all-expressed genes within the Meredith RNA-seq dataset. Robust Prdm1–associated genes were identified using a cut-off of $p < 0.0005$.

Table S1, Related to Supplemental Figure 3C. Prdm1 associated genes

Ap4e1	946	0.074295299	10162	2.957669E-01
Ap4m1	*947*	-0.001909644	5080	9.785896E-01
Ap4s1	948	0.15392937	15886	2.953792E-02
Ap5b1	949	-0.077394997	736.5	2.760195E-01
Ap5m1	*950*	0.115104158	13329	1.045863E-01
Ap5s1	951	0.279222645	19787	6.228498E-05
Ap5z1	952	0.026342437	6890	7.111832E-01
Apaf1	*953*	0.081733721	10725	2.499091E-01
Apba1	954	0.001856214	5412	9.791885E-01
Apba2	955	-0.016745103	4422	8.139420E-01
Apba3	*956*	0.128631155	14220	6.948312E-02
Apbb1	957	0.009222243	5737	8.968770E-01
Apbb1ip	958	0.069029749	9781	3.314148E-01
Apbb2	*959*	0.155333	15994	2.806908E-02
Apbb3	960	0.045124513	7951	5.257674E-01
Apc	961	0.055735427	8969	4.331064E-01
Apc-ps1	*962*		#N/A	1.000000E+00
Apc2	963	0.108607082	12591.5	1.258088E-01
Apcdd1	964	0.04384013	7855	5.376283E-01
Apcs	*965*		#N/A	1.000000E+00
Apeh	966	0.205857323	18454	3.451195E-03
Apex1	967	0.14730014	15493	3.739284E-02
Apex2	*968*	0.041432674	7708	5.602138E-01
Aph1a	969	-0.014705582	4530	8.362629E-01
Aph1b	970	-0.086750697	472.5	2.219141E-01
Aph1c	*971*	0.095278012	11717	1.795818E-01
Api5	972	0.087077415	11133	2.201716E-01
Apip	973	0.080039248	10593	2.598950E-01
Apitd1	*974*	-0.016731055	4437	8.140953E-01
Aplf	975	0.023952262	6746	7.363723E-01
Apln	976	0.016894796	6165	8.123094E-01
Aplnr	*977*	-0.054451513	1703	4.437940E-01
Aplp1	978	0.149757149	15635	3.429707E-02
Aplp2	979	0.114682214	13305	1.058729E-01
Apmap	*980*	0.143287579	15155	4.295495E-02
Apoa1	981	-7.57115E-05	5285	9.991510E-01
Apoa1bp	982	-0.029371027	3953	6.797132E-01
Apoa2	*983*	0.095072628	11701	1.805258E-01
Apoa4	984	0.033255409	7255	6.401537E-01
Apoa5	985		#N/A	1.000000E+00
Apob	*986*	-0.00568008	4892.5	9.363760E-01
Apobec1	987	0.197775156	18155	4.997200E-03
Apobec2	988	-0.029633734	3946	6.770084E-01

Spearman Rank correlation analysis performed between Prdm1 and all-expressed genes within the Meredith RNA-seq dataset. Robust Prdm1–associated genes were identified using a cut-off of $p < 0.0005$.

Table S1, Related to Supplemental Figure 3C. Prdm1 associated genes

Apobec3	*989*	0.214000566	18713	2.344004E-03
Apobec4	990	-0.054451513	1703	4.437940E-01
Apobr	991	0.113895956	13248	1.083034E-01
Apoc1	*992*	0.096569309	11814	1.737295E-01
Apoc2	993	-0.014176252	4561	8.420783E-01
Apoc3	994	-0.010004001	4744	8.881902E-01
Apoc4	*995*		#N/A	1.000000E+00
Apod	996	0.160517065	16367	2.317310E-02
Apoe	997	-0.009571687	4762	8.929924E-01
Apof	*998*	0.034928859	7327	6.234107E-01
Apoh	999	0.084230398	10936	2.356856E-01
Apol10a	1000	0.014301797	6015.5	8.406982E-01
Apol10b	*1001*	-0.10317255	224	1.459970E-01
Apol11a	1002		#N/A	1.000000E+00
Apol11b	1003	0.019546767	6306	7.835263E-01
Apol6	*1004*	0.04830418	8161	4.969864E-01
Apol7a	1005	-0.054450825	1984	4.437997E-01
Apol7b	1006	0.160304018	16323	2.335876E-02
Apol7c	*1007*	-0.058481216	1425	4.107528E-01
Apol7d	1008		#N/A	1.000000E+00
Apol7e	1009	0.213663002	18705	2.382563E-03
Apol8	*1010*		#N/A	1.000000E+00
Apol9a	1011	0.096148562	11784	1.756206E-01
Apol9b	1012	0.248386921	19488	3.904652E-04
Apold1	*1013*	0.189287957	17815	7.264505E-03
Apom	1014		#N/A	1.000000E+00
Apon	1015	0.143605682	15224	4.249002E-02
Apoo	*1016*	0.105541228	12396	1.369145E-01
Apoo-ps	1017	0.059200223	9167	4.050137E-01
Apool	1018	-0.036543663	3669	6.074354E-01
Apopt1	*1019*	0.105501832	12391	1.370619E-01
App	1020	0.153444441	15861	3.006041E-02
Appbp2	1021	0.176886433	17263	1.222224E-02
Appl1	*1022*	-0.047443009	2250	5.046982E-01
Appl2	1023	0.138596392	14859	5.032167E-02
Aprt	1024	0.022790269	6678	7.487224E-01
Aptx	*1025*	0.230559666	19178	1.021514E-03
Aqp1	1026	0.113548679	13094	1.093908E-01
Aqp11	1027	-0.082239396	616	2.469812E-01
Aqp12	*1028*	0.043243133	7816.5	5.431865E-01
Aqp2	1029		#N/A	1.000000E+00
Aqp3	1030	0.082850117	10818	2.434771E-01
Aqp4	*1031*	0.049509987	8254	4.862940E-01

Spearman Rank correlation analysis performed between Prdm1 and all-expressed genes within the Meredith RNA-seq dataset. Robust Prdm1–associated genes were identified using a cut-off of $p < 0.0005$.

Table S1, Related to Supplemental Figure 3C. Prdm1 associated genes

Aqp5	1032		#N/A	1.000000E+00
Aqp6	1033	0.014759448	6059.5	8.356716E-01
Aqp7	*1034*	0.190288113	17852	6.956539E-03
Aqp8	1035	-0.032131636	3840.5	6.515007E-01
Aqp9	1036	-0.086748028	498.5	2.219283E-01
Aqr	*1037*	0.091290037	11467	1.985722E-01
Ar	1038	0.122053407	13790	8.512034E-02
Araf	1039	0.232132554	19214	9.410891E-04
Arap1	*1040*	0.075766551	10327	2.862799E-01
Arap2	1041	0.111449542	12816	1.161469E-01
Arap3	1042	0.075110366	10254	2.904856E-01
Arc	*1043*	-0.083002077	605	2.426106E-01
Arcn1	1044	0.097475356	11872	1.697084E-01
Areg	1045	-0.117569446	123	9.731414E-02
Arf1	*1046*	0.144141752	15316	4.171608E-02
Arf2	1047	0.144403374	15330	4.134269E-02
Arf3	1048	0.245163186	19441	4.670941E-04
Arf4	*1049*	0.074267962	10160	2.959452E-01
Arf5	1050	0.184949213	17633	8.745635E-03
Arf6	1051	0.241721988	19388	5.640920E-04
Arfgap1	*1052*	0.231346611	19193	9.805185E-04
Arfgap2	1053	0.052111252	8478	4.636541E-01
Arfgap3	1054	0.15863761	16208	2.485563E-02
Arfgef1	*1055*	-0.009249884	4775	8.965696E-01
Arfgef2	1056	0.1100967	12724	1.206707E-01
Arfip1	1057	0.034122535	7300	6.314546E-01
Arfip2	*1058*	0.142979964	15140	4.340860E-02
Arfrp1	1059	0.258685647	19608	2.167988E-04
Arg1	1060	0.032115605	7188	6.516632E-01
Arg2	*1061*	0.013226053	5946	8.525386E-01
Arglu1	1062	0.135760757	14675	5.526379E-02
Arhgap1	1063	0.039558409	7606	5.781073E-01
Arhgap10	*1064*	0.048886683	8211	4.918056E-01
Arhgap11a	1065	0.139273303	14906	4.919789E-02
Arhgap12	1066	0.107905396	12548	1.282870E-01
Arhgap15	*1067*	0.111624021	12829	1.155732E-01
Arhgap17	1068	0.053815252	8854	4.491452E-01
Arhgap18	1069	0.085000945	10989	2.314129E-01
Arhgap19	*1070*	0.055844458	8976	4.322057E-01
Arhgap20	1071	-0.032762514	3799	6.451205E-01
Arhgap21	1072	0.008044997	5682	9.099813E-01
Arhgap22	*1073*	0.106031436	12428	1.350900E-01
Arhgap23	1074	0.028561444	6997	6.880742E-01

Spearman Rank correlation analysis performed between Prdm1 and all-expressed genes within the Meredith RNA-seq dataset. Robust Prdm1–associated genes were identified using a cut-off of $p < 0.0005$.

Table S1, Related to Supplemental Figure 3C. Prdm1 associated genes

Arhgap24	1075	0.119270964	13606	9.253325E-02
Arhgap25	*1076*	0.072654726	10040	3.065895E-01
Arhgap26	1077	0.097441693	11869	1.698566E-01
Arhgap27	1078	0.330829364	19987	1.712192E-06
Arhgap28	*1079*	0.014638494	6050	8.369995E-01
Arhgap29	1080	0.043575145	7838	5.400919E-01
Arhgap30	1081	0.107851408	12544	1.284792E-01
Arhgap31	*1082*	0.100161765	12073	1.581934E-01
Arhgap32	1083	0.12678587	14092	7.361310E-02
Arhgap33	1084	-0.103161098	248	1.460420E-01
Arhgap36	*1085*		#N/A	1.000000E+00
Arhgap39	1086	-0.158992544	15	2.453007E-02
Arhgap4	1087	0.088188805	11221	2.143172E-01
Arhgap40	*1088*		#N/A	1.000000E+00
Arhgap42	1089	0.055923604	8978	4.315525E-01
Arhgap44	1090	0.030989673	7129	6.631141E-01
Arhgap5	*1091*	0.025056695	6823	7.246962E-01
Arhgap6	1092	-0.01256849	4642	8.597926E-01
Arhgap8	1093	0.080178154	10610	2.590662E-01
Arhgap9	*1094*	0.08362653	10888	2.390725E-01
Arhgdia	1095	0.107138657	12506	1.310378E-01
Arhgdib	1096	0.207992683	18522	3.122502E-03
Arhgdig	*1097*		#N/A	1.000000E+00
Arhgef1	1098	0.051982079	8468	4.647644E-01
Arhgef10	1099	-0.023231589	4143	7.440241E-01
Arhgef10l	*1100*	-0.066663346	1288	3.482976E-01
Arhgef11	1101	0.066417701	9614	3.500806E-01
Arhgef12	1102	-0.000307851	5274	9.965481E-01
Arhgef15	*1103*	0.094890176	11694	1.813676E-01
Arhgef16	1104	-0.076814448	847	2.796484E-01
Arhgef17	1105	0.01913689	6287	7.879568E-01
Arhgef18	*1106*	0.035606799	7357	6.166818E-01
Arhgef19	1107	-0.038406171	3065	5.892384E-01
Arhgef2	1108	0.099023214	11999	1.629998E-01
Arhgef25	*1109*	0.003230329	5489	9.637905E-01
Arhgef26	1110	0.148193575	15542	3.624087E-02
Arhgef28	1111	0.000169417	5297	9.981003E-01
Arhgef3	*1112*	0.060458383	9236	3.950862E-01
Arhgef33	1113	0.08551953	11022	2.285683E-01
Arhgef37	1114	0.029746912	7051	6.758444E-01
Arhgef38	*1115*	0.194623275	18030	5.752000E-03
Arhgef39	1116	0.050054333	8302	4.815078E-01
Arhgef4	1117	0.031005997	7132	6.629475E-01

Spearman Rank correlation analysis performed between Prdm1 and all-expressed genes within the Meredith RNA-seq dataset. Robust Prdm1–associated genes were identified using a cut-off of $p < 0.0005$.

Table S1, Related to Supplemental Figure 3C. Prdm1 associated genes

Arhgef40	*1118*	0.10334132	12261	1.453351E-01
Arhgef5	1119	0.187095591	17721	7.982406E-03
Arhgef6	1120	0.212183709	18658	2.558430E-03
Arhgef7	*1121*	0.058166986	9104	4.132760E-01
Arhgef9	1122	0.064260159	9490	3.659878E-01
Arid1a	1123	0.233461943	19238	8.776992E-04
Arid1b	*1124*	0.107919926	12550	1.282353E-01
Arid2	1125	0.230216375	19155	1.039886E-03
Arid3a	1126	0.097473651	11871	1.697159E-01
Arid3b	*1127*	0.069769939	9830	3.262436E-01
Arid3c	1128		#N/A	1.000000E+00
Arid4a	1129	0.025907721	6866	7.157422E-01
Arid4b	*1130*	0.188189008	17770	7.616839E-03
Arid5a	1131	0.044773038	7918	5.289999E-01
Arid5b	1132	-0.035198173	3705	6.207338E-01
Arih1	*1133*	0.193687816	17979	5.994862E-03
Arih2	1134	0.026925641	6928	7.050830E-01
Arl1	1135	0.23151297	19200	9.720481E-04
Arl10	*1136*		#N/A	1.000000E+00
Arl11	1137	0.15058102	15687	3.330891E-02
Arl13a	1138	-0.062712762	1366	3.776676E-01
Arl13b	*1139*	0.162393855	16493	2.159183E-02
Arl14	1140	-0.002295611	5059.5	9.742636E-01
Arl14ep	1141	0.162277885	16483	2.168677E-02
Arl14epl	*1142*	0.021757451	6575	7.597536E-01
Arl15	1143	0.071582224	9955	3.138036E-01
Arl16	1144	0.076792683	10400	2.797851E-01
Arl2	*1145*	-0.056355383	1474	4.279992E-01
Arl2bp	1146	0.250799451	19516	3.409351E-04
Arl3	1147	-0.156027586	17	2.736564E-02
Arl4a	*1148*	-0.007903761	4826	9.115552E-01
Arl4c	1149	0.007169963	5633	9.197376E-01
Arl4d	1150	-0.043397509	2394.5	5.417465E-01
Arl5a	*1151*	0.164433809	16615	1.997966E-02
Arl5b	1152	0.172281708	17036	1.471055E-02
Arl5c	1153	0.022454528	6658	7.523029E-01
Arl6	*1154*	0.060061123	9216	3.982049E-01
Arl6ip1	1155	0.093592156	11620	1.874394E-01
Arl6ip4	1156	0.371117082	20017	6.304673E-08
Arl6ip5	*1157*	0.109722783	12694	1.219448E-01
Arl6ip6	1158	0.021723969	6567	7.601120E-01
Arl8a	1159	-0.021478179	4194	7.627449E-01
Arl8b	*1160*	0.173451705	17098	1.403958E-02

Spearman Rank correlation analysis performed between Prdm1 and all-expressed genes within the Meredith RNA-seq dataset. Robust Prdm1–associated genes were identified using a cut-off of $p < 0.0005$.

Table S1, Related to Supplemental Figure 3C. Prdm1 associated genes

Arl9	1161	-0.066855996	1239.5	3.469032E-01
Armc1	1162	0.156830828	16101	2.657102E-02
Armc10	*1163*	0.136176036	14710	5.451586E-02
Armc12	1164		#N/A	1.000000E+00
Armc2	1165	0.091272442	11462	1.986591E-01
Armc3	*1166*	0.036352429	7398	6.093178E-01
Armc4	1167	-0.000654998	5233	9.926556E-01
Armc5	1168	0.095563853	11740	1.782739E-01
Armc6	*1169*	0.023462819	6715	7.415662E-01
Armc7	1170	0.057275766	9051	4.204815E-01
Armc8	1171	0.056932386	9033	4.232772E-01
Armc9	*1172*	0.100498916	12090	1.567907E-01
Armcx1	1173	0.05012654	8306	4.808748E-01
Armcx2	1174	0.032580413	7211	6.469595E-01
Armcx3	*1175*	0.095713375	11750	1.775926E-01
Armcx4	1176	-0.112467236	144	1.128319E-01
Armcx5	1177	0.062762152	9376	3.772913E-01
Armcx6	*1178*	0.262645928	19651	1.717763E-04
Arnt	1179	0.183880895	17588	9.149109E-03
Arnt2	1180	0.163427903	16570	2.076111E-02
Arntl	*1181*	-0.044333853	2357	5.330532E-01
Arntl2	1182	-0.128196262	71	7.043905E-02
Arpc1a	1183	0.169967361	16927	1.612081E-02
Arpc1b	*1184*	0.178122439	17311	1.162079E-02
Arpc2	1185	0.163491057	16571	2.071128E-02
Arpc3	1186	0.110902592	12783	1.179597E-01
Arpc4	*1187*	0.254454377	19559	2.768994E-04
Arpc5	1188	0.101370702	12142	1.532072E-01
Arpc5l	1189	0.052771011	8580	4.580060E-01
Arpp19	*1190*	0.178335638	17321	1.151973E-02
Arpp21	1191	0.124625278	13947	7.869961E-02
Arr3	1192		#N/A	1.000000E+00
Arrb1	*1193*	0.009372818	5741	8.952028E-01
Arrb2	1194	-0.025671185	4080	7.182271E-01
Arrdc1	1195	0.205528816	18437	3.504447E-03
Arrdc2	*1196*	0.082428524	10783	2.458923E-01
Arrdc3	1197	0.105106695	12364	1.385474E-01
Arrdc4	1198	0.037456566	7455	5.984850E-01
Arrdc5	*1199*		#N/A	1.000000E+00
Arsa	1200	0.0040028	5516	9.551398E-01
Arsb	1201	0.059221874	9169	4.048416E-01
Arsg	*1202*	0.087072363	11132	2.201984E-01
Arsi	1203	0.053805701	8820	4.492258E-01

Spearman Rank correlation analysis performed between Prdm1 and all-expressed genes within the Meredith RNA-seq dataset. Robust Prdm1–associated genes were identified using a cut-off of $p < 0.0005$.

Table S1, Related to Supplemental Figure 3C. Prdm1 associated genes

Arsj	1204	0.103564782	12274	1.444622E-01
Arsk	*1205*	-0.031202762	3880	6.609408E-01
Art1	1206	-0.04601882	2290	5.175879E-01
Art2a-ps	1207	0.138673542	14866	5.019252E-02
Art2b	*1208*	-0.012131839	4666	8.646162E-01
Art3	1209	-0.024673834	4110	7.287368E-01
Art4	1210	-0.038406171	3065	5.892384E-01
Art5	*1211*	0.073928833	10125	2.981622E-01
Artn	1212		#N/A	1.000000E+00
Arv1	1213	-0.051082892	2180	4.725340E-01
Arvcf	*1214*	0.024212932	6765	7.336109E-01
Arx	1215	0.086412184	11087.5	2.237298E-01
Arxes1	1216	0.00860898	5710	9.037002E-01
Arxes2	*1217*	-0.0633173	1355	3.730776E-01
As3mt	1218	-0.02379994	4128	7.379875E-01
Asah1	1219	0.139722836	14932	4.846320E-02
Asah2	*1220*	0.071818561	9984	3.122045E-01
Asap1	1221	0.07523554	10281	2.896802E-01
Asap2	1222	0.071586547	9956	3.137743E-01
Asap3	*1223*	0.275904483	19763	7.670998E-05
Asb1	1224	0.123457182	13874	8.156558E-02
Asb10	1225	-0.001606767	5097.5	9.819847E-01
Asb11	*1226*	-0.095267438	357.5	1.796303E-01
Asb12	1227	0.154685737	15965	2.873847E-02
Asb13	1228	0.081299149	10684	2.524443E-01
Asb14	*1229*	-0.038406171	3065	5.892384E-01
Asb15	1230	0.043679362	7844	5.391223E-01
Asb16	1231	0.07277533	10046	3.057851E-01
Asb17	*1232*	0.012216999	5887	8.636750E-01
Asb18	1233	0.064123369	9476.5	3.670112E-01
Asb2	1234	0.148980053	15589	3.525180E-02
Asb3	*1235*	0.129272793	14259	6.809208E-02
Asb4	1236	0.109492511	12666.5	1.227347E-01
Asb5	1237	0.052728522	8550.5	4.583686E-01
Asb6	*1238*	0.072855419	10053	3.052518E-01
Asb7	1239	0.133520029	14509	5.944726E-02
Asb8	1240	0.042995839	7811	5.454971E-01
Asb9	*1241*	-0.077393023	832.5	2.760318E-01
Ascc1	1242	0.12528243	13989	7.712335E-02
Ascc2	1243	0.106484394	12459	1.334208E-01
Ascc3	*1244*	0.172781195	17068	1.442075E-02
Ascl1	1245	0.033484523	7265	6.378503E-01
Ascl2	1246	-0.018790377	4285	7.917077E-01

Spearman Rank correlation analysis performed between Prdm1 and all-expressed genes within the Meredith RNA-seq dataset. Robust Prdm1–associated genes were identified using a cut-off of $p < 0.0005$.

Table S1, Related to Supplemental Figure 3C. Prdm1 associated genes

Ascl3	*1247*	-0.054450825	1984	4.437997E-01
Ascl4	1248	0.08427422	10940	2.354411E-01
Ascl5	1249		#N/A	1.000000E+00
Asf1a	*1250*	0.160228571	16287	2.342481E-02
Asf1b	1251	0.116013642	13395	1.018552E-01
Asgr1	1252	-0.038406171	3065	5.892384E-01
Asgr2	*1253*		#N/A	1.000000E+00
Ash1l	1254	0.151165937	15724	3.262213E-02
Ash2l	1255	0.068958078	9773	3.319182E-01
Asic1	*1256*	-0.095280123	319	1.795721E-01
Asic2	1257	0.089807613	11331	2.059905E-01
Asic3	1258	-0.158297948	16	2.517064E-02
Asic4	*1259*	-0.038406171	3065	5.892384E-01
Asic5	1260	0.051226597	8402	4.712875E-01
Asl	1261	0.032889374	7231	6.438406E-01
Asmt	*1262*	0.138506292	14856	5.047285E-02
Asna1	1263	-0.007392057	4840	9.172601E-01
Asns	1264	0.238263921	19334	6.800381E-04
Asnsd1	*1265*	0.110143588	12729	1.205116E-01
Aspa	1266	0.052728522	8550.5	4.583686E-01
Aspdh	1267		#N/A	1.000000E+00
Aspg	*1268*	-0.018789992	4291.5	7.917119E-01
Asph	1269	0.078323296	10488	2.702834E-01
Asphd1	1270	-0.063731916	1350	3.699496E-01
Asphd2	*1271*		#N/A	1.000000E+00
Aspm	1272	0.16144672	16448	2.237778E-02
Aspn	1273	0.124113861	13917	7.994422E-02
Asprv1	*1274*	-0.01075067	4713	8.799057E-01
Aspscr1	1275	0.122022015	13786	8.520124E-02
Asrgl1	1276	0.145245661	15366	4.015959E-02
Ass1	*1277*	0.261158068	19640	1.875555E-04
Aste1	1278	0.104761269	12340	1.398561E-01
Astl	1279	-0.016730797	4444.5	8.140981E-01
Astn1	*1280*	0.007304668	5646	9.182348E-01
Astn2	1281	0.097885765	11906	1.679100E-01
Astx1a	1282		#N/A	1.000000E+00
Astx1b	*1283*		#N/A	1.000000E+00
Astx1c	1284		#N/A	1.000000E+00
Astx2	1285		#N/A	1.000000E+00
Astx3	*1286*		#N/A	1.000000E+00
Astx4a	1287		#N/A	1.000000E+00
Astx4b	1288	-0.038406171	3065	5.892384E-01
Astx4c	*1289*		#N/A	1.000000E+00

Spearman Rank correlation analysis performed between Prdm1 and all-expressed genes within the Meredith RNA-seq dataset. Robust Prdm1–associated genes were identified using a cut-off of $p < 0.0005$.

Table S1, Related to Supplemental Figure 3C. Prdm1 associated genes

Astx4d	1290		#N/A	1.000000E+00
Astx5	1291		#N/A	1.000000E+00
Astx6	*1292*		#N/A	1.000000E+00
Asun	1293	0.212048461	18651	2.575083E-03
Asxl1	1294	0.208007802	18523	3.120279E-03
Asxl2	*1295*	0.132209252	14426	6.201310E-02
Asxl3	1296	-0.071502132	936	3.143467E-01
Asz1	1297	0.011269971	5843	8.741515E-01
Atad1	*1298*	0.180274129	17417	1.063575E-02
Atad2	1299	0.163725477	16579	2.052723E-02
Atad2b	1300	-0.000586864	5237	9.934195E-01
Atad3a	*1301*	0.047005737	8080	5.086378E-01
Atad5	1302	0.105296292	12380	1.378331E-01
Atat1	1303	0.086003979	11053	2.259333E-01
Atcay	*1304*	-0.032450477	3820	6.482730E-01
Ate1	1305		#N/A	1.000000E+00
Atf1	1306	0.174142265	17130	1.365627E-02
Atf2	*1307*	0.069977379	9842	3.248038E-01
Atf3	1308	0.099230201	12012	1.621180E-01
Atf4	1309	0.029969532	7065	6.735570E-01
Atf5	*1310*	0.078681072	10511	2.680944E-01
Atf6	1311	0.128387586	14205	7.001720E-02
Atf6b	1312	0.178668005	17332	1.136372E-02
Atf7	*1313*	0.230258711	19171	1.037604E-03
Atf7ip	1314	0.168478082	16852	1.708944E-02
Atf7ip2	1315	0.109492511	12666.5	1.227347E-01
Atg10	*1316*	0.185801675	17673	8.435086E-03
Atg12	1317	0.106850203	12487	1.320844E-01
Atg13	1318	0.112445839	12885	1.129008E-01
Atg14	*1319*	0.084989533	10988	2.314758E-01
Atg16l1	1320	0.178926895	17350	1.124349E-02
Atg16l2	1321	0.286899739	19848	3.807400E-05
Atg2a	*1322*	-0.006764522	4858	9.242622E-01
Atg2b	1323	0.137328586	14777	5.248387E-02
Atg3	1324	0.096238972	11792	1.752130E-01
Atg4a	*1325*	-0.041204543	2446	5.623774E-01
Atg4a-ps	1326	0.097047874	11841	1.715969E-01
Atg4b	1327	-0.055725998	1499	4.331844E-01
Atg4c	*1328*	0.137759529	14806	5.174042E-02
Atg4d	1329	0.017602781	6207	8.045989E-01
Atg5	1330	0.114928139	13316	1.051215E-01
Atg7	*1331*	0.234046254	19258	8.511002E-04
Atg9a	1332	0.262745446	19653	1.707663E-04

Spearman Rank correlation analysis performed between Prdm1 and all-expressed genes within the Meredith RNA-seq dataset. Robust Prdm1–associated genes were identified using a cut-off of $p < 0.0005$.

Table S1, Related to Supplemental Figure 3C. Prdm1 associated genes

Atg9b	1333	0.192510301	17931	6.313577E-03
Athl1	*1334*	-0.117569446	123	9.731414E-02
Atic	1335	-0.104463649	211	1.409912E-01
Atl1	1336	-0.020772979	4215	7.703138E-01
Atl2	*1337*	0.141735362	15063	4.528526E-02
Atl3	1338	0.273757983	19748	8.765238E-05
Atm	1339	0.198978842	18197	4.733257E-03
Atmin	*1340*	0.048292983	8158	4.970863E-01
Atn1	1341	-0.035903019	3688	6.137516E-01
Atoh1	1342		#N/A	1.000000E+00
Atoh7	*1343*		#N/A	1.000000E+00
Atoh8	1344	-0.067435469	972	3.427303E-01
Atox1	1345	0.09636945	11799	1.746259E-01
Atp10a	*1346*	0.201936634	18323	4.137079E-03
Atp10b	1347	0.061622377	9308	3.860331E-01
Atp10d	1348	-0.069062458	958	3.311851E-01
Atp11a	*1349*	0.038935319	7575	5.841144E-01
Atp11b	1350	0.153553782	15869	2.994191E-02
Atp11c	1351	0.140548431	14991	4.713773E-02
Atp12a	*1352*	0.158981681	16224	2.453998E-02
Atp13a1	1353	0.044335639	7886	5.330367E-01
Atp13a2	1354	0.168502763	16854	1.707299E-02
Atp13a3	*1355*	0.193917243	17989	5.934465E-03
Atp13a4	1356	0.135057887	14624	5.654901E-02
Atp13a5	1357	0.042204622	7752	5.529223E-01
Atp1a1	*1358*	0.141914262	15077	4.501140E-02
Atp1a2	1359	0.085717556	11037	2.274886E-01
Atp1a3	1360	0.160304018	16323	2.335876E-02
Atp1a4	*1361*	-0.038406171	3065	5.892384E-01
Atp1b1	1362	0.16530503	16675	1.932357E-02
Atp1b2	1363	-0.056976985	1459.5	4.229135E-01
Atp1b3	*1364*	0.135977176	14698	5.487296E-02
Atp1b4	1365	0.052636197	8514	4.591570E-01
Atp2a1	1366	0.011520007	5852	8.713833E-01
Atp2a2	*1367*	0.138407965	14850	5.063826E-02
Atp2a3	1368	0.088252845	11227	2.139832E-01
Atp2b1	1369	0.100492017	12089	1.568193E-01
Atp2b2	*1370*	0.106818802	12483	1.321987E-01
Atp2b3	1371	0.108443559	12579	1.263830E-01
Atp2b4	1372	-0.01225016	4664	8.633086E-01
Atp2c1	*1373*	0.109179419	12635	1.238150E-01
Atp2c2	1374	0.064123369	9476.5	3.670112E-01
Atp4a	1375	-0.118722667	108	9.405297E-02

Spearman Rank correlation analysis performed between Prdm1 and all-expressed genes within the Meredith RNA-seq dataset. Robust Prdm1–associated genes were identified using a cut-off of $p < 0.0005$.

Table S1, Related to Supplemental Figure 3C. Prdm1 associated genes

Atp4b	*1376*	0.038312033	7515	5.901521E-01
Atp5a1	1377	0.136775056	14749	5.345178E-02
Atp5b	1378	0.21421885	18722	2.319372E-03
Atp5c1	*1379*	0.122610156	13821	8.369590E-02
Atp5d	1380	0.227171263	19082	1.216641E-03
Atp5e	1381	0.215305777	18756	2.200187E-03
Atp5f1	*1382*	0.169152145	16881	1.664492E-02
Atp5g1	1383	0.206341414	18471	3.374054E-03
Atp5g2	1384	0.163798818	16584	2.046994E-02
Atp5g3	*1385*	0.108684715	12602	1.255369E-01
Atp5h	1386	0.169899054	16923	1.616416E-02
Atp5j	1387	0.063085577	9393	3.748329E-01
Atp5j2	*1388*	0.167109958	16774	1.802355E-02
Atp5k	1389	0.121158397	13736	8.745095E-02
Atp5l	1390	0.185343968	17651	8.600589E-03
Atp5l-ps1	*1391*		#N/A	1.000000E+00
Atp5l2	1392		#N/A	1.000000E+00
Atp5o	1393	0.114348816	13277	1.068982E-01
Atp5s	*1394*	0.175745152	17205	1.280172E-02
Atp5sl	1395	0.191799033	17905	6.513347E-03
Atp6ap1	1396	0.042416731	7766	5.509269E-01
Atp6ap1l	*1397*	0.014301797	6015.5	8.406982E-01
Atp6ap2	1398	0.05432824	8885	4.448279E-01
Atp6v0a1	1399	0.02462331	6796	7.292706E-01
Atp6v0a2	*1400*	0.166007399	16705	1.880832E-02
Atp6v0a4	1401	-0.020575904	4219	7.724330E-01
Atp6v0b	1402	0.047085502	8084	5.079180E-01
Atp6v0c	*1403*	0.066867136	9637	3.468227E-01
Atp6v0d1	1404	0.23562402	19281	7.829393E-04
Atp6v0d2	1405	0.159065416	16228	2.446369E-02
Atp6v0e	*1406*	0.123095941	13848	8.246872E-02
Atp6v0e2	1407	-0.052816379	2144	4.576190E-01
Atp6v1a	1408	0.155851802	16032	2.754222E-02
Atp6v1b1	*1409*		#N/A	1.000000E+00
Atp6v1b2	1410	-0.111206352	149	1.169502E-01
Atp6v1c1	1411	0.011162995	5836	8.753363E-01
Atp6v1c2	*1412*	-0.066855996	1239.5	3.469032E-01
Atp6v1d	1413	0.199809755	18239	4.558432E-03
Atp6v1e1	1414	0.089194106	11286	2.091183E-01
Atp6v1e2	*1415*		#N/A	1.000000E+00
Atp6v1f	1416	0.176267723	17236	1.253345E-02
Atp6v1g1	1417	0.18225743	17503	9.793833E-03
Atp6v1g2	*1418*	-0.074279401	899	2.958706E-01

Spearman Rank correlation analysis performed between Prdm1 and all-expressed genes within the Meredith RNA-seq dataset. Robust Prdm1–associated genes were identified using a cut-off of $p < 0.0005$.

Table S1, Related to Supplemental Figure 3C. Prdm1 associated genes

Atp6v1g3	1419	-0.038030081	3632	5.928927E-01
Atp6v1h	1420	0.051797782	8455	4.663511E-01
Atp7a	*1421*	0.023359336	6710	7.426659E-01
Atp7b	1422	0.042113863	7746	5.537772E-01
Atp8a1	1423	0.240581204	19370	6.001505E-04
Atp8a2	*1424*	-0.047676545	2244	5.026007E-01
Atp8b1	1425	0.013406983	5961	8.505448E-01
Atp8b2	1426	0.163822051	16585	2.045182E-02
Atp8b3	*1427*	0.113548679	13094	1.093908E-01
Atp8b4	1428	0.017556608	6205	8.051012E-01
Atp8b5	1429	0.086558633	11108	2.229430E-01
Atp9a	*1430*	0.233654209	19249	8.688636E-04
Atp9b	1431	0.15606328	16041	2.732990E-02
Atpaf1	1432	0.118252325	13545	9.537238E-02
Atpaf2	*1433*	-0.006538581	4866	9.267847E-01
Atpbd4	1434	0.176904758	17264	1.221312E-02
Atpif1	1435	0.185223363	17647	8.644675E-03
Atr	*1436*	0.013634234	5975	8.480418E-01
Atraid	1437	0.117852769	13516	9.650474E-02
Atrip	1438	0.077280903	10427	2.767302E-01
Atrn	*1439*	0.121190981	13740	8.736521E-02
Atrnl1	1440	0.102268536	12195	1.495816E-01
Atrx	1441	0.194366871	18016	5.817676E-03
Atxn1	*1442*	0.134898165	14613	5.684449E-02
Atxn10	1443	0.120917846	13719	8.808598E-02
Atxn1l	1444	0.119309912	13609	9.242605E-02
Atxn2	*1445*	0.160372563	16355	2.329889E-02
Atxn2l	1446	0.069585675	9820	3.275260E-01
Atxn3	1447	0.159848927	16267	2.375963E-02
Atxn7	*1448*	0.089628906	11316	2.068981E-01
Atxn7l1	1449	0.162579754	16500	2.144040E-02
Atxn7l2	1450	0.021211577	6449	7.656037E-01
Atxn7l3	*1451*	0.198837373	18191	4.763614E-03
Atxn7l3b	1452	0.286019331	19841	4.031428E-05
AU015228	1453	0.075711532	10311	2.866309E-01
AU015336	*1454*		#N/A	1.000000E+00
AU015836	1455	0.143605682	15224	4.249002E-02
AU016765	1456	-0.018447724	4312.5	7.954215E-01
AU018091	*1457*	-0.041858835	2437	5.561828E-01
AU019823	1458	0.253499761	19545	2.924479E-04
AU019990	1459	0.006601358	5619	9.260838E-01
AU021092	*1460*	-0.054450825	1984	4.437997E-01
AU022252	1461	0.078542086	10498	2.689433E-01

Spearman Rank correlation analysis performed between Prdm1 and all-expressed genes within the Meredith RNA-seq dataset. Robust Prdm1–associated genes were identified using a cut-off of $p < 0.0005$.

Table S1, Related to Supplemental Figure 3C. Prdm1 associated genes

AU022751	1462		#N/A	1.000000E+00
AU023070	*1463*		#N/A	1.000000E+00
AU023871	1464	-0.033075221	3785	6.419675E-01
AU040320	1465	0.12706485	14120	7.297622E-02
AU040972	*1466*		#N/A	1.000000E+00
AU041133	1467	0.019135125	6286	7.879759E-01
Auh	1468	0.153558107	15870	2.993724E-02
Aunip	*1469*	0.071763977	9976	3.125733E-01
Aup1	1470	0.017652024	6209	8.040633E-01
Aurka	1471	0.084629528	10960	2.334655E-01
Aurkaip1	*1472*	0.106765038	12479	1.323946E-01
Aurkb	1473	0.093955675	11639	1.857241E-01
Aurkc	1474	-0.054450825	1984	4.437997E-01
Auts2	*1475*	0.214208619	18721	2.320521E-03
AUTS2	1476		#N/A	1.000000E+00
AV039307	1477	0.098546744	11961	1.650434E-01
AV064505	*1478*	-0.03122443	3877.5	6.607200E-01
AV099323	1479	-0.054450825	1984	4.437997E-01
AV320801	1480		#N/A	1.000000E+00
Aven	*1481*	0.159174658	16234	2.436446E-02
Avil	1482	-0.076074634	863	2.843194E-01
Avl9	1483	0.128392429	14207	7.000654E-02
Avp	*1484*	0.143605682	15224	4.249002E-02
Avpi1	1485	0.045333195	7958	5.238529E-01
Avpr1a	1486	-0.066858259	1029.5	3.468868E-01
Avpr1b	*1487*	-0.054451513	1703	4.437940E-01
Avpr2	1488		#N/A	1.000000E+00
AW011738	1489	0.045952076	8001	5.181961E-01
AW112010	*1490*	-0.003031437	5024	9.660185E-01
AW146154	1491	0.097186271	11853	1.709838E-01
AW209491	1492	-0.07444783	895	2.947738E-01
AW495222	*1493*	-0.067794452	970	3.401612E-01
AW549877	1494	0.15864694	16209	2.484702E-02
AW551984	1495	0.06879991	9761	3.330311E-01
AW554918	*1496*	0.077656798	10451	2.743935E-01
AW822073	1497		#N/A	1.000000E+00
AW822252	1498	-0.038406171	3065	5.892384E-01
Awat1	*1499*		#N/A	1.000000E+00
Awat2	1500	0.022714618	6671	7.495287E-01
Axdnd1	1501	-0.006217283	4875	9.303731E-01
Axin1	*1502*	0.134179169	14564	5.819041E-02
Axin2	1503	0.051280099	8410	4.708239E-01
Axl	1504	0.041241191	7702	5.620296E-01

Spearman Rank correlation analysis performed between Prdm1 and all-expressed genes within the Meredith RNA-seq dataset. Robust Prdm1–associated genes were identified using a cut-off of $p < 0.0005$.

Table S1, Related to Supplemental Figure 3C. Prdm1 associated genes

AY036118	*1505*	0.153028855	15843.5	3.051443E-02
AY074887	1506	0.166818393	16765	1.822826E-02
AY358078	1507	0.113548679	13094	1.093908E-01
AY512915	*1508*	-0.038406171	3065	5.892384E-01
AY512931	1509	0.068048738	9707	3.383489E-01
AY702102	1510		#N/A	1.000000E+00
AY702103	*1511*	-0.038406171	3065	5.892384E-01
AY761184	1512		#N/A	1.000000E+00
AY761185	1513	0.147627804	15514	3.696682E-02
Aym1	*1514*		#N/A	1.000000E+00
Azgp1	1515	-0.005348205	4909.5	9.400862E-01
Azi1	1516		#N/A	1.000000E+00
Azi2	*1517*	0.060308375	9227	3.962621E-01
Azin1	1518	0.125384054	13995	7.688189E-02
B020004C17Ril	1519	-0.038406171	3065	5.892384E-01
B020004J07Rik	*1520*	0.018446509	6243	7.954347E-01
B020018G12Ri	1521		#N/A	1.000000E+00
B020031M17R	1522		#N/A	1.000000E+00
B130006D01Ri	*1523*	0.064123369	9476.5	3.670112E-01
B130011K05Ril	1524		#N/A	1.000000E+00
B130024G19Ri	1525		#N/A	1.000000E+00
B130034C11Ril	*1526*	-0.033075221	3785	6.419675E-01
B130055M24R	1527	-0.038406171	3065	5.892384E-01
B230104C08Ril	1528	0.053267784	8685	4.537786E-01
B230104I21Rik	*1529*	-0.038406171	3065	5.892384E-01
B230112J18Rik	1530		#N/A	1.000000E+00
B230118H07Ri	1531	0.180789391	17438	1.041109E-02
B230119M05R	*1532*	0.052728522	8550.5	4.583686E-01
B230120H23Ri	1533	0.014295828	6003	8.407638E-01
B230206H07Ri	1534	0.200058801	18245	4.507175E-03
B230206L02Rik	*1535*	-0.029158944	3962	6.818997E-01
B230208B08Ril	1536		#N/A	1.000000E+00
B230216G23Ri	1537		#N/A	1.000000E+00
B230216N24Ri	*1538*		#N/A	1.000000E+00
B230217C12Ril	1539	0.077008503	10413	2.784319E-01
B230219D22Ri	1540	0.178441832	17323	1.146968E-02
B230307C23Ril	*1541*	-0.038406171	3065	5.892384E-01
B230312C02Ril	1542	-0.054451513	1703	4.437940E-01
B230314M03R	1543	-0.038406171	3065	5.892384E-01
B230317F23Rik	*1544*		#N/A	1.000000E+00
B230319C09Ril	1545	-0.038406171	3065	5.892384E-01
B230322F03Rik	1546		#N/A	1.000000E+00
B230325K18Ril	*1547*	-0.056977171	1458	4.229120E-01

Spearman Rank correlation analysis performed between Prdm1 and all-expressed genes within the Meredith RNA-seq dataset. Robust Prdm1–associated genes were identified using a cut-off of $p < 0.0005$.

Table S1, Related to Supplemental Figure 3C. Prdm1 associated genes

B230359F08Rik	1548		#N/A	1.000000E+00
B230364G03Ri	1549		#N/A	1.000000E+00
B230369F24Rik	*1550*	-0.032621296	3807	6.465464E-01
B230377A18Ril	1551		#N/A	1.000000E+00
B230378P21Ril	1552	0.014301797	6015.5	8.406982E-01
B230398E01Ril	*1553*	-0.032762514	3799	6.451205E-01
B2m	1554	0.22087349	18929	1.672529E-03
B330016D10Ri	1555	0.124264887	13924	7.957504E-02
B3galnt1	*1556*	0.022163003	6598	7.554161E-01
B3galnt2	1557	0.048965952	8218	4.911028E-01
B3galt1	1558	0.140716965	15003	4.687090E-02
B3galt2	*1559*	-0.011224063	4696	8.746599E-01
B3galt4	1560		#N/A	1.000000E+00
B3galt5	1561	0.009852849	5766	8.898687E-01
B3galt6	*1562*	0.114911972	13314	1.051708E-01
B3galtl	1563	0.021220644	6451	7.655065E-01
B3gat1	1564	0.022198316	6629.5	7.550388E-01
B3gat2	*1565*	0.15383339	15880	2.964072E-02
B3gat3	1566	-0.011610377	4683	8.703831E-01
B3gnt1	1567	-0.06094758	1385	3.912660E-01
B3gnt2	*1568*		#N/A	1.000000E+00
B3gnt3	1569	-0.077394997	736.5	2.760195E-01
B3gnt4	1570		#N/A	1.000000E+00
B3gnt5	*1571*	0.195074164	18049	5.638117E-03
B3gnt6	1572		#N/A	1.000000E+00
B3gnt7	1573	0.058436673	9120	4.111099E-01
B3gnt8	*1574*		#N/A	1.000000E+00
B3gnt9	1575		#N/A	1.000000E+00
B3gntl1	1576	-0.047851223	2241	5.010349E-01
B430010I23Rik	*1577*	0.071667294	9964.5	3.132274E-01
B430119L08Rik	1578		#N/A	1.000000E+00
B430202K04Ril	1579		#N/A	1.000000E+00
B430203G13Ri	*1580*		#N/A	1.000000E+00
B430212C06Ril	1581	-0.054451513	1703	4.437940E-01
B430218F22Rik	1582		#N/A	1.000000E+00
B430219N15Ri	*1583*		#N/A	1.000000E+00
B430305J03Rik	1584	-0.038406171	3065	5.892384E-01
B430306N03Ri	1585	0.133202515	14492	6.006063E-02
B430319F04Rik	*1586*		#N/A	1.000000E+00
B430319H21Ri	1587	-0.038406171	3065	5.892384E-01
B4galnt1	1588	0.07264705	10039	3.066407E-01
B4galnt2	*1589*	-0.066856562	1139	3.468991E-01
B4galnt3	1590	0.05227167	8488	4.622772E-01

Spearman Rank correlation analysis performed between Prdm1 and all-expressed genes within the Meredith RNA-seq dataset. Robust Prdm1–associated genes were identified using a cut-off of $p < 0.0005$.

Table S1, Related to Supplemental Figure 3C. Prdm1 associated genes

B4galnt4	1591		#N/A	1.000000E+00
B4galt1	*1592*	0.104180464	12305	1.420778E-01
B4galt2	1593	-0.054450825	1984	4.437997E-01
B4galt3	1594	0.157146259	16115	2.626443E-02
B4galt4	*1595*	0.093730544	11630	1.867851E-01
B4galt5	1596	0.067783549	9688	3.402391E-01
B4galt6	1597	0.191772439	17903	6.520925E-03
B4galt7	*1598*	0.051928084	8466	4.652290E-01
B530045E10Ril	1599	-0.038406171	3065	5.892384E-01
B630005N14Ri	1600	0.394929297	20027	7.160792E-09
B630019A10Ril	*1601*	-0.084142573	588	2.361761E-01
B630019K06Ril	1602	0.139966802	14950	4.806833E-02
B930036N10Ri	1603		#N/A	1.000000E+00
B930041F14Rik	*1604*	0.136699589	14741	5.358488E-02
B930078G14Ri	1605		#N/A	1.000000E+00
B930086A06Ril	1606		#N/A	1.000000E+00
B930094E09Ril	*1607*		#N/A	1.000000E+00
B930095G15Ri	1608	-0.050739304	2187	4.755215E-01
B9d1	1609	-0.074526674	894	2.942613E-01
B9d2	*1610*	0.046229613	8029	5.156695E-01
Baalc	1611	0.053805701	8820	4.492258E-01
Baat	1612	0.012766329	5917	8.576089E-01
Babam1	*1613*	0.081724772	10722	2.499611E-01
Bace1	1614	0.147187013	15488	3.754088E-02
Bace2	1615	0.079193438	10541	2.649807E-01
Bach1	*1616*	0.079644739	10571	2.622587E-01
Bach2	1617	0.202011352	18326	4.122936E-03
Bad	1618	0.13198167	14406	6.246773E-02
Bag1	*1619*	-0.018769487	4294	7.919340E-01
Bag2	1620	-0.066858259	1029.5	3.468868E-01
Bag3	1621	0.056068266	8989	4.303602E-01
Bag4	*1622*	0.125821883	14026	7.584857E-02
Bag5	1623	-0.062684079	1368	3.778862E-01
Bag6	1624	0.154417196	15917	2.902016E-02
Bahcc1	*1625*	0.069013241	9778	3.315307E-01
Bahd1	1626	0.069342385	9801	3.292242E-01
Bai1	1627	-0.017624044	4385	8.043676E-01
Bai2	*1628*	-0.054450825	1984	4.437997E-01
Bai3	1629	0.187055621	17718	7.996060E-03
Baiap2	1630	0.232880792	19228	9.049110E-04
Baiap2l1	*1631*	0.03921634	7589	5.814015E-01
Baiap2l2	1632	-0.077393517	797	2.760287E-01
Baiap3	1633	0.022198316	6629.5	7.550388E-01

Spearman Rank correlation analysis performed between Prdm1 and all-expressed genes within the Meredith RNA-seq dataset. Robust Prdm1–associated genes were identified using a cut-off of $p < 0.0005$.

Table S1, Related to Supplemental Figure 3C. Prdm1 associated genes

Bak1	*1634*	0.115801924	13380	1.024859E-01
Bambi	1635	0.119280226	13607	9.250775E-02
Bambi-ps1	1636		#N/A	1.000000E+00
Banf1	*1637*	0.229206018	19127	1.095732E-03
Banf2	1638		#N/A	1.000000E+00
Bank1	1639	0.017234173	6186	8.086110E-01
Banp	*1640*		#N/A	1.000000E+00
Bap1	1641	0.136879894	14758	5.326733E-02
Bard1	1642	0.228777051	19107	1.120265E-03
Barhl1	*1643*	0.146352159	15438	3.864880E-02
Barhl2	1644	0.101532815	12149	1.525477E-01
Barx1	1645	-0.035660602	3697	6.161491E-01
Barx2	*1646*	0.19182299	17906	6.506527E-03
Basp1	1647	0.093162236	11594	1.894830E-01
Batf	1648	-0.066410059	1291	3.501362E-01
Batf2	*1649*	-0.032450477	3820	6.482730E-01
Batf3	1650	0.064221989	9487	3.662732E-01
Bax	1651	0.115644784	13368	1.029560E-01
Baz1a	*1652*	0.201404368	18305	4.239099E-03
Baz1b	1653	0.047997509	8150	4.997255E-01
Baz2a	1654	0.145285988	15370	4.010367E-02
Baz2b	*1655*	0.047212214	8098	5.067756E-01
BB014433	1656		#N/A	1.000000E+00
BB019430	1657	-0.054450825	1984	4.437997E-01
BB031773	*1658*	0.160304018	16323	2.335876E-02
BB114351	1659		#N/A	1.000000E+00
BB218582	1660		#N/A	1.000000E+00
BB287469	*1661*		#N/A	1.000000E+00
BB365896	1662	0.038758411	7558.5	5.858252E-01
BB557941	1663	0.012838608	5920	8.568113E-01
Bbc3	*1664*	0.240685037	19374	5.967825E-04
Bbip1	1665	0.018091957	6226	7.992823E-01
Bbox1	1666	-0.086746694	532	2.219355E-01
Bbs1	*1667*	0.015243007	6083	8.303678E-01
Bbs10	1668	-0.05452075	1534	4.432138E-01
Bbs12	1669	0.15252299	15793	3.107495E-02
Bbs2	*1670*	-0.130920294	57	6.462424E-02
Bbs4	1671	-0.030051751	3924	6.727130E-01
Bbs5	1672	0.072737723	10042	3.060358E-01
Bbs7	*1673*	0.091207106	11455	1.989820E-01
Bbs9	1674	0.174000363	17127	1.373428E-02
Bbx	1675	0.126915486	14104	7.331664E-02
BC001981	*1676*		#N/A	1.000000E+00

Spearman Rank correlation analysis performed between Prdm1 and all-expressed genes within the Meredith RNA-seq dataset. Robust Prdm1–associated genes were identified using a cut-off of $p < 0.0005$.

Table S1, Related to Supplemental Figure 3C. Prdm1 associated genes

BC002059	1677	0.1544596	15936.5	2.897553E-02
BC002163	1678		#N/A	1.000000E+00
BC003331	*1679*	0.17265018	17059	1.449627E-02
BC003965	1680	0.049529101	8256	4.861255E-01
BC004004	1681	0.08223874	10762	2.469850E-01
BC005537	*1682*	0.058555547	9132	4.101573E-01
BC005561	1683	0.096969	11834	1.719470E-01
BC005624	1684	0.004492868	5538	9.496544E-01
BC005764	*1685*	0.113548679	13094	1.093908E-01
BC006965	1686	0.186429969	17698	8.212505E-03
BC007180	1687	-0.044084755	2367.5	5.353590E-01
BC016423	*1688*	0.098195988	11931	1.665600E-01
BC016548	1689		#N/A	1.000000E+00
BC016579	1690	-0.052932887	2143	4.566261E-01
BC017158	*1691*	0.135992952	14700	5.484456E-02
BC017643	1692	0.116204061	13409	1.012906E-01
BC018242	1693	0.040426146	7657	5.697899E-01
BC018473	*1694*	0.081337757	10689	2.522184E-01
BC018507	1695	0.082423957	10782	2.459186E-01
BC021614	1696	0.012152761	5883	8.643849E-01
BC021767	*1697*		#N/A	1.000000E+00
BC021785	1698	0.123772846	13890	8.078292E-02
BC021891	1699	0.152231315	15781	3.140211E-02
BC022687	*1700*		#N/A	1.000000E+00
BC022960	1701	-0.04310579	2401	5.444691E-01
BC023105	1702		#N/A	1.000000E+00
BC023719	*1703*	0.133546478	14511	5.939640E-02
BC023829	1704	0.08230197	10776	2.466206E-01
BC024139	1705	-0.038406171	3065	5.892384E-01
BC024582	*1706*	0.123931888	13906	8.039090E-02
BC024978	1707	-0.054450825	1984	4.437997E-01
BC025446	1708	0.160304018	16323	2.335876E-02
BC025920	*1709*	0.037566564	7462	5.974106E-01
BC026585	1710	0.107563085	12527	1.295096E-01
BC026590	1711	0.017088341	6177	8.101997E-01
BC027072	*1712*		#N/A	1.000000E+00
BC027231	1713	-0.059110554	1412	4.057268E-01
BC027582	1714		#N/A	1.000000E+00
BC028471	*1715*		#N/A	1.000000E+00
BC028528	1716	0.027299729	6944	7.011800E-01
BC028777	1717	-0.054450825	1984	4.437997E-01
BC029214	*1718*	0.206950947	18491	3.279142E-03
BC029722	1719	0.09211814	11526	1.945136E-01

Spearman Rank correlation analysis performed between Prdm1 and all-expressed genes within the Meredith RNA-seq dataset. Robust Prdm1–associated genes were identified using a cut-off of $p < 0.0005$.

Table S1, Related to Supplemental Figure 3C. Prdm1 associated genes

BC030307	1720	0.224628469	19035	1.384898E-03
BC030336	*1721*	0.02010988	6335	7.774508E-01
BC030476	1722	0.155651362	16013	2.774476E-02
BC030499	1723		#N/A	1.000000E+00
BC030500	*1724*	0.053267784	8685	4.537786E-01
BC030867	1725	0.051639438	8442	4.677167E-01
BC030870	1726	-0.041867883	2436	5.560973E-01
BC031181	*1727*	-0.021932148	4174	7.578842E-01
BC033916	1728	0.024627122	6797	7.292303E-01
BC034090	1729	-0.04456044	2352	5.309601E-01
BC034902	*1730*	-0.054451513	1703	4.437940E-01
BC035044	1731	0.122097983	13793	8.500559E-02
BC035947	1732	0.156129434	16056.5	2.726377E-02
BC037032	*1733*	0.22382035	19013	1.442673E-03
BC037034	1734	0.055605005	8962	4.341853E-01
BC039771	1735	0.05131611	8415	4.705120E-01
BC039966	*1736*		#N/A	1.000000E+00
BC042782	1737	0.080541011	10635	2.569098E-01
BC043934	1738		#N/A	1.000000E+00
BC046251	*1739*	-0.038406171	3065	5.892384E-01
BC046401	1740		#N/A	1.000000E+00
BC048403	1741	0.099915259	12061	1.592249E-01
BC048502	*1742*	0.225070239	19043	1.354217E-03
BC048507	1743		#N/A	1.000000E+00
BC048546	1744	0.056694516	9024	4.252202E-01
BC048562	*1745*	0.143605682	15224	4.249002E-02
BC048609	1746		#N/A	1.000000E+00
BC048644	1747		#N/A	1.000000E+00
BC048671	*1748*		#N/A	1.000000E+00
BC048679	1749		#N/A	1.000000E+00
BC048943	1750		#N/A	1.000000E+00
BC049352	*1751*	-0.056977727	1457	4.229074E-01
BC049635	1752	0.021316405	6469	7.644793E-01
BC049702	1753		#N/A	1.000000E+00
BC049715	*1754*		#N/A	1.000000E+00
BC049730	1755		#N/A	1.000000E+00
BC049762	1756		#N/A	1.000000E+00
BC050972	*1757*	-0.038406171	3065	5.892384E-01
BC051019	1758	-0.054451513	1703	4.437940E-01
BC051070	1759		#N/A	1.000000E+00
BC051076	*1760*	0.113548679	13094	1.093908E-01
BC051142	1761	-0.004355113	4973	9.511961E-01
BC051226	1762	0.021317126	6513.5	7.644715E-01

Spearman Rank correlation analysis performed between Prdm1 and all-expressed genes within the Meredith RNA-seq dataset. Robust Prdm1–associated genes were identified using a cut-off of $p < 0.0005$.

Table S1, Related to Supplemental Figure 3C. Prdm1 associated genes

BC051628	*1763*	0.160304018	16323	2.335876E-02
BC051665	1764	-0.054450825	1984	4.437997E-01
BC052040	1765	-0.152626439	25	3.095962E-02
BC052486	*1766*		#N/A	1.000000E+00
BC053393	1767	0.005798839	5586	9.350486E-01
BC053749	1768	-0.077397959	691.5	2.760011E-01
BC055111	*1769*	0.163311687	16560	2.085307E-02
BC055324	1770	0.080003672	10591	2.601075E-01
BC056474	1771	0.155977269	16037	2.741608E-02
BC061194	*1772*	0.053267784	8685	4.537786E-01
BC061195	1773		#N/A	1.000000E+00
BC061212	1774		#N/A	1.000000E+00
BC061237	*1775*	-0.038406171	3065	5.892384E-01
BC064078	1776	0.212122869	18653	2.565909E-03
BC065397	1777	0.37701417	20022	3.738483E-08
BC067074	*1778*	0.00043872	5339	9.950806E-01
BC068157	1779		#N/A	1.000000E+00
BC068281	1780	0.115694057	13375	1.028084E-01
BC080695	*1781*	-0.038406171	3065	5.892384E-01
BC089491	1782		#N/A	1.000000E+00
BC089597	1783	0.00030705	5316.5	9.965570E-01
BC094916	*1784*	0.139810206	14941	4.832148E-02
BC096441	1785		#N/A	1.000000E+00
Bc1	1786	-0.038406171	3065	5.892384E-01
BC100451	*1787*		#N/A	1.000000E+00
BC100530	1788	0.258895509	19613	2.141605E-04
BC106179	1789	0.038758411	7558.5	5.858252E-01
BC107364	*1790*	0.082762295	10811.5	2.439788E-01
BC117090	1791		#N/A	1.000000E+00
BC147527	1792	0.173094386	17084	1.424159E-02
Bcam	*1793*	0.017068952	6174	8.104110E-01
Bcan	1794	0.051002816	8384	4.732293E-01
Bcap29	1795	0.071314894	9938	3.156189E-01
Bcap31	*1796*	0.092426896	11544	1.930160E-01
Bcar1	1797	0.089423966	11303	2.079424E-01
Bcar3	1798	0.140723715	15004	4.686024E-02
Bcas1	*1799*	0.275027213	19760	8.101739E-05
Bcas2	1800	0.049753356	8274	4.841510E-01
Bcas3	1801	0.175066282	17167	1.315774E-02
Bcat1	*1802*	0.143064784	15144	4.328312E-02
Bcat2	1803	0.175929018	17217	1.270676E-02
Bccip	1804	0.176918321	17266	1.220638E-02
Bcdin3d	*1805*	-0.018790377	4285	7.917077E-01

Spearman Rank correlation analysis performed between Prdm1 and all-expressed genes within the Meredith RNA-seq dataset. Robust Prdm1–associated genes were identified using a cut-off of $p < 0.0005$.

Table S1, Related to Supplemental Figure 3C. Prdm1 associated genes

Bche	1806	0.156078818	16042	2.731435E-02
Bckdha	1807	0.037442509	7453	5.986224E-01
Bckdhb	*1808*	0.216890001	18817	2.036431E-03
Bckdk	1809	0.172592546	17053	1.452961E-02
Bcl10	1810	0.084042836	10922	2.367339E-01
Bcl11a	*1811*	0.203042314	18359	3.932189E-03
Bcl11b	1812	-0.06093662	1386	3.913514E-01
Bcl2	1813	0.150389672	15672	3.353624E-02
Bcl2a1a	*1814*	0.080180281	10611	2.590535E-01
Bcl2a1b	1815		#N/A	1.000000E+00
Bcl2a1c	1816	0.004267797	5529	9.521734E-01
Bcl2a1d	*1817*	-0.069442086	953	3.285276E-01
Bcl2l1	1818	0.112921208	12918	1.113772E-01
Bcl2l10	1819		#N/A	1.000000E+00
Bcl2l11	*1820*	0.166104224	16712	1.873824E-02
Bcl2l12	1821	0.213975622	18712	2.346833E-03
Bcl2l13	1822	-0.012368137	4654	8.620052E-01
Bcl2l14	*1823*	0.090547588	11382	2.022629E-01
Bcl2l15	1824	0.346624429	20002	4.955190E-07
Bcl2l2	1825		#N/A	1.000000E+00
Bcl3	*1826*	0.162141866	16474	2.179857E-02
Bcl6	1827	0.094073727	11647	1.851696E-01
Bcl6b	1828	0.240618115	19371	5.989512E-04
Bcl7a	*1829*	0.149082612	15595	3.512452E-02
Bcl7b	1830	0.004326664	5532	9.515145E-01
Bcl7c	1831	0.131733601	14387	6.296639E-02
Bcl9	*1832*	0.079446563	10559	2.634516E-01
Bcl9l	1833	0.16504086	16663	1.952051E-02
Bclaf1	1834	0.10880851	12612	1.251043E-01
Bcmo1	*1835*	0.096860935	11823	1.724276E-01
Bco2	1836	0.050540156	8340	4.772577E-01
Bcor	1837	0.106788053	12481	1.323107E-01
Bcorl1	*1838*	0.11297177	12926	1.112161E-01
Bcr	1839	0.000177476	5300	9.980100E-01
Bcs1l	1840	0.169425059	16900	1.646783E-02
Bdh1	*1841*	0.116274528	13412	1.010823E-01
Bdh2	1842	0.050544302	8344.5	4.772215E-01
Bdkrb1	1843	0.044321826	7884	5.331644E-01
Bdkrb2	*1844*	-0.078279033	662	2.705550E-01
Bdnf	1845	0.032611887	7212	6.466415E-01
Bdp1	1846	0.188553615	17782	7.498293E-03
Bean1	*1847*	0.212248203	18661	2.550523E-03
Becn1	1848	0.172855345	17070	1.437815E-02

Spearman Rank correlation analysis performed between Prdm1 and all-expressed genes within the Meredith RNA-seq dataset. Robust Prdm1–associated genes were identified using a cut-off of $p < 0.0005$.

Table S1, Related to Supplemental Figure 3C. Prdm1 associated genes

Begain	1849	0.160985049	16395	2.276975E-02
Bend3	*1850*	-0.09526621	368.5	1.796359E-01
Bend4	1851	0.193611138	17976	6.015169E-03
Bend5	1852	0.134524399	14591	5.754091E-02
Bend6	*1853*	0.151812785	15753	3.187668E-02
Bend7	1854	0.110428502	12749	1.195487E-01
Best1	1855	-0.054451513	1703	4.437940E-01
Best2	*1856*	0.113548679	13094	1.093908E-01
Best3	1857	0.081085405	10671.5	2.536978E-01
Best4-ps	1858		#N/A	1.000000E+00
Bet1	*1859*	0.160380369	16357	2.329208E-02
Bet1l	1860	0.207115466	18496	3.253941E-03
Bet3l	1861	0.117939373	13521	9.625840E-02
Bex1	*1862*	0.031633724	7168.5	6.565542E-01
Bex2	1863	-0.040797222	2455	5.662505E-01
Bex4	1864	-0.018790377	4285	7.917077E-01
Bex6	*1865*		#N/A	1.000000E+00
Bfar	1866	0.111490868	12820	1.160108E-01
Bfsp1	1867		#N/A	1.000000E+00
Bfsp2	*1868*	0.127275861	14133	7.249748E-02
Bglap	1869	0.046719608	8061	5.112243E-01
Bglap2	1870		#N/A	1.000000E+00
Bglap3	*1871*	-0.006695974	4861	9.250274E-01
Bgn	1872	0.08009402	10599	2.595679E-01
Bhlha15	1873	0.432830413	20030	1.543993E-10
Bhlha9	*1874*		#N/A	1.000000E+00
Bhlhb9	1875	-0.035422073	3702	6.185121E-01
Bhlhe22	1876	-0.086746694	532	2.219355E-01
Bhlhe23	*1877*	0.160987082	16412.5	2.276801E-02
Bhlhe40	1878	0.037874969	7478	5.944028E-01
Bhlhe41	1879	0.214513097	18728	2.286541E-03
Bhmt	*1880*	0.113748575	13240	1.087638E-01
Bhmt-ps1	1881		#N/A	1.000000E+00
Bhmt2	1882		#N/A	1.000000E+00
Bicc1	*1883*	0.000390886	5336	9.956170E-01
Bicd1	1884	0.037424115	7450	5.988021E-01
Bicd2	1885	0.112306013	12879	1.133520E-01
Bid	*1886*	0.219470343	18886	1.793322E-03
Bik	1887	0.008843101	5718	9.010945E-01
Bin1	1888	-0.093672939	381	1.870572E-01
Bin2	*1889*	0.231391667	19195	9.782177E-04
Bin3	1890	0.124503125	13939	7.899546E-02
Birc2	1891	0.212078899	18652	2.571326E-03

Spearman Rank correlation analysis performed between Prdm1 and all-expressed genes within the Meredith RNA-seq dataset. Robust Prdm1–associated genes were identified using a cut-off of $p < 0.0005$.

Table S1, Related to Supplemental Figure 3C. Prdm1 associated genes

Birc3	*1892*	0.198843115	18192	4.762379E-03
Birc5	1893	0.133683345	14527	5.913378E-02
Birc6	1894	0.22726422	19083	1.210861E-03
Birc7	*1895*	-0.035959028	3686	6.131982E-01
Bivm	1896	-0.024404057	4121	7.315884E-01
Blcap	1897	-0.076887693	845	2.791888E-01
Blk	*1898*	0.117859898	13517	9.648445E-02
Blm	1899	0.133193459	14490	6.007820E-02
Blmh	1900	-0.06826108	965	3.368402E-01
Blnk	*1901*	0.200502947	18263	4.417049E-03
Bloc1s1	1902	0.059302383	9176	4.042021E-01
Bloc1s2a	1903	0.021206548	6448	7.656577E-01
Bloc1s2b	*1904*	-0.038406171	3065	5.892384E-01
Bloc1s3	1905	0.110321409	12741	1.199099E-01
Bloc1s4	1906	0.030587992	7102	6.672184E-01
Bloc1s5	*1907*	0.024758572	6803	7.278419E-01
Bloc1s6	1908	0.18678858	17710	8.087815E-03
Blvra	1909	0.10477755	12341	1.397942E-01
Blvrb	*1910*	0.07075485	9891	3.194440E-01
Blzf1	1911	0.155366308	15996	2.803500E-02
Bmf	1912	0.086739883	11117	2.219719E-01
Bmi1	*1913*	0.060211057	9221	3.970261E-01
Bmp1	1914	0.058752365	9144	4.085829E-01
Bmp10	1915	-0.038406171	3065	5.892384E-01
Bmp15	*1916*		#N/A	1.000000E+00
Bmp2	1917	-0.011231622	4695	8.745762E-01
Bmp2k	1918	0.227634072	19094	1.188112E-03
Bmp3	*1919*	0.056653616	9018	4.255548E-01
Bmp4	1920	0.142231088	15095	4.452981E-02
Bmp5	1921	0.128345718	14200	7.010934E-02
Bmp6	*1922*	0.113329583	12944	1.100812E-01
Bmp7	1923	0.108281454	12568	1.269542E-01
Bmp8a	1924	-0.016619442	4453	8.153131E-01
Bmp8b	*1925*	0.112541015	12894	1.125944E-01
Bmper	1926	0.078902034	10526	2.667485E-01
Bmpr1a	1927	0.071265864	9934	3.159526E-01
Bmpr1b	*1928*	0.082921441	10823	2.430701E-01
Bmpr2	1929	0.162767216	16512	2.128863E-02
Bms1	1930	0.0508664	8365	4.744152E-01
Bmx	*1931*	-0.02568336	4078	7.180991E-01
Bmyc	1932	0.145734155	15393	3.948659E-02
Bnc1	1933	-0.049422551	2210	4.870652E-01
Bnc2	*1934*	-0.061097022	1383	3.901035E-01

Spearman Rank correlation analysis performed between Prdm1 and all-expressed genes within the Meredith RNA-seq dataset. Robust Prdm1–associated genes were identified using a cut-off of $p < 0.0005$.

Table S1, Related to Supplemental Figure 3C. Prdm1 associated genes

Bnip1	1935	0.133833346	14541	5.884706E-02
Bnip2	1936	0.121218136	13741	8.729381E-02
Bnip3	*1937*	0.028169323	6979	6.921375E-01
Bnip3l	1938	0.08394856	10918	2.372621E-01
Bnipl	1939	0.126933796	14110.5	7.327484E-02
Boc	*1940*	0.014493865	6041	8.385878E-01
Bod1	1941	0.127841011	14178	7.122784E-02
Bod1l	1942	0.192967302	17949	6.188127E-03
Bok	*1943*	0.112715046	12904	1.120360E-01
Bola1	1944	0.274528843	19753	8.356440E-05
Bola2	1945	0.023561454	6723	7.405186E-01
Bola3	*1946*	0.030760457	7114	6.654549E-01
Boll	1947	0.079925022	10588	2.605778E-01
Bop1	1948	0.079903417	10586	2.607071E-01
Bora	*1949*	0.166447088	16746	1.849188E-02
Bpgm	1950	0.127493012	14150	7.200748E-02
Bphl	1951	0.0115687	5856	8.708444E-01
Bpi	*1952*	0.077242587	10424.5	2.769691E-01
Bpifa1	1953	0.098211008	11933	1.664949E-01
Bpifa2	1954	0.01420171	6000	8.417984E-01
Bpifa3	*1955*		#N/A	1.000000E+00
Bpifa5	1956	0.021316405	6469	7.644793E-01
Bpifa6	1957	-0.001606757	5107.5	9.819849E-01
Bpifb1	*1958*	0.047870267	8136	5.008643E-01
Bpifb2	1959	0.11037053	12744.5	1.197441E-01
Bpifb3	1960	0.053805701	8820	4.492258E-01
Bpifb4	*1961*	-0.014457256	4544	8.389900E-01
Bpifb5	1962	0.113548679	13094	1.093908E-01
Bpifb6	1963	0.133515162	14508	5.945662E-02
Bpifb9a	*1964*	-0.038406171	3065	5.892384E-01
Bpifb9b	1965	-0.054450825	1984	4.437997E-01
Bpifc	1966	-0.015090437	4506	8.320404E-01
Bpnt1	*1967*	0.173948143	17120	1.376308E-02
Bptf	1968	0.266445996	19689	1.369185E-04
Braf	1969	0.16712858	16776	1.801054E-02
Brap	*1970*	0.198425367	18182	4.853026E-03
Brat1	1971	0.371251774	20018	6.230586E-08
Brca1	1972	0.070778965	9894	3.192787E-01
Brca2	*1973*	0.154953508	15975	2.845991E-02
Brcc3	1974	0.21981981	18897	1.762515E-03
Brd1	1975	0.173993428	17125	1.373810E-02
Brd2	*1976*	0.251181866	19521	3.336420E-04
Brd3	1977	0.302381398	19913	1.350087E-05

Spearman Rank correlation analysis performed between Prdm1 and all-expressed genes within the Meredith RNA-seq dataset. Robust Prdm1–associated genes were identified using a cut-off of $p < 0.0005$.

Table S1, Related to Supplemental Figure 3C. Prdm1 associated genes

Brd4	1978	0.254929348	19565	2.694529E-04
Brd7	*1979*	0.161161175	16431	2.261952E-02
Brd8	1980	0.190495185	17864	6.894251E-03
Brd9	1981	0.049967456	8294	4.822700E-01
Brdt	*1982*	0.112746182	12905	1.119363E-01
Bre	1983	0.222780832	18976	1.520233E-03
Brf1	1984	-0.098724278	288	1.642798E-01
Brf2	*1985*	0.084809642	10973	2.324685E-01
Bri3	1986	0.230787827	19183	1.009469E-03
Bri3bp	1987	0.055565018	8957	4.345164E-01
Bricd5	*1988*		#N/A	1.000000E+00
Brip1	1989	0.086712731	11114	2.221172E-01
Brix1	1990	-0.011951751	4671	8.666070E-01
Brk1	*1991*	0.129169165	14250	6.831519E-02
Brms1	1992	-0.045141331	2330	5.256130E-01
Brms1l	1993	0.048709108	8196	4.933819E-01
Brox	*1994*	0.149466679	15617	3.465134E-02
Brpf1	1995	0.114396558	13281	1.067509E-01
Brpf3	1996	0.143533374	15162	4.259533E-02
Brs3	*1997*		#N/A	1.000000E+00
Brsk1	1998	-0.045149205	2323	5.255407E-01
Brsk2	1999	0.116483788	13430	1.004656E-01
Brwd1	*2000*	0.17679232	17256	1.226913E-02
Brwd3	2001	0.110290513	12737	1.200143E-01
Bscl2	2002	0.108415289	12577	1.264825E-01
Bsdc1	*2003*	0.022311228	6651	7.538327E-01
Bsg	2004	0.258973284	19615	2.131904E-04
Bsn	2005	0.127592132	14160	7.178472E-02
Bsnd	*2006*		#N/A	1.000000E+00
Bsph1	2007	0.119462067	13624	9.200819E-02
Bsph2	2008	0.022198316	6629.5	7.550388E-01
Bspry	*2009*	0.068355238	9730	3.361726E-01
Bst1	2010	0.038256775	7507	5.906887E-01
Bst2	2011	0.148784397	15580	3.549569E-02
Bsx	*2012*	-0.054451513	1703	4.437940E-01
Btaf1	2013	0.13597057	14697	5.488486E-02
Btbd1	2014	0.295546565	19888	2.149548E-05
Btbd10	*2015*	0.12081257	13712	8.836505E-02
Btbd11	2016	0.06249932	9356	3.792963E-01
Btbd16	2017	0.061296112	9282	3.885579E-01
Btbd17	*2018*	0.051226597	8402	4.712875E-01
Btbd19	2019	0.200320451	18253	4.453883E-03
Btbd2	2020	-0.016617855	4454	8.153304E-01

Spearman Rank correlation analysis performed between Prdm1 and all-expressed genes within the Meredith RNA-seq dataset. Robust Prdm1–associated genes were identified using a cut-off of $p < 0.0005$.

Table S1, Related to Supplemental Figure 3C. Prdm1 associated genes

Btbd3	2021	0.107094354	12502	1.311981E-01
Btbd6	2022	-0.004525938	4966	9.492843E-01
Btbd7	2023	0.122621961	13822	8.366591E-02
Btbd8	2024	0.062274458	9342	3.810169E-01
Btbd9	2025	0.220069911	18902	1.740765E-03
Btc	2026	0.124500454	13938	7.900194E-02
Btd	2027	0.169510793	16903	1.641254E-02
Btf3	2028	0.118793504	13572	9.385552E-02
Btf3l4	2029	-0.018239827	4330	7.976770E-01
Btg1	2030	0.131902399	14401	6.262672E-02
Btg2	2031	0.219974009	18899	1.749076E-03
Btg3	2032	0.24069692	19375	5.963981E-04
Btg4	2033		#N/A	1.000000E+00
Btk	2034	0.021694935	6561	7.604229E-01
Btla	2035	0.199946652	18241	4.530192E-03
Btn1a1	2036	-0.077397959	691.5	2.760011E-01
Btn2a2	2037		#N/A	1.000000E+00
Btnl1	2038	-0.066855996	1239.5	3.469032E-01
Btnl10	2039	0.021317126	6513.5	7.644715E-01
Btnl2	2040	0.049935541	8292	4.825501E-01
Btnl4	2041		#N/A	1.000000E+00
Btnl5	2042	-0.038406171	3065	5.892384E-01
Btnl6	2043		#N/A	1.000000E+00
Btnl7	2044	-0.054450825	1984	4.437997E-01
Btnl9	2045	0.230235201	19161.5	1.038871E-03
Btrc	2046	0.065070705	9539	3.599600E-01
Bub1	2047	0.131692731	14384	6.304886E-02
Bub1b	2048	0.184680486	17624	8.845612E-03
Bub3	2049	0.304091358	19917	1.199575E-05
Bud13	2050	0.048339595	8164	4.966706E-01
Bud31	2051	0.064579722	9510	3.636039E-01
Bves	2052	0.197412985	18138	5.079161E-03
BX284639.1	2053		#N/A	1.000000E+00
BX537302.1	2054		#N/A	1.000000E+00
BX890553.1	2055		#N/A	1.000000E+00
Bysl	2056	0.178283606	17318	1.154432E-02
Bzrap1	2057	0.033122587	7247	6.414905E-01
Bzw1	2058	0.270890072	19724	1.045666E-04
Bzw2	2059	-0.050664917	2189	4.761696E-01
C030005K06Ril	2060		#N/A	1.000000E+00
C030005K15Ril	2061	-0.038406171	3065	5.892384E-01
C030006K11Ril	2062		#N/A	1.000000E+00
C030013C21Ril	2063		#N/A	1.000000E+00

Spearman Rank correlation analysis performed between Prdm1 and all-expressed genes within the Meredith RNA-seq dataset. Robust Prdm1–associated genes were identified using a cut-off of $p < 0.0005$.

Table S1, Related to Supplemental Figure 3C. Prdm1 associated genes

C030013D06Ri	2064		#N/A	1.000000E+00
C030014I23Rik	2065	-0.038406171	3065	5.892384E-01
C030017D09Ri	*2066*		#N/A	1.000000E+00
C030017K20Ril	2067	-0.066856562	1139	3.468991E-01
C030034I22Rik	2068	-0.054450825	1984	4.437997E-01
C030037D09Ri	*2069*	-0.090864643	409	2.006808E-01
C030037F17Rik	2070		#N/A	1.000000E+00
C030039L03Rik	2071	-0.117594402	114	9.724263E-02
C030046E11Rik	*2072*	0.144410234	15331	4.133294E-02
C030047K22Ril	2073	0.036718426	7416	6.057174E-01
C030048H21Ri	2074	-0.038406171	3065	5.892384E-01
C130021I20Rik	*2075*		#N/A	1.000000E+00
C130026I21Rik	2076	-0.012924566	4624	8.558630E-01
C130026L21Rik	2077		#N/A	1.000000E+00
C130032M10R	*2078*		#N/A	1.000000E+00
C130036L24Rik	2079	0.107442763	12520	1.299414E-01
C130040N14Ri	2080		#N/A	1.000000E+00
C130046K22Ril	*2081*	0.074307668	10164	2.956863E-01
C130050O18Ri	2082	-0.038406171	3065	5.892384E-01
C130060K24Ril	2083	0.053267784	8685	4.537786E-01
C130071C03Ril	*2084*	-0.046020353	2287	5.175739E-01
C130073F10Rik	2085	0.053267784	8685	4.537786E-01
C130074G19Ri	2086	-0.015147598	4503	8.314137E-01
C130079G13Ri	*2087*	0.297119942	19891	1.933312E-05
C130080G10Ri	2088		#N/A	1.000000E+00
C1d	2089	0.133048883	14479	6.035927E-02
C1galt1	*2090*	0.090157259	11362	2.042231E-01
C1galt1c1	2091	0.067060147	9650	3.454294E-01
C1qa	2092	0.067355949	9664	3.433010E-01
C1qb	*2093*	-0.07390252	904	2.983346E-01
C1qbp	2094	0.09748001	11873	1.696880E-01
C1qc	2095	0.11307379	12932	1.108916E-01
C1ql1	*2096*		#N/A	1.000000E+00
C1ql2	2097		#N/A	1.000000E+00
C1ql3	2098	0.196907797	18120	5.195503E-03
C1ql4	*2099*		#N/A	1.000000E+00
C1qtnf1	2100	0.064668769	9516.5	3.629413E-01
C1qtnf2	2101		#N/A	1.000000E+00
C1qtnf3	*2102*	-0.029715959	3940	6.761626E-01
C1qtnf4	2103	0.083645335	10891	2.389665E-01
C1qtnf5	2104		#N/A	1.000000E+00
C1qtnf6	*2105*	-0.0123722	4653	8.619603E-01
C1qtnf7	2106	-0.031504378	3860.5	6.578695E-01

Spearman Rank correlation analysis performed between Prdm1 and all-expressed genes within the Meredith RNA-seq dataset. Robust Prdm1–associated genes were identified using a cut-off of $p < 0.0005$.

Table S1, Related to Supplemental Figure 3C. Prdm1 associated genes

C1qtnf9	2107	0.1544596	15936.5	2.897553E-02
C1ra	*2108*	-0.008126314	4817	9.090753E-01
C1rl	2109	0.283561606	19822	4.724315E-05
C1s	2110	0.189933863	17838	7.064262E-03
C2	*2111*	0.1215893	13763	8.632258E-02
C230004F18Rik	2112	-0.054451513	1703	4.437940E-01
C230012O17Ri	2113	-0.054450825	1984	4.437997E-01
C230014O12Ri	*2114*	0.128611054	14218	6.952707E-02
C230029F24Rik	2115	0.026285635	6885	7.117783E-01
C230030N03Ri	2116		#N/A	1.000000E+00
C230034O21Ri	*2117*		#N/A	1.000000E+00
C230035I16Rik	2118		#N/A	1.000000E+00
C230037L18Rik	2119	0.053805701	8820	4.492258E-01
C230038L03Rik	*2120*		#N/A	1.000000E+00
C230052I12Rik	2121	-0.079872507	646	2.608922E-01
C230062I16Rik	2122		#N/A	1.000000E+00
C230066G23Ri	*2123*		#N/A	1.000000E+00
C230081A13Ril	2124	0.13866772	14863	5.020226E-02
C2cd2	2125	0.051893804	8462	4.655240E-01
C2cd2l	*2126*	0.129797163	14290	6.697222E-02
C2cd3	2127	0.238787608	19346	6.611707E-04
C2cd4a	2128		#N/A	1.000000E+00
C2cd4b	*2129*	0.032887976	7230	6.438547E-01
C2cd4c	2130	0.015676693	6106.5	8.256174E-01
C2cd4d	2131		#N/A	1.000000E+00
C2cd5	*2132*	0.128492326	14212	6.978712E-02
C3	2133	0.051901302	8463	4.654595E-01
C330005M16R	2134	0.023976542	6751.5	7.361149E-01
C330006A16Ril	*2135*	0.094591414	11678	1.827521E-01
C330006D17Ri	2136		#N/A	1.000000E+00
C330007P06Ril	2137	0.161313343	16442	2.249042E-02
C330008G21Ri	*2138*		#N/A	1.000000E+00
C330011M18R	2139		#N/A	1.000000E+00
C330013F16Rik	2140	0.00069212	5348	9.922393E-01
C330013J21Rik	*2141*	0.194131481	17998	5.878558E-03
C330018A13Ril	2142	-0.066856562	1139	3.468991E-01
C330018D20Ri	2143	0.0235855	6724	7.402632E-01
C330019F10Rik	*2144*	-0.066858259	1029.5	3.468868E-01
C330021F23Rik	2145	0.08324418	10851	2.412346E-01
C330027C09Ril	2146	0.139768057	14936	4.838981E-02
C3ar1	*2147*	0.250633682	19514	3.441423E-04
C430049B03Ril	2148	-0.038406171	3065	5.892384E-01
C4a	2149	0.247517371	19475	4.098967E-04

Spearman Rank correlation analysis performed between Prdm1 and all-expressed genes within the Meredith RNA-seq dataset. Robust Prdm1–associated genes were identified using a cut-off of $p < 0.0005$.

Table S1, Related to Supplemental Figure 3C. Prdm1 associated genes

C4b	*2150*	0.113450971	12955	1.096982E-01
C4bp	2151	0.198202869	18176	4.901936E-03
C530005A16Ril	2152	0.187373896	17736	7.887910E-03
C530008M17R	*2153*	0.154428928	15920	2.900781E-02
C530025M09R	2154		#N/A	1.000000E+00
C530030P08Ril	2155		#N/A	1.000000E+00
C5ar1	*2156*	0.280371224	19801	5.791605E-05
C5ar2	2157	0.248056187	19481	3.977531E-04
C6	2158	0.139090962	14897	4.949853E-02
C630004L07Rik	*2159*		#N/A	1.000000E+00
C630004M23R	2160	-0.038406171	3065	5.892384E-01
C630020P19Ril	2161	0.124142885	13920	7.987317E-02
C630028M04R	*2162*	-0.052956957	2140	4.564211E-01
C630043F03Rik	2163	0.156362	16074	2.703237E-02
C630050I24Rik	2164		#N/A	1.000000E+00
C7	*2165*	0.053267784	8685	4.537786E-01
C730034F03Rik	2166	0.053267784	8685	4.537786E-01
C77080	2167	0.055911325	8977	4.316538E-01
C77370	*2168*	0.095414617	11731	1.789559E-01
C78197	2169		#N/A	1.000000E+00
C78339	2170	0.032527723	7205	6.474920E-01
C79798	*2171*	0.048448014	8175.5	4.957045E-01
C86695	2172		#N/A	1.000000E+00
C87198	2173	-0.028573361	3976	6.879508E-01
C87414	*2174*	0.021712246	6564	7.602375E-01
C87436	2175	0.203122882	18364	3.917622E-03
C87499	2176	-0.038406171	3065	5.892384E-01
C87977	*2177*		#N/A	1.000000E+00
C8a	2178	0.050870414	8368.5	4.743803E-01
C8b	2179	-0.054451513	1703	4.437940E-01
C8g	*2180*	0.020875719	6412.5	7.692098E-01
C9	2181	0.10542092	12386	1.373651E-01
C920011F04Rik	2182		#N/A	1.000000E+00
C920021L13Rik	*2183*	0.11227883	12874	1.134399E-01
C920025E04Ril	2184	0.131264139	14365	6.391903E-02
CAAA0109815(	2185		#N/A	1.000000E+00
CAAA0111838:	*2186*		#N/A	1.000000E+00
CAAA0114733:	2187		#N/A	1.000000E+00
CAAA0116572(	2188		#N/A	1.000000E+00
CAAA0120120!	*2189*	0.04653282	8046	5.129165E-01
CAAA0120511:	2190		#N/A	1.000000E+00
CAAA0120511:	2191		#N/A	1.000000E+00
Caap1	*2192*	0.168872888	16870	1.682785E-02

Spearman Rank correlation analysis performed between Prdm1 and all-expressed genes within the Meredith RNA-seq dataset. Robust Prdm1–associated genes were identified using a cut-off of $p < 0.0005$.

Table S1, Related to Supplemental Figure 3C. Prdm1 associated genes

Cab39	2193	0.145734982	15394	3.948546E-02
Cab39l	2194	0.095954024	11772	1.765001E-01
Cabin1	*2195*	-0.064020205	1340	3.677841E-01
Cables1	2196	0.136712675	14743	5.356178E-02
Cables2	2197	-0.038952885	2490	5.839446E-01
Cabp1	*2198*	0.113548679	13094	1.093908E-01
CABP2	2199		#N/A	1.000000E+00
Cabp2	2200	-0.095267438	357.5	1.796303E-01
CABP4	*2201*		#N/A	1.000000E+00
Cabp4	2202	-0.033388476	3771	6.388155E-01
Cabp5	2203	-0.054451513	1703	4.437940E-01
Cabp7	*2204*	-0.054451513	1703	4.437940E-01
Cabs1	2205		#N/A	1.000000E+00
Cabyr	2206	0.066684034	9630	3.481476E-01
Cacfd1	*2207*	0.170134905	16937	1.601489E-02
Cachd1	2208	0.09578559	11751	1.772643E-01
Cacna1a	2209	0.097976411	11912	1.675147E-01
Cacna1b	*2210*	0.010737872	5808	8.800475E-01
Cacna1c	2211	0.060467503	9237	3.950148E-01
Cacna1d	2212	0.121597419	13764	8.630143E-02
Cacna1e	*2213*	0.272539291	19739	9.449976E-05
Cacna1f	2214	0.08427276	10939	2.354493E-01
Cacna1g	2215	-0.039013493	2489	5.833591E-01
Cacna1h	*2216*	0.143843749	15301	4.214484E-02
Cacna1i	2217	-0.043687937	2382.5	5.390426E-01
Cacna1s	2218	0.09570627	11749	1.776250E-01
Cacna2d1	*2219*	-0.021790481	4179	7.594000E-01
Cacna2d2	2220	-0.087370453	440	2.186170E-01
Cacna2d3	2221	0.185426276	17657	8.570616E-03
Cacna2d4	*2222*	0.380628741	20024	2.699801E-08
Cacnb1	2223	-0.064850436	1330	3.615919E-01
Cacnb2	2224	0.174428842	17142	1.349991E-02
Cacnb3	*2225*	0.136353093	14719	5.419953E-02
Cacnb4	2226	0.224302883	19026	1.407916E-03
Cacng1	2227	-0.038406171	3065	5.892384E-01
Cacng2	*2228*	0.018475335	6248	7.951221E-01
Cacng3	2229	0.093297167	11600	1.888398E-01
Cacng4	2230	0.047616944	8119	5.031356E-01
Cacng5	*2231*	0.128340356	14199	7.012114E-02
CACNG6	2232		#N/A	1.000000E+00
Cacng6	2233	0.038758411	7558.5	5.858252E-01
CACNG7	*2234*		#N/A	1.000000E+00
Cacng7	2235	0.163687615	16576	2.055686E-02

Spearman Rank correlation analysis performed between Prdm1 and all-expressed genes within the Meredith RNA-seq dataset. Robust Prdm1–associated genes were identified using a cut-off of $p < 0.0005$.

Table S1, Related to Supplemental Figure 3C. Prdm1 associated genes

CACNG8	2236		#N/A	1.000000E+00
Cacng8	2237	-0.016768635	4421	8.136853E-01
Cactin	2238	0.104235083	12310	1.418678E-01
Cacul1	2239	0.066358585	9611	3.505106E-01
Cacybp	2240	0.084446129	10951	2.344838E-01
Cad	2241	0.198917972	18195	4.746297E-03
Cadm1	2242	-0.017900518	4373	8.013619E-01
Cadm2	2243	0.062584663	9363	3.786446E-01
Cadm3	2244	0.044430029	7894	5.321643E-01
Cadm4	2245	0.231483645	19198	9.735363E-04
Cadps	2246	0.104332498	12314	1.414937E-01
Cadps2	2247	0.209744214	18568	2.874331E-03
Cage1	2248	0.071650057	9960	3.133441E-01
Calb1	2249	-0.050250995	2193	4.797849E-01
Calb2	2250	-0.035064643	3709	6.220604E-01
Calca	2251	0.027104325	6935	7.032178E-01
Calcb	2252	0.032727915	7222	6.454697E-01
Calcoco1	2253	0.053564643	8775	4.512629E-01
Calcoco2	2254	0.061348161	9289	3.881545E-01
Calcr	2255	-0.024459186	4118	7.310054E-01
Calcrl	2256	-0.002272917	5061	9.745179E-01
Cald1	2257	0.200577737	18266	4.402033E-03
Calhm1	2258		#N/A	1.000000E+00
Calhm2	2259	0.191202169	17883	6.685319E-03
Calhm3	2260		#N/A	1.000000E+00
Calm1	2261	0.102054753	12184	1.504389E-01
Calm2	2262	0.151422097	15740	3.232517E-02
Calm3	2263	0.209952506	18571	2.846040E-03
Calm4	2264	0.008861248	5721	9.008925E-01
CALM5	2265		#N/A	1.000000E+00
Calm5	2266	-0.001223987	5172.5	9.862760E-01
Calml3	2267	0.083925466	10914	2.373916E-01
Calml4	2268	0.072619803	10037	3.068227E-01
Caln1	2269	0.274638028	19754	8.300005E-05
Calr	2270	0.208749963	18542	3.012918E-03
Calr3	2271	0.109652638	12689	1.221850E-01
Calr4	2272	0.073881149	10116	2.984748E-01
Calu	2273	0.222858001	18981	1.514347E-03
Caly	2274	0.020875719	6412.5	7.692098E-01
Camk1	2275	-0.02568498	4077	7.180821E-01
Camk1d	2276	0.110542807	12762	1.191640E-01
Camk1g	2277	-0.038406171	3065	5.892384E-01
Camk2a	2278	0.026888217	6924	7.054739E-01

Spearman Rank correlation analysis performed between Prdm1 and all-expressed genes within the Meredith RNA-seq dataset. Robust Prdm1–associated genes were identified using a cut-off of $p < 0.0005$.

Table S1, Related to Supplemental Figure 3C. Prdm1 associated genes

Camk2b	*2279*	0.035418857	7345	6.185440E-01
Camk2d	2280	0.086731215	11115	2.220183E-01
Camk2g	2281	0.012858659	5921	8.565901E-01
Camk2n1	*2282*	-0.012324058	4660	8.624922E-01
Camk2n2	2283		#N/A	1.000000E+00
Camk4	2284	0.32611916	19980	2.445847E-06
Camkk1	*2285*	-0.054450825	1984	4.437997E-01
Camkk2	2286	0.211469077	18632	2.647540E-03
Camkmt	2287	0.189653763	17829	7.150486E-03
Camkv	*2288*	0.105930644	12416	1.354636E-01
Caml	2289	-0.014751333	4523	8.357607E-01
Camp	2290	0.057449274	9058	4.190730E-01
Camsap1	*2291*	0.179367252	17375	1.104156E-02
Camsap2	2292	0.105824503	12409	1.358579E-01
Camsap3	2293	-0.018447724	4312.5	7.954215E-01
Camta1	*2294*	0.169409207	16898	1.647807E-02
Camta2	2295	0.060135791	9220	3.976176E-01
Cand1	2296	0.148650607	15572	3.566329E-02
Cand2	*2297*	0.217274604	18826	1.998388E-03
Cant1	2298	0.090944916	11430	2.002817E-01
Canx	2299	0.143825407	15299	4.217136E-02
Cap1	*2300*	0.168466267	16848	1.709733E-02
Cap2	2301	-0.016101237	4468	8.209733E-01
Capg	2302	0.111816833	12838	1.149418E-01
Capn1	*2303*	0.002560998	5445	9.712895E-01
Capn10	2304	0.011761576	5865	8.687103E-01
Capn11	2305	0.050544302	8344.5	4.772215E-01
Capn12	*2306*	0.132928286	14472.5	6.059456E-02
Capn13	2307	0.090013747	11344.5	2.049472E-01
Capn2	2308	0.114142933	13262	1.075352E-01
Capn3	*2309*	0.034085846	7299	6.318216E-01
Capn5	2310	0.079734056	10574	2.617222E-01
Capn6	2311	-0.018105422	4347	7.991361E-01
Capn7	*2312*	0.131455224	14372	6.352986E-02
Capn8	2313	0.042747748	7785	5.478200E-01
Capn9	2314	-0.038406171	3065	5.892384E-01
Capns1	*2315*	0.212723727	18684	2.492908E-03
Capns2	2316		#N/A	1.000000E+00
Caprin1	2317	0.208137121	18528	3.101327E-03
Caprin2	*2318*	-0.0106716	4718	8.807824E-01
Caps2	2319	-0.00439051	4970	9.507999E-01
Capsl	2320	0.056228748	8996	4.290396E-01
Capza1	*2321*	0.187192307	17724	7.949453E-03

Spearman Rank correlation analysis performed between Prdm1 and all-expressed genes within the Meredith RNA-seq dataset. Robust Prdm1–associated genes were identified using a cut-off of $p < 0.0005$.

Table S1, Related to Supplemental Figure 3C. Prdm1 associated genes

Capza2	2322	0.211664952	18640	2.622840E-03
Capza3	2323		#N/A	1.000000E+00
Capzb	*2324*	0.118182878	13540	9.556844E-02
Car1	2325	-0.058190577	1431	4.130862E-01
Car10	2326	0.096031535	11777	1.761493E-01
Car11	*2327*		#N/A	1.000000E+00
Car12	2328	-0.064127737	1339	3.669785E-01
Car13	2329	-0.00060215	5236	9.932481E-01
Car14	*2330*	-0.038406171	3065	5.892384E-01
Car15	2331		#N/A	1.000000E+00
Car2	2332	0.014391234	6035	8.397154E-01
Car3	*2333*	-0.016731055	4437	8.140953E-01
Car4	2334	-0.066855996	1239.5	3.469032E-01
Car5a	2335	0.189835705	17833	7.094372E-03
Car5b	*2336*	0.205956149	18456	3.435319E-03
Car6	2337	-0.054450825	1984	4.437997E-01
Car7	2338	0.062609447	9366	3.784554E-01
Car8	*2339*	0.25374542	19549	2.883709E-04
Car9	2340	-0.077393517	797	2.760287E-01
Card10	2341	0.053267784	8685	4.537786E-01
Card11	*2342*	0.226273875	19075	1.273754E-03
Card14	2343	0.043994852	7864	5.361925E-01
Card6	2344	0.128938347	14235	6.881428E-02
Card9	*2345*	0.064871773	9530	3.614336E-01
Carf	2346	0.08005777	10594	2.597843E-01
Carhsp1	2347	0.040892142	7688	5.653468E-01
Carkd	*2348*	0.129825779	14291	6.691155E-02
Carm1	2349	-0.02152362	4192	7.622579E-01
CARNS1	2350		#N/A	1.000000E+00
Carns1	*2351*	-0.110563117	176	1.190958E-01
Cars	2352	0.078613483	10505	2.685070E-01
Cars2	2353	0.133935942	14547	5.865162E-02
Cartpt	*2354*	-0.077394997	736.5	2.760195E-01
Casc1	2355	0.166871358	16767	1.819092E-02
Casc3	2356	0.211500788	18633	2.643527E-03
Casc4	*2357*	0.089426059	11306	2.079318E-01
Casc5	2358	0.084672209	10965	2.332290E-01
Casd1	2359	0.155922038	16033	2.747155E-02
Cask	*2360*	0.137082215	14762	5.291287E-02
Caskin1	2361	-0.054451513	1703	4.437940E-01
Caskin2	2362	0.112234769	12869	1.135825E-01
Casp1	*2363*	0.167548448	16801	1.771944E-02
Casp12	2364	0.001540578	5392	9.827267E-01

Spearman Rank correlation analysis performed between Prdm1 and all-expressed genes within the Meredith RNA-seq dataset. Robust Prdm1–associated genes were identified using a cut-off of $p < 0.0005$.

Table S1, Related to Supplemental Figure 3C. Prdm1 associated genes

Casp14	2365	0.043936017	7859	5.367383E-01
Casp16	*2366*	-0.038406171	3065	5.892384E-01
Casp2	2367	0.169062487	16876	1.670346E-02
Casp3	2368	0.06247049	9352	3.795166E-01
Casp4	*2369*	0.170630281	16961	1.570528E-02
Casp6	2370	0.173661639	17105	1.392207E-02
Casp7	2371	0.064394753	9498	3.649825E-01
Casp8	*2372*	0.216671362	18809	2.058351E-03
Casp8ap2	2373	0.163380822	16567	2.079832E-02
Casp9	2374	-0.016999596	4414	8.111669E-01
Casq1	*2375*	0.154540397	15956.5	2.889064E-02
Casq2	2376	-0.027893871	4003.5	6.949972E-01
Casr	2377	0.167842382	16812	1.751810E-02
Cass4	*2378*	-0.029485741	3951	6.785316E-01
Cast	2379	-0.008811785	4793	9.014430E-01
Casz1	2380	0.033654072	7274	6.361481E-01
Cat	*2381*	-0.013621479	4585	8.481823E-01
Catsper1	2382	0.020875719	6412.5	7.692098E-01
Catsper2	2383	0.036638439	7410	6.065034E-01
Catsper3	*2384*	0.023910612	6743	7.368138E-01
Catsper4	2385	-0.000841282	5208.5	9.905669E-01
Catsperb	2386	-0.021709326	4183	7.602688E-01
Catsperd	*2387*	0.127888414	14184	7.112218E-02
Catsperg1	2388	0.070468735	9872	3.214097E-01
Catsperg2	2389		#N/A	1.000000E+00
Cav1	*2390*	0.051686201	8447	4.673132E-01
Cav2	2391	-0.117567396	127.5	9.732002E-02
Cav3	2392		#N/A	1.000000E+00
Cbfa2t2	*2393*	0.250811439	19517	3.407042E-04
Cbfa2t2-ps1	2394		#N/A	1.000000E+00
Cbfa2t3	2395	0.10699237	12496	1.315678E-01
Cbfb	*2396*	0.064022949	9469	3.677636E-01
Cbl	2397	0.052600479	8511	4.594622E-01
Cblb	2398	0.036233296	7392	6.104918E-01
Cblc	*2399*	0.045362169	7961	5.235874E-01
Cbll1	2400	0.245570088	19448	4.567074E-04
Cbln1	2401	0.113548679	13094	1.093908E-01
Cbln2	*2402*	-0.05944409	1406	4.030780E-01
Cbln3	2403	0.215379807	18761	2.192276E-03
Cbln4	2404	0.106443551	12457	1.335707E-01
Cbr1	*2405*	0.210860293	18606	2.725662E-03
Cbr2	2406	0.019372041	6298	7.854141E-01
Cbr3	2407	0.0681467	9711	3.376523E-01

Spearman Rank correlation analysis performed between Prdm1 and all-expressed genes within the Meredith RNA-seq dataset. Robust Prdm1–associated genes were identified using a cut-off of $p < 0.0005$.

Table S1, Related to Supplemental Figure 3C. Prdm1 associated genes

Cbr4	*2408*	-0.044929902	2337	5.275560E-01
Cbs	2409		#N/A	1.000000E+00
Cbwd1	2410	0.086614676	11110	2.226424E-01
Cbx1	*2411*	0.22198465	18957	1.582195E-03
Cbx2	2412	0.116573468	13439	1.002023E-01
Cbx3	2413	0.23495818	19271	8.110665E-04
Cbx3-ps3	*2414*		#N/A	1.000000E+00
Cbx4	2415	0.259237841	19619	2.099211E-04
Cbx5	2416	0.188237079	17773	7.601115E-03
Cbx6	*2417*		#N/A	1.000000E+00
Cbx7	2418	0.081043719	10664	2.539428E-01
Cbx8	2419	0.044705035	7913	5.296265E-01
Cby1	*2420*	-0.09352169	383	1.877733E-01
Cby3	2421		#N/A	1.000000E+00
Cc2d1a	2422	0.098363969	11940	1.658324E-01
Cc2d1b	*2423*	0.043293526	7822	5.427162E-01
Cc2d2a	2424	0.054149586	8873	4.463288E-01
Ccar1	2425	0.096646347	11818	1.733849E-01
Ccbe1	*2426*	0.029292365	7034	6.805238E-01
Ccbl1	2427	0.235555264	19279	7.858016E-04
Ccbl2	2428	-0.005922062	4881	9.336715E-01
Ccbp2	*2429*	-0.033782655	3744	6.348583E-01
Ccdc101	2430	-0.031412249	3871	6.588071E-01
Ccdc102a	2431	-0.038406171	3065	5.892384E-01
Ccdc103	*2432*		#N/A	1.000000E+00
Ccdc104	2433	0.172124238	17030	1.480296E-02
Ccdc105	2434		#N/A	1.000000E+00
Ccdc106	*2435*		#N/A	1.000000E+00
Ccdc107	2436	0.000171283	5298	9.980794E-01
Ccdc108	2437	0.136018244	14704	5.479905E-02
Ccdc109b	*2438*	-0.081210802	629	2.529619E-01
Ccdc11	2439	0.061424981	9295	3.875595E-01
Ccdc110	2440		#N/A	1.000000E+00
Ccdc111	*2441*	0.081006874	10662	2.541595E-01
Ccdc112	2442	-0.020872974	4208	7.692393E-01
Ccdc113	2443	-0.012847096	4630	8.567177E-01
Ccdc114	*2444*	0.167670613	16805	1.763552E-02
Ccdc115	2445	0.015021389	6071	8.327976E-01
Ccdc116	2446	0.16919636	16884	1.661612E-02
Ccdc117	*2447*	0.119809729	13654	9.105908E-02
Ccdc12	2448	0.040676028	7676	5.674053E-01
Ccdc120	2449	0.194378195	18017	5.814761E-03
Ccdc121	*2450*		#N/A	1.000000E+00

Spearman Rank correlation analysis performed between Prdm1 and all-expressed genes within the Meredith RNA-seq dataset. Robust Prdm1–associated genes were identified using a cut-off of $p < 0.0005$.

Table S1, Related to Supplemental Figure 3C. Prdm1 associated genes

Ccdc122	2451	0.042089948	7744	5.540026E-01
Ccdc124	2452	0.033192502	7251	6.407867E-01
Ccdc125	*2453*	0.256232761	19578	2.499643E-04
Ccdc126	2454	0.089059487	11277	2.098092E-01
Ccdc127	2455	0.080315785	10620	2.582468E-01
Ccdc129	*2456*	-0.003881933	4986	9.564931E-01
Ccdc13	2457	0.020875719	6412.5	7.692098E-01
Ccdc130	2458	0.079857473	10581	2.609822E-01
Ccdc132	*2459*	0.044624434	7908	5.303697E-01
Ccdc134	2460	0.001779633	5409	9.800469E-01
Ccdc135	2461	0.092854521	11568	1.909556E-01
Ccdc136	*2462*	0.042866343	7801.5	5.467090E-01
Ccdc137	2463	0.183124249	17553	9.444763E-03
Ccdc138	2464	0.118640087	13564	9.428356E-02
Ccdc14	*2465*	-0.030875227	3902	6.642825E-01
Ccdc141	2466	0.122379617	13809	8.428339E-02
Ccdc142	2467		#N/A	1.000000E+00
Ccdc144b	*2468*	-0.004812426	4947	9.460788E-01
Ccdc146	2469	0.128919254	14233	6.885570E-02
Ccdc147	2470	0.0094221	5743	8.946550E-01
Ccdc148	*2471*	0.260783265	19633	1.917382E-04
Ccdc149	2472	0.18078967	17439	1.041097E-02
Ccdc15	2473	0.144308904	15324	4.147720E-02
Ccdc150	*2474*	0.02402764	6756	7.355734E-01
Ccdc151	2475	0.086412184	11087.5	2.237298E-01
Ccdc152	2476	-0.038406171	3065	5.892384E-01
Ccdc153	*2477*	0.055345023	8948	4.363405E-01
Ccdc154	2478	-0.054450825	1984	4.437997E-01
Ccdc155	2479	-0.038406171	3065	5.892384E-01
Ccdc157	*2480*	0.102385002	12204	1.491161E-01
Ccdc158	2481	0.074100692	10142	2.970373E-01
Ccdc159	2482	0.012253703	5890	8.632695E-01
Ccdc160	*2483*	-0.014585286	4537	8.375838E-01
Ccdc162	2484	0.149378768	15612	3.475917E-02
Ccdc163	2485	0.107013539	12497	1.314910E-01
CCDC166	*2486*	-0.130639588	64	6.520466E-02
Ccdc166	2487		#N/A	1.000000E+00
Ccdc167	2488	0.152178751	15779	3.146138E-02
Ccdc169	*2489*	0.048672774	8193	4.937048E-01
Ccdc17	2490	0.11187381	12842	1.147558E-01
Ccdc170	2491	0.053267784	8685	4.537786E-01
Ccdc171	*2492*	0.133095052	14484	6.026940E-02
Ccdc172	2493	0.113548679	13094	1.093908E-01

Spearman Rank correlation analysis performed between Prdm1 and all-expressed genes within the Meredith RNA-seq dataset. Robust Prdm1–associated genes were identified using a cut-off of $p < 0.0005$.

Table S1, Related to Supplemental Figure 3C. Prdm1 associated genes

Ccdc173	2494	0.149779334	15637	3.427014E-02
Ccdc174	*2495*	-0.066258776	1295	3.512373E-01
Ccdc175	2496	0.172462554	17048	1.460504E-02
Ccdc176	2497	0.020414046	6346	7.741747E-01
Ccdc177	*2498*	0.120056887	13668	9.038910E-02
Ccdc178	2499	0.130757583	14339	6.496016E-02
Ccdc18	2500	0.008334778	5694	9.067533E-01
Ccdc181	*2501*	0.062339095	9345	3.805218E-01
Ccdc19	2502	-0.038406171	3065	5.892384E-01
Ccdc22	2503	-0.15959809	13	2.398311E-02
Ccdc23	*2504*	0.029163762	7026	6.818500E-01
Ccdc24	2505	-0.054450825	1984	4.437997E-01
Ccdc25	2506	-0.050391241	2190	4.785583E-01
Ccdc27	*2507*	0.143605682	15224	4.249002E-02
Ccdc28a	2508	0.036080282	7385	6.120011E-01
Ccdc28b	2509	0.036667352	7412	6.062192E-01
Ccdc3	*2510*	0.029180568	7027	6.816767E-01
Ccdc30	2511	0.241747218	19390	5.633177E-04
Ccdc32	2512	0.200630641	18270	4.391439E-03
Ccdc33	*2513*	0.092566909	11554	1.923396E-01
Ccdc34	2514	0.184430616	17615	8.939481E-03
Ccdc36	2515	0.146069833	15417	3.902966E-02
Ccdc37	*2516*	-0.054450825	1984	4.437997E-01
Ccdc38	2517	0.216516446	18803	2.074012E-03
Ccdc39	2518	-0.095269893	339	1.796190E-01
Ccdc40	*2519*	0.143605682	15224	4.249002E-02
Ccdc41	2520	0.087250798	11147	2.192508E-01
Ccdc42	2521	-0.077393517	797	2.760287E-01
Ccdc42b	*2522*		#N/A	1.000000E+00
Ccdc43	2523	-0.002834065	5040	9.682298E-01
Ccdc47	2524	0.115050507	13324	1.047492E-01
Ccdc50	*2525*	0.161590579	16454	2.225684E-02
Ccdc51	2526	0.07521908	10268	2.897860E-01
Ccdc53	2527	0.025429064	6846	7.207737E-01
Ccdc54	*2528*		#N/A	1.000000E+00
Ccdc55	2529	0.080397092	10625	2.577636E-01
Ccdc57	2530	0.120717133	13705	8.861865E-02
Ccdc58	*2531*	0.044952411	7928	5.273490E-01
Ccdc59	2532	0.120990355	13724	8.789417E-02
Ccdc6	2533	0.00189708	5416	9.787304E-01
Ccdc60	*2534*	0.120260305	13678	8.984065E-02
Ccdc61	2535	0.063277051	9410	3.733821E-01
Ccdc62	2536	0.037035813	7432	6.026028E-01

Spearman Rank correlation analysis performed between Prdm1 and all-expressed genes within the Meredith RNA-seq dataset. Robust Prdm1–associated genes were identified using a cut-off of $p < 0.0005$.

Table S1, Related to Supplemental Figure 3C. Prdm1 associated genes

Ccdc63	*2537*	-0.035812443	3690	6.146469E-01
Ccdc64	2538	0.02938571	7037	6.795619E-01
Ccdc64b	2539	0.155700209	16017	2.769529E-02
Ccdc65	*2540*	-0.065367286	1320.5	3.577700E-01
Ccdc66	2541	0.048127902	8153	4.985599E-01
Ccdc67	2542	-0.086748028	498.5	2.219283E-01
Ccdc68	*2543*	0.109492511	12666.5	1.227347E-01
Ccdc69	2544	0.019195966	6289	7.873178E-01
Ccdc7	2545	0.021462784	6550	7.629099E-01
Ccdc70	*2546*		#N/A	1.000000E+00
Ccdc71	2547	0.066538508	9622	3.492030E-01
Ccdc71l	2548	0.095158665	11708	1.801299E-01
Ccdc73	*2549*	-0.014689556	4532	8.364389E-01
Ccdc74a	2550		#N/A	1.000000E+00
Ccdc77	2551	0.253707279	19548	2.890004E-04
Ccdc78	*2552*	0.157810027	16167	2.562916E-02
Ccdc79	2553	0.070422707	9867.5	3.217267E-01
Ccdc8	2554	-0.016731055	4437	8.140953E-01
Ccdc80	*2555*	-0.017250621	4403	8.084319E-01
Ccdc81	2556	0.160987082	16412.5	2.276801E-02
Ccdc82	2557	0.010812126	5812	8.792243E-01
Ccdc83	*2558*	0.015119737	6076	8.317191E-01
Ccdc84	2559	0.174792207	17153	1.330391E-02
Ccdc85a	2560	0.080611511	10642	2.564923E-01
Ccdc85b	*2561*	0.259681353	19621	2.045449E-04
Ccdc85c	2562	-0.054451513	1703	4.437940E-01
Ccdc86	2563	0.106660005	12474	1.327780E-01
Ccdc87	*2564*	-0.066855996	1239.5	3.469032E-01
Ccdc88a	2565	0.218664994	18863	1.866196E-03
Ccdc88b	2566	-0.012164901	4665	8.642508E-01
Ccdc88c	*2567*	0.129569713	14277	6.745611E-02
Ccdc89	2568	0.113548679	13094	1.093908E-01
Ccdc9	2569	0.125812026	14024	7.587171E-02
Ccdc90b	*2570*	0.157289522	16118	2.612619E-02
Ccdc91	2571	0.087336624	11151	2.187961E-01
Ccdc92	2572	0.012063329	5879	8.653734E-01
Ccdc93	*2573*	0.091635091	11498	1.968737E-01
Ccdc94	2574	-0.056165168	1479.5	4.295625E-01
Ccdc96	2575	-0.054451513	1703	4.437940E-01
Ccdc97	*2576*	0.000561985	5345	9.936985E-01
Ccer1	2577		#N/A	1.000000E+00
Cchcr1	2578	0.108378772	12575	1.266111E-01
Ccin	*2579*		#N/A	1.000000E+00

Spearman Rank correlation analysis performed between Prdm1 and all-expressed genes within the Meredith RNA-seq dataset. Robust Prdm1–associated genes were identified using a cut-off of $p < 0.0005$.

Table S1, Related to Supplemental Figure 3C. Prdm1 associated genes

Cck	2580	0.047553853	8117	5.037021E-01
Cckar	2581	-0.017417495	4389.5	8.066150E-01
Cckbr	2582	0.069583234	9819	3.275431E-01
Ccl1	2583	-0.01155825	4686	8.709600E-01
Ccl11	2584	0.099671942	12038	1.602480E-01
Ccl12	2585	0.151119933	15719	3.267570E-02
Ccl17	2586	0.129070682	14243	6.852778E-02
Ccl19	2587	0.049794798	8275	4.837866E-01
CCL19	2588		#N/A	1.000000E+00
Ccl19-ps1	2589		#N/A	1.000000E+00
Ccl19-ps3	2590		#N/A	1.000000E+00
Ccl2	2591	-0.152977527	21	3.057090E-02
Ccl20	2592	0.084475517	10953	2.343204E-01
Ccl21a	2593	0.136866913	14757	5.329014E-02
CCL21A	2594		#N/A	1.000000E+00
Ccl21b	2595		#N/A	1.000000E+00
Ccl21c	2596		#N/A	1.000000E+00
Ccl22	2597	0.010816036	5813	8.791810E-01
Ccl24	2598	0.094766939	11688	1.819377E-01
Ccl25	2599	-0.056393236	1473	4.276885E-01
Ccl26	2600		#N/A	1.000000E+00
CCL27	2601		#N/A	1.000000E+00
Ccl27a	2602	-0.007371671	4841	9.174875E-01
Ccl27b	2603		#N/A	1.000000E+00
Ccl28	2604	-0.017867668	4375	8.017189E-01
Ccl3	2605	-0.086747139	513	2.219331E-01
Ccl4	2606		#N/A	1.000000E+00
Ccl5	2607	-0.053200001	2135	4.543541E-01
Ccl6	2608	0.077723243	10455	2.739819E-01
Ccl7	2609	-0.033388476	3771	6.388155E-01
Ccl8	2610	0.050540156	8340	4.772577E-01
Ccl9	2611	0.04870949	8197	4.933785E-01
Ccm2	2612	0.07031175	9862	3.224916E-01
Ccm2l	2613	-0.084370481	584	2.349047E-01
Ccna1	2614	-0.038406171	3065	5.892384E-01
Ccna2	2615	0.09716792	11849	1.710650E-01
Ccnb1	2616	0.026890552	6925	7.054495E-01
Ccnb1ip1	2617	-0.028762603	3970	6.859931E-01
Ccnb2	2618	0.204004265	18391	3.761421E-03
Ccnb2-ps	2619	0.193852493	17987	5.951455E-03
Ccnb3	2620	0.175971092	17219	1.268512E-02
Ccnc	2621	0.093818573	11633	1.863697E-01
Ccnd1	2622	0.187486581	17746	7.849932E-03

Spearman Rank correlation analysis performed between Prdm1 and all-expressed genes within the Meredith RNA-seq dataset. Robust Prdm1–associated genes were identified using a cut-off of $p < 0.0005$.

Table S1, Related to Supplemental Figure 3C. Prdm1 associated genes

Ccnd2	2623	0.211073938	18613	2.698011E-03
Ccnd3	*2624*	0.058938104	9155	4.071004E-01
Ccnd3-ps	2625		#N/A	1.000000E+00
Ccndbp1	2626	0.022320012	6652	7.537389E-01
Ccne1	*2627*	0.169603406	16909	1.635300E-02
Ccne2	2628	0.159154216	16233	2.438300E-02
Ccnf	2629	0.258368525	19604	2.208429E-04
Ccng1	*2630*	0.135196631	14641	5.629338E-02
Ccng2	2631	0.199762647	18238	4.568186E-03
Ccnh	2632	0.121613942	13765	8.625840E-02
Ccni	*2633*	0.07726586	10426	2.768240E-01
Ccnj	2634	0.064209434	9486	3.663671E-01
Ccnjl	2635	0.078862747	10522	2.669875E-01
Ccnk	*2636*	0.126037304	14049	7.534430E-02
Ccnl1	2637	0.101361697	12141	1.532439E-01
Ccnl2	2638	0.198149657	18173	4.913698E-03
Ccno	*2639*		#N/A	1.000000E+00
Ccnt1	2640	0.038803016	7572	5.853936E-01
Ccnt2	2641	0.2150684	18741	2.225730E-03
Ccny	*2642*	0.16697562	16770	1.811762E-02
Ccnyl1	2643	0.084062911	10925	2.366216E-01
Ccp110	2644	0.103637783	12278	1.441779E-01
Ccpg1	*2645*	0.021316405	6469	7.644793E-01
Ccr1	2646	0.047034628	8081	5.083770E-01
Ccr10	2647	0.058426979	9119	4.111876E-01
Ccr1l1	*2648*		#N/A	1.000000E+00
Ccr2	2649	-0.032450477	3820	6.482730E-01
Ccr3	2650	-0.038406171	3065	5.892384E-01
Ccr4	*2651*	0.098752258	11976	1.641596E-01
Ccr5	2652	0.178834242	17346	1.128639E-02
Ccr6	2653	0.096217463	11788	1.753099E-01
Ccr7	*2654*	-0.043988262	2372	5.362536E-01
Ccr8	2655		#N/A	1.000000E+00
Ccr9	2656	0.089292148	11293	2.086162E-01
Ccrl1	*2657*	-0.124267681	80	7.956822E-02
Ccrl2	2658	0.172321714	17041	1.468715E-02
Ccrn4l	2659	0.06536037	9551	3.578210E-01
Ccs	*2660*	0.052347185	8491	4.616299E-01
Ccsap	2661	0.069852103	9835	3.256728E-01
Ccser1	2662	0.161956074	16466	2.195210E-02
Ccser2	*2663*	0.077423929	10438	2.758395E-01
Cct2	2664	0.154051015	15893	2.940808E-02
Cct3	2665	0.190722412	17869	6.826473E-03

Spearman Rank correlation analysis performed between Prdm1 and all-expressed genes within the Meredith RNA-seq dataset. Robust Prdm1–associated genes were identified using a cut-off of $p < 0.0005$.

Table S1, Related to Supplemental Figure 3C. Prdm1 associated genes

Cct3-ps1	*2666*		#N/A	1.000000E+00
Cct4	2667	0.139817741	14942	4.830927E-02
Cct5	2668	0.134685513	14599	5.723986E-02
Cct6a	*2669*	0.100728742	12107	1.558399E-01
Cct6b	2670	-0.030557439	3909	6.675310E-01
Cct7	2671	0.218857981	18869	1.848491E-03
Cct8	*2672*	0.162940238	16541	2.114937E-02
Cct8l1	2673		#N/A	1.000000E+00
Ccz1	2674	0.119449632	13622	9.204228E-02
Cd101	*2675*	0.004149159	5524.5	9.535014E-01
Cd109	2676	-0.043910309	2375	5.369768E-01
Cd14	2677	-0.047588187	2245	5.033938E-01
Cd151	*2678*	0.14014286	14966	4.778503E-02
Cd160	2679	0.127545848	14155	7.188866E-02
Cd163	2680	-0.037912944	3635	5.940329E-01
Cd163l1	*2681*	-0.054451513	1703	4.437940E-01
Cd164	2682	0.109406953	12648	1.230292E-01
Cd164l2	2683	0.00030705	5316.5	9.965570E-01
Cd177	*2684*	0.090555233	11385.5	2.022247E-01
Cd180	2685	0.187481612	17743	7.851603E-03
Cd19	2686	0.139440393	14918	4.892373E-02
Cd1d1	*2687*	0.032526691	7204	6.475024E-01
Cd1d2	2688	-0.066856562	1139	3.468991E-01
Cd2	2689	0.238474293	19337	6.723999E-04
Cd200	*2690*	0.01010594	5776	8.870584E-01
Cd200r1	2691		#N/A	1.000000E+00
Cd200r2	2692	0.038154341	7488	5.916841E-01
Cd200r3	*2693*	0.020875719	6412.5	7.692098E-01
Cd200r4	2694	-0.038406171	3065	5.892384E-01
Cd207	2695	0.192355888	17926	6.356475E-03
Cd209a	*2696*	-0.038406171	3065	5.892384E-01
Cd209b	2697	-0.103163388	239	1.460330E-01
Cd209c	2698	-0.068468991	963	3.353671E-01
Cd209d	*2699*		#N/A	1.000000E+00
Cd209e	2700		#N/A	1.000000E+00
Cd209f	2701		#N/A	1.000000E+00
Cd209g	*2702*		#N/A	1.000000E+00
Cd22	2703	0.149438133	15616	3.468632E-02
Cd226	2704	-0.054451513	1703	4.437940E-01
Cd244	*2705*	-0.013636366	4583	8.480184E-01
Cd247	2706	0.047663982	8121	5.027134E-01
Cd248	2707		#N/A	1.000000E+00
Cd24a	*2708*	0.200801887	18277	4.357304E-03

Spearman Rank correlation analysis performed between Prdm1 and all-expressed genes within the Meredith RNA-seq dataset. Robust Prdm1–associated genes were identified using a cut-off of $p < 0.0005$.

Table S1, Related to Supplemental Figure 3C. Prdm1 associated genes

Cd27	2709	0.125961639	14043	7.552111E-02
Cd274	2710	0.092842753	11566	1.910121E-01
Cd276	*2711*	-0.052317278	2154	4.618862E-01
Cd28	2712	0.172428478	17047	1.462487E-02
Cd2ap	2713	0.078788338	10517	2.674405E-01
Cd2bp2	*2714*	0.123870798	13895	8.054130E-02
Cd300a	2715	0.125908356	14034	7.564582E-02
Cd300c	2716		#N/A	1.000000E+00
Cd300e	*2717*	-0.018790474	4278.5	7.917066E-01
Cd300lb	2718		#N/A	1.000000E+00
Cd300ld	2719	-0.047954856	2235	5.001071E-01
Cd300lf	*2720*	-0.05643476	1471.5	4.273478E-01
Cd300lg	2721	-0.013938399	4570	8.446941E-01
Cd300lh	2722	0.047153255	8091.5	5.073070E-01
Cd302	*2723*	0.001752412	5408	9.803521E-01
Cd320	2724	0.098838736	11983	1.637888E-01
Cd33	2725	0.042807012	7792.5	5.472647E-01
Cd34	*2726*	-0.066254108	1297.5	3.512713E-01
Cd36	2727	0.233612713	19247	8.707636E-04
Cd37	2728	0.164653465	16635	1.981245E-02
Cd38	*2729*	0.017335533	6192	8.075073E-01
Cd3d	2730	-0.00713876	4848	9.200857E-01
Cd3e	2731	0.075711532	10311	2.866309E-01
Cd3eap	*2732*	0.131117112	14359	6.421980E-02
Cd3g	2733	0.233261371	19232	8.870045E-04
Cd4	2734	0.088527983	11238	2.125529E-01
Cd40	*2735*	0.053964457	8863	4.478871E-01
Cd40lg	2736	-0.081570854	625	2.508571E-01
Cd44	2737	0.170358086	16950	1.587475E-02
Cd46	*2738*	0.048983255	8219	4.909495E-01
Cd47	2739	0.196978378	18123	5.179106E-03
Cd48	2740	0.197887874	18161	4.971934E-03
Cd5	*2741*	0.214091288	18717	2.333738E-03
Cd52	2742	0.153302514	15854	3.021481E-02
Cd53	2743	0.220053108	18901	1.742219E-03
Cd55	*2744*	0.042838447	7799	5.469702E-01
Cd59a	2745	0.256359272	19582	2.481443E-04
Cd59b	2746	0.014301797	6015.5	8.406982E-01
Cd5l	*2747*	0.197705265	18150	5.012924E-03
Cd6	2748	0.028348077	6985	6.902841E-01
Cd63	2749	0.004899068	5558	9.451095E-01
Cd63-ps	*2750*	0.099248004	12013	1.620423E-01
Cd68	2751	0.229317647	19130	1.089429E-03

Spearman Rank correlation analysis performed between Prdm1 and all-expressed genes within the Meredith RNA-seq dataset. Robust Prdm1–associated genes were identified using a cut-off of $p < 0.0005$.

Table S1, Related to Supplemental Figure 3C. Prdm1 associated genes

Cd69	2752	0.190352299	17855	6.937178E-03
Cd7	*2753*	-0.058038449	1435	4.143107E-01
Cd70	2754	0.017209246	6185	8.088825E-01
Cd72	2755	0.01430379	6027	8.406763E-01
Cd74	*2756*	0.150421734	15674	3.349806E-02
Cd79a	2757	0.233624389	19248	8.702286E-04
Cd79b	2758	0.165555434	16681	1.913849E-02
Cd80	*2759*	0.067672663	9683	3.410314E-01
Cd81	2760	0.087832421	11190	2.161822E-01
Cd82	2761	-0.009533373	4764	8.934182E-01
Cd83	*2762*	0.004805579	5551	9.461554E-01
Cd84	2763	0.066444363	9616	3.498868E-01
Cd86	2764	0.009083389	5735	8.984212E-01
Cd8a	*2765*	-0.056705422	1466.5	4.251310E-01
Cd8b1	2766	0.053805701	8820	4.492258E-01
Cd9	2767	0.173832887	17114	1.382685E-02
Cd9-ps	*2768*		#N/A	1.000000E+00
Cd93	2769	0.181318748	17458	1.018467E-02
Cd96	2770	0.225306028	19056	1.338097E-03
Cd97	*2771*	0.169263878	16891	1.657222E-02
Cd99l2	2772	0.080248299	10617	2.586484E-01
Cda	2773	0.055980215	8983	4.310857E-01
Cdadc1	*2774*	0.192662046	17937	6.271672E-03
Cdan1	2775	0.17022321	16941	1.595931E-02
Cdc123	2776	0.143177432	15146	4.311693E-02
Cdc14a	*2777*	0.102747771	12229	1.476731E-01
Cdc14b	2778	0.250602829	19513	3.447423E-04
Cdc16	2779	0.209317173	18559	2.933132E-03
Cdc20	*2780*	0.139452968	14919	4.890315E-02
Cdc20b	2781	0.163644566	16574	2.059059E-02
Cdc23	2782	0.099859029	12052	1.594609E-01
Cdc25a	*2783*	0.10909432	12632	1.241099E-01
Cdc25b	2784	0.122968233	13841	8.278992E-02
Cdc25c	2785	0.10414762	12302	1.422043E-01
Cdc26	*2786*	0.132526052	14448	6.138478E-02
Cdc27	2787	0.154196547	15905	2.925338E-02
Cdc34	2788	0.095945552	11768	1.765385E-01
Cdc34-ps	*2789*		#N/A	1.000000E+00
Cdc37	2790	0.155416866	16001	2.798333E-02
Cdc37l1	2791	0.210180895	18579	2.815309E-03
Cdc40	*2792*	0.203020332	18357	3.936171E-03
Cdc42	2793	0.231307319	19192	9.825290E-04
Cdc42bpa	2794	0.197744536	18153	5.004083E-03

Spearman Rank correlation analysis performed between Prdm1 and all-expressed genes within the Meredith RNA-seq dataset. Robust Prdm1–associated genes were identified using a cut-off of $p < 0.0005$.

Table S1, Related to Supplemental Figure 3C. Prdm1 associated genes

Cdc42bpb	*2795*	3.05596E-05	5292	9.996573E-01
Cdc42bpg	2796	0.055022511	8927	4.390225E-01
Cdc42ep1	2797	0.160987082	16412.5	2.276801E-02
Cdc42ep2	*2798*	0.037755174	7474	5.955703E-01
Cdc42ep3	2799	0.063505096	9435	3.716588E-01
Cdc42ep4	2800	0.145090425	15358	4.037548E-02
Cdc42ep5	*2801*	-0.103161098	248	1.460420E-01
Cdc42se1	2802	0.199273596	18214	4.670563E-03
Cdc42se2	2803	0.2417546	19391	5.630913E-04
Cdc45	*2804*	0.068214465	9716	3.371710E-01
Cdc5l	2805	0.219210514	18880	1.816545E-03
Cdc6	2806	0.011972659	5874	8.663758E-01
Cdc7	*2807*	0.343955948	19998	6.139500E-07
Cdc73	2808	0.1104879	12757	1.193487E-01
Cdca2	2809	0.053513468	8773	4.516960E-01
Cdca3	*2810*	0.192032967	17912	6.447029E-03
Cdca4	2811	0.065026895	9537	3.602842E-01
Cdca5	2812	0.229637051	19141	1.071579E-03
Cdca7	*2813*	0.160377606	16356	2.329449E-02
Cdca7l	2814	-0.01584433	4478	8.237829E-01
Cdca8	2815	0.104177398	12304	1.420896E-01
Cdcp1	*2816*	0.059153718	9164	4.053834E-01
Cdcp2	2817	-0.001224019	5151	9.862757E-01
Cdh1	2818	0.102203384	12192	1.498425E-01
Cdh10	*2819*	0.076720648	10393	2.802378E-01
Cdh11	2820	0.034074637	7296	6.319338E-01
Cdh12	2821	0.052171343	8483	4.631381E-01
Cdh13	*2822*	0.109412229	12650	1.230110E-01
Cdh15	2823	-0.054450825	1984	4.437997E-01
Cdh16	2824	-0.054451513	1703	4.437940E-01
Cdh17	*2825*	-0.038360444	3625	5.896821E-01
Cdh18	2826	0.274724451	19755	8.255589E-05
Cdh19	2827	0.013384031	5957	8.507976E-01
Cdh2	*2828*	0.147607941	15513	3.699253E-02
Cdh20	2829	0.046591528	8054	5.123843E-01
Cdh22	2830	0.018772884	6263	7.918972E-01
Cdh23	*2831*	-0.06392253	1341	3.685169E-01
Cdh24	2832		#N/A	1.000000E+00
Cdh26	2833	-0.103161098	248	1.460420E-01
Cdh3	*2834*	-0.026510602	4048	7.094223E-01
Cdh4	2835	0.008550035	5708	9.043564E-01
Cdh5	2836	0.170260502	16944	1.593589E-02
Cdh6	*2837*	0.067965012	9702	3.389449E-01

Spearman Rank correlation analysis performed between Prdm1 and all-expressed genes within the Meredith RNA-seq dataset. Robust Prdm1–associated genes were identified using a cut-off of $p < 0.0005$.

Table S1, Related to Supplemental Figure 3C. Prdm1 associated genes

Cdh7	2838	0.132880541	14470	6.068792E-02
Cdh8	2839	0.008247916	5689	9.077207E-01
Cdh9	*2840*	-0.096892634	302	1.722865E-01
Cdhr1	2841	0.045587558	7973	5.215241E-01
Cdhr2	2842	-0.072010768	928	3.109078E-01
Cdhr3	*2843*	0.013384031	5957	8.507976E-01
Cdhr4	2844		#N/A	1.000000E+00
Cdhr5	2845	-0.031519386	3856	6.577169E-01
Cdip1	*2846*	0.065387319	9552	3.576224E-01
Cdipt	2847	0.089904982	11337	2.054972E-01
Cdk1	2848	0.177303675	17281	1.201621E-02
Cdk10	*2849*		#N/A	1.000000E+00
Cdk11b	2850	0.170040128	16933	1.607473E-02
Cdk12	2851	0.172378921	17045	1.465375E-02
Cdk13	*2852*	0.108251478	12566	1.270601E-01
Cdk14	2853	0.00405576	5520	9.545469E-01
Cdk15	2854	-0.007634534	4834	9.145562E-01
Cdk16	*2855*	-0.001508361	5117	9.830879E-01
Cdk17	2856	0.04526187	7955	5.245069E-01
Cdk18	2857	0.148898943	15584	3.535273E-02
Cdk19	*2858*	0.0404913	7663	5.691677E-01
Cdk2	2859	0.087439111	11158	2.182539E-01
Cdk20	2860	0.035940058	7378.5	6.133856E-01
Cdk2ap1	*2861*	0.133960398	14548	5.860511E-02
Cdk2ap2	2862	0.219398797	18885	1.799689E-03
CDK2AP2	2863		#N/A	1.000000E+00
Cdk3-ps	*2864*	0.053805701	8820	4.492258E-01
Cdk4	2865	0.19563157	18069	5.500123E-03
Cdk5	2866	0.248856367	19494	3.803332E-04
Cdk5r1	*2867*	0.061363089	9291	3.880388E-01
Cdk5r2	2868		#N/A	1.000000E+00
Cdk5rap1	2869	0.080590897	10638	2.566143E-01
Cdk5rap2	*2870*	0.171074854	16977	1.543188E-02
Cdk5rap3	2871	0.13121363	14362	6.402223E-02
Cdk6	2872	0.172622821	17057	1.451209E-02
Cdk7	*2873*	0.038544114	7529	5.879006E-01
Cdk8	2874	0.170463567	16953	1.580889E-02
Cdk9	2875	0.184179393	17598	9.034747E-03
Cdkal1	*2876*	0.114502848	13293	1.064235E-01
Cdkl1	2877	-0.054451513	1703	4.437940E-01
Cdkl2	2878	-0.137735147	43	5.178225E-02
Cdkl3	*2879*	-0.029997911	3926	6.732657E-01
Cdkl4	2880	0.07385929	10115	2.986181E-01

Spearman Rank correlation analysis performed between Prdm1 and all-expressed genes within the Meredith RNA-seq dataset. Robust Prdm1–associated genes were identified using a cut-off of $p < 0.0005$.

Table S1, Related to Supplemental Figure 3C. Prdm1 associated genes

Cdkl5	2881	0.039154913	7586	5.819940E-01
Cdkn1a	2882	0.094238314	11658	1.843985E-01
Cdkn1b	2883	0.245727295	19451	4.527521E-04
Cdkn1c	2884	-0.103520753	218	1.446339E-01
Cdkn2a	2885	0.03969068	7611	5.768358E-01
Cdkn2aip	2886	0.142343787	15102	4.435954E-02
Cdkn2aipnl	2887	0.134284441	14580	5.799172E-02
Cdkn2b	2888	0.049075783	8221	4.901300E-01
Cdkn2c	2889	0.080468463	10631	2.573399E-01
Cdkn2d	2890	0.160523921	16368	2.316715E-02
Cdkn3	2891	0.048848669	8207	4.921428E-01
Cdnf	2892	0.088199096	11222	2.142635E-01
Cdo1	2893	0.205072605	18429	3.579630E-03
Cdon	2894	-0.026931952	4032	7.050171E-01
Cdpf1	2895	0.062810953	9381	3.769197E-01
Cdr1	2896		#N/A	1.000000E+00
Cdr2	2897	-0.057428267	1446	4.192434E-01
Cdr2l	2898	0.138031034	14826	5.127654E-02
Cdrt4	2899		#N/A	1.000000E+00
Cds1	2900	-0.142749903	34.5	4.375050E-02
Cds2	2901	0.045018484	7934	5.267415E-01
Cdsn	2902	0.06838666	9734	3.359500E-01
Cdt1	2903		#N/A	1.000000E+00
Cdv3	2904	0.172950829	17076	1.432347E-02
Cdx1	2905	-0.04309166	2413	5.446012E-01
Cdx2	2906		#N/A	1.000000E+00
Cdx4	2907		#N/A	1.000000E+00
Cdyl	2908	0.281312239	19808	5.455303E-05
Cdyl2	2909	0.25325108	19543	2.966296E-04
Ceacam1	2910	0.03037917	7087.5	6.693560E-01
Ceacam10	2911	0.066456156	9618	3.498011E-01
Ceacam11	2912		#N/A	1.000000E+00
Ceacam12	2913		#N/A	1.000000E+00
Ceacam13	2914	0.260179967	19627	1.986542E-04
Ceacam14	2915		#N/A	1.000000E+00
Ceacam15	2916	-0.028666921	3973	6.869827E-01
Ceacam16	2917	-0.020772979	4215	7.703138E-01
Ceacam18	2918	0.028229807	6981	6.915102E-01
Ceacam19	2919	0.124877452	13961	7.809169E-02
Ceacam2	2920	0.090858474	11420.5	2.007115E-01
Ceacam20	2921	-0.065964527	1307	3.533852E-01
Ceacam3	2922	0.113548679	13094	1.093908E-01
Ceacam5	2923	-0.038406171	3065	5.892384E-01

Spearman Rank correlation analysis performed between Prdm1 and all-expressed genes within the Meredith RNA-seq dataset. Robust Prdm1–associated genes were identified using a cut-off of $p < 0.0005$.

Table S1, Related to Supplemental Figure 3C. Prdm1 associated genes

Ceacam9	*2924*	-0.038406171	3065	5.892384E-01
Cebpa	2925	0.030179781	7073	6.713996E-01
Cebpb	2926	0.130165555	14306	6.619451E-02
Cebpd	*2927*	0.04911982	8224	4.897402E-01
Cebpe	2928		#N/A	1.000000E+00
Cebpg	2929	0.229556838	19138	1.076036E-03
Cebpz	*2930*	0.103747805	12281	1.437502E-01
Cecr2	2931	0.061351524	9290	3.881284E-01
Cecr5	2932	0.026092871	6877	7.137992E-01
Cecr6	*2933*		#N/A	1.000000E+00
Cel	2934	-0.038545798	2499	5.878843E-01
Cela1	2935	0.323986658	19976	2.868806E-06
Cela2a	*2936*		#N/A	1.000000E+00
Cela3b	2937	-0.081772856	623	2.496816E-01
Celf1	2938	0.177702553	17298	1.182212E-02
Celf2	*2939*	0.186762841	17709	8.096709E-03
Celf3	2940	0.109930598	12709.5	1.212354E-01
Celf4	2941	0.111841885	12840	1.148600E-01
Celf5	*2942*	0.099068343	12002	1.628073E-01
Celf6	2943	0.044371442	7889	5.327057E-01
Celsr1	2944	0.05106711	8391	4.726710E-01
Celsr2	*2945*	-0.001261706	5129.5	9.858531E-01
Celsr3	2946		#N/A	1.000000E+00
Cend1	2947	-0.054450825	1984	4.437997E-01
Cenpa	*2948*	0.372370849	20019	5.646577E-08
Cenpb	2949		#N/A	1.000000E+00
Cenpc1	2950	0.115272949	13341	1.040752E-01
Cenpe	*2951*	0.129123453	14247	6.841380E-02
Cenpf	2952	0.130041007	14302	6.645662E-02
Cenph	2953	-0.012486303	4648	8.607001E-01
Cenpi	*2954*	-0.015879747	4474	8.233954E-01
Cenpj	2955	-0.004245441	4976	9.524236E-01
Cenpk	2956	0.004066294	5521	9.544290E-01
Cenpl	*2957*	0.177604847	17291	1.186941E-02
Cenpm	2958	0.069323931	9798	3.293533E-01
Cenpn	2959	0.186664113	17704	8.130901E-03
Cenpo	*2960*	-0.08311518	602	2.419671E-01
Cenpp	2961	0.051196703	8397	4.715467E-01
Cenpq	2962	0.023972878	6750	7.361538E-01
Cenpt	*2963*	-0.117567396	127.5	9.732002E-02
Cenpv	2964	0.084978735	10986	2.315353E-01
Cenpw	2965	0.159557242	16252	2.401967E-02
Cep104	*2966*	-0.047728281	2242	5.021367E-01

Spearman Rank correlation analysis performed between Prdm1 and all-expressed genes within the Meredith RNA-seq dataset. Robust Prdm1–associated genes were identified using a cut-off of $p < 0.0005$.

Table S1, Related to Supplemental Figure 3C. Prdm1 associated genes

Cep110	2967	0.120779424	13710	8.845306E-02
Cep112	2968	0.280614063	19805	5.703001E-05
Cep120	*2969*	0.189111957	17807	7.319938E-03
Cep128	2970	0.065664635	9569	3.555827E-01
Cep135	2971	0.122396173	13811	8.424109E-02
Cep152	*2972*	0.107376646	12517	1.301792E-01
Cep164	2973	0.064265321	9491	3.659492E-01
Cep170	2974	0.122908367	13838	8.294084E-02
Cep170b	*2975*	0.124704555	13950	7.850809E-02
Cep19	2976	-0.037080339	3659	6.021665E-01
Cep192	2977	0.163318579	16561	2.084761E-02
Cep250	*2978*	0.087601843	11174	2.173950E-01
Cep290	2979	-0.073984647	903	2.977965E-01
Cep350	2980	0.169593543	16907	1.635933E-02
Cep41	*2981*	-0.073291779	917	3.023564E-01
Cep44	2982	0.000329802	5332	9.963019E-01
Cep55	2983	0.130723385	14336	6.503095E-02
Cep57	*2984*	0.207618483	18516	3.177972E-03
Cep57l1	2985	0.172376884	17044	1.465494E-02
Cep63	2986	0.011526561	5855	8.713107E-01
Cep68	*2987*	0.030723142	7110	6.658363E-01
Cep70	2988	0.079143743	10539	2.652816E-01
Cep72	2989	0.014233777	6001	8.414459E-01
Cep76	*2990*	0.050167519	8309	4.805158E-01
Cep78	2991	0.143188783	15147	4.310021E-02
Cep85	2992	0.054952913	8923	4.396025E-01
Cep85l	*2993*	0.076363259	10372	2.824909E-01
Cep89	2994	0.067137321	9652	3.448733E-01
Cep95	2995	0.054289869	8882	4.451500E-01
Cep97	*2996*	0.1563139	16073	2.708009E-02
Cept1	2997	0.222851915	18979	1.514811E-03
Cer1	2998	0.264806563	19676	1.510561E-04
Cercam	*2999*	0.098906751	11988	1.634976E-01
Cerk	3000	0.19332664	17960	6.091056E-03
Cerkl	3001	0.079849353	10579	2.610309E-01
Cers1	*3002*		#N/A	1.000000E+00
Cers2	3003	0.210855073	18605	2.726341E-03
Cers3	3004	0.213925639	18710	2.352513E-03
Cers4	*3005*	0.059622254	9192	4.016673E-01
Cers5	3006	0.044764087	7917	5.290824E-01
Cers6	3007	0.140380956	14981	4.740413E-02
Ces1a	*3008*	-0.06485515	1325.5	3.615570E-01
Ces1b	3009	0.052728522	8550.5	4.583686E-01

Spearman Rank correlation analysis performed between Prdm1 and all-expressed genes within the Meredith RNA-seq dataset. Robust Prdm1–associated genes were identified using a cut-off of $p < 0.0005$.

Table S1, Related to Supplemental Figure 3C. Prdm1 associated genes

Ces1c	3010	0.113548679	13094	1.093908E-01
Ces1d	*3011*	0.02758255	6956	6.982344E-01
Ces1e	3012	-0.001959544	5078	9.780303E-01
Ces1f	3013	0.033690558	7279	6.357820E-01
Ces1g	*3014*	0.163007706	16546	2.109529E-02
Ces1h	3015	-0.076325867	852	2.827273E-01
Ces2a	3016	0.002251949	5429	9.747529E-01
Ces2b	*3017*	0.162808768	16526.5	2.125512E-02
Ces2c	3018		#N/A	1.000000E+00
Ces2d-ps	3019		#N/A	1.000000E+00
Ces2e	*3020*	-0.003048459	5022	9.658278E-01
Ces2f	3021		#N/A	1.000000E+00
Ces2g	3022	0.014050303	5992	8.434632E-01
Ces2h	*3023*	0.162808768	16526.5	2.125512E-02
Ces3a	3024	-0.054450825	1984	4.437997E-01
Ces3b	3025		#N/A	1.000000E+00
Ces4a	*3026*	-0.016731055	4437	8.140953E-01
Ces5a	3027	0.015107379	6075	8.318546E-01
Cetn1	3028		#N/A	1.000000E+00
Cetn2	*3029*	0.0153454	6087	8.292456E-01
Cetn3	3030	0.089118165	11280	2.095078E-01
Cetn4	3031	0.041590052	7716	5.587235E-01
Cfb	*3032*	0.085144665	10996	2.306220E-01
Cfc1	3033	-0.086746694	532	2.219355E-01
Cfd	3034		#N/A	1.000000E+00
Cfdp1	*3035*	0.186400752	17697	8.222739E-03
Cfh	3036	0.074194755	10154.5	2.964228E-01
Cfhr1	3037	0.072416169	10023	3.081846E-01
Cfhr2	*3038*	0.166369694	16740.5	1.854724E-02
Cfhr3	3039		#N/A	1.000000E+00
Cfi	3040	-0.046941708	2262	5.092160E-01
Cfl1	*3041*	0.13321722	14494	6.003210E-02
Cfl2	3042	0.197694476	18148	5.015355E-03
Cflar	3043	0.05403259	8867	4.473132E-01
Cfp	*3044*	0.082065264	10752	2.479867E-01
Cftr	3045	0.054825898	8910	4.406622E-01
Cga	3046	0.058054502	9093	4.141814E-01
Cggbp1	*3047*	0.075352005	10292	2.889321E-01
Cgn	3048	0.022686709	6667	7.498262E-01
Cgnl1	3049	0.04749705	8114	5.042124E-01
Cgref1	*3050*	-0.054450825	1984	4.437997E-01
Cgrrf1	3051	0.062666004	9370	3.780240E-01
Ch25h	3052	-0.087580317	438	2.175085E-01

Spearman Rank correlation analysis performed between Prdm1 and all-expressed genes within the Meredith RNA-seq dataset. Robust Prdm1–associated genes were identified using a cut-off of $p < 0.0005$.

Table S1, Related to Supplemental Figure 3C. Prdm1 associated genes

Chac1	*3053*	0.061321653	9287	3.883599E-01
Chac2	3054	-0.004833901	4946	9.458385E-01
Chad	3055		#N/A	1.000000E+00
Chadl	*3056*	-0.066858259	1029.5	3.468868E-01
Chaf1a	3057	0.184517627	17619	8.906694E-03
Chaf1b	3058	0.196899914	18119	5.197337E-03
Champ1	*3059*	0.01754834	6203	8.051911E-01
Chat	3060	-0.038667572	2497	5.867045E-01
Chchd1	3061	0.143523888	15161	4.260917E-02
Chchd10	*3062*	0.186756548	17708	8.098884E-03
Chchd2	3063	0.110473299	12756	1.193978E-01
Chchd3	3064	0.230018282	19151	1.050625E-03
Chchd4	*3065*	0.03980566	7619	5.757316E-01
Chchd5	3066	0.013303735	5951	8.516824E-01
Chchd6	3067	0.098191469	11930	1.665796E-01
Chchd7	*3068*	0.053659305	8782	4.504623E-01
Chd1	3069	0.11602065	13396	1.018344E-01
Chd1l	3070	-0.103079555	259	1.463627E-01
Chd2	*3071*	0.142778093	15133	4.370849E-02
Chd3	3072	0.04926212	8242	4.884818E-01
Chd4	3073	0.16303816	16549	2.107091E-02
Chd5	*3074*	-0.018105422	4347	7.991361E-01
Chd6	3075	0.042307783	7761	5.519514E-01
Chd7	3076	0.241433133	19383	5.730279E-04
Chd8	*3077*	0.225800452	19068	1.304865E-03
Chd9	3078	0.061508714	9303	3.869116E-01
Chdc2	3079	-0.054451513	1703	4.437940E-01
Chdh	*3080*	0.055430874	8954	4.356281E-01
Chek1	3081	0.149061785	15594	3.515033E-02
Chek2	3082	0.038402332	7519	5.892756E-01
Cherp	*3083*	0.040482463	7662	5.692521E-01
Chfr	3084	0.129769214	14287	6.703153E-02
Chga	3085	-0.054004368	2112	4.475509E-01
Chgb	*3086*	-0.066855996	1239.5	3.469032E-01
Chi3l1	3087	-0.001598289	5114	9.820798E-01
Chi3l3	3088	-0.103159953	253.5	1.460465E-01
Chi3l4	*3089*	-0.044417492	2353	5.322801E-01
Chi3l7	3090	-0.038406171	3065	5.892384E-01
Chia	3091	0.073928833	10125	2.981622E-01
Chic1	*3092*	0.155318432	15993	2.808400E-02
Chic2	3093	0.182171651	17490	9.828990E-03
Chid1	3094	0.115640917	13367	1.029676E-01
Chit1	*3095*	0.260492779	19630	1.950397E-04

Spearman Rank correlation analysis performed between Prdm1 and all-expressed genes within the Meredith RNA-seq dataset. Robust Prdm1–associated genes were identified using a cut-off of $p < 0.0005$.

Table S1, Related to Supplemental Figure 3C. Prdm1 associated genes

Chka	3096	0.076793021	10401	2.797830E-01
Chkb	3097	0.116338609	13419	1.008931E-01
Chl1	*3098*	0.129531505	14273	6.753768E-02
Chm	3099	0.14507872	15356	4.039179E-02
Chml	3100		#N/A	1.000000E+00
Chmp1a	*3101*	0.141476015	15049	4.568475E-02
Chmp2a	3102	0.142086039	15085	4.474976E-02
Chmp2b	3103	0.108216739	12564	1.271828E-01
Chmp3	*3104*	0.136509056	14725	5.392215E-02
Chmp4b	3105	0.142149199	15092	4.465387E-02
Chmp4c	3106	0.187963516	17761	7.690986E-03
Chmp5	*3107*	0.079864871	10583	2.609379E-01
Chmp6	3108	-0.047885934	2238	5.007240E-01
Chmp7	3109	-0.040460981	2465	5.694572E-01
Chn1	*3110*	0.079428471	10555	2.635607E-01
Chn2	3111	-0.0255332	4085	7.196780E-01
Chodl	3112	-0.00529697	4914	9.406591E-01
Chordc1	*3113*	0.075719183	10323	2.865821E-01
Chp1	3114	0.145044275	15355	4.043984E-02
Chp2	3115		#N/A	1.000000E+00
Chpf	*3116*	0.22544513	19059	1.328670E-03
Chpf2	3117	0.122658568	13825	8.357295E-02
Chpt1	3118	-0.021757768	4181	7.597502E-01
Chrac1	*3119*	0.141193787	15030	4.612281E-02
Chrd	3120	-0.066856562	1139	3.468991E-01
Chrdl1	3121	0.065618682	9568	3.559202E-01
Chrdl2	*3122*	-0.077393517	797	2.760287E-01
Chrm1	3123	0.125901835	14033	7.566110E-02
Chrm2	3124	-0.008225977	4812	9.079651E-01
Chrm3	*3125*		#N/A	1.000000E+00
Chrm4	3126	0.280545747	19804	5.727797E-05
Chrm5	3127		#N/A	1.000000E+00
Chrna1	*3128*	-0.087423725	439	2.183352E-01
Chrna10	3129	0.012872949	5923	8.564324E-01
Chrna2	3130	-0.055858199	1494	4.320923E-01
Chrna3	*3131*	-0.04297985	2416	5.456467E-01
Chrna4	3132	0.09390163	11638	1.859784E-01
Chrna5	3133	0.10536343	12382	1.375808E-01
Chrna6	*3134*		#N/A	1.000000E+00
Chrna7	3135	0.138482117	14855	5.051347E-02
Chrna9	3136	-0.018138782	4334.5	7.987739E-01
Chrnb1	*3137*	0.183572848	17575	9.268472E-03
Chrnb2	3138	-0.008927098	4789	9.001599E-01

Spearman Rank correlation analysis performed between Prdm1 and all-expressed genes within the Meredith RNA-seq dataset. Robust Prdm1–associated genes were identified using a cut-off of $p < 0.0005$.

Table S1, Related to Supplemental Figure 3C. Prdm1 associated genes

Chrnb3	3139	0.230235201	19161.5	1.038871E-03
Chrnb4	*3140*	0.05681053	9028	4.242719E-01
Chrnd	3141	0.086412184	11087.5	2.237298E-01
Chrne	3142	0.216901297	18818	2.035305E-03
Chrng	*3143*	0.13535932	14653	5.599484E-02
Chst1	3144	0.067501148	9675	3.422593E-01
Chst10	3145	0.091416084	11479	1.979505E-01
Chst11	*3146*	0.061301602	9283	3.885153E-01
Chst12	3147	0.078427874	10493	2.696423E-01
Chst13	3148		#N/A	1.000000E+00
Chst14	*3149*	0.051183806	8395	4.716585E-01
Chst15	3150		#N/A	1.000000E+00
Chst2	3151	0.155021072	15981	2.838999E-02
Chst3	*3152*	-0.010887018	4707	8.783942E-01
Chst4	3153	0.074574898	10201.5	2.939481E-01
Chst5	3154	-0.038406171	3065	5.892384E-01
Chst7	*3155*	-0.075958455	865	2.850576E-01
Chst8	3156	0.154547993	15958	2.888267E-02
Chst9	3157	0.184131833	17596	9.052883E-03
Chsy1	*3158*	0.085068188	10992	2.310426E-01
Chsy3	3159	0.112907751	12915	1.114201E-01
Chtf18	3160	-0.046594725	2272	5.123554E-01
Chtf8	*3161*	0.063206915	9402	3.739131E-01
Chtop	3162	0.139301128	14910	4.915215E-02
Chuk	3163	0.184231969	17599	9.014736E-03
Churc1	*3164*	0.229347185	19131	1.087767E-03
Ciao1	3165	0.169060156	16875	1.670499E-02
Ciapin1	3166	0.134610824	14595	5.737926E-02
Cib1	*3167*	0.052512005	8502	4.602187E-01
Cib2	3168	0.148815681	15581	3.545660E-02
Cib3	3169	0.027015673	6933	7.041430E-01
Cib4	*3170*	0.167150607	16780	1.799517E-02
Cic	3171	0.178748246	17341	1.132633E-02
Cidea	3172	-0.003935858	4985	9.558893E-01
Cideb	*3173*	0.090624692	11395	2.018774E-01
Cidec	3174	-0.003703707	4996.5	9.584887E-01
Ciita	3175	0.052913878	8589	4.567880E-01
Cilp	*3176*	-0.132819589	55	6.080727E-02
Cilp2	3177	-0.066855996	1239.5	3.469032E-01
Cinp	3178	0.047448705	8112	5.046470E-01
Cir1	*3179*	0.070560874	9877	3.207758E-01
Cirbp	3180	0.016481689	6142	8.168168E-01
Cirh1a	3181	0.151993211	15768	3.167135E-02

Spearman Rank correlation analysis performed between Prdm1 and all-expressed genes within the Meredith RNA-seq dataset. Robust Prdm1–associated genes were identified using a cut-off of $p < 0.0005$.

Table S1, Related to Supplemental Figure 3C. Prdm1 associated genes

Cisd1	*3182*	0.147773484	15522	3.677873E-02
Cisd2	3183	-0.04741589	2251	5.049421E-01
Cisd3	3184	0.086491304	11106	2.233044E-01
Cish	*3185*	0.101186211	12131	1.539603E-01
Cit	3186	0.003654471	5501	9.590400E-01
Cited1	3187	-0.130647122	60	6.518903E-02
Cited2	*3188*	0.028374479	6987	6.900105E-01
Cited4	3189	0.023709146	6733	7.389508E-01
Ciz1	3190	0.001592976	5396	9.821393E-01
CK137956	*3191*	0.056382636	9004	4.277755E-01
Ckap2	3192	0.17256955	17051	1.454293E-02
Ckap2l	3193	-0.017214276	4405	8.088277E-01
Ckap4	*3194*	0.263051091	19658	1.676992E-04
Ckap5	3195	0.163606171	16573	2.062072E-02
Ckb	3196	0.120382315	13686	8.951297E-02
Cklf	*3197*	0.012252309	5889	8.632849E-01
Ckm	3198	-0.054451513	1703	4.437940E-01
Ckmt1	3199	0.026851289	6923	7.058597E-01
Ckmt2	*3200*	-0.054451513	1703	4.437940E-01
Cks1b	3201	0.174341914	17138	1.354717E-02
Cks2	3202	0.202451893	18340	4.040431E-03
Clasp1	*3203*	0.071166909	9922	3.166267E-01
Clasp2	3204	0.261099685	19638	1.882014E-04
Clasrp	3205	-0.095104171	373	1.803806E-01
Clca1	*3206*	-0.041649122	2441	5.581647E-01
Clca2	3207	0.132376382	14442	6.168097E-02
Clca3	3208	0.098659694	11967	1.645573E-01
Clca4	*3209*	0.075638426	10301	2.870978E-01
Clca5	3210	-0.06373106	1351	3.699560E-01
Clca6	3211	-0.038406171	3065	5.892384E-01
Clcc1	*3212*	-0.007690328	4832	9.139342E-01
CLCF1	3213		#N/A	1.000000E+00
Clcf1	3214	0.127586428	14159	7.179752E-02
Clcn1	*3215*	-0.038406171	3065	5.892384E-01
Clcn2	3216	-0.073235387	918	3.027296E-01
Clcn3	3217	0.121707974	13773	8.601387E-02
Clcn4-2	*3218*	0.224412058	19029	1.400159E-03
Clcn5	3219	0.090067197	11350	2.046773E-01
Clcn6	3220	0.032414876	7200	6.486331E-01
Clcn7	*3221*	-0.047943284	2237	5.002106E-01
Clcnka	3222	0.000307056	5327	9.965570E-01
Clcnkb	3223	-0.054450825	1984	4.437997E-01
Cldn1	*3224*	0.05727052	9050	4.205242E-01

Spearman Rank correlation analysis performed between Prdm1 and all-expressed genes within the Meredith RNA-seq dataset. Robust Prdm1–associated genes were identified using a cut-off of $p < 0.0005$.

Table S1, Related to Supplemental Figure 3C. Prdm1 associated genes

Cldn10	3225	0.048745218	8199	4.930612E-01
Cldn11	3226	-0.095273984	329	1.796002E-01
Cldn12	*3227*	0.097594719	11876	1.691839E-01
Cldn13	3228	-0.015594995	4492	8.265118E-01
Cldn14	3229	0.040730281	7679	5.668882E-01
Cldn15	*3230*		#N/A	1.000000E+00
Cldn16	3231	0.053267784	8685	4.537786E-01
Cldn17	3232		#N/A	1.000000E+00
Cldn18	*3233*	0.189660518	17830	7.148396E-03
Cldn19	3234	0.090338046	11372	2.033135E-01
Cldn2	3235	-0.054450825	1984	4.437997E-01
Cldn20	*3236*		#N/A	1.000000E+00
Cldn22	3237		#N/A	1.000000E+00
Cldn23	3238	0.096640119	11817	1.734127E-01
Cldn24	*3239*		#N/A	1.000000E+00
Cldn25	3240	-0.033385506	3776	6.388454E-01
Cldn26	3241	-0.038406171	3065	5.892384E-01
Cldn3	*3242*	0.061201148	9271	3.892947E-01
Cldn4	3243	0.03100988	7133	6.629079E-01
Cldn5	3244		#N/A	1.000000E+00
Cldn6	*3245*		#N/A	1.000000E+00
Cldn7	3246	0.087814601	11184	2.162758E-01
Cldn8	3247		#N/A	1.000000E+00
Cldn9	*3248*	-0.054451513	1703	4.437940E-01
Cldnd2	3249		#N/A	1.000000E+00
Clec10a	3250	-0.000458472	5251.5	9.948592E-01
Clec11a	*3251*	0.021317126	6513.5	7.644715E-01
Clec12a	3252	0.102420595	12207	1.489740E-01
Clec12b	3253	0.051668288	8445	4.674677E-01
Clec14a	*3254*		#N/A	1.000000E+00
Clec16a	3255	0.195270704	18055	5.589111E-03
Clec18a	3256		#N/A	1.000000E+00
Clec1a	*3257*	-0.038406171	3065	5.892384E-01
Clec1b	3258	-0.038406171	3065	5.892384E-01
Clec2d	3259	0.097769915	11889	1.684162E-01
Clec2e	*3260*	0.090770309	11407	2.011506E-01
Clec2f	3261		#N/A	1.000000E+00
Clec2g	3262	0.112947378	12923	1.112938E-01
Clec2h	*3263*	-0.097049929	300	1.715878E-01
Clec2i	3264	-0.076676991	849	2.805124E-01
Clec2j	3265		#N/A	1.000000E+00
Clec2l	*3266*	0.032887553	7229	6.438590E-01
Clec3a	3267	-0.054451513	1703	4.437940E-01

Spearman Rank correlation analysis performed between Prdm1 and all-expressed genes within the Meredith RNA-seq dataset. Robust Prdm1–associated genes were identified using a cut-off of $p < 0.0005$.

Table S1, Related to Supplemental Figure 3C. Prdm1 associated genes

Clec3b	3268	-0.038406171	3065	5.892384E-01
Clec4a1	*3269*	0.052682083	8520	4.587650E-01
Clec4a2	3270	0.100656832	12100	1.561370E-01
Clec4a3	3271	0.082762295	10811.5	2.439788E-01
Clec4a4	*3272*	0.074574898	10201.5	2.939481E-01
Clec4b1	3273	0.111877797	12843	1.147428E-01
Clec4b2	3274	-0.054450825	1984	4.437997E-01
Clec4d	*3275*		#N/A	1.000000E+00
Clec4e	3276	0.021100249	6443	7.667985E-01
Clec4f	3277		#N/A	1.000000E+00
Clec4g	*3278*		#N/A	1.000000E+00
Clec4n	3279	0.021316405	6469	7.644793E-01
Clec5a	3280	0.121153549	13735	8.746371E-02
Clec7a	*3281*	0.03908867	7584	5.826333E-01
Clec9a	3282	0.164977106	16656	1.956830E-02
Clgn	3283	0.107709621	12535	1.289851E-01
Clhc1	*3284*	-0.032445878	3830.5	6.483195E-01
Clic1	3285	-0.009660939	4760	8.920007E-01
Clic3	3286	0.033918136	7290	6.335006E-01
Clic4	*3287*	0.18443356	17616	8.938370E-03
Clic5	3288	0.074891238	10228	2.918993E-01
Clic6	3289	-0.004937246	4940	9.446824E-01
Clint1	*3290*	0.061026651	9261	3.906506E-01
Clip1	3291	0.047665755	8122	5.026975E-01
Clip2	3292	0.116281208	13414	1.010625E-01
Clip3	*3293*	-0.12719401	74	7.268288E-02
Clip4	3294	0.277880509	19778	6.778208E-05
Clk1	3295	0.215118128	18747	2.220357E-03
Clk2	*3296*	0.021959133	6587	7.575956E-01
Clk3	3297	0.211720566	18643	2.615865E-03
Clk4	3298	0.204594585	18409	3.659967E-03
Clmn	*3299*	0.151456984	15742	3.228490E-02
Clmp	3300	0.127735219	14171	7.146412E-02
Cln3	3301	0.067898624	9697	3.394180E-01
Cln5	*3302*	-0.027739829	4008	6.965983E-01
Cln6	3303	0.025805192	6862	7.168189E-01
Cln8	3304	-0.020857868	4209	7.694016E-01
Clnk	*3305*	0.049687465	8268	4.847307E-01
Clns1a	3306	0.116833086	13455	9.944299E-02
Clock	3307	0.155711591	16019	2.768377E-02
Clp1	*3308*	0.024333747	6776	7.323322E-01
Clpb	3309	0.142420105	15106	4.424455E-02
Clpp	3310	0.127855734	14179	7.119501E-02

Spearman Rank correlation analysis performed between Prdm1 and all-expressed genes within the Meredith RNA-seq dataset. Robust Prdm1–associated genes were identified using a cut-off of $p < 0.0005$.

Table S1, Related to Supplemental Figure 3C. Prdm1 associated genes

Clps	*3311*	0.048846555	8205	4.921616E-01
Clpsl2	3312		#N/A	1.000000E+00
Clptm1	3313	0.078580498	10501	2.687085E-01
Clptm1l	*3314*	0.175150574	17173	1.311307E-02
Clpx	3315	-0.033726176	3749	6.354247E-01
Clrn1	3316	-0.066142368	1305	3.520860E-01
Clrn2	*3317*		#N/A	1.000000E+00
Clrn3	3318	-0.090704007	410	2.014813E-01
Clspn	3319	0.050656289	8354	4.762448E-01
Clstn1	*3320*	0.142621377	15125	4.394249E-02
Clstn2	3321	0.212225353	18660	2.553322E-03
Clstn3	3322	-0.054451513	1703	4.437940E-01
Clta	*3323*	0.181773583	17476	9.993601E-03
Cltb	3324	-0.017165773	4406	8.093561E-01
Cltc	3325	0.110430156	12750	1.195431E-01
Clu	*3326*	0.169505618	16902	1.641587E-02
Cluap1	3327	0.170582274	16959	1.573505E-02
Cluh	3328	-0.049717527	2202	4.844662E-01
Clvs1	*3329*	0.229369377	19133	1.086520E-03
Clvs2	3330	0.03037917	7087.5	6.693560E-01
Clybl	3331	0.043422703	7831	5.415117E-01
Cma1	*3332*		#N/A	1.000000E+00
Cma2	3333	0.022198129	6607.5	7.550408E-01
Cmah	3334	0.165900382	16698	1.888605E-02
Cmas	*3335*	0.275680998	19761	7.778642E-05
Cmbl	3336	0.119740638	13643	9.124707E-02
Cmc1	3337	0.181771329	17475	9.994540E-03
Cmc2	*3338*	0.203934932	18386	3.773502E-03
Cmip	3339	0.114051464	13254	1.078192E-01
Cmklr1	3340	0.02273662	6676	7.492941E-01
Cml1	*3341*	0.012289581	5893	8.628731E-01
Cml2	3342	-0.054451513	1703	4.437940E-01
Cml3	3343	0.08254322	10792	2.452336E-01
Cml5	*3344*		#N/A	1.000000E+00
Cmpk1	3345	0.172962545	17079	1.431677E-02
Cmpk2	3346	0.170885678	16970	1.554770E-02
Cmss1	*3347*	0.085665042	11029	2.277745E-01
Cmtm1	3348	0.142100814	15090	4.472731E-02
Cmtm2a	3349	-0.054450825	1984	4.437997E-01
Cmtm2b	*3350*	0.053267784	8685	4.537786E-01
Cmtm3	3351	0.200334124	18255	4.451114E-03
Cmtm4	3352	0.197392538	18136	5.083825E-03
Cmtm5	*3353*	-0.038406171	3065	5.892384E-01

Spearman Rank correlation analysis performed between Prdm1 and all-expressed genes within the Meredith RNA-seq dataset. Robust Prdm1–associated genes were identified using a cut-off of $p < 0.0005$.

Table S1, Related to Supplemental Figure 3C. Prdm1 associated genes

Cmtm6	3354	0.219109141	18877	1.825679E-03
Cmtm7	3355	0.234892082	19268	8.139089E-04
Cmtm8	*3356*	0.095087779	11702	1.804561E-01
Cmya5	3357	0.230518953	19177	1.023677E-03
CN725425	3358		#N/A	1.000000E+00
Cnbd1	*3359*	0.184565106	17620	8.888848E-03
Cnbd2	3360	-0.022991113	4148	7.465831E-01
Cnbp	3361	0.214913966	18736	2.242493E-03
Cndp1	*3362*	0.160304018	16323	2.335876E-02
Cndp2	3363	0.123263509	13861	8.204879E-02
Cnep1r1	3364	-0.01373878	4576	8.468909E-01
Cnfn	*3365*	0.204368167	18403	3.698584E-03
Cnga1	3366	0.147801977	15525	3.674204E-02
Cnga2	3367	-0.004180534	4979	9.531502E-01
Cnga3	*3368*	0.073457216	10092	3.012635E-01
Cnga4	3369	0.021316405	6469	7.644793E-01
Cngb1	3370	-0.078863145	656	2.669851E-01
Cngb3	*3371*	0.109554681	12683	1.225210E-01
Cnih	3372	0.324949194	19977	2.669927E-06
Cnih2	3373	0.234957193	19270	8.111089E-04
Cnih3	*3374*	0.120403984	13687	8.945487E-02
Cnih4	3375	0.121021107	13728	8.781293E-02
Cnksr1	3376	-0.005535001	4899	9.379977E-01
Cnksr2	*3377*	0.105078114	12362	1.386554E-01
Cnksr3	3378	0.088410243	11234	2.131641E-01
Cnn1	3379	0.217214187	18824	2.004321E-03
Cnn2	*3380*	0.116194092	13408	1.013201E-01
Cnn3	3381	0.147408125	15501	3.725198E-02
Cnnm1	3382	0.177288042	17280	1.202388E-02
Cnnm2	*3383*	-0.011240472	4693	8.744782E-01
Cnnm3	3384	0.099399961	12026	1.613974E-01
Cnnm4	3385	0.038953452	7576	5.839392E-01
Cnot1	*3386*	-0.008446607	4804	9.055079E-01
Cnot10	3387	0.10303689	12245	1.465307E-01
Cnot11	3388	-0.027697273	4009	6.970408E-01
Cnot2	*3389*	0.095177621	11709	1.800428E-01
Cnot3	3390	0.107366779	12515	1.302147E-01
Cnot4	3391	0.172880679	17072	1.436363E-02
Cnot6	*3392*	0.096536853	11811	1.738748E-01
Cnot6l	3393	0.202799557	18349	3.976373E-03
Cnot7	3394	0.103881827	12290	1.432306E-01
Cnot8	*3395*	0.098810314	11980	1.639106E-01
Cnp	3396	0.191392498	17891	6.630045E-03

Spearman Rank correlation analysis performed between Prdm1 and all-expressed genes within the Meredith RNA-seq dataset. Robust Prdm1–associated genes were identified using a cut-off of $p < 0.0005$.

Table S1, Related to Supplemental Figure 3C. Prdm1 associated genes

Cnppd1	3397	0.157482855	16127	2.594062E-02
Cnpy1	*3398*	-0.086750697	472.5	2.219141E-01
Cnpy2	3399	0.226284949	19076	1.273035E-03
Cnpy3	3400	-0.051913642	2166	4.653533E-01
Cnpy4	*3401*	-0.031305766	3873	6.598913E-01
Cnr1	3402	0.04245963	7771	5.505238E-01
Cnr2	3403	0.282601205	19814	5.024363E-05
Cnrip1	*3404*	-0.057607724	1440	4.177891E-01
Cnst	3405	0.014813578	6063	8.350776E-01
Cntd1	3406	0.113548679	13094	1.093908E-01
Cntf	*3407*	0.230247091	19167.5	1.038230E-03
Cntfr	3408	0.072798883	10047.5	3.056282E-01
Cntln	3409	0.087155486	11142	2.197566E-01
Cntn1	*3410*	0.090237084	11366	2.038211E-01
Cntn2	3411	0.023161353	6701	7.447712E-01
Cntn3	3412	0.108754908	12606	1.252915E-01
Cntn4	*3413*	0.100178484	12074	1.581236E-01
Cntn5	3414	0.045565235	7971	5.217283E-01
Cntn6	3415	0.059293865	9175	4.042697E-01
Cntnap1	*3416*	0.096538046	11812	1.738695E-01
Cntnap2	3417	0.07500381	10250	2.911725E-01
Cntnap3	3418	-0.043558441	2390	5.402474E-01
Cntnap4	*3419*	0.07248392	10030	3.077310E-01
Cntnap5a	3420	0.067389457	9667	3.430605E-01
Cntnap5b	3421	0.062424037	9348	3.798718E-01
Cntnap5c	*3422*	0.037252326	7447	6.004823E-01
Cntrob	3423	0.088714796	11252	2.115856E-01
Coa3	3424	0.213510918	18698	2.400123E-03
Coa4	*3425*	0.066863463	9636	3.468492E-01
Coa5	3426	0.100929569	12116	1.550127E-01
Coa6	3427	0.054578295	8899	4.427320E-01
Coasy	*3428*	0.063582354	9442	3.710761E-01
Cobl	3429	0.04985195	8279	4.832843E-01
Cobll1	3430	0.02521128	6838	7.230670E-01
Coch	*3431*	0.182869536	17545	9.546176E-03
Cog1	3432	-0.018391103	4325	7.960356E-01
Cog2	3433	-0.020945493	4206	7.684602E-01
Cog3	*3434*	0.005331992	5575	9.402675E-01
Cog4	3435	0.098870224	11985	1.636539E-01
Cog5	3436	0.11760684	13500	9.720701E-02
Cog6	*3437*	0.15841664	16196	2.506018E-02
Cog7	3438	-0.009035226	4782	8.989570E-01
Cog8	3439	0.038458717	7524	5.887286E-01

Spearman Rank correlation analysis performed between Prdm1 and all-expressed genes within the Meredith RNA-seq dataset. Robust Prdm1–associated genes were identified using a cut-off of $p < 0.0005$.

Table S1, Related to Supplemental Figure 3C. Prdm1 associated genes

Coil	*3440*	0.110708928	12772	1.186068E-01
Col10a1	3441	0.220658701	18921.5	1.690525E-03
Col11a1	3442	0.069471461	9814	3.283226E-01
Col11a2	*3443*	0.020875719	6412.5	7.692098E-01
Col12a1	3444	0.144245491	15321	4.156769E-02
Col13a1	3445	0.017429875	6200	8.064803E-01
Col14a1	*3446*	-0.066290166	1294	3.510086E-01
Col15a1	3447	0.073627268	10106	3.001428E-01
Col16a1	3448	-0.055831836	1495	4.323099E-01
Col17a1	*3449*	0.031455842	7156	6.583634E-01
Col18a1	3450	0.037019973	7429	6.027581E-01
Col19a1	3451	0.10110209	12122	1.543047E-01
Col1a1	*3452*	0.17653292	17245	1.239921E-02
Col1a2	3453	0.2175743	18836	1.969194E-03
Col20a1	3454	0.006755553	5623	9.243623E-01
Col22a1	*3455*	0.004799858	5548	9.462194E-01
Col23a1	3456	0.182759623	17540	9.590234E-03
Col24a1	3457	0.102609741	12221	1.482209E-01
Col25a1	*3458*	0.088892833	11264	2.106667E-01
Col26a1	3459	0.1512252	15728	3.255322E-02
Col27a1	3460	0.092427349	11545	1.930138E-01
Col28a1	*3461*	-0.002645698	5043	9.703404E-01
Col2a1	3462	0.041362097	7705	5.608827E-01
Col3a1	3463	0.100913773	12114	1.550776E-01
Col4a1	*3464*	0.120594086	13698	8.894648E-02
Col4a2	3465	0.050214501	8319	4.801044E-01
Col4a3	3466	0.097860766	11899	1.680191E-01
Col4a3bp	*3467*	0.113893865	13247	1.083099E-01
Col4a4	3468	0.01495636	6069	8.335109E-01
Col4a5	3469	0.004191511	5526	9.530273E-01
Col4a6	*3470*	0.071429707	9945	3.148384E-01
Col5a1	3471	-0.020009293	4240	7.785351E-01
Col5a2	3472	0.005153222	5571	9.422667E-01
Col5a3	*3473*	-0.013436406	4596	8.502206E-01
Col6a1	3474	-0.037143255	3657	6.015501E-01
Col6a2	3475	-0.009034938	4783	8.989602E-01
Col6a3	*3476*	0.222376383	18965	1.551426E-03
Col6a4	3477	0.131662391	14380	6.311014E-02
Col6a5	3478	0.033650157	7273	6.361873E-01
Col6a6	*3479*	0.050834422	8363	4.746934E-01
Col7a1	3480	0.181737366	17473	1.000870E-02
Col8a1	3481	-0.051196631	2177	4.715473E-01
Col8a2	*3482*	0.068941254	9771	3.320365E-01

Spearman Rank correlation analysis performed between Prdm1 and all-expressed genes within the Meredith RNA-seq dataset. Robust Prdm1–associated genes were identified using a cut-off of $p < 0.0005$.

Table S1, Related to Supplemental Figure 3C. Prdm1 associated genes

Col9a1	3483	0.078911886	10527	2.666886E-01
Col9a2	3484	-0.052662801	2148	4.589297E-01
Col9a3	*3485*	-0.095267438	357.5	1.796303E-01
Colec10	3486	0.133999302	14551	5.853118E-02
Colec11	3487	-0.038406171	3065	5.892384E-01
Colec12	*3488*	0.094520659	11675	1.830811E-01
Colgalt1	3489	0.03530232	7343	6.197000E-01
Colgalt2	3490	0.055427664	8953	4.356548E-01
Colq	*3491*	0.109388648	12646	1.230922E-01
Commd1	3492	0.073413497	10087	3.015521E-01
Commd10	3493	0.19005812	17848	7.026309E-03
Commd2	*3494*	0.287799109	19854	3.590692E-05
Commd3	3495	0.008445476	5702	9.055205E-01
Commd4	3496	0.095650806	11746	1.778775E-01
Commd5	*3497*	0.061634704	9310	3.859379E-01
Commd6	3498	0.114617798	13299	1.060704E-01
Commd7	3499	-0.0630576	1361	3.750452E-01
Commd8	*3500*	0.102591466	12216	1.482935E-01
Commd9	3501	0.019032906	6283	7.890819E-01
Comp	3502	0.101466313	12146	1.528180E-01
Comt	*3503*	0.194602075	18029	5.757405E-03
Comtd1	3504	0.067032693	9646	3.456274E-01
Copa	3505	0.153544696	15868	2.995175E-02
Copb1	*3506*	0.15682489	16100	2.657682E-02
Copb2	3507	0.123689995	13886	8.098776E-02
Cope	3508	0.107644859	12533	1.292167E-01
Copg1	*3509*	0.200627053	18269	4.392157E-03
Copg2	3510	0.018891942	6272	7.906078E-01
Coprs	3511	0.020582982	6375	7.723568E-01
Cops2	*3512*	0.181656798	17469	1.004235E-02
Cops3	3513	0.022419089	6656	7.526811E-01
Cops4	3514	0.282735526	19815	4.981346E-05
Cops5	*3515*	0.025062297	6824	7.246372E-01
Cops6	3516	0.147578179	15512	3.703108E-02
Cops7a	3517	0.104799213	12345	1.397119E-01
Cops7b	*3518*	-0.057960113	1436	4.149420E-01
Cops8	3519	0.069827347	9833	3.258448E-01
Copz1	3520	0.275710902	19762	7.764157E-05
Copz2	*3521*	0.13779908	14810	5.167263E-02
Coq10a	3522	-0.097572869	296	1.692798E-01
Coq10b	3523	0.098376766	11945	1.657771E-01
Coq2	*3524*	0.064591022	9511	3.635197E-01
Coq3	3525	0.280515613	19802	5.738767E-05

Spearman Rank correlation analysis performed between Prdm1 and all-expressed genes within the Meredith RNA-seq dataset. Robust Prdm1–associated genes were identified using a cut-off of $p < 0.0005$.

Table S1, Related to Supplemental Figure 3C. Prdm1 associated genes

Coq4	3526	0.08038953	10624	2.578085E-01
Coq5	3527	-0.085062934	571	2.310716E-01
Coq6	3528	0.180945733	17444	1.034376E-02
Coq7	3529	0.285623986	19839	4.136015E-05
Coq9	3530	0.090792906	11413	2.010380E-01
Corin	3531	-0.077618291	665	2.746323E-01
Coro1a	3532	0.204008808	18392	3.760631E-03
Coro1b	3533	0.029245225	7032	6.810098E-01
CORO1B	3534		#N/A	1.000000E+00
Coro1c	3535	0.169712619	16913	1.628302E-02
Coro2a	3536	0.164752948	16640	1.973712E-02
Coro2b	3537	0.02650298	6902.5	7.095021E-01
Coro6	3538	0.053805701	8820	4.492258E-01
Coro7	3539	-0.094863839	377	1.814893E-01
Cort	3540		#N/A	1.000000E+00
Cotl1	3541	0.16317941	16552	2.095817E-02
Cox10	3542	0.171162832	16982	1.537827E-02
Cox11	3543	0.162433875	16494	2.155916E-02
Cox14	3544	-0.007587009	4836	9.150861E-01
Cox15	3545	0.140634215	14994	4.700175E-02
Cox16	3546		#N/A	1.000000E+00
Cox17	3547	0.194416184	18019	5.804993E-03
Cox18	3548	0.110331354	12743	1.198763E-01
Cox19	3549	0.084055694	10924	2.366620E-01
Cox20	3550	0.097710322	11884	1.686770E-01
Cox20-ps	3551	-0.054450825	1984	4.437997E-01
Cox4i1	3552	0.176788248	17255	1.227117E-02
Cox4i2	3553	-0.038406171	3065	5.892384E-01
Cox5a	3554	0.202400234	18339	4.050028E-03
Cox5b	3555	0.214942834	18739	2.239351E-03
Cox6a1	3556	0.138276017	14841	5.086094E-02
Cox6a2	3557	0.061063853	9264	3.903613E-01
Cox6b1	3558	0.074341925	10167	2.954631E-01
Cox6b2	3559	0.237835912	19323	6.958259E-04
Cox6c	3560	0.254893175	19563	2.700134E-04
Cox7a1	3561	0.002785337	5460	9.687757E-01
Cox7a2	3562	0.134471879	14587	5.763933E-02
Cox7a2l	3563	0.16196993	16467	2.194061E-02
Cox7b	3564	0.134087721	14558	5.836346E-02
Cox7b2	3565	-0.086475676	551	2.233884E-01
Cox7c	3566	0.166706174	16758	1.830758E-02
Cox8a	3567	0.163254798	16556	2.089822E-02
Cox8b	3568	-0.054450825	1984	4.437997E-01

Spearman Rank correlation analysis performed between Prdm1 and all-expressed genes within the Meredith RNA-seq dataset. Robust Prdm1–associated genes were identified using a cut-off of $p < 0.0005$.

Table S1, Related to Supplemental Figure 3C. Prdm1 associated genes

Cox8c	*3569*		#N/A	1.000000E+00
Cp	3570	0.107822355	12542	1.285828E-01
Cpa1	3571	-0.054450825	1984	4.437997E-01
Cpa2	*3572*	-0.004525891	4968	9.492848E-01
Cpa3	3573	-0.086748028	498.5	2.219283E-01
Cpa4	3574	0.020875719	6412.5	7.692098E-01
Cpa5	*3575*		#N/A	1.000000E+00
Cpa6	3576	-0.007905335	4825	9.115376E-01
Cpb1	3577		#N/A	1.000000E+00
Cpb2	*3578*	0.099773852	12047	1.598189E-01
Cpd	3579	0.000786981	5351	9.911757E-01
Cpe	3580	-0.000425434	5261	9.952296E-01
Cpeb1	*3581*	0.150972413	15711	3.284801E-02
Cpeb2	3582	0.095123979	11705	1.802895E-01
Cpeb3	3583	0.201857724	18319	4.152063E-03
Cpeb4	*3584*	0.130121151	14304	6.628786E-02
Cped1	3585	0.315096668	19954	5.506286E-06
Cphx1	3586		#N/A	1.000000E+00
Cphx3	*3587*	-0.077394997	736.5	2.760195E-01
Cplx1	3588	-0.017760534	4379.5	8.028834E-01
Cplx2	3589	0.273484217	19747	8.914878E-05
Cplx3	*3590*	-0.001224019	5151	9.862757E-01
Cplx4	3591	-0.018105422	4347	7.991361E-01
Cpm	3592	0.040464051	7659	5.694279E-01
Cpn1	*3593*	0.001570878	5394	9.823871E-01
Cpn2	3594	0.06350131	9434	3.716874E-01
Cpne1	3595	0.280975953	19806	5.573320E-05
Cpne2	*3596*	0.002540907	5444	9.715147E-01
Cpne3	3597	0.027645807	6958	6.975762E-01
Cpne4	3598	0.10338091	12265	1.451802E-01
Cpne5	*3599*	0.098581733	11962	1.648927E-01
Cpne6	3600	0.136623399	14736	5.371954E-02
Cpne7	3601	-0.016222939	4467	8.196432E-01
Cpne8	*3602*	0.260011105	19624	2.006312E-04
Cpne9	3603	0.133893988	14545	5.873148E-02
Cpox	3604	0.076855278	10406	2.793922E-01
Cpped1	*3605*	0.133647163	14522	5.920311E-02
Cpq	3606	0.157978965	16173	2.546960E-02
Cps1	3607	0.10680064	12482	1.322648E-01
Cpsf1	*3608*	0.178295355	17320	1.153876E-02
Cpsf2	3609	0.171871766	17011	1.495219E-02
Cpsf3	3610	0.039881076	7636	5.750079E-01
Cpsf3l	*3611*	0.081163278	10680	2.532407E-01

Spearman Rank correlation analysis performed between Prdm1 and all-expressed genes within the Meredith RNA-seq dataset. Robust Prdm1–associated genes were identified using a cut-off of $p < 0.0005$.

Table S1, Related to Supplemental Figure 3C. Prdm1 associated genes

Cpsf4	3612	0.211618584	18636	2.628668E-03
Cpsf4l	3613	-0.000841282	5208.5	9.905669E-01
Cpsf6	*3614*	0.194904097	18039	5.680832E-03
Cpsf7	3615	0.179117445	17364	1.115571E-02
Cpt1a	3616	0.079441739	10558	2.634807E-01
Cpt1b	*3617*		#N/A	1.000000E+00
Cpt1c	3618	-0.01419892	4560	8.418290E-01
Cpt2	3619	-0.01823252	4331	7.977563E-01
Cpvl	*3620*	-0.042309921	2428	5.519313E-01
Cpxcr1	3621	0.014301797	6015.5	8.406982E-01
Cpxm1	3622	0.221470905	18946	1.623392E-03
Cpxm2	*3623*		#N/A	1.000000E+00
Cpz	3624		#N/A	1.000000E+00
Cr1l	3625	0.117043598	13469	9.883066E-02
Cr2	*3626*	0.081997594	10746	2.483783E-01
CR293526.1	3627	-0.086750697	472.5	2.219141E-01
CR974586.1	3628		#N/A	1.000000E+00
CR974586.2	*3629*		#N/A	1.000000E+00
CR974586.3	3630		#N/A	1.000000E+00
CR974586.4	3631		#N/A	1.000000E+00
Crabp1	*3632*	-0.031503431	3866.5	6.578792E-01
Crabp2	3633	-0.011543767	4687	8.711203E-01
Cradd	3634	-0.068534333	962	3.349051E-01
Cramp1l	*3635*	0.019837077	6326	7.803924E-01
Crat	3636	0.044369568	7888	5.327230E-01
Crb1	3637	-0.00499136	4934	9.440771E-01
Crb2	*3638*	-0.054450825	1984	4.437997E-01
Crb3	3639	-0.021607471	4185	7.613596E-01
Crbn	3640	0.15794358	16172	2.550295E-02
Crcp	*3641*	0.135228384	14643	5.623501E-02
Crct1	3642	0.037226511	7445	6.007349E-01
Creb1	3643	0.03679358	7423	6.049792E-01
Creb3	*3644*	0.197703849	18149	5.013243E-03
Creb3l1	3645	0.150057697	15655	3.393375E-02
Creb3l2	3646	0.103808138	12286	1.435161E-01
Creb3l3	*3647*	-0.103159572	257	1.460480E-01
Creb3l4	3648		#N/A	1.000000E+00
Creb5	3649	0.081669522	10715	2.502825E-01
Crebbp	*3650*	0.0309042	7122	6.639866E-01
Crebl2	3651	0.153967334	15887	2.949735E-02
Crebrf	3652	0.257004273	19591	2.390553E-04
Crebzf	*3653*	0.043812734	7853	5.378828E-01
Creg1	3654	0.047853148	8130	5.010176E-01

Spearman Rank correlation analysis performed between Prdm1 and all-expressed genes within the Meredith RNA-seq dataset. Robust Prdm1–associated genes were identified using a cut-off of $p < 0.0005$.

Table S1, Related to Supplemental Figure 3C. Prdm1 associated genes

Creg2	3655	0.07902323	10533	2.660123E-01
Creld1	*3656*	0.082725826	10807	2.441874E-01
Creld2	3657	0.150578338	15686	3.331209E-02
Crem	3658	0.299439287	19906	1.651711E-05
Crh	*3659*	0.048881343	8210	4.918530E-01
Crhbp	3660	-0.017228458	4404	8.086733E-01
Crhr1	3661	0.014301797	6015.5	8.406982E-01
Crhr2	*3662*	0.177862052	17305	1.174529E-02
Crim1	3663	0.169800698	16917	1.622677E-02
Crip1	3664	-0.094905844	375	1.812952E-01
Crip2	*3665*	0.09501982	11698	1.807692E-01
Crip3	3666	0.046022306	8007	5.175561E-01
Cript	3667	0.084508254	10954	2.341385E-01
Crisp1	*3668*	-0.133121778	53	6.021742E-02
Crisp2	3669	-0.092349452	396	1.933908E-01
Crisp3	3670	0.053805701	8820	4.492258E-01
Crisp4	*3671*	-0.066858259	1029.5	3.468868E-01
Crispld1	3672	-0.054862425	1524	4.403573E-01
Crispld2	3673	0.167420724	16793	1.780756E-02
Crk	*3674*	0.144912824	15350	4.062365E-02
Crkl	3675	0.150201987	15663	3.376049E-02
Crlf1	3676		#N/A	1.000000E+00
Crlf2	*3677*	0.154983914	15977	2.842842E-02
Crlf3	3678	0.238769959	19345	6.617986E-04
Crls1	3679	0.097760976	11888	1.684553E-01
Crmp1	*3680*	0.047293351	8104	5.060448E-01
Crnkl1	3681	0.00887264	5723	9.007658E-01
Crnn	3682	-0.066855996	1239.5	3.469032E-01
Crocc	*3683*	-0.066858259	1029.5	3.468868E-01
Crot	3684	0.089010326	11274	2.100619E-01
Crp	3685	0.134753306	14607	5.711357E-02
Crry-ps	*3686*		#N/A	1.000000E+00
Crtac1	3687	0.053580006	8776	4.511329E-01
Crtam	3688	-0.001241011	5132	9.860852E-01
Crtap	*3689*	0.085099076	10994	2.308727E-01
Crtc1	3690	-0.119258427	107	9.256778E-02
Crtc2	3691	0.001836912	5411	9.794049E-01
Crtc3	*3692*	0.104227318	12309	1.418976E-01
Crx	3693	-0.001543167	5116	9.826977E-01
Crxos1	3694	0.02664691	6912	7.079962E-01
Cry1	*3695*	0.067473639	9672	3.424565E-01
Cry2	3696	0.186858137	17713	8.063826E-03
Cryaa	3697	-0.095267847	349	1.796284E-01

Spearman Rank correlation analysis performed between Prdm1 and all-expressed genes within the Meredith RNA-seq dataset. Robust Prdm1–associated genes were identified using a cut-off of $p < 0.0005$.

Table S1, Related to Supplemental Figure 3C. Prdm1 associated genes

Cryab	3698	0.017752724	6215	8.029683E-01
Cryba1	3699	-0.07739401	763.5	2.760257E-01
Cryba2	3700	0.209575308	18565	2.897460E-03
Cryba4	3701	-0.044269403	2360.5	5.336493E-01
Crybb1	3702	0.075893947	10339	2.854681E-01
Crybb2	3703	-0.018103658	4360	7.991553E-01
Crybb3	3704	-0.055062056	1521	4.386932E-01
Crybg3	3705	0.104953054	12351	1.391284E-01
Cryga	3706		#N/A	1.000000E+00
Crygb	3707	0.021316405	6469	7.644793E-01
Crygc	3708	0.052728522	8550.5	4.583686E-01
Crygd	3709	0.125961639	14043	7.552111E-02
Cryge	3710	0.109488806	12652	1.227474E-01
Crygf	3711		#N/A	1.000000E+00
Crygn	3712	0.021316405	6469	7.644793E-01
Crygs	3713		#N/A	1.000000E+00
Cryl1	3714	0.140226946	14970	4.765022E-02
Crym	3715	0.083454659	10873	2.400427E-01
Cryz	3716	0.071271838	9935	3.159119E-01
Cryzl1	3717		#N/A	1.000000E+00
Cs	3718	0.131052443	14354	6.435246E-02
Csad	3719	0.015661246	6101	8.257865E-01
Csdc2	3720		#N/A	1.000000E+00
Csde1	3721	0.128985607	14238	6.871185E-02
Cse1l	3722	0.249464522	19502	3.675700E-04
Csf1	3723	-0.007118269	4851	9.203143E-01
Csf1r	3724	0.024068775	6759	7.351376E-01
Csf2	3725	-0.0278972	4001.5	6.949626E-01
Csf2ra	3726	0.103014323	12244	1.466197E-01
Csf2rb	3727	0.175067721	17168	1.315697E-02
Csf2rb2	3728	-0.003586378	5002	9.598026E-01
Csf3	3729	-0.077393517	797	2.760287E-01
Csf3r	3730	0.0815268	10702	2.511140E-01
Csgalnact1	3731	0.152921711	15838	3.063242E-02
Csgalnact2	3732	0.037664281	7467	5.964568E-01
Csk	3733	0.115863544	13385	1.023020E-01
Csl	3734		#N/A	1.000000E+00
Csmd1	3735	-0.006489487	4869	9.273330E-01
Csmd2	3736	0.139963269	14949	4.807403E-02
Csmd3	3737	0.196897561	18117	5.197885E-03
Csn1s1	3738	0.013289408	5950	8.518403E-01
Csn1s2a	3739	-0.023549043	4135	7.406504E-01
Csn1s2b	3740	-0.065367286	1320.5	3.577700E-01

Spearman Rank correlation analysis performed between Prdm1 and all-expressed genes within the Meredith RNA-seq dataset. Robust Prdm1–associated genes were identified using a cut-off of $p < 0.0005$.

Table S1, Related to Supplemental Figure 3C. Prdm1 associated genes

Csn2	3741	-0.048500101	2228	4.952407E-01
Csn3	3742	0.028521017	6995	6.884927E-01
Csnk1a1	3743	0.15327648	15852	3.024320E-02
Csnk1d	3744	0.220471378	18916	1.706363E-03
Csnk1e	3745	0.099583464	12033	1.606212E-01
Csnk1g1	3746	0.081603483	10710	2.506670E-01
Csnk1g2	3747	0.083798149	10904	2.381065E-01
Csnk1g3	3748	0.220271101	18908	1.723447E-03
Csnk2a1	3749	0.246705544	19467	4.288437E-04
Csnk2a1-ps	3750		#N/A	1.000000E+00
Csnk2a2	3751	0.11221754	12868	1.136383E-01
Csnk2b	3752	0.077382804	10435	2.760954E-01
Csnka2ip	3753		#N/A	1.000000E+00
Cspg4	3754	0.175461156	17186	1.294961E-02
Cspg5	3755	-0.03087642	3893.5	6.642703E-01
Cspp1	3756	0.109768449	12697	1.217887E-01
Csprs	3757		#N/A	1.000000E+00
Csrnp1	3758		#N/A	1.000000E+00
Csrnp2	3759	0.040522903	7666	5.688660E-01
Csrnp3	3760	-0.010464407	4729	8.830803E-01
Csrp1	3761	0.116450375	13428	1.005639E-01
Csrp2	3762	-0.073556357	912	3.006098E-01
Csrp2bp	3763	0.123128676	13852	8.238655E-02
Csrp3	3764	0.075710566	10305	2.866371E-01
Cst10	3765	-0.086746694	532	2.219355E-01
Cst11	3766	-0.019476419	4255.5	7.842862E-01
Cst12	3767	0.007210755	5639	9.192825E-01
Cst13	3768		#N/A	1.000000E+00
Cst3	3769	0.193694635	17980	5.993058E-03
Cst6	3770	0.071589618	9957	3.137535E-01
Cst7	3771	0.103172005	12249	1.459992E-01
Cst8	3772	-0.027274045	4026	7.014477E-01
Cst9	3773	-0.047266735	2257	5.062844E-01
Csta	3774	0.021316405	6469	7.644793E-01
Cstad	3775	0.009422622	5744	8.946492E-01
Cstb	3776	0.16374755	16581	2.050997E-02
Cstf1	3777	0.097894102	11907	1.678736E-01
Cstf2	3778	0.194888627	18038	5.684732E-03
Cstf2t	3779	0.077152675	10420	2.775303E-01
Cstf3	3780	0.114134099	13261	1.075626E-01
Cstl1	3781		#N/A	1.000000E+00
CT009711.1	3782	-0.077393517	797	2.760287E-01
CT009715.1	3783	0.117939782	13522	9.625724E-02

Spearman Rank correlation analysis performed between Prdm1 and all-expressed genes within the Meredith RNA-seq dataset. Robust Prdm1–associated genes were identified using a cut-off of $p < 0.0005$.

Table S1, Related to Supplemental Figure 3C. Prdm1 associated genes

CT009757.1	3784	-0.038406171	3065	5.892384E-01
CT010433.1	3785	0.160304018	16323	2.335876E-02
CT010433.2	3786	0.214840127	18734	2.250548E-03
CT010439.1	3787	-0.04172333	2439	5.574630E-01
CT010583.1	3788	-0.038406171	3065	5.892384E-01
CT025557.1	3789		#N/A	1.000000E+00
CT025573.1	3790	0.113548679	13094	1.093908E-01
CT025592.1	3791	-0.054451513	1703	4.437940E-01
CT025592.2	3792	0.110455893	12754.5	1.194564E-01
CT025649.1	3793		#N/A	1.000000E+00
CT025653.1	3794		#N/A	1.000000E+00
CT025694.1	3795	-0.054451513	1703	4.437940E-01
CT030159.1	3796	0.106882912	12488	1.319654E-01
CT030166.1	3797		#N/A	1.000000E+00
CT030259.1	3798	-0.017997289	4369	8.003105E-01
CT030259.2	3799	0.101635452	12160	1.521312E-01
CT030259.3	3800		#N/A	1.000000E+00
CT030702.1	3801	0.225396681	19058	1.331947E-03
CT030736.1	3802	0.127284236	14137	7.247853E-02
CT030740.1	3803	-0.000108159	5281	9.987872E-01
CT033751.1	3804	0.182398428	17520.5	9.736288E-03
CT033751.2	3805	0.244418811	19431	4.866632E-04
CT033755.1	3806	0.006858698	5626	9.232110E-01
CT033785.1	3807		#N/A	1.000000E+00
CT485613.1	3808	0.22728507	19086	1.209568E-03
CT572983.1	3809	0.052009968	8471	4.645246E-01
CT572998.1	3810		#N/A	1.000000E+00
CT573100.1	3811	0.113548679	13094	1.093908E-01
CT868723.1	3812		#N/A	1.000000E+00
CT954326.1	3813	0.145563214	15382	3.972101E-02
Ctag2	3814		#N/A	1.000000E+00
Ctage5	3815	0.121728408	13775	8.596081E-02
Ctbp1	3816	0.182596721	17534	9.655864E-03
Ctbp2	3817	0.156129434	16056.5	2.726377E-02
Ctbs	3818	0.115526838	13359	1.033100E-01
Ctc1	3819	0.14279925	15135	4.367698E-02
Ctcf	3820	0.08742787	11156	2.183133E-01
Ctcfl	3821	0.037452981	7454	5.985200E-01
Ctdnep1	3822	0.179870005	17396	1.081493E-02
Ctdp1	3823	0.063598998	9443	3.709506E-01
Ctdsp1	3824	0.102460412	12210	1.488152E-01
Ctdsp2	3825	0.224044228	19019	1.426451E-03
Ctdspl	3826	-0.134961146	50	5.672782E-02

Spearman Rank correlation analysis performed between Prdm1 and all-expressed genes within the Meredith RNA-seq dataset. Robust Prdm1–associated genes were identified using a cut-off of $p < 0.0005$.

Table S1, Related to Supplemental Figure 3C. Prdm1 associated genes

Ctdspl2	3827	0.050409669	8333	4.783972E-01
Ctf1	3828	0.058760424	9145	4.085185E-01
Ctf2	3829	-0.038406171	3065	5.892384E-01
Ctgf	3830	-0.038406171	3065	5.892384E-01
Cth	3831	-0.016743206	4423	8.139627E-01
Cthrc1	3832	0.053267784	8685	4.537786E-01
Ctif	3833	0.022208006	6643	7.549352E-01
Ctla2a	3834	0.089677397	11319	2.066515E-01
Ctla2b	3835		#N/A	1.000000E+00
Ctla4	3836	0.070305057	9859	3.225378E-01
Ctnna1	3837	0.089464703	11308	2.077345E-01
Ctnna2	3838	0.116556414	13438	1.002523E-01
Ctnna3	3839	0.137502053	14790	5.218355E-02
Ctnnal1	3840	0.076583856	10386	2.810987E-01
Ctnnb1	3841	0.128675687	14225	6.938584E-02
Ctnnbip1	3842	0.033113809	7246	6.415789E-01
Ctnnbl1	3843	0.144755478	15341	4.084460E-02
Ctnnd1	3844	0.134450848	14586	5.767878E-02
Ctnnd2	3845	0.062488208	9355	3.793812E-01
Ctns	3846	0.017100287	6178	8.100695E-01
Ctps	3847	0.144086569	15315	4.179520E-02
Ctps2	3848	0.054257792	8880	4.454194E-01
Ctr9	3849	0.173736495	17109	1.388037E-02
Ctrb1	3850	-0.104232692	213	1.418769E-01
Ctrc	3851	0.180384025	17425	1.058748E-02
Ctrl	3852	-0.103159572	257	1.460480E-01
Cts3	3853		#N/A	1.000000E+00
Cts6	3854	-0.054451513	1703	4.437940E-01
Cts7	3855	-0.054451513	1703	4.437940E-01
Cts8	3856	0.067806315	9690	3.400766E-01
Ctsa	3857	0.003782594	5506	9.576053E-01
Ctsb	3858	0.125567505	14009	7.644755E-02
Ctsc	3859	0.186136983	17688	8.315642E-03
Ctsd	3860	0.167250722	16784	1.792543E-02
Ctse	3861	0.236038451	19289	7.658890E-04
Ctsf	3862	-0.103161098	248	1.460420E-01
Ctsg	3863		#N/A	1.000000E+00
Ctsh	3864	0.163011044	16547	2.109261E-02
Ctsj	3865		#N/A	1.000000E+00
Ctsk	3866	-0.054450825	1984	4.437997E-01
Ctsl	3867	0.075947791	10346	2.851254E-01
Ctsll3	3868		#N/A	1.000000E+00
Ctsm	3869		#N/A	1.000000E+00

Spearman Rank correlation analysis performed between Prdm1 and all-expressed genes within the Meredith RNA-seq dataset. Robust Prdm1–associated genes were identified using a cut-off of $p < 0.0005$.

Table S1, Related to Supplemental Figure 3C. Prdm1 associated genes

Ctso	3870	0.195439792	18061	5.547256E-03
Ctsq	3871		#N/A	1.000000E+00
Ctsr	3872		#N/A	1.000000E+00
Ctss	3873	0.228627615	19106	1.128929E-03
Ctsw	3874	0.094812763	11691	1.817256E-01
Ctsz	3875	0.132738166	14460	6.096702E-02
Cttn	3876	0.175406402	17183	1.297830E-02
Cttnbp2	3877	0.138980035	14887	4.968217E-02
Cttnbp2nl	3878	-0.033178769	3779	6.409249E-01
Ctu1	3879	-0.187721732	2	7.771203E-03
Ctu2	3880		#N/A	1.000000E+00
Ctxn1	3881	0.121661436	13770	8.613483E-02
Ctxn2	3882	0.270943938	19725	1.042225E-04
Ctxn3	3883	0.049853204	8280.5	4.832733E-01
Cubn	3884	0.103929723	12292	1.430452E-01
Cuedc1	3885	0.122420926	13812	8.417788E-02
Cuedc2	3886	-0.07965345	649	2.622063E-01
Cul1	3887	0.16604279	16706	1.878268E-02
Cul2	3888	0.066298093	9609	3.509509E-01
Cul3	3889	0.23354304	19243	8.739623E-04
Cul4a	3890	0.112285824	12876	1.134173E-01
Cul4b	3891	0.054592493	8900	4.426132E-01
Cul5	3892	0.04627836	8033	5.152264E-01
Cul7	3893		#N/A	1.000000E+00
Cul9	3894	0.111966339	12851	1.144541E-01
Cuta	3895	0.115676259	13372	1.028617E-01
Cutc	3896	-0.033930747	3741	6.333743E-01
Cux1	3897	0.18493114	17630	8.752328E-03
Cux2	3898	-0.010724482	4714	8.801960E-01
Cuzd1	3899		#N/A	1.000000E+00
Cwc15	3900	0.056284795	8998	4.285789E-01
Cwc22	3901	0.004991945	5563	9.440705E-01
Cwc25	3902	0.097367942	11862	1.701815E-01
Cwc27	3903	0.050139917	8307	4.807576E-01
Cwf19l1	3904	-0.068925666	960	3.321461E-01
Cwf19l2	3905	0.138938824	14879	4.975054E-02
Cwh43	3906	-0.018105422	4347	7.991361E-01
Cx3cl1	3907	0.075734994	10325	2.864812E-01
Cx3cr1	3908	0.208929239	18546	2.987491E-03
Cxadr	3909	0.15071622	15694	3.314909E-02
Cxcl1	3910	-0.029761321	3938	6.756962E-01
Cxcl10	3911	0.107798274	12541	1.286686E-01
Cxcl11	3912	0.058012237	9087	4.145219E-01

Spearman Rank correlation analysis performed between Prdm1 and all-expressed genes within the Meredith RNA-seq dataset. Robust Prdm1–associated genes were identified using a cut-off of $p < 0.0005$.

Table S1, Related to Supplemental Figure 3C. Prdm1 associated genes

Cxcl12	3913	0.098813378	11981	1.638975E-01
Cxcl13	3914	0.057912417	9081	4.153267E-01
Cxcl14	3915	-0.029726549	3939	6.760537E-01
Cxcl16	3916	0.04736617	8107	5.053893E-01
Cxcl17	3917	0.053805701	8820	4.492258E-01
Cxcl2	3918	0.102826503	12235	1.473614E-01
Cxcl3	3919	0.146272995	15431	3.875528E-02
Cxcl5	3920	-0.019133303	4265.5	7.879956E-01
Cxcl9	3921	0.126129577	14054	7.512914E-02
Cxcr1	3922		#N/A	1.000000E+00
Cxcr2	3923	0.00213502	5425	9.760634E-01
Cxcr3	3924	0.021100249	6443	7.667985E-01
Cxcr4	3925	0.14860903	15567	3.571551E-02
Cxcr5	3926	0.072215154	10010	3.095329E-01
Cxcr6	3927	0.152272071	15784	3.135622E-02
Cxcr7	3928	0.104550343	12327	1.406599E-01
Cxx1a	3929	0.137332099	14778.5	5.247777E-02
Cxx1b	3930	0.153837618	15881	2.963618E-02
Cxx1c	3931	-0.038406171	3065	5.892384E-01
Cxxc1	3932	0.161586539	16453	2.226023E-02
Cxxc4	3933	0.159709731	16261	2.388342E-02
Cxxc5	3934	0.003746422	5504	9.580104E-01
Cyb5	3935	0.024085427	6761	7.349612E-01
Cyb561	3936	-0.089575821	421	2.071682E-01
Cyb561a3	3937	-0.077393517	797	2.760287E-01
Cyb561d1	3938	0.117389843	13483	9.783002E-02
Cyb561d2	3939	0.14607758	15420	3.901916E-02
Cyb5b	3940	0.105387212	12384	1.374916E-01
Cyb5d1	3941	-0.054451513	1703	4.437940E-01
Cyb5d2	3942	0.300729841	19910	1.512299E-05
Cyb5r1	3943	0.14899401	15591	3.523445E-02
Cyb5r2	3944	-0.066855996	1239.5	3.469032E-01
Cyb5r3	3945	0.091011565	11438	1.999507E-01
Cyb5r4	3946	0.049548011	8257	4.859588E-01
Cyb5rl	3947	0.034690783	7321	6.257811E-01
Cyba	3948	0.056123305	8992	4.299070E-01
Cybb	3949	0.10010158	12070	1.584447E-01
Cybrd1	3950	-0.02051289	4220	7.731109E-01
Cyc1	3951	0.045833507	7992	5.192774E-01
Cycs	3952	0.191965904	17908	6.465979E-03
Cyct	3953		#N/A	1.000000E+00
Cyfip1	3954	0.0251834	6834	7.233607E-01
Cyfip2	3955	0.147093957	15482	3.766303E-02

Spearman Rank correlation analysis performed between Prdm1 and all-expressed genes within the Meredith RNA-seq dataset. Robust Prdm1–associated genes were identified using a cut-off of $p < 0.0005$.

Table S1, Related to Supplemental Figure 3C. Prdm1 associated genes

Cygb	3956	0.142562843	15118	4.403015E-02
Cyhr1	3957	0.088018043	11206	2.152094E-01
Cylc1	3958		#N/A	1.000000E+00
Cylc2	3959	-0.038406171	3065	5.892384E-01
Cyld	3960	0.154485367	15954	2.894843E-02
Cym	3961	0.051912239	8464	4.653653E-01
Cyp11a1	3962		#N/A	1.000000E+00
Cyp11b1	3963		#N/A	1.000000E+00
Cyp11b2	3964		#N/A	1.000000E+00
Cyp17a1	3965	-0.077393023	832.5	2.760318E-01
Cyp19a1	3966	0.026735483	6913	7.070700E-01
Cyp1a1	3967	0.022198316	6629.5	7.550388E-01
Cyp1a2	3968	0.180448558	17427	1.055922E-02
Cyp1b1	3969	0.102840458	12236	1.473062E-01
Cyp20a1	3970	0.016566657	6149	8.158893E-01
Cyp21a1	3971		#N/A	1.000000E+00
Cyp21a2-ps	3972		#N/A	1.000000E+00
Cyp24a1	3973	0.127545848	14155	7.188866E-02
Cyp26a1	3974	-0.016563575	4455	8.159229E-01
Cyp26b1	3975	0.03192256	7183	6.536208E-01
Cyp26c1	3976	-0.038406171	3065	5.892384E-01
Cyp27a1	3977	-0.017630547	4384	8.042969E-01
Cyp27b1	3978	-0.038406171	3065	5.892384E-01
Cyp2a12	3979	0.164478601	16623.5	1.994547E-02
Cyp2a22	3980		#N/A	1.000000E+00
Cyp2a4	3981	-0.038406171	3065	5.892384E-01
Cyp2a5	3982	-0.058931392	1418	4.071539E-01
Cyp2ab1	3983	-0.038406171	3065	5.892384E-01
Cyp2b10	3984	0.051998862	8470	4.646201E-01
Cyp2b13	3985	0.191624125	17899.5	6.563329E-03
Cyp2b19	3986	0.090719737	11405	2.014028E-01
Cyp2b23	3987	0.002119669	5422.5	9.762355E-01
Cyp2b9	3988		#N/A	1.000000E+00
Cyp2c29	3989	0.04461475	7907	5.304590E-01
Cyp2c37	3990	0.021316405	6469	7.644793E-01
Cyp2c38	3991	-0.038406171	3065	5.892384E-01
Cyp2c39	3992	0.090858474	11420.5	2.007115E-01
Cyp2c40	3993	0.113548679	13094	1.093908E-01
Cyp2c44	3994	-0.054451513	1703	4.437940E-01
Cyp2c50	3995	0.075715395	10317.5	2.866063E-01
Cyp2c53-ps	3996	-0.077393517	797	2.760287E-01
Cyp2c54	3997	0.143605682	15224	4.249002E-02
Cyp2c55	3998	0.216630157	18807.5	2.062506E-03

Spearman Rank correlation analysis performed between Prdm1 and all-expressed genes within the Meredith RNA-seq dataset. Robust Prdm1–associated genes were identified using a cut-off of $p < 0.0005$.

Table S1, Related to Supplemental Figure 3C. Prdm1 associated genes

Cyp2c65	3999	0.143605682	15224	4.249002E-02
Cyp2c66	4000	0.118423938	13553	9.488927E-02
Cyp2c67	4001	0.137500499	14788.5	5.218623E-02
Cyp2c68	4002	0.113548679	13094	1.093908E-01
Cyp2c69	4003	-0.038406171	3065	5.892384E-01
Cyp2c70	4004	0.176086238	17227.5	1.262605E-02
Cyp2d10	4005		#N/A	1.000000E+00
Cyp2d11	4006		#N/A	1.000000E+00
Cyp2d12	4007	0.180827196	17441	1.039478E-02
Cyp2d22	4008	0.156129434	16056.5	2.726377E-02
Cyp2d26	4009	-0.077393517	797	2.760287E-01
Cyp2d34	4010	0.031631143	7166	6.565804E-01
Cyp2d37-ps	4011		#N/A	1.000000E+00
Cyp2d40	4012	-0.077393517	797	2.760287E-01
Cyp2d9	4013	0.037700968	7469	5.960989E-01
Cyp2e1	4014	-0.00694057	4856	9.222972E-01
Cyp2f2	4015	0.176525741	17244	1.240283E-02
Cyp2g1	4016	0.010004366	5772	8.881861E-01
Cyp2j11	4017	-0.054451513	1703	4.437940E-01
Cyp2j12	4018	0.030939332	7124	6.636279E-01
Cyp2j13	4019	0.001172547	5371	9.868527E-01
Cyp2j5	4020	0.090858474	11420.5	2.007115E-01
Cyp2j6	4021	0.042490456	7773	5.502342E-01
Cyp2j7-ps	4022		#N/A	1.000000E+00
Cyp2j8	4023	0.076500612	10381	2.816235E-01
Cyp2j9	4024	0.0912448	11461	1.987957E-01
Cyp2r1	4025		#N/A	1.000000E+00
Cyp2s1	4026	-0.05397389	2116	4.478076E-01
Cyp2t4	4027		#N/A	1.000000E+00
Cyp2u1	4028	0.012386327	5896	8.618043E-01
Cyp2w1	4029		#N/A	1.000000E+00
Cyp39a1	4030	0.055831852	8974	4.323098E-01
Cyp3a11	4031	0.045741501	7985	5.201173E-01
Cyp3a13	4032	0.07424677	10158	2.960834E-01
Cyp3a16	4033		#N/A	1.000000E+00
Cyp3a25	4034	0.226732518	19080	1.244265E-03
Cyp3a41a	4035		#N/A	1.000000E+00
Cyp3a41b	4036	0.113548679	13094	1.093908E-01
Cyp3a44	4037	0.114826602	13313	1.054312E-01
Cyp3a57	4038	0.095286212	11719	1.795442E-01
Cyp3a59	4039	0.138076839	14829.5	5.119862E-02
Cyp46a1	4040		#N/A	1.000000E+00
Cyp4a10	4041		#N/A	1.000000E+00

Spearman Rank correlation analysis performed between Prdm1 and all-expressed genes within the Meredith RNA-seq dataset. Robust Prdm1–associated genes were identified using a cut-off of $p < 0.0005$.

Table S1, Related to Supplemental Figure 3C. Prdm1 associated genes

Cyp4a12a	4042	-0.038406171	3065	5.892384E-01
Cyp4a12b	4043	-0.038406171	3065	5.892384E-01
Cyp4a14	4044		#N/A	1.000000E+00
Cyp4a28-ps	4045		#N/A	1.000000E+00
Cyp4a29-ps	4046		#N/A	1.000000E+00
Cyp4a30b-ps	4047		#N/A	1.000000E+00
Cyp4a31	4048	-0.054451513	1703	4.437940E-01
Cyp4a32	4049	0.24708487	19474	4.198920E-04
Cyp4b1	4050	-0.038406171	3065	5.892384E-01
Cyp4f13	4051	-0.085088033	570	2.309335E-01
Cyp4f14	4052	0.156129434	16056.5	2.726377E-02
Cyp4f15	4053	0.08917136	11285	2.092349E-01
Cyp4f16	4054	0.022414248	6655	7.527328E-01
Cyp4f17	4055	0.161633006	16455	2.222128E-02
Cyp4f18	4056	-0.013636015	4584	8.480222E-01
Cyp4f39	4057	0.090013747	11344.5	2.049472E-01
Cyp4f40	4058	0.18218536	17494	9.823364E-03
Cyp4f41-ps	4059	-0.077393517	797	2.760287E-01
Cyp4v3	4060	0.26733108	19701	1.298100E-04
Cyp4x1	4061	-0.054451513	1703	4.437940E-01
Cyp51	4062	0.082388017	10780	2.461253E-01
Cyp7a1	4063	-0.066856562	1139	3.468991E-01
Cyp7b1	4064	0.196140179	18090	5.376858E-03
Cyp8b1	4065	-0.038406171	3065	5.892384E-01
Cypt1	4066		#N/A	1.000000E+00
Cypt12	4067		#N/A	1.000000E+00
Cypt14	4068		#N/A	1.000000E+00
Cypt15	4069		#N/A	1.000000E+00
Cypt2	4070		#N/A	1.000000E+00
Cypt3	4071	-0.038406171	3065	5.892384E-01
Cypt4	4072		#N/A	1.000000E+00
Cyr61	4073	0.030786003	7115	6.651939E-01
Cys1	4074	-0.038406171	3065	5.892384E-01
Cysltr1	4075	0.075715395	10317.5	2.866063E-01
Cysltr2	4076	0.075183564	10256	2.900144E-01
Cystm1	4077	-0.01472462	4524	8.360539E-01
Cyth1	4078	0.154442048	15921	2.899400E-02
Cyth2	4079	0.159256812	16240	2.429007E-02
Cyth3	4080	0.046055799	8015	5.172511E-01
Cyth4	4081	-0.091022343	408	1.998973E-01
Cytip	4082	0.211298002	18625	2.669285E-03
Cytl1	4083	-0.136803985	45	5.340083E-02
Cyyr1	4084	0.073412947	10086	3.015557E-01

Spearman Rank correlation analysis performed between Prdm1 and all-expressed genes within the Meredith RNA-seq dataset. Robust Prdm1–associated genes were identified using a cut-off of $p < 0.0005$.

Table S1, Related to Supplemental Figure 3C. Prdm1 associated genes

D030018L15Ril	4085	-0.110581467	154.5	1.190342E-01
D030025P21Ri	4086	0.146474008	15446	3.848540E-02
D030028A08Ri	4087	0.018300674	6239	7.970167E-01
D030046N08Ri	4088		#N/A	1.000000E+00
D030047H15Ri	4089	-0.007947234	4823	9.110707E-01
D030055H07Ri	4090	0.144516882	15334	4.118157E-02
D030056L22Ril	4091	0.094338414	11661	1.839306E-01
D030062O11Ri	4092	-0.054450825	1984	4.437997E-01
D10Bwg1379e	4093	0.251207346	19522	3.331612E-04
D10Jhu81e	4094	0.105760258	12406	1.360970E-01
D10Wsu102e	4095	-0.01349353	4593	8.495914E-01
D10Wsu52e	4096	0.095952835	11771	1.765055E-01
D11Bhm181e	4097		#N/A	1.000000E+00
D11Wsu47e	4098	-0.015720333	4488	8.251397E-01
D130007C19Ri	4099	-0.066855996	1239.5	3.469032E-01
D130040H23Ri	4100	0.119365921	13613	9.227206E-02
D130043K22Ri	4101	0.159196659	16236	2.434452E-02
D130052B06Ri	4102		#N/A	1.000000E+00
D130058E05Ril	4103		#N/A	1.000000E+00
D130062J21Rik	4104		#N/A	1.000000E+00
D13Ertd608e	4105		#N/A	1.000000E+00
D14Abb1e	4106	0.120926472	13720	8.806314E-02
D15Ertd621e	4107	0.234922889	19269	8.125830E-04
D16Ertd472e	4108	0.046215092	8028	5.158015E-01
D17H6S53E	4109	0.036299592	7397	6.098383E-01
D17H6S56E-5	4110	0.172935827	17074	1.433205E-02
D17Wsu104e	4111	0.086986747	11127	2.206541E-01
D17Wsu92e	4112	0.099755176	12045	1.598974E-01
D19Bwg1357e	4113	0.150003285	15651	3.399928E-02
D19Ertd737e	4114	0.034920164	7326	6.234972E-01
D1Ertd622e	4115	0.092916238	11577	1.906596E-01
D1Pas1	4116	0.162808768	16526.5	2.125512E-02
D230002A01Ri	4117		#N/A	1.000000E+00
D230022J07Rik	4118	-0.067178213	975	3.445789E-01
D230025D16Ri	4119	0.11968365	13636	9.140236E-02
D2hgdh	4120	0.0013795	5383	9.845325E-01
D2Wsu81e	4121	-0.012390013	4652	8.617636E-01
D330020A13Ri	4122		#N/A	1.000000E+00
D330022K07Ri	4123	0.020414096	6347	7.741742E-01
D330023K18Ri	4124	-0.096243034	307	1.751947E-01
D330041H03Ri	4125	0.080608939	10641	2.565075E-01
D330045A20Ri	4126	0.046903336	8069	5.095627E-01
D330050G23Ri	4127	0.021317126	6513.5	7.644715E-01

Spearman Rank correlation analysis performed between Prdm1 and all-expressed genes within the Meredith RNA-seq dataset. Robust Prdm1–associated genes were identified using a cut-off of $p < 0.0005$.

Table S1, Related to Supplemental Figure 3C. Prdm1 associated genes

D3Bwg0562e	4128	0.219051398	18876	1.830901E-03
D3Ertd254e	4129	0.021710506	6562	7.602562E-01
D3Ertd751e	4130	-0.028431257	3983	6.894222E-01
D430001F17Ril	4131	-0.117606715	112	9.720737E-02
D430019H16Ri	4132	0.017344733	6193	8.074071E-01
D430040D24Ri	4133	0.002889057	5467	9.676137E-01
D430041D05Ri	4134	-0.073748814	910	2.993434E-01
D430042O09Ri	4135	0.00247089	5441	9.722993E-01
D4Wsu53e	4136	0.021550868	6557	7.619660E-01
D530049N12Ri	4137		#N/A	1.000000E+00
D5Ertd577e	4138	-0.038406171	3065	5.892384E-01
D5Ertd579e	4139	0.035679641	7359	6.159607E-01
D630002J18Rik	4140		#N/A	1.000000E+00
D630003M21R	4141	0.225551504	19062	1.321502E-03
D630004N19Ri	4142		#N/A	1.000000E+00
D630008O14Ri	4143	0.003850092	5511	9.568496E-01
D630011A20Ri	4144		#N/A	1.000000E+00
D630014O11Ri	4145		#N/A	1.000000E+00
D630023F18Ril	4146	0.001698577	5406	9.809555E-01
D630024D03Ri	4147	0.053805701	8820	4.492258E-01
D630028G08Ri	4148		#N/A	1.000000E+00
D630029K05Ri	4149	-0.038406171	3065	5.892384E-01
D630032N06Ri	4150	-0.027457748	4016	6.995337E-01
D630033O11Ri	4151	-0.077393517	797	2.760287E-01
D630036H23Ri	4152		#N/A	1.000000E+00
D630037F22Ril	4153	0.088626723	11247	2.120412E-01
D630039A03Ri	4154	-0.038406171	3065	5.892384E-01
D630044L22Ril	4155		#N/A	1.000000E+00
D630045J12Rik	4156	0.26631744	19687	1.379807E-04
D6Ertd474e	4157	0.006716541	5621	9.247978E-01
D6Ertd527e	4158	-0.038406171	3065	5.892384E-01
D6Wsu163e	4159	0.152774977	15810	3.079465E-02
D730003I15Rik	4160		#N/A	1.000000E+00
D730039F16Ril	4161	0.148488939	15561	3.586670E-02
D730045A05Ri	4162		#N/A	1.000000E+00
D730045B01Ri	4163		#N/A	1.000000E+00
D730048I06Rik	4164	-0.066855996	1239.5	3.469032E-01
D7Ertd443e	4165	-0.000465734	5245	9.947777E-01
D830013O20Ri	4166	-0.038406171	3065	5.892384E-01
D830014E11Ril	4167		#N/A	1.000000E+00
D830015G02Ri	4168	-0.038406171	3065	5.892384E-01
D830025C05Ri	4169	-0.066858259	1029.5	3.468868E-01
D830026I12Rik	4170	-0.038406171	3065	5.892384E-01

Spearman Rank correlation analysis performed between Prdm1 and all-expressed genes within the Meredith RNA-seq dataset. Robust Prdm1–associated genes were identified using a cut-off of $p < 0.0005$.

Table S1, Related to Supplemental Figure 3C. Prdm1 associated genes

D830030K20Ri	4171	-0.045438257	2313.5	5.228904E-01
D830039M14R	4172		#N/A	1.000000E+00
D830044D21Ri	4173	-0.038406171	3065	5.892384E-01
D830046C22Ri	4174		#N/A	1.000000E+00
D830050J10Rik	4175	-0.032131498	3842.5	6.515021E-01
D8Ertd738e	4176	0.10377585	12283	1.436414E-01
D8Ertd82e	4177	0.088245113	11225	2.140235E-01
D930007J09Rik	4178		#N/A	1.000000E+00
D930015E06Ril	4179	0.139831548	14943	4.828691E-02
D930015M05R	4180	-0.066856562	1139	3.468991E-01
D930020B18Ri	4181	0.081011829	10663	2.541303E-01
D930028M14R	4182		#N/A	1.000000E+00
D930048N14Ri	4183	0.126933796	14110.5	7.327484E-02
Daam1	4184	0.147736807	15521	3.682601E-02
Daam2	4185	-0.077394997	736.5	2.760195E-01
Dab1	4186	0.082190886	10759	2.472611E-01
Dab2	4187	0.047228893	8102	5.066253E-01
Dab2ip	4188	0.155789091	16025	2.760545E-02
Dach1	4189	0.194151389	18003	5.873387E-03
Dach2	4190	0.151172886	15725	3.261404E-02
Dact1	4191	-0.019133303	4265.5	7.879956E-01
Dact2	4192	0.176601372	17248	1.236477E-02
Dact3	4193	0.023956928	6747	7.363228E-01
Dad1	4194	0.216080291	18788	2.118688E-03
Daf2	4195	-0.09526621	368.5	1.796359E-01
Dag1	4196	-0.003083382	5020	9.654366E-01
Dagla	4197	-0.04310276	2409.5	5.444975E-01
Daglb	4198	0.069656001	9826	3.270362E-01
Dak	4199	-0.024185002	4125	7.339066E-01
Dalrd3	4200	0.150000244	15650	3.400295E-02
Dancr	4201	0.023219662	6706	7.441510E-01
Dand5	4202	0.165758767	16689	1.898934E-02
Dao	4203	0.071763446	9975	3.125769E-01
Dap	4204	0.13988966	14945	4.819290E-02
Dap3	4205	0.160276881	16291	2.338250E-02
Dapk1	4206	0.212289484	18664	2.545474E-03
Dapk2	4207	0.034410247	7309	6.285793E-01
Dapk3	4208	0.130121316	14305	6.628751E-02
Dapl1	4209	0.192152724	17918	6.413313E-03
Dapp1	4210	0.061414984	9292	3.876369E-01
Darc	4211	-0.038406171	3065	5.892384E-01
Dars	4212	0.150825955	15703	3.301984E-02
Dars2	4213	0.071868171	9988	3.118695E-01

Spearman Rank correlation analysis performed between Prdm1 and all-expressed genes within the Meredith RNA-seq dataset. Robust Prdm1–associated genes were identified using a cut-off of $p < 0.0005$.

Table S1, Related to Supplemental Figure 3C. Prdm1 associated genes

Daw1	4214	0.155338244	15995	2.806371E-02
Daxx	4215	0.153027164	15842	3.051629E-02
Dazap1	4216	0.211717845	18642	2.616206E-03
Dazap2	4217	0.199110269	18206	4.705209E-03
Dazl	4218	0.208934416	18547	2.986759E-03
Dbc1	4219	-0.033500503	3763	6.376898E-01
Dbf4	4220	0.081673559	10716	2.502590E-01
Dbh	4221	-0.076074634	863	2.843194E-01
Dbi	4222	0.103194767	12252	1.459098E-01
Dbil5	4223	-0.004782526	4949	9.464133E-01
Dbn1	4224	0.081751252	10726	2.498071E-01
Dbndd1	4225	-0.038406171	3065	5.892384E-01
Dbndd2	4226	-0.059870047	1398	3.997102E-01
Dbnl	4227	0.098187437	11929	1.665971E-01
Dbp	4228	0.024611236	6795	7.293982E-01
Dbpht2	4229	-0.000841282	5208.5	9.905669E-01
Dbr1	4230	0.114646945	13302	1.059810E-01
Dbt	4231	-0.002511032	5049	9.718494E-01
Dbx1	4232	0.052728522	8550.5	4.583686E-01
Dbx2	4233	-0.153030191	20	3.051296E-02
Dcaf10	4234	0.293065852	19875	2.537469E-05
Dcaf11	4235	0.034080038	7297	6.318798E-01
Dcaf12	4236	0.230445178	19175	1.027608E-03
Dcaf12l1	4237	0.18254467	17533	9.676917E-03
Dcaf12l2	4238	0.091690749	11501	1.966007E-01
Dcaf13	4239	0.025214092	6839	7.230373E-01
Dcaf15	4240	0.024786526	6804	7.275467E-01
Dcaf17	4241	0.149667463	15628	3.440613E-02
Dcaf4	4242	-0.000350896	5272	9.960654E-01
Dcaf5	4243	0.122424442	13813	8.416890E-02
Dcaf6	4244	0.062509913	9357	3.792154E-01
Dcaf7	4245	0.079228381	10547	2.647693E-01
Dcaf8	4246	0.088009705	11204	2.152530E-01
Dcakd	4247	0.192249645	17922	6.386142E-03
Dcbld1	4248	0.0648644	9529	3.614883E-01
Dcbld2	4249	0.019673591	6315	7.821568E-01
Dcc	4250	0.144696244	15338	4.092804E-02
Dcdc2a	4251	0.097870786	11903	1.679754E-01
Dcdc2b	4252	0.088187776	11217	2.143225E-01
Dcdc2c	4253	-0.038406171	3065	5.892384E-01
Dcdc5	4254	0.12433814	13930	7.939647E-02
Dchs1	4255	0.134955226	14616	5.673878E-02
Dck	4256	0.277553451	19777	6.918903E-05

Spearman Rank correlation analysis performed between Prdm1 and all-expressed genes within the Meredith RNA-seq dataset. Robust Prdm1–associated genes were identified using a cut-off of $p < 0.0005$.

Table S1, Related to Supplemental Figure 3C. Prdm1 associated genes

Dclk1	4257	-0.020324095	4229	7.751431E-01
Dclk2	4258	0.17018461	16939	1.598359E-02
Dclk3	4259	0.053267784	8685	4.537786E-01
Dclre1a	4260	0.062235092	9339	3.813186E-01
Dclre1b	4261	0.156615991	16085	2.678159E-02
Dclre1c	4262	0.043395046	7829	5.417694E-01
Dcn	4263	0.119340236	13610	9.234265E-02
Dcp1a	4264	0.063561776	9439	3.712312E-01
Dcp1b	4265	0.184006486	17591	9.100837E-03
Dcp2	4266	0.000400162	5337	9.955130E-01
Dcpp1	4267	-0.004644149	4952	9.479615E-01
Dcpp2	4268	-0.017417228	4393	8.066179E-01
Dcpp3	4269	0.020434681	6357	7.739526E-01
Dcps	4270	0.263362411	19663	1.646280E-04
Dcst1	4271	-0.086746694	532	2.219355E-01
Dcstamp	4272	0.07521908	10268	2.897860E-01
Dct	4273	-0.038406171	3065	5.892384E-01
Dctd	4274	0.143867514	15302	4.211052E-02
Dctn1	4275	0.189422857	17819	7.222271E-03
Dctn2	4276	0.135829927	14683	5.513863E-02
Dctn3	4277	0.079317976	10551	2.642276E-01
Dctn4	4278	0.024284914	6773	7.328490E-01
Dctn5	4279	0.076192101	10360	2.835742E-01
Dctn6	4280	0.252502149	19533	3.095612E-04
Dctpp1	4281	0.140616282	14993	4.703015E-02
Dcun1d1	4282	0.027879025	6968	6.951514E-01
Dcun1d2	4283	0.065559436	9563	3.563556E-01
Dcun1d3	4284	-0.074937871	885	2.915980E-01
Dcun1d4	4285	0.060840238	9252	3.921024E-01
Dcun1d5	4286	0.15825028	16189	2.521513E-02
Dcx	4287	0.015597548	6098	8.264839E-01
Dcxr	4288	0.150166118	15661	3.380349E-02
Dda1	4289	0.15658512	16082	2.681196E-02
Ddah1	4290	0.130849293	14342	6.477065E-02
Ddah2	4291	0.143608461	15283	4.248598E-02
Ddb1	4292	0.075329088	10289	2.890792E-01
Ddb2	4293	0.184269877	17603	9.000331E-03
Ddc	4294	-0.010615485	4720	8.814046E-01
Ddhd1	4295	0.072244123	10011	3.093384E-01
Ddhd2	4296	0.233580127	19246	8.722583E-04
Ddi1	4297		#N/A	1.000000E+00
Ddi2	4298	0.145992243	15409	3.913488E-02
Ddit3	4299	0.070688751	9888	3.198974E-01

Spearman Rank correlation analysis performed between Prdm1 and all-expressed genes within the Meredith RNA-seq dataset. Robust Prdm1–associated genes were identified using a cut-off of $p < 0.0005$.

Table S1, Related to Supplemental Figure 3C. Prdm1 associated genes

Ddit4	4300	0.105572413	12398	1.367978E-01
Ddit4l	4301	0.102290763	12197	1.494926E-01
Ddn	4302	0.053267784	8685	4.537786E-01
Ddo	4303	0.059962683	9210	3.989800E-01
Ddost	4304	0.19080337	17872	6.802469E-03
Ddr1	4305	0.01695422	6169	8.116615E-01
Ddr2	4306	0.023227827	6707	7.440641E-01
Ddrgk1	4307	0.043315214	7826	5.425139E-01
Ddt	4308	0.169979171	16928	1.611332E-02
Ddx1	4309	0.093815761	11632	1.863830E-01
Ddx10	4310	0.040824404	7685	5.659916E-01
Ddx11	4311	0.041222397	7701	5.622080E-01
Ddx17	4312	0.177975867	17308	1.169073E-02
Ddx18	4313	0.153864477	15884	2.960739E-02
Ddx19a	4314	0.2012697	18300	4.265267E-03
Ddx19b	4315	0.036714767	7415	6.057533E-01
Ddx20	4316	0.017664477	6210	8.039279E-01
Ddx21	4317	0.177960066	17307	1.169829E-02
Ddx23	4318	0.220539952	18917	1.700549E-03
Ddx24	4319	0.148278255	15546	3.613326E-02
Ddx25	4320	0.027169996	6936.5	7.025327E-01
Ddx26b	4321	0.160982518	16389	2.277191E-02
Ddx27	4322	0.006204395	5604	9.305171E-01
Ddx28	4323	0.045441462	7962	5.228611E-01
Ddx31	4324	-0.014785404	4520	8.353868E-01
Ddx39	4325	0.116620639	13442	1.000640E-01
Ddx39b	4326	0.189183112	17809	7.297482E-03
Ddx3x	4327	0.142657394	15130	4.388861E-02
Ddx3y	4328	-0.014125284	4565	8.426387E-01
Ddx4	4329	-0.038406171	3065	5.892384E-01
Ddx41	4330	0.117291391	13479	9.811372E-02
Ddx42	4331	0.072867846	10055	3.051691E-01
Ddx43	4332	0.089699614	11323	2.065386E-01
Ddx46	4333	0.215585549	18780	2.170424E-03
Ddx47	4334	0.002772635	5459	9.689181E-01
Ddx49	4335	0.201191211	18298	4.280585E-03
Ddx5	4336	0.207387095	18511	3.212716E-03
Ddx50	4337	0.089891765	11336	2.055641E-01
Ddx51	4338	-0.034883881	3713	6.238582E-01
Ddx52	4339	-0.025339422	4090	7.217173E-01
Ddx54	4340	0.191636508	17901	6.559779E-03
Ddx55	4341	-0.038436189	2504	5.889471E-01
Ddx56	4342	0.256275334	19581	2.493505E-04

Spearman Rank correlation analysis performed between Prdm1 and all-expressed genes within the Meredith RNA-seq dataset. Robust Prdm1–associated genes were identified using a cut-off of $p < 0.0005$.

Table S1, Related to Supplemental Figure 3C. Prdm1 associated genes

Ddx58	4343	0.299229166	19905	1.675529E-05
Ddx59	4344	0.127217354	14130	7.262996E-02
Ddx6	4345	0.181703625	17470	1.002278E-02
Ddx60	4346	0.175473932	17188	1.294293E-02
Deaf1	4347	0.096913727	11830	1.721927E-01
Dear1	4348		#N/A	1.000000E+00
Deb1	4349	-0.013168482	4607	8.531732E-01
Decr1	4350	-0.011947511	4673	8.666539E-01
Decr2	4351	0.058143741	9098	4.134630E-01
Dedd	4352	0.163367222	16564	2.080908E-02
Dedd2	4353	-0.0575688	1442	4.181043E-01
Def6	4354	0.259820546	19622	2.028843E-04
Def8	4355	0.08312767	10842	2.418961E-01
Defa-ps1	4356		#N/A	1.000000E+00
Defa-ps10	4357		#N/A	1.000000E+00
Defa-ps11	4358		#N/A	1.000000E+00
Defa-ps12	4359	-0.038406171	3065	5.892384E-01
Defa-ps13	4360		#N/A	1.000000E+00
Defa-ps14	4361		#N/A	1.000000E+00
Defa-ps15	4362		#N/A	1.000000E+00
Defa-ps16	4363		#N/A	1.000000E+00
Defa-ps17	4364		#N/A	1.000000E+00
Defa-ps18	4365		#N/A	1.000000E+00
Defa-ps3	4366		#N/A	1.000000E+00
Defa-ps4	4367		#N/A	1.000000E+00
Defa-ps5	4368		#N/A	1.000000E+00
Defa-ps6	4369		#N/A	1.000000E+00
Defa-ps7	4370		#N/A	1.000000E+00
Defa-ps8	4371		#N/A	1.000000E+00
Defa-ps9	4372		#N/A	1.000000E+00
Defa-rs1	4373		#N/A	1.000000E+00
Defa-rs7	4374		#N/A	1.000000E+00
Defa17	4375		#N/A	1.000000E+00
Defa2	4376		#N/A	1.000000E+00
Defa20	4377		#N/A	1.000000E+00
Defa21	4378		#N/A	1.000000E+00
Defa22	4379		#N/A	1.000000E+00
Defa23	4380		#N/A	1.000000E+00
Defa24	4381	-0.019476618	4254	7.842841E-01
Defa25	4382		#N/A	1.000000E+00
Defa26	4383	0.053805701	8820	4.492258E-01
Defa3	4384		#N/A	1.000000E+00
Defa5	4385		#N/A	1.000000E+00

Spearman Rank correlation analysis performed between Prdm1 and all-expressed genes within the Meredith RNA-seq dataset. Robust Prdm1–associated genes were identified using a cut-off of $p < 0.0005$.

Table S1, Related to Supplemental Figure 3C. Prdm1 associated genes

Defb1	4386	-0.119274589	106	9.252327E-02
Defb10	4387		#N/A	1.000000E+00
Defb11	4388		#N/A	1.000000E+00
Defb12	4389		#N/A	1.000000E+00
Defb13	4390	-0.038406171	3065	5.892384E-01
Defb14	4391		#N/A	1.000000E+00
Defb15	4392		#N/A	1.000000E+00
Defb18	4393		#N/A	1.000000E+00
Defb19	4394	-0.010690624	4716	8.805714E-01
Defb2	4395		#N/A	1.000000E+00
Defb20	4396		#N/A	1.000000E+00
Defb21	4397		#N/A	1.000000E+00
Defb22	4398		#N/A	1.000000E+00
Defb23	4399		#N/A	1.000000E+00
Defb25	4400	-0.113990599	138	1.080085E-01
Defb26	4401		#N/A	1.000000E+00
Defb28	4402	-0.054450825	1984	4.437997E-01
Defb29	4403		#N/A	1.000000E+00
Defb3	4404	-0.093274543	385	1.889476E-01
Defb30	4405	0.014915162	6067	8.339629E-01
Defb33	4406		#N/A	1.000000E+00
Defb34	4407	0.126933796	14110.5	7.327484E-02
Defb35	4408	-0.054451513	1703	4.437940E-01
Defb36	4409		#N/A	1.000000E+00
Defb37	4410	0.090013747	11344.5	2.049472E-01
Defb38	4411		#N/A	1.000000E+00
Defb39	4412	0.182294195	17509.5	9.778799E-03
Defb4	4413		#N/A	1.000000E+00
Defb40	4414		#N/A	1.000000E+00
Defb41	4415		#N/A	1.000000E+00
Defb42	4416	0.137896377	14815	5.150617E-02
Defb43	4417	0.143605682	15224	4.249002E-02
Defb44-ps	4418		#N/A	1.000000E+00
Defb45	4419	-0.066855996	1239.5	3.469032E-01
Defb46	4420		#N/A	1.000000E+00
Defb47	4421		#N/A	1.000000E+00
Defb48	4422	-0.03262021	3808.5	6.465574E-01
Defb5	4423	0.052728522	8550.5	4.583686E-01
Defb50	4424	0.253098112	19540	2.992293E-04
Defb6	4425	-0.092672603	388	1.918301E-01
Defb7	4426		#N/A	1.000000E+00
Defb8	4427		#N/A	1.000000E+00
Defb9	4428		#N/A	1.000000E+00

Spearman Rank correlation analysis performed between Prdm1 and all-expressed genes within the Meredith RNA-seq dataset. Robust Prdm1–associated genes were identified using a cut-off of $p < 0.0005$.

Table S1, Related to Supplemental Figure 3C. Prdm1 associated genes

Degs1	4429	0.060416475	9233	3.954145E-01
Degs2	4430	0.092880705	11571	1.908300E-01
Dek	4431	0.159948053	16276	2.367182E-02
Dennd1a	4432	0.066939168	9640	3.463023E-01
Dennd1b	4433	0.225378279	19057	1.333193E-03
Dennd1c	4434	0.138967795	14884	4.970247E-02
Dennd2a	4435	0.220643305	18920	1.691821E-03
Dennd2c	4436	-0.03087642	3893.5	6.642703E-01
Dennd2d	4437	0.146321414	15437	3.869012E-02
Dennd3	4438	0.129873754	14294	6.680992E-02
Dennd4a	4439	0.165842254	16691	1.892839E-02
Dennd4b	4440	0.083488325	10876	2.398524E-01
Dennd4c	4441	0.099971126	12065	1.589907E-01
Dennd5a	4442	0.047379838	8110	5.052664E-01
Dennd5b	4443	0.227854651	19098	1.174732E-03
Dennd6a	4444	0.00275	5457	9.691717E-01
Dennd6b	4445	-0.064853076	1327.5	3.615723E-01
Denr	4446	0.036170745	7390	6.111085E-01
Depdc1a	4447	0.172172156	17032	1.477479E-02
Depdc1b	4448	0.074194755	10154.5	2.964228E-01
Depdc5	4449	0.063395121	9423	3.724893E-01
Depdc7	4450	-0.063816366	1346	3.693144E-01
Deptor	4451	0.22341635	18997	1.472376E-03
Dera	4452	-0.00940434	4769	8.948524E-01
Derl1	4453	0.210519956	18594	2.770241E-03
Derl2	4454	0.073016906	10064	3.041781E-01
Derl3	4455	0.201071451	18292	4.304055E-03
Des	4456	0.039985729	7640	5.740043E-01
Desi1	4457	0.239519289	19358	6.356178E-04
Desi2	4458	0.061703806	9316	3.854045E-01
Det1	4459	0.029327721	7036	6.801594E-01
Dexi	4460	-0.009344337	4772	8.955195E-01
Dffa	4461	0.001951535	5417	9.781200E-01
Dffb	4462	-0.073859495	905	2.986168E-01
Dfna5	4463	0.101257949	12134	1.536672E-01
Dfnb59	4464		#N/A	1.000000E+00
Dgat1	4465	0.126426503	14073	7.444013E-02
Dgat2	4466	0.146219734	15428	3.882705E-02
Dgat2l6	4467	-0.054451513	1703	4.437940E-01
Dgcr14	4468	0.20476766	18417	3.630694E-03
Dgcr2	4469	0.141706201	15060	4.533004E-02
Dgcr6	4470	0.003812335	5507	9.572723E-01
Dgcr8	4471	0.022269101	6648	7.542826E-01

Spearman Rank correlation analysis performed between Prdm1 and all-expressed genes within the Meredith RNA-seq dataset. Robust Prdm1–associated genes were identified using a cut-off of $p < 0.0005$.

Table S1, Related to Supplemental Figure 3C. Prdm1 associated genes

Dgka	4472	0.123128343	13851	8.238738E-02
Dgkb	4473	0.154185198	15904	2.926542E-02
Dgkd	4474	0.188637356	17785	7.471299E-03
Dgke	4475	0.029746151	7049	6.758522E-01
Dgkg	4476	0.040188652	7646	5.720607E-01
Dgkh	4477	0.015084982	6073	8.321002E-01
Dgki	4478	0.154460628	15951	2.897444E-02
Dgkk	4479	0.140791952	15009	4.675259E-02
Dgkq	4480	0.055947446	8981	4.313559E-01
Dgkz	4481	-0.117327814	129	9.800869E-02
Dguok	4482	0.047973976	8148	4.999360E-01
Dhcr24	4483	0.11674698	13452	9.969431E-02
Dhcr7	4484	0.038479095	7527	5.885310E-01
Dhdds	4485	0.08013248	10603	2.593385E-01
Dhdh	4486	0.03837825	7517	5.895093E-01
Dhfr	4487	0.18924285	17811	7.278676E-03
Dhh	4488	-0.089119381	427	2.095016E-01
Dhodh	4489	0.119195242	13601	9.274196E-02
Dhps	4490	0.266569229	19692	1.359074E-04
Dhrs1	4491	0.213685646	18707	2.379959E-03
Dhrs11	4492	-0.029826107	3930	6.750303E-01
Dhrs13	4493	-0.066858259	1029.5	3.468868E-01
Dhrs2	4494	-0.054450825	1984	4.437997E-01
Dhrs3	4495	-0.014534487	4539	8.381417E-01
Dhrs4	4496	-0.062254405	1377	3.811705E-01
Dhrs7	4497	0.111016713	12789	1.175796E-01
Dhrs7b	4498	0.105494775	12390	1.370883E-01
Dhrs7c	4499	0.157799267	16153.5	2.563935E-02
Dhrs9	4500		#N/A	1.000000E+00
Dhtkd1	4501	0.005940238	5593	9.334684E-01
Dhx15	4502	0.127835438	14176	7.124027E-02
Dhx16	4503	0.2368403	19303	7.338673E-04
Dhx29	4504	0.101701462	12163	1.518639E-01
Dhx30	4505	0.19730935	18135	5.102836E-03
Dhx32	4506	-0.07675331	848	2.800325E-01
Dhx33	4507	0.22733066	19088	1.206745E-03
Dhx34	4508	0.008009438	5680	9.103775E-01
Dhx35	4509	0.016814353	6163	8.131866E-01
Dhx36	4510	0.197743593	18152	5.004295E-03
Dhx37	4511	0.099472751	12029	1.610892E-01
Dhx38	4512	0.038271356	7512	5.905471E-01
Dhx40	4513	-0.041392978	2445	5.605900E-01
Dhx57	4514	0.001173126	5372	9.868463E-01

Spearman Rank correlation analysis performed between Prdm1 and all-expressed genes within the Meredith RNA-seq dataset. Robust Prdm1–associated genes were identified using a cut-off of $p < 0.0005$.

Table S1, Related to Supplemental Figure 3C. Prdm1 associated genes

Dhx58	4515	0.07901916	10532	2.660370E-01
Dhx8	4516	0.177429213	17286	1.195483E-02
Dhx9	4517	0.210141001	18576	2.820655E-03
Diablo	4518	0.156594531	16083	2.680270E-02
Diap1	4519	0.11289685	12914	1.114549E-01
Diap2	4520	0.120538797	13696	8.909411E-02
Diap3	4521	0.080633963	10643	2.563594E-01
Dicer1	4522	0.059990681	9212	3.987594E-01
Dido1	4523	0.25639336	19584	2.476560E-04
Diexf	4524	0.03586321	7371	6.141450E-01
Dimt1	4525	-0.018274917	4329	7.972962E-01
Dio1	4526	0.067371636	9665	3.431884E-01
Dio2	4527	0.154685737	15965	2.873847E-02
Dio3	4528		#N/A	1.000000E+00
Dip2a	4529	0.116945552	13460	9.911548E-02
Dip2b	4530	0.065788061	9576	3.546772E-01
Dip2c	4531	0.09404977	11646	1.852820E-01
Diras1	4532	0.113548679	13094	1.093908E-01
Diras2	4533	0.095242741	11712	1.797436E-01
Dirc2	4534	-0.039554616	2478	5.781437E-01
Dis3	4535	0.201035802	18290	4.311063E-03
Dis3l	4536	-0.015735975	4487	8.249686E-01
Dis3l2	4537	0.115224964	13337	1.042203E-01
Disc1	4538	0.17030269	16948	1.590943E-02
Disp1	4539	-0.072103925	927	3.102807E-01
Disp2	4540	-0.044269403	2360.5	5.336493E-01
Dixdc1	4541	-0.008297113	4810	9.071727E-01
Dkc1	4542	0.303658872	19915	1.236066E-05
Dkk1	4543	0.050013664	8298	4.818645E-01
Dkk2	4544	-0.057325901	1449	4.200743E-01
Dkk3	4545	-0.066855996	1239.5	3.469032E-01
Dkk4	4546	0.050747022	8358	4.754542E-01
Dkkl1	4547	-0.038406171	3065	5.892384E-01
Dlat	4548	0.23794777	19327	6.916675E-04
Dlc1	4549	0.098937212	11989	1.633673E-01
Dld	4550	0.160245804	16288	2.340971E-02
Dlec1	4551	0.074493743	10177	2.944753E-01
Dleu2	4552	0.233868984	19254	8.590904E-04
Dleu7	4553	0.080328904	10621	2.581688E-01
Dlg1	4554	0.07129369	9937	3.157632E-01
Dlg2	4555	0.05654075	9010	4.264789E-01
Dlg3	4556	0.135152088	14633	5.637534E-02
Dlg4	4557	-0.015893136	4473	8.232490E-01

Spearman Rank correlation analysis performed between Prdm1 and all-expressed genes within the Meredith RNA-seq dataset. Robust Prdm1–associated genes were identified using a cut-off of $p < 0.0005$.

Table S1, Related to Supplemental Figure 3C. Prdm1 associated genes

Dlg5	4558	0.273306036	19745	9.013555E-05
Dlgap1	4559	0.125154612	13978	7.742792E-02
Dlgap2	4560	0.072743475	10043	3.059975E-01
Dlgap3	4561	0.004457268	5536	9.500528E-01
Dlgap4	4562	0.189629297	17826	7.158062E-03
Dlgap5	4563	0.120001323	13663	9.053938E-02
Dlk1	4564	0.03213828	7189	6.514334E-01
Dlk2	4565		#N/A	1.000000E+00
Dll1	4566	0.018868923	6270	7.908570E-01
Dll3	4567	0.135251089	14644	5.619330E-02
Dll4	4568	0.002803894	5462	9.685678E-01
Dlst	4569	0.042270851	7757	5.522989E-01
Dlx1	4570	0.014301797	6015.5	8.406982E-01
Dlx1as	4571	0.099681749	12039	1.602066E-01
Dlx2	4572		#N/A	1.000000E+00
Dlx3	4573	0.102703024	12227	1.478505E-01
Dlx4	4574		#N/A	1.000000E+00
Dlx5	4575	-0.037492726	3646	5.981317E-01
Dlx6	4576	0.053805701	8820	4.492258E-01
Dlx6as1	4577	-0.089056705	429	2.098235E-01
Dlx6as2	4578	-0.10317255	224	1.459970E-01
Dmap1	4579	0.11763195	13502	9.713512E-02
Dmbt1	4580	-0.026912364	4033	7.052217E-01
Dmbx1	4581	0.02780156	6965	6.959565E-01
Dmc1	4582	0.016691396	6158	8.145279E-01
Dmd	4583	0.155402198	16000	2.799831E-02
Dmgdh	4584	-0.093622225	382	1.872971E-01
Dmkn	4585	-0.01401652	4567	8.438348E-01
Dmp1	4586	0.014301797	6015.5	8.406982E-01
Dmpk	4587	-0.069050313	959	3.312704E-01
Dmrt1	4588	0.107923698	12551	1.282219E-01
Dmrt2	4589	-0.025270207	4092	7.224462E-01
Dmrt3	4590	0.147827029	15530	3.670980E-02
Dmrta1	4591	-0.066856562	1139	3.468991E-01
Dmrta2	4592	0.21917948	18879	1.819337E-03
Dmrtb1	4593	0.071237894	9931.5	3.161430E-01
Dmrtc1a	4594		#N/A	1.000000E+00
Dmrtc1b	4595	-0.043103239	2406	5.444930E-01
Dmrtc1c1	4596		#N/A	1.000000E+00
Dmrtc1c2	4597		#N/A	1.000000E+00
Dmrtc2	4598		#N/A	1.000000E+00
Dmtf1	4599	0.101764319	12166	1.516096E-01
Dmwd	4600	0.150982277	15712	3.283647E-02

Spearman Rank correlation analysis performed between Prdm1 and all-expressed genes within the Meredith RNA-seq dataset. Robust Prdm1–associated genes were identified using a cut-off of $p < 0.0005$.

Table S1, Related to Supplemental Figure 3C. Prdm1 associated genes

Dmxl1	4601	0.185711039	17668	8.467633E-03
Dmxl2	4602	0.081939415	10741	2.487152E-01
Dna2	4603	0.36324731	20014	1.246175E-07
Dnaaf1	4604	-0.038406171	3065	5.892384E-01
Dnaaf2	4605	-0.058319877	1427	4.120472E-01
Dnaaf3	4606	0.124734454	13952	7.843595E-02
Dnahc1	4607	-0.103161098	248	1.460420E-01
Dnahc10	4608	0.015866138	6117	8.235443E-01
Dnahc11	4609	0.164294718	16610	2.008618E-02
Dnahc12	4610	0.139580572	14930	4.869472E-02
Dnahc14	4611	-0.038406171	3065	5.892384E-01
Dnahc17	4612	-0.010595526	4722	8.816260E-01
Dnahc2	4613	0.059879149	9202	3.996384E-01
Dnahc3	4614	0.031402782	7153	6.589034E-01
Dnahc5	4615	0.041450714	7709	5.600429E-01
Dnahc6	4616	0.068447507	9737	3.355192E-01
Dnahc7a	4617	-0.022600122	4154	7.507495E-01
Dnahc7b	4618	-0.023611387	4133	7.399884E-01
Dnahc7c	4619		#N/A	1.000000E+00
Dnahc8	4620	0.125347648	13994	7.696832E-02
Dnahc9	4621	-0.026296396	4053	7.116655E-01
Dnaic1	4622	0.058960827	9157	4.069192E-01
Dnaic2	4623	0.042791386	7790	5.474111E-01
Dnaja1	4624	0.045879491	7994	5.188579E-01
Dnaja1-ps	4625		#N/A	1.000000E+00
Dnaja2	4626	0.097504995	11874	1.695781E-01
Dnaja3	4627	0.145128419	15360	4.032255E-02
Dnaja4	4628	0.062174897	9335	3.817802E-01
Dnajb1	4629	-0.059170575	1411	4.052494E-01
Dnajb11	4630	0.22436686	19028	1.403365E-03
Dnajb12	4631	0.086286593	11071	2.244061E-01
Dnajb13	4632	0.020875719	6412.5	7.692098E-01
Dnajb14	4633	0.121279356	13744	8.713301E-02
Dnajb2	4634	0.127545848	14155	7.188866E-02
Dnajb3	4635		#N/A	1.000000E+00
Dnajb4	4636	0.10121821	12132	1.538295E-01
Dnajb5	4637	0.092460016	11547	1.928558E-01
Dnajb6	4638	0.172541573	17050	1.455914E-02
Dnajb7	4639		#N/A	1.000000E+00
Dnajb8	4640		#N/A	1.000000E+00
Dnajb9	4641	0.074964984	10247	2.914230E-01
Dnajc1	4642	0.073648151	10107	3.000053E-01
Dnajc10	4643	0.074692441	10223	2.931857E-01

Spearman Rank correlation analysis performed between Prdm1 and all-expressed genes within the Meredith RNA-seq dataset. Robust Prdm1–associated genes were identified using a cut-off of $p < 0.0005$.

Table S1, Related to Supplemental Figure 3C. Prdm1 associated genes

Dnajc11	4644	0.102411321	12206	1.490110E-01
Dnajc12	4645	0.010338745	5788	8.844745E-01
Dnajc13	4646	0.175884284	17211	1.272981E-02
Dnajc14	4647	0.087398731	11155	2.184674E-01
Dnajc15	4648	0.051051549	8390	4.728061E-01
Dnajc16	4649	-0.011986104	4668	8.662272E-01
Dnajc17	4650	0.135586527	14665	5.558010E-02
Dnajc18	4651	0.08816811	11212	2.144251E-01
Dnajc19	4652	0.266008976	19684	1.405611E-04
Dnajc19-ps	4653		#N/A	1.000000E+00
Dnajc2	4654	0.095881865	11761	1.768272E-01
Dnajc21	4655	0.081731929	10724	2.499195E-01
Dnajc22	4656	0.150605244	15688	3.328023E-02
Dnajc24	4657	0.137284944	14773	5.255965E-02
Dnajc25	4658		#N/A	1.000000E+00
Dnajc27	4659	0.00142176	5386	9.840588E-01
Dnajc28	4660	0.098723281	11974	1.642840E-01
Dnajc3	4661	0.206549823	18480	3.341326E-03
Dnajc30	4662	0.126797816	14093	7.358573E-02
Dnajc4	4663	0.161273478	16438	2.252418E-02
Dnajc5	4664	0.241090936	19380	5.837833E-04
Dnajc5b	4665	0.126833222	14098	7.350468E-02
Dnajc5g	4666		#N/A	1.000000E+00
Dnajc6	4667	0.141414038	15042	4.578065E-02
Dnajc7	4668	0.071228262	9930	3.162086E-01
Dnajc8	4669	0.210902585	18609	2.720168E-03
Dnajc9	4670	0.11809907	13535	9.580546E-02
Dnalc1	4671	0.1329668	14475	6.051934E-02
Dnalc4	4672	-0.049152225	2215	4.894535E-01
Dnali1	4673	-0.077393517	797	2.760287E-01
Dnase1	4674		#N/A	1.000000E+00
Dnase1l1	4675	-0.005905051	4883	9.338616E-01
Dnase1l2	4676		#N/A	1.000000E+00
Dnase1l3	4677	0.131704487	14385	6.302513E-02
Dnase2a	4678	0.089116482	11279	2.095165E-01
Dnase2b	4679	0.020875719	6412.5	7.692098E-01
Dnd1	4680	-0.077393517	797	2.760287E-01
Dner	4681	0.211852869	18647	2.599340E-03
Dnhd1	4682	-0.066856562	1139	3.468991E-01
Dnlz	4683	0.241931731	19395	5.576844E-04
Dnm1	4684	0.257096359	19593	2.377833E-04
Dnm1l	4685	-0.014167946	4562	8.421696E-01
Dnm2	4686	-0.092210016	399	1.940671E-01

Spearman Rank correlation analysis performed between Prdm1 and all-expressed genes within the Meredith RNA-seq dataset. Robust Prdm1–associated genes were identified using a cut-off of $p < 0.0005$.

Table S1, Related to Supplemental Figure 3C. Prdm1 associated genes

Dnm3	4687	0.145278487	15368	4.011407E-02
Dnm3os	4688	0.158765548	16214	2.473785E-02
Dnmbp	4689	0.023970155	6749	7.361826E-01
Dnmt1	4690	0.153783071	15878	2.969473E-02
Dnmt3a	4691	0.201463148	18308	4.227722E-03
Dnmt3b	4692	0.171749599	17006	1.502487E-02
Dnmt3l	4693	-0.024734409	4106	7.280970E-01
Dnmt3l-ps1	4694		#N/A	1.000000E+00
Dnpep	4695	0.033422375	7262	6.384748E-01
Dnph1	4696		#N/A	1.000000E+00
Dntt	4697	0.189678849	17831	7.142726E-03
Dnttip1	4698	0.239326371	19352	6.422652E-04
Dnttip2	4699	0.161763634	16459	2.211210E-02
Doc2a	4700		#N/A	1.000000E+00
Doc2b	4701	-5.84597E-05	5289	9.993445E-01
Doc2g	4702	0.269027073	19712	1.171431E-04
Dock1	4703	0.19123775	17886	6.674954E-03
Dock10	4704	0.114223103	13266	1.072868E-01
Dock11	4705	0.166399347	16744	1.852601E-02
Dock2	4706	0.264429668	19670	1.544935E-04
Dock3	4707	0.091218839	11458	1.989240E-01
Dock4	4708	0.053719793	8785	4.499512E-01
Dock5	4709	-0.00294235	5027	9.670166E-01
Dock6	4710	0.120319203	13680	8.968235E-02
Dock7	4711	0.092859333	11570	1.909325E-01
Dock8	4712	0.083162239	10846	2.416997E-01
Dock9	4713	0.026018043	6872	7.145843E-01
Dohh	4714	0.092576731	11555	1.922922E-01
Dok1	4715	0.088389889	11232	2.132699E-01
Dok2	4716	0.10415664	12303	1.421695E-01
Dok3	4717	0.136295665	14715	5.430196E-02
Dok4	4718	-0.054450825	1984	4.437997E-01
Dok5	4719	0.195466395	18062	5.540696E-03
Dok6	4720	0.102162023	12189	1.500083E-01
Dok7	4721		#N/A	1.000000E+00
Dolk	4722	0.007556839	5660	9.154225E-01
Dolpp1	4723	-0.037725919	3640	5.958556E-01
Dom3z	4724	0.265174335	19679	1.477708E-04
Donson	4725	0.024788944	6806	7.275212E-01
Dopey1	4726	0.192003518	17910	6.455344E-03
Dopey2	4727	-0.019282712	4261	7.863798E-01
Dos	4728	-0.01854844	4299	7.943294E-01
Dot1l	4729	0.091787531	11507	1.961267E-01

Spearman Rank correlation analysis performed between Prdm1 and all-expressed genes within the Meredith RNA-seq dataset. Robust Prdm1–associated genes were identified using a cut-off of $p < 0.0005$.

Table S1, Related to Supplemental Figure 3C. Prdm1 associated genes

Doxl2	4730	0.021316405	6469	7.644793E-01
Dpagt1	4731	-0.041043099	2450	5.639110E-01
Dpcd	4732	0.080005724	10592	2.600952E-01
Dpcr1	4733	-0.077394997	736.5	2.760195E-01
Dpep1	4734	-0.038406171	3065	5.892384E-01
Dpep2	4735	-0.047495195	2249	5.042291E-01
Dpep3	4736	0.053267784	8685	4.537786E-01
Dpf1	4737	-0.127736605	72	7.146102E-02
Dpf2	4738	0.103997572	12296	1.427830E-01
Dpf3	4739	0.061022271	9260	3.906847E-01
Dph1	4740	0.177183403	17274	1.207528E-02
Dph2	4741	0.120017604	13665	9.049532E-02
Dph3	4742	0.067874494	9696	3.395901E-01
Dph3b-ps	4743		#N/A	1.000000E+00
Dph5	4744	0.127730388	14170	7.147493E-02
Dpm1	4745	0.189248826	17814	7.276797E-03
Dpm2	4746	-0.080068772	640	2.597186E-01
Dpm3	4747	0.210679799	18598	2.749222E-03
Dpp10	4748	-0.005149795	4923	9.423050E-01
Dpp3	4749	0.120598934	13699	8.893355E-02
Dpp4	4750	0.183138073	17554	9.439286E-03
Dpp6	4751	0.150926447	15710	3.290186E-02
Dpp7	4752	0.115231038	13338	1.042019E-01
Dpp8	4753	0.022681482	6666	7.498819E-01
Dpp9	4754	0.173776711	17111	1.385802E-02
Dppa1	4755	0.078637604	10506	2.683597E-01
Dppa2	4756	0.108608001	12596	1.258056E-01
Dppa3	4757	-0.025305863	4091	7.220707E-01
Dppa4	4758	0.038758411	7558.5	5.858252E-01
Dppa5a	4759	-0.038406171	3065	5.892384E-01
Dpt	4760	0.313873225	19948	6.013445E-06
Dpy19l1	4761	0.122330608	13806	8.440871E-02
Dpy19l2	4762	0.11012449	12726.5	1.205764E-01
Dpy19l3	4763	0.005130712	5570	9.425184E-01
Dpy19l4	4764	0.16488002	16648	1.964126E-02
Dpy30	4765	0.095069425	11700	1.805406E-01
Dpyd	4766	0.131959778	14402	6.251161E-02
Dpys	4767	-0.071011266	939	3.176890E-01
Dpysl2	4768	0.127593335	14161	7.178202E-02
Dpysl3	4769	0.153076478	15847	3.046210E-02
Dpysl4	4770	-0.038406171	3065	5.892384E-01
Dpysl5	4771	0.085709587	11035	2.275319E-01
DQ267100	4772		#N/A	1.000000E+00

Spearman Rank correlation analysis performed between Prdm1 and all-expressed genes within the Meredith RNA-seq dataset. Robust Prdm1–associated genes were identified using a cut-off of $p < 0.0005$.

Table S1, Related to Supplemental Figure 3C. Prdm1 associated genes

DQ267101	4773		#N/A	1.000000E+00
DQ267102	4774		#N/A	1.000000E+00
Dqx1	4775	0.286914287	19849	3.803799E-05
Dr1	4776	0.190457661	17861	6.905501E-03
Dram1	4777	0.085836195	11045	2.268434E-01
Dram2	4778	0.161047886	16427	2.271605E-02
Drap1	4779	0.150044182	15653	3.395002E-02
Draxin	4780	0.149244134	15606	3.492486E-02
Drc1	4781	0.078778383	10516	2.675011E-01
Drd1a	4782	0.128244019	14198	7.033356E-02
Drd2	4783	0.111155589	12799	1.171184E-01
Drd3	4784	0.053267784	8685	4.537786E-01
Drd4	4785	0.050882593	8374	4.742743E-01
Drd5	4786	-0.077394997	736.5	2.760195E-01
Drg1	4787	0.024730663	6800	7.281366E-01
Drg2	4788	0.04110179	7697	5.633533E-01
Drosha	4789	0.026247911	6883	7.121737E-01
Drp2	4790	0.061577707	9306	3.863782E-01
Drr1	4791		#N/A	1.000000E+00
Dsc1	4792	-0.07511691	882	2.904435E-01
Dsc2	4793	0.200363408	18256	4.445188E-03
Dsc3	4794	0.176637097	17250	1.234683E-02
Dscam	4795	0.008911042	5725	9.003385E-01
Dscaml1	4796	0.159077068	16229	2.445309E-02
Dscc1	4797	0.083274386	10854	2.410633E-01
Dscr3	4798	-0.061480916	1382	3.871266E-01
Dse	4799	0.251521844	19524	3.272801E-04
Dsel	4800	-0.054450825	1984	4.437997E-01
Dsg1a	4801	-0.012253259	4663	8.632744E-01
Dsg1b	4802	0.012501906	5903	8.605278E-01
Dsg1c	4803	0.126933796	14110.5	7.327484E-02
Dsg2	4804	0.119201708	13602	9.272412E-02
Dsg3	4805	0.070871548	9903	3.186445E-01
Dsg4	4806	-0.043108183	2399.5	5.444468E-01
Dsn1	4807	0.158599529	16207	2.489077E-02
Dsp	4808	0.045027028	7936	5.266630E-01
Dspp	4809	-0.038406171	3065	5.892384E-01
Dst	4810	0.16040691	16360	2.326894E-02
Dstn	4811	0.082541698	10791	2.452423E-01
Dstyk	4812	0.189248085	17813	7.277030E-03
Dtd1	4813	0.150720757	15695	3.314374E-02
Dtd2	4814	-0.019712614	4247	7.817356E-01
Dtl	4815	0.097159073	11848	1.711042E-01

Spearman Rank correlation analysis performed between Prdm1 and all-expressed genes within the Meredith RNA-seq dataset. Robust Prdm1–associated genes were identified using a cut-off of $p < 0.0005$.

Table S1, Related to Supplemental Figure 3C. Prdm1 associated genes

Dtna	4816	0.040425397	7656	5.697971E-01
Dtnb	4817	0.004844781	5552	9.457168E-01
Dtnbp1	4818	0.23112093	19188	9.921178E-04
Dtwd1	4819	0.021481239	6551	7.627121E-01
Dtwd2	4820	0.272149736	19737	9.679217E-05
Dtx1	4821		#N/A	1.000000E+00
Dtx2	4822	0.057844315	9077	4.158764E-01
Dtx3	4823	0.142398917	15103	4.427645E-02
Dtx3l	4824	0.039832855	7628	5.754705E-01
Dtx4	4825	0.084858329	10977	2.321996E-01
Dtymk	4826	0.131596022	14376	6.324436E-02
Duox1	4827	-0.057251138	1451	4.206817E-01
Duox2	4828	-0.054451513	1703	4.437940E-01
Duoxa1	4829	-0.086748028	498.5	2.219283E-01
Duoxa2	4830		#N/A	1.000000E+00
Dupd1	4831	-0.02665934	4041	7.078662E-01
Dus1l	4832	0.085527581	11023	2.285243E-01
Dus2l	4833	0.07235124	10017	3.086197E-01
Dus3l	4834	0.096013564	11773	1.762306E-01
Dus4l	4835	0.080174568	10609	2.590875E-01
Dusp1	4836	0.105124636	12365	1.384797E-01
Dusp10	4837	0.07430416	10163	2.957092E-01
Dusp11	4838	0.043494572	7834	5.408421E-01
Dusp12	4839	0.090989303	11435	2.000612E-01
DUSP12	4840		#N/A	1.000000E+00
Dusp13	4841		#N/A	1.000000E+00
Dusp14	4842	0.042818192	7795	5.471599E-01
Dusp15	4843	-0.019711781	4248	7.817446E-01
Dusp16	4844	0.03772291	7473	5.958849E-01
Dusp18	4845	0.061268549	9279	3.887717E-01
Dusp19	4846		#N/A	1.000000E+00
Dusp2	4847	0.121557382	13759	8.640576E-02
Dusp21	4848		#N/A	1.000000E+00
Dusp22	4849	0.140411624	14982	4.735525E-02
Dusp23	4850	0.351932458	20005	3.216226E-07
Dusp26	4851		#N/A	1.000000E+00
Dusp27	4852	0.036956623	7426	6.033792E-01
Dusp28	4853	0.014580377	6048	8.376377E-01
Dusp3	4854	0.042890217	7803	5.464855E-01
Dusp4	4855	0.197162361	18127	5.136583E-03
Dusp5	4856	0.201556228	18312	4.209763E-03
Dusp6	4857	0.150569097	15685	3.332304E-02
Dusp7	4858	0.035899589	7373	6.137855E-01

Spearman Rank correlation analysis performed between Prdm1 and all-expressed genes within the Meredith RNA-seq dataset. Robust Prdm1–associated genes were identified using a cut-off of $p < 0.0005$.

Dusp8	4859	0.289519631	19863	3.208115E-05
Dusp9	4860		#N/A	1.000000E+00
Dut	4861	0.142093555	15086	4.473834E-02
Duxbl1	4862	0.167496789	16797	1.775504E-02
Duxbl2	4863		#N/A	1.000000E+00
Duxbl3	4864		#N/A	1.000000E+00
Dvl1	4865	0.125613699	14012	7.633849E-02
Dvl2	4866	0.047379728	8109	5.052673E-01
Dvl3	4867	0.092183311	11529	1.941968E-01
DXBay18	4868		#N/A	1.000000E+00
Dydc1	4869	0.272744585	19743	9.331223E-05
Dydc2	4870	-0.077394997	736.5	2.760195E-01
Dym	4871	0.054978301	8925	4.393909E-01
Dynap	4872		#N/A	1.000000E+00
Dync1h1	4873	0.139012991	14888	4.962755E-02
Dync1i1	4874	-0.151692998	27	3.201362E-02
Dync1i2	4875	0.044837989	7925	5.284018E-01
Dync1li1	4876	0.11478357	13311	1.055627E-01
Dync1li2	4877	0.244113718	19428	4.949010E-04
Dync2h1	4878	0.169222486	16889	1.659912E-02
Dync2li1	4879	-0.017361098	4400	8.072289E-01
Dynll1	4880	0.051446741	8422	4.693816E-01
Dynll2	4881	0.237037987	19310	7.261647E-04
Dynlrb1	4882	0.195958488	18086	5.420605E-03
Dynlrb2	4883		#N/A	1.000000E+00
Dynlt1-ps1	4884	-0.054451513	1703	4.437940E-01
Dynlt1a	4885	0.152789767	15822	3.077826E-02
Dynlt1b	4886		#N/A	1.000000E+00
Dynlt1c	4887	0.113548679	13094	1.093908E-01
Dynlt1f	4888	0.136327395	14717	5.424535E-02
Dynlt3	4889	0.09898287	11993	1.631721E-01
Dyrk1a	4890	0.05362368	8780	4.507635E-01
Dyrk1b	4891	-0.038406171	3065	5.892384E-01
Dyrk2	4892	-0.003703707	4996.5	9.584887E-01
Dyrk3	4893	0.050174861	8310	4.804515E-01
Dyrk4	4894	0.109492511	12666.5	1.227347E-01
Dysf	4895	0.114332169	13275	1.069496E-01
Dytn	4896	-0.049207144	2214	4.889678E-01
Dyx1c1	4897	0.119733492	13642	9.126653E-02
Dzank1	4898	0.114166725	13263	1.074615E-01
Dzip1	4899	0.034533769	7313	6.273466E-01
Dzip1l	4900	0.166369602	16739	1.854730E-02
Dzip3	4901	0.01098436	5823	8.773154E-01

Spearman Rank correlation analysis performed between Prdm1 and all-expressed genes within the Meredith RNA-seq dataset. Robust Prdm1–associated genes were identified using a cut-off of $p < 0.0005$.

Table S1, Related to Supplemental Figure 3C. Prdm1 associated genes

E030002O03Ri	4902		#N/A	1.000000E+00
E030010N08Ri	4903	0.143605682	15224	4.249002E-02
E030011O05Ri	4904		#N/A	1.000000E+00
E030013I19Rik	4905	-0.077394997	736.5	2.760195E-01
E030018B13Ril	4906		#N/A	1.000000E+00
E030019B06Ril	4907	0.21549506	18774.5	2.180011E-03
E030024N20Ri	4908	0.102076072	12186	1.503533E-01
E030025P04Rik	4909		#N/A	1.000000E+00
E030030I06Rik	4910		#N/A	1.000000E+00
E030037K01Ril	4911		#N/A	1.000000E+00
E030042O20Ri	4912	0.053267784	8685	4.537786E-01
E130003G02Ril	4913		#N/A	1.000000E+00
E130006D01Ril	4914	0.008796978	5716	9.016077E-01
E130012A19Ril	4915	-0.017607112	4386	8.045518E-01
E130018N17Ri	4916		#N/A	1.000000E+00
E130018O15Ri	4917		#N/A	1.000000E+00
E130102H24Ril	4918	0.008212935	5687	9.081104E-01
E130111B04Ril	4919		#N/A	1.000000E+00
E130114P18Rik	4920	0.087349554	11154	2.187276E-01
E130116L18Rik	4921		#N/A	1.000000E+00
E130120K24Rik	4922	-0.054451513	1703	4.437940E-01
E130201H02Ril	4923	-0.038406171	3065	5.892384E-01
E130208F15Rik	4924	-0.013116419	4619	8.537472E-01
E130215H24Ril	4925		#N/A	1.000000E+00
E130218I03Rik	4926	0.053267784	8685	4.537786E-01
E130304I02Rik	4927		#N/A	1.000000E+00
E130307A14Ril	4928	0.051727943	8450	4.669531E-01
E130308A19Ril	4929	-0.087798702	435	2.163593E-01
E130309D02Ril	4930	0.184299856	17606	8.988954E-03
E130309D14Ril	4931	-0.054451513	1703	4.437940E-01
E130309F12Rik	4932	0.111090927	12795	1.173330E-01
E130311K13Rik	4933	0.15925638	16239	2.429046E-02
E130317F20Rik	4934	0.135444262	14660	5.583949E-02
E230001N04Ri	4935	-0.077393517	797	2.760287E-01
E230008N13Ri	4936	-0.084599775	579.5	2.336305E-01
E230015B07Ril	4937	0.021317126	6513.5	7.644715E-01
E230015J15Rik	4938	0.236193938	19292	7.595808E-04
E230016K23Rik	4939	-0.026146235	4061	7.132396E-01
E230016M11Ri	4940	0.202637545	18344	4.006109E-03
E230019M04Ri	4941	0.122363806	13807	8.432381E-02
E230025N22Ri	4942		#N/A	1.000000E+00
E2f1	4943	0.092494121	11550	1.926910E-01
E2f2	4944	0.049585392	8260	4.856295E-01

Spearman Rank correlation analysis performed between Prdm1 and all-expressed genes within the Meredith RNA-seq dataset. Robust Prdm1–associated genes were identified using a cut-off of $p < 0.0005$.

Table S1, Related to Supplemental Figure 3C. Prdm1 associated genes

E2f3	4945	-0.109383787	186	1.231090E-01
E2f4	4946	0.139573993	14929	4.870544E-02
E2f5	4947	0.069130005	9790	3.307113E-01
E2f6	4948	-0.086748028	498.5	2.219283E-01
E2f7	4949	0.015973581	6123	8.223691E-01
E2f8	4950	0.295048075	19885	2.222693E-05
E330009J07Rik	4951	0.132879842	14468	6.068929E-02
E330010L02Rik	4952	-0.001224019	5151	9.862757E-01
E330012B07Ril	4953	-0.038406171	3065	5.892384E-01
E330013P04Ril	4954	0.104465642	12323	1.409836E-01
E330014E10Rik	4955		#N/A	1.000000E+00
E330017A01Ril	4956		#N/A	1.000000E+00
E330017L17Rik	4957	-0.038406171	3065	5.892384E-01
E330020D12Ril	4958	-0.012832382	4633	8.568800E-01
E330021D16Ril	4959	0.143605682	15224	4.249002E-02
E330034G19Ril	4960		#N/A	1.000000E+00
E430018J23Rik	4961	-0.103161098	248	1.460420E-01
E430024P14Rik	4962	0.044087692	7873	5.353318E-01
E430025E21Rik	4963	-0.042280919	2429	5.522042E-01
E4f1	4964	-0.06230382	1376	3.807919E-01
E530001F21Rik	4965		#N/A	1.000000E+00
E530001K10Rik	4966	-0.038406171	3065	5.892384E-01
Eaf1	4967	0.066754836	9634	3.476349E-01
Eaf2	4968	0.210402465	18589	2.785783E-03
Eapp	4969	0.126035587	14048	7.534831E-02
Ear-ps10	4970	-0.077394997	736.5	2.760195E-01
Ear-ps12	4971		#N/A	1.000000E+00
Ear-ps2	4972		#N/A	1.000000E+00
Ear-ps3	4973		#N/A	1.000000E+00
Ear-ps4	4974		#N/A	1.000000E+00
Ear-ps5	4975		#N/A	1.000000E+00
Ear-ps7	4976		#N/A	1.000000E+00
Ear-ps8	4977		#N/A	1.000000E+00
Ear-ps9	4978		#N/A	1.000000E+00
Ear1	4979	0.135761191	14676	5.526300E-02
Ear10	4980		#N/A	1.000000E+00
Ear11	4981	0.004686517	5543	9.474875E-01
Ear14	4982		#N/A	1.000000E+00
Ear2	4983	0.04250271	7775	5.501191E-01
Ear5	4984	0.030597817	7103	6.671179E-01
Ear6	4985	0.018113026	6227	7.990536E-01
Ears2	4986	0.021160338	6445	7.661535E-01
Ebag9	4987	0.278103365	19779	6.683883E-05

Spearman Rank correlation analysis performed between Prdm1 and all-expressed genes within the Meredith RNA-seq dataset. Robust Prdm1–associated genes were identified using a cut-off of $p < 0.0005$.

Table S1, Related to Supplemental Figure 3C. Prdm1 associated genes

Ebf1	4988	0.011748807	5864	8.688515E-01
Ebf2	4989	0.224586185	19034	1.387868E-03
Ebf3	4990	0.178672823	17333	1.136147E-02
Ebf4	4991	0.092993822	11586	1.902879E-01
Ebi3	4992	0.154046568	15891	2.941282E-02
Ebna1bp2	4993	0.163979743	16595	2.032920E-02
Ebp	4994	0.050797253	8361	4.750169E-01
Ebpl	4995	0.110180269	12731	1.203873E-01
Ecd	4996	0.241555264	19385	5.692339E-04
Ece1	4997	0.136368278	14721	5.417247E-02
Ece2	4998	0.071690436	9969	3.130707E-01
Ecel1	4999	0.106474601	12458	1.334567E-01
Ech1	5000	0.083505704	10878	2.397543E-01
Echdc1	5001	0.066155519	9602	3.519901E-01
Echdc2	5002	0.197637703	18145	5.028166E-03
Echdc3	5003	0.029746912	7051	6.758444E-01
Echs1	5004	0.208088472	18526	3.108444E-03
Eci1	5005	0.193090999	17952	6.154557E-03
Eci2	5006	-0.022403512	4164	7.528474E-01
Eci3	5007	-0.054450825	1984	4.437997E-01
Ecm1	5008	0.013164107	5940.5	8.532214E-01
Ecm2	5009	0.274166616	19749	8.546255E-05
Ecscr	5010	-0.082039473	618	2.481359E-01
Ecsit	5011	-0.05909562	1413	4.058456E-01
Ect2	5012	0.099873114	12053	1.594017E-01
Ect2l	5013	-0.055982321	1487	4.310683E-01
Eda	5014	0.034185023	7301	6.308296E-01
Eda2r	5015	-0.042473514	2422	5.503934E-01
Edar	5016	0.048719338	8198	4.932910E-01
Edaradd	5017	0.098238382	11934	1.663762E-01
Edc3	5018	-0.032235472	3835	6.504488E-01
Edc4	5019	0.065214569	9547	3.588967E-01
Eddm3b	5020		#N/A	1.000000E+00
Edem1	5021	0.18691723	17715	8.043495E-03
Edem2	5022	0.240637643	19372	5.983176E-04
Edem3	5023	0.122440804	13815	8.412715E-02
Edf1	5024	0.154986299	15978	2.842596E-02
Edil3	5025	-0.001421642	5122	9.840601E-01
Edn1	5026	0.170983934	16973	1.548745E-02
Edn2	5027	-0.086746694	532	2.219355E-01
Edn3	5028	0.143605682	15224	4.249002E-02
Ednra	5029	-0.077393023	832.5	2.760318E-01
Ednrb	5030	0.002851298	5463	9.680367E-01

Spearman Rank correlation analysis performed between Prdm1 and all-expressed genes within the Meredith RNA-seq dataset. Robust Prdm1–associated genes were identified using a cut-off of $p < 0.0005$.

Table S1, Related to Supplemental Figure 3C. Prdm1 associated genes

Eea1	5031	0.198935827	18196	4.742469E-03
Eed	5032	-0.065749775	1311	3.549579E-01
Eef1a1	5033	0.197874797	18160	4.974860E-03
Eef1a2	5034	-0.066856562	1139	3.468991E-01
Eef1b2	5035	0.193307724	17959	6.096131E-03
Eef1d	5036	0.080160077	10607	2.591739E-01
Eef1e1	5037	-0.062991739	1362	3.755452E-01
Eef1g	5038	0.133751809	14535	5.900277E-02
Eef2	5039	0.234841625	19267	8.160848E-04
Eef2k	5040	0.182062737	17485	9.873788E-03
Eefsec	5041	0.064747682	9525	3.623548E-01
Eepd1	5042	0.107401303	12519	1.300905E-01
Efcab1	5043	-0.044084755	2367.5	5.353590E-01
Efcab10	5044		#N/A	1.000000E+00
Efcab11	5045	0.167197059	16782	1.796278E-02
Efcab12	5046	-0.077397959	691.5	2.760011E-01
Efcab14	5047	0.009656364	5759	8.920515E-01
Efcab2	5048	0.199413393	18223	4.641090E-03
Efcab3	5049	-0.086748028	498.5	2.219283E-01
Efcab4a	5050	-0.095267847	349	1.796284E-01
Efcab4b	5051	-0.038406171	3065	5.892384E-01
Efcab5	5052	0.038259902	7508	5.906584E-01
Efcab6	5053	0.283316976	19820	4.799100E-05
Efcab7	5054	0.140120004	14964	4.782173E-02
Efcab8	5055	-0.027295868	4023	7.012203E-01
Efcab9	5056	0.143605682	15224	4.249002E-02
Efcc1	5057	0.071748509	9973	3.126779E-01
Efemp1	5058	0.109492511	12666.5	1.227347E-01
Efemp2	5059	-0.038406171	3065	5.892384E-01
Efhb	5060	0.023143321	6700	7.449631E-01
Efhc1	5061	0.071237894	9931.5	3.161430E-01
Efhc2	5062	-0.002837208	5039	9.681946E-01
Efhd1	5063	0.135602549	14667	5.555095E-02
Efhd2	5064	0.158672571	16210	2.482340E-02
Efna1	5065	0.022942289	6691	7.471029E-01
Efna2	5066	0.064405489	9500	3.649024E-01
Efna3	5067	-0.086748028	498.5	2.219283E-01
Efna4	5068	0.098670824	11970	1.645094E-01
Efna5	5069	0.032185577	7192	6.509542E-01
Efnb1	5070	-0.025671002	4081	7.182290E-01
Efnb2	5071	0.091326898	11474	1.983903E-01
Efnb3	5072	-0.110564915	172	1.190898E-01
Efr3a	5073	0.24001353	19364	6.188764E-04

Spearman Rank correlation analysis performed between Prdm1 and all-expressed genes within the Meredith RNA-seq dataset. Robust Prdm1–associated genes were identified using a cut-off of $p < 0.0005$.

Table S1, Related to Supplemental Figure 3C. Prdm1 associated genes

Efr3b	5074	0.081992079	10745	2.484102E-01
Efs	5075	0.061213006	9272	3.892026E-01
Eftud1	5076	0.172318342	17039	1.468912E-02
Eftud2	5077	0.016002593	6125	8.220518E-01
Egf	5078	0.046581843	8052	5.124721E-01
Egfem1	5079	0.055837408	8975	4.322639E-01
Egfl6	5080	-0.095269893	339	1.796190E-01
Egfl7	5081		#N/A	1.000000E+00
Egfl8	5082	0.000307048	5309	9.965571E-01
Egflam	5083	0.016380545	6141	8.179214E-01
Egfr	5084	0.086310252	11072	2.242786E-01
Egln1	5085	0.03857175	7531	5.876328E-01
Egln2	5086	0.257223325	19595	2.360398E-04
Egln3	5087	0.012963628	5935	8.554321E-01
Egr1	5088	0.208379521	18535	3.066081E-03
Egr2	5089	0.236785637	19301	7.360105E-04
Egr3	5090		#N/A	1.000000E+00
Egr4	5091		#N/A	1.000000E+00
Ehbp1	5092	0.249890418	19506	3.588696E-04
Ehbp1l1	5093	0.147108677	15484	3.764369E-02
Ehd1	5094	0.114953532	13319	1.050442E-01
Ehd2	5095	-0.103163388	239	1.460330E-01
Ehd3	5096	-0.015109708	4505	8.318291E-01
Ehd4	5097	0.239132505	19351	6.490098E-04
Ehf	5098	0.134850329	14609	5.693323E-02
Ehhadh	5099	0.03105623	7134	6.624350E-01
Ehmt1	5100	0.146168313	15424	3.889645E-02
Ehmt2	5101	0.14070546	15002	4.688908E-02
Ei24	5102	0.114329819	13274	1.069568E-01
Eid1	5103	0.126892076	14101	7.337011E-02
Eid2	5104	0.152557246	15795	3.103672E-02
Eid2b	5105	-0.073223148	919	3.028106E-01
Eif1	5106	0.146210868	15427	3.883901E-02
Eif1a	5107	0.166526759	16749	1.843503E-02
Eif1ad	5108	-0.123741044	93	8.086150E-02
Eif1ax	5109	0.108996566	12623	1.244493E-01
Eif1b	5110	0.120359959	13684	8.957294E-02
Eif2a	5111	0.116411748	13427	1.006776E-01
Eif2ak1	5112	0.023939778	6744	7.365046E-01
Eif2ak2	5113	0.202102822	18332	4.105682E-03
Eif2ak3	5114	0.120718835	13706	8.861413E-02
Eif2ak4	5115	0.104406515	12318	1.412100E-01
Eif2b1	5116	0.094366796	11666	1.837981E-01

Spearman Rank correlation analysis performed between Prdm1 and all-expressed genes within the Meredith RNA-seq dataset. Robust Prdm1–associated genes were identified using a cut-off of $p < 0.0005$.

Table S1, Related to Supplemental Figure 3C. Prdm1 associated genes

Eif2b2	5117	-0.016826798	4420	8.130509E-01
Eif2b3	5118	0.048993954	8220	4.908547E-01
Eif2b4	5119	0.171899493	17013	1.493574E-02
Eif2b5	5120	0.019842605	6327	7.803328E-01
Eif2c5	5121	0.109492511	12666.5	1.227347E-01
Eif2d	5122	0.146847142	15469	3.798864E-02
Eif2s1	5123	0.085711268	11036	2.275228E-01
Eif2s2	5124	0.165686979	16688	1.904188E-02
Eif2s3x	5125	0.173188363	17089	1.418822E-02
Eif2s3y	5126	-0.054451513	1703	4.437940E-01
Eif3a	5127	0.14091335	15016	4.656157E-02
Eif3b	5128	0.052619102	8513	4.593030E-01
Eif3c	5129	0.114188342	13264	1.073945E-01
Eif3d	5130	0.047220605	8100	5.067000E-01
Eif3e	5131	0.17686096	17260	1.223491E-02
Eif3f	5132	0.113387984	12948	1.098968E-01
Eif3g	5133	0.142961811	15139	4.343550E-02
Eif3h	5134	0.155824763	16030	2.756947E-02
Eif3i	5135	0.129682676	14281	6.721543E-02
Eif3j1	5136	0.062245212	9340	3.812410E-01
Eif3j2	5137	0.090664356	11399	2.016792E-01
Eif3k	5138	0.195907432	18080	5.432955E-03
Eif3l	5139	0.08283127	10817	2.435847E-01
Eif3m	5140	0.24479285	19436	4.767371E-04
Eif3s6-ps1	5141		#N/A	1.000000E+00
Eif3s6-ps2	5142		#N/A	1.000000E+00
Eif4a-ps4	5143		#N/A	1.000000E+00
Eif4a1	5144	0.210540555	18595	2.767524E-03
Eif4a2	5145	0.17599794	17221	1.267132E-02
Eif4a3	5146	0.241136426	19382	5.823428E-04
Eif4b	5147	0.193160528	17955	6.135759E-03
Eif4e	5148	0.185773634	17670	8.445144E-03
Eif4e1b	5149	0.207506955	18514	3.194676E-03
Eif4e2	5150	0.120504384	13693	8.918609E-02
Eif4e3	5151	0.161177128	16433	2.260596E-02
Eif4ebp1	5152	-0.005415472	4903	9.393341E-01
Eif4ebp2	5153	-0.014440079	4547	8.391787E-01
Eif4ebp3	5154		#N/A	1.000000E+00
Eif4enif1	5155	0.18880386	17790	7.417882E-03
Eif4g1	5156	0.247761758	19478	4.043469E-04
Eif4g2	5157	0.158927742	16220	2.458924E-02
Eif4g3	5158	0.258687318	19609	2.167776E-04
Eif4h	5159	0.200427295	18258	4.432285E-03

Spearman Rank correlation analysis performed between Prdm1 and all-expressed genes within the Meredith RNA-seq dataset. Robust Prdm1–associated genes were identified using a cut-off of $p < 0.0005$.

Table S1, Related to Supplemental Figure 3C. Prdm1 associated genes

Eif5	5160	0.149895727	15645	3.412914E-02
Eif5a	5161	0.136053856	14706	5.473503E-02
Eif5a2	5162	0.101772214	12167	1.515777E-01
Eif5al3-ps	5163		#N/A	1.000000E+00
Eif5b	5164	0.120017346	13664	9.049602E-02
Eif6	5165	0.197473965	18140	5.065278E-03
Elac1	5166	-0.082711661	609	2.442685E-01
Elac2	5167	0.087759947	11182	2.165629E-01
Elane	5168		#N/A	1.000000E+00
Elavl1	5169	0.177768777	17302	1.179017E-02
Elavl2	5170	0.127655415	14166	7.164278E-02
Elavl3	5171	0.112637	12897	1.122862E-01
Elavl4	5172	0.13232978	14434	6.177344E-02
Elf1	5173	0.173273816	17092	1.413983E-02
Elf2	5174	0.103278754	12256	1.455802E-01
Elf3	5175	0.055537925	8956	4.347408E-01
Elf4	5176	0.08143808	10697	2.516319E-01
Elf5	5177	0.103488543	12268	1.447596E-01
Elfn1	5178	-0.054451513	1703	4.437940E-01
Elfn2	5179	-0.038406171	3065	5.892384E-01
Elk1	5180	0.154090026	15898	2.936655E-02
Elk3	5181	0.074946222	10237	2.915441E-01
Elk4	5182	0.18432488	17609	8.979468E-03
Ell	5183	-0.070040243	949	3.243683E-01
Ell2	5184	0.181887077	17480	9.946422E-03
Ell3	5185	-0.077393517	797	2.760287E-01
Elmo1	5186	0.132671627	14457	6.109781E-02
Elmo2	5187	0.257033329	19592	2.386533E-04
Elmo3	5188	-0.103043329	260	1.465054E-01
Elmod1	5189	0.009713475	5762	8.914170E-01
Elmod2	5190	0.05129999	8413	4.706516E-01
Elmod3	5191	-0.140257424	39	4.760144E-02
Elmsan1	5192	0.065720427	9574	3.551732E-01
Eln	5193	0.092260427	11534.5	1.938224E-01
Elof1	5194	0.120767656	13708	8.848433E-02
Elovl1	5195	0.07338248	10085	3.017569E-01
Elovl2	5196	-0.057396204	1448	4.195035E-01
Elovl3	5197		#N/A	1.000000E+00
Elovl4	5198	0.05165509	8443	4.675816E-01
Elovl5	5199	0.033552685	7271	6.371658E-01
Elovl6	5200	0.238927152	19348	6.562253E-04
Elovl7	5201	0.011408473	5848	8.726179E-01
Elp2	5202	0.065598483	9567	3.560686E-01

Spearman Rank correlation analysis performed between Prdm1 and all-expressed genes within the Meredith RNA-seq dataset. Robust Prdm1–associated genes were identified using a cut-off of $p < 0.0005$.

Table S1, Related to Supplemental Figure 3C. Prdm1 associated genes

Elp3	5203	0.039241568	7590	5.811583E-01
Elp4	5204	0.094040072	11644	1.853276E-01
Elp5	5205	0.133031964	14477	6.039224E-02
Elp6	5206	0.082793528	10815	2.438003E-01
Eltd1	5207	0.165024821	16659	1.953252E-02
Emb	5208	0.079298448	10550	2.643456E-01
Emc1	5209		#N/A	1.000000E+00
Emc10	5210	0.201115816	18293	4.295347E-03
Emc2	5211	0.205407499	18434	3.524299E-03
Emc3	5212	0.036238497	7393	6.104405E-01
Emc4	5213	0.162146741	16476	2.179456E-02
Emc6	5214	0.10518626	12371	1.382473E-01
Emc7	5215	0.138869926	14874	4.986501E-02
Emc8	5216	0.049629791	8264	4.852384E-01
Emc9	5217	-0.053677122	2127	4.503117E-01
Emcn	5218	-0.083080312	604	2.421653E-01
Emd	5219	0.197602734	18144	5.036071E-03
Eme1	5220	0.185212763	17646	8.648559E-03
Eme2	5221	0.061726007	9318	3.852333E-01
Emg1	5222	0.029026454	7016	6.832670E-01
Emid1	5223	0.154135904	15900	2.931776E-02
Emilin1	5224	0.157799267	16153.5	2.563935E-02
Emilin2	5225	0.069781313	9831	3.261646E-01
Emilin3	5226		#N/A	1.000000E+00
Eml1	5227	0.10468691	12334	1.401391E-01
Eml2	5228	0.13731808	14776	5.250210E-02
Eml3	5229	-0.091232926	407	1.988544E-01
Eml4	5230	0.201539918	18310	4.212905E-03
Eml5	5231	0.061232988	9275	3.890476E-01
Eml6	5232	0.215244303	18754	2.206776E-03
Emp1	5233	0.021757451	6575	7.597536E-01
Emp2	5234	-0.008496452	4800	9.049529E-01
Emp3	5235	0.31762973	19961	4.582430E-06
Emr1	5236	-0.062385801	1374	3.801643E-01
Emr4	5237	0.114535596	13297	1.063228E-01
Emx1	5238		#N/A	1.000000E+00
Emx2	5239	0.150712695	15693	3.315325E-02
Emx2os	5240	-0.000380503	5266	9.957334E-01
En1	5241	-0.043104355	2402.5	5.444826E-01
En2	5242	0.216985709	18819	2.026903E-03
Enah	5243	0.106334301	12446	1.339722E-01
Enam	5244	-0.066858259	1029.5	3.468868E-01
Enc1	5245	-0.003662293	4999	9.589525E-01

Spearman Rank correlation analysis performed between Prdm1 and all-expressed genes within the Meredith RNA-seq dataset. Robust Prdm1–associated genes were identified using a cut-off of $p < 0.0005$.

Table S1, Related to Supplemental Figure 3C. Prdm1 associated genes

Endod1	5246	0.179085271	17361	1.117049E-02
Endog	5247	-0.050163324	2195	4.805525E-01
Endou	5248	0.260935546	19636	1.900284E-04
Endov	5249	0.014561849	6046	8.378412E-01
Eng	5250	-0.07559277	873	2.873897E-01
Engase	5251	0.109488806	12652	1.227474E-01
Enho	5252	0.095698452	11748	1.776605E-01
Enkd1	5253	-0.026648858	4042	7.079758E-01
Enkur	5254	0.034504526	7312	6.276384E-01
Eno1	5255	0.089415753	11302	2.079844E-01
Eno2	5256	-0.115873668	132	1.022718E-01
Eno3	5257	-0.056777103	1464	4.245450E-01
Eno4	5258	0.055158631	8935	4.378894E-01
Enoph1	5259	0.163021067	16548	2.108459E-02
Enox1	5260	0.125248455	13986	7.720421E-02
Enox2	5261	0.056975013	9037	4.229296E-01
Enpep	5262	-0.124960991	78	7.789114E-02
Enpp1	5263	0.02354724	6722	7.406695E-01
Enpp2	5264	0.102937952	12241	1.469209E-01
Enpp3	5265	0.04497701	7931	5.271228E-01
Enpp4	5266	0.138240003	14839	5.092186E-02
Enpp5	5267	0.072424366	10024	3.081297E-01
Enpp6	5268	-0.001881841	5081	9.789012E-01
Enpp7	5269		#N/A	1.000000E+00
Ensa	5270	0.11533654	13350	1.038831E-01
ENSMUSG0000(	5271		#N/A	1.000000E+00
ENSMUSG0000(	5272	-0.038406171	3065	5.892384E-01
ENSMUSG0000(	5273		#N/A	1.000000E+00
ENSMUSG0000(	5274		#N/A	1.000000E+00
ENSMUSG0000(	5275		#N/A	1.000000E+00
Enthd1	5276	0.10456771	12330	1.405936E-01
Enthd2	5277	-0.00568008	4892.5	9.363760E-01
Entpd1	5278	0.180519897	17430	1.052807E-02
Entpd2	5279		#N/A	1.000000E+00
Entpd3	5280	0.086451709	11101	2.235172E-01
Entpd4	5281		#N/A	1.000000E+00
Entpd5	5282	0.103319151	12259	1.454219E-01
Entpd6	5283	0.037512577	7459	5.979378E-01
Entpd7	5284	0.153021112	15841	3.052294E-02
Entpd8	5285	0.088187776	11217	2.143225E-01
Eny2	5286	0.196348451	18100	5.327099E-03
Eogt	5287	-0.014693911	4531	8.363911E-01
Eomes	5288	0.132160369	14421	6.211052E-02

Spearman Rank correlation analysis performed between Prdm1 and all-expressed genes within the Meredith RNA-seq dataset. Robust Prdm1–associated genes were identified using a cut-off of $p < 0.0005$.

Table S1, Related to Supplemental Figure 3C. Prdm1 associated genes

Ep300	5289	0.194275441	18008	5.841256E-03
Ep400	5290	0.206034942	18461	3.422708E-03
Epas1	5291	-0.009933774	4748	8.889700E-01
Epb4.1	5292	0.038222263	7491	5.910240E-01
Epb4.1l1	5293	0.082946902	10826	2.429250E-01
Epb4.1l2	5294	0.135217986	14642	5.625412E-02
Epb4.1l3	5295	0.050984359	8382	4.733897E-01
Epb4.1l4a	5296	0.05368997	8783	4.502031E-01
Epb4.1l4b	5297	0.139345342	14913	4.907953E-02
Epb4.1l5	5298	0.168396041	16844	1.714425E-02
Epb4.2	5299	-0.038605994	2498	5.873010E-01
Epb4.9	5300	0.041744214	7727	5.572656E-01
Epc1	5301	0.2333941	19235	8.808366E-04
Epc2	5302	0.096158858	11786	1.755742E-01
Epcam	5303	0.077312074	10431	2.765359E-01
Epdr1	5304	0.075420155	10295	2.884949E-01
Epg5	5305	0.132078097	14416	6.227477E-02
Epgn	5306	-0.001989505	5076	9.776944E-01
Epha1	5307	0.04617463	8025.5	5.161695E-01
Epha10	5308	-0.032450477	3820	6.482730E-01
Epha2	5309	-0.012609522	4640	8.593396E-01
Epha3	5310	0.06247867	9354	3.794541E-01
Epha4	5311	0.128243992	14197	7.033362E-02
Epha5	5312	-0.045780602	2296	5.197603E-01
Epha6	5313	0.168410304	16845	1.713471E-02
Epha7	5314	0.185365619	17653	8.592695E-03
Epha8	5315	-0.054450825	1984	4.437997E-01
Ephb1	5316	0.162792559	16515	2.126818E-02
Ephb2	5317	0.033469217	7264	6.380041E-01
Ephb3	5318	0.068729137	9753	3.335298E-01
Ephb4	5319	0.030696312	7108	6.661106E-01
Ephb6	5320	-0.012831654	4634	8.568880E-01
Ephx1	5321	0.145888448	15404	3.927601E-02
Ephx2	5322	0.175935981	17218	1.270318E-02
Ephx3	5323	0.044427593	7893	5.321868E-01
Ephx4	5324	-0.038406171	3065	5.892384E-01
Epm2a	5325	0.038027626	7484	5.929165E-01
Epm2aip1	5326	0.180638098	17435	1.047662E-02
Epn1	5327	0.201122614	18294	4.294014E-03
Epn2	5328	0.068383803	9732	3.359702E-01
Epn3	5329	0.096497938	11807	1.740492E-01
Epo	5330	0.116909779	13458	9.921956E-02
Epor	5331	-0.031503296	3869	6.578806E-01

Spearman Rank correlation analysis performed between Prdm1 and all-expressed genes within the Meredith RNA-seq dataset. Robust Prdm1–associated genes were identified using a cut-off of $p < 0.0005$.

Table S1, Related to Supplemental Figure 3C. Prdm1 associated genes

Eppin	5332		#N/A	1.000000E+00
Eprs	5333	0.100370761	12084	1.573228E-01
Eps15	5334	0.082902306	10822	2.431793E-01
Eps15l1	5335	0.018539215	6252	7.944294E-01
Eps8	5336	0.140950284	15018	4.650359E-02
Eps8l1	5337	-0.084599775	579.5	2.336305E-01
Eps8l2	5338	0.067595597	9679	3.415828E-01
Eps8l3	5339	0.280130118	19799	5.880855E-05
Epsti1	5340	0.014736569	6056	8.359228E-01
Ept1	5341	0.028867879	7008	6.849049E-01
Epx	5342	-0.130642413	62	6.519880E-02
Epyc	5343	-0.043139297	2397	5.441561E-01
Eqtn	5344	0.014301797	6015.5	8.406982E-01
Eral1	5345	-0.00382017	4989	9.571846E-01
Erap1	5346	0.047137302	8086	5.074508E-01
Eras	5347		#N/A	1.000000E+00
Erbb2	5348	0.021877775	6584	7.584659E-01
Erbb2ip	5349	0.113634008	13234	1.091228E-01
Erbb3	5350	0.111028415	12790	1.175407E-01
Erbb4	5351	0.218596935	18860	1.872476E-03
Erc1	5352	-0.037143797	3656	6.015448E-01
Erc2	5353	0.162748291	16509	2.130391E-02
Ercc1	5354	0.213710456	18708	2.377108E-03
Ercc2	5355	0.202633022	18343	4.006942E-03
Ercc3	5356	0.089335606	11294	2.083939E-01
Ercc4	5357	0.213312487	18694	2.423212E-03
Ercc5	5358	0.139287244	14908	4.917497E-02
Ercc6	5359	0.179051188	17357	1.118617E-02
Ercc6l	5360	0.01389846	5985	8.451336E-01
Ercc6l2	5361	0.098600079	11963	1.648137E-01
Ercc8	5362	-0.056174365	1477	4.294868E-01
Erdr1	5363	0.130992485	14352	6.447565E-02
Ereg	5364	-0.066855996	1239.5	3.469032E-01
Erf	5365	0.11952563	13627	9.183408E-02
Erg	5366	0.019221589	6292	7.870407E-01
Ergic1	5367	0.285129081	19835	4.270542E-05
Ergic2	5368	0.160706702	16375	2.300892E-02
Ergic3	5369	0.149687285	15629	3.438200E-02
Erh	5370	0.162847522	16535	2.122390E-02
Eri1	5371	0.243243389	19420	5.191150E-04
Eri2	5372	0.123873979	13896	8.053346E-02
Eri3	5373	0.030730922	7112	6.657568E-01
Erich1	5374	0.059264689	9172	4.045014E-01

Spearman Rank correlation analysis performed between Prdm1 and all-expressed genes within the Meredith RNA-seq dataset. Robust Prdm1–associated genes were identified using a cut-off of $p < 0.0005$.

Erich2	5375	0.130435587	14320	6.562915E-02
Erlec1	5376	0.102922373	12240	1.469824E-01
Erlin1	5377	0.115145633	13333	1.044605E-01
Erlin2	5378	0.118092599	13534	9.582378E-02
Ermap	5379	0.082113823	10754	2.477060E-01
Ermn	5380	0.023067505	6695.5	7.457699E-01
Ermp1	5381	-0.023642377	4132	7.396594E-01
Ern1	5382	0.161826644	16460	2.205960E-02
Ern2	5383	0.041672289	7724	5.579456E-01
Ero1l	5384	0.053886544	8857	4.485438E-01
Ero1lb	5385	0.168123521	16831	1.732739E-02
Erp27	5386	0.043302928	7823.5	5.426285E-01
Erp29	5387	0.162584837	16501	2.143628E-02
Erp44	5388	0.170632657	16962	1.570380E-02
Errfi1	5389	-0.096130856	308	1.757005E-01
Erv3	5390	0.08698414	11126	2.206680E-01
Esam	5391	0.014039659	5990.5	8.435803E-01
Esco1	5392	0.125494124	14004	7.662105E-02
Esco2	5393	0.192304984	17923	6.370674E-03
Esd	5394	0.170103154	16935	1.603492E-02
Esf1	5395	0.017109984	6179	8.099639E-01
Esm1	5396	0.109492511	12666.5	1.227347E-01
Espl1	5397	0.219148571	18878	1.822121E-03
Espn	5398	0.113757381	13243	1.087363E-01
Espnl	5399	0.111946412	12848	1.145190E-01
Esr1	5400	0.187693548	17750	7.780602E-03
Esr2	5401	0.149916028	15647	3.410460E-02
Esrp1	5402	0.107745754	12537	1.288560E-01
Esrp2	5403	-0.005928302	4879	9.336018E-01
Esrra	5404	0.010854016	5818	8.787600E-01
Esrrb	5405	0.080147333	10606	2.592499E-01
Esrrg	5406	0.032667535	7217	6.460794E-01
Esx1	5407	-0.016730797	4444.5	8.140981E-01
Esyt1	5408	0.122057111	13791	8.511080E-02
Esyt2	5409	0.075902012	10341	2.854167E-01
Esyt3	5410	-0.045078076	2331	5.261939E-01
Etaa1	5411	0.025399405	6844	7.210859E-01
Etd	5412	-0.038406171	3065	5.892384E-01
Etf1	5413	0.15049266	15681	3.341372E-02
Etfa	5414	0.043154604	7814	5.440131E-01
Etfb	5415	0.183687303	17581	9.223962E-03
Etfdh	5416	0.141212558	15031	4.609357E-02
Ethe1	5417	-0.075832819	869	2.858574E-01

Spearman Rank correlation analysis performed between Prdm1 and all-expressed genes within the Meredith RNA-seq dataset. Robust Prdm1–associated genes were identified using a cut-off of $p < 0.0005$.

Etl4	5418	0.112686049	12899	1.121289E-01
Etnk1	5419	0.185800357	17671	8.435559E-03
Etnk2	5420	-0.066855996	1239.5	3.469032E-01
Etohd2	5421	0.093023511	11589	1.901458E-01
Etohi1	5422	-0.046818519	2264	5.103294E-01
Etos1	5423		#N/A	1.000000E+00
Ets1	5424	0.105659781	12400	1.364715E-01
Ets2	5425	0.18036276	17423	1.059680E-02
Etv1	5426	0.016927692	6167	8.119507E-01
Etv2	5427	-0.018447724	4312.5	7.954215E-01
Etv3	5428	-0.005350225	4905	9.400636E-01
Etv4	5429		#N/A	1.000000E+00
Etv5	5430	0.104055394	12299	1.425597E-01
Etv6	5431	0.049084799	8222	4.900502E-01
EU599041	5432		#N/A	1.000000E+00
Eva1a	5433	-0.006764406	4859	9.242635E-01
Eva1b	5434	0.081085405	10671.5	2.536978E-01
Eva1c	5435	0.093442992	11610	1.881466E-01
Evc	5436	0.179605946	17387	1.093345E-02
Evc2	5437	0.094177202	11654	1.846845E-01
Evi2a	5438		#N/A	1.000000E+00
Evi2b	5439		#N/A	1.000000E+00
Evi5	5440	0.151248332	15731	3.252636E-02
Evi5l	5441	0.002889027	5466	9.676140E-01
Evl	5442	0.098071529	11919	1.671006E-01
Evpl	5443	0.113548679	13094	1.093908E-01
Evx1	5444		#N/A	1.000000E+00
Evx2	5445	-0.038406171	3065	5.892384E-01
Ewsr1	5446	0.196796519	18113	5.221449E-03
Exd1	5447	0.23596152	19287	7.690280E-04
Exd2	5448	0.136914935	14759	5.320580E-02
Exo1	5449	-0.055110399	1514	4.382907E-01
Exo5	5450	0.0603758	9229	3.957333E-01
Exoc1	5451	0.052499402	8500	4.603265E-01
Exoc2	5452	0.043244316	7818	5.431754E-01
Exoc3	5453	0.137283825	14772	5.256159E-02
Exoc3l	5454	0.154156363	15902	2.929603E-02
Exoc3l2	5455		#N/A	1.000000E+00
Exoc3l4	5456	0.155435546	16003	2.796426E-02
Exoc4	5457	0.125511525	14005	7.657988E-02
Exoc5	5458	0.123432763	13873	8.162638E-02
Exoc6	5459	0.043699758	7845	5.389327E-01
Exoc6b	5460	0.036617309	7409	6.067111E-01

Spearman Rank correlation analysis performed between Prdm1 and all-expressed genes within the Meredith RNA-seq dataset. Robust Prdm1–associated genes were identified using a cut-off of $p < 0.0005$.

Table S1, Related to Supplemental Figure 3C. Prdm1 associated genes

Exoc7	5461	0.049241173	8239	4.886669E-01
Exoc8	5462	0.161915249	16464	2.198596E-02
Exog	5463	0.135968379	14696	5.488880E-02
Exosc1	5464	0.08341382	10859	2.402736E-01
Exosc10	5465	0.111032285	12791	1.175278E-01
Exosc2	5466	0.246649224	19465	4.301878E-04
Exosc3	5467	0.242998394	19415	5.261262E-04
Exosc4	5468	0.026947819	6929	7.048514E-01
Exosc5	5469	0.162350821	16488	2.162702E-02
Exosc7	5470	0.183512413	17571	9.292051E-03
Exosc8	5471	0.062888664	9385	3.763285E-01
Exosc9	5472	0.026801641	6918	7.063785E-01
Exph5	5473	-0.013653719	4581	8.478273E-01
Ext1	5474	0.158098961	16179	2.535678E-02
Ext2	5475	-0.028748729	3971	6.861365E-01
Extl1	5476	0.189038636	17804	7.343142E-03
Extl2	5477	0.041750872	7728	5.572026E-01
Extl3	5478	0.126168417	14055	7.503872E-02
Eya1	5479	0.127367577	14144	7.229020E-02
Eya2	5480	0.089119027	11281	2.095034E-01
Eya3	5481	0.068896046	9766	3.323544E-01
Eya4	5482	0.095694989	11747	1.776763E-01
Ezh1	5483	0.072845333	10052	3.053189E-01
Ezh2	5484	0.004211183	5527	9.528071E-01
Ezr	5485	0.209339306	18560	2.930058E-03
F10	5486	0.297991561	19893	1.822543E-05
F11	5487		#N/A	1.000000E+00
F11r	5488	0.042451774	7770	5.505976E-01
F12	5489	-0.077397959	691.5	2.760011E-01
F13a1	5490	0.114514974	13294	1.063862E-01
F13b	5491		#N/A	1.000000E+00
F2	5492	-0.025872915	4071	7.161077E-01
F2r	5493	-0.02444093	4120	7.311985E-01
F2rl1	5494	0.128668775	14223	6.940093E-02
F2rl2	5495	0.058422446	9118	4.112240E-01
F2rl3	5496		#N/A	1.000000E+00
F3	5497		#N/A	1.000000E+00
F5	5498	0.030436322	7093	6.687707E-01
F630028O10Ril	5499		#N/A	1.000000E+00
F630206G17Ril	5500	-0.086755146	452	2.218903E-01
F7	5501		#N/A	1.000000E+00
F730016J06Rik	5502	-0.038406171	3065	5.892384E-01
F730035M05Ri	5503	0.161379506	16445	2.243448E-02

Spearman Rank correlation analysis performed between Prdm1 and all-expressed genes within the Meredith RNA-seq dataset. Robust Prdm1–associated genes were identified using a cut-off of $p < 0.0005$.

Table S1, Related to Supplemental Figure 3C. Prdm1 associated genes

F730035P03Rik	5504		#N/A	1.000000E+00
F730043M19Ri	5505	-0.009146045	4777	8.977244E-01
F8	5506	0.110303104	12738	1.199718E-01
F830001A07Ril	5507		#N/A	1.000000E+00
F830016B08Rik	5508	0.119517931	13626	9.185515E-02
F830045P16Rik	5509	0.201275365	18301	4.264163E-03
F830104G03Ril	5510		#N/A	1.000000E+00
F830208F22Rik	5511	0.152789767	15822	3.077826E-02
F8a	5512	-0.028056866	3994	6.933045E-01
F9	5513	0.149307379	15610	3.484694E-02
F930015N05Ril	5514	0.095485525	11737	1.786316E-01
F930017D23Ril	5515	-0.038406171	3065	5.892384E-01
Fa2h	5516	-0.077394997	736.5	2.760195E-01
Faah	5517	0.047353455	8106	5.055037E-01
Fabp1	5518	-0.022810177	4153	7.485102E-01
Fabp12	5519	0.130625651	14329	6.523359E-02
Fabp2	5520	-0.05506313	1518.5	4.386842E-01
Fabp3	5521	-0.077393517	797	2.760287E-01
Fabp3-ps1	5522	-0.054451513	1703	4.437940E-01
Fabp4	5523	-0.01443196	4548	8.392679E-01
Fabp5	5524	0.085747534	11041	2.273254E-01
Fabp5l2	5525	0.074574898	10201.5	2.939481E-01
Fabp6	5526	-0.041606406	2443	5.585688E-01
Fabp7	5527	0.015425617	6092	8.283668E-01
Fabp9	5528	0.010378602	5790	8.840323E-01
Fadd	5529	0.26509619	19678	1.484632E-04
Fads1	5530	0.199262284	18213	4.672955E-03
Fads2	5531		#N/A	1.000000E+00
Fads3	5532	0.053805701	8820	4.492258E-01
Fads6	5533	-0.060217297	1394	3.969771E-01
Faf1	5534	0.088999137	11271	2.101194E-01
Faf2	5535	0.244741538	19435	4.780876E-04
Fah	5536	0.109723712	12695	1.219417E-01
Fahd1	5537	-0.044956576	2336	5.273107E-01
Fahd2a	5538	-0.026662732	4040	7.078307E-01
Faim	5539	0.237765544	19321	6.984537E-04
Faim2	5540		#N/A	1.000000E+00
Faim3	5541	0.019630193	6310.5	7.826254E-01
Fam101a	5542	-0.054451513	1703	4.437940E-01
Fam101b	5543	0.02306759	6697	7.457690E-01
Fam102a	5544	0.03251256	7203	6.476453E-01
Fam102b	5545	0.062830068	9382	3.767742E-01
Fam103a1	5546	0.185127403	17642	8.679896E-03

Spearman Rank correlation analysis performed between Prdm1 and all-expressed genes within the Meredith RNA-seq dataset. Robust Prdm1–associated genes were identified using a cut-off of $p < 0.0005$.

Table S1, Related to Supplemental Figure 3C. Prdm1 associated genes

Fam104a	5547	0.122198654	13796	8.474687E-02
Fam105a	5548	0.094408179	11669	1.836051E-01
Fam105b	5549	0.024880457	6811	7.265553E-01
Fam107a	5550	-0.038406171	3065	5.892384E-01
Fam107b	5551	0.126007751	14046	7.541332E-02
Fam109a	5552	0.102990161	12243	1.467149E-01
Fam109b	5553	0.074440157	10174	2.948237E-01
Fam110a	5554	-0.07725558	840	2.768881E-01
Fam110b	5555	0.080069072	10596	2.597169E-01
Fam110c	5556	0.229057459	19118.5	1.104172E-03
Fam111a	5557	0.162800764	16517	2.126157E-02
Fam114a1	5558	-0.021084173	4203	7.669710E-01
Fam114a2	5559	0.146379503	15441	3.861208E-02
Fam115a	5560	0.092685532	11561	1.917679E-01
Fam115c	5561	0.021969493	6590	7.574848E-01
Fam115e	5562		#N/A	1.000000E+00
Fam117a	5563	0.187398415	17737	7.879632E-03
Fam117b	5564	0.00349039	5497	9.608776E-01
Fam118a	5565	0.106657109	12473	1.327885E-01
Fam118b	5566	0.164696033	16636	1.978019E-02
Fam120a	5567	0.071610018	9958	3.136153E-01
Fam120b	5568	0.08905661	11275	2.098240E-01
Fam120c	5569	0.169623495	16910	1.634010E-02
Fam122a	5570		#N/A	1.000000E+00
Fam122b	5571	-4.34584E-06	5290	9.999513E-01
Fam122c	5572	-0.011940535	4674	8.667310E-01
Fam124a	5573	-0.043099124	2412	5.445314E-01
Fam124b	5574	0.037241736	7446	6.005859E-01
Fam126a	5575	0.157008316	16107	2.639813E-02
Fam126b	5576	0.323352677	19972	3.007420E-06
Fam129a	5577	0.018537362	6251	7.944495E-01
Fam129b	5578	-0.039388577	2486	5.797417E-01
Fam129c	5579	-0.01735111	4401	8.073377E-01
Fam131a	5580	0.092906132	11575	1.907080E-01
Fam131b	5581	0.021316405	6469	7.644793E-01
Fam131c	5582	-0.001224019	5151	9.862757E-01
Fam132a	5583	-0.031959651	3847	6.532445E-01
Fam132b	5584	-0.054450825	1984	4.437997E-01
Fam133b	5585	0.248904968	19495	3.792983E-04
Fam134a	5586	0.039180333	7587	5.817488E-01
Fam134b	5587	0.047084283	8083	5.079290E-01
Fam134c	5588	-0.047683093	2243	5.025420E-01
Fam135a	5589	0.110742069	12774	1.184958E-01

Spearman Rank correlation analysis performed between Prdm1 and all-expressed genes within the Meredith RNA-seq dataset. Robust Prdm1–associated genes were identified using a cut-off of $p < 0.0005$.

Table S1, Related to Supplemental Figure 3C. Prdm1 associated genes

Fam135b	5590	0.03271953	7219	6.455544E-01
Fam136a	5591	0.194288896	18009	5.837781E-03
Fam136b-ps	5592	-0.038406171	3065	5.892384E-01
Fam13a	5593	0.052386961	8494	4.612891E-01
Fam13b	5594	0.194684559	18033	5.736401E-03
Fam13c	5595	0.026796026	6916.5	7.064372E-01
Fam149a	5596	0.010074338	5774	8.874092E-01
Fam149b	5597	-0.058126587	1432	4.136010E-01
Fam150a	5598	0.024216053	6769	7.335779E-01
Fam150b	5599	0.113548679	13094	1.093908E-01
Fam151a	5600		#N/A	1.000000E+00
Fam151b	5601	0.110281477	12736	1.200448E-01
Fam154a	5602	0.09836549	11941	1.658258E-01
Fam154b	5603	0.024787432	6805	7.275372E-01
Fam155a	5604	-0.02300802	4147	7.464030E-01
Fam159a	5605	-0.038406171	3065	5.892384E-01
Fam159b	5606		#N/A	1.000000E+00
Fam160a1	5607		#N/A	1.000000E+00
Fam160a2	5608	-0.005110205	4929	9.427478E-01
Fam160b1	5609	0.041044812	7694	5.638947E-01
Fam160b2	5610	0.231538567	19201	9.707508E-04
Fam161a	5611	0.057569271	9063	4.181005E-01
Fam161b	5612	0.095097935	11704	1.804093E-01
Fam162a	5613	0.086145259	11062	2.251689E-01
Fam162b	5614	0.070933959	9909	3.182174E-01
Fam163a	5615	0.085210477	11003	2.302605E-01
Fam163b	5616	-0.054451513	1703	4.437940E-01
Fam166a	5617		#N/A	1.000000E+00
Fam166b	5618		#N/A	1.000000E+00
Fam167a	5619	-0.134339517	51	5.788799E-02
Fam167b	5620		#N/A	1.000000E+00
Fam168a	5621	0.200160774	18246	4.486338E-03
Fam168b	5622	0.256376874	19583	2.478921E-04
Fam169a	5623	0.192221358	17920	6.394061E-03
Fam169b	5624	0.089844014	11333	2.058060E-01
Fam170a	5625		#N/A	1.000000E+00
Fam170b	5626	-0.038406171	3065	5.892384E-01
Fam171a1	5627	0.090150352	11361	2.042579E-01
Fam171a2	5628		#N/A	1.000000E+00
Fam171b	5629	-0.043956732	2374	5.365461E-01
Fam172a	5630	0.076262901	10365	2.831258E-01
Fam173a	5631	-0.053771653	2125	4.495132E-01
Fam173b	5632	0.170467704	16954	1.580631E-02

Spearman Rank correlation analysis performed between Prdm1 and all-expressed genes within the Meredith RNA-seq dataset. Robust Prdm1–associated genes were identified using a cut-off of $p < 0.0005$.

Table S1, Related to Supplemental Figure 3C. Prdm1 associated genes

Fam174a	5633	0.115967105	13394	1.019936E-01
Fam174b	5634	0.157743185	16138	2.569253E-02
Fam175a	5635	0.131709259	14386	6.301550E-02
Fam175b	5636	-0.06293879	1364	3.759474E-01
Fam177a	5637	-0.054451513	1703	4.437940E-01
Fam178a	5638	0.112287085	12877	1.134132E-01
Fam178b	5639	0.158458979	16197	2.502087E-02
Fam179a	5640	0.01952482	6303	7.837634E-01
Fam179b	5641	0.17538557	17182	1.298922E-02
Fam180a	5642		#N/A	1.000000E+00
Fam181a	5643		#N/A	1.000000E+00
Fam181b	5644	-0.038406171	3065	5.892384E-01
Fam183b	5645	0.084000076	10921	2.369734E-01
Fam184a	5646	0.043627162	7840	5.396078E-01
Fam184b	5647	0.133887297	14544	5.874422E-02
Fam185a	5648	0.140890676	15013	4.659720E-02
Fam186a	5649	0.038758411	7558.5	5.858252E-01
Fam186b	5650	0.053267784	8685	4.537786E-01
Fam187a	5651		#N/A	1.000000E+00
Fam187b	5652	-0.141333168	36	4.590603E-02
Fam188a	5653	0.159738504	16262	2.385779E-02
Fam188b	5654	0.112494909	12888	1.127428E-01
Fam189a1	5655	0.067229724	9656	3.442082E-01
Fam189a2	5656	0.10194313	12180	1.508880E-01
Fam189b	5657	0.119001128	13589	9.327869E-02
Fam192a	5658	0.107176018	12507	1.309027E-01
Fam193a	5659	0.003118758	5481	9.650403E-01
Fam193b	5660	0.094177239	11655	1.846843E-01
Fam194a	5661		#N/A	1.000000E+00
Fam195a	5662	0.002330858	5434	9.738686E-01
Fam195b	5663	0.004747394	5545	9.468063E-01
Fam196a	5664	0.176057181	17226	1.264094E-02
Fam196b	5665	0.101149406	12126	1.541109E-01
Fam198a	5666	0.093070123	11593	1.899229E-01
Fam198b	5667	0.093456405	11611	1.880829E-01
Fam199x	5668	0.054401684	8888	4.442117E-01
Fam19a1	5669	0.07773764	10456	2.738927E-01
Fam19a2	5670	0.060671886	9243	3.934162E-01
Fam19a3	5671	-0.054451513	1703	4.437940E-01
Fam19a4	5672	-0.073748888	909	2.993430E-01
Fam19a5	5673	0.115191646	13335	1.043211E-01
Fam203a	5674	0.153840847	15882	2.963272E-02
FAM205A	5675		#N/A	1.000000E+00

Spearman Rank correlation analysis performed between Prdm1 and all-expressed genes within the Meredith RNA-seq dataset. Robust Prdm1–associated genes were identified using a cut-off of $p < 0.0005$.

Table S1, Related to Supplemental Figure 3C. Prdm1 associated genes

Fam207a	5676	0.059412756	9180	4.033264E-01
Fam209	5677	-0.018103565	4364	7.991563E-01
Fam20a	5678	0.017980762	6222	8.004901E-01
Fam20b	5679	0.212494056	18677	2.520586E-03
Fam20c	5680	0.221075562	18935	1.655759E-03
Fam21	5681	0.077044236	10415	2.782083E-01
Fam210a	5682	0.083627979	10889	2.390644E-01
Fam210b	5683	0.179852559	17395	1.082273E-02
Fam211a	5684	-0.00174887	5083.5	9.803918E-01
Fam211b	5685	0.175816077	17208	1.276501E-02
Fam212a	5686	0.03256067	7209	6.471590E-01
Fam212b	5687	0.015967557	6122	8.224350E-01
Fam213a	5688	-0.012324481	4659	8.624875E-01
Fam213b	5689	0.145697743	15391	3.953643E-02
Fam214a	5690	0.210741494	18602	2.741149E-03
Fam214b	5691	0.041988167	7738	5.549622E-01
Fam216a	5692	-0.066572953	1289	3.489530E-01
Fam216b	5693	-0.038406171	3065	5.892384E-01
Fam217a	5694	0.081730759	10723	2.499263E-01
Fam217b	5695	0.013226902	5947	8.525292E-01
Fam219a	5696	-0.017395	4398	8.068599E-01
Fam219b	5697	0.157290995	16119	2.612477E-02
Fam220-ps	5698		#N/A	1.000000E+00
Fam220a	5699	-0.008960556	4785	8.997876E-01
Fam221a	5700	-0.023777265	4129	7.382280E-01
Fam221b	5701	0.113548679	13094	1.093908E-01
Fam222a	5702	-0.027897274	4000	6.949618E-01
Fam222b	5703	0.073729169	10111	2.994725E-01
Fam227a	5704	0.314304484	19952	5.829811E-06
Fam227b	5705	0.305578432	19923	1.081737E-05
Fam228a	5706		#N/A	1.000000E+00
Fam228b	5707	0.167715801	16807.5	1.760456E-02
Fam229a	5708	-0.005348205	4909.5	9.400862E-01
Fam229b	5709	0.012682557	5913	8.585334E-01
Fam24a	5710		#N/A	1.000000E+00
Fam25c	5711	-0.028387685	3984	6.898736E-01
Fam26d	5712	0.131777656	14391	6.287760E-02
Fam26e	5713	-0.038406171	3065	5.892384E-01
Fam26f	5714	-0.03437936	3729	6.288877E-01
Fam32a	5715	0.083518669	10880	2.396811E-01
Fam35a	5716	0.128242399	14196	7.033713E-02
Fam3a	5717	0.062841221	9383	3.766894E-01
Fam3b	5718	0.091821677	11510	1.959596E-01

Spearman Rank correlation analysis performed between Prdm1 and all-expressed genes within the Meredith RNA-seq dataset. Robust Prdm1–associated genes were identified using a cut-off of $p < 0.0005$.

Table S1, Related to Supplemental Figure 3C. Prdm1 associated genes

Fam3c	5719	0.068732079	9754	3.335091E-01
Fam43a	5720	0.156792699	16097	2.660829E-02
Fam43b	5721	-0.038406171	3065	5.892384E-01
Fam45a	5722	-0.008322112	4809	9.068943E-01
Fam46a	5723	0.091118642	11450	1.994198E-01
Fam46b	5724	-0.057522116	1443	4.184825E-01
Fam46c	5725	0.210735899	18601	2.741880E-03
Fam46d	5726	-0.066858259	1029.5	3.468868E-01
Fam47c	5727		#N/A	1.000000E+00
Fam47e	5728	-0.038406171	3065	5.892384E-01
Fam49a	5729	0.012106635	5880	8.648947E-01
Fam49b	5730	0.081597259	10709	2.507033E-01
Fam50a	5731	-0.002221957	5065	9.750891E-01
Fam50b	5732		#N/A	1.000000E+00
Fam53a	5733	-0.024388043	4123	7.317578E-01
Fam53b	5734		#N/A	1.000000E+00
Fam53c	5735	0.138947029	14882	4.973692E-02
Fam57a	5736	0.216712165	18813	2.054244E-03
Fam57b	5737		#N/A	1.000000E+00
Fam58b	5738	0.238047986	19328	6.879614E-04
Fam5b	5739	0.254862363	19562	2.704917E-04
Fam5c	5740	0.018062711	6224	7.996000E-01
Fam60a	5741	0.068093668	9708	3.380293E-01
Fam63a	5742	0.164278277	16608	2.009880E-02
Fam63b	5743	0.015347314	6089	8.292247E-01
Fam64a	5744	0.115059355	13325	1.047224E-01
Fam65a	5745	0.205698127	18443	3.476909E-03
Fam65b	5746	0.194714387	18034	5.728823E-03
Fam65c	5747	0.053805701	8820	4.492258E-01
Fam69a	5748	0.050208015	8317	4.801611E-01
Fam69b	5749		#N/A	1.000000E+00
Fam69c	5750	0.11330175	12940	1.101691E-01
Fam71a	5751		#N/A	1.000000E+00
Fam71b	5752	-0.084830261	573	2.323546E-01
Fam71d	5753	0.127545848	14155	7.188866E-02
Fam71e1	5754	0.143605682	15224	4.249002E-02
Fam71e2	5755		#N/A	1.000000E+00
Fam71f1	5756	-0.038406171	3065	5.892384E-01
Fam71f2	5757	-0.038406171	3065	5.892384E-01
Fam72a	5758	0.162363957	16491	2.161627E-02
Fam73a	5759	0.122953744	13840	8.282643E-02
Fam73b	5760	0.085160361	10999	2.305358E-01
Fam76a	5761	0.093357895	11604	1.885509E-01

Spearman Rank correlation analysis performed between Prdm1 and all-expressed genes within the Meredith RNA-seq dataset. Robust Prdm1–associated genes were identified using a cut-off of $p < 0.0005$.

Fam76b	5762	0.103211363	12253	1.458446E-01
Fam78a	5763	0.136345144	14718	5.421370E-02
Fam78b	5764	-0.01069511	4715	8.805217E-01
Fam81a	5765	0.042844892	7800	5.469099E-01
Fam83a	5766	0.287769273	19853	3.597691E-05
Fam83b	5767	0.108871866	12614	1.248833E-01
Fam83c	5768		#N/A	1.000000E+00
Fam83d	5769	0.323576575	19974	2.957754E-06
Fam83e	5770	-0.038406171	3065	5.892384E-01
Fam83f	5771	-0.069290151	956	3.295896E-01
Fam83g	5772	0.120120914	13670	9.021619E-02
Fam83h	5773	0.153789105	15879	2.968825E-02
Fam84a	5774	0.045164933	7952	5.253963E-01
Fam84b	5775	-0.014383343	4549	8.398021E-01
Fam86	5776	0.09602474	11774	1.761800E-01
Fam89a	5777	0.037289612	7448	6.001174E-01
Fam89b	5778	0.085170231	11001	2.304816E-01
Fam92a	5779	0.085348727	11011	2.295024E-01
Fam92b	5780		#N/A	1.000000E+00
Fam96a	5781	0.283787454	19824	4.656246E-05
Fam96b	5782	0.106648219	12468	1.328210E-01
Fam98a	5783	0.200223072	18249	4.473650E-03
Fam98b	5784	0.19557826	18066	5.513189E-03
Fam98c	5785	0.023187074	6703	7.444976E-01
Fan1	5786	0.116679448	13445	9.989178E-02
Fanca	5787	0.204777328	18418	3.629065E-03
Fancb	5788	0.046772676	8063	5.107441E-01
Fancc	5789	0.285249071	19836	4.237553E-05
Fancd2	5790	-0.042879483	2419	5.465860E-01
Fancd2os	5791	-0.054450825	1984	4.437997E-01
Fance	5792	0.100455846	12088	1.569694E-01
Fancf	5793	0.125996265	14045	7.544016E-02
Fancg	5794	0.047957473	8146	5.000837E-01
Fanci	5795	0.074194755	10154.5	2.964228E-01
Fancl	5796	0.037979296	7481	5.933869E-01
Fancm	5797	0.076042467	10349	2.845236E-01
Fank1	5798	-0.037038626	3660	6.025753E-01
Fap	5799	0.033450719	7263	6.381900E-01
Far1	5800	0.227489563	19093	1.196954E-03
Far2	5801	0.062330828	9344	3.805851E-01
Farp1	5802	0.014532059	6044	8.381683E-01
Farp2	5803	0.218976013	18872	1.837738E-03
Fars2	5804	0.044126323	7877	5.349739E-01

Spearman Rank correlation analysis performed between Prdm1 and all-expressed genes within the Meredith RNA-seq dataset. Robust Prdm1–associated genes were identified using a cut-off of $p < 0.0005$.

Table S1, Related to Supplemental Figure 3C. Prdm1 associated genes

Farsa	5805	0.013422251	5962	8.503765E-01
Farsb	5806	0.24494114	19437	4.728539E-04
Fas	5807	0.073489738	10096	3.010489E-01
Fasl	5808		#N/A	1.000000E+00
Fasn	5809	0.144222994	15320	4.159983E-02
Fastk	5810	0.031253314	7140	6.604257E-01
Fastkd1	5811	-0.057594411	1441	4.178969E-01
Fastkd2	5812	0.122225117	13799	8.467896E-02
Fastkd3	5813	0.060398545	9230	3.955551E-01
Fastkd5	5814		#N/A	1.000000E+00
Fat1	5815	0.092033596	11519	1.949252E-01
Fat2	5816	0.000944191	5357	9.894130E-01
Fat3	5817	0.239828456	19362	6.250971E-04
Fat4	5818	0.111318123	12810	1.165805E-01
Fate1	5819	0.052728522	8550.5	4.583686E-01
Fau	5820	0.219222605	18881	1.815458E-03
Fau-ps1	5821		#N/A	1.000000E+00
Fau-ps2	5822		#N/A	1.000000E+00
Faxc	5823	0.037876556	7479	5.943873E-01
Fbf1	5824	-0.130639902	63	6.520401E-02
Fbl	5825	0.058471164	9122	4.108334E-01
Fblim1	5826	0.101272658	12137	1.536071E-01
Fbll1	5827		#N/A	1.000000E+00
Fbln1	5828	0.05962361	9193	4.016566E-01
Fbln2	5829	0.094142023	11652	1.848493E-01
Fbln5	5830	-0.030295313	3919	6.702152E-01
Fbln7	5831	-0.054451513	1703	4.437940E-01
Fbn1	5832	-0.088352268	432	2.134656E-01
Fbn2	5833	0.068631108	9748	3.342214E-01
Fbp1	5834	-0.077397959	691.5	2.760011E-01
Fbp2	5835	-0.0278972	4001.5	6.949626E-01
Fbrs	5836	0.068657688	9751	3.340338E-01
Fbrsl1	5837	0.106068958	12431	1.349511E-01
Fbxl12	5838	0.130860657	14344	6.474719E-02
Fbxl13	5839	-0.023729282	4131	7.387371E-01
Fbxl14	5840	0.086084663	11060	2.254965E-01
Fbxl15	5841	0.011502627	5850	8.715756E-01
Fbxl16	5842		#N/A	1.000000E+00
Fbxl17	5843	-0.008955396	4786	8.998450E-01
Fbxl18	5844	-0.085516921	563	2.285825E-01
Fbxl19	5845	0.036285795	7395	6.099743E-01
Fbxl2	5846	0.199295315	18217	4.665973E-03
Fbxl20	5847	0.065971282	9589	3.533358E-01

Spearman Rank correlation analysis performed between Prdm1 and all-expressed genes within the Meredith RNA-seq dataset. Robust Prdm1–associated genes were identified using a cut-off of $p < 0.0005$.

Table S1, Related to Supplemental Figure 3C. Prdm1 associated genes

Fbxl21	5848	-0.110562757	179.5	1.190970E-01
Fbxl22	5849	0.207284648	18503	3.228208E-03
Fbxl3	5850	0.104405288	12317	1.412147E-01
Fbxl4	5851	-0.138523915	41	5.044325E-02
Fbxl5	5852	0.204167592	18397	3.733100E-03
Fbxl6	5853	0.004146278	5523	9.535336E-01
Fbxl7	5854	0.066713521	9633	3.479341E-01
Fbxl8	5855	0.007505513	5655	9.159948E-01
Fbxo10	5856	0.139622466	14931	4.862644E-02
Fbxo11	5857	0.165647707	16685	1.907068E-02
Fbxo15	5858	-0.023561621	4134	7.405168E-01
Fbxo16	5859	-0.103166823	231.5	1.460195E-01
Fbxo17	5860	0.021916108	6585	7.580558E-01
Fbxo18	5861	0.020945619	6436	7.684588E-01
Fbxo2	5862	-0.086746694	532	2.219355E-01
Fbxo21	5863	0.248076836	19483	3.972944E-04
Fbxo22	5864	0.008999393	5730	8.993556E-01
Fbxo24	5865		#N/A	1.000000E+00
Fbxo25	5866	0.017008745	6171	8.110672E-01
Fbxo27	5867	-0.018447724	4312.5	7.954215E-01
Fbxo28	5868	-0.043806568	2376	5.379400E-01
Fbxo3	5869	0.170255446	16943	1.593907E-02
Fbxo30	5870	0.13308991	14483	6.027940E-02
Fbxo31	5871	-0.009858737	4750	8.898034E-01
Fbxo32	5872	0.135864194	14689	5.507671E-02
Fbxo33	5873	0.05947132	9184	4.028622E-01
Fbxo34	5874	0.078896283	10525	2.667835E-01
Fbxo36	5875	0.126320884	14068	7.468463E-02
Fbxo38	5876	0.145803732	15399	3.939151E-02
Fbxo39	5877		#N/A	1.000000E+00
Fbxo4	5878	0.235194525	19272	8.009777E-04
Fbxo40	5879		#N/A	1.000000E+00
Fbxo41	5880	0.270001639	19718	1.103975E-04
Fbxo42	5881	0.047486825	8113	5.043043E-01
Fbxo43	5882	0.279374404	19788	6.169047E-05
Fbxo44	5883	-0.005810168	4887	9.349220E-01
Fbxo45	5884	0.171091175	16978	1.542192E-02
Fbxo46	5885	0.183846989	17586	9.162180E-03
Fbxo47	5886	-0.016264162	4465	8.191927E-01
Fbxo48	5887	0.143605682	15224	4.249002E-02
Fbxo5	5888	0.136833046	14754	5.334969E-02
Fbxo6	5889	0.034357485	7305	6.291062E-01
Fbxo7	5890	0.026599557	6910	7.084915E-01

Spearman Rank correlation analysis performed between Prdm1 and all-expressed genes within the Meredith RNA-seq dataset. Robust Prdm1–associated genes were identified using a cut-off of $p < 0.0005$.

Table S1, Related to Supplemental Figure 3C. Prdm1 associated genes

Fbxo8	5891	0.134181663	14565	5.818569E-02
Fbxo9	5892	-0.018458082	4301	7.953092E-01
Fbxw10	5893	0.05175716	8454	4.667012E-01
Fbxw11	5894	0.161855168	16461	2.203587E-02
Fbxw13	5895	0.015788425	6112	8.243946E-01
Fbxw14	5896	0.158060666	16178	2.539274E-02
Fbxw15	5897	-0.054451513	1703	4.437940E-01
Fbxw16	5898	-0.032131498	3842.5	6.515021E-01
Fbxw17	5899	0.174025936	17128	1.372019E-02
Fbxw18	5900	-0.054451513	1703	4.437940E-01
Fbxw19	5901	-0.038406171	3065	5.892384E-01
Fbxw2	5902	0.103449601	12267	1.449116E-01
Fbxw20	5903	0.053805701	8820	4.492258E-01
Fbxw21	5904	0.249403277	19500	3.688371E-04
Fbxw22	5905	0.053805701	8820	4.492258E-01
Fbxw24	5906		#N/A	1.000000E+00
Fbxw25	5907	-0.066856562	1139	3.468991E-01
Fbxw26	5908	-0.07739401	763.5	2.760257E-01
Fbxw28	5909	0.053267784	8685	4.537786E-01
Fbxw4	5910	-0.082013253	620	2.482876E-01
Fbxw5	5911	0.106646565	12467	1.328271E-01
Fbxw7	5912	0.121853188	13780	8.563734E-02
Fbxw8	5913	0.164758612	16641	1.973284E-02
Fbxw9	5914	0.053704799	8784	4.500779E-01
Fcamr	5915	0.022198316	6629.5	7.550388E-01
Fcer1a	5916		#N/A	1.000000E+00
Fcer1g	5917	0.137561686	14795	5.208064E-02
Fcer2a	5918	0.113641312	13235	1.090999E-01
Fcf1	5919	0.179928142	17398	1.078899E-02
Fcgbp	5920	0.135054478	14623	5.655531E-02
Fcgr1	5921	0.060514514	9238	3.946468E-01
Fcgr2b	5922	0.004903552	5559.5	9.450593E-01
Fcgr3	5923	0.015129558	6077	8.316114E-01
Fcgr4	5924	0.047724464	8124	5.021709E-01
Fcgrt	5925	0.043946654	7861	5.366396E-01
Fcho1	5926	0.178866943	17348	1.127123E-02
Fcho2	5927	0.090783195	11411	2.010864E-01
Fchsd1	5928	-0.04310276	2409.5	5.444975E-01
Fchsd2	5929	0.117496422	13492	9.752363E-02
Fcna	5930	-0.077393517	797	2.760287E-01
Fcnb	5931		#N/A	1.000000E+00
Fcor	5932		#N/A	1.000000E+00
Fcrl1	5933	0.19120528	17884	6.684412E-03

Spearman Rank correlation analysis performed between Prdm1 and all-expressed genes within the Meredith RNA-seq dataset. Robust Prdm1–associated genes were identified using a cut-off of $p < 0.0005$.

Table S1, Related to Supplemental Figure 3C. Prdm1 associated genes

Fcrl5	5934		#N/A	1.000000E+00
Fcrl6	5935	-0.027019998	4028	7.040978E-01
Fcrla	5936	0.239480429	19357	6.369516E-04
Fcrlb	5937		#N/A	1.000000E+00
Fcrls	5938	-0.001224019	5151	9.862757E-01
Fdft1	5939	0.159898584	16270	2.371561E-02
Fdps	5940	0.141955087	15080	4.494911E-02
Fdx1	5941	0.036038039	7383	6.124180E-01
Fdx1l	5942	0.131051893	14353	6.435359E-02
Fdxacb1	5943	-0.063313294	1356	3.731079E-01
Fdxr	5944	0.095277197	11716	1.795855E-01
Fech	5945	0.199181321	18210	4.690109E-03
Fem1a	5946	0.133751132	14534	5.900407E-02
Fem1b	5947	0.159104516	16231	2.442813E-02
Fem1c	5948	0.141714926	15061	4.531664E-02
Fen1	5949	0.132941347	14474	6.056904E-02
Fer	5950	-0.058060059	1434	4.141366E-01
Fer1l4	5951	-0.038406171	3065	5.892384E-01
Fer1l5	5952	0.113548679	13094	1.093908E-01
Fer1l6	5953	0.010874103	5819	8.785374E-01
Ferd3l	5954		#N/A	1.000000E+00
Fermt1	5955	0.099022442	11998	1.630031E-01
Fermt2	5956	0.025270798	6841	7.224400E-01
Fermt3	5957	-0.011735389	4677	8.690000E-01
Fes	5958	-0.00833974	4806	9.066980E-01
Fetub	5959	-0.09526621	368.5	1.796359E-01
Fev	5960	0.154646726	15961	2.877924E-02
Fez1	5961	0.03037917	7087.5	6.693560E-01
Fez2	5962	-0.023441808	4141	7.417895E-01
Fezf1	5963	-0.054450825	1984	4.437997E-01
Fezf2	5964	0.039575823	7608	5.779398E-01
Ffar1	5965		#N/A	1.000000E+00
Ffar2	5966	0.142784335	15134	4.369919E-02
Ffar3	5967		#N/A	1.000000E+00
Ffar4	5968	-0.066858259	1029.5	3.468868E-01
Fga	5969	-0.086748028	498.5	2.219283E-01
Fgb	5970	-0.028140487	3992	6.924367E-01
Fgd1	5971	0.051376193	8418	4.699919E-01
Fgd2	5972	0.032909762	7233	6.436350E-01
Fgd3	5973	0.134154998	14562	5.823610E-02
Fgd4	5974	0.006505018	5616	9.271595E-01
Fgd5	5975	0.022117163	6595	7.559060E-01
Fgd6	5976	0.03049981	7097	6.681207E-01

Spearman Rank correlation analysis performed between Prdm1 and all-expressed genes within the Meredith RNA-seq dataset. Robust Prdm1–associated genes were identified using a cut-off of $p < 0.0005$.

Table S1, Related to Supplemental Figure 3C. Prdm1 associated genes

Fgf1	5977	-0.057404374	1447	4.194372E-01
Fgf10	5978	-0.078573565	659	2.687509E-01
Fgf11	5979	0.105994793	12421	1.352257E-01
Fgf12	5980	-0.044754161	2348	5.291738E-01
Fgf13	5981	0.104493467	12325	1.408772E-01
Fgf14	5982	0.069404702	9807	3.287887E-01
Fgf15	5983		#N/A	1.000000E+00
Fgf16	5984		#N/A	1.000000E+00
Fgf17	5985	-0.038406171	3065	5.892384E-01
Fgf18	5986		#N/A	1.000000E+00
Fgf2	5987	0.014301797	6015.5	8.406982E-01
Fgf20	5988	-0.031190668	3881.5	6.610641E-01
Fgf21	5989	-0.008791638	4796	9.016672E-01
Fgf22	5990		#N/A	1.000000E+00
Fgf23	5991	-0.038406171	3065	5.892384E-01
Fgf3	5992	-0.054450825	1984	4.437997E-01
Fgf4	5993		#N/A	1.000000E+00
Fgf5	5994	0.105636658	12399	1.365578E-01
Fgf6	5995	-0.038406171	3065	5.892384E-01
Fgf7	5996	0.143605682	15224	4.249002E-02
Fgf8	5997	-0.032762514	3799	6.451205E-01
Fgf9	5998	0.018651794	6260	7.932092E-01
Fgfbp1	5999	0.137727506	14804	5.179536E-02
Fgfbp3	6000	0.018797371	6268	7.916319E-01
Fgfr1	6001	0.094349042	11664	1.838810E-01
Fgfr1op	6002	0.196717158	18110	5.240023E-03
Fgfr1op2	6003	0.141453953	15047	4.571886E-02
Fgfr2	6004	0.169071581	16877	1.669752E-02
Fgfr3	6005	0.008682857	5712	9.028778E-01
Fgfr4	6006		#N/A	1.000000E+00
Fgfrl1	6007	0.020434681	6357	7.739526E-01
Fgg	6008	-0.130638019	66	6.520792E-02
Fggy	6009	0.103374222	12263	1.452063E-01
Fgl1	6010	-0.018447724	4312.5	7.954215E-01
Fgl2	6011	0.21648961	18801	2.076736E-03
Fgr	6012	0.101670244	12161	1.519903E-01
Fh1	6013	0.24809882	19484	3.968066E-04
Fhad1	6014	-0.073640603	911	3.000550E-01
Fhdc1	6015	0.071744492	9972	3.127051E-01
Fhit	6016	0.263352867	19662	1.647214E-04
Fhl1	6017	-0.021372519	4197	7.638775E-01
Fhl2	6018	0.127284236	14137	7.247853E-02
Fhl3	6019	0.056614468	9015	4.258752E-01

Spearman Rank correlation analysis performed between Prdm1 and all-expressed genes within the Meredith RNA-seq dataset. Robust Prdm1–associated genes were identified using a cut-off of $p < 0.0005$.

Fhl4	6020	0.014759448	6059.5	8.356716E-01
Fhl5	6021		#N/A	1.000000E+00
Fhod1	6022	-0.117175895	130	9.844737E-02
Fhod3	6023	-0.014529376	4540	8.381978E-01
Fibcd1	6024	0.020875719	6412.5	7.692098E-01
Fibin	6025	-0.038406171	3065	5.892384E-01
Fibp	6026	-0.15295271	22	3.059824E-02
Ficd	6027	0.019200096	6290	7.872731E-01
Fig4	6028	-0.051493861	2173	4.689742E-01
Figf	6029	0.053267784	8685	4.537786E-01
Figla	6030		#N/A	1.000000E+00
Fign	6031	0.246739175	19469	4.280430E-04
Fignl1	6032	0.141275339	15032	4.599587E-02
Fignl2	6033	0.051018269	8386	4.730951E-01
Filip1	6034	0.225149075	19048	1.348808E-03
Filip1l	6035	0.146846865	15468	3.798901E-02
Fip1l1	6036	0.092615437	11557	1.921055E-01
Fis1	6037	-0.081203003	630	2.530077E-01
Fitm1	6038	-0.038406171	3065	5.892384E-01
Fitm2	6039	0.026992021	6930	7.043899E-01
Fiz1	6040	0.09135458	11477	1.982537E-01
Fjx1	6041	-0.038406171	3065	5.892384E-01
Fkbp10	6042	-0.038406171	3065	5.892384E-01
Fkbp11	6043	0.271619438	19730	9.999665E-05
Fkbp14	6044		#N/A	1.000000E+00
Fkbp15	6045	0.042762258	7786	5.476840E-01
Fkbp1a	6046	0.233884081	19255	8.584072E-04
Fkbp1b	6047	-0.005447284	4901	9.389784E-01
Fkbp2	6048	0.201499875	18309	4.220628E-03
Fkbp3	6049	0.155713808	16020	2.768152E-02
Fkbp4	6050	0.110413948	12748	1.195977E-01
Fkbp5	6051	0.127764882	14173	7.139781E-02
Fkbp6	6052	0.219781289	18894.5	1.765887E-03
Fkbp7	6053	0.112889218	12912	1.114792E-01
Fkbp8	6054	0.192766294	17942	6.243028E-03
Fkbp9	6055	0.040321764	7650	5.707874E-01
Fkbpl	6056	-0.005125499	4927	9.425767E-01
Fkrp	6057	0.102811388	12234	1.474212E-01
Fktn	6058	0.190298217	17853	6.953488E-03
Flad1	6059	0.123843577	13892	8.060839E-02
Flcn	6060	-0.010112613	4740	8.869843E-01
Flg	6061	0.083630981	10890	2.390474E-01
Flg2	6062		#N/A	1.000000E+00

Spearman Rank correlation analysis performed between Prdm1 and all-expressed genes within the Meredith RNA-seq dataset. Robust Prdm1–associated genes were identified using a cut-off of $p < 0.0005$.

Table S1, Related to Supplemental Figure 3C. Prdm1 associated genes

Fli1	6063	0.179009741	17355	1.120525E-02
Flii	6064	0.121644702	13768	8.617835E-02
Flna	6065	-0.022033813	4171	7.567970E-01
Flnb	6066	0.129060507	14241	6.854977E-02
Flnc	6067	-0.054514719	1539	4.432644E-01
Flot1	6068	0.180509308	17429	1.053269E-02
Flot2	6069	-0.032696239	3806	6.457895E-01
Flrt1	6070	-0.001202082	5176.5	9.865216E-01
Flrt2	6071	0.118896395	13578	9.356931E-02
Flrt3	6072	0.115060879	13326	1.047177E-01
Flt1	6073	0.072432597	10026	3.080746E-01
Flt3	6074	0.146617372	15455	3.829389E-02
Flt3l	6075		#N/A	1.000000E+00
Flt4	6076	0.134865579	14610	5.690493E-02
Flywch1	6077	-0.053194508	2136	4.544008E-01
Flywch2	6078	0.076881974	10407	2.792247E-01
Fmn1	6079	0.071826662	9985	3.121497E-01
Fmn2	6080	0.102748244	12230	1.476712E-01
Fmnl1	6081	0.171091198	16979	1.542190E-02
Fmnl2	6082	0.098463615	11952	1.654019E-01
Fmnl3	6083	0.173981536	17123	1.374466E-02
Fmo1	6084	-0.077393517	797	2.760287E-01
Fmo2	6085	0.009870742	5767	8.896700E-01
Fmo3	6086	-0.077393023	832.5	2.760318E-01
Fmo4	6087		#N/A	1.000000E+00
Fmo5	6088	-0.04516216	2319	5.254218E-01
Fmo6	6089		#N/A	1.000000E+00
Fmo9	6090	0.091544822	11492	1.973170E-01
Fmod	6091		#N/A	1.000000E+00
Fmr1	6092	0.150821376	15702	3.302523E-02
Fmr1nb	6093	0.053267784	8685	4.537786E-01
Fn1	6094	-0.085368865	568	2.293922E-01
Fn3k	6095	0.117156079	13473	9.850471E-02
Fn3krp	6096	0.027888984	6969	6.950480E-01
Fnbp1	6097	0.253011866	19538	3.007044E-04
Fnbp1l	6098	0.140939867	15017	4.651994E-02
Fnbp4	6099	0.1791973	17367	1.111911E-02
Fnd3c2	6100	-0.077397959	691.5	2.760011E-01
Fndc1	6101	-0.053184555	2137	4.544853E-01
Fndc3a	6102	0.184989315	17635	8.730802E-03
Fndc3b	6103	0.089704707	11324	2.065128E-01
Fndc3c1	6104	-0.086748028	498.5	2.219283E-01
Fndc4	6105	-0.038406171	3065	5.892384E-01

Spearman Rank correlation analysis performed between Prdm1 and all-expressed genes within the Meredith RNA-seq dataset. Robust Prdm1–associated genes were identified using a cut-off of $p < 0.0005$.

Table S1, Related to Supplemental Figure 3C. Prdm1 associated genes

Fndc5	6106	0.112702055	12902	1.120776E-01
Fndc7	6107	0.10056402	12094	1.565209E-01
Fndc8	6108	0.116326563	13417	1.009286E-01
Fndc9	6109	0.323877033	19975	2.892331E-06
Fnip1	6110	0.163913793	16589	2.038040E-02
Fnip2	6111	0.218517457	18856	1.879835E-03
Fnta	6112	0.243815812	19425	5.030691E-04
Fntb	6113	-0.082946522	606	2.429271E-01
Focad	6114	0.105534732	12395	1.369388E-01
Folh1	6115	-0.017417228	4393	8.066179E-01
Folr1	6116	0.098251687	11936	1.663185E-01
Folr2	6117	-0.086747139	513	2.219331E-01
Folr4	6118	0.186725681	17707	8.109563E-03
Fopnl	6119	0.133212613	14493	6.004104E-02
Fos	6120	0.163956149	16591	2.034750E-02
Fosb	6121	0.156905643	16103	2.649802E-02
Fosl1	6122	0.010726378	5806	8.801750E-01
Fosl2	6123	0.142566059	15120	4.402533E-02
Foxa1	6124	0.107603682	12529	1.293641E-01
Foxa2	6125	0.1544596	15936.5	2.897553E-02
Foxa3	6126	0.064665329	9515	3.629669E-01
Foxb1	6127	0.064668769	9516.5	3.629413E-01
Foxb2	6128		#N/A	1.000000E+00
Foxc1	6129	0.129425567	14269	6.776425E-02
Foxc2	6130		#N/A	1.000000E+00
Foxd1	6131	0.113548679	13094	1.093908E-01
Foxd2	6132	0.155689991	16016	2.770563E-02
Foxd3	6133	0.02691967	6926	7.051454E-01
Foxd4	6134		#N/A	1.000000E+00
Foxe1	6135	-0.033388476	3771	6.388155E-01
Foxe3	6136		#N/A	1.000000E+00
Foxf1	6137		#N/A	1.000000E+00
Foxf2	6138	0.021317126	6513.5	7.644715E-01
Foxg1	6139	0.193478205	17972	6.050522E-03
Foxh1	6140		#N/A	1.000000E+00
Foxi1	6141	0.155008799	15980	2.840268E-02
Foxi2	6142	-0.032762514	3799	6.451205E-01
Foxi3	6143		#N/A	1.000000E+00
Foxj1	6144	-0.038542464	2500	5.879166E-01
Foxj2	6145	0.017423216	6197	8.065527E-01
Foxj3	6146	0.026996848	6932	7.043395E-01
Foxk1	6147	0.215518312	18778	2.177544E-03
Foxk2	6148	0.075130726	10255	2.903545E-01

Spearman Rank correlation analysis performed between Prdm1 and all-expressed genes within the Meredith RNA-seq dataset. Robust Prdm1–associated genes were identified using a cut-off of $p < 0.0005$.

Table S1, Related to Supplemental Figure 3C. Prdm1 associated genes

FOXL1	6149		#N/A	1.000000E+00
Foxl1	6150		#N/A	1.000000E+00
Foxl2	6151	0.198043012	18167	4.937348E-03
Foxm1	6152	0.081851594	10734	2.492244E-01
Foxn1	6153	-0.00578499	4888	9.352034E-01
Foxn2	6154	0.175677837	17199	1.283664E-02
Foxn3	6155	0.114085877	13258	1.077123E-01
Foxn4	6156		#N/A	1.000000E+00
Foxo1	6157	0.176916199	17265	1.220744E-02
Foxo3	6158	0.090962001	11431	2.001968E-01
Foxo4	6159	0.095947815	11769	1.765283E-01
Foxo6	6160		#N/A	1.000000E+00
Foxp1	6161	0.038382704	7518	5.894661E-01
Foxp2	6162	0.132173252	14423	6.208484E-02
Foxp3	6163	-0.038406171	3065	5.892384E-01
Foxp4	6164	-0.030505792	3915	6.680595E-01
Foxq1	6165	-0.071441434	937	3.147588E-01
Foxr1	6166	-0.070188339	945	3.233438E-01
Foxr2	6167	0.132263077	14430	6.190598E-02
Foxred1	6168	0.124845825	13959	7.816773E-02
Foxred2	6169	0.019154678	6288	7.877644E-01
Foxs1	6170	0.053805701	8820	4.492258E-01
Fpgs	6171	-0.004080971	4983	9.542647E-01
Fpgt	6172	0.052705781	8522	4.585627E-01
Fpr-rs3	6173		#N/A	1.000000E+00
Fpr-rs4	6174		#N/A	1.000000E+00
Fpr-rs6	6175	-0.054450825	1984	4.437997E-01
Fpr-rs7	6176	-0.038406171	3065	5.892384E-01
Fpr1	6177		#N/A	1.000000E+00
Fpr2	6178		#N/A	1.000000E+00
Fpr3	6179	0.020431343	6349	7.739885E-01
Fra10ac1	6180	0.026449658	6900	7.100603E-01
Fras1	6181	0.091595131	11496	1.970698E-01
Frat1	6182		#N/A	1.000000E+00
Frat2	6183	-0.016551874	4456	8.160506E-01
Frem1	6184	0.071856047	9987	3.119513E-01
Frem2	6185	0.092968202	11581	1.904106E-01
Frem3	6186	0.020875719	6412.5	7.692098E-01
Frg1	6187	-0.051125986	2179	4.721600E-01
Frk	6188	0.080121914	10601	2.594015E-01
Frmd3	6189	-0.014525416	4541	8.382413E-01
Frmd4a	6190	0.108022665	12554	1.278702E-01
Frmd4b	6191	0.245144563	19440	4.675747E-04

Spearman Rank correlation analysis performed between Prdm1 and all-expressed genes within the Meredith RNA-seq dataset. Robust Prdm1–associated genes were identified using a cut-off of $p < 0.0005$.

Frmd5	6192	0.115902701	13387	1.021853E-01
Frmd6	6193	0.242940286	19413	5.278020E-04
Frmd7	6194	0.021317126	6513.5	7.644715E-01
Frmd8	6195	0.10640591	12453	1.337089E-01
Frmpd1	6196	0.09781383	11894	1.682242E-01
Frmpd3	6197	0.197522372	18141	5.054280E-03
Frmpd4	6198	0.067325091	9660	3.435227E-01
Frrs1	6199	0.319613279	19962	3.963940E-06
Frrs1l	6200	-0.038406171	3065	5.892384E-01
Frs2	6201	0.112079596	12858	1.140857E-01
Frs3	6202	0.091064291	11443	1.996892E-01
Fry	6203	0.191088247	17881	6.718599E-03
Fryl	6204	0.237695238	19319	7.010883E-04
Frzb	6205	0.113548679	13094	1.093908E-01
Fsbp	6206	0.092046888	11520	1.948604E-01
Fscb	6207		#N/A	1.000000E+00
Fscn1	6208	0.088406031	11233	2.131860E-01
Fscn2	6209		#N/A	1.000000E+00
Fscn3	6210		#N/A	1.000000E+00
Fsd1	6211		#N/A	1.000000E+00
Fsd1l	6212	0.098370652	11943	1.658035E-01
Fsd2	6213	-0.110568513	165.5	1.190777E-01
Fshb	6214	-0.038406171	3065	5.892384E-01
Fshr	6215	0.134263791	14577	5.803065E-02
Fsip1	6216	0.080406705	10626.5	2.577065E-01
Fsip2	6217	0.08479514	10971	2.325487E-01
Fst	6218	-0.066858259	1029.5	3.468868E-01
Fstl1	6219	0.264167709	19668	1.569256E-04
Fstl3	6220	0.039184473	7588	5.817089E-01
Fstl4	6221	-0.029278786	3957	6.806638E-01
Fstl5	6222	0.18585385	17676	8.416401E-03
Ftcd	6223		#N/A	1.000000E+00
Fth-ps2	6224	-0.004526355	4961.5	9.492796E-01
Fth1	6225	0.125854604	14029	7.577180E-02
Fthl17	6226		#N/A	1.000000E+00
Ftl1	6227	0.119011905	13590	9.324883E-02
Ftl2	6228		#N/A	1.000000E+00
Ftmt	6229		#N/A	1.000000E+00
Fto	6230	0.0915361	11490	1.973599E-01
Ftsj1	6231	0.206333103	18469	3.375365E-03
Ftsj2	6232	0.040432604	7658	5.697282E-01
Ftsj3	6233	0.197810537	18158	4.989257E-03
Ftsjd1	6234	-0.028054547	3995	6.933286E-01

Table S1, Related to Supplemental Figure 3C. Prdm1 associated genes

Ftsjd2	6235	0.190543633	17866	6.879750E-03
Ftx	6236	0.08835102	11231	2.134721E-01
Fubp1	6237	0.062913346	9387	3.761408E-01
Fubp3	6238	0.20467456	18413	3.646414E-03
Fuca1	6239	0.143548135	15163	4.257382E-02
Fuca2	6240	0.107970193	12552	1.280566E-01
Fuk	6241		#N/A	1.000000E+00
Fundc1	6242	0.159565433	16254	2.401234E-02
Fundc2	6243	0.157028699	16108	2.637834E-02
Fuom	6244	0.067540636	9677	3.419764E-01
Furin	6245	0.146698472	15462	3.818592E-02
Fus	6246	0.124927735	13964	7.797093E-02
Fut1	6247	0.017259388	6187	8.083364E-01
Fut10	6248	0.215880936	18785	2.139399E-03
Fut11	6249	0.062536606	9359	3.790115E-01
Fut2	6250	-0.025085703	4097	7.243904E-01
Fut4	6251	0.254453806	19558	2.769084E-04
Fut7	6252	-0.038406171	3065	5.892384E-01
Fut8	6253	0.117616264	13501	9.718002E-02
Fut9	6254	0.087817123	11185	2.162625E-01
Fuz	6255	0.011086897	5830	8.761793E-01
Fv1	6256		#N/A	1.000000E+00
Fxn	6257	0.279968863	19797	5.941267E-05
Fxr1	6258	0.020107194	6334	7.774798E-01
Fxr2	6259	-0.060322927	1393	3.961480E-01
Fxyd1	6260		#N/A	1.000000E+00
Fxyd2	6261	0.01853406	6250	7.944853E-01
Fxyd3	6262	0.06119663	9270	3.893297E-01
Fxyd4	6263	0.178780143	17343	1.131150E-02
Fxyd5	6264	0.200171396	18248	4.484172E-03
Fxyd6	6265	-0.066856562	1139	3.468991E-01
Fxyd7	6266	0.143605682	15224	4.249002E-02
Fyb	6267	0.076719326	10392	2.802461E-01
Fyco1	6268	0.045550888	7970	5.218595E-01
Fyn	6269	0.224490888	19030	1.394582E-03
Fyttd1	6270	0.211352684	18627	2.662317E-03
Fzd1	6271	-0.002688612	5042	9.698595E-01
Fzd10	6272	0.070263217	9855	3.228265E-01
Fzd2	6273	0.204610267	18410	3.657306E-03
Fzd3	6274	0.005510157	5580	9.382755E-01
Fzd4	6275		#N/A	1.000000E+00
Fzd5	6276	0.149828474	15640	3.421055E-02
Fzd6	6277	0.1330046	14476	6.044558E-02

Spearman Rank correlation analysis performed between Prdm1 and all-expressed genes within the Meredith RNA-seq dataset. Robust Prdm1–associated genes were identified using a cut-off of $p < 0.0005$.

Table S1, Related to Supplemental Figure 3C. Prdm1 associated genes

Fzd7	6278	0.107529322	12525	1.296306E-01
Fzd8	6279	-0.077393517	797	2.760287E-01
Fzd9	6280		#N/A	1.000000E+00
Fzr1	6281	0.17939066	17376	1.103091E-02
G0s2	6282	0.306161917	19927	1.038568E-05
G2e3	6283	0.156792014	16096	2.660896E-02
G3bp1	6284	0.176035523	17223	1.265204E-02
G3bp2	6285	0.21047068	18592	2.776750E-03
G430049J08Rik	6286		#N/A	1.000000E+00
G430095P16Ri	6287	-0.054451513	1703	4.437940E-01
G530011O06Ri	6288	0.126231953	14060	7.489100E-02
G530012D18Ri	6289	0.09266156	11560	1.918833E-01
G630016G05Ri	6290	0.151934152	15763	3.173844E-02
G630018N14Ri	6291	-0.080003204	642	2.601103E-01
G630090E17Ril	6292	0.169197971	16885	1.661507E-02
G6pc	6293	0.113548679	13094	1.093908E-01
G6pc2	6294	0.0910137	11439	1.999401E-01
G6pc3	6295	0.024554175	6791	7.300012E-01
G6pd2	6296		#N/A	1.000000E+00
G6pdx	6297	0.222943048	18987	1.507884E-03
G930045G22Ri	6298	0.000307056	5327	9.965570E-01
Gaa	6299	-0.147803701	30	3.673982E-02
Gab1	6300	0.061818055	9324	3.845236E-01
Gab2	6301	0.17929892	17372	1.107268E-02
Gab3	6302	0.046909261	8070	5.095092E-01
Gabarap	6303	0.075280133	10284	2.893936E-01
Gabarapl1	6304	-0.007971848	4822	9.107964E-01
Gabarapl2	6305	0.216710502	18812	2.054411E-03
Gabbr1	6306	0.049528646	8255	4.861295E-01
Gabbr2	6307	0.028381078	6988	6.899421E-01
Gabpa	6308	0.097031058	11839	1.716715E-01
Gabpb1	6309	0.083731928	10898	2.384789E-01
Gabpb2	6310	-0.074635	891	2.935581E-01
Gabra1	6311	0.145799381	15398	3.939745E-02
Gabra2	6312	0.039668815	7610	5.770459E-01
Gabra3	6313	-0.001202082	5176.5	9.865216E-01
Gabra4	6314	-0.070398998	943	3.218900E-01
Gabra5	6315	0.161746903	16458	2.212605E-02
Gabra6	6316	0.020875719	6412.5	7.692098E-01
Gabrb1	6317	0.089274018	11292	2.087090E-01
Gabrb2	6318	0.033230702	7253	6.404023E-01
Gabrb3	6319	-0.079026468	654	2.659927E-01
Gabrd	6320	0.045959241	8002	5.181308E-01

Spearman Rank correlation analysis performed between Prdm1 and all-expressed genes within the Meredith RNA-seq dataset. Robust Prdm1–associated genes were identified using a cut-off of $p < 0.0005$.

Table S1, Related to Supplemental Figure 3C. Prdm1 associated genes

Gabre	6321	-0.038406171	3065	5.892384E-01
Gabrg1	6322	0.05349265	8771	4.518723E-01
Gabrg2	6323	-0.086750697	472.5	2.219141E-01
Gabrg3	6324	0.155485174	16007	2.791366E-02
Gabrp	6325	0.132862036	14467	6.072413E-02
Gabrq	6326		#N/A	1.000000E+00
Gabrr1	6327	-0.086755146	452	2.218903E-01
Gabrr2	6328	0.021317126	6513.5	7.644715E-01
Gabrr3	6329	0.053267784	8685	4.537786E-01
Gad1	6330	-0.044376133	2354	5.326623E-01
Gad1-ps	6331		#N/A	1.000000E+00
Gad2	6332	0.129949994	14296	6.664868E-02
Gadd45a	6333	0.055422374	8952	4.356986E-01
Gadd45b	6334	0.071799192	9983	3.123353E-01
Gadd45g	6335	0.083337383	10856	2.407063E-01
Gadd45gip1	6336	0.117440405	13487	9.768457E-02
Gadl1	6337	0.006829572	5625	9.235361E-01
Gak	6338	0.082892074	10821	2.432376E-01
Gal	6339	-0.001224019	5151	9.862757E-01
Gal3st1	6340	0.133232038	14496	6.000338E-02
Gal3st2	6341	-0.008139554	4815	9.089278E-01
Gal3st3	6342	0.266527982	19691	1.362450E-04
Gal3st4	6343	-0.027767303	4007	6.963126E-01
Galc	6344	-0.044759954	2347	5.291205E-01
Gale	6345	0.092660893	11559	1.918865E-01
Galk1	6346	-0.101472602	267	1.527924E-01
Galk2	6347	0.137663298	14800	5.190567E-02
Galm	6348	0.157407785	16124	2.601254E-02
Galns	6349	0.177186178	17275	1.207392E-02
Galnt1	6350	0.017425153	6198	8.065317E-01
Galnt10	6351	0.085376384	11014	2.293510E-01
Galnt11	6352	0.108065351	12557	1.277188E-01
Galnt12	6353	0.078113084	10472	2.715751E-01
Galnt13	6354	0.168472435	16851	1.709321E-02
Galnt14	6355	0.129228051	14255	6.818834E-02
Galnt15	6356	0.053267784	8685	4.537786E-01
Galnt16	6357	-0.016832394	4419	8.129899E-01
Galnt18	6358	0.127867404	14182	7.116899E-02
Galnt2	6359	-0.013472233	4595	8.498259E-01
Galnt3	6360	0.144313771	15325	4.147026E-02
Galnt4	6361		#N/A	1.000000E+00
Galnt5	6362	0.158245927	16188	2.521919E-02
Galnt6	6363	-0.08324675	599	2.412200E-01

Spearman Rank correlation analysis performed between Prdm1 and all-expressed genes within the Meredith RNA-seq dataset. Robust Prdm1–associated genes were identified using a cut-off of $p < 0.0005$.

Galnt7	6364	0.054153118	8874	4.462991E-01
Galnt9	6365	0.141418724	15043	4.577339E-02
Galntl5	6366	-0.038406171	3065	5.892384E-01
Galntl6	6367	0.187929024	17759	7.702384E-03
Galp	6368		#N/A	1.000000E+00
Galr1	6369		#N/A	1.000000E+00
Galr2	6370	-0.038406171	3065	5.892384E-01
Galt	6371	0.079614724	10565	2.624391E-01
Gamt	6372	0.068210352	9713.5	3.372002E-01
Gan	6373	0.140446452	14984	4.729980E-02
Ganab	6374	0.19583962	18078	5.449398E-03
Ganc	6375	0.108300914	12569	1.268855E-01
Gap43	6376	0.147730595	15519	3.683402E-02
Gapdh	6377	0.12862244	14219	6.950218E-02
GAPDH	6378	0.207130702	18497	3.251616E-03
Gapdhs	6379	0.061285945	9281	3.886367E-01
Gapt	6380	0.107877037	12547	1.283879E-01
Gapvd1	6381	0.148933282	15586	3.530997E-02
Gar1	6382	0.323562561	19973	2.960840E-06
Garem	6383	0.282085341	19810	5.192839E-05
Gareml	6384		#N/A	1.000000E+00
Garnl3	6385	0.210403024	18590	2.785709E-03
Gars	6386	0.098123707	11921	1.668738E-01
Gart	6387	0.029408987	7039	6.793221E-01
Gas1	6388	0.050280495	8325	4.795268E-01
Gas2	6389	0.027198336	6939	7.022371E-01
Gas2l1	6390	-0.057755117	1438	4.165969E-01
Gas2l2	6391	-0.038406171	3065	5.892384E-01
Gas2l3	6392	0.169407097	16897	1.647944E-02
Gas5	6393	0.101943012	12179	1.508885E-01
Gas6	6394	0.054434122	8889	4.439397E-01
Gas7	6395	0.144696647	15339	4.092747E-02
Gas8	6396	0.199091454	18204	4.709215E-03
Gast	6397	0.14341533	15158	4.276772E-02
Gata1	6398	0.127871888	14183	7.115900E-02
Gata2	6399	0.108608001	12596	1.258056E-01
Gata3	6400	0.219675204	18892	1.775204E-03
Gata4	6401	0.038722635	7542	5.861714E-01
Gata5	6402	0.074900209	10229	2.918413E-01
Gata6	6403	-0.095280123	319	1.795721E-01
Gatad1	6404	0.141774525	15066	4.522520E-02
Gatad2a	6405	0.027541539	6951	6.986613E-01
Gatad2b	6406	-0.036898332	3663	6.039511E-01

Spearman Rank correlation analysis performed between Prdm1 and all-expressed genes within the Meredith RNA-seq dataset. Robust Prdm1–associated genes were identified using a cut-off of $p < 0.0005$.

Table S1, Related to Supplemental Figure 3C. Prdm1 associated genes

Gatc	6407	0.000507554	5340	9.943088E-01
Gatm	6408	0.026405151	6897	7.105263E-01
Gatsl2	6409	0.083780578	10903	2.382053E-01
Gatsl3	6410	0.060045238	9214	3.983299E-01
Gba	6411	0.110744769	12775	1.184868E-01
Gba2	6412	0.052948806	8592	4.564905E-01
Gbas	6413	0.099362737	12024	1.615552E-01
Gbe1	6414	0.20665539	18483	3.324858E-03
Gbf1	6415	-0.010805841	4708	8.792940E-01
Gbgt1	6416		#N/A	1.000000E+00
Gbp10	6417		#N/A	1.000000E+00
GBP11	6418	-0.038406171	3065	5.892384E-01
Gbp11	6419	0.032778047	7225	6.449637E-01
Gbp2	6420	0.133782506	14538	5.894411E-02
Gbp2b	6421	-0.00567934	4894	9.363842E-01
Gbp3	6422	0.173571945	17103	1.397217E-02
Gbp4	6423		#N/A	1.000000E+00
Gbp5	6424	0.155785912	16023	2.760866E-02
Gbp6	6425	0.114502016	13292	1.064261E-01
Gbp7	6426	0.134835681	14608	5.696042E-02
Gbp8	6427	0.115541093	13362	1.032671E-01
Gbp9	6428	0.068151061	9712	3.376213E-01
Gbx1	6429	-0.086750697	472.5	2.219141E-01
Gbx2	6430		#N/A	1.000000E+00
Gc	6431	0.093510595	11614	1.878259E-01
Gca	6432	0.115468516	13355	1.034853E-01
Gcat	6433	0.011106841	5831	8.759584E-01
Gcc1	6434	0.00820427	5686	9.082069E-01
Gcc2	6435	-0.039428838	2483	5.793541E-01
Gcdh	6436	0.026548742	6907	7.090232E-01
Gcfc2	6437	0.205384532	18432	3.528069E-03
Gcg	6438	-0.04601882	2290	5.175879E-01
Gcgr	6439	0.021316405	6469	7.644793E-01
Gch1	6440	0.08020281	10612	2.589193E-01
Gchfr	6441	0.150418855	15673	3.350148E-02
Gck	6442	-0.015750861	4485	8.248056E-01
Gckr	6443	0.052728522	8550.5	4.583686E-01
Gclc	6444	0.045784418	7990	5.197255E-01
Gclm	6445	0.014427031	6039	8.393221E-01
Gcm1	6446	0.028518853	6994	6.885151E-01
Gcm2	6447	0.113548679	13094	1.093908E-01
Gcn1l1	6448	0.217613332	18838	1.965420E-03
Gcnt1	6449	0.049650035	8266	4.850602E-01

Spearman Rank correlation analysis performed between Prdm1 and all-expressed genes within the Meredith RNA-seq dataset. Robust Prdm1–associated genes were identified using a cut-off of $p < 0.0005$.

Table S1, Related to Supplemental Figure 3C. Prdm1 associated genes

Gcnt2	6450	0.116967138	13462	9.905272E-02
Gcnt3	6451	0.074161834	10146	2.966378E-01
Gcnt4	6452	0.195063819	18048	5.640707E-03
Gcnt7	6453	0.053267784	8685	4.537786E-01
Gcom1	6454	0.074405963	10170	2.950462E-01
Gcsam	6455	0.276448718	19768	7.414679E-05
Gcsh	6456	0.107275543	12513	1.305434E-01
Gda	6457	0.061117573	9269	3.899438E-01
Gdap1	6458	0.147822381	15529	3.671578E-02
Gdap1l1	6459	0.024072636	6760	7.350967E-01
Gdap2	6460	0.227413918	19090	1.201606E-03
Gde1	6461	0.119451644	13623	9.203677E-02
Gdf1	6462		#N/A	1.000000E+00
Gdf10	6463	0.022198129	6607.5	7.550408E-01
Gdf11	6464	0.11941585	13620	9.213496E-02
Gdf15	6465	-0.027500697	4014	6.990864E-01
Gdf2	6466	0.052728522	8550.5	4.583686E-01
Gdf3	6467	-0.027942038	3999	6.944968E-01
Gdf5	6468		#N/A	1.000000E+00
Gdf6	6469	-0.054451513	1703	4.437940E-01
Gdf7	6470		#N/A	1.000000E+00
Gdf9	6471	-0.038406171	3065	5.892384E-01
Gdi1	6472	0.006268346	5608	9.298027E-01
Gdi2	6473	0.194641965	18031	5.747239E-03
Gdnf	6474		#N/A	1.000000E+00
Gdpd1	6475	-0.044872671	2339	5.280826E-01
Gdpd2	6476	-0.090091982	416	2.045522E-01
Gdpd3	6477	-0.034077311	3735	6.319071E-01
Gdpd4	6478	0.149003884	15592	3.522219E-02
Gdpd5	6479	-0.027436468	4018	6.997553E-01
Gdpgp1	6480	-0.053516446	2131	4.516708E-01
Gem	6481	0.013160523	5939	8.532609E-01
Gemin2	6482	-0.124203487	91	7.972497E-02
Gemin4	6483		#N/A	1.000000E+00
Gemin5	6484	-0.066683974	1284	3.481481E-01
Gemin6	6485	0.045863277	7993	5.190058E-01
Gemin7	6486	0.103737797	12280	1.437891E-01
Gemin8	6487	-0.009948652	4746	8.888048E-01
Gen1	6488	-0.059612595	1404	4.017437E-01
Get4	6489	0.057343702	9054	4.199297E-01
Gfap	6490		#N/A	1.000000E+00
Gfer	6491	-0.046406568	2275	5.140619E-01
Gfi1	6492		#N/A	1.000000E+00

Spearman Rank correlation analysis performed between Prdm1 and all-expressed genes within the Meredith RNA-seq dataset. Robust Prdm1–associated genes were identified using a cut-off of $p < 0.0005$.

Gfi1b	6493	0.170796633	16966	1.560248E-02
Gfm1	6494	0.127968668	14187	7.094357E-02
Gfm2	6495	0.110093762	12723	1.206806E-01
Gfod1	6496	0.305270012	19922	1.105237E-05
Gfod2	6497	-0.006541487	4864	9.267523E-01
Gfpt1	6498	0.102138085	12188	1.501043E-01
Gfpt2	6499	0.005994889	5597	9.328578E-01
Gfra1	6500	-0.058460897	1426	4.109157E-01
Gfra2	6501	0.0762063	10361	2.834843E-01
Gfra3	6502	0.046388389	8039	5.142269E-01
Gfra4	6503	0.07642123	10374	2.821246E-01
Gfral	6504	-0.038406171	3065	5.892384E-01
Gga1	6505	0.187362067	17734	7.891906E-03
Gga2	6506	0.204969663	18425	3.596795E-03
Gga3	6507	0.036283768	7394	6.099943E-01
Ggact	6508	0.185706995	17667	8.469088E-03
Ggct	6509	0.136500928	14724	5.393658E-02
Ggcx	6510	0.109076279	12630	1.241725E-01
Ggh	6511	0.066704186	9631	3.480017E-01
Ggn	6512	-0.038406171	3065	5.892384E-01
Ggnbp1	6513	0.154774761	15969	2.864560E-02
Ggnbp2	6514	0.201794921	18317	4.164023E-03
Ggps1	6515	0.041488151	7710	5.596882E-01
Ggt1	6516		#N/A	1.000000E+00
Ggt5	6517	0.18053009	17432	1.052362E-02
Ggt6	6518	-0.066856562	1139	3.468991E-01
Ggt7	6519	0.001131589	5368.5	9.873120E-01
Ggta1	6520	-0.006052621	4878	9.322127E-01
Gh	6521	-0.095474596	310	1.786816E-01
Ghdc	6522	0.008971529	5728	8.996656E-01
Ghitm	6523	0.190687762	17868	6.836770E-03
Ghr	6524	0.025198531	6837	7.232013E-01
Ghrh	6525	-0.055603591	1504	4.341970E-01
Ghrhr	6526	0.053805701	8820	4.492258E-01
Ghrl	6527		#N/A	1.000000E+00
Ghsr	6528	-0.034367378	3730	6.290074E-01
Gid4	6529	0.188758216	17787	7.432492E-03
Gid8	6530	0.04008464	7643	5.730565E-01
Gif	6531	0.176630984	17249	1.234990E-02
Gigyf1	6532	0.124836729	13958	7.818960E-02
Gigyf2	6533	0.080288157	10618	2.584111E-01
Gimap1	6534	0.127207191	14129	7.265300E-02
Gimap3	6535	0.021100114	6440.5	7.667999E-01

Spearman Rank correlation analysis performed between Prdm1 and all-expressed genes within the Meredith RNA-seq dataset. Robust Prdm1–associated genes were identified using a cut-off of $p < 0.0005$.

Table S1, Related to Supplemental Figure 3C. Prdm1 associated genes

Gimap4	6536	0.082965747	10831	2.428176E-01
Gimap5	6537	0.09320178	11596	1.892943E-01
Gimap6	6538	0.277457398	19776	6.960741E-05
Gimap7	6539		#N/A	1.000000E+00
Gimap8	6540	0.036149801	7388	6.113151E-01
Gimap9	6541	-0.020341626	4228	7.749544E-01
Gin1	6542	0.111541462	12822	1.158444E-01
Ginm1	6543	0.171415064	16991	1.522547E-02
Gins1	6544	0.176019984	17222	1.266001E-02
Gins2	6545	-0.055112337	1513	4.382746E-01
Gins3	6546	0.097901945	11908	1.678394E-01
Gins4	6547	-0.070178981	947	3.234084E-01
Gip	6548	-0.032445878	3830.5	6.483195E-01
Gipc1	6549	0.010000157	5771	8.882328E-01
Gipc2	6550	0.128979516	14237	6.872505E-02
Gipc3	6551		#N/A	1.000000E+00
Gipr	6552	0.021317126	6513.5	7.644715E-01
Git1	6553	-0.105036413	208	1.388129E-01
Git2	6554	0.129285606	14260	6.806454E-02
Gja1	6555	0.086939039	11122	2.209084E-01
Gja10	6556		#N/A	1.000000E+00
Gja3	6557	-0.000841282	5208.5	9.905669E-01
Gja4	6558	0.191568442	17896	6.579312E-03
Gja5	6559	0.244371692	19430	4.879272E-04
Gja6	6560		#N/A	1.000000E+00
Gja8	6561		#N/A	1.000000E+00
Gjb1	6562		#N/A	1.000000E+00
Gjb2	6563	0.025804085	6861	7.168306E-01
Gjb3	6564	0.248683456	19492	3.840364E-04
Gjb4	6565		#N/A	1.000000E+00
Gjb5	6566		#N/A	1.000000E+00
Gjb6	6567	-0.043445382	2393	5.413003E-01
Gjc1	6568	0.099023315	12000	1.629994E-01
Gjc2	6569	0.052728522	8550.5	4.583686E-01
Gjc3	6570	0.053521971	8774	4.516240E-01
Gjd2	6571	0.14001476	14956.5	4.799102E-02
Gjd3	6572		#N/A	1.000000E+00
Gjd4	6573		#N/A	1.000000E+00
Gje1	6574	0.100386359	12085	1.572579E-01
Gk2	6575		#N/A	1.000000E+00
Gk5	6576	0.122303309	13803	8.447858E-02
Gkap1	6577	0.064098701	9470	3.671959E-01
Gkn1	6578		#N/A	1.000000E+00

Spearman Rank correlation analysis performed between Prdm1 and all-expressed genes within the Meredith RNA-seq dataset. Robust Prdm1–associated genes were identified using a cut-off of $p < 0.0005$.

Table S1, Related to Supplemental Figure 3C. Prdm1 associated genes

Gkn2	6579	-0.00045847	5257	9.948592E-01
Gkn3	6580		#N/A	1.000000E+00
Gla	6581	0.179485703	17382	1.098779E-02
Glb1	6582	0.061226111	9273	3.891009E-01
Glb1l	6583	0.014200371	5999	8.418131E-01
Glb1l2	6584	-0.037145338	3655	6.015297E-01
Glb1l3	6585	-0.103180568	220	1.459655E-01
Glcci1	6586	0.300580446	19909	1.527848E-05
Glce	6587	0.086059816	11058	2.256310E-01
Gldc	6588	0.129441896	14271	6.772929E-02
Gldn	6589	0.138395448	14849	5.065935E-02
Gle1	6590	0.094455464	11671	1.833847E-01
Glg1	6591	0.109643053	12687	1.222179E-01
Gli1	6592		#N/A	1.000000E+00
Gli2	6593	0.0928823	11572	1.908223E-01
Gli3	6594	0.223544482	19004	1.462895E-03
Glipr1	6595	0.160951581	16387	2.279839E-02
Glipr1l1	6596	0.053267784	8685	4.537786E-01
Glipr1l2	6597	-0.001606757	5107.5	9.819849E-01
Glipr2	6598	0.12667846	14082	7.385950E-02
Glis1	6599	0.049136369	8225	4.895938E-01
Glis2	6600	0.077085587	10417	2.779496E-01
Glis3	6601	0.029583403	7045	6.775262E-01
Glmn	6602	0.014540589	6045	8.380746E-01
Glns-ps1	6603	-0.038406171	3065	5.892384E-01
Glo1	6604	0.076529381	10383	2.814421E-01
Glod4	6605	0.075776661	10329	2.862154E-01
Glod5	6606	-0.086747139	513	2.219331E-01
Glp1r	6607	0.18218536	17494	9.823364E-03
Glp2r	6608	0.203933334	18385	3.773781E-03
Glra1	6609	0.076358508	10371	2.825209E-01
Glra2	6610	0.099892398	12057	1.593208E-01
Glra3	6611	0.134200998	14569.5	5.814916E-02
Glra4	6612	-0.054450825	1984	4.437997E-01
Glrb	6613	-0.089078668	428	2.097106E-01
Glrp1	6614	0.022714618	6671	7.495287E-01
Glrx	6615	0.148464423	15560	3.589764E-02
Glrx2	6616	0.233490965	19241	8.763602E-04
Glrx3	6617	0.141859698	15074	4.509478E-02
Glrx5	6618	0.036094869	7387	6.118571E-01
Gls	6619	0.126228734	14058	7.489847E-02
Gls2	6620	0.045443441	7963	5.228429E-01
Glt1d1	6621	0.177103168	17271	1.211483E-02

Spearman Rank correlation analysis performed between Prdm1 and all-expressed genes within the Meredith RNA-seq dataset. Robust Prdm1–associated genes were identified using a cut-off of $p < 0.0005$.

Table S1, Related to Supplemental Figure 3C. Prdm1 associated genes

Glt28d2	6622		#N/A	1.000000E+00
Glt6d1	6623	0.020875719	6412.5	7.692098E-01
Glt8d1	6624	0.079426076	10554	2.635751E-01
Glt8d2	6625	0.125864883	14030	7.574770E-02
Gltp	6626	0.118563372	13561	9.449819E-02
Gltpd1	6627	0.026447933	6899	7.100784E-01
Gltpd2	6628		#N/A	1.000000E+00
Gltscr1	6629	0.195730002	18073	5.476072E-03
Gltscr1l	6630	0.150648284	15689	3.322932E-02
Gltscr2	6631	0.193412372	17964	6.068098E-03
Glud1	6632	0.194512826	18025	5.780209E-03
Glul	6633	0.018275922	6237	7.972853E-01
Glyat	6634	0.113548679	13094	1.093908E-01
Glyatl3	6635		#N/A	1.000000E+00
Glycam1	6636	-0.064332465	1337.5	3.654475E-01
Glyctk	6637	-0.072292286	926	3.090151E-01
Glyr1	6638	0.112640571	12898	1.122747E-01
Gm10000	6639	0.049839576	8278	4.833930E-01
Gm10006	6640		#N/A	1.000000E+00
Gm10010	6641	-0.038406171	3065	5.892384E-01
Gm10011	6642		#N/A	1.000000E+00
Gm10012	6643		#N/A	1.000000E+00
Gm10013	6644		#N/A	1.000000E+00
Gm10015	6645		#N/A	1.000000E+00
Gm10020	6646		#N/A	1.000000E+00
Gm10024	6647		#N/A	1.000000E+00
Gm10025	6648		#N/A	1.000000E+00
Gm10029	6649	0.11394017	13251	1.081656E-01
Gm10030	6650		#N/A	1.000000E+00
Gm10031	6651		#N/A	1.000000E+00
Gm10032	6652		#N/A	1.000000E+00
Gm10033	6653	0.113548679	13094	1.093908E-01
Gm10036	6654	0.07877506	10515	2.675214E-01
Gm10037	6655	0.035940058	7378.5	6.133856E-01
Gm10038	6656		#N/A	1.000000E+00
Gm10039	6657	0.001876253	5414	9.789639E-01
Gm10040	6658		#N/A	1.000000E+00
Gm10042	6659		#N/A	1.000000E+00
Gm10044	6660	-0.031894347	3852	6.539071E-01
Gm10045	6661		#N/A	1.000000E+00
Gm10047	6662		#N/A	1.000000E+00
Gm10048	6663		#N/A	1.000000E+00
Gm10051	6664	0.083008808	10832	2.425723E-01

Spearman Rank correlation analysis performed between Prdm1 and all-expressed genes within the Meredith RNA-seq dataset. Robust Prdm1–associated genes were identified using a cut-off of $p < 0.0005$.

Table S1, Related to Supplemental Figure 3C. Prdm1 associated genes

Gm10052	6665		#N/A	1.000000E+00
Gm10053	6666		#N/A	1.000000E+00
Gm10055	6667	-0.038406171	3065	5.892384E-01
Gm10056	6668		#N/A	1.000000E+00
Gm10057	6669		#N/A	1.000000E+00
Gm10058	6670	0.113548679	13094	1.093908E-01
Gm10059	6671	0.058035813	9088	4.143319E-01
Gm10061	6672		#N/A	1.000000E+00
Gm10062	6673		#N/A	1.000000E+00
Gm10064	6674	0.188804702	17792.5	7.417613E-03
Gm10065	6675		#N/A	1.000000E+00
Gm10067	6676		#N/A	1.000000E+00
Gm10068	6677	0.153028855	15843.5	3.051443E-02
Gm10069	6678	0.019843878	6328	7.803191E-01
Gm10071	6679	-0.054451513	1703	4.437940E-01
Gm10073	6680	0.052728522	8550.5	4.583686E-01
Gm10074	6681	-0.038406171	3065	5.892384E-01
Gm10075	6682		#N/A	1.000000E+00
Gm10076	6683	0.175814976	17207	1.276558E-02
Gm10080	6684		#N/A	1.000000E+00
Gm10081	6685		#N/A	1.000000E+00
Gm10083	6686		#N/A	1.000000E+00
Gm10086	6687	0.014915162	6067	8.339629E-01
Gm10087	6688		#N/A	1.000000E+00
Gm10088	6689		#N/A	1.000000E+00
Gm10092	6690	0.187798503	17756	7.745652E-03
Gm10093	6691	0.104913529	12350	1.392781E-01
Gm10094	6692	0.068888425	9765	3.324080E-01
Gm10096	6693		#N/A	1.000000E+00
Gm10097	6694		#N/A	1.000000E+00
Gm101	6695	-0.001606757	5107.5	9.819849E-01
Gm10100	6696		#N/A	1.000000E+00
Gm10101	6697		#N/A	1.000000E+00
Gm10104	6698		#N/A	1.000000E+00
Gm10105	6699		#N/A	1.000000E+00
Gm10108	6700		#N/A	1.000000E+00
Gm10109	6701		#N/A	1.000000E+00
Gm10110	6702	-0.005060844	4931	9.432999E-01
Gm10112	6703		#N/A	1.000000E+00
Gm10113	6704		#N/A	1.000000E+00
Gm10115	6705		#N/A	1.000000E+00
Gm10116	6706	0.081085405	10671.5	2.536978E-01
Gm10118	6707	-0.054451513	1703	4.437940E-01

Spearman Rank correlation analysis performed between Prdm1 and all-expressed genes within the Meredith RNA-seq dataset. Robust Prdm1–associated genes were identified using a cut-off of $p < 0.0005$.

Table S1, Related to Supplemental Figure 3C. Prdm1 associated genes

Gm10123	6708	-0.00418459	4978	9.531047E-01
Gm10125	6709	0.177740176	17300	1.180396E-02
Gm10126	6710	-0.086755146	452	2.218903E-01
Gm10128	6711		#N/A	1.000000E+00
Gm10129	6712		#N/A	1.000000E+00
Gm10130	6713	0.013842768	5982.5	8.457464E-01
Gm10131	6714		#N/A	1.000000E+00
Gm10132	6715	-0.038406171	3065	5.892384E-01
Gm10134	6716		#N/A	1.000000E+00
Gm10135	6717	0.052728522	8550.5	4.583686E-01
Gm10136	6718	0.082269134	10768	2.468098E-01
Gm10138	6719		#N/A	1.000000E+00
Gm10139	6720		#N/A	1.000000E+00
Gm10142	6721		#N/A	1.000000E+00
Gm10143	6722	0.216566415	18804	2.068949E-03
Gm10145	6723		#N/A	1.000000E+00
Gm10146	6724		#N/A	1.000000E+00
Gm10147	6725		#N/A	1.000000E+00
Gm10152	6726	-0.038406171	3065	5.892384E-01
Gm10153	6727		#N/A	1.000000E+00
Gm10154	6728		#N/A	1.000000E+00
Gm10155	6729	0.194131481	17998	5.878558E-03
Gm10156	6730		#N/A	1.000000E+00
Gm10157	6731		#N/A	1.000000E+00
Gm10160	6732		#N/A	1.000000E+00
Gm10161	6733	-0.054451513	1703	4.437940E-01
Gm10163	6734	-0.054451513	1703	4.437940E-01
Gm10166	6735	-0.038406171	3065	5.892384E-01
Gm10167	6736	0.065215125	9548	3.588926E-01
Gm10169	6737	0.071777786	9979.5	3.124800E-01
Gm10171	6738		#N/A	1.000000E+00
Gm10172	6739		#N/A	1.000000E+00
Gm10175	6740	0.14618614	15425	3.887238E-02
Gm10176	6741		#N/A	1.000000E+00
Gm10177	6742	0.126800162	14094	7.358036E-02
Gm10179	6743		#N/A	1.000000E+00
Gm10180	6744	0.105103187	12363	1.385607E-01
Gm10181	6745		#N/A	1.000000E+00
Gm10182	6746		#N/A	1.000000E+00
Gm10184	6747		#N/A	1.000000E+00
Gm10186	6748	0.105244186	12375	1.380291E-01
Gm10188	6749		#N/A	1.000000E+00
Gm10190	6750		#N/A	1.000000E+00

Spearman Rank correlation analysis performed between Prdm1 and all-expressed genes within the Meredith RNA-seq dataset. Robust Prdm1–associated genes were identified using a cut-off of $p < 0.0005$.

Table S1, Related to Supplemental Figure 3C. Prdm1 associated genes

Gm10192	6751		#N/A	1.000000E+00
Gm10197	6752		#N/A	1.000000E+00
Gm10198	6753		#N/A	1.000000E+00
Gm10203	6754		#N/A	1.000000E+00
Gm10205	6755		#N/A	1.000000E+00
Gm10212	6756		#N/A	1.000000E+00
Gm10217	6757		#N/A	1.000000E+00
Gm10220	6758		#N/A	1.000000E+00
Gm10221	6759	0.160268395	16289	2.338993E-02
Gm10222	6760		#N/A	1.000000E+00
Gm10224	6761		#N/A	1.000000E+00
Gm10226	6762		#N/A	1.000000E+00
Gm10228	6763		#N/A	1.000000E+00
Gm10229	6764	0.21549506	18774.5	2.180011E-03
Gm10230	6765		#N/A	1.000000E+00
Gm10231	6766		#N/A	1.000000E+00
Gm10232	6767	0.038758411	7558.5	5.858252E-01
Gm10233	6768		#N/A	1.000000E+00
Gm10234	6769		#N/A	1.000000E+00
Gm10237	6770	-0.038406171	3065	5.892384E-01
Gm10238	6771		#N/A	1.000000E+00
Gm10240	6772		#N/A	1.000000E+00
Gm10241	6773		#N/A	1.000000E+00
Gm10242	6774		#N/A	1.000000E+00
Gm10243	6775	0.053267784	8685	4.537786E-01
Gm10244	6776	-0.04310292	2408	5.444960E-01
Gm10247	6777		#N/A	1.000000E+00
Gm10248	6778	0.109772198	12698	1.217758E-01
Gm10250	6779	0.29815023	19895	1.803036E-05
Gm10251	6780		#N/A	1.000000E+00
Gm10253	6781		#N/A	1.000000E+00
Gm10254	6782		#N/A	1.000000E+00
Gm10256	6783		#N/A	1.000000E+00
Gm10257	6784		#N/A	1.000000E+00
Gm10258	6785		#N/A	1.000000E+00
Gm10259	6786		#N/A	1.000000E+00
Gm10260	6787	0.096064254	11779	1.760014E-01
Gm10263	6788	0.168119368	16830	1.733019E-02
Gm10264	6789		#N/A	1.000000E+00
Gm10267	6790		#N/A	1.000000E+00
Gm10268	6791		#N/A	1.000000E+00
Gm10269	6792	0.134200998	14569.5	5.814916E-02
Gm10271	6793	-0.045149205	2323	5.255407E-01

Spearman Rank correlation analysis performed between Prdm1 and all-expressed genes within the Meredith RNA-seq dataset. Robust Prdm1–associated genes were identified using a cut-off of $p < 0.0005$.

Table S1, Related to Supplemental Figure 3C. Prdm1 associated genes

Gm10272	6794		#N/A	1.000000E+00
Gm10273	6795	-0.054451513	1703	4.437940E-01
Gm10275	6796		#N/A	1.000000E+00
Gm10277	6797		#N/A	1.000000E+00
Gm10282	6798	0.050870414	8368.5	4.743803E-01
Gm10283	6799	0.113548679	13094	1.093908E-01
Gm10284	6800		#N/A	1.000000E+00
Gm10286	6801		#N/A	1.000000E+00
Gm10287	6802		#N/A	1.000000E+00
Gm10288	6803	0.061962843	9329	3.834090E-01
Gm10291	6804		#N/A	1.000000E+00
Gm10293	6805		#N/A	1.000000E+00
Gm10294	6806	-0.038406171	3065	5.892384E-01
Gm10295	6807		#N/A	1.000000E+00
Gm10298	6808		#N/A	1.000000E+00
Gm10300	6809		#N/A	1.000000E+00
Gm10302	6810		#N/A	1.000000E+00
Gm10304	6811		#N/A	1.000000E+00
Gm10305	6812		#N/A	1.000000E+00
Gm10306	6813		#N/A	1.000000E+00
Gm10307	6814	0.175914003	17215	1.271449E-02
Gm10308	6815		#N/A	1.000000E+00
Gm10309	6816	-0.038406171	3065	5.892384E-01
Gm10310	6817		#N/A	1.000000E+00
Gm10311	6818	0.215438595	18765	2.186012E-03
Gm10312	6819	-0.038406171	3065	5.892384E-01
Gm10313	6820	-0.038406171	3065	5.892384E-01
Gm10318	6821		#N/A	1.000000E+00
Gm10319	6822	0.086412184	11087.5	2.237298E-01
Gm10320	6823	0.053267784	8685	4.537786E-01
Gm10322	6824		#N/A	1.000000E+00
Gm10323	6825		#N/A	1.000000E+00
Gm10324	6826	0.13222823	14427	6.197532E-02
Gm10327	6827		#N/A	1.000000E+00
Gm10330	6828		#N/A	1.000000E+00
Gm10334	6829		#N/A	1.000000E+00
Gm10335	6830	0.238698508	19341	6.643463E-04
Gm10337	6831		#N/A	1.000000E+00
Gm10338	6832		#N/A	1.000000E+00
Gm10339	6833	-0.038406171	3065	5.892384E-01
Gm10340	6834		#N/A	1.000000E+00
Gm10343	6835	0.03037917	7087.5	6.693560E-01
Gm10344	6836	0.215438595	18765	2.186012E-03

Spearman Rank correlation analysis performed between Prdm1 and all-expressed genes within the Meredith RNA-seq dataset. Robust Prdm1–associated genes were identified using a cut-off of $p < 0.0005$.

Table S1, Related to Supplemental Figure 3C. Prdm1 associated genes

Gm10349	6837		#N/A	1.000000E+00
Gm10351	6838		#N/A	1.000000E+00
Gm10352	6839		#N/A	1.000000E+00
Gm10354	6840	0.113548679	13094	1.093908E-01
Gm10355	6841		#N/A	1.000000E+00
Gm10356	6842		#N/A	1.000000E+00
Gm10357	6843		#N/A	1.000000E+00
Gm10358	6844		#N/A	1.000000E+00
Gm10359	6845		#N/A	1.000000E+00
Gm10360	6846		#N/A	1.000000E+00
Gm10364	6847	-0.086755146	452	2.218903E-01
Gm10369	6848		#N/A	1.000000E+00
Gm10371	6849	0.145902217	15405	3.925726E-02
Gm10373	6850	0.069206068	9794	3.301782E-01
Gm10375	6851		#N/A	1.000000E+00
Gm10376	6852		#N/A	1.000000E+00
Gm10377	6853		#N/A	1.000000E+00
Gm10378	6854		#N/A	1.000000E+00
Gm10382	6855	-0.086747139	513	2.219331E-01
Gm10384	6856		#N/A	1.000000E+00
Gm10392	6857		#N/A	1.000000E+00
Gm10396	6858		#N/A	1.000000E+00
Gm10398	6859		#N/A	1.000000E+00
Gm10399	6860		#N/A	1.000000E+00
Gm10401	6861	-0.086755146	452	2.218903E-01
Gm10406	6862	-0.005759909	4889	9.354837E-01
Gm10408	6863		#N/A	1.000000E+00
Gm10409	6864		#N/A	1.000000E+00
Gm10410	6865	-0.038406171	3065	5.892384E-01
Gm10413	6866		#N/A	1.000000E+00
Gm10417	6867		#N/A	1.000000E+00
Gm10418	6868		#N/A	1.000000E+00
Gm10419	6869	0.143605682	15224	4.249002E-02
Gm10420	6870	-0.095269893	339	1.796190E-01
Gm10424	6871		#N/A	1.000000E+00
Gm10428	6872		#N/A	1.000000E+00
Gm1043	6873	-0.038406171	3065	5.892384E-01
Gm10430	6874		#N/A	1.000000E+00
Gm10433	6875	0.021757707	6579.5	7.597509E-01
Gm10434	6876		#N/A	1.000000E+00
Gm10435	6877		#N/A	1.000000E+00
Gm10436	6878	-0.038406171	3065	5.892384E-01
Gm10439	6879		#N/A	1.000000E+00

Spearman Rank correlation analysis performed between Prdm1 and all-expressed genes within the Meredith RNA-seq dataset. Robust Prdm1–associated genes were identified using a cut-off of $p < 0.0005$.

Table S1, Related to Supplemental Figure 3C. Prdm1 associated genes

Gm10443	6880		#N/A	1.000000E+00
Gm10444	6881		#N/A	1.000000E+00
Gm10447	6882		#N/A	1.000000E+00
Gm1045	6883	0.156129434	16056.5	2.726377E-02
Gm10451	6884	0.232542596	19220	9.211017E-04
Gm10462	6885		#N/A	1.000000E+00
Gm10463	6886		#N/A	1.000000E+00
Gm10466	6887	0.113548679	13094	1.093908E-01
Gm10471	6888	-0.066855996	1239.5	3.469032E-01
Gm10472	6889		#N/A	1.000000E+00
Gm10475	6890	0.109492511	12666.5	1.227347E-01
Gm10476	6891	-0.066858259	1029.5	3.468868E-01
Gm10477	6892		#N/A	1.000000E+00
Gm10478	6893		#N/A	1.000000E+00
Gm10479	6894		#N/A	1.000000E+00
Gm10480	6895		#N/A	1.000000E+00
Gm10481	6896		#N/A	1.000000E+00
Gm10482	6897		#N/A	1.000000E+00
Gm10483	6898		#N/A	1.000000E+00
Gm10485	6899	0.186433927	17699	8.211120E-03
Gm10486	6900		#N/A	1.000000E+00
Gm10487	6901		#N/A	1.000000E+00
Gm10488	6902		#N/A	1.000000E+00
Gm10490	6903		#N/A	1.000000E+00
Gm10491	6904		#N/A	1.000000E+00
Gm10493	6905		#N/A	1.000000E+00
Gm10497	6906	0.119182415	13598	9.277735E-02
Gm10499	6907	-0.038406171	3065	5.892384E-01
Gm10501	6908	0.111240562	12805	1.168369E-01
Gm10505	6909	-0.086746694	532	2.219355E-01
Gm10509	6910		#N/A	1.000000E+00
Gm10517	6911		#N/A	1.000000E+00
Gm10518	6912	-0.066858259	1029.5	3.468868E-01
Gm10521	6913		#N/A	1.000000E+00
Gm10530	6914	0.046578968	8051	5.124982E-01
Gm10540	6915		#N/A	1.000000E+00
Gm10542	6916		#N/A	1.000000E+00
Gm10543	6917		#N/A	1.000000E+00
Gm10545	6918		#N/A	1.000000E+00
Gm10548	6919	0.176431821	17242	1.245024E-02
Gm10549	6920		#N/A	1.000000E+00
Gm10552	6921	0.086835521	11120	2.214607E-01
Gm10553	6922		#N/A	1.000000E+00

Spearman Rank correlation analysis performed between Prdm1 and all-expressed genes within the Meredith RNA-seq dataset. Robust Prdm1–associated genes were identified using a cut-off of $p < 0.0005$.

Table S1, Related to Supplemental Figure 3C. Prdm1 associated genes

Gm10557	6923	0.046025613	8011	5.175260E-01
Gm10558	6924		#N/A	1.000000E+00
Gm10562	6925		#N/A	1.000000E+00
Gm10563	6926	0.013458335	5965.5	8.499790E-01
Gm10564	6927		#N/A	1.000000E+00
Gm10566	6928		#N/A	1.000000E+00
Gm10567	6929	-0.038406171	3065	5.892384E-01
Gm10570	6930		#N/A	1.000000E+00
Gm10571	6931		#N/A	1.000000E+00
Gm10574	6932	0.272127167	19736	9.692657E-05
Gm10575	6933	0.056660782	9020	4.254961E-01
Gm10576	6934	0.143605682	15224	4.249002E-02
Gm10577	6935		#N/A	1.000000E+00
Gm10580	6936		#N/A	1.000000E+00
Gm10581	6937		#N/A	1.000000E+00
Gm10582	6938		#N/A	1.000000E+00
Gm10583	6939		#N/A	1.000000E+00
Gm10586	6940		#N/A	1.000000E+00
Gm10590	6941		#N/A	1.000000E+00
Gm10591	6942		#N/A	1.000000E+00
Gm10592	6943		#N/A	1.000000E+00
Gm10593	6944		#N/A	1.000000E+00
Gm10595	6945		#N/A	1.000000E+00
GM10597	6946		#N/A	1.000000E+00
Gm10597	6947		#N/A	1.000000E+00
Gm10600	6948		#N/A	1.000000E+00
Gm10601	6949		#N/A	1.000000E+00
Gm10602	6950		#N/A	1.000000E+00
Gm10604	6951		#N/A	1.000000E+00
Gm10608	6952		#N/A	1.000000E+00
Gm10610	6953	0.021316405	6469	7.644793E-01
Gm10612	6954		#N/A	1.000000E+00
Gm10615	6955	-0.054450825	1984	4.437997E-01
Gm10617	6956	0.032562904	7210	6.471364E-01
Gm10619	6957		#N/A	1.000000E+00
Gm10620	6958		#N/A	1.000000E+00
Gm10621	6959	-0.054450825	1984	4.437997E-01
Gm10629	6960	0.134018302	14553	5.849511E-02
Gm10631	6961		#N/A	1.000000E+00
Gm10634	6962		#N/A	1.000000E+00
Gm10638	6963	0.113548679	13094	1.093908E-01
Gm10639	6964	-0.053971255	2119	4.478298E-01
Gm10642	6965		#N/A	1.000000E+00

Spearman Rank correlation analysis performed between Prdm1 and all-expressed genes within the Meredith RNA-seq dataset. Robust Prdm1–associated genes were identified using a cut-off of $p < 0.0005$.

Table S1, Related to Supplemental Figure 3C. Prdm1 associated genes

Gm10643	6966	0.082762295	10811.5	2.439788E-01
Gm10644	6967	-0.066856562	1139	3.468991E-01
Gm10645	6968	0.162808768	16526.5	2.125512E-02
Gm10647	6969		#N/A	1.000000E+00
Gm10649	6970	0.01016948	5779	8.863530E-01
Gm10650	6971		#N/A	1.000000E+00
Gm10651	6972		#N/A	1.000000E+00
Gm10653	6973	0.144855397	15347	4.070418E-02
Gm10654	6974		#N/A	1.000000E+00
Gm10655	6975		#N/A	1.000000E+00
Gm10657	6976	-0.066856562	1139	3.468991E-01
Gm10658	6977	0.051686351	8448	4.673119E-01
Gm10660	6978	-0.038406171	3065	5.892384E-01
Gm10662	6979		#N/A	1.000000E+00
Gm10663	6980		#N/A	1.000000E+00
Gm10664	6981	0.100892498	12113	1.551651E-01
Gm10665	6982		#N/A	1.000000E+00
Gm10666	6983		#N/A	1.000000E+00
Gm10668	6984		#N/A	1.000000E+00
Gm10670	6985		#N/A	1.000000E+00
Gm10676	6986		#N/A	1.000000E+00
Gm10678	6987		#N/A	1.000000E+00
Gm10679	6988		#N/A	1.000000E+00
Gm10680	6989	-0.054450825	1984	4.437997E-01
Gm10681	6990	0.074574898	10201.5	2.939481E-01
Gm10684	6991	0.288785172	19858	3.366474E-05
Gm10689	6992	0.018928849	6275	7.902082E-01
Gm10691	6993	-0.066858259	1029.5	3.468868E-01
Gm10693	6994		#N/A	1.000000E+00
Gm10696	6995	-0.000458472	5251.5	9.948592E-01
Gm10697	6996		#N/A	1.000000E+00
Gm10698	6997		#N/A	1.000000E+00
Gm10699	6998		#N/A	1.000000E+00
Gm1070	6999		#N/A	1.000000E+00
Gm10701	7000	0.055649073	8963	4.338206E-01
Gm10702	7001		#N/A	1.000000E+00
Gm10704	7002	0.082597991	10793	2.449195E-01
Gm10705	7003		#N/A	1.000000E+00
Gm10706	7004	-0.038406171	3065	5.892384E-01
Gm10709	7005		#N/A	1.000000E+00
Gm10710	7006		#N/A	1.000000E+00
Gm10711	7007		#N/A	1.000000E+00
Gm10712	7008		#N/A	1.000000E+00

Spearman Rank correlation analysis performed between Prdm1 and all-expressed genes within the Meredith RNA-seq dataset. Robust Prdm1–associated genes were identified using a cut-off of $p < 0.0005$.

Gm10713	7009	-0.095280123	319	1.795721E-01
Gm10714	7010	-0.066858259	1029.5	3.468868E-01
Gm10715	7011	-0.017417585	4387	8.066140E-01
Gm10717	7012	0.123000325	13842	8.270911E-02
Gm10718	7013	-0.066409866	1292.5	3.501376E-01
Gm10719	7014	0.025659071	6855	7.183544E-01
Gm10720	7015	0.091544822	11492	1.973170E-01
Gm10721	7016	-0.054451513	1703	4.437940E-01
Gm10722	7017	-0.018105422	4347	7.991361E-01
Gm10728	7018		#N/A	1.000000E+00
Gm10729	7019		#N/A	1.000000E+00
Gm10731	7020	-0.038406171	3065	5.892384E-01
Gm10735	7021		#N/A	1.000000E+00
Gm10736	7022		#N/A	1.000000E+00
Gm10737	7023		#N/A	1.000000E+00
Gm10742	7024		#N/A	1.000000E+00
Gm10748	7025		#N/A	1.000000E+00
Gm10750	7026		#N/A	1.000000E+00
Gm10754	7027		#N/A	1.000000E+00
Gm10760	7028		#N/A	1.000000E+00
Gm10762	7029	0.220658701	18921.5	1.690525E-03
Gm10764	7030		#N/A	1.000000E+00
Gm10766	7031		#N/A	1.000000E+00
Gm10767	7032	0.110628604	12767	1.188760E-01
Gm10768	7033	-0.066856562	1139	3.468991E-01
Gm10770	7034		#N/A	1.000000E+00
Gm10772	7035		#N/A	1.000000E+00
Gm10774	7036		#N/A	1.000000E+00
Gm10775	7037	-0.054450825	1984	4.437997E-01
Gm10778	7038	-0.038406171	3065	5.892384E-01
Gm1078	7039	0.127634088	14162	7.169059E-02
Gm10780	7040		#N/A	1.000000E+00
Gm10782	7041		#N/A	1.000000E+00
Gm10784	7042		#N/A	1.000000E+00
Gm10785	7043	-0.110563117	176	1.190958E-01
Gm10787	7044		#N/A	1.000000E+00
Gm10794	7045		#N/A	1.000000E+00
Gm10797	7046		#N/A	1.000000E+00
Gm10799	7047		#N/A	1.000000E+00
Gm10800	7048	0.074247553	10159	2.960783E-01
Gm10801	7049	0.139275949	14907	4.919354E-02
Gm10803	7050		#N/A	1.000000E+00
Gm10804	7051	-0.038406171	3065	5.892384E-01

Table S1, Related to Supplemental Figure 3C. Prdm1 associated genes

Gm10805	7052		#N/A	1.000000E+00
Gm10807	7053		#N/A	1.000000E+00
Gm10812	7054	-0.066855996	1239.5	3.469032E-01
Gm10813	7055		#N/A	1.000000E+00
Gm10818	7056		#N/A	1.000000E+00
Gm10819	7057		#N/A	1.000000E+00
Gm1082	7058		#N/A	1.000000E+00
Gm10837	7059		#N/A	1.000000E+00
Gm10840	7060		#N/A	1.000000E+00
Gm10842	7061		#N/A	1.000000E+00
Gm10845	7062		#N/A	1.000000E+00
Gm10849	7063		#N/A	1.000000E+00
Gm10855	7064		#N/A	1.000000E+00
Gm10858	7065		#N/A	1.000000E+00
Gm10860	7066		#N/A	1.000000E+00
Gm10863	7067	0.025142859	6832.5	7.237880E-01
Gm10874	7068		#N/A	1.000000E+00
Gm10912	7069		#N/A	1.000000E+00
Gm10913	7070	-0.038406171	3065	5.892384E-01
Gm10916	7071		#N/A	1.000000E+00
Gm10921	7072		#N/A	1.000000E+00
Gm10922	7073		#N/A	1.000000E+00
Gm10923	7074	0.053267784	8685	4.537786E-01
Gm10931	7075		#N/A	1.000000E+00
Gm10935	7076		#N/A	1.000000E+00
Gm10936	7077		#N/A	1.000000E+00
Gm10941	7078	0.003398203	5495	9.619101E-01
Gm10944	7079		#N/A	1.000000E+00
Gm10945	7080	-0.054451513	1703	4.437940E-01
Gm10959	7081	-0.054451513	1703	4.437940E-01
Gm10961	7082		#N/A	1.000000E+00
Gm10964	7083	0.183289421	17561	9.379511E-03
Gm10972	7084		#N/A	1.000000E+00
Gm10974	7085	-0.039421692	2485	5.794229E-01
Gm10985	7086		#N/A	1.000000E+00
Gm10999	7087		#N/A	1.000000E+00
Gm11007	7088		#N/A	1.000000E+00
Gm11008	7089		#N/A	1.000000E+00
Gm11009	7090		#N/A	1.000000E+00
Gm11011	7091		#N/A	1.000000E+00
Gm11020	7092		#N/A	1.000000E+00
Gm11025	7093		#N/A	1.000000E+00
Gm11027	7094		#N/A	1.000000E+00

Spearman Rank correlation analysis performed between Prdm1 and all-expressed genes within the Meredith RNA-seq dataset. Robust Prdm1–associated genes were identified using a cut-off of $p < 0.0005$.

Table S1, Related to Supplemental Figure 3C. Prdm1 associated genes

Gm11032	7095		#N/A	1.000000E+00
Gm11033	7096	-0.084830261	573	2.323546E-01
Gm11034	7097		#N/A	1.000000E+00
Gm11037	7098		#N/A	1.000000E+00
Gm11042	7099	-0.016730797	4444.5	8.140981E-01
Gm11052	7100		#N/A	1.000000E+00
Gm11060	7101		#N/A	1.000000E+00
Gm11065	7102		#N/A	1.000000E+00
Gm11077	7103	-0.038406171	3065	5.892384E-01
Gm11084	7104	0.116353988	13422	1.008478E-01
Gm11096	7105		#N/A	1.000000E+00
Gm11099	7106	0.053267784	8685	4.537786E-01
Gm1110	7107	0.159026488	16225	2.449913E-02
Gm11100	7108	-0.054450825	1984	4.437997E-01
Gm11105	7109		#N/A	1.000000E+00
Gm11107	7110		#N/A	1.000000E+00
Gm11109	7111		#N/A	1.000000E+00
Gm11110	7112	-0.081277267	627	2.525725E-01
Gm11111	7113		#N/A	1.000000E+00
Gm11114	7114		#N/A	1.000000E+00
Gm11115	7115		#N/A	1.000000E+00
Gm11116	7116		#N/A	1.000000E+00
Gm11127	7117	0.178691694	17337	1.135267E-02
Gm11128	7118	0.151345634	15738	3.241357E-02
Gm11131	7119	0.242406278	19401	5.434344E-04
Gm11143	7120		#N/A	1.000000E+00
Gm11146	7121	-0.066856562	1139	3.468991E-01
Gm11149	7122		#N/A	1.000000E+00
Gm11168	7123	0.05251099	8501	4.602274E-01
Gm11172	7124		#N/A	1.000000E+00
Gm11175	7125		#N/A	1.000000E+00
Gm11184	7126	0.022961479	6693	7.468986E-01
Gm11185	7127		#N/A	1.000000E+00
Gm11186	7128		#N/A	1.000000E+00
Gm11187	7129		#N/A	1.000000E+00
Gm11188	7130		#N/A	1.000000E+00
Gm11189	7131		#N/A	1.000000E+00
Gm11190	7132	0.053805701	8820	4.492258E-01
Gm11191	7133	-0.054451513	1703	4.437940E-01
Gm11192	7134		#N/A	1.000000E+00
Gm11193	7135		#N/A	1.000000E+00
Gm11194	7136	0.153926051	15885	2.954147E-02
Gm11195	7137		#N/A	1.000000E+00

Spearman Rank correlation analysis performed between Prdm1 and all-expressed genes within the Meredith RNA-seq dataset. Robust Prdm1–associated genes were identified using a cut-off of $p < 0.0005$.

Table S1, Related to Supplemental Figure 3C. Prdm1 associated genes

Gm11196	7138		#N/A	1.000000E+00
Gm11197	7139		#N/A	1.000000E+00
Gm11198	7140	-0.038406171	3065	5.892384E-01
Gm11199	7141		#N/A	1.000000E+00
Gm11200	7142	0.164478601	16623.5	1.994547E-02
Gm11201	7143	-0.038406171	3065	5.892384E-01
Gm11202	7144	-0.001224019	5151	9.862757E-01
Gm11203	7145		#N/A	1.000000E+00
Gm11204	7146		#N/A	1.000000E+00
Gm11205	7147	-0.110568513	165.5	1.190777E-01
Gm11206	7148		#N/A	1.000000E+00
Gm11207	7149		#N/A	1.000000E+00
Gm11209	7150		#N/A	1.000000E+00
Gm11210	7151		#N/A	1.000000E+00
Gm11211	7152		#N/A	1.000000E+00
Gm11212	7153		#N/A	1.000000E+00
Gm11213	7154	-0.038406171	3065	5.892384E-01
Gm11216	7155		#N/A	1.000000E+00
Gm11217	7156	0.178178847	17315	1.159398E-02
Gm11218	7157		#N/A	1.000000E+00
Gm11219	7158		#N/A	1.000000E+00
Gm11220	7159		#N/A	1.000000E+00
Gm11221	7160		#N/A	1.000000E+00
Gm11222	7161		#N/A	1.000000E+00
Gm11223	7162	0.057825313	9074	4.160298E-01
Gm11224	7163		#N/A	1.000000E+00
Gm11225	7164		#N/A	1.000000E+00
Gm11226	7165		#N/A	1.000000E+00
Gm11227	7166		#N/A	1.000000E+00
Gm11228	7167	0.035918913	7376	6.135946E-01
Gm11229	7168		#N/A	1.000000E+00
Gm1123	7169	-0.038406171	3065	5.892384E-01
Gm11231	7170		#N/A	1.000000E+00
Gm11232	7171	0.00030705	5316.5	9.965570E-01
Gm11233	7172		#N/A	1.000000E+00
Gm11234	7173	0.074574898	10201.5	2.939481E-01
Gm11235	7174		#N/A	1.000000E+00
Gm11236	7175		#N/A	1.000000E+00
Gm11237	7176		#N/A	1.000000E+00
Gm11238	7177	0.053267784	8685	4.537786E-01
Gm11239	7178		#N/A	1.000000E+00
Gm11240	7179	-0.066856562	1139	3.468991E-01
Gm11241	7180		#N/A	1.000000E+00

Spearman Rank correlation analysis performed between Prdm1 and all-expressed genes within the Meredith RNA-seq dataset. Robust Prdm1–associated genes were identified using a cut-off of $p < 0.0005$.

Gm11242	7181	-0.038406171	3065	5.892384E-01
Gm11243	7182	-0.018447724	4312.5	7.954215E-01
Gm11244	7183		#N/A	1.000000E+00
Gm11245	7184		#N/A	1.000000E+00
Gm11246	7185		#N/A	1.000000E+00
Gm11247	7186		#N/A	1.000000E+00
Gm11248	7187		#N/A	1.000000E+00
Gm11249	7188		#N/A	1.000000E+00
Gm11250	7189		#N/A	1.000000E+00
Gm11251	7190	-0.038406171	3065	5.892384E-01
Gm11252	7191		#N/A	1.000000E+00
Gm11253	7192	0.014301797	6015.5	8.406982E-01
Gm11254	7193	-0.038406171	3065	5.892384E-01
Gm11255	7194		#N/A	1.000000E+00
Gm11256	7195		#N/A	1.000000E+00
Gm11257	7196		#N/A	1.000000E+00
Gm11258	7197	-0.038406171	3065	5.892384E-01
Gm11259	7198		#N/A	1.000000E+00
Gm11260	7199		#N/A	1.000000E+00
Gm11261	7200		#N/A	1.000000E+00
Gm11262	7201		#N/A	1.000000E+00
Gm11263	7202		#N/A	1.000000E+00
Gm11264	7203		#N/A	1.000000E+00
Gm11266	7204	0.049855249	8285.5	4.832553E-01
Gm11267	7205		#N/A	1.000000E+00
Gm11268	7206		#N/A	1.000000E+00
Gm11269	7207		#N/A	1.000000E+00
Gm11270	7208		#N/A	1.000000E+00
Gm11271	7209	0.152789767	15822	3.077826E-02
Gm11273	7210	0.079756392	10576	2.615882E-01
Gm11274	7211		#N/A	1.000000E+00
Gm11278	7212		#N/A	1.000000E+00
Gm11279	7213		#N/A	1.000000E+00
Gm11280	7214	-0.054451513	1703	4.437940E-01
Gm11281	7215	0.06902692	9779.5	3.314346E-01
Gm11282	7216		#N/A	1.000000E+00
Gm11284	7217		#N/A	1.000000E+00
Gm11285	7218	0.162785326	16514	2.127402E-02
Gm11290	7219		#N/A	1.000000E+00
Gm11291	7220		#N/A	1.000000E+00
Gm11292	7221		#N/A	1.000000E+00
Gm11295	7222	0.160304018	16323	2.335876E-02
Gm11298	7223		#N/A	1.000000E+00

Table S1, Related to Supplemental Figure 3C. Prdm1 associated genes

Gm11300	7224		#N/A	1.000000E+00
Gm11307	7225	-0.077397959	691.5	2.760011E-01
Gm11309	7226		#N/A	1.000000E+00
Gm11313	7227		#N/A	1.000000E+00
Gm11314	7228		#N/A	1.000000E+00
Gm11318	7229		#N/A	1.000000E+00
Gm11320	7230		#N/A	1.000000E+00
Gm11321	7231		#N/A	1.000000E+00
Gm11322	7232		#N/A	1.000000E+00
Gm11325	7233		#N/A	1.000000E+00
Gm11331	7234		#N/A	1.000000E+00
Gm11333	7235		#N/A	1.000000E+00
Gm11334	7236		#N/A	1.000000E+00
Gm11335	7237		#N/A	1.000000E+00
Gm11336	7238		#N/A	1.000000E+00
Gm11337	7239	0.34182415	19996	7.275601E-07
Gm11338	7240		#N/A	1.000000E+00
Gm11339	7241	0.230217498	19157	1.039825E-03
Gm11340	7242		#N/A	1.000000E+00
Gm11341	7243	0.109492511	12666.5	1.227347E-01
Gm11342	7244		#N/A	1.000000E+00
Gm11343	7245	0.113548679	13094	1.093908E-01
Gm11344	7246	0.021317126	6513.5	7.644715E-01
Gm11345	7247		#N/A	1.000000E+00
Gm11346	7248	0.156002604	16038	2.739067E-02
Gm11348	7249		#N/A	1.000000E+00
Gm11349	7250		#N/A	1.000000E+00
Gm11350	7251		#N/A	1.000000E+00
Gm11351	7252	0.038229357	7499.5	5.909551E-01
Gm11352	7253		#N/A	1.000000E+00
Gm11353	7254	0.050544302	8344.5	4.772215E-01
Gm11354	7255	0.097564782	11875	1.693153E-01
Gm11355	7256	-0.038406171	3065	5.892384E-01
Gm11356	7257		#N/A	1.000000E+00
Gm11357	7258		#N/A	1.000000E+00
Gm11358	7259		#N/A	1.000000E+00
Gm11359	7260	0.143605682	15224	4.249002E-02
Gm11360	7261		#N/A	1.000000E+00
Gm11361	7262		#N/A	1.000000E+00
Gm11362	7263	-0.038406171	3065	5.892384E-01
Gm11363	7264	-0.038406171	3065	5.892384E-01
Gm11364	7265		#N/A	1.000000E+00
Gm11365	7266		#N/A	1.000000E+00

Spearman Rank correlation analysis performed between Prdm1 and all-expressed genes within the Meredith RNA-seq dataset. Robust Prdm1–associated genes were identified using a cut-off of $p < 0.0005$.

Table S1, Related to Supplemental Figure 3C. Prdm1 associated genes

Gm11366	7267		#N/A	1.000000E+00
Gm11367	7268		#N/A	1.000000E+00
Gm11368	7269		#N/A	1.000000E+00
Gm11369	7270		#N/A	1.000000E+00
Gm11370	7271		#N/A	1.000000E+00
Gm11371	7272		#N/A	1.000000E+00
Gm11372	7273		#N/A	1.000000E+00
Gm11373	7274	-0.038406171	3065	5.892384E-01
Gm11374	7275		#N/A	1.000000E+00
Gm11375	7276	-0.011591882	4684	8.705878E-01
Gm11376	7277	-0.016267086	4463.5	8.191608E-01
Gm11377	7278		#N/A	1.000000E+00
Gm11378	7279		#N/A	1.000000E+00
Gm11379	7280		#N/A	1.000000E+00
Gm11380	7281	0.053267784	8685	4.537786E-01
Gm11381	7282		#N/A	1.000000E+00
Gm11382	7283		#N/A	1.000000E+00
Gm11383	7284		#N/A	1.000000E+00
Gm11384	7285		#N/A	1.000000E+00
Gm11385	7286		#N/A	1.000000E+00
Gm11386	7287		#N/A	1.000000E+00
Gm11387	7288		#N/A	1.000000E+00
Gm11388	7289		#N/A	1.000000E+00
Gm11389	7290		#N/A	1.000000E+00
Gm11393	7291		#N/A	1.000000E+00
Gm11397	7292		#N/A	1.000000E+00
Gm11398	7293		#N/A	1.000000E+00
Gm11399	7294		#N/A	1.000000E+00
Gm1140	7295		#N/A	1.000000E+00
Gm11400	7296	0.113548679	13094	1.093908E-01
Gm11401	7297	-0.066856562	1139	3.468991E-01
Gm11402	7298		#N/A	1.000000E+00
Gm11403	7299		#N/A	1.000000E+00
Gm11404	7300		#N/A	1.000000E+00
Gm11405	7301		#N/A	1.000000E+00
Gm11406	7302		#N/A	1.000000E+00
Gm11407	7303	0.021317126	6513.5	7.644715E-01
Gm11408	7304	-0.038406171	3065	5.892384E-01
Gm11409	7305		#N/A	1.000000E+00
Gm1141	7306		#N/A	1.000000E+00
Gm11410	7307	0.113379154	12947	1.099247E-01
Gm11411	7308	-0.038406171	3065	5.892384E-01
Gm11412	7309		#N/A	1.000000E+00

Spearman Rank correlation analysis performed between Prdm1 and all-expressed genes within the Meredith RNA-seq dataset. Robust Prdm1–associated genes were identified using a cut-off of $p < 0.0005$.

Table S1, Related to Supplemental Figure 3C. Prdm1 associated genes

Gm11413	7310	0.14781234	15527	3.672870E-02
Gm11414	7311		#N/A	1.000000E+00
Gm11415	7312	0.113548679	13094	1.093908E-01
Gm11416	7313		#N/A	1.000000E+00
Gm11417	7314		#N/A	1.000000E+00
Gm11418	7315		#N/A	1.000000E+00
Gm11419	7316		#N/A	1.000000E+00
Gm11420	7317		#N/A	1.000000E+00
Gm11421	7318		#N/A	1.000000E+00
Gm11422	7319		#N/A	1.000000E+00
Gm11423	7320	0.032463519	7201	6.481411E-01
Gm11424	7321		#N/A	1.000000E+00
Gm11425	7322		#N/A	1.000000E+00
Gm11426	7323	-0.038406171	3065	5.892384E-01
Gm11427	7324		#N/A	1.000000E+00
Gm11429	7325		#N/A	1.000000E+00
Gm11430	7326		#N/A	1.000000E+00
Gm11431	7327	-0.038406171	3065	5.892384E-01
Gm11432	7328		#N/A	1.000000E+00
Gm11433	7329		#N/A	1.000000E+00
Gm11434	7330		#N/A	1.000000E+00
Gm11435	7331	-0.038406171	3065	5.892384E-01
Gm11437	7332	0.021317126	6513.5	7.644715E-01
Gm11438	7333	-0.038406171	3065	5.892384E-01
Gm11439	7334		#N/A	1.000000E+00
Gm11440	7335		#N/A	1.000000E+00
Gm11442	7336		#N/A	1.000000E+00
Gm11443	7337		#N/A	1.000000E+00
Gm11444	7338	0.01934762	6296	7.856781E-01
Gm11445	7339		#N/A	1.000000E+00
Gm11446	7340	-0.066856562	1139	3.468991E-01
Gm11447	7341		#N/A	1.000000E+00
Gm11448	7342		#N/A	1.000000E+00
Gm11449	7343		#N/A	1.000000E+00
Gm11450	7344	0.04534714	7959	5.237251E-01
Gm11451	7345		#N/A	1.000000E+00
Gm11452	7346		#N/A	1.000000E+00
Gm11453	7347		#N/A	1.000000E+00
Gm11454	7348	-0.013540297	4588.5	8.490763E-01
Gm11455	7349		#N/A	1.000000E+00
Gm11456	7350		#N/A	1.000000E+00
Gm11457	7351	0.242418018	19402.5	5.430862E-04
Gm11458	7352	0.02421538	6767	7.335850E-01

Spearman Rank correlation analysis performed between Prdm1 and all-expressed genes within the Meredith RNA-seq dataset. Robust Prdm1–associated genes were identified using a cut-off of $p < 0.0005$.

Table S1, Related to Supplemental Figure 3C. Prdm1 associated genes

Gm11459	7353		#N/A	1.000000E+00
Gm11460	7354		#N/A	1.000000E+00
Gm11461	7355		#N/A	1.000000E+00
Gm11462	7356	-0.054451513	1703	4.437940E-01
Gm11463	7357	0.125079149	13973	7.760819E-02
Gm11464	7358		#N/A	1.000000E+00
Gm11465	7359		#N/A	1.000000E+00
Gm11466	7360		#N/A	1.000000E+00
Gm11467	7361		#N/A	1.000000E+00
Gm11468	7362		#N/A	1.000000E+00
Gm11469	7363	0.089371505	11299	2.082104E-01
Gm11470	7364	-0.054450825	1984	4.437997E-01
Gm11471	7365		#N/A	1.000000E+00
Gm11472	7366	0.030061148	7068	6.726166E-01
Gm11473	7367	0.134200998	14569.5	5.814916E-02
Gm11474	7368	-0.066856562	1139	3.468991E-01
Gm11475	7369		#N/A	1.000000E+00
Gm11476	7370	0.024351285	6778	7.321467E-01
Gm11477	7371		#N/A	1.000000E+00
Gm11478	7372	-0.004937246	4940	9.446824E-01
Gm11479	7373		#N/A	1.000000E+00
Gm11480	7374		#N/A	1.000000E+00
Gm11481	7375		#N/A	1.000000E+00
Gm11482	7376		#N/A	1.000000E+00
Gm11483	7377	0.203601309	18374.5	3.832123E-03
Gm11484	7378		#N/A	1.000000E+00
Gm11485	7379		#N/A	1.000000E+00
Gm11486	7380		#N/A	1.000000E+00
Gm11487	7381		#N/A	1.000000E+00
Gm11488	7382		#N/A	1.000000E+00
Gm11489	7383		#N/A	1.000000E+00
Gm11490	7384		#N/A	1.000000E+00
Gm11491	7385	0.013521743	5971	8.492806E-01
Gm11492	7386		#N/A	1.000000E+00
Gm11493	7387		#N/A	1.000000E+00
Gm11494	7388	-0.038406171	3065	5.892384E-01
Gm11496	7389		#N/A	1.000000E+00
Gm11497	7390		#N/A	1.000000E+00
Gm11498	7391		#N/A	1.000000E+00
Gm11499	7392		#N/A	1.000000E+00
Gm11500	7393	-0.054451513	1703	4.437940E-01
Gm11501	7394		#N/A	1.000000E+00
Gm11502	7395		#N/A	1.000000E+00

Spearman Rank correlation analysis performed between Prdm1 and all-expressed genes within the Meredith RNA-seq dataset. Robust Prdm1–associated genes were identified using a cut-off of $p < 0.0005$.

Table S1, Related to Supplemental Figure 3C. Prdm1 associated genes

Gm11504	7396		#N/A	1.000000E+00
Gm11505	7397		#N/A	1.000000E+00
Gm11506	7398	-0.016225238	4466	8.196180E-01
Gm11507	7399		#N/A	1.000000E+00
Gm11508	7400	0.010605938	5797	8.815105E-01
Gm11509	7401		#N/A	1.000000E+00
Gm11510	7402		#N/A	1.000000E+00
Gm11511	7403		#N/A	1.000000E+00
Gm11512	7404	0.139566339	14926	4.871793E-02
Gm11513	7405		#N/A	1.000000E+00
Gm11514	7406	0.133599303	14516	5.929493E-02
Gm11515	7407		#N/A	1.000000E+00
Gm11516	7408		#N/A	1.000000E+00
Gm11517	7409	0.152090827	15775	3.156073E-02
Gm11518	7410		#N/A	1.000000E+00
Gm11520	7411	0.193815607	17983	5.961153E-03
Gm11521	7412		#N/A	1.000000E+00
Gm11522	7413		#N/A	1.000000E+00
Gm11523	7414		#N/A	1.000000E+00
Gm11524	7415	-0.038406171	3065	5.892384E-01
Gm11525	7416		#N/A	1.000000E+00
Gm11526	7417		#N/A	1.000000E+00
Gm11527	7418	0.020434681	6357	7.739526E-01
Gm11528	7419		#N/A	1.000000E+00
Gm11529	7420		#N/A	1.000000E+00
Gm11531	7421		#N/A	1.000000E+00
Gm11532	7422	0.020740754	6384	7.706602E-01
Gm11533	7423		#N/A	1.000000E+00
Gm11534	7424	0.113548679	13094	1.093908E-01
Gm11535	7425	-0.038406171	3065	5.892384E-01
Gm11536	7426	0.1544596	15936.5	2.897553E-02
Gm11537	7427		#N/A	1.000000E+00
Gm11539	7428		#N/A	1.000000E+00
Gm11540	7429	0.048448014	8175.5	4.957045E-01
Gm11541	7430	0.038758411	7558.5	5.858252E-01
Gm11542	7431	-0.038406171	3065	5.892384E-01
Gm11543	7432		#N/A	1.000000E+00
Gm11544	7433	0.132800502	14465	6.084469E-02
Gm11545	7434	-0.010903374	4706	8.782129E-01
Gm11546	7435		#N/A	1.000000E+00
Gm11547	7436	0.139572743	14928	4.870748E-02
Gm11548	7437	0.020143681	6339	7.770865E-01
Gm11549	7438	-0.038406171	3065	5.892384E-01

Spearman Rank correlation analysis performed between Prdm1 and all-expressed genes within the Meredith RNA-seq dataset. Robust Prdm1–associated genes were identified using a cut-off of $p < 0.0005$.

Table S1, Related to Supplemental Figure 3C. Prdm1 associated genes

Gm11550	7439		#N/A	1.000000E+00
Gm11551	7440	-0.018790377	4285	7.917077E-01
Gm11553	7441		#N/A	1.000000E+00
Gm11554	7442		#N/A	1.000000E+00
Gm11555	7443		#N/A	1.000000E+00
Gm11556	7444		#N/A	1.000000E+00
Gm11557	7445		#N/A	1.000000E+00
Gm11558	7446	-0.038406171	3065	5.892384E-01
Gm11559	7447	-0.077397959	691.5	2.760011E-01
Gm11560	7448	0.073928833	10125	2.981622E-01
Gm11561	7449		#N/A	1.000000E+00
Gm11562	7450		#N/A	1.000000E+00
Gm11563	7451		#N/A	1.000000E+00
Gm11564	7452		#N/A	1.000000E+00
Gm11565	7453		#N/A	1.000000E+00
Gm11566	7454		#N/A	1.000000E+00
Gm11567	7455		#N/A	1.000000E+00
Gm11568	7456		#N/A	1.000000E+00
Gm11569	7457		#N/A	1.000000E+00
Gm11571	7458		#N/A	1.000000E+00
Gm11572	7459	-0.001223995	5170	9.862759E-01
Gm11573	7460	0.160304018	16323	2.335876E-02
Gm11574	7461	0.076253456	10364	2.831856E-01
Gm11575	7462		#N/A	1.000000E+00
Gm11576	7463		#N/A	1.000000E+00
Gm11577	7464		#N/A	1.000000E+00
Gm11578	7465		#N/A	1.000000E+00
Gm11579	7466	-0.038406171	3065	5.892384E-01
Gm11581	7467		#N/A	1.000000E+00
Gm11582	7468		#N/A	1.000000E+00
Gm11583	7469	0.044087692	7873	5.353318E-01
Gm11584	7470		#N/A	1.000000E+00
Gm11585	7471	-0.038406171	3065	5.892384E-01
Gm11586	7472		#N/A	1.000000E+00
Gm11587	7473		#N/A	1.000000E+00
Gm11588	7474	-0.026612978	4044	7.083511E-01
Gm11590	7475		#N/A	1.000000E+00
Gm11591	7476		#N/A	1.000000E+00
Gm11592	7477		#N/A	1.000000E+00
Gm11594	7478		#N/A	1.000000E+00
Gm11595	7479		#N/A	1.000000E+00
Gm11596	7480		#N/A	1.000000E+00
Gm11597	7481	0.053267784	8685	4.537786E-01

Spearman Rank correlation analysis performed between Prdm1 and all-expressed genes within the Meredith RNA-seq dataset. Robust Prdm1–associated genes were identified using a cut-off of $p < 0.0005$.

Table S1, Related to Supplemental Figure 3C. Prdm1 associated genes

Gm11598	7482	0.090555233	11385.5	2.022247E-01
Gm11599	7483	-0.038406171	3065	5.892384E-01
Gm11600	7484	0.113548679	13094	1.093908E-01
Gm11601	7485	0.001442702	5387.5	9.838240E-01
Gm11602	7486	0.073540537	10103	3.007140E-01
Gm11603	7487	0.113548679	13094	1.093908E-01
Gm11604	7488		#N/A	1.000000E+00
Gm11605	7489		#N/A	1.000000E+00
Gm11606	7490		#N/A	1.000000E+00
Gm11607	7491	-0.054451513	1703	4.437940E-01
Gm11608	7492	0.113548679	13094	1.093908E-01
Gm11609	7493		#N/A	1.000000E+00
Gm11610	7494	0.310581347	19940	7.607524E-06
Gm11611	7495	0.053267784	8685	4.537786E-01
Gm11612	7496	0.152789767	15822	3.077826E-02
Gm11613	7497	-0.066856562	1139	3.468991E-01
Gm11614	7498	-0.054451513	1703	4.437940E-01
Gm11615	7499		#N/A	1.000000E+00
Gm11616	7500		#N/A	1.000000E+00
Gm11617	7501	-0.038406171	3065	5.892384E-01
Gm11618	7502		#N/A	1.000000E+00
Gm11619	7503	-0.054451513	1703	4.437940E-01
Gm11620	7504		#N/A	1.000000E+00
Gm11621	7505	0.113548679	13094	1.093908E-01
Gm11622	7506		#N/A	1.000000E+00
Gm11623	7507		#N/A	1.000000E+00
Gm11624	7508		#N/A	1.000000E+00
Gm11625	7509		#N/A	1.000000E+00
Gm11626	7510		#N/A	1.000000E+00
Gm11627	7511	0.020875719	6412.5	7.692098E-01
Gm11628	7512	0.143605682	15224	4.249002E-02
Gm11629	7513	-0.054451513	1703	4.437940E-01
Gm11630	7514		#N/A	1.000000E+00
Gm11631	7515	-0.054451513	1703	4.437940E-01
Gm11632	7516		#N/A	1.000000E+00
Gm11633	7517	-0.066856562	1139	3.468991E-01
Gm11634	7518	-0.018105422	4347	7.991361E-01
Gm11635	7519		#N/A	1.000000E+00
Gm11636	7520		#N/A	1.000000E+00
Gm11637	7521	-0.038406171	3065	5.892384E-01
Gm11638	7522		#N/A	1.000000E+00
Gm11639	7523	0.073115751	10073	3.035222E-01
Gm11640	7524		#N/A	1.000000E+00

Spearman Rank correlation analysis performed between Prdm1 and all-expressed genes within the Meredith RNA-seq dataset. Robust Prdm1–associated genes were identified using a cut-off of $p < 0.0005$.

Table S1, Related to Supplemental Figure 3C. Prdm1 associated genes

Gm11641	7525	0.150429395	15675	3.348894E-02
Gm11642	7526		#N/A	1.000000E+00
Gm11643	7527		#N/A	1.000000E+00
Gm11644	7528		#N/A	1.000000E+00
Gm11645	7529		#N/A	1.000000E+00
Gm11646	7530		#N/A	1.000000E+00
Gm11647	7531		#N/A	1.000000E+00
Gm11648	7532	0.148620224	15568	3.570144E-02
Gm11649	7533		#N/A	1.000000E+00
Gm11650	7534		#N/A	1.000000E+00
Gm11651	7535		#N/A	1.000000E+00
Gm11652	7536		#N/A	1.000000E+00
Gm11653	7537		#N/A	1.000000E+00
Gm11655	7538		#N/A	1.000000E+00
Gm11656	7539		#N/A	1.000000E+00
Gm11657	7540		#N/A	1.000000E+00
Gm11658	7541	-0.038406171	3065	5.892384E-01
Gm11659	7542	-0.038406171	3065	5.892384E-01
Gm11660	7543		#N/A	1.000000E+00
Gm11661	7544		#N/A	1.000000E+00
Gm11662	7545	0.1544596	15936.5	2.897553E-02
Gm11663	7546		#N/A	1.000000E+00
Gm11665	7547		#N/A	1.000000E+00
Gm11666	7548	0.113548679	13094	1.093908E-01
Gm11667	7549		#N/A	1.000000E+00
Gm11668	7550		#N/A	1.000000E+00
Gm11669	7551		#N/A	1.000000E+00
Gm11670	7552		#N/A	1.000000E+00
Gm11671	7553	0.16887006	16869	1.682971E-02
Gm11672	7554	-0.038406171	3065	5.892384E-01
Gm11673	7555		#N/A	1.000000E+00
Gm11674	7556		#N/A	1.000000E+00
Gm11675	7557	0.1544596	15936.5	2.897553E-02
Gm11676	7558		#N/A	1.000000E+00
Gm11677	7559		#N/A	1.000000E+00
Gm11678	7560		#N/A	1.000000E+00
Gm11679	7561		#N/A	1.000000E+00
Gm11680	7562		#N/A	1.000000E+00
Gm11681	7563	0.063365727	9418	3.727114E-01
Gm11682	7564		#N/A	1.000000E+00
Gm11683	7565		#N/A	1.000000E+00
Gm11684	7566		#N/A	1.000000E+00
Gm11685	7567	-0.054451513	1703	4.437940E-01

Spearman Rank correlation analysis performed between Prdm1 and all-expressed genes within the Meredith RNA-seq dataset. Robust Prdm1–associated genes were identified using a cut-off of $p < 0.0005$.

Table S1, Related to Supplemental Figure 3C. Prdm1 associated genes

Gm11686	7568	0.090858474	11420.5	2.007115E-01
Gm11687	7569	-0.038406171	3065	5.892384E-01
Gm11688	7570	-0.036320164	3677	6.096356E-01
Gm11689	7571		#N/A	1.000000E+00
Gm11690	7572		#N/A	1.000000E+00
Gm11691	7573		#N/A	1.000000E+00
Gm11692	7574		#N/A	1.000000E+00
Gm11693	7575		#N/A	1.000000E+00
Gm11694	7576	-0.095280123	319	1.795721E-01
Gm11695	7577		#N/A	1.000000E+00
Gm11696	7578	0.004891637	5557	9.451926E-01
Gm11697	7579		#N/A	1.000000E+00
Gm11698	7580	0.106001553	12425	1.352007E-01
Gm11699	7581		#N/A	1.000000E+00
Gm11700	7582		#N/A	1.000000E+00
Gm11701	7583		#N/A	1.000000E+00
Gm11702	7584		#N/A	1.000000E+00
Gm11703	7585	0.113548679	13094	1.093908E-01
Gm11704	7586		#N/A	1.000000E+00
Gm11705	7587		#N/A	1.000000E+00
Gm11706	7588		#N/A	1.000000E+00
Gm11707	7589		#N/A	1.000000E+00
Gm11708	7590		#N/A	1.000000E+00
Gm11709	7591		#N/A	1.000000E+00
Gm11710	7592		#N/A	1.000000E+00
Gm11711	7593		#N/A	1.000000E+00
Gm11712	7594		#N/A	1.000000E+00
Gm11713	7595		#N/A	1.000000E+00
Gm11714	7596		#N/A	1.000000E+00
Gm11715	7597	0.182294195	17509.5	9.778799E-03
Gm11716	7598	0.287823186	19855	3.585053E-05
Gm11718	7599		#N/A	1.000000E+00
Gm11719	7600	-0.066858259	1029.5	3.468868E-01
Gm11721	7601		#N/A	1.000000E+00
Gm11722	7602		#N/A	1.000000E+00
Gm11723	7603		#N/A	1.000000E+00
Gm11724	7604	0.087844585	11191	2.161184E-01
Gm11725	7605	0.072570383	10034	3.071528E-01
Gm11726	7606	-0.054450825	1984	4.437997E-01
Gm11728	7607		#N/A	1.000000E+00
Gm11729	7608		#N/A	1.000000E+00
Gm11730	7609		#N/A	1.000000E+00
Gm11731	7610		#N/A	1.000000E+00

Spearman Rank correlation analysis performed between Prdm1 and all-expressed genes within the Meredith RNA-seq dataset. Robust Prdm1–associated genes were identified using a cut-off of $p < 0.0005$.

Table S1, Related to Supplemental Figure 3C. Prdm1 associated genes

Gm11732	7611		#N/A	1.000000E+00
Gm11733	7612		#N/A	1.000000E+00
Gm11734	7613		#N/A	1.000000E+00
Gm11735	7614	-0.054451513	1703	4.437940E-01
Gm11736	7615		#N/A	1.000000E+00
Gm11737	7616		#N/A	1.000000E+00
Gm11738	7617		#N/A	1.000000E+00
Gm11739	7618	0.113548679	13094	1.093908E-01
Gm11742	7619		#N/A	1.000000E+00
Gm11743	7620		#N/A	1.000000E+00
Gm11744	7621	0.081317556	10686	2.523366E-01
Gm11745	7622		#N/A	1.000000E+00
Gm11746	7623		#N/A	1.000000E+00
Gm11747	7624	0.031318251	7145	6.597641E-01
Gm11748	7625		#N/A	1.000000E+00
Gm11749	7626		#N/A	1.000000E+00
Gm11750	7627		#N/A	1.000000E+00
Gm11751	7628	-0.006714509	4860	9.248205E-01
Gm11752	7629		#N/A	1.000000E+00
Gm11753	7630		#N/A	1.000000E+00
Gm11754	7631		#N/A	1.000000E+00
Gm11755	7632	0.216041607	18787	2.122693E-03
Gm11756	7633		#N/A	1.000000E+00
Gm11757	7634		#N/A	1.000000E+00
Gm11758	7635		#N/A	1.000000E+00
Gm11759	7636	0.038758411	7558.5	5.858252E-01
Gm11760	7637	0.074180476	10151	2.965160E-01
Gm11761	7638	0.22212366	18960	1.571213E-03
Gm11762	7639		#N/A	1.000000E+00
Gm11763	7640		#N/A	1.000000E+00
Gm11764	7641		#N/A	1.000000E+00
Gm11765	7642	0.164723247	16638	1.975959E-02
Gm11766	7643		#N/A	1.000000E+00
Gm11767	7644		#N/A	1.000000E+00
Gm11768	7645	-0.066856562	1139	3.468991E-01
Gm11769	7646	-0.054450825	1984	4.437997E-01
Gm11770	7647		#N/A	1.000000E+00
Gm11771	7648		#N/A	1.000000E+00
Gm11772	7649	-0.066855996	1239.5	3.469032E-01
Gm11773	7650		#N/A	1.000000E+00
Gm11774	7651		#N/A	1.000000E+00
Gm11775	7652	0.021317126	6513.5	7.644715E-01
Gm11779	7653		#N/A	1.000000E+00

Spearman Rank correlation analysis performed between Prdm1 and all-expressed genes within the Meredith RNA-seq dataset. Robust Prdm1–associated genes were identified using a cut-off of $p < 0.0005$.

Table S1, Related to Supplemental Figure 3C. Prdm1 associated genes

Gm11780	7654	-0.054451513	1703	4.437940E-01
Gm11781	7655		#N/A	1.000000E+00
Gm11782	7656		#N/A	1.000000E+00
Gm11783	7657		#N/A	1.000000E+00
Gm11784	7658		#N/A	1.000000E+00
Gm11785	7659		#N/A	1.000000E+00
Gm11786	7660		#N/A	1.000000E+00
Gm11787	7661	-0.038406171	3065	5.892384E-01
Gm11788	7662		#N/A	1.000000E+00
Gm11789	7663	-0.038406171	3065	5.892384E-01
Gm11790	7664		#N/A	1.000000E+00
Gm11791	7665		#N/A	1.000000E+00
Gm11792	7666		#N/A	1.000000E+00
Gm11793	7667		#N/A	1.000000E+00
Gm11794	7668		#N/A	1.000000E+00
Gm11795	7669		#N/A	1.000000E+00
Gm11796	7670		#N/A	1.000000E+00
Gm11797	7671		#N/A	1.000000E+00
Gm11798	7672		#N/A	1.000000E+00
Gm11799	7673		#N/A	1.000000E+00
Gm11800	7674		#N/A	1.000000E+00
Gm11801	7675		#N/A	1.000000E+00
Gm11802	7676		#N/A	1.000000E+00
Gm11803	7677	-0.054450825	1984	4.437997E-01
Gm11804	7678		#N/A	1.000000E+00
Gm11805	7679		#N/A	1.000000E+00
Gm11807	7680		#N/A	1.000000E+00
Gm11808	7681	0.18596828	17679	8.375549E-03
Gm11809	7682		#N/A	1.000000E+00
Gm11810	7683	-0.000841282	5208.5	9.905669E-01
Gm11812	7684		#N/A	1.000000E+00
Gm11813	7685		#N/A	1.000000E+00
Gm11814	7686		#N/A	1.000000E+00
Gm11815	7687		#N/A	1.000000E+00
Gm11816	7688		#N/A	1.000000E+00
Gm11817	7689	-0.038406171	3065	5.892384E-01
Gm11819	7690		#N/A	1.000000E+00
Gm11820	7691		#N/A	1.000000E+00
Gm11821	7692		#N/A	1.000000E+00
Gm11822	7693	0.038758411	7558.5	5.858252E-01
Gm11823	7694	0.064555735	9508	3.637825E-01
Gm11824	7695	-0.038406171	3065	5.892384E-01
Gm11825	7696		#N/A	1.000000E+00

Spearman Rank correlation analysis performed between Prdm1 and all-expressed genes within the Meredith RNA-seq dataset. Robust Prdm1–associated genes were identified using a cut-off of $p < 0.0005$.

Table S1, Related to Supplemental Figure 3C. Prdm1 associated genes

Gm11826	7697	-0.038406171	3065	5.892384E-01
Gm11827	7698		#N/A	1.000000E+00
Gm11829	7699	-0.038406171	3065	5.892384E-01
Gm11830	7700	0.162808768	16526.5	2.125512E-02
Gm11831	7701		#N/A	1.000000E+00
Gm11832	7702		#N/A	1.000000E+00
Gm11833	7703		#N/A	1.000000E+00
Gm11834	7704		#N/A	1.000000E+00
Gm11835	7705		#N/A	1.000000E+00
Gm11836	7706	0.020611413	6377.5	7.720510E-01
Gm11837	7707	-0.054450825	1984	4.437997E-01
Gm11838	7708		#N/A	1.000000E+00
Gm11839	7709	-0.038406171	3065	5.892384E-01
Gm11840	7710		#N/A	1.000000E+00
Gm11841	7711		#N/A	1.000000E+00
Gm11842	7712	-0.038406171	3065	5.892384E-01
Gm11843	7713		#N/A	1.000000E+00
Gm11844	7714		#N/A	1.000000E+00
Gm11845	7715	-0.038406171	3065	5.892384E-01
Gm11846	7716	-0.038406171	3065	5.892384E-01
Gm11847	7717	0.163266121	16558	2.088922E-02
Gm11848	7718		#N/A	1.000000E+00
Gm11849	7719	0.08926081	11290	2.087766E-01
Gm11850	7720		#N/A	1.000000E+00
Gm11851	7721		#N/A	1.000000E+00
Gm11852	7722		#N/A	1.000000E+00
Gm11853	7723		#N/A	1.000000E+00
Gm11854	7724		#N/A	1.000000E+00
Gm11855	7725	0.113548679	13094	1.093908E-01
Gm11856	7726		#N/A	1.000000E+00
Gm11857	7727		#N/A	1.000000E+00
Gm11858	7728		#N/A	1.000000E+00
Gm11859	7729		#N/A	1.000000E+00
Gm11860	7730	0.127723427	14169	7.149050E-02
Gm11861	7731	0.116691532	13446.5	9.985642E-02
Gm11862	7732		#N/A	1.000000E+00
Gm11863	7733		#N/A	1.000000E+00
Gm11864	7734		#N/A	1.000000E+00
Gm11865	7735		#N/A	1.000000E+00
Gm11866	7736		#N/A	1.000000E+00
Gm11867	7737	0.052118433	8479	4.635924E-01
Gm11868	7738		#N/A	1.000000E+00
Gm11869	7739	-0.054451513	1703	4.437940E-01

Spearman Rank correlation analysis performed between Prdm1 and all-expressed genes within the Meredith RNA-seq dataset. Robust Prdm1–associated genes were identified using a cut-off of $p < 0.0005$.

Table S1, Related to Supplemental Figure 3C. Prdm1 associated genes

Gm11870	7740		#N/A	1.000000E+00
Gm11871	7741		#N/A	1.000000E+00
Gm11872	7742		#N/A	1.000000E+00
Gm11873	7743		#N/A	1.000000E+00
Gm11874	7744		#N/A	1.000000E+00
Gm11875	7745	0.115443826	13352	1.035597E-01
Gm11876	7746	-0.054451513	1703	4.437940E-01
Gm11877	7747		#N/A	1.000000E+00
Gm11878	7748	0.021757707	6579.5	7.597509E-01
Gm11879	7749		#N/A	1.000000E+00
Gm11880	7750		#N/A	1.000000E+00
Gm11881	7751		#N/A	1.000000E+00
Gm11882	7752		#N/A	1.000000E+00
Gm11883	7753		#N/A	1.000000E+00
Gm11884	7754	0.046648584	8058	5.118674E-01
Gm11885	7755		#N/A	1.000000E+00
Gm11886	7756		#N/A	1.000000E+00
Gm11887	7757		#N/A	1.000000E+00
Gm11888	7758	0.088187776	11217	2.143225E-01
Gm11889	7759		#N/A	1.000000E+00
Gm11890	7760		#N/A	1.000000E+00
Gm11891	7761	-0.038406171	3065	5.892384E-01
Gm11892	7762	0.152789767	15822	3.077826E-02
Gm11893	7763		#N/A	1.000000E+00
Gm11894	7764		#N/A	1.000000E+00
Gm11895	7765		#N/A	1.000000E+00
Gm11896	7766		#N/A	1.000000E+00
Gm11897	7767		#N/A	1.000000E+00
Gm11898	7768		#N/A	1.000000E+00
Gm11899	7769		#N/A	1.000000E+00
Gm11900	7770		#N/A	1.000000E+00
Gm11901	7771		#N/A	1.000000E+00
Gm11902	7772		#N/A	1.000000E+00
Gm11903	7773		#N/A	1.000000E+00
Gm11904	7774		#N/A	1.000000E+00
Gm11905	7775		#N/A	1.000000E+00
Gm11906	7776	0.244423179	19432	4.865462E-04
Gm11907	7777		#N/A	1.000000E+00
Gm11908	7778	-0.038406171	3065	5.892384E-01
Gm11909	7779		#N/A	1.000000E+00
Gm11910	7780	0.113548679	13094	1.093908E-01
Gm11911	7781	-0.038406171	3065	5.892384E-01
Gm11912	7782		#N/A	1.000000E+00

Spearman Rank correlation analysis performed between Prdm1 and all-expressed genes within the Meredith RNA-seq dataset. Robust Prdm1–associated genes were identified using a cut-off of $p < 0.0005$.

Gm11913	7783	0.053267784	8685	4.537786E-01
Gm11914	7784		#N/A	1.000000E+00
Gm11915	7785		#N/A	1.000000E+00
Gm11916	7786		#N/A	1.000000E+00
Gm11917	7787	0.053267784	8685	4.537786E-01
Gm11918	7788		#N/A	1.000000E+00
Gm11919	7789		#N/A	1.000000E+00
Gm11920	7790		#N/A	1.000000E+00
Gm11921	7791		#N/A	1.000000E+00
Gm11922	7792	-0.038406171	3065	5.892384E-01
Gm11923	7793	0.053805701	8820	4.492258E-01
Gm11924	7794	0.224069878	19021	1.424603E-03
Gm11925	7795		#N/A	1.000000E+00
Gm11926	7796		#N/A	1.000000E+00
Gm11927	7797		#N/A	1.000000E+00
Gm11928	7798	-0.038406171	3065	5.892384E-01
Gm11929	7799		#N/A	1.000000E+00
Gm11930	7800	0.221357237	18941	1.632638E-03
Gm11931	7801		#N/A	1.000000E+00
Gm11932	7802		#N/A	1.000000E+00
Gm11933	7803		#N/A	1.000000E+00
Gm11934	7804	-0.086750697	472.5	2.219141E-01
Gm11935	7805	-0.038406171	3065	5.892384E-01
Gm11936	7806		#N/A	1.000000E+00
Gm11937	7807		#N/A	1.000000E+00
Gm11938	7808		#N/A	1.000000E+00
Gm11939	7809		#N/A	1.000000E+00
Gm11940	7810		#N/A	1.000000E+00
Gm11941	7811		#N/A	1.000000E+00
Gm11942	7812	0.237088581	19311	7.242054E-04
Gm11943	7813		#N/A	1.000000E+00
Gm11944	7814	-0.001611344	5093	9.819334E-01
Gm11945	7815	0.061066885	9265	3.903377E-01
Gm11946	7816		#N/A	1.000000E+00
Gm11947	7817		#N/A	1.000000E+00
Gm11948	7818		#N/A	1.000000E+00
Gm11949	7819		#N/A	1.000000E+00
Gm11950	7820		#N/A	1.000000E+00
Gm11951	7821		#N/A	1.000000E+00
Gm11952	7822		#N/A	1.000000E+00
Gm11953	7823		#N/A	1.000000E+00
Gm11954	7824		#N/A	1.000000E+00
Gm11955	7825		#N/A	1.000000E+00

Spearman Rank correlation analysis performed between Prdm1 and all-expressed genes within the Meredith RNA-seq dataset. Robust Prdm1–associated genes were identified using a cut-off of $p < 0.0005$.

Table S1, Related to Supplemental Figure 3C. Prdm1 associated genes

Gm11956	7826	0.003512661	5499	9.606282E-01
Gm11957	7827		#N/A	1.000000E+00
Gm11958	7828		#N/A	1.000000E+00
Gm11959	7829		#N/A	1.000000E+00
Gm11960	7830		#N/A	1.000000E+00
Gm11961	7831		#N/A	1.000000E+00
Gm11962	7832		#N/A	1.000000E+00
Gm11963	7833	0.075715395	10317.5	2.866063E-01
Gm11964	7834		#N/A	1.000000E+00
Gm11965	7835		#N/A	1.000000E+00
Gm11966	7836		#N/A	1.000000E+00
Gm11967	7837		#N/A	1.000000E+00
Gm11969	7838	-0.086750697	472.5	2.219141E-01
Gm11970	7839	0.12046769	13690.5	8.928425E-02
Gm11971	7840		#N/A	1.000000E+00
Gm11972	7841	0.022080709	6593	7.562956E-01
Gm11973	7842	-0.002881543	5033	9.676978E-01
Gm11974	7843	0.075846361	10334	2.857711E-01
Gm11975	7844	0.135169958	14636.5	5.634245E-02
Gm11976	7845		#N/A	1.000000E+00
Gm11977	7846		#N/A	1.000000E+00
Gm11978	7847	-0.103163388	239	1.460330E-01
Gm11979	7848		#N/A	1.000000E+00
Gm11980	7849		#N/A	1.000000E+00
Gm11981	7850	0.143605682	15224	4.249002E-02
Gm11982	7851		#N/A	1.000000E+00
Gm11984	7852		#N/A	1.000000E+00
Gm11985	7853		#N/A	1.000000E+00
Gm11986	7854	0.090858474	11420.5	2.007115E-01
Gm11987	7855		#N/A	1.000000E+00
Gm11988	7856		#N/A	1.000000E+00
Gm11989	7857	-0.054451513	1703	4.437940E-01
Gm11990	7858	-0.054451513	1703	4.437940E-01
Gm11991	7859		#N/A	1.000000E+00
Gm11992	7860	-0.066856562	1139	3.468991E-01
Gm11993	7861		#N/A	1.000000E+00
Gm11995	7862		#N/A	1.000000E+00
Gm11996	7863	0.172715298	17063	1.445869E-02
Gm11997	7864	0.046025613	8011	5.175260E-01
Gm11998	7865	0.013164497	5942.5	8.532171E-01
Gm11999	7866	0.042126718	7748	5.536561E-01
Gm12	7867		#N/A	1.000000E+00
Gm12000	7868		#N/A	1.000000E+00

Spearman Rank correlation analysis performed between Prdm1 and all-expressed genes within the Meredith RNA-seq dataset. Robust Prdm1–associated genes were identified using a cut-off of $p < 0.0005$.

Table S1, Related to Supplemental Figure 3C. Prdm1 associated genes

Gm12001	7869		#N/A	1.000000E+00
Gm12002	7870	0.109492511	12666.5	1.227347E-01
Gm12003	7871	-0.038406171	3065	5.892384E-01
Gm12004	7872		#N/A	1.000000E+00
Gm12005	7873	-0.066858259	1029.5	3.468868E-01
Gm12006	7874		#N/A	1.000000E+00
Gm12007	7875		#N/A	1.000000E+00
Gm12008	7876		#N/A	1.000000E+00
Gm12009	7877		#N/A	1.000000E+00
Gm12010	7878	0.086412184	11087.5	2.237298E-01
Gm12011	7879	0.091324416	11473	1.984025E-01
Gm12012	7880		#N/A	1.000000E+00
Gm12013	7881	-0.043015135	2414	5.453166E-01
Gm12014	7882		#N/A	1.000000E+00
Gm12015	7883		#N/A	1.000000E+00
Gm12016	7884	-0.066856562	1139	3.468991E-01
Gm12017	7885	-0.038406171	3065	5.892384E-01
Gm12018	7886		#N/A	1.000000E+00
Gm12019	7887		#N/A	1.000000E+00
Gm12020	7888	0.156129434	16056.5	2.726377E-02
Gm12021	7889		#N/A	1.000000E+00
Gm12022	7890		#N/A	1.000000E+00
Gm12023	7891	0.122365833	13808	8.431862E-02
Gm12024	7892		#N/A	1.000000E+00
Gm12025	7893	0.111610737	12827	1.156168E-01
Gm12026	7894		#N/A	1.000000E+00
Gm12027	7895		#N/A	1.000000E+00
Gm12028	7896		#N/A	1.000000E+00
Gm12029	7897	0.164478601	16623.5	1.994547E-02
Gm12030	7898		#N/A	1.000000E+00
Gm12031	7899		#N/A	1.000000E+00
Gm12033	7900		#N/A	1.000000E+00
Gm12034	7901	-0.054451513	1703	4.437940E-01
Gm12035	7902	0.090858474	11420.5	2.007115E-01
Gm12036	7903		#N/A	1.000000E+00
Gm12037	7904		#N/A	1.000000E+00
Gm12038	7905	0.018446509	6243	7.954347E-01
Gm12039	7906	0.1544596	15936.5	2.897553E-02
Gm12040	7907		#N/A	1.000000E+00
Gm12041	7908		#N/A	1.000000E+00
Gm12042	7909		#N/A	1.000000E+00
Gm12043	7910		#N/A	1.000000E+00
Gm12044	7911		#N/A	1.000000E+00

Spearman Rank correlation analysis performed between Prdm1 and all-expressed genes within the Meredith RNA-seq dataset. Robust Prdm1–associated genes were identified using a cut-off of $p < 0.0005$.

Table S1, Related to Supplemental Figure 3C. Prdm1 associated genes

Gm12045	7912		#N/A	1.000000E+00
Gm12046	7913		#N/A	1.000000E+00
Gm12047	7914		#N/A	1.000000E+00
Gm12050	7915		#N/A	1.000000E+00
Gm12051	7916	-0.018447724	4312.5	7.954215E-01
Gm12052	7917		#N/A	1.000000E+00
Gm12053	7918	0.070871548	9903	3.186445E-01
Gm12054	7919		#N/A	1.000000E+00
Gm12055	7920		#N/A	1.000000E+00
Gm12056	7921		#N/A	1.000000E+00
Gm12057	7922	0.107050601	12499	1.313566E-01
Gm12058	7923		#N/A	1.000000E+00
Gm12059	7924	0.127284236	14137	7.247853E-02
Gm12060	7925	0.261703377	19645	1.816216E-04
Gm12061	7926		#N/A	1.000000E+00
Gm12062	7927	-0.066858259	1029.5	3.468868E-01
Gm12063	7928		#N/A	1.000000E+00
Gm12064	7929	0.113548679	13094	1.093908E-01
Gm12065	7930		#N/A	1.000000E+00
Gm12066	7931		#N/A	1.000000E+00
Gm12067	7932		#N/A	1.000000E+00
Gm12068	7933	0.090716057	11404	2.014212E-01
Gm12069	7934	-0.005348205	4909.5	9.400862E-01
Gm12070	7935	0.173314327	17093	1.411695E-02
Gm12071	7936		#N/A	1.000000E+00
Gm12072	7937		#N/A	1.000000E+00
Gm12073	7938		#N/A	1.000000E+00
Gm12074	7939	-0.054451513	1703	4.437940E-01
Gm12075	7940	-0.038406171	3065	5.892384E-01
Gm12077	7941		#N/A	1.000000E+00
Gm12079	7942		#N/A	1.000000E+00
Gm12080	7943		#N/A	1.000000E+00
Gm12081	7944	-0.038406171	3065	5.892384E-01
Gm12082	7945	-0.005348232	4906.5	9.400859E-01
Gm12084	7946		#N/A	1.000000E+00
Gm12085	7947	0.021317126	6513.5	7.644715E-01
Gm12086	7948		#N/A	1.000000E+00
Gm12087	7949	-0.038406171	3065	5.892384E-01
Gm12088	7950		#N/A	1.000000E+00
Gm12089	7951	0.054640123	8904	4.422146E-01
Gm12090	7952	0.045056653	7941.5	5.263908E-01
Gm12091	7953	0.22313283	18990	1.493553E-03
Gm12092	7954	-0.018789992	4291.5	7.917119E-01

Spearman Rank correlation analysis performed between Prdm1 and all-expressed genes within the Meredith RNA-seq dataset. Robust Prdm1–associated genes were identified using a cut-off of $p < 0.0005$.

Table S1, Related to Supplemental Figure 3C. Prdm1 associated genes

Gm12093	7955		#N/A	1.000000E+00
Gm12094	7956	0.082269134	10768	2.468098E-01
Gm12095	7957		#N/A	1.000000E+00
Gm12096	7958		#N/A	1.000000E+00
Gm12097	7959		#N/A	1.000000E+00
Gm12098	7960	-0.054451513	1703	4.437940E-01
Gm12099	7961		#N/A	1.000000E+00
Gm12100	7962		#N/A	1.000000E+00
Gm12101	7963		#N/A	1.000000E+00
Gm12102	7964		#N/A	1.000000E+00
Gm12103	7965		#N/A	1.000000E+00
Gm12104	7966		#N/A	1.000000E+00
Gm12105	7967		#N/A	1.000000E+00
Gm12106	7968	-0.054450825	1984	4.437997E-01
Gm12107	7969		#N/A	1.000000E+00
Gm12108	7970	0.00030705	5316.5	9.965570E-01
Gm12109	7971		#N/A	1.000000E+00
Gm12110	7972	-0.054451513	1703	4.437940E-01
Gm12111	7973	-0.013118278	4616	8.537267E-01
Gm12112	7974		#N/A	1.000000E+00
Gm12114	7975		#N/A	1.000000E+00
Gm12115	7976	-0.054451513	1703	4.437940E-01
Gm12116	7977	-0.066856562	1139	3.468991E-01
Gm12117	7978		#N/A	1.000000E+00
Gm12118	7979	-0.038406171	3065	5.892384E-01
Gm12119	7980		#N/A	1.000000E+00
Gm12120	7981		#N/A	1.000000E+00
Gm12121	7982	-0.038406171	3065	5.892384E-01
Gm12122	7983		#N/A	1.000000E+00
Gm12123	7984	-0.038406171	3065	5.892384E-01
Gm12124	7985		#N/A	1.000000E+00
Gm12125	7986		#N/A	1.000000E+00
Gm12126	7987		#N/A	1.000000E+00
Gm12127	7988		#N/A	1.000000E+00
Gm12128	7989		#N/A	1.000000E+00
Gm12129	7990		#N/A	1.000000E+00
Gm12130	7991	-0.054451513	1703	4.437940E-01
Gm12131	7992		#N/A	1.000000E+00
Gm12132	7993	0.05961818	9191	4.016995E-01
Gm12133	7994		#N/A	1.000000E+00
Gm12134	7995		#N/A	1.000000E+00
Gm12135	7996		#N/A	1.000000E+00
Gm12136	7997		#N/A	1.000000E+00

Spearman Rank correlation analysis performed between Prdm1 and all-expressed genes within the Meredith RNA-seq dataset. Robust Prdm1–associated genes were identified using a cut-off of $p < 0.0005$.

Table S1, Related to Supplemental Figure 3C. Prdm1 associated genes

Gm12137	7998		#N/A	1.000000E+00
Gm12138	7999		#N/A	1.000000E+00
Gm12139	8000		#N/A	1.000000E+00
Gm12140	8001	0.113548679	13094	1.093908E-01
Gm12141	8002	-0.001224019	5151	9.862757E-01
Gm12142	8003		#N/A	1.000000E+00
Gm12143	8004		#N/A	1.000000E+00
Gm12144	8005		#N/A	1.000000E+00
Gm12145	8006	-0.103180568	220	1.459655E-01
Gm12146	8007	0.016115908	6130	8.208130E-01
Gm12147	8008	-0.066856562	1139	3.468991E-01
Gm12148	8009		#N/A	1.000000E+00
Gm12149	8010		#N/A	1.000000E+00
Gm12150	8011		#N/A	1.000000E+00
Gm12151	8012		#N/A	1.000000E+00
Gm12152	8013	0.212474793	18672.5	2.522920E-03
Gm12153	8014	-0.018105422	4347	7.991361E-01
Gm12154	8015		#N/A	1.000000E+00
Gm12155	8016	0.18247897	17528	9.703550E-03
Gm12156	8017		#N/A	1.000000E+00
Gm12157	8018		#N/A	1.000000E+00
Gm12158	8019	-0.038406171	3065	5.892384E-01
Gm12159	8020		#N/A	1.000000E+00
Gm12160	8021	0.188804702	17792.5	7.417613E-03
Gm12161	8022		#N/A	1.000000E+00
Gm12162	8023	0.098009372	11916.5	1.673711E-01
Gm12163	8024	0.150673323	15691	3.319973E-02
Gm12164	8025		#N/A	1.000000E+00
Gm12165	8026	-0.066858259	1029.5	3.468868E-01
Gm12166	8027	0.035463419	7346	6.181023E-01
Gm12167	8028	-0.066855996	1239.5	3.469032E-01
Gm12168	8029		#N/A	1.000000E+00
Gm12169	8030		#N/A	1.000000E+00
Gm12170	8031		#N/A	1.000000E+00
Gm12171	8032	-0.038406171	3065	5.892384E-01
Gm12173	8033		#N/A	1.000000E+00
Gm12174	8034		#N/A	1.000000E+00
Gm12175	8035		#N/A	1.000000E+00
Gm12176	8036	0.082269134	10768	2.468098E-01
Gm12177	8037		#N/A	1.000000E+00
Gm12178	8038		#N/A	1.000000E+00
Gm12179	8039		#N/A	1.000000E+00
Gm12180	8040		#N/A	1.000000E+00

Spearman Rank correlation analysis performed between Prdm1 and all-expressed genes within the Meredith RNA-seq dataset. Robust Prdm1–associated genes were identified using a cut-off of $p < 0.0005$.

Table S1, Related to Supplemental Figure 3C. Prdm1 associated genes

Gm12181	8041		#N/A	1.000000E+00
Gm12182	8042	-0.054451513	1703	4.437940E-01
Gm12183	8043	-0.038406171	3065	5.892384E-01
Gm12184	8044	0.08942406	11304	2.079420E-01
Gm12185	8045	0.115309956	13347	1.039633E-01
Gm12186	8046		#N/A	1.000000E+00
Gm12187	8047		#N/A	1.000000E+00
Gm12188	8048		#N/A	1.000000E+00
Gm12189	8049		#N/A	1.000000E+00
Gm12190	8050	0.156129434	16056.5	2.726377E-02
Gm12191	8051		#N/A	1.000000E+00
Gm12192	8052		#N/A	1.000000E+00
Gm12193	8053		#N/A	1.000000E+00
Gm12194	8054		#N/A	1.000000E+00
Gm12195	8055		#N/A	1.000000E+00
Gm12196	8056		#N/A	1.000000E+00
Gm12197	8057		#N/A	1.000000E+00
Gm12198	8058	-0.054451513	1703	4.437940E-01
Gm12199	8059		#N/A	1.000000E+00
Gm12200	8060		#N/A	1.000000E+00
Gm12201	8061	0.099105556	12004	1.626486E-01
Gm12202	8062	0.132263077	14430	6.190598E-02
Gm12203	8063		#N/A	1.000000E+00
Gm12204	8064		#N/A	1.000000E+00
Gm12205	8065		#N/A	1.000000E+00
Gm12206	8066		#N/A	1.000000E+00
Gm12207	8067		#N/A	1.000000E+00
Gm12208	8068		#N/A	1.000000E+00
Gm12209	8069		#N/A	1.000000E+00
Gm12210	8070		#N/A	1.000000E+00
Gm12212	8071		#N/A	1.000000E+00
Gm12213	8072		#N/A	1.000000E+00
Gm12214	8073	0.199994562	18242	4.520347E-03
Gm12216	8074	0.083884044	10911	2.376240E-01
Gm12217	8075		#N/A	1.000000E+00
Gm12218	8076	-0.066858259	1029.5	3.468868E-01
Gm12219	8077		#N/A	1.000000E+00
Gm12220	8078		#N/A	1.000000E+00
Gm12221	8079		#N/A	1.000000E+00
Gm12222	8080	-0.054450825	1984	4.437997E-01
Gm12223	8081		#N/A	1.000000E+00
Gm12224	8082		#N/A	1.000000E+00
Gm12225	8083		#N/A	1.000000E+00

Spearman Rank correlation analysis performed between Prdm1 and all-expressed genes within the Meredith RNA-seq dataset. Robust Prdm1–associated genes were identified using a cut-off of $p < 0.0005$.

Table S1, Related to Supplemental Figure 3C. Prdm1 associated genes

Gm12226	8084		#N/A	1.000000E+00
Gm12227	8085		#N/A	1.000000E+00
Gm12228	8086	-0.13063896	65	6.520596E-02
Gm12229	8087		#N/A	1.000000E+00
Gm12230	8088	-0.074665178	890	2.933624E-01
Gm12231	8089		#N/A	1.000000E+00
Gm12233	8090	0.112857834	12910	1.115794E-01
Gm12234	8091		#N/A	1.000000E+00
Gm12235	8092		#N/A	1.000000E+00
Gm12236	8093		#N/A	1.000000E+00
Gm12237	8094		#N/A	1.000000E+00
Gm12238	8095		#N/A	1.000000E+00
Gm12239	8096	0.136180045	14712	5.450868E-02
Gm12240	8097		#N/A	1.000000E+00
Gm12241	8098		#N/A	1.000000E+00
Gm12242	8099	-0.054451513	1703	4.437940E-01
Gm12243	8100		#N/A	1.000000E+00
Gm12244	8101		#N/A	1.000000E+00
Gm12245	8102		#N/A	1.000000E+00
Gm12246	8103		#N/A	1.000000E+00
Gm12247	8104		#N/A	1.000000E+00
Gm12248	8105	-0.038406171	3065	5.892384E-01
Gm12249	8106		#N/A	1.000000E+00
Gm12250	8107	0.144927158	15351	4.060358E-02
Gm12251	8108		#N/A	1.000000E+00
Gm12252	8109		#N/A	1.000000E+00
Gm12253	8110	0.01536649	6091	8.290146E-01
Gm12254	8111	0.020434681	6357	7.739526E-01
Gm12255	8112		#N/A	1.000000E+00
Gm12256	8113		#N/A	1.000000E+00
Gm12257	8114		#N/A	1.000000E+00
Gm12258	8115	-0.038406171	3065	5.892384E-01
Gm12259	8116	0.053267784	8685	4.537786E-01
Gm12260	8117		#N/A	1.000000E+00
Gm12261	8118	0.230247091	19167.5	1.038230E-03
Gm12262	8119		#N/A	1.000000E+00
Gm12263	8120		#N/A	1.000000E+00
Gm12264	8121	0.128238977	14195	7.034469E-02
Gm12265	8122		#N/A	1.000000E+00
Gm12266	8123		#N/A	1.000000E+00
Gm12267	8124	0.157896862	16170	2.554704E-02
Gm12268	8125		#N/A	1.000000E+00
Gm12269	8126		#N/A	1.000000E+00

Spearman Rank correlation analysis performed between Prdm1 and all-expressed genes within the Meredith RNA-seq dataset. Robust Prdm1–associated genes were identified using a cut-off of $p < 0.0005$.

Table S1, Related to Supplemental Figure 3C. Prdm1 associated genes

Gm12271	8127		#N/A	1.000000E+00
Gm12272	8128		#N/A	1.000000E+00
Gm12273	8129		#N/A	1.000000E+00
Gm12274	8130		#N/A	1.000000E+00
Gm12275	8131		#N/A	1.000000E+00
Gm12276	8132	-0.054450825	1984	4.437997E-01
Gm12278	8133		#N/A	1.000000E+00
Gm12279	8134	0.052728522	8550.5	4.583686E-01
Gm12280	8135		#N/A	1.000000E+00
Gm12281	8136		#N/A	1.000000E+00
Gm12282	8137		#N/A	1.000000E+00
Gm12283	8138		#N/A	1.000000E+00
Gm12284	8139		#N/A	1.000000E+00
Gm12285	8140	0.117779503	13513	9.671354E-02
Gm12286	8141	-0.066858259	1029.5	3.468868E-01
Gm12287	8142		#N/A	1.000000E+00
Gm12288	8143		#N/A	1.000000E+00
Gm12289	8144		#N/A	1.000000E+00
Gm12290	8145	0.113548679	13094	1.093908E-01
Gm12291	8146		#N/A	1.000000E+00
Gm12292	8147	-0.038406171	3065	5.892384E-01
Gm12293	8148		#N/A	1.000000E+00
Gm12294	8149	0.149612093	15625	3.447360E-02
Gm12295	8150	0.099014893	11995.5	1.630354E-01
Gm12296	8151	0.138431037	14853	5.059940E-02
Gm12297	8152		#N/A	1.000000E+00
Gm12298	8153	0.133384855	14503	5.970775E-02
Gm12299	8154		#N/A	1.000000E+00
Gm12300	8155		#N/A	1.000000E+00
Gm12301	8156	-0.038406171	3065	5.892384E-01
GM12302	8157		#N/A	1.000000E+00
Gm12302	8158		#N/A	1.000000E+00
Gm12303	8159		#N/A	1.000000E+00
Gm12304	8160	0.074574898	10201.5	2.939481E-01
Gm12305	8161		#N/A	1.000000E+00
Gm12306	8162		#N/A	1.000000E+00
Gm12307	8163		#N/A	1.000000E+00
Gm12308	8164	-0.038406171	3065	5.892384E-01
Gm12309	8165		#N/A	1.000000E+00
Gm12310	8166		#N/A	1.000000E+00
Gm12311	8167	0.021317126	6513.5	7.644715E-01
Gm12312	8168		#N/A	1.000000E+00
Gm12313	8169		#N/A	1.000000E+00

Spearman Rank correlation analysis performed between Prdm1 and all-expressed genes within the Meredith RNA-seq dataset. Robust Prdm1–associated genes were identified using a cut-off of $p < 0.0005$.

Table S1, Related to Supplemental Figure 3C. Prdm1 associated genes

Gm12314	8170		#N/A	1.000000E+00
Gm12315	8171	-0.038406171	3065	5.892384E-01
Gm12316	8172		#N/A	1.000000E+00
Gm12317	8173	0.083452862	10867	2.400529E-01
Gm12318	8174	0.119194027	13599.5	9.274531E-02
Gm12319	8175		#N/A	1.000000E+00
Gm12320	8176	-0.09526621	368.5	1.796359E-01
Gm12321	8177		#N/A	1.000000E+00
Gm12322	8178		#N/A	1.000000E+00
Gm12324	8179	0.113548679	13094	1.093908E-01
Gm12325	8180		#N/A	1.000000E+00
Gm12326	8181		#N/A	1.000000E+00
Gm12327	8182		#N/A	1.000000E+00
Gm12328	8183	0.069653266	9825	3.270553E-01
Gm12329	8184		#N/A	1.000000E+00
Gm12331	8185		#N/A	1.000000E+00
Gm12332	8186	0.143605682	15224	4.249002E-02
Gm12333	8187		#N/A	1.000000E+00
Gm12335	8188		#N/A	1.000000E+00
Gm12336	8189		#N/A	1.000000E+00
Gm12337	8190	-0.038406171	3065	5.892384E-01
Gm12338	8191	0.204453557	18406	3.683977E-03
Gm12339	8192	0.119528081	13628	9.182737E-02
Gm12340	8193		#N/A	1.000000E+00
Gm12341	8194	0.162808768	16526.5	2.125512E-02
Gm12343	8195		#N/A	1.000000E+00
Gm12344	8196		#N/A	1.000000E+00
Gm12345	8197		#N/A	1.000000E+00
Gm12346	8198	0.029119575	7023	6.823059E-01
Gm12347	8199		#N/A	1.000000E+00
Gm12348	8200		#N/A	1.000000E+00
Gm12349	8201	-0.066855996	1239.5	3.469032E-01
Gm12350	8202		#N/A	1.000000E+00
Gm12351	8203	0.054596945	8902	4.425759E-01
Gm12352	8204	0.162808768	16526.5	2.125512E-02
Gm12353	8205	-0.067176455	976	3.445915E-01
Gm12354	8206		#N/A	1.000000E+00
Gm12355	8207	0.11060705	12766	1.189483E-01
Gm12356	8208		#N/A	1.000000E+00
Gm12357	8209	0.119194027	13599.5	9.274531E-02
Gm12358	8210		#N/A	1.000000E+00
Gm12359	8211	0.133870184	14542	5.877682E-02
Gm12360	8212	-0.001224019	5151	9.862757E-01

Spearman Rank correlation analysis performed between Prdm1 and all-expressed genes within the Meredith RNA-seq dataset. Robust Prdm1–associated genes were identified using a cut-off of $p < 0.0005$.

Table S1, Related to Supplemental Figure 3C. Prdm1 associated genes

Gm12361	8213	-0.038406171	3065	5.892384E-01
Gm12362	8214		#N/A	1.000000E+00
Gm12363	8215		#N/A	1.000000E+00
Gm12364	8216		#N/A	1.000000E+00
Gm12365	8217		#N/A	1.000000E+00
Gm12366	8218		#N/A	1.000000E+00
Gm12367	8219	0.02650298	6902.5	7.095021E-01
Gm12368	8220		#N/A	1.000000E+00
Gm12369	8221		#N/A	1.000000E+00
Gm12370	8222		#N/A	1.000000E+00
Gm12371	8223	0.039898539	7638	5.748403E-01
Gm12372	8224		#N/A	1.000000E+00
Gm12374	8225	-0.117606715	112	9.720737E-02
Gm12375	8226		#N/A	1.000000E+00
Gm12376	8227		#N/A	1.000000E+00
Gm12377	8228		#N/A	1.000000E+00
Gm12378	8229		#N/A	1.000000E+00
Gm12379	8230	0.081085405	10671.5	2.536978E-01
Gm12380	8231	-0.038406171	3065	5.892384E-01
Gm12381	8232	0.207334017	18507	3.220734E-03
Gm12382	8233		#N/A	1.000000E+00
Gm12383	8234		#N/A	1.000000E+00
Gm12384	8235		#N/A	1.000000E+00
Gm12385	8236		#N/A	1.000000E+00
Gm12386	8237		#N/A	1.000000E+00
Gm12387	8238		#N/A	1.000000E+00
Gm12388	8239	-0.038406171	3065	5.892384E-01
Gm12389	8240		#N/A	1.000000E+00
Gm12390	8241	-0.038406171	3065	5.892384E-01
Gm12391	8242	-0.054451513	1703	4.437940E-01
Gm12392	8243		#N/A	1.000000E+00
Gm12393	8244		#N/A	1.000000E+00
Gm12394	8245	-0.023525528	4137	7.409001E-01
Gm12395	8246		#N/A	1.000000E+00
Gm12396	8247	0.000286338	5305	9.967893E-01
Gm12397	8248		#N/A	1.000000E+00
Gm12398	8249		#N/A	1.000000E+00
Gm12399	8250	0.166148435	16721.5	1.870631E-02
Gm12400	8251		#N/A	1.000000E+00
Gm12401	8252	0.113548679	13094	1.093908E-01
Gm12402	8253		#N/A	1.000000E+00
Gm12403	8254		#N/A	1.000000E+00
Gm12404	8255		#N/A	1.000000E+00

Spearman Rank correlation analysis performed between Prdm1 and all-expressed genes within the Meredith RNA-seq dataset. Robust Prdm1–associated genes were identified using a cut-off of $p < 0.0005$.

Table S1, Related to Supplemental Figure 3C. Prdm1 associated genes

Gm12405	8256		#N/A	1.000000E+00
Gm12406	8257	-0.038406171	3065	5.892384E-01
Gm12407	8258	-0.038406171	3065	5.892384E-01
Gm12408	8259	-0.077394997	736.5	2.760195E-01
Gm12409	8260		#N/A	1.000000E+00
Gm12410	8261		#N/A	1.000000E+00
Gm12411	8262		#N/A	1.000000E+00
Gm12412	8263	0.152789767	15822	3.077826E-02
Gm12413	8264		#N/A	1.000000E+00
Gm12414	8265		#N/A	1.000000E+00
Gm12415	8266		#N/A	1.000000E+00
Gm12416	8267	0.014301797	6015.5	8.406982E-01
Gm12417	8268		#N/A	1.000000E+00
Gm12418	8269		#N/A	1.000000E+00
Gm12419	8270		#N/A	1.000000E+00
Gm12420	8271		#N/A	1.000000E+00
Gm12421	8272		#N/A	1.000000E+00
Gm12422	8273		#N/A	1.000000E+00
Gm12423	8274		#N/A	1.000000E+00
Gm12424	8275		#N/A	1.000000E+00
Gm12425	8276		#N/A	1.000000E+00
Gm12426	8277		#N/A	1.000000E+00
Gm12427	8278		#N/A	1.000000E+00
Gm12428	8279		#N/A	1.000000E+00
Gm12429	8280		#N/A	1.000000E+00
Gm12430	8281	0.210141715	18577	2.820559E-03
Gm12431	8282		#N/A	1.000000E+00
Gm12432	8283		#N/A	1.000000E+00
Gm12433	8284		#N/A	1.000000E+00
Gm12434	8285		#N/A	1.000000E+00
Gm12435	8286		#N/A	1.000000E+00
Gm12436	8287	-0.000505063	5241	9.943367E-01
Gm12437	8288		#N/A	1.000000E+00
Gm12438	8289		#N/A	1.000000E+00
Gm12439	8290		#N/A	1.000000E+00
Gm12440	8291	-0.054450825	1984	4.437997E-01
Gm12441	8292		#N/A	1.000000E+00
Gm12442	8293		#N/A	1.000000E+00
Gm12443	8294	-0.054450825	1984	4.437997E-01
Gm12444	8295		#N/A	1.000000E+00
Gm12445	8296		#N/A	1.000000E+00
Gm12446	8297	0.090373357	11373	2.031362E-01
Gm12447	8298		#N/A	1.000000E+00

Spearman Rank correlation analysis performed between Prdm1 and all-expressed genes within the Meredith RNA-seq dataset. Robust Prdm1–associated genes were identified using a cut-off of $p < 0.0005$.

Table S1, Related to Supplemental Figure 3C. Prdm1 associated genes

Gm12448	8299		#N/A	1.000000E+00
Gm12449	8300		#N/A	1.000000E+00
Gm12450	8301		#N/A	1.000000E+00
Gm12451	8302	0.045449022	7964	5.227918E-01
Gm12452	8303		#N/A	1.000000E+00
Gm12453	8304		#N/A	1.000000E+00
Gm12454	8305	0.013727565	5977	8.470143E-01
Gm12455	8306		#N/A	1.000000E+00
Gm12457	8307		#N/A	1.000000E+00
Gm12458	8308	-0.054451513	1703	4.437940E-01
Gm12459	8309		#N/A	1.000000E+00
Gm12460	8310	-0.066858259	1029.5	3.468868E-01
Gm12461	8311	0.144811278	15343	4.076613E-02
Gm12462	8312	0.031945794	7184	6.533850E-01
Gm12463	8313	-0.038406171	3065	5.892384E-01
Gm12464	8314		#N/A	1.000000E+00
Gm12465	8315		#N/A	1.000000E+00
Gm12466	8316	-0.000841282	5208.5	9.905669E-01
Gm12467	8317		#N/A	1.000000E+00
Gm12468	8318	0.121478848	13752	8.661069E-02
Gm12469	8319		#N/A	1.000000E+00
Gm12470	8320		#N/A	1.000000E+00
Gm12472	8321	-0.033388476	3771	6.388155E-01
Gm12473	8322	0.053267784	8685	4.537786E-01
Gm12474	8323	-0.038406171	3065	5.892384E-01
Gm12475	8324		#N/A	1.000000E+00
Gm12477	8325		#N/A	1.000000E+00
Gm12478	8326		#N/A	1.000000E+00
Gm12479	8327		#N/A	1.000000E+00
Gm12480	8328		#N/A	1.000000E+00
Gm12481	8329	0.078366084	10491	2.700209E-01
Gm12482	8330		#N/A	1.000000E+00
Gm12483	8331		#N/A	1.000000E+00
Gm12484	8332		#N/A	1.000000E+00
Gm12485	8333		#N/A	1.000000E+00
Gm12486	8334		#N/A	1.000000E+00
Gm12488	8335		#N/A	1.000000E+00
Gm12489	8336		#N/A	1.000000E+00
Gm12490	8337		#N/A	1.000000E+00
Gm12492	8338	0.053267784	8685	4.537786E-01
Gm12493	8339	-0.038406171	3065	5.892384E-01
Gm12494	8340	-0.038406171	3065	5.892384E-01
Gm12495	8341		#N/A	1.000000E+00

Spearman Rank correlation analysis performed between Prdm1 and all-expressed genes within the Meredith RNA-seq dataset. Robust Prdm1–associated genes were identified using a cut-off of $p < 0.0005$.

Table S1, Related to Supplemental Figure 3C. Prdm1 associated genes

Gm12496	8342		#N/A	1.000000E+00
Gm12497	8343	-0.038406171	3065	5.892384E-01
Gm12498	8344		#N/A	1.000000E+00
Gm12499	8345		#N/A	1.000000E+00
Gm12500	8346		#N/A	1.000000E+00
Gm12501	8347		#N/A	1.000000E+00
Gm12502	8348		#N/A	1.000000E+00
Gm12503	8349		#N/A	1.000000E+00
Gm12504	8350	0.154605914	15960	2.882196E-02
Gm12505	8351	0.113548679	13094	1.093908E-01
Gm12506	8352		#N/A	1.000000E+00
Gm12507	8353		#N/A	1.000000E+00
Gm12508	8354		#N/A	1.000000E+00
Gm12509	8355		#N/A	1.000000E+00
Gm12510	8356	-0.014844367	4517.5	8.347397E-01
Gm12511	8357	-0.038406171	3065	5.892384E-01
Gm12512	8358		#N/A	1.000000E+00
Gm12513	8359		#N/A	1.000000E+00
Gm12514	8360	0.064506649	9506	3.641481E-01
Gm12515	8361		#N/A	1.000000E+00
Gm12516	8362	-0.066858259	1029.5	3.468868E-01
Gm12517	8363	0.065421341	9556	3.573717E-01
Gm12518	8364	-0.038406171	3065	5.892384E-01
Gm12519	8365	0.113548679	13094	1.093908E-01
Gm12520	8366		#N/A	1.000000E+00
Gm12521	8367		#N/A	1.000000E+00
Gm12522	8368	-0.012506891	4645	8.604728E-01
Gm12523	8369		#N/A	1.000000E+00
Gm12524	8370	-0.014844367	4517.5	8.347397E-01
Gm12525	8371		#N/A	1.000000E+00
Gm12526	8372		#N/A	1.000000E+00
Gm12527	8373		#N/A	1.000000E+00
Gm12529	8374		#N/A	1.000000E+00
Gm12530	8375	-0.086747139	513	2.219331E-01
Gm12531	8376	0.179282898	17371	1.107999E-02
Gm12532	8377	-0.066856562	1139	3.468991E-01
Gm12534	8378		#N/A	1.000000E+00
Gm12535	8379		#N/A	1.000000E+00
Gm12536	8380	-0.038406171	3065	5.892384E-01
Gm12537	8381		#N/A	1.000000E+00
Gm12538	8382		#N/A	1.000000E+00
Gm12539	8383		#N/A	1.000000E+00
Gm12540	8384		#N/A	1.000000E+00

Spearman Rank correlation analysis performed between Prdm1 and all-expressed genes within the Meredith RNA-seq dataset. Robust Prdm1–associated genes were identified using a cut-off of $p < 0.0005$.

Table S1, Related to Supplemental Figure 3C. Prdm1 associated genes

Gm12541	8385		#N/A	1.000000E+00
Gm12542	8386		#N/A	1.000000E+00
Gm12543	8387		#N/A	1.000000E+00
Gm12550	8388		#N/A	1.000000E+00
Gm12551	8389		#N/A	1.000000E+00
Gm12564	8390		#N/A	1.000000E+00
Gm12565	8391		#N/A	1.000000E+00
Gm12566	8392		#N/A	1.000000E+00
Gm12567	8393		#N/A	1.000000E+00
Gm12568	8394		#N/A	1.000000E+00
Gm12569	8395		#N/A	1.000000E+00
Gm12570	8396	0.113548679	13094	1.093908E-01
Gm12571	8397		#N/A	1.000000E+00
Gm12572	8398		#N/A	1.000000E+00
Gm12573	8399		#N/A	1.000000E+00
Gm12574	8400		#N/A	1.000000E+00
Gm12575	8401		#N/A	1.000000E+00
Gm12576	8402	0.053267784	8685	4.537786E-01
Gm12577	8403		#N/A	1.000000E+00
Gm12578	8404	-0.038406171	3065	5.892384E-01
Gm12579	8405		#N/A	1.000000E+00
Gm12580	8406	0.194131481	17998	5.878558E-03
Gm12582	8407	0.143605682	15224	4.249002E-02
Gm12583	8408		#N/A	1.000000E+00
Gm12584	8409		#N/A	1.000000E+00
Gm12585	8410		#N/A	1.000000E+00
Gm12586	8411		#N/A	1.000000E+00
Gm12587	8412		#N/A	1.000000E+00
Gm12588	8413	0.143605682	15224	4.249002E-02
Gm12589	8414	-0.001224019	5151	9.862757E-01
Gm12590	8415		#N/A	1.000000E+00
Gm12591	8416		#N/A	1.000000E+00
Gm12592	8417	0.082273309	10774	2.467857E-01
Gm12593	8418	0.207920483	18520	3.133136E-03
Gm12594	8419		#N/A	1.000000E+00
Gm12596	8420		#N/A	1.000000E+00
Gm12597	8421		#N/A	1.000000E+00
Gm12598	8422		#N/A	1.000000E+00
Gm12599	8423	0.152789767	15822	3.077826E-02
Gm12600	8424		#N/A	1.000000E+00
Gm12601	8425		#N/A	1.000000E+00
Gm12602	8426	-0.038406171	3065	5.892384E-01
Gm12603	8427	-0.054451513	1703	4.437940E-01

Spearman Rank correlation analysis performed between Prdm1 and all-expressed genes within the Meredith RNA-seq dataset. Robust Prdm1–associated genes were identified using a cut-off of $p < 0.0005$.

Table S1, Related to Supplemental Figure 3C. Prdm1 associated genes

Gm12604	8428		#N/A	1.000000E+00
Gm12605	8429		#N/A	1.000000E+00
Gm12606	8430	0.167124971	16775	1.801306E-02
Gm12607	8431		#N/A	1.000000E+00
Gm12608	8432	0.03826864	7511	5.905735E-01
Gm12609	8433	-0.038406171	3065	5.892384E-01
Gm12610	8434	0.049260381	8241	4.884972E-01
Gm12611	8435		#N/A	1.000000E+00
Gm12612	8436		#N/A	1.000000E+00
Gm12613	8437		#N/A	1.000000E+00
Gm12614	8438		#N/A	1.000000E+00
Gm12615	8439		#N/A	1.000000E+00
Gm12616	8440		#N/A	1.000000E+00
Gm12617	8441	0.143605682	15224	4.249002E-02
Gm12618	8442	0.053267784	8685	4.537786E-01
Gm12619	8443		#N/A	1.000000E+00
Gm12620	8444		#N/A	1.000000E+00
Gm12621	8445		#N/A	1.000000E+00
Gm12622	8446		#N/A	1.000000E+00
Gm12623	8447		#N/A	1.000000E+00
Gm12624	8448		#N/A	1.000000E+00
Gm12625	8449		#N/A	1.000000E+00
Gm12626	8450		#N/A	1.000000E+00
Gm12627	8451		#N/A	1.000000E+00
Gm12628	8452	0.090858474	11420.5	2.007115E-01
Gm12629	8453		#N/A	1.000000E+00
Gm12630	8454	0.071667294	9964.5	3.132274E-01
Gm12631	8455		#N/A	1.000000E+00
Gm12632	8456		#N/A	1.000000E+00
Gm12633	8457		#N/A	1.000000E+00
Gm12634	8458	0.135864161	14688	5.507677E-02
Gm12635	8459		#N/A	1.000000E+00
Gm12636	8460		#N/A	1.000000E+00
Gm12637	8461		#N/A	1.000000E+00
Gm12638	8462		#N/A	1.000000E+00
Gm12639	8463		#N/A	1.000000E+00
Gm12640	8464		#N/A	1.000000E+00
Gm12641	8465	0.274487927	19751	8.377681E-05
Gm12642	8466	0.053267784	8685	4.537786E-01
Gm12643	8467	0.021317126	6513.5	7.644715E-01
Gm12644	8468		#N/A	1.000000E+00
Gm12645	8469		#N/A	1.000000E+00
Gm12646	8470		#N/A	1.000000E+00

Spearman Rank correlation analysis performed between Prdm1 and all-expressed genes within the Meredith RNA-seq dataset. Robust Prdm1–associated genes were identified using a cut-off of $p < 0.0005$.

Table S1, Related to Supplemental Figure 3C. Prdm1 associated genes

Gm12647	8471		#N/A	1.000000E+00
Gm12648	8472	-0.100891849	270	1.551678E-01
Gm12649	8473	0.057091801	9041	4.219780E-01
Gm12650	8474		#N/A	1.000000E+00
Gm12651	8475		#N/A	1.000000E+00
Gm12652	8476		#N/A	1.000000E+00
Gm12653	8477		#N/A	1.000000E+00
Gm12654	8478		#N/A	1.000000E+00
Gm12655	8479	0.077465926	10440	2.755784E-01
Gm12656	8480	-0.038406171	3065	5.892384E-01
Gm12657	8481	0.284666306	19831	4.400053E-05
Gm12658	8482	-0.001224019	5151	9.862757E-01
Gm12659	8483	-0.054450825	1984	4.437997E-01
Gm12660	8484		#N/A	1.000000E+00
Gm12661	8485		#N/A	1.000000E+00
Gm12663	8486		#N/A	1.000000E+00
Gm12664	8487		#N/A	1.000000E+00
Gm12665	8488		#N/A	1.000000E+00
Gm12666	8489		#N/A	1.000000E+00
Gm12667	8490		#N/A	1.000000E+00
Gm12668	8491		#N/A	1.000000E+00
Gm12669	8492		#N/A	1.000000E+00
Gm12670	8493		#N/A	1.000000E+00
Gm12671	8494		#N/A	1.000000E+00
Gm12673	8495		#N/A	1.000000E+00
Gm12674	8496		#N/A	1.000000E+00
Gm12675	8497		#N/A	1.000000E+00
Gm12676	8498		#N/A	1.000000E+00
Gm12677	8499		#N/A	1.000000E+00
Gm12678	8500		#N/A	1.000000E+00
Gm12679	8501	-0.038406171	3065	5.892384E-01
Gm12680	8502	0.159931226	16274	2.368670E-02
Gm12681	8503		#N/A	1.000000E+00
Gm12682	8504	-0.086746694	532	2.219355E-01
Gm12683	8505		#N/A	1.000000E+00
Gm12684	8506		#N/A	1.000000E+00
Gm12685	8507		#N/A	1.000000E+00
Gm12686	8508		#N/A	1.000000E+00
Gm12687	8509		#N/A	1.000000E+00
Gm12688	8510	-0.081059573	632	2.538496E-01
Gm12689	8511		#N/A	1.000000E+00
Gm12690	8512		#N/A	1.000000E+00
Gm12691	8513	0.06902692	9779.5	3.314346E-01

Spearman Rank correlation analysis performed between Prdm1 and all-expressed genes within the Meredith RNA-seq dataset. Robust Prdm1–associated genes were identified using a cut-off of $p < 0.0005$.

Table S1, Related to Supplemental Figure 3C. Prdm1 associated genes

Gm12692	8514		#N/A	1.000000E+00
Gm12693	8515	-0.054451513	1703	4.437940E-01
Gm12694	8516	-0.054450825	1984	4.437997E-01
Gm12695	8517	0.019738126	6316	7.814602E-01
Gm12696	8518	-0.038406171	3065	5.892384E-01
Gm12697	8519		#N/A	1.000000E+00
Gm12698	8520		#N/A	1.000000E+00
Gm12699	8521	0.099271295	12017.5	1.619434E-01
Gm12700	8522	-0.038406171	3065	5.892384E-01
Gm12701	8523		#N/A	1.000000E+00
Gm12702	8524		#N/A	1.000000E+00
Gm12703	8525	0.160987082	16412.5	2.276801E-02
Gm12704	8526		#N/A	1.000000E+00
Gm12705	8527	-0.054451513	1703	4.437940E-01
Gm12706	8528		#N/A	1.000000E+00
Gm12707	8529		#N/A	1.000000E+00
Gm12708	8530		#N/A	1.000000E+00
Gm12709	8531		#N/A	1.000000E+00
Gm12710	8532	0.113548679	13094	1.093908E-01
Gm12711	8533		#N/A	1.000000E+00
Gm12712	8534		#N/A	1.000000E+00
Gm12713	8535		#N/A	1.000000E+00
Gm12714	8536	0.176757831	17253	1.228636E-02
Gm12715	8537	0.049855249	8285.5	4.832553E-01
Gm12716	8538		#N/A	1.000000E+00
Gm12717	8539		#N/A	1.000000E+00
Gm12718	8540	-0.086748028	498.5	2.219283E-01
Gm12719	8541		#N/A	1.000000E+00
Gm12720	8542	-0.038406171	3065	5.892384E-01
Gm12721	8543		#N/A	1.000000E+00
Gm12722	8544		#N/A	1.000000E+00
Gm12723	8545		#N/A	1.000000E+00
Gm12724	8546	-0.066856562	1139	3.468991E-01
Gm12725	8547	0.072770281	10045	3.058188E-01
Gm12726	8548	-0.066858259	1029.5	3.468868E-01
Gm12727	8549		#N/A	1.000000E+00
Gm12728	8550	0.139045799	14892.5	4.957323E-02
Gm12729	8551		#N/A	1.000000E+00
Gm12730	8552		#N/A	1.000000E+00
Gm12731	8553		#N/A	1.000000E+00
Gm12732	8554	0.174446546	17144	1.349030E-02
Gm12733	8555		#N/A	1.000000E+00
Gm12734	8556		#N/A	1.000000E+00

Spearman Rank correlation analysis performed between Prdm1 and all-expressed genes within the Meredith RNA-seq dataset. Robust Prdm1–associated genes were identified using a cut-off of $p < 0.0005$.

Table S1, Related to Supplemental Figure 3C. Prdm1 associated genes

Gm12735	8557		#N/A	1.000000E+00
Gm12736	8558	-0.038406171	3065	5.892384E-01
Gm12737	8559		#N/A	1.000000E+00
Gm12738	8560	0.044087692	7873	5.353318E-01
Gm12739	8561	0.21857011	18859	1.874957E-03
Gm12740	8562		#N/A	1.000000E+00
Gm12741	8563	-0.038406171	3065	5.892384E-01
Gm12742	8564		#N/A	1.000000E+00
Gm12743	8565	0.064294989	9494	3.657275E-01
Gm12744	8566	0.111301969	12807	1.166338E-01
Gm12745	8567		#N/A	1.000000E+00
Gm12746	8568		#N/A	1.000000E+00
Gm12747	8569	-0.038406171	3065	5.892384E-01
Gm12748	8570	-0.054450825	1984	4.437997E-01
Gm12749	8571	0.113548679	13094	1.093908E-01
Gm12750	8572		#N/A	1.000000E+00
Gm12751	8573	0.113523015	12956	1.094715E-01
Gm12752	8574		#N/A	1.000000E+00
Gm12753	8575		#N/A	1.000000E+00
Gm12754	8576	0.021742267	6570	7.599161E-01
Gm12755	8577	-0.038406171	3065	5.892384E-01
Gm12756	8578		#N/A	1.000000E+00
Gm12757	8579	0.098384184	11948.5	1.657450E-01
Gm12758	8580	-0.077397959	691.5	2.760011E-01
Gm12760	8581	0.159393965	16248	2.416630E-02
Gm12762	8582	-0.038406171	3065	5.892384E-01
Gm12763	8583	-0.038406171	3065	5.892384E-01
Gm12764	8584	-0.054451513	1703	4.437940E-01
Gm12765	8585		#N/A	1.000000E+00
Gm12766	8586	-0.030877746	3889	6.642567E-01
Gm12768	8587		#N/A	1.000000E+00
Gm12770	8588		#N/A	1.000000E+00
Gm12771	8589		#N/A	1.000000E+00
Gm12773	8590		#N/A	1.000000E+00
Gm12774	8591		#N/A	1.000000E+00
Gm12778	8592	0.031352699	7148	6.594133E-01
Gm12779	8593		#N/A	1.000000E+00
Gm12780	8594	0.230235201	19161.5	1.038871E-03
Gm12781	8595	0.13472209	14604	5.717169E-02
Gm12782	8596		#N/A	1.000000E+00
Gm12783	8597	0.160304018	16323	2.335876E-02
Gm12784	8598	0.053805701	8820	4.492258E-01
Gm12785	8599		#N/A	1.000000E+00

Spearman Rank correlation analysis performed between Prdm1 and all-expressed genes within the Meredith RNA-seq dataset. Robust Prdm1–associated genes were identified using a cut-off of $p < 0.0005$.

Table S1, Related to Supplemental Figure 3C. Prdm1 associated genes

Gm12786	8600		#N/A	1.000000E+00
Gm12787	8601	0.113548679	13094	1.093908E-01
Gm12788	8602		#N/A	1.000000E+00
Gm12789	8603	-0.054451513	1703	4.437940E-01
Gm12790	8604		#N/A	1.000000E+00
Gm12791	8605		#N/A	1.000000E+00
Gm12792	8606		#N/A	1.000000E+00
Gm12793	8607		#N/A	1.000000E+00
Gm12794	8608		#N/A	1.000000E+00
Gm12795	8609	0.021317126	6513.5	7.644715E-01
Gm12796	8610	0.109488806	12652	1.227474E-01
Gm12797	8611		#N/A	1.000000E+00
Gm12798	8612		#N/A	1.000000E+00
Gm12799	8613		#N/A	1.000000E+00
Gm128	8614	0.113548679	13094	1.093908E-01
Gm12800	8615		#N/A	1.000000E+00
Gm12801	8616		#N/A	1.000000E+00
Gm12802	8617	0.192939236	17948	6.195767E-03
Gm12803	8618	-0.054450825	1984	4.437997E-01
Gm12804	8619		#N/A	1.000000E+00
Gm12805	8620		#N/A	1.000000E+00
Gm12806	8621		#N/A	1.000000E+00
Gm12807	8622	-0.054450825	1984	4.437997E-01
Gm12808	8623		#N/A	1.000000E+00
Gm12811	8624	0.186999719	17717	8.015191E-03
Gm12812	8625		#N/A	1.000000E+00
Gm12813	8626	0.346243231	20001	5.109858E-07
Gm12814	8627		#N/A	1.000000E+00
Gm12815	8628		#N/A	1.000000E+00
Gm12816	8629		#N/A	1.000000E+00
Gm12817	8630		#N/A	1.000000E+00
Gm12818	8631		#N/A	1.000000E+00
Gm12819	8632		#N/A	1.000000E+00
Gm12820	8633		#N/A	1.000000E+00
Gm12821	8634		#N/A	1.000000E+00
Gm12823	8635	-0.038406171	3065	5.892384E-01
Gm12825	8636		#N/A	1.000000E+00
Gm12826	8637		#N/A	1.000000E+00
Gm12827	8638		#N/A	1.000000E+00
Gm12828	8639		#N/A	1.000000E+00
Gm12829	8640	0.07521908	10268	2.897860E-01
Gm12830	8641		#N/A	1.000000E+00
Gm12831	8642		#N/A	1.000000E+00

Spearman Rank correlation analysis performed between Prdm1 and all-expressed genes within the Meredith RNA-seq dataset. Robust Prdm1–associated genes were identified using a cut-off of $p < 0.0005$.

Table S1, Related to Supplemental Figure 3C. Prdm1 associated genes

Gm12832	8643		#N/A	1.000000E+00
Gm12833	8644		#N/A	1.000000E+00
Gm12834	8645		#N/A	1.000000E+00
Gm12835	8646	0.157799267	16153.5	2.563935E-02
Gm12836	8647		#N/A	1.000000E+00
Gm12837	8648	-0.038406171	3065	5.892384E-01
Gm12838	8649	-0.038406171	3065	5.892384E-01
Gm12839	8650		#N/A	1.000000E+00
Gm12840	8651		#N/A	1.000000E+00
Gm12841	8652	0.171113116	16980	1.540854E-02
Gm12842	8653	0.221578254	18948	1.614703E-03
Gm12843	8654	-0.038406171	3065	5.892384E-01
Gm12844	8655		#N/A	1.000000E+00
Gm12845	8656		#N/A	1.000000E+00
Gm12846	8657	0.020875719	6412.5	7.692098E-01
Gm12847	8658		#N/A	1.000000E+00
Gm12848	8659	-0.038406171	3065	5.892384E-01
Gm12849	8660	0.074574898	10201.5	2.939481E-01
Gm12850	8661		#N/A	1.000000E+00
Gm12851	8662	0.160985049	16395	2.276975E-02
Gm12852	8663		#N/A	1.000000E+00
Gm12853	8664	-0.038406171	3065	5.892384E-01
Gm12854	8665		#N/A	1.000000E+00
Gm12855	8666		#N/A	1.000000E+00
Gm12856	8667		#N/A	1.000000E+00
Gm12857	8668		#N/A	1.000000E+00
Gm12858	8669		#N/A	1.000000E+00
Gm12859	8670		#N/A	1.000000E+00
Gm12860	8671		#N/A	1.000000E+00
Gm12861	8672		#N/A	1.000000E+00
Gm12862	8673		#N/A	1.000000E+00
Gm12863	8674	-0.038406171	3065	5.892384E-01
Gm12864	8675		#N/A	1.000000E+00
Gm12865	8676		#N/A	1.000000E+00
Gm12866	8677	0.109492511	12666.5	1.227347E-01
Gm12867	8678	-0.054450825	1984	4.437997E-01
Gm12868	8679		#N/A	1.000000E+00
Gm12869	8680	-0.038406171	3065	5.892384E-01
Gm12870	8681		#N/A	1.000000E+00
Gm12871	8682	0.197280667	18134	5.109405E-03
Gm12872	8683		#N/A	1.000000E+00
Gm12873	8684	-0.07739401	763.5	2.760257E-01
Gm12874	8685		#N/A	1.000000E+00

Spearman Rank correlation analysis performed between Prdm1 and all-expressed genes within the Meredith RNA-seq dataset. Robust Prdm1–associated genes were identified using a cut-off of $p < 0.0005$.

Table S1, Related to Supplemental Figure 3C. Prdm1 associated genes

Gm12875	8686		#N/A	1.000000E+00
Gm12876	8687	0.053267784	8685	4.537786E-01
Gm12877	8688	0.074574898	10201.5	2.939481E-01
Gm12878	8689		#N/A	1.000000E+00
Gm12879	8690		#N/A	1.000000E+00
Gm12880	8691		#N/A	1.000000E+00
Gm12881	8692	-0.054451513	1703	4.437940E-01
Gm12882	8693	0.222864427	18982	1.513858E-03
Gm12883	8694	0.091279115	11463.5	1.986261E-01
Gm12884	8695		#N/A	1.000000E+00
Gm12885	8696		#N/A	1.000000E+00
Gm12886	8697	0.003080023	5476	9.654742E-01
Gm12887	8698		#N/A	1.000000E+00
Gm12888	8699	0.052728522	8550.5	4.583686E-01
Gm12889	8700		#N/A	1.000000E+00
Gm12890	8701		#N/A	1.000000E+00
Gm12891	8702		#N/A	1.000000E+00
Gm12892	8703	0.19429475	18010.5	5.836269E-03
Gm12893	8704		#N/A	1.000000E+00
Gm12895	8705		#N/A	1.000000E+00
Gm12896	8706		#N/A	1.000000E+00
Gm12897	8707	-7.57115E-05	5285	9.991510E-01
Gm12898	8708		#N/A	1.000000E+00
Gm12899	8709		#N/A	1.000000E+00
Gm129	8710	0.063158813	9398	3.742776E-01
Gm12900	8711	0.019032971	6284	7.890812E-01
Gm12901	8712	0.127422667	14147	7.216592E-02
Gm12902	8713		#N/A	1.000000E+00
Gm12903	8714		#N/A	1.000000E+00
Gm12904	8715		#N/A	1.000000E+00
Gm12905	8716	-0.066856562	1139	3.468991E-01
Gm12906	8717		#N/A	1.000000E+00
Gm12907	8718	0.160304018	16323	2.335876E-02
Gm12908	8719	0.19939461	18220	4.645040E-03
Gm12909	8720	0.194131481	17998	5.878558E-03
Gm12910	8721	0.160987082	16412.5	2.276801E-02
Gm12911	8722		#N/A	1.000000E+00
Gm12912	8723	-0.027407398	4019	7.000581E-01
Gm12913	8724		#N/A	1.000000E+00
Gm12914	8725		#N/A	1.000000E+00
Gm12915	8726		#N/A	1.000000E+00
Gm12916	8727		#N/A	1.000000E+00
Gm12917	8728	-0.038406171	3065	5.892384E-01

Spearman Rank correlation analysis performed between Prdm1 and all-expressed genes within the Meredith RNA-seq dataset. Robust Prdm1–associated genes were identified using a cut-off of $p < 0.0005$.

Table S1, Related to Supplemental Figure 3C. Prdm1 associated genes

Gm12918	8729		#N/A	1.000000E+00
Gm12919	8730		#N/A	1.000000E+00
Gm12920	8731		#N/A	1.000000E+00
Gm12922	8732		#N/A	1.000000E+00
Gm12923	8733		#N/A	1.000000E+00
Gm12924	8734		#N/A	1.000000E+00
Gm12925	8735		#N/A	1.000000E+00
Gm12926	8736		#N/A	1.000000E+00
Gm12927	8737		#N/A	1.000000E+00
Gm12928	8738		#N/A	1.000000E+00
Gm12929	8739		#N/A	1.000000E+00
Gm12930	8740		#N/A	1.000000E+00
Gm12931	8741		#N/A	1.000000E+00
Gm12932	8742		#N/A	1.000000E+00
Gm12933	8743	0.109492511	12666.5	1.227347E-01
Gm12934	8744		#N/A	1.000000E+00
Gm12935	8745		#N/A	1.000000E+00
Gm12936	8746	0.182294195	17509.5	9.778799E-03
Gm12937	8747		#N/A	1.000000E+00
Gm12939	8748		#N/A	1.000000E+00
Gm12940	8749	0.140089296	14961	4.787107E-02
Gm12941	8750		#N/A	1.000000E+00
Gm12942	8751	-0.120657592	99	8.877717E-02
Gm12943	8752	0.062099902	9332	3.823558E-01
Gm12944	8753	0.031784044	7174	6.550269E-01
Gm12945	8754		#N/A	1.000000E+00
Gm12946	8755		#N/A	1.000000E+00
Gm12947	8756	0.195939369	18083	5.425227E-03
Gm12948	8757		#N/A	1.000000E+00
Gm12949	8758		#N/A	1.000000E+00
Gm12950	8759		#N/A	1.000000E+00
Gm12951	8760		#N/A	1.000000E+00
Gm12952	8761		#N/A	1.000000E+00
Gm12953	8762	0.051175111	8394	4.717339E-01
Gm12954	8763		#N/A	1.000000E+00
Gm12955	8764		#N/A	1.000000E+00
Gm12956	8765		#N/A	1.000000E+00
Gm12957	8766		#N/A	1.000000E+00
Gm12958	8767		#N/A	1.000000E+00
Gm12959	8768	-0.038406171	3065	5.892384E-01
Gm12960	8769	0.113548679	13094	1.093908E-01
Gm12961	8770		#N/A	1.000000E+00
Gm12962	8771	0.05903225	9158	4.063502E-01

Spearman Rank correlation analysis performed between Prdm1 and all-expressed genes within the Meredith RNA-seq dataset. Robust Prdm1–associated genes were identified using a cut-off of $p < 0.0005$.

Gm12963	8772		#N/A	1.000000E+00
Gm12966	8773		#N/A	1.000000E+00
Gm12967	8774	0.188526222	17781	7.507142E-03
Gm12968	8775	-0.032450477	3820	6.482730E-01
Gm12969	8776		#N/A	1.000000E+00
Gm12970	8777		#N/A	1.000000E+00
Gm12971	8778		#N/A	1.000000E+00
Gm12972	8779		#N/A	1.000000E+00
Gm12973	8780	-0.038406171	3065	5.892384E-01
Gm12974	8781		#N/A	1.000000E+00
Gm12976	8782	-0.038406171	3065	5.892384E-01
Gm12977	8783	0.053267784	8685	4.537786E-01
Gm12978	8784		#N/A	1.000000E+00
Gm12979	8785	0.097267047	11858	1.706267E-01
Gm12980	8786	0.089371505	11299	2.082104E-01
Gm12981	8787	0.113548679	13094	1.093908E-01
Gm12982	8788		#N/A	1.000000E+00
Gm12983	8789		#N/A	1.000000E+00
Gm12984	8790	0.113548679	13094	1.093908E-01
Gm12985	8791		#N/A	1.000000E+00
Gm12986	8792		#N/A	1.000000E+00
Gm12987	8793		#N/A	1.000000E+00
Gm12988	8794	0.160987082	16412.5	2.276801E-02
Gm12989	8795		#N/A	1.000000E+00
Gm12990	8796		#N/A	1.000000E+00
Gm12991	8797	0.127764143	14172	7.139946E-02
Gm12992	8798	0.051251246	8408	4.710739E-01
Gm12993	8799		#N/A	1.000000E+00
Gm12994	8800	-0.038406171	3065	5.892384E-01
Gm12996	8801	-0.038406171	3065	5.892384E-01
Gm12997	8802		#N/A	1.000000E+00
Gm12998	8803		#N/A	1.000000E+00
Gm12999	8804	0.160304018	16323	2.335876E-02
Gm13000	8805		#N/A	1.000000E+00
Gm13001	8806		#N/A	1.000000E+00
Gm13002	8807		#N/A	1.000000E+00
Gm13003	8808		#N/A	1.000000E+00
Gm13005	8809		#N/A	1.000000E+00
Gm13006	8810		#N/A	1.000000E+00
Gm13007	8811		#N/A	1.000000E+00
Gm13008	8812	0.064732329	9521	3.624688E-01
Gm13009	8813	-0.020022635	4238	7.783912E-01
Gm13010	8814		#N/A	1.000000E+00

Table S1, Related to Supplemental Figure 3C. Prdm1 associated genes

Gm13011	8815	0.085013068	10990	2.313461E-01
Gm13012	8816	0.068212994	9715	3.371815E-01
Gm13013	8817		#N/A	1.000000E+00
Gm13015	8818	0.143605682	15224	4.249002E-02
Gm13016	8819		#N/A	1.000000E+00
Gm13017	8820		#N/A	1.000000E+00
Gm13018	8821		#N/A	1.000000E+00
Gm13019	8822		#N/A	1.000000E+00
Gm13021	8823	-0.054450825	1984	4.437997E-01
Gm13022	8824		#N/A	1.000000E+00
Gm13023	8825	-0.038406171	3065	5.892384E-01
Gm13024	8826		#N/A	1.000000E+00
Gm13025	8827		#N/A	1.000000E+00
Gm13026	8828	0.075328288	10286.5	2.890843E-01
Gm13027	8829		#N/A	1.000000E+00
Gm13028	8830	0.029944433	7063	6.738148E-01
Gm13029	8831	-0.038406171	3065	5.892384E-01
Gm13030	8832		#N/A	1.000000E+00
Gm13031	8833	0.049475173	8253	4.866010E-01
Gm13032	8834	-0.054451513	1703	4.437940E-01
Gm13033	8835		#N/A	1.000000E+00
Gm13034	8836		#N/A	1.000000E+00
Gm13035	8837		#N/A	1.000000E+00
Gm13036	8838		#N/A	1.000000E+00
Gm13037	8839		#N/A	1.000000E+00
Gm13038	8840	0.158026551	16176	2.542481E-02
Gm13039	8841		#N/A	1.000000E+00
Gm13040	8842		#N/A	1.000000E+00
Gm13041	8843		#N/A	1.000000E+00
Gm13042	8844		#N/A	1.000000E+00
GM13043	8845		#N/A	1.000000E+00
Gm13043	8846		#N/A	1.000000E+00
Gm13044	8847		#N/A	1.000000E+00
Gm13045	8848		#N/A	1.000000E+00
Gm13046	8849		#N/A	1.000000E+00
Gm13047	8850	0.113548679	13094	1.093908E-01
Gm13049	8851	-0.066858259	1029.5	3.468868E-01
Gm1305	8852	0.049218433	8236	4.888680E-01
Gm13050	8853		#N/A	1.000000E+00
Gm13051	8854	0.104687489	12335	1.401369E-01
Gm13052	8855	0.022983011	6694	7.466693E-01
Gm13053	8856		#N/A	1.000000E+00
Gm13054	8857	-0.038406171	3065	5.892384E-01

Spearman Rank correlation analysis performed between Prdm1 and all-expressed genes within the Meredith RNA-seq dataset. Robust Prdm1–associated genes were identified using a cut-off of $p < 0.0005$.

Table S1, Related to Supplemental Figure 3C. Prdm1 associated genes

Gm13055	8858		#N/A	1.000000E+00
Gm13056	8859		#N/A	1.000000E+00
Gm13057	8860		#N/A	1.000000E+00
Gm13058	8861		#N/A	1.000000E+00
Gm13059	8862		#N/A	1.000000E+00
Gm13061	8863		#N/A	1.000000E+00
Gm13062	8864		#N/A	1.000000E+00
Gm13063	8865		#N/A	1.000000E+00
Gm13064	8866		#N/A	1.000000E+00
Gm13065	8867	-0.030530331	3913	6.678084E-01
Gm13066	8868		#N/A	1.000000E+00
Gm13067	8869	-0.038406171	3065	5.892384E-01
Gm13068	8870		#N/A	1.000000E+00
Gm13069	8871		#N/A	1.000000E+00
Gm13070	8872		#N/A	1.000000E+00
Gm13071	8873		#N/A	1.000000E+00
Gm13073	8874		#N/A	1.000000E+00
Gm13074	8875	0.109492511	12666.5	1.227347E-01
Gm13075	8876	-0.054451513	1703	4.437940E-01
Gm13076	8877	0.113548679	13094	1.093908E-01
Gm13077	8878		#N/A	1.000000E+00
Gm13078	8879	0.082269134	10768	2.468098E-01
Gm13079	8880	0.112525134	12892	1.126455E-01
Gm13080	8881		#N/A	1.000000E+00
Gm13081	8882		#N/A	1.000000E+00
Gm13082	8883		#N/A	1.000000E+00
Gm13083	8884		#N/A	1.000000E+00
Gm13084	8885		#N/A	1.000000E+00
Gm13085	8886		#N/A	1.000000E+00
Gm13086	8887		#N/A	1.000000E+00
Gm13087	8888		#N/A	1.000000E+00
Gm13088	8889		#N/A	1.000000E+00
Gm13089	8890		#N/A	1.000000E+00
Gm13090	8891		#N/A	1.000000E+00
Gm13091	8892	-0.038406171	3065	5.892384E-01
Gm13092	8893	0.134962848	14618	5.672468E-02
Gm13093	8894		#N/A	1.000000E+00
Gm13094	8895	-0.054451513	1703	4.437940E-01
Gm13096	8896		#N/A	1.000000E+00
Gm13097	8897		#N/A	1.000000E+00
Gm13100	8898		#N/A	1.000000E+00
Gm13101	8899		#N/A	1.000000E+00
Gm13102	8900	-0.054451513	1703	4.437940E-01

Spearman Rank correlation analysis performed between Prdm1 and all-expressed genes within the Meredith RNA-seq dataset. Robust Prdm1–associated genes were identified using a cut-off of $p < 0.0005$.

Table S1, Related to Supplemental Figure 3C. Prdm1 associated genes

Gm13103	8901		#N/A	1.000000E+00
Gm13104	8902		#N/A	1.000000E+00
Gm13105	8903		#N/A	1.000000E+00
Gm13106	8904		#N/A	1.000000E+00
Gm13108	8905		#N/A	1.000000E+00
Gm13109	8906		#N/A	1.000000E+00
Gm13110	8907	0.113548679	13094	1.093908E-01
Gm13111	8908		#N/A	1.000000E+00
Gm13112	8909	-0.038406171	3065	5.892384E-01
Gm13113	8910		#N/A	1.000000E+00
Gm13114	8911		#N/A	1.000000E+00
Gm13115	8912		#N/A	1.000000E+00
Gm13116	8913		#N/A	1.000000E+00
Gm13117	8914		#N/A	1.000000E+00
Gm13118	8915		#N/A	1.000000E+00
Gm13119	8916		#N/A	1.000000E+00
Gm13120	8917		#N/A	1.000000E+00
Gm13121	8918	0.113548679	13094	1.093908E-01
Gm13122	8919		#N/A	1.000000E+00
Gm13123	8920		#N/A	1.000000E+00
Gm13124	8921		#N/A	1.000000E+00
Gm13125	8922		#N/A	1.000000E+00
Gm13126	8923		#N/A	1.000000E+00
Gm13127	8924		#N/A	1.000000E+00
Gm13128	8925		#N/A	1.000000E+00
Gm13130	8926		#N/A	1.000000E+00
Gm13131	8927		#N/A	1.000000E+00
Gm13132	8928		#N/A	1.000000E+00
Gm13133	8929		#N/A	1.000000E+00
Gm13134	8930	-0.038406171	3065	5.892384E-01
Gm13135	8931		#N/A	1.000000E+00
Gm13136	8932		#N/A	1.000000E+00
Gm13137	8933		#N/A	1.000000E+00
Gm13139	8934	-0.038406171	3065	5.892384E-01
Gm13140	8935		#N/A	1.000000E+00
Gm13141	8936		#N/A	1.000000E+00
Gm13142	8937		#N/A	1.000000E+00
Gm13144	8938		#N/A	1.000000E+00
Gm13145	8939		#N/A	1.000000E+00
Gm13146	8940		#N/A	1.000000E+00
Gm13147	8941	-0.038406171	3065	5.892384E-01
Gm13148	8942		#N/A	1.000000E+00
Gm13149	8943		#N/A	1.000000E+00

Spearman Rank correlation analysis performed between Prdm1 and all-expressed genes within the Meredith RNA-seq dataset. Robust Prdm1–associated genes were identified using a cut-off of $p < 0.0005$.

Table S1, Related to Supplemental Figure 3C. Prdm1 associated genes

Gm13150	8944		#N/A	1.000000E+00
Gm13151	8945		#N/A	1.000000E+00
Gm13152	8946	0.138972295	14885	4.969500E-02
Gm13153	8947		#N/A	1.000000E+00
Gm13154	8948	0.022355118	6653	7.533640E-01
Gm13155	8949		#N/A	1.000000E+00
Gm13156	8950		#N/A	1.000000E+00
Gm13157	8951	0.179146178	17365	1.114253E-02
Gm13158	8952		#N/A	1.000000E+00
Gm13160	8953		#N/A	1.000000E+00
Gm13161	8954		#N/A	1.000000E+00
Gm13162	8955		#N/A	1.000000E+00
Gm13163	8956		#N/A	1.000000E+00
Gm13165	8957	-0.035010307	3710	6.226006E-01
Gm13166	8958	0.113548679	13094	1.093908E-01
Gm13167	8959		#N/A	1.000000E+00
Gm13168	8960		#N/A	1.000000E+00
Gm13169	8961		#N/A	1.000000E+00
Gm13170	8962		#N/A	1.000000E+00
Gm13171	8963		#N/A	1.000000E+00
Gm13172	8964		#N/A	1.000000E+00
Gm13173	8965		#N/A	1.000000E+00
Gm13174	8966		#N/A	1.000000E+00
Gm13175	8967		#N/A	1.000000E+00
Gm13176	8968		#N/A	1.000000E+00
Gm13177	8969		#N/A	1.000000E+00
Gm13178	8970	-0.095267438	357.5	1.796303E-01
Gm13179	8971		#N/A	1.000000E+00
Gm13180	8972		#N/A	1.000000E+00
Gm13181	8973		#N/A	1.000000E+00
Gm13182	8974		#N/A	1.000000E+00
Gm13183	8975	0.020875719	6412.5	7.692098E-01
Gm13184	8976	-0.038406171	3065	5.892384E-01
Gm13185	8977		#N/A	1.000000E+00
Gm13186	8978		#N/A	1.000000E+00
Gm13187	8979	-0.038406171	3065	5.892384E-01
Gm13188	8980		#N/A	1.000000E+00
Gm13189	8981		#N/A	1.000000E+00
Gm13191	8982		#N/A	1.000000E+00
Gm13192	8983	-0.086755146	452	2.218903E-01
Gm13193	8984		#N/A	1.000000E+00
Gm13194	8985	0.009616656	5752	8.924927E-01
Gm13195	8986		#N/A	1.000000E+00

Spearman Rank correlation analysis performed between Prdm1 and all-expressed genes within the Meredith RNA-seq dataset. Robust Prdm1–associated genes were identified using a cut-off of $p < 0.0005$.

Gm13196	8987	-0.066858259	1029.5	3.468868E-01
Gm13197	8988		#N/A	1.000000E+00
Gm13198	8989		#N/A	1.000000E+00
Gm13199	8990		#N/A	1.000000E+00
Gm13200	8991	-0.038406171	3065	5.892384E-01
Gm13201	8992		#N/A	1.000000E+00
Gm13202	8993	0.053267784	8685	4.537786E-01
Gm13203	8994	-0.054450825	1984	4.437997E-01
Gm13204	8995		#N/A	1.000000E+00
Gm13205	8996	0.101138169	12125	1.541569E-01
Gm13206	8997		#N/A	1.000000E+00
Gm13207	8998	0.230217498	19157	1.039825E-03
Gm13209	8999	-0.054450825	1984	4.437997E-01
Gm13210	9000		#N/A	1.000000E+00
Gm13211	9001	-0.020022635	4238	7.783912E-01
Gm13212	9002	0.058353666	9111	4.117759E-01
Gm13213	9003		#N/A	1.000000E+00
Gm13214	9004		#N/A	1.000000E+00
Gm13215	9005	0.020875719	6412.5	7.692098E-01
Gm13216	9006		#N/A	1.000000E+00
Gm13217	9007		#N/A	1.000000E+00
Gm13218	9008		#N/A	1.000000E+00
Gm13219	9009	-0.066254108	1297.5	3.512713E-01
Gm1322	9010		#N/A	1.000000E+00
Gm13220	9011		#N/A	1.000000E+00
Gm13221	9012		#N/A	1.000000E+00
Gm13222	9013		#N/A	1.000000E+00
Gm13223	9014		#N/A	1.000000E+00
Gm13224	9015		#N/A	1.000000E+00
Gm13225	9016	0.012048174	5877	8.655410E-01
Gm13226	9017	0.146987249	15478	3.780352E-02
Gm13227	9018	-0.005348232	4906.5	9.400859E-01
Gm13228	9019		#N/A	1.000000E+00
Gm13229	9020	0.074574898	10201.5	2.939481E-01
Gm13230	9021		#N/A	1.000000E+00
Gm13231	9022	-0.038406171	3065	5.892384E-01
Gm13232	9023		#N/A	1.000000E+00
Gm13233	9024		#N/A	1.000000E+00
Gm13234	9025		#N/A	1.000000E+00
Gm13235	9026	0.113548679	13094	1.093908E-01
Gm13236	9027		#N/A	1.000000E+00
Gm13237	9028		#N/A	1.000000E+00
Gm13238	9029		#N/A	1.000000E+00

Table S1, Related to Supplemental Figure 3C. Prdm1 associated genes

Gm13239	9030		#N/A	1.000000E+00
Gm13240	9031		#N/A	1.000000E+00
Gm13241	9032		#N/A	1.000000E+00
Gm13242	9033		#N/A	1.000000E+00
Gm13243	9034		#N/A	1.000000E+00
Gm13244	9035		#N/A	1.000000E+00
Gm13245	9036	-0.038406171	3065	5.892384E-01
Gm13246	9037		#N/A	1.000000E+00
Gm13247	9038	0.097256674	11857	1.706725E-01
Gm13248	9039	0.045056653	7941.5	5.263908E-01
Gm13249	9040		#N/A	1.000000E+00
Gm13250	9041		#N/A	1.000000E+00
Gm13251	9042	-0.109162119	188	1.238749E-01
Gm13252	9043	-0.066858259	1029.5	3.468868E-01
Gm13254	9044		#N/A	1.000000E+00
Gm13255	9045		#N/A	1.000000E+00
Gm13256	9046	0.126288732	14063	7.475918E-02
Gm13257	9047	-0.054451513	1703	4.437940E-01
Gm13258	9048		#N/A	1.000000E+00
Gm13259	9049		#N/A	1.000000E+00
Gm13260	9050	-0.038406171	3065	5.892384E-01
Gm13261	9051	0.110677636	12769	1.187116E-01
Gm13262	9052	-0.038406171	3065	5.892384E-01
Gm13263	9053		#N/A	1.000000E+00
Gm13264	9054	-0.038406171	3065	5.892384E-01
Gm13265	9055	-0.038406171	3065	5.892384E-01
Gm13266	9056	0.157799267	16153.5	2.563935E-02
Gm13267	9057		#N/A	1.000000E+00
Gm13268	9058	0.021317126	6513.5	7.644715E-01
Gm13269	9059	-0.018447724	4312.5	7.954215E-01
Gm13270	9060	-0.038406171	3065	5.892384E-01
Gm13271	9061		#N/A	1.000000E+00
Gm13272	9062		#N/A	1.000000E+00
Gm13273	9063		#N/A	1.000000E+00
Gm13274	9064	-0.038406171	3065	5.892384E-01
Gm13275	9065		#N/A	1.000000E+00
Gm13276	9066		#N/A	1.000000E+00
Gm13277	9067		#N/A	1.000000E+00
Gm13278	9068		#N/A	1.000000E+00
Gm13279	9069		#N/A	1.000000E+00
Gm13280	9070		#N/A	1.000000E+00
Gm13281	9071		#N/A	1.000000E+00
Gm13282	9072		#N/A	1.000000E+00

Spearman Rank correlation analysis performed between Prdm1 and all-expressed genes within the Meredith RNA-seq dataset. Robust Prdm1–associated genes were identified using a cut-off of $p < 0.0005$.

Table S1, Related to Supplemental Figure 3C. Prdm1 associated genes

Gm13283	9073		#N/A	1.000000E+00
Gm13284	9074		#N/A	1.000000E+00
Gm13285	9075		#N/A	1.000000E+00
Gm13286	9076		#N/A	1.000000E+00
Gm13287	9077		#N/A	1.000000E+00
Gm13288	9078		#N/A	1.000000E+00
Gm13289	9079		#N/A	1.000000E+00
Gm13290	9080		#N/A	1.000000E+00
Gm13291	9081		#N/A	1.000000E+00
Gm13292	9082		#N/A	1.000000E+00
Gm13293	9083		#N/A	1.000000E+00
Gm13294	9084		#N/A	1.000000E+00
Gm13295	9085		#N/A	1.000000E+00
Gm13296	9086	-0.038406171	3065	5.892384E-01
Gm13297	9087	-0.038406171	3065	5.892384E-01
Gm13298	9088		#N/A	1.000000E+00
Gm13299	9089		#N/A	1.000000E+00
Gm1330	9090		#N/A	1.000000E+00
Gm13301	9091	-0.021149044	4201	7.662748E-01
Gm13303	9092		#N/A	1.000000E+00
Gm13304	9093		#N/A	1.000000E+00
Gm13305	9094		#N/A	1.000000E+00
Gm13306	9095		#N/A	1.000000E+00
Gm13307	9096		#N/A	1.000000E+00
GM13308	9097		#N/A	1.000000E+00
Gm13310	9098		#N/A	1.000000E+00
Gm13311	9099		#N/A	1.000000E+00
Gm13312	9100	-0.038406171	3065	5.892384E-01
Gm13313	9101		#N/A	1.000000E+00
Gm13314	9102		#N/A	1.000000E+00
Gm13315	9103		#N/A	1.000000E+00
Gm13316	9104		#N/A	1.000000E+00
Gm13317	9105		#N/A	1.000000E+00
Gm13318	9106	0.097701346	11881	1.687164E-01
Gm13319	9107		#N/A	1.000000E+00
Gm13320	9108		#N/A	1.000000E+00
Gm13321	9109	0.220173509	18904	1.731828E-03
Gm13322	9110		#N/A	1.000000E+00
Gm13323	9111		#N/A	1.000000E+00
Gm13324	9112		#N/A	1.000000E+00
Gm13325	9113		#N/A	1.000000E+00
Gm13326	9114	-0.054450825	1984	4.437997E-01
Gm13327	9115	0.137332099	14778.5	5.247777E-02

Spearman Rank correlation analysis performed between Prdm1 and all-expressed genes within the Meredith RNA-seq dataset. Robust Prdm1–associated genes were identified using a cut-off of $p < 0.0005$.

Table S1, Related to Supplemental Figure 3C. Prdm1 associated genes

Gm13328	9116		#N/A	1.000000E+00
Gm13329	9117		#N/A	1.000000E+00
Gm13330	9118	-0.038406171	3065	5.892384E-01
Gm13331	9119		#N/A	1.000000E+00
Gm13332	9120		#N/A	1.000000E+00
Gm13333	9121		#N/A	1.000000E+00
Gm13334	9122	0.143605682	15224	4.249002E-02
Gm13335	9123		#N/A	1.000000E+00
Gm13336	9124		#N/A	1.000000E+00
Gm13337	9125		#N/A	1.000000E+00
Gm13338	9126		#N/A	1.000000E+00
Gm13339	9127	0.004284676	5530	9.519845E-01
Gm13340	9128	0.246442879	19460	4.351457E-04
Gm13341	9129	0.252424138	19530	3.109379E-04
Gm13342	9130		#N/A	1.000000E+00
Gm13343	9131		#N/A	1.000000E+00
Gm13344	9132		#N/A	1.000000E+00
Gm13345	9133		#N/A	1.000000E+00
Gm13346	9134		#N/A	1.000000E+00
Gm13347	9135		#N/A	1.000000E+00
Gm13348	9136		#N/A	1.000000E+00
Gm13349	9137		#N/A	1.000000E+00
Gm1335	9138		#N/A	1.000000E+00
Gm13350	9139		#N/A	1.000000E+00
Gm13351	9140		#N/A	1.000000E+00
Gm13352	9141		#N/A	1.000000E+00
Gm13353	9142		#N/A	1.000000E+00
Gm13354	9143		#N/A	1.000000E+00
Gm13355	9144		#N/A	1.000000E+00
Gm13356	9145		#N/A	1.000000E+00
Gm13357	9146		#N/A	1.000000E+00
Gm13358	9147	-0.066858259	1029.5	3.468868E-01
Gm13359	9148		#N/A	1.000000E+00
Gm13360	9149		#N/A	1.000000E+00
Gm13361	9150		#N/A	1.000000E+00
Gm13362	9151		#N/A	1.000000E+00
Gm13363	9152		#N/A	1.000000E+00
Gm13364	9153	0.05275236	8579	4.581651E-01
Gm13365	9154		#N/A	1.000000E+00
Gm13366	9155	0.166148435	16721.5	1.870631E-02
Gm13367	9156		#N/A	1.000000E+00
Gm13368	9157	0.08394009	10917	2.373096E-01
Gm13369	9158	-0.000841282	5208.5	9.905669E-01

Spearman Rank correlation analysis performed between Prdm1 and all-expressed genes within the Meredith RNA-seq dataset. Robust Prdm1–associated genes were identified using a cut-off of $p < 0.0005$.

Table S1, Related to Supplemental Figure 3C. Prdm1 associated genes

Gm13370	9159		#N/A	1.000000E+00
Gm13371	9160		#N/A	1.000000E+00
Gm13372	9161		#N/A	1.000000E+00
Gm13373	9162	-0.038406171	3065	5.892384E-01
Gm13375	9163	0.113548679	13094	1.093908E-01
Gm13376	9164	-0.038406171	3065	5.892384E-01
Gm13377	9165	0.018794845	6266	7.916593E-01
Gm13378	9166		#N/A	1.000000E+00
Gm13379	9167	-0.038406171	3065	5.892384E-01
Gm13380	9168	-0.066855996	1239.5	3.469032E-01
Gm13381	9169	0.157799267	16153.5	2.563935E-02
Gm13382	9170		#N/A	1.000000E+00
Gm13383	9171		#N/A	1.000000E+00
Gm13384	9172		#N/A	1.000000E+00
Gm13385	9173	0.074574898	10201.5	2.939481E-01
Gm13386	9174		#N/A	1.000000E+00
Gm13387	9175	0.113548679	13094	1.093908E-01
Gm13388	9176		#N/A	1.000000E+00
Gm13389	9177		#N/A	1.000000E+00
Gm13391	9178	0.127090804	14122	7.291719E-02
Gm13392	9179		#N/A	1.000000E+00
Gm13393	9180		#N/A	1.000000E+00
Gm13394	9181		#N/A	1.000000E+00
Gm13396	9182		#N/A	1.000000E+00
Gm13397	9183	0.047870267	8136	5.008643E-01
Gm13398	9184	-0.066855996	1239.5	3.469032E-01
Gm13399	9185	0.071043989	9915	3.174654E-01
Gm13400	9186		#N/A	1.000000E+00
Gm13401	9187		#N/A	1.000000E+00
Gm13402	9188	0.090858474	11420.5	2.007115E-01
Gm13403	9189		#N/A	1.000000E+00
Gm13404	9190	-0.000841234	5228	9.905674E-01
Gm13405	9191		#N/A	1.000000E+00
Gm13406	9192		#N/A	1.000000E+00
Gm13407	9193	-0.001224019	5151	9.862757E-01
Gm13408	9194		#N/A	1.000000E+00
Gm13409	9195	0.050197193	8313.5	4.802559E-01
Gm13410	9196		#N/A	1.000000E+00
Gm13411	9197	0.229057459	19118.5	1.104172E-03
Gm13412	9198		#N/A	1.000000E+00
Gm13413	9199	-0.054451513	1703	4.437940E-01
Gm13414	9200		#N/A	1.000000E+00
Gm13415	9201	0.074574898	10201.5	2.939481E-01

Spearman Rank correlation analysis performed between Prdm1 and all-expressed genes within the Meredith RNA-seq dataset. Robust Prdm1–associated genes were identified using a cut-off of $p < 0.0005$.

Table S1, Related to Supplemental Figure 3C. Prdm1 associated genes

Gm13416	9202		#N/A	1.000000E+00
Gm13417	9203		#N/A	1.000000E+00
Gm13418	9204		#N/A	1.000000E+00
Gm13419	9205		#N/A	1.000000E+00
Gm13420	9206		#N/A	1.000000E+00
Gm13421	9207	0.160304018	16323	2.335876E-02
Gm13422	9208		#N/A	1.000000E+00
Gm13423	9209	-0.018790377	4285	7.917077E-01
Gm13424	9210		#N/A	1.000000E+00
Gm13425	9211		#N/A	1.000000E+00
Gm13426	9212		#N/A	1.000000E+00
Gm13427	9213	0.081004407	10661	2.541740E-01
Gm13428	9214	0.063431092	9426.5	3.722175E-01
Gm13429	9215		#N/A	1.000000E+00
Gm13430	9216		#N/A	1.000000E+00
Gm13431	9217	0.020434681	6357	7.739526E-01
Gm13432	9218		#N/A	1.000000E+00
Gm13433	9219		#N/A	1.000000E+00
Gm13434	9220		#N/A	1.000000E+00
Gm13435	9221		#N/A	1.000000E+00
Gm13436	9222		#N/A	1.000000E+00
Gm13437	9223	0.033795959	7286	6.347250E-01
Gm13438	9224		#N/A	1.000000E+00
Gm13439	9225		#N/A	1.000000E+00
Gm13440	9226	-7.57115E-05	5285	9.991510E-01
Gm13441	9227		#N/A	1.000000E+00
Gm13442	9228		#N/A	1.000000E+00
Gm13443	9229		#N/A	1.000000E+00
Gm13444	9230		#N/A	1.000000E+00
Gm13445	9231	-0.054451513	1703	4.437940E-01
Gm13446	9232		#N/A	1.000000E+00
Gm13447	9233		#N/A	1.000000E+00
Gm13448	9234		#N/A	1.000000E+00
Gm13449	9235	0.000542976	5344	9.939116E-01
Gm13450	9236		#N/A	1.000000E+00
Gm13451	9237		#N/A	1.000000E+00
Gm13452	9238		#N/A	1.000000E+00
Gm13453	9239		#N/A	1.000000E+00
Gm13454	9240		#N/A	1.000000E+00
Gm13455	9241		#N/A	1.000000E+00
Gm13456	9242	0.164478601	16623.5	1.994547E-02
Gm13457	9243		#N/A	1.000000E+00
Gm13458	9244		#N/A	1.000000E+00

Spearman Rank correlation analysis performed between Prdm1 and all-expressed genes within the Meredith RNA-seq dataset. Robust Prdm1–associated genes were identified using a cut-off of $p < 0.0005$.

Table S1, Related to Supplemental Figure 3C. Prdm1 associated genes

Gm13459	9245	0.049316404	8246.5	4.880022E-01
Gm13460	9246		#N/A	1.000000E+00
Gm13461	9247		#N/A	1.000000E+00
Gm13462	9248		#N/A	1.000000E+00
Gm13463	9249		#N/A	1.000000E+00
Gm13464	9250	-0.054451513	1703	4.437940E-01
Gm13465	9251		#N/A	1.000000E+00
Gm13466	9252	0.113548679	13094	1.093908E-01
Gm13467	9253	0.073256824	10083	3.025877E-01
Gm13468	9254		#N/A	1.000000E+00
Gm13469	9255	-0.054451513	1703	4.437940E-01
Gm13470	9256	-0.038406171	3065	5.892384E-01
Gm13471	9257		#N/A	1.000000E+00
Gm13472	9258	0.086412184	11087.5	2.237298E-01
Gm13473	9259		#N/A	1.000000E+00
Gm13474	9260		#N/A	1.000000E+00
Gm13475	9261		#N/A	1.000000E+00
Gm13476	9262	-0.01265031	4638	8.588894E-01
Gm13477	9263	0.113548679	13094	1.093908E-01
Gm13478	9264	-0.013118278	4616	8.537267E-01
Gm13479	9265	-0.038406171	3065	5.892384E-01
Gm13480	9266	0.073928833	10125	2.981622E-01
Gm13481	9267	0.059611756	9190	4.017503E-01
Gm13482	9268		#N/A	1.000000E+00
Gm13483	9269	-0.029782286	3934	6.754807E-01
Gm13484	9270		#N/A	1.000000E+00
Gm13485	9271		#N/A	1.000000E+00
Gm13486	9272		#N/A	1.000000E+00
Gm13487	9273		#N/A	1.000000E+00
Gm13488	9274		#N/A	1.000000E+00
Gm13489	9275	0.053267784	8685	4.537786E-01
Gm13490	9276	0.145119223	15359	4.033535E-02
Gm13491	9277		#N/A	1.000000E+00
Gm13492	9278	0.084141322	10930.5	2.361831E-01
Gm13493	9279		#N/A	1.000000E+00
Gm13494	9280		#N/A	1.000000E+00
Gm13495	9281		#N/A	1.000000E+00
Gm13496	9282		#N/A	1.000000E+00
Gm13497	9283	0.194131481	17998	5.878558E-03
Gm13498	9284	0.053912715	8858	4.483232E-01
Gm13499	9285		#N/A	1.000000E+00
Gm13500	9286		#N/A	1.000000E+00
Gm13501	9287		#N/A	1.000000E+00

Spearman Rank correlation analysis performed between Prdm1 and all-expressed genes within the Meredith RNA-seq dataset. Robust Prdm1–associated genes were identified using a cut-off of $p < 0.0005$.

Table S1, Related to Supplemental Figure 3C. Prdm1 associated genes

Gm13502	9288		#N/A	1.000000E+00
Gm13503	9289		#N/A	1.000000E+00
Gm13504	9290		#N/A	1.000000E+00
Gm13505	9291	-0.038406171	3065	5.892384E-01
Gm13506	9292		#N/A	1.000000E+00
Gm13507	9293	-0.077394997	736.5	2.760195E-01
Gm13508	9294	0.192815011	17945	6.229682E-03
Gm13509	9295		#N/A	1.000000E+00
Gm13510	9296		#N/A	1.000000E+00
Gm13511	9297		#N/A	1.000000E+00
Gm13517	9298		#N/A	1.000000E+00
Gm13518	9299		#N/A	1.000000E+00
Gm13519	9300		#N/A	1.000000E+00
Gm13520	9301		#N/A	1.000000E+00
Gm13521	9302		#N/A	1.000000E+00
Gm13522	9303		#N/A	1.000000E+00
Gm13523	9304		#N/A	1.000000E+00
Gm13524	9305		#N/A	1.000000E+00
Gm13525	9306	-0.031225586	3876	6.607082E-01
Gm13526	9307		#N/A	1.000000E+00
Gm13527	9308		#N/A	1.000000E+00
Gm13528	9309		#N/A	1.000000E+00
Gm13529	9310		#N/A	1.000000E+00
Gm13530	9311		#N/A	1.000000E+00
Gm13531	9312		#N/A	1.000000E+00
Gm13532	9313		#N/A	1.000000E+00
Gm13533	9314	-0.038406171	3065	5.892384E-01
Gm13534	9315		#N/A	1.000000E+00
Gm13535	9316	0.222868143	18983	1.513575E-03
Gm13536	9317		#N/A	1.000000E+00
Gm13537	9318		#N/A	1.000000E+00
Gm13538	9319	0.215109181	18744.5	2.221323E-03
Gm13539	9320	0.00823266	5688	9.078906E-01
Gm13541	9321	-0.032762514	3799	6.451205E-01
Gm13542	9322		#N/A	1.000000E+00
Gm13543	9323		#N/A	1.000000E+00
Gm13544	9324	0.159799776	16264	2.380328E-02
Gm13545	9325	-0.066858259	1029.5	3.468868E-01
Gm13546	9326	-0.054450825	1984	4.437997E-01
Gm13547	9327	-0.038406171	3065	5.892384E-01
Gm13548	9328		#N/A	1.000000E+00
Gm13549	9329		#N/A	1.000000E+00
Gm13550	9330		#N/A	1.000000E+00

Spearman Rank correlation analysis performed between Prdm1 and all-expressed genes within the Meredith RNA-seq dataset. Robust Prdm1–associated genes were identified using a cut-off of $p < 0.0005$.

Table S1, Related to Supplemental Figure 3C. Prdm1 associated genes

Gm13551	9331	-0.038406171	3065	5.892384E-01
Gm13552	9332		#N/A	1.000000E+00
Gm13553	9333		#N/A	1.000000E+00
Gm13554	9334	0.129222432	14253	6.820044E-02
Gm13555	9335		#N/A	1.000000E+00
Gm13556	9336		#N/A	1.000000E+00
Gm13557	9337		#N/A	1.000000E+00
Gm13558	9338	-0.038406171	3065	5.892384E-01
Gm13559	9339		#N/A	1.000000E+00
Gm13560	9340		#N/A	1.000000E+00
Gm13561	9341	-0.1524785	26	3.112466E-02
Gm13562	9342	-0.038406171	3065	5.892384E-01
Gm13563	9343	0.035537681	7353	6.173664E-01
Gm13564	9344		#N/A	1.000000E+00
Gm13565	9345		#N/A	1.000000E+00
Gm13566	9346		#N/A	1.000000E+00
Gm13567	9347	-0.038406171	3065	5.892384E-01
Gm13568	9348		#N/A	1.000000E+00
Gm13569	9349		#N/A	1.000000E+00
Gm13570	9350		#N/A	1.000000E+00
Gm13571	9351	-0.117579016	115	9.728672E-02
Gm13572	9352		#N/A	1.000000E+00
Gm13573	9353	-0.000841282	5208.5	9.905669E-01
Gm13574	9354		#N/A	1.000000E+00
Gm13575	9355		#N/A	1.000000E+00
Gm13576	9356		#N/A	1.000000E+00
Gm13577	9357		#N/A	1.000000E+00
Gm13578	9358		#N/A	1.000000E+00
Gm13579	9359		#N/A	1.000000E+00
Gm13580	9360	0.113548679	13094	1.093908E-01
Gm13581	9361	0.053267784	8685	4.537786E-01
Gm13582	9362	0.082269134	10768	2.468098E-01
Gm13583	9363	-0.054450825	1984	4.437997E-01
Gm13584	9364		#N/A	1.000000E+00
Gm13586	9365		#N/A	1.000000E+00
Gm13587	9366		#N/A	1.000000E+00
Gm13588	9367		#N/A	1.000000E+00
Gm13589	9368	0.01287414	5926	8.564193E-01
Gm13590	9369		#N/A	1.000000E+00
Gm13591	9370	0.113548679	13094	1.093908E-01
Gm13592	9371	-0.054451513	1703	4.437940E-01
Gm13593	9372	-0.066858259	1029.5	3.468868E-01
Gm13594	9373		#N/A	1.000000E+00

Spearman Rank correlation analysis performed between Prdm1 and all-expressed genes within the Meredith RNA-seq dataset. Robust Prdm1–associated genes were identified using a cut-off of $p < 0.0005$.

Table S1, Related to Supplemental Figure 3C. Prdm1 associated genes

Gm13595	9374		#N/A	1.000000E+00
Gm13596	9375		#N/A	1.000000E+00
Gm13597	9376	0.027453091	6950	6.995822E-01
Gm13598	9377	-0.124294167	79	7.950362E-02
Gm13599	9378	-0.095267438	357.5	1.796303E-01
Gm136	9379	0.113548679	13094	1.093908E-01
Gm13600	9380		#N/A	1.000000E+00
Gm13601	9381		#N/A	1.000000E+00
Gm13602	9382		#N/A	1.000000E+00
Gm13603	9383		#N/A	1.000000E+00
Gm13604	9384	-0.038406171	3065	5.892384E-01
Gm13605	9385		#N/A	1.000000E+00
Gm13606	9386		#N/A	1.000000E+00
Gm13607	9387		#N/A	1.000000E+00
Gm13608	9388		#N/A	1.000000E+00
Gm13609	9389		#N/A	1.000000E+00
Gm13610	9390	-0.029261571	3959.5	6.808413E-01
Gm13611	9391		#N/A	1.000000E+00
Gm13612	9392		#N/A	1.000000E+00
Gm13613	9393	0.022198316	6629.5	7.550388E-01
Gm13614	9394	0.010600707	5795.5	8.815685E-01
Gm13615	9395		#N/A	1.000000E+00
Gm13616	9396		#N/A	1.000000E+00
Gm13617	9397		#N/A	1.000000E+00
Gm13618	9398		#N/A	1.000000E+00
Gm13619	9399		#N/A	1.000000E+00
Gm13620	9400		#N/A	1.000000E+00
Gm13621	9401		#N/A	1.000000E+00
Gm13622	9402	-0.038406171	3065	5.892384E-01
Gm13623	9403	0.139045799	14892.5	4.957323E-02
Gm13624	9404		#N/A	1.000000E+00
Gm13625	9405	-0.017417138	4396	8.066189E-01
Gm13626	9406		#N/A	1.000000E+00
Gm13627	9407	-0.038406171	3065	5.892384E-01
Gm13629	9408	0.154470487	15953	2.896407E-02
Gm13630	9409		#N/A	1.000000E+00
Gm13631	9410		#N/A	1.000000E+00
Gm13632	9411	0.164478601	16623.5	1.994547E-02
Gm13633	9412		#N/A	1.000000E+00
Gm13634	9413	0.138134206	14834	5.110117E-02
Gm13635	9414		#N/A	1.000000E+00
Gm13636	9415		#N/A	1.000000E+00
Gm13637	9416	-0.038406171	3065	5.892384E-01

Spearman Rank correlation analysis performed between Prdm1 and all-expressed genes within the Meredith RNA-seq dataset. Robust Prdm1–associated genes were identified using a cut-off of $p < 0.0005$.

Table S1, Related to Supplemental Figure 3C. Prdm1 associated genes

Gm13638	9417		#N/A	1.000000E+00
Gm13640	9418		#N/A	1.000000E+00
Gm13641	9419	-0.038406171	3065	5.892384E-01
Gm13642	9420		#N/A	1.000000E+00
Gm13643	9421	-0.038406171	3065	5.892384E-01
Gm13644	9422	0.00588276	5590	9.341107E-01
Gm13645	9423		#N/A	1.000000E+00
Gm13647	9424		#N/A	1.000000E+00
Gm13648	9425	-0.04885054	2219	4.921262E-01
Gm13649	9426	0.113548679	13094	1.093908E-01
Gm13650	9427		#N/A	1.000000E+00
Gm13651	9428		#N/A	1.000000E+00
Gm13652	9429		#N/A	1.000000E+00
Gm13653	9430	-0.054451513	1703	4.437940E-01
Gm13655	9431		#N/A	1.000000E+00
Gm13656	9432		#N/A	1.000000E+00
Gm13657	9433		#N/A	1.000000E+00
Gm13658	9434		#N/A	1.000000E+00
Gm13659	9435		#N/A	1.000000E+00
Gm13660	9436	-0.054451513	1703	4.437940E-01
Gm13661	9437		#N/A	1.000000E+00
Gm13662	9438		#N/A	1.000000E+00
Gm13663	9439	0.002119669	5422.5	9.762355E-01
Gm13664	9440	0.053217079	8608	4.542091E-01
Gm13665	9441	-0.038406171	3065	5.892384E-01
Gm13666	9442		#N/A	1.000000E+00
Gm13667	9443	0.160304018	16323	2.335876E-02
Gm13668	9444		#N/A	1.000000E+00
Gm13669	9445		#N/A	1.000000E+00
Gm13670	9446		#N/A	1.000000E+00
Gm13672	9447		#N/A	1.000000E+00
Gm13673	9448		#N/A	1.000000E+00
Gm13674	9449		#N/A	1.000000E+00
Gm13675	9450		#N/A	1.000000E+00
Gm13676	9451		#N/A	1.000000E+00
Gm13677	9452		#N/A	1.000000E+00
Gm13678	9453		#N/A	1.000000E+00
Gm13679	9454	-0.066856562	1139	3.468991E-01
Gm13680	9455	0.080450175	10630	2.574485E-01
Gm13681	9456		#N/A	1.000000E+00
Gm13682	9457		#N/A	1.000000E+00
Gm13683	9458		#N/A	1.000000E+00
Gm13684	9459	0.048448014	8175.5	4.957045E-01

Spearman Rank correlation analysis performed between Prdm1 and all-expressed genes within the Meredith RNA-seq dataset. Robust Prdm1–associated genes were identified using a cut-off of $p < 0.0005$.

Table S1, Related to Supplemental Figure 3C. Prdm1 associated genes

Gm13685	9460		#N/A	1.000000E+00
Gm13686	9461		#N/A	1.000000E+00
Gm13687	9462		#N/A	1.000000E+00
Gm13688	9463	0.03904475	7581	5.830573E-01
Gm13689	9464		#N/A	1.000000E+00
Gm13690	9465		#N/A	1.000000E+00
Gm13691	9466		#N/A	1.000000E+00
Gm13692	9467		#N/A	1.000000E+00
Gm13693	9468		#N/A	1.000000E+00
Gm13694	9469		#N/A	1.000000E+00
Gm13695	9470		#N/A	1.000000E+00
Gm13696	9471		#N/A	1.000000E+00
Gm13697	9472		#N/A	1.000000E+00
Gm13698	9473		#N/A	1.000000E+00
Gm13700	9474		#N/A	1.000000E+00
Gm13701	9475		#N/A	1.000000E+00
Gm13702	9476		#N/A	1.000000E+00
Gm13703	9477	-0.038406171	3065	5.892384E-01
Gm13704	9478	0.02911945	7021	6.823072E-01
Gm13705	9479	0.168014365	16821	1.740122E-02
Gm13706	9480		#N/A	1.000000E+00
Gm13707	9481		#N/A	1.000000E+00
Gm13708	9482	0.192333123	17924	6.362821E-03
Gm13709	9483	0.053267784	8685	4.537786E-01
Gm13710	9484	0.21957656	18890	1.783907E-03
Gm13711	9485		#N/A	1.000000E+00
Gm13712	9486		#N/A	1.000000E+00
Gm13713	9487	-0.018105422	4347	7.991361E-01
Gm13714	9488	0.1544596	15936.5	2.897553E-02
Gm13715	9489		#N/A	1.000000E+00
Gm13716	9490		#N/A	1.000000E+00
Gm13717	9491	0.127542604	14151	7.189596E-02
Gm13718	9492		#N/A	1.000000E+00
Gm13719	9493	0.052728522	8550.5	4.583686E-01
Gm13720	9494	0.053267784	8685	4.537786E-01
Gm13721	9495		#N/A	1.000000E+00
Gm13722	9496	0.143605682	15224	4.249002E-02
Gm13723	9497		#N/A	1.000000E+00
Gm13724	9498		#N/A	1.000000E+00
Gm13725	9499	-0.038406171	3065	5.892384E-01
Gm13726	9500		#N/A	1.000000E+00
Gm13727	9501		#N/A	1.000000E+00
Gm13728	9502		#N/A	1.000000E+00

Spearman Rank correlation analysis performed between Prdm1 and all-expressed genes within the Meredith RNA-seq dataset. Robust Prdm1–associated genes were identified using a cut-off of $p < 0.0005$.

Table S1, Related to Supplemental Figure 3C. Prdm1 associated genes

Gm13729	9503		#N/A	1.000000E+00
Gm13730	9504		#N/A	1.000000E+00
Gm13731	9505		#N/A	1.000000E+00
Gm13732	9506		#N/A	1.000000E+00
Gm13733	9507		#N/A	1.000000E+00
Gm13734	9508		#N/A	1.000000E+00
Gm13735	9509	-0.000841282	5208.5	9.905669E-01
Gm13736	9510	0.237545808	19317	7.067184E-04
Gm13737	9511		#N/A	1.000000E+00
Gm13738	9512		#N/A	1.000000E+00
Gm13739	9513		#N/A	1.000000E+00
Gm13740	9514	0.113548679	13094	1.093908E-01
Gm13741	9515		#N/A	1.000000E+00
Gm13742	9516	0.086412184	11087.5	2.237298E-01
Gm13743	9517		#N/A	1.000000E+00
Gm13744	9518		#N/A	1.000000E+00
Gm13745	9519		#N/A	1.000000E+00
Gm13746	9520		#N/A	1.000000E+00
Gm13747	9521		#N/A	1.000000E+00
Gm13748	9522	0.143605682	15224	4.249002E-02
Gm13749	9523		#N/A	1.000000E+00
Gm13750	9524	0.143605682	15224	4.249002E-02
Gm13751	9525	-0.038406171	3065	5.892384E-01
Gm13752	9526	0.160304018	16323	2.335876E-02
Gm13753	9527		#N/A	1.000000E+00
Gm13754	9528	-0.038406171	3065	5.892384E-01
Gm13755	9529	0.17750575	17287	1.191754E-02
Gm13756	9530		#N/A	1.000000E+00
Gm13757	9531		#N/A	1.000000E+00
Gm13758	9532		#N/A	1.000000E+00
Gm13759	9533		#N/A	1.000000E+00
Gm13760	9534		#N/A	1.000000E+00
Gm13761	9535		#N/A	1.000000E+00
Gm13762	9536		#N/A	1.000000E+00
Gm13763	9537		#N/A	1.000000E+00
Gm13764	9538		#N/A	1.000000E+00
Gm13765	9539		#N/A	1.000000E+00
Gm13767	9540		#N/A	1.000000E+00
Gm13768	9541	0.194131481	17998	5.878558E-03
Gm13769	9542		#N/A	1.000000E+00
Gm13770	9543	0.086412184	11087.5	2.237298E-01
Gm13771	9544	0.029748956	7055	6.758233E-01
Gm13772	9545		#N/A	1.000000E+00

Spearman Rank correlation analysis performed between Prdm1 and all-expressed genes within the Meredith RNA-seq dataset. Robust Prdm1–associated genes were identified using a cut-off of $p < 0.0005$.

Table S1, Related to Supplemental Figure 3C. Prdm1 associated genes

Gm13773	9546		#N/A	1.000000E+00
Gm13775	9547		#N/A	1.000000E+00
Gm13776	9548	0.165207727	16671	1.939591E-02
Gm13777	9549	0.053267784	8685	4.537786E-01
Gm13778	9550	-0.038406171	3065	5.892384E-01
Gm13780	9551	-0.077394997	736.5	2.760195E-01
Gm13781	9552		#N/A	1.000000E+00
Gm13782	9553		#N/A	1.000000E+00
Gm13783	9554		#N/A	1.000000E+00
Gm13784	9555		#N/A	1.000000E+00
Gm13785	9556	-0.018103658	4360	7.991553E-01
Gm13786	9557		#N/A	1.000000E+00
Gm13787	9558		#N/A	1.000000E+00
Gm13788	9559		#N/A	1.000000E+00
Gm13789	9560	0.05249663	8499	4.603502E-01
Gm13790	9561		#N/A	1.000000E+00
Gm13791	9562	0.020875719	6412.5	7.692098E-01
Gm13792	9563		#N/A	1.000000E+00
Gm13793	9564		#N/A	1.000000E+00
Gm13794	9565		#N/A	1.000000E+00
Gm13795	9566		#N/A	1.000000E+00
Gm13796	9567		#N/A	1.000000E+00
Gm13797	9568		#N/A	1.000000E+00
Gm13798	9569	0.053267784	8685	4.537786E-01
Gm13799	9570		#N/A	1.000000E+00
Gm13800	9571		#N/A	1.000000E+00
Gm13801	9572		#N/A	1.000000E+00
Gm13802	9573		#N/A	1.000000E+00
Gm13803	9574		#N/A	1.000000E+00
Gm13804	9575	-0.054450825	1984	4.437997E-01
Gm13805	9576		#N/A	1.000000E+00
Gm13806	9577	0.053267784	8685	4.537786E-01
Gm13807	9578	0.030375908	7084	6.693894E-01
Gm13808	9579		#N/A	1.000000E+00
Gm13809	9580		#N/A	1.000000E+00
Gm13810	9581		#N/A	1.000000E+00
Gm13811	9582		#N/A	1.000000E+00
Gm13812	9583		#N/A	1.000000E+00
Gm13813	9584		#N/A	1.000000E+00
Gm13814	9585	0.001532305	5390	9.828195E-01
Gm13815	9586	0.15203904	15772	3.161938E-02
Gm13816	9587		#N/A	1.000000E+00
Gm13817	9588		#N/A	1.000000E+00

Spearman Rank correlation analysis performed between Prdm1 and all-expressed genes within the Meredith RNA-seq dataset. Robust Prdm1–associated genes were identified using a cut-off of $p < 0.0005$.

Table S1, Related to Supplemental Figure 3C. Prdm1 associated genes

Gm13818	9589		#N/A	1.000000E+00
Gm13819	9590		#N/A	1.000000E+00
Gm13820	9591		#N/A	1.000000E+00
Gm13821	9592		#N/A	1.000000E+00
Gm13822	9593	0.003915212	5513	9.561205E-01
Gm13823	9594		#N/A	1.000000E+00
Gm13824	9595	-0.006787075	4857	9.240105E-01
Gm13825	9596		#N/A	1.000000E+00
Gm13826	9597	0.087448212	11159	2.182058E-01
Gm13827	9598	-0.038406171	3065	5.892384E-01
Gm13828	9599		#N/A	1.000000E+00
Gm13829	9600		#N/A	1.000000E+00
Gm13830	9601	-0.059044806	1414	4.062502E-01
Gm13831	9602		#N/A	1.000000E+00
Gm13832	9603	0.083012292	10833	2.425524E-01
Gm13833	9604		#N/A	1.000000E+00
Gm13834	9605		#N/A	1.000000E+00
Gm13835	9606		#N/A	1.000000E+00
Gm13836	9607	0.113548679	13094	1.093908E-01
Gm13837	9608		#N/A	1.000000E+00
Gm13838	9609	0.113548679	13094	1.093908E-01
Gm13839	9610		#N/A	1.000000E+00
Gm13840	9611		#N/A	1.000000E+00
Gm13841	9612	0.113548679	13094	1.093908E-01
Gm13842	9613		#N/A	1.000000E+00
Gm13844	9614		#N/A	1.000000E+00
Gm13845	9615	0.102068551	12185	1.503835E-01
Gm13846	9616	-0.054451513	1703	4.437940E-01
Gm13847	9617	0.003160638	5485	9.645711E-01
Gm13848	9618		#N/A	1.000000E+00
Gm13849	9619	-0.077397959	691.5	2.760011E-01
Gm13850	9620		#N/A	1.000000E+00
Gm13851	9621	0.193353008	17961	6.083986E-03
Gm13852	9622		#N/A	1.000000E+00
Gm13853	9623	0.139174521	14901.5	4.936057E-02
Gm13854	9624	0.1544596	15936.5	2.897553E-02
Gm13855	9625		#N/A	1.000000E+00
Gm13856	9626		#N/A	1.000000E+00
Gm13857	9627	0.02401714	6754	7.356847E-01
Gm13858	9628		#N/A	1.000000E+00
Gm13859	9629	0.074574898	10201.5	2.939481E-01
Gm13860	9630		#N/A	1.000000E+00
Gm13861	9631		#N/A	1.000000E+00

Spearman Rank correlation analysis performed between Prdm1 and all-expressed genes within the Meredith RNA-seq dataset. Robust Prdm1–associated genes were identified using a cut-off of $p < 0.0005$.

Table S1, Related to Supplemental Figure 3C. Prdm1 associated genes

Gm13862	9632		#N/A	1.000000E+00
Gm13863	9633	-0.038406171	3065	5.892384E-01
Gm13864	9634		#N/A	1.000000E+00
Gm13865	9635		#N/A	1.000000E+00
Gm13866	9636		#N/A	1.000000E+00
Gm13867	9637		#N/A	1.000000E+00
Gm13868	9638	0.136427668	14723	5.406675E-02
Gm13869	9639		#N/A	1.000000E+00
Gm13870	9640		#N/A	1.000000E+00
Gm13871	9641		#N/A	1.000000E+00
Gm13872	9642		#N/A	1.000000E+00
Gm13873	9643	-0.067433705	973	3.427430E-01
Gm13874	9644		#N/A	1.000000E+00
Gm13875	9645	-0.11057391	159	1.190596E-01
Gm13876	9646		#N/A	1.000000E+00
Gm13877	9647		#N/A	1.000000E+00
Gm13878	9648		#N/A	1.000000E+00
Gm13879	9649	0.014301797	6015.5	8.406982E-01
Gm13880	9650		#N/A	1.000000E+00
Gm13881	9651	-0.038406171	3065	5.892384E-01
Gm13882	9652		#N/A	1.000000E+00
Gm13883	9653		#N/A	1.000000E+00
Gm13884	9654		#N/A	1.000000E+00
Gm13885	9655		#N/A	1.000000E+00
Gm13886	9656	0.218540914	18857	1.877660E-03
Gm13887	9657		#N/A	1.000000E+00
Gm13888	9658	0.113548679	13094	1.093908E-01
Gm13889	9659	0.022198316	6629.5	7.550388E-01
Gm13890	9660	0.106001553	12425	1.352007E-01
Gm13898	9661		#N/A	1.000000E+00
Gm13899	9662		#N/A	1.000000E+00
Gm13900	9663		#N/A	1.000000E+00
Gm13901	9664	0.082269134	10768	2.468098E-01
Gm13902	9665		#N/A	1.000000E+00
Gm13903	9666		#N/A	1.000000E+00
Gm13904	9667	0.113548679	13094	1.093908E-01
Gm13905	9668		#N/A	1.000000E+00
Gm13910	9669		#N/A	1.000000E+00
Gm13912	9670	0.111469887	12817.5	1.160799E-01
Gm13913	9671		#N/A	1.000000E+00
Gm13914	9672		#N/A	1.000000E+00
Gm13915	9673		#N/A	1.000000E+00
Gm13916	9674	0.053267784	8685	4.537786E-01

Spearman Rank correlation analysis performed between Prdm1 and all-expressed genes within the Meredith RNA-seq dataset. Robust Prdm1–associated genes were identified using a cut-off of $p < 0.0005$.

Table S1, Related to Supplemental Figure 3C. Prdm1 associated genes

Gm13919	9675	0.113548679	13094	1.093908E-01
Gm13920	9676		#N/A	1.000000E+00
Gm13921	9677		#N/A	1.000000E+00
Gm13922	9678	0.134200998	14569.5	5.814916E-02
Gm13923	9679		#N/A	1.000000E+00
Gm13924	9680		#N/A	1.000000E+00
Gm13925	9681	0.160987082	16412.5	2.276801E-02
Gm13928	9682		#N/A	1.000000E+00
Gm13929	9683		#N/A	1.000000E+00
Gm13930	9684		#N/A	1.000000E+00
Gm13932	9685		#N/A	1.000000E+00
Gm13935	9686	-0.029344249	3956	6.799891E-01
Gm13936	9687	-0.054451513	1703	4.437940E-01
Gm13937	9688		#N/A	1.000000E+00
Gm13938	9689	-0.066858259	1029.5	3.468868E-01
Gm13939	9690	0.113548679	13094	1.093908E-01
Gm13940	9691		#N/A	1.000000E+00
Gm13941	9692		#N/A	1.000000E+00
Gm13942	9693		#N/A	1.000000E+00
Gm13943	9694		#N/A	1.000000E+00
Gm13944	9695		#N/A	1.000000E+00
Gm13954	9696		#N/A	1.000000E+00
Gm13961	9697	0.109492511	12666.5	1.227347E-01
Gm13962	9698	-0.054451513	1703	4.437940E-01
Gm13963	9699	0.07124331	9933	3.161061E-01
Gm13964	9700		#N/A	1.000000E+00
Gm13965	9701		#N/A	1.000000E+00
Gm13966	9702		#N/A	1.000000E+00
Gm13967	9703		#N/A	1.000000E+00
Gm13972	9704		#N/A	1.000000E+00
Gm13973	9705		#N/A	1.000000E+00
Gm13974	9706	0.012185587	5886	8.640222E-01
Gm13975	9707		#N/A	1.000000E+00
Gm13976	9708		#N/A	1.000000E+00
Gm13977	9709	0.021317126	6513.5	7.644715E-01
Gm13981	9710	-0.038406171	3065	5.892384E-01
Gm13982	9711		#N/A	1.000000E+00
Gm13983	9712		#N/A	1.000000E+00
Gm13985	9713		#N/A	1.000000E+00
Gm13986	9714	-0.000385478	5264	9.956776E-01
Gm13988	9715		#N/A	1.000000E+00
Gm13989	9716		#N/A	1.000000E+00
Gm13990	9717	0.098384184	11948.5	1.657450E-01

Spearman Rank correlation analysis performed between Prdm1 and all-expressed genes within the Meredith RNA-seq dataset. Robust Prdm1–associated genes were identified using a cut-off of $p < 0.0005$.

Table S1, Related to Supplemental Figure 3C. Prdm1 associated genes

Gm13991	9718		#N/A	1.000000E+00
Gm13992	9719		#N/A	1.000000E+00
Gm13994	9720		#N/A	1.000000E+00
Gm13996	9721		#N/A	1.000000E+00
Gm13997	9722		#N/A	1.000000E+00
Gm13998	9723	-0.038406171	3065	5.892384E-01
Gm13999	9724	0.242240068	19399.5	5.483867E-04
Gm14000	9725	-0.038406171	3065	5.892384E-01
Gm14001	9726		#N/A	1.000000E+00
Gm14002	9727	0.022198129	6607.5	7.550408E-01
Gm14003	9728		#N/A	1.000000E+00
Gm14004	9729	-0.038406171	3065	5.892384E-01
Gm14005	9730	0.080135812	10605	2.593186E-01
Gm14006	9731		#N/A	1.000000E+00
Gm14007	9732	-0.038406171	3065	5.892384E-01
Gm14008	9733		#N/A	1.000000E+00
Gm14009	9734	-0.014336714	4553	8.403145E-01
Gm1401	9735		#N/A	1.000000E+00
Gm14010	9736		#N/A	1.000000E+00
Gm14011	9737		#N/A	1.000000E+00
Gm14012	9738		#N/A	1.000000E+00
Gm14013	9739		#N/A	1.000000E+00
Gm14014	9740		#N/A	1.000000E+00
Gm14015	9741	-0.066858259	1029.5	3.468868E-01
Gm14016	9742	-0.038406171	3065	5.892384E-01
Gm14017	9743		#N/A	1.000000E+00
Gm14018	9744		#N/A	1.000000E+00
Gm14019	9745		#N/A	1.000000E+00
Gm14020	9746	-0.038406171	3065	5.892384E-01
Gm14021	9747		#N/A	1.000000E+00
Gm14022	9748		#N/A	1.000000E+00
Gm14023	9749	-0.013165851	4609.5	8.532022E-01
Gm14024	9750	-0.066856562	1139	3.468991E-01
Gm14025	9751		#N/A	1.000000E+00
Gm14026	9752		#N/A	1.000000E+00
Gm14027	9753	0.012280354	5892	8.629750E-01
Gm14028	9754		#N/A	1.000000E+00
Gm14029	9755	0.008413232	5701	9.058796E-01
Gm14032	9756		#N/A	1.000000E+00
Gm14033	9757	-0.057249648	1452.5	4.206938E-01
Gm14034	9758	0.165986131	16703	1.882375E-02
Gm14035	9759		#N/A	1.000000E+00
Gm14036	9760	-0.054451513	1703	4.437940E-01

Spearman Rank correlation analysis performed between Prdm1 and all-expressed genes within the Meredith RNA-seq dataset. Robust Prdm1–associated genes were identified using a cut-off of $p < 0.0005$.

Table S1, Related to Supplemental Figure 3C. Prdm1 associated genes

Gm14037	9761		#N/A	1.000000E+00
Gm14038	9762	-0.038406171	3065	5.892384E-01
Gm14039	9763	0.080406705	10626.5	2.577065E-01
Gm14040	9764	-0.086755146	452	2.218903E-01
Gm14041	9765		#N/A	1.000000E+00
Gm14042	9766		#N/A	1.000000E+00
Gm14043	9767		#N/A	1.000000E+00
Gm14044	9768	0.167881089	16814	1.749173E-02
Gm14046	9769	-0.000841282	5208.5	9.905669E-01
Gm14047	9770	-0.016731569	4428.5	8.140896E-01
Gm14048	9771		#N/A	1.000000E+00
Gm14049	9772		#N/A	1.000000E+00
Gm14050	9773		#N/A	1.000000E+00
Gm14051	9774	0.076441859	10375	2.819943E-01
Gm14052	9775		#N/A	1.000000E+00
Gm14053	9776		#N/A	1.000000E+00
Gm14054	9777		#N/A	1.000000E+00
Gm14055	9778	-0.032450477	3820	6.482730E-01
Gm14056	9779	-0.054451513	1703	4.437940E-01
Gm14057	9780		#N/A	1.000000E+00
Gm14058	9781		#N/A	1.000000E+00
Gm14060	9782		#N/A	1.000000E+00
Gm14061	9783	0.133550827	14513	5.938804E-02
Gm14062	9784		#N/A	1.000000E+00
Gm14063	9785		#N/A	1.000000E+00
Gm14064	9786	-0.066858259	1029.5	3.468868E-01
Gm14065	9787	0.113548679	13094	1.093908E-01
Gm14066	9788	-0.025804166	4073	7.168297E-01
Gm14067	9789		#N/A	1.000000E+00
Gm14068	9790		#N/A	1.000000E+00
Gm14069	9791		#N/A	1.000000E+00
Gm14070	9792		#N/A	1.000000E+00
Gm14071	9793		#N/A	1.000000E+00
Gm14073	9794		#N/A	1.000000E+00
Gm14074	9795	0.077242095	10423	2.769722E-01
Gm14077	9796	0.114414374	13283	1.066960E-01
Gm14078	9797	0.021317126	6513.5	7.644715E-01
Gm14079	9798		#N/A	1.000000E+00
Gm14080	9799		#N/A	1.000000E+00
Gm14081	9800		#N/A	1.000000E+00
Gm14082	9801	-0.038406171	3065	5.892384E-01
Gm14083	9802		#N/A	1.000000E+00
Gm14085	9803	0.143605682	15224	4.249002E-02

Spearman Rank correlation analysis performed between Prdm1 and all-expressed genes within the Meredith RNA-seq dataset. Robust Prdm1–associated genes were identified using a cut-off of $p < 0.0005$.

Table S1, Related to Supplemental Figure 3C. Prdm1 associated genes

Gm14086	9804		#N/A	1.000000E+00
Gm14087	9805		#N/A	1.000000E+00
Gm14088	9806	0.053267784	8685	4.537786E-01
Gm14089	9807		#N/A	1.000000E+00
Gm14091	9808		#N/A	1.000000E+00
Gm14092	9809		#N/A	1.000000E+00
Gm14093	9810		#N/A	1.000000E+00
Gm14094	9811	0.064311388	9495	3.656050E-01
Gm14095	9812	0.014301797	6015.5	8.406982E-01
Gm14097	9813		#N/A	1.000000E+00
Gm14098	9814	0.053805701	8820	4.492258E-01
Gm14099	9815		#N/A	1.000000E+00
Gm14100	9816		#N/A	1.000000E+00
Gm14101	9817	0.031254132	7141	6.604173E-01
Gm14102	9818	-0.039426307	2484	5.793784E-01
Gm14103	9819		#N/A	1.000000E+00
Gm14104	9820		#N/A	1.000000E+00
Gm14105	9821		#N/A	1.000000E+00
Gm14106	9822		#N/A	1.000000E+00
Gm14107	9823		#N/A	1.000000E+00
Gm14108	9824		#N/A	1.000000E+00
Gm14109	9825		#N/A	1.000000E+00
Gm14110	9826	0.1544596	15936.5	2.897553E-02
Gm14111	9827	-0.038406171	3065	5.892384E-01
Gm14113	9828		#N/A	1.000000E+00
Gm14114	9829		#N/A	1.000000E+00
Gm14115	9830		#N/A	1.000000E+00
Gm14116	9831		#N/A	1.000000E+00
Gm14117	9832		#N/A	1.000000E+00
Gm14119	9833	0.113548679	13094	1.093908E-01
Gm14120	9834		#N/A	1.000000E+00
Gm14121	9835		#N/A	1.000000E+00
Gm14122	9836		#N/A	1.000000E+00
Gm14123	9837	-0.038406171	3065	5.892384E-01
Gm14124	9838	0.113548679	13094	1.093908E-01
Gm14125	9839	0.160304018	16323	2.335876E-02
Gm14126	9840		#N/A	1.000000E+00
Gm14127	9841		#N/A	1.000000E+00
Gm14128	9842		#N/A	1.000000E+00
Gm14129	9843		#N/A	1.000000E+00
Gm14130	9844	-0.038406171	3065	5.892384E-01
Gm14131	9845		#N/A	1.000000E+00
Gm14132	9846		#N/A	1.000000E+00

Spearman Rank correlation analysis performed between Prdm1 and all-expressed genes within the Meredith RNA-seq dataset. Robust Prdm1–associated genes were identified using a cut-off of $p < 0.0005$.

Table S1, Related to Supplemental Figure 3C. Prdm1 associated genes

Gm14133	9847	-0.0150378	4508	8.326176E-01
Gm14135	9848		#N/A	1.000000E+00
Gm14136	9849		#N/A	1.000000E+00
Gm14137	9850	-0.086435733	552	2.236031E-01
Gm14138	9851		#N/A	1.000000E+00
Gm14139	9852	0.143605682	15224	4.249002E-02
Gm14140	9853		#N/A	1.000000E+00
Gm14141	9854		#N/A	1.000000E+00
Gm14142	9855		#N/A	1.000000E+00
Gm14143	9856		#N/A	1.000000E+00
Gm14144	9857		#N/A	1.000000E+00
Gm14145	9858	-0.001606757	5107.5	9.819849E-01
Gm14146	9859	0.112003982	12854	1.143316E-01
Gm14147	9860		#N/A	1.000000E+00
Gm14148	9861		#N/A	1.000000E+00
Gm14149	9862		#N/A	1.000000E+00
Gm14150	9863	0.035127983	7338	6.214310E-01
Gm14151	9864	0.113548679	13094	1.093908E-01
Gm14152	9865	0.09604846	11778	1.760728E-01
Gm14153	9866		#N/A	1.000000E+00
Gm14154	9867	0.020434681	6357	7.739526E-01
Gm14155	9868	-0.038406171	3065	5.892384E-01
Gm14156	9869	0.053267784	8685	4.537786E-01
Gm14157	9870	-0.077394997	736.5	2.760195E-01
Gm14158	9871		#N/A	1.000000E+00
Gm14159	9872		#N/A	1.000000E+00
Gm14161	9873	0.132108418	14418	6.221420E-02
Gm14162	9874		#N/A	1.000000E+00
Gm14163	9875		#N/A	1.000000E+00
Gm14164	9876	0.123859632	13894	8.056881E-02
Gm14165	9877	0.00301757	5474	9.661739E-01
Gm14167	9878	0.199664175	18233	4.588636E-03
Gm14168	9879		#N/A	1.000000E+00
Gm14169	9880	-0.018105422	4347	7.991361E-01
Gm14170	9881		#N/A	1.000000E+00
Gm14172	9882	0.053267784	8685	4.537786E-01
Gm14173	9883	-0.038406171	3065	5.892384E-01
Gm14174	9884		#N/A	1.000000E+00
Gm14175	9885	-0.038406171	3065	5.892384E-01
Gm14176	9886	-0.103180568	220	1.459655E-01
Gm14177	9887		#N/A	1.000000E+00
Gm14178	9888		#N/A	1.000000E+00
Gm14179	9889		#N/A	1.000000E+00

Spearman Rank correlation analysis performed between Prdm1 and all-expressed genes within the Meredith RNA-seq dataset. Robust Prdm1–associated genes were identified using a cut-off of $p < 0.0005$.

Table S1, Related to Supplemental Figure 3C. Prdm1 associated genes

Gm14180	9890		#N/A	1.000000E+00
Gm14181	9891		#N/A	1.000000E+00
Gm14182	9892		#N/A	1.000000E+00
Gm14183	9893		#N/A	1.000000E+00
Gm14184	9894		#N/A	1.000000E+00
Gm14185	9895		#N/A	1.000000E+00
Gm14186	9896		#N/A	1.000000E+00
Gm14187	9897		#N/A	1.000000E+00
Gm14188	9898		#N/A	1.000000E+00
Gm14189	9899		#N/A	1.000000E+00
Gm14190	9900		#N/A	1.000000E+00
Gm14191	9901		#N/A	1.000000E+00
Gm14192	9902		#N/A	1.000000E+00
Gm14193	9903		#N/A	1.000000E+00
Gm14194	9904		#N/A	1.000000E+00
Gm14196	9905		#N/A	1.000000E+00
Gm14197	9906		#N/A	1.000000E+00
Gm14198	9907	-0.038406171	3065	5.892384E-01
Gm14199	9908		#N/A	1.000000E+00
Gm14200	9909	-0.038406171	3065	5.892384E-01
Gm14201	9910		#N/A	1.000000E+00
Gm14202	9911		#N/A	1.000000E+00
Gm14204	9912	0.136827758	14753	5.335899E-02
Gm14205	9913		#N/A	1.000000E+00
Gm14206	9914	-0.054450825	1984	4.437997E-01
Gm14207	9915	-0.011850199	4676	8.677300E-01
Gm14208	9916		#N/A	1.000000E+00
Gm14209	9917		#N/A	1.000000E+00
Gm14210	9918		#N/A	1.000000E+00
Gm14211	9919	0.113548679	13094	1.093908E-01
Gm14212	9920	-0.038406171	3065	5.892384E-01
Gm14213	9921		#N/A	1.000000E+00
Gm14215	9922		#N/A	1.000000E+00
Gm14216	9923	-0.003703707	4996.5	9.584887E-01
Gm14217	9924	0.08295169	10828	2.428977E-01
Gm14218	9925		#N/A	1.000000E+00
Gm14219	9926		#N/A	1.000000E+00
Gm14220	9927		#N/A	1.000000E+00
Gm14221	9928	0.113548679	13094	1.093908E-01
Gm14222	9929	-0.038406171	3065	5.892384E-01
Gm14223	9930	-0.038406171	3065	5.892384E-01
Gm14224	9931		#N/A	1.000000E+00
Gm14225	9932		#N/A	1.000000E+00

Spearman Rank correlation analysis performed between Prdm1 and all-expressed genes within the Meredith RNA-seq dataset. Robust Prdm1–associated genes were identified using a cut-off of $p < 0.0005$.

Table S1, Related to Supplemental Figure 3C. Prdm1 associated genes

Gm14226	9933		#N/A	1.000000E+00
Gm14227	9934	0.020875719	6412.5	7.692098E-01
Gm14228	9935		#N/A	1.000000E+00
Gm14229	9936		#N/A	1.000000E+00
Gm14230	9937		#N/A	1.000000E+00
Gm14231	9938	0.046025613	8011	5.175260E-01
Gm14232	9939	0.051571404	8436.5	4.683041E-01
Gm14233	9940	-0.038406171	3065	5.892384E-01
Gm14234	9941	0.160304018	16323	2.335876E-02
Gm14235	9942		#N/A	1.000000E+00
Gm14236	9943		#N/A	1.000000E+00
Gm14237	9944		#N/A	1.000000E+00
Gm14238	9945		#N/A	1.000000E+00
Gm14239	9946		#N/A	1.000000E+00
Gm14240	9947		#N/A	1.000000E+00
Gm14241	9948		#N/A	1.000000E+00
Gm14242	9949		#N/A	1.000000E+00
Gm14243	9950	0.160405811	16359	2.326989E-02
Gm14244	9951	-0.054450825	1984	4.437997E-01
Gm14245	9952		#N/A	1.000000E+00
Gm14246	9953		#N/A	1.000000E+00
Gm14247	9954		#N/A	1.000000E+00
Gm14248	9955	-0.066856562	1139	3.468991E-01
Gm14249	9956		#N/A	1.000000E+00
Gm14250	9957		#N/A	1.000000E+00
Gm14251	9958		#N/A	1.000000E+00
Gm14252	9959		#N/A	1.000000E+00
Gm14253	9960		#N/A	1.000000E+00
Gm14254	9961		#N/A	1.000000E+00
Gm14255	9962		#N/A	1.000000E+00
Gm14256	9963	0.117780885	13514	9.670959E-02
Gm14257	9964		#N/A	1.000000E+00
Gm14258	9965		#N/A	1.000000E+00
Gm14259	9966		#N/A	1.000000E+00
Gm14260	9967		#N/A	1.000000E+00
Gm14261	9968		#N/A	1.000000E+00
Gm14262	9969		#N/A	1.000000E+00
Gm14263	9970	-0.066856562	1139	3.468991E-01
Gm14264	9971	-0.077393517	797	2.760287E-01
Gm14265	9972		#N/A	1.000000E+00
Gm14266	9973	0.023213849	6705	7.442128E-01
Gm14267	9974		#N/A	1.000000E+00
Gm14268	9975		#N/A	1.000000E+00

Spearman Rank correlation analysis performed between Prdm1 and all-expressed genes within the Meredith RNA-seq dataset. Robust Prdm1–associated genes were identified using a cut-off of $p < 0.0005$.

Gm14269	9976		#N/A	1.000000E+00
Gm14270	9977	-0.038406171	3065	5.892384E-01
Gm14271	9978		#N/A	1.000000E+00
Gm14272	9979		#N/A	1.000000E+00
Gm14273	9980	0.113548679	13094	1.093908E-01
Gm14274	9981		#N/A	1.000000E+00
Gm14275	9982	0.026585227	6908	7.086414E-01
Gm14276	9983	-0.077394997	736.5	2.760195E-01
Gm14277	9984	0.009076312	5734	8.984999E-01
Gm14278	9985	0.074063958	10140	2.972775E-01
Gm14279	9986	-0.054451513	1703	4.437940E-01
Gm14280	9987	-0.066858259	1029.5	3.468868E-01
Gm14281	9988		#N/A	1.000000E+00
Gm14282	9989		#N/A	1.000000E+00
Gm14283	9990		#N/A	1.000000E+00
Gm14284	9991		#N/A	1.000000E+00
Gm14285	9992		#N/A	1.000000E+00
Gm14286	9993		#N/A	1.000000E+00
Gm14287	9994		#N/A	1.000000E+00
Gm14288	9995		#N/A	1.000000E+00
Gm14290	9996	0.048550359	8187	4.947934E-01
Gm14291	9997		#N/A	1.000000E+00
Gm14292	9998		#N/A	1.000000E+00
Gm14293	9999		#N/A	1.000000E+00
Gm14294	10000	-0.066856562	1139	3.468991E-01
Gm14295	10001	0.141631639	15054	4.544468E-02
Gm14296	10002		#N/A	1.000000E+00
Gm14297	10003		#N/A	1.000000E+00
Gm14298	10004		#N/A	1.000000E+00
Gm14299	10005		#N/A	1.000000E+00
Gm14300	10006		#N/A	1.000000E+00
Gm14301	10007		#N/A	1.000000E+00
Gm14302	10008	0.113548679	13094	1.093908E-01
Gm14303	10009		#N/A	1.000000E+00
Gm14304	10010		#N/A	1.000000E+00
Gm14305	10011	-0.004544711	4956.5	9.490742E-01
Gm14306	10012		#N/A	1.000000E+00
Gm14307	10013		#N/A	1.000000E+00
Gm14308	10014		#N/A	1.000000E+00
Gm14309	10015		#N/A	1.000000E+00
Gm14310	10016		#N/A	1.000000E+00
Gm14311	10017		#N/A	1.000000E+00
Gm14312	10018		#N/A	1.000000E+00

Table S1, Related to Supplemental Figure 3C. Prdm1 associated genes

Gm14313	10019		#N/A	1.000000E+00
Gm14314	10020		#N/A	1.000000E+00
Gm14317	10021	-0.038406171	3065	5.892384E-01
Gm14318	10022	-0.077397959	691.5	2.760011E-01
Gm14319	10023	0.229051473	19115	1.104513E-03
Gm14320	10024		#N/A	1.000000E+00
Gm14321	10025	-0.095280123	319	1.795721E-01
Gm14322	10026	-0.066858259	1029.5	3.468868E-01
Gm14323	10027	0.086412184	11087.5	2.237298E-01
Gm14324	10028		#N/A	1.000000E+00
Gm14325	10029	-0.080367313	637	2.579405E-01
Gm14326	10030	0.038664784	7538	5.867315E-01
Gm14327	10031		#N/A	1.000000E+00
Gm14328	10032	0.189988431	17842.5	7.047572E-03
Gm14329	10033		#N/A	1.000000E+00
Gm14330	10034		#N/A	1.000000E+00
Gm14331	10035		#N/A	1.000000E+00
Gm14332	10036		#N/A	1.000000E+00
Gm14333	10037		#N/A	1.000000E+00
Gm14334	10038		#N/A	1.000000E+00
Gm14335	10039		#N/A	1.000000E+00
Gm14336	10040	-0.054451513	1703	4.437940E-01
Gm14337	10041	-0.066858259	1029.5	3.468868E-01
Gm14339	10042		#N/A	1.000000E+00
Gm14340	10043		#N/A	1.000000E+00
Gm14341	10044		#N/A	1.000000E+00
Gm14342	10045	0.101170825	12130	1.540233E-01
Gm14343	10046	0.204838889	18419	3.618708E-03
Gm14344	10047		#N/A	1.000000E+00
Gm14345	10048		#N/A	1.000000E+00
Gm14346	10049		#N/A	1.000000E+00
Gm14347	10050		#N/A	1.000000E+00
Gm14348	10051		#N/A	1.000000E+00
Gm14349	10052		#N/A	1.000000E+00
Gm14350	10053		#N/A	1.000000E+00
Gm14351	10054		#N/A	1.000000E+00
Gm14352	10055		#N/A	1.000000E+00
Gm14353	10056		#N/A	1.000000E+00
Gm14354	10057		#N/A	1.000000E+00
Gm14355	10058		#N/A	1.000000E+00
Gm14356	10059		#N/A	1.000000E+00
Gm14357	10060		#N/A	1.000000E+00
Gm14358	10061		#N/A	1.000000E+00

Spearman Rank correlation analysis performed between Prdm1 and all-expressed genes within the Meredith RNA-seq dataset. Robust Prdm1–associated genes were identified using a cut-off of $p < 0.0005$.

Table S1, Related to Supplemental Figure 3C. Prdm1 associated genes

Gm14359	10062		#N/A	1.000000E+00
Gm14360	10063		#N/A	1.000000E+00
Gm14361	10064		#N/A	1.000000E+00
Gm14362	10065		#N/A	1.000000E+00
Gm14363	10066		#N/A	1.000000E+00
Gm14364	10067		#N/A	1.000000E+00
Gm14365	10068		#N/A	1.000000E+00
Gm14366	10069	-0.038406171	3065	5.892384E-01
Gm14367	10070		#N/A	1.000000E+00
Gm14368	10071		#N/A	1.000000E+00
Gm14369	10072		#N/A	1.000000E+00
Gm14370	10073		#N/A	1.000000E+00
Gm14371	10074	0.132773093	14462.5	6.089845E-02
Gm14372	10075		#N/A	1.000000E+00
Gm14373	10076	0.230235201	19161.5	1.038871E-03
Gm14374	10077		#N/A	1.000000E+00
Gm14375	10078		#N/A	1.000000E+00
Gm14376	10079		#N/A	1.000000E+00
Gm14377	10080		#N/A	1.000000E+00
Gm14378	10081	-0.077393517	797	2.760287E-01
Gm14379	10082	0.099425967	12027	1.612873E-01
Gm14380	10083		#N/A	1.000000E+00
Gm14381	10084		#N/A	1.000000E+00
Gm14382	10085		#N/A	1.000000E+00
Gm14383	10086		#N/A	1.000000E+00
Gm14385	10087		#N/A	1.000000E+00
Gm14387	10088		#N/A	1.000000E+00
Gm14388	10089		#N/A	1.000000E+00
Gm14389	10090	-0.054451513	1703	4.437940E-01
Gm14390	10091		#N/A	1.000000E+00
Gm14391	10092	-0.018319439	4327	7.968131E-01
Gm14392	10093		#N/A	1.000000E+00
Gm14393	10094	0.160304018	16323	2.335876E-02
Gm14394	10095		#N/A	1.000000E+00
Gm14395	10096		#N/A	1.000000E+00
Gm14396	10097		#N/A	1.000000E+00
Gm14397	10098		#N/A	1.000000E+00
Gm14398	10099		#N/A	1.000000E+00
Gm14399	10100		#N/A	1.000000E+00
Gm14400	10101	0.020875719	6412.5	7.692098E-01
Gm14401	10102	0.113548679	13094	1.093908E-01
Gm14402	10103	-0.038406171	3065	5.892384E-01
Gm14403	10104	0.13946837	14921	4.887795E-02

Spearman Rank correlation analysis performed between Prdm1 and all-expressed genes within the Meredith RNA-seq dataset. Robust Prdm1–associated genes were identified using a cut-off of $p < 0.0005$.

Table S1, Related to Supplemental Figure 3C. Prdm1 associated genes

Gm14404	10105		#N/A	1.000000E+00
Gm14405	10106		#N/A	1.000000E+00
Gm14406	10107	0.160987082	16412.5	2.276801E-02
Gm14407	10108	0.160304018	16323	2.335876E-02
Gm14408	10109		#N/A	1.000000E+00
Gm14409	10110		#N/A	1.000000E+00
Gm14410	10111		#N/A	1.000000E+00
Gm14411	10112		#N/A	1.000000E+00
Gm14412	10113		#N/A	1.000000E+00
Gm14413	10114		#N/A	1.000000E+00
Gm14414	10115		#N/A	1.000000E+00
Gm14415	10116		#N/A	1.000000E+00
Gm14416	10117		#N/A	1.000000E+00
Gm14417	10118		#N/A	1.000000E+00
Gm14418	10119		#N/A	1.000000E+00
Gm14419	10120		#N/A	1.000000E+00
Gm14420	10121	0.063365727	9418	3.727114E-01
Gm14421	10122		#N/A	1.000000E+00
Gm14422	10123		#N/A	1.000000E+00
Gm14423	10124		#N/A	1.000000E+00
Gm14424	10125		#N/A	1.000000E+00
Gm14425	10126		#N/A	1.000000E+00
Gm14426	10127		#N/A	1.000000E+00
Gm14427	10128	0.052728522	8550.5	4.583686E-01
Gm14428	10129		#N/A	1.000000E+00
Gm14429	10130		#N/A	1.000000E+00
Gm14431	10131		#N/A	1.000000E+00
Gm14434	10132		#N/A	1.000000E+00
Gm14435	10133		#N/A	1.000000E+00
Gm14437	10134		#N/A	1.000000E+00
Gm14438	10135		#N/A	1.000000E+00
Gm14439	10136	-0.037926739	3634	5.938986E-01
Gm14440	10137		#N/A	1.000000E+00
Gm14441	10138	-0.038406171	3065	5.892384E-01
Gm14442	10139		#N/A	1.000000E+00
Gm14443	10140	-0.033388476	3771	6.388155E-01
Gm14444	10141		#N/A	1.000000E+00
Gm14445	10142		#N/A	1.000000E+00
Gm14446	10143	0.052728522	8550.5	4.583686E-01
Gm14448	10144		#N/A	1.000000E+00
Gm14450	10145		#N/A	1.000000E+00
Gm14451	10146		#N/A	1.000000E+00
Gm14452	10147		#N/A	1.000000E+00

Spearman Rank correlation analysis performed between Prdm1 and all-expressed genes within the Meredith RNA-seq dataset. Robust Prdm1–associated genes were identified using a cut-off of $p < 0.0005$.

Table S1, Related to Supplemental Figure 3C. Prdm1 associated genes

Gm14453	10148	-0.054451513	1703	4.437940E-01
Gm14454	10149		#N/A	1.000000E+00
Gm14455	10150	0.068234091	9718	3.370317E-01
Gm14457	10151		#N/A	1.000000E+00
Gm14458	10152		#N/A	1.000000E+00
Gm14459	10153		#N/A	1.000000E+00
Gm14460	10154		#N/A	1.000000E+00
Gm14461	10155	-0.025600458	4083	7.189707E-01
Gm14463	10156		#N/A	1.000000E+00
Gm14464	10157		#N/A	1.000000E+00
Gm14465	10158		#N/A	1.000000E+00
Gm14466	10159		#N/A	1.000000E+00
Gm14467	10160		#N/A	1.000000E+00
Gm14469	10161		#N/A	1.000000E+00
Gm14470	10162		#N/A	1.000000E+00
Gm14471	10163	-0.038406171	3065	5.892384E-01
Gm14472	10164		#N/A	1.000000E+00
Gm14473	10165		#N/A	1.000000E+00
Gm14474	10166		#N/A	1.000000E+00
Gm14475	10167		#N/A	1.000000E+00
Gm14476	10168		#N/A	1.000000E+00
Gm14477	10169		#N/A	1.000000E+00
Gm14478	10170		#N/A	1.000000E+00
Gm14479	10171		#N/A	1.000000E+00
Gm14480	10172	0.053267784	8685	4.537786E-01
Gm14481	10173		#N/A	1.000000E+00
Gm14482	10174		#N/A	1.000000E+00
Gm14483	10175		#N/A	1.000000E+00
Gm14484	10176		#N/A	1.000000E+00
Gm14485	10177		#N/A	1.000000E+00
Gm14486	10178	-0.054450825	1984	4.437997E-01
Gm14487	10179		#N/A	1.000000E+00
Gm14488	10180	0.113548679	13094	1.093908E-01
Gm14489	10181		#N/A	1.000000E+00
Gm14490	10182	0.076815055	10404	2.796446E-01
Gm14491	10183		#N/A	1.000000E+00
Gm14492	10184	0.09716809	11850.5	1.710642E-01
Gm14493	10185		#N/A	1.000000E+00
Gm14494	10186		#N/A	1.000000E+00
Gm14496	10187	0.053267784	8685	4.537786E-01
Gm14497	10188		#N/A	1.000000E+00
Gm14499	10189		#N/A	1.000000E+00
Gm14500	10190		#N/A	1.000000E+00

Spearman Rank correlation analysis performed between Prdm1 and all-expressed genes within the Meredith RNA-seq dataset. Robust Prdm1–associated genes were identified using a cut-off of $p < 0.0005$.

Table S1, Related to Supplemental Figure 3C. Prdm1 associated genes

Gm14501	10191		#N/A	1.000000E+00
Gm14502	10192		#N/A	1.000000E+00
Gm14503	10193	-0.038406171	3065	5.892384E-01
Gm14504	10194		#N/A	1.000000E+00
Gm14505	10195	-0.066858259	1029.5	3.468868E-01
Gm14506	10196		#N/A	1.000000E+00
Gm14507	10197	-0.013166004	4608	8.532005E-01
Gm14508	10198		#N/A	1.000000E+00
Gm14509	10199		#N/A	1.000000E+00
Gm14510	10200		#N/A	1.000000E+00
Gm14511	10201		#N/A	1.000000E+00
Gm14512	10202		#N/A	1.000000E+00
Gm14513	10203	-0.066858259	1029.5	3.468868E-01
Gm14514	10204		#N/A	1.000000E+00
Gm14515	10205	-0.016731569	4428.5	8.140896E-01
Gm14516	10206	-0.054451513	1703	4.437940E-01
Gm14517	10207		#N/A	1.000000E+00
Gm14518	10208		#N/A	1.000000E+00
Gm14519	10209		#N/A	1.000000E+00
Gm14520	10210		#N/A	1.000000E+00
Gm14521	10211		#N/A	1.000000E+00
Gm14523	10212		#N/A	1.000000E+00
Gm14524	10213		#N/A	1.000000E+00
Gm14525	10214		#N/A	1.000000E+00
Gm14526	10215		#N/A	1.000000E+00
Gm14527	10216		#N/A	1.000000E+00
Gm14528	10217		#N/A	1.000000E+00
Gm14529	10218		#N/A	1.000000E+00
Gm14530	10219	0.156129434	16056.5	2.726377E-02
Gm14531	10220		#N/A	1.000000E+00
Gm14532	10221	0.037107831	7437	6.018971E-01
Gm14533	10222		#N/A	1.000000E+00
Gm14534	10223		#N/A	1.000000E+00
Gm14535	10224		#N/A	1.000000E+00
Gm14536	10225		#N/A	1.000000E+00
Gm14537	10226		#N/A	1.000000E+00
Gm14538	10227		#N/A	1.000000E+00
Gm14539	10228		#N/A	1.000000E+00
Gm14540	10229		#N/A	1.000000E+00
Gm14541	10230		#N/A	1.000000E+00
Gm14542	10231		#N/A	1.000000E+00
GM14543	10232		#N/A	1.000000E+00
Gm14543	10233		#N/A	1.000000E+00

Spearman Rank correlation analysis performed between Prdm1 and all-expressed genes within the Meredith RNA-seq dataset. Robust Prdm1–associated genes were identified using a cut-off of $p < 0.0005$.

Table S1, Related to Supplemental Figure 3C. Prdm1 associated genes

Gm14544	10234		#N/A	1.000000E+00
Gm14546	10235	0.109492511	12666.5	1.227347E-01
Gm14547	10236	-0.038406171	3065	5.892384E-01
Gm14548	10237	0.021316405	6469	7.644793E-01
Gm14549	10238		#N/A	1.000000E+00
Gm14550	10239		#N/A	1.000000E+00
Gm14551	10240	0.126933796	14110.5	7.327484E-02
Gm14552	10241		#N/A	1.000000E+00
Gm14553	10242	-0.054451513	1703	4.437940E-01
Gm14554	10243	0.038229357	7499.5	5.909551E-01
Gm14555	10244		#N/A	1.000000E+00
Gm14556	10245		#N/A	1.000000E+00
Gm14558	10246		#N/A	1.000000E+00
Gm14559	10247		#N/A	1.000000E+00
Gm14560	10248		#N/A	1.000000E+00
Gm14561	10249		#N/A	1.000000E+00
Gm14562	10250	-0.038406171	3065	5.892384E-01
Gm14563	10251		#N/A	1.000000E+00
Gm14564	10252	0.187258064	17729	7.927118E-03
Gm14565	10253	0.053267784	8685	4.537786E-01
Gm14566	10254		#N/A	1.000000E+00
Gm14567	10255	-0.038406171	3065	5.892384E-01
Gm14568	10256		#N/A	1.000000E+00
Gm14569	10257	0.053267784	8685	4.537786E-01
Gm14570	10258		#N/A	1.000000E+00
Gm14571	10259	-0.077397959	691.5	2.760011E-01
Gm14572	10260		#N/A	1.000000E+00
Gm14573	10261		#N/A	1.000000E+00
Gm14574	10262		#N/A	1.000000E+00
Gm14575	10263		#N/A	1.000000E+00
Gm14576	10264		#N/A	1.000000E+00
Gm14577	10265	-0.038406171	3065	5.892384E-01
Gm14578	10266		#N/A	1.000000E+00
Gm14579	10267		#N/A	1.000000E+00
Gm14580	10268		#N/A	1.000000E+00
Gm14581	10269		#N/A	1.000000E+00
Gm14582	10270		#N/A	1.000000E+00
Gm14584	10271		#N/A	1.000000E+00
Gm14585	10272		#N/A	1.000000E+00
Gm14586	10273		#N/A	1.000000E+00
Gm14587	10274		#N/A	1.000000E+00
Gm14588	10275		#N/A	1.000000E+00
Gm14589	10276		#N/A	1.000000E+00

Spearman Rank correlation analysis performed between Prdm1 and all-expressed genes within the Meredith RNA-seq dataset. Robust Prdm1–associated genes were identified using a cut-off of $p < 0.0005$.

Table S1, Related to Supplemental Figure 3C. Prdm1 associated genes

Gm14590	10277		#N/A	1.000000E+00
Gm14591	10278		#N/A	1.000000E+00
Gm14592	10279		#N/A	1.000000E+00
Gm14593	10280	-0.04661847	2270	5.121402E-01
Gm14594	10281		#N/A	1.000000E+00
Gm14595	10282		#N/A	1.000000E+00
Gm14596	10283		#N/A	1.000000E+00
Gm14597	10284		#N/A	1.000000E+00
Gm14598	10285		#N/A	1.000000E+00
Gm14599	10286		#N/A	1.000000E+00
Gm14600	10287		#N/A	1.000000E+00
Gm14601	10288		#N/A	1.000000E+00
Gm14602	10289	-0.054451513	1703	4.437940E-01
Gm14603	10290		#N/A	1.000000E+00
Gm14604	10291		#N/A	1.000000E+00
Gm14605	10292		#N/A	1.000000E+00
Gm14606	10293		#N/A	1.000000E+00
Gm14607	10294		#N/A	1.000000E+00
Gm14608	10295		#N/A	1.000000E+00
Gm14609	10296		#N/A	1.000000E+00
Gm14610	10297		#N/A	1.000000E+00
Gm14611	10298		#N/A	1.000000E+00
Gm14612	10299		#N/A	1.000000E+00
Gm14613	10300		#N/A	1.000000E+00
Gm14614	10301	0.157799267	16153.5	2.563935E-02
Gm14615	10302		#N/A	1.000000E+00
Gm14616	10303	0.113548679	13094	1.093908E-01
Gm14617	10304		#N/A	1.000000E+00
Gm14618	10305		#N/A	1.000000E+00
Gm14619	10306		#N/A	1.000000E+00
Gm14620	10307	0.053267784	8685	4.537786E-01
Gm14621	10308		#N/A	1.000000E+00
Gm14622	10309		#N/A	1.000000E+00
Gm14623	10310		#N/A	1.000000E+00
Gm14624	10311		#N/A	1.000000E+00
Gm14625	10312	-0.038406171	3065	5.892384E-01
Gm14626	10313		#N/A	1.000000E+00
Gm14627	10314		#N/A	1.000000E+00
Gm14628	10315		#N/A	1.000000E+00
Gm14629	10316		#N/A	1.000000E+00
Gm14630	10317		#N/A	1.000000E+00
Gm14631	10318		#N/A	1.000000E+00
Gm14632	10319		#N/A	1.000000E+00

Spearman Rank correlation analysis performed between Prdm1 and all-expressed genes within the Meredith RNA-seq dataset. Robust Prdm1–associated genes were identified using a cut-off of $p < 0.0005$.

Table S1, Related to Supplemental Figure 3C. Prdm1 associated genes

Gm14633	10320		#N/A	1.000000E+00
Gm14634	10321	0.066101155	9598	3.523868E-01
Gm14635	10322	-0.077394997	736.5	2.760195E-01
Gm14636	10323	-0.076317226	856.5	2.827820E-01
Gm14637	10324		#N/A	1.000000E+00
Gm14638	10325		#N/A	1.000000E+00
Gm14639	10326		#N/A	1.000000E+00
Gm14640	10327		#N/A	1.000000E+00
Gm14641	10328		#N/A	1.000000E+00
Gm14642	10329		#N/A	1.000000E+00
Gm14643	10330	0.132490652	14447	6.145473E-02
Gm14644	10331		#N/A	1.000000E+00
Gm14645	10332		#N/A	1.000000E+00
Gm14646	10333		#N/A	1.000000E+00
Gm14647	10334		#N/A	1.000000E+00
Gm14648	10335		#N/A	1.000000E+00
Gm14649	10336		#N/A	1.000000E+00
Gm14650	10337		#N/A	1.000000E+00
Gm14651	10338		#N/A	1.000000E+00
Gm14652	10339		#N/A	1.000000E+00
Gm14653	10340		#N/A	1.000000E+00
Gm14654	10341		#N/A	1.000000E+00
Gm14655	10342		#N/A	1.000000E+00
Gm14656	10343		#N/A	1.000000E+00
Gm14657	10344		#N/A	1.000000E+00
Gm14658	10345		#N/A	1.000000E+00
Gm14659	10346		#N/A	1.000000E+00
Gm14660	10347	0.152789767	15822	3.077826E-02
Gm14661	10348	0.035937227	7377	6.134136E-01
Gm14662	10349		#N/A	1.000000E+00
Gm14663	10350		#N/A	1.000000E+00
Gm14664	10351	-0.038406171	3065	5.892384E-01
Gm14665	10352		#N/A	1.000000E+00
Gm14666	10353		#N/A	1.000000E+00
Gm14667	10354		#N/A	1.000000E+00
Gm14668	10355		#N/A	1.000000E+00
Gm14669	10356		#N/A	1.000000E+00
Gm14670	10357		#N/A	1.000000E+00
Gm14671	10358		#N/A	1.000000E+00
Gm14672	10359		#N/A	1.000000E+00
Gm14673	10360		#N/A	1.000000E+00
Gm14674	10361		#N/A	1.000000E+00
Gm14675	10362		#N/A	1.000000E+00

Spearman Rank correlation analysis performed between Prdm1 and all-expressed genes within the Meredith RNA-seq dataset. Robust Prdm1–associated genes were identified using a cut-off of $p < 0.0005$.

Gm14676	10363		#N/A	1.000000E+00
Gm14677	10364		#N/A	1.000000E+00
Gm14678	10365		#N/A	1.000000E+00
Gm14679	10366		#N/A	1.000000E+00
Gm14680	10367		#N/A	1.000000E+00
Gm14681	10368	-0.016731055	4437	8.140953E-01
Gm14682	10369		#N/A	1.000000E+00
Gm14683	10370		#N/A	1.000000E+00
Gm14684	10371		#N/A	1.000000E+00
Gm14685	10372		#N/A	1.000000E+00
Gm14686	10373		#N/A	1.000000E+00
Gm14687	10374		#N/A	1.000000E+00
Gm14688	10375	0.053267784	8685	4.537786E-01
Gm14689	10376		#N/A	1.000000E+00
Gm14690	10377		#N/A	1.000000E+00
Gm14692	10378		#N/A	1.000000E+00
Gm14693	10379		#N/A	1.000000E+00
Gm14694	10380	-0.054450825	1984	4.437997E-01
Gm14695	10381		#N/A	1.000000E+00
Gm14696	10382		#N/A	1.000000E+00
Gm14697	10383	-0.086755146	452	2.218903E-01
Gm14698	10384	-0.018447724	4312.5	7.954215E-01
Gm14699	10385		#N/A	1.000000E+00
Gm14700	10386		#N/A	1.000000E+00
Gm14701	10387		#N/A	1.000000E+00
Gm14702	10388		#N/A	1.000000E+00
Gm14703	10389	0.126933796	14110.5	7.327484E-02
Gm14704	10390		#N/A	1.000000E+00
Gm14705	10391		#N/A	1.000000E+00
Gm14706	10392	-0.038406171	3065	5.892384E-01
Gm14707	10393	-0.038406171	3065	5.892384E-01
Gm14708	10394		#N/A	1.000000E+00
Gm14709	10395		#N/A	1.000000E+00
Gm14710	10396		#N/A	1.000000E+00
Gm14711	10397		#N/A	1.000000E+00
Gm14712	10398		#N/A	1.000000E+00
Gm14713	10399		#N/A	1.000000E+00
Gm14714	10400		#N/A	1.000000E+00
Gm14715	10401	-0.054450825	1984	4.437997E-01
Gm14716	10402		#N/A	1.000000E+00
Gm14717	10403		#N/A	1.000000E+00
Gm14718	10404	0.021452695	6549	7.630180E-01
Gm14719	10405		#N/A	1.000000E+00

Table S1, Related to Supplemental Figure 3C. Prdm1 associated genes

Gm14720	10406	0.143605682	15224	4.249002E-02
Gm14721	10407		#N/A	1.000000E+00
Gm14722	10408		#N/A	1.000000E+00
Gm14723	10409		#N/A	1.000000E+00
Gm14724	10410		#N/A	1.000000E+00
Gm14725	10411	0.143605682	15224	4.249002E-02
Gm14726	10412		#N/A	1.000000E+00
Gm14727	10413		#N/A	1.000000E+00
Gm14728	10414		#N/A	1.000000E+00
Gm14729	10415		#N/A	1.000000E+00
Gm14730	10416		#N/A	1.000000E+00
Gm14731	10417		#N/A	1.000000E+00
Gm14732	10418		#N/A	1.000000E+00
Gm14733	10419	-0.038406171	3065	5.892384E-01
Gm14734	10420		#N/A	1.000000E+00
Gm14735	10421		#N/A	1.000000E+00
Gm14736	10422		#N/A	1.000000E+00
Gm14737	10423		#N/A	1.000000E+00
Gm14738	10424		#N/A	1.000000E+00
Gm14739	10425		#N/A	1.000000E+00
Gm14740	10426		#N/A	1.000000E+00
Gm14741	10427		#N/A	1.000000E+00
Gm14742	10428		#N/A	1.000000E+00
Gm14743	10429		#N/A	1.000000E+00
Gm14744	10430		#N/A	1.000000E+00
Gm14745	10431		#N/A	1.000000E+00
Gm14746	10432	0.088187776	11217	2.143225E-01
Gm14747	10433	-0.038406171	3065	5.892384E-01
Gm14748	10434	-0.038406171	3065	5.892384E-01
Gm14749	10435		#N/A	1.000000E+00
Gm14750	10436		#N/A	1.000000E+00
Gm14751	10437		#N/A	1.000000E+00
Gm14752	10438	-0.038406171	3065	5.892384E-01
Gm14753	10439		#N/A	1.000000E+00
Gm14754	10440	-0.066858259	1029.5	3.468868E-01
Gm14755	10441		#N/A	1.000000E+00
Gm14756	10442		#N/A	1.000000E+00
Gm14757	10443		#N/A	1.000000E+00
Gm14758	10444		#N/A	1.000000E+00
Gm14759	10445		#N/A	1.000000E+00
Gm14760	10446		#N/A	1.000000E+00
Gm14761	10447	0.113548679	13094	1.093908E-01
Gm14762	10448	0.213663002	18705	2.382563E-03

Spearman Rank correlation analysis performed between Prdm1 and all-expressed genes within the Meredith RNA-seq dataset. Robust Prdm1–associated genes were identified using a cut-off of $p < 0.0005$.

Table S1, Related to Supplemental Figure 3C. Prdm1 associated genes

Gm14763	10449		#N/A	1.000000E+00
Gm14764	10450		#N/A	1.000000E+00
Gm14765	10451	0.157799267	16153.5	2.563935E-02
Gm14766	10452		#N/A	1.000000E+00
Gm14767	10453		#N/A	1.000000E+00
Gm14768	10454		#N/A	1.000000E+00
Gm14769	10455		#N/A	1.000000E+00
Gm14770	10456		#N/A	1.000000E+00
Gm14771	10457		#N/A	1.000000E+00
Gm14772	10458		#N/A	1.000000E+00
Gm14773	10459		#N/A	1.000000E+00
Gm14774	10460		#N/A	1.000000E+00
Gm14775	10461	0.105996145	12422	1.352207E-01
Gm14776	10462		#N/A	1.000000E+00
Gm14777	10463		#N/A	1.000000E+00
Gm14778	10464		#N/A	1.000000E+00
Gm14780	10465	0.219995286	18900	1.747229E-03
Gm14781	10466		#N/A	1.000000E+00
Gm14782	10467		#N/A	1.000000E+00
Gm14783	10468		#N/A	1.000000E+00
Gm14784	10469		#N/A	1.000000E+00
Gm14785	10470		#N/A	1.000000E+00
Gm14786	10471		#N/A	1.000000E+00
Gm14787	10472		#N/A	1.000000E+00
Gm14788	10473		#N/A	1.000000E+00
Gm14789	10474		#N/A	1.000000E+00
Gm14790	10475		#N/A	1.000000E+00
Gm14791	10476	-0.038406171	3065	5.892384E-01
Gm14792	10477		#N/A	1.000000E+00
Gm14793	10478		#N/A	1.000000E+00
Gm14794	10479	-0.038406171	3065	5.892384E-01
Gm14795	10480		#N/A	1.000000E+00
Gm14796	10481		#N/A	1.000000E+00
Gm14797	10482		#N/A	1.000000E+00
Gm14798	10483	0.06950556	9817	3.280846E-01
Gm14799	10484		#N/A	1.000000E+00
Gm14800	10485		#N/A	1.000000E+00
Gm14801	10486		#N/A	1.000000E+00
Gm14802	10487		#N/A	1.000000E+00
Gm14803	10488		#N/A	1.000000E+00
Gm14804	10489		#N/A	1.000000E+00
Gm14805	10490		#N/A	1.000000E+00
Gm14806	10491	-0.013118278	4616	8.537267E-01

Spearman Rank correlation analysis performed between Prdm1 and all-expressed genes within the Meredith RNA-seq dataset. Robust Prdm1–associated genes were identified using a cut-off of $p < 0.0005$.

Table S1, Related to Supplemental Figure 3C. Prdm1 associated genes

Gm14807	10492		#N/A	1.000000E+00
Gm14808	10493		#N/A	1.000000E+00
Gm14809	10494		#N/A	1.000000E+00
Gm14810	10495		#N/A	1.000000E+00
Gm14811	10496	0.113548679	13094	1.093908E-01
Gm14812	10497	0.046994573	8076	5.087386E-01
Gm14813	10498	0.113548679	13094	1.093908E-01
Gm14814	10499		#N/A	1.000000E+00
Gm14815	10500		#N/A	1.000000E+00
Gm14816	10501	0.215109181	18744.5	2.221323E-03
Gm14817	10502		#N/A	1.000000E+00
Gm14818	10503	0.116691532	13446.5	9.985642E-02
Gm14819	10504		#N/A	1.000000E+00
Gm14820	10505	0.182294195	17509.5	9.778799E-03
Gm14821	10506		#N/A	1.000000E+00
Gm14822	10507		#N/A	1.000000E+00
Gm14823	10508		#N/A	1.000000E+00
Gm14824	10509		#N/A	1.000000E+00
Gm14825	10510		#N/A	1.000000E+00
Gm14826	10511		#N/A	1.000000E+00
Gm14827	10512	-0.028921345	3966	6.843525E-01
Gm14828	10513		#N/A	1.000000E+00
Gm14829	10514		#N/A	1.000000E+00
Gm14830	10515		#N/A	1.000000E+00
Gm14831	10516		#N/A	1.000000E+00
Gm14832	10517		#N/A	1.000000E+00
Gm14833	10518		#N/A	1.000000E+00
Gm14834	10519	0.113548679	13094	1.093908E-01
Gm14835	10520		#N/A	1.000000E+00
Gm14836	10521		#N/A	1.000000E+00
Gm14837	10522		#N/A	1.000000E+00
Gm14838	10523		#N/A	1.000000E+00
Gm14839	10524		#N/A	1.000000E+00
Gm14840	10525		#N/A	1.000000E+00
Gm14841	10526		#N/A	1.000000E+00
Gm14842	10527		#N/A	1.000000E+00
Gm14843	10528		#N/A	1.000000E+00
Gm14844	10529		#N/A	1.000000E+00
Gm14845	10530		#N/A	1.000000E+00
Gm14846	10531	-0.054450825	1984	4.437997E-01
Gm14847	10532	-0.001224019	5151	9.862757E-01
Gm14848	10533		#N/A	1.000000E+00
Gm14849	10534		#N/A	1.000000E+00

Spearman Rank correlation analysis performed between Prdm1 and all-expressed genes within the Meredith RNA-seq dataset. Robust Prdm1–associated genes were identified using a cut-off of $p < 0.0005$.

Table S1, Related to Supplemental Figure 3C. Prdm1 associated genes

Gm14850	10535		#N/A	1.000000E+00
Gm14851	10536		#N/A	1.000000E+00
Gm14852	10537		#N/A	1.000000E+00
Gm14853	10538	0.1544596	15936.5	2.897553E-02
Gm14854	10539		#N/A	1.000000E+00
Gm14855	10540		#N/A	1.000000E+00
Gm14856	10541		#N/A	1.000000E+00
Gm14857	10542		#N/A	1.000000E+00
Gm14858	10543	-0.096707674	303	1.731109E-01
Gm14859	10544	0.046994573	8076	5.087386E-01
Gm14860	10545		#N/A	1.000000E+00
Gm14861	10546	-0.018447724	4312.5	7.954215E-01
Gm14862	10547		#N/A	1.000000E+00
Gm14863	10548		#N/A	1.000000E+00
Gm14864	10549		#N/A	1.000000E+00
Gm14865	10550		#N/A	1.000000E+00
Gm14866	10551		#N/A	1.000000E+00
Gm14867	10552		#N/A	1.000000E+00
Gm14868	10553		#N/A	1.000000E+00
Gm14869	10554		#N/A	1.000000E+00
Gm14870	10555		#N/A	1.000000E+00
Gm14871	10556	-0.066858259	1029.5	3.468868E-01
Gm14872	10557		#N/A	1.000000E+00
Gm14873	10558		#N/A	1.000000E+00
Gm14874	10559	-0.054451513	1703	4.437940E-01
Gm14875	10560		#N/A	1.000000E+00
Gm14876	10561		#N/A	1.000000E+00
Gm14877	10562	0.022714618	6671	7.495287E-01
Gm14878	10563		#N/A	1.000000E+00
Gm14879	10564	-0.038406171	3065	5.892384E-01
Gm14880	10565		#N/A	1.000000E+00
Gm14881	10566		#N/A	1.000000E+00
Gm14882	10567		#N/A	1.000000E+00
Gm14883	10568		#N/A	1.000000E+00
Gm14884	10569		#N/A	1.000000E+00
Gm14885	10570		#N/A	1.000000E+00
Gm14886	10571		#N/A	1.000000E+00
Gm14887	10572		#N/A	1.000000E+00
Gm14888	10573		#N/A	1.000000E+00
Gm14889	10574		#N/A	1.000000E+00
Gm14890	10575		#N/A	1.000000E+00
Gm14891	10576		#N/A	1.000000E+00
Gm14892	10577	0.178266262	17316	1.155253E-02

Spearman Rank correlation analysis performed between Prdm1 and all-expressed genes within the Meredith RNA-seq dataset. Robust Prdm1–associated genes were identified using a cut-off of $p < 0.0005$.

Table S1, Related to Supplemental Figure 3C. Prdm1 associated genes

Gm14893	10578		#N/A	1.000000E+00
Gm14894	10579		#N/A	1.000000E+00
Gm14895	10580	0.164478601	16623.5	1.994547E-02
Gm14896	10581		#N/A	1.000000E+00
Gm14897	10582		#N/A	1.000000E+00
Gm14898	10583		#N/A	1.000000E+00
Gm14899	10584	0.113548679	13094	1.093908E-01
Gm14900	10585		#N/A	1.000000E+00
Gm14901	10586	0.215438595	18765	2.186012E-03
Gm14902	10587		#N/A	1.000000E+00
Gm14903	10588	-0.054450825	1984	4.437997E-01
Gm14904	10589		#N/A	1.000000E+00
Gm14905	10590		#N/A	1.000000E+00
Gm14906	10591	0.143605682	15224	4.249002E-02
Gm14907	10592		#N/A	1.000000E+00
Gm14908	10593		#N/A	1.000000E+00
Gm14909	10594		#N/A	1.000000E+00
Gm14910	10595	-0.038406171	3065	5.892384E-01
Gm14911	10596		#N/A	1.000000E+00
Gm14912	10597	-0.038406171	3065	5.892384E-01
Gm14913	10598		#N/A	1.000000E+00
Gm14914	10599		#N/A	1.000000E+00
Gm14915	10600		#N/A	1.000000E+00
Gm14916	10601		#N/A	1.000000E+00
Gm14917	10602		#N/A	1.000000E+00
Gm14918	10603		#N/A	1.000000E+00
Gm14919	10604		#N/A	1.000000E+00
Gm14920	10605		#N/A	1.000000E+00
Gm14921	10606	-0.038406171	3065	5.892384E-01
Gm14922	10607		#N/A	1.000000E+00
Gm14923	10608	0.023709233	6735.5	7.389498E-01
Gm14925	10609		#N/A	1.000000E+00
Gm14926	10610		#N/A	1.000000E+00
Gm14927	10611		#N/A	1.000000E+00
Gm14928	10612		#N/A	1.000000E+00
Gm14929	10613		#N/A	1.000000E+00
Gm14930	10614		#N/A	1.000000E+00
Gm14931	10615		#N/A	1.000000E+00
Gm14932	10616		#N/A	1.000000E+00
Gm14933	10617		#N/A	1.000000E+00
Gm14934	10618		#N/A	1.000000E+00
Gm14935	10619		#N/A	1.000000E+00
Gm14936	10620	-0.054450825	1984	4.437997E-01

Spearman Rank correlation analysis performed between Prdm1 and all-expressed genes within the Meredith RNA-seq dataset. Robust Prdm1–associated genes were identified using a cut-off of $p < 0.0005$.

Table S1, Related to Supplemental Figure 3C. Prdm1 associated genes

Gm14937	10621		#N/A	1.000000E+00
Gm14938	10622	0.014301797	6015.5	8.406982E-01
Gm14939	10623	0.113548679	13094	1.093908E-01
Gm14940	10624		#N/A	1.000000E+00
Gm14941	10625		#N/A	1.000000E+00
Gm14942	10626		#N/A	1.000000E+00
Gm14943	10627		#N/A	1.000000E+00
Gm14944	10628		#N/A	1.000000E+00
Gm14945	10629		#N/A	1.000000E+00
Gm14946	10630		#N/A	1.000000E+00
Gm14947	10631		#N/A	1.000000E+00
Gm14950	10632		#N/A	1.000000E+00
Gm14951	10633		#N/A	1.000000E+00
Gm14957	10634	0.094644243	11681	1.825067E-01
Gm14963	10635	-0.009849908	4751	8.899014E-01
Gm14964	10636	-0.043688907	2381	5.390336E-01
Gm14965	10637	0.113548679	13094	1.093908E-01
Gm14966	10638	0.035066074	7333	6.220462E-01
Gm14967	10639		#N/A	1.000000E+00
Gm14968	10640		#N/A	1.000000E+00
Gm14969	10641		#N/A	1.000000E+00
Gm14970	10642		#N/A	1.000000E+00
Gm14971	10643		#N/A	1.000000E+00
Gm14972	10644		#N/A	1.000000E+00
Gm14973	10645		#N/A	1.000000E+00
Gm14974	10646		#N/A	1.000000E+00
Gm14975	10647	-0.000841282	5208.5	9.905669E-01
Gm14976	10648		#N/A	1.000000E+00
Gm14977	10649		#N/A	1.000000E+00
Gm14978	10650		#N/A	1.000000E+00
Gm14979	10651		#N/A	1.000000E+00
Gm14980	10652		#N/A	1.000000E+00
Gm14981	10653		#N/A	1.000000E+00
Gm14982	10654		#N/A	1.000000E+00
Gm14983	10655		#N/A	1.000000E+00
Gm14984	10656	-0.038406171	3065	5.892384E-01
Gm14985	10657		#N/A	1.000000E+00
Gm14986	10658		#N/A	1.000000E+00
Gm14987	10659		#N/A	1.000000E+00
Gm14988	10660		#N/A	1.000000E+00
Gm14989	10661	-0.038406171	3065	5.892384E-01
Gm14990	10662		#N/A	1.000000E+00
Gm14991	10663		#N/A	1.000000E+00

Spearman Rank correlation analysis performed between Prdm1 and all-expressed genes within the Meredith RNA-seq dataset. Robust Prdm1–associated genes were identified using a cut-off of $p < 0.0005$.

Table S1, Related to Supplemental Figure 3C. Prdm1 associated genes

Gm14992	10664		#N/A	1.000000E+00
Gm14993	10665		#N/A	1.000000E+00
Gm14994	10666		#N/A	1.000000E+00
Gm14995	10667		#N/A	1.000000E+00
Gm14996	10668		#N/A	1.000000E+00
Gm14997	10669		#N/A	1.000000E+00
Gm14998	10670		#N/A	1.000000E+00
Gm14999	10671		#N/A	1.000000E+00
Gm15000	10672		#N/A	1.000000E+00
Gm15001	10673		#N/A	1.000000E+00
Gm15002	10674	0.053267784	8685	4.537786E-01
Gm15003	10675		#N/A	1.000000E+00
Gm15004	10676		#N/A	1.000000E+00
Gm15005	10677		#N/A	1.000000E+00
Gm15006	10678		#N/A	1.000000E+00
Gm15007	10679	-0.038406171	3065	5.892384E-01
Gm15008	10680	0.143605682	15224	4.249002E-02
Gm15009	10681		#N/A	1.000000E+00
Gm15010	10682		#N/A	1.000000E+00
Gm15011	10683		#N/A	1.000000E+00
Gm15012	10684		#N/A	1.000000E+00
Gm15014	10685		#N/A	1.000000E+00
Gm15015	10686		#N/A	1.000000E+00
Gm15016	10687		#N/A	1.000000E+00
Gm15017	10688		#N/A	1.000000E+00
Gm15018	10689		#N/A	1.000000E+00
Gm15019	10690	-0.066856562	1139	3.468991E-01
Gm15020	10691		#N/A	1.000000E+00
Gm15021	10692		#N/A	1.000000E+00
Gm15022	10693		#N/A	1.000000E+00
Gm15023	10694		#N/A	1.000000E+00
Gm15024	10695	-0.038406171	3065	5.892384E-01
Gm15025	10696		#N/A	1.000000E+00
Gm15026	10697		#N/A	1.000000E+00
Gm15027	10698		#N/A	1.000000E+00
Gm15028	10699		#N/A	1.000000E+00
Gm15029	10700		#N/A	1.000000E+00
Gm15030	10701		#N/A	1.000000E+00
Gm15031	10702	0.136623399	14736	5.371954E-02
Gm15032	10703	0.148156609	15541	3.628793E-02
Gm15033	10704		#N/A	1.000000E+00
Gm15034	10705		#N/A	1.000000E+00
Gm15035	10706		#N/A	1.000000E+00

Spearman Rank correlation analysis performed between Prdm1 and all-expressed genes within the Meredith RNA-seq dataset. Robust Prdm1–associated genes were identified using a cut-off of $p < 0.0005$.

Table S1, Related to Supplemental Figure 3C. Prdm1 associated genes

Gm15036	10707		#N/A	1.000000E+00
Gm15037	10708		#N/A	1.000000E+00
Gm15038	10709		#N/A	1.000000E+00
Gm15039	10710		#N/A	1.000000E+00
Gm15040	10711		#N/A	1.000000E+00
Gm15041	10712		#N/A	1.000000E+00
Gm15042	10713		#N/A	1.000000E+00
Gm15043	10714		#N/A	1.000000E+00
Gm15044	10715		#N/A	1.000000E+00
Gm15045	10716		#N/A	1.000000E+00
Gm15046	10717	0.140899824	15014	4.658282E-02
Gm15047	10718		#N/A	1.000000E+00
Gm15048	10719		#N/A	1.000000E+00
Gm15049	10720		#N/A	1.000000E+00
Gm15050	10721		#N/A	1.000000E+00
Gm15051	10722	0.143605682	15224	4.249002E-02
Gm15052	10723		#N/A	1.000000E+00
Gm15053	10724	-0.05257233	2150	4.597028E-01
Gm15054	10725	-0.038406171	3065	5.892384E-01
Gm15055	10726	-0.054450825	1984	4.437997E-01
Gm15056	10727	-0.051544258	2171	4.685386E-01
Gm15057	10728		#N/A	1.000000E+00
Gm15058	10729		#N/A	1.000000E+00
Gm15060	10730		#N/A	1.000000E+00
Gm15061	10731		#N/A	1.000000E+00
Gm15062	10732		#N/A	1.000000E+00
Gm15063	10733	-0.038406171	3065	5.892384E-01
Gm15064	10734		#N/A	1.000000E+00
Gm15065	10735		#N/A	1.000000E+00
Gm15066	10736		#N/A	1.000000E+00
Gm15067	10737	0.038758411	7558.5	5.858252E-01
Gm15068	10738		#N/A	1.000000E+00
Gm15069	10739		#N/A	1.000000E+00
Gm15070	10740		#N/A	1.000000E+00
Gm15071	10741		#N/A	1.000000E+00
Gm15073	10742		#N/A	1.000000E+00
Gm15074	10743		#N/A	1.000000E+00
Gm15075	10744		#N/A	1.000000E+00
Gm15076	10745		#N/A	1.000000E+00
Gm15077	10746		#N/A	1.000000E+00
Gm15078	10747		#N/A	1.000000E+00
Gm15079	10748		#N/A	1.000000E+00
Gm15080	10749	0.113548679	13094	1.093908E-01

Spearman Rank correlation analysis performed between Prdm1 and all-expressed genes within the Meredith RNA-seq dataset. Robust Prdm1–associated genes were identified using a cut-off of $p < 0.0005$.

Table S1, Related to Supplemental Figure 3C. Prdm1 associated genes

Gm15081	10750		#N/A	1.000000E+00
Gm15082	10751	0.0815845	10706	2.507776E-01
Gm15083	10752		#N/A	1.000000E+00
Gm15084	10753		#N/A	1.000000E+00
Gm15085	10754		#N/A	1.000000E+00
Gm15086	10755		#N/A	1.000000E+00
Gm15087	10756		#N/A	1.000000E+00
Gm15088	10757		#N/A	1.000000E+00
Gm15089	10758		#N/A	1.000000E+00
Gm15090	10759		#N/A	1.000000E+00
Gm15091	10760		#N/A	1.000000E+00
Gm15092	10761		#N/A	1.000000E+00
Gm15093	10762		#N/A	1.000000E+00
Gm15094	10763		#N/A	1.000000E+00
Gm15095	10764		#N/A	1.000000E+00
Gm15096	10765		#N/A	1.000000E+00
Gm15097	10766	-0.038406171	3065	5.892384E-01
Gm15098	10767		#N/A	1.000000E+00
Gm15099	10768		#N/A	1.000000E+00
Gm15100	10769		#N/A	1.000000E+00
Gm15101	10770		#N/A	1.000000E+00
Gm15102	10771		#N/A	1.000000E+00
Gm15103	10772		#N/A	1.000000E+00
Gm15104	10773	-0.054450825	1984	4.437997E-01
Gm15105	10774		#N/A	1.000000E+00
Gm15106	10775		#N/A	1.000000E+00
Gm15107	10776	-0.054451513	1703	4.437940E-01
Gm15108	10777		#N/A	1.000000E+00
Gm15109	10778		#N/A	1.000000E+00
Gm15110	10779		#N/A	1.000000E+00
Gm15111	10780		#N/A	1.000000E+00
Gm15112	10781		#N/A	1.000000E+00
Gm15113	10782		#N/A	1.000000E+00
Gm15114	10783		#N/A	1.000000E+00
Gm15115	10784		#N/A	1.000000E+00
Gm15116	10785		#N/A	1.000000E+00
Gm15120	10786		#N/A	1.000000E+00
Gm15121	10787		#N/A	1.000000E+00
Gm15122	10788		#N/A	1.000000E+00
Gm15123	10789		#N/A	1.000000E+00
Gm15124	10790		#N/A	1.000000E+00
Gm15125	10791		#N/A	1.000000E+00
Gm15126	10792		#N/A	1.000000E+00

Spearman Rank correlation analysis performed between Prdm1 and all-expressed genes within the Meredith RNA-seq dataset. Robust Prdm1–associated genes were identified using a cut-off of $p < 0.0005$.

Table S1, Related to Supplemental Figure 3C. Prdm1 associated genes

Gm15127	10793	0.150673323	15691	3.319973E-02
Gm15128	10794	-0.001989505	5076	9.776944E-01
Gm15129	10795		#N/A	1.000000E+00
Gm15130	10796	-0.054451513	1703	4.437940E-01
Gm15131	10797	-0.000841282	5208.5	9.905669E-01
Gm15132	10798	0.215438595	18765	2.186012E-03
Gm15133	10799		#N/A	1.000000E+00
Gm15135	10800		#N/A	1.000000E+00
Gm15136	10801		#N/A	1.000000E+00
Gm15137	10802	-0.038406171	3065	5.892384E-01
Gm15138	10803		#N/A	1.000000E+00
Gm15140	10804		#N/A	1.000000E+00
Gm15141	10805		#N/A	1.000000E+00
Gm15142	10806		#N/A	1.000000E+00
Gm15143	10807		#N/A	1.000000E+00
Gm15145	10808		#N/A	1.000000E+00
Gm15146	10809		#N/A	1.000000E+00
Gm15147	10810		#N/A	1.000000E+00
Gm15148	10811	0.035669553	7358	6.160605E-01
Gm15149	10812		#N/A	1.000000E+00
Gm15151	10813	-0.038406171	3065	5.892384E-01
Gm15152	10814		#N/A	1.000000E+00
Gm15153	10815		#N/A	1.000000E+00
Gm15154	10816	0.053267784	8685	4.537786E-01
Gm15155	10817	0.222593758	18972	1.534589E-03
Gm15156	10818		#N/A	1.000000E+00
Gm15157	10819	-0.054451513	1703	4.437940E-01
Gm15158	10820		#N/A	1.000000E+00
Gm15159	10821	0.205571976	18439	3.497408E-03
Gm15160	10822		#N/A	1.000000E+00
Gm15161	10823		#N/A	1.000000E+00
Gm15162	10824	-0.018105422	4347	7.991361E-01
Gm15163	10825	0.020120596	6337.5	7.773353E-01
Gm15164	10826		#N/A	1.000000E+00
Gm15165	10827		#N/A	1.000000E+00
Gm15166	10828		#N/A	1.000000E+00
Gm15167	10829		#N/A	1.000000E+00
Gm15168	10830		#N/A	1.000000E+00
Gm15169	10831	-0.077393517	797	2.760287E-01
Gm15170	10832		#N/A	1.000000E+00
Gm15171	10833		#N/A	1.000000E+00
Gm15172	10834		#N/A	1.000000E+00
Gm15173	10835		#N/A	1.000000E+00

Spearman Rank correlation analysis performed between Prdm1 and all-expressed genes within the Meredith RNA-seq dataset. Robust Prdm1–associated genes were identified using a cut-off of $p < 0.0005$.

Table S1, Related to Supplemental Figure 3C. Prdm1 associated genes

Gm15174	10836		#N/A	1.000000E+00
Gm15175	10837		#N/A	1.000000E+00
Gm15176	10838		#N/A	1.000000E+00
Gm15177	10839		#N/A	1.000000E+00
Gm15178	10840		#N/A	1.000000E+00
Gm15179	10841	-0.038406171	3065	5.892384E-01
Gm15180	10842	0.157301384	16120	2.611477E-02
Gm15181	10843		#N/A	1.000000E+00
Gm15182	10844		#N/A	1.000000E+00
Gm15183	10845		#N/A	1.000000E+00
Gm15185	10846		#N/A	1.000000E+00
Gm15186	10847		#N/A	1.000000E+00
Gm15187	10848		#N/A	1.000000E+00
Gm15188	10849		#N/A	1.000000E+00
Gm15189	10850	0.135169958	14636.5	5.634245E-02
Gm15190	10851		#N/A	1.000000E+00
Gm15191	10852		#N/A	1.000000E+00
Gm15192	10853		#N/A	1.000000E+00
Gm15193	10854		#N/A	1.000000E+00
Gm15194	10855		#N/A	1.000000E+00
Gm15195	10856		#N/A	1.000000E+00
Gm15196	10857		#N/A	1.000000E+00
Gm15197	10858		#N/A	1.000000E+00
Gm15198	10859	0.13051396	14323	6.546580E-02
Gm15199	10860	-0.038406171	3065	5.892384E-01
Gm15200	10861		#N/A	1.000000E+00
Gm15201	10862	0.113548679	13094	1.093908E-01
Gm15202	10863		#N/A	1.000000E+00
Gm15203	10864		#N/A	1.000000E+00
Gm15204	10865	0.160985049	16395	2.276975E-02
Gm15205	10866		#N/A	1.000000E+00
Gm15206	10867		#N/A	1.000000E+00
Gm15207	10868		#N/A	1.000000E+00
Gm15208	10869	-0.038406171	3065	5.892384E-01
Gm15209	10870		#N/A	1.000000E+00
Gm15210	10871	0.151205412	15727	3.257621E-02
Gm15211	10872		#N/A	1.000000E+00
Gm15212	10873		#N/A	1.000000E+00
Gm15213	10874		#N/A	1.000000E+00
Gm15214	10875		#N/A	1.000000E+00
Gm15215	10876		#N/A	1.000000E+00
Gm15216	10877	-0.038406171	3065	5.892384E-01
Gm15217	10878		#N/A	1.000000E+00

Spearman Rank correlation analysis performed between Prdm1 and all-expressed genes within the Meredith RNA-seq dataset. Robust Prdm1–associated genes were identified using a cut-off of $p < 0.0005$.

Table S1, Related to Supplemental Figure 3C. Prdm1 associated genes

Gm15218	10879	0.173463964	17099	1.403269E-02
Gm15219	10880		#N/A	1.000000E+00
Gm15220	10881		#N/A	1.000000E+00
Gm15221	10882		#N/A	1.000000E+00
Gm15222	10883		#N/A	1.000000E+00
Gm15223	10884		#N/A	1.000000E+00
Gm15224	10885	0.052728522	8550.5	4.583686E-01
Gm15225	10886		#N/A	1.000000E+00
Gm15226	10887	0.156129434	16056.5	2.726377E-02
Gm15227	10888		#N/A	1.000000E+00
Gm15228	10889	-0.011049835	4701	8.765899E-01
Gm15229	10890		#N/A	1.000000E+00
Gm15230	10891	-0.038406171	3065	5.892384E-01
Gm15231	10892		#N/A	1.000000E+00
Gm15232	10893	-0.054450825	1984	4.437997E-01
Gm15233	10894		#N/A	1.000000E+00
Gm15234	10895		#N/A	1.000000E+00
Gm15235	10896	-0.086750697	472.5	2.219141E-01
Gm15236	10897		#N/A	1.000000E+00
Gm15237	10898		#N/A	1.000000E+00
Gm15238	10899		#N/A	1.000000E+00
Gm15239	10900	-0.054450825	1984	4.437997E-01
Gm15240	10901		#N/A	1.000000E+00
Gm15241	10902	0.118044943	13529	9.595879E-02
Gm15242	10903		#N/A	1.000000E+00
Gm15243	10904		#N/A	1.000000E+00
Gm15244	10905		#N/A	1.000000E+00
Gm15245	10906	-0.043104195	2404	5.444841E-01
Gm15246	10907	-0.086750697	472.5	2.219141E-01
Gm15247	10908		#N/A	1.000000E+00
Gm15249	10909		#N/A	1.000000E+00
Gm15250	10910		#N/A	1.000000E+00
Gm15251	10911		#N/A	1.000000E+00
Gm15252	10912		#N/A	1.000000E+00
Gm15253	10913		#N/A	1.000000E+00
Gm15254	10914		#N/A	1.000000E+00
Gm15255	10915		#N/A	1.000000E+00
Gm15256	10916		#N/A	1.000000E+00
Gm15257	10917		#N/A	1.000000E+00
Gm15258	10918		#N/A	1.000000E+00
Gm15259	10919		#N/A	1.000000E+00
Gm15260	10920		#N/A	1.000000E+00
Gm15261	10921	-0.032450477	3820	6.482730E-01

Spearman Rank correlation analysis performed between Prdm1 and all-expressed genes within the Meredith RNA-seq dataset. Robust Prdm1–associated genes were identified using a cut-off of $p < 0.0005$.

Table S1, Related to Supplemental Figure 3C. Prdm1 associated genes

Gm15262	10922	-0.038406171	3065	5.892384E-01
Gm15263	10923		#N/A	1.000000E+00
Gm15264	10924	-0.095273984	329	1.796002E-01
Gm15265	10925	-0.038406171	3065	5.892384E-01
Gm15266	10926	0.001354786	5380	9.848096E-01
Gm15267	10927		#N/A	1.000000E+00
Gm15268	10928	-0.038406171	3065	5.892384E-01
Gm15269	10929		#N/A	1.000000E+00
Gm1527	10930	-0.038406171	3065	5.892384E-01
Gm15270	10931	0.053805701	8820	4.492258E-01
Gm15271	10932		#N/A	1.000000E+00
Gm15272	10933		#N/A	1.000000E+00
Gm15273	10934		#N/A	1.000000E+00
Gm15274	10935		#N/A	1.000000E+00
Gm15275	10936		#N/A	1.000000E+00
Gm15276	10937		#N/A	1.000000E+00
Gm15279	10938		#N/A	1.000000E+00
Gm15280	10939		#N/A	1.000000E+00
Gm15281	10940		#N/A	1.000000E+00
Gm15282	10941		#N/A	1.000000E+00
Gm15283	10942	0.159364327	16244	2.419300E-02
Gm15284	10943		#N/A	1.000000E+00
Gm15285	10944		#N/A	1.000000E+00
Gm15286	10945	0.135796901	14680.5	5.519836E-02
Gm15287	10946	-0.038406171	3065	5.892384E-01
Gm15288	10947		#N/A	1.000000E+00
Gm15289	10948	0.051447952	8423	4.693711E-01
Gm15290	10949	0.046416802	8043	5.139690E-01
Gm15292	10950		#N/A	1.000000E+00
Gm15293	10951		#N/A	1.000000E+00
Gm15294	10952		#N/A	1.000000E+00
Gm15295	10953		#N/A	1.000000E+00
Gm15297	10954		#N/A	1.000000E+00
Gm15298	10955		#N/A	1.000000E+00
Gm15299	10956		#N/A	1.000000E+00
Gm15300	10957		#N/A	1.000000E+00
Gm15302	10958		#N/A	1.000000E+00
Gm15303	10959		#N/A	1.000000E+00
Gm15304	10960		#N/A	1.000000E+00
Gm15305	10961		#N/A	1.000000E+00
Gm15308	10962	-0.038406171	3065	5.892384E-01
Gm15309	10963		#N/A	1.000000E+00
Gm15310	10964	-0.038406171	3065	5.892384E-01

Spearman Rank correlation analysis performed between Prdm1 and all-expressed genes within the Meredith RNA-seq dataset. Robust Prdm1–associated genes were identified using a cut-off of $p < 0.0005$.

Table S1, Related to Supplemental Figure 3C. Prdm1 associated genes

Gm15311	10965		#N/A	1.000000E+00
Gm15312	10966		#N/A	1.000000E+00
Gm15313	10967		#N/A	1.000000E+00
Gm15314	10968		#N/A	1.000000E+00
Gm15315	10969		#N/A	1.000000E+00
Gm15316	10970		#N/A	1.000000E+00
Gm15317	10971	0.113548679	13094	1.093908E-01
Gm15318	10972	0.053267784	8685	4.537786E-01
Gm15319	10973		#N/A	1.000000E+00
Gm15320	10974	-0.012908807	4625	8.560368E-01
Gm15321	10975		#N/A	1.000000E+00
Gm15322	10976	-0.038406171	3065	5.892384E-01
Gm15323	10977	-0.054451513	1703	4.437940E-01
Gm15324	10978		#N/A	1.000000E+00
Gm15325	10979		#N/A	1.000000E+00
Gm15326	10980		#N/A	1.000000E+00
Gm15327	10981	0.02095764	6437.5	7.683297E-01
Gm15328	10982		#N/A	1.000000E+00
Gm15329	10983		#N/A	1.000000E+00
Gm15331	10984	-0.054450825	1984	4.437997E-01
Gm15332	10985		#N/A	1.000000E+00
Gm15333	10986		#N/A	1.000000E+00
Gm15334	10987		#N/A	1.000000E+00
Gm15335	10988	0.004903552	5559.5	9.450593E-01
Gm15336	10989	0.113548679	13094	1.093908E-01
Gm15337	10990	0.060351423	9228	3.959245E-01
Gm15338	10991		#N/A	1.000000E+00
Gm15339	10992		#N/A	1.000000E+00
Gm15340	10993	0.074574898	10201.5	2.939481E-01
Gm15341	10994	-0.037611332	3643.5	5.969735E-01
Gm15342	10995		#N/A	1.000000E+00
Gm15343	10996		#N/A	1.000000E+00
Gm15344	10997		#N/A	1.000000E+00
Gm15345	10998	-0.054450825	1984	4.437997E-01
Gm15346	10999	0.082269134	10768	2.468098E-01
Gm15347	11000	0.083242192	10850	2.412458E-01
Gm15348	11001	-0.066855996	1239.5	3.469032E-01
Gm15349	11002	0.131873214	14398	6.268535E-02
Gm15350	11003		#N/A	1.000000E+00
Gm15351	11004		#N/A	1.000000E+00
Gm15352	11005		#N/A	1.000000E+00
Gm15353	11006		#N/A	1.000000E+00
Gm15354	11007		#N/A	1.000000E+00

Spearman Rank correlation analysis performed between Prdm1 and all-expressed genes within the Meredith RNA-seq dataset. Robust Prdm1–associated genes were identified using a cut-off of $p < 0.0005$.

Table S1, Related to Supplemental Figure 3C. Prdm1 associated genes

Gm15355	11008	-0.038406171	3065	5.892384E-01
Gm15356	11009	-0.054450825	1984	4.437997E-01
Gm15357	11010		#N/A	1.000000E+00
Gm15358	11011		#N/A	1.000000E+00
Gm15359	11012		#N/A	1.000000E+00
Gm15361	11013		#N/A	1.000000E+00
Gm15362	11014		#N/A	1.000000E+00
Gm15363	11015		#N/A	1.000000E+00
Gm15364	11016		#N/A	1.000000E+00
Gm15365	11017		#N/A	1.000000E+00
Gm15366	11018	-0.038406171	3065	5.892384E-01
Gm15367	11019		#N/A	1.000000E+00
Gm15368	11020		#N/A	1.000000E+00
Gm15369	11021		#N/A	1.000000E+00
Gm15370	11022		#N/A	1.000000E+00
Gm15371	11023		#N/A	1.000000E+00
Gm15372	11024		#N/A	1.000000E+00
Gm15373	11025		#N/A	1.000000E+00
Gm15374	11026		#N/A	1.000000E+00
Gm15375	11027		#N/A	1.000000E+00
Gm15376	11028		#N/A	1.000000E+00
Gm15377	11029		#N/A	1.000000E+00
Gm15378	11030		#N/A	1.000000E+00
Gm15379	11031		#N/A	1.000000E+00
Gm15381	11032		#N/A	1.000000E+00
Gm15382	11033		#N/A	1.000000E+00
Gm15383	11034		#N/A	1.000000E+00
Gm15384	11035		#N/A	1.000000E+00
Gm15385	11036	-0.038406171	3065	5.892384E-01
Gm15386	11037	0.071667294	9964.5	3.132274E-01
Gm15387	11038		#N/A	1.000000E+00
Gm15388	11039	0.160987082	16412.5	2.276801E-02
Gm15389	11040	-0.054451513	1703	4.437940E-01
Gm15390	11041		#N/A	1.000000E+00
Gm15391	11042		#N/A	1.000000E+00
Gm15392	11043		#N/A	1.000000E+00
Gm15393	11044		#N/A	1.000000E+00
Gm15394	11045	-0.038406171	3065	5.892384E-01
Gm15395	11046		#N/A	1.000000E+00
Gm15396	11047	0.083256153	10852	2.411667E-01
Gm15397	11048	-0.014347514	4552	8.401958E-01
Gm15398	11049	0.072425441	10025	3.081225E-01
Gm15399	11050	-0.054450825	1984	4.437997E-01

Spearman Rank correlation analysis performed between Prdm1 and all-expressed genes within the Meredith RNA-seq dataset. Robust Prdm1–associated genes were identified using a cut-off of $p < 0.0005$.

Table S1, Related to Supplemental Figure 3C. Prdm1 associated genes

Gm15400	11051		#N/A	1.000000E+00
Gm15401	11052	-0.03641562	3676	6.086955E-01
Gm15402	11053		#N/A	1.000000E+00
Gm15403	11054		#N/A	1.000000E+00
Gm15404	11055	-0.054451513	1703	4.437940E-01
Gm15405	11056		#N/A	1.000000E+00
Gm15406	11057	0.175904409	17213	1.271943E-02
Gm15407	11058		#N/A	1.000000E+00
Gm15408	11059		#N/A	1.000000E+00
Gm15409	11060		#N/A	1.000000E+00
Gm15410	11061		#N/A	1.000000E+00
Gm15411	11062	0.183308051	17562	9.372176E-03
Gm15412	11063		#N/A	1.000000E+00
Gm15413	11064		#N/A	1.000000E+00
Gm15414	11065		#N/A	1.000000E+00
Gm15415	11066	-0.031504649	3858.5	6.578668E-01
Gm15416	11067	-0.054451513	1703	4.437940E-01
Gm15417	11068	0.15289972	15837	3.065669E-02
Gm15418	11069	-0.058728104	1421	4.087767E-01
Gm15419	11070		#N/A	1.000000E+00
Gm15420	11071	0.053805701	8820	4.492258E-01
Gm15421	11072		#N/A	1.000000E+00
Gm15422	11073		#N/A	1.000000E+00
Gm15423	11074		#N/A	1.000000E+00
Gm15425	11075	0.08570079	11031	2.275798E-01
Gm15426	11076		#N/A	1.000000E+00
Gm15427	11077	-0.038406171	3065	5.892384E-01
Gm15428	11078		#N/A	1.000000E+00
Gm15429	11079	0.113548679	13094	1.093908E-01
Gm15432	11080		#N/A	1.000000E+00
Gm15433	11081		#N/A	1.000000E+00
Gm15436	11082		#N/A	1.000000E+00
Gm15437	11083		#N/A	1.000000E+00
Gm15439	11084		#N/A	1.000000E+00
Gm15440	11085	0.108608001	12596	1.258056E-01
Gm15441	11086	0.218545756	18858	1.877212E-03
Gm15442	11087		#N/A	1.000000E+00
Gm15443	11088		#N/A	1.000000E+00
Gm15444	11089		#N/A	1.000000E+00
Gm15445	11090		#N/A	1.000000E+00
Gm15446	11091	0.017200525	6184	8.089775E-01
Gm15448	11092	0.053267784	8685	4.537786E-01
Gm15452	11093		#N/A	1.000000E+00

Spearman Rank correlation analysis performed between Prdm1 and all-expressed genes within the Meredith RNA-seq dataset. Robust Prdm1–associated genes were identified using a cut-off of $p < 0.0005$.

Table S1, Related to Supplemental Figure 3C. Prdm1 associated genes

Gm15453	11094		#N/A	1.000000E+00
Gm15454	11095		#N/A	1.000000E+00
Gm15455	11096	0.055037503	8929	4.388976E-01
Gm15457	11097		#N/A	1.000000E+00
Gm15458	11098		#N/A	1.000000E+00
Gm15459	11099	0.209274214	18558	2.939107E-03
Gm15460	11100		#N/A	1.000000E+00
Gm15461	11101		#N/A	1.000000E+00
Gm15462	11102		#N/A	1.000000E+00
Gm15464	11103	0.014301797	6015.5	8.406982E-01
Gm15465	11104		#N/A	1.000000E+00
Gm15466	11105		#N/A	1.000000E+00
Gm15467	11106	0.221407423	18944	1.628550E-03
Gm15469	11107		#N/A	1.000000E+00
Gm15470	11108		#N/A	1.000000E+00
Gm15471	11109	0.134200998	14569.5	5.814916E-02
Gm15472	11110		#N/A	1.000000E+00
Gm15473	11111	-0.038406171	3065	5.892384E-01
Gm15474	11112		#N/A	1.000000E+00
Gm15475	11113		#N/A	1.000000E+00
Gm15476	11114		#N/A	1.000000E+00
Gm15477	11115	0.082605987	10794.5	2.448737E-01
Gm15478	11116		#N/A	1.000000E+00
Gm15479	11117		#N/A	1.000000E+00
Gm15480	11118		#N/A	1.000000E+00
Gm15481	11119	-0.054451513	1703	4.437940E-01
Gm15482	11120		#N/A	1.000000E+00
Gm15483	11121	0.113548679	13094	1.093908E-01
Gm15484	11122	0.164478601	16623.5	1.994547E-02
Gm15485	11123	-0.038406171	3065	5.892384E-01
Gm15486	11124	0.020120596	6337.5	7.773353E-01
Gm15487	11125	0.119709523	13638	9.133183E-02
Gm15488	11126		#N/A	1.000000E+00
Gm15489	11127	0.015676693	6106.5	8.256174E-01
Gm15490	11128		#N/A	1.000000E+00
Gm15491	11129	0.049362576	8249	4.875945E-01
Gm15492	11130	-0.031923336	3850	6.536129E-01
Gm15493	11131		#N/A	1.000000E+00
Gm15494	11132	0.055981658	8984	4.310738E-01
Gm15495	11133	0.113548679	13094	1.093908E-01
Gm15496	11134		#N/A	1.000000E+00
Gm15497	11135		#N/A	1.000000E+00
Gm15498	11136	-0.077393517	797	2.760287E-01

Spearman Rank correlation analysis performed between Prdm1 and all-expressed genes within the Meredith RNA-seq dataset. Robust Prdm1–associated genes were identified using a cut-off of $p < 0.0005$.

Table S1, Related to Supplemental Figure 3C. Prdm1 associated genes

Gm15499	11137		#N/A	1.000000E+00
Gm15500	11138	-0.043185653	2396	5.437231E-01
Gm15501	11139	0.117358862	13482	9.791923E-02
Gm15502	11140		#N/A	1.000000E+00
Gm15503	11141		#N/A	1.000000E+00
Gm15504	11142		#N/A	1.000000E+00
Gm15506	11143	-0.108226343	193	1.271489E-01
Gm15507	11144	0.026327986	6888	7.113346E-01
Gm15508	11145		#N/A	1.000000E+00
Gm15509	11146		#N/A	1.000000E+00
Gm15510	11147		#N/A	1.000000E+00
Gm15511	11148	-0.038406171	3065	5.892384E-01
Gm15512	11149	-0.038406171	3065	5.892384E-01
Gm15513	11150	0.199285967	18215	4.667948E-03
Gm15514	11151	0.157334344	16121	2.608306E-02
Gm15515	11152		#N/A	1.000000E+00
Gm15516	11153	-0.038406171	3065	5.892384E-01
Gm15517	11154		#N/A	1.000000E+00
Gm15518	11155	0.132773093	14462.5	6.089845E-02
Gm15519	11156		#N/A	1.000000E+00
Gm15520	11157	0.02785819	6966	6.953679E-01
Gm15521	11158		#N/A	1.000000E+00
Gm15522	11159		#N/A	1.000000E+00
Gm15523	11160		#N/A	1.000000E+00
Gm15524	11161	-0.038406171	3065	5.892384E-01
Gm15525	11162		#N/A	1.000000E+00
Gm15526	11163		#N/A	1.000000E+00
Gm15527	11164	-0.000841282	5208.5	9.905669E-01
Gm15528	11165		#N/A	1.000000E+00
Gm15529	11166		#N/A	1.000000E+00
Gm1553	11167	-0.038406171	3065	5.892384E-01
Gm15530	11168		#N/A	1.000000E+00
Gm15531	11169	0.044087692	7873	5.353318E-01
Gm15532	11170		#N/A	1.000000E+00
Gm15533	11171		#N/A	1.000000E+00
Gm15534	11172		#N/A	1.000000E+00
Gm15535	11173		#N/A	1.000000E+00
Gm15536	11174		#N/A	1.000000E+00
Gm15537	11175		#N/A	1.000000E+00
Gm15538	11176		#N/A	1.000000E+00
Gm15539	11177		#N/A	1.000000E+00
Gm15540	11178	-0.038406171	3065	5.892384E-01
Gm15541	11179	-0.038406171	3065	5.892384E-01

Spearman Rank correlation analysis performed between Prdm1 and all-expressed genes within the Meredith RNA-seq dataset. Robust Prdm1–associated genes were identified using a cut-off of $p < 0.0005$.

Table S1, Related to Supplemental Figure 3C. Prdm1 associated genes

Gm15542	11180		#N/A	1.000000E+00
Gm15543	11181		#N/A	1.000000E+00
Gm15544	11182		#N/A	1.000000E+00
Gm15545	11183	0.120268202	13679	8.981942E-02
Gm15546	11184		#N/A	1.000000E+00
Gm15547	11185		#N/A	1.000000E+00
Gm15549	11186		#N/A	1.000000E+00
Gm15550	11187		#N/A	1.000000E+00
Gm15551	11188		#N/A	1.000000E+00
Gm15552	11189		#N/A	1.000000E+00
Gm15553	11190	-0.038406171	3065	5.892384E-01
Gm15554	11191	0.11976026	13647	9.119365E-02
Gm15555	11192	0.053267784	8685	4.537786E-01
Gm15556	11193	0.000307048	5309	9.965571E-01
Gm15557	11194		#N/A	1.000000E+00
Gm15558	11195		#N/A	1.000000E+00
Gm15559	11196	-0.054450825	1984	4.437997E-01
Gm15560	11197		#N/A	1.000000E+00
Gm15561	11198	-0.054450825	1984	4.437997E-01
Gm15562	11199		#N/A	1.000000E+00
Gm15563	11200	0.085246361	11005	2.300636E-01
Gm15564	11201	0.054345778	8886	4.446807E-01
Gm15565	11202	0.125958255	14040.5	7.552903E-02
Gm15566	11203		#N/A	1.000000E+00
Gm15567	11204	0.143605682	15224	4.249002E-02
Gm15568	11205	0.229051473	19115	1.104513E-03
Gm15569	11206		#N/A	1.000000E+00
Gm15570	11207	-0.038406171	3065	5.892384E-01
Gm15571	11208	0.113548679	13094	1.093908E-01
Gm15572	11209	0.143605682	15224	4.249002E-02
Gm15573	11210		#N/A	1.000000E+00
Gm15574	11211		#N/A	1.000000E+00
Gm15575	11212		#N/A	1.000000E+00
Gm15576	11213		#N/A	1.000000E+00
Gm15577	11214	-0.038406171	3065	5.892384E-01
Gm15578	11215	-0.054451513	1703	4.437940E-01
Gm15579	11216	-0.038406171	3065	5.892384E-01
Gm15580	11217	0.021317126	6513.5	7.644715E-01
Gm15581	11218	-0.013487614	4594	8.496565E-01
Gm15582	11219		#N/A	1.000000E+00
Gm15583	11220		#N/A	1.000000E+00
Gm15584	11221	0.154540397	15956.5	2.889064E-02
Gm15585	11222		#N/A	1.000000E+00

Spearman Rank correlation analysis performed between Prdm1 and all-expressed genes within the Meredith RNA-seq dataset. Robust Prdm1–associated genes were identified using a cut-off of $p < 0.0005$.

Table S1, Related to Supplemental Figure 3C. Prdm1 associated genes

Gm15586	11223		#N/A	1.000000E+00
Gm15587	11224	-0.054451513	1703	4.437940E-01
Gm15589	11225		#N/A	1.000000E+00
Gm15590	11226	0.047870267	8136	5.008643E-01
Gm15591	11227		#N/A	1.000000E+00
Gm15592	11228	0.053267784	8685	4.537786E-01
Gm15593	11229		#N/A	1.000000E+00
Gm15594	11230		#N/A	1.000000E+00
Gm15595	11231		#N/A	1.000000E+00
Gm15596	11232		#N/A	1.000000E+00
Gm15597	11233	-0.038406171	3065	5.892384E-01
Gm15598	11234		#N/A	1.000000E+00
Gm15599	11235	-0.038406171	3065	5.892384E-01
Gm156	11236	0.143605682	15224	4.249002E-02
Gm15600	11237	-0.066856562	1139	3.468991E-01
Gm15601	11238	0.133652732	14523	5.919244E-02
Gm15602	11239		#N/A	1.000000E+00
Gm15603	11240		#N/A	1.000000E+00
Gm15604	11241	0.012901672	5931	8.561156E-01
Gm15605	11242		#N/A	1.000000E+00
Gm15606	11243		#N/A	1.000000E+00
Gm15607	11244	0.022198316	6629.5	7.550388E-01
Gm15608	11245	-0.095267847	349	1.796284E-01
Gm15609	11246		#N/A	1.000000E+00
Gm15610	11247	-0.054451513	1703	4.437940E-01
Gm15611	11248		#N/A	1.000000E+00
Gm15612	11249	0.07521908	10268	2.897860E-01
Gm15613	11250	0.160987082	16412.5	2.276801E-02
Gm15614	11251	0.039852312	7632	5.752838E-01
Gm15615	11252		#N/A	1.000000E+00
Gm15616	11253		#N/A	1.000000E+00
Gm15617	11254		#N/A	1.000000E+00
Gm15618	11255		#N/A	1.000000E+00
Gm15619	11256	-0.066858259	1029.5	3.468868E-01
Gm15620	11257	0.01084242	5817	8.788885E-01
Gm15621	11258		#N/A	1.000000E+00
Gm15622	11259		#N/A	1.000000E+00
Gm15623	11260	0.205071268	18428	3.579853E-03
Gm15624	11261		#N/A	1.000000E+00
Gm15625	11262	0.160304018	16323	2.335876E-02
Gm15626	11263		#N/A	1.000000E+00
Gm15627	11264	-0.066855996	1239.5	3.469032E-01
Gm15628	11265		#N/A	1.000000E+00

Spearman Rank correlation analysis performed between Prdm1 and all-expressed genes within the Meredith RNA-seq dataset. Robust Prdm1–associated genes were identified using a cut-off of $p < 0.0005$.

Gm15629	11266	-0.054450825	1984	4.437997E-01
Gm15631	11267		#N/A	1.000000E+00
Gm15632	11268		#N/A	1.000000E+00
Gm15634	11269		#N/A	1.000000E+00
Gm15635	11270	0.020434681	6357	7.739526E-01
Gm15636	11271		#N/A	1.000000E+00
Gm15637	11272	-0.038406171	3065	5.892384E-01
Gm15638	11273	-0.032450477	3820	6.482730E-01
Gm1564	11274	0.053267784	8685	4.537786E-01
Gm15640	11275		#N/A	1.000000E+00
Gm15641	11276		#N/A	1.000000E+00
Gm15642	11277	0.109492511	12666.5	1.227347E-01
Gm15643	11278		#N/A	1.000000E+00
Gm15644	11279		#N/A	1.000000E+00
Gm15645	11280	0.124418529	13935	7.920087E-02
Gm15646	11281		#N/A	1.000000E+00
Gm15647	11282		#N/A	1.000000E+00
Gm15648	11283		#N/A	1.000000E+00
Gm15649	11284	-0.054451513	1703	4.437940E-01
Gm15650	11285		#N/A	1.000000E+00
Gm15651	11286		#N/A	1.000000E+00
Gm15652	11287		#N/A	1.000000E+00
Gm15653	11288	-0.038406171	3065	5.892384E-01
Gm15654	11289		#N/A	1.000000E+00
Gm15655	11290	0.015676693	6106.5	8.256174E-01
Gm15656	11291		#N/A	1.000000E+00
Gm15657	11292	-0.038406171	3065	5.892384E-01
Gm15658	11293	0.235526608	19277	7.869974E-04
Gm15659	11294		#N/A	1.000000E+00
Gm15660	11295		#N/A	1.000000E+00
Gm15661	11296	-0.038406171	3065	5.892384E-01
Gm15662	11297	0.021317126	6513.5	7.644715E-01
Gm15663	11298	-0.020923201	4207	7.686997E-01
Gm15664	11299		#N/A	1.000000E+00
Gm15665	11300		#N/A	1.000000E+00
Gm15666	11301		#N/A	1.000000E+00
Gm15667	11302		#N/A	1.000000E+00
Gm15668	11303	0.045056653	7941.5	5.263908E-01
Gm15669	11304	0.113548679	13094	1.093908E-01
Gm15670	11305		#N/A	1.000000E+00
Gm15671	11306		#N/A	1.000000E+00
Gm15672	11307		#N/A	1.000000E+00
Gm15673	11308		#N/A	1.000000E+00

Table S1, Related to Supplemental Figure 3C. Prdm1 associated genes

Gm15674	11309		#N/A	1.000000E+00
Gm15675	11310	-0.038406171	3065	5.892384E-01
Gm15676	11311		#N/A	1.000000E+00
Gm15677	11312		#N/A	1.000000E+00
Gm15678	11313		#N/A	1.000000E+00
Gm15679	11314	0.156487084	16079	2.690862E-02
Gm15680	11315	-0.088542228	430	2.124790E-01
Gm15681	11316		#N/A	1.000000E+00
Gm15682	11317	0.113548679	13094	1.093908E-01
Gm15684	11318	0.065901424	9582	3.538468E-01
Gm15685	11319		#N/A	1.000000E+00
Gm15686	11320	-0.110581467	154.5	1.190342E-01
Gm15687	11321		#N/A	1.000000E+00
Gm15688	11322		#N/A	1.000000E+00
Gm15689	11323	-0.066855996	1239.5	3.469032E-01
Gm15690	11324	0.029432427	7040	6.790806E-01
Gm15691	11325		#N/A	1.000000E+00
Gm15692	11326		#N/A	1.000000E+00
Gm15693	11327		#N/A	1.000000E+00
Gm15694	11328		#N/A	1.000000E+00
Gm15696	11329	0.139045799	14892.5	4.957323E-02
Gm15697	11330		#N/A	1.000000E+00
Gm15698	11331	0.120043024	13666	9.042658E-02
Gm15699	11332		#N/A	1.000000E+00
Gm15700	11333		#N/A	1.000000E+00
Gm15701	11334		#N/A	1.000000E+00
Gm15702	11335		#N/A	1.000000E+00
Gm15703	11336		#N/A	1.000000E+00
Gm15704	11337	0.160304018	16323	2.335876E-02
Gm15705	11338		#N/A	1.000000E+00
Gm15706	11339	0.03096103	7127	6.634064E-01
Gm15707	11340		#N/A	1.000000E+00
Gm15708	11341	-0.054451513	1703	4.437940E-01
Gm15709	11342		#N/A	1.000000E+00
Gm15710	11343	-0.064853076	1327.5	3.615723E-01
Gm15711	11344		#N/A	1.000000E+00
Gm15712	11345		#N/A	1.000000E+00
Gm15713	11346		#N/A	1.000000E+00
Gm15714	11347	0.113548679	13094	1.093908E-01
Gm15715	11348		#N/A	1.000000E+00
Gm15716	11349	0.07521908	10268	2.897860E-01
Gm15717	11350		#N/A	1.000000E+00
Gm15718	11351		#N/A	1.000000E+00

Spearman Rank correlation analysis performed between Prdm1 and all-expressed genes within the Meredith RNA-seq dataset. Robust Prdm1–associated genes were identified using a cut-off of $p < 0.0005$.

Table S1, Related to Supplemental Figure 3C. Prdm1 associated genes

Gm15719	11352	0.113548679	13094	1.093908E-01
Gm15720	11353		#N/A	1.000000E+00
Gm15721	11354	0.051571404	8436.5	4.683041E-01
Gm15722	11355	-0.038406171	3065	5.892384E-01
Gm15723	11356	-0.054450825	1984	4.437997E-01
Gm15724	11357		#N/A	1.000000E+00
Gm15725	11358	0.26009994	19625	1.995889E-04
Gm15726	11359		#N/A	1.000000E+00
Gm15727	11360	-0.004544711	4956.5	9.490742E-01
Gm15728	11361		#N/A	1.000000E+00
Gm15729	11362		#N/A	1.000000E+00
Gm15730	11363	0.053267784	8685	4.537786E-01
Gm15731	11364		#N/A	1.000000E+00
Gm15732	11365	0.195348153	18059	5.569905E-03
Gm15733	11366		#N/A	1.000000E+00
Gm15734	11367		#N/A	1.000000E+00
Gm15735	11368		#N/A	1.000000E+00
Gm15736	11369		#N/A	1.000000E+00
Gm15737	11370	0.07521908	10268	2.897860E-01
Gm15738	11371	0.021317126	6513.5	7.644715E-01
Gm15739	11372		#N/A	1.000000E+00
Gm15740	11373		#N/A	1.000000E+00
Gm15741	11374		#N/A	1.000000E+00
Gm15742	11375		#N/A	1.000000E+00
Gm15743	11376	-0.01403802	4566	8.435983E-01
Gm15744	11377		#N/A	1.000000E+00
Gm15745	11378	-0.004937246	4940	9.446824E-01
Gm15746	11379		#N/A	1.000000E+00
Gm15747	11380		#N/A	1.000000E+00
Gm15749	11381		#N/A	1.000000E+00
Gm15750	11382		#N/A	1.000000E+00
Gm15751	11383	-0.015374187	4496	8.289302E-01
Gm15752	11384		#N/A	1.000000E+00
Gm15753	11385	-0.038406171	3065	5.892384E-01
Gm15754	11386	0.1544596	15936.5	2.897553E-02
Gm15755	11387	-0.038406171	3065	5.892384E-01
Gm15756	11388	-0.038406171	3065	5.892384E-01
Gm15757	11389	0.03170671	7170	6.558125E-01
Gm15758	11390		#N/A	1.000000E+00
Gm15759	11391	0.038229357	7499.5	5.909551E-01
Gm15760	11392		#N/A	1.000000E+00
Gm15762	11393		#N/A	1.000000E+00
Gm15763	11394		#N/A	1.000000E+00

Spearman Rank correlation analysis performed between Prdm1 and all-expressed genes within the Meredith RNA-seq dataset. Robust Prdm1–associated genes were identified using a cut-off of $p < 0.0005$.

Table S1, Related to Supplemental Figure 3C. Prdm1 associated genes

Gm15764	11395	-0.066858259	1029.5	3.468868E-01
Gm15765	11396		#N/A	1.000000E+00
Gm15766	11397	-0.038406171	3065	5.892384E-01
Gm15767	11398	-0.07739401	763.5	2.760257E-01
Gm15770	11399		#N/A	1.000000E+00
Gm15772	11400	-0.066858259	1029.5	3.468868E-01
Gm15773	11401		#N/A	1.000000E+00
Gm15774	11402	-0.038406171	3065	5.892384E-01
Gm15775	11403		#N/A	1.000000E+00
Gm15776	11404	0.150673323	15691	3.319973E-02
Gm15777	11405		#N/A	1.000000E+00
Gm15778	11406		#N/A	1.000000E+00
Gm15779	11407	0.139335373	14912	4.909590E-02
Gm15780	11408		#N/A	1.000000E+00
Gm15781	11409		#N/A	1.000000E+00
Gm15782	11410		#N/A	1.000000E+00
Gm15783	11411		#N/A	1.000000E+00
Gm15784	11412		#N/A	1.000000E+00
Gm15785	11413		#N/A	1.000000E+00
Gm15786	11414		#N/A	1.000000E+00
Gm15787	11415	0.04611625	8018	5.167007E-01
Gm15788	11416		#N/A	1.000000E+00
Gm15789	11417		#N/A	1.000000E+00
Gm15790	11418		#N/A	1.000000E+00
Gm15791	11419		#N/A	1.000000E+00
Gm15792	11420		#N/A	1.000000E+00
Gm15793	11421		#N/A	1.000000E+00
Gm15794	11422	-0.077397959	691.5	2.760011E-01
Gm15795	11423		#N/A	1.000000E+00
Gm15796	11424	0.04777941	8127	5.016783E-01
Gm15797	11425		#N/A	1.000000E+00
Gm15798	11426	-0.036458426	3675	6.082741E-01
Gm15799	11427		#N/A	1.000000E+00
Gm15800	11428	0.057979896	9084	4.147825E-01
Gm15801	11429		#N/A	1.000000E+00
Gm15802	11430		#N/A	1.000000E+00
Gm15803	11431	-0.038406171	3065	5.892384E-01
Gm15806	11432		#N/A	1.000000E+00
Gm15807	11433		#N/A	1.000000E+00
Gm15808	11434		#N/A	1.000000E+00
Gm15809	11435		#N/A	1.000000E+00
Gm15810	11436	-0.038406171	3065	5.892384E-01
Gm15812	11437	0.160987082	16412.5	2.276801E-02

Spearman Rank correlation analysis performed between Prdm1 and all-expressed genes within the Meredith RNA-seq dataset. Robust Prdm1–associated genes were identified using a cut-off of $p < 0.0005$.

Table S1, Related to Supplemental Figure 3C. Prdm1 associated genes

Gm15813	11438		#N/A	1.000000E+00
Gm15815	11439		#N/A	1.000000E+00
Gm15816	11440	-0.038406171	3065	5.892384E-01
Gm15817	11441	0.094676268	11684	1.823581E-01
Gm15818	11442		#N/A	1.000000E+00
Gm15819	11443		#N/A	1.000000E+00
Gm15820	11444		#N/A	1.000000E+00
Gm15821	11445	-0.092611098	389	1.921265E-01
Gm15823	11446		#N/A	1.000000E+00
Gm15824	11447		#N/A	1.000000E+00
Gm15825	11448	0.090555233	11385.5	2.022247E-01
Gm15826	11449		#N/A	1.000000E+00
Gm15828	11450		#N/A	1.000000E+00
Gm15829	11451		#N/A	1.000000E+00
Gm15830	11452	-0.038406171	3065	5.892384E-01
Gm15831	11453	-0.038406171	3065	5.892384E-01
Gm15832	11454	0.11382315	13245	1.085306E-01
Gm15833	11455		#N/A	1.000000E+00
Gm15834	11456	-0.001224019	5151	9.862757E-01
Gm15835	11457		#N/A	1.000000E+00
Gm15836	11458		#N/A	1.000000E+00
Gm15838	11459		#N/A	1.000000E+00
Gm15839	11460	0.052728522	8550.5	4.583686E-01
Gm15840	11461		#N/A	1.000000E+00
Gm15841	11462		#N/A	1.000000E+00
Gm15842	11463		#N/A	1.000000E+00
Gm15843	11464	0.230247091	19167.5	1.038230E-03
Gm15844	11465		#N/A	1.000000E+00
Gm15845	11466	0.160304018	16323	2.335876E-02
Gm15846	11467		#N/A	1.000000E+00
Gm15847	11468		#N/A	1.000000E+00
Gm15848	11469	0.043970267	7863	5.364205E-01
Gm15849	11470		#N/A	1.000000E+00
Gm15850	11471		#N/A	1.000000E+00
Gm15851	11472	0.108608001	12596	1.258056E-01
Gm15852	11473		#N/A	1.000000E+00
Gm15853	11474		#N/A	1.000000E+00
Gm15854	11475	-0.038406171	3065	5.892384E-01
Gm15856	11476		#N/A	1.000000E+00
Gm15857	11477		#N/A	1.000000E+00
Gm15859	11478		#N/A	1.000000E+00
Gm15860	11479	-0.038406171	3065	5.892384E-01
Gm15861	11480		#N/A	1.000000E+00

Spearman Rank correlation analysis performed between Prdm1 and all-expressed genes within the Meredith RNA-seq dataset. Robust Prdm1–associated genes were identified using a cut-off of $p < 0.0005$.

Table S1, Related to Supplemental Figure 3C. Prdm1 associated genes

Gm15862	11481	0.1263153	14066	7.469757E-02
Gm15863	11482		#N/A	1.000000E+00
Gm15864	11483		#N/A	1.000000E+00
Gm15865	11484		#N/A	1.000000E+00
Gm15866	11485	0.020875719	6412.5	7.692098E-01
Gm15867	11486		#N/A	1.000000E+00
Gm15868	11487		#N/A	1.000000E+00
Gm15869	11488		#N/A	1.000000E+00
Gm1587	11489		#N/A	1.000000E+00
Gm15870	11490		#N/A	1.000000E+00
Gm15872	11491		#N/A	1.000000E+00
Gm15873	11492		#N/A	1.000000E+00
Gm15875	11493		#N/A	1.000000E+00
Gm15877	11494	0.1544596	15936.5	2.897553E-02
Gm15878	11495		#N/A	1.000000E+00
Gm15879	11496	0.021100249	6443	7.667985E-01
Gm15880	11497	0.038229357	7499.5	5.909551E-01
Gm15881	11498		#N/A	1.000000E+00
Gm15882	11499		#N/A	1.000000E+00
Gm15883	11500	0.098317006	11938	1.660356E-01
Gm15884	11501	0.167617989	16803	1.767163E-02
Gm15885	11502	-0.038406171	3065	5.892384E-01
Gm15886	11503	0.22727588	19085	1.210138E-03
Gm15888	11504	-0.016730711	4448.5	8.140990E-01
Gm15889	11505		#N/A	1.000000E+00
Gm15890	11506	-0.038406171	3065	5.892384E-01
Gm15891	11507	-0.077397959	691.5	2.760011E-01
Gm15892	11508	-0.103164533	236	1.460285E-01
Gm15893	11509	-0.038406171	3065	5.892384E-01
Gm15894	11510	-0.038406171	3065	5.892384E-01
Gm15895	11511		#N/A	1.000000E+00
Gm15896	11512	0.029748956	7055	6.758233E-01
Gm15897	11513		#N/A	1.000000E+00
Gm15898	11514		#N/A	1.000000E+00
Gm15899	11515		#N/A	1.000000E+00
Gm15900	11516		#N/A	1.000000E+00
Gm15901	11517		#N/A	1.000000E+00
Gm15902	11518		#N/A	1.000000E+00
Gm15903	11519		#N/A	1.000000E+00
Gm15904	11520	-0.038406171	3065	5.892384E-01
Gm15905	11521		#N/A	1.000000E+00
Gm15906	11522		#N/A	1.000000E+00
Gm15907	11523		#N/A	1.000000E+00

Spearman Rank correlation analysis performed between Prdm1 and all-expressed genes within the Meredith RNA-seq dataset. Robust Prdm1–associated genes were identified using a cut-off of $p < 0.0005$.

Table S1, Related to Supplemental Figure 3C. Prdm1 associated genes

Gm15908	11524	0.113548679	13094	1.093908E-01
Gm15910	11525	-0.018105422	4347	7.991361E-01
Gm15911	11526		#N/A	1.000000E+00
Gm15912	11527		#N/A	1.000000E+00
Gm15913	11528		#N/A	1.000000E+00
Gm15914	11529		#N/A	1.000000E+00
Gm15915	11530	0.157799267	16153.5	2.563935E-02
Gm15916	11531		#N/A	1.000000E+00
Gm15917	11532	-0.054451513	1703	4.437940E-01
Gm15918	11533	0.206475277	18477	3.353000E-03
Gm15919	11534	0.113548679	13094	1.093908E-01
Gm15920	11535		#N/A	1.000000E+00
Gm15921	11536		#N/A	1.000000E+00
Gm15922	11537		#N/A	1.000000E+00
Gm15923	11538		#N/A	1.000000E+00
Gm15925	11539		#N/A	1.000000E+00
Gm15926	11540		#N/A	1.000000E+00
Gm15927	11541	0.167475151	16796	1.776996E-02
Gm15928	11542		#N/A	1.000000E+00
Gm15929	11543	0.160304018	16323	2.335876E-02
Gm15930	11544		#N/A	1.000000E+00
Gm15931	11545		#N/A	1.000000E+00
Gm15932	11546	0.17482941	17158	1.328399E-02
Gm15933	11547		#N/A	1.000000E+00
Gm15934	11548		#N/A	1.000000E+00
Gm15935	11549		#N/A	1.000000E+00
Gm15936	11550		#N/A	1.000000E+00
Gm15937	11551	0.167147312	16779	1.799747E-02
Gm15938	11552	0.043531858	7835	5.404948E-01
Gm15939	11553	-0.054451513	1703	4.437940E-01
Gm15940	11554		#N/A	1.000000E+00
Gm15941	11555	-0.038406171	3065	5.892384E-01
Gm15942	11556	-0.054450825	1984	4.437997E-01
Gm15943	11557	0.135716015	14672	5.534488E-02
Gm15945	11558		#N/A	1.000000E+00
Gm15946	11559		#N/A	1.000000E+00
Gm15947	11560	0.113548679	13094	1.093908E-01
Gm15948	11561		#N/A	1.000000E+00
Gm15949	11562		#N/A	1.000000E+00
Gm15950	11563		#N/A	1.000000E+00
Gm15951	11564		#N/A	1.000000E+00
Gm15952	11565	0.257572431	19597	2.313069E-04
Gm15953	11566		#N/A	1.000000E+00

Spearman Rank correlation analysis performed between Prdm1 and all-expressed genes within the Meredith RNA-seq dataset. Robust Prdm1–associated genes were identified using a cut-off of $p < 0.0005$.

Table S1, Related to Supplemental Figure 3C. Prdm1 associated genes

Gm15954	11567		#N/A	1.000000E+00
Gm15956	11568	-0.077393517	797	2.760287E-01
Gm15957	11569		#N/A	1.000000E+00
Gm15958	11570	-0.066856562	1139	3.468991E-01
Gm15959	11571	-0.066856562	1139	3.468991E-01
Gm15961	11572		#N/A	1.000000E+00
Gm15962	11573	0.113548679	13094	1.093908E-01
Gm15963	11574		#N/A	1.000000E+00
Gm15964	11575	-0.038406171	3065	5.892384E-01
Gm15965	11576		#N/A	1.000000E+00
Gm15966	11577		#N/A	1.000000E+00
Gm15967	11578	-0.038406171	3065	5.892384E-01
Gm15968	11579		#N/A	1.000000E+00
Gm15969	11580		#N/A	1.000000E+00
Gm15970	11581		#N/A	1.000000E+00
Gm15971	11582		#N/A	1.000000E+00
Gm15972	11583		#N/A	1.000000E+00
Gm15974	11584		#N/A	1.000000E+00
Gm15975	11585		#N/A	1.000000E+00
Gm15976	11586	0.153441014	15860	3.006413E-02
Gm15978	11587		#N/A	1.000000E+00
Gm15979	11588		#N/A	1.000000E+00
Gm15980	11589		#N/A	1.000000E+00
Gm15981	11590		#N/A	1.000000E+00
Gm15982	11591		#N/A	1.000000E+00
Gm15983	11592	0.139174521	14901.5	4.936057E-02
Gm15984	11593	0.053805701	8820	4.492258E-01
Gm15985	11594	0.113548679	13094	1.093908E-01
Gm15986	11595		#N/A	1.000000E+00
Gm15987	11596	0.100828939	12112	1.554268E-01
Gm15988	11597		#N/A	1.000000E+00
Gm15989	11598		#N/A	1.000000E+00
Gm15990	11599		#N/A	1.000000E+00
Gm15991	11600	-0.077397959	691.5	2.760011E-01
Gm15992	11601		#N/A	1.000000E+00
Gm15994	11602	-0.066855996	1239.5	3.469032E-01
Gm15995	11603		#N/A	1.000000E+00
Gm15996	11604	0.143605682	15224	4.249002E-02
Gm15997	11605	0.022714618	6671	7.495287E-01
Gm15998	11606	-0.038406171	3065	5.892384E-01
Gm15999	11607		#N/A	1.000000E+00
Gm16000	11608	0.053267784	8685	4.537786E-01
Gm16001	11609		#N/A	1.000000E+00

Spearman Rank correlation analysis performed between Prdm1 and all-expressed genes within the Meredith RNA-seq dataset. Robust Prdm1–associated genes were identified using a cut-off of $p < 0.0005$.

Table S1, Related to Supplemental Figure 3C. Prdm1 associated genes

Gm16004	11610	-0.054451513	1703	4.437940E-01
Gm16005	11611	0.12097631	13721	8.793130E-02
Gm16006	11612		#N/A	1.000000E+00
Gm16007	11613		#N/A	1.000000E+00
Gm16008	11614		#N/A	1.000000E+00
Gm16010	11615	-0.018138782	4334.5	7.987739E-01
Gm16011	11616		#N/A	1.000000E+00
Gm16012	11617	0.143605682	15224	4.249002E-02
Gm16013	11618		#N/A	1.000000E+00
Gm16014	11619		#N/A	1.000000E+00
Gm16015	11620		#N/A	1.000000E+00
Gm16016	11621		#N/A	1.000000E+00
Gm16017	11622	0.157799267	16153.5	2.563935E-02
Gm16019	11623		#N/A	1.000000E+00
Gm16020	11624	0.072928367	10059	3.047665E-01
Gm16021	11625		#N/A	1.000000E+00
Gm16022	11626	-0.038406171	3065	5.892384E-01
Gm16023	11627	0.088954394	11267	2.103497E-01
Gm16024	11628		#N/A	1.000000E+00
Gm16025	11629		#N/A	1.000000E+00
Gm16026	11630	-0.000458481	5247	9.948591E-01
Gm16027	11631		#N/A	1.000000E+00
Gm16028	11632	-0.018447724	4312.5	7.954215E-01
Gm16029	11633		#N/A	1.000000E+00
Gm16030	11634		#N/A	1.000000E+00
Gm16031	11635		#N/A	1.000000E+00
Gm16032	11636		#N/A	1.000000E+00
Gm16033	11637	0.038758411	7558.5	5.858252E-01
Gm16034	11638	0.143605682	15224	4.249002E-02
Gm16035	11639		#N/A	1.000000E+00
Gm16036	11640	-0.054451513	1703	4.437940E-01
Gm16038	11641		#N/A	1.000000E+00
Gm16039	11642	0.221580021	18949	1.614561E-03
Gm16040	11643	0.143605682	15224	4.249002E-02
Gm16041	11644		#N/A	1.000000E+00
Gm16042	11645		#N/A	1.000000E+00
Gm16043	11646	0.156099909	16043	2.729327E-02
Gm16044	11647		#N/A	1.000000E+00
Gm16045	11648	0.053267784	8685	4.537786E-01
Gm16046	11649		#N/A	1.000000E+00
Gm16047	11650	0.043998631	7866	5.361574E-01
Gm16048	11651	-0.066855996	1239.5	3.469032E-01
Gm16049	11652		#N/A	1.000000E+00

Spearman Rank correlation analysis performed between Prdm1 and all-expressed genes within the Meredith RNA-seq dataset. Robust Prdm1–associated genes were identified using a cut-off of $p < 0.0005$.

Table S1, Related to Supplemental Figure 3C. Prdm1 associated genes

Gm1604A	11653	0.134885536	14611.5	5.686790E-02
Gm1604b	11654	-0.038406171	3065	5.892384E-01
Gm16050	11655		#N/A	1.000000E+00
Gm16052	11656		#N/A	1.000000E+00
Gm16053	11657		#N/A	1.000000E+00
Gm16054	11658	0.187620974	17748.5	7.804850E-03
Gm16055	11659		#N/A	1.000000E+00
Gm16056	11660		#N/A	1.000000E+00
Gm16057	11661		#N/A	1.000000E+00
Gm16058	11662		#N/A	1.000000E+00
Gm16059	11663		#N/A	1.000000E+00
Gm16060	11664	0.157799267	16153.5	2.563935E-02
Gm16061	11665	0.042807012	7792.5	5.472647E-01
Gm16062	11666	0.097250266	11856	1.707009E-01
Gm16063	11667	0.185980145	17680	8.371323E-03
Gm16064	11668		#N/A	1.000000E+00
Gm16065	11669	-0.066858259	1029.5	3.468868E-01
Gm16066	11670	0.175054299	17165	1.316410E-02
Gm16067	11671		#N/A	1.000000E+00
Gm16068	11672		#N/A	1.000000E+00
Gm16069	11673	-0.038406171	3065	5.892384E-01
Gm16070	11674	-0.038406171	3065	5.892384E-01
Gm16071	11675	0.021317126	6513.5	7.644715E-01
Gm16072	11676	0.114414374	13283	1.066960E-01
Gm16073	11677	-0.038406171	3065	5.892384E-01
Gm16074	11678		#N/A	1.000000E+00
Gm16075	11679	-0.038406171	3065	5.892384E-01
Gm16076	11680		#N/A	1.000000E+00
Gm16077	11681		#N/A	1.000000E+00
Gm16078	11682		#N/A	1.000000E+00
Gm16079	11683	-0.054450825	1984	4.437997E-01
Gm16080	11684		#N/A	1.000000E+00
Gm16081	11685		#N/A	1.000000E+00
Gm16082	11686		#N/A	1.000000E+00
Gm16083	11687	0.009920243	5768	8.891203E-01
Gm16084	11688	-0.038406171	3065	5.892384E-01
Gm16085	11689		#N/A	1.000000E+00
Gm16086	11690	-0.066855996	1239.5	3.469032E-01
Gm16087	11691		#N/A	1.000000E+00
Gm16088	11692		#N/A	1.000000E+00
Gm16089	11693	-0.054451513	1703	4.437940E-01
Gm16090	11694	0.071559655	9953	3.139566E-01
Gm16091	11695	0.067793754	9689	3.401662E-01

Spearman Rank correlation analysis performed between Prdm1 and all-expressed genes within the Meredith RNA-seq dataset. Robust Prdm1–associated genes were identified using a cut-off of $p < 0.0005$.

Table S1, Related to Supplemental Figure 3C. Prdm1 associated genes

Gm16092	11696	-0.038406171	3065	5.892384E-01
Gm16093	11697	0.029239252	7031	6.810714E-01
Gm16094	11698		#N/A	1.000000E+00
Gm16095	11699	-0.077394997	736.5	2.760195E-01
Gm16096	11700	0.011244345	5840	8.744353E-01
Gm16098	11701		#N/A	1.000000E+00
Gm16099	11702	0.021316405	6469	7.644793E-01
Gm16100	11703		#N/A	1.000000E+00
Gm16101	11704		#N/A	1.000000E+00
Gm16102	11705		#N/A	1.000000E+00
Gm16103	11706	-0.038406171	3065	5.892384E-01
Gm16104	11707		#N/A	1.000000E+00
Gm16105	11708	-0.069731363	951	3.265118E-01
Gm16106	11709	-0.038406171	3065	5.892384E-01
Gm16107	11710		#N/A	1.000000E+00
Gm16108	11711	-0.032762514	3799	6.451205E-01
Gm16110	11712		#N/A	1.000000E+00
Gm16111	11713	-0.054451513	1703	4.437940E-01
Gm16112	11714		#N/A	1.000000E+00
Gm16113	11715		#N/A	1.000000E+00
Gm16114	11716	0.113548679	13094	1.093908E-01
Gm16116	11717	-0.038406171	3065	5.892384E-01
Gm16117	11718		#N/A	1.000000E+00
Gm16118	11719		#N/A	1.000000E+00
Gm16119	11720	0.115257078	13340	1.041231E-01
Gm16120	11721		#N/A	1.000000E+00
Gm16121	11722		#N/A	1.000000E+00
Gm16122	11723		#N/A	1.000000E+00
Gm16123	11724	0.203598738	18373	3.832578E-03
Gm16124	11725	0.154019406	15889	2.944177E-02
Gm16125	11726		#N/A	1.000000E+00
Gm16126	11727	0.160304018	16323	2.335876E-02
Gm16127	11728		#N/A	1.000000E+00
Gm16128	11729		#N/A	1.000000E+00
Gm16129	11730	0.018225701	6233	7.978304E-01
Gm16130	11731	-0.038406171	3065	5.892384E-01
Gm16131	11732		#N/A	1.000000E+00
Gm16132	11733		#N/A	1.000000E+00
Gm16133	11734		#N/A	1.000000E+00
Gm16134	11735	0.00323985	5491	9.636838E-01
Gm16135	11736		#N/A	1.000000E+00
Gm16136	11737	0.021316405	6469	7.644793E-01
Gm16137	11738		#N/A	1.000000E+00

Spearman Rank correlation analysis performed between Prdm1 and all-expressed genes within the Meredith RNA-seq dataset. Robust Prdm1–associated genes were identified using a cut-off of $p < 0.0005$.

Table S1, Related to Supplemental Figure 3C. Prdm1 associated genes

Gm16138	11739		#N/A	1.000000E+00
Gm16139	11740		#N/A	1.000000E+00
Gm16140	11741		#N/A	1.000000E+00
Gm16141	11742	0.021316405	6469	7.644793E-01
Gm16142	11743		#N/A	1.000000E+00
Gm16143	11744		#N/A	1.000000E+00
Gm16144	11745		#N/A	1.000000E+00
Gm16145	11746	-0.003578438	5003	9.598915E-01
Gm16146	11747	-0.038406171	3065	5.892384E-01
Gm16147	11748		#N/A	1.000000E+00
Gm16148	11749	0.086412184	11087.5	2.237298E-01
Gm16149	11750		#N/A	1.000000E+00
Gm16150	11751	0.126933796	14110.5	7.327484E-02
Gm16151	11752		#N/A	1.000000E+00
Gm16152	11753	0.021317126	6513.5	7.644715E-01
Gm16153	11754	-0.038406171	3065	5.892384E-01
Gm16154	11755	0.268660779	19710	1.197767E-04
Gm16155	11756		#N/A	1.000000E+00
Gm16156	11757	-0.022047601	4170	7.566496E-01
Gm16157	11758	0.025486343	6848	7.201710E-01
Gm16158	11759	-0.038406171	3065	5.892384E-01
Gm16159	11760		#N/A	1.000000E+00
Gm16160	11761	-0.054450825	1984	4.437997E-01
Gm16161	11762		#N/A	1.000000E+00
Gm16162	11763	0.156129434	16056.5	2.726377E-02
Gm16163	11764	0.07494696	10242	2.915394E-01
Gm16164	11765		#N/A	1.000000E+00
Gm16165	11766		#N/A	1.000000E+00
Gm16166	11767	-0.038406171	3065	5.892384E-01
Gm16167	11768		#N/A	1.000000E+00
Gm16168	11769	0.056768718	9026	4.246135E-01
Gm16169	11770	-0.038406171	3065	5.892384E-01
Gm16170	11771	-0.038406171	3065	5.892384E-01
Gm16171	11772		#N/A	1.000000E+00
Gm16172	11773		#N/A	1.000000E+00
Gm16174	11774	0.084141322	10930.5	2.361831E-01
Gm16175	11775	-0.033388476	3771	6.388155E-01
Gm16177	11776		#N/A	1.000000E+00
Gm16178	11777	-0.066858259	1029.5	3.468868E-01
Gm16179	11778		#N/A	1.000000E+00
Gm16180	11779		#N/A	1.000000E+00
Gm16181	11780		#N/A	1.000000E+00
Gm16183	11781	-0.033702148	3752	6.356657E-01

Spearman Rank correlation analysis performed between Prdm1 and all-expressed genes within the Meredith RNA-seq dataset. Robust Prdm1–associated genes were identified using a cut-off of $p < 0.0005$.

Table S1, Related to Supplemental Figure 3C. Prdm1 associated genes

Gm16184	11782	-0.038406171	3065	5.892384E-01
Gm16185	11783		#N/A	1.000000E+00
Gm16186	11784		#N/A	1.000000E+00
Gm16187	11785	0.156648086	16086	2.675004E-02
Gm16188	11786	0.113548679	13094	1.093908E-01
Gm16189	11787		#N/A	1.000000E+00
Gm16190	11788		#N/A	1.000000E+00
Gm16191	11789	0.113548679	13094	1.093908E-01
Gm16192	11790		#N/A	1.000000E+00
Gm16193	11791	0.074574898	10201.5	2.939481E-01
Gm16194	11792	-0.066856562	1139	3.468991E-01
Gm16195	11793	-0.038406171	3065	5.892384E-01
Gm16196	11794	-0.066855996	1239.5	3.469032E-01
Gm16198	11795	-0.038406171	3065	5.892384E-01
Gm16199	11796		#N/A	1.000000E+00
Gm16200	11797		#N/A	1.000000E+00
Gm16201	11798	0.177606879	17292	1.186842E-02
Gm16202	11799		#N/A	1.000000E+00
Gm16203	11800		#N/A	1.000000E+00
Gm16204	11801		#N/A	1.000000E+00
Gm16205	11802		#N/A	1.000000E+00
Gm16206	11803	-0.038406171	3065	5.892384E-01
Gm16207	11804		#N/A	1.000000E+00
Gm16208	11805		#N/A	1.000000E+00
Gm16209	11806		#N/A	1.000000E+00
Gm16210	11807	-0.054450825	1984	4.437997E-01
Gm16211	11808		#N/A	1.000000E+00
Gm16212	11809		#N/A	1.000000E+00
Gm16213	11810		#N/A	1.000000E+00
Gm16214	11811		#N/A	1.000000E+00
Gm16215	11812		#N/A	1.000000E+00
Gm16216	11813	0.048448014	8175.5	4.957045E-01
Gm16217	11814		#N/A	1.000000E+00
Gm16218	11815	-0.038406171	3065	5.892384E-01
Gm16219	11816		#N/A	1.000000E+00
Gm16220	11817		#N/A	1.000000E+00
Gm16221	11818	0.109492511	12666.5	1.227347E-01
Gm16222	11819		#N/A	1.000000E+00
Gm16223	11820	0.062962713	9389.5	3.757656E-01
Gm16224	11821		#N/A	1.000000E+00
Gm16225	11822		#N/A	1.000000E+00
Gm16226	11823	0.113548679	13094	1.093908E-01
Gm16227	11824		#N/A	1.000000E+00

Spearman Rank correlation analysis performed between Prdm1 and all-expressed genes within the Meredith RNA-seq dataset. Robust Prdm1–associated genes were identified using a cut-off of $p < 0.0005$.

Table S1, Related to Supplemental Figure 3C. Prdm1 associated genes

Gm16228	11825		#N/A	1.000000E+00
Gm16229	11826		#N/A	1.000000E+00
Gm16230	11827	-0.018105422	4347	7.991361E-01
Gm16231	11828		#N/A	1.000000E+00
Gm16232	11829		#N/A	1.000000E+00
Gm16233	11830	-0.018447724	4312.5	7.954215E-01
Gm16234	11831		#N/A	1.000000E+00
Gm16235	11832	-0.002881632	5031	9.676968E-01
Gm16236	11833		#N/A	1.000000E+00
Gm16237	11834		#N/A	1.000000E+00
Gm16238	11835		#N/A	1.000000E+00
Gm16239	11836		#N/A	1.000000E+00
Gm16240	11837		#N/A	1.000000E+00
Gm16241	11838		#N/A	1.000000E+00
Gm16242	11839	-0.086748028	498.5	2.219283E-01
Gm16243	11840		#N/A	1.000000E+00
Gm16244	11841	-0.038406171	3065	5.892384E-01
Gm16245	11842		#N/A	1.000000E+00
Gm16246	11843	0.232443759	19217	9.258835E-04
Gm16248	11844		#N/A	1.000000E+00
Gm16249	11845	0.113548679	13094	1.093908E-01
Gm16250	11846		#N/A	1.000000E+00
Gm16251	11847		#N/A	1.000000E+00
Gm16252	11848	-0.077397959	691.5	2.760011E-01
Gm16253	11849	0.117975498	13525	9.615579E-02
Gm16254	11850		#N/A	1.000000E+00
Gm16256	11851		#N/A	1.000000E+00
Gm16257	11852		#N/A	1.000000E+00
Gm16259	11853	0.024994051	6819	7.253569E-01
Gm16260	11854		#N/A	1.000000E+00
Gm16261	11855	-0.077397959	691.5	2.760011E-01
Gm16262	11856		#N/A	1.000000E+00
Gm16263	11857	-0.038406171	3065	5.892384E-01
Gm16265	11858	0.053267784	8685	4.537786E-01
Gm16266	11859	0.097616157	11877	1.690898E-01
Gm16267	11860	0.229051473	19115	1.104513E-03
Gm16268	11861	0.043998631	7866	5.361574E-01
Gm16269	11862		#N/A	1.000000E+00
Gm16270	11863	-0.038406171	3065	5.892384E-01
Gm16271	11864	-0.023330139	4142	7.429763E-01
Gm16272	11865	0.048448014	8175.5	4.957045E-01
Gm16273	11866		#N/A	1.000000E+00
Gm16274	11867		#N/A	1.000000E+00

Spearman Rank correlation analysis performed between Prdm1 and all-expressed genes within the Meredith RNA-seq dataset. Robust Prdm1–associated genes were identified using a cut-off of $p < 0.0005$.

Table S1, Related to Supplemental Figure 3C. Prdm1 associated genes

Gm16275	11868	-0.054451513	1703	4.437940E-01
Gm16276	11869		#N/A	1.000000E+00
Gm16277	11870	0.196079198	18087	5.391505E-03
Gm16278	11871	0.064123369	9476.5	3.670112E-01
Gm16279	11872	0.072102623	10003	3.102894E-01
Gm16280	11873		#N/A	1.000000E+00
Gm16282	11874		#N/A	1.000000E+00
Gm16283	11875		#N/A	1.000000E+00
Gm16284	11876		#N/A	1.000000E+00
Gm16285	11877		#N/A	1.000000E+00
Gm16286	11878	0.124498714	13937	7.900616E-02
Gm16287	11879	-0.031039672	3885	6.626039E-01
Gm16288	11880	-0.054451513	1703	4.437940E-01
Gm16289	11881		#N/A	1.000000E+00
Gm16290	11882		#N/A	1.000000E+00
Gm16291	11883	-0.054451513	1703	4.437940E-01
Gm16292	11884		#N/A	1.000000E+00
Gm16294	11885		#N/A	1.000000E+00
Gm16295	11886		#N/A	1.000000E+00
Gm16296	11887		#N/A	1.000000E+00
Gm16297	11888		#N/A	1.000000E+00
Gm16298	11889	0.070535706	9874.5	3.209489E-01
Gm16299	11890	0.113548679	13094	1.093908E-01
Gm16300	11891		#N/A	1.000000E+00
Gm16301	11892		#N/A	1.000000E+00
Gm16302	11893	0.052728522	8550.5	4.583686E-01
Gm16303	11894		#N/A	1.000000E+00
Gm16304	11895		#N/A	1.000000E+00
Gm16305	11896	0.143605682	15224	4.249002E-02
Gm16306	11897		#N/A	1.000000E+00
Gm16307	11898		#N/A	1.000000E+00
Gm16308	11899		#N/A	1.000000E+00
Gm16309	11900		#N/A	1.000000E+00
Gm1631	11901	-0.038406171	3065	5.892384E-01
Gm16310	11902		#N/A	1.000000E+00
Gm16311	11903		#N/A	1.000000E+00
Gm16312	11904		#N/A	1.000000E+00
Gm16314	11905		#N/A	1.000000E+00
Gm16315	11906		#N/A	1.000000E+00
Gm16316	11907		#N/A	1.000000E+00
Gm16317	11908		#N/A	1.000000E+00
Gm16318	11909		#N/A	1.000000E+00
Gm16320	11910	-0.038406171	3065	5.892384E-01

Spearman Rank correlation analysis performed between Prdm1 and all-expressed genes within the Meredith RNA-seq dataset. Robust Prdm1–associated genes were identified using a cut-off of $p < 0.0005$.

Table S1, Related to Supplemental Figure 3C. Prdm1 associated genes

Gm16321	11911		#N/A	1.000000E+00
Gm16322	11912		#N/A	1.000000E+00
Gm16323	11913		#N/A	1.000000E+00
Gm16325	11914		#N/A	1.000000E+00
Gm16326	11915		#N/A	1.000000E+00
Gm16327	11916		#N/A	1.000000E+00
Gm16328	11917		#N/A	1.000000E+00
Gm16329	11918		#N/A	1.000000E+00
Gm16330	11919		#N/A	1.000000E+00
Gm16331	11920		#N/A	1.000000E+00
Gm16332	11921	0.218404973	18852	1.890294E-03
Gm16333	11922		#N/A	1.000000E+00
Gm16334	11923		#N/A	1.000000E+00
Gm16335	11924	0.089829065	11332	2.058817E-01
Gm16336	11925		#N/A	1.000000E+00
Gm16337	11926	0.087473475	11162	2.180723E-01
Gm16338	11927		#N/A	1.000000E+00
Gm16339	11928	0.09614611	11783	1.756317E-01
Gm16340	11929	-0.077397959	691.5	2.760011E-01
Gm16341	11930	0.078345378	10490	2.701479E-01
Gm16342	11931	0.141448608	15045.5	4.572713E-02
Gm16343	11932	0.145994164	15410	3.913227E-02
Gm16344	11933		#N/A	1.000000E+00
Gm16345	11934		#N/A	1.000000E+00
Gm16346	11935		#N/A	1.000000E+00
Gm16347	11936		#N/A	1.000000E+00
Gm16348	11937		#N/A	1.000000E+00
Gm16349	11938		#N/A	1.000000E+00
Gm16350	11939		#N/A	1.000000E+00
Gm16351	11940	0.113548679	13094	1.093908E-01
Gm16352	11941		#N/A	1.000000E+00
Gm16353	11942		#N/A	1.000000E+00
Gm16354	11943		#N/A	1.000000E+00
Gm16355	11944		#N/A	1.000000E+00
Gm16356	11945		#N/A	1.000000E+00
Gm16357	11946		#N/A	1.000000E+00
Gm16358	11947		#N/A	1.000000E+00
Gm16359	11948		#N/A	1.000000E+00
Gm16362	11949		#N/A	1.000000E+00
Gm16363	11950		#N/A	1.000000E+00
Gm16364	11951	-0.038406171	3065	5.892384E-01
Gm16365	11952	0.164573633	16634	1.987308E-02
Gm16367	11953		#N/A	1.000000E+00

Spearman Rank correlation analysis performed between Prdm1 and all-expressed genes within the Meredith RNA-seq dataset. Robust Prdm1–associated genes were identified using a cut-off of $p < 0.0005$.

Table S1, Related to Supplemental Figure 3C. Prdm1 associated genes

Gm16368	11954		#N/A	1.000000E+00
Gm16372	11955		#N/A	1.000000E+00
Gm16373	11956		#N/A	1.000000E+00
Gm16378	11957		#N/A	1.000000E+00
Gm16379	11958	0.143605682	15224	4.249002E-02
Gm16380	11959		#N/A	1.000000E+00
Gm16381	11960		#N/A	1.000000E+00
Gm16383	11961		#N/A	1.000000E+00
Gm16388	11962		#N/A	1.000000E+00
Gm16390	11963		#N/A	1.000000E+00
Gm16391	11964		#N/A	1.000000E+00
Gm16394	11965		#N/A	1.000000E+00
Gm16397	11966		#N/A	1.000000E+00
Gm16400	11967		#N/A	1.000000E+00
Gm16401	11968		#N/A	1.000000E+00
Gm16404	11969	0.143605682	15224	4.249002E-02
Gm16405	11970		#N/A	1.000000E+00
Gm16407	11971		#N/A	1.000000E+00
Gm16409	11972		#N/A	1.000000E+00
Gm16410	11973		#N/A	1.000000E+00
Gm16411	11974		#N/A	1.000000E+00
Gm16412	11975		#N/A	1.000000E+00
Gm16415	11976		#N/A	1.000000E+00
Gm16416	11977		#N/A	1.000000E+00
Gm16418	11978		#N/A	1.000000E+00
Gm16420	11979	-0.054450825	1984	4.437997E-01
Gm16421	11980		#N/A	1.000000E+00
Gm16423	11981		#N/A	1.000000E+00
Gm16425	11982	-0.038406171	3065	5.892384E-01
Gm16427	11983		#N/A	1.000000E+00
Gm16428	11984	-0.038406171	3065	5.892384E-01
Gm16429	11985		#N/A	1.000000E+00
Gm16430	11986		#N/A	1.000000E+00
Gm16431	11987		#N/A	1.000000E+00
Gm16432	11988	-0.031924044	3848.5	6.536057E-01
Gm16433	11989	0.082269134	10768	2.468098E-01
Gm16434	11990		#N/A	1.000000E+00
Gm16436	11991	-0.054451513	1703	4.437940E-01
Gm16437	11992		#N/A	1.000000E+00
Gm16439	11993		#N/A	1.000000E+00
Gm16440	11994		#N/A	1.000000E+00
Gm16441	11995		#N/A	1.000000E+00
Gm16442	11996		#N/A	1.000000E+00

Spearman Rank correlation analysis performed between Prdm1 and all-expressed genes within the Meredith RNA-seq dataset. Robust Prdm1–associated genes were identified using a cut-off of $p < 0.0005$.

Table S1, Related to Supplemental Figure 3C. Prdm1 associated genes

Gm16445	11997	-0.038406171	3065	5.892384E-01
Gm16451	11998		#N/A	1.000000E+00
Gm16456	11999		#N/A	1.000000E+00
Gm16458	12000	-0.066856562	1139	3.468991E-01
Gm16459	12001		#N/A	1.000000E+00
Gm16462	12002	0.074574898	10201.5	2.939481E-01
Gm16463	12003	0.040626191	7671	5.678805E-01
Gm16464	12004		#N/A	1.000000E+00
Gm16465	12005	-0.054451513	1703	4.437940E-01
Gm1647	12006	-0.066856562	1139	3.468991E-01
Gm16471	12007		#N/A	1.000000E+00
Gm16477	12008		#N/A	1.000000E+00
Gm16479	12009		#N/A	1.000000E+00
Gm16480	12010		#N/A	1.000000E+00
Gm16481	12011		#N/A	1.000000E+00
Gm16485	12012		#N/A	1.000000E+00
Gm16487	12013	-0.002881499	5034.5	9.676983E-01
Gm16490	12014	-0.032450477	3820	6.482730E-01
Gm16493	12015	0.151117939	15717	3.267803E-02
Gm16494	12016		#N/A	1.000000E+00
Gm16499	12017		#N/A	1.000000E+00
Gm16501	12018		#N/A	1.000000E+00
Gm16503	12019		#N/A	1.000000E+00
Gm16505	12020		#N/A	1.000000E+00
Gm16506	12021		#N/A	1.000000E+00
Gm16510	12022		#N/A	1.000000E+00
Gm16511	12023		#N/A	1.000000E+00
Gm16513	12024		#N/A	1.000000E+00
Gm16515	12025	0.109860796	12705	1.214733E-01
Gm16516	12026	-0.038406171	3065	5.892384E-01
Gm16518	12027		#N/A	1.000000E+00
Gm16519	12028	0.038229357	7499.5	5.909551E-01
GM16522	12029		#N/A	1.000000E+00
Gm16522	12030		#N/A	1.000000E+00
Gm16523	12031	0.087473475	11162	2.180723E-01
Gm16533	12032		#N/A	1.000000E+00
Gm16534	12033		#N/A	1.000000E+00
Gm16535	12034	-0.038406171	3065	5.892384E-01
Gm16536	12035	0.112909428	12916	1.114147E-01
Gm16537	12036		#N/A	1.000000E+00
Gm16538	12037	-0.038406171	3065	5.892384E-01
Gm16539	12038	0.036975773	7427	6.031915E-01
Gm16540	12039		#N/A	1.000000E+00

Spearman Rank correlation analysis performed between Prdm1 and all-expressed genes within the Meredith RNA-seq dataset. Robust Prdm1–associated genes were identified using a cut-off of $p < 0.0005$.

Table S1, Related to Supplemental Figure 3C. Prdm1 associated genes

Gm16541	12040	0.068272528	9723	3.367590E-01
Gm16542	12041	0.109492511	12666.5	1.227347E-01
Gm16543	12042		#N/A	1.000000E+00
Gm16545	12043		#N/A	1.000000E+00
Gm16546	12044		#N/A	1.000000E+00
Gm16547	12045	0.189988431	17842.5	7.047572E-03
Gm16548	12046	0.113548679	13094	1.093908E-01
Gm16549	12047		#N/A	1.000000E+00
Gm16550	12048		#N/A	1.000000E+00
Gm16551	12049	-0.038406171	3065	5.892384E-01
Gm16552	12050	0.038758411	7558.5	5.858252E-01
Gm16553	12051	0.01287414	5926	8.564193E-01
Gm16554	12052		#N/A	1.000000E+00
Gm16555	12053	-0.038406171	3065	5.892384E-01
Gm16556	12054	0.215438595	18765	2.186012E-03
Gm16557	12055		#N/A	1.000000E+00
Gm16558	12056		#N/A	1.000000E+00
Gm16559	12057		#N/A	1.000000E+00
Gm16560	12058		#N/A	1.000000E+00
Gm16561	12059		#N/A	1.000000E+00
Gm16563	12060		#N/A	1.000000E+00
Gm16564	12061	0.113548679	13094	1.093908E-01
Gm16565	12062	-0.038406171	3065	5.892384E-01
Gm16566	12063		#N/A	1.000000E+00
Gm16567	12064	-0.054450825	1984	4.437997E-01
Gm16568	12065		#N/A	1.000000E+00
Gm16569	12066		#N/A	1.000000E+00
Gm16570	12067	0.113548679	13094	1.093908E-01
Gm16571	12068		#N/A	1.000000E+00
Gm16572	12069		#N/A	1.000000E+00
Gm16573	12070	0.085259237	11006	2.299930E-01
Gm16574	12071		#N/A	1.000000E+00
Gm16575	12072		#N/A	1.000000E+00
Gm16576	12073	-0.004303875	4975	9.517696E-01
Gm16577	12074	0.156129434	16056.5	2.726377E-02
Gm16578	12075		#N/A	1.000000E+00
Gm16579	12076	-0.054451513	1703	4.437940E-01
Gm16580	12077		#N/A	1.000000E+00
Gm16581	12078		#N/A	1.000000E+00
Gm16582	12079		#N/A	1.000000E+00
Gm16583	12080	-0.038406171	3065	5.892384E-01
Gm16584	12081		#N/A	1.000000E+00
Gm16585	12082	0.118679452	13567	9.417358E-02

Spearman Rank correlation analysis performed between Prdm1 and all-expressed genes within the Meredith RNA-seq dataset. Robust Prdm1–associated genes were identified using a cut-off of $p < 0.0005$.

Table S1, Related to Supplemental Figure 3C. Prdm1 associated genes

Gm16586	12083	0.230189597	19153	1.041332E-03
Gm16587	12084		#N/A	1.000000E+00
Gm16588	12085		#N/A	1.000000E+00
Gm16589	12086	0.1544596	15936.5	2.897553E-02
Gm16599	12087	-0.032762514	3799	6.451205E-01
Gm166	12088	0.049164186	8229	4.893477E-01
Gm16602	12089		#N/A	1.000000E+00
Gm1661	12090	-0.086748028	498.5	2.219283E-01
Gm16618	12091		#N/A	1.000000E+00
Gm16638	12092	0.08295169	10828	2.428977E-01
Gm1667	12093		#N/A	1.000000E+00
Gm16686	12094		#N/A	1.000000E+00
Gm16710	12095	-0.038406171	3065	5.892384E-01
Gm16722	12096	0.055304739	8946	4.366750E-01
Gm16725	12097	-0.054451513	1703	4.437940E-01
Gm16726	12098		#N/A	1.000000E+00
Gm1673	12099	-0.066856562	1139	3.468991E-01
Gm16731	12100	0.052728522	8550.5	4.583686E-01
Gm16741	12101	0.053267784	8685	4.537786E-01
Gm16751	12102		#N/A	1.000000E+00
Gm16758	12103		#N/A	1.000000E+00
Gm16759	12104	0.007811906	5671	9.125789E-01
Gm16764	12105		#N/A	1.000000E+00
Gm16796	12106	0.120685946	13702	8.870165E-02
Gm16832	12107	0.070897485	9906	3.184670E-01
Gm16833	12108		#N/A	1.000000E+00
Gm16835	12109	-0.077393517	797	2.760287E-01
Gm16838	12110	-0.038406171	3065	5.892384E-01
Gm16876	12111	0.321384841	19969	3.479429E-06
Gm16933	12112	0.012608035	5907	8.593560E-01
Gm16968	12113		#N/A	1.000000E+00
Gm16976	12114	-0.019913532	4243	7.795677E-01
Gm1698	12115	-0.077393517	797	2.760287E-01
Gm16984	12116	-0.0170741	4411	8.103549E-01
Gm17014	12117	-0.038406171	3065	5.892384E-01
Gm17017	12118		#N/A	1.000000E+00
Gm17018	12119	0.083452862	10867	2.400529E-01
Gm17019	12120		#N/A	1.000000E+00
Gm17020	12121		#N/A	1.000000E+00
Gm17021	12122	-0.075592571	874.5	2.873910E-01
Gm17022	12123		#N/A	1.000000E+00
Gm17023	12124	-0.066858259	1029.5	3.468868E-01
Gm17024	12125		#N/A	1.000000E+00

Spearman Rank correlation analysis performed between Prdm1 and all-expressed genes within the Meredith RNA-seq dataset. Robust Prdm1–associated genes were identified using a cut-off of $p < 0.0005$.

Table S1, Related to Supplemental Figure 3C. Prdm1 associated genes

Gm17025	12126		#N/A	1.000000E+00
Gm17026	12127		#N/A	1.000000E+00
Gm17027	12128		#N/A	1.000000E+00
Gm17028	12129		#N/A	1.000000E+00
Gm17029	12130	-0.066856562	1139	3.468991E-01
Gm17030	12131		#N/A	1.000000E+00
Gm17031	12132		#N/A	1.000000E+00
Gm17032	12133		#N/A	1.000000E+00
Gm17033	12134		#N/A	1.000000E+00
Gm17034	12135		#N/A	1.000000E+00
Gm17035	12136		#N/A	1.000000E+00
Gm17036	12137	-0.054451513	1703	4.437940E-01
Gm17037	12138		#N/A	1.000000E+00
Gm17038	12139		#N/A	1.000000E+00
Gm17039	12140		#N/A	1.000000E+00
Gm17040	12141	-0.077394997	736.5	2.760195E-01
Gm17041	12142	0.075715395	10317.5	2.866063E-01
Gm17042	12143		#N/A	1.000000E+00
Gm17043	12144		#N/A	1.000000E+00
Gm17044	12145	-0.038406171	3065	5.892384E-01
Gm17045	12146		#N/A	1.000000E+00
Gm17046	12147		#N/A	1.000000E+00
Gm17047	12148		#N/A	1.000000E+00
Gm17048	12149		#N/A	1.000000E+00
Gm17051	12150		#N/A	1.000000E+00
Gm17052	12151		#N/A	1.000000E+00
Gm17053	12152	-0.038406171	3065	5.892384E-01
Gm17054	12153		#N/A	1.000000E+00
Gm17055	12154		#N/A	1.000000E+00
Gm17056	12155	0.039813567	7624	5.756557E-01
Gm17057	12156	0.038229357	7499.5	5.909551E-01
Gm17058	12157	0.070564029	9878	3.207541E-01
Gm17059	12158	0.052728522	8550.5	4.583686E-01
Gm17060	12159	0.091513222	11488	1.974724E-01
Gm17061	12160		#N/A	1.000000E+00
Gm17062	12161		#N/A	1.000000E+00
Gm17063	12162		#N/A	1.000000E+00
Gm17064	12163		#N/A	1.000000E+00
Gm17065	12164		#N/A	1.000000E+00
Gm17066	12165		#N/A	1.000000E+00
Gm17067	12166		#N/A	1.000000E+00
Gm17068	12167	-0.054451513	1703	4.437940E-01
Gm17069	12168		#N/A	1.000000E+00

Spearman Rank correlation analysis performed between Prdm1 and all-expressed genes within the Meredith RNA-seq dataset. Robust Prdm1–associated genes were identified using a cut-off of $p < 0.0005$.

Table S1, Related to Supplemental Figure 3C. Prdm1 associated genes

Gm17070	12169		#N/A	1.000000E+00
Gm17071	12170	0.082350327	10778	2.463421E-01
Gm17072	12171		#N/A	1.000000E+00
Gm17073	12172		#N/A	1.000000E+00
Gm17074	12173		#N/A	1.000000E+00
Gm17075	12174		#N/A	1.000000E+00
Gm17076	12175		#N/A	1.000000E+00
Gm17077	12176	0.044404926	7892	5.323962E-01
Gm17078	12177		#N/A	1.000000E+00
Gm17079	12178		#N/A	1.000000E+00
Gm17080	12179		#N/A	1.000000E+00
Gm17081	12180		#N/A	1.000000E+00
Gm17082	12181		#N/A	1.000000E+00
Gm17083	12182		#N/A	1.000000E+00
Gm17084	12183		#N/A	1.000000E+00
Gm17085	12184		#N/A	1.000000E+00
Gm17086	12185		#N/A	1.000000E+00
Gm17088	12186		#N/A	1.000000E+00
Gm17089	12187	0.253088704	19539	2.993899E-04
Gm17090	12188	0.023853825	6738	7.374159E-01
Gm17091	12189		#N/A	1.000000E+00
Gm17092	12190		#N/A	1.000000E+00
Gm17093	12191		#N/A	1.000000E+00
Gm17094	12192	-0.016730797	4444.5	8.140981E-01
Gm17096	12193		#N/A	1.000000E+00
Gm17097	12194		#N/A	1.000000E+00
Gm17098	12195		#N/A	1.000000E+00
Gm17099	12196	0.180524314	17431	1.052614E-02
Gm17100	12197		#N/A	1.000000E+00
Gm17101	12198	0.07521908	10268	2.897860E-01
Gm17102	12199	-0.045537741	2310	5.219798E-01
Gm17103	12200	-0.066858259	1029.5	3.468868E-01
Gm17104	12201		#N/A	1.000000E+00
Gm17105	12202		#N/A	1.000000E+00
Gm17106	12203	0.127941752	14186	7.100343E-02
Gm17107	12204		#N/A	1.000000E+00
Gm17108	12205		#N/A	1.000000E+00
Gm17109	12206		#N/A	1.000000E+00
Gm17110	12207		#N/A	1.000000E+00
Gm17111	12208		#N/A	1.000000E+00
Gm17112	12209		#N/A	1.000000E+00
Gm17113	12210	-0.038406171	3065	5.892384E-01
Gm17114	12211	-0.066855996	1239.5	3.469032E-01

Spearman Rank correlation analysis performed between Prdm1 and all-expressed genes within the Meredith RNA-seq dataset. Robust Prdm1–associated genes were identified using a cut-off of $p < 0.0005$.

Table S1, Related to Supplemental Figure 3C. Prdm1 associated genes

Gm17115	12212		#N/A	1.000000E+00
Gm17116	12213		#N/A	1.000000E+00
Gm17117	12214		#N/A	1.000000E+00
Gm17118	12215		#N/A	1.000000E+00
Gm17119	12216		#N/A	1.000000E+00
Gm17120	12217	-0.077393517	797	2.760287E-01
Gm17121	12218		#N/A	1.000000E+00
Gm17122	12219	-0.054451513	1703	4.437940E-01
Gm17124	12220		#N/A	1.000000E+00
Gm17126	12221		#N/A	1.000000E+00
Gm17129	12222		#N/A	1.000000E+00
Gm17130	12223		#N/A	1.000000E+00
Gm17131	12224		#N/A	1.000000E+00
Gm17132	12225		#N/A	1.000000E+00
Gm17133	12226	-0.054451513	1703	4.437940E-01
Gm17134	12227		#N/A	1.000000E+00
Gm17135	12228	0.095334825	11725	1.793213E-01
Gm17136	12229		#N/A	1.000000E+00
Gm17137	12230		#N/A	1.000000E+00
Gm17138	12231		#N/A	1.000000E+00
Gm17139	12232	0.167348311	16788.5	1.785769E-02
Gm17140	12233	-0.038406171	3065	5.892384E-01
Gm17141	12234		#N/A	1.000000E+00
Gm17142	12235		#N/A	1.000000E+00
Gm17146	12236		#N/A	1.000000E+00
Gm17147	12237		#N/A	1.000000E+00
Gm17148	12238		#N/A	1.000000E+00
Gm17149	12239		#N/A	1.000000E+00
Gm17150	12240	0.215438595	18765	2.186012E-03
Gm17151	12241		#N/A	1.000000E+00
Gm17152	12242		#N/A	1.000000E+00
Gm17153	12243	-0.038406171	3065	5.892384E-01
Gm17154	12244		#N/A	1.000000E+00
Gm17155	12245		#N/A	1.000000E+00
Gm17157	12246		#N/A	1.000000E+00
Gm17158	12247		#N/A	1.000000E+00
Gm17159	12248		#N/A	1.000000E+00
Gm17160	12249		#N/A	1.000000E+00
Gm17161	12250		#N/A	1.000000E+00
Gm17162	12251		#N/A	1.000000E+00
Gm17163	12252		#N/A	1.000000E+00
Gm17164	12253		#N/A	1.000000E+00
Gm17165	12254		#N/A	1.000000E+00

Spearman Rank correlation analysis performed between Prdm1 and all-expressed genes within the Meredith RNA-seq dataset. Robust Prdm1–associated genes were identified using a cut-off of $p < 0.0005$.

Table S1, Related to Supplemental Figure 3C. Prdm1 associated genes

Gm17166	12255		#N/A	1.000000E+00
Gm17167	12256		#N/A	1.000000E+00
Gm17168	12257		#N/A	1.000000E+00
Gm17169	12258		#N/A	1.000000E+00
Gm17170	12259		#N/A	1.000000E+00
Gm17171	12260	0.113548679	13094	1.093908E-01
Gm17172	12261		#N/A	1.000000E+00
Gm17173	12262	-0.038406171	3065	5.892384E-01
Gm17174	12263		#N/A	1.000000E+00
Gm17175	12264		#N/A	1.000000E+00
Gm17176	12265	0.013458335	5965.5	8.499790E-01
Gm17177	12266		#N/A	1.000000E+00
Gm17178	12267	0.09716809	11850.5	1.710642E-01
Gm17179	12268	-0.032069348	3844	6.521320E-01
Gm17180	12269		#N/A	1.000000E+00
Gm17181	12270		#N/A	1.000000E+00
Gm17182	12271		#N/A	1.000000E+00
Gm17183	12272		#N/A	1.000000E+00
Gm17184	12273		#N/A	1.000000E+00
Gm17186	12274		#N/A	1.000000E+00
Gm17187	12275		#N/A	1.000000E+00
Gm17188	12276	0.113548679	13094	1.093908E-01
Gm17189	12277	0.113548679	13094	1.093908E-01
Gm17191	12278		#N/A	1.000000E+00
Gm17192	12279		#N/A	1.000000E+00
Gm17193	12280		#N/A	1.000000E+00
Gm17194	12281		#N/A	1.000000E+00
Gm17195	12282		#N/A	1.000000E+00
Gm17196	12283		#N/A	1.000000E+00
Gm17197	12284		#N/A	1.000000E+00
Gm17198	12285		#N/A	1.000000E+00
Gm17199	12286		#N/A	1.000000E+00
Gm1720	12287	0.064123369	9476.5	3.670112E-01
Gm17200	12288	0.210111816	18575	2.824572E-03
Gm17201	12289	0.031129504	7136	6.616876E-01
Gm17202	12290	0.057250942	9049	4.206833E-01
Gm17203	12291		#N/A	1.000000E+00
Gm17204	12292		#N/A	1.000000E+00
Gm17205	12293		#N/A	1.000000E+00
Gm17206	12294		#N/A	1.000000E+00
Gm17207	12295		#N/A	1.000000E+00
Gm17208	12296	-0.038406171	3065	5.892384E-01
Gm17209	12297		#N/A	1.000000E+00

Spearman Rank correlation analysis performed between Prdm1 and all-expressed genes within the Meredith RNA-seq dataset. Robust Prdm1–associated genes were identified using a cut-off of $p < 0.0005$.

Table S1, Related to Supplemental Figure 3C. Prdm1 associated genes

Gm17210	12298		#N/A	1.000000E+00
Gm17212	12299		#N/A	1.000000E+00
Gm17213	12300		#N/A	1.000000E+00
Gm17214	12301		#N/A	1.000000E+00
Gm17215	12302		#N/A	1.000000E+00
Gm17216	12303		#N/A	1.000000E+00
Gm17217	12304		#N/A	1.000000E+00
Gm17218	12305		#N/A	1.000000E+00
Gm17219	12306	0.157799267	16153.5	2.563935E-02
Gm17221	12307		#N/A	1.000000E+00
Gm17222	12308		#N/A	1.000000E+00
Gm17223	12309		#N/A	1.000000E+00
Gm17224	12310		#N/A	1.000000E+00
Gm17225	12311		#N/A	1.000000E+00
Gm17226	12312		#N/A	1.000000E+00
Gm17227	12313		#N/A	1.000000E+00
Gm17228	12314		#N/A	1.000000E+00
Gm17229	12315		#N/A	1.000000E+00
Gm17230	12316		#N/A	1.000000E+00
Gm17231	12317	0.089160376	11284	2.092913E-01
Gm17232	12318	-0.038406171	3065	5.892384E-01
Gm17233	12319	0.000307056	5327	9.965570E-01
Gm17234	12320	-0.038406171	3065	5.892384E-01
Gm17235	12321		#N/A	1.000000E+00
Gm17244	12322		#N/A	1.000000E+00
Gm17251	12323	-0.038406171	3065	5.892384E-01
Gm17252	12324		#N/A	1.000000E+00
Gm17257	12325		#N/A	1.000000E+00
Gm17258	12326		#N/A	1.000000E+00
Gm17267	12327		#N/A	1.000000E+00
Gm17268	12328	-0.103166823	231.5	1.460195E-01
Gm17271	12329		#N/A	1.000000E+00
Gm17296	12330	0.051571404	8436.5	4.683041E-01
Gm17300	12331	0.03756524	7461	5.974235E-01
Gm17304	12332		#N/A	1.000000E+00
Gm17305	12333	-0.038406171	3065	5.892384E-01
Gm17309	12334		#N/A	1.000000E+00
Gm17324	12335		#N/A	1.000000E+00
Gm17330	12336		#N/A	1.000000E+00
Gm17332	12337		#N/A	1.000000E+00
Gm17333	12338	-0.117572864	119.5	9.730435E-02
Gm17334	12339		#N/A	1.000000E+00
Gm17337	12340	0.048463736	8183	4.955645E-01

Spearman Rank correlation analysis performed between Prdm1 and all-expressed genes within the Meredith RNA-seq dataset. Robust Prdm1–associated genes were identified using a cut-off of $p < 0.0005$.

Table S1, Related to Supplemental Figure 3C. Prdm1 associated genes

Gm17344	12341	-0.092175708	400	1.942337E-01
Gm17349	12342		#N/A	1.000000E+00
Gm17354	12343	-0.054450825	1984	4.437997E-01
Gm17359	12344	0.211629232	18637	2.627328E-03
Gm17361	12345		#N/A	1.000000E+00
Gm17364	12346		#N/A	1.000000E+00
Gm17365	12347		#N/A	1.000000E+00
Gm17366	12348		#N/A	1.000000E+00
Gm17374	12349		#N/A	1.000000E+00
Gm17376	12350		#N/A	1.000000E+00
Gm17377	12351		#N/A	1.000000E+00
Gm17382	12352		#N/A	1.000000E+00
Gm17383	12353		#N/A	1.000000E+00
Gm17384	12354	-0.001224019	5151	9.862757E-01
Gm17387	12355	0.075720569	10324	2.865732E-01
Gm17391	12356		#N/A	1.000000E+00
Gm17396	12357	-0.044268912	2363.5	5.336539E-01
Gm17402	12358	-0.038406171	3065	5.892384E-01
Gm17404	12359		#N/A	1.000000E+00
Gm17409	12360		#N/A	1.000000E+00
Gm17412	12361		#N/A	1.000000E+00
Gm17414	12362	-0.002842332	5037	9.681372E-01
Gm17415	12363		#N/A	1.000000E+00
Gm17416	12364	0.042990603	7810	5.455461E-01
Gm17420	12365		#N/A	1.000000E+00
Gm17421	12366		#N/A	1.000000E+00
Gm17428	12367		#N/A	1.000000E+00
Gm17430	12368	-0.038406171	3065	5.892384E-01
Gm17449	12369		#N/A	1.000000E+00
Gm17450	12370	0.16135414	16443	2.245591E-02
Gm17455	12371		#N/A	1.000000E+00
Gm17467	12372		#N/A	1.000000E+00
Gm17469	12373		#N/A	1.000000E+00
Gm17472	12374		#N/A	1.000000E+00
Gm17477	12375	-0.054451513	1703	4.437940E-01
Gm17482	12376	0.066070605	9595	3.526099E-01
Gm17484	12377	0.071667294	9964.5	3.132274E-01
Gm17490	12378		#N/A	1.000000E+00
Gm17494	12379	0.138464979	14854	5.054229E-02
Gm17495	12380		#N/A	1.000000E+00
Gm17509	12381		#N/A	1.000000E+00
Gm17511	12382	0.019770131	6319	7.811148E-01
Gm17521	12383		#N/A	1.000000E+00

Spearman Rank correlation analysis performed between Prdm1 and all-expressed genes within the Meredith RNA-seq dataset. Robust Prdm1–associated genes were identified using a cut-off of $p < 0.0005$.

Table S1, Related to Supplemental Figure 3C. Prdm1 associated genes

Gm17522	12384		#N/A	1.000000E+00
Gm17528	12385		#N/A	1.000000E+00
Gm17530	12386	0.073928833	10125	2.981622E-01
Gm17535	12387	0.021317126	6513.5	7.644715E-01
Gm17538	12388		#N/A	1.000000E+00
Gm17541	12389	-0.01259869	4641	8.594592E-01
Gm17542	12390		#N/A	1.000000E+00
Gm17545	12391	-0.038406171	3065	5.892384E-01
Gm17546	12392	0.124323758	13929	7.943150E-02
Gm17555	12393		#N/A	1.000000E+00
Gm17566	12394	0.194643714	18032	5.746794E-03
Gm17567	12395		#N/A	1.000000E+00
Gm17571	12396	0.1544596	15936.5	2.897553E-02
Gm17572	12397		#N/A	1.000000E+00
Gm17576	12398		#N/A	1.000000E+00
Gm17577	12399		#N/A	1.000000E+00
Gm1758	12400	0.113548679	13094	1.093908E-01
Gm17581	12401		#N/A	1.000000E+00
Gm17584	12402		#N/A	1.000000E+00
Gm17590	12403		#N/A	1.000000E+00
Gm17604	12404		#N/A	1.000000E+00
Gm17606	12405	-0.076912946	844	2.790305E-01
Gm17611	12406		#N/A	1.000000E+00
Gm17613	12407		#N/A	1.000000E+00
Gm17615	12408		#N/A	1.000000E+00
Gm17617	12409		#N/A	1.000000E+00
Gm17618	12410	-0.038406171	3065	5.892384E-01
Gm17622	12411		#N/A	1.000000E+00
Gm17631	12412		#N/A	1.000000E+00
Gm17641	12413		#N/A	1.000000E+00
Gm17651	12414		#N/A	1.000000E+00
Gm17654	12415		#N/A	1.000000E+00
Gm17655	12416		#N/A	1.000000E+00
Gm17657	12417		#N/A	1.000000E+00
Gm17660	12418	-0.038406171	3065	5.892384E-01
Gm17661	12419		#N/A	1.000000E+00
Gm17662	12420	0.113548679	13094	1.093908E-01
Gm17669	12421		#N/A	1.000000E+00
Gm17673	12422	0.071667294	9964.5	3.132274E-01
Gm17677	12423		#N/A	1.000000E+00
Gm17678	12424		#N/A	1.000000E+00
Gm17686	12425		#N/A	1.000000E+00
Gm17688	12426		#N/A	1.000000E+00

Spearman Rank correlation analysis performed between Prdm1 and all-expressed genes within the Meredith RNA-seq dataset. Robust Prdm1–associated genes were identified using a cut-off of $p < 0.0005$.

Table S1, Related to Supplemental Figure 3C. Prdm1 associated genes

Gm17689	12427		#N/A	1.000000E+00
Gm17693	12428		#N/A	1.000000E+00
Gm17705	12429	-0.038406171	3065	5.892384E-01
Gm17709	12430		#N/A	1.000000E+00
Gm17711	12431	-0.038406171	3065	5.892384E-01
Gm17720	12432	0.002673432	5455	9.700296E-01
Gm17727	12433	-0.038406171	3065	5.892384E-01
Gm17728	12434		#N/A	1.000000E+00
Gm17732	12435	0.222852157	18980	1.514792E-03
Gm17782	12436		#N/A	1.000000E+00
Gm17907	12437		#N/A	1.000000E+00
Gm17919	12438		#N/A	1.000000E+00
Gm17973	12439		#N/A	1.000000E+00
Gm18006	12440		#N/A	1.000000E+00
Gm18025	12441		#N/A	1.000000E+00
Gm18128	12442	-0.038406171	3065	5.892384E-01
Gm1815	12443		#N/A	1.000000E+00
Gm18159	12444		#N/A	1.000000E+00
Gm1818	12445	-0.066858259	1029.5	3.468868E-01
Gm18191	12446		#N/A	1.000000E+00
Gm18199	12447		#N/A	1.000000E+00
Gm18200	12448		#N/A	1.000000E+00
Gm18201	12449		#N/A	1.000000E+00
Gm1821	12450	0.230111086	19152	1.045581E-03
Gm1826	12451		#N/A	1.000000E+00
Gm18284	12452		#N/A	1.000000E+00
Gm18294	12453	0.086412184	11087.5	2.237298E-01
Gm18343	12454		#N/A	1.000000E+00
Gm18354	12455		#N/A	1.000000E+00
Gm18355	12456		#N/A	1.000000E+00
Gm18358	12457		#N/A	1.000000E+00
Gm18359	12458	0.022198316	6629.5	7.550388E-01
Gm18360	12459		#N/A	1.000000E+00
Gm18367	12460		#N/A	1.000000E+00
Gm18396	12461		#N/A	1.000000E+00
Gm18397	12462		#N/A	1.000000E+00
Gm1840	12463	-0.054451513	1703	4.437940E-01
Gm18415	12464		#N/A	1.000000E+00
Gm18416	12465		#N/A	1.000000E+00
Gm18417	12466		#N/A	1.000000E+00
Gm1848	12467		#N/A	1.000000E+00
Gm18489	12468		#N/A	1.000000E+00
Gm18513	12469		#N/A	1.000000E+00

Spearman Rank correlation analysis performed between Prdm1 and all-expressed genes within the Meredith RNA-seq dataset. Robust Prdm1–associated genes were identified using a cut-off of $p < 0.0005$.

Table S1, Related to Supplemental Figure 3C. Prdm1 associated genes

Gm18520	12470		#N/A	1.000000E+00
Gm18522	12471		#N/A	1.000000E+00
Gm18560	12472		#N/A	1.000000E+00
Gm18588	12473		#N/A	1.000000E+00
Gm18594	12474		#N/A	1.000000E+00
Gm18595	12475		#N/A	1.000000E+00
Gm18604	12476		#N/A	1.000000E+00
Gm1862	12477	-0.038406171	3065	5.892384E-01
Gm1866	12478		#N/A	1.000000E+00
Gm18733	12479		#N/A	1.000000E+00
Gm18734	12480		#N/A	1.000000E+00
Gm18737	12481		#N/A	1.000000E+00
Gm18779	12482		#N/A	1.000000E+00
Gm18808	12483		#N/A	1.000000E+00
Gm18833	12484		#N/A	1.000000E+00
Gm18856	12485		#N/A	1.000000E+00
Gm18889	12486		#N/A	1.000000E+00
Gm18953	12487		#N/A	1.000000E+00
Gm18958	12488	-0.038406171	3065	5.892384E-01
Gm19031	12489		#N/A	1.000000E+00
Gm19203	12490		#N/A	1.000000E+00
Gm19246	12491	0.019770131	6319	7.811148E-01
Gm19301	12492	0.072798883	10047.5	3.056282E-01
Gm19345	12493		#N/A	1.000000E+00
Gm19357	12494	0.123963302	13909	8.031364E-02
Gm19402	12495		#N/A	1.000000E+00
Gm19459	12496		#N/A	1.000000E+00
Gm1947	12497		#N/A	1.000000E+00
Gm19553	12498		#N/A	1.000000E+00
Gm19560	12499		#N/A	1.000000E+00
Gm1965	12500	0.096596777	11815	1.736066E-01
Gm1966	12501		#N/A	1.000000E+00
Gm19668	12502		#N/A	1.000000E+00
Gm1968	12503	0.117641498	13503	9.710780E-02
Gm19684	12504	0.143605682	15224	4.249002E-02
Gm1976	12505	0.0243831	6782	7.318101E-01
Gm1979	12506		#N/A	1.000000E+00
Gm1980	12507		#N/A	1.000000E+00
Gm19807	12508		#N/A	1.000000E+00
Gm19815	12509	0.159222778	16238	2.432086E-02
Gm19840	12510		#N/A	1.000000E+00
Gm1989	12511		#N/A	1.000000E+00
Gm19898	12512		#N/A	1.000000E+00

Spearman Rank correlation analysis performed between Prdm1 and all-expressed genes within the Meredith RNA-seq dataset. Robust Prdm1–associated genes were identified using a cut-off of $p < 0.0005$.

Table S1, Related to Supplemental Figure 3C. Prdm1 associated genes

Gm1992	12513	0.014301797	6015.5	8.406982E-01
Gm1993	12514		#N/A	1.000000E+00
Gm19932	12515		#N/A	1.000000E+00
Gm19961	12516		#N/A	1.000000E+00
Gm19965	12517		#N/A	1.000000E+00
Gm19967	12518		#N/A	1.000000E+00
Gm2000	12519	0.181124782	17452	1.026712E-02
Gm2001	12520		#N/A	1.000000E+00
Gm2003	12521	0.053267784	8685	4.537786E-01
Gm20036	12522		#N/A	1.000000E+00
Gm2004	12523		#N/A	1.000000E+00
Gm2005	12524		#N/A	1.000000E+00
Gm20069	12525	0.194131481	17998	5.878558E-03
Gm2007	12526		#N/A	1.000000E+00
Gm20091	12527	0.106001553	12425	1.352007E-01
Gm2012	12528		#N/A	1.000000E+00
Gm2016	12529		#N/A	1.000000E+00
Gm2018	12530	-0.038406171	3065	5.892384E-01
Gm2020	12531		#N/A	1.000000E+00
Gm2022	12532		#N/A	1.000000E+00
Gm2026	12533		#N/A	1.000000E+00
Gm20287	12534		#N/A	1.000000E+00
Gm2030	12535		#N/A	1.000000E+00
Gm20319	12536	0.103127317	12248	1.461748E-01
Gm20345	12537		#N/A	1.000000E+00
Gm2035	12538		#N/A	1.000000E+00
Gm20379	12539		#N/A	1.000000E+00
Gm20382	12540		#N/A	1.000000E+00
Gm20386	12541		#N/A	1.000000E+00
Gm20387	12542	0.113548679	13094	1.093908E-01
Gm20388	12543	0.216830031	18816	2.042422E-03
Gm20389	12544		#N/A	1.000000E+00
Gm20390	12545	0.139471492	14922	4.887285E-02
Gm20391	12546		#N/A	1.000000E+00
Gm20392	12547		#N/A	1.000000E+00
Gm20394	12548		#N/A	1.000000E+00
Gm20395	12549		#N/A	1.000000E+00
Gm20396	12550	-0.038406171	3065	5.892384E-01
Gm20397	12551		#N/A	1.000000E+00
Gm20399	12552		#N/A	1.000000E+00
Gm20400	12553		#N/A	1.000000E+00
Gm20401	12554		#N/A	1.000000E+00
Gm20402	12555		#N/A	1.000000E+00

Spearman Rank correlation analysis performed between Prdm1 and all-expressed genes within the Meredith RNA-seq dataset. Robust Prdm1–associated genes were identified using a cut-off of $p < 0.0005$.

Table S1, Related to Supplemental Figure 3C. Prdm1 associated genes

Gm20403	12556		#N/A	1.000000E+00
Gm20404	12557	-0.066858259	1029.5	3.468868E-01
Gm20405	12558		#N/A	1.000000E+00
Gm20406	12559		#N/A	1.000000E+00
Gm20407	12560		#N/A	1.000000E+00
Gm20408	12561	-0.038406171	3065	5.892384E-01
Gm20409	12562		#N/A	1.000000E+00
Gm20410	12563	0.074574898	10201.5	2.939481E-01
Gm20411	12564		#N/A	1.000000E+00
Gm20412	12565		#N/A	1.000000E+00
Gm20413	12566	-0.054451513	1703	4.437940E-01
Gm20414	12567		#N/A	1.000000E+00
Gm20415	12568		#N/A	1.000000E+00
Gm20416	12569		#N/A	1.000000E+00
Gm20417	12570	-0.056705607	1465	4.251295E-01
Gm20419	12571	-0.038406171	3065	5.892384E-01
Gm2042	12572	0.160304018	16323	2.335876E-02
Gm20420	12573		#N/A	1.000000E+00
Gm20421	12574		#N/A	1.000000E+00
Gm20422	12575	-0.054451513	1703	4.437940E-01
Gm20424	12576	0.097788826	11892	1.683335E-01
Gm20425	12577		#N/A	1.000000E+00
Gm20426	12578		#N/A	1.000000E+00
Gm20427	12579	0.041901464	7736	5.557803E-01
Gm20428	12580		#N/A	1.000000E+00
Gm20429	12581	-0.066855996	1239.5	3.469032E-01
Gm20430	12582	-0.038406171	3065	5.892384E-01
Gm20431	12583	0.152272219	15786	3.135605E-02
Gm20432	12584		#N/A	1.000000E+00
Gm20433	12585		#N/A	1.000000E+00
Gm20434	12586		#N/A	1.000000E+00
Gm20435	12587		#N/A	1.000000E+00
Gm20438	12588		#N/A	1.000000E+00
Gm2044	12589	-0.014198961	4559	8.418286E-01
Gm20441	12590	0.255669587	19573	2.582181E-04
Gm20442	12591	0.051225809	8399.5	4.712944E-01
Gm20443	12592		#N/A	1.000000E+00
Gm20444	12593		#N/A	1.000000E+00
Gm20445	12594		#N/A	1.000000E+00
Gm20446	12595	-0.038406171	3065	5.892384E-01
Gm20447	12596		#N/A	1.000000E+00
Gm20448	12597		#N/A	1.000000E+00
Gm20449	12598	0.083380721	10858	2.404609E-01

Spearman Rank correlation analysis performed between Prdm1 and all-expressed genes within the Meredith RNA-seq dataset. Robust Prdm1–associated genes were identified using a cut-off of $p < 0.0005$.

Table S1, Related to Supplemental Figure 3C. Prdm1 associated genes

Gm20450	12599		#N/A	1.000000E+00
Gm20451	12600		#N/A	1.000000E+00
Gm20452	12601		#N/A	1.000000E+00
Gm20453	12602		#N/A	1.000000E+00
Gm20454	12603		#N/A	1.000000E+00
Gm20455	12604		#N/A	1.000000E+00
Gm20456	12605		#N/A	1.000000E+00
Gm20457	12606	0.028465569	6991	6.890668E-01
Gm20458	12607	0.025185127	6835	7.233425E-01
Gm20459	12608	0.03378913	7285	6.347934E-01
Gm2046	12609		#N/A	1.000000E+00
Gm20460	12610	0.020873487	6389	7.692337E-01
Gm20461	12611		#N/A	1.000000E+00
Gm20462	12612		#N/A	1.000000E+00
Gm20463	12613	0.090089482	11353.5	2.045648E-01
Gm20464	12614	-0.054450825	1984	4.437997E-01
Gm20465	12615	0.089213881	11287	2.090170E-01
Gm20467	12616	0.053267784	8685	4.537786E-01
Gm20468	12617	-0.054451513	1703	4.437940E-01
Gm20469	12618	-0.038406171	3065	5.892384E-01
Gm20470	12619	-0.038406171	3065	5.892384E-01
Gm20471	12620	0.092790287	11564	1.912641E-01
Gm20472	12621	0.053267784	8685	4.537786E-01
Gm20473	12622		#N/A	1.000000E+00
Gm20474	12623	-0.054451513	1703	4.437940E-01
Gm20475	12624		#N/A	1.000000E+00
Gm20476	12625		#N/A	1.000000E+00
Gm20477	12626		#N/A	1.000000E+00
Gm20478	12627		#N/A	1.000000E+00
Gm20479	12628	0.113548679	13094	1.093908E-01
Gm20480	12629	0.160304018	16323	2.335876E-02
Gm20481	12630	0.113548679	13094	1.093908E-01
Gm20482	12631		#N/A	1.000000E+00
Gm20483	12632		#N/A	1.000000E+00
Gm20484	12633	-0.038406171	3065	5.892384E-01
Gm20485	12634		#N/A	1.000000E+00
Gm20486	12635		#N/A	1.000000E+00
Gm20488	12636	0.027253223	6941	7.016648E-01
Gm20489	12637	-0.066858259	1029.5	3.468868E-01
Gm20490	12638		#N/A	1.000000E+00
Gm20491	12639		#N/A	1.000000E+00
Gm20492	12640	0.09739768	11864	1.700504E-01
Gm20493	12641		#N/A	1.000000E+00

Spearman Rank correlation analysis performed between Prdm1 and all-expressed genes within the Meredith RNA-seq dataset. Robust Prdm1–associated genes were identified using a cut-off of $p < 0.0005$.

Table S1, Related to Supplemental Figure 3C. Prdm1 associated genes

Gm20495	12642		#N/A	1.000000E+00
Gm20496	12643	0.113548679	13094	1.093908E-01
Gm20497	12644	0.017370925	6195	8.071219E-01
Gm20498	12645	0.334487167	19991	1.292672E-06
Gm20499	12646	0.118067525	13532	9.589479E-02
Gm20500	12647		#N/A	1.000000E+00
Gm20501	12648		#N/A	1.000000E+00
Gm20503	12649	0.109693041	12692	1.220466E-01
Gm20504	12650		#N/A	1.000000E+00
Gm20505	12651		#N/A	1.000000E+00
Gm20506	12652	0.08372371	10896	2.385252E-01
Gm20507	12653	-0.045687615	2307	5.206095E-01
Gm20508	12654		#N/A	1.000000E+00
Gm20509	12655	-0.014199085	4558	8.418272E-01
Gm20510	12656	0.106047563	12429	1.350303E-01
Gm20511	12657		#N/A	1.000000E+00
Gm20512	12658		#N/A	1.000000E+00
Gm20513	12659	-0.082569945	612	2.450803E-01
Gm20514	12660	0.063431092	9426.5	3.722175E-01
Gm20515	12661		#N/A	1.000000E+00
Gm20516	12662		#N/A	1.000000E+00
Gm20517	12663	-0.124244479	85	7.962485E-02
Gm20518	12664		#N/A	1.000000E+00
Gm20519	12665	0.143605682	15224	4.249002E-02
Gm20520	12666		#N/A	1.000000E+00
Gm20521	12667	-0.021416554	4195	7.634054E-01
Gm20522	12668	-0.054450825	1984	4.437997E-01
Gm20523	12669		#N/A	1.000000E+00
Gm20524	12670		#N/A	1.000000E+00
Gm20525	12671		#N/A	1.000000E+00
Gm20526	12672		#N/A	1.000000E+00
Gm20527	12673	-0.038406171	3065	5.892384E-01
Gm20528	12674	0.15811232	16180	2.534425E-02
Gm20529	12675	0.056482135	9006	4.269593E-01
Gm20530	12676		#N/A	1.000000E+00
Gm20531	12677	-0.038406171	3065	5.892384E-01
Gm20532	12678	0.141448608	15045.5	4.572713E-02
Gm20534	12679		#N/A	1.000000E+00
Gm20535	12680		#N/A	1.000000E+00
Gm20536	12681	0.113548679	13094	1.093908E-01
Gm20537	12682	-0.013528215	4592	8.492093E-01
Gm20538	12683	0.140525339	14989	4.717438E-02
Gm20539	12684		#N/A	1.000000E+00

Spearman Rank correlation analysis performed between Prdm1 and all-expressed genes within the Meredith RNA-seq dataset. Robust Prdm1–associated genes were identified using a cut-off of $p < 0.0005$.

Table S1, Related to Supplemental Figure 3C. Prdm1 associated genes

Gm20540	12685		#N/A	1.000000E+00
Gm20541	12686		#N/A	1.000000E+00
Gm20542	12687		#N/A	1.000000E+00
Gm20543	12688		#N/A	1.000000E+00
Gm20544	12689		#N/A	1.000000E+00
Gm20545	12690		#N/A	1.000000E+00
Gm20546	12691		#N/A	1.000000E+00
Gm20547	12692		#N/A	1.000000E+00
Gm20548	12693	-0.054450825	1984	4.437997E-01
Gm20549	12694		#N/A	1.000000E+00
Gm20554	12695	-0.01274284	4636	8.578681E-01
Gm2056	12696		#N/A	1.000000E+00
Gm20563	12697		#N/A	1.000000E+00
Gm20579	12698		#N/A	1.000000E+00
Gm2058	12699		#N/A	1.000000E+00
Gm20580	12700		#N/A	1.000000E+00
Gm20581	12701		#N/A	1.000000E+00
Gm20594	12702	0.183436459	17565	9.321760E-03
Gm20604	12703		#N/A	1.000000E+00
Gm20605	12704		#N/A	1.000000E+00
Gm20608	12705		#N/A	1.000000E+00
Gm20609	12706		#N/A	1.000000E+00
Gm20610	12707		#N/A	1.000000E+00
Gm20611	12708	0.18841568	17778	7.542946E-03
Gm20612	12709		#N/A	1.000000E+00
Gm20613	12710		#N/A	1.000000E+00
Gm20614	12711	-0.038406171	3065	5.892384E-01
Gm20615	12712		#N/A	1.000000E+00
Gm20616	12713	-0.038406171	3065	5.892384E-01
Gm20617	12714	-0.038406171	3065	5.892384E-01
Gm20618	12715	0.056284828	8999	4.285787E-01
Gm20619	12716	0.013458335	5965.5	8.499790E-01
Gm20620	12717		#N/A	1.000000E+00
Gm20621	12718	0.07521908	10268	2.897860E-01
Gm20622	12719		#N/A	1.000000E+00
Gm20623	12720		#N/A	1.000000E+00
Gm20624	12721		#N/A	1.000000E+00
Gm20625	12722		#N/A	1.000000E+00
Gm20627	12723		#N/A	1.000000E+00
Gm20628	12724		#N/A	1.000000E+00
Gm20629	12725	0.057322888	9053	4.200987E-01
Gm20630	12726		#N/A	1.000000E+00
Gm20631	12727	0.050519119	8336	4.774413E-01

Spearman Rank correlation analysis performed between Prdm1 and all-expressed genes within the Meredith RNA-seq dataset. Robust Prdm1–associated genes were identified using a cut-off of $p < 0.0005$.

Table S1, Related to Supplemental Figure 3C. Prdm1 associated genes

Gm20632	12728	0.127545848	14155	7.188866E-02
Gm20633	12729	-0.054451513	1703	4.437940E-01
Gm20634	12730		#N/A	1.000000E+00
Gm20635	12731	0.167348311	16788.5	1.785769E-02
Gm20636	12732		#N/A	1.000000E+00
Gm20637	12733		#N/A	1.000000E+00
Gm20638	12734		#N/A	1.000000E+00
Gm20641	12735	0.113548679	13094	1.093908E-01
Gm20642	12736	0.012162535	5884	8.642769E-01
Gm20643	12737		#N/A	1.000000E+00
Gm20644	12738	0.1544596	15936.5	2.897553E-02
Gm20645	12739	0.147404823	15500	3.725628E-02
Gm20646	12740	0.178689437	17336	1.135372E-02
Gm20647	12741	0.085706783	11033	2.275472E-01
Gm20648	12742		#N/A	1.000000E+00
Gm20649	12743	-0.038406171	3065	5.892384E-01
Gm20650	12744	-0.038406171	3065	5.892384E-01
Gm20651	12745		#N/A	1.000000E+00
Gm20652	12746	0.143605682	15224	4.249002E-02
Gm20654	12747		#N/A	1.000000E+00
Gm20655	12748	-0.03087642	3893.5	6.642703E-01
Gm20656	12749	0.087479177	11164	2.180422E-01
Gm20657	12750	0.157799267	16153.5	2.563935E-02
Gm20658	12751	-0.038406171	3065	5.892384E-01
Gm20659	12752		#N/A	1.000000E+00
Gm2066	12753		#N/A	1.000000E+00
Gm20660	12754		#N/A	1.000000E+00
Gm20661	12755	0.052605775	8512	4.594169E-01
Gm20662	12756	-0.027985754	3998	6.940428E-01
Gm20663	12757	0.021317126	6513.5	7.644715E-01
Gm20664	12758		#N/A	1.000000E+00
Gm20665	12759		#N/A	1.000000E+00
Gm20667	12760	-0.054450825	1984	4.437997E-01
Gm20670	12761		#N/A	1.000000E+00
Gm20671	12762	0.197398666	18137	5.082427E-03
Gm20672	12763		#N/A	1.000000E+00
Gm20673	12764	-0.066858259	1029.5	3.468868E-01
Gm20674	12765		#N/A	1.000000E+00
Gm20675	12766		#N/A	1.000000E+00
Gm20677	12767		#N/A	1.000000E+00
Gm20678	12768		#N/A	1.000000E+00
Gm20679	12769	-0.054450825	1984	4.437997E-01
Gm20680	12770	0.136623399	14736	5.371954E-02

Spearman Rank correlation analysis performed between Prdm1 and all-expressed genes within the Meredith RNA-seq dataset. Robust Prdm1–associated genes were identified using a cut-off of $p < 0.0005$.

Table S1, Related to Supplemental Figure 3C. Prdm1 associated genes

Gm20681	12771		#N/A	1.000000E+00
Gm20682	12772	-0.038406171	3065	5.892384E-01
Gm20683	12773	0.181859984	17478	9.957666E-03
Gm20684	12774		#N/A	1.000000E+00
Gm20685	12775	-0.054451513	1703	4.437940E-01
Gm20686	12776		#N/A	1.000000E+00
Gm20687	12777	0.243847163	19426	5.022037E-04
Gm20688	12778		#N/A	1.000000E+00
Gm20689	12779		#N/A	1.000000E+00
Gm20690	12780		#N/A	1.000000E+00
Gm20691	12781		#N/A	1.000000E+00
Gm20692	12782		#N/A	1.000000E+00
Gm20693	12783		#N/A	1.000000E+00
Gm20694	12784		#N/A	1.000000E+00
Gm20695	12785	0.061569982	9305	3.864379E-01
Gm20696	12786	0.101598695	12156	1.522803E-01
Gm20698	12787		#N/A	1.000000E+00
Gm20699	12788		#N/A	1.000000E+00
Gm20700	12789	0.074574898	10201.5	2.939481E-01
Gm20701	12790	0.053267784	8685	4.537786E-01
Gm20702	12791		#N/A	1.000000E+00
Gm20703	12792	-0.038406171	3065	5.892384E-01
Gm20704	12793		#N/A	1.000000E+00
Gm20705	12794		#N/A	1.000000E+00
Gm20706	12795		#N/A	1.000000E+00
Gm20707	12796	0.051099165	8392	4.723927E-01
Gm20708	12797	0.134749856	14606	5.711999E-02
Gm20709	12798		#N/A	1.000000E+00
Gm20710	12799		#N/A	1.000000E+00
Gm20711	12800		#N/A	1.000000E+00
Gm20712	12801	0.165581808	16682	1.911909E-02
Gm20713	12802	-0.016048766	4470	8.215470E-01
Gm20714	12803	0.038758411	7558.5	5.858252E-01
Gm20715	12804	-0.077394997	736.5	2.760195E-01
Gm20716	12805		#N/A	1.000000E+00
Gm20717	12806		#N/A	1.000000E+00
Gm20718	12807		#N/A	1.000000E+00
Gm20719	12808		#N/A	1.000000E+00
Gm20721	12809	0.137954373	14817	5.140717E-02
Gm20726	12810		#N/A	1.000000E+00
Gm20727	12811		#N/A	1.000000E+00
Gm20730	12812		#N/A	1.000000E+00
Gm20731	12813		#N/A	1.000000E+00

Spearman Rank correlation analysis performed between Prdm1 and all-expressed genes within the Meredith RNA-seq dataset. Robust Prdm1–associated genes were identified using a cut-off of $p < 0.0005$.

Table S1, Related to Supplemental Figure 3C. Prdm1 associated genes

Gm20732	12814	0.137979016	14821	5.136514E-02
Gm20736	12815	-0.038406171	3065	5.892384E-01
Gm20737	12816		#N/A	1.000000E+00
Gm20738	12817		#N/A	1.000000E+00
Gm20746	12818	-0.054451513	1703	4.437940E-01
Gm20747	12819		#N/A	1.000000E+00
Gm2075	12820		#N/A	1.000000E+00
Gm20760	12821		#N/A	1.000000E+00
Gm20765	12822		#N/A	1.000000E+00
Gm20767	12823		#N/A	1.000000E+00
Gm20773	12824		#N/A	1.000000E+00
Gm20775	12825	-0.077397959	691.5	2.760011E-01
Gm20777	12826		#N/A	1.000000E+00
Gm20781	12827		#N/A	1.000000E+00
Gm20782	12828		#N/A	1.000000E+00
Gm20783	12829	-0.038406171	3065	5.892384E-01
Gm20784	12830		#N/A	1.000000E+00
Gm20791	12831		#N/A	1.000000E+00
Gm20793	12832		#N/A	1.000000E+00
Gm20794	12833		#N/A	1.000000E+00
Gm20795	12834		#N/A	1.000000E+00
Gm20796	12835		#N/A	1.000000E+00
Gm20797	12836		#N/A	1.000000E+00
Gm20798	12837		#N/A	1.000000E+00
Gm20799	12838		#N/A	1.000000E+00
Gm20800	12839		#N/A	1.000000E+00
Gm20801	12840		#N/A	1.000000E+00
Gm20803	12841		#N/A	1.000000E+00
Gm20804	12842		#N/A	1.000000E+00
Gm20805	12843		#N/A	1.000000E+00
Gm20806	12844		#N/A	1.000000E+00
Gm20808	12845		#N/A	1.000000E+00
Gm20809	12846		#N/A	1.000000E+00
Gm20810	12847		#N/A	1.000000E+00
Gm20812	12848		#N/A	1.000000E+00
Gm20813	12849		#N/A	1.000000E+00
Gm20815	12850		#N/A	1.000000E+00
Gm20816	12851		#N/A	1.000000E+00
Gm20818	12852		#N/A	1.000000E+00
Gm20821	12853		#N/A	1.000000E+00
Gm20822	12854		#N/A	1.000000E+00
Gm20823	12855		#N/A	1.000000E+00
Gm20825	12856		#N/A	1.000000E+00

Spearman Rank correlation analysis performed between Prdm1 and all-expressed genes within the Meredith RNA-seq dataset. Robust Prdm1–associated genes were identified using a cut-off of $p < 0.0005$.

Table S1, Related to Supplemental Figure 3C. Prdm1 associated genes

Gm20826	12857	#N/A	1.000000E+00
Gm20827	12858	#N/A	1.000000E+00
Gm20828	12859	#N/A	1.000000E+00
Gm20830	12860	#N/A	1.000000E+00
Gm20831	12861	#N/A	1.000000E+00
Gm20834	12862	#N/A	1.000000E+00
Gm20836	12863	#N/A	1.000000E+00
Gm20837	12864	#N/A	1.000000E+00
Gm20838	12865	#N/A	1.000000E+00
Gm20840	12866	#N/A	1.000000E+00
Gm20841	12867	#N/A	1.000000E+00
Gm20842	12868	#N/A	1.000000E+00
Gm20843	12869	#N/A	1.000000E+00
Gm20844	12870	#N/A	1.000000E+00
Gm20846	12871	#N/A	1.000000E+00
Gm20847	12872	#N/A	1.000000E+00
Gm20848	12873	#N/A	1.000000E+00
Gm20849	12874	#N/A	1.000000E+00
Gm20851	12875	#N/A	1.000000E+00
Gm20852	12876	#N/A	1.000000E+00
Gm20853	12877	#N/A	1.000000E+00
Gm20854	12878	#N/A	1.000000E+00
Gm20855	12879	#N/A	1.000000E+00
Gm20856	12880	#N/A	1.000000E+00
Gm20859	12881	#N/A	1.000000E+00
Gm20860	12882	#N/A	1.000000E+00
Gm20861	12883	#N/A	1.000000E+00
Gm20862	12884	#N/A	1.000000E+00
Gm20863	12885	#N/A	1.000000E+00
Gm20864	12886	#N/A	1.000000E+00
Gm20865	12887	#N/A	1.000000E+00
Gm20866	12888	#N/A	1.000000E+00
Gm20867	12889	#N/A	1.000000E+00
Gm20868	12890	#N/A	1.000000E+00
Gm20873	12891	#N/A	1.000000E+00
Gm20874	12892	#N/A	1.000000E+00
Gm20877	12893	#N/A	1.000000E+00
Gm20878	12894	#N/A	1.000000E+00
Gm20880	12895	#N/A	1.000000E+00
Gm20881	12896	#N/A	1.000000E+00
Gm20882	12897	#N/A	1.000000E+00
Gm20884	12898	#N/A	1.000000E+00
Gm20886	12899	#N/A	1.000000E+00

Spearman Rank correlation analysis performed between Prdm1 and all-expressed genes within the Meredith RNA-seq dataset. Robust Prdm1–associated genes were identified using a cut-off of $p < 0.0005$.

Table S1, Related to Supplemental Figure 3C. Prdm1 associated genes

Gm20887	12900		#N/A	1.000000E+00
Gm20888	12901		#N/A	1.000000E+00
Gm20889	12902		#N/A	1.000000E+00
Gm20891	12903		#N/A	1.000000E+00
Gm20892	12904		#N/A	1.000000E+00
Gm20893	12905		#N/A	1.000000E+00
Gm20895	12906		#N/A	1.000000E+00
Gm20896	12907	0.015676693	6106.5	8.256174E-01
Gm20898	12908		#N/A	1.000000E+00
Gm20899	12909		#N/A	1.000000E+00
Gm20900	12910		#N/A	1.000000E+00
Gm20901	12911	0.113548679	13094	1.093908E-01
Gm20902	12912		#N/A	1.000000E+00
Gm20904	12913		#N/A	1.000000E+00
Gm20907	12914		#N/A	1.000000E+00
Gm20909	12915		#N/A	1.000000E+00
Gm20910	12916		#N/A	1.000000E+00
Gm20912	12917		#N/A	1.000000E+00
Gm20913	12918		#N/A	1.000000E+00
Gm20914	12919		#N/A	1.000000E+00
Gm20916	12920		#N/A	1.000000E+00
Gm20917	12921		#N/A	1.000000E+00
Gm20918	12922		#N/A	1.000000E+00
Gm20919	12923		#N/A	1.000000E+00
Gm2092	12924		#N/A	1.000000E+00
Gm20921	12925		#N/A	1.000000E+00
Gm20922	12926		#N/A	1.000000E+00
Gm20923	12927		#N/A	1.000000E+00
Gm20924	12928		#N/A	1.000000E+00
Gm20925	12929		#N/A	1.000000E+00
Gm20926	12930		#N/A	1.000000E+00
Gm20927	12931		#N/A	1.000000E+00
Gm20930	12932		#N/A	1.000000E+00
Gm20931	12933		#N/A	1.000000E+00
Gm20932	12934		#N/A	1.000000E+00
Gm20933	12935		#N/A	1.000000E+00
Gm20934	12936		#N/A	1.000000E+00
Gm20935	12937		#N/A	1.000000E+00
Gm20936	12938		#N/A	1.000000E+00
Gm20937	12939		#N/A	1.000000E+00
Gm20938	12940		#N/A	1.000000E+00
Gm20939	12941	0.073702434	10110	2.996483E-01
Gm20945	12942	-0.066858259	1029.5	3.468868E-01

Spearman Rank correlation analysis performed between Prdm1 and all-expressed genes within the Meredith RNA-seq dataset. Robust Prdm1–associated genes were identified using a cut-off of $p < 0.0005$.

Table S1, Related to Supplemental Figure 3C. Prdm1 associated genes

Gm20946	12943		#N/A	1.000000E+00
Gm21002	12944		#N/A	1.000000E+00
Gm2101	12945		#N/A	1.000000E+00
Gm21028	12946		#N/A	1.000000E+00
Gm21060	12947		#N/A	1.000000E+00
Gm21065	12948		#N/A	1.000000E+00
Gm21076	12949		#N/A	1.000000E+00
Gm2108	12950		#N/A	1.000000E+00
Gm21085	12951		#N/A	1.000000E+00
Gm21092	12952	0.160987082	16412.5	2.276801E-02
Gm21093	12953		#N/A	1.000000E+00
Gm21094	12954		#N/A	1.000000E+00
Gm21095	12955		#N/A	1.000000E+00
Gm21098	12956		#N/A	1.000000E+00
Gm21103	12957		#N/A	1.000000E+00
Gm21111	12958		#N/A	1.000000E+00
Gm21114	12959		#N/A	1.000000E+00
Gm21117	12960		#N/A	1.000000E+00
Gm21118	12961		#N/A	1.000000E+00
Gm21119	12962		#N/A	1.000000E+00
Gm21127	12963		#N/A	1.000000E+00
Gm21142	12964		#N/A	1.000000E+00
Gm21151	12965		#N/A	1.000000E+00
Gm21155	12966		#N/A	1.000000E+00
Gm21160	12967		#N/A	1.000000E+00
Gm21163	12968		#N/A	1.000000E+00
Gm2117	12969		#N/A	1.000000E+00
Gm21170	12970		#N/A	1.000000E+00
Gm21171	12971		#N/A	1.000000E+00
Gm21180	12972		#N/A	1.000000E+00
Gm21182	12973		#N/A	1.000000E+00
Gm21184	12974		#N/A	1.000000E+00
Gm21188	12975		#N/A	1.000000E+00
Gm21198	12976		#N/A	1.000000E+00
Gm21201	12977		#N/A	1.000000E+00
Gm21209	12978		#N/A	1.000000E+00
Gm21241	12979		#N/A	1.000000E+00
Gm21242	12980		#N/A	1.000000E+00
Gm21244	12981		#N/A	1.000000E+00
Gm21245	12982		#N/A	1.000000E+00
Gm21247	12983		#N/A	1.000000E+00
Gm21248	12984		#N/A	1.000000E+00
Gm21249	12985		#N/A	1.000000E+00

Spearman Rank correlation analysis performed between Prdm1 and all-expressed genes within the Meredith RNA-seq dataset. Robust Prdm1–associated genes were identified using a cut-off of $p < 0.0005$.

Table S1, Related to Supplemental Figure 3C. Prdm1 associated genes

Gm21256	12986		#N/A	1.000000E+00
Gm21257	12987		#N/A	1.000000E+00
Gm21258	12988	0.165207727	16671	1.939591E-02
Gm21268	12989		#N/A	1.000000E+00
Gm21271	12990		#N/A	1.000000E+00
Gm21275	12991		#N/A	1.000000E+00
Gm21276	12992		#N/A	1.000000E+00
Gm2128	12993		#N/A	1.000000E+00
Gm21281	12994		#N/A	1.000000E+00
Gm21282	12995		#N/A	1.000000E+00
Gm21285	12996		#N/A	1.000000E+00
Gm21286	12997		#N/A	1.000000E+00
Gm21287	12998		#N/A	1.000000E+00
Gm21292	12999		#N/A	1.000000E+00
Gm21302	13000		#N/A	1.000000E+00
Gm21308	13001		#N/A	1.000000E+00
Gm21310	13002		#N/A	1.000000E+00
Gm21312	13003		#N/A	1.000000E+00
Gm21316	13004		#N/A	1.000000E+00
Gm21319	13005		#N/A	1.000000E+00
Gm21330	13006		#N/A	1.000000E+00
Gm21333	13007		#N/A	1.000000E+00
Gm21340	13008		#N/A	1.000000E+00
Gm21344	13009		#N/A	1.000000E+00
Gm21350	13010		#N/A	1.000000E+00
Gm21364	13011		#N/A	1.000000E+00
Gm21370	13012		#N/A	1.000000E+00
Gm21378	13013		#N/A	1.000000E+00
Gm21379	13014		#N/A	1.000000E+00
Gm21380	13015		#N/A	1.000000E+00
Gm21387	13016		#N/A	1.000000E+00
Gm21394	13017		#N/A	1.000000E+00
Gm21396	13018		#N/A	1.000000E+00
Gm21399	13019	0.083452862	10867	2.400529E-01
Gm21405	13020		#N/A	1.000000E+00
Gm21411	13021	-0.013424049	4598	8.503567E-01
Gm21412	13022		#N/A	1.000000E+00
Gm21425	13023		#N/A	1.000000E+00
Gm21427	13024		#N/A	1.000000E+00
Gm21435	13025		#N/A	1.000000E+00
Gm21440	13026		#N/A	1.000000E+00
Gm21443	13027		#N/A	1.000000E+00
Gm21447	13028		#N/A	1.000000E+00

Spearman Rank correlation analysis performed between Prdm1 and all-expressed genes within the Meredith RNA-seq dataset. Robust Prdm1–associated genes were identified using a cut-off of $p < 0.0005$.

Table S1, Related to Supplemental Figure 3C. Prdm1 associated genes

Gm21451	13029	0.113548679	13094	1.093908E-01
Gm21462	13030		#N/A	1.000000E+00
Gm21464	13031		#N/A	1.000000E+00
Gm21469	13032		#N/A	1.000000E+00
Gm21470	13033		#N/A	1.000000E+00
Gm21476	13034		#N/A	1.000000E+00
Gm21477	13035		#N/A	1.000000E+00
Gm21492	13036		#N/A	1.000000E+00
Gm21498	13037		#N/A	1.000000E+00
Gm21506	13038		#N/A	1.000000E+00
Gm21518	13039		#N/A	1.000000E+00
Gm21524	13040		#N/A	1.000000E+00
Gm21529	13041		#N/A	1.000000E+00
Gm21530	13042		#N/A	1.000000E+00
Gm21539	13043		#N/A	1.000000E+00
Gm21541	13044		#N/A	1.000000E+00
Gm21560	13045		#N/A	1.000000E+00
Gm21562	13046		#N/A	1.000000E+00
Gm21572	13047		#N/A	1.000000E+00
Gm21573	13048		#N/A	1.000000E+00
Gm21582	13049		#N/A	1.000000E+00
Gm21586	13050		#N/A	1.000000E+00
Gm21588	13051		#N/A	1.000000E+00
Gm21596	13052		#N/A	1.000000E+00
Gm21598	13053		#N/A	1.000000E+00
Gm21599	13054		#N/A	1.000000E+00
Gm216	13055	-0.001606757	5107.5	9.819849E-01
Gm21608	13056		#N/A	1.000000E+00
Gm21616	13057		#N/A	1.000000E+00
Gm21617	13058		#N/A	1.000000E+00
Gm21626	13059		#N/A	1.000000E+00
Gm2163	13060		#N/A	1.000000E+00
Gm21633	13061		#N/A	1.000000E+00
Gm21634	13062		#N/A	1.000000E+00
Gm21637	13063		#N/A	1.000000E+00
Gm21638	13064		#N/A	1.000000E+00
Gm21642	13065		#N/A	1.000000E+00
Gm21645	13066		#N/A	1.000000E+00
Gm2165	13067		#N/A	1.000000E+00
Gm21650	13068		#N/A	1.000000E+00
Gm21654	13069		#N/A	1.000000E+00
Gm21655	13070		#N/A	1.000000E+00
Gm21657	13071		#N/A	1.000000E+00

Spearman Rank correlation analysis performed between Prdm1 and all-expressed genes within the Meredith RNA-seq dataset. Robust Prdm1–associated genes were identified using a cut-off of $p < 0.0005$.

Table S1, Related to Supplemental Figure 3C. Prdm1 associated genes

Gm21658	13072		#N/A	1.000000E+00
Gm21660	13073		#N/A	1.000000E+00
Gm21661	13074		#N/A	1.000000E+00
Gm21671	13075		#N/A	1.000000E+00
Gm21672	13076		#N/A	1.000000E+00
Gm21677	13077		#N/A	1.000000E+00
Gm21679	13078		#N/A	1.000000E+00
Gm21681	13079		#N/A	1.000000E+00
Gm21683	13080		#N/A	1.000000E+00
Gm21685	13081	-0.038406171	3065	5.892384E-01
Gm21693	13082		#N/A	1.000000E+00
Gm21698	13083		#N/A	1.000000E+00
Gm21699	13084		#N/A	1.000000E+00
Gm21704	13085		#N/A	1.000000E+00
Gm21708	13086		#N/A	1.000000E+00
Gm21715	13087		#N/A	1.000000E+00
Gm21717	13088		#N/A	1.000000E+00
Gm21718	13089	-0.038406171	3065	5.892384E-01
Gm21719	13090		#N/A	1.000000E+00
Gm21720	13091		#N/A	1.000000E+00
Gm21721	13092		#N/A	1.000000E+00
Gm21722	13093		#N/A	1.000000E+00
Gm21723	13094		#N/A	1.000000E+00
Gm21724	13095		#N/A	1.000000E+00
Gm21725	13096		#N/A	1.000000E+00
Gm21726	13097	0.160987082	16412.5	2.276801E-02
Gm21728	13098		#N/A	1.000000E+00
Gm21729	13099	-0.054451513	1703	4.437940E-01
Gm21731	13100		#N/A	1.000000E+00
Gm21732	13101		#N/A	1.000000E+00
Gm21733	13102		#N/A	1.000000E+00
Gm21734	13103		#N/A	1.000000E+00
Gm21735	13104		#N/A	1.000000E+00
Gm21736	13105		#N/A	1.000000E+00
Gm21737	13106		#N/A	1.000000E+00
Gm21738	13107	-0.014940376	4512	8.336863E-01
Gm21739	13108		#N/A	1.000000E+00
Gm2174	13109		#N/A	1.000000E+00
Gm21740	13110		#N/A	1.000000E+00
Gm21741	13111		#N/A	1.000000E+00
Gm21742	13112		#N/A	1.000000E+00
Gm21743	13113		#N/A	1.000000E+00
Gm21744	13114		#N/A	1.000000E+00

Spearman Rank correlation analysis performed between Prdm1 and all-expressed genes within the Meredith RNA-seq dataset. Robust Prdm1–associated genes were identified using a cut-off of $p < 0.0005$.

Table S1, Related to Supplemental Figure 3C. Prdm1 associated genes

Gm21745	13115		#N/A	1.000000E+00
Gm21746	13116		#N/A	1.000000E+00
Gm21747	13117		#N/A	1.000000E+00
Gm21748	13118		#N/A	1.000000E+00
Gm21749	13119		#N/A	1.000000E+00
Gm21750	13120		#N/A	1.000000E+00
Gm21751	13121		#N/A	1.000000E+00
Gm21752	13122		#N/A	1.000000E+00
Gm21753	13123		#N/A	1.000000E+00
Gm21754	13124		#N/A	1.000000E+00
Gm21755	13125		#N/A	1.000000E+00
Gm21756	13126		#N/A	1.000000E+00
Gm21757	13127		#N/A	1.000000E+00
Gm21758	13128		#N/A	1.000000E+00
Gm21759	13129		#N/A	1.000000E+00
Gm21760	13130	-0.038406171	3065	5.892384E-01
Gm21761	13131		#N/A	1.000000E+00
Gm21762	13132		#N/A	1.000000E+00
Gm21763	13133		#N/A	1.000000E+00
Gm21764	13134		#N/A	1.000000E+00
Gm21765	13135		#N/A	1.000000E+00
Gm21766	13136		#N/A	1.000000E+00
Gm21767	13137		#N/A	1.000000E+00
Gm21768	13138		#N/A	1.000000E+00
Gm21769	13139	0.192355888	17926	6.356475E-03
Gm21771	13140		#N/A	1.000000E+00
Gm21772	13141		#N/A	1.000000E+00
Gm21773	13142		#N/A	1.000000E+00
Gm21774	13143		#N/A	1.000000E+00
Gm21775	13144		#N/A	1.000000E+00
Gm21776	13145		#N/A	1.000000E+00
Gm21777	13146		#N/A	1.000000E+00
Gm21778	13147		#N/A	1.000000E+00
Gm21779	13148		#N/A	1.000000E+00
Gm2178	13149	0.135169958	14636.5	5.634245E-02
Gm21780	13150		#N/A	1.000000E+00
Gm21781	13151	0.090429409	11376	2.028550E-01
Gm21782	13152		#N/A	1.000000E+00
Gm21783	13153		#N/A	1.000000E+00
Gm21784	13154		#N/A	1.000000E+00
Gm21785	13155		#N/A	1.000000E+00
Gm21786	13156		#N/A	1.000000E+00
Gm21787	13157		#N/A	1.000000E+00

Spearman Rank correlation analysis performed between Prdm1 and all-expressed genes within the Meredith RNA-seq dataset. Robust Prdm1–associated genes were identified using a cut-off of $p < 0.0005$.

Table S1, Related to Supplemental Figure 3C. Prdm1 associated genes

Gm21788	13158		#N/A	1.000000E+00
Gm21789	13159		#N/A	1.000000E+00
Gm21790	13160		#N/A	1.000000E+00
Gm21791	13161		#N/A	1.000000E+00
Gm21792	13162		#N/A	1.000000E+00
Gm21794	13163		#N/A	1.000000E+00
Gm21795	13164		#N/A	1.000000E+00
Gm21796	13165		#N/A	1.000000E+00
Gm21797	13166		#N/A	1.000000E+00
Gm21798	13167	-0.003089539	5019	9.653676E-01
Gm21799	13168		#N/A	1.000000E+00
Gm21800	13169		#N/A	1.000000E+00
Gm21801	13170		#N/A	1.000000E+00
Gm21802	13171		#N/A	1.000000E+00
Gm21803	13172		#N/A	1.000000E+00
Gm21804	13173		#N/A	1.000000E+00
Gm21805	13174		#N/A	1.000000E+00
Gm21806	13175		#N/A	1.000000E+00
Gm21807	13176		#N/A	1.000000E+00
Gm21808	13177		#N/A	1.000000E+00
Gm21809	13178		#N/A	1.000000E+00
Gm21810	13179		#N/A	1.000000E+00
Gm21811	13180	-0.054451513	1703	4.437940E-01
Gm21812	13181		#N/A	1.000000E+00
Gm21813	13182		#N/A	1.000000E+00
Gm21814	13183	-0.110568513	165.5	1.190777E-01
Gm21815	13184	-0.086750697	472.5	2.219141E-01
Gm21816	13185		#N/A	1.000000E+00
Gm21817	13186		#N/A	1.000000E+00
Gm21818	13187		#N/A	1.000000E+00
Gm21819	13188		#N/A	1.000000E+00
Gm2182	13189		#N/A	1.000000E+00
Gm21820	13190		#N/A	1.000000E+00
Gm21821	13191		#N/A	1.000000E+00
Gm21822	13192		#N/A	1.000000E+00
Gm21823	13193		#N/A	1.000000E+00
Gm21824	13194		#N/A	1.000000E+00
Gm21825	13195		#N/A	1.000000E+00
Gm21826	13196		#N/A	1.000000E+00
Gm21827	13197		#N/A	1.000000E+00
Gm21828	13198		#N/A	1.000000E+00
Gm21829	13199		#N/A	1.000000E+00
Gm21830	13200		#N/A	1.000000E+00

Spearman Rank correlation analysis performed between Prdm1 and all-expressed genes within the Meredith RNA-seq dataset. Robust Prdm1–associated genes were identified using a cut-off of $p < 0.0005$.

Table S1, Related to Supplemental Figure 3C. Prdm1 associated genes

Gm21832	13201		#N/A	1.000000E+00
Gm21833	13202		#N/A	1.000000E+00
Gm21834	13203		#N/A	1.000000E+00
Gm21835	13204		#N/A	1.000000E+00
Gm21836	13205		#N/A	1.000000E+00
Gm21837	13206		#N/A	1.000000E+00
Gm21838	13207		#N/A	1.000000E+00
Gm21839	13208		#N/A	1.000000E+00
Gm21840	13209		#N/A	1.000000E+00
Gm21841	13210		#N/A	1.000000E+00
Gm21842	13211		#N/A	1.000000E+00
Gm21843	13212		#N/A	1.000000E+00
Gm21844	13213		#N/A	1.000000E+00
Gm21845	13214		#N/A	1.000000E+00
Gm21846	13215		#N/A	1.000000E+00
GM21847	13216		#N/A	1.000000E+00
Gm21847	13217	-0.095280123	319	1.795721E-01
Gm21848	13218		#N/A	1.000000E+00
Gm21849	13219		#N/A	1.000000E+00
Gm21850	13220		#N/A	1.000000E+00
Gm21851	13221		#N/A	1.000000E+00
Gm21852	13222		#N/A	1.000000E+00
Gm21853	13223		#N/A	1.000000E+00
Gm21854	13224		#N/A	1.000000E+00
Gm21855	13225		#N/A	1.000000E+00
Gm21856	13226		#N/A	1.000000E+00
Gm21857	13227		#N/A	1.000000E+00
Gm21858	13228		#N/A	1.000000E+00
Gm21859	13229		#N/A	1.000000E+00
Gm21860	13230		#N/A	1.000000E+00
Gm21861	13231		#N/A	1.000000E+00
Gm21862	13232		#N/A	1.000000E+00
Gm21863	13233		#N/A	1.000000E+00
Gm21864	13234		#N/A	1.000000E+00
Gm21865	13235	-0.038406171	3065	5.892384E-01
Gm21866	13236		#N/A	1.000000E+00
Gm21867	13237		#N/A	1.000000E+00
Gm21868	13238		#N/A	1.000000E+00
Gm21869	13239		#N/A	1.000000E+00
Gm21870	13240		#N/A	1.000000E+00
Gm21871	13241		#N/A	1.000000E+00
Gm21872	13242		#N/A	1.000000E+00
Gm21873	13243		#N/A	1.000000E+00

Spearman Rank correlation analysis performed between Prdm1 and all-expressed genes within the Meredith RNA-seq dataset. Robust Prdm1–associated genes were identified using a cut-off of $p < 0.0005$.

Table S1, Related to Supplemental Figure 3C. Prdm1 associated genes

Gm21874	13244		#N/A	1.000000E+00
Gm21875	13245		#N/A	1.000000E+00
Gm21876	13246		#N/A	1.000000E+00
Gm21877	13247		#N/A	1.000000E+00
Gm21878	13248		#N/A	1.000000E+00
Gm21879	13249		#N/A	1.000000E+00
Gm21880	13250		#N/A	1.000000E+00
Gm21881	13251		#N/A	1.000000E+00
Gm21882	13252		#N/A	1.000000E+00
Gm21883	13253		#N/A	1.000000E+00
Gm21884	13254		#N/A	1.000000E+00
Gm21885	13255	-0.038406171	3065	5.892384E-01
Gm21886	13256		#N/A	1.000000E+00
Gm21887	13257		#N/A	1.000000E+00
Gm21889	13258		#N/A	1.000000E+00
Gm21890	13259		#N/A	1.000000E+00
Gm21891	13260		#N/A	1.000000E+00
Gm21892	13261		#N/A	1.000000E+00
Gm21893	13262		#N/A	1.000000E+00
Gm21894	13263		#N/A	1.000000E+00
Gm21895	13264		#N/A	1.000000E+00
Gm21896	13265		#N/A	1.000000E+00
Gm21897	13266		#N/A	1.000000E+00
Gm21898	13267		#N/A	1.000000E+00
Gm21899	13268		#N/A	1.000000E+00
Gm21900	13269		#N/A	1.000000E+00
Gm21901	13270		#N/A	1.000000E+00
Gm21902	13271		#N/A	1.000000E+00
Gm21903	13272		#N/A	1.000000E+00
Gm21904	13273		#N/A	1.000000E+00
Gm21905	13274	-0.045728293	2300	5.202379E-01
Gm21906	13275		#N/A	1.000000E+00
Gm21907	13276		#N/A	1.000000E+00
Gm21908	13277		#N/A	1.000000E+00
Gm21909	13278		#N/A	1.000000E+00
Gm21910	13279		#N/A	1.000000E+00
Gm21911	13280		#N/A	1.000000E+00
Gm21912	13281		#N/A	1.000000E+00
GM21913	13282		#N/A	1.000000E+00
Gm21913	13283		#N/A	1.000000E+00
Gm21914	13284		#N/A	1.000000E+00
Gm21915	13285		#N/A	1.000000E+00
Gm21916	13286		#N/A	1.000000E+00

Spearman Rank correlation analysis performed between Prdm1 and all-expressed genes within the Meredith RNA-seq dataset. Robust Prdm1–associated genes were identified using a cut-off of $p < 0.0005$.

Table S1, Related to Supplemental Figure 3C. Prdm1 associated genes

Gm21917	13287	-0.038406171	3065	5.892384E-01
Gm21918	13288		#N/A	1.000000E+00
Gm21919	13289		#N/A	1.000000E+00
Gm21920	13290		#N/A	1.000000E+00
Gm21921	13291		#N/A	1.000000E+00
Gm21922	13292		#N/A	1.000000E+00
Gm21923	13293		#N/A	1.000000E+00
Gm21924	13294		#N/A	1.000000E+00
Gm21943	13295		#N/A	1.000000E+00
Gm21945	13296		#N/A	1.000000E+00
Gm21946	13297		#N/A	1.000000E+00
Gm21947	13298		#N/A	1.000000E+00
Gm21950	13299		#N/A	1.000000E+00
Gm21951	13300		#N/A	1.000000E+00
Gm21953	13301		#N/A	1.000000E+00
Gm21954	13302	0.071776772	9978	3.124868E-01
Gm21955	13303		#N/A	1.000000E+00
Gm21956	13304		#N/A	1.000000E+00
Gm21957	13305		#N/A	1.000000E+00
Gm21958	13306		#N/A	1.000000E+00
Gm21959	13307	0.141884531	15076	4.505682E-02
Gm21960	13308	0.143605682	15224	4.249002E-02
Gm21961	13309	-0.015803621	4480	8.242283E-01
Gm21962	13310		#N/A	1.000000E+00
Gm21963	13311		#N/A	1.000000E+00
Gm21964	13312	0.052728522	8550.5	4.583686E-01
Gm21965	13313		#N/A	1.000000E+00
Gm21966	13314		#N/A	1.000000E+00
Gm21967	13315		#N/A	1.000000E+00
Gm21968	13316		#N/A	1.000000E+00
Gm21969	13317	0.027260918	6942	7.015846E-01
Gm21970	13318	0.116039739	13398	1.017777E-01
Gm21971	13319	-0.054451513	1703	4.437940E-01
Gm21972	13320	-0.028606	3975	6.876130E-01
Gm21973	13321	0.094984654	11697	1.809313E-01
Gm21974	13322	0.105133832	12366	1.384450E-01
Gm21975	13323	0.286286606	19845	3.962138E-05
Gm21976	13324	0.300498092	19908	1.536484E-05
Gm21977	13325	0.143605682	15224	4.249002E-02
Gm21978	13326		#N/A	1.000000E+00
Gm21979	13327		#N/A	1.000000E+00
Gm21980	13328		#N/A	1.000000E+00
Gm21981	13329	-0.038406171	3065	5.892384E-01

Spearman Rank correlation analysis performed between Prdm1 and all-expressed genes within the Meredith RNA-seq dataset. Robust Prdm1–associated genes were identified using a cut-off of $p < 0.0005$.

Table S1, Related to Supplemental Figure 3C. Prdm1 associated genes

Gm21982	13330	0.22553151	19061	1.322847E-03
Gm21983	13331	0.026047992	6874	7.142700E-01
Gm21984	13332	0.112118269	12862	1.139601E-01
Gm21985	13333	0.133160205	14486	6.014275E-02
Gm21986	13334	-0.00333412	5014	9.626279E-01
Gm21987	13335	-0.038406171	3065	5.892384E-01
Gm21988	13336	0.033288478	7257	6.398210E-01
Gm21989	13337	0.124055058	13912	8.008834E-02
Gm2199	13338		#N/A	1.000000E+00
Gm21990	13339		#N/A	1.000000E+00
Gm21991	13340		#N/A	1.000000E+00
Gm21992	13341	0.140018102	14958	4.798564E-02
Gm21994	13342		#N/A	1.000000E+00
Gm21998	13343		#N/A	1.000000E+00
Gm21999	13344		#N/A	1.000000E+00
Gm22	13345	-0.000929698	5187.5	9.895755E-01
Gm2200	13346		#N/A	1.000000E+00
Gm22000	13347		#N/A	1.000000E+00
Gm22001	13348		#N/A	1.000000E+00
Gm22002	13349		#N/A	1.000000E+00
Gm22003	13350		#N/A	1.000000E+00
Gm22004	13351		#N/A	1.000000E+00
Gm22005	13352		#N/A	1.000000E+00
Gm22006	13353		#N/A	1.000000E+00
Gm22007	13354		#N/A	1.000000E+00
Gm22008	13355		#N/A	1.000000E+00
Gm22009	13356		#N/A	1.000000E+00
Gm22010	13357		#N/A	1.000000E+00
Gm22011	13358		#N/A	1.000000E+00
Gm22012	13359		#N/A	1.000000E+00
Gm22013	13360		#N/A	1.000000E+00
Gm22014	13361		#N/A	1.000000E+00
Gm22015	13362		#N/A	1.000000E+00
Gm22016	13363		#N/A	1.000000E+00
Gm22017	13364		#N/A	1.000000E+00
Gm22018	13365		#N/A	1.000000E+00
Gm22019	13366		#N/A	1.000000E+00
Gm22020	13367		#N/A	1.000000E+00
Gm22021	13368		#N/A	1.000000E+00
Gm22022	13369		#N/A	1.000000E+00
Gm22023	13370		#N/A	1.000000E+00
Gm22024	13371		#N/A	1.000000E+00
Gm22025	13372		#N/A	1.000000E+00

Spearman Rank correlation analysis performed between Prdm1 and all-expressed genes within the Meredith RNA-seq dataset. Robust Prdm1–associated genes were identified using a cut-off of $p < 0.0005$.

Table S1, Related to Supplemental Figure 3C. Prdm1 associated genes

Gm22026	13373		#N/A	1.000000E+00
Gm22027	13374		#N/A	1.000000E+00
Gm22028	13375		#N/A	1.000000E+00
Gm22029	13376		#N/A	1.000000E+00
Gm22030	13377	0.160304018	16323	2.335876E-02
Gm22031	13378		#N/A	1.000000E+00
Gm22032	13379		#N/A	1.000000E+00
Gm22033	13380		#N/A	1.000000E+00
Gm22034	13381		#N/A	1.000000E+00
Gm22035	13382		#N/A	1.000000E+00
Gm22036	13383		#N/A	1.000000E+00
Gm22037	13384		#N/A	1.000000E+00
Gm22038	13385		#N/A	1.000000E+00
Gm22039	13386		#N/A	1.000000E+00
Gm22040	13387		#N/A	1.000000E+00
Gm22041	13388		#N/A	1.000000E+00
Gm22042	13389		#N/A	1.000000E+00
Gm22043	13390		#N/A	1.000000E+00
Gm22044	13391		#N/A	1.000000E+00
Gm22045	13392		#N/A	1.000000E+00
Gm22046	13393		#N/A	1.000000E+00
Gm22047	13394		#N/A	1.000000E+00
Gm22048	13395		#N/A	1.000000E+00
Gm22049	13396		#N/A	1.000000E+00
Gm22050	13397		#N/A	1.000000E+00
Gm22051	13398		#N/A	1.000000E+00
Gm22052	13399		#N/A	1.000000E+00
Gm22053	13400		#N/A	1.000000E+00
Gm22054	13401		#N/A	1.000000E+00
Gm22055	13402		#N/A	1.000000E+00
Gm22056	13403	-0.066858259	1029.5	3.468868E-01
Gm22057	13404		#N/A	1.000000E+00
Gm22058	13405		#N/A	1.000000E+00
Gm22059	13406		#N/A	1.000000E+00
Gm22060	13407		#N/A	1.000000E+00
Gm22061	13408		#N/A	1.000000E+00
Gm22062	13409		#N/A	1.000000E+00
Gm22063	13410		#N/A	1.000000E+00
Gm22064	13411	0.182398428	17520.5	9.736288E-03
Gm22065	13412		#N/A	1.000000E+00
Gm22066	13413		#N/A	1.000000E+00
Gm22067	13414		#N/A	1.000000E+00
Gm22068	13415		#N/A	1.000000E+00

Spearman Rank correlation analysis performed between Prdm1 and all-expressed genes within the Meredith RNA-seq dataset. Robust Prdm1–associated genes were identified using a cut-off of $p < 0.0005$.

Table S1, Related to Supplemental Figure 3C. Prdm1 associated genes

Gm22069	13416	#N/A	1.000000E+00
Gm22070	13417	#N/A	1.000000E+00
Gm22071	13418	#N/A	1.000000E+00
Gm22072	13419	#N/A	1.000000E+00
Gm22073	13420	#N/A	1.000000E+00
Gm22074	13421	#N/A	1.000000E+00
Gm22075	13422	#N/A	1.000000E+00
Gm22076	13423	#N/A	1.000000E+00
Gm22077	13424	#N/A	1.000000E+00
Gm22078	13425	#N/A	1.000000E+00
Gm22079	13426	#N/A	1.000000E+00
Gm22080	13427	#N/A	1.000000E+00
Gm22081	13428	#N/A	1.000000E+00
Gm22082	13429	#N/A	1.000000E+00
Gm22083	13430	#N/A	1.000000E+00
Gm22084	13431	#N/A	1.000000E+00
Gm22085	13432	#N/A	1.000000E+00
Gm22086	13433	#N/A	1.000000E+00
Gm22087	13434	#N/A	1.000000E+00
Gm22088	13435	#N/A	1.000000E+00
Gm22089	13436	#N/A	1.000000E+00
Gm22090	13437	#N/A	1.000000E+00
Gm22091	13438	#N/A	1.000000E+00
Gm22092	13439	#N/A	1.000000E+00
Gm22093	13440	#N/A	1.000000E+00
Gm22094	13441	#N/A	1.000000E+00
Gm22095	13442	#N/A	1.000000E+00
Gm22096	13443	#N/A	1.000000E+00
Gm22097	13444	#N/A	1.000000E+00
Gm22098	13445	#N/A	1.000000E+00
Gm22099	13446	#N/A	1.000000E+00
Gm22100	13447	#N/A	1.000000E+00
Gm22101	13448	#N/A	1.000000E+00
Gm22102	13449	#N/A	1.000000E+00
Gm22103	13450	#N/A	1.000000E+00
Gm22104	13451	#N/A	1.000000E+00
Gm22105	13452	#N/A	1.000000E+00
Gm22106	13453	#N/A	1.000000E+00
Gm22107	13454	#N/A	1.000000E+00
Gm22108	13455	#N/A	1.000000E+00
Gm22109	13456	#N/A	1.000000E+00
Gm22110	13457	#N/A	1.000000E+00
Gm22111	13458	#N/A	1.000000E+00

Spearman Rank correlation analysis performed between Prdm1 and all-expressed genes within the Meredith RNA-seq dataset. Robust Prdm1–associated genes were identified using a cut-off of $p < 0.0005$.

Table S1, Related to Supplemental Figure 3C. Prdm1 associated genes

Gm22112	13459		#N/A	1.000000E+00
Gm22113	13460		#N/A	1.000000E+00
Gm22114	13461		#N/A	1.000000E+00
Gm22115	13462		#N/A	1.000000E+00
Gm22116	13463		#N/A	1.000000E+00
Gm22117	13464		#N/A	1.000000E+00
Gm22118	13465		#N/A	1.000000E+00
Gm22119	13466		#N/A	1.000000E+00
Gm22120	13467		#N/A	1.000000E+00
Gm22121	13468		#N/A	1.000000E+00
Gm22122	13469		#N/A	1.000000E+00
Gm22123	13470		#N/A	1.000000E+00
Gm22124	13471		#N/A	1.000000E+00
Gm22125	13472		#N/A	1.000000E+00
Gm22126	13473		#N/A	1.000000E+00
Gm22127	13474		#N/A	1.000000E+00
Gm22128	13475		#N/A	1.000000E+00
Gm22129	13476		#N/A	1.000000E+00
Gm22130	13477		#N/A	1.000000E+00
Gm22131	13478		#N/A	1.000000E+00
Gm22132	13479		#N/A	1.000000E+00
Gm22133	13480	0.081896798	10737	2.489622E-01
Gm22134	13481		#N/A	1.000000E+00
Gm22135	13482		#N/A	1.000000E+00
Gm22136	13483		#N/A	1.000000E+00
Gm22137	13484		#N/A	1.000000E+00
Gm22138	13485		#N/A	1.000000E+00
Gm22139	13486		#N/A	1.000000E+00
Gm2214	13487		#N/A	1.000000E+00
Gm22140	13488		#N/A	1.000000E+00
Gm22141	13489		#N/A	1.000000E+00
Gm22142	13490		#N/A	1.000000E+00
Gm22143	13491		#N/A	1.000000E+00
Gm22144	13492		#N/A	1.000000E+00
Gm22145	13493		#N/A	1.000000E+00
Gm22146	13494		#N/A	1.000000E+00
Gm22147	13495		#N/A	1.000000E+00
Gm22148	13496		#N/A	1.000000E+00
Gm22149	13497		#N/A	1.000000E+00
Gm22150	13498		#N/A	1.000000E+00
Gm22151	13499		#N/A	1.000000E+00
Gm22152	13500		#N/A	1.000000E+00
Gm22153	13501		#N/A	1.000000E+00

Spearman Rank correlation analysis performed between Prdm1 and all-expressed genes within the Meredith RNA-seq dataset. Robust Prdm1–associated genes were identified using a cut-off of $p < 0.0005$.

Table S1, Related to Supplemental Figure 3C. Prdm1 associated genes

Gm22154	13502		#N/A	1.000000E+00
Gm22155	13503		#N/A	1.000000E+00
Gm22156	13504		#N/A	1.000000E+00
Gm22157	13505		#N/A	1.000000E+00
Gm22158	13506		#N/A	1.000000E+00
Gm22159	13507		#N/A	1.000000E+00
Gm22160	13508		#N/A	1.000000E+00
Gm22161	13509		#N/A	1.000000E+00
Gm22162	13510		#N/A	1.000000E+00
Gm22163	13511		#N/A	1.000000E+00
Gm22164	13512		#N/A	1.000000E+00
Gm22165	13513		#N/A	1.000000E+00
Gm22166	13514		#N/A	1.000000E+00
Gm22167	13515		#N/A	1.000000E+00
Gm22168	13516		#N/A	1.000000E+00
Gm22169	13517		#N/A	1.000000E+00
Gm22170	13518		#N/A	1.000000E+00
Gm22171	13519		#N/A	1.000000E+00
Gm22172	13520		#N/A	1.000000E+00
Gm22173	13521		#N/A	1.000000E+00
Gm22174	13522		#N/A	1.000000E+00
Gm22175	13523		#N/A	1.000000E+00
Gm22176	13524		#N/A	1.000000E+00
Gm22177	13525		#N/A	1.000000E+00
Gm22178	13526		#N/A	1.000000E+00
Gm22179	13527	-0.066549458	1290	3.491235E-01
Gm22180	13528		#N/A	1.000000E+00
Gm22181	13529		#N/A	1.000000E+00
Gm22182	13530		#N/A	1.000000E+00
Gm22183	13531		#N/A	1.000000E+00
Gm22184	13532		#N/A	1.000000E+00
Gm22185	13533		#N/A	1.000000E+00
Gm22186	13534		#N/A	1.000000E+00
Gm22187	13535		#N/A	1.000000E+00
Gm22188	13536		#N/A	1.000000E+00
Gm22189	13537		#N/A	1.000000E+00
Gm22190	13538		#N/A	1.000000E+00
Gm22191	13539		#N/A	1.000000E+00
Gm22192	13540		#N/A	1.000000E+00
Gm22193	13541		#N/A	1.000000E+00
Gm22194	13542		#N/A	1.000000E+00
Gm22195	13543		#N/A	1.000000E+00
Gm22196	13544		#N/A	1.000000E+00

Spearman Rank correlation analysis performed between Prdm1 and all-expressed genes within the Meredith RNA-seq dataset. Robust Prdm1–associated genes were identified using a cut-off of $p < 0.0005$.

Table S1, Related to Supplemental Figure 3C. Prdm1 associated genes

Gm22197	13545		#N/A	1.000000E+00
Gm22198	13546		#N/A	1.000000E+00
Gm22199	13547		#N/A	1.000000E+00
Gm22200	13548		#N/A	1.000000E+00
Gm22201	13549		#N/A	1.000000E+00
Gm22202	13550		#N/A	1.000000E+00
Gm22203	13551		#N/A	1.000000E+00
Gm22204	13552		#N/A	1.000000E+00
Gm22205	13553		#N/A	1.000000E+00
Gm22206	13554		#N/A	1.000000E+00
Gm22207	13555		#N/A	1.000000E+00
Gm22208	13556		#N/A	1.000000E+00
Gm22209	13557		#N/A	1.000000E+00
Gm22210	13558		#N/A	1.000000E+00
Gm22211	13559		#N/A	1.000000E+00
Gm22212	13560		#N/A	1.000000E+00
Gm22213	13561		#N/A	1.000000E+00
Gm22214	13562		#N/A	1.000000E+00
Gm22215	13563		#N/A	1.000000E+00
Gm22216	13564		#N/A	1.000000E+00
Gm22217	13565		#N/A	1.000000E+00
Gm22218	13566		#N/A	1.000000E+00
Gm22219	13567		#N/A	1.000000E+00
Gm22220	13568		#N/A	1.000000E+00
Gm22221	13569		#N/A	1.000000E+00
Gm22222	13570		#N/A	1.000000E+00
Gm22223	13571		#N/A	1.000000E+00
Gm22224	13572		#N/A	1.000000E+00
Gm22225	13573		#N/A	1.000000E+00
Gm22226	13574		#N/A	1.000000E+00
Gm22227	13575		#N/A	1.000000E+00
Gm22228	13576		#N/A	1.000000E+00
Gm22229	13577	0.052728522	8550.5	4.583686E-01
Gm2223	13578		#N/A	1.000000E+00
Gm22230	13579		#N/A	1.000000E+00
Gm22231	13580		#N/A	1.000000E+00
Gm22232	13581		#N/A	1.000000E+00
Gm22233	13582		#N/A	1.000000E+00
Gm22234	13583		#N/A	1.000000E+00
Gm22235	13584		#N/A	1.000000E+00
Gm22236	13585		#N/A	1.000000E+00
Gm22237	13586		#N/A	1.000000E+00
Gm22238	13587		#N/A	1.000000E+00

Spearman Rank correlation analysis performed between Prdm1 and all-expressed genes within the Meredith RNA-seq dataset. Robust Prdm1–associated genes were identified using a cut-off of $p < 0.0005$.

Table S1, Related to Supplemental Figure 3C. Prdm1 associated genes

Gm22239	13588		#N/A	1.000000E+00
Gm22240	13589		#N/A	1.000000E+00
Gm22241	13590		#N/A	1.000000E+00
Gm22242	13591		#N/A	1.000000E+00
Gm22243	13592		#N/A	1.000000E+00
Gm22244	13593		#N/A	1.000000E+00
Gm22245	13594		#N/A	1.000000E+00
Gm22246	13595		#N/A	1.000000E+00
Gm22247	13596		#N/A	1.000000E+00
Gm22248	13597		#N/A	1.000000E+00
Gm22249	13598		#N/A	1.000000E+00
Gm22250	13599		#N/A	1.000000E+00
Gm22251	13600		#N/A	1.000000E+00
Gm22252	13601		#N/A	1.000000E+00
Gm22253	13602		#N/A	1.000000E+00
Gm22254	13603		#N/A	1.000000E+00
Gm22255	13604		#N/A	1.000000E+00
Gm22256	13605	-0.054451513	1703	4.437940E-01
Gm22257	13606		#N/A	1.000000E+00
Gm22258	13607		#N/A	1.000000E+00
Gm22259	13608	0.143605682	15224	4.249002E-02
Gm22260	13609		#N/A	1.000000E+00
Gm22261	13610		#N/A	1.000000E+00
Gm22262	13611		#N/A	1.000000E+00
Gm22263	13612		#N/A	1.000000E+00
Gm22264	13613		#N/A	1.000000E+00
Gm22265	13614		#N/A	1.000000E+00
Gm22266	13615		#N/A	1.000000E+00
Gm22267	13616		#N/A	1.000000E+00
Gm22268	13617		#N/A	1.000000E+00
Gm22269	13618		#N/A	1.000000E+00
Gm22270	13619		#N/A	1.000000E+00
Gm22271	13620		#N/A	1.000000E+00
Gm22272	13621		#N/A	1.000000E+00
Gm22273	13622		#N/A	1.000000E+00
Gm22274	13623		#N/A	1.000000E+00
Gm22275	13624		#N/A	1.000000E+00
Gm22276	13625		#N/A	1.000000E+00
Gm22277	13626		#N/A	1.000000E+00
Gm22278	13627		#N/A	1.000000E+00
Gm22279	13628		#N/A	1.000000E+00
Gm22280	13629		#N/A	1.000000E+00
Gm22281	13630		#N/A	1.000000E+00

Spearman Rank correlation analysis performed between Prdm1 and all-expressed genes within the Meredith RNA-seq dataset. Robust Prdm1–associated genes were identified using a cut-off of $p < 0.0005$.

Table S1, Related to Supplemental Figure 3C. Prdm1 associated genes

Gm22282	13631	#N/A	1.000000E+00
Gm22283	13632	#N/A	1.000000E+00
Gm22284	13633	#N/A	1.000000E+00
Gm22285	13634	#N/A	1.000000E+00
Gm22286	13635	#N/A	1.000000E+00
Gm22287	13636	#N/A	1.000000E+00
Gm22288	13637	#N/A	1.000000E+00
Gm22289	13638	#N/A	1.000000E+00
Gm22290	13639	#N/A	1.000000E+00
Gm22291	13640	#N/A	1.000000E+00
Gm22292	13641	#N/A	1.000000E+00
Gm22293	13642	#N/A	1.000000E+00
Gm22294	13643	#N/A	1.000000E+00
Gm22295	13644	#N/A	1.000000E+00
Gm22296	13645	#N/A	1.000000E+00
Gm22297	13646	#N/A	1.000000E+00
Gm22298	13647	#N/A	1.000000E+00
Gm22299	13648	#N/A	1.000000E+00
Gm22300	13649	#N/A	1.000000E+00
Gm22301	13650	#N/A	1.000000E+00
Gm22302	13651	#N/A	1.000000E+00
Gm22303	13652	#N/A	1.000000E+00
Gm22304	13653	#N/A	1.000000E+00
Gm22305	13654	#N/A	1.000000E+00
Gm22306	13655	#N/A	1.000000E+00
Gm22307	13656	#N/A	1.000000E+00
Gm22308	13657	#N/A	1.000000E+00
Gm22309	13658	#N/A	1.000000E+00
Gm2231	13659	#N/A	1.000000E+00
Gm22310	13660	#N/A	1.000000E+00
Gm22311	13661	#N/A	1.000000E+00
Gm22312	13662	#N/A	1.000000E+00
Gm22313	13663	#N/A	1.000000E+00
Gm22314	13664	#N/A	1.000000E+00
Gm22315	13665	#N/A	1.000000E+00
Gm22316	13666	#N/A	1.000000E+00
Gm22317	13667	#N/A	1.000000E+00
Gm22318	13668	#N/A	1.000000E+00
Gm22319	13669	#N/A	1.000000E+00
Gm22320	13670	#N/A	1.000000E+00
Gm22321	13671	#N/A	1.000000E+00
Gm22322	13672	#N/A	1.000000E+00
Gm22323	13673	#N/A	1.000000E+00

Spearman Rank correlation analysis performed between Prdm1 and all-expressed genes within the Meredith RNA-seq dataset. Robust Prdm1–associated genes were identified using a cut-off of $p < 0.0005$.

Table S1, Related to Supplemental Figure 3C. Prdm1 associated genes

Gm22324	13674		#N/A	1.000000E+00
Gm22325	13675		#N/A	1.000000E+00
Gm22326	13676		#N/A	1.000000E+00
Gm22327	13677		#N/A	1.000000E+00
Gm22328	13678		#N/A	1.000000E+00
Gm22329	13679		#N/A	1.000000E+00
Gm22330	13680		#N/A	1.000000E+00
Gm22331	13681		#N/A	1.000000E+00
Gm22332	13682		#N/A	1.000000E+00
Gm22333	13683		#N/A	1.000000E+00
Gm22334	13684		#N/A	1.000000E+00
Gm22335	13685		#N/A	1.000000E+00
Gm22336	13686		#N/A	1.000000E+00
Gm22337	13687		#N/A	1.000000E+00
Gm22338	13688		#N/A	1.000000E+00
Gm22339	13689	0.113548679	13094	1.093908E-01
Gm22340	13690		#N/A	1.000000E+00
Gm22341	13691		#N/A	1.000000E+00
Gm22342	13692		#N/A	1.000000E+00
Gm22343	13693		#N/A	1.000000E+00
Gm22344	13694		#N/A	1.000000E+00
Gm22345	13695		#N/A	1.000000E+00
Gm22346	13696		#N/A	1.000000E+00
Gm22347	13697		#N/A	1.000000E+00
Gm22348	13698		#N/A	1.000000E+00
Gm22349	13699		#N/A	1.000000E+00
Gm22350	13700		#N/A	1.000000E+00
Gm22351	13701		#N/A	1.000000E+00
Gm22352	13702		#N/A	1.000000E+00
Gm22353	13703		#N/A	1.000000E+00
Gm22354	13704		#N/A	1.000000E+00
Gm22355	13705		#N/A	1.000000E+00
Gm22356	13706		#N/A	1.000000E+00
Gm22357	13707		#N/A	1.000000E+00
Gm22358	13708		#N/A	1.000000E+00
Gm22359	13709		#N/A	1.000000E+00
Gm22360	13710		#N/A	1.000000E+00
Gm22361	13711		#N/A	1.000000E+00
Gm22362	13712		#N/A	1.000000E+00
Gm22363	13713		#N/A	1.000000E+00
Gm22364	13714		#N/A	1.000000E+00
Gm22365	13715		#N/A	1.000000E+00
Gm22366	13716		#N/A	1.000000E+00

Spearman Rank correlation analysis performed between Prdm1 and all-expressed genes within the Meredith RNA-seq dataset. Robust Prdm1–associated genes were identified using a cut-off of $p < 0.0005$.

Table S1, Related to Supplemental Figure 3C. Prdm1 associated genes

Gm22367	13717		#N/A	1.000000E+00
Gm22368	13718	0.02095764	6437.5	7.683297E-01
Gm22369	13719		#N/A	1.000000E+00
Gm2237	13720	-0.017417495	4389.5	8.066150E-01
Gm22370	13721		#N/A	1.000000E+00
Gm22371	13722		#N/A	1.000000E+00
Gm22372	13723		#N/A	1.000000E+00
Gm22373	13724		#N/A	1.000000E+00
Gm22374	13725		#N/A	1.000000E+00
Gm22375	13726		#N/A	1.000000E+00
Gm22376	13727		#N/A	1.000000E+00
Gm22377	13728		#N/A	1.000000E+00
Gm22378	13729		#N/A	1.000000E+00
Gm22379	13730		#N/A	1.000000E+00
Gm22380	13731		#N/A	1.000000E+00
Gm22381	13732		#N/A	1.000000E+00
Gm22382	13733		#N/A	1.000000E+00
Gm22383	13734		#N/A	1.000000E+00
Gm22384	13735		#N/A	1.000000E+00
Gm22385	13736		#N/A	1.000000E+00
Gm22386	13737		#N/A	1.000000E+00
Gm22387	13738		#N/A	1.000000E+00
Gm22388	13739		#N/A	1.000000E+00
Gm22389	13740		#N/A	1.000000E+00
Gm22390	13741		#N/A	1.000000E+00
Gm22391	13742		#N/A	1.000000E+00
Gm22392	13743		#N/A	1.000000E+00
Gm22393	13744		#N/A	1.000000E+00
Gm22394	13745		#N/A	1.000000E+00
Gm22395	13746		#N/A	1.000000E+00
Gm22396	13747		#N/A	1.000000E+00
Gm22398	13748		#N/A	1.000000E+00
Gm22399	13749		#N/A	1.000000E+00
Gm22400	13750		#N/A	1.000000E+00
Gm22401	13751		#N/A	1.000000E+00
Gm22402	13752		#N/A	1.000000E+00
Gm22403	13753		#N/A	1.000000E+00
Gm22404	13754		#N/A	1.000000E+00
Gm22405	13755	0.162808768	16526.5	2.125512E-02
Gm22406	13756		#N/A	1.000000E+00
Gm22407	13757		#N/A	1.000000E+00
Gm22408	13758		#N/A	1.000000E+00
Gm22409	13759		#N/A	1.000000E+00

Spearman Rank correlation analysis performed between Prdm1 and all-expressed genes within the Meredith RNA-seq dataset. Robust Prdm1–associated genes were identified using a cut-off of $p < 0.0005$.

Table S1, Related to Supplemental Figure 3C. Prdm1 associated genes

Gm22410	13760		#N/A	1.000000E+00
Gm22411	13761		#N/A	1.000000E+00
Gm22412	13762		#N/A	1.000000E+00
Gm22413	13763		#N/A	1.000000E+00
Gm22414	13764		#N/A	1.000000E+00
Gm22415	13765		#N/A	1.000000E+00
Gm22416	13766		#N/A	1.000000E+00
Gm22417	13767		#N/A	1.000000E+00
Gm22418	13768	-0.038406171	3065	5.892384E-01
Gm22419	13769		#N/A	1.000000E+00
Gm22420	13770		#N/A	1.000000E+00
Gm22421	13771		#N/A	1.000000E+00
Gm22422	13772		#N/A	1.000000E+00
Gm22423	13773		#N/A	1.000000E+00
Gm22424	13774		#N/A	1.000000E+00
Gm22425	13775		#N/A	1.000000E+00
Gm22426	13776	-0.038406171	3065	5.892384E-01
Gm22427	13777		#N/A	1.000000E+00
Gm22428	13778		#N/A	1.000000E+00
Gm22429	13779		#N/A	1.000000E+00
Gm22430	13780		#N/A	1.000000E+00
Gm22431	13781		#N/A	1.000000E+00
Gm22432	13782		#N/A	1.000000E+00
Gm22433	13783		#N/A	1.000000E+00
Gm22434	13784		#N/A	1.000000E+00
Gm22435	13785		#N/A	1.000000E+00
Gm22436	13786		#N/A	1.000000E+00
Gm22437	13787		#N/A	1.000000E+00
Gm22438	13788		#N/A	1.000000E+00
Gm22439	13789		#N/A	1.000000E+00
Gm2244	13790		#N/A	1.000000E+00
Gm22440	13791		#N/A	1.000000E+00
Gm22441	13792		#N/A	1.000000E+00
Gm22442	13793		#N/A	1.000000E+00
Gm22443	13794		#N/A	1.000000E+00
Gm22444	13795		#N/A	1.000000E+00
Gm22445	13796		#N/A	1.000000E+00
Gm22446	13797		#N/A	1.000000E+00
Gm22447	13798		#N/A	1.000000E+00
Gm22448	13799		#N/A	1.000000E+00
Gm22449	13800		#N/A	1.000000E+00
Gm2245	13801	0.021317126	6513.5	7.644715E-01
Gm22450	13802		#N/A	1.000000E+00

Spearman Rank correlation analysis performed between Prdm1 and all-expressed genes within the Meredith RNA-seq dataset. Robust Prdm1–associated genes were identified using a cut-off of $p < 0.0005$.

Table S1, Related to Supplemental Figure 3C. Prdm1 associated genes

Gm22451	13803	#N/A	1.000000E+00
Gm22452	13804	#N/A	1.000000E+00
Gm22453	13805	#N/A	1.000000E+00
Gm22454	13806	#N/A	1.000000E+00
Gm22455	13807	#N/A	1.000000E+00
Gm22456	13808	#N/A	1.000000E+00
Gm22457	13809	#N/A	1.000000E+00
Gm22458	13810	#N/A	1.000000E+00
Gm22459	13811	#N/A	1.000000E+00
Gm22460	13812	#N/A	1.000000E+00
Gm22461	13813	#N/A	1.000000E+00
Gm22462	13814	#N/A	1.000000E+00
Gm22463	13815	#N/A	1.000000E+00
Gm22464	13816	#N/A	1.000000E+00
Gm22465	13817	#N/A	1.000000E+00
Gm22466	13818	#N/A	1.000000E+00
Gm22467	13819	#N/A	1.000000E+00
Gm22468	13820	#N/A	1.000000E+00
Gm22469	13821	#N/A	1.000000E+00
Gm22470	13822	#N/A	1.000000E+00
Gm22471	13823	#N/A	1.000000E+00
Gm22472	13824	#N/A	1.000000E+00
Gm22473	13825	#N/A	1.000000E+00
Gm22474	13826	#N/A	1.000000E+00
Gm22475	13827	#N/A	1.000000E+00
Gm22476	13828	#N/A	1.000000E+00
Gm22477	13829	#N/A	1.000000E+00
Gm22478	13830	#N/A	1.000000E+00
Gm22479	13831	#N/A	1.000000E+00
Gm22480	13832	#N/A	1.000000E+00
Gm22481	13833	#N/A	1.000000E+00
Gm22482	13834	#N/A	1.000000E+00
Gm22483	13835	#N/A	1.000000E+00
Gm22484	13836	#N/A	1.000000E+00
Gm22485	13837	#N/A	1.000000E+00
Gm22486	13838	#N/A	1.000000E+00
Gm22487	13839	#N/A	1.000000E+00
Gm22488	13840	#N/A	1.000000E+00
Gm22489	13841	#N/A	1.000000E+00
Gm22490	13842	#N/A	1.000000E+00
Gm22491	13843	#N/A	1.000000E+00
Gm22492	13844	#N/A	1.000000E+00
Gm22493	13845	#N/A	1.000000E+00

Spearman Rank correlation analysis performed between Prdm1 and all-expressed genes within the Meredith RNA-seq dataset. Robust Prdm1–associated genes were identified using a cut-off of $p < 0.0005$.

Gm22494	13846		#N/A	1.000000E+00
Gm22495	13847		#N/A	1.000000E+00
Gm22496	13848		#N/A	1.000000E+00
Gm22497	13849		#N/A	1.000000E+00
Gm22498	13850		#N/A	1.000000E+00
Gm22499	13851		#N/A	1.000000E+00
Gm22500	13852		#N/A	1.000000E+00
Gm22501	13853		#N/A	1.000000E+00
Gm22502	13854		#N/A	1.000000E+00
Gm22503	13855		#N/A	1.000000E+00
Gm22504	13856		#N/A	1.000000E+00
Gm22505	13857		#N/A	1.000000E+00
Gm22506	13858		#N/A	1.000000E+00
Gm22507	13859		#N/A	1.000000E+00
Gm22508	13860		#N/A	1.000000E+00
Gm22509	13861	-0.038406171	3065	5.892384E-01
Gm22510	13862		#N/A	1.000000E+00
Gm22511	13863		#N/A	1.000000E+00
Gm22512	13864		#N/A	1.000000E+00
Gm22513	13865	-0.095280123	319	1.795721E-01
Gm22514	13866	-0.038406171	3065	5.892384E-01
Gm22515	13867		#N/A	1.000000E+00
Gm22516	13868		#N/A	1.000000E+00
Gm22517	13869		#N/A	1.000000E+00
Gm22518	13870		#N/A	1.000000E+00
Gm22519	13871		#N/A	1.000000E+00
Gm22520	13872		#N/A	1.000000E+00
Gm22521	13873		#N/A	1.000000E+00
Gm22522	13874		#N/A	1.000000E+00
Gm22523	13875		#N/A	1.000000E+00
Gm22524	13876		#N/A	1.000000E+00
Gm22525	13877		#N/A	1.000000E+00
Gm22526	13878		#N/A	1.000000E+00
Gm22527	13879		#N/A	1.000000E+00
Gm22528	13880		#N/A	1.000000E+00
Gm22529	13881	-0.066858259	1029.5	3.468868E-01
Gm22530	13882		#N/A	1.000000E+00
Gm22531	13883		#N/A	1.000000E+00
Gm22532	13884		#N/A	1.000000E+00
Gm22533	13885		#N/A	1.000000E+00
Gm22534	13886		#N/A	1.000000E+00
Gm22535	13887		#N/A	1.000000E+00
Gm22536	13888		#N/A	1.000000E+00

Spearman Rank correlation analysis performed between Prdm1 and all-expressed genes within the Meredith RNA-seq dataset. Robust Prdm1–associated genes were identified using a cut-off of $p < 0.0005$.

Table S1, Related to Supplemental Figure 3C. Prdm1 associated genes

Gm22537	13889		#N/A	1.000000E+00
Gm22538	13890		#N/A	1.000000E+00
Gm22539	13891		#N/A	1.000000E+00
Gm22540	13892		#N/A	1.000000E+00
Gm22541	13893	-0.038406171	3065	5.892384E-01
Gm22542	13894		#N/A	1.000000E+00
Gm22543	13895		#N/A	1.000000E+00
Gm22544	13896		#N/A	1.000000E+00
Gm22545	13897		#N/A	1.000000E+00
Gm22546	13898		#N/A	1.000000E+00
Gm22547	13899		#N/A	1.000000E+00
Gm22548	13900		#N/A	1.000000E+00
Gm22549	13901		#N/A	1.000000E+00
Gm22550	13902		#N/A	1.000000E+00
Gm22551	13903		#N/A	1.000000E+00
Gm22552	13904		#N/A	1.000000E+00
Gm22553	13905		#N/A	1.000000E+00
Gm22554	13906		#N/A	1.000000E+00
Gm22555	13907		#N/A	1.000000E+00
Gm22556	13908		#N/A	1.000000E+00
Gm22557	13909		#N/A	1.000000E+00
Gm22558	13910		#N/A	1.000000E+00
Gm22559	13911		#N/A	1.000000E+00
Gm22560	13912		#N/A	1.000000E+00
Gm22561	13913		#N/A	1.000000E+00
Gm22562	13914		#N/A	1.000000E+00
Gm22563	13915		#N/A	1.000000E+00
Gm22564	13916		#N/A	1.000000E+00
Gm22565	13917		#N/A	1.000000E+00
Gm22566	13918		#N/A	1.000000E+00
Gm22567	13919	-0.086677076	550	2.223080E-01
Gm22568	13920		#N/A	1.000000E+00
Gm22569	13921		#N/A	1.000000E+00
Gm22570	13922		#N/A	1.000000E+00
Gm22571	13923		#N/A	1.000000E+00
Gm22572	13924		#N/A	1.000000E+00
Gm22573	13925		#N/A	1.000000E+00
Gm22574	13926		#N/A	1.000000E+00
Gm22575	13927		#N/A	1.000000E+00
Gm22576	13928		#N/A	1.000000E+00
Gm22577	13929		#N/A	1.000000E+00
Gm22578	13930		#N/A	1.000000E+00
Gm22579	13931		#N/A	1.000000E+00

Spearman Rank correlation analysis performed between Prdm1 and all-expressed genes within the Meredith RNA-seq dataset. Robust Prdm1–associated genes were identified using a cut-off of $p < 0.0005$.

Table S1, Related to Supplemental Figure 3C. Prdm1 associated genes

Gm22580	13932		#N/A	1.000000E+00
Gm22581	13933		#N/A	1.000000E+00
Gm22582	13934	0.160304018	16323	2.335876E-02
Gm22583	13935		#N/A	1.000000E+00
Gm22584	13936		#N/A	1.000000E+00
Gm22585	13937		#N/A	1.000000E+00
Gm22586	13938		#N/A	1.000000E+00
Gm22587	13939		#N/A	1.000000E+00
Gm22588	13940		#N/A	1.000000E+00
Gm22589	13941		#N/A	1.000000E+00
Gm22590	13942		#N/A	1.000000E+00
Gm22591	13943		#N/A	1.000000E+00
Gm22592	13944		#N/A	1.000000E+00
Gm22593	13945		#N/A	1.000000E+00
Gm22594	13946		#N/A	1.000000E+00
Gm22595	13947		#N/A	1.000000E+00
Gm22596	13948		#N/A	1.000000E+00
Gm22597	13949		#N/A	1.000000E+00
Gm22598	13950		#N/A	1.000000E+00
Gm22599	13951		#N/A	1.000000E+00
Gm22600	13952		#N/A	1.000000E+00
Gm22601	13953		#N/A	1.000000E+00
Gm22602	13954		#N/A	1.000000E+00
Gm22603	13955		#N/A	1.000000E+00
Gm22604	13956		#N/A	1.000000E+00
Gm22605	13957		#N/A	1.000000E+00
Gm22606	13958		#N/A	1.000000E+00
Gm22607	13959		#N/A	1.000000E+00
Gm22608	13960		#N/A	1.000000E+00
Gm22609	13961		#N/A	1.000000E+00
Gm22610	13962		#N/A	1.000000E+00
Gm22611	13963		#N/A	1.000000E+00
Gm22612	13964		#N/A	1.000000E+00
Gm22613	13965		#N/A	1.000000E+00
Gm22614	13966		#N/A	1.000000E+00
Gm22615	13967		#N/A	1.000000E+00
Gm22616	13968		#N/A	1.000000E+00
Gm22617	13969		#N/A	1.000000E+00
Gm22618	13970		#N/A	1.000000E+00
Gm22619	13971		#N/A	1.000000E+00
Gm22620	13972		#N/A	1.000000E+00
Gm22621	13973		#N/A	1.000000E+00
Gm22622	13974		#N/A	1.000000E+00

Spearman Rank correlation analysis performed between Prdm1 and all-expressed genes within the Meredith RNA-seq dataset. Robust Prdm1–associated genes were identified using a cut-off of $p < 0.0005$.

Table S1, Related to Supplemental Figure 3C. Prdm1 associated genes

Gm22623	13975		#N/A	1.000000E+00
Gm22624	13976		#N/A	1.000000E+00
Gm22625	13977		#N/A	1.000000E+00
Gm22626	13978		#N/A	1.000000E+00
Gm22627	13979		#N/A	1.000000E+00
Gm22628	13980		#N/A	1.000000E+00
Gm22629	13981		#N/A	1.000000E+00
Gm22630	13982		#N/A	1.000000E+00
Gm22631	13983		#N/A	1.000000E+00
Gm22632	13984		#N/A	1.000000E+00
Gm22633	13985		#N/A	1.000000E+00
Gm22634	13986		#N/A	1.000000E+00
Gm22635	13987		#N/A	1.000000E+00
Gm22636	13988		#N/A	1.000000E+00
Gm22637	13989		#N/A	1.000000E+00
Gm22638	13990		#N/A	1.000000E+00
Gm22639	13991		#N/A	1.000000E+00
Gm22640	13992		#N/A	1.000000E+00
Gm22641	13993	0.024986083	6818	7.254409E-01
Gm22642	13994		#N/A	1.000000E+00
Gm22643	13995		#N/A	1.000000E+00
Gm22644	13996		#N/A	1.000000E+00
Gm22645	13997	0.017609465	6208	8.045262E-01
Gm22646	13998		#N/A	1.000000E+00
Gm22647	13999		#N/A	1.000000E+00
Gm22648	14000		#N/A	1.000000E+00
Gm22649	14001	0.085741307	11039	2.273593E-01
Gm22650	14002		#N/A	1.000000E+00
Gm22651	14003		#N/A	1.000000E+00
Gm22652	14004		#N/A	1.000000E+00
Gm22654	14005		#N/A	1.000000E+00
Gm22655	14006		#N/A	1.000000E+00
Gm22656	14007	0.156129434	16056.5	2.726377E-02
Gm22657	14008		#N/A	1.000000E+00
Gm22658	14009		#N/A	1.000000E+00
Gm22659	14010		#N/A	1.000000E+00
Gm22660	14011		#N/A	1.000000E+00
Gm22661	14012		#N/A	1.000000E+00
Gm22662	14013		#N/A	1.000000E+00
Gm22663	14014		#N/A	1.000000E+00
Gm22664	14015		#N/A	1.000000E+00
Gm22665	14016		#N/A	1.000000E+00
Gm22666	14017		#N/A	1.000000E+00

Spearman Rank correlation analysis performed between Prdm1 and all-expressed genes within the Meredith RNA-seq dataset. Robust Prdm1–associated genes were identified using a cut-off of $p < 0.0005$.

Table S1, Related to Supplemental Figure 3C. Prdm1 associated genes

Gm22667	14018		#N/A	1.000000E+00
Gm22668	14019		#N/A	1.000000E+00
Gm22669	14020		#N/A	1.000000E+00
Gm22670	14021		#N/A	1.000000E+00
Gm22671	14022		#N/A	1.000000E+00
Gm22672	14023		#N/A	1.000000E+00
Gm22673	14024		#N/A	1.000000E+00
Gm22674	14025		#N/A	1.000000E+00
Gm22675	14026		#N/A	1.000000E+00
Gm22676	14027		#N/A	1.000000E+00
Gm22677	14028		#N/A	1.000000E+00
Gm22678	14029		#N/A	1.000000E+00
Gm22679	14030		#N/A	1.000000E+00
Gm22680	14031		#N/A	1.000000E+00
Gm22681	14032		#N/A	1.000000E+00
Gm22682	14033		#N/A	1.000000E+00
Gm22683	14034		#N/A	1.000000E+00
Gm22684	14035		#N/A	1.000000E+00
Gm22685	14036		#N/A	1.000000E+00
Gm22686	14037		#N/A	1.000000E+00
Gm22687	14038		#N/A	1.000000E+00
Gm22688	14039		#N/A	1.000000E+00
Gm22689	14040		#N/A	1.000000E+00
Gm22690	14041		#N/A	1.000000E+00
Gm22691	14042		#N/A	1.000000E+00
Gm22692	14043		#N/A	1.000000E+00
Gm22693	14044		#N/A	1.000000E+00
Gm22694	14045		#N/A	1.000000E+00
Gm22695	14046		#N/A	1.000000E+00
Gm22696	14047		#N/A	1.000000E+00
Gm22697	14048		#N/A	1.000000E+00
Gm22698	14049		#N/A	1.000000E+00
Gm22699	14050		#N/A	1.000000E+00
Gm22700	14051		#N/A	1.000000E+00
Gm22701	14052		#N/A	1.000000E+00
Gm22702	14053		#N/A	1.000000E+00
Gm22703	14054		#N/A	1.000000E+00
Gm22704	14055		#N/A	1.000000E+00
Gm22706	14056		#N/A	1.000000E+00
Gm22707	14057		#N/A	1.000000E+00
Gm22708	14058		#N/A	1.000000E+00
Gm22709	14059		#N/A	1.000000E+00
Gm22710	14060	-0.054450825	1984	4.437997E-01

Spearman Rank correlation analysis performed between Prdm1 and all-expressed genes within the Meredith RNA-seq dataset. Robust Prdm1–associated genes were identified using a cut-off of $p < 0.0005$.

Table S1, Related to Supplemental Figure 3C. Prdm1 associated genes

Gm22711	14061		#N/A	1.000000E+00
Gm22712	14062		#N/A	1.000000E+00
Gm22713	14063		#N/A	1.000000E+00
Gm22714	14064		#N/A	1.000000E+00
Gm22715	14065		#N/A	1.000000E+00
Gm22716	14066		#N/A	1.000000E+00
Gm22717	14067		#N/A	1.000000E+00
Gm22718	14068		#N/A	1.000000E+00
Gm22719	14069		#N/A	1.000000E+00
Gm22720	14070		#N/A	1.000000E+00
Gm22721	14071		#N/A	1.000000E+00
Gm22722	14072		#N/A	1.000000E+00
Gm22723	14073		#N/A	1.000000E+00
Gm22724	14074	-0.038406171	3065	5.892384E-01
Gm22725	14075		#N/A	1.000000E+00
Gm22726	14076		#N/A	1.000000E+00
Gm22727	14077		#N/A	1.000000E+00
Gm22728	14078		#N/A	1.000000E+00
Gm22729	14079		#N/A	1.000000E+00
Gm22730	14080		#N/A	1.000000E+00
Gm22731	14081		#N/A	1.000000E+00
Gm22732	14082		#N/A	1.000000E+00
Gm22733	14083		#N/A	1.000000E+00
Gm22734	14084		#N/A	1.000000E+00
Gm22735	14085		#N/A	1.000000E+00
Gm22736	14086		#N/A	1.000000E+00
Gm22737	14087		#N/A	1.000000E+00
Gm22738	14088		#N/A	1.000000E+00
Gm22739	14089	0.052728522	8550.5	4.583686E-01
Gm22740	14090		#N/A	1.000000E+00
Gm22741	14091		#N/A	1.000000E+00
Gm22742	14092	-0.077397959	691.5	2.760011E-01
Gm22743	14093		#N/A	1.000000E+00
Gm22744	14094		#N/A	1.000000E+00
Gm22745	14095		#N/A	1.000000E+00
Gm22746	14096		#N/A	1.000000E+00
Gm22747	14097		#N/A	1.000000E+00
Gm22748	14098		#N/A	1.000000E+00
Gm22749	14099		#N/A	1.000000E+00
Gm22750	14100		#N/A	1.000000E+00
Gm22751	14101		#N/A	1.000000E+00
Gm22752	14102		#N/A	1.000000E+00
Gm22753	14103		#N/A	1.000000E+00

Spearman Rank correlation analysis performed between Prdm1 and all-expressed genes within the Meredith RNA-seq dataset. Robust Prdm1–associated genes were identified using a cut-off of $p < 0.0005$.

Gm22754	14104	#N/A	1.000000E+00
Gm22755	14105	#N/A	1.000000E+00
Gm22756	14106	#N/A	1.000000E+00
Gm22757	14107	#N/A	1.000000E+00
Gm22758	14108	#N/A	1.000000E+00
Gm22759	14109	#N/A	1.000000E+00
Gm22760	14110	#N/A	1.000000E+00
Gm22761	14111	#N/A	1.000000E+00
Gm22762	14112	#N/A	1.000000E+00
Gm22763	14113	#N/A	1.000000E+00
Gm22764	14114	#N/A	1.000000E+00
Gm22765	14115	#N/A	1.000000E+00
Gm22766	14116	#N/A	1.000000E+00
Gm22767	14117	#N/A	1.000000E+00
Gm22768	14118	#N/A	1.000000E+00
Gm22769	14119	#N/A	1.000000E+00
Gm22770	14120	#N/A	1.000000E+00
Gm22771	14121	#N/A	1.000000E+00
Gm22772	14122	#N/A	1.000000E+00
Gm22773	14123	#N/A	1.000000E+00
Gm22774	14124	#N/A	1.000000E+00
Gm22775	14125	#N/A	1.000000E+00
Gm22776	14126	#N/A	1.000000E+00
Gm22777	14127	#N/A	1.000000E+00
Gm22778	14128	#N/A	1.000000E+00
Gm22779	14129	#N/A	1.000000E+00
Gm22780	14130	#N/A	1.000000E+00
Gm22781	14131	#N/A	1.000000E+00
Gm22782	14132	#N/A	1.000000E+00
Gm22783	14133	#N/A	1.000000E+00
Gm22784	14134	#N/A	1.000000E+00
Gm22785	14135	#N/A	1.000000E+00
Gm22786	14136	#N/A	1.000000E+00
Gm22787	14137	#N/A	1.000000E+00
Gm22788	14138	#N/A	1.000000E+00
Gm22789	14139	#N/A	1.000000E+00
Gm22790	14140	#N/A	1.000000E+00
Gm22791	14141	#N/A	1.000000E+00
Gm22792	14142	#N/A	1.000000E+00
Gm22793	14143	#N/A	1.000000E+00
Gm22794	14144	#N/A	1.000000E+00
Gm22795	14145	#N/A	1.000000E+00
Gm22796	14146	#N/A	1.000000E+00

Spearman Rank correlation analysis performed between Prdm1 and all-expressed genes within the Meredith RNA-seq dataset. Robust Prdm1–associated genes were identified using a cut-off of $p < 0.0005$.

Gm22797	14147	#N/A	1.000000E+00
Gm22798	14148	#N/A	1.000000E+00
Gm22799	14149	#N/A	1.000000E+00
Gm22800	14150	#N/A	1.000000E+00
Gm22801	14151	#N/A	1.000000E+00
Gm22802	14152	#N/A	1.000000E+00
Gm22803	14153	#N/A	1.000000E+00
Gm22804	14154	#N/A	1.000000E+00
Gm22805	14155	#N/A	1.000000E+00
Gm22806	14156	#N/A	1.000000E+00
Gm22807	14157	#N/A	1.000000E+00
Gm22808	14158	#N/A	1.000000E+00
Gm22809	14159	#N/A	1.000000E+00
Gm22810	14160	#N/A	1.000000E+00
Gm22811	14161	#N/A	1.000000E+00
Gm22812	14162	#N/A	1.000000E+00
Gm22813	14163	#N/A	1.000000E+00
Gm22814	14164	#N/A	1.000000E+00
Gm22815	14165	#N/A	1.000000E+00
Gm22816	14166	#N/A	1.000000E+00
Gm22817	14167	#N/A	1.000000E+00
Gm22818	14168	#N/A	1.000000E+00
Gm22819	14169	#N/A	1.000000E+00
Gm22820	14170	#N/A	1.000000E+00
Gm22821	14171	#N/A	1.000000E+00
Gm22822	14172	#N/A	1.000000E+00
Gm22823	14173	#N/A	1.000000E+00
Gm22824	14174	#N/A	1.000000E+00
Gm22825	14175	#N/A	1.000000E+00
Gm22826	14176	#N/A	1.000000E+00
Gm22827	14177	#N/A	1.000000E+00
Gm22828	14178	#N/A	1.000000E+00
Gm22829	14179	#N/A	1.000000E+00
Gm22830	14180	#N/A	1.000000E+00
Gm22831	14181	#N/A	1.000000E+00
Gm22832	14182	#N/A	1.000000E+00
Gm22833	14183	#N/A	1.000000E+00
Gm22834	14184	#N/A	1.000000E+00
Gm22835	14185	#N/A	1.000000E+00
Gm22836	14186	#N/A	1.000000E+00
Gm22837	14187	#N/A	1.000000E+00
Gm22838	14188	#N/A	1.000000E+00
Gm22839	14189	#N/A	1.000000E+00

Gm22840	14190		#N/A	1.000000E+00
Gm22841	14191		#N/A	1.000000E+00
Gm22842	14192		#N/A	1.000000E+00
Gm22843	14193		#N/A	1.000000E+00
Gm22844	14194		#N/A	1.000000E+00
Gm22845	14195		#N/A	1.000000E+00
Gm22846	14196		#N/A	1.000000E+00
Gm22847	14197		#N/A	1.000000E+00
Gm22848	14198		#N/A	1.000000E+00
Gm22849	14199		#N/A	1.000000E+00
Gm22850	14200		#N/A	1.000000E+00
Gm22851	14201		#N/A	1.000000E+00
Gm22852	14202		#N/A	1.000000E+00
Gm22853	14203		#N/A	1.000000E+00
Gm22854	14204		#N/A	1.000000E+00
Gm22855	14205		#N/A	1.000000E+00
Gm22856	14206		#N/A	1.000000E+00
Gm22857	14207		#N/A	1.000000E+00
Gm22858	14208		#N/A	1.000000E+00
Gm22859	14209		#N/A	1.000000E+00
Gm22860	14210		#N/A	1.000000E+00
Gm22861	14211		#N/A	1.000000E+00
Gm22862	14212		#N/A	1.000000E+00
Gm22863	14213		#N/A	1.000000E+00
Gm22864	14214		#N/A	1.000000E+00
Gm22865	14215		#N/A	1.000000E+00
Gm22866	14216		#N/A	1.000000E+00
Gm22867	14217		#N/A	1.000000E+00
Gm22868	14218		#N/A	1.000000E+00
Gm22869	14219		#N/A	1.000000E+00
Gm22870	14220		#N/A	1.000000E+00
Gm22871	14221		#N/A	1.000000E+00
Gm22872	14222		#N/A	1.000000E+00
Gm22873	14223		#N/A	1.000000E+00
Gm22874	14224		#N/A	1.000000E+00
Gm22875	14225		#N/A	1.000000E+00
Gm22876	14226	-0.038406171	3065	5.892384E-01
Gm22877	14227		#N/A	1.000000E+00
Gm22878	14228		#N/A	1.000000E+00
Gm22879	14229		#N/A	1.000000E+00
Gm2288	14230		#N/A	1.000000E+00
Gm22880	14231		#N/A	1.000000E+00
Gm22881	14232		#N/A	1.000000E+00

Spearman Rank correlation analysis performed between Prdm1 and all-expressed genes within the Meredith RNA-seq dataset. Robust Prdm1–associated genes were identified using a cut-off of $p < 0.0005$.

Gm22882	14233		#N/A	1.000000E+00
Gm22883	14234		#N/A	1.000000E+00
Gm22884	14235		#N/A	1.000000E+00
Gm22885	14236		#N/A	1.000000E+00
Gm22886	14237		#N/A	1.000000E+00
Gm22887	14238		#N/A	1.000000E+00
Gm22888	14239		#N/A	1.000000E+00
Gm22889	14240		#N/A	1.000000E+00
Gm22890	14241		#N/A	1.000000E+00
Gm22891	14242		#N/A	1.000000E+00
Gm22892	14243		#N/A	1.000000E+00
Gm22893	14244		#N/A	1.000000E+00
Gm22894	14245		#N/A	1.000000E+00
Gm22895	14246		#N/A	1.000000E+00
Gm22896	14247		#N/A	1.000000E+00
Gm22897	14248		#N/A	1.000000E+00
Gm22898	14249		#N/A	1.000000E+00
Gm22899	14250		#N/A	1.000000E+00
Gm22900	14251		#N/A	1.000000E+00
Gm22901	14252		#N/A	1.000000E+00
Gm22902	14253		#N/A	1.000000E+00
Gm22903	14254		#N/A	1.000000E+00
Gm22904	14255		#N/A	1.000000E+00
Gm22905	14256		#N/A	1.000000E+00
Gm22906	14257		#N/A	1.000000E+00
Gm22907	14258		#N/A	1.000000E+00
Gm22908	14259		#N/A	1.000000E+00
Gm22909	14260		#N/A	1.000000E+00
Gm22910	14261	0.134200998	14569.5	5.814916E-02
Gm22911	14262		#N/A	1.000000E+00
Gm22912	14263		#N/A	1.000000E+00
Gm22913	14264		#N/A	1.000000E+00
Gm22914	14265		#N/A	1.000000E+00
Gm22915	14266		#N/A	1.000000E+00
Gm22916	14267		#N/A	1.000000E+00
Gm22917	14268		#N/A	1.000000E+00
Gm22918	14269		#N/A	1.000000E+00
Gm22919	14270		#N/A	1.000000E+00
Gm22920	14271		#N/A	1.000000E+00
Gm22921	14272	-0.054451513	1703	4.437940E-01
Gm22922	14273		#N/A	1.000000E+00
Gm22923	14274		#N/A	1.000000E+00
Gm22924	14275		#N/A	1.000000E+00

Spearman Rank correlation analysis performed between Prdm1 and all-expressed genes within the Meredith RNA-seq dataset. Robust Prdm1–associated genes were identified using a cut-off of $p < 0.0005$.

Table S1, Related to Supplemental Figure 3C. Prdm1 associated genes

Gm22925	14276		#N/A	1.000000E+00
Gm22926	14277		#N/A	1.000000E+00
Gm22927	14278		#N/A	1.000000E+00
Gm22928	14279		#N/A	1.000000E+00
Gm22929	14280		#N/A	1.000000E+00
Gm22930	14281		#N/A	1.000000E+00
Gm22931	14282		#N/A	1.000000E+00
Gm22932	14283		#N/A	1.000000E+00
Gm22933	14284		#N/A	1.000000E+00
Gm22934	14285		#N/A	1.000000E+00
Gm22935	14286		#N/A	1.000000E+00
Gm22936	14287		#N/A	1.000000E+00
Gm22937	14288		#N/A	1.000000E+00
Gm22938	14289		#N/A	1.000000E+00
Gm22939	14290		#N/A	1.000000E+00
Gm22940	14291		#N/A	1.000000E+00
Gm22941	14292		#N/A	1.000000E+00
Gm22942	14293		#N/A	1.000000E+00
Gm22943	14294		#N/A	1.000000E+00
Gm22944	14295		#N/A	1.000000E+00
Gm22945	14296		#N/A	1.000000E+00
Gm22946	14297		#N/A	1.000000E+00
Gm22947	14298		#N/A	1.000000E+00
Gm22948	14299		#N/A	1.000000E+00
Gm22949	14300		#N/A	1.000000E+00
Gm22950	14301		#N/A	1.000000E+00
Gm22951	14302		#N/A	1.000000E+00
Gm22952	14303		#N/A	1.000000E+00
Gm22953	14304		#N/A	1.000000E+00
Gm22954	14305		#N/A	1.000000E+00
Gm22955	14306		#N/A	1.000000E+00
Gm22956	14307		#N/A	1.000000E+00
Gm22957	14308		#N/A	1.000000E+00
Gm22958	14309		#N/A	1.000000E+00
Gm22959	14310		#N/A	1.000000E+00
Gm22960	14311		#N/A	1.000000E+00
Gm22961	14312		#N/A	1.000000E+00
Gm22962	14313	-0.038406171	3065	5.892384E-01
Gm22963	14314		#N/A	1.000000E+00
Gm22964	14315		#N/A	1.000000E+00
Gm22965	14316		#N/A	1.000000E+00
Gm22966	14317		#N/A	1.000000E+00
Gm22967	14318		#N/A	1.000000E+00

Spearman Rank correlation analysis performed between Prdm1 and all-expressed genes within the Meredith RNA-seq dataset. Robust Prdm1–associated genes were identified using a cut-off of $p < 0.0005$.

Table S1, Related to Supplemental Figure 3C. Prdm1 associated genes

Gm22968	14319		#N/A	1.000000E+00
Gm22969	14320		#N/A	1.000000E+00
Gm22970	14321		#N/A	1.000000E+00
Gm22971	14322		#N/A	1.000000E+00
Gm22972	14323		#N/A	1.000000E+00
Gm22973	14324		#N/A	1.000000E+00
Gm22974	14325		#N/A	1.000000E+00
Gm22975	14326		#N/A	1.000000E+00
Gm22976	14327		#N/A	1.000000E+00
Gm22977	14328		#N/A	1.000000E+00
Gm22978	14329		#N/A	1.000000E+00
Gm22979	14330		#N/A	1.000000E+00
Gm22980	14331		#N/A	1.000000E+00
Gm22981	14332		#N/A	1.000000E+00
Gm22982	14333		#N/A	1.000000E+00
Gm22983	14334		#N/A	1.000000E+00
Gm22984	14335	0.113548679	13094	1.093908E-01
Gm22985	14336		#N/A	1.000000E+00
Gm22986	14337		#N/A	1.000000E+00
Gm22987	14338		#N/A	1.000000E+00
Gm22988	14339		#N/A	1.000000E+00
Gm22989	14340		#N/A	1.000000E+00
Gm22990	14341		#N/A	1.000000E+00
Gm22991	14342		#N/A	1.000000E+00
Gm22992	14343		#N/A	1.000000E+00
Gm22993	14344		#N/A	1.000000E+00
Gm22994	14345		#N/A	1.000000E+00
Gm22995	14346		#N/A	1.000000E+00
Gm22996	14347		#N/A	1.000000E+00
Gm22997	14348		#N/A	1.000000E+00
Gm22998	14349		#N/A	1.000000E+00
Gm22999	14350		#N/A	1.000000E+00
Gm23000	14351		#N/A	1.000000E+00
Gm23001	14352		#N/A	1.000000E+00
Gm23002	14353		#N/A	1.000000E+00
Gm23003	14354		#N/A	1.000000E+00
Gm23004	14355		#N/A	1.000000E+00
Gm23005	14356		#N/A	1.000000E+00
Gm23006	14357		#N/A	1.000000E+00
Gm23007	14358		#N/A	1.000000E+00
Gm23008	14359		#N/A	1.000000E+00
Gm23009	14360		#N/A	1.000000E+00
Gm2301	14361	0.0515562	8431	4.684355E-01

Spearman Rank correlation analysis performed between Prdm1 and all-expressed genes within the Meredith RNA-seq dataset. Robust Prdm1–associated genes were identified using a cut-off of $p < 0.0005$.

Table S1, Related to Supplemental Figure 3C. Prdm1 associated genes

Gm23010	14362	#N/A	1.000000E+00
Gm23011	14363	#N/A	1.000000E+00
Gm23012	14364	#N/A	1.000000E+00
Gm23013	14365	#N/A	1.000000E+00
Gm23014	14366	#N/A	1.000000E+00
Gm23015	14367	#N/A	1.000000E+00
Gm23016	14368	#N/A	1.000000E+00
Gm23017	14369	#N/A	1.000000E+00
Gm23018	14370	#N/A	1.000000E+00
Gm23019	14371	#N/A	1.000000E+00
Gm2302	14372	#N/A	1.000000E+00
Gm23020	14373	#N/A	1.000000E+00
Gm23021	14374	#N/A	1.000000E+00
Gm23022	14375	#N/A	1.000000E+00
Gm23023	14376	#N/A	1.000000E+00
Gm23024	14377	#N/A	1.000000E+00
Gm23025	14378	#N/A	1.000000E+00
Gm23026	14379	#N/A	1.000000E+00
Gm23027	14380	#N/A	1.000000E+00
Gm23028	14381	#N/A	1.000000E+00
Gm23029	14382	#N/A	1.000000E+00
Gm23030	14383	#N/A	1.000000E+00
Gm23031	14384	#N/A	1.000000E+00
Gm23032	14385	#N/A	1.000000E+00
Gm23033	14386	#N/A	1.000000E+00
Gm23034	14387	#N/A	1.000000E+00
Gm23035	14388	#N/A	1.000000E+00
Gm23036	14389	#N/A	1.000000E+00
Gm23037	14390	#N/A	1.000000E+00
Gm23038	14391	#N/A	1.000000E+00
Gm23039	14392	#N/A	1.000000E+00
Gm23040	14393	#N/A	1.000000E+00
Gm23041	14394	#N/A	1.000000E+00
Gm23042	14395	#N/A	1.000000E+00
Gm23043	14396	#N/A	1.000000E+00
Gm23044	14397	#N/A	1.000000E+00
Gm23045	14398	#N/A	1.000000E+00
Gm23046	14399	#N/A	1.000000E+00
Gm23047	14400	#N/A	1.000000E+00
Gm23048	14401	#N/A	1.000000E+00
Gm23049	14402	#N/A	1.000000E+00
Gm23050	14403	#N/A	1.000000E+00
Gm23051	14404	#N/A	1.000000E+00

Spearman Rank correlation analysis performed between Prdm1 and all-expressed genes within the Meredith RNA-seq dataset. Robust Prdm1–associated genes were identified using a cut-off of $p < 0.0005$.

Table S1, Related to Supplemental Figure 3C. Prdm1 associated genes

Gm23052	14405		#N/A	1.000000E+00
Gm23053	14406		#N/A	1.000000E+00
Gm23054	14407		#N/A	1.000000E+00
Gm23055	14408		#N/A	1.000000E+00
Gm23056	14409		#N/A	1.000000E+00
Gm23057	14410		#N/A	1.000000E+00
Gm23058	14411		#N/A	1.000000E+00
Gm23059	14412		#N/A	1.000000E+00
Gm23060	14413		#N/A	1.000000E+00
Gm23061	14414	0.074699258	10224	2.931415E-01
Gm23062	14415		#N/A	1.000000E+00
Gm23063	14416		#N/A	1.000000E+00
Gm23064	14417		#N/A	1.000000E+00
Gm23065	14418		#N/A	1.000000E+00
Gm23066	14419		#N/A	1.000000E+00
Gm23067	14420		#N/A	1.000000E+00
Gm23068	14421		#N/A	1.000000E+00
Gm23069	14422		#N/A	1.000000E+00
Gm23070	14423		#N/A	1.000000E+00
Gm23071	14424		#N/A	1.000000E+00
Gm23072	14425		#N/A	1.000000E+00
Gm23073	14426		#N/A	1.000000E+00
Gm23074	14427		#N/A	1.000000E+00
Gm23075	14428		#N/A	1.000000E+00
Gm23076	14429		#N/A	1.000000E+00
Gm23077	14430		#N/A	1.000000E+00
Gm23078	14431		#N/A	1.000000E+00
Gm23079	14432		#N/A	1.000000E+00
Gm2308	14433		#N/A	1.000000E+00
Gm23080	14434		#N/A	1.000000E+00
Gm23081	14435		#N/A	1.000000E+00
Gm23082	14436		#N/A	1.000000E+00
Gm23083	14437		#N/A	1.000000E+00
Gm23084	14438		#N/A	1.000000E+00
Gm23085	14439		#N/A	1.000000E+00
Gm23086	14440		#N/A	1.000000E+00
Gm23087	14441		#N/A	1.000000E+00
Gm23088	14442		#N/A	1.000000E+00
Gm23089	14443		#N/A	1.000000E+00
Gm2309	14444		#N/A	1.000000E+00
Gm23090	14445		#N/A	1.000000E+00
Gm23092	14446	-0.038406171	3065	5.892384E-01
Gm23093	14447		#N/A	1.000000E+00

Spearman Rank correlation analysis performed between Prdm1 and all-expressed genes within the Meredith RNA-seq dataset. Robust Prdm1–associated genes were identified using a cut-off of $p < 0.0005$.

Table S1, Related to Supplemental Figure 3C. Prdm1 associated genes

Gm23094	14448		#N/A	1.000000E+00
Gm23095	14449		#N/A	1.000000E+00
Gm23096	14450		#N/A	1.000000E+00
Gm23097	14451		#N/A	1.000000E+00
Gm23098	14452		#N/A	1.000000E+00
Gm23099	14453		#N/A	1.000000E+00
Gm2310	14454		#N/A	1.000000E+00
Gm23100	14455	-0.038406171	3065	5.892384E-01
Gm23101	14456		#N/A	1.000000E+00
Gm23102	14457		#N/A	1.000000E+00
Gm23103	14458		#N/A	1.000000E+00
Gm23104	14459		#N/A	1.000000E+00
Gm23105	14460		#N/A	1.000000E+00
Gm23106	14461		#N/A	1.000000E+00
Gm23108	14462		#N/A	1.000000E+00
Gm23109	14463		#N/A	1.000000E+00
Gm23110	14464		#N/A	1.000000E+00
Gm23111	14465		#N/A	1.000000E+00
Gm23112	14466		#N/A	1.000000E+00
Gm23113	14467		#N/A	1.000000E+00
Gm23114	14468		#N/A	1.000000E+00
Gm23115	14469		#N/A	1.000000E+00
Gm23116	14470		#N/A	1.000000E+00
Gm23117	14471		#N/A	1.000000E+00
Gm23118	14472		#N/A	1.000000E+00
Gm23119	14473		#N/A	1.000000E+00
Gm23120	14474		#N/A	1.000000E+00
Gm23121	14475		#N/A	1.000000E+00
Gm23122	14476		#N/A	1.000000E+00
Gm23123	14477		#N/A	1.000000E+00
Gm23124	14478		#N/A	1.000000E+00
Gm23125	14479		#N/A	1.000000E+00
Gm23126	14480		#N/A	1.000000E+00
Gm23127	14481		#N/A	1.000000E+00
Gm23128	14482		#N/A	1.000000E+00
Gm23129	14483		#N/A	1.000000E+00
Gm23130	14484		#N/A	1.000000E+00
Gm23131	14485		#N/A	1.000000E+00
Gm23132	14486		#N/A	1.000000E+00
Gm23133	14487		#N/A	1.000000E+00
Gm23134	14488		#N/A	1.000000E+00
Gm23135	14489		#N/A	1.000000E+00
Gm23136	14490		#N/A	1.000000E+00

Spearman Rank correlation analysis performed between Prdm1 and all-expressed genes within the Meredith RNA-seq dataset. Robust Prdm1–associated genes were identified using a cut-off of $p < 0.0005$.

Table S1, Related to Supplemental Figure 3C. Prdm1 associated genes

Gm23137	14491		#N/A	1.000000E+00
Gm23138	14492		#N/A	1.000000E+00
Gm23139	14493		#N/A	1.000000E+00
Gm23140	14494		#N/A	1.000000E+00
Gm23141	14495		#N/A	1.000000E+00
Gm23142	14496		#N/A	1.000000E+00
Gm23143	14497		#N/A	1.000000E+00
Gm23144	14498		#N/A	1.000000E+00
Gm23145	14499		#N/A	1.000000E+00
Gm23146	14500		#N/A	1.000000E+00
Gm23147	14501		#N/A	1.000000E+00
Gm23148	14502		#N/A	1.000000E+00
Gm23149	14503		#N/A	1.000000E+00
Gm23150	14504		#N/A	1.000000E+00
Gm23151	14505		#N/A	1.000000E+00
Gm23152	14506		#N/A	1.000000E+00
Gm23153	14507	0.106151421	12436	1.346463E-01
Gm23154	14508		#N/A	1.000000E+00
Gm23155	14509		#N/A	1.000000E+00
Gm23156	14510		#N/A	1.000000E+00
Gm23157	14511		#N/A	1.000000E+00
Gm23158	14512		#N/A	1.000000E+00
Gm23159	14513		#N/A	1.000000E+00
Gm23160	14514		#N/A	1.000000E+00
Gm23161	14515		#N/A	1.000000E+00
Gm23162	14516		#N/A	1.000000E+00
Gm23163	14517		#N/A	1.000000E+00
Gm23164	14518		#N/A	1.000000E+00
Gm23165	14519		#N/A	1.000000E+00
Gm23166	14520		#N/A	1.000000E+00
Gm23167	14521		#N/A	1.000000E+00
Gm23168	14522		#N/A	1.000000E+00
Gm23169	14523		#N/A	1.000000E+00
Gm23170	14524		#N/A	1.000000E+00
Gm23171	14525		#N/A	1.000000E+00
Gm23172	14526		#N/A	1.000000E+00
Gm23173	14527		#N/A	1.000000E+00
Gm23174	14528		#N/A	1.000000E+00
Gm23175	14529		#N/A	1.000000E+00
Gm23176	14530		#N/A	1.000000E+00
Gm23177	14531		#N/A	1.000000E+00
Gm23178	14532		#N/A	1.000000E+00
Gm23179	14533		#N/A	1.000000E+00

Spearman Rank correlation analysis performed between Prdm1 and all-expressed genes within the Meredith RNA-seq dataset. Robust Prdm1–associated genes were identified using a cut-off of $p < 0.0005$.

Table S1, Related to Supplemental Figure 3C. Prdm1 associated genes

Gm2318	14534		#N/A	1.000000E+00
Gm23180	14535		#N/A	1.000000E+00
Gm23181	14536		#N/A	1.000000E+00
Gm23182	14537		#N/A	1.000000E+00
Gm23183	14538		#N/A	1.000000E+00
Gm23184	14539		#N/A	1.000000E+00
Gm23185	14540		#N/A	1.000000E+00
Gm23186	14541		#N/A	1.000000E+00
Gm23187	14542		#N/A	1.000000E+00
Gm23188	14543		#N/A	1.000000E+00
Gm23189	14544		#N/A	1.000000E+00
Gm23190	14545		#N/A	1.000000E+00
Gm23191	14546		#N/A	1.000000E+00
Gm23192	14547		#N/A	1.000000E+00
Gm23193	14548	-0.054451513	1703	4.437940E-01
Gm23194	14549		#N/A	1.000000E+00
Gm23195	14550		#N/A	1.000000E+00
Gm23196	14551		#N/A	1.000000E+00
Gm23197	14552		#N/A	1.000000E+00
Gm23198	14553		#N/A	1.000000E+00
Gm23199	14554		#N/A	1.000000E+00
Gm23200	14555		#N/A	1.000000E+00
Gm23201	14556		#N/A	1.000000E+00
Gm23202	14557		#N/A	1.000000E+00
Gm23203	14558		#N/A	1.000000E+00
Gm23204	14559		#N/A	1.000000E+00
Gm23205	14560		#N/A	1.000000E+00
Gm23206	14561		#N/A	1.000000E+00
Gm23207	14562		#N/A	1.000000E+00
Gm23208	14563		#N/A	1.000000E+00
Gm23209	14564		#N/A	1.000000E+00
Gm23210	14565		#N/A	1.000000E+00
Gm23211	14566		#N/A	1.000000E+00
Gm23212	14567		#N/A	1.000000E+00
Gm23213	14568		#N/A	1.000000E+00
Gm23214	14569		#N/A	1.000000E+00
Gm23215	14570	-0.038406171	3065	5.892384E-01
Gm23216	14571		#N/A	1.000000E+00
Gm23217	14572		#N/A	1.000000E+00
Gm23218	14573		#N/A	1.000000E+00
Gm23219	14574		#N/A	1.000000E+00
Gm23220	14575		#N/A	1.000000E+00
Gm23221	14576		#N/A	1.000000E+00

Spearman Rank correlation analysis performed between Prdm1 and all-expressed genes within the Meredith RNA-seq dataset. Robust Prdm1–associated genes were identified using a cut-off of $p < 0.0005$.

Table S1, Related to Supplemental Figure 3C. Prdm1 associated genes

Gm23222	14577	#N/A	1.000000E+00
Gm23223	14578	#N/A	1.000000E+00
Gm23224	14579	#N/A	1.000000E+00
Gm23225	14580	#N/A	1.000000E+00
Gm23226	14581	#N/A	1.000000E+00
Gm23227	14582	#N/A	1.000000E+00
Gm23228	14583	#N/A	1.000000E+00
Gm23229	14584	#N/A	1.000000E+00
Gm23230	14585	#N/A	1.000000E+00
Gm23231	14586	#N/A	1.000000E+00
Gm23232	14587	#N/A	1.000000E+00
Gm23233	14588	#N/A	1.000000E+00
Gm23234	14589	#N/A	1.000000E+00
Gm23235	14590	#N/A	1.000000E+00
Gm23236	14591	#N/A	1.000000E+00
Gm23237	14592	#N/A	1.000000E+00
Gm23238	14593	#N/A	1.000000E+00
Gm23239	14594	#N/A	1.000000E+00
Gm23240	14595	#N/A	1.000000E+00
Gm23241	14596	#N/A	1.000000E+00
Gm23242	14597	#N/A	1.000000E+00
Gm23243	14598	#N/A	1.000000E+00
Gm23244	14599	#N/A	1.000000E+00
Gm23245	14600	#N/A	1.000000E+00
Gm23246	14601	#N/A	1.000000E+00
Gm23247	14602	#N/A	1.000000E+00
Gm23248	14603	#N/A	1.000000E+00
Gm23249	14604	#N/A	1.000000E+00
Gm23250	14605	#N/A	1.000000E+00
Gm23251	14606	#N/A	1.000000E+00
Gm23252	14607	#N/A	1.000000E+00
Gm23253	14608	#N/A	1.000000E+00
Gm23254	14609	#N/A	1.000000E+00
Gm23255	14610	#N/A	1.000000E+00
Gm23256	14611	#N/A	1.000000E+00
Gm23257	14612	#N/A	1.000000E+00
Gm23258	14613	#N/A	1.000000E+00
Gm23259	14614	#N/A	1.000000E+00
Gm23260	14615	#N/A	1.000000E+00
Gm23261	14616	#N/A	1.000000E+00
Gm23262	14617	#N/A	1.000000E+00
Gm23263	14618	#N/A	1.000000E+00
Gm23264	14619	#N/A	1.000000E+00

Spearman Rank correlation analysis performed between Prdm1 and all-expressed genes within the Meredith RNA-seq dataset. Robust Prdm1–associated genes were identified using a cut-off of $p < 0.0005$.

Table S1, Related to Supplemental Figure 3C. Prdm1 associated genes

Gm23265	14620	#N/A	1.000000E+00
Gm23266	14621	#N/A	1.000000E+00
Gm23267	14622	#N/A	1.000000E+00
Gm23268	14623	#N/A	1.000000E+00
Gm23269	14624	#N/A	1.000000E+00
Gm23270	14625	#N/A	1.000000E+00
Gm23271	14626	#N/A	1.000000E+00
Gm23272	14627	#N/A	1.000000E+00
Gm23273	14628	#N/A	1.000000E+00
Gm23274	14629	#N/A	1.000000E+00
Gm23275	14630	#N/A	1.000000E+00
Gm23276	14631	#N/A	1.000000E+00
Gm23277	14632	#N/A	1.000000E+00
Gm23278	14633	#N/A	1.000000E+00
Gm23279	14634	#N/A	1.000000E+00
Gm23280	14635	#N/A	1.000000E+00
Gm23281	14636	#N/A	1.000000E+00
Gm23282	14637	#N/A	1.000000E+00
Gm23283	14638	#N/A	1.000000E+00
Gm23284	14639	#N/A	1.000000E+00
Gm23285	14640	#N/A	1.000000E+00
Gm23286	14641	#N/A	1.000000E+00
Gm23287	14642	#N/A	1.000000E+00
Gm23288	14643	#N/A	1.000000E+00
Gm23289	14644	#N/A	1.000000E+00
Gm23290	14645	#N/A	1.000000E+00
Gm23291	14646	#N/A	1.000000E+00
Gm23292	14647	#N/A	1.000000E+00
Gm23293	14648	#N/A	1.000000E+00
Gm23294	14649	#N/A	1.000000E+00
Gm23295	14650	#N/A	1.000000E+00
Gm23296	14651	#N/A	1.000000E+00
Gm23297	14652	#N/A	1.000000E+00
Gm23298	14653	#N/A	1.000000E+00
Gm23299	14654	#N/A	1.000000E+00
Gm23300	14655	#N/A	1.000000E+00
Gm23301	14656	#N/A	1.000000E+00
Gm23302	14657	#N/A	1.000000E+00
Gm23303	14658	#N/A	1.000000E+00
Gm23304	14659	#N/A	1.000000E+00
Gm23305	14660	#N/A	1.000000E+00
Gm23306	14661	#N/A	1.000000E+00
Gm23307	14662	#N/A	1.000000E+00

Spearman Rank correlation analysis performed between Prdm1 and all-expressed genes within the Meredith RNA-seq dataset. Robust Prdm1–associated genes were identified using a cut-off of $p < 0.0005$.

Table S1, Related to Supplemental Figure 3C. Prdm1 associated genes

Gm23308	14663	#N/A	1.000000E+00
Gm23309	14664	#N/A	1.000000E+00
Gm23310	14665	#N/A	1.000000E+00
Gm23311	14666	#N/A	1.000000E+00
Gm23312	14667	#N/A	1.000000E+00
Gm23313	14668	#N/A	1.000000E+00
Gm23314	14669	#N/A	1.000000E+00
Gm23315	14670	#N/A	1.000000E+00
Gm23316	14671	#N/A	1.000000E+00
Gm23317	14672	#N/A	1.000000E+00
Gm23318	14673	#N/A	1.000000E+00
Gm23319	14674	#N/A	1.000000E+00
Gm23320	14675	#N/A	1.000000E+00
Gm23321	14676	#N/A	1.000000E+00
Gm23322	14677	#N/A	1.000000E+00
Gm23323	14678	#N/A	1.000000E+00
Gm23324	14679	#N/A	1.000000E+00
Gm23325	14680	#N/A	1.000000E+00
Gm23326	14681	#N/A	1.000000E+00
Gm23327	14682	#N/A	1.000000E+00
Gm23328	14683	#N/A	1.000000E+00
Gm23329	14684	#N/A	1.000000E+00
Gm23330	14685	#N/A	1.000000E+00
Gm23331	14686	#N/A	1.000000E+00
Gm23332	14687	#N/A	1.000000E+00
Gm23333	14688	#N/A	1.000000E+00
Gm23334	14689	#N/A	1.000000E+00
Gm23335	14690	#N/A	1.000000E+00
Gm23336	14691	#N/A	1.000000E+00
Gm23337	14692	#N/A	1.000000E+00
Gm23338	14693	#N/A	1.000000E+00
Gm23339	14694	#N/A	1.000000E+00
Gm23340	14695	#N/A	1.000000E+00
Gm23341	14696	#N/A	1.000000E+00
Gm23342	14697	#N/A	1.000000E+00
Gm23343	14698	#N/A	1.000000E+00
Gm23344	14699	#N/A	1.000000E+00
Gm23345	14700	#N/A	1.000000E+00
Gm23346	14701	#N/A	1.000000E+00
Gm23347	14702	#N/A	1.000000E+00
Gm23348	14703	#N/A	1.000000E+00
Gm23349	14704	#N/A	1.000000E+00
Gm23350	14705	#N/A	1.000000E+00

Spearman Rank correlation analysis performed between Prdm1 and all-expressed genes within the Meredith RNA-seq dataset. Robust Prdm1–associated genes were identified using a cut-off of $p < 0.0005$.

Table S1, Related to Supplemental Figure 3C. Prdm1 associated genes

Gm23351	14706	#N/A	1.000000E+00
Gm23352	14707	#N/A	1.000000E+00
Gm23353	14708	#N/A	1.000000E+00
Gm23354	14709	#N/A	1.000000E+00
Gm23355	14710	#N/A	1.000000E+00
Gm23356	14711	#N/A	1.000000E+00
Gm23357	14712	#N/A	1.000000E+00
Gm23358	14713	#N/A	1.000000E+00
Gm23359	14714	#N/A	1.000000E+00
Gm23360	14715	#N/A	1.000000E+00
Gm23361	14716	#N/A	1.000000E+00
Gm23362	14717	#N/A	1.000000E+00
Gm23363	14718	#N/A	1.000000E+00
Gm23364	14719	#N/A	1.000000E+00
Gm23365	14720	#N/A	1.000000E+00
Gm23366	14721	#N/A	1.000000E+00
Gm23367	14722	#N/A	1.000000E+00
Gm23368	14723	#N/A	1.000000E+00
Gm23369	14724	#N/A	1.000000E+00
Gm23370	14725	#N/A	1.000000E+00
Gm23371	14726	#N/A	1.000000E+00
Gm23372	14727	#N/A	1.000000E+00
Gm23373	14728	#N/A	1.000000E+00
Gm23374	14729	#N/A	1.000000E+00
Gm23375	14730	#N/A	1.000000E+00
Gm23376	14731	#N/A	1.000000E+00
Gm23377	14732	#N/A	1.000000E+00
Gm23378	14733	#N/A	1.000000E+00
Gm23379	14734	#N/A	1.000000E+00
Gm23380	14735	#N/A	1.000000E+00
Gm23381	14736	#N/A	1.000000E+00
Gm23382	14737	#N/A	1.000000E+00
Gm23383	14738	#N/A	1.000000E+00
Gm23384	14739	#N/A	1.000000E+00
Gm23385	14740	#N/A	1.000000E+00
Gm23386	14741	#N/A	1.000000E+00
Gm23387	14742	#N/A	1.000000E+00
Gm23388	14743	#N/A	1.000000E+00
Gm23389	14744	#N/A	1.000000E+00
Gm23390	14745	#N/A	1.000000E+00
Gm23391	14746	#N/A	1.000000E+00
Gm23392	14747	#N/A	1.000000E+00
Gm23393	14748	#N/A	1.000000E+00

Spearman Rank correlation analysis performed between Prdm1 and all-expressed genes within the Meredith RNA-seq dataset. Robust Prdm1–associated genes were identified using a cut-off of $p < 0.0005$.

Table S1, Related to Supplemental Figure 3C. Prdm1 associated genes

Gm23394	14749		#N/A	1.000000E+00
Gm23395	14750		#N/A	1.000000E+00
Gm23396	14751		#N/A	1.000000E+00
Gm23397	14752		#N/A	1.000000E+00
Gm23398	14753		#N/A	1.000000E+00
Gm23399	14754		#N/A	1.000000E+00
Gm23400	14755		#N/A	1.000000E+00
Gm23401	14756		#N/A	1.000000E+00
Gm23402	14757		#N/A	1.000000E+00
Gm23403	14758		#N/A	1.000000E+00
Gm23404	14759	0.166148435	16721.5	1.870631E-02
Gm23405	14760		#N/A	1.000000E+00
Gm23406	14761		#N/A	1.000000E+00
Gm23407	14762		#N/A	1.000000E+00
Gm23408	14763		#N/A	1.000000E+00
Gm23409	14764		#N/A	1.000000E+00
Gm23410	14765		#N/A	1.000000E+00
Gm23411	14766		#N/A	1.000000E+00
Gm23412	14767		#N/A	1.000000E+00
Gm23413	14768		#N/A	1.000000E+00
Gm23414	14769		#N/A	1.000000E+00
Gm23415	14770		#N/A	1.000000E+00
Gm23416	14771		#N/A	1.000000E+00
Gm23417	14772		#N/A	1.000000E+00
Gm23418	14773		#N/A	1.000000E+00
Gm23419	14774		#N/A	1.000000E+00
Gm23420	14775		#N/A	1.000000E+00
Gm23421	14776		#N/A	1.000000E+00
Gm23422	14777		#N/A	1.000000E+00
Gm23423	14778		#N/A	1.000000E+00
Gm23424	14779		#N/A	1.000000E+00
Gm23425	14780		#N/A	1.000000E+00
Gm23426	14781		#N/A	1.000000E+00
Gm23427	14782		#N/A	1.000000E+00
Gm23428	14783		#N/A	1.000000E+00
Gm23429	14784		#N/A	1.000000E+00
Gm2343	14785		#N/A	1.000000E+00
Gm23430	14786		#N/A	1.000000E+00
Gm23431	14787		#N/A	1.000000E+00
Gm23432	14788		#N/A	1.000000E+00
Gm23433	14789		#N/A	1.000000E+00
Gm23434	14790		#N/A	1.000000E+00
Gm23435	14791		#N/A	1.000000E+00

Spearman Rank correlation analysis performed between Prdm1 and all-expressed genes within the Meredith RNA-seq dataset. Robust Prdm1–associated genes were identified using a cut-off of $p < 0.0005$.

Table S1, Related to Supplemental Figure 3C. Prdm1 associated genes

Gm23436	14792		#N/A	1.000000E+00
Gm23437	14793		#N/A	1.000000E+00
Gm23438	14794		#N/A	1.000000E+00
Gm23439	14795		#N/A	1.000000E+00
Gm23440	14796		#N/A	1.000000E+00
Gm23441	14797		#N/A	1.000000E+00
Gm23442	14798		#N/A	1.000000E+00
Gm23443	14799		#N/A	1.000000E+00
Gm23444	14800		#N/A	1.000000E+00
Gm23445	14801		#N/A	1.000000E+00
Gm23446	14802		#N/A	1.000000E+00
Gm23447	14803		#N/A	1.000000E+00
Gm23448	14804		#N/A	1.000000E+00
Gm23449	14805		#N/A	1.000000E+00
Gm23450	14806		#N/A	1.000000E+00
Gm23451	14807		#N/A	1.000000E+00
Gm23452	14808		#N/A	1.000000E+00
Gm23453	14809		#N/A	1.000000E+00
Gm23454	14810		#N/A	1.000000E+00
Gm23455	14811		#N/A	1.000000E+00
Gm23456	14812		#N/A	1.000000E+00
Gm23457	14813		#N/A	1.000000E+00
Gm23458	14814		#N/A	1.000000E+00
Gm23459	14815		#N/A	1.000000E+00
Gm23460	14816		#N/A	1.000000E+00
Gm23461	14817		#N/A	1.000000E+00
Gm23462	14818		#N/A	1.000000E+00
Gm23463	14819		#N/A	1.000000E+00
Gm23464	14820		#N/A	1.000000E+00
Gm23465	14821		#N/A	1.000000E+00
Gm23466	14822		#N/A	1.000000E+00
Gm23467	14823		#N/A	1.000000E+00
Gm23468	14824		#N/A	1.000000E+00
Gm23469	14825		#N/A	1.000000E+00
Gm23470	14826	0.07521908	10268	2.897860E-01
Gm23471	14827		#N/A	1.000000E+00
Gm23472	14828		#N/A	1.000000E+00
Gm23473	14829		#N/A	1.000000E+00
Gm23474	14830		#N/A	1.000000E+00
Gm23475	14831		#N/A	1.000000E+00
Gm23476	14832		#N/A	1.000000E+00
Gm23477	14833		#N/A	1.000000E+00
Gm23478	14834		#N/A	1.000000E+00

Spearman Rank correlation analysis performed between Prdm1 and all-expressed genes within the Meredith RNA-seq dataset. Robust Prdm1–associated genes were identified using a cut-off of $p < 0.0005$.

Table S1, Related to Supplemental Figure 3C. Prdm1 associated genes

Gm23479	14835		#N/A	1.000000E+00
Gm2348	14836		#N/A	1.000000E+00
Gm23480	14837		#N/A	1.000000E+00
Gm23481	14838	-0.038406171	3065	5.892384E-01
Gm23482	14839		#N/A	1.000000E+00
Gm23483	14840		#N/A	1.000000E+00
Gm23484	14841		#N/A	1.000000E+00
Gm23485	14842		#N/A	1.000000E+00
Gm23486	14843		#N/A	1.000000E+00
Gm23487	14844		#N/A	1.000000E+00
Gm23488	14845		#N/A	1.000000E+00
Gm23489	14846		#N/A	1.000000E+00
Gm23490	14847		#N/A	1.000000E+00
Gm23491	14848		#N/A	1.000000E+00
Gm23492	14849		#N/A	1.000000E+00
Gm23493	14850	0.044242998	7881	5.338936E-01
Gm23494	14851		#N/A	1.000000E+00
Gm23495	14852		#N/A	1.000000E+00
Gm23496	14853		#N/A	1.000000E+00
Gm23497	14854		#N/A	1.000000E+00
Gm23498	14855		#N/A	1.000000E+00
Gm23499	14856		#N/A	1.000000E+00
Gm2350	14857	0.071855013	9986	3.119583E-01
Gm23500	14858		#N/A	1.000000E+00
Gm23501	14859		#N/A	1.000000E+00
Gm23502	14860		#N/A	1.000000E+00
Gm23503	14861		#N/A	1.000000E+00
Gm23504	14862		#N/A	1.000000E+00
Gm23505	14863		#N/A	1.000000E+00
Gm23506	14864		#N/A	1.000000E+00
Gm23507	14865	-0.038406171	3065	5.892384E-01
Gm23508	14866		#N/A	1.000000E+00
Gm23509	14867		#N/A	1.000000E+00
Gm23510	14868		#N/A	1.000000E+00
Gm23511	14869		#N/A	1.000000E+00
Gm23512	14870	-0.054450825	1984	4.437997E-01
Gm23513	14871		#N/A	1.000000E+00
Gm23514	14872		#N/A	1.000000E+00
Gm23515	14873		#N/A	1.000000E+00
Gm23516	14874		#N/A	1.000000E+00
Gm23517	14875		#N/A	1.000000E+00
Gm23518	14876		#N/A	1.000000E+00
Gm23519	14877		#N/A	1.000000E+00

Spearman Rank correlation analysis performed between Prdm1 and all-expressed genes within the Meredith RNA-seq dataset. Robust Prdm1–associated genes were identified using a cut-off of $p < 0.0005$.

Gm23520	14878	#N/A	1.000000E+00
Gm23521	14879	#N/A	1.000000E+00
Gm23522	14880	#N/A	1.000000E+00
Gm23523	14881	#N/A	1.000000E+00
Gm23524	14882	#N/A	1.000000E+00
Gm23525	14883	#N/A	1.000000E+00
Gm23526	14884	#N/A	1.000000E+00
Gm23527	14885	#N/A	1.000000E+00
Gm23528	14886	#N/A	1.000000E+00
Gm23529	14887	#N/A	1.000000E+00
Gm23530	14888	#N/A	1.000000E+00
Gm23531	14889	#N/A	1.000000E+00
Gm23532	14890	#N/A	1.000000E+00
Gm23533	14891	#N/A	1.000000E+00
Gm23534	14892	#N/A	1.000000E+00
Gm23535	14893	#N/A	1.000000E+00
Gm23536	14894	#N/A	1.000000E+00
Gm23537	14895	#N/A	1.000000E+00
Gm23538	14896	#N/A	1.000000E+00
Gm23539	14897	#N/A	1.000000E+00
Gm23540	14898	#N/A	1.000000E+00
Gm23541	14899	#N/A	1.000000E+00
Gm23542	14900	#N/A	1.000000E+00
Gm23543	14901	#N/A	1.000000E+00
Gm23544	14902	#N/A	1.000000E+00
Gm23545	14903	#N/A	1.000000E+00
Gm23546	14904	#N/A	1.000000E+00
Gm23547	14905	#N/A	1.000000E+00
Gm23548	14906	#N/A	1.000000E+00
Gm23549	14907	#N/A	1.000000E+00
Gm23550	14908	#N/A	1.000000E+00
Gm23551	14909	#N/A	1.000000E+00
Gm23552	14910	#N/A	1.000000E+00
Gm23553	14911	#N/A	1.000000E+00
Gm23554	14912	#N/A	1.000000E+00
Gm23555	14913	#N/A	1.000000E+00
Gm23556	14914	#N/A	1.000000E+00
Gm23557	14915	#N/A	1.000000E+00
Gm23558	14916	#N/A	1.000000E+00
Gm23559	14917	#N/A	1.000000E+00
Gm23560	14918	#N/A	1.000000E+00
Gm23561	14919	#N/A	1.000000E+00
Gm23562	14920	#N/A	1.000000E+00

Table S1, Related to Supplemental Figure 3C. Prdm1 associated genes

Gm23563	14921	#N/A	1.000000E+00
Gm23564	14922	#N/A	1.000000E+00
Gm23565	14923	#N/A	1.000000E+00
Gm23566	14924	#N/A	1.000000E+00
Gm23567	14925	#N/A	1.000000E+00
Gm23568	14926	#N/A	1.000000E+00
Gm23569	14927	#N/A	1.000000E+00
Gm23570	14928	#N/A	1.000000E+00
Gm23571	14929	#N/A	1.000000E+00
Gm23572	14930	#N/A	1.000000E+00
Gm23573	14931	#N/A	1.000000E+00
Gm23574	14932	#N/A	1.000000E+00
Gm23575	14933	#N/A	1.000000E+00
Gm23576	14934	#N/A	1.000000E+00
Gm23577	14935	#N/A	1.000000E+00
Gm23578	14936	#N/A	1.000000E+00
Gm23579	14937	#N/A	1.000000E+00
Gm23580	14938	#N/A	1.000000E+00
Gm23581	14939	#N/A	1.000000E+00
Gm23582	14940	#N/A	1.000000E+00
Gm23583	14941	#N/A	1.000000E+00
Gm23584	14942	#N/A	1.000000E+00
Gm23585	14943	#N/A	1.000000E+00
Gm23586	14944	#N/A	1.000000E+00
Gm23587	14945	#N/A	1.000000E+00
Gm23588	14946	#N/A	1.000000E+00
Gm23589	14947	#N/A	1.000000E+00
Gm23590	14948	#N/A	1.000000E+00
Gm23591	14949	#N/A	1.000000E+00
Gm23592	14950	#N/A	1.000000E+00
Gm23593	14951	#N/A	1.000000E+00
Gm23594	14952	#N/A	1.000000E+00
Gm23595	14953	#N/A	1.000000E+00
Gm23596	14954	#N/A	1.000000E+00
Gm23597	14955	#N/A	1.000000E+00
Gm23598	14956	#N/A	1.000000E+00
Gm23599	14957	#N/A	1.000000E+00
Gm23600	14958	#N/A	1.000000E+00
Gm23601	14959	#N/A	1.000000E+00
Gm23602	14960	#N/A	1.000000E+00
Gm23603	14961	#N/A	1.000000E+00
Gm23604	14962	#N/A	1.000000E+00
Gm23605	14963	#N/A	1.000000E+00

Spearman Rank correlation analysis performed between Prdm1 and all-expressed genes within the Meredith RNA-seq dataset. Robust Prdm1–associated genes were identified using a cut-off of $p < 0.0005$.

Table S1, Related to Supplemental Figure 3C. Prdm1 associated genes

Gm23606	14964		#N/A	1.000000E+00
Gm23607	14965		#N/A	1.000000E+00
Gm23608	14966		#N/A	1.000000E+00
Gm23609	14967		#N/A	1.000000E+00
Gm23610	14968		#N/A	1.000000E+00
Gm23611	14969		#N/A	1.000000E+00
Gm23612	14970		#N/A	1.000000E+00
Gm23613	14971		#N/A	1.000000E+00
Gm23614	14972		#N/A	1.000000E+00
Gm23615	14973		#N/A	1.000000E+00
Gm23616	14974		#N/A	1.000000E+00
Gm23617	14975		#N/A	1.000000E+00
Gm23618	14976		#N/A	1.000000E+00
Gm23619	14977		#N/A	1.000000E+00
Gm23620	14978		#N/A	1.000000E+00
Gm23621	14979		#N/A	1.000000E+00
Gm23622	14980	-0.054451513	1703	4.437940E-01
Gm23623	14981		#N/A	1.000000E+00
Gm23624	14982		#N/A	1.000000E+00
Gm23625	14983		#N/A	1.000000E+00
Gm23626	14984		#N/A	1.000000E+00
Gm23627	14985		#N/A	1.000000E+00
Gm23628	14986		#N/A	1.000000E+00
Gm23629	14987		#N/A	1.000000E+00
Gm23630	14988		#N/A	1.000000E+00
Gm23631	14989		#N/A	1.000000E+00
Gm23632	14990		#N/A	1.000000E+00
Gm23633	14991		#N/A	1.000000E+00
Gm23634	14992		#N/A	1.000000E+00
Gm23635	14993		#N/A	1.000000E+00
Gm23636	14994		#N/A	1.000000E+00
Gm23637	14995		#N/A	1.000000E+00
Gm23638	14996		#N/A	1.000000E+00
Gm23639	14997		#N/A	1.000000E+00
Gm23640	14998		#N/A	1.000000E+00
Gm23641	14999		#N/A	1.000000E+00
Gm23642	15000		#N/A	1.000000E+00
Gm23643	15001		#N/A	1.000000E+00
Gm23644	15002		#N/A	1.000000E+00
Gm23645	15003		#N/A	1.000000E+00
Gm23646	15004		#N/A	1.000000E+00
Gm23647	15005		#N/A	1.000000E+00
Gm23648	15006		#N/A	1.000000E+00

Spearman Rank correlation analysis performed between Prdm1 and all-expressed genes within the Meredith RNA-seq dataset. Robust Prdm1–associated genes were identified using a cut-off of $p < 0.0005$.

Gm23649	15007	#N/A	1.000000E+00
Gm23650	15008	#N/A	1.000000E+00
Gm23651	15009	#N/A	1.000000E+00
Gm23652	15010	#N/A	1.000000E+00
Gm23653	15011	#N/A	1.000000E+00
Gm23654	15012	#N/A	1.000000E+00
Gm23655	15013	#N/A	1.000000E+00
Gm23656	15014	#N/A	1.000000E+00
Gm23657	15015	#N/A	1.000000E+00
Gm23658	15016	#N/A	1.000000E+00
Gm23659	15017	#N/A	1.000000E+00
Gm23660	15018	#N/A	1.000000E+00
Gm23661	15019	#N/A	1.000000E+00
Gm23662	15020	#N/A	1.000000E+00
Gm23663	15021	#N/A	1.000000E+00
Gm23664	15022	#N/A	1.000000E+00
Gm23665	15023	#N/A	1.000000E+00
Gm23666	15024	#N/A	1.000000E+00
Gm23667	15025	#N/A	1.000000E+00
Gm23668	15026	#N/A	1.000000E+00
Gm23669	15027	#N/A	1.000000E+00
Gm23670	15028	#N/A	1.000000E+00
Gm23671	15029	#N/A	1.000000E+00
Gm23672	15030	#N/A	1.000000E+00
Gm23673	15031	#N/A	1.000000E+00
Gm23674	15032	#N/A	1.000000E+00
Gm23675	15033	#N/A	1.000000E+00
Gm23676	15034	#N/A	1.000000E+00
Gm23677	15035	#N/A	1.000000E+00
Gm23678	15036	#N/A	1.000000E+00
Gm23679	15037	#N/A	1.000000E+00
Gm23680	15038	#N/A	1.000000E+00
Gm23681	15039	#N/A	1.000000E+00
Gm23682	15040	#N/A	1.000000E+00
Gm23683	15041	#N/A	1.000000E+00
Gm23684	15042	#N/A	1.000000E+00
Gm23685	15043	#N/A	1.000000E+00
Gm23686	15044	#N/A	1.000000E+00
Gm23687	15045	#N/A	1.000000E+00
Gm23688	15046	#N/A	1.000000E+00
Gm23689	15047	#N/A	1.000000E+00
Gm23690	15048	#N/A	1.000000E+00
Gm23691	15049	#N/A	1.000000E+00

Table S1, Related to Supplemental Figure 3C. Prdm1 associated genes

Gm23692	15050		#N/A	1.000000E+00
Gm23693	15051		#N/A	1.000000E+00
Gm23694	15052		#N/A	1.000000E+00
Gm23695	15053		#N/A	1.000000E+00
Gm23696	15054		#N/A	1.000000E+00
Gm23697	15055		#N/A	1.000000E+00
Gm23698	15056		#N/A	1.000000E+00
Gm23699	15057		#N/A	1.000000E+00
Gm23700	15058		#N/A	1.000000E+00
Gm23701	15059		#N/A	1.000000E+00
Gm23702	15060		#N/A	1.000000E+00
Gm23703	15061		#N/A	1.000000E+00
Gm23704	15062		#N/A	1.000000E+00
Gm23705	15063		#N/A	1.000000E+00
Gm23706	15064		#N/A	1.000000E+00
Gm23707	15065		#N/A	1.000000E+00
Gm23708	15066		#N/A	1.000000E+00
Gm23709	15067		#N/A	1.000000E+00
Gm23711	15068		#N/A	1.000000E+00
Gm23712	15069		#N/A	1.000000E+00
Gm23713	15070		#N/A	1.000000E+00
Gm23714	15071		#N/A	1.000000E+00
Gm23715	15072		#N/A	1.000000E+00
Gm23716	15073		#N/A	1.000000E+00
Gm23717	15074		#N/A	1.000000E+00
Gm23718	15075		#N/A	1.000000E+00
Gm23719	15076		#N/A	1.000000E+00
Gm23720	15077		#N/A	1.000000E+00
Gm23721	15078		#N/A	1.000000E+00
Gm23722	15079		#N/A	1.000000E+00
Gm23723	15080		#N/A	1.000000E+00
Gm23724	15081		#N/A	1.000000E+00
Gm23725	15082		#N/A	1.000000E+00
Gm23726	15083		#N/A	1.000000E+00
Gm23727	15084		#N/A	1.000000E+00
Gm23728	15085		#N/A	1.000000E+00
Gm23729	15086		#N/A	1.000000E+00
Gm2373	15087	-0.016731055	4437	8.140953E-01
Gm23730	15088		#N/A	1.000000E+00
Gm23731	15089		#N/A	1.000000E+00
Gm23732	15090		#N/A	1.000000E+00
Gm23733	15091		#N/A	1.000000E+00
Gm23734	15092		#N/A	1.000000E+00

Spearman Rank correlation analysis performed between Prdm1 and all-expressed genes within the Meredith RNA-seq dataset. Robust Prdm1–associated genes were identified using a cut-off of $p < 0.0005$.

Table S1, Related to Supplemental Figure 3C. Prdm1 associated genes

Gm23735	15093	#N/A	1.000000E+00
Gm23736	15094	#N/A	1.000000E+00
Gm23737	15095	#N/A	1.000000E+00
Gm23738	15096	#N/A	1.000000E+00
Gm23739	15097	#N/A	1.000000E+00
Gm23740	15098	#N/A	1.000000E+00
Gm23741	15099	#N/A	1.000000E+00
Gm23742	15100	#N/A	1.000000E+00
Gm23743	15101	#N/A	1.000000E+00
Gm23744	15102	#N/A	1.000000E+00
Gm23745	15103	#N/A	1.000000E+00
Gm23746	15104	#N/A	1.000000E+00
Gm23747	15105	#N/A	1.000000E+00
Gm23748	15106	#N/A	1.000000E+00
Gm23749	15107	#N/A	1.000000E+00
Gm23750	15108	#N/A	1.000000E+00
Gm23751	15109	#N/A	1.000000E+00
Gm23752	15110	#N/A	1.000000E+00
Gm23753	15111	#N/A	1.000000E+00
Gm23754	15112	#N/A	1.000000E+00
Gm23755	15113	#N/A	1.000000E+00
Gm23756	15114	#N/A	1.000000E+00
Gm23757	15115	#N/A	1.000000E+00
Gm23758	15116	#N/A	1.000000E+00
Gm23759	15117	#N/A	1.000000E+00
Gm23760	15118	#N/A	1.000000E+00
Gm23761	15119	#N/A	1.000000E+00
Gm23762	15120	#N/A	1.000000E+00
Gm23763	15121	#N/A	1.000000E+00
Gm23764	15122	#N/A	1.000000E+00
Gm23765	15123	#N/A	1.000000E+00
Gm23766	15124	#N/A	1.000000E+00
Gm23767	15125	#N/A	1.000000E+00
Gm23768	15126	#N/A	1.000000E+00
Gm23769	15127	#N/A	1.000000E+00
Gm23770	15128	#N/A	1.000000E+00
Gm23771	15129	#N/A	1.000000E+00
Gm23772	15130	#N/A	1.000000E+00
Gm23773	15131	#N/A	1.000000E+00
Gm23774	15132	#N/A	1.000000E+00
Gm23775	15133	#N/A	1.000000E+00
Gm23776	15134	#N/A	1.000000E+00
Gm23777	15135	#N/A	1.000000E+00

Spearman Rank correlation analysis performed between Prdm1 and all-expressed genes within the Meredith RNA-seq dataset. Robust Prdm1–associated genes were identified using a cut-off of $p < 0.0005$.

Table S1, Related to Supplemental Figure 3C. Prdm1 associated genes

Gm23778	15136		#N/A	1.000000E+00
Gm23779	15137		#N/A	1.000000E+00
Gm23780	15138		#N/A	1.000000E+00
Gm23781	15139		#N/A	1.000000E+00
Gm23782	15140		#N/A	1.000000E+00
Gm23783	15141		#N/A	1.000000E+00
Gm23784	15142		#N/A	1.000000E+00
Gm23785	15143		#N/A	1.000000E+00
Gm23786	15144		#N/A	1.000000E+00
Gm23787	15145		#N/A	1.000000E+00
Gm23788	15146		#N/A	1.000000E+00
Gm23789	15147		#N/A	1.000000E+00
Gm23790	15148		#N/A	1.000000E+00
Gm23791	15149		#N/A	1.000000E+00
Gm23792	15150		#N/A	1.000000E+00
Gm23793	15151		#N/A	1.000000E+00
Gm23794	15152		#N/A	1.000000E+00
Gm23795	15153		#N/A	1.000000E+00
Gm23796	15154		#N/A	1.000000E+00
Gm23797	15155		#N/A	1.000000E+00
Gm23798	15156		#N/A	1.000000E+00
Gm23799	15157		#N/A	1.000000E+00
Gm23800	15158		#N/A	1.000000E+00
Gm23801	15159		#N/A	1.000000E+00
Gm23802	15160		#N/A	1.000000E+00
Gm23803	15161	-0.038406171	3065	5.892384E-01
Gm23804	15162		#N/A	1.000000E+00
Gm23805	15163		#N/A	1.000000E+00
Gm23806	15164		#N/A	1.000000E+00
Gm23807	15165		#N/A	1.000000E+00
Gm23808	15166		#N/A	1.000000E+00
Gm23809	15167	0.05392473	8859	4.482219E-01
Gm2381	15168	0.099271295	12017.5	1.619434E-01
Gm23810	15169		#N/A	1.000000E+00
Gm23811	15170		#N/A	1.000000E+00
Gm23812	15171		#N/A	1.000000E+00
Gm23813	15172		#N/A	1.000000E+00
Gm23814	15173		#N/A	1.000000E+00
Gm23815	15174		#N/A	1.000000E+00
Gm23816	15175		#N/A	1.000000E+00
Gm23817	15176		#N/A	1.000000E+00
Gm23818	15177		#N/A	1.000000E+00
Gm23819	15178		#N/A	1.000000E+00

Spearman Rank correlation analysis performed between Prdm1 and all-expressed genes within the Meredith RNA-seq dataset. Robust Prdm1–associated genes were identified using a cut-off of $p < 0.0005$.

Table S1, Related to Supplemental Figure 3C. Prdm1 associated genes

Gm2382	15179	0.091775499	11506	1.961855E-01
Gm23820	15180		#N/A	1.000000E+00
Gm23821	15181		#N/A	1.000000E+00
Gm23822	15182		#N/A	1.000000E+00
Gm23823	15183		#N/A	1.000000E+00
Gm23824	15184		#N/A	1.000000E+00
Gm23825	15185		#N/A	1.000000E+00
Gm23826	15186		#N/A	1.000000E+00
Gm23827	15187		#N/A	1.000000E+00
Gm23828	15188		#N/A	1.000000E+00
Gm23829	15189		#N/A	1.000000E+00
Gm23830	15190		#N/A	1.000000E+00
Gm23831	15191		#N/A	1.000000E+00
Gm23832	15192		#N/A	1.000000E+00
Gm23833	15193		#N/A	1.000000E+00
Gm23834	15194		#N/A	1.000000E+00
Gm23835	15195		#N/A	1.000000E+00
Gm23836	15196		#N/A	1.000000E+00
Gm23837	15197		#N/A	1.000000E+00
Gm23838	15198		#N/A	1.000000E+00
Gm23839	15199		#N/A	1.000000E+00
Gm23840	15200	0.158529356	16203	2.495566E-02
Gm23841	15201		#N/A	1.000000E+00
Gm23842	15202		#N/A	1.000000E+00
Gm23843	15203		#N/A	1.000000E+00
Gm23844	15204		#N/A	1.000000E+00
Gm23845	15205		#N/A	1.000000E+00
Gm23846	15206		#N/A	1.000000E+00
Gm23847	15207		#N/A	1.000000E+00
Gm23848	15208		#N/A	1.000000E+00
Gm23849	15209		#N/A	1.000000E+00
Gm23850	15210		#N/A	1.000000E+00
Gm23851	15211		#N/A	1.000000E+00
Gm23852	15212		#N/A	1.000000E+00
Gm23853	15213		#N/A	1.000000E+00
Gm23854	15214		#N/A	1.000000E+00
Gm23855	15215		#N/A	1.000000E+00
Gm23856	15216		#N/A	1.000000E+00
Gm23857	15217		#N/A	1.000000E+00
Gm23858	15218		#N/A	1.000000E+00
Gm23859	15219		#N/A	1.000000E+00
Gm23860	15220		#N/A	1.000000E+00
Gm23861	15221		#N/A	1.000000E+00

Spearman Rank correlation analysis performed between Prdm1 and all-expressed genes within the Meredith RNA-seq dataset. Robust Prdm1–associated genes were identified using a cut-off of $p < 0.0005$.

Table S1, Related to Supplemental Figure 3C. Prdm1 associated genes

Gm23862	15222		#N/A	1.000000E+00
Gm23863	15223		#N/A	1.000000E+00
Gm23864	15224		#N/A	1.000000E+00
Gm23865	15225		#N/A	1.000000E+00
Gm23866	15226		#N/A	1.000000E+00
Gm23867	15227		#N/A	1.000000E+00
Gm23868	15228		#N/A	1.000000E+00
Gm23869	15229		#N/A	1.000000E+00
Gm23870	15230		#N/A	1.000000E+00
Gm23871	15231	-0.038406171	3065	5.892384E-01
Gm23872	15232		#N/A	1.000000E+00
Gm23873	15233		#N/A	1.000000E+00
Gm23874	15234		#N/A	1.000000E+00
Gm23875	15235		#N/A	1.000000E+00
Gm23876	15236		#N/A	1.000000E+00
Gm23877	15237		#N/A	1.000000E+00
Gm23878	15238		#N/A	1.000000E+00
Gm23879	15239		#N/A	1.000000E+00
Gm23880	15240		#N/A	1.000000E+00
Gm23881	15241		#N/A	1.000000E+00
Gm23882	15242		#N/A	1.000000E+00
Gm23883	15243		#N/A	1.000000E+00
Gm23884	15244		#N/A	1.000000E+00
Gm23885	15245		#N/A	1.000000E+00
Gm23886	15246		#N/A	1.000000E+00
Gm23887	15247		#N/A	1.000000E+00
Gm23888	15248		#N/A	1.000000E+00
Gm23889	15249		#N/A	1.000000E+00
Gm2389	15250	0.152789767	15822	3.077826E-02
Gm23890	15251		#N/A	1.000000E+00
Gm23891	15252		#N/A	1.000000E+00
Gm23892	15253		#N/A	1.000000E+00
Gm23893	15254		#N/A	1.000000E+00
Gm23894	15255		#N/A	1.000000E+00
Gm23895	15256		#N/A	1.000000E+00
Gm23896	15257		#N/A	1.000000E+00
Gm23897	15258		#N/A	1.000000E+00
Gm23898	15259		#N/A	1.000000E+00
Gm23899	15260		#N/A	1.000000E+00
Gm23900	15261		#N/A	1.000000E+00
Gm23901	15262		#N/A	1.000000E+00
Gm23902	15263		#N/A	1.000000E+00
Gm23903	15264		#N/A	1.000000E+00

Spearman Rank correlation analysis performed between Prdm1 and all-expressed genes within the Meredith RNA-seq dataset. Robust Prdm1–associated genes were identified using a cut-off of $p < 0.0005$.

Table S1, Related to Supplemental Figure 3C. Prdm1 associated genes

Gm23904	15265		#N/A	1.000000E+00
Gm23905	15266		#N/A	1.000000E+00
Gm23906	15267		#N/A	1.000000E+00
Gm23907	15268		#N/A	1.000000E+00
Gm23908	15269		#N/A	1.000000E+00
Gm23909	15270		#N/A	1.000000E+00
Gm23910	15271		#N/A	1.000000E+00
Gm23911	15272		#N/A	1.000000E+00
Gm23912	15273		#N/A	1.000000E+00
Gm23913	15274		#N/A	1.000000E+00
Gm23914	15275		#N/A	1.000000E+00
Gm23915	15276		#N/A	1.000000E+00
Gm23916	15277		#N/A	1.000000E+00
Gm23917	15278		#N/A	1.000000E+00
Gm23918	15279		#N/A	1.000000E+00
Gm23919	15280		#N/A	1.000000E+00
Gm23920	15281		#N/A	1.000000E+00
Gm23921	15282		#N/A	1.000000E+00
Gm23922	15283		#N/A	1.000000E+00
Gm23923	15284		#N/A	1.000000E+00
Gm23924	15285		#N/A	1.000000E+00
Gm23925	15286		#N/A	1.000000E+00
Gm23926	15287		#N/A	1.000000E+00
Gm23927	15288	-0.077394997	736.5	2.760195E-01
Gm23928	15289		#N/A	1.000000E+00
Gm23929	15290		#N/A	1.000000E+00
Gm23930	15291		#N/A	1.000000E+00
Gm23931	15292		#N/A	1.000000E+00
Gm23932	15293		#N/A	1.000000E+00
Gm23933	15294		#N/A	1.000000E+00
Gm23934	15295		#N/A	1.000000E+00
Gm23935	15296	0.137767657	14808	5.172648E-02
Gm23936	15297		#N/A	1.000000E+00
Gm23937	15298		#N/A	1.000000E+00
Gm23938	15299		#N/A	1.000000E+00
Gm23939	15300		#N/A	1.000000E+00
Gm23940	15301		#N/A	1.000000E+00
Gm23941	15302		#N/A	1.000000E+00
Gm23942	15303		#N/A	1.000000E+00
Gm23943	15304		#N/A	1.000000E+00
Gm23944	15305		#N/A	1.000000E+00
Gm23945	15306		#N/A	1.000000E+00
Gm23946	15307		#N/A	1.000000E+00

Spearman Rank correlation analysis performed between Prdm1 and all-expressed genes within the Meredith RNA-seq dataset. Robust Prdm1–associated genes were identified using a cut-off of $p < 0.0005$.

Gm23947	15308	#N/A	1.000000E+00
Gm23948	15309	#N/A	1.000000E+00
Gm23949	15310	#N/A	1.000000E+00
Gm23950	15311	#N/A	1.000000E+00
Gm23951	15312	#N/A	1.000000E+00
Gm23952	15313	#N/A	1.000000E+00
Gm23953	15314	#N/A	1.000000E+00
Gm23954	15315	#N/A	1.000000E+00
Gm23955	15316	#N/A	1.000000E+00
Gm23956	15317	#N/A	1.000000E+00
Gm23957	15318	#N/A	1.000000E+00
Gm23958	15319	#N/A	1.000000E+00
Gm23959	15320	#N/A	1.000000E+00
Gm23960	15321	#N/A	1.000000E+00
Gm23961	15322	#N/A	1.000000E+00
Gm23962	15323	#N/A	1.000000E+00
Gm23963	15324	#N/A	1.000000E+00
Gm23964	15325	#N/A	1.000000E+00
Gm23965	15326	#N/A	1.000000E+00
Gm23966	15327	#N/A	1.000000E+00
Gm23967	15328	#N/A	1.000000E+00
Gm23968	15329	#N/A	1.000000E+00
Gm23969	15330	#N/A	1.000000E+00
Gm23970	15331	#N/A	1.000000E+00
Gm23971	15332	#N/A	1.000000E+00
Gm23972	15333	#N/A	1.000000E+00
Gm23973	15334	#N/A	1.000000E+00
Gm23974	15335	#N/A	1.000000E+00
Gm23975	15336	#N/A	1.000000E+00
Gm23976	15337	#N/A	1.000000E+00
Gm23977	15338	#N/A	1.000000E+00
Gm23978	15339	#N/A	1.000000E+00
Gm23979	15340	#N/A	1.000000E+00
Gm23980	15341	#N/A	1.000000E+00
Gm23981	15342	#N/A	1.000000E+00
Gm23982	15343	#N/A	1.000000E+00
Gm23983	15344	#N/A	1.000000E+00
Gm23984	15345	#N/A	1.000000E+00
Gm23985	15346	#N/A	1.000000E+00
Gm23986	15347	#N/A	1.000000E+00
Gm23987	15348	#N/A	1.000000E+00
Gm23988	15349	#N/A	1.000000E+00
Gm23989	15350	#N/A	1.000000E+00

Spearman Rank correlation analysis performed between Prdm1 and all-expressed genes within the Meredith RNA-seq dataset. Robust Prdm1–associated genes were identified using a cut-off of $p < 0.0005$.

Table S1, Related to Supplemental Figure 3C. Prdm1 associated genes

Gm2399	15351		#N/A	1.000000E+00
Gm23990	15352		#N/A	1.000000E+00
Gm23991	15353		#N/A	1.000000E+00
Gm23992	15354		#N/A	1.000000E+00
Gm23993	15355		#N/A	1.000000E+00
Gm23994	15356		#N/A	1.000000E+00
Gm23995	15357		#N/A	1.000000E+00
Gm23996	15358		#N/A	1.000000E+00
Gm23997	15359		#N/A	1.000000E+00
Gm23998	15360		#N/A	1.000000E+00
Gm23999	15361		#N/A	1.000000E+00
Gm24000	15362		#N/A	1.000000E+00
Gm24001	15363		#N/A	1.000000E+00
Gm24002	15364		#N/A	1.000000E+00
Gm24003	15365		#N/A	1.000000E+00
Gm24004	15366	-0.038406171	3065	5.892384E-01
Gm24005	15367		#N/A	1.000000E+00
Gm24006	15368		#N/A	1.000000E+00
Gm24007	15369		#N/A	1.000000E+00
Gm24008	15370		#N/A	1.000000E+00
Gm24009	15371		#N/A	1.000000E+00
Gm24010	15372		#N/A	1.000000E+00
Gm24011	15373		#N/A	1.000000E+00
Gm24012	15374		#N/A	1.000000E+00
Gm24013	15375		#N/A	1.000000E+00
Gm24014	15376		#N/A	1.000000E+00
Gm24015	15377		#N/A	1.000000E+00
Gm24016	15378		#N/A	1.000000E+00
Gm24017	15379		#N/A	1.000000E+00
Gm24018	15380		#N/A	1.000000E+00
Gm24019	15381		#N/A	1.000000E+00
Gm24020	15382		#N/A	1.000000E+00
Gm24021	15383		#N/A	1.000000E+00
Gm24022	15384		#N/A	1.000000E+00
Gm24023	15385		#N/A	1.000000E+00
Gm24024	15386		#N/A	1.000000E+00
Gm24025	15387		#N/A	1.000000E+00
Gm24026	15388		#N/A	1.000000E+00
Gm24027	15389		#N/A	1.000000E+00
Gm24028	15390		#N/A	1.000000E+00
Gm24029	15391		#N/A	1.000000E+00
Gm24030	15392		#N/A	1.000000E+00
Gm24031	15393		#N/A	1.000000E+00

Spearman Rank correlation analysis performed between Prdm1 and all-expressed genes within the Meredith RNA-seq dataset. Robust Prdm1–associated genes were identified using a cut-off of $p < 0.0005$.

Table S1, Related to Supplemental Figure 3C. Prdm1 associated genes

Gm24032	15394		#N/A	1.000000E+00
Gm24033	15395		#N/A	1.000000E+00
Gm24034	15396		#N/A	1.000000E+00
Gm24035	15397		#N/A	1.000000E+00
Gm24036	15398		#N/A	1.000000E+00
Gm24037	15399	-0.038406171	3065	5.892384E-01
Gm24038	15400		#N/A	1.000000E+00
Gm24039	15401		#N/A	1.000000E+00
Gm24040	15402		#N/A	1.000000E+00
Gm24041	15403		#N/A	1.000000E+00
Gm24042	15404		#N/A	1.000000E+00
Gm24043	15405		#N/A	1.000000E+00
Gm24044	15406		#N/A	1.000000E+00
Gm24045	15407		#N/A	1.000000E+00
Gm24046	15408		#N/A	1.000000E+00
Gm24047	15409		#N/A	1.000000E+00
Gm24048	15410		#N/A	1.000000E+00
Gm24049	15411		#N/A	1.000000E+00
Gm24050	15412		#N/A	1.000000E+00
Gm24051	15413		#N/A	1.000000E+00
Gm24052	15414		#N/A	1.000000E+00
Gm24053	15415		#N/A	1.000000E+00
Gm24054	15416		#N/A	1.000000E+00
Gm24055	15417		#N/A	1.000000E+00
Gm24056	15418		#N/A	1.000000E+00
Gm24057	15419		#N/A	1.000000E+00
Gm24058	15420		#N/A	1.000000E+00
Gm24059	15421		#N/A	1.000000E+00
Gm24060	15422		#N/A	1.000000E+00
Gm24061	15423		#N/A	1.000000E+00
Gm24062	15424	-0.038406171	3065	5.892384E-01
Gm24063	15425		#N/A	1.000000E+00
Gm24064	15426		#N/A	1.000000E+00
Gm24065	15427		#N/A	1.000000E+00
Gm24066	15428		#N/A	1.000000E+00
Gm24067	15429		#N/A	1.000000E+00
Gm24068	15430		#N/A	1.000000E+00
Gm24069	15431		#N/A	1.000000E+00
Gm24070	15432		#N/A	1.000000E+00
Gm24071	15433		#N/A	1.000000E+00
Gm24072	15434		#N/A	1.000000E+00
Gm24073	15435		#N/A	1.000000E+00
Gm24074	15436		#N/A	1.000000E+00

Spearman Rank correlation analysis performed between Prdm1 and all-expressed genes within the Meredith RNA-seq dataset. Robust Prdm1–associated genes were identified using a cut-off of $p < 0.0005$.

Table S1, Related to Supplemental Figure 3C. Prdm1 associated genes

Gm24075	15437		#N/A	1.000000E+00
Gm24076	15438		#N/A	1.000000E+00
Gm24077	15439		#N/A	1.000000E+00
Gm24078	15440		#N/A	1.000000E+00
Gm24079	15441		#N/A	1.000000E+00
Gm24080	15442		#N/A	1.000000E+00
Gm24081	15443		#N/A	1.000000E+00
Gm24082	15444		#N/A	1.000000E+00
Gm24083	15445		#N/A	1.000000E+00
Gm24084	15446		#N/A	1.000000E+00
Gm24085	15447		#N/A	1.000000E+00
Gm24086	15448		#N/A	1.000000E+00
Gm24087	15449		#N/A	1.000000E+00
Gm24088	15450		#N/A	1.000000E+00
Gm24089	15451		#N/A	1.000000E+00
Gm24090	15452		#N/A	1.000000E+00
Gm24091	15453		#N/A	1.000000E+00
Gm24092	15454		#N/A	1.000000E+00
Gm24093	15455		#N/A	1.000000E+00
Gm24094	15456		#N/A	1.000000E+00
Gm24095	15457		#N/A	1.000000E+00
Gm24096	15458		#N/A	1.000000E+00
Gm24097	15459		#N/A	1.000000E+00
Gm24098	15460	0.157799267	16153.5	2.563935E-02
Gm24099	15461		#N/A	1.000000E+00
Gm24100	15462		#N/A	1.000000E+00
Gm24101	15463		#N/A	1.000000E+00
Gm24102	15464		#N/A	1.000000E+00
Gm24103	15465		#N/A	1.000000E+00
Gm24104	15466		#N/A	1.000000E+00
Gm24105	15467	0.037551558	7460	5.975571E-01
Gm24106	15468		#N/A	1.000000E+00
Gm24107	15469		#N/A	1.000000E+00
Gm24108	15470		#N/A	1.000000E+00
Gm24109	15471		#N/A	1.000000E+00
Gm24110	15472		#N/A	1.000000E+00
Gm24111	15473		#N/A	1.000000E+00
Gm24112	15474		#N/A	1.000000E+00
Gm24113	15475		#N/A	1.000000E+00
Gm24114	15476		#N/A	1.000000E+00
Gm24115	15477		#N/A	1.000000E+00
Gm24116	15478		#N/A	1.000000E+00
Gm24117	15479		#N/A	1.000000E+00

Spearman Rank correlation analysis performed between Prdm1 and all-expressed genes within the Meredith RNA-seq dataset. Robust Prdm1–associated genes were identified using a cut-off of $p < 0.0005$.

Table S1, Related to Supplemental Figure 3C. Prdm1 associated genes

Gm24118	15480		#N/A	1.000000E+00
Gm24119	15481		#N/A	1.000000E+00
Gm24120	15482		#N/A	1.000000E+00
Gm24121	15483		#N/A	1.000000E+00
Gm24122	15484		#N/A	1.000000E+00
Gm24123	15485		#N/A	1.000000E+00
Gm24124	15486		#N/A	1.000000E+00
Gm24125	15487		#N/A	1.000000E+00
Gm24126	15488		#N/A	1.000000E+00
Gm24127	15489		#N/A	1.000000E+00
Gm24128	15490		#N/A	1.000000E+00
Gm24129	15491		#N/A	1.000000E+00
Gm24130	15492		#N/A	1.000000E+00
Gm24131	15493		#N/A	1.000000E+00
Gm24132	15494		#N/A	1.000000E+00
Gm24133	15495		#N/A	1.000000E+00
Gm24134	15496		#N/A	1.000000E+00
Gm24135	15497		#N/A	1.000000E+00
Gm24136	15498		#N/A	1.000000E+00
Gm24137	15499		#N/A	1.000000E+00
Gm24138	15500		#N/A	1.000000E+00
Gm24139	15501		#N/A	1.000000E+00
Gm24140	15502	-0.054451513	1703	4.437940E-01
Gm24141	15503		#N/A	1.000000E+00
Gm24142	15504		#N/A	1.000000E+00
Gm24143	15505		#N/A	1.000000E+00
Gm24144	15506		#N/A	1.000000E+00
Gm24145	15507		#N/A	1.000000E+00
Gm24146	15508		#N/A	1.000000E+00
Gm24147	15509		#N/A	1.000000E+00
Gm24148	15510		#N/A	1.000000E+00
Gm24149	15511		#N/A	1.000000E+00
Gm2415	15512	0.155250525	15992	2.815363E-02
Gm24150	15513		#N/A	1.000000E+00
Gm24151	15514		#N/A	1.000000E+00
Gm24152	15515		#N/A	1.000000E+00
Gm24153	15516		#N/A	1.000000E+00
Gm24154	15517		#N/A	1.000000E+00
Gm24155	15518		#N/A	1.000000E+00
Gm24156	15519		#N/A	1.000000E+00
Gm24157	15520		#N/A	1.000000E+00
Gm24158	15521		#N/A	1.000000E+00
Gm24159	15522		#N/A	1.000000E+00

Spearman Rank correlation analysis performed between Prdm1 and all-expressed genes within the Meredith RNA-seq dataset. Robust Prdm1–associated genes were identified using a cut-off of $p < 0.0005$.

Table S1, Related to Supplemental Figure 3C. Prdm1 associated genes

Gm24160	15523		#N/A	1.000000E+00
Gm24161	15524		#N/A	1.000000E+00
Gm24162	15525		#N/A	1.000000E+00
Gm24163	15526		#N/A	1.000000E+00
Gm24164	15527		#N/A	1.000000E+00
Gm24165	15528		#N/A	1.000000E+00
Gm24166	15529		#N/A	1.000000E+00
Gm24167	15530		#N/A	1.000000E+00
Gm24168	15531		#N/A	1.000000E+00
Gm24169	15532		#N/A	1.000000E+00
Gm24170	15533		#N/A	1.000000E+00
Gm24171	15534		#N/A	1.000000E+00
Gm24172	15535		#N/A	1.000000E+00
Gm24173	15536		#N/A	1.000000E+00
Gm24174	15537		#N/A	1.000000E+00
Gm24175	15538		#N/A	1.000000E+00
Gm24176	15539		#N/A	1.000000E+00
Gm24177	15540		#N/A	1.000000E+00
Gm24178	15541		#N/A	1.000000E+00
Gm24179	15542		#N/A	1.000000E+00
Gm24180	15543		#N/A	1.000000E+00
Gm24181	15544		#N/A	1.000000E+00
Gm24182	15545		#N/A	1.000000E+00
Gm24183	15546		#N/A	1.000000E+00
Gm24184	15547		#N/A	1.000000E+00
Gm24185	15548		#N/A	1.000000E+00
Gm24186	15549		#N/A	1.000000E+00
Gm24187	15550	0.123921859	13904	8.041557E-02
Gm24188	15551		#N/A	1.000000E+00
Gm24189	15552		#N/A	1.000000E+00
Gm24190	15553		#N/A	1.000000E+00
Gm24191	15554		#N/A	1.000000E+00
Gm24192	15555		#N/A	1.000000E+00
Gm24193	15556		#N/A	1.000000E+00
Gm24194	15557		#N/A	1.000000E+00
Gm24195	15558		#N/A	1.000000E+00
Gm24196	15559		#N/A	1.000000E+00
Gm24197	15560		#N/A	1.000000E+00
Gm24198	15561		#N/A	1.000000E+00
Gm24199	15562		#N/A	1.000000E+00
Gm2420	15563		#N/A	1.000000E+00
Gm24200	15564		#N/A	1.000000E+00
Gm24201	15565		#N/A	1.000000E+00

Spearman Rank correlation analysis performed between Prdm1 and all-expressed genes within the Meredith RNA-seq dataset. Robust Prdm1–associated genes were identified using a cut-off of $p < 0.0005$.

Table S1, Related to Supplemental Figure 3C. Prdm1 associated genes

Gm24202	15566		#N/A	1.000000E+00
Gm24203	15567		#N/A	1.000000E+00
Gm24204	15568	-0.038406171	3065	5.892384E-01
Gm24205	15569		#N/A	1.000000E+00
Gm24206	15570		#N/A	1.000000E+00
Gm24207	15571		#N/A	1.000000E+00
Gm24208	15572		#N/A	1.000000E+00
Gm24209	15573		#N/A	1.000000E+00
Gm24210	15574		#N/A	1.000000E+00
Gm24211	15575		#N/A	1.000000E+00
Gm24212	15576		#N/A	1.000000E+00
Gm24213	15577		#N/A	1.000000E+00
Gm24214	15578		#N/A	1.000000E+00
Gm24215	15579		#N/A	1.000000E+00
Gm24216	15580		#N/A	1.000000E+00
Gm24217	15581		#N/A	1.000000E+00
Gm24218	15582		#N/A	1.000000E+00
Gm24219	15583		#N/A	1.000000E+00
Gm24220	15584		#N/A	1.000000E+00
Gm24221	15585		#N/A	1.000000E+00
Gm24222	15586		#N/A	1.000000E+00
Gm24223	15587		#N/A	1.000000E+00
Gm24224	15588		#N/A	1.000000E+00
Gm24225	15589		#N/A	1.000000E+00
Gm24226	15590		#N/A	1.000000E+00
Gm24227	15591		#N/A	1.000000E+00
Gm24228	15592		#N/A	1.000000E+00
Gm24229	15593		#N/A	1.000000E+00
Gm2423	15594		#N/A	1.000000E+00
Gm24230	15595		#N/A	1.000000E+00
Gm24231	15596		#N/A	1.000000E+00
Gm24232	15597		#N/A	1.000000E+00
Gm24233	15598		#N/A	1.000000E+00
Gm24234	15599		#N/A	1.000000E+00
Gm24235	15600		#N/A	1.000000E+00
Gm24236	15601		#N/A	1.000000E+00
Gm24237	15602		#N/A	1.000000E+00
Gm24238	15603		#N/A	1.000000E+00
Gm24239	15604		#N/A	1.000000E+00
Gm24240	15605		#N/A	1.000000E+00
Gm24241	15606		#N/A	1.000000E+00
Gm24242	15607		#N/A	1.000000E+00
Gm24243	15608		#N/A	1.000000E+00

Spearman Rank correlation analysis performed between Prdm1 and all-expressed genes within the Meredith RNA-seq dataset. Robust Prdm1–associated genes were identified using a cut-off of $p < 0.0005$.

Table S1, Related to Supplemental Figure 3C. Prdm1 associated genes

Gm24244	15609	0.021317126	6513.5	7.644715E-01
Gm24245	15610	0.021317126	6513.5	7.644715E-01
Gm24246	15611		#N/A	1.000000E+00
Gm24247	15612		#N/A	1.000000E+00
Gm24248	15613		#N/A	1.000000E+00
Gm24249	15614		#N/A	1.000000E+00
Gm24250	15615		#N/A	1.000000E+00
Gm24251	15616		#N/A	1.000000E+00
Gm24252	15617		#N/A	1.000000E+00
Gm24253	15618		#N/A	1.000000E+00
Gm24254	15619		#N/A	1.000000E+00
Gm24255	15620		#N/A	1.000000E+00
Gm24256	15621		#N/A	1.000000E+00
Gm24257	15622		#N/A	1.000000E+00
Gm24258	15623		#N/A	1.000000E+00
Gm24259	15624		#N/A	1.000000E+00
Gm24260	15625		#N/A	1.000000E+00
Gm24261	15626		#N/A	1.000000E+00
Gm24262	15627		#N/A	1.000000E+00
Gm24263	15628		#N/A	1.000000E+00
Gm24264	15629		#N/A	1.000000E+00
Gm24265	15630		#N/A	1.000000E+00
Gm24266	15631		#N/A	1.000000E+00
Gm24267	15632		#N/A	1.000000E+00
Gm24268	15633		#N/A	1.000000E+00
Gm24269	15634		#N/A	1.000000E+00
Gm24270	15635		#N/A	1.000000E+00
Gm24271	15636		#N/A	1.000000E+00
Gm24272	15637	0.065904853	9584	3.538218E-01
Gm24273	15638		#N/A	1.000000E+00
Gm24274	15639		#N/A	1.000000E+00
Gm24275	15640		#N/A	1.000000E+00
Gm24276	15641	0.035516473	7349	6.175765E-01
Gm24277	15642		#N/A	1.000000E+00
Gm24278	15643		#N/A	1.000000E+00
Gm24279	15644		#N/A	1.000000E+00
Gm24280	15645		#N/A	1.000000E+00
Gm24281	15646		#N/A	1.000000E+00
Gm24282	15647		#N/A	1.000000E+00
Gm24283	15648		#N/A	1.000000E+00
Gm24284	15649		#N/A	1.000000E+00
Gm24285	15650		#N/A	1.000000E+00
Gm24286	15651		#N/A	1.000000E+00

Spearman Rank correlation analysis performed between Prdm1 and all-expressed genes within the Meredith RNA-seq dataset. Robust Prdm1–associated genes were identified using a cut-off of $p < 0.0005$.

Table S1, Related to Supplemental Figure 3C. Prdm1 associated genes

Gm24287	15652		#N/A	1.000000E+00
Gm24288	15653		#N/A	1.000000E+00
Gm24289	15654		#N/A	1.000000E+00
Gm24290	15655		#N/A	1.000000E+00
Gm24291	15656		#N/A	1.000000E+00
Gm24292	15657		#N/A	1.000000E+00
Gm24293	15658		#N/A	1.000000E+00
Gm24294	15659		#N/A	1.000000E+00
Gm24295	15660		#N/A	1.000000E+00
Gm24296	15661		#N/A	1.000000E+00
Gm24297	15662		#N/A	1.000000E+00
Gm24298	15663		#N/A	1.000000E+00
Gm24299	15664		#N/A	1.000000E+00
Gm24300	15665		#N/A	1.000000E+00
Gm24301	15666		#N/A	1.000000E+00
Gm24302	15667		#N/A	1.000000E+00
Gm24303	15668		#N/A	1.000000E+00
Gm24304	15669		#N/A	1.000000E+00
Gm24305	15670		#N/A	1.000000E+00
Gm24306	15671		#N/A	1.000000E+00
Gm24307	15672		#N/A	1.000000E+00
Gm24308	15673		#N/A	1.000000E+00
Gm24309	15674		#N/A	1.000000E+00
Gm2431	15675		#N/A	1.000000E+00
Gm24310	15676		#N/A	1.000000E+00
Gm24311	15677	0.053267784	8685	4.537786E-01
Gm24312	15678		#N/A	1.000000E+00
Gm24313	15679		#N/A	1.000000E+00
Gm24314	15680		#N/A	1.000000E+00
Gm24315	15681		#N/A	1.000000E+00
Gm24316	15682		#N/A	1.000000E+00
Gm24317	15683		#N/A	1.000000E+00
Gm24318	15684		#N/A	1.000000E+00
Gm24319	15685		#N/A	1.000000E+00
Gm24320	15686		#N/A	1.000000E+00
Gm24321	15687		#N/A	1.000000E+00
Gm24322	15688		#N/A	1.000000E+00
Gm24323	15689		#N/A	1.000000E+00
Gm24324	15690		#N/A	1.000000E+00
Gm24325	15691		#N/A	1.000000E+00
Gm24326	15692		#N/A	1.000000E+00
Gm24327	15693		#N/A	1.000000E+00
Gm24328	15694		#N/A	1.000000E+00

Spearman Rank correlation analysis performed between Prdm1 and all-expressed genes within the Meredith RNA-seq dataset. Robust Prdm1–associated genes were identified using a cut-off of $p < 0.0005$.

Table S1, Related to Supplemental Figure 3C. Prdm1 associated genes

Gm24329	15695		#N/A	1.000000E+00
Gm24330	15696		#N/A	1.000000E+00
Gm24331	15697		#N/A	1.000000E+00
Gm24332	15698		#N/A	1.000000E+00
Gm24333	15699		#N/A	1.000000E+00
Gm24334	15700		#N/A	1.000000E+00
Gm24335	15701		#N/A	1.000000E+00
Gm24336	15702		#N/A	1.000000E+00
Gm24337	15703		#N/A	1.000000E+00
Gm24338	15704		#N/A	1.000000E+00
Gm24339	15705		#N/A	1.000000E+00
Gm24340	15706		#N/A	1.000000E+00
Gm24341	15707		#N/A	1.000000E+00
Gm24342	15708		#N/A	1.000000E+00
Gm24343	15709		#N/A	1.000000E+00
Gm24344	15710		#N/A	1.000000E+00
Gm24345	15711		#N/A	1.000000E+00
Gm24346	15712		#N/A	1.000000E+00
Gm24347	15713		#N/A	1.000000E+00
Gm24348	15714		#N/A	1.000000E+00
Gm24349	15715		#N/A	1.000000E+00
Gm24350	15716		#N/A	1.000000E+00
Gm24351	15717		#N/A	1.000000E+00
Gm24352	15718		#N/A	1.000000E+00
Gm24354	15719		#N/A	1.000000E+00
Gm24355	15720		#N/A	1.000000E+00
Gm24356	15721		#N/A	1.000000E+00
Gm24357	15722		#N/A	1.000000E+00
Gm24358	15723		#N/A	1.000000E+00
Gm24359	15724		#N/A	1.000000E+00
Gm24360	15725		#N/A	1.000000E+00
Gm24361	15726	-0.038406171	3065	5.892384E-01
Gm24362	15727		#N/A	1.000000E+00
Gm24363	15728		#N/A	1.000000E+00
Gm24364	15729		#N/A	1.000000E+00
Gm24365	15730		#N/A	1.000000E+00
Gm24366	15731		#N/A	1.000000E+00
Gm24368	15732		#N/A	1.000000E+00
Gm24369	15733		#N/A	1.000000E+00
Gm24370	15734	-0.038406171	3065	5.892384E-01
Gm24371	15735		#N/A	1.000000E+00
Gm24372	15736		#N/A	1.000000E+00
Gm24373	15737		#N/A	1.000000E+00

Spearman Rank correlation analysis performed between Prdm1 and all-expressed genes within the Meredith RNA-seq dataset. Robust Prdm1–associated genes were identified using a cut-off of $p < 0.0005$.

Table S1, Related to Supplemental Figure 3C. Prdm1 associated genes

Gm24374	15738		#N/A	1.000000E+00
Gm24375	15739		#N/A	1.000000E+00
Gm24376	15740		#N/A	1.000000E+00
Gm24377	15741		#N/A	1.000000E+00
Gm24378	15742		#N/A	1.000000E+00
Gm24379	15743		#N/A	1.000000E+00
Gm24380	15744		#N/A	1.000000E+00
Gm24381	15745		#N/A	1.000000E+00
Gm24382	15746		#N/A	1.000000E+00
Gm24383	15747		#N/A	1.000000E+00
Gm24384	15748		#N/A	1.000000E+00
Gm24385	15749		#N/A	1.000000E+00
Gm24386	15750		#N/A	1.000000E+00
Gm24387	15751		#N/A	1.000000E+00
Gm24388	15752		#N/A	1.000000E+00
Gm24389	15753		#N/A	1.000000E+00
Gm24390	15754		#N/A	1.000000E+00
Gm24391	15755		#N/A	1.000000E+00
Gm24392	15756		#N/A	1.000000E+00
Gm24393	15757		#N/A	1.000000E+00
Gm24394	15758		#N/A	1.000000E+00
Gm24395	15759		#N/A	1.000000E+00
Gm24396	15760		#N/A	1.000000E+00
Gm24397	15761		#N/A	1.000000E+00
Gm24398	15762		#N/A	1.000000E+00
Gm24399	15763		#N/A	1.000000E+00
Gm24400	15764		#N/A	1.000000E+00
Gm24401	15765		#N/A	1.000000E+00
Gm24402	15766		#N/A	1.000000E+00
Gm24403	15767		#N/A	1.000000E+00
Gm24404	15768		#N/A	1.000000E+00
Gm24405	15769		#N/A	1.000000E+00
Gm24406	15770		#N/A	1.000000E+00
Gm24407	15771	0.109930598	12709.5	1.212354E-01
Gm24408	15772		#N/A	1.000000E+00
Gm24409	15773		#N/A	1.000000E+00
Gm24410	15774		#N/A	1.000000E+00
Gm24411	15775		#N/A	1.000000E+00
Gm24412	15776		#N/A	1.000000E+00
Gm24413	15777		#N/A	1.000000E+00
Gm24414	15778		#N/A	1.000000E+00
Gm24415	15779		#N/A	1.000000E+00
Gm24416	15780		#N/A	1.000000E+00

Spearman Rank correlation analysis performed between Prdm1 and all-expressed genes within the Meredith RNA-seq dataset. Robust Prdm1–associated genes were identified using a cut-off of $p < 0.0005$.

Gm24417	15781	#N/A	1.000000E+00
Gm24418	15782	#N/A	1.000000E+00
Gm24419	15783	#N/A	1.000000E+00
Gm24420	15784	#N/A	1.000000E+00
Gm24421	15785	#N/A	1.000000E+00
Gm24422	15786	#N/A	1.000000E+00
Gm24423	15787	#N/A	1.000000E+00
Gm24424	15788	#N/A	1.000000E+00
Gm24425	15789	#N/A	1.000000E+00
Gm24426	15790	#N/A	1.000000E+00
Gm24427	15791	#N/A	1.000000E+00
Gm24428	15792	#N/A	1.000000E+00
Gm24429	15793	#N/A	1.000000E+00
Gm24430	15794	#N/A	1.000000E+00
Gm24431	15795	#N/A	1.000000E+00
Gm24432	15796	#N/A	1.000000E+00
Gm24433	15797	#N/A	1.000000E+00
Gm24434	15798	#N/A	1.000000E+00
Gm24435	15799	#N/A	1.000000E+00
Gm24436	15800	#N/A	1.000000E+00
Gm24437	15801	#N/A	1.000000E+00
Gm24438	15802	#N/A	1.000000E+00
Gm24439	15803	#N/A	1.000000E+00
Gm24440	15804	#N/A	1.000000E+00
Gm24441	15805	#N/A	1.000000E+00
Gm24442	15806	#N/A	1.000000E+00
Gm24443	15807	#N/A	1.000000E+00
Gm24444	15808	#N/A	1.000000E+00
Gm24445	15809	#N/A	1.000000E+00
Gm24446	15810	#N/A	1.000000E+00
Gm24447	15811	#N/A	1.000000E+00
Gm24448	15812	#N/A	1.000000E+00
Gm24449	15813	#N/A	1.000000E+00
Gm24450	15814	#N/A	1.000000E+00
Gm24451	15815	#N/A	1.000000E+00
Gm24452	15816	#N/A	1.000000E+00
Gm24453	15817	#N/A	1.000000E+00
Gm24454	15818	#N/A	1.000000E+00
Gm24455	15819	#N/A	1.000000E+00
Gm24456	15820	#N/A	1.000000E+00
Gm24457	15821	#N/A	1.000000E+00
Gm24458	15822	#N/A	1.000000E+00
Gm24459	15823	#N/A	1.000000E+00

Gm24460	15824	#N/A	1.000000E+00
Gm24461	15825	#N/A	1.000000E+00
Gm24462	15826	#N/A	1.000000E+00
Gm24463	15827	#N/A	1.000000E+00
Gm24464	15828	#N/A	1.000000E+00
Gm24465	15829	#N/A	1.000000E+00
Gm24466	15830	#N/A	1.000000E+00
Gm24467	15831	#N/A	1.000000E+00
Gm24468	15832	#N/A	1.000000E+00
Gm24469	15833	#N/A	1.000000E+00
Gm24470	15834	#N/A	1.000000E+00
Gm24471	15835	#N/A	1.000000E+00
Gm24472	15836	#N/A	1.000000E+00
Gm24473	15837	#N/A	1.000000E+00
Gm24474	15838	#N/A	1.000000E+00
Gm24475	15839	#N/A	1.000000E+00
Gm24476	15840	#N/A	1.000000E+00
Gm24477	15841	#N/A	1.000000E+00
Gm24478	15842	#N/A	1.000000E+00
Gm24479	15843	#N/A	1.000000E+00
Gm24480	15844	#N/A	1.000000E+00
Gm24481	15845	#N/A	1.000000E+00
Gm24482	15846	#N/A	1.000000E+00
Gm24483	15847	#N/A	1.000000E+00
Gm24484	15848	#N/A	1.000000E+00
Gm24485	15849	#N/A	1.000000E+00
Gm24486	15850	#N/A	1.000000E+00
Gm24487	15851	#N/A	1.000000E+00
Gm24488	15852	#N/A	1.000000E+00
Gm24489	15853	#N/A	1.000000E+00
Gm24490	15854	#N/A	1.000000E+00
Gm24491	15855	#N/A	1.000000E+00
Gm24492	15856	#N/A	1.000000E+00
Gm24493	15857	#N/A	1.000000E+00
Gm24494	15858	#N/A	1.000000E+00
Gm24495	15859	#N/A	1.000000E+00
Gm24496	15860	#N/A	1.000000E+00
Gm24497	15861	#N/A	1.000000E+00
Gm24498	15862	#N/A	1.000000E+00
Gm24499	15863	#N/A	1.000000E+00
Gm24500	15864	#N/A	1.000000E+00
Gm24501	15865	#N/A	1.000000E+00
Gm24502	15866	#N/A	1.000000E+00

Table S1, Related to Supplemental Figure 3C. Prdm1 associated genes

Gm24503	15867		#N/A	1.000000E+00
Gm24504	15868		#N/A	1.000000E+00
Gm24505	15869		#N/A	1.000000E+00
Gm24506	15870		#N/A	1.000000E+00
Gm24507	15871		#N/A	1.000000E+00
Gm24508	15872	-0.038406171	3065	5.892384E-01
Gm24509	15873		#N/A	1.000000E+00
Gm24510	15874		#N/A	1.000000E+00
Gm24511	15875	-0.054451513	1703	4.437940E-01
Gm24512	15876		#N/A	1.000000E+00
Gm24513	15877		#N/A	1.000000E+00
Gm24514	15878		#N/A	1.000000E+00
Gm24515	15879		#N/A	1.000000E+00
Gm24516	15880		#N/A	1.000000E+00
Gm24517	15881	-0.038406171	3065	5.892384E-01
Gm24518	15882		#N/A	1.000000E+00
Gm24519	15883		#N/A	1.000000E+00
Gm24520	15884		#N/A	1.000000E+00
Gm24521	15885		#N/A	1.000000E+00
Gm24522	15886		#N/A	1.000000E+00
Gm24523	15887		#N/A	1.000000E+00
Gm24524	15888		#N/A	1.000000E+00
Gm24525	15889		#N/A	1.000000E+00
Gm24526	15890		#N/A	1.000000E+00
Gm24527	15891		#N/A	1.000000E+00
Gm24528	15892		#N/A	1.000000E+00
Gm24529	15893		#N/A	1.000000E+00
Gm24530	15894		#N/A	1.000000E+00
Gm24531	15895		#N/A	1.000000E+00
Gm24532	15896		#N/A	1.000000E+00
Gm24533	15897		#N/A	1.000000E+00
Gm24534	15898		#N/A	1.000000E+00
Gm24535	15899		#N/A	1.000000E+00
Gm24536	15900		#N/A	1.000000E+00
Gm24537	15901		#N/A	1.000000E+00
Gm24538	15902		#N/A	1.000000E+00
Gm24539	15903		#N/A	1.000000E+00
Gm24540	15904		#N/A	1.000000E+00
Gm24541	15905		#N/A	1.000000E+00
Gm24542	15906		#N/A	1.000000E+00
Gm24543	15907		#N/A	1.000000E+00
Gm24544	15908		#N/A	1.000000E+00
Gm24545	15909		#N/A	1.000000E+00

Spearman Rank correlation analysis performed between Prdm1 and all-expressed genes within the Meredith RNA-seq dataset. Robust Prdm1–associated genes were identified using a cut-off of $p < 0.0005$.

Table S1, Related to Supplemental Figure 3C. Prdm1 associated genes

Gm24546	15910		#N/A	1.000000E+00
Gm24547	15911		#N/A	1.000000E+00
Gm24548	15912		#N/A	1.000000E+00
Gm24549	15913		#N/A	1.000000E+00
Gm24550	15914		#N/A	1.000000E+00
Gm24551	15915		#N/A	1.000000E+00
Gm24552	15916		#N/A	1.000000E+00
Gm24553	15917		#N/A	1.000000E+00
Gm24554	15918		#N/A	1.000000E+00
Gm24555	15919		#N/A	1.000000E+00
Gm24556	15920		#N/A	1.000000E+00
Gm24557	15921		#N/A	1.000000E+00
Gm24558	15922		#N/A	1.000000E+00
Gm24559	15923		#N/A	1.000000E+00
Gm24560	15924		#N/A	1.000000E+00
Gm24561	15925		#N/A	1.000000E+00
Gm24562	15926		#N/A	1.000000E+00
Gm24563	15927		#N/A	1.000000E+00
Gm24564	15928		#N/A	1.000000E+00
Gm24565	15929		#N/A	1.000000E+00
Gm24566	15930		#N/A	1.000000E+00
Gm24567	15931		#N/A	1.000000E+00
Gm24568	15932		#N/A	1.000000E+00
Gm24569	15933		#N/A	1.000000E+00
Gm24570	15934		#N/A	1.000000E+00
Gm24571	15935		#N/A	1.000000E+00
Gm24572	15936		#N/A	1.000000E+00
Gm24573	15937		#N/A	1.000000E+00
Gm24574	15938		#N/A	1.000000E+00
Gm24575	15939		#N/A	1.000000E+00
Gm24576	15940		#N/A	1.000000E+00
Gm24577	15941		#N/A	1.000000E+00
Gm24578	15942		#N/A	1.000000E+00
Gm24579	15943		#N/A	1.000000E+00
Gm24580	15944		#N/A	1.000000E+00
Gm24581	15945		#N/A	1.000000E+00
Gm24582	15946		#N/A	1.000000E+00
Gm24583	15947		#N/A	1.000000E+00
Gm24584	15948		#N/A	1.000000E+00
Gm24585	15949		#N/A	1.000000E+00
Gm24586	15950		#N/A	1.000000E+00
Gm24587	15951		#N/A	1.000000E+00
Gm24588	15952	0.149110542	15597	3.508992E-02

Spearman Rank correlation analysis performed between Prdm1 and all-expressed genes within the Meredith RNA-seq dataset. Robust Prdm1–associated genes were identified using a cut-off of $p < 0.0005$.

Table S1, Related to Supplemental Figure 3C. Prdm1 associated genes

Gm24589	15953		#N/A	1.000000E+00
Gm24590	15954		#N/A	1.000000E+00
Gm24591	15955		#N/A	1.000000E+00
Gm24592	15956		#N/A	1.000000E+00
Gm24593	15957		#N/A	1.000000E+00
Gm24594	15958		#N/A	1.000000E+00
Gm24595	15959		#N/A	1.000000E+00
Gm24596	15960		#N/A	1.000000E+00
Gm24597	15961		#N/A	1.000000E+00
Gm24598	15962		#N/A	1.000000E+00
Gm24599	15963		#N/A	1.000000E+00
Gm24600	15964		#N/A	1.000000E+00
Gm24601	15965		#N/A	1.000000E+00
Gm24602	15966		#N/A	1.000000E+00
Gm24603	15967		#N/A	1.000000E+00
Gm24604	15968		#N/A	1.000000E+00
Gm24605	15969		#N/A	1.000000E+00
Gm24606	15970		#N/A	1.000000E+00
Gm24607	15971		#N/A	1.000000E+00
Gm24608	15972		#N/A	1.000000E+00
Gm24609	15973		#N/A	1.000000E+00
Gm24610	15974		#N/A	1.000000E+00
Gm24611	15975		#N/A	1.000000E+00
Gm24612	15976		#N/A	1.000000E+00
Gm24613	15977		#N/A	1.000000E+00
Gm24614	15978		#N/A	1.000000E+00
Gm24615	15979		#N/A	1.000000E+00
Gm24616	15980		#N/A	1.000000E+00
Gm24617	15981		#N/A	1.000000E+00
Gm24618	15982		#N/A	1.000000E+00
Gm24619	15983		#N/A	1.000000E+00
Gm24620	15984		#N/A	1.000000E+00
Gm24621	15985		#N/A	1.000000E+00
Gm24622	15986		#N/A	1.000000E+00
Gm24623	15987		#N/A	1.000000E+00
Gm24624	15988		#N/A	1.000000E+00
Gm24625	15989		#N/A	1.000000E+00
Gm24626	15990		#N/A	1.000000E+00
Gm24627	15991		#N/A	1.000000E+00
Gm24628	15992		#N/A	1.000000E+00
Gm24629	15993		#N/A	1.000000E+00
Gm24630	15994		#N/A	1.000000E+00
Gm24631	15995	-0.054451513	1703	4.437940E-01

Spearman Rank correlation analysis performed between Prdm1 and all-expressed genes within the Meredith RNA-seq dataset. Robust Prdm1–associated genes were identified using a cut-off of $p < 0.0005$.

Table S1, Related to Supplemental Figure 3C. Prdm1 associated genes

Gm24632	15996		#N/A	1.000000E+00
Gm24633	15997		#N/A	1.000000E+00
Gm24634	15998		#N/A	1.000000E+00
Gm24635	15999		#N/A	1.000000E+00
Gm24636	16000		#N/A	1.000000E+00
Gm24637	16001		#N/A	1.000000E+00
Gm24638	16002		#N/A	1.000000E+00
Gm24639	16003		#N/A	1.000000E+00
Gm24640	16004		#N/A	1.000000E+00
Gm24641	16005		#N/A	1.000000E+00
Gm24642	16006		#N/A	1.000000E+00
Gm24643	16007		#N/A	1.000000E+00
Gm24644	16008		#N/A	1.000000E+00
Gm24645	16009		#N/A	1.000000E+00
Gm24646	16010		#N/A	1.000000E+00
Gm24647	16011		#N/A	1.000000E+00
Gm24648	16012		#N/A	1.000000E+00
Gm24649	16013		#N/A	1.000000E+00
Gm24650	16014		#N/A	1.000000E+00
Gm24651	16015		#N/A	1.000000E+00
Gm24652	16016		#N/A	1.000000E+00
Gm24653	16017		#N/A	1.000000E+00
Gm24654	16018		#N/A	1.000000E+00
Gm24655	16019		#N/A	1.000000E+00
Gm24656	16020		#N/A	1.000000E+00
Gm24657	16021		#N/A	1.000000E+00
Gm24658	16022		#N/A	1.000000E+00
Gm24659	16023		#N/A	1.000000E+00
Gm24660	16024		#N/A	1.000000E+00
Gm24661	16025		#N/A	1.000000E+00
Gm24662	16026	0.021317126	6513.5	7.644715E-01
Gm24663	16027		#N/A	1.000000E+00
Gm24664	16028		#N/A	1.000000E+00
Gm24665	16029		#N/A	1.000000E+00
Gm24666	16030		#N/A	1.000000E+00
Gm24667	16031		#N/A	1.000000E+00
Gm24668	16032		#N/A	1.000000E+00
Gm24669	16033		#N/A	1.000000E+00
Gm24670	16034		#N/A	1.000000E+00
Gm24671	16035		#N/A	1.000000E+00
Gm24672	16036		#N/A	1.000000E+00
Gm24673	16037		#N/A	1.000000E+00
Gm24674	16038		#N/A	1.000000E+00

Spearman Rank correlation analysis performed between Prdm1 and all-expressed genes within the Meredith RNA-seq dataset. Robust Prdm1–associated genes were identified using a cut-off of $p < 0.0005$.

Table S1, Related to Supplemental Figure 3C. Prdm1 associated genes

Gm24675	16039		#N/A	1.000000E+00
Gm24676	16040		#N/A	1.000000E+00
Gm24677	16041		#N/A	1.000000E+00
Gm24678	16042		#N/A	1.000000E+00
Gm24679	16043		#N/A	1.000000E+00
Gm24680	16044		#N/A	1.000000E+00
Gm24681	16045		#N/A	1.000000E+00
Gm24682	16046		#N/A	1.000000E+00
Gm24683	16047		#N/A	1.000000E+00
Gm24684	16048		#N/A	1.000000E+00
Gm24685	16049		#N/A	1.000000E+00
Gm24686	16050		#N/A	1.000000E+00
Gm24687	16051		#N/A	1.000000E+00
Gm24688	16052		#N/A	1.000000E+00
Gm24689	16053		#N/A	1.000000E+00
Gm24690	16054		#N/A	1.000000E+00
Gm24691	16055		#N/A	1.000000E+00
Gm24692	16056		#N/A	1.000000E+00
Gm24693	16057		#N/A	1.000000E+00
Gm24694	16058		#N/A	1.000000E+00
Gm24695	16059		#N/A	1.000000E+00
Gm24696	16060		#N/A	1.000000E+00
Gm24697	16061		#N/A	1.000000E+00
Gm24698	16062		#N/A	1.000000E+00
Gm24699	16063		#N/A	1.000000E+00
Gm24700	16064		#N/A	1.000000E+00
Gm24701	16065		#N/A	1.000000E+00
Gm24702	16066		#N/A	1.000000E+00
Gm24703	16067		#N/A	1.000000E+00
Gm24704	16068		#N/A	1.000000E+00
Gm24705	16069		#N/A	1.000000E+00
Gm24706	16070		#N/A	1.000000E+00
Gm24707	16071		#N/A	1.000000E+00
Gm24708	16072		#N/A	1.000000E+00
Gm24709	16073		#N/A	1.000000E+00
Gm24710	16074		#N/A	1.000000E+00
Gm24711	16075		#N/A	1.000000E+00
Gm24712	16076		#N/A	1.000000E+00
Gm24713	16077	0.075711049	10308	2.866340E-01
Gm24714	16078		#N/A	1.000000E+00
Gm24715	16079		#N/A	1.000000E+00
Gm24716	16080		#N/A	1.000000E+00
Gm24717	16081		#N/A	1.000000E+00

Spearman Rank correlation analysis performed between Prdm1 and all-expressed genes within the Meredith RNA-seq dataset. Robust Prdm1–associated genes were identified using a cut-off of $p < 0.0005$.

Gm24718	16082	#N/A	1.000000E+00
Gm24719	16083	#N/A	1.000000E+00
Gm24720	16084	#N/A	1.000000E+00
Gm24721	16085	#N/A	1.000000E+00
Gm24722	16086	#N/A	1.000000E+00
Gm24723	16087	#N/A	1.000000E+00
Gm24724	16088	#N/A	1.000000E+00
Gm24725	16089	#N/A	1.000000E+00
Gm24726	16090	#N/A	1.000000E+00
Gm24727	16091	#N/A	1.000000E+00
Gm24728	16092	#N/A	1.000000E+00
Gm24729	16093	#N/A	1.000000E+00
Gm24730	16094	#N/A	1.000000E+00
Gm24731	16095	#N/A	1.000000E+00
Gm24732	16096	#N/A	1.000000E+00
Gm24733	16097	#N/A	1.000000E+00
Gm24734	16098	#N/A	1.000000E+00
Gm24735	16099	#N/A	1.000000E+00
Gm24736	16100	#N/A	1.000000E+00
Gm24737	16101	#N/A	1.000000E+00
Gm24738	16102	#N/A	1.000000E+00
Gm24739	16103	#N/A	1.000000E+00
Gm24740	16104	#N/A	1.000000E+00
Gm24741	16105	#N/A	1.000000E+00
Gm24742	16106	#N/A	1.000000E+00
Gm24743	16107	#N/A	1.000000E+00
Gm24744	16108	#N/A	1.000000E+00
Gm24745	16109	#N/A	1.000000E+00
Gm24746	16110	#N/A	1.000000E+00
Gm24747	16111	#N/A	1.000000E+00
Gm24748	16112	#N/A	1.000000E+00
Gm24749	16113	#N/A	1.000000E+00
Gm24750	16114	#N/A	1.000000E+00
Gm24751	16115	#N/A	1.000000E+00
Gm24752	16116	#N/A	1.000000E+00
Gm24753	16117	#N/A	1.000000E+00
Gm24754	16118	#N/A	1.000000E+00
Gm24755	16119	#N/A	1.000000E+00
Gm24756	16120	#N/A	1.000000E+00
Gm24757	16121	#N/A	1.000000E+00
Gm24758	16122	#N/A	1.000000E+00
Gm24759	16123	#N/A	1.000000E+00
Gm24760	16124	#N/A	1.000000E+00

Table S1, Related to Supplemental Figure 3C. Prdm1 associated genes

Gm24761	16125	#N/A	1.000000E+00
Gm24762	16126	#N/A	1.000000E+00
Gm24763	16127	#N/A	1.000000E+00
Gm24764	16128	#N/A	1.000000E+00
Gm24765	16129	#N/A	1.000000E+00
Gm24766	16130	#N/A	1.000000E+00
Gm24767	16131	#N/A	1.000000E+00
Gm24768	16132	#N/A	1.000000E+00
Gm24769	16133	#N/A	1.000000E+00
Gm24770	16134	#N/A	1.000000E+00
Gm24771	16135	#N/A	1.000000E+00
Gm24772	16136	#N/A	1.000000E+00
Gm24773	16137	#N/A	1.000000E+00
Gm24774	16138	#N/A	1.000000E+00
Gm24775	16139	#N/A	1.000000E+00
Gm24776	16140	#N/A	1.000000E+00
Gm24777	16141	#N/A	1.000000E+00
Gm24778	16142	#N/A	1.000000E+00
Gm24779	16143	#N/A	1.000000E+00
Gm24780	16144	#N/A	1.000000E+00
Gm24781	16145	#N/A	1.000000E+00
Gm24782	16146	#N/A	1.000000E+00
Gm24783	16147	#N/A	1.000000E+00
Gm24784	16148	#N/A	1.000000E+00
Gm24785	16149	#N/A	1.000000E+00
Gm24786	16150	#N/A	1.000000E+00
Gm24787	16151	#N/A	1.000000E+00
Gm24788	16152	#N/A	1.000000E+00
Gm24789	16153	#N/A	1.000000E+00
Gm24790	16154	#N/A	1.000000E+00
Gm24791	16155	#N/A	1.000000E+00
Gm24792	16156	#N/A	1.000000E+00
Gm24793	16157	#N/A	1.000000E+00
Gm24794	16158	#N/A	1.000000E+00
Gm24795	16159	#N/A	1.000000E+00
Gm24796	16160	#N/A	1.000000E+00
Gm24797	16161	#N/A	1.000000E+00
Gm24798	16162	#N/A	1.000000E+00
Gm24799	16163	#N/A	1.000000E+00
Gm24800	16164	#N/A	1.000000E+00
Gm24801	16165	#N/A	1.000000E+00
Gm24802	16166	#N/A	1.000000E+00
Gm24803	16167	#N/A	1.000000E+00

Spearman Rank correlation analysis performed between Prdm1 and all-expressed genes within the Meredith RNA-seq dataset. Robust Prdm1–associated genes were identified using a cut-off of $p < 0.0005$.

Gm24804	16168		#N/A	1.000000E+00
Gm24805	16169		#N/A	1.000000E+00
Gm24806	16170		#N/A	1.000000E+00
Gm24807	16171		#N/A	1.000000E+00
Gm24808	16172		#N/A	1.000000E+00
Gm24809	16173		#N/A	1.000000E+00
Gm24810	16174		#N/A	1.000000E+00
Gm24811	16175		#N/A	1.000000E+00
Gm24812	16176	0.113548679	13094	1.093908E-01
Gm24813	16177		#N/A	1.000000E+00
Gm24814	16178		#N/A	1.000000E+00
Gm24815	16179		#N/A	1.000000E+00
Gm24816	16180		#N/A	1.000000E+00
Gm24817	16181		#N/A	1.000000E+00
Gm24818	16182		#N/A	1.000000E+00
Gm24819	16183		#N/A	1.000000E+00
Gm24820	16184		#N/A	1.000000E+00
Gm24822	16185		#N/A	1.000000E+00
Gm24823	16186		#N/A	1.000000E+00
Gm24824	16187		#N/A	1.000000E+00
Gm24825	16188		#N/A	1.000000E+00
Gm24826	16189		#N/A	1.000000E+00
Gm24827	16190		#N/A	1.000000E+00
Gm24828	16191		#N/A	1.000000E+00
Gm24829	16192		#N/A	1.000000E+00
Gm24830	16193		#N/A	1.000000E+00
Gm24831	16194		#N/A	1.000000E+00
Gm24832	16195		#N/A	1.000000E+00
Gm24833	16196		#N/A	1.000000E+00
Gm24834	16197		#N/A	1.000000E+00
Gm24835	16198		#N/A	1.000000E+00
Gm24836	16199		#N/A	1.000000E+00
Gm24837	16200		#N/A	1.000000E+00
Gm24838	16201		#N/A	1.000000E+00
Gm24839	16202		#N/A	1.000000E+00
Gm24840	16203		#N/A	1.000000E+00
Gm24841	16204		#N/A	1.000000E+00
Gm24842	16205		#N/A	1.000000E+00
Gm24843	16206		#N/A	1.000000E+00
Gm24844	16207		#N/A	1.000000E+00
Gm24845	16208		#N/A	1.000000E+00
Gm24846	16209		#N/A	1.000000E+00
Gm24847	16210		#N/A	1.000000E+00

Spearman Rank correlation analysis performed between Prdm1 and all-expressed genes within the Meredith RNA-seq dataset. Robust Prdm1–associated genes were identified using a cut-off of $p < 0.0005$.

Table S1, Related to Supplemental Figure 3C. Prdm1 associated genes

Gm24848	16211		#N/A	1.000000E+00
Gm24849	16212		#N/A	1.000000E+00
Gm24850	16213		#N/A	1.000000E+00
Gm24851	16214		#N/A	1.000000E+00
Gm24852	16215	0.053267784	8685	4.537786E-01
Gm24853	16216		#N/A	1.000000E+00
Gm24854	16217		#N/A	1.000000E+00
Gm24855	16218		#N/A	1.000000E+00
Gm24856	16219		#N/A	1.000000E+00
Gm24857	16220		#N/A	1.000000E+00
Gm24858	16221		#N/A	1.000000E+00
Gm24859	16222		#N/A	1.000000E+00
Gm24860	16223		#N/A	1.000000E+00
Gm24861	16224		#N/A	1.000000E+00
Gm24862	16225		#N/A	1.000000E+00
Gm24863	16226		#N/A	1.000000E+00
Gm24864	16227		#N/A	1.000000E+00
Gm24865	16228		#N/A	1.000000E+00
Gm24866	16229		#N/A	1.000000E+00
Gm24867	16230		#N/A	1.000000E+00
Gm24868	16231		#N/A	1.000000E+00
Gm24869	16232		#N/A	1.000000E+00
Gm24870	16233	-0.038406171	3065	5.892384E-01
Gm24871	16234		#N/A	1.000000E+00
Gm24872	16235		#N/A	1.000000E+00
Gm24873	16236		#N/A	1.000000E+00
Gm24874	16237		#N/A	1.000000E+00
Gm24875	16238		#N/A	1.000000E+00
Gm24876	16239		#N/A	1.000000E+00
Gm24877	16240		#N/A	1.000000E+00
Gm24878	16241		#N/A	1.000000E+00
Gm24879	16242		#N/A	1.000000E+00
Gm24880	16243		#N/A	1.000000E+00
Gm24881	16244		#N/A	1.000000E+00
Gm24882	16245		#N/A	1.000000E+00
Gm24883	16246		#N/A	1.000000E+00
Gm24884	16247		#N/A	1.000000E+00
Gm24885	16248		#N/A	1.000000E+00
Gm24886	16249		#N/A	1.000000E+00
Gm24887	16250		#N/A	1.000000E+00
Gm24888	16251		#N/A	1.000000E+00
Gm24889	16252		#N/A	1.000000E+00
Gm24890	16253		#N/A	1.000000E+00

Spearman Rank correlation analysis performed between Prdm1 and all-expressed genes within the Meredith RNA-seq dataset. Robust Prdm1–associated genes were identified using a cut-off of $p < 0.0005$.

Gm24891	16254		#N/A	1.000000E+00
Gm24892	16255		#N/A	1.000000E+00
Gm24893	16256		#N/A	1.000000E+00
Gm24894	16257		#N/A	1.000000E+00
Gm24895	16258		#N/A	1.000000E+00
Gm24896	16259		#N/A	1.000000E+00
Gm24897	16260		#N/A	1.000000E+00
Gm24898	16261		#N/A	1.000000E+00
Gm24899	16262		#N/A	1.000000E+00
Gm24900	16263		#N/A	1.000000E+00
Gm24901	16264		#N/A	1.000000E+00
Gm24902	16265		#N/A	1.000000E+00
Gm24903	16266		#N/A	1.000000E+00
Gm24904	16267		#N/A	1.000000E+00
Gm24905	16268	0.021317126	6513.5	7.644715E-01
Gm24906	16269		#N/A	1.000000E+00
Gm24907	16270		#N/A	1.000000E+00
Gm24908	16271		#N/A	1.000000E+00
Gm24909	16272		#N/A	1.000000E+00
Gm24910	16273		#N/A	1.000000E+00
Gm24911	16274		#N/A	1.000000E+00
Gm24912	16275		#N/A	1.000000E+00
Gm24913	16276		#N/A	1.000000E+00
Gm24914	16277		#N/A	1.000000E+00
Gm24915	16278		#N/A	1.000000E+00
Gm24916	16279		#N/A	1.000000E+00
Gm24917	16280		#N/A	1.000000E+00
Gm24918	16281		#N/A	1.000000E+00
Gm24919	16282		#N/A	1.000000E+00
Gm24920	16283		#N/A	1.000000E+00
Gm24921	16284		#N/A	1.000000E+00
Gm24922	16285		#N/A	1.000000E+00
Gm24923	16286		#N/A	1.000000E+00
Gm24924	16287		#N/A	1.000000E+00
Gm24925	16288		#N/A	1.000000E+00
Gm24926	16289		#N/A	1.000000E+00
Gm24927	16290		#N/A	1.000000E+00
Gm24928	16291		#N/A	1.000000E+00
Gm24929	16292		#N/A	1.000000E+00
Gm24930	16293		#N/A	1.000000E+00
Gm24931	16294		#N/A	1.000000E+00
Gm24932	16295		#N/A	1.000000E+00
Gm24933	16296		#N/A	1.000000E+00

Table S1, Related to Supplemental Figure 3C. Prdm1 associated genes

Gm24934	16297		#N/A	1.000000E+00
Gm24935	16298		#N/A	1.000000E+00
Gm24936	16299		#N/A	1.000000E+00
Gm24937	16300		#N/A	1.000000E+00
Gm24938	16301		#N/A	1.000000E+00
Gm24939	16302		#N/A	1.000000E+00
Gm24940	16303		#N/A	1.000000E+00
Gm24941	16304		#N/A	1.000000E+00
Gm24942	16305	0.113548679	13094	1.093908E-01
Gm24943	16306		#N/A	1.000000E+00
Gm24944	16307		#N/A	1.000000E+00
Gm24945	16308		#N/A	1.000000E+00
Gm24946	16309		#N/A	1.000000E+00
Gm24947	16310		#N/A	1.000000E+00
Gm24948	16311		#N/A	1.000000E+00
Gm24949	16312		#N/A	1.000000E+00
Gm24950	16313		#N/A	1.000000E+00
Gm24951	16314		#N/A	1.000000E+00
Gm24952	16315		#N/A	1.000000E+00
Gm24953	16316		#N/A	1.000000E+00
Gm24954	16317		#N/A	1.000000E+00
Gm24955	16318		#N/A	1.000000E+00
Gm24956	16319		#N/A	1.000000E+00
Gm24957	16320		#N/A	1.000000E+00
Gm24958	16321		#N/A	1.000000E+00
Gm24959	16322		#N/A	1.000000E+00
Gm24960	16323		#N/A	1.000000E+00
Gm24961	16324		#N/A	1.000000E+00
Gm24962	16325		#N/A	1.000000E+00
Gm24963	16326		#N/A	1.000000E+00
Gm24964	16327		#N/A	1.000000E+00
Gm24965	16328		#N/A	1.000000E+00
Gm24966	16329		#N/A	1.000000E+00
Gm24967	16330		#N/A	1.000000E+00
Gm24968	16331		#N/A	1.000000E+00
Gm24969	16332		#N/A	1.000000E+00
Gm24970	16333		#N/A	1.000000E+00
Gm24971	16334		#N/A	1.000000E+00
Gm24972	16335		#N/A	1.000000E+00
Gm24973	16336		#N/A	1.000000E+00
Gm24974	16337		#N/A	1.000000E+00
Gm24975	16338		#N/A	1.000000E+00
Gm24976	16339		#N/A	1.000000E+00

Spearman Rank correlation analysis performed between Prdm1 and all-expressed genes within the Meredith RNA-seq dataset. Robust Prdm1–associated genes were identified using a cut-off of $p < 0.0005$.

Table S1, Related to Supplemental Figure 3C. Prdm1 associated genes

Gm24977	16340	#N/A	1.000000E+00
Gm24978	16341	#N/A	1.000000E+00
Gm24979	16342	#N/A	1.000000E+00
Gm24980	16343	#N/A	1.000000E+00
Gm24981	16344	#N/A	1.000000E+00
Gm24982	16345	#N/A	1.000000E+00
Gm24983	16346	#N/A	1.000000E+00
Gm24984	16347	#N/A	1.000000E+00
Gm24985	16348	#N/A	1.000000E+00
Gm24986	16349	#N/A	1.000000E+00
Gm24987	16350	#N/A	1.000000E+00
Gm24988	16351	#N/A	1.000000E+00
Gm24989	16352	#N/A	1.000000E+00
Gm24990	16353	#N/A	1.000000E+00
Gm24991	16354	#N/A	1.000000E+00
Gm24992	16355	#N/A	1.000000E+00
Gm24993	16356	#N/A	1.000000E+00
Gm24994	16357	#N/A	1.000000E+00
Gm24995	16358	#N/A	1.000000E+00
Gm24996	16359	#N/A	1.000000E+00
Gm24997	16360	#N/A	1.000000E+00
Gm24998	16361	#N/A	1.000000E+00
Gm24999	16362	#N/A	1.000000E+00
Gm25000	16363	#N/A	1.000000E+00
Gm25001	16364	#N/A	1.000000E+00
Gm25002	16365	#N/A	1.000000E+00
Gm25003	16366	#N/A	1.000000E+00
Gm25004	16367	#N/A	1.000000E+00
Gm25005	16368	#N/A	1.000000E+00
Gm25006	16369	#N/A	1.000000E+00
Gm25007	16370	#N/A	1.000000E+00
Gm25008	16371	#N/A	1.000000E+00
Gm25009	16372	#N/A	1.000000E+00
Gm25010	16373	#N/A	1.000000E+00
Gm25011	16374	#N/A	1.000000E+00
Gm25012	16375	#N/A	1.000000E+00
Gm25013	16376	#N/A	1.000000E+00
Gm25014	16377	#N/A	1.000000E+00
Gm25015	16378	#N/A	1.000000E+00
Gm25016	16379	#N/A	1.000000E+00
Gm25017	16380	#N/A	1.000000E+00
Gm25018	16381	#N/A	1.000000E+00
Gm25019	16382	#N/A	1.000000E+00

Spearman Rank correlation analysis performed between Prdm1 and all-expressed genes within the Meredith RNA-seq dataset. Robust Prdm1–associated genes were identified using a cut-off of $p < 0.0005$.

Table S1, Related to Supplemental Figure 3C. Prdm1 associated genes

Gm25020	16383	#N/A	1.000000E+00
Gm25021	16384	#N/A	1.000000E+00
Gm25022	16385	#N/A	1.000000E+00
Gm25023	16386	#N/A	1.000000E+00
Gm25024	16387	#N/A	1.000000E+00
Gm25025	16388	#N/A	1.000000E+00
Gm25026	16389	#N/A	1.000000E+00
Gm25027	16390	#N/A	1.000000E+00
Gm25028	16391	#N/A	1.000000E+00
Gm25029	16392	#N/A	1.000000E+00
Gm25030	16393	#N/A	1.000000E+00
Gm25031	16394	#N/A	1.000000E+00
Gm25032	16395	#N/A	1.000000E+00
Gm25033	16396	#N/A	1.000000E+00
Gm25034	16397	#N/A	1.000000E+00
Gm25035	16398	#N/A	1.000000E+00
Gm25036	16399	#N/A	1.000000E+00
Gm25037	16400	#N/A	1.000000E+00
Gm25038	16401	#N/A	1.000000E+00
Gm25039	16402	#N/A	1.000000E+00
Gm25040	16403	#N/A	1.000000E+00
Gm25041	16404	#N/A	1.000000E+00
Gm25042	16405	#N/A	1.000000E+00
Gm25043	16406	#N/A	1.000000E+00
Gm25044	16407	#N/A	1.000000E+00
Gm25045	16408	#N/A	1.000000E+00
Gm25046	16409	#N/A	1.000000E+00
Gm25047	16410	#N/A	1.000000E+00
Gm25048	16411	#N/A	1.000000E+00
Gm25049	16412	#N/A	1.000000E+00
Gm25050	16413	#N/A	1.000000E+00
Gm25051	16414	#N/A	1.000000E+00
Gm25052	16415	#N/A	1.000000E+00
Gm25053	16416	#N/A	1.000000E+00
Gm25054	16417	#N/A	1.000000E+00
Gm25055	16418	#N/A	1.000000E+00
Gm25056	16419	#N/A	1.000000E+00
Gm25057	16420	#N/A	1.000000E+00
Gm25058	16421	#N/A	1.000000E+00
Gm25059	16422	#N/A	1.000000E+00
Gm25060	16423	#N/A	1.000000E+00
Gm25061	16424	#N/A	1.000000E+00
Gm25062	16425	#N/A	1.000000E+00

Spearman Rank correlation analysis performed between Prdm1 and all-expressed genes within the Meredith RNA-seq dataset. Robust Prdm1–associated genes were identified using a cut-off of $p < 0.0005$.

Gm25063	16426	#N/A	1.000000E+00
Gm25064	16427	#N/A	1.000000E+00
Gm25065	16428	#N/A	1.000000E+00
Gm25066	16429	#N/A	1.000000E+00
Gm25067	16430	#N/A	1.000000E+00
Gm25068	16431	#N/A	1.000000E+00
Gm25069	16432	#N/A	1.000000E+00
Gm25070	16433	#N/A	1.000000E+00
Gm25071	16434	#N/A	1.000000E+00
Gm25072	16435	#N/A	1.000000E+00
Gm25073	16436	#N/A	1.000000E+00
Gm25074	16437	#N/A	1.000000E+00
Gm25075	16438	#N/A	1.000000E+00
Gm25076	16439	#N/A	1.000000E+00
Gm25077	16440	#N/A	1.000000E+00
Gm25078	16441	#N/A	1.000000E+00
Gm25079	16442	#N/A	1.000000E+00
Gm25080	16443	#N/A	1.000000E+00
Gm25081	16444	#N/A	1.000000E+00
Gm25082	16445	#N/A	1.000000E+00
Gm25083	16446	#N/A	1.000000E+00
Gm25084	16447	#N/A	1.000000E+00
Gm25085	16448	#N/A	1.000000E+00
Gm25086	16449	#N/A	1.000000E+00
Gm25087	16450	#N/A	1.000000E+00
Gm25088	16451	#N/A	1.000000E+00
Gm25089	16452	#N/A	1.000000E+00
Gm25090	16453	#N/A	1.000000E+00
Gm25091	16454	#N/A	1.000000E+00
Gm25092	16455	#N/A	1.000000E+00
Gm25093	16456	#N/A	1.000000E+00
Gm25094	16457	#N/A	1.000000E+00
Gm25095	16458	#N/A	1.000000E+00
Gm25096	16459	#N/A	1.000000E+00
Gm25097	16460	#N/A	1.000000E+00
Gm25098	16461	#N/A	1.000000E+00
Gm25099	16462	#N/A	1.000000E+00
Gm25100	16463	#N/A	1.000000E+00
Gm25101	16464	#N/A	1.000000E+00
Gm25102	16465	#N/A	1.000000E+00
Gm25103	16466	#N/A	1.000000E+00
Gm25104	16467	#N/A	1.000000E+00
Gm25105	16468	#N/A	1.000000E+00

Table S1, Related to Supplemental Figure 3C. Prdm1 associated genes

Gm25106	16469		#N/A	1.000000E+00
Gm25107	16470		#N/A	1.000000E+00
Gm25108	16471		#N/A	1.000000E+00
Gm25109	16472		#N/A	1.000000E+00
Gm25110	16473		#N/A	1.000000E+00
Gm25111	16474		#N/A	1.000000E+00
Gm25112	16475		#N/A	1.000000E+00
Gm25113	16476		#N/A	1.000000E+00
Gm25114	16477		#N/A	1.000000E+00
Gm25115	16478		#N/A	1.000000E+00
Gm25116	16479		#N/A	1.000000E+00
Gm25117	16480		#N/A	1.000000E+00
Gm25118	16481		#N/A	1.000000E+00
Gm25119	16482		#N/A	1.000000E+00
Gm25120	16483		#N/A	1.000000E+00
Gm25121	16484		#N/A	1.000000E+00
Gm25122	16485		#N/A	1.000000E+00
Gm25123	16486		#N/A	1.000000E+00
Gm25124	16487		#N/A	1.000000E+00
Gm25125	16488		#N/A	1.000000E+00
Gm25126	16489		#N/A	1.000000E+00
Gm25127	16490		#N/A	1.000000E+00
Gm25128	16491		#N/A	1.000000E+00
Gm25129	16492		#N/A	1.000000E+00
Gm25130	16493		#N/A	1.000000E+00
Gm25131	16494		#N/A	1.000000E+00
Gm25132	16495		#N/A	1.000000E+00
Gm25133	16496		#N/A	1.000000E+00
Gm25134	16497		#N/A	1.000000E+00
Gm25135	16498		#N/A	1.000000E+00
Gm25136	16499		#N/A	1.000000E+00
Gm25137	16500		#N/A	1.000000E+00
Gm25138	16501		#N/A	1.000000E+00
Gm25139	16502	-0.038406171	3065	5.892384E-01
Gm25140	16503		#N/A	1.000000E+00
Gm25141	16504		#N/A	1.000000E+00
Gm25142	16505		#N/A	1.000000E+00
Gm25143	16506		#N/A	1.000000E+00
Gm25144	16507		#N/A	1.000000E+00
Gm25145	16508		#N/A	1.000000E+00
Gm25146	16509		#N/A	1.000000E+00
Gm25147	16510		#N/A	1.000000E+00
Gm25148	16511		#N/A	1.000000E+00

Spearman Rank correlation analysis performed between Prdm1 and all-expressed genes within the Meredith RNA-seq dataset. Robust Prdm1–associated genes were identified using a cut-off of $p < 0.0005$.

Table S1, Related to Supplemental Figure 3C. Prdm1 associated genes

Gm25149	16512	#N/A	1.000000E+00
Gm25150	16513	#N/A	1.000000E+00
Gm25151	16514	#N/A	1.000000E+00
Gm25152	16515	#N/A	1.000000E+00
Gm25153	16516	#N/A	1.000000E+00
Gm25154	16517	#N/A	1.000000E+00
Gm25155	16518	#N/A	1.000000E+00
Gm25156	16519	#N/A	1.000000E+00
Gm25157	16520	#N/A	1.000000E+00
Gm25158	16521	#N/A	1.000000E+00
Gm25159	16522	#N/A	1.000000E+00
Gm25160	16523	#N/A	1.000000E+00
Gm25161	16524	#N/A	1.000000E+00
Gm25162	16525	#N/A	1.000000E+00
Gm25163	16526	#N/A	1.000000E+00
Gm25164	16527	#N/A	1.000000E+00
Gm25165	16528	#N/A	1.000000E+00
Gm25166	16529	#N/A	1.000000E+00
Gm25167	16530	#N/A	1.000000E+00
Gm25168	16531	#N/A	1.000000E+00
Gm25169	16532	#N/A	1.000000E+00
Gm25170	16533	#N/A	1.000000E+00
Gm25171	16534	#N/A	1.000000E+00
Gm25172	16535	#N/A	1.000000E+00
Gm25173	16536	#N/A	1.000000E+00
Gm25174	16537	#N/A	1.000000E+00
Gm25175	16538	#N/A	1.000000E+00
Gm25176	16539	#N/A	1.000000E+00
Gm25177	16540	#N/A	1.000000E+00
Gm25178	16541	#N/A	1.000000E+00
Gm25179	16542	#N/A	1.000000E+00
Gm25180	16543	#N/A	1.000000E+00
Gm25181	16544	#N/A	1.000000E+00
Gm25182	16545	#N/A	1.000000E+00
Gm25183	16546	#N/A	1.000000E+00
Gm25184	16547	#N/A	1.000000E+00
Gm25185	16548	#N/A	1.000000E+00
Gm25186	16549	#N/A	1.000000E+00
Gm25187	16550	#N/A	1.000000E+00
Gm25188	16551	#N/A	1.000000E+00
Gm25189	16552	#N/A	1.000000E+00
Gm25190	16553	#N/A	1.000000E+00
Gm25191	16554	#N/A	1.000000E+00

Spearman Rank correlation analysis performed between Prdm1 and all-expressed genes within the Meredith RNA-seq dataset. Robust Prdm1–associated genes were identified using a cut-off of $p < 0.0005$.

Table S1, Related to Supplemental Figure 3C. Prdm1 associated genes

Gm25192	16555		#N/A	1.000000E+00
Gm25193	16556		#N/A	1.000000E+00
Gm25194	16557		#N/A	1.000000E+00
Gm25195	16558		#N/A	1.000000E+00
Gm25196	16559		#N/A	1.000000E+00
Gm25197	16560		#N/A	1.000000E+00
Gm25198	16561		#N/A	1.000000E+00
Gm25199	16562		#N/A	1.000000E+00
Gm25200	16563		#N/A	1.000000E+00
Gm25201	16564		#N/A	1.000000E+00
Gm25202	16565		#N/A	1.000000E+00
Gm25203	16566	0.084636591	10962	2.334264E-01
Gm25204	16567		#N/A	1.000000E+00
Gm25205	16568		#N/A	1.000000E+00
Gm25206	16569		#N/A	1.000000E+00
Gm25207	16570		#N/A	1.000000E+00
Gm25208	16571		#N/A	1.000000E+00
Gm25209	16572		#N/A	1.000000E+00
Gm25210	16573		#N/A	1.000000E+00
Gm25211	16574		#N/A	1.000000E+00
Gm25212	16575		#N/A	1.000000E+00
Gm25213	16576		#N/A	1.000000E+00
Gm25214	16577		#N/A	1.000000E+00
Gm25215	16578		#N/A	1.000000E+00
Gm25216	16579		#N/A	1.000000E+00
Gm25217	16580		#N/A	1.000000E+00
Gm25218	16581		#N/A	1.000000E+00
Gm25219	16582		#N/A	1.000000E+00
Gm25220	16583		#N/A	1.000000E+00
Gm25221	16584		#N/A	1.000000E+00
Gm25222	16585		#N/A	1.000000E+00
Gm25223	16586		#N/A	1.000000E+00
Gm25224	16587		#N/A	1.000000E+00
Gm25225	16588	0.030727477	7111	6.657920E-01
Gm25226	16589		#N/A	1.000000E+00
Gm25227	16590		#N/A	1.000000E+00
Gm25228	16591		#N/A	1.000000E+00
Gm25229	16592		#N/A	1.000000E+00
Gm25230	16593		#N/A	1.000000E+00
Gm25231	16594		#N/A	1.000000E+00
Gm25232	16595		#N/A	1.000000E+00
Gm25233	16596		#N/A	1.000000E+00
Gm25234	16597		#N/A	1.000000E+00

Spearman Rank correlation analysis performed between Prdm1 and all-expressed genes within the Meredith RNA-seq dataset. Robust Prdm1–associated genes were identified using a cut-off of $p < 0.0005$.

Table S1, Related to Supplemental Figure 3C. Prdm1 associated genes

Gm25235	16598		#N/A	1.000000E+00
Gm25236	16599		#N/A	1.000000E+00
Gm25237	16600		#N/A	1.000000E+00
Gm25238	16601	0.160304018	16323	2.335876E-02
Gm25239	16602		#N/A	1.000000E+00
Gm25240	16603		#N/A	1.000000E+00
Gm25241	16604		#N/A	1.000000E+00
Gm25242	16605		#N/A	1.000000E+00
Gm25243	16606		#N/A	1.000000E+00
Gm25244	16607		#N/A	1.000000E+00
Gm25245	16608		#N/A	1.000000E+00
Gm25246	16609		#N/A	1.000000E+00
Gm25247	16610		#N/A	1.000000E+00
Gm25248	16611	-0.038406171	3065	5.892384E-01
Gm25249	16612		#N/A	1.000000E+00
Gm25250	16613		#N/A	1.000000E+00
Gm25251	16614		#N/A	1.000000E+00
Gm25252	16615		#N/A	1.000000E+00
Gm25253	16616		#N/A	1.000000E+00
Gm25254	16617		#N/A	1.000000E+00
Gm25255	16618		#N/A	1.000000E+00
Gm25256	16619		#N/A	1.000000E+00
Gm25257	16620		#N/A	1.000000E+00
Gm25258	16621		#N/A	1.000000E+00
Gm25259	16622		#N/A	1.000000E+00
Gm25260	16623		#N/A	1.000000E+00
Gm25261	16624		#N/A	1.000000E+00
Gm25262	16625		#N/A	1.000000E+00
Gm25263	16626		#N/A	1.000000E+00
Gm25264	16627		#N/A	1.000000E+00
Gm25265	16628		#N/A	1.000000E+00
Gm25266	16629		#N/A	1.000000E+00
Gm25267	16630		#N/A	1.000000E+00
Gm25268	16631		#N/A	1.000000E+00
Gm25269	16632		#N/A	1.000000E+00
Gm25270	16633		#N/A	1.000000E+00
Gm25271	16634		#N/A	1.000000E+00
Gm25272	16635		#N/A	1.000000E+00
Gm25273	16636		#N/A	1.000000E+00
Gm25274	16637		#N/A	1.000000E+00
Gm25275	16638		#N/A	1.000000E+00
Gm25276	16639		#N/A	1.000000E+00
Gm25277	16640		#N/A	1.000000E+00

Spearman Rank correlation analysis performed between Prdm1 and all-expressed genes within the Meredith RNA-seq dataset. Robust Prdm1–associated genes were identified using a cut-off of $p < 0.0005$.

Table S1, Related to Supplemental Figure 3C. Prdm1 associated genes

Gm25278	16641		#N/A	1.000000E+00
Gm25279	16642		#N/A	1.000000E+00
Gm25280	16643		#N/A	1.000000E+00
Gm25281	16644		#N/A	1.000000E+00
Gm25282	16645		#N/A	1.000000E+00
Gm25283	16646		#N/A	1.000000E+00
Gm25284	16647	0.212479273	18674.5	2.522377E-03
Gm25285	16648		#N/A	1.000000E+00
Gm25286	16649		#N/A	1.000000E+00
Gm25287	16650		#N/A	1.000000E+00
Gm25288	16651		#N/A	1.000000E+00
Gm25289	16652		#N/A	1.000000E+00
Gm25290	16653		#N/A	1.000000E+00
Gm25291	16654		#N/A	1.000000E+00
Gm25292	16655		#N/A	1.000000E+00
Gm25293	16656		#N/A	1.000000E+00
Gm25294	16657		#N/A	1.000000E+00
Gm25295	16658		#N/A	1.000000E+00
Gm25296	16659		#N/A	1.000000E+00
Gm25297	16660		#N/A	1.000000E+00
Gm25298	16661		#N/A	1.000000E+00
Gm25299	16662		#N/A	1.000000E+00
Gm25300	16663		#N/A	1.000000E+00
Gm25301	16664		#N/A	1.000000E+00
Gm25302	16665		#N/A	1.000000E+00
Gm25303	16666		#N/A	1.000000E+00
Gm25304	16667		#N/A	1.000000E+00
Gm25305	16668		#N/A	1.000000E+00
Gm25306	16669		#N/A	1.000000E+00
Gm25307	16670		#N/A	1.000000E+00
Gm25308	16671		#N/A	1.000000E+00
Gm25309	16672		#N/A	1.000000E+00
Gm2531	16673		#N/A	1.000000E+00
Gm25310	16674		#N/A	1.000000E+00
Gm25311	16675		#N/A	1.000000E+00
Gm25312	16676		#N/A	1.000000E+00
Gm25313	16677		#N/A	1.000000E+00
Gm25314	16678		#N/A	1.000000E+00
Gm25315	16679		#N/A	1.000000E+00
Gm25316	16680		#N/A	1.000000E+00
Gm25317	16681	0.073928833	10125	2.981622E-01
Gm25318	16682		#N/A	1.000000E+00
Gm25319	16683		#N/A	1.000000E+00

Spearman Rank correlation analysis performed between Prdm1 and all-expressed genes within the Meredith RNA-seq dataset. Robust Prdm1–associated genes were identified using a cut-off of $p < 0.0005$.

Table S1, Related to Supplemental Figure 3C. Prdm1 associated genes

Gm25320	16684		#N/A	1.000000E+00
Gm25321	16685		#N/A	1.000000E+00
Gm25322	16686		#N/A	1.000000E+00
Gm25323	16687		#N/A	1.000000E+00
Gm25324	16688		#N/A	1.000000E+00
Gm25325	16689		#N/A	1.000000E+00
Gm25326	16690		#N/A	1.000000E+00
Gm25327	16691		#N/A	1.000000E+00
Gm25328	16692		#N/A	1.000000E+00
Gm25329	16693		#N/A	1.000000E+00
Gm25330	16694		#N/A	1.000000E+00
Gm25331	16695		#N/A	1.000000E+00
Gm25332	16696		#N/A	1.000000E+00
Gm25333	16697		#N/A	1.000000E+00
Gm25334	16698		#N/A	1.000000E+00
Gm25335	16699		#N/A	1.000000E+00
Gm25336	16700		#N/A	1.000000E+00
Gm25337	16701		#N/A	1.000000E+00
Gm25338	16702		#N/A	1.000000E+00
Gm25339	16703		#N/A	1.000000E+00
Gm25340	16704		#N/A	1.000000E+00
Gm25341	16705		#N/A	1.000000E+00
Gm25342	16706		#N/A	1.000000E+00
Gm25343	16707		#N/A	1.000000E+00
Gm25344	16708		#N/A	1.000000E+00
Gm25345	16709		#N/A	1.000000E+00
Gm25346	16710		#N/A	1.000000E+00
Gm25347	16711		#N/A	1.000000E+00
Gm25348	16712		#N/A	1.000000E+00
Gm25349	16713		#N/A	1.000000E+00
Gm25350	16714		#N/A	1.000000E+00
Gm25351	16715		#N/A	1.000000E+00
Gm25352	16716		#N/A	1.000000E+00
Gm25353	16717		#N/A	1.000000E+00
Gm25354	16718		#N/A	1.000000E+00
Gm25355	16719		#N/A	1.000000E+00
Gm25356	16720		#N/A	1.000000E+00
Gm25357	16721		#N/A	1.000000E+00
Gm25358	16722		#N/A	1.000000E+00
Gm25359	16723		#N/A	1.000000E+00
Gm25360	16724	0.143605682	15224	4.249002E-02
Gm25361	16725		#N/A	1.000000E+00
Gm25362	16726		#N/A	1.000000E+00

Spearman Rank correlation analysis performed between Prdm1 and all-expressed genes within the Meredith RNA-seq dataset. Robust Prdm1–associated genes were identified using a cut-off of $p < 0.0005$.

Gm25363	16727	#N/A	1.000000E+00
Gm25364	16728	#N/A	1.000000E+00
Gm25365	16729	#N/A	1.000000E+00
Gm25366	16730	#N/A	1.000000E+00
Gm25367	16731	#N/A	1.000000E+00
Gm25368	16732	#N/A	1.000000E+00
Gm25369	16733	#N/A	1.000000E+00
Gm25370	16734	#N/A	1.000000E+00
Gm25371	16735	#N/A	1.000000E+00
Gm25372	16736	#N/A	1.000000E+00
Gm25373	16737	#N/A	1.000000E+00
Gm25374	16738	#N/A	1.000000E+00
Gm25375	16739	#N/A	1.000000E+00
Gm25376	16740	#N/A	1.000000E+00
Gm25377	16741	#N/A	1.000000E+00
Gm25378	16742	#N/A	1.000000E+00
Gm25379	16743	#N/A	1.000000E+00
Gm25380	16744	#N/A	1.000000E+00
Gm25381	16745	#N/A	1.000000E+00
Gm25382	16746	#N/A	1.000000E+00
Gm25383	16747	#N/A	1.000000E+00
Gm25384	16748	#N/A	1.000000E+00
Gm25385	16749	#N/A	1.000000E+00
Gm25386	16750	#N/A	1.000000E+00
Gm25387	16751	#N/A	1.000000E+00
Gm25388	16752	#N/A	1.000000E+00
Gm25389	16753	#N/A	1.000000E+00
Gm25390	16754	#N/A	1.000000E+00
Gm25391	16755	#N/A	1.000000E+00
Gm25392	16756	#N/A	1.000000E+00
Gm25393	16757	#N/A	1.000000E+00
Gm25394	16758	#N/A	1.000000E+00
Gm25395	16759	#N/A	1.000000E+00
Gm25396	16760	#N/A	1.000000E+00
Gm25397	16761	#N/A	1.000000E+00
Gm25398	16762	#N/A	1.000000E+00
Gm25399	16763	#N/A	1.000000E+00
Gm25400	16764	#N/A	1.000000E+00
Gm25401	16765	#N/A	1.000000E+00
Gm25402	16766	#N/A	1.000000E+00
Gm25403	16767	#N/A	1.000000E+00
Gm25404	16768	#N/A	1.000000E+00
Gm25405	16769	#N/A	1.000000E+00

Spearman Rank correlation analysis performed between Prdm1 and all-expressed genes within the Meredith RNA-seq dataset. Robust Prdm1–associated genes were identified using a cut-off of $p < 0.0005$.

Table S1, Related to Supplemental Figure 3C. Prdm1 associated genes

Gm25406	16770	#N/A	1.000000E+00
Gm25407	16771	#N/A	1.000000E+00
Gm25408	16772	#N/A	1.000000E+00
Gm25409	16773	#N/A	1.000000E+00
Gm2541	16774	#N/A	1.000000E+00
Gm25410	16775	#N/A	1.000000E+00
Gm25411	16776	#N/A	1.000000E+00
Gm25412	16777	#N/A	1.000000E+00
Gm25413	16778	#N/A	1.000000E+00
Gm25414	16779	#N/A	1.000000E+00
Gm25415	16780	#N/A	1.000000E+00
Gm25416	16781	#N/A	1.000000E+00
Gm25417	16782	#N/A	1.000000E+00
Gm25418	16783	#N/A	1.000000E+00
Gm25419	16784	#N/A	1.000000E+00
Gm25420	16785	#N/A	1.000000E+00
Gm25421	16786	#N/A	1.000000E+00
Gm25422	16787	#N/A	1.000000E+00
Gm25423	16788	#N/A	1.000000E+00
Gm25424	16789	#N/A	1.000000E+00
Gm25425	16790	#N/A	1.000000E+00
Gm25426	16791	#N/A	1.000000E+00
Gm25427	16792	#N/A	1.000000E+00
Gm25428	16793	#N/A	1.000000E+00
Gm25429	16794	#N/A	1.000000E+00
Gm25430	16795	#N/A	1.000000E+00
Gm25431	16796	#N/A	1.000000E+00
Gm25432	16797	#N/A	1.000000E+00
Gm25433	16798	#N/A	1.000000E+00
Gm25434	16799	#N/A	1.000000E+00
Gm25435	16800	#N/A	1.000000E+00
Gm25436	16801	#N/A	1.000000E+00
Gm25437	16802	#N/A	1.000000E+00
Gm25438	16803	#N/A	1.000000E+00
Gm25439	16804	#N/A	1.000000E+00
Gm25440	16805	#N/A	1.000000E+00
Gm25441	16806	#N/A	1.000000E+00
Gm25442	16807	#N/A	1.000000E+00
Gm25443	16808	#N/A	1.000000E+00
Gm25444	16809	#N/A	1.000000E+00
Gm25445	16810	#N/A	1.000000E+00
Gm25446	16811	#N/A	1.000000E+00
Gm25447	16812	#N/A	1.000000E+00

Spearman Rank correlation analysis performed between Prdm1 and all-expressed genes within the Meredith RNA-seq dataset. Robust Prdm1–associated genes were identified using a cut-off of $p < 0.0005$.

Gm25448	16813	#N/A	1.000000E+00
Gm25449	16814	#N/A	1.000000E+00
Gm25450	16815	#N/A	1.000000E+00
Gm25451	16816	#N/A	1.000000E+00
Gm25452	16817	#N/A	1.000000E+00
Gm25453	16818	#N/A	1.000000E+00
Gm25454	16819	#N/A	1.000000E+00
Gm25455	16820	#N/A	1.000000E+00
Gm25456	16821	#N/A	1.000000E+00
Gm25457	16822	#N/A	1.000000E+00
Gm25458	16823	#N/A	1.000000E+00
Gm25459	16824	#N/A	1.000000E+00
Gm25460	16825	#N/A	1.000000E+00
Gm25461	16826	#N/A	1.000000E+00
Gm25462	16827	#N/A	1.000000E+00
Gm25463	16828	#N/A	1.000000E+00
Gm25464	16829	#N/A	1.000000E+00
Gm25465	16830	#N/A	1.000000E+00
Gm25466	16831	#N/A	1.000000E+00
Gm25467	16832	#N/A	1.000000E+00
Gm25468	16833	#N/A	1.000000E+00
Gm25469	16834	#N/A	1.000000E+00
Gm25470	16835	#N/A	1.000000E+00
Gm25471	16836	#N/A	1.000000E+00
Gm25472	16837	#N/A	1.000000E+00
Gm25473	16838	#N/A	1.000000E+00
Gm25474	16839	#N/A	1.000000E+00
Gm25475	16840	#N/A	1.000000E+00
Gm25476	16841	#N/A	1.000000E+00
Gm25477	16842	#N/A	1.000000E+00
Gm25478	16843	#N/A	1.000000E+00
Gm25479	16844	#N/A	1.000000E+00
Gm25480	16845	#N/A	1.000000E+00
Gm25481	16846	#N/A	1.000000E+00
Gm25482	16847	#N/A	1.000000E+00
Gm25483	16848	#N/A	1.000000E+00
Gm25484	16849	#N/A	1.000000E+00
Gm25485	16850	#N/A	1.000000E+00
Gm25486	16851	#N/A	1.000000E+00
Gm25487	16852	#N/A	1.000000E+00
Gm25488	16853	#N/A	1.000000E+00
Gm25489	16854	#N/A	1.000000E+00
Gm25490	16855	#N/A	1.000000E+00

Gm25491	16856		#N/A	1.000000E+00
Gm25492	16857		#N/A	1.000000E+00
Gm25493	16858		#N/A	1.000000E+00
Gm25494	16859		#N/A	1.000000E+00
Gm25495	16860		#N/A	1.000000E+00
Gm25496	16861		#N/A	1.000000E+00
Gm25497	16862		#N/A	1.000000E+00
Gm25498	16863		#N/A	1.000000E+00
Gm25499	16864		#N/A	1.000000E+00
Gm25500	16865		#N/A	1.000000E+00
Gm25501	16866		#N/A	1.000000E+00
Gm25502	16867		#N/A	1.000000E+00
Gm25503	16868		#N/A	1.000000E+00
Gm25504	16869		#N/A	1.000000E+00
Gm25505	16870		#N/A	1.000000E+00
Gm25506	16871		#N/A	1.000000E+00
Gm25507	16872		#N/A	1.000000E+00
Gm25508	16873		#N/A	1.000000E+00
Gm25509	16874		#N/A	1.000000E+00
Gm25510	16875		#N/A	1.000000E+00
Gm25511	16876		#N/A	1.000000E+00
Gm25512	16877		#N/A	1.000000E+00
Gm25513	16878		#N/A	1.000000E+00
Gm25514	16879	-0.038406171	3065	5.892384E-01
Gm25515	16880		#N/A	1.000000E+00
Gm25516	16881		#N/A	1.000000E+00
Gm25517	16882		#N/A	1.000000E+00
Gm25518	16883		#N/A	1.000000E+00
Gm25519	16884		#N/A	1.000000E+00
Gm25520	16885		#N/A	1.000000E+00
Gm25521	16886		#N/A	1.000000E+00
Gm25522	16887		#N/A	1.000000E+00
Gm25523	16888		#N/A	1.000000E+00
Gm25524	16889		#N/A	1.000000E+00
Gm25525	16890		#N/A	1.000000E+00
Gm25526	16891		#N/A	1.000000E+00
Gm25527	16892		#N/A	1.000000E+00
Gm25528	16893		#N/A	1.000000E+00
Gm25529	16894		#N/A	1.000000E+00
Gm25530	16895		#N/A	1.000000E+00
Gm25531	16896		#N/A	1.000000E+00
Gm25532	16897		#N/A	1.000000E+00
Gm25533	16898		#N/A	1.000000E+00

Table S1, Related to Supplemental Figure 3C. Prdm1 associated genes

Gm25534	16899	#N/A	1.000000E+00
Gm25535	16900	#N/A	1.000000E+00
Gm25536	16901	#N/A	1.000000E+00
Gm25537	16902	#N/A	1.000000E+00
Gm25538	16903	#N/A	1.000000E+00
Gm25539	16904	#N/A	1.000000E+00
Gm25540	16905	#N/A	1.000000E+00
Gm25541	16906	#N/A	1.000000E+00
Gm25542	16907	#N/A	1.000000E+00
Gm25543	16908	#N/A	1.000000E+00
Gm25544	16909	#N/A	1.000000E+00
Gm25545	16910	#N/A	1.000000E+00
Gm25546	16911	#N/A	1.000000E+00
Gm25547	16912	#N/A	1.000000E+00
Gm25548	16913	#N/A	1.000000E+00
Gm25549	16914	#N/A	1.000000E+00
Gm25550	16915	#N/A	1.000000E+00
Gm25551	16916	#N/A	1.000000E+00
Gm25552	16917	#N/A	1.000000E+00
Gm25553	16918	#N/A	1.000000E+00
Gm25554	16919	#N/A	1.000000E+00
Gm25555	16920	#N/A	1.000000E+00
Gm25556	16921	#N/A	1.000000E+00
Gm25557	16922	#N/A	1.000000E+00
Gm25558	16923	#N/A	1.000000E+00
Gm25559	16924	#N/A	1.000000E+00
Gm25560	16925	#N/A	1.000000E+00
Gm25561	16926	#N/A	1.000000E+00
Gm25562	16927	#N/A	1.000000E+00
Gm25563	16928	#N/A	1.000000E+00
Gm25564	16929	#N/A	1.000000E+00
Gm25565	16930	#N/A	1.000000E+00
Gm25566	16931	#N/A	1.000000E+00
Gm25567	16932	#N/A	1.000000E+00
Gm25568	16933	#N/A	1.000000E+00
Gm25569	16934	#N/A	1.000000E+00
Gm25570	16935	#N/A	1.000000E+00
Gm25571	16936	#N/A	1.000000E+00
Gm25572	16937	#N/A	1.000000E+00
Gm25573	16938	#N/A	1.000000E+00
Gm25574	16939	#N/A	1.000000E+00
Gm25575	16940	#N/A	1.000000E+00
Gm25576	16941	#N/A	1.000000E+00

Spearman Rank correlation analysis performed between Prdm1 and all-expressed genes within the Meredith RNA-seq dataset. Robust Prdm1–associated genes were identified using a cut-off of $p < 0.0005$.

Table S1, Related to Supplemental Figure 3C. Prdm1 associated genes

Gm25577	16942		#N/A	1.000000E+00
Gm25578	16943		#N/A	1.000000E+00
Gm25579	16944		#N/A	1.000000E+00
Gm25580	16945	0.053267784	8685	4.537786E-01
Gm25581	16946	0.053267784	8685	4.537786E-01
Gm25582	16947		#N/A	1.000000E+00
Gm25583	16948		#N/A	1.000000E+00
Gm25584	16949		#N/A	1.000000E+00
Gm25585	16950		#N/A	1.000000E+00
Gm25586	16951		#N/A	1.000000E+00
Gm25587	16952	-0.038406171	3065	5.892384E-01
Gm25588	16953		#N/A	1.000000E+00
Gm25589	16954		#N/A	1.000000E+00
Gm25590	16955		#N/A	1.000000E+00
Gm25591	16956		#N/A	1.000000E+00
Gm25592	16957		#N/A	1.000000E+00
Gm25593	16958	0.160304018	16323	2.335876E-02
Gm25594	16959		#N/A	1.000000E+00
Gm25595	16960	-0.054451513	1703	4.437940E-01
Gm25596	16961		#N/A	1.000000E+00
Gm25597	16962		#N/A	1.000000E+00
Gm25598	16963		#N/A	1.000000E+00
Gm25599	16964		#N/A	1.000000E+00
Gm25600	16965		#N/A	1.000000E+00
Gm25601	16966		#N/A	1.000000E+00
Gm25602	16967		#N/A	1.000000E+00
Gm25603	16968	-0.038406171	3065	5.892384E-01
Gm25604	16969		#N/A	1.000000E+00
Gm25605	16970		#N/A	1.000000E+00
Gm25606	16971		#N/A	1.000000E+00
Gm25607	16972		#N/A	1.000000E+00
Gm25608	16973		#N/A	1.000000E+00
Gm25609	16974		#N/A	1.000000E+00
Gm25610	16975		#N/A	1.000000E+00
Gm25611	16976		#N/A	1.000000E+00
Gm25612	16977		#N/A	1.000000E+00
Gm25613	16978		#N/A	1.000000E+00
Gm25614	16979		#N/A	1.000000E+00
Gm25615	16980		#N/A	1.000000E+00
Gm25616	16981		#N/A	1.000000E+00
Gm25617	16982		#N/A	1.000000E+00
Gm25618	16983		#N/A	1.000000E+00
Gm25619	16984		#N/A	1.000000E+00

Spearman Rank correlation analysis performed between Prdm1 and all-expressed genes within the Meredith RNA-seq dataset. Robust Prdm1–associated genes were identified using a cut-off of $p < 0.0005$.

Table S1, Related to Supplemental Figure 3C. Prdm1 associated genes

Gm25620	16985	#N/A	1.000000E+00
Gm25621	16986	#N/A	1.000000E+00
Gm25622	16987	#N/A	1.000000E+00
Gm25623	16988	#N/A	1.000000E+00
Gm25624	16989	#N/A	1.000000E+00
Gm25625	16990	#N/A	1.000000E+00
Gm25626	16991	#N/A	1.000000E+00
Gm25627	16992	#N/A	1.000000E+00
Gm25628	16993	#N/A	1.000000E+00
Gm25629	16994	#N/A	1.000000E+00
Gm25630	16995	#N/A	1.000000E+00
Gm25631	16996	#N/A	1.000000E+00
Gm25632	16997	#N/A	1.000000E+00
Gm25633	16998	#N/A	1.000000E+00
Gm25634	16999	#N/A	1.000000E+00
Gm25635	17000	#N/A	1.000000E+00
Gm25636	17001	#N/A	1.000000E+00
Gm25637	17002	#N/A	1.000000E+00
Gm25638	17003	#N/A	1.000000E+00
Gm25639	17004	#N/A	1.000000E+00
Gm2564	17005	#N/A	1.000000E+00
Gm25640	17006	#N/A	1.000000E+00
Gm25641	17007	#N/A	1.000000E+00
Gm25642	17008	#N/A	1.000000E+00
Gm25643	17009	#N/A	1.000000E+00
Gm25644	17010	#N/A	1.000000E+00
Gm25645	17011	#N/A	1.000000E+00
Gm25646	17012	#N/A	1.000000E+00
Gm25647	17013	#N/A	1.000000E+00
Gm25648	17014	#N/A	1.000000E+00
Gm25649	17015	#N/A	1.000000E+00
Gm25650	17016	#N/A	1.000000E+00
Gm25651	17017	#N/A	1.000000E+00
Gm25652	17018	#N/A	1.000000E+00
Gm25653	17019	#N/A	1.000000E+00
Gm25654	17020	#N/A	1.000000E+00
Gm25655	17021	#N/A	1.000000E+00
Gm25656	17022	#N/A	1.000000E+00
Gm25657	17023	#N/A	1.000000E+00
Gm25658	17024	#N/A	1.000000E+00
Gm25659	17025	#N/A	1.000000E+00
Gm2566	17026	#N/A	1.000000E+00
Gm25660	17027	#N/A	1.000000E+00

Spearman Rank correlation analysis performed between Prdm1 and all-expressed genes within the Meredith RNA-seq dataset. Robust Prdm1–associated genes were identified using a cut-off of $p < 0.0005$.

Table S1, Related to Supplemental Figure 3C. Prdm1 associated genes

Gm25661	17028		#N/A	1.000000E+00
Gm25662	17029		#N/A	1.000000E+00
Gm25663	17030		#N/A	1.000000E+00
Gm25664	17031		#N/A	1.000000E+00
Gm25665	17032		#N/A	1.000000E+00
Gm25666	17033		#N/A	1.000000E+00
Gm25667	17034		#N/A	1.000000E+00
Gm25668	17035		#N/A	1.000000E+00
Gm25669	17036		#N/A	1.000000E+00
Gm25670	17037		#N/A	1.000000E+00
Gm25671	17038		#N/A	1.000000E+00
Gm25672	17039		#N/A	1.000000E+00
Gm25673	17040		#N/A	1.000000E+00
Gm25674	17041		#N/A	1.000000E+00
Gm25675	17042		#N/A	1.000000E+00
Gm25676	17043		#N/A	1.000000E+00
Gm25677	17044		#N/A	1.000000E+00
Gm25678	17045		#N/A	1.000000E+00
Gm25679	17046		#N/A	1.000000E+00
Gm2568	17047		#N/A	1.000000E+00
Gm25680	17048		#N/A	1.000000E+00
Gm25681	17049		#N/A	1.000000E+00
Gm25682	17050	-0.038406171	3065	5.892384E-01
Gm25683	17051		#N/A	1.000000E+00
Gm25684	17052		#N/A	1.000000E+00
Gm25685	17053		#N/A	1.000000E+00
Gm25686	17054		#N/A	1.000000E+00
Gm25687	17055		#N/A	1.000000E+00
Gm25688	17056		#N/A	1.000000E+00
Gm25689	17057		#N/A	1.000000E+00
Gm25690	17058		#N/A	1.000000E+00
Gm25691	17059	0.053267784	8685	4.537786E-01
Gm25692	17060		#N/A	1.000000E+00
Gm25693	17061		#N/A	1.000000E+00
Gm25694	17062		#N/A	1.000000E+00
Gm25695	17063		#N/A	1.000000E+00
Gm25696	17064		#N/A	1.000000E+00
Gm25697	17065		#N/A	1.000000E+00
Gm25698	17066		#N/A	1.000000E+00
Gm25699	17067		#N/A	1.000000E+00
Gm25700	17068		#N/A	1.000000E+00
Gm25701	17069		#N/A	1.000000E+00
Gm25702	17070		#N/A	1.000000E+00

Spearman Rank correlation analysis performed between Prdm1 and all-expressed genes within the Meredith RNA-seq dataset. Robust Prdm1–associated genes were identified using a cut-off of $p < 0.0005$.

Table S1, Related to Supplemental Figure 3C. Prdm1 associated genes

Gm25703	17071		#N/A	1.000000E+00
Gm25704	17072		#N/A	1.000000E+00
Gm25705	17073		#N/A	1.000000E+00
Gm25706	17074		#N/A	1.000000E+00
Gm25707	17075		#N/A	1.000000E+00
Gm25708	17076		#N/A	1.000000E+00
Gm25709	17077		#N/A	1.000000E+00
Gm25710	17078		#N/A	1.000000E+00
Gm25711	17079		#N/A	1.000000E+00
Gm25712	17080		#N/A	1.000000E+00
Gm25713	17081		#N/A	1.000000E+00
Gm25714	17082		#N/A	1.000000E+00
Gm25715	17083		#N/A	1.000000E+00
Gm25716	17084		#N/A	1.000000E+00
Gm25717	17085		#N/A	1.000000E+00
Gm25718	17086		#N/A	1.000000E+00
Gm25719	17087		#N/A	1.000000E+00
Gm25720	17088		#N/A	1.000000E+00
Gm25721	17089		#N/A	1.000000E+00
Gm25722	17090		#N/A	1.000000E+00
Gm25723	17091		#N/A	1.000000E+00
Gm25724	17092		#N/A	1.000000E+00
Gm25725	17093		#N/A	1.000000E+00
Gm25726	17094		#N/A	1.000000E+00
Gm25727	17095		#N/A	1.000000E+00
Gm25728	17096		#N/A	1.000000E+00
Gm25729	17097		#N/A	1.000000E+00
Gm25730	17098		#N/A	1.000000E+00
Gm25731	17099		#N/A	1.000000E+00
Gm25732	17100		#N/A	1.000000E+00
Gm25733	17101		#N/A	1.000000E+00
Gm25734	17102		#N/A	1.000000E+00
Gm25735	17103	0.152789767	15822	3.077826E-02
Gm25736	17104		#N/A	1.000000E+00
Gm25737	17105		#N/A	1.000000E+00
Gm25738	17106		#N/A	1.000000E+00
Gm25739	17107		#N/A	1.000000E+00
Gm2574	17108		#N/A	1.000000E+00
Gm25740	17109		#N/A	1.000000E+00
Gm25741	17110		#N/A	1.000000E+00
Gm25742	17111		#N/A	1.000000E+00
Gm25743	17112		#N/A	1.000000E+00
Gm25744	17113		#N/A	1.000000E+00

Spearman Rank correlation analysis performed between Prdm1 and all-expressed genes within the Meredith RNA-seq dataset. Robust Prdm1–associated genes were identified using a cut-off of $p < 0.0005$.

Table S1, Related to Supplemental Figure 3C. Prdm1 associated genes

Gm25745	17114	0.020463014	6369	7.736476E-01
Gm25746	17115	-0.038406171	3065	5.892384E-01
Gm25747	17116		#N/A	1.000000E+00
Gm25748	17117		#N/A	1.000000E+00
Gm25749	17118		#N/A	1.000000E+00
Gm25750	17119		#N/A	1.000000E+00
Gm25751	17120		#N/A	1.000000E+00
Gm25752	17121		#N/A	1.000000E+00
Gm25753	17122		#N/A	1.000000E+00
Gm25754	17123		#N/A	1.000000E+00
Gm25755	17124		#N/A	1.000000E+00
Gm25756	17125		#N/A	1.000000E+00
Gm25757	17126		#N/A	1.000000E+00
Gm25758	17127		#N/A	1.000000E+00
Gm25759	17128		#N/A	1.000000E+00
Gm25760	17129		#N/A	1.000000E+00
Gm25761	17130		#N/A	1.000000E+00
Gm25762	17131		#N/A	1.000000E+00
Gm25763	17132		#N/A	1.000000E+00
Gm25764	17133		#N/A	1.000000E+00
Gm25765	17134		#N/A	1.000000E+00
Gm25766	17135		#N/A	1.000000E+00
Gm25767	17136		#N/A	1.000000E+00
Gm25768	17137		#N/A	1.000000E+00
Gm25769	17138		#N/A	1.000000E+00
Gm25770	17139		#N/A	1.000000E+00
Gm25771	17140		#N/A	1.000000E+00
Gm25772	17141		#N/A	1.000000E+00
Gm25773	17142		#N/A	1.000000E+00
Gm25774	17143		#N/A	1.000000E+00
Gm25775	17144		#N/A	1.000000E+00
Gm25776	17145		#N/A	1.000000E+00
Gm25777	17146		#N/A	1.000000E+00
Gm25778	17147		#N/A	1.000000E+00
Gm25779	17148		#N/A	1.000000E+00
Gm25780	17149		#N/A	1.000000E+00
Gm25781	17150	-0.038406171	3065	5.892384E-01
Gm25782	17151		#N/A	1.000000E+00
Gm25783	17152		#N/A	1.000000E+00
Gm25784	17153	0.097880814	11904	1.679316E-01
Gm25785	17154		#N/A	1.000000E+00
Gm25786	17155		#N/A	1.000000E+00
Gm25787	17156		#N/A	1.000000E+00

Spearman Rank correlation analysis performed between Prdm1 and all-expressed genes within the Meredith RNA-seq dataset. Robust Prdm1–associated genes were identified using a cut-off of $p < 0.0005$.

Table S1, Related to Supplemental Figure 3C. Prdm1 associated genes

Gm25788	17157		#N/A	1.000000E+00
Gm25789	17158	0.086624598	11111	2.225892E-01
Gm25790	17159		#N/A	1.000000E+00
Gm25791	17160		#N/A	1.000000E+00
Gm25792	17161		#N/A	1.000000E+00
Gm25793	17162		#N/A	1.000000E+00
Gm25794	17163		#N/A	1.000000E+00
Gm25795	17164		#N/A	1.000000E+00
Gm25796	17165		#N/A	1.000000E+00
Gm25797	17166		#N/A	1.000000E+00
Gm25798	17167		#N/A	1.000000E+00
Gm25799	17168		#N/A	1.000000E+00
Gm25800	17169		#N/A	1.000000E+00
Gm25801	17170		#N/A	1.000000E+00
Gm25802	17171		#N/A	1.000000E+00
Gm25803	17172		#N/A	1.000000E+00
Gm25804	17173		#N/A	1.000000E+00
Gm25805	17174		#N/A	1.000000E+00
Gm25806	17175		#N/A	1.000000E+00
Gm25807	17176	-0.038406171	3065	5.892384E-01
Gm25808	17177		#N/A	1.000000E+00
Gm25809	17178		#N/A	1.000000E+00
Gm25810	17179		#N/A	1.000000E+00
Gm25811	17180	-0.038406171	3065	5.892384E-01
Gm25812	17181		#N/A	1.000000E+00
Gm25813	17182		#N/A	1.000000E+00
Gm25814	17183		#N/A	1.000000E+00
Gm25815	17184		#N/A	1.000000E+00
Gm25816	17185		#N/A	1.000000E+00
Gm25817	17186		#N/A	1.000000E+00
Gm25818	17187		#N/A	1.000000E+00
Gm25819	17188		#N/A	1.000000E+00
Gm25820	17189		#N/A	1.000000E+00
Gm25821	17190		#N/A	1.000000E+00
Gm25822	17191	0.298324667	19897	1.781817E-05
Gm25823	17192		#N/A	1.000000E+00
Gm25824	17193		#N/A	1.000000E+00
Gm25825	17194		#N/A	1.000000E+00
Gm25826	17195		#N/A	1.000000E+00
Gm25827	17196		#N/A	1.000000E+00
Gm25828	17197		#N/A	1.000000E+00
Gm25829	17198		#N/A	1.000000E+00
Gm25830	17199		#N/A	1.000000E+00

Spearman Rank correlation analysis performed between Prdm1 and all-expressed genes within the Meredith RNA-seq dataset. Robust Prdm1–associated genes were identified using a cut-off of $p < 0.0005$.

Table S1, Related to Supplemental Figure 3C. Prdm1 associated genes

Gm25831	17200	#N/A	1.000000E+00
Gm25832	17201	#N/A	1.000000E+00
Gm25833	17202	#N/A	1.000000E+00
Gm25834	17203	#N/A	1.000000E+00
Gm25835	17204	#N/A	1.000000E+00
Gm25836	17205	#N/A	1.000000E+00
Gm25837	17206	#N/A	1.000000E+00
Gm25838	17207	#N/A	1.000000E+00
Gm25839	17208	#N/A	1.000000E+00
Gm25840	17209	#N/A	1.000000E+00
Gm25841	17210	#N/A	1.000000E+00
Gm25842	17211	#N/A	1.000000E+00
Gm25843	17212	#N/A	1.000000E+00
Gm25844	17213	#N/A	1.000000E+00
Gm25845	17214	#N/A	1.000000E+00
Gm25846	17215	#N/A	1.000000E+00
Gm25847	17216	#N/A	1.000000E+00
Gm25848	17217	#N/A	1.000000E+00
Gm25849	17218	#N/A	1.000000E+00
Gm25850	17219	#N/A	1.000000E+00
Gm25851	17220	#N/A	1.000000E+00
Gm25852	17221	#N/A	1.000000E+00
Gm25853	17222	#N/A	1.000000E+00
Gm25854	17223	#N/A	1.000000E+00
Gm25855	17224	#N/A	1.000000E+00
Gm25856	17225	#N/A	1.000000E+00
Gm25857	17226	#N/A	1.000000E+00
Gm25858	17227	#N/A	1.000000E+00
Gm25859	17228	#N/A	1.000000E+00
Gm25860	17229	#N/A	1.000000E+00
Gm25861	17230	#N/A	1.000000E+00
Gm25862	17231	#N/A	1.000000E+00
Gm25863	17232	#N/A	1.000000E+00
Gm25864	17233	#N/A	1.000000E+00
Gm25865	17234	#N/A	1.000000E+00
Gm25866	17235	#N/A	1.000000E+00
Gm25867	17236	#N/A	1.000000E+00
Gm25868	17237	#N/A	1.000000E+00
Gm25869	17238	#N/A	1.000000E+00
Gm25870	17239	#N/A	1.000000E+00
Gm25871	17240	#N/A	1.000000E+00
Gm25872	17241	#N/A	1.000000E+00
Gm25873	17242	#N/A	1.000000E+00

Spearman Rank correlation analysis performed between Prdm1 and all-expressed genes within the Meredith RNA-seq dataset. Robust Prdm1–associated genes were identified using a cut-off of $p < 0.0005$.

Table S1, Related to Supplemental Figure 3C. Prdm1 associated genes

Gm25874	17243		#N/A	1.000000E+00
Gm25875	17244		#N/A	1.000000E+00
Gm25876	17245		#N/A	1.000000E+00
Gm25877	17246		#N/A	1.000000E+00
Gm25878	17247		#N/A	1.000000E+00
Gm25879	17248		#N/A	1.000000E+00
Gm25880	17249		#N/A	1.000000E+00
Gm25881	17250		#N/A	1.000000E+00
Gm25882	17251		#N/A	1.000000E+00
Gm25883	17252		#N/A	1.000000E+00
Gm25884	17253		#N/A	1.000000E+00
Gm25885	17254		#N/A	1.000000E+00
Gm25886	17255		#N/A	1.000000E+00
Gm25887	17256		#N/A	1.000000E+00
Gm25888	17257		#N/A	1.000000E+00
Gm25889	17258		#N/A	1.000000E+00
Gm25890	17259		#N/A	1.000000E+00
Gm25891	17260		#N/A	1.000000E+00
Gm25892	17261		#N/A	1.000000E+00
Gm25893	17262		#N/A	1.000000E+00
Gm25894	17263		#N/A	1.000000E+00
Gm25895	17264		#N/A	1.000000E+00
Gm25896	17265		#N/A	1.000000E+00
Gm25897	17266		#N/A	1.000000E+00
Gm25898	17267		#N/A	1.000000E+00
Gm25899	17268		#N/A	1.000000E+00
Gm25900	17269		#N/A	1.000000E+00
Gm25901	17270		#N/A	1.000000E+00
Gm25902	17271		#N/A	1.000000E+00
Gm25903	17272		#N/A	1.000000E+00
Gm25904	17273		#N/A	1.000000E+00
Gm25905	17274		#N/A	1.000000E+00
Gm25906	17275		#N/A	1.000000E+00
Gm25907	17276		#N/A	1.000000E+00
Gm25908	17277		#N/A	1.000000E+00
Gm25909	17278		#N/A	1.000000E+00
Gm25910	17279		#N/A	1.000000E+00
Gm25911	17280	-0.038406171	3065	5.892384E-01
Gm25912	17281		#N/A	1.000000E+00
Gm25913	17282		#N/A	1.000000E+00
Gm25914	17283		#N/A	1.000000E+00
Gm25915	17284		#N/A	1.000000E+00
Gm25916	17285		#N/A	1.000000E+00

Spearman Rank correlation analysis performed between Prdm1 and all-expressed genes within the Meredith RNA-seq dataset. Robust Prdm1–associated genes were identified using a cut-off of $p < 0.0005$.

Table S1, Related to Supplemental Figure 3C. Prdm1 associated genes

Gm25917	17286	#N/A	1.000000E+00
Gm25918	17287	#N/A	1.000000E+00
Gm25919	17288	#N/A	1.000000E+00
Gm25920	17289	#N/A	1.000000E+00
Gm25921	17290	#N/A	1.000000E+00
Gm25922	17291	#N/A	1.000000E+00
Gm25923	17292	#N/A	1.000000E+00
Gm25924	17293	#N/A	1.000000E+00
Gm25925	17294	#N/A	1.000000E+00
Gm25926	17295	#N/A	1.000000E+00
Gm25927	17296	#N/A	1.000000E+00
Gm25928	17297	#N/A	1.000000E+00
Gm25929	17298	#N/A	1.000000E+00
Gm25930	17299	#N/A	1.000000E+00
Gm25931	17300	#N/A	1.000000E+00
Gm25932	17301	#N/A	1.000000E+00
Gm25933	17302	#N/A	1.000000E+00
Gm25934	17303	#N/A	1.000000E+00
Gm25935	17304	#N/A	1.000000E+00
Gm25936	17305	#N/A	1.000000E+00
Gm25937	17306	#N/A	1.000000E+00
Gm25938	17307	#N/A	1.000000E+00
Gm25939	17308	#N/A	1.000000E+00
Gm25940	17309	#N/A	1.000000E+00
Gm25941	17310	#N/A	1.000000E+00
Gm25942	17311	#N/A	1.000000E+00
Gm25943	17312	#N/A	1.000000E+00
Gm25944	17313	#N/A	1.000000E+00
Gm25945	17314	#N/A	1.000000E+00
Gm25946	17315	#N/A	1.000000E+00
Gm25947	17316	#N/A	1.000000E+00
Gm25948	17317	#N/A	1.000000E+00
Gm25949	17318	#N/A	1.000000E+00
Gm25950	17319	#N/A	1.000000E+00
Gm25951	17320	#N/A	1.000000E+00
Gm25952	17321	#N/A	1.000000E+00
Gm25953	17322	#N/A	1.000000E+00
Gm25954	17323	#N/A	1.000000E+00
Gm25955	17324	#N/A	1.000000E+00
Gm25956	17325	#N/A	1.000000E+00
Gm25957	17326	#N/A	1.000000E+00
Gm25958	17327	#N/A	1.000000E+00
Gm25959	17328	#N/A	1.000000E+00

Spearman Rank correlation analysis performed between Prdm1 and all-expressed genes within the Meredith RNA-seq dataset. Robust Prdm1–associated genes were identified using a cut-off of $p < 0.0005$.

Table S1, Related to Supplemental Figure 3C. Prdm1 associated genes

Gm25960	17329		#N/A	1.000000E+00
Gm25961	17330		#N/A	1.000000E+00
Gm25962	17331		#N/A	1.000000E+00
Gm25963	17332		#N/A	1.000000E+00
Gm25964	17333		#N/A	1.000000E+00
Gm25965	17334		#N/A	1.000000E+00
Gm25966	17335		#N/A	1.000000E+00
Gm25967	17336		#N/A	1.000000E+00
Gm25968	17337		#N/A	1.000000E+00
Gm25969	17338		#N/A	1.000000E+00
Gm25970	17339		#N/A	1.000000E+00
Gm25971	17340		#N/A	1.000000E+00
Gm25972	17341		#N/A	1.000000E+00
Gm25973	17342		#N/A	1.000000E+00
Gm25974	17343		#N/A	1.000000E+00
Gm25975	17344		#N/A	1.000000E+00
Gm25976	17345		#N/A	1.000000E+00
Gm25977	17346		#N/A	1.000000E+00
Gm25978	17347		#N/A	1.000000E+00
Gm25979	17348		#N/A	1.000000E+00
Gm25980	17349		#N/A	1.000000E+00
Gm25981	17350		#N/A	1.000000E+00
Gm25982	17351		#N/A	1.000000E+00
Gm25983	17352		#N/A	1.000000E+00
Gm25984	17353		#N/A	1.000000E+00
Gm25985	17354		#N/A	1.000000E+00
Gm25986	17355		#N/A	1.000000E+00
Gm25987	17356		#N/A	1.000000E+00
Gm25988	17357		#N/A	1.000000E+00
Gm25989	17358		#N/A	1.000000E+00
Gm25990	17359		#N/A	1.000000E+00
Gm25991	17360		#N/A	1.000000E+00
Gm25992	17361	0.042807012	7792.5	5.472647E-01
Gm25993	17362		#N/A	1.000000E+00
Gm25994	17363		#N/A	1.000000E+00
Gm25995	17364		#N/A	1.000000E+00
Gm25996	17365		#N/A	1.000000E+00
Gm25997	17366		#N/A	1.000000E+00
Gm25998	17367		#N/A	1.000000E+00
Gm25999	17368		#N/A	1.000000E+00
Gm26000	17369		#N/A	1.000000E+00
Gm26001	17370		#N/A	1.000000E+00
Gm26002	17371		#N/A	1.000000E+00

Spearman Rank correlation analysis performed between Prdm1 and all-expressed genes within the Meredith RNA-seq dataset. Robust Prdm1–associated genes were identified using a cut-off of $p < 0.0005$.

Table S1, Related to Supplemental Figure 3C. Prdm1 associated genes

Gm26003	17372		#N/A	1.000000E+00
Gm26004	17373		#N/A	1.000000E+00
Gm26005	17374		#N/A	1.000000E+00
Gm26006	17375		#N/A	1.000000E+00
Gm26007	17376		#N/A	1.000000E+00
Gm26008	17377		#N/A	1.000000E+00
Gm26009	17378		#N/A	1.000000E+00
Gm26010	17379		#N/A	1.000000E+00
Gm26011	17380		#N/A	1.000000E+00
Gm26012	17381		#N/A	1.000000E+00
Gm26013	17382		#N/A	1.000000E+00
Gm26014	17383		#N/A	1.000000E+00
Gm26015	17384		#N/A	1.000000E+00
Gm26016	17385	0.113548679	13094	1.093908E-01
Gm26017	17386		#N/A	1.000000E+00
Gm26018	17387		#N/A	1.000000E+00
Gm26019	17388		#N/A	1.000000E+00
Gm26020	17389		#N/A	1.000000E+00
Gm26021	17390		#N/A	1.000000E+00
Gm26022	17391		#N/A	1.000000E+00
Gm26023	17392	0.075715395	10317.5	2.866063E-01
Gm26024	17393		#N/A	1.000000E+00
Gm26025	17394		#N/A	1.000000E+00
Gm26026	17395		#N/A	1.000000E+00
Gm26027	17396		#N/A	1.000000E+00
Gm26028	17397		#N/A	1.000000E+00
Gm26029	17398		#N/A	1.000000E+00
Gm26030	17399		#N/A	1.000000E+00
Gm26031	17400		#N/A	1.000000E+00
Gm26032	17401		#N/A	1.000000E+00
Gm26033	17402		#N/A	1.000000E+00
Gm26034	17403		#N/A	1.000000E+00
Gm26035	17404		#N/A	1.000000E+00
Gm26036	17405		#N/A	1.000000E+00
Gm26037	17406		#N/A	1.000000E+00
Gm26038	17407		#N/A	1.000000E+00
Gm26039	17408	-0.038406171	3065	5.892384E-01
Gm26040	17409		#N/A	1.000000E+00
Gm26041	17410		#N/A	1.000000E+00
Gm26042	17411		#N/A	1.000000E+00
Gm26043	17412		#N/A	1.000000E+00
Gm26044	17413		#N/A	1.000000E+00
Gm26045	17414		#N/A	1.000000E+00

Spearman Rank correlation analysis performed between Prdm1 and all-expressed genes within the Meredith RNA-seq dataset. Robust Prdm1–associated genes were identified using a cut-off of $p < 0.0005$.

Table S1, Related to Supplemental Figure 3C. Prdm1 associated genes

Gm26046	17415		#N/A	1.000000E+00
Gm26047	17416		#N/A	1.000000E+00
Gm26048	17417		#N/A	1.000000E+00
Gm26049	17418		#N/A	1.000000E+00
Gm26050	17419		#N/A	1.000000E+00
Gm26051	17420		#N/A	1.000000E+00
Gm26052	17421		#N/A	1.000000E+00
Gm26053	17422		#N/A	1.000000E+00
Gm26054	17423		#N/A	1.000000E+00
Gm26055	17424		#N/A	1.000000E+00
Gm26056	17425		#N/A	1.000000E+00
Gm26057	17426		#N/A	1.000000E+00
Gm26058	17427		#N/A	1.000000E+00
Gm26059	17428		#N/A	1.000000E+00
Gm2606	17429		#N/A	1.000000E+00
Gm26060	17430		#N/A	1.000000E+00
Gm26061	17431		#N/A	1.000000E+00
Gm26062	17432	-0.038406171	3065	5.892384E-01
Gm26063	17433		#N/A	1.000000E+00
Gm26064	17434		#N/A	1.000000E+00
Gm26065	17435		#N/A	1.000000E+00
Gm26066	17436		#N/A	1.000000E+00
Gm26067	17437		#N/A	1.000000E+00
Gm26068	17438		#N/A	1.000000E+00
Gm26069	17439		#N/A	1.000000E+00
Gm26070	17440		#N/A	1.000000E+00
Gm26071	17441		#N/A	1.000000E+00
Gm26072	17442		#N/A	1.000000E+00
Gm26073	17443		#N/A	1.000000E+00
Gm26074	17444		#N/A	1.000000E+00
Gm26075	17445		#N/A	1.000000E+00
Gm26076	17446		#N/A	1.000000E+00
Gm26077	17447		#N/A	1.000000E+00
Gm26078	17448		#N/A	1.000000E+00
Gm26079	17449		#N/A	1.000000E+00
Gm26080	17450		#N/A	1.000000E+00
Gm26081	17451		#N/A	1.000000E+00
Gm26082	17452		#N/A	1.000000E+00
Gm26083	17453	0.149581868	15621	3.451048E-02
Gm26084	17454		#N/A	1.000000E+00
Gm26085	17455		#N/A	1.000000E+00
Gm26086	17456		#N/A	1.000000E+00
Gm26087	17457		#N/A	1.000000E+00

Spearman Rank correlation analysis performed between Prdm1 and all-expressed genes within the Meredith RNA-seq dataset. Robust Prdm1–associated genes were identified using a cut-off of $p < 0.0005$.

Table S1, Related to Supplemental Figure 3C. Prdm1 associated genes

Gm26088	17458		#N/A	1.000000E+00
Gm26089	17459		#N/A	1.000000E+00
Gm26090	17460		#N/A	1.000000E+00
Gm26091	17461	-0.054450825	1984	4.437997E-01
Gm26092	17462		#N/A	1.000000E+00
Gm26094	17463		#N/A	1.000000E+00
Gm26095	17464		#N/A	1.000000E+00
Gm26096	17465		#N/A	1.000000E+00
Gm26097	17466		#N/A	1.000000E+00
Gm26098	17467		#N/A	1.000000E+00
Gm26099	17468		#N/A	1.000000E+00
Gm26100	17469		#N/A	1.000000E+00
Gm26101	17470		#N/A	1.000000E+00
Gm26102	17471		#N/A	1.000000E+00
Gm26103	17472		#N/A	1.000000E+00
Gm26104	17473		#N/A	1.000000E+00
Gm26105	17474		#N/A	1.000000E+00
Gm26106	17475		#N/A	1.000000E+00
Gm26107	17476		#N/A	1.000000E+00
Gm26108	17477		#N/A	1.000000E+00
Gm26109	17478		#N/A	1.000000E+00
Gm26110	17479		#N/A	1.000000E+00
Gm26111	17480		#N/A	1.000000E+00
Gm26112	17481		#N/A	1.000000E+00
Gm26113	17482		#N/A	1.000000E+00
Gm26114	17483		#N/A	1.000000E+00
Gm26115	17484		#N/A	1.000000E+00
Gm26116	17485		#N/A	1.000000E+00
Gm26117	17486		#N/A	1.000000E+00
Gm26118	17487		#N/A	1.000000E+00
Gm26119	17488		#N/A	1.000000E+00
Gm26120	17489		#N/A	1.000000E+00
Gm26121	17490		#N/A	1.000000E+00
Gm26122	17491		#N/A	1.000000E+00
Gm26123	17492		#N/A	1.000000E+00
Gm26124	17493		#N/A	1.000000E+00
Gm26125	17494		#N/A	1.000000E+00
Gm26126	17495		#N/A	1.000000E+00
Gm26127	17496		#N/A	1.000000E+00
Gm26128	17497		#N/A	1.000000E+00
Gm26129	17498		#N/A	1.000000E+00
Gm26130	17499		#N/A	1.000000E+00
Gm26131	17500		#N/A	1.000000E+00

Spearman Rank correlation analysis performed between Prdm1 and all-expressed genes within the Meredith RNA-seq dataset. Robust Prdm1–associated genes were identified using a cut-off of $p < 0.0005$.

Table S1, Related to Supplemental Figure 3C. Prdm1 associated genes

Gm26132	17501		#N/A	1.000000E+00
Gm26133	17502		#N/A	1.000000E+00
Gm26134	17503		#N/A	1.000000E+00
Gm26135	17504		#N/A	1.000000E+00
Gm26136	17505		#N/A	1.000000E+00
Gm26137	17506		#N/A	1.000000E+00
Gm26138	17507		#N/A	1.000000E+00
Gm26139	17508		#N/A	1.000000E+00
Gm26140	17509		#N/A	1.000000E+00
Gm26141	17510		#N/A	1.000000E+00
Gm26142	17511		#N/A	1.000000E+00
Gm26143	17512		#N/A	1.000000E+00
Gm26144	17513		#N/A	1.000000E+00
Gm26145	17514		#N/A	1.000000E+00
Gm26146	17515		#N/A	1.000000E+00
Gm26147	17516		#N/A	1.000000E+00
Gm26148	17517		#N/A	1.000000E+00
Gm26149	17518		#N/A	1.000000E+00
Gm26150	17519		#N/A	1.000000E+00
Gm26151	17520		#N/A	1.000000E+00
Gm26152	17521		#N/A	1.000000E+00
Gm26153	17522		#N/A	1.000000E+00
Gm26154	17523		#N/A	1.000000E+00
Gm26155	17524		#N/A	1.000000E+00
Gm26156	17525		#N/A	1.000000E+00
Gm26157	17526		#N/A	1.000000E+00
Gm26158	17527		#N/A	1.000000E+00
Gm26159	17528		#N/A	1.000000E+00
Gm26160	17529		#N/A	1.000000E+00
Gm26161	17530		#N/A	1.000000E+00
Gm26162	17531		#N/A	1.000000E+00
Gm26163	17532		#N/A	1.000000E+00
Gm26164	17533		#N/A	1.000000E+00
Gm26165	17534		#N/A	1.000000E+00
Gm26166	17535	-0.038406171	3065	5.892384E-01
Gm26167	17536		#N/A	1.000000E+00
Gm26168	17537		#N/A	1.000000E+00
Gm26169	17538		#N/A	1.000000E+00
Gm2617	17539		#N/A	1.000000E+00
Gm26170	17540		#N/A	1.000000E+00
Gm26171	17541		#N/A	1.000000E+00
Gm26172	17542		#N/A	1.000000E+00
Gm26173	17543		#N/A	1.000000E+00

Spearman Rank correlation analysis performed between Prdm1 and all-expressed genes within the Meredith RNA-seq dataset. Robust Prdm1–associated genes were identified using a cut-off of $p < 0.0005$.

Table S1, Related to Supplemental Figure 3C. Prdm1 associated genes

Gm26174	17544		#N/A	1.000000E+00
Gm26175	17545		#N/A	1.000000E+00
Gm26176	17546		#N/A	1.000000E+00
Gm26177	17547		#N/A	1.000000E+00
Gm26178	17548		#N/A	1.000000E+00
Gm26179	17549		#N/A	1.000000E+00
Gm26180	17550		#N/A	1.000000E+00
Gm26181	17551		#N/A	1.000000E+00
Gm26182	17552		#N/A	1.000000E+00
Gm26183	17553		#N/A	1.000000E+00
Gm26184	17554		#N/A	1.000000E+00
Gm26185	17555		#N/A	1.000000E+00
Gm26186	17556		#N/A	1.000000E+00
Gm26187	17557		#N/A	1.000000E+00
Gm26188	17558		#N/A	1.000000E+00
Gm26189	17559		#N/A	1.000000E+00
Gm2619	17560	-0.054451513	1703	4.437940E-01
Gm26190	17561		#N/A	1.000000E+00
Gm26191	17562	-0.066856562	1139	3.468991E-01
Gm26192	17563		#N/A	1.000000E+00
Gm26193	17564		#N/A	1.000000E+00
Gm26194	17565		#N/A	1.000000E+00
Gm26195	17566		#N/A	1.000000E+00
Gm26196	17567		#N/A	1.000000E+00
Gm26197	17568		#N/A	1.000000E+00
Gm26198	17569		#N/A	1.000000E+00
Gm26199	17570		#N/A	1.000000E+00
Gm26200	17571		#N/A	1.000000E+00
Gm26201	17572		#N/A	1.000000E+00
Gm26202	17573		#N/A	1.000000E+00
Gm26203	17574		#N/A	1.000000E+00
Gm26204	17575		#N/A	1.000000E+00
Gm26205	17576	-0.038406171	3065	5.892384E-01
Gm26206	17577		#N/A	1.000000E+00
Gm26207	17578		#N/A	1.000000E+00
Gm26208	17579		#N/A	1.000000E+00
Gm26209	17580		#N/A	1.000000E+00
Gm26210	17581		#N/A	1.000000E+00
Gm26211	17582		#N/A	1.000000E+00
Gm26212	17583		#N/A	1.000000E+00
Gm26213	17584		#N/A	1.000000E+00
Gm26214	17585		#N/A	1.000000E+00
Gm26215	17586		#N/A	1.000000E+00

Spearman Rank correlation analysis performed between Prdm1 and all-expressed genes within the Meredith RNA-seq dataset. Robust Prdm1–associated genes were identified using a cut-off of $p < 0.0005$.

Gm26216	17587		#N/A	1.000000E+00
Gm26217	17588		#N/A	1.000000E+00
Gm26218	17589		#N/A	1.000000E+00
Gm26219	17590		#N/A	1.000000E+00
Gm26220	17591		#N/A	1.000000E+00
Gm26221	17592		#N/A	1.000000E+00
Gm26222	17593		#N/A	1.000000E+00
Gm26223	17594		#N/A	1.000000E+00
Gm26224	17595		#N/A	1.000000E+00
Gm26225	17596		#N/A	1.000000E+00
Gm26226	17597		#N/A	1.000000E+00
Gm26227	17598		#N/A	1.000000E+00
Gm26228	17599		#N/A	1.000000E+00
Gm26229	17600		#N/A	1.000000E+00
Gm26230	17601		#N/A	1.000000E+00
Gm26231	17602		#N/A	1.000000E+00
Gm26232	17603		#N/A	1.000000E+00
Gm26233	17604		#N/A	1.000000E+00
Gm26234	17605	-0.038406171	3065	5.892384E-01
Gm26235	17606		#N/A	1.000000E+00
Gm26236	17607		#N/A	1.000000E+00
Gm26237	17608		#N/A	1.000000E+00
Gm26238	17609		#N/A	1.000000E+00
Gm26239	17610		#N/A	1.000000E+00
Gm26240	17611		#N/A	1.000000E+00
Gm26241	17612		#N/A	1.000000E+00
Gm26242	17613		#N/A	1.000000E+00
Gm26243	17614		#N/A	1.000000E+00
Gm26244	17615		#N/A	1.000000E+00
Gm26245	17616		#N/A	1.000000E+00
Gm26246	17617		#N/A	1.000000E+00
Gm26247	17618		#N/A	1.000000E+00
Gm26248	17619		#N/A	1.000000E+00
Gm26249	17620		#N/A	1.000000E+00
Gm26250	17621		#N/A	1.000000E+00
Gm26251	17622		#N/A	1.000000E+00
Gm26252	17623		#N/A	1.000000E+00
Gm26253	17624		#N/A	1.000000E+00
Gm26254	17625		#N/A	1.000000E+00
Gm26255	17626		#N/A	1.000000E+00
Gm26256	17627		#N/A	1.000000E+00
Gm26257	17628		#N/A	1.000000E+00
Gm26258	17629		#N/A	1.000000E+00

Table S1, Related to Supplemental Figure 3C. Prdm1 associated genes

Gm26259	17630		#N/A	1.000000E+00
Gm26260	17631		#N/A	1.000000E+00
Gm26261	17632		#N/A	1.000000E+00
Gm26262	17633		#N/A	1.000000E+00
Gm26263	17634		#N/A	1.000000E+00
Gm26264	17635		#N/A	1.000000E+00
Gm26265	17636		#N/A	1.000000E+00
Gm26266	17637		#N/A	1.000000E+00
Gm26267	17638		#N/A	1.000000E+00
Gm26268	17639		#N/A	1.000000E+00
Gm26269	17640		#N/A	1.000000E+00
Gm26270	17641		#N/A	1.000000E+00
Gm26271	17642		#N/A	1.000000E+00
Gm26272	17643		#N/A	1.000000E+00
Gm26273	17644		#N/A	1.000000E+00
Gm26274	17645		#N/A	1.000000E+00
Gm26275	17646		#N/A	1.000000E+00
Gm26276	17647		#N/A	1.000000E+00
Gm26277	17648		#N/A	1.000000E+00
Gm26278	17649		#N/A	1.000000E+00
Gm26279	17650	0.1544596	15936.5	2.897553E-02
Gm26280	17651		#N/A	1.000000E+00
Gm26281	17652		#N/A	1.000000E+00
Gm26282	17653		#N/A	1.000000E+00
Gm26283	17654		#N/A	1.000000E+00
Gm26284	17655		#N/A	1.000000E+00
Gm26285	17656	0.113548679	13094	1.093908E-01
Gm26286	17657		#N/A	1.000000E+00
Gm26287	17658		#N/A	1.000000E+00
Gm26288	17659		#N/A	1.000000E+00
Gm26289	17660		#N/A	1.000000E+00
Gm26290	17661		#N/A	1.000000E+00
Gm26291	17662		#N/A	1.000000E+00
Gm26292	17663		#N/A	1.000000E+00
Gm26293	17664		#N/A	1.000000E+00
Gm26294	17665		#N/A	1.000000E+00
Gm26295	17666		#N/A	1.000000E+00
Gm26296	17667		#N/A	1.000000E+00
Gm26297	17668		#N/A	1.000000E+00
Gm26298	17669		#N/A	1.000000E+00
Gm26299	17670		#N/A	1.000000E+00
Gm26300	17671		#N/A	1.000000E+00
Gm26301	17672		#N/A	1.000000E+00

Spearman Rank correlation analysis performed between Prdm1 and all-expressed genes within the Meredith RNA-seq dataset. Robust Prdm1–associated genes were identified using a cut-off of $p < 0.0005$.

Table S1, Related to Supplemental Figure 3C. Prdm1 associated genes

Gm26302	17673		#N/A	1.000000E+00
Gm26303	17674		#N/A	1.000000E+00
Gm26304	17675		#N/A	1.000000E+00
Gm26305	17676		#N/A	1.000000E+00
Gm26306	17677		#N/A	1.000000E+00
Gm26307	17678		#N/A	1.000000E+00
Gm26308	17679		#N/A	1.000000E+00
Gm26309	17680		#N/A	1.000000E+00
Gm26310	17681		#N/A	1.000000E+00
Gm26311	17682		#N/A	1.000000E+00
Gm26312	17683		#N/A	1.000000E+00
Gm26313	17684		#N/A	1.000000E+00
Gm26314	17685		#N/A	1.000000E+00
Gm26315	17686		#N/A	1.000000E+00
Gm26316	17687		#N/A	1.000000E+00
Gm26317	17688		#N/A	1.000000E+00
Gm26318	17689		#N/A	1.000000E+00
Gm26319	17690		#N/A	1.000000E+00
Gm26320	17691		#N/A	1.000000E+00
Gm26321	17692		#N/A	1.000000E+00
Gm26322	17693		#N/A	1.000000E+00
Gm26323	17694		#N/A	1.000000E+00
Gm26324	17695		#N/A	1.000000E+00
Gm26325	17696	0.026465769	6901	7.098916E-01
Gm26326	17697		#N/A	1.000000E+00
Gm26327	17698	-0.038406171	3065	5.892384E-01
Gm26328	17699		#N/A	1.000000E+00
Gm26329	17700		#N/A	1.000000E+00
Gm26330	17701		#N/A	1.000000E+00
Gm26331	17702		#N/A	1.000000E+00
Gm26332	17703		#N/A	1.000000E+00
Gm26333	17704		#N/A	1.000000E+00
Gm26334	17705		#N/A	1.000000E+00
Gm26335	17706		#N/A	1.000000E+00
Gm26336	17707		#N/A	1.000000E+00
Gm26337	17708		#N/A	1.000000E+00
Gm26338	17709		#N/A	1.000000E+00
Gm26339	17710		#N/A	1.000000E+00
Gm26340	17711		#N/A	1.000000E+00
Gm26341	17712		#N/A	1.000000E+00
Gm26342	17713		#N/A	1.000000E+00
Gm26343	17714		#N/A	1.000000E+00
Gm26344	17715		#N/A	1.000000E+00

Spearman Rank correlation analysis performed between Prdm1 and all-expressed genes within the Meredith RNA-seq dataset. Robust Prdm1–associated genes were identified using a cut-off of $p < 0.0005$.

Table S1, Related to Supplemental Figure 3C. Prdm1 associated genes

Gm26345	17716		#N/A	1.000000E+00
Gm26346	17717		#N/A	1.000000E+00
Gm26347	17718		#N/A	1.000000E+00
Gm26348	17719		#N/A	1.000000E+00
Gm26349	17720		#N/A	1.000000E+00
Gm2635	17721		#N/A	1.000000E+00
Gm26350	17722		#N/A	1.000000E+00
Gm26351	17723		#N/A	1.000000E+00
Gm26352	17724		#N/A	1.000000E+00
Gm26353	17725		#N/A	1.000000E+00
Gm26354	17726		#N/A	1.000000E+00
Gm26355	17727		#N/A	1.000000E+00
Gm26356	17728		#N/A	1.000000E+00
Gm26357	17729		#N/A	1.000000E+00
Gm26358	17730		#N/A	1.000000E+00
Gm26359	17731		#N/A	1.000000E+00
Gm26360	17732		#N/A	1.000000E+00
Gm26361	17733		#N/A	1.000000E+00
Gm26362	17734		#N/A	1.000000E+00
Gm26363	17735		#N/A	1.000000E+00
Gm26364	17736		#N/A	1.000000E+00
Gm26365	17737		#N/A	1.000000E+00
Gm26366	17738		#N/A	1.000000E+00
Gm26367	17739		#N/A	1.000000E+00
Gm26368	17740		#N/A	1.000000E+00
Gm26369	17741		#N/A	1.000000E+00
Gm26370	17742		#N/A	1.000000E+00
Gm26371	17743		#N/A	1.000000E+00
Gm26372	17744		#N/A	1.000000E+00
Gm26373	17745		#N/A	1.000000E+00
Gm26374	17746		#N/A	1.000000E+00
Gm26375	17747		#N/A	1.000000E+00
Gm26376	17748		#N/A	1.000000E+00
Gm26377	17749	0.184970571	17634	8.737733E-03
Gm26378	17750		#N/A	1.000000E+00
Gm26379	17751		#N/A	1.000000E+00
Gm26380	17752		#N/A	1.000000E+00
Gm26381	17753		#N/A	1.000000E+00
Gm26382	17754		#N/A	1.000000E+00
Gm26383	17755		#N/A	1.000000E+00
Gm26384	17756	-0.018105422	4347	7.991361E-01
Gm26385	17757		#N/A	1.000000E+00
Gm26386	17758		#N/A	1.000000E+00

Spearman Rank correlation analysis performed between Prdm1 and all-expressed genes within the Meredith RNA-seq dataset. Robust Prdm1–associated genes were identified using a cut-off of $p < 0.0005$.

Table S1, Related to Supplemental Figure 3C. Prdm1 associated genes

Gm26387	17759		#N/A	1.000000E+00
Gm26388	17760	-0.066856562	1139	3.468991E-01
Gm26389	17761		#N/A	1.000000E+00
Gm2639	17762		#N/A	1.000000E+00
Gm26390	17763		#N/A	1.000000E+00
Gm26391	17764		#N/A	1.000000E+00
Gm26392	17765		#N/A	1.000000E+00
Gm26393	17766		#N/A	1.000000E+00
Gm26394	17767		#N/A	1.000000E+00
Gm26395	17768		#N/A	1.000000E+00
Gm26396	17769		#N/A	1.000000E+00
Gm26397	17770		#N/A	1.000000E+00
Gm26398	17771		#N/A	1.000000E+00
Gm26399	17772		#N/A	1.000000E+00
Gm26400	17773		#N/A	1.000000E+00
Gm26401	17774		#N/A	1.000000E+00
Gm26402	17775		#N/A	1.000000E+00
Gm26403	17776		#N/A	1.000000E+00
Gm26404	17777		#N/A	1.000000E+00
Gm26405	17778		#N/A	1.000000E+00
Gm26406	17779		#N/A	1.000000E+00
Gm26407	17780	-0.038406171	3065	5.892384E-01
Gm26408	17781		#N/A	1.000000E+00
Gm26409	17782		#N/A	1.000000E+00
Gm26410	17783		#N/A	1.000000E+00
Gm26411	17784		#N/A	1.000000E+00
Gm26412	17785		#N/A	1.000000E+00
Gm26413	17786		#N/A	1.000000E+00
Gm26414	17787		#N/A	1.000000E+00
Gm26415	17788		#N/A	1.000000E+00
Gm26416	17789		#N/A	1.000000E+00
Gm26417	17790		#N/A	1.000000E+00
Gm26418	17791		#N/A	1.000000E+00
Gm26419	17792		#N/A	1.000000E+00
Gm26420	17793		#N/A	1.000000E+00
Gm26421	17794		#N/A	1.000000E+00
Gm26422	17795		#N/A	1.000000E+00
Gm26423	17796		#N/A	1.000000E+00
Gm26424	17797		#N/A	1.000000E+00
Gm26425	17798		#N/A	1.000000E+00
Gm26426	17799		#N/A	1.000000E+00
Gm26427	17800		#N/A	1.000000E+00
Gm26428	17801		#N/A	1.000000E+00

Spearman Rank correlation analysis performed between Prdm1 and all-expressed genes within the Meredith RNA-seq dataset. Robust Prdm1–associated genes were identified using a cut-off of $p < 0.0005$.

Table S1, Related to Supplemental Figure 3C. Prdm1 associated genes

Gm26429	17802		#N/A	1.000000E+00
Gm26430	17803		#N/A	1.000000E+00
Gm26431	17804		#N/A	1.000000E+00
Gm26432	17805		#N/A	1.000000E+00
Gm26433	17806		#N/A	1.000000E+00
Gm26434	17807		#N/A	1.000000E+00
Gm26435	17808		#N/A	1.000000E+00
Gm26436	17809		#N/A	1.000000E+00
Gm26437	17810		#N/A	1.000000E+00
Gm26438	17811		#N/A	1.000000E+00
Gm26439	17812		#N/A	1.000000E+00
Gm26440	17813		#N/A	1.000000E+00
Gm26441	17814		#N/A	1.000000E+00
Gm26442	17815		#N/A	1.000000E+00
Gm26443	17816		#N/A	1.000000E+00
Gm26444	17817		#N/A	1.000000E+00
Gm26445	17818	-0.038406171	3065	5.892384E-01
Gm26446	17819		#N/A	1.000000E+00
Gm26447	17820		#N/A	1.000000E+00
Gm26448	17821		#N/A	1.000000E+00
Gm26449	17822		#N/A	1.000000E+00
Gm26450	17823		#N/A	1.000000E+00
Gm26451	17824		#N/A	1.000000E+00
Gm26452	17825		#N/A	1.000000E+00
Gm26453	17826		#N/A	1.000000E+00
Gm26454	17827		#N/A	1.000000E+00
Gm26455	17828		#N/A	1.000000E+00
Gm26456	17829		#N/A	1.000000E+00
Gm26457	17830		#N/A	1.000000E+00
Gm26458	17831		#N/A	1.000000E+00
Gm26459	17832		#N/A	1.000000E+00
Gm26460	17833		#N/A	1.000000E+00
Gm26461	17834		#N/A	1.000000E+00
Gm26462	17835		#N/A	1.000000E+00
Gm26463	17836		#N/A	1.000000E+00
Gm26464	17837		#N/A	1.000000E+00
Gm26465	17838		#N/A	1.000000E+00
Gm26466	17839		#N/A	1.000000E+00
Gm26467	17840		#N/A	1.000000E+00
Gm26468	17841		#N/A	1.000000E+00
Gm26469	17842		#N/A	1.000000E+00
Gm26470	17843		#N/A	1.000000E+00
Gm26471	17844		#N/A	1.000000E+00

Spearman Rank correlation analysis performed between Prdm1 and all-expressed genes within the Meredith RNA-seq dataset. Robust Prdm1–associated genes were identified using a cut-off of $p < 0.0005$.

Table S1, Related to Supplemental Figure 3C. Prdm1 associated genes

Gm26472	17845		#N/A	1.000000E+00
Gm26473	17846		#N/A	1.000000E+00
Gm26474	17847		#N/A	1.000000E+00
Gm26475	17848		#N/A	1.000000E+00
Gm26476	17849		#N/A	1.000000E+00
Gm26477	17850		#N/A	1.000000E+00
Gm26478	17851		#N/A	1.000000E+00
Gm26479	17852		#N/A	1.000000E+00
Gm26480	17853		#N/A	1.000000E+00
Gm26481	17854		#N/A	1.000000E+00
Gm26482	17855		#N/A	1.000000E+00
Gm26483	17856		#N/A	1.000000E+00
Gm26484	17857		#N/A	1.000000E+00
Gm26485	17858		#N/A	1.000000E+00
Gm26486	17859		#N/A	1.000000E+00
Gm26487	17860		#N/A	1.000000E+00
Gm26488	17861		#N/A	1.000000E+00
Gm26489	17862		#N/A	1.000000E+00
Gm26490	17863		#N/A	1.000000E+00
Gm26491	17864		#N/A	1.000000E+00
Gm26492	17865		#N/A	1.000000E+00
Gm26493	17866		#N/A	1.000000E+00
Gm26494	17867		#N/A	1.000000E+00
Gm26495	17868		#N/A	1.000000E+00
Gm26496	17869		#N/A	1.000000E+00
Gm26497	17870		#N/A	1.000000E+00
Gm26498	17871		#N/A	1.000000E+00
Gm26499	17872		#N/A	1.000000E+00
Gm26500	17873		#N/A	1.000000E+00
Gm26501	17874		#N/A	1.000000E+00
Gm26502	17875		#N/A	1.000000E+00
Gm26503	17876		#N/A	1.000000E+00
Gm26504	17877		#N/A	1.000000E+00
Gm26505	17878	-0.054450825	1984	4.437997E-01
Gm26506	17879		#N/A	1.000000E+00
Gm266	17880	0.02819721	6980	6.918482E-01
Gm2663	17881		#N/A	1.000000E+00
Gm2666	17882		#N/A	1.000000E+00
Gm2673	17883		#N/A	1.000000E+00
Gm2709	17884		#N/A	1.000000E+00
Gm2735	17885	0.040176447	7645	5.721775E-01
Gm2759	17886		#N/A	1.000000E+00
Gm2768	17887		#N/A	1.000000E+00

Spearman Rank correlation analysis performed between Prdm1 and all-expressed genes within the Meredith RNA-seq dataset. Robust Prdm1–associated genes were identified using a cut-off of $p < 0.0005$.

Table S1, Related to Supplemental Figure 3C. Prdm1 associated genes

Gm2777	17888		#N/A	1.000000E+00
Gm2784	17889		#N/A	1.000000E+00
Gm2788	17890	0.220660567	18924	1.690368E-03
Gm2790	17891		#N/A	1.000000E+00
Gm2792	17892	0.185404186	17655	8.578651E-03
Gm2799	17893		#N/A	1.000000E+00
Gm2800	17894		#N/A	1.000000E+00
Gm281	17895	-0.066856562	1139	3.468991E-01
Gm2810	17896		#N/A	1.000000E+00
Gm2822	17897		#N/A	1.000000E+00
Gm2825	17898		#N/A	1.000000E+00
Gm2830	17899	0.033643073	7272	6.362584E-01
Gm2832	17900	0.052728522	8550.5	4.583686E-01
Gm2840	17901		#N/A	1.000000E+00
Gm2854	17902		#N/A	1.000000E+00
Gm2855	17903		#N/A	1.000000E+00
Gm2862	17904		#N/A	1.000000E+00
Gm2863	17905		#N/A	1.000000E+00
Gm2864	17906		#N/A	1.000000E+00
Gm2866	17907		#N/A	1.000000E+00
Gm2869	17908		#N/A	1.000000E+00
Gm2871	17909		#N/A	1.000000E+00
Gm2877	17910		#N/A	1.000000E+00
Gm2878	17911		#N/A	1.000000E+00
Gm2888	17912		#N/A	1.000000E+00
Gm2892	17913		#N/A	1.000000E+00
Gm2895	17914	0.104782751	12343	1.397745E-01
Gm2897	17915		#N/A	1.000000E+00
Gm2904	17916		#N/A	1.000000E+00
Gm2913	17917		#N/A	1.000000E+00
Gm2916	17918		#N/A	1.000000E+00
Gm2927	17919		#N/A	1.000000E+00
Gm2930	17920		#N/A	1.000000E+00
Gm2933	17921		#N/A	1.000000E+00
Gm2951	17922		#N/A	1.000000E+00
Gm2956	17923		#N/A	1.000000E+00
Gm2959	17924		#N/A	1.000000E+00
Gm2962	17925		#N/A	1.000000E+00
Gm2964	17926		#N/A	1.000000E+00
Gm2966	17927		#N/A	1.000000E+00
Gm2974	17928		#N/A	1.000000E+00
Gm2976	17929		#N/A	1.000000E+00
Gm2986	17930	-0.038406171	3065	5.892384E-01

Spearman Rank correlation analysis performed between Prdm1 and all-expressed genes within the Meredith RNA-seq dataset. Robust Prdm1–associated genes were identified using a cut-off of $p < 0.0005$.

Table S1, Related to Supplemental Figure 3C. Prdm1 associated genes

Gm2991	17931		#N/A	1.000000E+00
Gm2a	17932	0.246321121	19458	4.380959E-04
Gm3002	17933	0.058867472	9149	4.076638E-01
Gm3005	17934		#N/A	1.000000E+00
Gm3008	17935		#N/A	1.000000E+00
Gm3012	17936		#N/A	1.000000E+00
Gm3015	17937		#N/A	1.000000E+00
Gm3020	17938		#N/A	1.000000E+00
Gm3025	17939		#N/A	1.000000E+00
Gm3027	17940		#N/A	1.000000E+00
Gm3029	17941		#N/A	1.000000E+00
Gm3030	17942		#N/A	1.000000E+00
Gm3033	17943		#N/A	1.000000E+00
Gm3043	17944		#N/A	1.000000E+00
Gm3047	17945		#N/A	1.000000E+00
Gm3053	17946		#N/A	1.000000E+00
Gm3054	17947		#N/A	1.000000E+00
Gm3055	17948		#N/A	1.000000E+00
Gm3072	17949	-0.038406171	3065	5.892384E-01
Gm3076	17950		#N/A	1.000000E+00
Gm3077	17951		#N/A	1.000000E+00
Gm3084	17952		#N/A	1.000000E+00
Gm3086	17953	0.145351509	15374	4.001295E-02
Gm3091	17954		#N/A	1.000000E+00
Gm3095	17955		#N/A	1.000000E+00
Gm3099	17956		#N/A	1.000000E+00
Gm3102	17957		#N/A	1.000000E+00
Gm3104	17958		#N/A	1.000000E+00
Gm3106	17959		#N/A	1.000000E+00
Gm3111	17960		#N/A	1.000000E+00
Gm3115	17961		#N/A	1.000000E+00
Gm3123	17962		#N/A	1.000000E+00
Gm3127	17963	0.038758411	7558.5	5.858252E-01
Gm3138	17964		#N/A	1.000000E+00
GM3139	17965		#N/A	1.000000E+00
Gm3139	17966		#N/A	1.000000E+00
Gm3140	17967		#N/A	1.000000E+00
Gm3141	17968		#N/A	1.000000E+00
GM3147	17969		#N/A	1.000000E+00
Gm3147	17970		#N/A	1.000000E+00
Gm3149	17971		#N/A	1.000000E+00
Gm3150	17972	-0.038406171	3065	5.892384E-01
Gm3159	17973		#N/A	1.000000E+00

Spearman Rank correlation analysis performed between Prdm1 and all-expressed genes within the Meredith RNA-seq dataset. Robust Prdm1–associated genes were identified using a cut-off of $p < 0.0005$.

Table S1, Related to Supplemental Figure 3C. Prdm1 associated genes

Gm3160	17974		#N/A	1.000000E+00
Gm3164	17975		#N/A	1.000000E+00
Gm3170	17976		#N/A	1.000000E+00
Gm3173	17977	-0.054451513	1703	4.437940E-01
Gm3176	17978		#N/A	1.000000E+00
Gm318	17979		#N/A	1.000000E+00
Gm3182	17980		#N/A	1.000000E+00
Gm3183	17981		#N/A	1.000000E+00
Gm3187	17982	-0.038406171	3065	5.892384E-01
Gm3191	17983		#N/A	1.000000E+00
Gm3194	17984		#N/A	1.000000E+00
Gm3208	17985		#N/A	1.000000E+00
Gm3211	17986		#N/A	1.000000E+00
Gm3216	17987		#N/A	1.000000E+00
Gm3221	17988		#N/A	1.000000E+00
Gm3222	17989		#N/A	1.000000E+00
Gm3233	17990		#N/A	1.000000E+00
Gm3235	17991		#N/A	1.000000E+00
Gm3238	17992		#N/A	1.000000E+00
Gm3239	17993		#N/A	1.000000E+00
Gm3242	17994		#N/A	1.000000E+00
Gm3244	17995		#N/A	1.000000E+00
Gm3248	17996	-0.038406171	3065	5.892384E-01
Gm3250	17997		#N/A	1.000000E+00
Gm3252	17998	0.143605682	15224	4.249002E-02
Gm3259	17999		#N/A	1.000000E+00
Gm3261	18000		#N/A	1.000000E+00
Gm3264	18001	-0.038406171	3065	5.892384E-01
Gm3267	18002		#N/A	1.000000E+00
Gm3269	18003		#N/A	1.000000E+00
Gm3272	18004		#N/A	1.000000E+00
Gm3275	18005		#N/A	1.000000E+00
Gm3278	18006		#N/A	1.000000E+00
GM3286	18007		#N/A	1.000000E+00
Gm3286	18008	-0.038406171	3065	5.892384E-01
Gm3287	18009		#N/A	1.000000E+00
Gm3294	18010	0.061664819	9312	3.857054E-01
Gm3298	18011		#N/A	1.000000E+00
Gm3307	18012	0.143605682	15224	4.249002E-02
Gm3317	18013		#N/A	1.000000E+00
Gm3326	18014		#N/A	1.000000E+00
Gm3327	18015		#N/A	1.000000E+00
Gm3329	18016		#N/A	1.000000E+00

Spearman Rank correlation analysis performed between Prdm1 and all-expressed genes within the Meredith RNA-seq dataset. Robust Prdm1–associated genes were identified using a cut-off of $p < 0.0005$.

Table S1, Related to Supplemental Figure 3C. Prdm1 associated genes

Gm3336	18017	-0.134267676	52	5.802332E-02
Gm3339	18018		#N/A	1.000000E+00
Gm3344	18019		#N/A	1.000000E+00
Gm3345	18020		#N/A	1.000000E+00
Gm3348	18021		#N/A	1.000000E+00
Gm3355	18022		#N/A	1.000000E+00
Gm3362	18023		#N/A	1.000000E+00
Gm3370	18024		#N/A	1.000000E+00
Gm3371	18025	-0.038406171	3065	5.892384E-01
Gm3373	18026		#N/A	1.000000E+00
Gm3376	18027		#N/A	1.000000E+00
Gm3383	18028	-0.038406171	3065	5.892384E-01
Gm340	18029		#N/A	1.000000E+00
Gm3402	18030		#N/A	1.000000E+00
Gm3404	18031		#N/A	1.000000E+00
Gm3409	18032		#N/A	1.000000E+00
Gm3411	18033	-0.038406171	3065	5.892384E-01
Gm3415	18034		#N/A	1.000000E+00
Gm3417	18035		#N/A	1.000000E+00
Gm3424	18036		#N/A	1.000000E+00
Gm3435	18037	0.054850556	8911	4.404564E-01
Gm3436	18038		#N/A	1.000000E+00
Gm3443	18039	-0.054450825	1984	4.437997E-01
Gm3448	18040		#N/A	1.000000E+00
Gm3453	18041	0.143605682	15224	4.249002E-02
Gm3460	18042		#N/A	1.000000E+00
Gm3468	18043	0.052728522	8550.5	4.583686E-01
Gm3470	18044		#N/A	1.000000E+00
Gm3476	18045		#N/A	1.000000E+00
Gm3486	18046		#N/A	1.000000E+00
Gm3488	18047		#N/A	1.000000E+00
Gm3495	18048		#N/A	1.000000E+00
Gm3500	18049	-0.066858259	1029.5	3.468868E-01
Gm3512	18050		#N/A	1.000000E+00
Gm3513	18051		#N/A	1.000000E+00
Gm3526	18052		#N/A	1.000000E+00
Gm3531	18053		#N/A	1.000000E+00
Gm3542	18054		#N/A	1.000000E+00
Gm3543	18055		#N/A	1.000000E+00
Gm355	18056		#N/A	1.000000E+00
Gm3550	18057	0.053805701	8820	4.492258E-01
Gm3555	18058		#N/A	1.000000E+00
Gm3558	18059		#N/A	1.000000E+00

Spearman Rank correlation analysis performed between Prdm1 and all-expressed genes within the Meredith RNA-seq dataset. Robust Prdm1–associated genes were identified using a cut-off of $p < 0.0005$.

Table S1, Related to Supplemental Figure 3C. Prdm1 associated genes

Gm3563	18060	-0.038406171	3065	5.892384E-01
Gm3571	18061	0.110550608	12763	1.191378E-01
Gm3573	18062		#N/A	1.000000E+00
Gm3584	18063		#N/A	1.000000E+00
Gm3585	18064		#N/A	1.000000E+00
Gm3591	18065		#N/A	1.000000E+00
Gm3594	18066		#N/A	1.000000E+00
Gm3604	18067	-0.054450825	1984	4.437997E-01
Gm3608	18068		#N/A	1.000000E+00
Gm3611	18069		#N/A	1.000000E+00
Gm3616	18070		#N/A	1.000000E+00
Gm3617	18071		#N/A	1.000000E+00
Gm3618	18072		#N/A	1.000000E+00
Gm362	18073		#N/A	1.000000E+00
Gm3623	18074	-0.066858259	1029.5	3.468868E-01
Gm3625	18075		#N/A	1.000000E+00
Gm3629	18076	0.042546099	7777	5.497117E-01
Gm3633	18077		#N/A	1.000000E+00
Gm3636	18078		#N/A	1.000000E+00
Gm364	18079	0.18218536	17494	9.823364E-03
Gm3642	18080		#N/A	1.000000E+00
Gm3644	18081		#N/A	1.000000E+00
Gm3646	18082		#N/A	1.000000E+00
Gm3654	18083		#N/A	1.000000E+00
Gm3657	18084		#N/A	1.000000E+00
Gm3665	18085		#N/A	1.000000E+00
Gm3667	18086		#N/A	1.000000E+00
Gm3669	18087		#N/A	1.000000E+00
Gm3675	18088		#N/A	1.000000E+00
Gm3676	18089		#N/A	1.000000E+00
Gm3677	18090		#N/A	1.000000E+00
Gm3681	18091		#N/A	1.000000E+00
Gm3683	18092		#N/A	1.000000E+00
Gm3685	18093	-0.038406171	3065	5.892384E-01
Gm3687	18094	0.160304018	16323	2.335876E-02
Gm3695	18095		#N/A	1.000000E+00
Gm3696	18096	-0.038406171	3065	5.892384E-01
Gm3699	18097		#N/A	1.000000E+00
Gm3701	18098		#N/A	1.000000E+00
Gm3706	18099		#N/A	1.000000E+00
Gm371	18100		#N/A	1.000000E+00
Gm3712	18101		#N/A	1.000000E+00
Gm3715	18102		#N/A	1.000000E+00

Spearman Rank correlation analysis performed between Prdm1 and all-expressed genes within the Meredith RNA-seq dataset. Robust Prdm1–associated genes were identified using a cut-off of $p < 0.0005$.

Table S1, Related to Supplemental Figure 3C. Prdm1 associated genes

Gm3719	18103	0.143605682	15224	4.249002E-02
Gm3727	18104		#N/A	1.000000E+00
Gm3739	18105	0.182294195	17509.5	9.778799E-03
Gm3750	18106		#N/A	1.000000E+00
Gm3752	18107	-0.038406171	3065	5.892384E-01
Gm3755	18108		#N/A	1.000000E+00
Gm3756	18109		#N/A	1.000000E+00
Gm3757	18110		#N/A	1.000000E+00
Gm3760	18111		#N/A	1.000000E+00
Gm3763	18112		#N/A	1.000000E+00
Gm3765	18113		#N/A	1.000000E+00
Gm378	18114		#N/A	1.000000E+00
Gm3788	18115	-0.038406171	3065	5.892384E-01
Gm379	18116		#N/A	1.000000E+00
Gm381	18117		#N/A	1.000000E+00
Gm3810	18118		#N/A	1.000000E+00
Gm382	18119	0.053267784	8685	4.537786E-01
Gm3828	18120		#N/A	1.000000E+00
Gm3833	18121	-0.038406171	3065	5.892384E-01
Gm3835	18122		#N/A	1.000000E+00
Gm3839	18123		#N/A	1.000000E+00
Gm3848	18124		#N/A	1.000000E+00
Gm3851	18125		#N/A	1.000000E+00
Gm3852	18126		#N/A	1.000000E+00
Gm3867	18127	-0.038406171	3065	5.892384E-01
Gm3880	18128	0.021317126	6513.5	7.644715E-01
Gm3883	18129		#N/A	1.000000E+00
Gm3892	18130		#N/A	1.000000E+00
GM3893	18131		#N/A	1.000000E+00
Gm3893	18132		#N/A	1.000000E+00
Gm3896	18133		#N/A	1.000000E+00
Gm3898	18134		#N/A	1.000000E+00
Gm3912	18135		#N/A	1.000000E+00
Gm3928	18136		#N/A	1.000000E+00
Gm3931	18137		#N/A	1.000000E+00
Gm3940	18138		#N/A	1.000000E+00
Gm3943	18139		#N/A	1.000000E+00
Gm3944	18140		#N/A	1.000000E+00
Gm3956	18141		#N/A	1.000000E+00
Gm3959	18142		#N/A	1.000000E+00
Gm3962	18143		#N/A	1.000000E+00
Gm3965	18144		#N/A	1.000000E+00
Gm3968	18145		#N/A	1.000000E+00

Spearman Rank correlation analysis performed between Prdm1 and all-expressed genes within the Meredith RNA-seq dataset. Robust Prdm1–associated genes were identified using a cut-off of $p < 0.0005$.

Table S1, Related to Supplemental Figure 3C. Prdm1 associated genes

Gm3972	18146		#N/A	1.000000E+00
Gm3977	18147		#N/A	1.000000E+00
Gm3978	18148		#N/A	1.000000E+00
Gm3980	18149		#N/A	1.000000E+00
Gm3981	18150		#N/A	1.000000E+00
Gm3984	18151		#N/A	1.000000E+00
Gm3985	18152	-0.040669096	2458	5.674714E-01
Gm3987	18153		#N/A	1.000000E+00
Gm3989	18154		#N/A	1.000000E+00
Gm3993	18155		#N/A	1.000000E+00
Gm3994	18156		#N/A	1.000000E+00
Gm3996	18157		#N/A	1.000000E+00
Gm3998	18158		#N/A	1.000000E+00
Gm40	18159	0.021316405	6469	7.644793E-01
Gm4001	18160		#N/A	1.000000E+00
Gm4003	18161		#N/A	1.000000E+00
Gm4011	18162		#N/A	1.000000E+00
Gm4014	18163		#N/A	1.000000E+00
Gm4016	18164		#N/A	1.000000E+00
Gm4018	18165		#N/A	1.000000E+00
Gm4023	18166		#N/A	1.000000E+00
Gm4027	18167		#N/A	1.000000E+00
Gm4070	18168	0.152789767	15822	3.077826E-02
Gm4073	18169	-0.038406171	3065	5.892384E-01
Gm4076	18170	0.000307056	5327	9.965570E-01
Gm41	18171		#N/A	1.000000E+00
Gm410	18172	0.011525191	5854	8.713259E-01
Gm4117	18173	0.307760528	19933	9.285051E-06
Gm4128	18174	0.113548679	13094	1.093908E-01
Gm4133	18175		#N/A	1.000000E+00
Gm4134	18176		#N/A	1.000000E+00
Gm4141	18177		#N/A	1.000000E+00
Gm4149	18178	0.110062269	12719	1.207876E-01
Gm4152	18179		#N/A	1.000000E+00
Gm4172	18180		#N/A	1.000000E+00
Gm4175	18181		#N/A	1.000000E+00
Gm4177	18182		#N/A	1.000000E+00
Gm4179	18183		#N/A	1.000000E+00
Gm4181	18184		#N/A	1.000000E+00
Gm4187	18185		#N/A	1.000000E+00
Gm4189	18186	-0.000458472	5251.5	9.948592E-01
Gm4201	18187		#N/A	1.000000E+00
Gm4202	18188		#N/A	1.000000E+00

Spearman Rank correlation analysis performed between Prdm1 and all-expressed genes within the Meredith RNA-seq dataset. Robust Prdm1–associated genes were identified using a cut-off of $p < 0.0005$.

Table S1, Related to Supplemental Figure 3C. Prdm1 associated genes

Gm4204	18189	0.179439217	17378	1.100886E-02
Gm4207	18190		#N/A	1.000000E+00
Gm4214	18191		#N/A	1.000000E+00
Gm4216	18192		#N/A	1.000000E+00
Gm4219	18193	-0.038406171	3065	5.892384E-01
Gm4220	18194		#N/A	1.000000E+00
Gm4222	18195		#N/A	1.000000E+00
Gm4234	18196		#N/A	1.000000E+00
Gm4245	18197		#N/A	1.000000E+00
Gm4246	18198		#N/A	1.000000E+00
Gm4252	18199		#N/A	1.000000E+00
Gm4256	18200		#N/A	1.000000E+00
Gm4271	18201		#N/A	1.000000E+00
Gm4279	18202		#N/A	1.000000E+00
Gm428	18203		#N/A	1.000000E+00
Gm4285	18204	0.212616774	18681	2.505763E-03
Gm4287	18205		#N/A	1.000000E+00
Gm4294	18206		#N/A	1.000000E+00
Gm4297	18207	0.098871246	11986	1.636496E-01
Gm4301	18208		#N/A	1.000000E+00
Gm4302	18209		#N/A	1.000000E+00
Gm4303	18210		#N/A	1.000000E+00
Gm4305	18211		#N/A	1.000000E+00
Gm4307	18212		#N/A	1.000000E+00
Gm4308	18213		#N/A	1.000000E+00
Gm4312	18214		#N/A	1.000000E+00
Gm4340	18215		#N/A	1.000000E+00
Gm4342	18216		#N/A	1.000000E+00
Gm4345	18217		#N/A	1.000000E+00
Gm4353	18218		#N/A	1.000000E+00
Gm436	18219		#N/A	1.000000E+00
Gm438	18220	-0.095267438	357.5	1.796303E-01
Gm44	18221		#N/A	1.000000E+00
Gm4409	18222	0.045892028	7996	5.187436E-01
Gm4425	18223	0.113548679	13094	1.093908E-01
Gm4450	18224		#N/A	1.000000E+00
Gm4459	18225		#N/A	1.000000E+00
Gm4461	18226	-0.038406171	3065	5.892384E-01
Gm4462	18227		#N/A	1.000000E+00
Gm4468	18228	0.254164912	19553	2.815309E-04
Gm4473	18229	0.021317126	6513.5	7.644715E-01
Gm4477	18230		#N/A	1.000000E+00
Gm4479	18231	-0.066858259	1029.5	3.468868E-01

Spearman Rank correlation analysis performed between Prdm1 and all-expressed genes within the Meredith RNA-seq dataset. Robust Prdm1–associated genes were identified using a cut-off of $p < 0.0005$.

Table S1, Related to Supplemental Figure 3C. Prdm1 associated genes

Gm4482	18232		#N/A	1.000000E+00
Gm4487	18233		#N/A	1.000000E+00
Gm4491	18234		#N/A	1.000000E+00
Gm4498	18235		#N/A	1.000000E+00
Gm4524	18236		#N/A	1.000000E+00
Gm4535	18237		#N/A	1.000000E+00
Gm454	18238		#N/A	1.000000E+00
Gm4540	18239	0.115147259	13334	1.044556E-01
Gm4553	18240		#N/A	1.000000E+00
Gm4558	18241		#N/A	1.000000E+00
Gm4559	18242		#N/A	1.000000E+00
Gm4567	18243		#N/A	1.000000E+00
Gm4577	18244		#N/A	1.000000E+00
Gm4596	18245		#N/A	1.000000E+00
Gm46	18246		#N/A	1.000000E+00
Gm4604	18247		#N/A	1.000000E+00
Gm4609	18248		#N/A	1.000000E+00
Gm4613	18249	-0.038406171	3065	5.892384E-01
Gm4617	18250		#N/A	1.000000E+00
Gm4631	18251		#N/A	1.000000E+00
Gm4636	18252	-0.054451513	1703	4.437940E-01
Gm4660	18253		#N/A	1.000000E+00
Gm4675	18254	0.009995747	5770	8.882818E-01
Gm4705	18255		#N/A	1.000000E+00
Gm4707	18256		#N/A	1.000000E+00
Gm4723	18257		#N/A	1.000000E+00
Gm4724	18258		#N/A	1.000000E+00
Gm4725	18259		#N/A	1.000000E+00
Gm4726	18260		#N/A	1.000000E+00
Gm4732	18261		#N/A	1.000000E+00
Gm4735	18262		#N/A	1.000000E+00
Gm4736	18263		#N/A	1.000000E+00
Gm4737	18264	0.102539695	12212	1.484995E-01
Gm4744	18265		#N/A	1.000000E+00
Gm4745	18266		#N/A	1.000000E+00
Gm4746	18267		#N/A	1.000000E+00
Gm4750	18268		#N/A	1.000000E+00
Gm4758	18269		#N/A	1.000000E+00
Gm4759	18270	-0.038406171	3065	5.892384E-01
Gm4760	18271		#N/A	1.000000E+00
Gm4761	18272	0.145821843	15402	3.936680E-02
Gm4763	18273		#N/A	1.000000E+00
Gm4764	18274	-0.038406171	3065	5.892384E-01

Spearman Rank correlation analysis performed between Prdm1 and all-expressed genes within the Meredith RNA-seq dataset. Robust Prdm1–associated genes were identified using a cut-off of $p < 0.0005$.

Table S1, Related to Supplemental Figure 3C. Prdm1 associated genes

Gm4767	18275		#N/A	1.000000E+00
Gm4772	18276		#N/A	1.000000E+00
Gm4773	18277		#N/A	1.000000E+00
Gm4775	18278	0.089371505	11299	2.082104E-01
Gm4776	18279	-0.031503837	3862	6.578750E-01
Gm4778	18280		#N/A	1.000000E+00
Gm4779	18281		#N/A	1.000000E+00
Gm4781	18282		#N/A	1.000000E+00
Gm4782	18283		#N/A	1.000000E+00
Gm4784	18284	0.081085405	10671.5	2.536978E-01
Gm4787	18285		#N/A	1.000000E+00
Gm4788	18286	0.004149159	5524.5	9.535014E-01
Gm4789	18287		#N/A	1.000000E+00
Gm4791	18288	0.066135487	9601	3.521362E-01
Gm4792	18289		#N/A	1.000000E+00
Gm4793	18290		#N/A	1.000000E+00
Gm4794	18291	0.053267784	8685	4.537786E-01
Gm4795	18292		#N/A	1.000000E+00
Gm4799	18293		#N/A	1.000000E+00
Gm4810	18294		#N/A	1.000000E+00
Gm4822	18295		#N/A	1.000000E+00
Gm4825	18296	0.014301797	6015.5	8.406982E-01
Gm4830	18297		#N/A	1.000000E+00
Gm4831	18298		#N/A	1.000000E+00
Gm4832	18299	0.155036818	15983	2.837371E-02
Gm4835	18300		#N/A	1.000000E+00
Gm4836	18301		#N/A	1.000000E+00
Gm4841	18302	-0.080575548	635	2.567052E-01
Gm4845	18303		#N/A	1.000000E+00
Gm4846	18304	0.212479273	18674.5	2.522377E-03
Gm4847	18305	-0.054451513	1703	4.437940E-01
Gm4852	18306		#N/A	1.000000E+00
Gm4853	18307	0.113548679	13094	1.093908E-01
Gm4858	18308		#N/A	1.000000E+00
Gm4861	18309		#N/A	1.000000E+00
Gm4862	18310		#N/A	1.000000E+00
Gm4868	18311		#N/A	1.000000E+00
Gm4871	18312		#N/A	1.000000E+00
Gm4875	18313		#N/A	1.000000E+00
Gm4876	18314	0.056955735	9035	4.230868E-01
Gm4877	18315	0.113548679	13094	1.093908E-01
Gm4879	18316	0.113548679	13094	1.093908E-01
Gm4883	18317	0.146695052	15461	3.819046E-02

Spearman Rank correlation analysis performed between Prdm1 and all-expressed genes within the Meredith RNA-seq dataset. Robust Prdm1–associated genes were identified using a cut-off of $p < 0.0005$.

Table S1, Related to Supplemental Figure 3C. Prdm1 associated genes

Gm4884	18318		#N/A	1.000000E+00
Gm4886	18319		#N/A	1.000000E+00
Gm4887	18320		#N/A	1.000000E+00
Gm4889	18321		#N/A	1.000000E+00
Gm4894	18322	-0.038406171	3065	5.892384E-01
Gm4895	18323		#N/A	1.000000E+00
Gm4897	18324		#N/A	1.000000E+00
Gm4899	18325		#N/A	1.000000E+00
Gm4904	18326		#N/A	1.000000E+00
Gm4906	18327		#N/A	1.000000E+00
Gm4907	18328	0.020875719	6412.5	7.692098E-01
Gm4908	18329		#N/A	1.000000E+00
Gm4909	18330		#N/A	1.000000E+00
Gm4910	18331		#N/A	1.000000E+00
Gm4911	18332		#N/A	1.000000E+00
Gm4912	18333		#N/A	1.000000E+00
Gm4913	18334		#N/A	1.000000E+00
Gm4914	18335		#N/A	1.000000E+00
Gm4915	18336		#N/A	1.000000E+00
Gm4916	18337		#N/A	1.000000E+00
Gm4917	18338		#N/A	1.000000E+00
Gm4918	18339		#N/A	1.000000E+00
Gm4919	18340		#N/A	1.000000E+00
Gm4922	18341		#N/A	1.000000E+00
Gm4924	18342	0.103830896	12287	1.434279E-01
Gm4925	18343	0.238084897	19329	6.866010E-04
Gm4926	18344	0.091901353	11512	1.955702E-01
Gm4937	18345	0.160987082	16412.5	2.276801E-02
Gm4938	18346		#N/A	1.000000E+00
Gm4943	18347	-0.038406171	3065	5.892384E-01
Gm4944	18348	0.047227116	8101	5.066413E-01
Gm4945	18349	0.013458335	5965.5	8.499790E-01
Gm4950	18350	0.086412184	11087.5	2.237298E-01
Gm4951	18351	0.159864342	16268	2.374596E-02
Gm4952	18352	0.070806516	9899	3.190899E-01
Gm4953	18353	0.053267784	8685	4.537786E-01
Gm4954	18354		#N/A	1.000000E+00
Gm4955	18355	0.135891096	14691	5.502814E-02
Gm4956	18356		#N/A	1.000000E+00
Gm4963	18357	-0.038406171	3065	5.892384E-01
Gm4968	18358	-0.001224019	5151	9.862757E-01
Gm4969	18359	-0.032450477	3820	6.482730E-01
Gm4975	18360	0.053267784	8685	4.537786E-01

Spearman Rank correlation analysis performed between Prdm1 and all-expressed genes within the Meredith RNA-seq dataset. Robust Prdm1–associated genes were identified using a cut-off of $p < 0.0005$.

Table S1, Related to Supplemental Figure 3C. Prdm1 associated genes

Gm4978	18361		#N/A	1.000000E+00
Gm498	18362	0.113548679	13094	1.093908E-01
Gm4980	18363	-0.038406171	3065	5.892384E-01
Gm4981	18364		#N/A	1.000000E+00
Gm4982	18365		#N/A	1.000000E+00
Gm4984	18366		#N/A	1.000000E+00
Gm4985	18367		#N/A	1.000000E+00
Gm4986	18368	-0.054451513	1703	4.437940E-01
Gm4987	18369	0.022714618	6671	7.495287E-01
Gm4989	18370		#N/A	1.000000E+00
Gm4991	18371		#N/A	1.000000E+00
Gm4992	18372		#N/A	1.000000E+00
Gm4993	18373		#N/A	1.000000E+00
Gm4994	18374		#N/A	1.000000E+00
Gm4995	18375		#N/A	1.000000E+00
Gm4996	18376		#N/A	1.000000E+00
Gm4997	18377	-0.028518853	3979	6.885151E-01
Gm5039	18378	0.154685737	15965	2.873847E-02
Gm5045	18379	-0.038406171	3065	5.892384E-01
Gm5046	18380		#N/A	1.000000E+00
Gm5054	18381	0.043085995	7813	5.446541E-01
Gm5060	18382		#N/A	1.000000E+00
Gm5062	18383		#N/A	1.000000E+00
Gm5065	18384	0.177258985	17279	1.203813E-02
Gm5067	18385		#N/A	1.000000E+00
Gm5068	18386	-0.038406171	3065	5.892384E-01
Gm5069	18387	0.24319479	19418	5.204989E-04
Gm5070	18388	0.068957493	9772	3.319224E-01
Gm5071	18389		#N/A	1.000000E+00
Gm5072	18390		#N/A	1.000000E+00
Gm5073	18391		#N/A	1.000000E+00
Gm5077	18392	-0.054451513	1703	4.437940E-01
Gm5082	18393		#N/A	1.000000E+00
Gm5083	18394	-0.100192745	275	1.580641E-01
Gm5084	18395	-0.066858259	1029.5	3.468868E-01
Gm5087	18396	-0.048613109	2226	4.942352E-01
Gm5089	18397	-0.000458481	5247	9.948591E-01
Gm5093	18398	0.033862427	7288	6.340588E-01
Gm5096	18399	0.047870267	8136	5.008643E-01
Gm5097	18400	-0.038406171	3065	5.892384E-01
Gm5105	18401	0.090185518	11364	2.040807E-01
Gm5108	18402	-0.002894001	5028	9.675583E-01
Gm5111	18403	0.05099615	8383	4.732872E-01

Spearman Rank correlation analysis performed between Prdm1 and all-expressed genes within the Meredith RNA-seq dataset. Robust Prdm1–associated genes were identified using a cut-off of $p < 0.0005$.

Table S1, Related to Supplemental Figure 3C. Prdm1 associated genes

Gm5113	18404	0.168018651	16822.5	1.739831E-02
Gm5114	18405		#N/A	1.000000E+00
Gm5116	18406		#N/A	1.000000E+00
Gm5117	18407		#N/A	1.000000E+00
Gm5121	18408	0.030953612	7126	6.634821E-01
Gm5123	18409		#N/A	1.000000E+00
Gm5124	18410	0.071777786	9979.5	3.124800E-01
Gm5125	18411		#N/A	1.000000E+00
Gm5127	18412	-0.076318229	853	2.827756E-01
Gm5128	18413	-0.038406171	3065	5.892384E-01
Gm5129	18414		#N/A	1.000000E+00
Gm5132	18415		#N/A	1.000000E+00
Gm5133	18416		#N/A	1.000000E+00
Gm5134	18417	0.0231232	6699	7.451772E-01
Gm5135	18418		#N/A	1.000000E+00
Gm5138	18419		#N/A	1.000000E+00
Gm5139	18420		#N/A	1.000000E+00
Gm514	18421	0.102661913	12224.5	1.480137E-01
Gm5141	18422	0.04617463	8025.5	5.161695E-01
Gm5142	18423		#N/A	1.000000E+00
Gm5145	18424		#N/A	1.000000E+00
Gm5148	18425	0.074180476	10151	2.965160E-01
Gm5150	18426	-0.042194928	2432	5.530136E-01
Gm5152	18427		#N/A	1.000000E+00
Gm5155	18428	-0.066856562	1139	3.468991E-01
Gm5160	18429		#N/A	1.000000E+00
Gm5161	18430		#N/A	1.000000E+00
Gm5166	18431		#N/A	1.000000E+00
Gm5167	18432		#N/A	1.000000E+00
Gm5168	18433		#N/A	1.000000E+00
Gm5169	18434	-0.038406171	3065	5.892384E-01
Gm5174	18435		#N/A	1.000000E+00
Gm5185	18436		#N/A	1.000000E+00
Gm5187	18437		#N/A	1.000000E+00
Gm5196	18438		#N/A	1.000000E+00
Gm5215	18439	0.152108591	15776	3.154064E-02
Gm5218	18440		#N/A	1.000000E+00
Gm5225	18441		#N/A	1.000000E+00
Gm5227	18442	-0.038406171	3065	5.892384E-01
Gm5228	18443	0.160987082	16412.5	2.276801E-02
Gm5229	18444		#N/A	1.000000E+00
Gm5239	18445	0.043998631	7866	5.361574E-01
Gm5244	18446	-0.054451513	1703	4.437940E-01

Spearman Rank correlation analysis performed between Prdm1 and all-expressed genes within the Meredith RNA-seq dataset. Robust Prdm1–associated genes were identified using a cut-off of $p < 0.0005$.

Table S1, Related to Supplemental Figure 3C. Prdm1 associated genes

Gm525	18447	0.008596065	5709	9.038439E-01
Gm5250	18448		#N/A	1.000000E+00
Gm5255	18449		#N/A	1.000000E+00
Gm5257	18450	-0.038406171	3065	5.892384E-01
Gm5258	18451	0.024817824	6809	7.272163E-01
Gm5263	18452		#N/A	1.000000E+00
Gm5265	18453	0.143605682	15224	4.249002E-02
Gm527	18454	0.000707108	5349	9.920713E-01
Gm5270	18455	0.143605682	15224	4.249002E-02
Gm5277	18456	-0.066855996	1239.5	3.469032E-01
Gm5278	18457	-0.001224019	5151	9.862757E-01
Gm5283	18458		#N/A	1.000000E+00
Gm5286	18459		#N/A	1.000000E+00
Gm5287	18460		#N/A	1.000000E+00
Gm5292	18461	0.156129434	16056.5	2.726377E-02
Gm5294	18462		#N/A	1.000000E+00
Gm53	18463	-0.038406171	3065	5.892384E-01
Gm5301	18464		#N/A	1.000000E+00
Gm5303	18465		#N/A	1.000000E+00
Gm5316	18466		#N/A	1.000000E+00
Gm5319	18467		#N/A	1.000000E+00
Gm5321	18468		#N/A	1.000000E+00
Gm5327	18469		#N/A	1.000000E+00
Gm5329	18470		#N/A	1.000000E+00
Gm5334	18471		#N/A	1.000000E+00
Gm5341	18472	-0.038406171	3065	5.892384E-01
Gm5346	18473		#N/A	1.000000E+00
Gm5353	18474	-0.054451513	1703	4.437940E-01
Gm5356	18475	0.12587337	14031	7.572780E-02
Gm5379	18476		#N/A	1.000000E+00
Gm5380	18477		#N/A	1.000000E+00
Gm5381	18478		#N/A	1.000000E+00
Gm5382	18479		#N/A	1.000000E+00
Gm5383	18480		#N/A	1.000000E+00
Gm5384	18481	0.095454531	11735	1.787733E-01
Gm5385	18482		#N/A	1.000000E+00
Gm5386	18483	0.113548679	13094	1.093908E-01
Gm5387	18484		#N/A	1.000000E+00
Gm5388	18485		#N/A	1.000000E+00
Gm5389	18486		#N/A	1.000000E+00
Gm5390	18487		#N/A	1.000000E+00
Gm5391	18488	-0.038406171	3065	5.892384E-01
Gm5392	18489	0.018928849	6275	7.902082E-01

Spearman Rank correlation analysis performed between Prdm1 and all-expressed genes within the Meredith RNA-seq dataset. Robust Prdm1–associated genes were identified using a cut-off of $p < 0.0005$.

Table S1, Related to Supplemental Figure 3C. Prdm1 associated genes

Gm5393	18490		#N/A	1.000000E+00
Gm5394	18491		#N/A	1.000000E+00
Gm5395	18492		#N/A	1.000000E+00
Gm5396	18493		#N/A	1.000000E+00
Gm5397	18494		#N/A	1.000000E+00
Gm5398	18495		#N/A	1.000000E+00
Gm5400	18496		#N/A	1.000000E+00
Gm5401	18497		#N/A	1.000000E+00
Gm5402	18498		#N/A	1.000000E+00
Gm5403	18499		#N/A	1.000000E+00
Gm5405	18500		#N/A	1.000000E+00
Gm5406	18501		#N/A	1.000000E+00
Gm5407	18502		#N/A	1.000000E+00
Gm5409	18503		#N/A	1.000000E+00
Gm5414	18504		#N/A	1.000000E+00
Gm5415	18505	0.135118563	14629	5.643710E-02
Gm5416	18506		#N/A	1.000000E+00
Gm5417	18507		#N/A	1.000000E+00
Gm5419	18508		#N/A	1.000000E+00
Gm5420	18509	-0.054450825	1984	4.437997E-01
Gm5422	18510	-0.000841282	5208.5	9.905669E-01
Gm5423	18511		#N/A	1.000000E+00
Gm5424	18512	0.082762295	10811.5	2.439788E-01
Gm5426	18513	0.009014794	5732	8.991843E-01
Gm5428	18514	-0.038406171	3065	5.892384E-01
Gm5431	18515	0.038808337	7573	5.853421E-01
Gm5434	18516	0.137500499	14788.5	5.218623E-02
Gm5435	18517		#N/A	1.000000E+00
Gm5436	18518	0.143605682	15224	4.249002E-02
Gm5444	18519	-0.031924044	3848.5	6.536057E-01
Gm5445	18520	-0.066858259	1029.5	3.468868E-01
Gm5446	18521	-0.038406171	3065	5.892384E-01
Gm5447	18522	-0.038406171	3065	5.892384E-01
Gm5449	18523	0.164478601	16623.5	1.994547E-02
Gm5451	18524	-0.038406171	3065	5.892384E-01
Gm5453	18525	0.053805701	8820	4.492258E-01
Gm5454	18526		#N/A	1.000000E+00
Gm5455	18527		#N/A	1.000000E+00
Gm5456	18528		#N/A	1.000000E+00
Gm5457	18529		#N/A	1.000000E+00
Gm5458	18530	0.091938796	11514	1.953874E-01
Gm5459	18531		#N/A	1.000000E+00
Gm5460	18532	-0.038406171	3065	5.892384E-01

Spearman Rank correlation analysis performed between Prdm1 and all-expressed genes within the Meredith RNA-seq dataset. Robust Prdm1–associated genes were identified using a cut-off of $p < 0.0005$.

Table S1, Related to Supplemental Figure 3C. Prdm1 associated genes

Gm5464	18533	0.079416656	10553	2.636320E-01
Gm5466	18534		#N/A	1.000000E+00
Gm5468	18535		#N/A	1.000000E+00
Gm5471	18536	0.088989401	11270	2.101695E-01
Gm5472	18537		#N/A	1.000000E+00
Gm5473	18538		#N/A	1.000000E+00
Gm5475	18539	0.129719214	14283	6.713774E-02
Gm5478	18540	-0.066855996	1239.5	3.469032E-01
Gm5479	18541		#N/A	1.000000E+00
Gm5480	18542	-0.026446782	4049	7.100904E-01
Gm5483	18543	0.052728522	8550.5	4.583686E-01
Gm5494	18544	-0.086747139	513	2.219331E-01
Gm5496	18545		#N/A	1.000000E+00
Gm5499	18546	0.113548679	13094	1.093908E-01
Gm5501	18547		#N/A	1.000000E+00
Gm5506	18548	0.210510486	18593	2.771491E-03
Gm5507	18549	-0.038406171	3065	5.892384E-01
Gm5510	18550		#N/A	1.000000E+00
Gm5513	18551	-0.038406171	3065	5.892384E-01
Gm5514	18552	0.143605682	15224	4.249002E-02
Gm5518	18553		#N/A	1.000000E+00
Gm5519	18554		#N/A	1.000000E+00
Gm5526	18555	0.021317126	6513.5	7.644715E-01
Gm5528	18556	-0.038406171	3065	5.892384E-01
Gm5529	18557		#N/A	1.000000E+00
Gm5530	18558		#N/A	1.000000E+00
Gm5531	18559	0.053267784	8685	4.537786E-01
Gm5532	18560	0.169813104	16919	1.621886E-02
Gm5533	18561		#N/A	1.000000E+00
Gm5535	18562	0.13354917	14512	5.939122E-02
Gm5537	18563		#N/A	1.000000E+00
Gm5538	18564	-0.038406171	3065	5.892384E-01
Gm5540	18565		#N/A	1.000000E+00
Gm5544	18566	0.091544822	11492	1.973170E-01
Gm5546	18567		#N/A	1.000000E+00
Gm5548	18568		#N/A	1.000000E+00
Gm5549	18569	-0.038406171	3065	5.892384E-01
Gm5555	18570		#N/A	1.000000E+00
Gm5558	18571	-0.000841282	5208.5	9.905669E-01
Gm5559	18572		#N/A	1.000000E+00
Gm5560	18573		#N/A	1.000000E+00
Gm5561	18574		#N/A	1.000000E+00
Gm5564	18575		#N/A	1.000000E+00

Spearman Rank correlation analysis performed between Prdm1 and all-expressed genes within the Meredith RNA-seq dataset. Robust Prdm1–associated genes were identified using a cut-off of $p < 0.0005$.

Table S1, Related to Supplemental Figure 3C. Prdm1 associated genes

Gm5566	18576		#N/A	1.000000E+00
Gm5570	18577	0.063365727	9418	3.727114E-01
Gm5576	18578		#N/A	1.000000E+00
Gm5577	18579	0.134526338	14592	5.753728E-02
Gm5578	18580		#N/A	1.000000E+00
Gm5580	18581		#N/A	1.000000E+00
Gm5581	18582	-0.038406171	3065	5.892384E-01
Gm5582	18583		#N/A	1.000000E+00
Gm5583	18584		#N/A	1.000000E+00
Gm5590	18585		#N/A	1.000000E+00
Gm5591	18586		#N/A	1.000000E+00
Gm5592	18587		#N/A	1.000000E+00
Gm5593	18588		#N/A	1.000000E+00
Gm5594	18589		#N/A	1.000000E+00
Gm5595	18590	-0.084440248	582	2.345165E-01
Gm5600	18591		#N/A	1.000000E+00
Gm5601	18592	0.066707032	9632	3.479811E-01
Gm5602	18593		#N/A	1.000000E+00
Gm5607	18594	-0.042375177	2425	5.513176E-01
Gm561	18595	0.051934249	8467	4.651759E-01
Gm5611	18596	0.038758411	7558.5	5.858252E-01
Gm5612	18597		#N/A	1.000000E+00
Gm5614	18598	-0.038406171	3065	5.892384E-01
Gm5615	18599	-0.086750697	472.5	2.219141E-01
Gm5616	18600		#N/A	1.000000E+00
Gm5617	18601	0.055385133	8950	4.360076E-01
Gm5619	18602	0.113548679	13094	1.093908E-01
Gm5620	18603	-0.086750697	472.5	2.219141E-01
Gm5621	18604	-0.076076434	860	2.843079E-01
Gm5622	18605	-0.057249648	1452.5	4.206938E-01
Gm5624	18606		#N/A	1.000000E+00
Gm5627	18607	-0.054450825	1984	4.437997E-01
Gm5633	18608	0.04546004	7966	5.226910E-01
Gm5634	18609		#N/A	1.000000E+00
Gm5635	18610		#N/A	1.000000E+00
Gm5637	18611		#N/A	1.000000E+00
Gm5638	18612		#N/A	1.000000E+00
Gm5639	18613		#N/A	1.000000E+00
Gm5640	18614		#N/A	1.000000E+00
Gm5641	18615		#N/A	1.000000E+00
Gm5643	18616	0.090712775	11403	2.014375E-01
Gm5644	18617	0.002449701	5440	9.725367E-01
Gm5645	18618		#N/A	1.000000E+00

Spearman Rank correlation analysis performed between Prdm1 and all-expressed genes within the Meredith RNA-seq dataset. Robust Prdm1–associated genes were identified using a cut-off of $p < 0.0005$.

Table S1, Related to Supplemental Figure 3C. Prdm1 associated genes

Gm5646	18619		#N/A	1.000000E+00
Gm5648	18620		#N/A	1.000000E+00
Gm5656	18621		#N/A	1.000000E+00
Gm5662	18622	0.113548679	13094	1.093908E-01
Gm5665	18623	-0.044270386	2358	5.336402E-01
Gm5670	18624	-0.054451513	1703	4.437940E-01
Gm5678	18625		#N/A	1.000000E+00
Gm568	18626		#N/A	1.000000E+00
Gm5682	18627		#N/A	1.000000E+00
Gm5687	18628	-0.038406171	3065	5.892384E-01
Gm5689	18629		#N/A	1.000000E+00
Gm5697	18630		#N/A	1.000000E+00
Gm5698	18631		#N/A	1.000000E+00
Gm5699	18632	0.162808768	16526.5	2.125512E-02
Gm5705	18633		#N/A	1.000000E+00
Gm5708	18634		#N/A	1.000000E+00
Gm5709	18635	0.24756935	19476	4.087104E-04
Gm572	18636	0.117559753	13496	9.734193E-02
Gm5721	18637		#N/A	1.000000E+00
Gm5724	18638	-0.016731569	4428.5	8.140896E-01
Gm5725	18639		#N/A	1.000000E+00
Gm5726	18640		#N/A	1.000000E+00
Gm5728	18641		#N/A	1.000000E+00
Gm5741	18642		#N/A	1.000000E+00
Gm5742	18643	-0.066858259	1029.5	3.468868E-01
Gm5745	18644		#N/A	1.000000E+00
Gm5751	18645		#N/A	1.000000E+00
Gm5752	18646		#N/A	1.000000E+00
Gm5753	18647		#N/A	1.000000E+00
Gm5754	18648		#N/A	1.000000E+00
Gm5755	18649		#N/A	1.000000E+00
Gm5757	18650		#N/A	1.000000E+00
Gm5759	18651	0.053267784	8685	4.537786E-01
Gm5760	18652		#N/A	1.000000E+00
Gm5761	18653		#N/A	1.000000E+00
Gm5762	18654	-0.054451513	1703	4.437940E-01
Gm5763	18655		#N/A	1.000000E+00
Gm5764	18656	0.088653549	11249	2.119024E-01
Gm5766	18657	0.098061128	11918	1.671459E-01
Gm5771	18658		#N/A	1.000000E+00
Gm5773	18659		#N/A	1.000000E+00
Gm5776	18660	0.238192961	19332	6.826325E-04
Gm5777	18661	-0.066156793	1302	3.519808E-01

Spearman Rank correlation analysis performed between Prdm1 and all-expressed genes within the Meredith RNA-seq dataset. Robust Prdm1–associated genes were identified using a cut-off of $p < 0.0005$.

Table S1, Related to Supplemental Figure 3C. Prdm1 associated genes

Gm5778	18662	0.204739563	18415	3.635432E-03
Gm5784	18663		#N/A	1.000000E+00
Gm5786	18664	-0.054451513	1703	4.437940E-01
Gm5792	18665		#N/A	1.000000E+00
Gm5793	18666		#N/A	1.000000E+00
Gm5795	18667		#N/A	1.000000E+00
Gm5796	18668	-0.086748028	498.5	2.219283E-01
Gm5797	18669	0.05724748	9048	4.207114E-01
Gm5798	18670		#N/A	1.000000E+00
Gm5799	18671		#N/A	1.000000E+00
Gm5800	18672	0.113548679	13094	1.093908E-01
Gm5801	18673		#N/A	1.000000E+00
Gm5803	18674	0.164478601	16623.5	1.994547E-02
Gm5805	18675		#N/A	1.000000E+00
Gm5806	18676	-0.038406171	3065	5.892384E-01
Gm5808	18677		#N/A	1.000000E+00
Gm5809	18678		#N/A	1.000000E+00
Gm581	18679		#N/A	1.000000E+00
Gm5812	18680		#N/A	1.000000E+00
Gm5814	18681		#N/A	1.000000E+00
Gm5815	18682		#N/A	1.000000E+00
Gm5819	18683		#N/A	1.000000E+00
Gm5822	18684	0.1544596	15936.5	2.897553E-02
Gm5828	18685		#N/A	1.000000E+00
Gm5830	18686		#N/A	1.000000E+00
Gm5832	18687		#N/A	1.000000E+00
Gm5834	18688	0.021317126	6513.5	7.644715E-01
Gm5843	18689		#N/A	1.000000E+00
Gm5844	18690	0.091107583	11449	1.994746E-01
Gm5849	18691		#N/A	1.000000E+00
Gm5852	18692		#N/A	1.000000E+00
Gm5859	18693		#N/A	1.000000E+00
Gm5860	18694	0.168625279	16859	1.699150E-02
Gm5861	18695		#N/A	1.000000E+00
Gm5862	18696		#N/A	1.000000E+00
Gm5863	18697	0.021317126	6513.5	7.644715E-01
Gm5866	18698		#N/A	1.000000E+00
Gm5867	18699		#N/A	1.000000E+00
Gm5868	18700	-0.103166823	231.5	1.460195E-01
Gm5871	18701		#N/A	1.000000E+00
Gm5873	18702		#N/A	1.000000E+00
Gm5874	18703		#N/A	1.000000E+00
Gm5878	18704		#N/A	1.000000E+00

Spearman Rank correlation analysis performed between Prdm1 and all-expressed genes within the Meredith RNA-seq dataset. Robust Prdm1–associated genes were identified using a cut-off of $p < 0.0005$.

Table S1, Related to Supplemental Figure 3C. Prdm1 associated genes

Gm5879	18705		#N/A	1.000000E+00
Gm5883	18706	-0.001224019	5151	9.862757E-01
Gm5884	18707	0.176720821	17252	1.230487E-02
Gm5885	18708		#N/A	1.000000E+00
Gm5889	18709		#N/A	1.000000E+00
Gm5891	18710		#N/A	1.000000E+00
Gm5893	18711	0.195544906	18064	5.521378E-03
Gm5898	18712		#N/A	1.000000E+00
Gm5899	18713		#N/A	1.000000E+00
Gm590	18714	0.113548679	13094	1.093908E-01
Gm5900	18715	0.271024979	19726	1.037069E-04
Gm5901	18716		#N/A	1.000000E+00
Gm5903	18717		#N/A	1.000000E+00
Gm5908	18718	0.141397021	15040	4.580701E-02
Gm5909	18719		#N/A	1.000000E+00
Gm5912	18720		#N/A	1.000000E+00
Gm5913	18721		#N/A	1.000000E+00
Gm5915	18722	0.021317126	6513.5	7.644715E-01
Gm5916	18723	-0.038406171	3065	5.892384E-01
Gm5918	18724	0.044155818	7878	5.347007E-01
Gm5919	18725		#N/A	1.000000E+00
Gm5921	18726	0.160304018	16323	2.335876E-02
Gm5924	18727		#N/A	1.000000E+00
Gm5925	18728		#N/A	1.000000E+00
Gm5926	18729		#N/A	1.000000E+00
Gm5929	18730		#N/A	1.000000E+00
Gm5930	18731	-0.054450825	1984	4.437997E-01
Gm5931	18732		#N/A	1.000000E+00
Gm5934	18733	0.207821138	18518	3.147821E-03
Gm5935	18734		#N/A	1.000000E+00
Gm5936	18735		#N/A	1.000000E+00
Gm5937	18736		#N/A	1.000000E+00
Gm5938	18737	0.021317126	6513.5	7.644715E-01
Gm5939	18738		#N/A	1.000000E+00
Gm5940	18739	0.211289804	18623	2.670332E-03
Gm5941	18740	-0.066856562	1139	3.468991E-01
Gm5942	18741	0.138291906	14844	5.083408E-02
Gm5943	18742		#N/A	1.000000E+00
Gm5944	18743	-0.038406171	3065	5.892384E-01
Gm5946	18744		#N/A	1.000000E+00
Gm5947	18745	0.143605682	15224	4.249002E-02
Gm595	18746	0.022198129	6607.5	7.550408E-01
Gm5958	18747	0.083452862	10867	2.400529E-01

Spearman Rank correlation analysis performed between Prdm1 and all-expressed genes within the Meredith RNA-seq dataset. Robust Prdm1–associated genes were identified using a cut-off of $p < 0.0005$.

Table S1, Related to Supplemental Figure 3C. Prdm1 associated genes

Gm5963	18748	0.00784376	5674	9.122239E-01
Gm5965	18749		#N/A	1.000000E+00
Gm5967	18750	0.014301797	6015.5	8.406982E-01
Gm597	18751		#N/A	1.000000E+00
Gm5970	18752		#N/A	1.000000E+00
Gm5973	18753		#N/A	1.000000E+00
Gm5977	18754	0.038757099	7545	5.858379E-01
Gm5989	18755		#N/A	1.000000E+00
Gm6004	18756		#N/A	1.000000E+00
Gm6019	18757		#N/A	1.000000E+00
Gm6020	18758		#N/A	1.000000E+00
Gm6023	18759	0.111780213	12835	1.150615E-01
Gm6024	18760		#N/A	1.000000E+00
Gm6025	18761	-0.066858259	1029.5	3.468868E-01
Gm6026	18762		#N/A	1.000000E+00
Gm6027	18763		#N/A	1.000000E+00
Gm6030	18764	0.068624675	9747	3.342668E-01
Gm6031	18765		#N/A	1.000000E+00
Gm6034	18766	-0.066856562	1139	3.468991E-01
Gm6038	18767		#N/A	1.000000E+00
Gm6039	18768		#N/A	1.000000E+00
Gm6040	18769	-0.032762514	3799	6.451205E-01
Gm6043	18770		#N/A	1.000000E+00
Gm6044	18771		#N/A	1.000000E+00
Gm6047	18772		#N/A	1.000000E+00
Gm6054	18773		#N/A	1.000000E+00
Gm6055	18774	-0.038406171	3065	5.892384E-01
Gm6058	18775		#N/A	1.000000E+00
Gm6061	18776		#N/A	1.000000E+00
Gm6062	18777	0.113548679	13094	1.093908E-01
Gm6065	18778	0.046025613	8011	5.175260E-01
Gm6067	18779	0.089762094	11328	2.062214E-01
Gm6071	18780	0.048448014	8175.5	4.957045E-01
Gm6079	18781		#N/A	1.000000E+00
Gm608	18782	0.092982169	11584	1.903437E-01
Gm6081	18783		#N/A	1.000000E+00
Gm6083	18784		#N/A	1.000000E+00
Gm6084	18785		#N/A	1.000000E+00
Gm6086	18786	0.071481206	9950	3.144888E-01
Gm6088	18787	0.085706783	11033	2.275472E-01
Gm609	18788	-0.077397959	691.5	2.760011E-01
Gm6091	18789		#N/A	1.000000E+00
Gm6092	18790		#N/A	1.000000E+00

Spearman Rank correlation analysis performed between Prdm1 and all-expressed genes within the Meredith RNA-seq dataset. Robust Prdm1–associated genes were identified using a cut-off of $p < 0.0005$.

Table S1, Related to Supplemental Figure 3C. Prdm1 associated genes

Gm6096	18791	-0.045149205	2323	5.255407E-01
Gm6097	18792		#N/A	1.000000E+00
Gm6098	18793		#N/A	1.000000E+00
Gm6100	18794	0.083452862	10867	2.400529E-01
Gm6104	18795		#N/A	1.000000E+00
Gm6113	18796		#N/A	1.000000E+00
Gm6115	18797		#N/A	1.000000E+00
Gm6116	18798	0.022198316	6629.5	7.550388E-01
Gm6117	18799		#N/A	1.000000E+00
Gm6121	18800		#N/A	1.000000E+00
Gm6132	18801		#N/A	1.000000E+00
Gm6133	18802	0.096985606	11836.5	1.718733E-01
Gm6134	18803	-0.038406171	3065	5.892384E-01
Gm6136	18804	0.175810729	17206	1.276778E-02
Gm6139	18805		#N/A	1.000000E+00
Gm614	18806		#N/A	1.000000E+00
Gm6141	18807		#N/A	1.000000E+00
Gm6142	18808	0.113548679	13094	1.093908E-01
Gm6152	18809		#N/A	1.000000E+00
Gm6153	18810		#N/A	1.000000E+00
Gm6155	18811	0.027375091	6948	7.003947E-01
Gm6158	18812		#N/A	1.000000E+00
Gm6161	18813		#N/A	1.000000E+00
Gm6164	18814		#N/A	1.000000E+00
Gm6166	18815	0.109642127	12686	1.222210E-01
Gm6168	18816		#N/A	1.000000E+00
Gm6169	18817	-0.018447724	4312.5	7.954215E-01
Gm6175	18818		#N/A	1.000000E+00
Gm6176	18819		#N/A	1.000000E+00
Gm6177	18820	-0.066858259	1029.5	3.468868E-01
Gm6180	18821		#N/A	1.000000E+00
Gm6181	18822		#N/A	1.000000E+00
Gm6182	18823		#N/A	1.000000E+00
Gm6184	18824		#N/A	1.000000E+00
Gm6189	18825	-0.054451513	1703	4.437940E-01
Gm6195	18826		#N/A	1.000000E+00
Gm6205	18827		#N/A	1.000000E+00
Gm6206	18828	0.074574898	10201.5	2.939481E-01
Gm6207	18829		#N/A	1.000000E+00
Gm6211	18830		#N/A	1.000000E+00
Gm6214	18831		#N/A	1.000000E+00
Gm6215	18832		#N/A	1.000000E+00
Gm6221	18833		#N/A	1.000000E+00

Spearman Rank correlation analysis performed between Prdm1 and all-expressed genes within the Meredith RNA-seq dataset. Robust Prdm1–associated genes were identified using a cut-off of $p < 0.0005$.

Table S1, Related to Supplemental Figure 3C. Prdm1 associated genes

Gm6222	18834	-0.124258856	81	7.958975E-02
Gm6223	18835		#N/A	1.000000E+00
Gm6226	18836		#N/A	1.000000E+00
Gm6228	18837		#N/A	1.000000E+00
Gm6238	18838		#N/A	1.000000E+00
Gm6245	18839		#N/A	1.000000E+00
Gm6253	18840		#N/A	1.000000E+00
Gm6254	18841		#N/A	1.000000E+00
Gm6257	18842		#N/A	1.000000E+00
Gm6263	18843		#N/A	1.000000E+00
Gm6264	18844	-0.038406171	3065	5.892384E-01
Gm6265	18845	0.126933796	14110.5	7.327484E-02
Gm6268	18846		#N/A	1.000000E+00
Gm6269	18847		#N/A	1.000000E+00
Gm6270	18848	-0.038406171	3065	5.892384E-01
Gm6272	18849	0.04235856	7764	5.514738E-01
Gm6274	18850	-0.036680126	3667	6.060937E-01
Gm6275	18851		#N/A	1.000000E+00
Gm6276	18852	-0.066858259	1029.5	3.468868E-01
Gm6285	18853	0.062793037	9379	3.770561E-01
Gm6286	18854		#N/A	1.000000E+00
Gm6288	18855	-0.103166823	231.5	1.460195E-01
Gm6289	18856		#N/A	1.000000E+00
Gm6291	18857	0.083035263	10837	2.424217E-01
Gm6292	18858		#N/A	1.000000E+00
Gm6293	18859	-0.038406171	3065	5.892384E-01
Gm6298	18860		#N/A	1.000000E+00
Gm6304	18861		#N/A	1.000000E+00
Gm6305	18862		#N/A	1.000000E+00
Gm6309	18863		#N/A	1.000000E+00
Gm6310	18864	0.113548679	13094	1.093908E-01
Gm6311	18865		#N/A	1.000000E+00
Gm6313	18866	-0.014870399	4515	8.344540E-01
Gm6316	18867	0.143605682	15224	4.249002E-02
Gm6322	18868		#N/A	1.000000E+00
Gm6325	18869	0.074574898	10201.5	2.939481E-01
Gm6327	18870		#N/A	1.000000E+00
Gm6335	18871	-0.054451513	1703	4.437940E-01
Gm6337	18872		#N/A	1.000000E+00
Gm6341	18873	-0.054451513	1703	4.437940E-01
Gm6344	18874		#N/A	1.000000E+00
Gm6351	18875		#N/A	1.000000E+00
Gm6356	18876		#N/A	1.000000E+00

Spearman Rank correlation analysis performed between Prdm1 and all-expressed genes within the Meredith RNA-seq dataset. Robust Prdm1–associated genes were identified using a cut-off of $p < 0.0005$.

Table S1, Related to Supplemental Figure 3C. Prdm1 associated genes

Gm6358	18877		#N/A	1.000000E+00
Gm6361	18878		#N/A	1.000000E+00
Gm6365	18879		#N/A	1.000000E+00
GM6367	18880		#N/A	1.000000E+00
Gm6367	18881	0.076051619	10351.5	2.844655E-01
Gm6368	18882	0.020721525	6382	7.708670E-01
Gm6369	18883	-0.038406171	3065	5.892384E-01
Gm6370	18884		#N/A	1.000000E+00
Gm6373	18885	-0.038406171	3065	5.892384E-01
Gm6374	18886		#N/A	1.000000E+00
Gm6377	18887	3.34562E-05	5293	9.996249E-01
Gm6378	18888	-0.095273984	329	1.796002E-01
Gm6382	18889		#N/A	1.000000E+00
Gm6395	18890	0.134087541	14557	5.836380E-02
Gm6397	18891		#N/A	1.000000E+00
Gm6401	18892		#N/A	1.000000E+00
Gm6404	18893	0.242080059	19397	5.531936E-04
Gm6406	18894		#N/A	1.000000E+00
Gm6407	18895	-0.038406171	3065	5.892384E-01
Gm6408	18896		#N/A	1.000000E+00
Gm6415	18897	-0.038406171	3065	5.892384E-01
Gm6418	18898		#N/A	1.000000E+00
Gm6419	18899		#N/A	1.000000E+00
Gm6420	18900		#N/A	1.000000E+00
Gm6421	18901		#N/A	1.000000E+00
Gm6425	18902	0.050870414	8368.5	4.743803E-01
Gm6427	18903		#N/A	1.000000E+00
Gm6428	18904	-0.054451513	1703	4.437940E-01
Gm6429	18905	0.074574898	10201.5	2.939481E-01
Gm6430	18906		#N/A	1.000000E+00
Gm6432	18907	-0.054451513	1703	4.437940E-01
Gm6433	18908	-0.000841282	5208.5	9.905669E-01
Gm6434	18909		#N/A	1.000000E+00
Gm6441	18910		#N/A	1.000000E+00
Gm6444	18911	0.053267784	8685	4.537786E-01
Gm6446	18912		#N/A	1.000000E+00
Gm6447	18913		#N/A	1.000000E+00
Gm6451	18914		#N/A	1.000000E+00
Gm6455	18915		#N/A	1.000000E+00
Gm6457	18916		#N/A	1.000000E+00
Gm6460	18917		#N/A	1.000000E+00
Gm6461	18918		#N/A	1.000000E+00
Gm6462	18919	0.086412184	11087.5	2.237298E-01

Spearman Rank correlation analysis performed between Prdm1 and all-expressed genes within the Meredith RNA-seq dataset. Robust Prdm1–associated genes were identified using a cut-off of $p < 0.0005$.

Table S1, Related to Supplemental Figure 3C. Prdm1 associated genes

Gm6465	18920		#N/A	1.000000E+00
Gm6467	18921		#N/A	1.000000E+00
Gm6468	18922		#N/A	1.000000E+00
Gm6471	18923		#N/A	1.000000E+00
Gm6472	18924		#N/A	1.000000E+00
Gm6478	18925		#N/A	1.000000E+00
Gm648	18926	-0.038406171	3065	5.892384E-01
Gm6480	18927		#N/A	1.000000E+00
Gm6482	18928	0.152789767	15822	3.077826E-02
Gm6483	18929	-0.054450825	1984	4.437997E-01
Gm6484	18930	-0.054450825	1984	4.437997E-01
Gm6485	18931		#N/A	1.000000E+00
Gm6486	18932		#N/A	1.000000E+00
Gm6489	18933	-0.054451513	1703	4.437940E-01
Gm649	18934		#N/A	1.000000E+00
Gm6490	18935		#N/A	1.000000E+00
Gm6491	18936	-0.038406171	3065	5.892384E-01
Gm6493	18937		#N/A	1.000000E+00
Gm6498	18938	0.109643527	12688	1.222162E-01
Gm650	18939		#N/A	1.000000E+00
Gm6500	18940	0.152789767	15822	3.077826E-02
Gm6502	18941		#N/A	1.000000E+00
Gm6505	18942		#N/A	1.000000E+00
Gm6506	18943		#N/A	1.000000E+00
Gm6507	18944		#N/A	1.000000E+00
Gm6509	18945		#N/A	1.000000E+00
Gm6517	18946	-0.038406171	3065	5.892384E-01
Gm6526	18947		#N/A	1.000000E+00
Gm6531	18948	0.038700666	7541	5.863841E-01
Gm6536	18949		#N/A	1.000000E+00
Gm6537	18950		#N/A	1.000000E+00
Gm6539	18951		#N/A	1.000000E+00
Gm6541	18952		#N/A	1.000000E+00
Gm6542	18953		#N/A	1.000000E+00
Gm6548	18954	0.140683365	15000	4.692400E-02
Gm6563	18955	-0.038406171	3065	5.892384E-01
Gm6564	18956	0.156717411	16094	2.668201E-02
Gm6565	18957		#N/A	1.000000E+00
Gm6568	18958		#N/A	1.000000E+00
Gm6569	18959	0.020875719	6412.5	7.692098E-01
Gm6570	18960		#N/A	1.000000E+00
Gm6574	18961		#N/A	1.000000E+00
Gm6576	18962	0.113548679	13094	1.093908E-01

Spearman Rank correlation analysis performed between Prdm1 and all-expressed genes within the Meredith RNA-seq dataset. Robust Prdm1–associated genes were identified using a cut-off of $p < 0.0005$.

Gm6578	18963	-0.000841282	5208.5	9.905669E-01
Gm6580	18964		#N/A	1.000000E+00
Gm6583	18965		#N/A	1.000000E+00
Gm6584	18966	-0.038406171	3065	5.892384E-01
Gm6586	18967		#N/A	1.000000E+00
Gm6587	18968		#N/A	1.000000E+00
Gm6588	18969	0.021317126	6513.5	7.644715E-01
Gm6591	18970		#N/A	1.000000E+00
Gm6592	18971		#N/A	1.000000E+00
Gm6594	18972		#N/A	1.000000E+00
Gm6604	18973		#N/A	1.000000E+00
Gm6605	18974	0.125673067	14015	7.619852E-02
Gm6611	18975		#N/A	1.000000E+00
Gm6612	18976		#N/A	1.000000E+00
Gm6614	18977	-0.052308206	2155	4.619640E-01
Gm6619	18978		#N/A	1.000000E+00
Gm6620	18979		#N/A	1.000000E+00
Gm6623	18980		#N/A	1.000000E+00
Gm6625	18981		#N/A	1.000000E+00
Gm6630	18982		#N/A	1.000000E+00
Gm6632	18983		#N/A	1.000000E+00
Gm6633	18984		#N/A	1.000000E+00
Gm6640	18985		#N/A	1.000000E+00
Gm6644	18986		#N/A	1.000000E+00
Gm6645	18987		#N/A	1.000000E+00
Gm6647	18988		#N/A	1.000000E+00
Gm6650	18989		#N/A	1.000000E+00
Gm6654	18990	0.064123369	9476.5	3.670112E-01
Gm6658	18991		#N/A	1.000000E+00
Gm6659	18992		#N/A	1.000000E+00
Gm6660	18993		#N/A	1.000000E+00
Gm6664	18994		#N/A	1.000000E+00
Gm6665	18995		#N/A	1.000000E+00
Gm6673	18996		#N/A	1.000000E+00
Gm6676	18997		#N/A	1.000000E+00
Gm6682	18998		#N/A	1.000000E+00
Gm6684	18999		#N/A	1.000000E+00
Gm6685	19000		#N/A	1.000000E+00
Gm6686	19001		#N/A	1.000000E+00
Gm6689	19002	0.074574898	10201.5	2.939481E-01
Gm6695	19003		#N/A	1.000000E+00
Gm6696	19004		#N/A	1.000000E+00
Gm6706	19005		#N/A	1.000000E+00

Table S1, Related to Supplemental Figure 3C. Prdm1 associated genes

Gm6710	19006		#N/A	1.000000E+00
Gm6712	19007	-0.086746694	532	2.219355E-01
Gm6723	19008		#N/A	1.000000E+00
Gm6726	19009		#N/A	1.000000E+00
Gm6728	19010		#N/A	1.000000E+00
Gm6729	19011		#N/A	1.000000E+00
Gm6733	19012		#N/A	1.000000E+00
Gm6736	19013		#N/A	1.000000E+00
Gm6740	19014		#N/A	1.000000E+00
Gm6741	19015		#N/A	1.000000E+00
Gm6743	19016		#N/A	1.000000E+00
Gm6744	19017		#N/A	1.000000E+00
Gm6746	19018		#N/A	1.000000E+00
Gm6754	19019		#N/A	1.000000E+00
Gm6755	19020		#N/A	1.000000E+00
Gm6756	19021	0.113548679	13094	1.093908E-01
Gm6758	19022		#N/A	1.000000E+00
Gm6760	19023		#N/A	1.000000E+00
Gm6762	19024		#N/A	1.000000E+00
GM6763	19025		#N/A	1.000000E+00
Gm6763	19026		#N/A	1.000000E+00
Gm6767	19027	0.184948651	17632	8.745843E-03
Gm6768	19028	0.060718693	9247	3.930507E-01
Gm6772	19029	-0.054450825	1984	4.437997E-01
Gm6776	19030		#N/A	1.000000E+00
Gm6781	19031		#N/A	1.000000E+00
Gm6783	19032		#N/A	1.000000E+00
Gm6787	19033	-0.074938591	884	2.915934E-01
Gm6788	19034	-0.038406171	3065	5.892384E-01
Gm6792	19035	0.143843347	15300	4.214543E-02
Gm6793	19036	-0.038406171	3065	5.892384E-01
Gm6797	19037		#N/A	1.000000E+00
Gm6798	19038		#N/A	1.000000E+00
Gm6802	19039		#N/A	1.000000E+00
Gm6803	19040		#N/A	1.000000E+00
Gm6806	19041		#N/A	1.000000E+00
Gm6807	19042	0.044495949	7898.5	5.315554E-01
Gm6808	19043	0.126933796	14110.5	7.327484E-02
Gm6811	19044		#N/A	1.000000E+00
Gm6812	19045		#N/A	1.000000E+00
Gm6816	19046		#N/A	1.000000E+00
Gm6821	19047		#N/A	1.000000E+00
Gm6822	19048		#N/A	1.000000E+00

Spearman Rank correlation analysis performed between Prdm1 and all-expressed genes within the Meredith RNA-seq dataset. Robust Prdm1–associated genes were identified using a cut-off of $p < 0.0005$.

Table S1, Related to Supplemental Figure 3C. Prdm1 associated genes

Gm6823	19049		#N/A	1.000000E+00
Gm6826	19050		#N/A	1.000000E+00
Gm6829	19051		#N/A	1.000000E+00
Gm6838	19052		#N/A	1.000000E+00
Gm684	19053	-0.044561427	2351	5.309510E-01
Gm6851	19054	0.132263077	14430	6.190598E-02
Gm6863	19055	0.022714618	6671	7.495287E-01
Gm6871	19056	0.053267784	8685	4.537786E-01
Gm6872	19057		#N/A	1.000000E+00
Gm6877	19058		#N/A	1.000000E+00
Gm6878	19059		#N/A	1.000000E+00
Gm6880	19060		#N/A	1.000000E+00
Gm6881	19061	0.113548679	13094	1.093908E-01
Gm6882	19062		#N/A	1.000000E+00
Gm6884	19063		#N/A	1.000000E+00
Gm6887	19064	-0.038406171	3065	5.892384E-01
Gm6890	19065		#N/A	1.000000E+00
Gm6893	19066		#N/A	1.000000E+00
Gm6897	19067		#N/A	1.000000E+00
Gm6899	19068		#N/A	1.000000E+00
Gm6900	19069	0.044087692	7873	5.353318E-01
Gm6901	19070		#N/A	1.000000E+00
Gm6902	19071		#N/A	1.000000E+00
Gm6903	19072		#N/A	1.000000E+00
Gm6904	19073	-0.083624126	595	2.390861E-01
Gm6910	19074		#N/A	1.000000E+00
Gm6912	19075		#N/A	1.000000E+00
Gm6913	19076	-0.066156793	1302	3.519808E-01
Gm6917	19077		#N/A	1.000000E+00
Gm6918	19078		#N/A	1.000000E+00
Gm6919	19079		#N/A	1.000000E+00
Gm6921	19080	-0.054450825	1984	4.437997E-01
Gm6923	19081		#N/A	1.000000E+00
Gm6927	19082		#N/A	1.000000E+00
Gm6938	19083		#N/A	1.000000E+00
Gm6939	19084	0.113548679	13094	1.093908E-01
Gm694	19085		#N/A	1.000000E+00
Gm6952	19086		#N/A	1.000000E+00
Gm6954	19087	0.075715395	10317.5	2.866063E-01
Gm6956	19088		#N/A	1.000000E+00
Gm6960	19089		#N/A	1.000000E+00
Gm6963	19090		#N/A	1.000000E+00
Gm6964	19091		#N/A	1.000000E+00

Spearman Rank correlation analysis performed between Prdm1 and all-expressed genes within the Meredith RNA-seq dataset. Robust Prdm1–associated genes were identified using a cut-off of $p < 0.0005$.

Table S1, Related to Supplemental Figure 3C. Prdm1 associated genes

Gm6969	19092		#N/A	1.000000E+00
Gm6970	19093		#N/A	1.000000E+00
Gm6973	19094		#N/A	1.000000E+00
Gm6977	19095	0.188804702	17792.5	7.417613E-03
Gm6982	19096		#N/A	1.000000E+00
Gm6984	19097	-0.054451513	1703	4.437940E-01
Gm6985	19098		#N/A	1.000000E+00
Gm6987	19099	0.167392262	16790	1.782725E-02
Gm6990	19100	-0.038406171	3065	5.892384E-01
Gm6994	19101	0.012356402	5895	8.621349E-01
Gm7008	19102	0.113548679	13094	1.093908E-01
Gm7023	19103		#N/A	1.000000E+00
Gm7027	19104	-0.066858259	1029.5	3.468868E-01
Gm7029	19105		#N/A	1.000000E+00
Gm7030	19106	0.096408587	11801	1.744501E-01
Gm7031	19107		#N/A	1.000000E+00
Gm7052	19108		#N/A	1.000000E+00
Gm7058	19109		#N/A	1.000000E+00
Gm7061	19110		#N/A	1.000000E+00
Gm7071	19111		#N/A	1.000000E+00
Gm7073	19112	0.110455893	12754.5	1.194564E-01
Gm7075	19113		#N/A	1.000000E+00
Gm7076	19114		#N/A	1.000000E+00
Gm7079	19115	0.142599903	15122	4.397463E-02
Gm7083	19116		#N/A	1.000000E+00
Gm7091	19117	0.14133844	15036	4.589785E-02
Gm7092	19118	0.271759936	19732	9.913811E-05
Gm7094	19119		#N/A	1.000000E+00
Gm7097	19120		#N/A	1.000000E+00
Gm7099	19121		#N/A	1.000000E+00
Gm7102	19122	0.052043297	8474	4.642380E-01
Gm7104	19123		#N/A	1.000000E+00
Gm7108	19124		#N/A	1.000000E+00
Gm711	19125	0.013930108	5986	8.447854E-01
Gm7110	19126	0.065875074	9581	3.540397E-01
Gm7113	19127		#N/A	1.000000E+00
Gm7117	19128		#N/A	1.000000E+00
Gm7120	19129	0.010547879	5793	8.821544E-01
Gm7123	19130	0.025544243	6849.5	7.195619E-01
Gm7125	19131		#N/A	1.000000E+00
Gm7129	19132	-0.038406171	3065	5.892384E-01
Gm7134	19133		#N/A	1.000000E+00
Gm7137	19134		#N/A	1.000000E+00

Spearman Rank correlation analysis performed between Prdm1 and all-expressed genes within the Meredith RNA-seq dataset. Robust Prdm1–associated genes were identified using a cut-off of $p < 0.0005$.

Table S1, Related to Supplemental Figure 3C. Prdm1 associated genes

Gm7138	19135		#N/A	1.000000E+00
Gm7146	19136		#N/A	1.000000E+00
Gm7148	19137		#N/A	1.000000E+00
Gm715	19138		#N/A	1.000000E+00
Gm7150	19139		#N/A	1.000000E+00
Gm7152	19140		#N/A	1.000000E+00
Gm7153	19141		#N/A	1.000000E+00
Gm7155	19142	-0.038406171	3065	5.892384E-01
Gm7157	19143		#N/A	1.000000E+00
Gm7158	19144		#N/A	1.000000E+00
Gm7162	19145		#N/A	1.000000E+00
Gm7164	19146		#N/A	1.000000E+00
Gm7168	19147	-0.038406171	3065	5.892384E-01
Gm7171	19148		#N/A	1.000000E+00
Gm7173	19149	0.156129434	16056.5	2.726377E-02
Gm7180	19150		#N/A	1.000000E+00
Gm7189	19151	-0.038406171	3065	5.892384E-01
Gm7190	19152	0.113548679	13094	1.093908E-01
Gm7199	19153	-0.038406171	3065	5.892384E-01
Gm7204	19154		#N/A	1.000000E+00
Gm7206	19155	0.224798783	19038.5	1.372995E-03
Gm7209	19156		#N/A	1.000000E+00
Gm7211	19157		#N/A	1.000000E+00
Gm7212	19158		#N/A	1.000000E+00
Gm7219	19159	0.021317126	6513.5	7.644715E-01
Gm7221	19160	0.157799267	16153.5	2.563935E-02
Gm7222	19161	-0.077397959	691.5	2.760011E-01
Gm7224	19162		#N/A	1.000000E+00
Gm7230	19163		#N/A	1.000000E+00
Gm7233	19164	-0.038406171	3065	5.892384E-01
Gm7235	19165		#N/A	1.000000E+00
Gm7236	19166		#N/A	1.000000E+00
Gm7237	19167		#N/A	1.000000E+00
Gm7241	19168		#N/A	1.000000E+00
Gm7244	19169		#N/A	1.000000E+00
Gm7247	19170	0.191594759	17897	6.571754E-03
Gm7251	19171		#N/A	1.000000E+00
Gm7257	19172	-0.066856562	1139	3.468991E-01
Gm7258	19173		#N/A	1.000000E+00
Gm7271	19174		#N/A	1.000000E+00
Gm7275	19175		#N/A	1.000000E+00
Gm7276	19176		#N/A	1.000000E+00
Gm7278	19177		#N/A	1.000000E+00

Spearman Rank correlation analysis performed between Prdm1 and all-expressed genes within the Meredith RNA-seq dataset. Robust Prdm1–associated genes were identified using a cut-off of $p < 0.0005$.

Table S1, Related to Supplemental Figure 3C. Prdm1 associated genes

Gm7281	19178	0.160987082	16412.5	2.276801E-02
Gm7284	19179		#N/A	1.000000E+00
Gm7289	19180	0.053267784	8685	4.537786E-01
Gm7290	19181	-0.015750907	4484	8.248051E-01
Gm7292	19182		#N/A	1.000000E+00
Gm7293	19183		#N/A	1.000000E+00
Gm7308	19184	-0.038406171	3065	5.892384E-01
Gm7309	19185		#N/A	1.000000E+00
Gm7312	19186	0.099271295	12017.5	1.619434E-01
Gm7316	19187		#N/A	1.000000E+00
Gm732	19188		#N/A	1.000000E+00
Gm7324	19189		#N/A	1.000000E+00
Gm7325	19190		#N/A	1.000000E+00
Gm7327	19191		#N/A	1.000000E+00
Gm7331	19192	-0.029078739	3963	6.827273E-01
Gm7332	19193		#N/A	1.000000E+00
Gm7334	19194	0.07494696	10242	2.915394E-01
Gm7335	19195		#N/A	1.000000E+00
Gm7336	19196		#N/A	1.000000E+00
Gm7337	19197	0.143876722	15304	4.209722E-02
Gm7340	19198		#N/A	1.000000E+00
Gm7346	19199		#N/A	1.000000E+00
Gm7347	19200		#N/A	1.000000E+00
Gm7350	19201		#N/A	1.000000E+00
Gm7352	19202		#N/A	1.000000E+00
GM7353	19203		#N/A	1.000000E+00
Gm7353	19204	0.174173615	17132	1.363909E-02
Gm7361	19205		#N/A	1.000000E+00
Gm7363	19206	0.151984883	15767	3.168080E-02
Gm7365	19207		#N/A	1.000000E+00
Gm7367	19208		#N/A	1.000000E+00
Gm7375	19209		#N/A	1.000000E+00
Gm7381	19210		#N/A	1.000000E+00
Gm7386	19211		#N/A	1.000000E+00
Gm7391	19212		#N/A	1.000000E+00
Gm7393	19213	0.108608001	12596	1.258056E-01
Gm7398	19214		#N/A	1.000000E+00
Gm7399	19215		#N/A	1.000000E+00
Gm7405	19216		#N/A	1.000000E+00
Gm7408	19217		#N/A	1.000000E+00
Gm7415	19218		#N/A	1.000000E+00
Gm7416	19219	-0.054451513	1703	4.437940E-01
Gm7418	19220	-0.066858259	1029.5	3.468868E-01

Spearman Rank correlation analysis performed between Prdm1 and all-expressed genes within the Meredith RNA-seq dataset. Robust Prdm1–associated genes were identified using a cut-off of $p < 0.0005$.

Table S1, Related to Supplemental Figure 3C. Prdm1 associated genes

Gm7420	19221		#N/A	1.000000E+00
Gm7421	19222		#N/A	1.000000E+00
Gm7426	19223	-0.066855996	1239.5	3.469032E-01
Gm7429	19224	-0.038406171	3065	5.892384E-01
Gm7437	19225	-0.038406171	3065	5.892384E-01
Gm7438	19226		#N/A	1.000000E+00
Gm7452	19227		#N/A	1.000000E+00
Gm7457	19228	0.199325797	18218	4.659538E-03
Gm7461	19229		#N/A	1.000000E+00
Gm7485	19230	-0.038406171	3065	5.892384E-01
Gm7488	19231		#N/A	1.000000E+00
Gm7489	19232		#N/A	1.000000E+00
Gm7493	19233		#N/A	1.000000E+00
Gm7494	19234		#N/A	1.000000E+00
Gm7502	19235		#N/A	1.000000E+00
Gm7518	19236		#N/A	1.000000E+00
Gm7534	19237	-0.095269893	339	1.796190E-01
Gm7535	19238		#N/A	1.000000E+00
Gm7536	19239	-0.054450825	1984	4.437997E-01
Gm7541	19240		#N/A	1.000000E+00
Gm7546	19241	0.090858474	11420.5	2.007115E-01
Gm7551	19242		#N/A	1.000000E+00
Gm7568	19243	-0.054451513	1703	4.437940E-01
Gm757	19244		#N/A	1.000000E+00
Gm7571	19245		#N/A	1.000000E+00
Gm7579	19246		#N/A	1.000000E+00
Gm7580	19247		#N/A	1.000000E+00
Gm7582	19248		#N/A	1.000000E+00
Gm7584	19249		#N/A	1.000000E+00
Gm7589	19250	0.115284896	13343	1.040390E-01
Gm7590	19251		#N/A	1.000000E+00
Gm7592	19252	0.102857666	12237	1.472381E-01
Gm7598	19253		#N/A	1.000000E+00
Gm7599	19254	0.143605682	15224	4.249002E-02
Gm7609	19255	0.098701214	11972	1.643788E-01
Gm7613	19256		#N/A	1.000000E+00
Gm7616	19257	-0.038406171	3065	5.892384E-01
Gm7618	19258	-0.025769148	4074	7.171976E-01
Gm7628	19259		#N/A	1.000000E+00
Gm7638	19260		#N/A	1.000000E+00
Gm7645	19261		#N/A	1.000000E+00
Gm7647	19262		#N/A	1.000000E+00
Gm7648	19263		#N/A	1.000000E+00

Spearman Rank correlation analysis performed between Prdm1 and all-expressed genes within the Meredith RNA-seq dataset. Robust Prdm1–associated genes were identified using a cut-off of $p < 0.0005$.

Table S1, Related to Supplemental Figure 3C. Prdm1 associated genes

Gm765	19264	0.082225743	10761	2.470600E-01
Gm7651	19265		#N/A	1.000000E+00
Gm7657	19266		#N/A	1.000000E+00
Gm7658	19267	0.1544596	15936.5	2.897553E-02
Gm766	19268		#N/A	1.000000E+00
Gm7664	19269		#N/A	1.000000E+00
Gm7665	19270		#N/A	1.000000E+00
Gm7666	19271		#N/A	1.000000E+00
Gm7670	19272	0.032964975	7235	6.430784E-01
Gm7676	19273	-0.038406171	3065	5.892384E-01
GM7682	19274		#N/A	1.000000E+00
Gm7682	19275		#N/A	1.000000E+00
Gm7694	19276	-0.00042541	5262	9.952299E-01
Gm7696	19277		#N/A	1.000000E+00
Gm7700	19278		#N/A	1.000000E+00
Gm7701	19279		#N/A	1.000000E+00
Gm7707	19280	-0.038406171	3065	5.892384E-01
Gm7713	19281		#N/A	1.000000E+00
Gm7714	19282	-0.038406171	3065	5.892384E-01
Gm7722	19283	0.098633919	11965	1.646681E-01
Gm7729	19284		#N/A	1.000000E+00
Gm773	19285		#N/A	1.000000E+00
Gm7731	19286		#N/A	1.000000E+00
Gm7732	19287		#N/A	1.000000E+00
Gm7735	19288		#N/A	1.000000E+00
Gm7736	19289		#N/A	1.000000E+00
Gm7742	19290		#N/A	1.000000E+00
Gm7746	19291		#N/A	1.000000E+00
Gm7781	19292	-0.038406171	3065	5.892384E-01
Gm7785	19293		#N/A	1.000000E+00
Gm7790	19294		#N/A	1.000000E+00
Gm7792	19295		#N/A	1.000000E+00
Gm7799	19296	-0.018447724	4312.5	7.954215E-01
Gm7803	19297		#N/A	1.000000E+00
Gm7805	19298		#N/A	1.000000E+00
Gm7808	19299		#N/A	1.000000E+00
Gm7809	19300	0.053267784	8685	4.537786E-01
Gm7816	19301	-0.038406171	3065	5.892384E-01
GM7819	19302		#N/A	1.000000E+00
Gm7820	19303	0.020611413	6377.5	7.720510E-01
Gm7821	19304		#N/A	1.000000E+00
Gm7823	19305		#N/A	1.000000E+00
Gm7831	19306	-0.066858259	1029.5	3.468868E-01

Spearman Rank correlation analysis performed between Prdm1 and all-expressed genes within the Meredith RNA-seq dataset. Robust Prdm1–associated genes were identified using a cut-off of $p < 0.0005$.

Table S1, Related to Supplemental Figure 3C. Prdm1 associated genes

Gm7834	19307		#N/A	1.000000E+00
Gm7840	19308		#N/A	1.000000E+00
Gm7841	19309		#N/A	1.000000E+00
Gm7846	19310		#N/A	1.000000E+00
Gm7847	19311		#N/A	1.000000E+00
Gm7849	19312		#N/A	1.000000E+00
Gm7851	19313		#N/A	1.000000E+00
Gm7853	19314		#N/A	1.000000E+00
Gm7854	19315	0.143605682	15224	4.249002E-02
Gm7855	19316		#N/A	1.000000E+00
Gm7856	19317		#N/A	1.000000E+00
Gm7857	19318	0.053267784	8685	4.537786E-01
Gm7860	19319	0.123668478	13883.5	8.104102E-02
Gm7861	19320		#N/A	1.000000E+00
Gm7862	19321		#N/A	1.000000E+00
Gm7866	19322	-0.038406171	3065	5.892384E-01
Gm7869	19323		#N/A	1.000000E+00
Gm7873	19324		#N/A	1.000000E+00
Gm7876	19325		#N/A	1.000000E+00
Gm7879	19326		#N/A	1.000000E+00
Gm7882	19327		#N/A	1.000000E+00
Gm7887	19328		#N/A	1.000000E+00
Gm7901	19329	-0.054451513	1703	4.437940E-01
Gm7903	19330		#N/A	1.000000E+00
Gm7905	19331		#N/A	1.000000E+00
Gm7910	19332		#N/A	1.000000E+00
Gm7916	19333	0.18218536	17494	9.823364E-03
Gm7925	19334		#N/A	1.000000E+00
Gm7927	19335	0.053267784	8685	4.537786E-01
Gm7929	19336		#N/A	1.000000E+00
Gm7931	19337		#N/A	1.000000E+00
Gm7932	19338		#N/A	1.000000E+00
Gm7935	19339	-0.038406171	3065	5.892384E-01
Gm7936	19340		#N/A	1.000000E+00
Gm7942	19341		#N/A	1.000000E+00
Gm7945	19342		#N/A	1.000000E+00
Gm7950	19343		#N/A	1.000000E+00
Gm7951	19344		#N/A	1.000000E+00
Gm7953	19345		#N/A	1.000000E+00
Gm7954	19346		#N/A	1.000000E+00
Gm7958	19347		#N/A	1.000000E+00
Gm7964	19348		#N/A	1.000000E+00
Gm7970	19349		#N/A	1.000000E+00

Spearman Rank correlation analysis performed between Prdm1 and all-expressed genes within the Meredith RNA-seq dataset. Robust Prdm1–associated genes were identified using a cut-off of $p < 0.0005$.

Table S1, Related to Supplemental Figure 3C. Prdm1 associated genes

Gm7973	19350		#N/A	1.000000E+00
Gm7974	19351		#N/A	1.000000E+00
Gm7978	19352		#N/A	1.000000E+00
Gm7980	19353		#N/A	1.000000E+00
Gm7982	19354		#N/A	1.000000E+00
Gm7984	19355		#N/A	1.000000E+00
Gm7991	19356		#N/A	1.000000E+00
Gm7995	19357		#N/A	1.000000E+00
Gm8005	19358		#N/A	1.000000E+00
Gm8011	19359		#N/A	1.000000E+00
Gm8018	19360	-0.066856562	1139	3.468991E-01
Gm8019	19361	-0.054451513	1703	4.437940E-01
Gm8020	19362		#N/A	1.000000E+00
Gm8024	19363		#N/A	1.000000E+00
Gm8032	19364		#N/A	1.000000E+00
Gm8034	19365		#N/A	1.000000E+00
Gm8036	19366		#N/A	1.000000E+00
Gm8038	19367		#N/A	1.000000E+00
Gm8040	19368		#N/A	1.000000E+00
Gm8046	19369		#N/A	1.000000E+00
Gm8050	19370		#N/A	1.000000E+00
Gm8051	19371	-0.038406171	3065	5.892384E-01
Gm8053	19372		#N/A	1.000000E+00
Gm8054	19373	0.050870414	8368.5	4.743803E-01
Gm8055	19374		#N/A	1.000000E+00
Gm8057	19375		#N/A	1.000000E+00
Gm806	19376		#N/A	1.000000E+00
Gm8061	19377		#N/A	1.000000E+00
Gm8062	19378		#N/A	1.000000E+00
Gm8064	19379		#N/A	1.000000E+00
Gm8065	19380		#N/A	1.000000E+00
Gm8068	19381		#N/A	1.000000E+00
Gm8072	19382		#N/A	1.000000E+00
Gm8074	19383		#N/A	1.000000E+00
Gm8078	19384	-0.038406171	3065	5.892384E-01
Gm8080	19385		#N/A	1.000000E+00
Gm8082	19386		#N/A	1.000000E+00
Gm8084	19387		#N/A	1.000000E+00
Gm8087	19388		#N/A	1.000000E+00
Gm8089	19389	-0.054451513	1703	4.437940E-01
Gm8091	19390	0.075338888	10291	2.890163E-01
Gm8093	19391	0.022198316	6629.5	7.550388E-01
Gm8094	19392		#N/A	1.000000E+00

Spearman Rank correlation analysis performed between Prdm1 and all-expressed genes within the Meredith RNA-seq dataset. Robust Prdm1–associated genes were identified using a cut-off of $p < 0.0005$.

Table S1, Related to Supplemental Figure 3C. Prdm1 associated genes

Gm8097	19393	0.053267784	8685	4.537786E-01
Gm8098	19394		#N/A	1.000000E+00
Gm8104	19395		#N/A	1.000000E+00
Gm8107	19396	-0.054451513	1703	4.437940E-01
Gm8108	19397		#N/A	1.000000E+00
Gm8111	19398		#N/A	1.000000E+00
Gm8112	19399		#N/A	1.000000E+00
Gm8113	19400	0.02911945	7021	6.823072E-01
Gm8114	19401		#N/A	1.000000E+00
Gm8116	19402		#N/A	1.000000E+00
Gm8121	19403	0.048448014	8175.5	4.957045E-01
Gm8122	19404		#N/A	1.000000E+00
Gm8126	19405		#N/A	1.000000E+00
Gm8127	19406		#N/A	1.000000E+00
Gm8129	19407	-0.062487898	1372	3.793836E-01
Gm813	19408		#N/A	1.000000E+00
Gm8138	19409		#N/A	1.000000E+00
Gm8145	19410	-0.038406171	3065	5.892384E-01
Gm815	19411	0.021317126	6513.5	7.644715E-01
Gm8152	19412	0.165207727	16671	1.939591E-02
Gm8159	19413		#N/A	1.000000E+00
Gm8161	19414		#N/A	1.000000E+00
Gm8163	19415	-0.038406171	3065	5.892384E-01
Gm8164	19416		#N/A	1.000000E+00
Gm8165	19417		#N/A	1.000000E+00
Gm8172	19418		#N/A	1.000000E+00
Gm8174	19419		#N/A	1.000000E+00
Gm8178	19420		#N/A	1.000000E+00
Gm8180	19421		#N/A	1.000000E+00
Gm8181	19422		#N/A	1.000000E+00
Gm8185	19423		#N/A	1.000000E+00
Gm8186	19424		#N/A	1.000000E+00
Gm8194	19425		#N/A	1.000000E+00
Gm8199	19426		#N/A	1.000000E+00
Gm8200	19427	-0.086748473	486.5	2.219259E-01
Gm8202	19428		#N/A	1.000000E+00
Gm8203	19429	0.135395611	14655	5.592842E-02
Gm8206	19430		#N/A	1.000000E+00
Gm8207	19431		#N/A	1.000000E+00
Gm8210	19432		#N/A	1.000000E+00
Gm8212	19433	-0.038406171	3065	5.892384E-01
Gm8213	19434	0.13721726	14769	5.267736E-02
Gm8214	19435		#N/A	1.000000E+00

Spearman Rank correlation analysis performed between Prdm1 and all-expressed genes within the Meredith RNA-seq dataset. Robust Prdm1–associated genes were identified using a cut-off of $p < 0.0005$.

Table S1, Related to Supplemental Figure 3C. Prdm1 associated genes

Gm8215	19436		#N/A	1.000000E+00
Gm8216	19437		#N/A	1.000000E+00
Gm8217	19438	0.015835655	6114	8.238778E-01
Gm8220	19439		#N/A	1.000000E+00
Gm8221	19440	-0.038406171	3065	5.892384E-01
Gm8224	19441		#N/A	1.000000E+00
Gm8225	19442	-0.038406171	3065	5.892384E-01
Gm8226	19443		#N/A	1.000000E+00
Gm8229	19444		#N/A	1.000000E+00
Gm8232	19445	-0.038406171	3065	5.892384E-01
Gm8237	19446		#N/A	1.000000E+00
Gm8244	19447		#N/A	1.000000E+00
Gm8246	19448		#N/A	1.000000E+00
Gm8247	19449		#N/A	1.000000E+00
Gm8250	19450		#N/A	1.000000E+00
Gm8251	19451		#N/A	1.000000E+00
Gm8256	19452		#N/A	1.000000E+00
Gm8257	19453		#N/A	1.000000E+00
Gm8258	19454		#N/A	1.000000E+00
Gm826	19455	0.073928833	10125	2.981622E-01
Gm8260	19456		#N/A	1.000000E+00
Gm8261	19457		#N/A	1.000000E+00
Gm8265	19458		#N/A	1.000000E+00
Gm8266	19459		#N/A	1.000000E+00
Gm8267	19460	-0.033702148	3752	6.356657E-01
Gm8271	19461		#N/A	1.000000E+00
Gm8279	19462		#N/A	1.000000E+00
Gm8281	19463	0.052728522	8550.5	4.583686E-01
Gm8289	19464		#N/A	1.000000E+00
Gm829	19465		#N/A	1.000000E+00
Gm8292	19466	0.164226607	16605	2.013851E-02
Gm8297	19467		#N/A	1.000000E+00
Gm8298	19468	0.187485437	17744.5	7.850316E-03
Gm830	19469	-0.038406171	3065	5.892384E-01
Gm8300	19470	0.122641288	13823	8.361682E-02
Gm8302	19471	-0.038406171	3065	5.892384E-01
Gm8303	19472		#N/A	1.000000E+00
Gm8305	19473		#N/A	1.000000E+00
Gm831	19474	-0.038406171	3065	5.892384E-01
Gm8310	19475		#N/A	1.000000E+00
Gm8312	19476	-0.038406171	3065	5.892384E-01
Gm8317	19477	0.1544596	15936.5	2.897553E-02
Gm8320	19478		#N/A	1.000000E+00

Spearman Rank correlation analysis performed between Prdm1 and all-expressed genes within the Meredith RNA-seq dataset. Robust Prdm1–associated genes were identified using a cut-off of $p < 0.0005$.

Table S1, Related to Supplemental Figure 3C. Prdm1 associated genes

Gm8323	19479		#N/A	1.000000E+00
Gm8324	19480		#N/A	1.000000E+00
Gm8325	19481	-0.020085617	4236	7.777123E-01
Gm8327	19482		#N/A	1.000000E+00
Gm833	19483	0.215438595	18765	2.186012E-03
Gm8331	19484		#N/A	1.000000E+00
Gm8332	19485		#N/A	1.000000E+00
Gm8334	19486		#N/A	1.000000E+00
Gm8338	19487		#N/A	1.000000E+00
Gm8340	19488		#N/A	1.000000E+00
Gm8344	19489		#N/A	1.000000E+00
Gm8346	19490		#N/A	1.000000E+00
Gm8355	19491	0.082269134	10768	2.468098E-01
Gm8356	19492		#N/A	1.000000E+00
Gm8358	19493		#N/A	1.000000E+00
Gm8362	19494		#N/A	1.000000E+00
Gm8366	19495		#N/A	1.000000E+00
Gm8369	19496	-0.043701509	2380	5.389164E-01
Gm8374	19497		#N/A	1.000000E+00
Gm8378	19498		#N/A	1.000000E+00
Gm8379	19499		#N/A	1.000000E+00
Gm8383	19500		#N/A	1.000000E+00
Gm8385	19501		#N/A	1.000000E+00
Gm839	19502		#N/A	1.000000E+00
Gm8394	19503	0.021317126	6513.5	7.644715E-01
Gm8396	19504		#N/A	1.000000E+00
Gm8398	19505	-0.066856562	1139	3.468991E-01
Gm8399	19506		#N/A	1.000000E+00
Gm8401	19507		#N/A	1.000000E+00
Gm8402	19508		#N/A	1.000000E+00
Gm8410	19509		#N/A	1.000000E+00
Gm8412	19510	-0.038406171	3065	5.892384E-01
Gm8420	19511	-0.055896814	1492	4.317736E-01
Gm8421	19512		#N/A	1.000000E+00
Gm8424	19513		#N/A	1.000000E+00
Gm8425	19514	-0.077397959	691.5	2.760011E-01
Gm8428	19515		#N/A	1.000000E+00
Gm8429	19516	-0.066858259	1029.5	3.468868E-01
Gm8430	19517	0.068292321	9725	3.366186E-01
Gm8439	19518	-0.038406171	3065	5.892384E-01
Gm8444	19519	0.05654886	9011	4.264125E-01
Gm8453	19520		#N/A	1.000000E+00
Gm8459	19521		#N/A	1.000000E+00

Spearman Rank correlation analysis performed between Prdm1 and all-expressed genes within the Meredith RNA-seq dataset. Robust Prdm1–associated genes were identified using a cut-off of $p < 0.0005$.

Table S1, Related to Supplemental Figure 3C. Prdm1 associated genes

Gm8464	19522		#N/A	1.000000E+00
Gm8473	19523		#N/A	1.000000E+00
Gm8475	19524		#N/A	1.000000E+00
Gm8482	19525		#N/A	1.000000E+00
Gm8488	19526		#N/A	1.000000E+00
Gm8494	19527		#N/A	1.000000E+00
Gm8501	19528	-0.054450825	1984	4.437997E-01
Gm8503	19529		#N/A	1.000000E+00
Gm8508	19530		#N/A	1.000000E+00
Gm8521	19531		#N/A	1.000000E+00
Gm8522	19532	0.160304018	16323	2.335876E-02
Gm8526	19533	0.035805373	7366	6.147168E-01
Gm853	19534	-0.038406171	3065	5.892384E-01
Gm8532	19535		#N/A	1.000000E+00
Gm8534	19536	-0.000841282	5208.5	9.905669E-01
Gm8540	19537	-0.038406171	3065	5.892384E-01
Gm8545	19538		#N/A	1.000000E+00
Gm8546	19539		#N/A	1.000000E+00
Gm8550	19540		#N/A	1.000000E+00
Gm8566	19541	0.1544596	15936.5	2.897553E-02
Gm8569	19542		#N/A	1.000000E+00
Gm8577	19543		#N/A	1.000000E+00
Gm8580	19544	0.075841759	10333	2.858004E-01
Gm8582	19545		#N/A	1.000000E+00
Gm8584	19546		#N/A	1.000000E+00
Gm8587	19547		#N/A	1.000000E+00
Gm8595	19548	0.074574898	10201.5	2.939481E-01
Gm8597	19549		#N/A	1.000000E+00
Gm8603	19550		#N/A	1.000000E+00
Gm8606	19551		#N/A	1.000000E+00
Gm8615	19552		#N/A	1.000000E+00
Gm8617	19553		#N/A	1.000000E+00
Gm8618	19554	0.135796901	14680.5	5.519836E-02
Gm8619	19555		#N/A	1.000000E+00
Gm8623	19556		#N/A	1.000000E+00
Gm8624	19557	0.097996299	11914	1.674280E-01
Gm8644	19558		#N/A	1.000000E+00
Gm8648	19559	0.101112751	12124	1.542610E-01
Gm8653	19560		#N/A	1.000000E+00
Gm8659	19561		#N/A	1.000000E+00
Gm8660	19562		#N/A	1.000000E+00
Gm8661	19563		#N/A	1.000000E+00
Gm8662	19564	0.163104102	16550	2.101821E-02

Spearman Rank correlation analysis performed between Prdm1 and all-expressed genes within the Meredith RNA-seq dataset. Robust Prdm1–associated genes were identified using a cut-off of $p < 0.0005$.

Table S1, Related to Supplemental Figure 3C. Prdm1 associated genes

Gm8666	19565		#N/A	1.000000E+00
Gm8668	19566		#N/A	1.000000E+00
Gm867	19567		#N/A	1.000000E+00
Gm8674	19568		#N/A	1.000000E+00
Gm8677	19569		#N/A	1.000000E+00
Gm8682	19570		#N/A	1.000000E+00
Gm8688	19571		#N/A	1.000000E+00
Gm8692	19572		#N/A	1.000000E+00
Gm8693	19573		#N/A	1.000000E+00
Gm8696	19574		#N/A	1.000000E+00
Gm8701	19575		#N/A	1.000000E+00
Gm8711	19576		#N/A	1.000000E+00
Gm8715	19577		#N/A	1.000000E+00
Gm872	19578	-0.053449131	2133	4.522408E-01
Gm8720	19579		#N/A	1.000000E+00
Gm8722	19580		#N/A	1.000000E+00
Gm8723	19581		#N/A	1.000000E+00
Gm8724	19582	0.064123369	9476.5	3.670112E-01
Gm8730	19583	0.053267784	8685	4.537786E-01
Gm8731	19584	0.020875719	6412.5	7.692098E-01
Gm8737	19585		#N/A	1.000000E+00
Gm8738	19586		#N/A	1.000000E+00
Gm8741	19587		#N/A	1.000000E+00
Gm8749	19588	-0.038406171	3065	5.892384E-01
Gm8750	19589		#N/A	1.000000E+00
Gm8752	19590		#N/A	1.000000E+00
Gm8754	19591		#N/A	1.000000E+00
Gm8757	19592		#N/A	1.000000E+00
Gm8761	19593		#N/A	1.000000E+00
Gm8765	19594		#N/A	1.000000E+00
Gm8770	19595		#N/A	1.000000E+00
Gm8773	19596	-0.004525914	4967	9.492846E-01
Gm8775	19597		#N/A	1.000000E+00
Gm8778	19598		#N/A	1.000000E+00
Gm8783	19599		#N/A	1.000000E+00
Gm8787	19600	-0.117577649	117	9.729063E-02
Gm8801	19601	-0.054451513	1703	4.437940E-01
Gm8802	19602		#N/A	1.000000E+00
Gm8805	19603	0.122200231	13797	8.474282E-02
Gm8806	19604	-0.038406171	3065	5.892384E-01
Gm8807	19605	0.160304018	16323	2.335876E-02
Gm8809	19606		#N/A	1.000000E+00
Gm8810	19607		#N/A	1.000000E+00

Spearman Rank correlation analysis performed between Prdm1 and all-expressed genes within the Meredith RNA-seq dataset. Robust Prdm1–associated genes were identified using a cut-off of $p < 0.0005$.

Table S1, Related to Supplemental Figure 3C. Prdm1 associated genes

Gm8812	19608		#N/A	1.000000E+00
Gm8813	19609		#N/A	1.000000E+00
Gm8814	19610	-0.038406171	3065	5.892384E-01
Gm8815	19611		#N/A	1.000000E+00
Gm8817	19612	-0.054451513	1703	4.437940E-01
Gm8818	19613		#N/A	1.000000E+00
Gm8822	19614	0.188232164	17772	7.602721E-03
Gm8832	19615	0.113548679	13094	1.093908E-01
Gm8835	19616		#N/A	1.000000E+00
Gm8837	19617	-0.038406171	3065	5.892384E-01
Gm8839	19618		#N/A	1.000000E+00
Gm884	19619	0.130020918	14300	6.649897E-02
Gm8840	19620	0.070535706	9874.5	3.209489E-01
Gm8844	19621		#N/A	1.000000E+00
Gm8849	19622		#N/A	1.000000E+00
Gm8855	19623		#N/A	1.000000E+00
Gm8857	19624		#N/A	1.000000E+00
Gm8858	19625	0.172390605	17046	1.464693E-02
Gm8864	19626	0.114414374	13283	1.066960E-01
Gm8865	19627		#N/A	1.000000E+00
Gm8868	19628		#N/A	1.000000E+00
Gm8871	19629	-0.038406171	3065	5.892384E-01
Gm8876	19630		#N/A	1.000000E+00
Gm8877	19631		#N/A	1.000000E+00
Gm8878	19632		#N/A	1.000000E+00
Gm8879	19633		#N/A	1.000000E+00
Gm8880	19634	0.050883637	8377	4.742652E-01
Gm8882	19635		#N/A	1.000000E+00
Gm8888	19636		#N/A	1.000000E+00
Gm8890	19637		#N/A	1.000000E+00
Gm8893	19638		#N/A	1.000000E+00
Gm8894	19639		#N/A	1.000000E+00
Gm8895	19640		#N/A	1.000000E+00
Gm8897	19641		#N/A	1.000000E+00
Gm8898	19642		#N/A	1.000000E+00
Gm8902	19643		#N/A	1.000000E+00
Gm8906	19644		#N/A	1.000000E+00
Gm8908	19645		#N/A	1.000000E+00
Gm8909	19646	0.205742887	18446	3.469662E-03
Gm8910	19647		#N/A	1.000000E+00
Gm8914	19648		#N/A	1.000000E+00
Gm8916	19649		#N/A	1.000000E+00
Gm8922	19650		#N/A	1.000000E+00

Spearman Rank correlation analysis performed between Prdm1 and all-expressed genes within the Meredith RNA-seq dataset. Robust Prdm1–associated genes were identified using a cut-off of $p < 0.0005$.

Table S1, Related to Supplemental Figure 3C. Prdm1 associated genes

Gm8923	19651	-0.054450825	1984	4.437997E-01
Gm8926	19652		#N/A	1.000000E+00
Gm8935	19653	-0.013540297	4588.5	8.490763E-01
Gm8946	19654		#N/A	1.000000E+00
Gm8973	19655	0.045056653	7941.5	5.263908E-01
Gm8974	19656		#N/A	1.000000E+00
Gm8975	19657	0.02122684	6452	7.654400E-01
Gm8978	19658	-0.038406171	3065	5.892384E-01
Gm8979	19659		#N/A	1.000000E+00
Gm8980	19660		#N/A	1.000000E+00
Gm8988	19661		#N/A	1.000000E+00
Gm8989	19662		#N/A	1.000000E+00
Gm8991	19663		#N/A	1.000000E+00
Gm8994	19664	0.113548679	13094	1.093908E-01
Gm8995	19665	0.270858703	19723	1.047675E-04
Gm9	19666		#N/A	1.000000E+00
Gm9001	19667		#N/A	1.000000E+00
Gm9002	19668	0.049855249	8285.5	4.832553E-01
Gm9003	19669		#N/A	1.000000E+00
Gm9005	19670		#N/A	1.000000E+00
Gm9006	19671		#N/A	1.000000E+00
Gm9008	19672		#N/A	1.000000E+00
Gm9009	19673		#N/A	1.000000E+00
Gm9013	19674		#N/A	1.000000E+00
Gm9017	19675	-0.054450825	1984	4.437997E-01
Gm9025	19676	0.021317126	6513.5	7.644715E-01
Gm9027	19677		#N/A	1.000000E+00
Gm9034	19678		#N/A	1.000000E+00
Gm904	19679		#N/A	1.000000E+00
Gm9040	19680		#N/A	1.000000E+00
Gm9041	19681	-0.038406171	3065	5.892384E-01
Gm9043	19682		#N/A	1.000000E+00
Gm9044	19683		#N/A	1.000000E+00
Gm9045	19684		#N/A	1.000000E+00
Gm9046	19685		#N/A	1.000000E+00
Gm9047	19686		#N/A	1.000000E+00
Gm9048	19687		#N/A	1.000000E+00
Gm9049	19688		#N/A	1.000000E+00
Gm9050	19689		#N/A	1.000000E+00
Gm9051	19690		#N/A	1.000000E+00
Gm9059	19691	-0.057516983	1444	4.185241E-01
Gm906	19692		#N/A	1.000000E+00
Gm9060	19693		#N/A	1.000000E+00

Spearman Rank correlation analysis performed between Prdm1 and all-expressed genes within the Meredith RNA-seq dataset. Robust Prdm1–associated genes were identified using a cut-off of $p < 0.0005$.

Table S1, Related to Supplemental Figure 3C. Prdm1 associated genes

Gm9062	19694		#N/A	1.000000E+00
Gm9064	19695		#N/A	1.000000E+00
Gm9071	19696		#N/A	1.000000E+00
Gm9075	19697		#N/A	1.000000E+00
Gm9078	19698		#N/A	1.000000E+00
Gm9083	19699		#N/A	1.000000E+00
Gm9085	19700		#N/A	1.000000E+00
Gm9089	19701		#N/A	1.000000E+00
Gm9095	19702		#N/A	1.000000E+00
Gm9097	19703		#N/A	1.000000E+00
Gm9103	19704		#N/A	1.000000E+00
Gm9104	19705	-0.054451513	1703	4.437940E-01
Gm9105	19706		#N/A	1.000000E+00
Gm9109	19707		#N/A	1.000000E+00
Gm9112	19708	0.022198316	6629.5	7.550388E-01
Gm9115	19709		#N/A	1.000000E+00
Gm9116	19710		#N/A	1.000000E+00
Gm9117	19711		#N/A	1.000000E+00
Gm9119	19712		#N/A	1.000000E+00
Gm9125	19713	0.076478035	10378.5	2.817660E-01
Gm9126	19714		#N/A	1.000000E+00
Gm9133	19715		#N/A	1.000000E+00
Gm9141	19716		#N/A	1.000000E+00
Gm9143	19717		#N/A	1.000000E+00
Gm9144	19718		#N/A	1.000000E+00
Gm9157	19719		#N/A	1.000000E+00
Gm9159	19720	0.187781716	17753	7.751233E-03
Gm9164	19721		#N/A	1.000000E+00
Gm9166	19722	0.230235201	19161.5	1.038871E-03
Gm9167	19723		#N/A	1.000000E+00
Gm9174	19724		#N/A	1.000000E+00
Gm9183	19725	0.113548679	13094	1.093908E-01
Gm9200	19726	0.160304018	16323	2.335876E-02
Gm9208	19727		#N/A	1.000000E+00
Gm9218	19728		#N/A	1.000000E+00
Gm9225	19729		#N/A	1.000000E+00
Gm9234	19730		#N/A	1.000000E+00
Gm9242	19731		#N/A	1.000000E+00
Gm9244	19732		#N/A	1.000000E+00
Gm9246	19733		#N/A	1.000000E+00
Gm9252	19734	-0.038406171	3065	5.892384E-01
Gm9257	19735		#N/A	1.000000E+00
Gm9266	19736	0.164478601	16623.5	1.994547E-02

Spearman Rank correlation analysis performed between Prdm1 and all-expressed genes within the Meredith RNA-seq dataset. Robust Prdm1–associated genes were identified using a cut-off of $p < 0.0005$.

Table S1, Related to Supplemental Figure 3C. Prdm1 associated genes

Gm9268	19737		#N/A	1.000000E+00
Gm9285	19738	-0.054451513	1703	4.437940E-01
Gm9286	19739	-0.077397959	691.5	2.760011E-01
Gm9294	19740	0.285444231	19837	4.184408E-05
Gm9308	19741		#N/A	1.000000E+00
Gm9311	19742		#N/A	1.000000E+00
Gm9312	19743		#N/A	1.000000E+00
Gm9320	19744	0.061835554	9325	3.843888E-01
Gm933	19745		#N/A	1.000000E+00
Gm9359	19746		#N/A	1.000000E+00
Gm9364	19747		#N/A	1.000000E+00
Gm9372	19748	0.090581236	11391	2.020946E-01
Gm9376	19749	-0.054450825	1984	4.437997E-01
Gm9378	19750	-0.054450825	1984	4.437997E-01
Gm9385	19751	0.113548679	13094	1.093908E-01
Gm9386	19752		#N/A	1.000000E+00
Gm9387	19753		#N/A	1.000000E+00
Gm9392	19754		#N/A	1.000000E+00
Gm9396	19755		#N/A	1.000000E+00
Gm9399	19756		#N/A	1.000000E+00
Gm94	19757	0.051838141	8457	4.660034E-01
Gm9427	19758		#N/A	1.000000E+00
Gm9428	19759		#N/A	1.000000E+00
Gm9429	19760		#N/A	1.000000E+00
Gm9430	19761	0.11037053	12744.5	1.197441E-01
Gm9431	19762		#N/A	1.000000E+00
Gm9432	19763		#N/A	1.000000E+00
Gm9434	19764		#N/A	1.000000E+00
Gm9435	19765		#N/A	1.000000E+00
Gm9436	19766	0.024955854	6814	7.257598E-01
Gm9439	19767		#N/A	1.000000E+00
Gm9440	19768		#N/A	1.000000E+00
Gm9457	19769	0.197901953	18163	4.968787E-03
Gm9458	19770		#N/A	1.000000E+00
Gm9476	19771		#N/A	1.000000E+00
Gm9493	19772	-0.054451513	1703	4.437940E-01
Gm9498	19773		#N/A	1.000000E+00
Gm9506	19774		#N/A	1.000000E+00
Gm9507	19775		#N/A	1.000000E+00
Gm9513	19776	-0.066855996	1239.5	3.469032E-01
Gm9516	19777	-0.066858259	1029.5	3.468868E-01
Gm9529	19778		#N/A	1.000000E+00
Gm9531	19779		#N/A	1.000000E+00

Spearman Rank correlation analysis performed between Prdm1 and all-expressed genes within the Meredith RNA-seq dataset. Robust Prdm1–associated genes were identified using a cut-off of $p < 0.0005$.

Table S1, Related to Supplemental Figure 3C. Prdm1 associated genes

Gm9550	19780		#N/A	1.000000E+00
Gm9573	19781	0.022198316	6629.5	7.550388E-01
Gm9574	19782		#N/A	1.000000E+00
Gm9575	19783	0.143605682	15224	4.249002E-02
Gm9577	19784		#N/A	1.000000E+00
Gm9581	19785	-0.038406171	3065	5.892384E-01
Gm9587	19786		#N/A	1.000000E+00
Gm9593	19787		#N/A	1.000000E+00
Gm9597	19788		#N/A	1.000000E+00
Gm9599	19789	-0.038406171	3065	5.892384E-01
Gm960	19790	0.021317126	6513.5	7.644715E-01
Gm9602	19791		#N/A	1.000000E+00
Gm9603	19792		#N/A	1.000000E+00
Gm9611	19793		#N/A	1.000000E+00
Gm9612	19794		#N/A	1.000000E+00
Gm9613	19795		#N/A	1.000000E+00
Gm9630	19796		#N/A	1.000000E+00
Gm9631	19797	0.152789767	15822	3.077826E-02
Gm9637	19798		#N/A	1.000000E+00
Gm9670	19799	0.198121949	18171	4.919833E-03
Gm9671	19800		#N/A	1.000000E+00
Gm9672	19801		#N/A	1.000000E+00
Gm9673	19802		#N/A	1.000000E+00
Gm9696	19803	0.186034874	17683	8.351855E-03
Gm9697	19804		#N/A	1.000000E+00
Gm97	19805	0.075715395	10317.5	2.866063E-01
Gm9703	19806	-0.054451513	1703	4.437940E-01
Gm9705	19807	0.143605682	15224	4.249002E-02
Gm9719	19808		#N/A	1.000000E+00
Gm9725	19809	0.020875719	6412.5	7.692098E-01
Gm9726	19810	0.109930598	12709.5	1.212354E-01
Gm9727	19811	-0.054451513	1703	4.437940E-01
Gm973	19812	-0.05397389	2116	4.478076E-01
Gm9732	19813	-0.038406171	3065	5.892384E-01
Gm9733	19814	-0.038406171	3065	5.892384E-01
Gm9742	19815		#N/A	1.000000E+00
Gm9745	19816		#N/A	1.000000E+00
Gm9747	19817		#N/A	1.000000E+00
Gm9750	19818	0.05327231	8763	4.537402E-01
Gm9752	19819		#N/A	1.000000E+00
Gm9754	19820	-0.038406171	3065	5.892384E-01
Gm9755	19821	0.079220671	10546	2.648159E-01
Gm9758	19822	-0.054450825	1984	4.437997E-01

Spearman Rank correlation analysis performed between Prdm1 and all-expressed genes within the Meredith RNA-seq dataset. Robust Prdm1–associated genes were identified using a cut-off of $p < 0.0005$.

Table S1, Related to Supplemental Figure 3C. Prdm1 associated genes

Gm9761	19823		#N/A	1.000000E+00
Gm9762	19824		#N/A	1.000000E+00
Gm9763	19825		#N/A	1.000000E+00
Gm9765	19826	0.02421538	6767	7.335850E-01
Gm9767	19827		#N/A	1.000000E+00
Gm9769	19828	-0.006583341	4862	9.262850E-01
Gm9770	19829		#N/A	1.000000E+00
Gm9772	19830		#N/A	1.000000E+00
Gm9774	19831	0.188062479	17764	7.658366E-03
Gm9776	19832	0.094514885	11673	1.831080E-01
Gm9780	19833	0.143605682	15224	4.249002E-02
Gm9783	19834	-0.038406171	3065	5.892384E-01
Gm9785	19835		#N/A	1.000000E+00
Gm9788	19836		#N/A	1.000000E+00
Gm9789	19837		#N/A	1.000000E+00
Gm9790	19838	-0.044861116	2341	5.281889E-01
Gm9791	19839	0.038758411	7558.5	5.858252E-01
Gm9795	19840		#N/A	1.000000E+00
Gm9796	19841		#N/A	1.000000E+00
Gm9797	19842	0.106652326	12469	1.328060E-01
Gm9799	19843	0.223150728	18991	1.492208E-03
Gm9800	19844	-0.038406171	3065	5.892384E-01
Gm9801	19845	0.053267784	8685	4.537786E-01
Gm9803	19846	0.151115048	15716	3.268140E-02
Gm9804	19847		#N/A	1.000000E+00
Gm9805	19848		#N/A	1.000000E+00
Gm9806	19849		#N/A	1.000000E+00
Gm9812	19850		#N/A	1.000000E+00
Gm9814	19851		#N/A	1.000000E+00
Gm9816	19852	0.02824322	6983	6.913711E-01
Gm9817	19853		#N/A	1.000000E+00
Gm9818	19854		#N/A	1.000000E+00
Gm9821	19855		#N/A	1.000000E+00
Gm9823	19856		#N/A	1.000000E+00
Gm9824	19857		#N/A	1.000000E+00
Gm9825	19858	0.125262852	13987	7.716994E-02
Gm9826	19859		#N/A	1.000000E+00
Gm9828	19860	-0.016731569	4428.5	8.140896E-01
Gm9830	19861		#N/A	1.000000E+00
Gm9833	19862		#N/A	1.000000E+00
Gm9835	19863		#N/A	1.000000E+00
Gm9836	19864		#N/A	1.000000E+00
Gm9837	19865		#N/A	1.000000E+00

Spearman Rank correlation analysis performed between Prdm1 and all-expressed genes within the Meredith RNA-seq dataset. Robust Prdm1–associated genes were identified using a cut-off of $p < 0.0005$.

Table S1, Related to Supplemental Figure 3C. Prdm1 associated genes

Gm9839	19866		#N/A	1.000000E+00
Gm9840	19867	0.128369979	14203	7.005593E-02
Gm9843	19868	0.254159861	19552	2.816124E-04
Gm9844	19869	0.164478601	16623.5	1.994547E-02
Gm9845	19870		#N/A	1.000000E+00
Gm9846	19871	0.193423952	17967	6.065003E-03
Gm9847	19872		#N/A	1.000000E+00
Gm9848	19873		#N/A	1.000000E+00
Gm9850	19874	0.053805701	8820	4.492258E-01
Gm9855	19875	-0.018970142	4272	7.897612E-01
Gm9857	19876		#N/A	1.000000E+00
Gm9864	19877		#N/A	1.000000E+00
Gm9866	19878	0.115914734	13389	1.021495E-01
Gm9867	19879		#N/A	1.000000E+00
Gm9869	19880		#N/A	1.000000E+00
Gm9870	19881		#N/A	1.000000E+00
Gm9871	19882		#N/A	1.000000E+00
Gm9873	19883		#N/A	1.000000E+00
Gm9874	19884	-0.054451513	1703	4.437940E-01
Gm9875	19885	0.074574898	10201.5	2.939481E-01
Gm9877	19886	0.215109181	18744.5	2.221323E-03
Gm9881	19887		#N/A	1.000000E+00
Gm9884	19888		#N/A	1.000000E+00
Gm9885	19889		#N/A	1.000000E+00
Gm9887	19890		#N/A	1.000000E+00
Gm9888	19891		#N/A	1.000000E+00
Gm9889	19892	-0.038406171	3065	5.892384E-01
Gm9890	19893		#N/A	1.000000E+00
Gm9892	19894	0.01947445	6301	7.843075E-01
Gm9894	19895		#N/A	1.000000E+00
Gm9897	19896	-0.086748473	486.5	2.219259E-01
Gm9898	19897	-0.077394997	736.5	2.760195E-01
Gm9899	19898		#N/A	1.000000E+00
Gm9900	19899	0.169024144	16871	1.672855E-02
Gm9901	19900		#N/A	1.000000E+00
Gm9905	19901	-0.038406171	3065	5.892384E-01
Gm9912	19902		#N/A	1.000000E+00
Gm9913	19903		#N/A	1.000000E+00
Gm9915	19904		#N/A	1.000000E+00
Gm9920	19905	0.003819599	5509	9.571910E-01
Gm9921	19906		#N/A	1.000000E+00
Gm9922	19907		#N/A	1.000000E+00
Gm9923	19908		#N/A	1.000000E+00

Spearman Rank correlation analysis performed between Prdm1 and all-expressed genes within the Meredith RNA-seq dataset. Robust Prdm1–associated genes were identified using a cut-off of $p < 0.0005$.

Table S1, Related to Supplemental Figure 3C. Prdm1 associated genes

Gm9924	19909		#N/A	1.000000E+00
Gm9925	19910		#N/A	1.000000E+00
Gm9929	19911	0.147781682	15523	3.676817E-02
Gm9930	19912		#N/A	1.000000E+00
Gm9931	19913		#N/A	1.000000E+00
Gm9932	19914		#N/A	1.000000E+00
Gm9933	19915		#N/A	1.000000E+00
Gm9934	19916	0.053805701	8820	4.492258E-01
Gm9936	19917		#N/A	1.000000E+00
Gm9938	19918		#N/A	1.000000E+00
Gm9939	19919		#N/A	1.000000E+00
Gm9944	19920		#N/A	1.000000E+00
Gm9945	19921		#N/A	1.000000E+00
Gm9946	19922	0.053267784	8685	4.537786E-01
Gm9947	19923	-0.054450825	1984	4.437997E-01
Gm9949	19924		#N/A	1.000000E+00
Gm9951	19925	0.162808768	16526.5	2.125512E-02
Gm9954	19926		#N/A	1.000000E+00
Gm9955	19927		#N/A	1.000000E+00
Gm9956	19928		#N/A	1.000000E+00
Gm9958	19929	0.124975963	13967	7.785524E-02
Gm9959	19930		#N/A	1.000000E+00
Gm996	19931	-0.038406171	3065	5.892384E-01
Gm9961	19932		#N/A	1.000000E+00
Gm9962	19933		#N/A	1.000000E+00
Gm9964	19934		#N/A	1.000000E+00
Gm9966	19935		#N/A	1.000000E+00
Gm9967	19936		#N/A	1.000000E+00
Gm9968	19937	-0.038406171	3065	5.892384E-01
Gm9969	19938		#N/A	1.000000E+00
Gm9970	19939		#N/A	1.000000E+00
Gm9972	19940		#N/A	1.000000E+00
Gm9974	19941	0.143605682	15224	4.249002E-02
Gm9978	19942		#N/A	1.000000E+00
Gm9979	19943		#N/A	1.000000E+00
Gm9982	19944	-0.066855996	1239.5	3.469032E-01
Gm9985	19945		#N/A	1.000000E+00
Gm9987	19946		#N/A	1.000000E+00
Gm9988	19947		#N/A	1.000000E+00
Gm9989	19948		#N/A	1.000000E+00
Gm9990	19949	-0.038406171	3065	5.892384E-01
Gm9991	19950		#N/A	1.000000E+00
Gm9992	19951		#N/A	1.000000E+00

Spearman Rank correlation analysis performed between Prdm1 and all-expressed genes within the Meredith RNA-seq dataset. Robust Prdm1–associated genes were identified using a cut-off of $p < 0.0005$.

Table S1, Related to Supplemental Figure 3C. Prdm1 associated genes

Gm9994	19952		#N/A	1.000000E+00
Gm9996	19953		#N/A	1.000000E+00
Gm9999	19954	-0.054451513	1703	4.437940E-01
Gmcl1	19955	0.119767078	13648	9.117509E-02
Gmcl1l	19956		#N/A	1.000000E+00
Gmds	19957	0.197459562	18139	5.068554E-03
Gmeb1	19958	-0.046919932	2263	5.094128E-01
Gmeb2	19959	0.10359501	12276	1.443444E-01
Gmfb	19960	0.164134413	16600	2.020954E-02
Gmfg	19961	0.320221682	19967	3.790731E-06
Gmip	19962	-0.038527908	2502	5.880577E-01
Gml	19963	-0.054450825	1984	4.437997E-01
Gmnc	19964	0.055761012	8971	4.328950E-01
Gmnn	19965	0.261629559	19644	1.824145E-04
Gmppa	19966	-0.042411445	2424	5.509766E-01
Gmppb	19967	0.206924533	18490	3.283204E-03
Gmpr	19968	0.100362265	12083	1.573581E-01
Gmpr2	19969	0.079464059	10561	2.633461E-01
Gmps	19970	0.278272316	19781	6.613196E-05
Gna11	19971	0.382220452	20026	2.336331E-08
Gna12	19972	-0.037330448	3652	5.997180E-01
Gna13	19973	0.104781485	12342	1.397793E-01
Gna14	19974	0.063663265	9448	3.704664E-01
Gna15	19975	0.132151224	14420	6.212876E-02
Gnai1	19976	-0.086755146	452	2.218903E-01
Gnai2	19977	0.111204383	12803	1.169567E-01
Gnai3	19978	0.190806611	17874	6.801509E-03
Gnal	19979	-0.000341796	5273	9.961674E-01
Gnao1	19980	0.094024065	11643	1.854027E-01
Gnaq	19981	0.073527938	10101	3.007971E-01
Gnas	19982	0.202033806	18328	4.118695E-03
Gnat1	19983		#N/A	1.000000E+00
Gnat2	19984		#N/A	1.000000E+00
Gnat3	19985	0.059533327	9187	4.023710E-01
Gnaz	19986	0.097837199	11896	1.681221E-01
Gnb1	19987	0.154418108	15918	2.901920E-02
Gnb1l	19988	0.074565752	10182	2.940075E-01
Gnb2	19989	0.146489143	15447	3.846514E-02
Gnb2l1	19990	0.064291737	9493	3.657518E-01
Gnb3	19991	0.115627573	13365	1.030076E-01
Gnb4	19992	0.109317081	12645	1.233391E-01
Gnb5	19993	0.113548679	13094	1.093908E-01
Gne	19994	0.14167721	15058	4.537458E-02

Spearman Rank correlation analysis performed between Prdm1 and all-expressed genes within the Meredith RNA-seq dataset. Robust Prdm1–associated genes were identified using a cut-off of $p < 0.0005$.

Table S1, Related to Supplemental Figure 3C. Prdm1 associated genes

Gng10	19995	0.13027023	14309	6.597488E-02
Gng11	19996		#N/A	1.000000E+00
Gng12	19997	0.169395404	16895	1.648700E-02
Gng13	19998	-0.004955569	4936	9.444774E-01
Gng2	19999	0.042391968	7765	5.511597E-01
Gng2-ps1	20000		#N/A	1.000000E+00
Gng3	20001		#N/A	1.000000E+00
Gng4	20002	-0.084830261	573	2.323546E-01
Gng5	20003	0.166506933	16748	1.844916E-02
Gng7	20004	-0.043969054	2373	5.364318E-01
Gng8	20005		#N/A	1.000000E+00
Gngt1	20006	0.035759538	7363	6.151701E-01
Gngt2	20007	0.070688113	9887	3.199018E-01
Gnl1	20008	0.006188346	5603	9.306964E-01
Gnl2	20009	0.06263939	9369	3.782270E-01
Gnl3	20010	0.062215088	9337	3.814719E-01
Gnl3l	20011	0.050368875	8329	4.787538E-01
Gnmt	20012	0.097706346	11883	1.686945E-01
Gnpat	20013	-0.008208278	4813	9.081622E-01
Gnpda1	20014	-0.059739893	1401	4.007374E-01
Gnpda2	20015	0.109200439	12637	1.237422E-01
Gnpnat1	20016	0.162837334	16534	2.123210E-02
Gnptab	20017	0.20059954	18268	4.397664E-03
Gnptg	20018	0.215297073	18755	2.201119E-03
Gnrh1	20019		#N/A	1.000000E+00
Gnrhr	20020	0.029748956	7055	6.758233E-01
Gns	20021	0.063757729	9455	3.697554E-01
Golga1	20022	0.229567162	19139	1.075462E-03
Golga2	20023	-0.043619662	2388	5.396776E-01
Golga3	20024	0.074407242	10171	2.950378E-01
Golga4	20025	0.137802237	14811	5.166722E-02
Golga5	20026	0.083539225	10883	2.395650E-01
Golga7	20027	0.100264438	12077	1.577652E-01
Golga7b	20028	0.114452027	13288	1.065799E-01
Golgb1	20029	0.07493809	10233	2.915966E-01
Golim4	20030	0.142097976	15088	4.473162E-02
Golm1	20031	-0.035190045	3706	6.208145E-01
Golph3	20032	0.10239995	12205	1.490564E-01
Golph3l	20033	0.125486528	14002	7.663902E-02
Golt1a	20034	0.109930598	12709.5	1.212354E-01
Golt1b	20035	0.076128641	10355	2.839766E-01
Gon4l	20036	0.11069577	12770	1.186508E-01
Gopc	20037	0.147197057	15489	3.752772E-02

Spearman Rank correlation analysis performed between Prdm1 and all-expressed genes within the Meredith RNA-seq dataset. Robust Prdm1–associated genes were identified using a cut-off of $p < 0.0005$.

Table S1, Related to Supplemental Figure 3C. Prdm1 associated genes

Gorab	20038	-0.000722302	5232	9.919009E-01
Gorasp1	20039	-0.056076923	1485	4.302889E-01
Gorasp2	20040	0.194142951	18002	5.875578E-03
Gosr1	20041	0.052821922	8583	4.575717E-01
Gosr2	20042	0.025382361	6843	7.212653E-01
Got1	20043	0.049213149	8235	4.889147E-01
Got1l1	20044		#N/A	1.000000E+00
Got2	20045	0.204092264	18395	3.746138E-03
Got2-ps1	20046		#N/A	1.000000E+00
Gp1ba	20047		#N/A	1.000000E+00
Gp1bb	20048	0.139227275	14905	4.927364E-02
Gp2	20049	-0.052133786	2159	4.634605E-01
Gp49a	20050	0.070846738	9901	3.188143E-01
Gp5	20051		#N/A	1.000000E+00
Gp6	20052	-0.018103565	4364	7.991563E-01
Gp9	20053	-0.038406171	3065	5.892384E-01
Gpa33	20054	0.066028768	9590	3.529155E-01
Gpaa1	20055	0.169035165	16872	1.672134E-02
Gpam	20056	-0.00147522	5118	9.834594E-01
Gpank1	20057	0.222467007	18968	1.544386E-03
Gpat2	20058	-0.065882987	1309	3.539818E-01
Gpatch1	20059	0.087781835	11183	2.164479E-01
Gpatch11	20060	0.004802157	5550	9.461936E-01
Gpatch2	20061	0.121344075	13747	8.696329E-02
Gpatch2l	20062	0.229354333	19132	1.087365E-03
Gpatch3	20063	0.143250593	15152	4.300929E-02
Gpatch4	20064	0.07102673	9912	3.175833E-01
Gpatch8	20065	0.100507374	12091	1.567556E-01
Gpbar1	20066		#N/A	1.000000E+00
Gpbp1	20067	0.094540544	11676	1.829886E-01
Gpbp1l1	20068	0.10168634	12162	1.519251E-01
Gpc1	20069	0.109752891	12696	1.218418E-01
Gpc2	20070	-0.054450825	1984	4.437997E-01
Gpc3	20071	0.110982983	12787	1.176918E-01
Gpc4	20072	0.256262428	19580	2.495364E-04
Gpc5	20073	0.120497608	13692	8.920420E-02
Gpc6	20074	0.210361735	18587	2.791190E-03
Gpcpd1	20075	0.093845112	11634	1.862446E-01
Gpd1	20076	0.170346484	16949	1.588201E-02
Gpd1l	20077	0.223916042	19014	1.435719E-03
Gpd2	20078	0.035512762	7348	6.176133E-01
Gpha2	20079		#N/A	1.000000E+00
Gphb5	20080	0.113548679	13094	1.093908E-01

Spearman Rank correlation analysis performed between Prdm1 and all-expressed genes within the Meredith RNA-seq dataset. Robust Prdm1–associated genes were identified using a cut-off of $p < 0.0005$.

Table S1, Related to Supplemental Figure 3C. Prdm1 associated genes

Gphn	20081	0.058170723	9105	4.132459E-01
Gpi1	20082	0.115030692	13321	1.048094E-01
Gpihbp1	20083	-0.038406171	3065	5.892384E-01
Gpkow	20084	0.032660685	7216	6.461486E-01
Gpld1	20085	0.167107623	16773	1.802518E-02
Gpm6a	20086	0.175855748	17210	1.274453E-02
Gpm6b	20087	0.135912151	14692	5.499015E-02
Gpn1	20088	0.102338339	12202	1.493024E-01
Gpn2	20089	0.069333332	9799	3.292875E-01
Gpn3	20090	0.052652354	8515	4.590190E-01
Gpnmb	20091	0.223419373	18998	1.472151E-03
Gpr1	20092	-0.077397959	691.5	2.760011E-01
Gpr101	20093		#N/A	1.000000E+00
Gpr107	20094	0.186584685	17701	8.158502E-03
Gpr108	20095	0.181078813	17450	1.028675E-02
Gpr110	20096	0.104332452	12313	1.414939E-01
Gpr111	20097	-0.038406171	3065	5.892384E-01
Gpr112	20098	0.076208861	10362	2.834680E-01
Gpr113	20099	0.0670144	9645	3.457593E-01
Gpr114	20100	0.154458225	15922	2.897697E-02
Gpr115	20101	-0.04514386	2328	5.255898E-01
Gpr116	20102	0.049234487	8237	4.887260E-01
Gpr119	20103		#N/A	1.000000E+00
Gpr12	20104	0.297699265	19892	1.859003E-05
Gpr123	20105		#N/A	1.000000E+00
Gpr124	20106	-0.018105422	4347	7.991361E-01
Gpr125	20107	0.007630774	5663	9.145981E-01
Gpr126	20108	0.12432226	13928	7.943515E-02
Gpr128	20109	0.037120402	7438	6.017740E-01
Gpr132	20110	0.023524814	6720	7.409077E-01
Gpr133	20111	-0.038257571	3627	5.906810E-01
Gpr135	20112		#N/A	1.000000E+00
Gpr137	20113	-0.009802645	4754	8.904264E-01
Gpr137b	20114	0.075355495	10293	2.889097E-01
Gpr137c	20115	0.03812495	7487	5.919699E-01
Gpr139	20116	-0.032450477	3820	6.482730E-01
Gpr141	20117	0.195738513	18075	5.473996E-03
Gpr142	20118	0.072887938	10056	3.050354E-01
Gpr143	20119	0.168466304	16849.5	1.709730E-02
Gpr144	20120		#N/A	1.000000E+00
Gpr146	20121	0.141830562	15072	4.513936E-02
Gpr149	20122	-0.018105422	4347	7.991361E-01
Gpr15	20123		#N/A	1.000000E+00

Spearman Rank correlation analysis performed between Prdm1 and all-expressed genes within the Meredith RNA-seq dataset. Robust Prdm1–associated genes were identified using a cut-off of $p < 0.0005$.

Table S1, Related to Supplemental Figure 3C. Prdm1 associated genes

Gpr150	20124	-0.107936164	195	1.281776E-01
Gpr151	20125		#N/A	1.000000E+00
GPR152	20126		#N/A	1.000000E+00
Gpr152	20127	0.053267784	8685	4.537786E-01
Gpr153	20128	0.131401892	14371	6.363829E-02
Gpr155	20129	0.298711236	19900	1.735634E-05
Gpr156	20130	0.077569934	10446	2.749323E-01
Gpr157	20131	0.144153654	15318	4.169903E-02
Gpr158	20132	0.057961769	9083	4.149287E-01
Gpr160	20133	-0.058624568	1423	4.096047E-01
Gpr161	20134	0.137190443	14768	5.272406E-02
Gpr162	20135	-0.075578854	876	2.874787E-01
Gpr165	20136	0.089371505	11299	2.082104E-01
Gpr17	20137	-0.095270302	333	1.796171E-01
Gpr171	20138	0.1699933	16929	1.610437E-02
Gpr173	20139	0.213588796	18702	2.391117E-03
Gpr174	20140	0.216575906	18805	2.067988E-03
Gpr176	20141	-0.043779528	2378	5.381912E-01
Gpr179	20142	-0.084130927	589	2.362412E-01
Gpr18	20143	0.078564613	10499	2.688056E-01
Gpr180	20144	-0.00993968	4747	8.889044E-01
Gpr182	20145	0.073928833	10125	2.981622E-01
Gpr183	20146	0.022752206	6677	7.491280E-01
Gpr19	20147	-0.072874406	923.5	3.051254E-01
Gpr20	20148	0.053267784	8685	4.537786E-01
Gpr21	20149		#N/A	1.000000E+00
Gpr22	20150	0.113548679	13094	1.093908E-01
Gpr25	20151	0.071098393	9917	3.170940E-01
Gpr26	20152	0.079124235	10538	2.653998E-01
Gpr27	20153		#N/A	1.000000E+00
Gpr3	20154		#N/A	1.000000E+00
Gpr30	20155		#N/A	1.000000E+00
Gpr31b	20156		#N/A	1.000000E+00
Gpr33	20157	0.053267784	8685	4.537786E-01
Gpr34	20158	0.307212507	19931	9.649266E-06
Gpr35	20159	-0.046961184	2261	5.090401E-01
Gpr37	20160	-0.00174858	5086	9.803950E-01
Gpr37l1	20161		#N/A	1.000000E+00
Gpr39	20162	0.067463565	9671	3.425288E-01
Gpr4	20163	0.065207999	9546	3.589452E-01
Gpr44	20164		#N/A	1.000000E+00
Gpr45	20165	0.175890964	17212	1.272636E-02
Gpr50	20166	0.000542784	5343	9.939138E-01

Spearman Rank correlation analysis performed between Prdm1 and all-expressed genes within the Meredith RNA-seq dataset. Robust Prdm1–associated genes were identified using a cut-off of $p < 0.0005$.

Table S1, Related to Supplemental Figure 3C. Prdm1 associated genes

Gpr55	20167	-0.001606757	5107.5	9.819849E-01
Gpr56	20168	0.042836145	7797	5.469918E-01
Gpr6	20169		#N/A	1.000000E+00
Gpr61	20170		#N/A	1.000000E+00
Gpr62	20171	-0.038406171	3065	5.892384E-01
Gpr63	20172	0.126710802	14086	7.378524E-02
Gpr64	20173	0.086341218	11074	2.241117E-01
Gpr65	20174	0.071917374	9992	3.115374E-01
Gpr68	20175	0.109285069	12643	1.234496E-01
Gpr75	20176	-0.054451513	1703	4.437940E-01
Gpr81	20177		#N/A	1.000000E+00
Gpr82	20178	0.072930141	10060	3.047547E-01
Gpr83	20179	-0.066855996	1239.5	3.469032E-01
Gpr84	20180	0.00030705	5316.5	9.965570E-01
Gpr85	20181	-0.066855996	1239.5	3.469032E-01
Gpr87	20182	-0.075782308	871	2.861794E-01
Gpr88	20183	0.00030705	5316.5	9.965570E-01
Gpr89	20184	0.045072175	7946	5.262481E-01
Gpr97	20185	0.003986658	5515	9.553205E-01
Gpr98	20186	0.103327451	12260	1.453894E-01
Gprasp1	20187	0.002637408	5453	9.704333E-01
Gprasp2	20188	0.110398473	12747	1.196499E-01
Gprc5a	20189	-0.08224987	615	2.469208E-01
Gprc5b	20190	0.157646331	16135	2.578459E-02
Gprc5c	20191	0.121159865	13737	8.744708E-02
Gprc5d	20192		#N/A	1.000000E+00
Gprc6a	20193	0.174434051	17143	1.349708E-02
Gprin1	20194	-0.066856562	1139	3.468991E-01
Gprin2	20195		#N/A	1.000000E+00
Gprin3	20196	0.030551373	7099	6.675930E-01
Gps1	20197	0.078106373	10471	2.716165E-01
Gps2	20198	0.174901716	17159	1.324534E-02
Gpsm1	20199	0.013377923	5953	8.508649E-01
Gpsm2	20200	0.164295135	16611	2.008586E-02
Gpsm3	20201		#N/A	1.000000E+00
Gpt	20202	0.065942381	9585	3.535471E-01
Gpt2	20203	0.195022715	18046	5.651009E-03
Gpx1	20204	0.048536319	8186	4.949183E-01
Gpx2	20205	-0.038406171	3065	5.892384E-01
Gpx2-ps1	20206		#N/A	1.000000E+00
Gpx3	20207	-0.037547309	3645	5.975986E-01
Gpx4	20208	0.084909397	10984	2.319177E-01
Gpx5	20209	0.04229078	7759	5.521114E-01

Spearman Rank correlation analysis performed between Prdm1 and all-expressed genes within the Meredith RNA-seq dataset. Robust Prdm1–associated genes were identified using a cut-off of $p < 0.0005$.

Table S1, Related to Supplemental Figure 3C. Prdm1 associated genes

Gpx6	20210	0.166148774	16726	1.870606E-02
Gpx7	20211	0.12872012	14226	6.928888E-02
Gpx8	20212	-0.031504378	3860.5	6.578695E-01
Gramd1a	20213	0.111094143	12796	1.173223E-01
Gramd1b	20214	0.011068989	5829	8.763777E-01
Gramd1c	20215	0.070599642	9881	3.205094E-01
Gramd2	20216	0.19352919	17974	6.036941E-03
Gramd3	20217	-0.121955059	96	8.537398E-02
Gramd4	20218	0.11627777	13413	1.010727E-01
Grap	20219	0.076275963	10367	2.830431E-01
Grap2	20220	-0.020397608	4226	7.743516E-01
Grasp	20221	0.158124175	16182	2.533313E-02
Grb10	20222	0.10722672	12511	1.307196E-01
Grb14	20223	0.176216262	17235	1.255965E-02
Grb2	20224	0.241099537	19381	5.835107E-04
Grb7	20225	0.061505061	9302	3.869398E-01
Grcc10	20226	0.094000938	11642	1.855114E-01
Greb1	20227	0.11410347	13259	1.076577E-01
Greb1l	20228	0.125786641	14022	7.593133E-02
Grem1	20229	-0.035704478	3695	6.157149E-01
Grem2	20230	0.077124196	10419	2.777083E-01
Grhl1	20231	-0.029549729	3949	6.778728E-01
Grhl2	20232	0.082080304	10753	2.478998E-01
Grhl3	20233	-0.167197901	6	1.796220E-02
Grhpr	20234	0.025753526	6859	7.173617E-01
Gria1	20235	0.186889564	17714	8.053008E-03
Gria2	20236	0.041509741	7712	5.594838E-01
Gria3	20237	0.030806028	7117	6.649893E-01
Gria4	20238	0.165861581	16693	1.891430E-02
Grid1	20239	0.129233273	14256	6.817710E-02
Grid2	20240	0.14913953	15600	3.505405E-02
Grid2ip	20241	0.159709007	16260	2.388407E-02
Grifin	20242	0.044495949	7898.5	5.315554E-01
Grik1	20243	0.064774253	9526	3.621574E-01
Grik2	20244	0.183470777	17568	9.308326E-03
Grik3	20245	0.094788843	11690	1.818363E-01
Grik4	20246	0.036412382	7402	6.087273E-01
Grik5	20247	0.06319827	9401	3.739786E-01
Grin1	20248	0.069666882	9828	3.269605E-01
Grin2a	20249	0.12147295	13750	8.662610E-02
Grin2b	20250	0.279919178	19796	5.959998E-05
Grin2c	20251	0.097790885	11893	1.683245E-01
Grin2d	20252	0.149614145	15626	3.447110E-02

Spearman Rank correlation analysis performed between Prdm1 and all-expressed genes within the Meredith RNA-seq dataset. Robust Prdm1–associated genes were identified using a cut-off of $p < 0.0005$.

Table S1, Related to Supplemental Figure 3C. Prdm1 associated genes

Grin3a	20253	-0.055733137	1498	4.331254E-01
Grin3b	20254		#N/A	1.000000E+00
Grina	20255	0.066046075	9592	3.527891E-01
Grip1	20256	0.121749889	13777	8.590505E-02
Grip2	20257	-0.066858259	1029.5	3.468868E-01
Gripap1	20258	0.118262998	13546	9.534228E-02
Grk1	20259	0.077930565	10465	2.727002E-01
Grk4	20260	0.05690159	9032	4.235285E-01
Grk5	20261	0.170211975	16940	1.596638E-02
Grk6	20262	0.123132558	13854	8.237681E-02
Grlf1	20263	0.046926284	8071	5.093554E-01
Grm1	20264	0.081651454	10713	2.503876E-01
Grm2	20265	-0.054451513	1703	4.437940E-01
Grm3	20266	0.135356219	14652	5.600052E-02
Grm4	20267	0.056755928	9025	4.247181E-01
Grm5	20268	0.179052737	17358	1.118545E-02
Grm6	20269	0.068272528	9723	3.367590E-01
Grm7	20270	0.061329146	9288	3.883018E-01
Grm8	20271	0.016686743	6157	8.145787E-01
Grn	20272	0.109782327	12699	1.217412E-01
Grp	20273	-0.135563455	49	5.562210E-02
Grpel1	20274	0.074324436	10165	2.955770E-01
Grpel2	20275	0.066765182	9635	3.475600E-01
Grpr	20276	-0.010944517	4705	8.777570E-01
Grrp1	20277		#N/A	1.000000E+00
Grsf1	20278	-0.019099907	4269	7.883569E-01
Grtp1	20279	0.231877902	19208	9.537020E-04
Grwd1	20280	0.187455406	17742	7.860422E-03
Grxcr1	20281	0.038266143	7510	5.905977E-01
Grxcr2	20282	-0.000929698	5187.5	9.895755E-01
Gsap	20283	0.024464056	6787	7.309539E-01
Gsc	20284		#N/A	1.000000E+00
Gsc2	20285		#N/A	1.000000E+00
Gsdma	20286	0.157779192	16141	2.565838E-02
Gsdma2	20287	0.010798401	5811	8.793765E-01
Gsdma3	20288		#N/A	1.000000E+00
Gsdmc	20289	0.001131589	5368.5	9.873120E-01
Gsdmc2	20290	-0.054450825	1984	4.437997E-01
Gsdmc3	20291		#N/A	1.000000E+00
Gsdmc4	20292	-0.066855996	1239.5	3.469032E-01
Gsdmcl1	20293		#N/A	1.000000E+00
Gsdmcl2	20294	-0.038406171	3065	5.892384E-01
Gsdmd	20295	0.109409176	12649	1.230215E-01

Spearman Rank correlation analysis performed between Prdm1 and all-expressed genes within the Meredith RNA-seq dataset. Robust Prdm1–associated genes were identified using a cut-off of $p < 0.0005$.

Table S1, Related to Supplemental Figure 3C. Prdm1 associated genes

Gse1	20296		#N/A	1.000000E+00
Gsg1	20297		#N/A	1.000000E+00
Gsg1l	20298	0.007535724	5659	9.156579E-01
Gsg2	20299	-0.048162875	2231	4.982475E-01
Gsk3a	20300	0.038439725	7521	5.889128E-01
Gsk3b	20301	0.154989523	15979	2.842262E-02
Gskip	20302	0.014064526	5993	8.433068E-01
Gsn	20303	0.14516757	15363	4.026807E-02
Gspt1	20304	0.150484376	15680	3.342356E-02
Gspt2	20305	-0.001201765	5178	9.865252E-01
Gsr	20306	0.164282768	16609	2.009535E-02
Gss	20307	-0.033740883	3747	6.352772E-01
Gsta1	20308	0.109930598	12709.5	1.212354E-01
Gsta2	20309	0.08817098	11213	2.144102E-01
Gsta3	20310	-0.054451513	1703	4.437940E-01
Gsta4	20311	-0.076317426	854.5	2.827807E-01
Gstcd	20312	0.155793965	16026	2.760053E-02
Gstk1	20313	0.089510156	11310	2.075028E-01
Gstm1	20314	0.029106731	7019	6.824385E-01
Gstm2	20315	-0.04611596	2284	5.167034E-01
Gstm3	20316	0.154685737	15965	2.873847E-02
Gstm4	20317	0.082650137	10800	2.446207E-01
Gstm5	20318	0.128631226	14221	6.948297E-02
Gstm6	20319	0.001630623	5400	9.817173E-01
Gstm7	20320	0.052728522	8550.5	4.583686E-01
Gsto1	20321	0.08438217	10949	2.348397E-01
Gsto2	20322	0.052728522	8550.5	4.583686E-01
Gstp1	20323	0.180950102	17445	1.034189E-02
GSTP1	20324		#N/A	1.000000E+00
GSTP2	20325		#N/A	1.000000E+00
Gstp2	20326		#N/A	1.000000E+00
Gstt1	20327	0.012116759	5881	8.647828E-01
Gstt2	20328	0.098133001	11922	1.668334E-01
Gstt3	20329		#N/A	1.000000E+00
Gstt4	20330	0.043302928	7823.5	5.426285E-01
Gstz1	20331	-0.128891955	69	6.891495E-02
Gsx1	20332		#N/A	1.000000E+00
Gsx2	20333	0.074574898	10201.5	2.939481E-01
Gt(ROSA)26Sor	20334	0.072981378	10063	3.044141E-01
Gtdc1	20335	0.064733272	9522	3.624618E-01
Gtdc2	20336	0.182155895	17489	9.835459E-03
Gtf2a1	20337	0.118629879	13562	9.431210E-02
Gtf2a1l	20338	-0.054451513	1703	4.437940E-01

Spearman Rank correlation analysis performed between Prdm1 and all-expressed genes within the Meredith RNA-seq dataset. Robust Prdm1–associated genes were identified using a cut-off of $p < 0.0005$.

Table S1, Related to Supplemental Figure 3C. Prdm1 associated genes

Gtf2a2	20339	0.030813102	7118	6.649170E-01
Gtf2b	20340	0.136162614	14708	5.453990E-02
Gtf2e1	20341	0.117597012	13499	9.723516E-02
Gtf2e2	20342	-0.009869054	4749	8.896888E-01
Gtf2f1	20343	0.026390793	6895	7.106767E-01
Gtf2f2	20344	0.091666944	11499	1.967174E-01
Gtf2h1	20345	-0.005131871	4926	9.425055E-01
Gtf2h2	20346	0.092226669	11531	1.939862E-01
Gtf2h3	20347	0.048493886	8185	4.952960E-01
Gtf2h4	20348	-0.052777323	2145	4.579521E-01
Gtf2h5	20349	0.114420685	13285	1.066765E-01
Gtf2i	20350	0.119743784	13644	9.123850E-02
Gtf2ird1	20351	0.118557886	13560	9.451355E-02
Gtf2ird2	20352	-0.050888172	2184	4.742258E-01
Gtf3a	20353	0.007616978	5661	9.147519E-01
Gtf3c1	20354	0.074991908	10248	2.912493E-01
Gtf3c2	20355	0.125908815	14035	7.564475E-02
Gtf3c3	20356	0.050077378	8303	4.813057E-01
Gtf3c4	20357	0.17142404	16994	1.522006E-02
Gtf3c5	20358	0.082433336	10784	2.458647E-01
Gtf3c6	20359	0.058078064	9094	4.139916E-01
Gtl3	20360	0.153642605	15873	2.984595E-02
Gtpbp1	20361	0.187237498	17727	7.934098E-03
Gtpbp10	20362	0.193648535	17977	6.005257E-03
Gtpbp2	20363	0.10649217	12460	1.333923E-01
Gtpbp3	20364	0.087427881	11157	2.183133E-01
Gtpbp4	20365	0.003117162	5480	9.650581E-01
Gtpbp5	20366	-0.054451513	1703	4.437940E-01
Gtpbp6	20367	-0.034644519	3723	6.262422E-01
Gtpbp8	20368	0.190841105	17876	6.791306E-03
Gtse1	20369	0.199024564	18199	4.723482E-03
Gtsf1	20370	-0.014677583	4535	8.365703E-01
Gtsf1l	20371		#N/A	1.000000E+00
Guca1a	20372		#N/A	1.000000E+00
Guca1b	20373	0.07521908	10268	2.897860E-01
Guca2a	20374		#N/A	1.000000E+00
Guca2b	20375	-0.129032486	68	6.861037E-02
Gucd1	20376	0.269057217	19713	1.169288E-04
Gucy1a2	20377	-0.00461923	4954	9.482404E-01
Gucy1a3	20378	-0.030875624	3897.5	6.642784E-01
Gucy1b2	20379	-0.090503066	412	2.024858E-01
Gucy1b3	20380	-0.054450825	1984	4.437997E-01
Gucy2c	20381	0.012885631	5930	8.562925E-01

Spearman Rank correlation analysis performed between Prdm1 and all-expressed genes within the Meredith RNA-seq dataset. Robust Prdm1–associated genes were identified using a cut-off of $p < 0.0005$.

Table S1, Related to Supplemental Figure 3C. Prdm1 associated genes

Gucy2d	20382	0.074574898	10201.5	2.939481E-01
Gucy2e	20383	-0.066858259	1029.5	3.468868E-01
Gucy2f	20384	0.045603613	7974	5.213773E-01
Gucy2g	20385	0.042988444	7809	5.455663E-01
Guf1	20386	0.107496223	12521	1.297494E-01
Guk1	20387	0.211918042	18650	2.591234E-03
Gulo	20388	0.068343175	9728	3.362581E-01
Gulp1	20389	0.08787711	11194	2.159477E-01
Gusb	20390	0.22998991	19150	1.052171E-03
Gvin1	20391		#N/A	1.000000E+00
Gxylt1	20392	0.051610658	8440	4.679652E-01
Gxylt2	20393	0.082641852	10799	2.446681E-01
Gyg	20394	0.047860509	8131	5.009517E-01
Gyk	20395	-0.113508093	141	1.095184E-01
Gykl1	20396		#N/A	1.000000E+00
Gyltl1b	20397	0.014703935	6052	8.362810E-01
Gypa	20398	-0.054450825	1984	4.437997E-01
Gypc	20399	0.028874997	7010	6.848314E-01
Gys1	20400	0.065307376	9549	3.582117E-01
Gys2	20401	-0.018790377	4285	7.917077E-01
Gzf1	20402	0.098891118	11987	1.635645E-01
Gzma	20403		#N/A	1.000000E+00
Gzmb	20404	0.192384755	17928	6.348436E-03
Gzmc	20405	-0.000381563	5265	9.957215E-01
Gzmd	20406		#N/A	1.000000E+00
Gzme	20407	-0.086746694	532	2.219355E-01
Gzmf	20408	-0.019066625	4270	7.887170E-01
Gzmg	20409	-0.054450825	1984	4.437997E-01
Gzmk	20410	0.053267784	8685	4.537786E-01
Gzmm	20411	0.178096663	17310	1.163307E-02
Gzmn	20412	-0.038406171	3065	5.892384E-01
H13	20413	0.152848717	15834	3.071303E-02
H19	20414	-0.064332465	1337.5	3.654475E-01
H1f0	20415	0.181631745	17466	1.005284E-02
H1fnt	20416	-0.054450825	1984	4.437997E-01
H1foo	20417	-0.085763499	558	2.272386E-01
H1fx	20418	-7.57115E-05	5285	9.991510E-01
H2-Aa	20419	0.148205572	15543	3.622561E-02
H2-Ab1	20420	0.140265672	14971	4.758824E-02
H2-Bl	20421	-0.038406171	3065	5.892384E-01
H2-D1	20422	0.240321197	19365	6.086614E-04
H2-DMa	20423	0.09397182	11641	1.856482E-01
H2-DMb1	20424	0.106432488	12456	1.336113E-01

Spearman Rank correlation analysis performed between Prdm1 and all-expressed genes within the Meredith RNA-seq dataset. Robust Prdm1–associated genes were identified using a cut-off of $p < 0.0005$.

Table S1, Related to Supplemental Figure 3C. Prdm1 associated genes

H2-DMb2	20425	0.140777459	15007	4.677543E-02
H2-Ea-ps	20426	-0.000108159	5282	9.987872E-01
H2-Eb1	20427	0.172959205	17078	1.431868E-02
H2-Eb2	20428	0.080862954	10653	2.550069E-01
H2-K1	20429	0.236292339	19295	7.556134E-04
H2-K2	20430	0.097046679	11840	1.716022E-01
H2-Ke2	20431	0.003778022	5505	9.576565E-01
H2-Ke6	20432	0.022198316	6629.5	7.550388E-01
H2-M1	20433	-0.038406171	3065	5.892384E-01
H2-M10.1	20434	-0.02063596	4217	7.717870E-01
H2-M10.2	20435		#N/A	1.000000E+00
H2-M10.3	20436		#N/A	1.000000E+00
H2-M10.4	20437		#N/A	1.000000E+00
H2-M10.5	20438		#N/A	1.000000E+00
H2-M10.6	20439		#N/A	1.000000E+00
H2-M11	20440		#N/A	1.000000E+00
H2-M2	20441	0.109214357	12640	1.236941E-01
H2-M3	20442	0.268316321	19707	1.223038E-04
H2-M5	20443	-0.054451513	1703	4.437940E-01
H2-M6-ps	20444		#N/A	1.000000E+00
H2-M9	20445	-0.066858259	1029.5	3.468868E-01
H2-Oa	20446	0.067053506	9647	3.454773E-01
H2-Ob	20447	0.162669517	16505	2.136761E-02
H2-Pa	20448		#N/A	1.000000E+00
H2-Pb	20449	-0.038406171	3065	5.892384E-01
H2-Q1	20450	0.044085114	7870	5.353557E-01
H2-Q10	20451	0.185801619	17672	8.435106E-03
H2-Q2	20452	0.009550304	5750	8.932301E-01
H2-Q3	20453		#N/A	1.000000E+00
H2-Q4	20454	0.145335835	15373	4.003464E-02
H2-Q5	20455	0.111965064	12850	1.144583E-01
H2-Q6	20456	0.203039673	18358	3.932667E-03
H2-Q7	20457	0.160568696	16370	2.312830E-02
H2-T10	20458		#N/A	1.000000E+00
H2-T22	20459	0.2963267	19890	2.039623E-05
H2-T23	20460	0.113041017	12930	1.109957E-01
H2-T24	20461	-0.082655555	611	2.445896E-01
H2-T3	20462	-0.029653683	3944	6.768031E-01
H2afb1	20463		#N/A	1.000000E+00
H2afb2-ps	20464		#N/A	1.000000E+00
H2afb3-ps	20465		#N/A	1.000000E+00
H2afj	20466	0.091322149	11472	1.984137E-01
H2afv	20467	0.148033351	15539	3.644522E-02

Spearman Rank correlation analysis performed between Prdm1 and all-expressed genes within the Meredith RNA-seq dataset. Robust Prdm1–associated genes were identified using a cut-off of $p < 0.0005$.

Table S1, Related to Supplemental Figure 3C. Prdm1 associated genes

H2afx	20468	0.194943201	18042	5.670985E-03
H2afy	20469	0.082858894	10819	2.434270E-01
H2afy2	20470	0.041703187	7726	5.576534E-01
H2afy3	20471	0.015288781	6086	8.298661E-01
H2afz	20472	0.266679287	19696	1.350103E-04
H2bfm	20473		#N/A	1.000000E+00
H3f3a	20474	0.183865799	17587	9.154926E-03
H3f3a-ps1	20475		#N/A	1.000000E+00
H3f3a-ps2	20476		#N/A	1.000000E+00
H3f3b	20477	0.177748623	17301	1.179988E-02
H3f3c	20478		#N/A	1.000000E+00
H60b	20479		#N/A	1.000000E+00
H60c	20480		#N/A	1.000000E+00
H6pd	20481	0.078816909	10519	2.672665E-01
Haao	20482	-0.000186695	5277	9.979066E-01
Habp2	20483	-0.029775693	3935	6.755485E-01
Habp4	20484	0.027327732	6946	7.008882E-01
Hace1	20485	0.013346699	5952	8.512090E-01
Hacl1	20486	0.078004935	10468	2.722414E-01
Hadh	20487	0.162925739	16540	2.116101E-02
Hadha	20488	0.147371674	15497	3.729948E-02
Hadhb	20489	0.096961238	11833	1.719815E-01
Hagh	20490	0.1381979	14837	5.099315E-02
Haghl	20491	0.180999054	17448	1.032089E-02
Hal	20492	0.068982303	9774	3.317480E-01
Hamp	20493	0.082119497	10756	2.476733E-01
Hamp2	20494	-0.00566732	4896	9.365186E-01
Hand1	20495	0.021757451	6575	7.597536E-01
Hand2	20496		#N/A	1.000000E+00
Hao1	20497	0.061486189	9300	3.870858E-01
Hao2	20498	-0.028518853	3979	6.885151E-01
Hap1	20499	-0.146442802	33	3.852719E-02
Hapln1	20500	0.032749452	7223	6.452523E-01
Hapln2	20501	0.090625548	11396.5	2.018731E-01
Hapln3	20502	0.053267784	8685	4.537786E-01
Hapln4	20503	-0.038406171	3065	5.892384E-01
Harbi1	20504	0.111490672	12819	1.160115E-01
Hars	20505	0.159705018	16259	2.388763E-02
Hars2	20506	0.190355558	17857	6.936196E-03
Has1	20507	-0.054451513	1703	4.437940E-01
Has2	20508	0.145811445	15401	3.938099E-02
Has2as	20509	0.03028139	7077	6.703579E-01
Has3	20510	-0.054450825	1984	4.437997E-01

Spearman Rank correlation analysis performed between Prdm1 and all-expressed genes within the Meredith RNA-seq dataset. Robust Prdm1–associated genes were identified using a cut-off of $p < 0.0005$.

Table S1, Related to Supplemental Figure 3C. Prdm1 associated genes

Hat1	20511	0.136364805	14720	5.417866E-02
Haus1	20512	0.131775325	14390	6.288229E-02
Haus2	20513	0.186208817	17690	8.290249E-03
Haus3	20514	0.133656879	14525	5.918449E-02
Haus4	20515	0.15188108	15759	3.179882E-02
Haus5	20516		#N/A	1.000000E+00
Haus6	20517	0.186613883	17702	8.148346E-03
Haus7	20518	0.185136957	17643	8.676383E-03
Haus8	20519	-0.04648573	2273	5.133436E-01
Havcr1	20520	0.028016658	6975	6.937219E-01
Havcr2	20521	0.061422606	9294	3.875779E-01
Hax1	20522	0.183524164	17573	9.287462E-03
Hba-a1	20523	0.112355243	12883	1.131930E-01
Hba-a2	20524		#N/A	1.000000E+00
Hba-ps4	20525	0.143605682	15224	4.249002E-02
Hba-x	20526		#N/A	1.000000E+00
Hbb-b1	20527		#N/A	1.000000E+00
Hbb-b2	20528	-0.054450825	1984	4.437997E-01
Hbb-bh0	20529		#N/A	1.000000E+00
Hbb-bh1	20530		#N/A	1.000000E+00
Hbb-bh2	20531		#N/A	1.000000E+00
Hbb-bh3	20532		#N/A	1.000000E+00
Hbb-y	20533		#N/A	1.000000E+00
Hbegf	20534	0.058037207	9089	4.143207E-01
Hbp1	20535	0.091794206	11508	1.960940E-01
Hbq1a	20536		#N/A	1.000000E+00
Hbq1b	20537		#N/A	1.000000E+00
Hbs1l	20538	0.041072953	7696	5.636273E-01
Hc	20539	0.085921893	11051	2.263782E-01
Hccs	20540	0.178841616	17347	1.128297E-02
Hcfc1	20541	0.269970779	19717	1.106054E-04
Hcfc1r1	20542	0.058218215	9106	4.128640E-01
Hcfc2	20543	0.104744296	12337	1.399207E-01
Hck	20544	0.105530604	12394	1.369542E-01
Hcls1	20545	0.208996068	18551	2.978062E-03
Hcn1	20546	0.008990549	5729	8.994540E-01
Hcn2	20547	0.098182091	11928	1.666203E-01
Hcn3	20548	0.038424289	7520	5.890626E-01
Hcn4	20549	-0.038406171	3065	5.892384E-01
Hcrt	20550	0.211254406	18621	2.674853E-03
Hcrtr1	20551	-0.054450825	1984	4.437997E-01
Hcrtr2	20552	0.125761757	14019	7.598980E-02
Hcst	20553	0.048446374	8169.5	4.957191E-01

Spearman Rank correlation analysis performed between Prdm1 and all-expressed genes within the Meredith RNA-seq dataset. Robust Prdm1–associated genes were identified using a cut-off of $p < 0.0005$.

Table S1, Related to Supplemental Figure 3C. Prdm1 associated genes

Hdac1	20554	0.099737094	12043	1.599735E-01
Hdac10	20555	-0.025519895	4087	7.198180E-01
Hdac11	20556	-0.109390588	185	1.230856E-01
Hdac2	20557	0.157804682	16166	2.563422E-02
Hdac3	20558	0.074920061	10231	2.917131E-01
Hdac4	20559	-0.026071928	4066	7.140189E-01
Hdac5	20560	0.378908738	20023	3.153656E-08
Hdac6	20561	0.020872904	6388	7.692400E-01
Hdac7	20562	0.232418418	19216	9.271132E-04
Hdac8	20563	0.059448319	9182	4.030445E-01
Hdac9	20564	0.030279784	7076	6.703743E-01
Hdc	20565	-0.038157769	3629	5.916508E-01
Hddc2	20566	-0.041199194	2447	5.624282E-01
Hddc3	20567	0.027576796	6954	6.982943E-01
Hdgf	20568	0.134016799	14552	5.849796E-02
Hdgfl1	20569	0.326859571	19982	2.313422E-06
Hdgfrp2	20570	-0.055958449	1489	4.312652E-01
Hdgfrp3	20571	0.13403247	14554	5.846822E-02
Hdhd1a	20572		#N/A	1.000000E+00
Hdhd2	20573	0.152823111	15833	3.074135E-02
Hdhd3	20574	-0.050001529	2197	4.819710E-01
Hdlbp	20575	0.139084883	14896	4.950858E-02
Hdx	20576	-0.005137179	4925	9.424461E-01
Heatr1	20577	0.063042084	9391	3.751629E-01
Heatr2	20578	0.106601347	12466	1.329924E-01
Heatr3	20579	0.123498388	13875	8.146307E-02
Heatr5a	20580	0.167007339	16771	1.809537E-02
Heatr5b	20581	0.045659684	7977	5.208648E-01
Heatr6	20582	0.036844037	7424	6.044839E-01
Hebp1	20583	0.082019019	10747	2.482543E-01
Hebp2	20584	0.038569477	7530	5.876548E-01
Heca	20585	0.05362659	8781	4.507389E-01
Hectd1	20586	0.064132433	9481	3.669433E-01
Hectd2	20587	0.266988026	19698	1.325233E-04
Hectd3	20588	0.072401342	10021	3.082839E-01
Hecw1	20589	0.130766389	14340	6.494194E-02
Hecw2	20590	0.123204779	13859	8.219577E-02
Heg1	20591	0.086241987	11068	2.246466E-01
Helb	20592	0.06854444	9743	3.348336E-01
Hells	20593	0.080649047	10644	2.562702E-01
Helq	20594	0.02396839	6748	7.362013E-01
Helt	20595	0.038229357	7499.5	5.909551E-01
Helz	20596	0.128656165	14222	6.942847E-02

Spearman Rank correlation analysis performed between Prdm1 and all-expressed genes within the Meredith RNA-seq dataset. Robust Prdm1–associated genes were identified using a cut-off of $p < 0.0005$.

Table S1, Related to Supplemental Figure 3C. Prdm1 associated genes

Helz2	20597	0.198664125	18186	4.801030E-03
Hemgn	20598	0.157776642	16140	2.566080E-02
Hemk1	20599	0.121785821	13778	8.581185E-02
Hemt1	20600		#N/A	1.000000E+00
Henmt1	20601	0.073540302	10102	3.007156E-01
Hepacam	20602	0.099088553	12003	1.627211E-01
Hepacam2	20603	-0.016267086	4463.5	8.191608E-01
Heph	20604	0.082452722	10786	2.457533E-01
Hephl1	20605	-0.066856562	1139	3.468991E-01
Herc1	20606	0.233426093	19236	8.793558E-04
Herc2	20607	0.143167928	15145	4.313093E-02
Herc3	20608	0.264771077	19675	1.513766E-04
Herc4	20609	0.119058817	13592	9.311892E-02
Herc6	20610	0.123921279	13903	8.041700E-02
Herpud1	20611	0.164724142	16639	1.975891E-02
Herpud2	20612	0.219574816	18889	1.784061E-03
Hes1	20613	0.244293294	19429	4.900368E-04
Hes2	20614	0.020434681	6357	7.739526E-01
Hes3	20615		#N/A	1.000000E+00
Hes5	20616		#N/A	1.000000E+00
Hes6	20617	0.066130899	9599	3.521697E-01
Hes7	20618	-0.018367686	4326	7.962897E-01
Hesx1	20619		#N/A	1.000000E+00
Hexa	20620	-0.052366011	2153	4.614686E-01
Hexb	20621	0.155520056	16008	2.787813E-02
Hexdc	20622	-0.001316536	5127	9.852384E-01
Hexim1	20623	-0.007749173	4830	9.132782E-01
Hexim2	20624	-0.077397959	691.5	2.760011E-01
Hey1	20625	-0.074347366	898	2.954277E-01
Hey2	20626	0.00030705	5316.5	9.965570E-01
Heyl	20627	-0.034829567	3714	6.243988E-01
Hfe	20628	0.015859017	6115	8.236222E-01
Hfe2	20629		#N/A	1.000000E+00
Hfm1	20630	0.124606742	13946	7.874445E-02
Hgd	20631	-0.035124528	3708	6.214653E-01
Hgf	20632	0.030875887	7121	6.642757E-01
Hgfac	20633	-0.077394997	736.5	2.760195E-01
Hgs	20634	0.001705583	5407	9.808770E-01
Hgsnat	20635	0.046388008	8038	5.142304E-01
Hhat	20636	0.106653608	12471	1.328013E-01
Hhatl	20637	0.073928833	10125	2.981622E-01
Hhex	20638	0.021100114	6440.5	7.667999E-01
Hhip	20639	0.190944875	17878	6.760693E-03

Spearman Rank correlation analysis performed between Prdm1 and all-expressed genes within the Meredith RNA-seq dataset. Robust Prdm1–associated genes were identified using a cut-off of $p < 0.0005$.

Table S1, Related to Supplemental Figure 3C. Prdm1 associated genes

Hhipl1	20640	-0.054451513	1703	4.437940E-01
Hhipl2	20641	-0.038406171	3065	5.892384E-01
Hhla1	20642	-0.054450825	1984	4.437997E-01
Hiat1	20643	0.158929887	16221	2.458727E-02
Hiatl1	20644	0.125399756	13999	7.684463E-02
Hibadh	20645	0.15045939	15678	3.345326E-02
Hibch	20646	0.031365391	7150	6.592841E-01
Hic1	20647	0.090767581	11406	2.011642E-01
Hic2	20648	-0.045149205	2323	5.255407E-01
Hid1	20649	0.32732703	19983	2.233363E-06
Hif1a	20650	0.189318236	17816	7.255006E-03
Hif1an	20651	0.011981518	5875	8.662779E-01
Hif3a	20652	0.086154251	11063	2.251203E-01
Higd1a	20653	0.141736575	15064	4.528340E-02
Higd1b	20654	0.008412329	5700	9.058896E-01
Higd1c	20655		#N/A	1.000000E+00
Higd2a	20656	0.204894735	18423	3.609336E-03
Hilpda	20657	-0.046364296	2277	5.144457E-01
Hils1	20658	-0.066858259	1029.5	3.468868E-01
Hinfp	20659	0.047886471	8142	5.007192E-01
Hint1	20660	0.152484263	15791	3.111822E-02
Hint2	20661	0.057222367	9047	4.209156E-01
Hint3	20662	0.27734483	19775	7.010076E-05
Hip1	20663	0.221308219	18939	1.636640E-03
Hip1r	20664	0.115906961	13388	1.021726E-01
Hipk1	20665	0.185389754	17654	8.583904E-03
Hipk2	20666	0.083420905	10860	2.402336E-01
Hipk3	20667	0.223652047	19010	1.454980E-03
Hipk4	20668		#N/A	1.000000E+00
Hira	20669	-0.034433115	3728	6.283510E-01
Hirip3	20670	0.139290888	14909	4.916898E-02
Hist1h1a	20671	0.027326276	6945	7.009033E-01
Hist1h1b	20672	0.195907074	18079	5.433042E-03
Hist1h1c	20673	0.135117981	14627	5.643817E-02
Hist1h1d	20674	0.012390518	5897	8.617580E-01
Hist1h1e	20675	-0.077397959	691.5	2.760011E-01
Hist1h1t	20676		#N/A	1.000000E+00
Hist1h2aa	20677	-0.038406171	3065	5.892384E-01
Hist1h2ab	20678		#N/A	1.000000E+00
Hist1h2ac	20679	0.035982677	7381	6.129647E-01
Hist1h2ad	20680		#N/A	1.000000E+00
Hist1h2ae	20681	-0.038406171	3065	5.892384E-01
Hist1h2af	20682		#N/A	1.000000E+00

Spearman Rank correlation analysis performed between Prdm1 and all-expressed genes within the Meredith RNA-seq dataset. Robust Prdm1–associated genes were identified using a cut-off of $p < 0.0005$.

Table S1, Related to Supplemental Figure 3C. Prdm1 associated genes

Hist1h2ag	20683	0.157799267	16153.5	2.563935E-02
Hist1h2ah	20684		#N/A	1.000000E+00
Hist1h2ai	20685	0.084141322	10930.5	2.361831E-01
Hist1h2aj	20686		#N/A	1.000000E+00
Hist1h2ak	20687	0.216630157	18807.5	2.062506E-03
Hist1h2al	20688	0.053267784	8685	4.537786E-01
Hist1h2an	20689		#N/A	1.000000E+00
Hist1h2ao	20690		#N/A	1.000000E+00
Hist1h2ap	20691		#N/A	1.000000E+00
Hist1h2ba	20692		#N/A	1.000000E+00
Hist1h2bb	20693	0.020434854	6365.5	7.739507E-01
Hist1h2bc	20694	0.003818086	5508	9.572079E-01
Hist1h2be	20695	0.080485	10633	2.572419E-01
Hist1h2bf	20696		#N/A	1.000000E+00
Hist1h2bg	20697	-0.066856562	1139	3.468991E-01
Hist1h2bh	20698	-0.077397959	691.5	2.760011E-01
Hist1h2bj	20699		#N/A	1.000000E+00
Hist1h2bk	20700	0.113548679	13094	1.093908E-01
Hist1h2bl	20701		#N/A	1.000000E+00
Hist1h2bm	20702		#N/A	1.000000E+00
Hist1h2bn	20703		#N/A	1.000000E+00
Hist1h2bp	20704	-0.001224019	5151	9.862757E-01
Hist1h2bq	20705		#N/A	1.000000E+00
Hist1h2br	20706	-0.038406171	3065	5.892384E-01
Hist1h3a	20707		#N/A	1.000000E+00
Hist1h3b	20708		#N/A	1.000000E+00
Hist1h3c	20709		#N/A	1.000000E+00
Hist1h3d	20710	-0.066855996	1239.5	3.469032E-01
Hist1h3e	20711		#N/A	1.000000E+00
Hist1h3f	20712		#N/A	1.000000E+00
Hist1h3g	20713		#N/A	1.000000E+00
Hist1h3h	20714	0.082762295	10811.5	2.439788E-01
Hist1h3i	20715		#N/A	1.000000E+00
Hist1h4a	20716		#N/A	1.000000E+00
Hist1h4b	20717	0.113548679	13094	1.093908E-01
Hist1h4c	20718		#N/A	1.000000E+00
Hist1h4d	20719	-0.054451513	1703	4.437940E-01
Hist1h4f	20720		#N/A	1.000000E+00
Hist1h4h	20721	-0.066858259	1029.5	3.468868E-01
Hist1h4i	20722	0.106148563	12435	1.346569E-01
Hist1h4j	20723		#N/A	1.000000E+00
Hist1h4k	20724		#N/A	1.000000E+00
Hist1h4m	20725	0.113548679	13094	1.093908E-01

Spearman Rank correlation analysis performed between Prdm1 and all-expressed genes within the Meredith RNA-seq dataset. Robust Prdm1–associated genes were identified using a cut-off of $p < 0.0005$.

Table S1, Related to Supplemental Figure 3C. Prdm1 associated genes

Hist1h4n	20726		#N/A	1.000000E+00
Hist2h2aa1	20727		#N/A	1.000000E+00
Hist2h2aa2	20728		#N/A	1.000000E+00
Hist2h2ab	20729		#N/A	1.000000E+00
Hist2h2ac	20730	0.1544596	15936.5	2.897553E-02
Hist2h2bb	20731	0.007273308	5644	9.185846E-01
Hist2h2be	20732	-0.082108181	617	2.477386E-01
Hist2h3b	20733		#N/A	1.000000E+00
Hist2h3c1	20734	-0.020402993	4224	7.742937E-01
Hist2h3c2	20735	0.143605682	15224	4.249002E-02
Hist2h4	20736		#N/A	1.000000E+00
Hist3h2a	20737	0.126029843	14047	7.536172E-02
Hist3h2ba	20738	-0.066855996	1239.5	3.469032E-01
Hist3h2bb-ps	20739		#N/A	1.000000E+00
Hist4h4	20740	-0.071561139	935	3.139465E-01
Hivep1	20741	0.075871101	10337	2.856135E-01
Hivep2	20742	0.319718379	19963	3.933490E-06
Hivep3	20743	0.13175816	14388	6.291688E-02
Hjurp	20744	0.205503726	18436	3.508544E-03
Hk1	20745	0.123308638	13865	8.193599E-02
Hk2	20746	0.000429647	5338	9.951824E-01
Hk3	20747	-0.09526662	364	1.796340E-01
Hkdc1	20748	-0.054451513	1703	4.437940E-01
Hlcs	20749	0.077475346	10441	2.755198E-01
Hlf	20750	0.142203	15093	4.457233E-02
Hltf	20751	0.167718125	16809	1.760297E-02
Hlx	20752	0.110085291	12722	1.207094E-01
Hmbox1	20753	0.171567507	17000	1.513377E-02
Hmbs	20754	-0.004536314	4958	9.491682E-01
Hmcn1	20755	0.022525843	6660	7.515419E-01
Hmcn2	20756	-0.084599571	581	2.336317E-01
Hmg1l1	20757		#N/A	1.000000E+00
Hmg20a	20758	0.051280463	8411	4.708208E-01
Hmg20b	20759	0.04357341	7837	5.401080E-01
Hmga1	20760	0.202939339	18355	3.950877E-03
Hmga1-rs1	20761	0.029748956	7055	6.758233E-01
Hmga2	20762	0.06609486	9597	3.524328E-01
Hmgb1	20763	0.13123519	14363	6.397816E-02
Hmgb1-ps1	20764		#N/A	1.000000E+00
Hmgb1-ps2	20765		#N/A	1.000000E+00
Hmgb1-ps3	20766		#N/A	1.000000E+00
Hmgb1-ps4	20767		#N/A	1.000000E+00
Hmgb1-ps5	20768		#N/A	1.000000E+00

Spearman Rank correlation analysis performed between Prdm1 and all-expressed genes within the Meredith RNA-seq dataset. Robust Prdm1–associated genes were identified using a cut-off of $p < 0.0005$.

Table S1, Related to Supplemental Figure 3C. Prdm1 associated genes

Hmgb1-ps6	20769		#N/A	1.000000E+00
Hmgb1-rs18	20770		#N/A	1.000000E+00
Hmgb1l	20771		#N/A	1.000000E+00
Hmgb2	20772	0.108197661	12562	1.272503E-01
Hmgb3	20773	0.042177276	7750	5.531798E-01
Hmgb4	20774		#N/A	1.000000E+00
Hmgcl	20775	0.008475682	5705	9.051842E-01
Hmgcll1	20776	0.122243653	13800	8.463143E-02
Hmgcr	20777	0.175578238	17193	1.288846E-02
Hmgcs1	20778	0.239360002	19354	6.411017E-04
Hmgcs2	20779	-0.038406171	3065	5.892384E-01
Hmgn1	20780	0.007750938	5668	9.132585E-01
Hmgn2	20781	0.105004222	12356	1.389347E-01
Hmgn2-ps1	20782		#N/A	1.000000E+00
Hmgn2l6	20783		#N/A	1.000000E+00
Hmgn3	20784	0.25323698	19541	2.968683E-04
Hmgn5	20785	0.286891814	19847	3.809364E-05
Hmgxb3	20786	0.148988469	15590	3.524134E-02
Hmgxb4	20787	0.142457085	15108	4.418892E-02
Hmha1	20788	0.132405988	14444	6.162229E-02
Hmmr	20789	0.204982111	18426	3.594715E-03
Hmox1	20790	-0.07484919	887	2.921710E-01
Hmox2	20791	0.18121199	17454	1.022998E-02
Hmx1	20792	0.283561236	19821	4.724427E-05
Hmx2	20793	-0.004525984	4965	9.492838E-01
Hmx3	20794		#N/A	1.000000E+00
Hn1	20795	0.116105955	13400	1.015812E-01
Hn1l	20796	0.104961772	12352	1.390953E-01
Hnf1a	20797	0.12630859	14065	7.471313E-02
Hnf1b	20798	-0.03367206	3757.5	6.359676E-01
Hnf4a	20799	-0.117577649	117	9.729063E-02
Hnf4g	20800	0.060401964	9231	3.955283E-01
Hnmt	20801	0.084533834	10956	2.339965E-01
Hnrnpa0	20802	0.134499214	14588	5.758809E-02
Hnrnpa1	20803	0.117451041	13488	9.765400E-02
Hnrnpa1l2-ps	20804		#N/A	1.000000E+00
Hnrnpa2b1	20805	0.165045343	16664	1.951715E-02
Hnrnpa3	20806	0.185583475	17663	8.513629E-03
Hnrnpab	20807	0.131345586	14369	6.375292E-02
Hnrnpc	20808	0.19502126	18044	5.651374E-03
Hnrnpd	20809	0.272621756	19741	9.402105E-05
Hnrnpf	20810	0.215464825	18770	2.183222E-03
Hnrnph1	20811	0.097271791	11859	1.706058E-01

Spearman Rank correlation analysis performed between Prdm1 and all-expressed genes within the Meredith RNA-seq dataset. Robust Prdm1–associated genes were identified using a cut-off of $p < 0.0005$.

Table S1, Related to Supplemental Figure 3C. Prdm1 associated genes

Hnrnph2	20812	0.244950271	19438	4.726158E-04
Hnrnph3	20813	0.209189431	18557	2.950932E-03
Hnrnpk	20814	0.263591343	19665	1.624032E-04
Hnrnpl	20815	0.123373684	13869	8.177362E-02
Hnrnpm	20816	0.171964162	17018	1.489742E-02
Hnrnpr	20817	0.012427107	5899	8.613539E-01
Hnrnpu	20818	0.158687514	16212	2.480963E-02
Hnrnpul1	20819	0.050535895	8337	4.772949E-01
Hnrnpul2	20820	0.180569476	17433	1.050646E-02
Hnrpdl	20821	0.117643061	13504	9.710332E-02
Hnrpll	20822	0.039013825	7579	5.833559E-01
Hoga1	20823	-0.003389066	5010	9.620124E-01
Homer1	20824	0.200314614	18252	4.455066E-03
Homer2	20825	0.119798283	13653	9.109020E-02
Homer3	20826	-0.049738088	2201	4.842853E-01
Homez	20827	0.022825799	6682	7.483438E-01
Hook1	20828	0.114287821	13271	1.070866E-01
Hook2	20829	0.098208102	11932	1.665075E-01
Hook3	20830	0.080301132	10619	2.583340E-01
Hopx	20831	0.091212984	11457	1.989530E-01
Hormad1	20832	-0.001224019	5151	9.862757E-01
Hormad2	20833	0.088141885	11210	2.145620E-01
Hotair	20834	0.129781147	14289	6.700621E-02
Hoxa1	20835	-0.004526355	4961.5	9.492796E-01
Hoxa10	20836	0.112984011	12929	1.111771E-01
Hoxa11	20837	-0.054450825	1984	4.437997E-01
Hoxa11as	20838	0.053267784	8685	4.537786E-01
Hoxa13	20839		#N/A	1.000000E+00
Hoxa2	20840	0.113548679	13094	1.093908E-01
Hoxa3	20841	0.125831775	14027	7.582536E-02
Hoxa4	20842		#N/A	1.000000E+00
Hoxa5	20843	0.325933867	19979	2.480100E-06
Hoxa6	20844		#N/A	1.000000E+00
Hoxa7	20845	0.00030705	5316.5	9.965570E-01
Hoxa9	20846	0.014759448	6059.5	8.356716E-01
Hoxb1	20847	0.171954201	17016	1.490332E-02
Hoxb13	20848		#N/A	1.000000E+00
Hoxb2	20849	-0.016730797	4444.5	8.140981E-01
Hoxb3	20850	0.109492511	12666.5	1.227347E-01
Hoxb3os	20851	-0.077397959	691.5	2.760011E-01
Hoxb4	20852	-0.060615773	1389	3.938547E-01
Hoxb5	20853	0.182294195	17509.5	9.778799E-03
Hoxb6	20854	0.176189557	17233	1.257326E-02

Spearman Rank correlation analysis performed between Prdm1 and all-expressed genes within the Meredith RNA-seq dataset. Robust Prdm1–associated genes were identified using a cut-off of $p < 0.0005$.

Table S1, Related to Supplemental Figure 3C. Prdm1 associated genes

Hoxb7	20855	0.07417953	10147.5	2.965222E-01
Hoxb8	20856		#N/A	1.000000E+00
Hoxb9	20857	-0.020397608	4226	7.743516E-01
Hoxc10	20858	0.035233935	7339	6.203787E-01
Hoxc11	20859	-0.042212104	2430	5.528519E-01
Hoxc12	20860		#N/A	1.000000E+00
Hoxc13	20861	0.119869459	13657	9.089680E-02
Hoxc4	20862	0.047035654	8082	5.083678E-01
Hoxc5	20863	-0.066856562	1139	3.468991E-01
Hoxc6	20864	-0.034734605	3721	6.253445E-01
Hoxc8	20865	0.021169772	6446	7.660523E-01
Hoxc9	20866	0.113313879	12941	1.101308E-01
Hoxd1	20867	0.303827466	19916	1.221717E-05
Hoxd10	20868	-0.038406171	3065	5.892384E-01
Hoxd11	20869	0.000307056	5327	9.965570E-01
Hoxd12	20870	-0.038406171	3065	5.892384E-01
Hoxd13	20871		#N/A	1.000000E+00
Hoxd3	20872	0.074412153	10173	2.950059E-01
Hoxd8	20873	0.126047776	14050.5	7.531986E-02
Hoxd9	20874	0.083810603	10907.5	2.380365E-01
Hp	20875		#N/A	1.000000E+00
Hp1bp3	20876	0.15996458	16278	2.365720E-02
Hpca	20877	0.051192803	8396	4.715805E-01
Hpcal1	20878	0.073138162	10075	3.033736E-01
Hpcal4	20879	-0.054450825	1984	4.437997E-01
Hpd	20880		#N/A	1.000000E+00
Hpdl	20881		#N/A	1.000000E+00
Hpgd	20882	0.114920019	13315	1.051463E-01
Hpgds	20883	0.022384717	6654	7.530480E-01
Hpn	20884	-0.084104413	591	2.363894E-01
Hprt	20885	0.117434529	13486	9.770147E-02
Hps1	20886	0.060900264	9256	3.916345E-01
Hps3	20887	0.111977052	12853	1.144192E-01
Hps4	20888	0.114083018	13257	1.077212E-01
Hps5	20889	0.043268441	7821	5.429503E-01
Hps6	20890		#N/A	1.000000E+00
Hpse	20891	-0.001261706	5129.5	9.858531E-01
Hpse2	20892	-0.029791128	3933	6.753898E-01
Hpx	20893	0.177720372	17299	1.181352E-02
Hr	20894	0.170837211	16968	1.557750E-02
Hras1	20895	0.061086985	9267	3.901815E-01
Hrasls	20896	-0.038406171	3065	5.892384E-01
Hrasls5	20897	-0.038406171	3065	5.892384E-01

Spearman Rank correlation analysis performed between Prdm1 and all-expressed genes within the Meredith RNA-seq dataset. Robust Prdm1–associated genes were identified using a cut-off of $p < 0.0005$.

Table S1, Related to Supplemental Figure 3C. Prdm1 associated genes

Hrc	20898	0.012453251	5902	8.610651E-01
Hrct1	20899	0.160985049	16395	2.276975E-02
Hrg	20900		#N/A	1.000000E+00
Hrh1	20901	0.109492511	12666.5	1.227347E-01
Hrh2	20902	0.053805701	8820	4.492258E-01
Hrh3	20903		#N/A	1.000000E+00
Hrh4	20904		#N/A	1.000000E+00
Hrk	20905	0.050762316	8360	4.753210E-01
Hrnr	20906	-0.002388981	5053	9.732172E-01
Hrsp12	20907	0.092078421	11522	1.947069E-01
Hs1bp3	20908	-0.041790669	2438	5.568266E-01
Hs2st1	20909	0.020833977	6387	7.696583E-01
Hs3st1	20910	0.102039652	12183	1.504996E-01
Hs3st2	20911	-0.056705422	1466.5	4.251310E-01
Hs3st3a1	20912	0.081702959	10718	2.500879E-01
Hs3st3b1	20913	0.065867296	9580	3.540967E-01
Hs3st4	20914	0.070053867	9844	3.242739E-01
Hs3st5	20915	0.055505485	8955	4.350096E-01
Hs3st6	20916	-0.054450825	1984	4.437997E-01
Hs6st1	20917	0.125206552	13983	7.730404E-02
Hs6st2	20918	-0.085393164	567	2.292591E-01
Hs6st3	20919	0.226848298	19081	1.236920E-03
Hsbp1	20920	0.001611129	5397	9.819358E-01
Hsbp1l1	20921	0.01769628	6211	8.035820E-01
Hscb	20922	0.079433791	10557	2.635286E-01
Hsd11b1	20923	0.06904595	9784	3.313010E-01
Hsd11b2	20924	0.084902384	10983	2.319564E-01
Hsd17b1	20925		#N/A	1.000000E+00
Hsd17b10	20926	0.068008178	9703	3.386375E-01
Hsd17b11	20927	0.095027709	11699	1.807328E-01
Hsd17b12	20928	0.045661906	7979	5.208445E-01
Hsd17b13	20929	0.149711195	15633	3.435291E-02
Hsd17b14	20930	0.054887085	8914	4.401515E-01
Hsd17b2	20931	0.165268379	16673	1.935079E-02
Hsd17b3	20932	-0.017760534	4379.5	8.028834E-01
Hsd17b4	20933	0.162655665	16503	2.137883E-02
Hsd17b6	20934	0.049238404	8238	4.886914E-01
Hsd17b7	20935	0.07988056	10584	2.608440E-01
Hsd3b1	20936		#N/A	1.000000E+00
Hsd3b2	20937	-0.038406171	3065	5.892384E-01
Hsd3b3	20938	-0.032762514	3799	6.451205E-01
Hsd3b4	20939		#N/A	1.000000E+00
Hsd3b5	20940		#N/A	1.000000E+00

Spearman Rank correlation analysis performed between Prdm1 and all-expressed genes within the Meredith RNA-seq dataset. Robust Prdm1–associated genes were identified using a cut-off of $p < 0.0005$.

Table S1, Related to Supplemental Figure 3C. Prdm1 associated genes

Hsd3b6	20941	-0.038406171	3065	5.892384E-01
Hsd3b7	20942	0.13894338	14881	4.974298E-02
Hsdl1	20943	0.024859637	6810	7.267750E-01
Hsdl2	20944	-0.033729799	3748	6.353884E-01
Hsf1	20945	0.167141172	16777	1.800175E-02
Hsf2	20946	0.220578778	18918	1.697266E-03
Hsf2bp	20947	0.17027948	16946	1.592399E-02
Hsf3	20948	0.04366723	7843	5.392351E-01
Hsf4	20949	-0.038406171	3065	5.892384E-01
Hsf5	20950	-0.01467878	4534	8.365572E-01
Hsfy2	20951		#N/A	1.000000E+00
Hsh2d	20952	0.095311519	11723	1.794281E-01
Hsp25-ps1	20953		#N/A	1.000000E+00
Hsp90aa1	20954	0.148425612	15556	3.594665E-02
Hsp90ab1	20955	0.227291603	19087	1.209163E-03
Hsp90b1	20956	0.163717943	16578	2.053312E-02
Hspa12a	20957	-0.066855996	1239.5	3.469032E-01
Hspa12b	20958		#N/A	1.000000E+00
Hspa13	20959	0.240830019	19376	5.921089E-04
Hspa14	20960	0.134717966	14602	5.717937E-02
Hspa1a	20961	-0.029815037	3932	6.751441E-01
Hspa1b	20962	0.032244463	7195	6.503578E-01
Hspa1l	20963	0.113548679	13094	1.093908E-01
Hspa2	20964	0.092425336	11543	1.930235E-01
Hspa4	20965	0.130366236	14316	6.577397E-02
Hspa4l	20966	0.089581345	11315	2.071401E-01
Hspa5	20967	0.179649063	17389	1.091402E-02
Hspa8	20968	0.182807799	17543	9.570901E-03
Hspa9	20969	0.151128852	15722	3.266531E-02
Hspa9-ps1	20970	-0.038406171	3065	5.892384E-01
Hspb1	20971	0.024362028	6780	7.320330E-01
Hspb11	20972	0.086323664	11073	2.242063E-01
Hspb2	20973	-0.089735319	419	2.063573E-01
Hspb3	20974	-0.038406171	3065	5.892384E-01
Hspb6	20975	0.125184303	13981	7.735708E-02
Hspb7	20976	-0.054451513	1703	4.437940E-01
Hspb8	20977	-0.10493537	209	1.391953E-01
Hspb9	20978		#N/A	1.000000E+00
Hspbap1	20979	0.171875545	17012	1.494994E-02
Hspbp1	20980	0.054917261	8918	4.398998E-01
Hspd1	20981	0.14867023	15573	3.563867E-02
Hspe1	20982	0.139888935	14944	4.819407E-02
Hspe1-ps2	20983	0.134200998	14569.5	5.814916E-02

Spearman Rank correlation analysis performed between Prdm1 and all-expressed genes within the Meredith RNA-seq dataset. Robust Prdm1–associated genes were identified using a cut-off of $p < 0.0005$.

Table S1, Related to Supplemental Figure 3C. Prdm1 associated genes

Hspe1-ps3	20984		#N/A	1.000000E+00
Hspe1-ps4	20985	0.19429475	18010.5	5.836269E-03
Hspe1-ps5	20986	0.098384184	11948.5	1.657450E-01
Hspe1-ps6	20987	-0.038406171	3065	5.892384E-01
Hspg2	20988	0.021493289	6553	7.625829E-01
Hsph1	20989	0.116309751	13416	1.009783E-01
Htatip2	20990	-0.006309443	4872	9.293437E-01
Htatsf1	20991	0.229797758	19145	1.062699E-03
Htr1a	20992	-0.038406171	3065	5.892384E-01
Htr1b	20993		#N/A	1.000000E+00
Htr1d	20994	-0.001131589	5180	9.873120E-01
Htr1f	20995	0.050197193	8313.5	4.802559E-01
Htr2a	20996	0.091005261	11437	1.999820E-01
Htr2b	20997	-0.054451513	1703	4.437940E-01
Htr2c	20998	0.049353478	8248	4.876748E-01
Htr3a	20999		#N/A	1.000000E+00
Htr3b	21000		#N/A	1.000000E+00
Htr4	21001	-0.025064814	4099	7.246106E-01
Htr5a	21002	0.049853204	8280.5	4.832733E-01
Htr5b	21003	0.113548679	13094	1.093908E-01
Htr6	21004	-0.038406171	3065	5.892384E-01
Htr7	21005	0.055256588	8943	4.370750E-01
Htra1	21006	0.112283533	12875	1.134247E-01
Htra2	21007	0.035573719	7355	6.170094E-01
Htra3	21008	-0.066855996	1239.5	3.469032E-01
Htra4	21009	0.074574898	10201.5	2.939481E-01
Htt	21010	0.05020569	8316	4.801815E-01
Hunk	21011	0.06506067	9538	3.600343E-01
Hus1	21012	0.069369035	9803	3.290379E-01
Hus1b	21013	-0.066855996	1239.5	3.469032E-01
Huwe1	21014	0.127379261	14145	7.226382E-02
Hvcn1	21015	0.107199063	12509	1.308195E-01
Hyal1	21016		#N/A	1.000000E+00
Hyal2	21017	-0.021000025	4204	7.678745E-01
Hyal3	21018	0.317255042	19960	4.709124E-06
Hyal4	21019		#N/A	1.000000E+00
Hyal5	21020	-0.038406171	3065	5.892384E-01
Hyal6	21021		#N/A	1.000000E+00
Hydin	21022	0.091287689	11466	1.985838E-01
Hyi	21023	0.063249257	9407	3.735925E-01
Hyls1	21024	0.102192422	12191	1.498864E-01
Hyou1	21025	0.207000165	18493	3.271584E-03
Hypk	21026	0.222327924	18962	1.555202E-03

Spearman Rank correlation analysis performed between Prdm1 and all-expressed genes within the Meredith RNA-seq dataset. Robust Prdm1–associated genes were identified using a cut-off of $p < 0.0005$.

Table S1, Related to Supplemental Figure 3C. Prdm1 associated genes

I0C0044D17Rik	21027		#N/A	1.000000E+00
I830012O16Rik	21028	-0.046018991	2288	5.175864E-01
I830077J02Rik	21029	0.072343587	10013	3.086710E-01
I830134H01Rik	21030		#N/A	1.000000E+00
Iah1	21031	0.171918567	17014	1.492443E-02
Iapp	21032	-0.023471279	4140	7.414764E-01
Iars	21033	0.100631266	12099	1.562427E-01
Iars2	21034	0.206024363	18460	3.424399E-03
Iba57	21035	-0.136796716	47	5.341363E-02
Ibsp	21036	0.050539378	8338	4.772645E-01
Ibtk	21037	-0.013347246	4603	8.512029E-01
Ica1	21038	-0.026839438	4037	7.059835E-01
Ica1l	21039	0.000307056	5327	9.965570E-01
Icam1	21040	0.125316436	13991	7.704248E-02
Icam2	21041	0.199474208	18226	4.628320E-03
Icam4	21042		#N/A	1.000000E+00
Icam5	21043	0.046174239	8024	5.161731E-01
Ick	21044	0.137679697	14802	5.187748E-02
Icmt	21045	0.082686521	10804	2.444123E-01
Icos	21046	0.108794958	12609	1.251516E-01
Icosl	21047	0.101446035	12145	1.529005E-01
Ict1	21048	-0.008796298	4795	9.016153E-01
Id1	21049	0.143950482	15310	4.199085E-02
Id2	21050	0.048377467	8167	4.963330E-01
Id3	21051	0.085268219	11007	2.299437E-01
Id4	21052	-0.044084038	2369.5	5.353657E-01
Ide	21053	0.182249074	17502	9.797253E-03
Idh1	21054	0.0023025	5432	9.741864E-01
Idh2	21055	0.279839503	19795	5.990151E-05
Idh3a	21056	0.163648228	16575	2.058772E-02
Idh3b	21057	0.163979165	16594	2.032964E-02
Idh3g	21058	0.196526129	18104	5.284977E-03
Idi1	21059	0.144306946	15323	4.147999E-02
Idi2	21060	-0.099709698	278	1.600889E-01
Idnk	21061	0.12924569	14258	6.815038E-02
Ido1	21062	-0.03181808	3854	6.546813E-01
Ido2	21063	-0.077393517	797	2.760287E-01
Ids	21064	0.136719299	14744	5.355009E-02
Idua	21065	-0.027834677	4005	6.956123E-01
Ier2	21066	0.146768706	15465	3.809261E-02
Ier3	21067	0.120760421	13707	8.850355E-02
Ier3ip1	21068	0.078260962	10482	2.706660E-01
Ier5	21069	0.234082207	19259	8.494880E-04

Spearman Rank correlation analysis performed between Prdm1 and all-expressed genes within the Meredith RNA-seq dataset. Robust Prdm1–associated genes were identified using a cut-off of $p < 0.0005$.

Table S1, Related to Supplemental Figure 3C. Prdm1 associated genes

Ier5l	21070	0.050386475	8331	4.786000E-01
Iffo1	21071	0.292798333	19874	2.583040E-05
Iffo2	21072	0.096907099	11829	1.722222E-01
Ifi202b	21073	0.0769125	10409	2.790333E-01
Ifi203	21074	0.14795634	15537	3.654379E-02
Ifi204	21075	0.029809596	7058	6.752000E-01
Ifi205	21076	0.169327912	16894	1.653068E-02
Ifi27l1	21077	0.00084939	5352	9.904760E-01
Ifi27l2a	21078	0.172626889	17058	1.450974E-02
Ifi27l2b	21079	-0.086746694	532	2.219355E-01
Ifi30	21080	0.062604331	9365	3.784944E-01
Ifi35	21081	0.112588221	12896	1.124427E-01
Ifi44	21082	0.178706376	17339	1.134583E-02
Ifi44l	21083	-0.038406171	3065	5.892384E-01
Ifi47	21084	0.128346572	14201	7.010746E-02
Ifih1	21085	0.062753878	9374	3.773543E-01
Ifit1	21086	0.040081572	7642	5.730859E-01
Ifit2	21087	0.05738948	9055	4.195581E-01
Ifit3	21088	0.154846616	15971	2.857083E-02
Ifitm1	21089	0.105917259	12415	1.355133E-01
Ifitm10	21090	0.164110636	16598	2.022790E-02
Ifitm2	21091	0.168832893	16868	1.685419E-02
Ifitm3	21092	0.14929563	15609	3.486140E-02
Ifitm5	21093	-0.054450825	1984	4.437997E-01
Ifitm6	21094	0.021317126	6513.5	7.644715E-01
Ifitm7	21095	0.026796026	6916.5	7.064372E-01
Ifltd1	21096	0.055812156	8973	4.324725E-01
Ifna-ps1	21097		#N/A	1.000000E+00
Ifna1	21098		#N/A	1.000000E+00
Ifna11	21099		#N/A	1.000000E+00
Ifna12	21100		#N/A	1.000000E+00
Ifna13	21101	-0.054450825	1984	4.437997E-01
Ifna14	21102		#N/A	1.000000E+00
Ifna2	21103		#N/A	1.000000E+00
Ifna4	21104		#N/A	1.000000E+00
Ifna5	21105		#N/A	1.000000E+00
Ifna6	21106		#N/A	1.000000E+00
Ifna7	21107		#N/A	1.000000E+00
Ifna9	21108		#N/A	1.000000E+00
Ifnab	21109		#N/A	1.000000E+00
Ifnar1	21110	0.007182838	5635	9.195939E-01
Ifnar2	21111	-0.031922863	3851	6.536177E-01
Ifnb1	21112	-0.038406171	3065	5.892384E-01

Spearman Rank correlation analysis performed between Prdm1 and all-expressed genes within the Meredith RNA-seq dataset. Robust Prdm1–associated genes were identified using a cut-off of $p < 0.0005$.

Table S1, Related to Supplemental Figure 3C. Prdm1 associated genes

Ifne	21113		#N/A	1.000000E+00
Ifng	21114		#N/A	1.000000E+00
Ifngr1	21115	0.221613171	18950	1.611886E-03
Ifngr2	21116	0.19574932	18076	5.471363E-03
Ifnk	21117		#N/A	1.000000E+00
Ifnl2	21118		#N/A	1.000000E+00
Ifnl3	21119	-0.077394997	736.5	2.760195E-01
Ifnlr1	21120	0.254042866	19550	2.835052E-04
Ifnz	21121		#N/A	1.000000E+00
Ifrd1	21122	0.078810432	10518	2.673059E-01
Ifrd2	21123	0.069469478	9813	3.283364E-01
Ift122	21124	0.054570758	8897	4.427951E-01
Ift140	21125	0.071127236	9920	3.168973E-01
Ift172	21126	0.079747046	10575	2.616443E-01
Ift20	21127	0.13718675	14767	5.273049E-02
Ift27	21128	0.160046341	16280	2.358502E-02
Ift43	21129	0.052184486	8485	4.630252E-01
Ift46	21130	0.03382862	7287	6.343976E-01
Ift52	21131	0.016517327	6144	8.164278E-01
Ift57	21132	0.048866806	8209	4.919819E-01
Ift74	21133	-0.038525115	2503	5.880848E-01
Ift80	21134	0.14033017	14978	4.748516E-02
Ift81	21135	-0.019575849	4251	7.832122E-01
Ift88	21136	0.037034529	7431	6.026154E-01
Igbp1	21137	-0.035780486	3691	6.149629E-01
Igbp1b	21138		#N/A	1.000000E+00
Igdcc3	21139	-0.038406171	3065	5.892384E-01
Igdcc4	21140	0.049722731	8271	4.844204E-01
Igf1	21141	0.102605069	12220	1.482395E-01
Igf1r	21142	-0.079267236	651	2.645343E-01
Igf2	21143	0.044814733	7922	5.286160E-01
Igf2as	21144	0.224798783	19038.5	1.372995E-03
Igf2bp1	21145	0.067460452	9670	3.425511E-01
Igf2bp2	21146	0.243380842	19421	5.152194E-04
Igf2bp3	21147	0.143955284	15311	4.198394E-02
Igf2r	21148	0.14685985	15473	3.797182E-02
Igfals	21149	0.123920638	13902	8.041858E-02
Igfbp1	21150	0.007210755	5639	9.192825E-01
Igfbp2	21151	0.182724654	17537	9.604289E-03
Igfbp3	21152	0.012581169	5905	8.596526E-01
Igfbp4	21153	0.046712992	8060	5.112842E-01
Igfbp5	21154	0.102178866	12190	1.499407E-01
Igfbp6	21155	0.00649074	5615	9.273190E-01

Spearman Rank correlation analysis performed between Prdm1 and all-expressed genes within the Meredith RNA-seq dataset. Robust Prdm1–associated genes were identified using a cut-off of $p < 0.0005$.

Table S1, Related to Supplemental Figure 3C. Prdm1 associated genes

Igfbp7	21156	0.019026779	6282	7.891482E-01
Igfbpl1	21157	0.057285264	9052	4.204044E-01
Igfl3	21158	-0.038406171	3065	5.892384E-01
Igflr1	21159	0.072856215	10054	3.052465E-01
Igfn1	21160	-0.054515073	1536	4.432614E-01
Igha	21161	0.042078685	7743	5.541087E-01
Ighd1-1	21162		#N/A	1.000000E+00
Ighd2-3	21163		#N/A	1.000000E+00
Ighd2-4	21164		#N/A	1.000000E+00
Ighd2-5	21165		#N/A	1.000000E+00
Ighd2-6	21166		#N/A	1.000000E+00
Ighd2-7	21167		#N/A	1.000000E+00
Ighd2-8	21168		#N/A	1.000000E+00
Ighd3-1	21169		#N/A	1.000000E+00
Ighd3-2	21170		#N/A	1.000000E+00
Ighd4-1	21171		#N/A	1.000000E+00
Ighd5-1	21172		#N/A	1.000000E+00
Ighd5-2	21173		#N/A	1.000000E+00
Ighd5-3	21174		#N/A	1.000000E+00
Ighd5-4	21175		#N/A	1.000000E+00
Ighd5-5	21176		#N/A	1.000000E+00
Ighd5-6	21177		#N/A	1.000000E+00
Ighd5-7	21178		#N/A	1.000000E+00
Ighd5-8	21179		#N/A	1.000000E+00
Ighd6-1	21180		#N/A	1.000000E+00
Ighd6-2	21181		#N/A	1.000000E+00
Ighe	21182	0.217285915	18829	1.997279E-03
Ighg1	21183	-0.110563117	176	1.190958E-01
Ighg2b	21184	-0.029767681	3937	6.756308E-01
Ighg2c	21185	-0.047558827	2246	5.036574E-01
Ighg3	21186	0.071901326	9991	3.116457E-01
Ighj1	21187		#N/A	1.000000E+00
Ighj2	21188		#N/A	1.000000E+00
Ighj3	21189		#N/A	1.000000E+00
Ighj4	21190		#N/A	1.000000E+00
Ighm	21191	0.199056384	18201	4.716691E-03
Ighmbp2	21192	-0.038809378	2492	5.853321E-01
Ighv1-12	21193		#N/A	1.000000E+00
Ighv1-14	21194		#N/A	1.000000E+00
Ighv1-15	21195		#N/A	1.000000E+00
Ighv1-18	21196		#N/A	1.000000E+00
Ighv1-19	21197		#N/A	1.000000E+00
Ighv1-2	21198		#N/A	1.000000E+00

Spearman Rank correlation analysis performed between Prdm1 and all-expressed genes within the Meredith RNA-seq dataset. Robust Prdm1–associated genes were identified using a cut-off of $p < 0.0005$.

Table S1, Related to Supplemental Figure 3C. Prdm1 associated genes

Ighv1-20	21199		#N/A	1.000000E+00
Ighv1-22	21200		#N/A	1.000000E+00
Ighv1-23	21201		#N/A	1.000000E+00
Ighv1-24	21202		#N/A	1.000000E+00
Ighv1-26	21203		#N/A	1.000000E+00
Ighv1-30	21204		#N/A	1.000000E+00
Ighv1-31	21205		#N/A	1.000000E+00
Ighv1-34	21206		#N/A	1.000000E+00
Ighv1-36	21207		#N/A	1.000000E+00
Ighv1-37	21208		#N/A	1.000000E+00
Ighv1-39	21209		#N/A	1.000000E+00
Ighv1-4	21210		#N/A	1.000000E+00
Ighv1-42	21211		#N/A	1.000000E+00
Ighv1-43	21212		#N/A	1.000000E+00
Ighv1-47	21213		#N/A	1.000000E+00
Ighv1-49	21214		#N/A	1.000000E+00
Ighv1-5	21215		#N/A	1.000000E+00
Ighv1-50	21216		#N/A	1.000000E+00
Ighv1-52	21217		#N/A	1.000000E+00
Ighv1-53	21218		#N/A	1.000000E+00
Ighv1-54	21219		#N/A	1.000000E+00
Ighv1-55	21220	0.160304018	16323	2.335876E-02
Ighv1-56	21221		#N/A	1.000000E+00
Ighv1-58	21222		#N/A	1.000000E+00
Ighv1-59	21223		#N/A	1.000000E+00
Ighv1-61	21224	-0.054450825	1984	4.437997E-01
Ighv1-62-1	21225		#N/A	1.000000E+00
Ighv1-62-2	21226		#N/A	1.000000E+00
Ighv1-62-3	21227		#N/A	1.000000E+00
Ighv1-63	21228		#N/A	1.000000E+00
Ighv1-64	21229		#N/A	1.000000E+00
Ighv1-66	21230		#N/A	1.000000E+00
Ighv1-67	21231	-0.038406171	3065	5.892384E-01
Ighv1-69	21232		#N/A	1.000000E+00
Ighv1-7	21233		#N/A	1.000000E+00
Ighv1-71	21234		#N/A	1.000000E+00
Ighv1-72	21235		#N/A	1.000000E+00
Ighv1-73	21236		#N/A	1.000000E+00
Ighv1-75	21237	-0.054450825	1984	4.437997E-01
Ighv1-76	21238		#N/A	1.000000E+00
Ighv1-77	21239	-0.054450825	1984	4.437997E-01
Ighv1-78	21240		#N/A	1.000000E+00
Ighv1-80	21241		#N/A	1.000000E+00

Spearman Rank correlation analysis performed between Prdm1 and all-expressed genes within the Meredith RNA-seq dataset. Robust Prdm1–associated genes were identified using a cut-off of $p < 0.0005$.

Table S1, Related to Supplemental Figure 3C. Prdm1 associated genes

Ighv1-81	21242		#N/A	1.000000E+00
Ighv1-82	21243		#N/A	1.000000E+00
Ighv1-83	21244		#N/A	1.000000E+00
Ighv1-84	21245		#N/A	1.000000E+00
Ighv1-85	21246		#N/A	1.000000E+00
Ighv1-9	21247		#N/A	1.000000E+00
Ighv10-1	21248		#N/A	1.000000E+00
Ighv10-3	21249		#N/A	1.000000E+00
Ighv11-1	21250		#N/A	1.000000E+00
Ighv11-2	21251		#N/A	1.000000E+00
Ighv12-3	21252		#N/A	1.000000E+00
Ighv13-1	21253		#N/A	1.000000E+00
Ighv13-2	21254		#N/A	1.000000E+00
Ighv14-1	21255		#N/A	1.000000E+00
Ighv14-2	21256		#N/A	1.000000E+00
Ighv14-3	21257		#N/A	1.000000E+00
Ighv14-4	21258		#N/A	1.000000E+00
Ighv15-2	21259		#N/A	1.000000E+00
Ighv16-1	21260		#N/A	1.000000E+00
Ighv2-1	21261		#N/A	1.000000E+00
Ighv2-2	21262		#N/A	1.000000E+00
Ighv2-3	21263		#N/A	1.000000E+00
Ighv2-4	21264		#N/A	1.000000E+00
Ighv2-5	21265		#N/A	1.000000E+00
Ighv2-6	21266		#N/A	1.000000E+00
Ighv2-6-8	21267		#N/A	1.000000E+00
Ighv2-7	21268		#N/A	1.000000E+00
Ighv2-9	21269		#N/A	1.000000E+00
Ighv2-9-1	21270		#N/A	1.000000E+00
Ighv3-1	21271		#N/A	1.000000E+00
Ighv3-3	21272		#N/A	1.000000E+00
Ighv3-5	21273		#N/A	1.000000E+00
Ighv3-6	21274		#N/A	1.000000E+00
Ighv3-8	21275		#N/A	1.000000E+00
Ighv4-1	21276		#N/A	1.000000E+00
Ighv5-12	21277		#N/A	1.000000E+00
Ighv5-15	21278		#N/A	1.000000E+00
Ighv5-16	21279	-0.038406171	3065	5.892384E-01
Ighv5-17	21280		#N/A	1.000000E+00
Ighv5-2	21281		#N/A	1.000000E+00
Ighv5-4	21282		#N/A	1.000000E+00
Ighv5-6	21283		#N/A	1.000000E+00
Ighv5-9	21284		#N/A	1.000000E+00

Spearman Rank correlation analysis performed between Prdm1 and all-expressed genes within the Meredith RNA-seq dataset. Robust Prdm1–associated genes were identified using a cut-off of $p < 0.0005$.

Table S1, Related to Supplemental Figure 3C. Prdm1 associated genes

Ighv5-9-1	21285		#N/A	1.000000E+00
Ighv6-3	21286		#N/A	1.000000E+00
Ighv6-4	21287		#N/A	1.000000E+00
Ighv6-5	21288		#N/A	1.000000E+00
Ighv6-6	21289		#N/A	1.000000E+00
Ighv7-1	21290		#N/A	1.000000E+00
Ighv7-2	21291		#N/A	1.000000E+00
Ighv7-3	21292		#N/A	1.000000E+00
Ighv7-4	21293		#N/A	1.000000E+00
Ighv8-11	21294		#N/A	1.000000E+00
Ighv8-12	21295		#N/A	1.000000E+00
Ighv8-13	21296		#N/A	1.000000E+00
Ighv8-14	21297		#N/A	1.000000E+00
Ighv8-4	21298		#N/A	1.000000E+00
Ighv8-5	21299		#N/A	1.000000E+00
Ighv8-6	21300		#N/A	1.000000E+00
Ighv8-8	21301		#N/A	1.000000E+00
Ighv8-9	21302		#N/A	1.000000E+00
Ighv9-1	21303		#N/A	1.000000E+00
Ighv9-2	21304		#N/A	1.000000E+00
Ighv9-3	21305		#N/A	1.000000E+00
Ighv9-4	21306		#N/A	1.000000E+00
Igj	21307	0.168273393	16838	1.722646E-02
Igkc	21308	0.27180562	19734	9.886043E-05
Igkj1	21309	0.161221974	16435	2.256786E-02
Igkj2	21310	0.250724521	19515	3.423813E-04
Igkj3	21311		#N/A	1.000000E+00
Igkj4	21312		#N/A	1.000000E+00
Igkj5	21313	-0.038406171	3065	5.892384E-01
Igkv1-110	21314		#N/A	1.000000E+00
Igkv1-115	21315		#N/A	1.000000E+00
Igkv1-117	21316		#N/A	1.000000E+00
Igkv1-122	21317		#N/A	1.000000E+00
Igkv1-131	21318		#N/A	1.000000E+00
Igkv1-132	21319		#N/A	1.000000E+00
Igkv1-133	21320		#N/A	1.000000E+00
Igkv1-135	21321	0.230247091	19167.5	1.038230E-03
Igkv1-35	21322		#N/A	1.000000E+00
Igkv1-88	21323		#N/A	1.000000E+00
Igkv1-99	21324		#N/A	1.000000E+00
Igkv10-94	21325	0.157799267	16153.5	2.563935E-02
Igkv10-95	21326		#N/A	1.000000E+00
Igkv10-96	21327		#N/A	1.000000E+00

Spearman Rank correlation analysis performed between Prdm1 and all-expressed genes within the Meredith RNA-seq dataset. Robust Prdm1–associated genes were identified using a cut-off of $p < 0.0005$.

Table S1, Related to Supplemental Figure 3C. Prdm1 associated genes

Igkv11-125	21328		#N/A	1.000000E+00
Igkv12-38	21329	0.159922834	16273	2.369413E-02
Igkv12-41	21330		#N/A	1.000000E+00
Igkv12-44	21331		#N/A	1.000000E+00
Igkv12-46	21332		#N/A	1.000000E+00
Igkv12-47	21333		#N/A	1.000000E+00
Igkv12-89	21334		#N/A	1.000000E+00
Igkv12-98	21335		#N/A	1.000000E+00
Igkv13-84	21336		#N/A	1.000000E+00
Igkv13-87	21337		#N/A	1.000000E+00
Igkv14-100	21338	-0.054450825	1984	4.437997E-01
Igkv14-111	21339		#N/A	1.000000E+00
Igkv14-126	21340		#N/A	1.000000E+00
Igkv14-130	21341		#N/A	1.000000E+00
Igkv15-103	21342		#N/A	1.000000E+00
Igkv16-104	21343		#N/A	1.000000E+00
Igkv17-121	21344		#N/A	1.000000E+00
Igkv17-127	21345		#N/A	1.000000E+00
Igkv17-134	21346		#N/A	1.000000E+00
Igkv18-36	21347		#N/A	1.000000E+00
Igkv19-93	21348	0.160304018	16323	2.335876E-02
Igkv2-109	21349		#N/A	1.000000E+00
Igkv2-112	21350		#N/A	1.000000E+00
Igkv2-116	21351		#N/A	1.000000E+00
Igkv2-137	21352		#N/A	1.000000E+00
Igkv2-95-2	21353		#N/A	1.000000E+00
Igkv3-1	21354		#N/A	1.000000E+00
Igkv3-10	21355		#N/A	1.000000E+00
Igkv3-12	21356		#N/A	1.000000E+00
Igkv3-2	21357		#N/A	1.000000E+00
Igkv3-3	21358		#N/A	1.000000E+00
Igkv3-4	21359		#N/A	1.000000E+00
Igkv3-5	21360		#N/A	1.000000E+00
Igkv3-7	21361		#N/A	1.000000E+00
Igkv3-9	21362		#N/A	1.000000E+00
Igkv4-50	21363		#N/A	1.000000E+00
Igkv4-53	21364		#N/A	1.000000E+00
Igkv4-54	21365		#N/A	1.000000E+00
Igkv4-55	21366	0.230235201	19161.5	1.038871E-03
Igkv4-56	21367	0.16677321	16760	1.826016E-02
Igkv4-57	21368		#N/A	1.000000E+00
Igkv4-57-1	21369		#N/A	1.000000E+00
Igkv4-58	21370		#N/A	1.000000E+00

Spearman Rank correlation analysis performed between Prdm1 and all-expressed genes within the Meredith RNA-seq dataset. Robust Prdm1–associated genes were identified using a cut-off of $p < 0.0005$.

Table S1, Related to Supplemental Figure 3C. Prdm1 associated genes

Igkv4-59	21371		#N/A	1.000000E+00
Igkv4-61	21372		#N/A	1.000000E+00
Igkv4-62	21373		#N/A	1.000000E+00
Igkv4-63	21374		#N/A	1.000000E+00
Igkv4-68	21375		#N/A	1.000000E+00
Igkv4-69	21376		#N/A	1.000000E+00
Igkv4-70	21377	0.053267784	8685	4.537786E-01
Igkv4-71	21378		#N/A	1.000000E+00
Igkv4-72	21379		#N/A	1.000000E+00
Igkv4-73	21380		#N/A	1.000000E+00
Igkv4-74	21381		#N/A	1.000000E+00
Igkv4-78	21382		#N/A	1.000000E+00
Igkv4-79	21383	0.230247091	19167.5	1.038230E-03
Igkv4-80	21384		#N/A	1.000000E+00
Igkv4-81	21385		#N/A	1.000000E+00
Igkv4-86	21386		#N/A	1.000000E+00
Igkv4-90	21387		#N/A	1.000000E+00
Igkv4-91	21388		#N/A	1.000000E+00
Igkv4-92	21389		#N/A	1.000000E+00
Igkv5-37	21390		#N/A	1.000000E+00
Igkv5-39	21391		#N/A	1.000000E+00
Igkv5-43	21392		#N/A	1.000000E+00
Igkv5-45	21393		#N/A	1.000000E+00
Igkv5-48	21394		#N/A	1.000000E+00
Igkv6-13	21395		#N/A	1.000000E+00
Igkv6-14	21396		#N/A	1.000000E+00
Igkv6-15	21397		#N/A	1.000000E+00
Igkv6-17	21398		#N/A	1.000000E+00
Igkv6-20	21399		#N/A	1.000000E+00
Igkv6-23	21400	0.157799267	16153.5	2.563935E-02
Igkv6-25	21401		#N/A	1.000000E+00
Igkv6-29	21402		#N/A	1.000000E+00
Igkv6-32	21403		#N/A	1.000000E+00
Igkv7-33	21404		#N/A	1.000000E+00
Igkv8-16	21405		#N/A	1.000000E+00
Igkv8-18	21406		#N/A	1.000000E+00
Igkv8-19	21407	0.160304018	16323	2.335876E-02
Igkv8-21	21408		#N/A	1.000000E+00
Igkv8-24	21409		#N/A	1.000000E+00
Igkv8-26	21410		#N/A	1.000000E+00
Igkv8-27	21411		#N/A	1.000000E+00
Igkv8-28	21412		#N/A	1.000000E+00
Igkv8-30	21413		#N/A	1.000000E+00

Spearman Rank correlation analysis performed between Prdm1 and all-expressed genes within the Meredith RNA-seq dataset. Robust Prdm1–associated genes were identified using a cut-off of $p < 0.0005$.

Table S1, Related to Supplemental Figure 3C. Prdm1 associated genes

Igkv9-120	21414	0.192510301	17931	6.313577E-03
Igkv9-123	21415		#N/A	1.000000E+00
Igkv9-124	21416	-0.038406171	3065	5.892384E-01
Igkv9-129	21417	0.192510301	17931	6.313577E-03
Iglc2	21418	0.176684321	17251	1.232315E-02
Igll1	21419	0.056854809	9031	4.239103E-01
Iglon5	21420	0.09963975	12036	1.603837E-01
Iglv1	21421	0.204893336	18422	3.609570E-03
Iglv2	21422	0.223431786	18999	1.471231E-03
Iglv3	21423		#N/A	1.000000E+00
Igsf1	21424	-0.086746694	532	2.219355E-01
Igsf10	21425	-0.053967919	2121	4.478579E-01
Igsf11	21426	0.203856214	18382	3.787260E-03
Igsf21	21427	-0.027439248	4017	6.997263E-01
Igsf23	21428	-0.1596035	12	2.397827E-02
Igsf3	21429	0.100823092	12111	1.554509E-01
Igsf5	21430	0.026844851	6921	7.059270E-01
Igsf6	21431	0.074393452	10169	2.951276E-01
Igsf8	21432	-0.014211745	4556	8.416880E-01
Igsf9	21433	0.042257059	7755	5.524287E-01
Igsf9b	21434	-0.038406171	3065	5.892384E-01
Igtp	21435		#N/A	1.000000E+00
Ihh	21436	-0.054451513	1703	4.437940E-01
Iigp1	21437	0.070192015	9851	3.233184E-01
Ik	21438	0.186794135	17711	8.085897E-03
Ikbip	21439	0.196814827	18114	5.217172E-03
Ikbkap	21440	0.110709715	12773	1.186041E-01
Ikbkb	21441	0.123610006	13879	8.118591E-02
Ikbke	21442	0.07283492	10051	3.053882E-01
Ikbkg	21443	0.119362669	13611	9.228099E-02
Ikzf1	21444	0.16010006	16281	2.353769E-02
Ikzf2	21445	0.231596476	19203	9.678216E-04
Ikzf3	21446	0.185426978	17658	8.570361E-03
Ikzf4	21447	-0.081824812	622	2.493799E-01
Ikzf5	21448		#N/A	1.000000E+00
Il10	21449	0.016079389	6129	8.212122E-01
Il10ra	21450	0.079178788	10540	2.650694E-01
Il10rb	21451	0.120688013	13703	8.869615E-02
Il11	21452	0.10155015	12150	1.524773E-01
Il11ra1	21453	0.090987108	11433	2.000721E-01
IL11RA2	21454		#N/A	1.000000E+00
Il11ra2	21455		#N/A	1.000000E+00
Il12a	21456	0.072899763	10057	3.049567E-01

Spearman Rank correlation analysis performed between Prdm1 and all-expressed genes within the Meredith RNA-seq dataset. Robust Prdm1–associated genes were identified using a cut-off of $p < 0.0005$.

Table S1, Related to Supplemental Figure 3C. Prdm1 associated genes

Il12b	21457	0.160304018	16323	2.335876E-02
Il12rb1	21458	0.080489631	10634	2.572144E-01
Il12rb2	21459	-0.074202458	901	2.963725E-01
Il13	21460	-0.048240487	2230	4.975546E-01
Il13ra1	21461	0.071554829	9952	3.139893E-01
Il13ra2	21462	0.058515249	9127	4.104801E-01
Il15	21463	0.155384488	15997	2.801641E-02
Il15ra	21464	0.103608092	12277	1.442935E-01
Il16	21465	0.07293741	10061	3.047063E-01
Il17a	21466		#N/A	1.000000E+00
Il17b	21467	0.00030705	5316.5	9.965570E-01
Il17c	21468		#N/A	1.000000E+00
Il17d	21469	0.036193362	7391	6.108855E-01
Il17f	21470	0.230297472	19173	1.035519E-03
Il17ra	21471	0.135935867	14695	5.494738E-02
Il17rb	21472	0.090013747	11344.5	2.049472E-01
Il17rc	21473	-0.066856562	1139	3.468991E-01
Il17rd	21474	0.006351887	5611	9.288696E-01
Il17re	21475	0.240677171	19373	5.970370E-04
Il18	21476	0.138760766	14870	5.004684E-02
Il18bp	21477	-0.086108159	555	2.253695E-01
Il18r1	21478	0.052682409	8521	4.587623E-01
Il18rap	21479	0.047870267	8136	5.008643E-01
Il19	21480	0.038229357	7499.5	5.909551E-01
Il1a	21481	0.073018262	10065	3.041691E-01
Il1b	21482	0.105783443	12408	1.360106E-01
Il1f10	21483	0.127284236	14137	7.247853E-02
Il1f5	21484	0.032652684	7214	6.462294E-01
Il1f6	21485	0.145333066	15372	4.003847E-02
Il1f8	21486	-0.054451513	1703	4.437940E-01
Il1f9	21487	0.011965191	5871	8.664584E-01
Il1r1	21488	0.107618485	12530	1.293111E-01
Il1r2	21489	0.050876437	8372	4.743279E-01
Il1rap	21490	0.07616294	10359	2.837591E-01
Il1rapl1	21491	0.129599726	14279	6.739210E-02
Il1rapl2	21492	0.241041852	19378	5.853412E-04
Il1rl1	21493	0.019392376	6299	7.851944E-01
Il1rl2	21494	-0.008338062	4807	9.067167E-01
Il1rn	21495	0.053960585	8862	4.479197E-01
Il2	21496	0.28102567	19807	5.555722E-05
Il20	21497		#N/A	1.000000E+00
Il20ra	21498	0.11194457	12847	1.145250E-01
Il20rb	21499	-0.025969098	4067	7.150979E-01

Spearman Rank correlation analysis performed between Prdm1 and all-expressed genes within the Meredith RNA-seq dataset. Robust Prdm1–associated genes were identified using a cut-off of $p < 0.0005$.

Table S1, Related to Supplemental Figure 3C. Prdm1 associated genes

Il21	21500	0.138246696	14840	5.091053E-02
Il21r	21501	0.008387622	5698	9.061648E-01
Il22	21502		#N/A	1.000000E+00
Il22ra1	21503		#N/A	1.000000E+00
Il22ra2	21504		#N/A	1.000000E+00
Il23a	21505	0.092122531	11527	1.944923E-01
Il23r	21506	0.13332014	14499	5.983280E-02
Il24	21507	0.090776969	11408	2.011174E-01
Il25	21508	0.192196617	17919	6.400995E-03
Il27	21509	0.070235085	9854	3.230208E-01
Il27ra	21510	-0.049601089	2206	4.854912E-01
Il2ra	21511	-0.032349397	3834	6.492955E-01
Il2rb	21512	0.316024598	19957	5.149012E-06
Il2rg	21513	0.067646562	9681	3.412181E-01
Il3	21514		#N/A	1.000000E+00
Il31	21515		#N/A	1.000000E+00
Il31ra	21516	0.052070603	8475	4.640033E-01
Il33	21517	-0.04681591	2265	5.103530E-01
Il34	21518	0.062349602	9346	3.804414E-01
Il3ra	21519	0.061707572	9317	3.853755E-01
Il4	21520	-0.034947638	3711	6.232238E-01
Il4i1	21521	0.01622469	6133	8.196240E-01
Il4ra	21522	0.026810865	6919	7.062821E-01
Il5	21523	0.04447041	7896	5.317913E-01
Il5ra	21524	0.007627507	5662	9.146345E-01
Il6	21525	0.002889175	5468.5	9.676123E-01
Il6ra	21526	0.04250173	7774	5.501283E-01
Il6st	21527	0.15941569	16250	2.414675E-02
Il7	21528	0.074913331	10230	2.917565E-01
Il7r	21529	-0.036220745	3678	6.106155E-01
Il8	21530	-0.001989505	5076	9.776944E-01
Il9	21531	-0.054450825	1984	4.437997E-01
Il9r	21532	0.01672612	6159	8.141491E-01
Ildr1	21533	0.070146974	9850	3.236297E-01
Ildr2	21534	0.116124916	13402	1.015250E-01
Ilf2	21535	0.056686412	9022	4.252865E-01
Ilf3	21536	0.222478978	18969	1.543459E-03
Ilk	21537	0.081182346	10681	2.531288E-01
Ilkap	21538	0.169500568	16901	1.641913E-02
Iltifb	21539	0.049901454	8291	4.828494E-01
Ilvbl	21540	-0.002317262	5056	9.740210E-01
Immp1l	21541	0.061482508	9299	3.871143E-01
Immp2l	21542	0.123657953	13882	8.106709E-02

Spearman Rank correlation analysis performed between Prdm1 and all-expressed genes within the Meredith RNA-seq dataset. Robust Prdm1–associated genes were identified using a cut-off of $p < 0.0005$.

Table S1, Related to Supplemental Figure 3C. Prdm1 associated genes

Immt	21543	0.116532256	13436	1.003232E-01
Imp3	21544	0.103781166	12284	1.436208E-01
Imp4	21545	0.144987745	15353	4.051880E-02
Impa1	21546	0.143487898	15160	4.266168E-02
Impa2	21547	0.076017683	10348	2.846811E-01
Impact	21548	0.073440269	10090	3.013753E-01
Impad1	21549	0.148700968	15575	3.560013E-02
Impdh1	21550	0.089802134	11329	2.060183E-01
Impdh2	21551	0.228253427	19102	1.150893E-03
Impg1	21552	-0.002645692	5044	9.703405E-01
Impg2	21553	0.016769042	6161	8.136809E-01
Ina	21554	0.074574898	10201.5	2.939481E-01
Inadl	21555	0.086376449	11075	2.239221E-01
Inca1	21556	0.093705288	11626	1.869044E-01
Incenp	21557	0.170381695	16951	1.585999E-02
Inf2	21558	-0.01052833	4727	8.823713E-01
Ing1	21559	0.083064615	10839	2.422546E-01
Ing2	21560	-0.034742828	3720	6.252626E-01
Ing3	21561	0.240547695	19369	6.012411E-04
Ing4	21562	0.011393556	5846	8.727830E-01
Ing5	21563	0.179097826	17363	1.116472E-02
Inha	21564	0.049151123	8227	4.894632E-01
Inhba	21565	0.097397819	11865.5	1.700498E-01
Inhbb	21566	-0.00121545	5175	9.863717E-01
Inhbc	21567	0.11540986	13351	1.036620E-01
Inhbe	21568	-0.054450825	1984	4.437997E-01
Inip	21569	0.097237982	11855	1.707551E-01
Inmt	21570	0.113548679	13094	1.093908E-01
Ino80	21571	0.222869955	18984	1.513437E-03
Ino80b	21572	-0.000363323	5268	9.959261E-01
Ino80c	21573	-0.019485247	4253	7.841909E-01
Ino80d	21574	0.217096253	18820	2.015949E-03
Ino80e	21575	0.037188417	7443	6.011079E-01
Inpp1	21576	0.176036794	17224	1.265139E-02
Inpp4a	21577	0.078153924	10474	2.713239E-01
Inpp4b	21578	0.179271581	17370	1.108516E-02
Inpp5a	21579	0.167907132	16816	1.747401E-02
Inpp5b	21580	-0.010537821	4725	8.822660E-01
Inpp5d	21581	-0.012505308	4646	8.604903E-01
Inpp5e	21582	0.00366409	5502	9.589323E-01
Inpp5f	21583	0.073099576	10070	3.036295E-01
Inpp5j	21584	0.067446588	9669	3.426505E-01
Inpp5k	21585	-0.05406584	2111	4.470333E-01

Spearman Rank correlation analysis performed between Prdm1 and all-expressed genes within the Meredith RNA-seq dataset. Robust Prdm1–associated genes were identified using a cut-off of $p < 0.0005$.

Table S1, Related to Supplemental Figure 3C. Prdm1 associated genes

Inppl1	21586	-0.031444509	3870	6.584787E-01
Ins1	21587	-0.038406171	3065	5.892384E-01
Ins2	21588	0.066934503	9639	3.463360E-01
Insc	21589	0.044265616	7883	5.336844E-01
Insig1	21590	0.191597136	17898	6.571072E-03
Insig2	21591	0.242976492	19414	5.267573E-04
Insl3	21592		#N/A	1.000000E+00
Insl5	21593	0.157799267	16153.5	2.563935E-02
Insl6	21594	0.123898088	13898	8.047408E-02
Insm1	21595	0.055969667	8982	4.311727E-01
Insm2	21596	0.276998777	19774	7.163801E-05
Insr	21597	0.118384553	13551	9.499997E-02
Insrr	21598	-0.019476419	4255.5	7.842862E-01
Ints1	21599	0.033493558	7266	6.377596E-01
Ints10	21600	0.104015789	12297	1.427126E-01
Ints12	21601	0.162301912	16484	2.166707E-02
Ints2	21602	0.116388198	13425	1.007469E-01
Ints3	21603	0.21374881	18709	2.372707E-03
Ints4	21604	0.017359205	6194	8.072495E-01
Ints5	21605	0.16337423	16565	2.080354E-02
Ints6	21606	0.055034035	8928	4.389265E-01
Ints7	21607	0.185001439	17637	8.726322E-03
Ints8	21608	0.091702229	11503	1.965444E-01
Ints9	21609	0.044511468	7903	5.314121E-01
Intu	21610	0.101889727	12173	1.511033E-01
Invs	21611	0.185020627	17640	8.719236E-03
Ip6k1	21612	0.067873792	9695	3.395951E-01
Ip6k2	21613	0.09720128	11854	1.709174E-01
Ip6k3	21614	-0.020846058	4210	7.695285E-01
Ipcef1	21615	0.045706233	7983	5.204394E-01
Ipmk	21616	0.27947289	19789	6.130751E-05
Ipo11	21617	0.127635534	14163	7.168735E-02
Ipo13	21618	-0.124240886	89	7.963362E-02
Ipo4	21619	0.0595593	9188	4.021654E-01
Ipo5	21620	0.258980331	19616	2.131027E-04
Ipo7	21621	-0.026107704	4064	7.136436E-01
Ipo8	21622	0.057610533	9065	4.177664E-01
Ipo9	21623	0.171345742	16988	1.526733E-02
Ipp	21624	0.149692286	15630	3.437591E-02
Ippk	21625	0.234153934	19260	8.462801E-04
Iqca	21626	0.024211531	6764	7.336258E-01
Iqcb1	21627	0.152958375	15839	3.059200E-02
Iqcc	21628	0.038001878	7483	5.931671E-01

Spearman Rank correlation analysis performed between Prdm1 and all-expressed genes within the Meredith RNA-seq dataset. Robust Prdm1–associated genes were identified using a cut-off of $p < 0.0005$.

Table S1, Related to Supplemental Figure 3C. Prdm1 associated genes

Iqcd	21629	0.078162402	10476	2.712717E-01
Iqce	21630	0.129940414	14295	6.666892E-02
Iqcf1	21631		#N/A	1.000000E+00
Iqcf3	21632	-0.066858259	1029.5	3.468868E-01
Iqcf4	21633		#N/A	1.000000E+00
Iqcf5	21634		#N/A	1.000000E+00
Iqcf6	21635		#N/A	1.000000E+00
Iqcg	21636	0.023646334	6729	7.396174E-01
Iqch	21637	0.053031048	8596	4.557904E-01
Iqcj	21638	-0.067524471	971	3.420922E-01
Iqck	21639	-0.057284478	1450	4.204107E-01
Iqgap1	21640	0.306907293	19930	9.857943E-06
Iqgap2	21641	0.141421848	15044	4.576855E-02
Iqgap3	21642	0.072584795	10035	3.070565E-01
Iqsec1	21643	0.216320029	18795	2.094024E-03
Iqsec2	21644	-0.010802581	4709	8.793302E-01
Iqsec3	21645	0.105186936	12372.5	1.382448E-01
Iqub	21646	-0.077394997	736.5	2.760195E-01
Irak1	21647	0.113602032	13232	1.092232E-01
Irak1bp1	21648	0.274524696	19752	8.358591E-05
Irak2	21649	0.183568401	17574	9.270205E-03
Irak3	21650	-0.070184319	946	3.233715E-01
Irak4	21651	0.165184723	16668	1.941305E-02
Ireb2	21652	0.050204681	8315	4.801903E-01
Irf1	21653	0.059899399	9204	3.994787E-01
Irf2	21654	0.067378475	9666	3.431393E-01
Irf2bp1	21655	0.097696981	11880	1.687355E-01
Irf2bp2	21656	0.084589863	10959	2.336855E-01
Irf2bpl	21657	0.173012353	17081	1.428833E-02
Irf3	21658	0.10438242	12316	1.413023E-01
Irf4	21659	0.178777223	17342	1.131286E-02
Irf5	21660	0.084048198	10923	2.367039E-01
Irf6	21661	0.026355823	6891	7.110430E-01
Irf7	21662	0.166088484	16710	1.874961E-02
Irf8	21663		#N/A	1.000000E+00
Irf9	21664	0.063940743	9466	3.683802E-01
Irg1	21665	0.040402371	7655	5.700170E-01
Irgc1	21666		#N/A	1.000000E+00
Irgm1	21667	0.078584301	10502	2.686853E-01
Irgm2	21668	0.092897914	11574	1.907474E-01
Irgq	21669	0.018788391	6265	7.917292E-01
Irs1	21670	0.139198142	14903	4.932163E-02
Irs2	21671	0.02022543	6340	7.762058E-01

Spearman Rank correlation analysis performed between Prdm1 and all-expressed genes within the Meredith RNA-seq dataset. Robust Prdm1–associated genes were identified using a cut-off of $p < 0.0005$.

Table S1, Related to Supplemental Figure 3C. Prdm1 associated genes

Irs3	21672	-0.019133303	4265.5	7.879956E-01
Irs4	21673		#N/A	1.000000E+00
Irx1	21674	-0.038406171	3065	5.892384E-01
Irx2	21675	-0.066855996	1239.5	3.469032E-01
Irx3	21676	0.114441026	13287	1.066138E-01
Irx4	21677	-0.042549465	2421	5.496801E-01
Irx5	21678	0.08295169	10828	2.428977E-01
Irx6	21679	0.014759354	6057	8.356727E-01
Isca1	21680	0.201449134	18307	4.230432E-03
Isca2	21681	0.135852324	14686	5.509815E-02
Iscu	21682	0.101262639	12136	1.536480E-01
Isg15	21683	0.160117327	16283	2.352250E-02
Isg20	21684	0.143891679	15305	4.207563E-02
Isg20l2	21685	0.064256902	9489	3.660121E-01
Isl1	21686	0.12571298	14017	7.610453E-02
Isl2	21687	-0.016730883	4441	8.140971E-01
Islr	21688	-0.028518853	3979	6.885151E-01
Islr2	21689	0.109930598	12709.5	1.212354E-01
Ism1	21690	0.062962713	9389.5	3.757656E-01
Ism2	21691	-0.159702875	10	2.388954E-02
Isoc1	21692	0.155080818	15986	2.832828E-02
Isoc2a	21693	0.081495676	10700	2.512956E-01
Isoc2b	21694	0.04774701	8125	5.019687E-01
Ispd	21695	0.024955505	6813	7.257634E-01
Ist1	21696	0.229050571	19113	1.104565E-03
Isx	21697	-0.038406171	3065	5.892384E-01
Isy1	21698	0.129554986	14275	6.748754E-02
Isyna1	21699	0.078445208	10494	2.695361E-01
Itch	21700	0.261923232	19648	1.792791E-04
Itfg1	21701	0.120778437	13709	8.845568E-02
Itfg2	21702	0.015918937	6119	8.229668E-01
Itfg3	21703	0.047150168	8088	5.073348E-01
Itga1	21704	0.006560648	5617	9.265383E-01
Itga10	21705		#N/A	1.000000E+00
Itga11	21706	0.081911793	10738	2.488753E-01
Itga2	21707	0.150057962	15656	3.393343E-02
Itga2b	21708	0.065591389	9566	3.561207E-01
Itga3	21709	0.053034478	8597	4.557612E-01
Itga4	21710	0.058303583	9109	4.121780E-01
Itga5	21711	0.042423157	7767	5.508666E-01
Itga6	21712	0.087824486	11189	2.162239E-01
Itga7	21713	-0.066855996	1239.5	3.469032E-01
Itga8	21714	0.084753533	10969	2.327788E-01

Spearman Rank correlation analysis performed between Prdm1 and all-expressed genes within the Meredith RNA-seq dataset. Robust Prdm1–associated genes were identified using a cut-off of $p < 0.0005$.

Table S1, Related to Supplemental Figure 3C. Prdm1 associated genes

Itga9	21715	-0.016283528	4461	8.189812E-01
Itgad	21716	0.123271483	13863	8.202885E-02
Itgae	21717	0.01033243	5787	8.845446E-01
Itgal	21718	0.09643514	11804	1.743309E-01
Itgam	21719	0.094517948	11674	1.830937E-01
Itgav	21720	0.089688839	11321	2.065934E-01
Itgax	21721	0.022120944	6596	7.558656E-01
Itgb1	21722	0.197776096	18157	4.996989E-03
Itgb1bp1	21723	0.151332958	15736	3.242824E-02
Itgb1bp2	21724		#N/A	1.000000E+00
Itgb2	21725	0.168020852	16824	1.739682E-02
Itgb2l	21726	0.052728522	8550.5	4.583686E-01
Itgb3	21727	0.186721709	17706	8.110938E-03
Itgb3bp	21728	0.172604686	17054	1.452258E-02
Itgb4	21729	0.061496483	9301	3.870062E-01
Itgb5	21730	0.007182901	5636	9.195932E-01
Itgb6	21731	0.174227594	17134	1.360955E-02
Itgb7	21732	0.085373796	11013	2.293652E-01
Itgb8	21733	0.019928633	6331	7.794048E-01
Itgbl1	21734	0.124138289	13919	7.988442E-02
Itih1	21735	-0.089747066	418	2.062977E-01
Itih2	21736	-0.110565275	170	1.190886E-01
Itih3	21737	-0.077393517	797	2.760287E-01
Itih4	21738	0.121676221	13772	8.609638E-02
Itih5	21739	0.076634683	10388	2.807786E-01
Itih5l-ps	21740	-0.038406171	3065	5.892384E-01
Itk	21741	-0.077393517	797	2.760287E-01
Itln1	21742	0.114076322	13256	1.077420E-01
Itm2a	21743	0.167921687	16817	1.746412E-02
Itm2b	21744	0.178703346	17338	1.134724E-02
Itm2c	21745	0.107864698	12546	1.284319E-01
Itpa	21746	0.070803659	9898	3.191094E-01
Itpa-ps1	21747		#N/A	1.000000E+00
Itpa-ps3	21748		#N/A	1.000000E+00
Itpk1	21749	0.12166502	13771	8.612551E-02
Itpka	21750		#N/A	1.000000E+00
Itpkb	21751	0.013283999	5949	8.518999E-01
Itpkc	21752	0.020875719	6412.5	7.692098E-01
Itpr1	21753	0.060826545	9251	3.922091E-01
Itpr2	21754	0.120853346	13716	8.825687E-02
Itpr3	21755	0.139132561	14899	4.942981E-02
Itprip	21756		#N/A	1.000000E+00
Itpripl1	21757	0.209988713	18572	2.841148E-03

Spearman Rank correlation analysis performed between Prdm1 and all-expressed genes within the Meredith RNA-seq dataset. Robust Prdm1–associated genes were identified using a cut-off of $p < 0.0005$.

Table S1, Related to Supplemental Figure 3C. Prdm1 associated genes

Itpripl2	21758	0.15085442	15706	3.298639E-02
Itsn1	21759	0.237148068	19313	7.219079E-04
Itsn2	21760	0.188785402	17788	7.423787E-03
Ivd	21761	-0.0275497	4011	6.985763E-01
Ivl	21762	0.046678342	8059	5.115979E-01
Ivns1abp	21763	0.1393784	14914	4.902530E-02
Iws1	21764	0.16233739	16486	2.163801E-02
Iyd	21765	-0.077393023	832.5	2.760318E-01
Izumo1	21766	0.127861054	14181	7.118315E-02
Izumo2	21767		#N/A	1.000000E+00
Izumo3	21768		#N/A	1.000000E+00
Izumo4	21769	-0.016730797	4444.5	8.140981E-01
Jag1	21770	0.085801643	11043	2.270312E-01
Jag2	21771	0.209357601	18561	2.927519E-03
Jagn1	21772	0.140835567	15011	4.668389E-02
Jak1	21773	0.249195458	19497	3.731670E-04
Jak2	21774	0.194312925	18012	5.831578E-03
Jak3	21775	0.056137524	8994	4.297900E-01
Jakmip1	21776	0.32674526	19981	2.333411E-06
Jakmip2	21777	0.140809638	15010	4.672472E-02
Jakmip3	21778	0.053267784	8685	4.537786E-01
Jam2	21779	0.15431986	15913.5	2.912285E-02
Jam3	21780	0.056497014	9007	4.268373E-01
Jarid2	21781	-0.03966069	2476	5.771239E-01
Jazf1	21782	0.106263376	12443	1.342333E-01
Jdp2	21783	0.011143476	5835	8.755525E-01
Jhdm1d	21784	0.22521289	19052	1.344444E-03
Jkamp	21785	0.197932783	18165	4.961900E-03
Jmjd1c	21786	0.191475594	17894	6.606041E-03
Jmjd4	21787	-0.027521433	4012	6.988706E-01
Jmjd6	21788	0.013571951	5973	8.487277E-01
Jmjd8	21789		#N/A	1.000000E+00
Jmy	21790	0.102026257	12182	1.505535E-01
Josd1	21791	0.171339009	16987	1.527140E-02
Josd2	21792	0.097404869	11868	1.700187E-01
Jph1	21793	-0.020501916	4221	7.732290E-01
Jph2	21794		#N/A	1.000000E+00
Jph3	21795	0.192785357	17943	6.237803E-03
Jph4	21796	-0.030875227	3902	6.642825E-01
Jpx	21797	0.165050462	16665	1.951332E-02
Jrk	21798	-0.098869215	286	1.636583E-01
Jrkl	21799	0.001683721	5404	9.811221E-01
Jsrp1	21800		#N/A	1.000000E+00

Spearman Rank correlation analysis performed between Prdm1 and all-expressed genes within the Meredith RNA-seq dataset. Robust Prdm1–associated genes were identified using a cut-off of $p < 0.0005$.

Table S1, Related to Supplemental Figure 3C. Prdm1 associated genes

Jtb	21801	0.11545349	13354	1.035306E-01
Jun	21802	0.129331126	14263	6.796676E-02
Junb	21803	0.046127707	8019	5.165965E-01
Jund	21804	0.165848162	16692	1.892408E-02
Jup	21805	0.054159829	8875	4.462427E-01
Kalrn	21806	0.090030492	11347	2.048626E-01
Kank1	21807	-0.013659192	4580	8.477670E-01
Kank2	21808	0.057757995	9072	4.165736E-01
Kank3	21809	0.177787186	17303	1.178130E-02
Kank4	21810	-0.043397509	2394.5	5.417465E-01
Kansl1	21811	0.142602856	15123	4.397021E-02
Kansl1l	21812	0.109707025	12693	1.219988E-01
Kansl2	21813	0.024693952	6798	7.285243E-01
Kansl3	21814	0.112947282	12922	1.112941E-01
Kap	21815	-0.038406171	3065	5.892384E-01
Kars	21816	0.007836333	5672	9.123066E-01
Kars-ps1	21817		#N/A	1.000000E+00
Kat2a	21818	-0.007604038	4835	9.148962E-01
Kat2b	21819	0.167539457	16800	1.772563E-02
Kat2b-ps	21820		#N/A	1.000000E+00
Kat5	21821	0.019879439	6329	7.799355E-01
Kat6a	21822	0.156382082	16076	2.701247E-02
Kat6b	21823	0.132349786	14438	6.173373E-02
Kat7	21824	0.110245038	12732	1.201680E-01
Kat8	21825	-0.097203805	299	1.709062E-01
Katna1	21826	0.141993179	15081	4.489104E-02
Katnal1	21827	0.062456123	9350	3.796265E-01
Katnal2	21828	0.027169996	6936.5	7.025327E-01
Katnb1	21829	0.131683792	14383	6.306691E-02
Katnbl1	21830	0.201584155	18314	4.204388E-03
Kazald1	21831	0.109785234	12700	1.217313E-01
Kazn	21832	0.060648351	9241	3.936001E-01
Kbtbd11	21833	0.063785513	9457	3.695464E-01
Kbtbd12	21834	0.212474793	18672.5	2.522920E-03
Kbtbd13	21835	0.143605682	15224	4.249002E-02
Kbtbd2	21836	0.153628284	15872	2.986141E-02
Kbtbd3	21837	0.120329844	13681	8.965378E-02
Kbtbd4	21838	-0.019610903	4250	7.828337E-01
Kbtbd7	21839	0.145558815	15381	3.972706E-02
Kbtbd8	21840	0.160304018	16323	2.335876E-02
Kcmf1	21841	0.098475438	11953	1.653509E-01
Kcna1	21842	0.091532011	11489	1.973800E-01
Kcna10	21843		#N/A	1.000000E+00

Spearman Rank correlation analysis performed between Prdm1 and all-expressed genes within the Meredith RNA-seq dataset. Robust Prdm1–associated genes were identified using a cut-off of $p < 0.0005$.

Table S1, Related to Supplemental Figure 3C. Prdm1 associated genes

Kcna2	21844		#N/A	1.000000E+00
Kcna3	21845		#N/A	1.000000E+00
Kcna4	21846	-0.038406171	3065	5.892384E-01
Kcna5	21847	0.080437091	10628	2.575261E-01
Kcna6	21848	-0.054450825	1984	4.437997E-01
Kcna7	21849	-0.054450825	1984	4.437997E-01
Kcnab1	21850	-0.001013269	5182	9.886385E-01
Kcnab2	21851	0.081878104	10735	2.490706E-01
Kcnab3	21852	-0.038406171	3065	5.892384E-01
Kcnb1	21853	0.008453583	5703	9.054303E-01
Kcnb2	21854	-0.170016722	5	1.608954E-02
Kcnc1	21855	0.033780247	7284	6.348825E-01
Kcnc2	21856	0.198535432	18184	4.828994E-03
Kcnc3	21857	0.196346019	18099	5.327678E-03
Kcnc4	21858	0.013458335	5965.5	8.499790E-01
Kcnd1	21859	-0.045728293	2300	5.202379E-01
Kcnd2	21860	0.168391356	16843	1.714738E-02
Kcnd3	21861	0.141409873	15041	4.578710E-02
Kcne1	21862	-0.03367206	3757.5	6.359676E-01
Kcne1l	21863	0.053267784	8685	4.537786E-01
Kcne2	21864	0.113548679	13094	1.093908E-01
Kcne3	21865		#N/A	1.000000E+00
Kcne4	21866		#N/A	1.000000E+00
Kcnf1	21867	-0.017680437	4382	8.037543E-01
Kcng1	21868	-0.066856562	1139	3.468991E-01
Kcng2	21869	-0.015245563	4499	8.303397E-01
Kcng3	21870	-0.004937246	4940	9.446824E-01
Kcng4	21871	-0.054450825	1984	4.437997E-01
Kcnh1	21872	0.182469449	17527	9.707415E-03
Kcnh2	21873		#N/A	1.000000E+00
Kcnh3	21874	0.111057495	12792	1.174440E-01
Kcnh4	21875	-0.038406171	3065	5.892384E-01
Kcnh5	21876	-0.030530444	3912	6.678072E-01
Kcnh6	21877	0.113435011	12953	1.097485E-01
Kcnh7	21878	0.004412567	5535	9.505531E-01
Kcnh8	21879	-0.106662315	201	1.327695E-01
Kcnip1	21880	0.192723005	17941	6.254908E-03
Kcnip2	21881	0.131075152	14357	6.430585E-02
Kcnip3	21882	0.060939882	9258	3.913260E-01
Kcnip4	21883	0.183830158	17585	9.168674E-03
Kcnj1	21884	0.012925476	5932.5	8.558530E-01
Kcnj10	21885	0.164080385	16597	2.025127E-02
Kcnj11	21886		#N/A	1.000000E+00

Spearman Rank correlation analysis performed between Prdm1 and all-expressed genes within the Meredith RNA-seq dataset. Robust Prdm1–associated genes were identified using a cut-off of $p < 0.0005$.

Table S1, Related to Supplemental Figure 3C. Prdm1 associated genes

Kcnj12	21887	0.076051619	10351.5	2.844655E-01
Kcnj13	21888		#N/A	1.000000E+00
Kcnj14	21889	0.113548679	13094	1.093908E-01
Kcnj15	21890	0.100315976	12080	1.575506E-01
Kcnj16	21891	0.090112489	11358	2.044487E-01
Kcnj2	21892	0.047553918	8118	5.037015E-01
Kcnj3	21893	-0.002893582	5029	9.675630E-01
Kcnj4	21894		#N/A	1.000000E+00
Kcnj5	21895	0.04927978	8243	4.883258E-01
Kcnj6	21896	0.095378147	11728	1.791228E-01
Kcnj8	21897	-0.077393517	797	2.760287E-01
Kcnj9	21898	0.143605682	15224	4.249002E-02
Kcnk1	21899	-0.130662507	59	6.515711E-02
Kcnk10	21900	0.13842601	14852	5.060787E-02
Kcnk12	21901	0.053267784	8685	4.537786E-01
Kcnk13	21902	-0.052956803	2141.5	4.564224E-01
Kcnk15	21903		#N/A	1.000000E+00
Kcnk16	21904		#N/A	1.000000E+00
Kcnk18	21905	-0.038406171	3065	5.892384E-01
Kcnk2	21906	0.072446339	10027	3.079826E-01
Kcnk3	21907	0.151693558	15749	3.201298E-02
Kcnk4	21908		#N/A	1.000000E+00
Kcnk5	21909	-0.066856562	1139	3.468991E-01
Kcnk6	21910	0.091075017	11444	1.996360E-01
Kcnk7	21911		#N/A	1.000000E+00
Kcnk9	21912	0.135737051	14674	5.530674E-02
Kcnma1	21913	0.030473645	7096	6.683886E-01
Kcnmb1	21914	0.049855249	8285.5	4.832553E-01
Kcnmb2	21915	0.018577752	6256	7.940117E-01
Kcnmb3	21916	0.143605682	15224	4.249002E-02
Kcnmb4	21917	0.025109852	6829	7.241358E-01
Kcnn1	21918	0.07679841	10402	2.797492E-01
Kcnn2	21919	0.110303596	12739	1.199701E-01
Kcnn3	21920	0.051021642	8388	4.730658E-01
Kcnn4	21921	0.181396826	17461	1.015164E-02
Kcnq1	21922	0.216690421	18811	2.056432E-03
Kcnq2	21923	0.001542776	5393	9.827021E-01
Kcnq3	21924	0.158955193	16222	2.456416E-02
Kcnq4	21925	0.053267784	8685	4.537786E-01
Kcnq5	21926	0.11898852	13587	9.331364E-02
Kcnrg	21927		#N/A	1.000000E+00
Kcns1	21928	0.081437712	10695.5	2.516340E-01
Kcns2	21929		#N/A	1.000000E+00

Spearman Rank correlation analysis performed between Prdm1 and all-expressed genes within the Meredith RNA-seq dataset. Robust Prdm1–associated genes were identified using a cut-off of $p < 0.0005$.

Table S1, Related to Supplemental Figure 3C. Prdm1 associated genes

Kcns3	21930	0.175556251	17191	1.289992E-02
Kcnt1	21931	-0.110561318	183	1.191018E-01
Kcnt2	21932	0.018638001	6259	7.933586E-01
Kcnu1	21933	-0.056165168	1479.5	4.295625E-01
Kcnv1	21934	-0.038406171	3065	5.892384E-01
Kcnv2	21935	0.087473475	11162	2.180723E-01
Kcp	21936		#N/A	1.000000E+00
Kctd1	21937	0.12391731	13900	8.042676E-02
Kctd10	21938	0.160272993	16290	2.338590E-02
Kctd11	21939	-0.005523476	4900	9.381266E-01
Kctd12	21940	-0.105385025	205	1.374998E-01
Kctd12b	21941	0.047714834	8123	5.022573E-01
Kctd13	21942	-0.110581467	154.5	1.190342E-01
Kctd14	21943	0.086735788	11116	2.219938E-01
Kctd15	21944	0.025224402	6840	7.229287E-01
Kctd16	21945	0.007176943	5634	9.196597E-01
Kctd17	21946	-0.054450825	1984	4.437997E-01
Kctd18	21947	0.004801241	5549	9.462039E-01
Kctd19	21948	0.031318251	7145	6.597641E-01
Kctd2	21949	0.18948198	17822	7.203830E-03
Kctd20	21950	0.007688707	5666	9.139522E-01
Kctd21	21951	-0.066141792	1306	3.520902E-01
Kctd3	21952	-0.070328947	944	3.223730E-01
Kctd4	21953	0.059761445	9198	4.005672E-01
Kctd5	21954	0.071476705	9949	3.145193E-01
Kctd6	21955	-0.016538996	4457	8.161912E-01
Kctd7	21956	0.110814925	12779	1.182522E-01
Kctd8	21957	0.022908516	6686	7.474626E-01
Kctd9	21958	0.132011625	14411	6.240773E-02
Kdelc1	21959	0.1296362	14280	6.731437E-02
Kdelc2	21960	0.077960379	10466	2.725162E-01
Kdelr1	21961	0.183509894	17570	9.293035E-03
Kdelr2	21962	0.203711494	18377	3.812672E-03
Kdelr3	21963	0.092058516	11521	1.948038E-01
Kdm1a	21964	0.154318781	15912	2.912399E-02
Kdm1b	21965	0.069922841	9839	3.251820E-01
Kdm2a	21966	0.133275426	14498	5.991932E-02
Kdm2b	21967	0.19611343	18089	5.383278E-03
Kdm3a	21968	0.070125584	9847	3.237776E-01
Kdm3b	21969	0.156011046	16039	2.738221E-02
Kdm4a	21970	0.06714591	9653	3.448115E-01
Kdm4b	21971	-0.096433027	306	1.743404E-01
Kdm4c	21972	0.075202736	10257	2.898911E-01

Spearman Rank correlation analysis performed between Prdm1 and all-expressed genes within the Meredith RNA-seq dataset. Robust Prdm1–associated genes were identified using a cut-off of $p < 0.0005$.

Table S1, Related to Supplemental Figure 3C. Prdm1 associated genes

Kdm4d	21973	0.038273027	7513	5.905309E-01
Kdm5a	21974	0.17650374	17243	1.241392E-02
Kdm5b	21975	0.132010638	14410	6.240971E-02
Kdm5c	21976	0.149770329	15636	3.428107E-02
Kdm5d	21977	0.053267784	8685	4.537786E-01
Kdm6a	21978	0.187925884	17758	7.703423E-03
Kdm6b	21979	0.119795954	13652	9.109653E-02
Kdm8	21980	0.112455116	12886	1.128709E-01
Kdr	21981	0.288392388	19857	3.454158E-05
Kdsr	21982	0.180259436	17416	1.064222E-02
Keap1	21983	-0.045796408	2295	5.196160E-01
Keg1	21984	-0.054451513	1703	4.437940E-01
Kel	21985		#N/A	1.000000E+00
Kera	21986	0.143242796	15151	4.302075E-02
Khdc1a	21987		#N/A	1.000000E+00
Khdc1b	21988		#N/A	1.000000E+00
Khdc1c	21989		#N/A	1.000000E+00
Khdc3	21990	-0.054450825	1984	4.437997E-01
Khdrbs1	21991	0.200554346	18264	4.406724E-03
Khdrbs2	21992	0.231690674	19205	9.630743E-04
Khdrbs3	21993	-0.125004012	77	7.778803E-02
Khk	21994	0.107134311	12505	1.310535E-01
Khnyn	21995	0.173941263	17119	1.376688E-02
Khsrp	21996	0.200310879	18251	4.455823E-03
Kidins220	21997	0.188371844	17776	7.557186E-03
Kif11	21998	0.173173558	17088	1.419661E-02
Kif12	21999		#N/A	1.000000E+00
Kif13a	22000	0.07809032	10470	2.717153E-01
Kif13b	22001	0.03419007	7302	6.307792E-01
Kif14	22002	0.164249148	16606	2.012118E-02
Kif15	22003	0.290697583	19867	2.968715E-05
Kif16b	22004	0.106593616	12465	1.330207E-01
Kif17	22005	-0.000841282	5208.5	9.905669E-01
Kif18a	22006	-0.014915985	4514	8.339539E-01
Kif18b	22007	0.112018769	12857	1.142835E-01
Kif19a	22008	0.088616827	11243	2.120925E-01
Kif1a	22009	0.176329384	17239	1.250213E-02
Kif1b	22010	0.200049398	18243	4.509101E-03
Kif1c	22011	0.135350025	14651	5.601186E-02
Kif20a	22012	0.25520023	19567	2.652899E-04
Kif20b	22013	0.304728619	19919	1.147661E-05
Kif21a	22014	0.027551888	6952	6.985535E-01
Kif21b	22015	0.073458634	10093	3.012541E-01

Spearman Rank correlation analysis performed between Prdm1 and all-expressed genes within the Meredith RNA-seq dataset. Robust Prdm1–associated genes were identified using a cut-off of $p < 0.0005$.

Table S1, Related to Supplemental Figure 3C. Prdm1 associated genes

Kif22	22016	0.173114411	17085	1.423020E-02
Kif22-ps	22017		#N/A	1.000000E+00
Kif23	22018	0.122565808	13820	8.380866E-02
Kif24	22019	0.144927267	15352	4.060342E-02
Kif26a	22020	0.195521665	18063	5.527090E-03
Kif26b	22021	-0.055042703	1522	4.388543E-01
Kif27	22022	-0.000841282	5208.5	9.905669E-01
Kif2a	22023	0.051309304	8414	4.705710E-01
Kif2b	22024	0.113548679	13094	1.093908E-01
Kif2c	22025	0.031161085	7137	6.613656E-01
Kif3a	22026	0.191212523	17885	6.682301E-03
Kif3b	22027	0.052132152	8480	4.634746E-01
Kif3c	22028	0.061868611	9327	3.841342E-01
Kif4	22029	0.152041229	15773	3.161689E-02
Kif4-ps	22030		#N/A	1.000000E+00
Kif5a	22031	0.01816215	6232	7.985202E-01
Kif5b	22032	0.143800536	15298	4.220732E-02
Kif5c	22033	0.146528521	15449	3.841249E-02
Kif6	22034	0.092533317	11551	1.925017E-01
Kif7	22035	0.018781238	6264	7.918067E-01
Kif9	22036	0.101704315	12164	1.518523E-01
Kifap3	22037	0.142529356	15116	4.408037E-02
Kifc1	22038	-0.095269074	345	1.796228E-01
Kifc2	22039	0.049429886	8250	4.870004E-01
Kifc3	22040	0.071728442	9971	3.128136E-01
Kifc5b	22041	0.185942386	17678	8.384778E-03
Kifc5c-ps	22042		#N/A	1.000000E+00
Kin	22043	0.055081658	8931	4.385300E-01
Kir3dl1	22044	-0.038406171	3065	5.892384E-01
Kir3dl2	22045	0.052728522	8550.5	4.583686E-01
Kirrel	22046	0.070711587	9890	3.197407E-01
Kirrel2	22047	0.225488562	19060	1.325739E-03
Kirrel3	22048	-0.055421109	1507	4.357091E-01
Kis2	22049		#N/A	1.000000E+00
Kiss1	22050	0.182294195	17509.5	9.778799E-03
Kiss1r	22051		#N/A	1.000000E+00
Kit	22052	0.028521742	6996	6.884852E-01
Kitl	22053	0.01707371	6175	8.103591E-01
Kl	22054	0.183200677	17557	9.414520E-03
Klb	22055	-0.038406171	3065	5.892384E-01
Klc1	22056	-0.037782244	3638	5.953064E-01
Klc2	22057	-0.005909836	4882	9.338082E-01
Klc3	22058	0.098665179	11969	1.645337E-01

Spearman Rank correlation analysis performed between Prdm1 and all-expressed genes within the Meredith RNA-seq dataset. Robust Prdm1–associated genes were identified using a cut-off of $p < 0.0005$.

Table S1, Related to Supplemental Figure 3C. Prdm1 associated genes

Klc4	22059	-0.098159135	293	1.667200E-01
Klf1	22060	0.174550711	17147	1.343390E-02
Klf10	22061	0.023619242	6728	7.399050E-01
Klf11	22062	-0.078736128	658	2.677586E-01
Klf12	22063	0.092962076	11580	1.904399E-01
Klf13	22064	0.160889741	16382	2.285140E-02
Klf14	22065	0.150467275	15679	3.344389E-02
Klf15	22066	-0.038406171	3065	5.892384E-01
Klf16	22067	0.145754035	15396	3.945941E-02
Klf17	22068	0.18817735	17767	7.620657E-03
Klf2	22069	0.165667117	16687	1.905644E-02
Klf3	22070	0.098168578	11927	1.666790E-01
Klf4	22071	0.221037074	18933	1.658941E-03
Klf5	22072	0.205967934	18457	3.433430E-03
Klf6	22073	0.134354056	14583	5.786063E-02
Klf7	22074	0.054904964	8916	4.400024E-01
Klf8	22075	0.013800383	5979	8.462128E-01
Klf9	22076	0.137558261	14794	5.208655E-02
Klhdc1	22077	0.063938373	9465	3.683980E-01
Klhdc10	22078	0.01595164	6121	8.226091E-01
Klhdc2	22079	0.16280313	16519	2.125966E-02
Klhdc3	22080	0.096155043	11785	1.755914E-01
Klhdc4	22081	0.089057262	11276	2.098206E-01
Klhdc7a	22082	-0.003041262	5023	9.659084E-01
Klhdc7b	22083		#N/A	1.000000E+00
Klhdc8a	22084	0.061457467	9297	3.873080E-01
Klhdc8b	22085	-0.0595331	1405	4.023728E-01
Klhdc9	22086	0.180331819	17422	1.061039E-02
Klhl1	22087	0.153863276	15883	2.960868E-02
Klhl10	22088	-0.018790474	4278.5	7.917066E-01
Klhl11	22089	-0.077393023	832.5	2.760318E-01
Klhl12	22090	-0.063166931	1357	3.742161E-01
Klhl13	22091	0.131680025	14382	6.307452E-02
Klhl14	22092	0.222098274	18959	1.573213E-03
Klhl15	22093	0.107097769	12503	1.311858E-01
Klhl17	22094	-0.030153016	3921	6.716741E-01
Klhl18	22095	0.085718324	11038	2.274844E-01
Klhl2	22096	0.175608343	17195	1.287278E-02
Klhl20	22097	0.09745401	11870	1.698024E-01
Klhl21	22098	0.11637016	13423	1.008001E-01
Klhl22	22099	-0.118346992	109	9.510564E-02
Klhl23	22100	-0.046110909	2285	5.167494E-01
Klhl24	22101	0.190532668	17865	6.883029E-03

Spearman Rank correlation analysis performed between Prdm1 and all-expressed genes within the Meredith RNA-seq dataset. Robust Prdm1–associated genes were identified using a cut-off of $p < 0.0005$.

Table S1, Related to Supplemental Figure 3C. Prdm1 associated genes

Klhl25	22102	0.137091913	14763	5.289592E-02
Klhl26	22103	0.019531273	6304	7.836937E-01
Klhl28	22104	0.151753404	15751	3.194450E-02
Klhl29	22105	0.14939197	15613	3.474296E-02
Klhl3	22106	0.074460569	10176	2.946909E-01
Klhl30	22107	0.063365727	9418	3.727114E-01
Klhl31	22108	0.28894265	19859	3.331911E-05
Klhl32	22109	0.125404699	14000	7.683291E-02
Klhl33	22110		#N/A	1.000000E+00
Klhl34	22111		#N/A	1.000000E+00
Klhl35	22112	0.000307048	5309	9.965571E-01
Klhl36	22113	0.041622118	7719	5.584201E-01
Klhl38	22114	-0.038406171	3065	5.892384E-01
Klhl4	22115	0.075936443	10345	2.851976E-01
Klhl40	22116	0.224158595	19022	1.418228E-03
Klhl41	22117	0.06823271	9717	3.370415E-01
Klhl42	22118	0.117865358	13518	9.646890E-02
Klhl5	22119	0.123735023	13889	8.087638E-02
Klhl6	22120	0.11593599	13391	1.020862E-01
Klhl7	22121	0.086406576	11076	2.237599E-01
Klhl8	22122	-0.079983478	644	2.602282E-01
Klhl9	22123	0.148329088	15549	3.606880E-02
Klk1	22124	0.011033199	5825	8.767743E-01
Klk10	22125	0.020875719	6412.5	7.692098E-01
Klk11	22126	0.102971158	12242	1.467899E-01
Klk12	22127	0.050196163	8311	4.802649E-01
Klk13	22128	0.104492917	12324	1.408793E-01
Klk14	22129	0.037024818	7430	6.027106E-01
Klk15	22130		#N/A	1.000000E+00
Klk1b1	22131	-0.001224019	5151	9.862757E-01
Klk1b11	22132	0.051334071	8416	4.703565E-01
Klk1b16	22133	0.002568575	5447.5	9.712046E-01
Klk1b21	22134	-0.033318989	3778	6.395141E-01
Klk1b22	22135	0.13512616	14630	5.642310E-02
Klk1b24	22136	0.090568686	11390	2.021574E-01
Klk1b26	22137	0.055223732	8937	4.373481E-01
Klk1b27	22138	0.113324719	12943	1.100965E-01
Klk1b3	22139	0.130860052	14343	6.474844E-02
Klk1b4	22140	0.052657832	8516	4.589722E-01
Klk1b5	22141	0.073221412	10082	3.028221E-01
Klk1b7-ps	22142	0.271786773	19733	9.897490E-05
Klk1b8	22143	0.181437002	17464	1.013468E-02
Klk1b9	22144	0.150220751	15664	3.373801E-02

Spearman Rank correlation analysis performed between Prdm1 and all-expressed genes within the Meredith RNA-seq dataset. Robust Prdm1–associated genes were identified using a cut-off of $p < 0.0005$.

Table S1, Related to Supplemental Figure 3C. Prdm1 associated genes

Klk4	22145	0.152336998	15789	3.128323E-02
Klk5	22146	0.055675782	8967	4.335996E-01
Klk6	22147	0.123692473	13887	8.098162E-02
Klk7	22148	0.204999983	18427	3.591732E-03
Klk8	22149	-0.026174589	4058	7.129423E-01
Klk9	22150	0.185164397	17644	8.666303E-03
Klkb1	22151		#N/A	1.000000E+00
Klra1	22152	0.038758411	7558.5	5.858252E-01
Klra10	22153	-0.054451513	1703	4.437940E-01
Klra11-ps	22154	-0.038406171	3065	5.892384E-01
Klra13-ps	22155	0.113548679	13094	1.093908E-01
Klra14	22156		#N/A	1.000000E+00
Klra17	22157	0.031385694	7152	6.590774E-01
Klra2	22158	-0.038406171	3065	5.892384E-01
Klra3	22159	0.217153959	18822	2.010252E-03
Klra4	22160	0.072015234	10001	3.108778E-01
Klra5	22161	0.011635093	5858.5	8.701096E-01
Klra6	22162		#N/A	1.000000E+00
Klra7	22163		#N/A	1.000000E+00
Klra8	22164	0.049599818	8262	4.855024E-01
Klra9	22165	0.087115402	11136	2.199696E-01
Klrb1	22166	-0.038406171	3065	5.892384E-01
Klrb1-ps1	22167		#N/A	1.000000E+00
Klrb1a	22168	0.073498585	10098.5	3.009906E-01
Klrb1b	22169	0.070653297	9885	3.201408E-01
Klrb1c	22170	0.053267784	8685	4.537786E-01
Klrb1f	22171	0.022198129	6607.5	7.550408E-01
Klrc1	22172		#N/A	1.000000E+00
Klrc2	22173	-0.054450825	1984	4.437997E-01
Klrc3	22174		#N/A	1.000000E+00
Klrd1	22175		#N/A	1.000000E+00
Klre1	22176		#N/A	1.000000E+00
Klrg1	22177	0.004869018	5555	9.454456E-01
Klrg2	22178		#N/A	1.000000E+00
Klri1	22179	-0.10317255	224	1.459970E-01
Klri2	22180	0.236910787	19304	7.311123E-04
Klrk1	22181	-0.049355564	2212	4.876564E-01
Kmo	22182	0.289744936	19864	3.160962E-05
Kncn	22183		#N/A	1.000000E+00
Kndc1	22184	0.163971365	16593	2.033569E-02
Kng1	22185	-0.056155848	1482	4.296392E-01
Kng2	22186	0.07262924	10038	3.067596E-01
Knop1	22187	0.213580931	18700	2.392025E-03

Spearman Rank correlation analysis performed between Prdm1 and all-expressed genes within the Meredith RNA-seq dataset. Robust Prdm1–associated genes were identified using a cut-off of $p < 0.0005$.

Table S1, Related to Supplemental Figure 3C. Prdm1 associated genes

Knstrn	22188	0.164429043	16614	1.998331E-02
Kntc1	22189	0.16194334	16465	2.196265E-02
Kpna1	22190	0.101002996	12118	1.547110E-01
Kpna2	22191	0.165548488	16680	1.914360E-02
Kpna3	22192	0.035511864	7347	6.176221E-01
Kpna4	22193	0.056020019	8987	4.307576E-01
Kpna6	22194	0.002494081	5442	9.720394E-01
Kpna7	22195	0.063369885	9422	3.726800E-01
Kpnb1	22196	0.214480073	18727	2.290204E-03
Kprp	22197		#N/A	1.000000E+00
Kptn	22198	-0.034115011	3734	6.315299E-01
Kras	22199	0.044956044	7929	5.273156E-01
Krba1	22200	0.048932849	8216	4.913963E-01
Krcc1	22201	0.132459869	14446	6.151561E-02
Kremen1	22202	0.166370594	16742	1.854659E-02
Kremen2	22203	0.115097481	13328	1.046066E-01
Kri1	22204	0.047882033	8140	5.007589E-01
Krit1	22205	0.257112156	19594	2.375657E-04
Krr1	22206	0.150433507	15676	3.348404E-02
Krt1	22207		#N/A	1.000000E+00
Krt10	22208	0.043858155	7857	5.374609E-01
Krt12	22209	-0.092534296	394	1.924970E-01
Krt13	22210	0.221831612	18953	1.594366E-03
Krt14	22211	0.187201647	17725	7.946277E-03
Krt15	22212	0.166550721	16751	1.841797E-02
Krt16	22213	0.105754904	12404	1.361169E-01
Krt17	22214	0.119574283	13631	9.170099E-02
Krt18	22215	0.044726608	7915	5.294277E-01
Krt19	22216	0.199139885	18208	4.698910E-03
Krt2	22217	0.143605682	15224	4.249002E-02
Krt20	22218	-0.055976559	1488	4.311158E-01
Krt222	22219	0.024523744	6790	7.303229E-01
Krt23	22220	-0.001246449	5131	9.860242E-01
Krt24	22221	0.116990239	13464	9.898558E-02
Krt25	22222	-0.038406171	3065	5.892384E-01
Krt26	22223	-0.054450825	1984	4.437997E-01
Krt27	22224	-0.077394997	736.5	2.760195E-01
Krt28	22225	0.053267784	8685	4.537786E-01
Krt31	22226		#N/A	1.000000E+00
Krt32	22227		#N/A	1.000000E+00
Krt33a	22228		#N/A	1.000000E+00
Krt33b	22229	-0.043686321	2387	5.390576E-01
Krt34	22230		#N/A	1.000000E+00

Spearman Rank correlation analysis performed between Prdm1 and all-expressed genes within the Meredith RNA-seq dataset. Robust Prdm1–associated genes were identified using a cut-off of $p < 0.0005$.

Table S1, Related to Supplemental Figure 3C. Prdm1 associated genes

Krt35	22231		#N/A	1.000000E+00
Krt36	22232	-0.054450825	1984	4.437997E-01
Krt39	22233		#N/A	1.000000E+00
Krt4	22234	0.10096257	12117	1.548771E-01
Krt40	22235		#N/A	1.000000E+00
Krt42	22236	0.36885076	20016	7.685852E-08
Krt5	22237	0.099881938	12055	1.593647E-01
Krt6a	22238		#N/A	1.000000E+00
Krt6b	22239		#N/A	1.000000E+00
Krt7	22240	0.169627124	16912	1.633778E-02
Krt71	22241	0.114277824	13270	1.071175E-01
Krt72	22242		#N/A	1.000000E+00
Krt73	22243	0.103180128	12251	1.459672E-01
Krt74	22244	-0.002881632	5031	9.676968E-01
Krt75	22245	-0.044269403	2360.5	5.336493E-01
Krt76	22246	0.008522339	5706	9.046647E-01
Krt77	22247	-0.046310542	2279	5.149340E-01
Krt78	22248	0.086101506	11061	2.254054E-01
Krt79	22249	0.225122142	19046	1.350653E-03
Krt8	22250	0.09356141	11618	1.875850E-01
Krt80	22251		#N/A	1.000000E+00
Krt81	22252	0.106389689	12452	1.337685E-01
Krt82	22253	-0.066856562	1139	3.468991E-01
Krt83	22254		#N/A	1.000000E+00
Krt84	22255	0.195276729	18056	5.587615E-03
Krt86	22256	-0.038406171	3065	5.892384E-01
Krt9	22257	0.001871989	5413	9.790117E-01
Krtap1-3	22258	-0.038406171	3065	5.892384E-01
Krtap1-4	22259		#N/A	1.000000E+00
Krtap1-5	22260		#N/A	1.000000E+00
Krtap10-10	22261	-0.066856562	1139	3.468991E-01
Krtap10-4	22262		#N/A	1.000000E+00
Krtap11-1	22263		#N/A	1.000000E+00
Krtap12-1	22264		#N/A	1.000000E+00
Krtap13	22265		#N/A	1.000000E+00
Krtap13-1	22266	0.113548679	13094	1.093908E-01
Krtap14	22267		#N/A	1.000000E+00
Krtap16-1	22268		#N/A	1.000000E+00
Krtap16-3	22269		#N/A	1.000000E+00
Krtap17-1	22270		#N/A	1.000000E+00
Krtap19-1	22271		#N/A	1.000000E+00
Krtap19-2	22272	0.088187776	11217	2.143225E-01
Krtap19-3	22273		#N/A	1.000000E+00

Spearman Rank correlation analysis performed between Prdm1 and all-expressed genes within the Meredith RNA-seq dataset. Robust Prdm1–associated genes were identified using a cut-off of $p < 0.0005$.

Table S1, Related to Supplemental Figure 3C. Prdm1 associated genes

Krtap19-4	22274		#N/A	1.000000E+00
Krtap19-5	22275		#N/A	1.000000E+00
Krtap19-9a	22276		#N/A	1.000000E+00
Krtap19-9b	22277		#N/A	1.000000E+00
Krtap2-4	22278	0.049316404	8246.5	4.880022E-01
Krtap20-2	22279		#N/A	1.000000E+00
Krtap21-1	22280		#N/A	1.000000E+00
Krtap22-2	22281		#N/A	1.000000E+00
Krtap24-1	22282		#N/A	1.000000E+00
Krtap26-1	22283		#N/A	1.000000E+00
Krtap27-1	22284		#N/A	1.000000E+00
Krtap29-1	22285		#N/A	1.000000E+00
Krtap3-1	22286		#N/A	1.000000E+00
Krtap3-2	22287		#N/A	1.000000E+00
Krtap3-3	22288		#N/A	1.000000E+00
Krtap31-1	22289		#N/A	1.000000E+00
Krtap31-2	22290		#N/A	1.000000E+00
Krtap4-1	22291		#N/A	1.000000E+00
Krtap4-13	22292		#N/A	1.000000E+00
Krtap4-16	22293	0.113548679	13094	1.093908E-01
Krtap4-2	22294		#N/A	1.000000E+00
Krtap4-6	22295		#N/A	1.000000E+00
Krtap4-7	22296		#N/A	1.000000E+00
Krtap4-8	22297	-0.054450825	1984	4.437997E-01
Krtap4-9	22298	-0.038406171	3065	5.892384E-01
Krtap5-1	22299		#N/A	1.000000E+00
Krtap5-2	22300		#N/A	1.000000E+00
Krtap5-3	22301		#N/A	1.000000E+00
Krtap5-4	22302		#N/A	1.000000E+00
Krtap5-5	22303		#N/A	1.000000E+00
Krtap6-1	22304	0.160304018	16323	2.335876E-02
Krtap6-2	22305		#N/A	1.000000E+00
Krtap6-3	22306		#N/A	1.000000E+00
Krtap6-5	22307		#N/A	1.000000E+00
Krtap7-1	22308		#N/A	1.000000E+00
Krtap8-1	22309	0.052728522	8550.5	4.583686E-01
Krtap9-1	22310		#N/A	1.000000E+00
Krtap9-3	22311		#N/A	1.000000E+00
Krtap9-5	22312		#N/A	1.000000E+00
Krtcap2	22313	0.166376018	16743	1.854271E-02
Krtcap3	22314	-0.013386064	4600	8.507752E-01
Krtdap	22315	0.045775839	7988	5.198038E-01
Ksr1	22316	0.03944219	7600	5.792255E-01

Spearman Rank correlation analysis performed between Prdm1 and all-expressed genes within the Meredith RNA-seq dataset. Robust Prdm1–associated genes were identified using a cut-off of $p < 0.0005$.

Table S1, Related to Supplemental Figure 3C. Prdm1 associated genes

Ksr2	22317	-0.051641267	2170	4.677009E-01
Kti12	22318	0.103691533	12279	1.439689E-01
Ktn1	22319	0.151910781	15760	3.176502E-02
Kxd1	22320	0.170845712	16969	1.557227E-02
Ky	22321	-0.067129303	977	3.449311E-01
Kynu	22322	0.146221714	15429	3.882438E-02
L1cam	22323	0.212829388	18685	2.480268E-03
L1td1	22324	0.166148435	16721.5	1.870631E-02
L2hgdh	22325	0.037661079	7466	5.964881E-01
L3hypdh	22326	-0.095273984	329	1.796002E-01
L3mbtl1	22327	-0.054451513	1703	4.437940E-01
L3mbtl2	22328	0.332818055	19988	1.470223E-06
L3mbtl3	22329	0.151241703	15729	3.253405E-02
L3mbtl4	22330	-0.028451763	3981	6.892098E-01
l7Rn6	22331	0.069353547	9802	3.291462E-01
Lacc1	22332	0.119069334	13593	9.308982E-02
Lace1	22333	0.069745874	9829	3.264109E-01
Lactb	22334	0.077668131	10452	2.743233E-01
Lactb2	22335	0.053597633	8777	4.509838E-01
Lactbl1	22336	-0.054451513	1703	4.437940E-01
Lad1	22337	0.015927217	6120	8.228762E-01
Lag3	22338	-0.06283942	1365	3.767031E-01
Lage3	22339	0.132092128	14417	6.224673E-02
Lair1	22340	0.259841367	19623	2.026370E-04
Lalba	22341	0.022198316	6629.5	7.550388E-01
Lama1	22342	-0.132917633	54	6.061538E-02
Lama2	22343	0.00681372	5624	9.237130E-01
Lama3	22344	0.187136581	17722	7.968425E-03
Lama4	22345	0.372765018	20020	5.453700E-08
Lama5	22346	-0.05424326	2106.5	4.455415E-01
Lamb1	22347	0.002101964	5420	9.764339E-01
Lamb2	22348	0.01894811	6277	7.899997E-01
Lamb3	22349	0.178508006	17328	1.143859E-02
Lamc1	22350	0.083704125	10894	2.386354E-01
Lamc2	22351	0.020767521	6385	7.703725E-01
Lamc3	22352	0.124066528	13913	8.006021E-02
Lamp1	22353	0.182117194	17488	9.851367E-03
Lamp2	22354	0.088261151	11228	2.139400E-01
Lamp3	22355	0.053805701	8820	4.492258E-01
Lamp5	22356	-0.054451513	1703	4.437940E-01
Lamr1-ps1	22357		#N/A	1.000000E+00
Lamtor1	22358	0.214995076	18740	2.233675E-03
Lamtor2	22359	0.139804018	14940	4.833151E-02

Spearman Rank correlation analysis performed between Prdm1 and all-expressed genes within the Meredith RNA-seq dataset. Robust Prdm1–associated genes were identified using a cut-off of $p < 0.0005$.

Table S1, Related to Supplemental Figure 3C. Prdm1 associated genes

Lamtor3	22360	0.251559076	19525	3.265903E-04
Lamtor4	22361	0.124594387	13945	7.877434E-02
Lamtor5	22362	0.102771264	12231	1.475800E-01
Lancl1	22363	0.053942462	8860	4.480724E-01
Lancl2	22364	0.196219196	18092	5.357931E-03
Lancl3	22365	0.232551114	19221	9.206907E-04
Lao1	22366	0.052728522	8550.5	4.583686E-01
Lap3	22367	0.230692968	19181	1.014461E-03
Laptm4a	22368	0.14942475	15615	3.470273E-02
Laptm4b	22369	0.118940474	13582	9.344691E-02
Laptm5	22370	0.16817696	16833	1.729134E-02
Large	22371	0.011521109	5853	8.713711E-01
Larp1	22372	-0.034177647	3731	6.309034E-01
Larp1b	22373	0.13731197	14775	5.251271E-02
Larp4	22374	0.099933301	12063	1.591492E-01
Larp4b	22375	0.082021667	10749	2.482389E-01
Larp6	22376	0.260974778	19637	1.895902E-04
Larp7	22377	0.013834746	5980	8.458347E-01
Lars	22378	0.170548414	16958	1.575608E-02
Lars2	22379	0.070798869	9897	3.191423E-01
Las1l	22380	0.103228698	12254	1.457766E-01
Lasp1	22381	0.182987467	17551	9.499103E-03
Lat	22382	-0.086750697	472.5	2.219141E-01
Lat2	22383	0.330816735	19986	1.713843E-06
Lats1	22384	0.268086565	19706	1.240170E-04
Lats2	22385	0.172945165	17075	1.432671E-02
Lax1	22386	0.313558718	19947	6.150814E-06
Layn	22387		#N/A	1.000000E+00
Lbh	22388	0.095925034	11765	1.766315E-01
Lbp	22389	0.030690022	7107	6.661749E-01
Lbr	22390	0.282962013	19817	4.909597E-05
Lbx1	22391	0.113548679	13094	1.093908E-01
Lbx2	22392		#N/A	1.000000E+00
Lca5	22393	0.155460101	16004	2.793921E-02
Lca5l	22394	0.177700537	17297	1.182310E-02
Lcat	22395	0.107029169	12498	1.314343E-01
Lce1a1	22396		#N/A	1.000000E+00
Lce1a2	22397		#N/A	1.000000E+00
Lce1b	22398		#N/A	1.000000E+00
Lce1c	22399	0.053805701	8820	4.492258E-01
Lce1d	22400	0.113548679	13094	1.093908E-01
Lce1e	22401	-0.03087642	3893.5	6.642703E-01
Lce1f	22402	-0.066855996	1239.5	3.469032E-01

Spearman Rank correlation analysis performed between Prdm1 and all-expressed genes within the Meredith RNA-seq dataset. Robust Prdm1–associated genes were identified using a cut-off of $p < 0.0005$.

Table S1, Related to Supplemental Figure 3C. Prdm1 associated genes

Lce1g	22403		#N/A	1.000000E+00
Lce1h	22404	-0.054450825	1984	4.437997E-01
Lce1i	22405		#N/A	1.000000E+00
Lce1j	22406		#N/A	1.000000E+00
Lce1k	22407		#N/A	1.000000E+00
Lce1l	22408		#N/A	1.000000E+00
Lce1m	22409	0.022198129	6607.5	7.550408E-01
Lce3a	22410	0.022198129	6607.5	7.550408E-01
Lce3b	22411	0.072346372	10016	3.086524E-01
Lce3c	22412	0.049631625	8265	4.852223E-01
Lce3d	22413	-0.066855996	1239.5	3.469032E-01
Lce3e	22414	-0.033387902	3775	6.388213E-01
Lce3f	22415	0.191624125	17899.5	6.563329E-03
Lce6a	22416		#N/A	1.000000E+00
Lck	22417	0.16326336	16557	2.089142E-02
Lclat1	22418	0.041669876	7723	5.579684E-01
Lcmt1	22419	0.095340688	11726	1.792944E-01
Lcmt2	22420	-0.040575525	2461	5.683638E-01
Lcn10	22421	0.113548679	13094	1.093908E-01
Lcn11	22422	0.031481901	7159	6.580982E-01
Lcn12	22423		#N/A	1.000000E+00
Lcn2	22424	0.042819219	7796	5.471503E-01
Lcn3	22425	0.052012842	8472	4.644998E-01
Lcn4	22426		#N/A	1.000000E+00
Lcn5	22427		#N/A	1.000000E+00
Lcn6	22428	-0.043102601	2411	5.444990E-01
Lcn8	22429	0.108022805	12555	1.278697E-01
Lcn9	22430	-0.066856562	1139	3.468991E-01
Lcor	22431	0.092107838	11525	1.945637E-01
Lcorl	22432	0.146574188	15452	3.835149E-02
Lcp1	22433	0.084320117	10944	2.351853E-01
Lcp2	22434	0.11002164	12716	1.209256E-01
Lct	22435	0.022198316	6629.5	7.550388E-01
Lctl	22436	0.086412184	11087.5	2.237298E-01
Ldb1	22437	0.043328376	7828	5.423911E-01
Ldb2	22438	-0.030541249	3911	6.676966E-01
Ldb3	22439	0.148751941	15578	3.553629E-02
Ldha	22440	0.113050992	12931	1.109640E-01
Ldha-ps	22441		#N/A	1.000000E+00
Ldha-ps2	22442		#N/A	1.000000E+00
Ldhb	22443	0.033672043	7275	6.359677E-01
Ldhc	22444	-0.066855996	1239.5	3.469032E-01
Ldhd	22445	-0.038406171	3065	5.892384E-01

Spearman Rank correlation analysis performed between Prdm1 and all-expressed genes within the Meredith RNA-seq dataset. Robust Prdm1–associated genes were identified using a cut-off of $p < 0.0005$.

Table S1, Related to Supplemental Figure 3C. Prdm1 associated genes

Ldlr	22446	0.091225962	11460	1.988888E-01
Ldlrad1	22447	-0.077393517	797	2.760287E-01
Ldlrad2	22448		#N/A	1.000000E+00
Ldlrad3	22449	0.035093153	7335	6.217771E-01
Ldlrad4	22450	0.100214523	12076	1.579733E-01
Ldlrap1	22451	0.183457229	17567	9.313627E-03
Ldoc1	22452		#N/A	1.000000E+00
Ldoc1l	22453	-0.159605123	11	2.397682E-02
Leap2	22454		#N/A	1.000000E+00
Lect1	22455	0.154197943	15906	2.925190E-02
Lect2	22456	-0.054450825	1984	4.437997E-01
Lef1	22457	0.234565958	19262	8.280677E-04
Lefty1	22458	-0.054451513	1703	4.437940E-01
Lefty2	22459	0.125622994	14014	7.631656E-02
Lekr1	22460	0.05186258	8460	4.657929E-01
Lelp1	22461		#N/A	1.000000E+00
Lemd1	22462	0.020875719	6412.5	7.692098E-01
Lemd2	22463	-0.117843617	110	9.653080E-02
Lemd3	22464	0.155400095	15999	2.800046E-02
Lenep	22465	0.037138591	7439	6.015958E-01
Leng1	22466	0.2431092	19417	5.229444E-04
Leng8	22467	0.110752118	12776	1.184622E-01
Leng9	22468	-0.049044803	2216	4.904043E-01
Leo1	22469	0.268504308	19708	1.209185E-04
Lep	22470	0.030441472	7094	6.687180E-01
Lepr	22471	-0.108944764	190	1.246295E-01
Lepre1	22472	0.112289979	12878	1.134039E-01
Leprel1	22473	-0.007148316	4847	9.199791E-01
Leprel2	22474	0.045297129	7957	5.241836E-01
Leprel4	22475	0.047952846	8145	5.001251E-01
Leprot	22476	0.123927533	13905	8.040161E-02
Leprotl1	22477	-0.052730097	2146	4.583551E-01
Letm1	22478	0.024486526	6788	7.307163E-01
Letm2	22479	0.049655228	8267	4.850145E-01
Letmd1	22480	0.038728396	7543	5.861157E-01
Lfng	22481	0.013496627	5970	8.495572E-01
Lgals1	22482	0.091103223	11448	1.994962E-01
Lgals12	22483	-0.076475461	851	2.817822E-01
Lgals2	22484	0.07756975	10445	2.749334E-01
Lgals3	22485	0.062541135	9360	3.789769E-01
Lgals3bp	22486	0.230622821	19180	1.018167E-03
Lgals4	22487	-0.03336962	3777	6.390050E-01
Lgals7	22488	0.05966434	9195	4.013345E-01

Spearman Rank correlation analysis performed between Prdm1 and all-expressed genes within the Meredith RNA-seq dataset. Robust Prdm1–associated genes were identified using a cut-off of $p < 0.0005$.

Table S1, Related to Supplemental Figure 3C. Prdm1 associated genes

Lgals8	22489	-0.023536313	4136	7.407856E-01
Lgals9	22490	0.226391348	19077	1.266141E-03
Lgalsl	22491	0.125037933	13971	7.770680E-02
Lgi1	22492	0.053267784	8685	4.537786E-01
Lgi2	22493	-0.027158545	4027	7.026521E-01
Lgi3	22494	0.113548679	13094	1.093908E-01
Lgi4	22495	-0.077393517	797	2.760287E-01
Lgmn	22496	0.179233992	17369	1.110233E-02
Lgr4	22497	0.023864432	6739	7.373035E-01
Lgr5	22498	0.169200018	16886	1.661374E-02
Lgr6	22499	0.041880363	7734	5.559795E-01
Lgsn	22500	0.182294195	17509.5	9.778799E-03
Lhb	22501	-0.054450825	1984	4.437997E-01
Lhcgr	22502	-0.038905448	2491	5.844031E-01
Lhfp	22503	-0.073534861	913	3.007514E-01
Lhfpl1	22504	-0.079808513	647	2.612756E-01
Lhfpl2	22505	0.090480835	11378	2.025972E-01
Lhfpl4	22506	-0.055897591	1490	4.317672E-01
Lhfpl5	22507		#N/A	1.000000E+00
Lhpp	22508	0.17521735	17177	1.307777E-02
Lhx1	22509	-0.086750697	472.5	2.219141E-01
Lhx2	22510	-0.001224019	5151	9.862757E-01
Lhx3	22511	0.053805701	8820	4.492258E-01
Lhx4	22512	-0.026362141	4052	7.109768E-01
Lhx5	22513	0.022150667	6597	7.555479E-01
Lhx6	22514	-0.054451513	1703	4.437940E-01
Lhx8	22515	-0.000458472	5251.5	9.948592E-01
Lhx9	22516	-0.015853609	4477	8.236814E-01
Lias	22517	0.216575965	18806	2.067982E-03
Lif	22518	0.126804285	14096	7.357092E-02
Lifr	22519	0.160914326	16384	2.283031E-02
Lig1	22520	0.016116384	6131	8.208078E-01
Lig3	22521	0.119768344	13649	9.117165E-02
Lig4	22522	0.178142216	17312	1.161139E-02
Lilra5	22523	-0.038406171	3065	5.892384E-01
Lilra6	22524	-0.002295618	5058	9.742635E-01
Lilrb3	22525	0.044831733	7924	5.284594E-01
Lilrb4	22526	-0.045587209	2309	5.215273E-01
LIM2	22527		#N/A	1.000000E+00
Lim2	22528	0.113548679	13094	1.093908E-01
Lima1	22529	0.102902231	12239	1.470620E-01
Limch1	22530	0.137151752	14766	5.279149E-02
Limd1	22531	0.242216875	19398	5.490811E-04

Spearman Rank correlation analysis performed between Prdm1 and all-expressed genes within the Meredith RNA-seq dataset. Robust Prdm1–associated genes were identified using a cut-off of $p < 0.0005$.

Table S1, Related to Supplemental Figure 3C. Prdm1 associated genes

Limd2	22532	0.146850332	15470	3.798442E-02
Lime1	22533	0.272704884	19742	9.354078E-05
Limk1	22534	0.075558126	10298	2.876113E-01
Limk2	22535	0.125947966	14039	7.555310E-02
Lims1	22536	0.09256271	11553	1.923598E-01
Lims2	22537	0.021712385	6565.5	7.602361E-01
Lin28a	22538	0.122873305	13833	8.302933E-02
Lin28b	22539	0.079102939	10537	2.655289E-01
Lin37	22540	0.178476572	17326	1.145335E-02
Lin52	22541	-0.014440868	4545	8.391700E-01
Lin54	22542	0.308208228	19935	8.997222E-06
Lin7a	22543	0.08484659	10976	2.322644E-01
Lin7b	22544		#N/A	1.000000E+00
Lin7c	22545	0.243390119	19422	5.149574E-04
Lin9	22546	0.03377091	7282	6.349761E-01
Lingo1	22547	-0.120421769	100	8.940721E-02
Lingo2	22548	0.171703931	17005	1.505212E-02
Lingo3	22549	-0.054450825	1984	4.437997E-01
Lingo4	22550	0.007193855	5637	9.194710E-01
Lins	22551	0.140107858	14963	4.784124E-02
Lipa	22552	0.179943552	17399	1.078213E-02
Lipc	22553	-0.06294514	1363	3.758992E-01
Lipe	22554	0.090625548	11396.5	2.018731E-01
Lipf	22555		#N/A	1.000000E+00
Lipg	22556	-0.055481532	1506	4.352081E-01
Liph	22557	0.208855742	18545	2.997892E-03
Lipi	22558	0.097931037	11910	1.677125E-01
Lipk	22559	0.013384031	5957	8.507976E-01
Lipm	22560	0.133697015	14528	5.910760E-02
Lipn	22561		#N/A	1.000000E+00
Lipo1	22562	0.00847082	5704	9.052383E-01
Lipo2	22563		#N/A	1.000000E+00
Lipo4	22564		#N/A	1.000000E+00
Lipt1	22565	-0.021844527	4177	7.588217E-01
Lipt2	22566	0.075715395	10317.5	2.866063E-01
Litaf	22567	0.135795459	14679	5.520097E-02
Lix1	22568	0.147707398	15518	3.686395E-02
Lix1l	22569	0.053201652	8606	4.543401E-01
Llgl1	22570	0.013994371	5987	8.440784E-01
Llgl2	22571	0.22231415	18961	1.556277E-03
Llph	22572	0.043634234	7841	5.395420E-01
Lman1	22573	0.166130284	16716	1.871941E-02
Lman1l	22574	0.052728522	8550.5	4.583686E-01

Spearman Rank correlation analysis performed between Prdm1 and all-expressed genes within the Meredith RNA-seq dataset. Robust Prdm1–associated genes were identified using a cut-off of $p < 0.0005$.

Table S1, Related to Supplemental Figure 3C. Prdm1 associated genes

Lman2	22575	-0.019474483	4257	7.843071E-01
Lman2l	22576	0.003200928	5488	9.641198E-01
Lmbr1	22577	-0.119533139	105	9.181353E-02
Lmbr1l	22578	-0.05904458	1416	4.062520E-01
Lmbrd1	22579	0.229785429	19144	1.063378E-03
Lmbrd2	22580	-0.03401942	3736.5	6.324864E-01
Lmcd1	22581	0.052397028	8495	4.612029E-01
Lmf1	22582	0.012020704	5876	8.658446E-01
Lmf2	22583	0.190436611	17858	6.911819E-03
Lmln	22584	0.084230376	10935	2.356857E-01
Lmna	22585	0.074705421	10225	2.931016E-01
Lmnb1	22586	0.174796254	17154	1.330175E-02
Lmnb2	22587	-0.034498587	3727	6.276976E-01
Lmo1	22588	-0.054450825	1984	4.437997E-01
Lmo2	22589	-0.022592672	4155	7.508290E-01
Lmo3	22590	0.209682188	18567	2.882805E-03
Lmo4	22591	0.104208705	12308	1.419692E-01
Lmo7	22592	0.073160627	10076	3.032247E-01
Lmod1	22593	-0.053969499	2120	4.478446E-01
Lmod2	22594		#N/A	1.000000E+00
Lmod3	22595		#N/A	1.000000E+00
Lmtk2	22596	0.095436157	11734	1.788573E-01
Lmtk3	22597	0.07521908	10268	2.897860E-01
Lmx1a	22598	-0.025177768	4094	7.234201E-01
Lmx1b	22599	0.129052734	14240	6.856658E-02
Lnp	22600	0.06439799	9499	3.649584E-01
Lnpep	22601	0.127187026	14127	7.269871E-02
Lnx1	22602	-0.024482303	4115	7.307610E-01
Lnx2	22603	0.186640687	17703	8.139033E-03
Loh12cr1	22604	0.03747533	7456	5.983017E-01
Lonp1	22605	0.068094185	9709	3.380256E-01
Lonp2	22606	0.110270571	12734	1.200817E-01
Lonrf1	22607	0.104997048	12355	1.389618E-01
Lonrf2	22608	0.013460056	5969	8.499601E-01
Lonrf3	22609	0.187485437	17744.5	7.850316E-03
Lor	22610		#N/A	1.000000E+00
Lox	22611	-0.084142775	587	2.361749E-01
Loxhd1	22612	0.135533264	14663	5.567710E-02
Loxl1	22613	-0.054451513	1703	4.437940E-01
Loxl2	22614	0.1797594	17391	1.086443E-02
Loxl3	22615	-0.018965124	4273	7.898155E-01
Loxl4	22616	0.053805701	8820	4.492258E-01
Lpar1	22617	0.103545029	12272	1.445392E-01

Spearman Rank correlation analysis performed between Prdm1 and all-expressed genes within the Meredith RNA-seq dataset. Robust Prdm1–associated genes were identified using a cut-off of $p < 0.0005$.

Table S1, Related to Supplemental Figure 3C. Prdm1 associated genes

Lpar2	22618	0.140054162	14959	4.792758E-02
Lpar3	22619	0.058608903	9135	4.097301E-01
Lpar4	22620	0.211082887	18614	2.696859E-03
Lpar5	22621	0.088187776	11217	2.143225E-01
Lpar6	22622	0.058162342	9103	4.133133E-01
Lpcat1	22623	0.096463981	11805	1.742015E-01
Lpcat2	22624	-0.015854169	4476	8.236753E-01
Lpcat2b	22625		#N/A	1.000000E+00
Lpcat3	22626	0.010680632	5803	8.806822E-01
Lpcat4	22627	0.077293569	10429	2.766512E-01
Lpgat1	22628	0.137356587	14783	5.243530E-02
Lphn1	22629	-0.010143766	4739	8.866385E-01
Lphn2	22630	0.11557245	13363	1.031730E-01
Lphn3	22631	0.18281975	17544	9.566110E-03
Lpin1	22632	0.222383379	18966	1.550881E-03
Lpin2	22633	0.04209006	7745	5.540015E-01
Lpin3	22634	0.056093733	8990	4.301504E-01
Lpl	22635	0.209118994	18553	2.960789E-03
Lpo	22636	-0.011883981	4675	8.673564E-01
Lpp	22637	0.172212847	17034	1.475090E-02
Lpxn	22638	0.169560076	16904	1.638083E-02
Lrat	22639	0.041674158	7725	5.579279E-01
Lrba	22640	0.108948418	12619	1.246167E-01
Lrch1	22641	0.115519775	13358	1.033312E-01
Lrch2	22642	0.11766179	13507	9.704975E-02
Lrch3	22643	0.212362973	18670	2.536508E-03
Lrch4	22644	0.17768727	17295	1.182951E-02
Lrcol1	22645	-0.031503431	3866.5	6.578792E-01
Lrdd	22646	-0.066856562	1139	3.468991E-01
Lrfn1	22647		#N/A	1.000000E+00
Lrfn2	22648	0.006572933	5618	9.264012E-01
Lrfn3	22649	-0.017417228	4393	8.066179E-01
Lrfn4	22650	0.041210904	7700	5.623171E-01
Lrfn5	22651	0.188625881	17784	7.474992E-03
Lrg1	22652		#N/A	1.000000E+00
Lrguk	22653	0.08971082	11325	2.064817E-01
Lrif1	22654	-0.003191159	5018	9.642292E-01
Lrig1	22655	0.000903554	5356	9.898687E-01
Lrig2	22656	0.141688855	15059	4.535668E-02
Lrig3	22657	0.009623493	5755	8.924167E-01
Lrit1	22658	0.147480918	15502	3.715729E-02
Lrit2	22659	0.146302305	15435	3.871583E-02
Lrit3	22660		#N/A	1.000000E+00

Spearman Rank correlation analysis performed between Prdm1 and all-expressed genes within the Meredith RNA-seq dataset. Robust Prdm1–associated genes were identified using a cut-off of $p < 0.0005$.

Table S1, Related to Supplemental Figure 3C. Prdm1 associated genes

Lrmp	22661	0.15956001	16253	2.401719E-02
Lrp1	22662	0.184251161	17602	9.007441E-03
Lrp10	22663	0.2468449	19471	4.255347E-04
Lrp11	22664	0.12665107	14080	7.392244E-02
Lrp12	22665	0.097863677	11901	1.680064E-01
Lrp1b	22666	0.104136921	12301	1.422455E-01
Lrp2	22667	-0.004113901	4982	9.538961E-01
Lrp2bp	22668	0.269786612	19715	1.118538E-04
Lrp3	22669	0.021508422	6554	7.624208E-01
Lrp4	22670	0.146968014	15477	3.782889E-02
Lrp5	22671	-0.053903836	2122	4.483980E-01
Lrp6	22672	0.199696324	18235	4.581951E-03
Lrp8	22673	-0.088396715	431	2.132344E-01
Lrpap1	22674	-0.100441293	273	1.570298E-01
Lrpprc	22675	0.125281351	13988	7.712591E-02
Lrr1	22676	-0.066855996	1239.5	3.469032E-01
Lrrc1	22677	0.04439163	7890	5.325191E-01
Lrrc10	22678		#N/A	1.000000E+00
Lrrc10b	22679	-0.038406171	3065	5.892384E-01
Lrrc14	22680	-0.00799254	4819	9.105658E-01
Lrrc14b	22681	0.071544388	9951	3.140601E-01
Lrrc15	22682	0.064113467	9472	3.670853E-01
Lrrc16a	22683	0.188152514	17765	7.628796E-03
Lrrc16b	22684	0.087060757	11130	2.202602E-01
Lrrc17	22685	-0.046734007	2267	5.110940E-01
Lrrc18	22686	-0.086748473	486.5	2.219259E-01
Lrrc19	22687	0.051467968	8425	4.691980E-01
Lrrc2	22688	0.130864873	14345	6.473850E-02
Lrrc20	22689	0.067007849	9644	3.458066E-01
Lrrc23	22690	0.026389085	6894	7.106946E-01
Lrrc24	22691	0.024966895	6816	7.256433E-01
Lrrc25	22692	0.07494696	10242	2.915394E-01
Lrrc26	22693	0.072345988	10015	3.086549E-01
Lrrc27	22694	0.050544302	8344.5	4.772215E-01
Lrrc28	22695	0.093006861	11587	1.902255E-01
Lrrc29	22696	0.160304018	16323	2.335876E-02
Lrrc3	22697	0.150261418	15665	3.368934E-02
Lrrc30	22698	-0.066855996	1239.5	3.469032E-01
Lrrc31	22699	-0.054450825	1984	4.437997E-01
Lrrc32	22700		#N/A	1.000000E+00
Lrrc33	22701	-0.029911917	3929	6.741487E-01
Lrrc34	22702	0.191455875	17893	6.611731E-03
Lrrc36	22703	0.228819993	19109	1.117786E-03

Spearman Rank correlation analysis performed between Prdm1 and all-expressed genes within the Meredith RNA-seq dataset. Robust Prdm1–associated genes were identified using a cut-off of $p < 0.0005$.

Table S1, Related to Supplemental Figure 3C. Prdm1 associated genes

Lrrc37a	22704	0.240460724	19367	6.040805E-04
LRRC38	22705		#N/A	1.000000E+00
Lrrc38	22706	-0.038406171	3065	5.892384E-01
Lrrc39	22707	0.109072852	12629	1.241844E-01
Lrrc3b	22708	0.138305446	14847	5.081120E-02
Lrrc4	22709	0.097780658	11890	1.683692E-01
Lrrc40	22710	-0.08581103	557	2.269802E-01
Lrrc41	22711	0.298055717	19894	1.814632E-05
Lrrc42	22712	0.014425406	6038	8.393399E-01
Lrrc43	22713	0.043745824	7847	5.385044E-01
Lrrc45	22714	0.137867085	14814	5.155624E-02
Lrrc46	22715		#N/A	1.000000E+00
Lrrc47	22716	-0.081227385	628	2.528647E-01
Lrrc48	22717	-0.075400473	878	2.886211E-01
Lrrc49	22718	-0.052377946	2152	4.613663E-01
Lrrc4b	22719	0.057904871	9079	4.153876E-01
Lrrc4c	22720	0.153323115	15855	3.019235E-02
Lrrc51	22721	0.065477321	9560	3.569596E-01
Lrrc52	22722	-0.054450825	1984	4.437997E-01
Lrrc55	22723	0.187843751	17757	7.730628E-03
Lrrc56	22724	0.194942712	18041	5.671108E-03
Lrrc57	22725	-0.026723685	4039	7.071933E-01
Lrrc58	22726	0.129174933	14251	6.830276E-02
Lrrc59	22727	0.193462735	17971	6.054648E-03
Lrrc6	22728	0.104830865	12348	1.395917E-01
Lrrc61	22729	0.04234566	7763	5.515951E-01
Lrrc63	22730	0.103943745	12294	1.429910E-01
Lrrc66	22731	0.016372126	6140	8.180133E-01
Lrrc69	22732	0.09404285	11645	1.853145E-01
Lrrc7	22733	-0.011641459	4680	8.700392E-01
Lrrc70	22734	0.188941405	17800.5	7.374013E-03
Lrrc71	22735	-0.005759256	4890	9.354910E-01
Lrrc72	22736	-0.086748028	498.5	2.219283E-01
Lrrc73	22737	-0.054450825	1984	4.437997E-01
Lrrc8a	22738	-0.001340544	5126	9.849693E-01
Lrrc8b	22739	-0.077516254	667	2.752656E-01
Lrrc8c	22740	0.11438755	13280	1.067787E-01
Lrrc8d	22741	0.148916261	15585	3.533116E-02
Lrrc8e	22742	0.101314183	12138	1.534376E-01
Lrrc9	22743	0.200566941	18265	4.404198E-03
Lrrcc1	22744	0.014080885	5994	8.431269E-01
Lrrd1	22745	0.073928833	10125	2.981622E-01
Lrrfip1	22746	0.133245041	14497	5.997817E-02

Spearman Rank correlation analysis performed between Prdm1 and all-expressed genes within the Meredith RNA-seq dataset. Robust Prdm1–associated genes were identified using a cut-off of $p < 0.0005$.

Table S1, Related to Supplemental Figure 3C. Prdm1 associated genes

Lrrfip2	22747	0.22883833	19110	1.116730E-03
Lrriq1	22748	0.057930216	9082	4.151831E-01
Lrriq3	22749	0.034681085	7320	6.258778E-01
Lrriq4	22750	0.142299573	15098	4.442628E-02
Lrrk1	22751	-0.059335646	1410	4.039381E-01
Lrrk2	22752	0.080069422	10597	2.597148E-01
Lrrn1	22753	-0.046309849	2280	5.149403E-01
Lrrn2	22754	0.06641968	9615	3.500662E-01
Lrrn3	22755	0.214769063	18731	2.258325E-03
Lrrn4	22756	-0.032762514	3799	6.451205E-01
Lrrn4cl	22757	-0.015234904	4501	8.304566E-01
Lrrtm1	22758	-0.018612583	4297	7.936341E-01
Lrrtm2	22759	-0.066858259	1029.5	3.468868E-01
Lrrtm3	22760	-0.026764444	4038	7.067672E-01
Lrrtm4	22761	0.229174695	19126	1.097507E-03
Lrsam1	22762	-0.099575951	281	1.606530E-01
Lrtm1	22763	0.047539246	8115	5.038333E-01
Lrtm2	22764	0.090982489	11432	2.000951E-01
Lrwd1	22765	0.044798452	7920	5.287659E-01
Lsamp	22766	0.151929296	15762	3.174396E-02
Lsg1	22767	-0.099492042	282	1.610076E-01
Lsm1	22768	0.086013268	11055	2.258830E-01
Lsm10	22769	0.116552035	13437	1.002652E-01
Lsm11	22770	0.140163633	14968	4.775170E-02
Lsm12	22771	0.216354417	18796	2.090508E-03
Lsm14a	22772	0.221882031	18954	1.590346E-03
Lsm14b	22773	0.048855225	8208	4.920847E-01
Lsm2	22774	0.083613264	10887	2.391473E-01
Lsm3	22775	0.087990277	11203	2.153547E-01
Lsm4	22776	0.092541547	11552	1.924620E-01
Lsm5	22777	0.173872165	17116	1.380509E-02
Lsm6	22778	0.212316577	18666	2.542165E-03
Lsm7	22779	0.098818048	11982	1.638775E-01
Lsmd1	22780	0.255382038	19570	2.625295E-04
Lsmem1	22781	0.053805701	8820	4.492258E-01
Lsp1	22782	0.073480426	10095	3.011104E-01
Lsr	22783	0.121929844	13782	8.543911E-02
Lss	22784	0.138956319	14883	4.972150E-02
Lst1	22785	-0.170607291	3	1.571953E-02
Lta	22786	0.045940049	8000	5.183057E-01
Lta4h	22787	0.252930096	19536	3.021092E-04
Ltb	22788	0.045025423	7935	5.266777E-01
Ltb4r1	22789		#N/A	1.000000E+00

Spearman Rank correlation analysis performed between Prdm1 and all-expressed genes within the Meredith RNA-seq dataset. Robust Prdm1–associated genes were identified using a cut-off of $p < 0.0005$.

Table S1, Related to Supplemental Figure 3C. Prdm1 associated genes

Ltb4r2	22790	-0.066855996	1239.5	3.469032E-01
Ltbp1	22791	0.100314892	12079	1.575551E-01
Ltbp2	22792	0.108802596	12611	1.251249E-01
Ltbp3	22793	0.242831356	19410	5.309566E-04
Ltbp4	22794	-0.035368481	3703	6.190436E-01
Ltbr	22795	-0.023519283	4138	7.409664E-01
Ltc4s	22796	0.187063314	17719	7.993430E-03
Ltf	22797	0.181862146	17479	9.956769E-03
Ltk	22798	-0.086746694	532	2.219355E-01
Ltn1	22799	0.125614165	14013	7.633739E-02
Ltv1	22800	0.109987429	12715	1.210419E-01
Luc7l	22801	0.086213261	11064	2.248016E-01
Luc7l2	22802	0.133561761	14514	5.936703E-02
Luc7l3	22803	0.162912151	16539	2.117193E-02
Lum	22804	0.186093593	17687	8.331013E-03
Lurap1	22805	0.00211968	5424	9.762354E-01
Lurap1l	22806	-0.005282475	4915	9.408212E-01
Luzp1	22807	0.133084517	14482	6.028990E-02
Luzp2	22808	-0.000498786	5243	9.944071E-01
Luzp4	22809	-0.038406171	3065	5.892384E-01
Lxn	22810	0.208655285	18540	3.026426E-03
Ly6a	22811	0.23549191	19276	7.884476E-04
Ly6c1	22812	0.157129229	16114	2.628090E-02
Ly6c2	22813	0.171017938	16974	1.546664E-02
Ly6d	22814	0.144705125	15340	4.091552E-02
Ly6e	22815	0.208285565	18531	3.079699E-03
Ly6f	22816	0.129163658	14249	6.832707E-02
Ly6g	22817		#N/A	1.000000E+00
Ly6g5b	22818		#N/A	1.000000E+00
Ly6g5c	22819	0.087115402	11136	2.199696E-01
Ly6g6c	22820	-0.018069136	4367	7.995302E-01
Ly6g6d	22821	0.113548679	13094	1.093908E-01
Ly6g6e	22822	0.034428378	7310	6.283983E-01
Ly6g6f	22823	-0.066856562	1139	3.468991E-01
Ly6h	22824	0.003111067	5479	9.651264E-01
Ly6i	22825	0.265069794	19677	1.486978E-04
Ly6k	22826	-0.059732681	1402	4.007944E-01
Ly75	22827	-0.024688316	4108	7.285838E-01
Ly86	22828	0.166313336	16734	1.858764E-02
Ly9	22829	0.018139774	6231	7.987632E-01
Ly96	22830	0.140873575	15012	4.662409E-02
Lyar	22831	0.189168977	17808	7.301938E-03
Lyg1	22832	-0.038406171	3065	5.892384E-01

Spearman Rank correlation analysis performed between Prdm1 and all-expressed genes within the Meredith RNA-seq dataset. Robust Prdm1–associated genes were identified using a cut-off of $p < 0.0005$.

Table S1, Related to Supplemental Figure 3C. Prdm1 associated genes

Lyg2	22833	-0.066855996	1239.5	3.469032E-01
Lyl1	22834	0.236040652	19290	7.657994E-04
Lyn	22835	0.070644622	9884	3.202004E-01
Lynx1	22836	0.160671741	16374	2.303911E-02
Lypd1	22837	-0.092610278	390	1.921304E-01
Lypd2	22838	0.086241884	11067	2.246472E-01
Lypd3	22839	-0.035721065	3694	6.155507E-01
Lypd4	22840		#N/A	1.000000E+00
Lypd5	22841		#N/A	1.000000E+00
Lypd6	22842	0.017124558	6181	8.098051E-01
Lypd6b	22843	0.138934599	14878	4.975755E-02
Lypd8	22844	0.085742485	11040	2.273529E-01
Lypla1	22845	0.216031539	18786	2.123736E-03
Lypla2	22846	-0.115103636	133	1.045879E-01
Lyplal1	22847	-0.031243917	3875	6.605214E-01
Lyrm1	22848	-0.03493818	3712	6.233179E-01
Lyrm2	22849	-0.053539184	2129	4.514783E-01
Lyrm4	22850	0.16415436	16601	2.019416E-02
Lyrm5	22851	-0.034137033	3733	6.313096E-01
Lyrm7	22852	-0.003870974	4987	9.566158E-01
Lyrm9	22853	0.044755263	7916	5.291637E-01
Lysmd1	22854	0.007882062	5675	9.117970E-01
Lysmd2	22855	0.018756826	6261	7.920711E-01
Lysmd3	22856	-0.047130985	2258	5.075078E-01
Lysmd4	22857	0.002079108	5419	9.766901E-01
Lyst	22858	-0.033882246	3742	6.338602E-01
Lyve1	22859		#N/A	1.000000E+00
Lyz1	22860	0.12985535	14293	6.684889E-02
Lyz2	22861	0.136519991	14728	5.390275E-02
Lyzl1	22862	-0.038406171	3065	5.892384E-01
Lyzl4	22863	0.031580877	7163	6.570915E-01
Lyzl6	22864	-0.038406171	3065	5.892384E-01
Lzic	22865	-0.039136703	2488	5.821697E-01
Lztfl1	22866	0.124582102	13942	7.880408E-02
Lztr1	22867	0.072826106	10050	3.054469E-01
Lzts1	22868		#N/A	1.000000E+00
Lzts2	22869	0.016631101	6154	8.151859E-01
Lzts3	22870	0.038966697	7577	5.838112E-01
M1ap	22871	0.044341012	7887	5.329870E-01
M5C1000I18Ri	22872		#N/A	1.000000E+00
M6pr	22873	0.173969569	17122	1.375126E-02
M6pr-ps	22874	-0.038406171	3065	5.892384E-01
Maats1	22875	0.359389511	20011	1.728892E-07

Spearman Rank correlation analysis performed between Prdm1 and all-expressed genes within the Meredith RNA-seq dataset. Robust Prdm1–associated genes were identified using a cut-off of $p < 0.0005$.

Table S1, Related to Supplemental Figure 3C. Prdm1 associated genes

Mab21l1	22876		#N/A	1.000000E+00
Mab21l2	22877	0.126345628	14070	7.462729E-02
Mab21l3	22878	0.020446139	6367	7.738293E-01
Macc1	22879	0.089966769	11340	2.051846E-01
Macf1	22880	0.048466596	8184	4.955390E-01
Macrod1	22881	0.087004907	11128	2.205574E-01
Macrod2	22882	0.172045542	17023	1.484934E-02
Mad1l1	22883	0.08705129	11129	2.203105E-01
Mad2l1	22884	0.193685626	17978	5.995441E-03
Mad2l1bp	22885	0.18989228	17836	7.077004E-03
Mad2l2	22886	0.090314903	11369	2.034298E-01
Madcam1	22887	-0.098728625	287	1.642611E-01
Madd	22888	0.103123765	12247	1.461888E-01
Maea	22889	0.092227963	11532	1.939799E-01
Mael	22890	0.09782265	11895	1.681856E-01
Maf	22891	0.012407912	5898	8.615659E-01
Maf1	22892	0.045497401	7968	5.223490E-01
Mafa	22893		#N/A	1.000000E+00
Mafb	22894	0.08887799	11262	2.107432E-01
Maff	22895	0.030660296	7105	6.664789E-01
Mafg	22896	0.040811219	7684	5.661172E-01
Mafk	22897	-0.026555789	4045	7.089494E-01
Mag	22898	-0.066855996	1239.5	3.469032E-01
Magea1	22899		#N/A	1.000000E+00
Magea10	22900		#N/A	1.000000E+00
Magea2	22901	0.053805701	8820	4.492258E-01
Magea3	22902		#N/A	1.000000E+00
Magea4	22903		#N/A	1.000000E+00
Magea5	22904	0.053805701	8820	4.492258E-01
Magea6	22905		#N/A	1.000000E+00
Magea7-ps	22906		#N/A	1.000000E+00
Magea8	22907	-0.038406171	3065	5.892384E-01
Magea9-ps	22908		#N/A	1.000000E+00
Mageb1	22909		#N/A	1.000000E+00
Mageb10-ps	22910		#N/A	1.000000E+00
Mageb16	22911	0.110451417	12753	1.194715E-01
Mageb16-ps1	22912	0.134156412	14563	5.823343E-02
Mageb17-ps	22913		#N/A	1.000000E+00
Mageb18	22914	0.002670127	5454	9.700667E-01
Mageb2	22915		#N/A	1.000000E+00
Mageb3	22916		#N/A	1.000000E+00
Mageb4	22917	0.049711368	8269.5	4.845204E-01
Mageb5	22918		#N/A	1.000000E+00

Spearman Rank correlation analysis performed between Prdm1 and all-expressed genes within the Meredith RNA-seq dataset. Robust Prdm1–associated genes were identified using a cut-off of $p < 0.0005$.

Table S1, Related to Supplemental Figure 3C. Prdm1 associated genes

Mageb6-ps	22919		#N/A	1.000000E+00
Mageb7-ps	22920		#N/A	1.000000E+00
Mageb8-ps	22921		#N/A	1.000000E+00
Maged1	22922	0.168096747	16828	1.734547E-02
Maged2	22923	-0.147534892	31	3.708720E-02
Magee1	22924	0.150861061	15707	3.297859E-02
Magee2	22925	-0.066856562	1139	3.468991E-01
Mageh1	22926	-0.036462717	3674	6.082318E-01
Magel2	22927	0.182782588	17541	9.581014E-03
Magi1	22928	0.059249261	9170	4.046240E-01
Magi2	22929	0.083228595	10849	2.413230E-01
Magi3	22930	0.056690831	9023	4.252503E-01
Magix	22931		#N/A	1.000000E+00
Magoh	22932	0.173719299	17108	1.388994E-02
Magohb	22933	-0.083091457	603	2.421020E-01
Magt1	22934	0.263349032	19661	1.647589E-04
Mak	22935	0.06519917	9545	3.590104E-01
Mak16	22936	0.007153849	5632	9.199173E-01
Mal	22937	0.147914742	15536	3.659713E-02
Mal2	22938	0.066398311	9613	3.502216E-01
Malat1	22939	0.085910532	11049	2.264399E-01
Mall	22940	0.053334538	8765	4.532122E-01
Malsu1	22941	0.184464048	17618	8.926870E-03
Malt1	22942	0.070915189	9907	3.183458E-01
Mamdc2	22943	0.060293992	9225	3.963750E-01
Mamdc4	22944	0.202743674	18347	3.986607E-03
Maml1	22945	0.123576909	13876	8.126802E-02
Maml2	22946	0.107372543	12516	1.301939E-01
Maml3	22947	0.105489804	12389	1.371069E-01
Mamld1	22948	0.005127047	5569	9.425594E-01
Mamstr	22949	-0.058964418	1417	4.068906E-01
Man1a	22950	0.117778578	13512	9.671618E-02
Man1a2	22951	0.182012999	17481	9.894307E-03
Man1b1	22952	0.152664495	15803	3.091728E-02
Man1c1	22953	-0.077394997	736.5	2.760195E-01
Man2a1	22954	0.200880252	18281	4.341763E-03
Man2a2	22955	0.156294151	16071	2.709971E-02
Man2b1	22956	0.333050908	19990	1.444127E-06
Man2b2	22957	0.166776043	16761	1.825816E-02
Man2c1	22958	0.017512373	6202	8.055825E-01
Manba	22959	0.149996791	15649	3.400711E-02
Manbal	22960	0.06103454	9262	3.905893E-01
Manea	22961	0.164834096	16645	1.967586E-02

Spearman Rank correlation analysis performed between Prdm1 and all-expressed genes within the Meredith RNA-seq dataset. Robust Prdm1–associated genes were identified using a cut-off of $p < 0.0005$.

Table S1, Related to Supplemental Figure 3C. Prdm1 associated genes

Maneal	22962		#N/A	1.000000E+00
Manf	22963	0.244586758	19433	4.821829E-04
Mansc1	22964	-0.008636405	4799	9.033949E-01
Mansc4	22965	-0.038406171	3065	5.892384E-01
Maoa	22966	-0.032786133	3792	6.448821E-01
Maob	22967	-0.018447724	4312.5	7.954215E-01
Map10	22968	0.113413019	12950	1.098179E-01
Map1a	22969	0.106361707	12449	1.338714E-01
Map1b	22970	0.14737808	15498	3.729113E-02
Map1lc3a	22971	0.070841795	9900	3.188482E-01
Map1lc3b	22972	0.105244255	12376	1.380289E-01
Map1s	22973	0.051241322	8406	4.711599E-01
Map2	22974	0.143224561	15149	4.304756E-02
Map2k1	22975	0.196920179	18121	5.192623E-03
Map2k2	22976	0.116695036	13448	9.984617E-02
Map2k3	22977	0.282427457	19812	5.080525E-05
Map2k4	22978	0.134259959	14576	5.803787E-02
Map2k5	22979	0.178143717	17313	1.161067E-02
Map2k6	22980	-0.065471476	1317	3.570026E-01
Map2k7	22981	0.211424798	18630	2.653153E-03
Map3k1	22982	0.223561779	19005	1.461620E-03
Map3k10	22983	-0.069425018	954	3.286468E-01
Map3k11	22984	0.096425912	11802	1.743723E-01
Map3k12	22985	0.332922381	19989	1.458476E-06
Map3k13	22986	0.043800991	7851	5.379918E-01
Map3k14	22987	0.026254869	6884	7.121007E-01
Map3k15	22988	0.003353641	5493	9.624092E-01
Map3k19	22989	0.022714618	6671	7.495287E-01
Map3k2	22990	0.078985248	10529	2.662429E-01
Map3k3	22991	0.018577642	6255	7.940129E-01
Map3k4	22992	-0.046759832	2266	5.108603E-01
Map3k5	22993	0.193595838	17975	6.019229E-03
Map3k6	22994	-0.057683817	1439	4.171734E-01
Map3k7	22995	0.095299928	11721	1.794812E-01
Map3k7cl	22996	0.10105166	12121	1.545114E-01
Map3k8	22997	0.053039692	8598	4.557169E-01
Map3k9	22998	0.170124188	16936	1.602165E-02
Map4	22999	0.182332285	17518	9.763245E-03
Map4k1	23000	-0.019812926	4246	7.806530E-01
Map4k2	23001	0.009842818	5765	8.899802E-01
Map4k3	23002	0.09028495	11368	2.035803E-01
Map4k4	23003	0.07019353	9852	3.233079E-01
Map4k5	23004	-0.028924431	3965	6.843206E-01

Spearman Rank correlation analysis performed between Prdm1 and all-expressed genes within the Meredith RNA-seq dataset. Robust Prdm1–associated genes were identified using a cut-off of $p < 0.0005$.

Table S1, Related to Supplemental Figure 3C. Prdm1 associated genes

Map6	23005	0.107499692	12522	1.297369E-01
Map6d1	23006	-0.011982025	4669	8.662723E-01
Map7	23007	0.061311122	9285	3.884415E-01
Map7d1	23008	-0.073332793	916	3.020852E-01
Map7d2	23009	-0.033075363	3782	6.419661E-01
Map9	23010	-0.062542627	1369	3.789655E-01
Mapk1	23011	0.1842503	17601	9.007767E-03
Mapk10	23012	0.127332491	14142	7.236943E-02
Mapk11	23013	0.030093255	7069	6.722871E-01
Mapk12	23014	0.048242947	8155	4.975327E-01
Mapk13	23015	0.212529438	18678	2.516304E-03
Mapk14	23016	0.028971901	7013	6.838303E-01
Mapk15	23017	-0.075578258	877	2.874825E-01
Mapk1ip1	23018	-0.017060204	4412	8.105063E-01
Mapk1ip1l	23019	0.242504283	19405	5.405338E-04
Mapk3	23020	0.049208287	8234	4.889577E-01
Mapk4	23021	0.033679392	7277	6.358940E-01
Mapk6	23022	0.153973429	15888	2.949084E-02
Mapk7	23023	0.143605682	15224	4.249002E-02
Mapk8	23024	0.136531907	14729	5.388161E-02
Mapk8ip1	23025	0.062592915	9364	3.785816E-01
Mapk8ip2	23026	0.147215365	15490	3.750374E-02
Mapk8ip3	23027	-0.030827194	3905	6.647731E-01
Mapk9	23028	0.061654753	9311	3.857831E-01
Mapkap1	23029	0.123411712	13872	8.167882E-02
Mapkapk2	23030	0.090204528	11365	2.039850E-01
Mapkapk3	23031	-0.083270165	597	2.410872E-01
Mapkapk5	23032	0.10995954	12714	1.211368E-01
Mapkbp1	23033	0.077120397	10418	2.777320E-01
Mapre1	23034	0.163898321	16587	2.039243E-02
Mapre2	23035	0.163378354	16566	2.080027E-02
Mapre3	23036	0.193455893	17969	6.056474E-03
Mapt	23037	0.158015271	16175	2.543542E-02
Marcks	23038	0.164899713	16650	1.962644E-02
Marcksl1	23039	0.076274864	10366	2.830500E-01
Marcksl1-ps4	23040		#N/A	1.000000E+00
Marco	23041	-0.001748557	5087	9.803953E-01
Marf1	23042	0.146568058	15451	3.835968E-02
Mark1	23043	0.130882758	14346	6.470160E-02
Mark2	23044	0.186227595	17692	8.283623E-03
Mark3	23045	0.191966881	17909	6.465702E-03
Mark4	23046	0.113548679	13094	1.093908E-01
Mars	23047	0.088844831	11258	2.109142E-01

Spearman Rank correlation analysis performed between Prdm1 and all-expressed genes within the Meredith RNA-seq dataset. Robust Prdm1–associated genes were identified using a cut-off of $p < 0.0005$.

Table S1, Related to Supplemental Figure 3C. Prdm1 associated genes

Mars2	23048	-0.086746694	532	2.219355E-01
Marveld1	23049	-0.052201118	2157	4.628825E-01
Marveld2	23050	0.14755888	15509	3.705609E-02
Marveld3	23051	0.126560862	14078	7.413005E-02
Mas1	23052	0.10080002	12110	1.555459E-01
Masp1	23053	0.038758411	7558.5	5.858252E-01
Masp2	23054	-0.034017029	3738	6.325104E-01
Mast1	23055	-0.066855996	1239.5	3.469032E-01
Mast2	23056	0.058399747	9117	4.114061E-01
Mast3	23057	-0.005216573	4921	9.415582E-01
Mast4	23058	0.231982141	19212	9.485204E-04
Mastl	23059	0.124351295	13931	7.936443E-02
Mat1a	23060	0.045646452	7976	5.209857E-01
Mat2a	23061	0.09474995	11687	1.820164E-01
Mat2b	23062	0.136601497	14734	5.375830E-02
Matk	23063	-0.077393517	797	2.760287E-01
Matn1	23064	-0.055303062	1511	4.366889E-01
Matn2	23065	-0.03122443	3877.5	6.607200E-01
Matn3	23066	0.048786593	8203	4.926938E-01
Matn4	23067	0.039817136	7626	5.756214E-01
Matr3	23068	0.229159521	19124	1.098367E-03
Matr3-ps2	23069		#N/A	1.000000E+00
Mau2	23070	0.185018287	17638	8.720100E-03
Mavs	23071	0.113549911	13230	1.093869E-01
Max	23072	0.047882046	8141	5.007588E-01
Maz	23073	0.233349522	19234	8.829037E-04
Mb	23074	0.249453261	19501	3.678027E-04
Mb21d1	23075	0.216143469	18790	2.112163E-03
Mb21d2	23076	0.016619596	6153	8.153114E-01
Mbd1	23077	0.083742521	10899	2.384193E-01
Mbd2	23078	0.116499741	13432	1.004187E-01
Mbd3	23079	0.124321033	13927	7.943814E-02
Mbd3l1	23080		#N/A	1.000000E+00
Mbd3l2	23081		#N/A	1.000000E+00
Mbd4	23082	0.252453915	19531	3.104118E-04
Mbd5	23083	0.071980349	9996	3.111128E-01
Mbd6	23084	0.172621805	17056	1.451268E-02
Mbip	23085	-0.020252219	4231	7.759172E-01
Mbl1	23086		#N/A	1.000000E+00
Mbl2	23087	-0.056976309	1461	4.229190E-01
Mblac1	23088	-0.035957214	3687	6.132162E-01
Mblac2	23089	0.201971861	18325	4.130406E-03
Mbnl1	23090	0.168564135	16855	1.703213E-02

Spearman Rank correlation analysis performed between Prdm1 and all-expressed genes within the Meredith RNA-seq dataset. Robust Prdm1–associated genes were identified using a cut-off of $p < 0.0005$.

Table S1, Related to Supplemental Figure 3C. Prdm1 associated genes

Mbnl2	23091	0.182423863	17524	9.725938E-03
Mbnl3	23092	0.214916858	18737	2.242178E-03
Mboat1	23093	0.172349916	17043	1.467068E-02
Mboat2	23094	0.017950683	6220	8.008168E-01
Mboat4	23095	0.15431986	15913.5	2.912285E-02
Mboat7	23096	0.24951995	19504	3.664267E-04
Mbp	23097	0.105271091	12377	1.379279E-01
Mbtd1	23098	0.111384447	12814	1.163615E-01
Mbtps1	23099	0.112577023	12895	1.124787E-01
Mbtps2	23100	-0.092047261	402	1.948586E-01
Mc1r	23101	0.015250113	6084	8.302899E-01
Mc2r	23102		#N/A	1.000000E+00
Mc3r	23103	-0.086746694	532	2.219355E-01
Mc4r	23104		#N/A	1.000000E+00
Mc5r	23105		#N/A	1.000000E+00
Mcam	23106	0.059302509	9177	4.042011E-01
Mcat	23107	-0.005847582	4886	9.345039E-01
Mcc	23108	0.235875408	19285	7.725556E-04
Mccc1	23109	0.107599973	12528	1.293774E-01
Mccc2	23110	-0.047955793	2234	5.000987E-01
Mcee	23111	0.135322771	14648	5.606179E-02
Mcf2	23112	0.135713265	14671	5.534986E-02
Mcf2l	23113	0.081096208	10676	2.536344E-01
Mcfd2	23114	0.03945642	7602	5.790885E-01
Mchr1	23115		#N/A	1.000000E+00
Mcidas	23116	0.117648041	13505	9.708907E-02
Mcl1	23117	0.229952859	19148	1.054194E-03
Mcm10	23118	0.221264544	18937	1.640214E-03
Mcm2	23119	0.170267014	16945	1.593181E-02
Mcm3	23120	0.060947756	9259	3.912647E-01
Mcm3ap	23121	0.058681424	9140	4.091499E-01
Mcm4	23122	0.041620532	7718	5.584351E-01
Mcm5	23123	0.088056098	11208	2.150103E-01
Mcm6	23124	0.126169064	14056	7.503721E-02
Mcm7	23125	0.254238952	19554	2.803395E-04
Mcm8	23126	0.04075343	7682	5.666676E-01
Mcm9	23127	0.089922619	11338	2.054079E-01
Mcmbp	23128	0.077901437	10464	2.728800E-01
Mcmdc2	23129	0.014145583	5997	8.424154E-01
Mcoln1	23130	-0.09843274	290	1.655352E-01
Mcoln2	23131	-0.054451513	1703	4.437940E-01
Mcoln3	23132	0.099021826	11997	1.630058E-01
Mcph1	23133	0.111144857	12798	1.171540E-01

Spearman Rank correlation analysis performed between Prdm1 and all-expressed genes within the Meredith RNA-seq dataset. Robust Prdm1–associated genes were identified using a cut-off of $p < 0.0005$.

Table S1, Related to Supplemental Figure 3C. Prdm1 associated genes

Mcpt1	23134	0.067410452	9668	3.429098E-01
Mcpt2	23135	0.008358222	5696	9.064922E-01
Mcpt4	23136	0.019622181	6307	7.827119E-01
Mcpt8	23137		#N/A	1.000000E+00
Mcpt9	23138	0.160985049	16395	2.276975E-02
Mcrs1	23139	0.145717343	15392	3.950960E-02
Mctp1	23140	0.141627536	15053	4.545100E-02
Mctp2	23141	0.058538156	9129	4.102966E-01
Mcts1	23142	0.068471602	9739	3.353487E-01
Mcts2	23143		#N/A	1.000000E+00
Mcu	23144	0.153737332	15876	2.974390E-02
Mcur1	23145	0.175701229	17200	1.282449E-02
Mdc1	23146	0.027394336	6949	7.001942E-01
Mdfi	23147	0.118493635	13556	9.469362E-02
Mdfic	23148	0.069621699	9822	3.272751E-01
Mdga1	23149	0.061693915	9315	3.854808E-01
Mdga2	23150	0.138591795	14858	5.032937E-02
Mdh1	23151	0.173574755	17104	1.397060E-02
Mdh1b	23152	0.154470253	15952	2.896432E-02
Mdh2	23153	0.152659231	15801	3.092313E-02
Mdk	23154	0.05135613	8417	4.701655E-01
Mdk-ps1	23155		#N/A	1.000000E+00
Mdm1	23156	-0.027785726	4006	6.961211E-01
Mdm2	23157	0.19283337	17946	6.224660E-03
Mdm4	23158	0.016070044	6128	8.213143E-01
Mdm4-ps	23159	0.012744805	5915	8.578464E-01
Mdn1	23160	0.112015317	12856	1.142947E-01
Mdp1	23161	0.249696247	19505	3.628123E-04
Me1	23162	0.034266595	7303	6.300142E-01
Me2	23163	0.103175885	12250	1.459839E-01
Me3	23164	0.083156763	10844	2.417308E-01
Mea1	23165		#N/A	1.000000E+00
Meaf6	23166	0.125164022	13979	7.740546E-02
Mecom	23167	0.149574225	15620	3.451981E-02
Mecp2	23168	0.171445725	16995	1.520699E-02
Mecr	23169	0.024943633	6812	7.258887E-01
Med1	23170	0.141337429	15034	4.589942E-02
Med10	23171	0.107531615	12526	1.296224E-01
Med11	23172	0.101572416	12152	1.523869E-01
Med12	23173		#N/A	1.000000E+00
Med12l	23174	0.172619081	17055	1.451425E-02
Med13	23175	0.184608141	17622	8.872700E-03
Med13l	23176	0.079073372	10536	2.657081E-01

Spearman Rank correlation analysis performed between Prdm1 and all-expressed genes within the Meredith RNA-seq dataset. Robust Prdm1–associated genes were identified using a cut-off of $p < 0.0005$.

Table S1, Related to Supplemental Figure 3C. Prdm1 associated genes

Med14	23177	0.243044012	19416	5.248142E-04
Med15	23178	0.080370173	10623	2.579235E-01
Med16	23179	-0.070104572	948	3.239230E-01
Med17	23180	0.235817251	19284	7.749465E-04
Med18	23181	0.19044223	17859	6.910132E-03
Med19	23182	0.068363149	9731	3.361165E-01
Med20	23183	-0.069611023	952	3.273494E-01
Med21	23184	0.126770904	14091	7.364739E-02
Med22	23185	-0.012827129	4635	8.569380E-01
Med23	23186	0.088445619	11237	2.129803E-01
Med24	23187	0.022309955	6650	7.538463E-01
Med25	23188	0.082686349	10803	2.444133E-01
Med26	23189	0.034544804	7314	6.272365E-01
Med27	23190	-0.033757455	3745	6.351110E-01
Med28	23191	0.109670785	12690	1.221228E-01
Med29	23192	0.121116003	13733	8.756259E-02
Med30	23193	0.177052494	17270	1.213987E-02
Med31	23194	0.081416229	10693	2.517596E-01
Med4	23195	0.035057983	7332	6.221266E-01
Med6	23196	0.050957074	8381	4.736268E-01
Med7	23197	0.184825154	17626	8.791665E-03
Med8	23198	0.157405288	16123	2.601493E-02
Med9	23199	0.039403765	7598	5.795955E-01
Medag	23200	0.071281508	9936	3.158461E-01
Mef2a	23201	0.283767263	19823	4.662294E-05
Mef2b	23202		#N/A	1.000000E+00
Mef2c	23203	0.19555971	18065	5.517742E-03
Mef2d	23204	-0.060327902	1392	3.961090E-01
Mefv	23205	-0.038406171	3065	5.892384E-01
Meg3	23206	0.121147798	13734	8.747885E-02
Megf10	23207	-0.03367206	3757.5	6.359676E-01
Megf11	23208	0.028586367	6998	6.878162E-01
Megf6	23209	0.039081303	7583	5.827044E-01
Megf8	23210	0.135105198	14626	5.646173E-02
Megf9	23211	0.162870552	16536	2.120537E-02
Mei1	23212	-0.000841282	5208.5	9.905669E-01
Mei4	23213	0.207718114	18517	3.163116E-03
Meig1	23214	0.011046632	5827	8.766254E-01
Meiob	23215	0.029748956	7055	6.758233E-01
Meis1	23216	0.122470469	13817	8.405148E-02
Meis2	23217	0.039696568	7612	5.767792E-01
Meis3	23218	-0.076867526	846	2.793153E-01
Melk	23219	0.141115032	15023	4.624568E-02

Spearman Rank correlation analysis performed between Prdm1 and all-expressed genes within the Meredith RNA-seq dataset. Robust Prdm1–associated genes were identified using a cut-off of $p < 0.0005$.

Table S1, Related to Supplemental Figure 3C. Prdm1 associated genes

Memo1	23220	0.322838994	19970	3.124384E-06
Men1	23221	0.077344187	10433	2.763359E-01
Meox1	23222	-0.057249462	1454	4.206953E-01
Meox2	23223	0.145740311	15395	3.947817E-02
Mep1a	23224	0.017892286	6218	8.014513E-01
Mep1b	23225	0.045902613	7998	5.186470E-01
Mepce	23226	0.008319394	5692	9.069246E-01
Mepe	23227		#N/A	1.000000E+00
Mertk	23228	0.042807012	7792.5	5.472647E-01
Mesdc1	23229	-0.11473558	136	1.057095E-01
Mesdc2	23230	0.1297741	14288	6.702116E-02
Mesp1	23231		#N/A	1.000000E+00
Mesp2	23232	-0.038406171	3065	5.892384E-01
Mest	23233	-0.036509548	3670	6.077710E-01
Met	23234	-0.045994976	2292	5.178051E-01
Metap1	23235	0.106676757	12475	1.327168E-01
Metap1d	23236	0.093407486	11608	1.883152E-01
Metap2	23237	0.166299607	16733	1.859750E-02
Metrn	23238		#N/A	1.000000E+00
Metrnl	23239	0.08215698	10758	2.474568E-01
Mettl1	23240	-0.043582549	2389	5.400230E-01
Mettl10	23241		#N/A	1.000000E+00
Mettl11b	23242	0.048891809	8213	4.917602E-01
Mettl13	23243	0.038378175	7516	5.895100E-01
Mettl14	23244	0.075533148	10297	2.877711E-01
Mettl15	23245	-0.11509548	134	1.046127E-01
Mettl16	23246	0.044558246	7904	5.309803E-01
Mettl17	23247	0.044399821	7891	5.324434E-01
Mettl18	23248	0.021317126	6513.5	7.644715E-01
Mettl2	23249	0.051508848	8428	4.688446E-01
Mettl20	23250	0.061689155	9314	3.855176E-01
Mettl21a	23251	0.070924527	9908	3.182819E-01
Mettl21b	23252	-0.038406171	3065	5.892384E-01
Mettl21c	23253	0.113548679	13094	1.093908E-01
Mettl21d	23254	0.027194139	6938	7.022809E-01
Mettl21e	23255	-0.052956803	2141.5	4.564224E-01
Mettl22	23256	-0.11057391	159	1.190596E-01
Mettl23	23257	0.089731748	11327	2.063754E-01
Mettl24	23258	-0.018103658	4360	7.991553E-01
Mettl25	23259	0.094188726	11656	1.846305E-01
Mettl3	23260	0.133883991	14543	5.875052E-02
Mettl4	23261	0.065178262	9543	3.591648E-01
Mettl4-ps1	23262	-0.024625472	4111.5	7.292477E-01

Spearman Rank correlation analysis performed between Prdm1 and all-expressed genes within the Meredith RNA-seq dataset. Robust Prdm1–associated genes were identified using a cut-off of $p < 0.0005$.

Table S1, Related to Supplemental Figure 3C. Prdm1 associated genes

Mettl5	23263	0.202250279	18336	4.078003E-03
Mettl6	23264	-0.048669988	2224	4.937295E-01
Mettl7a1	23265	0.035825662	7367	6.145162E-01
Mettl7a2	23266		#N/A	1.000000E+00
Mettl7a3	23267	-0.054451513	1703	4.437940E-01
Mettl7b	23268		#N/A	1.000000E+00
Mettl8	23269	0.004496179	5539	9.496173E-01
Mettl9	23270	0.049178683	8232	4.892195E-01
Mex3a	23271	0.018795523	6267	7.916520E-01
Mex3b	23272	0.094606913	11679	1.826801E-01
Mex3c	23273	0.084580479	10958	2.337376E-01
Mex3d	23274	0.195016544	18043	5.652557E-03
Mfap1a	23275	0.105031268	12358	1.388324E-01
Mfap1b	23276	0.089559368	11314	2.072520E-01
Mfap2	23277	-0.066856562	1139	3.468991E-01
Mfap3	23278	0.146637074	15456	3.826764E-02
Mfap3l	23279	-0.047882068	2239	5.007586E-01
Mfap4	23280		#N/A	1.000000E+00
Mfap5	23281	-0.007972119	4821	9.107934E-01
Mff	23282	0.109685683	12691	1.220718E-01
Mfge8	23283	0.050021624	8301	4.817947E-01
Mfhas1	23284	0.133800441	14539	5.890986E-02
Mfi2	23285	0.036529766	7407	6.075721E-01
Mfn1	23286	0.186083751	17686	8.334503E-03
Mfn2	23287	0.238150437	19331	6.841916E-04
Mfng	23288	0.187697425	17751	7.779308E-03
Mfrp	23289	-0.038406171	3065	5.892384E-01
Mfsd1	23290	0.325659706	19978	2.531621E-06
Mfsd10	23291	0.11270428	12903	1.120704E-01
Mfsd11	23292	0.058368974	9113	4.116530E-01
Mfsd12	23293	0.048442073	8168	4.957574E-01
Mfsd2a	23294	0.066063354	9594	3.526629E-01
Mfsd2b	23295	-0.003471895	5008	9.610847E-01
Mfsd3	23296	-0.077393517	797	2.760287E-01
Mfsd4	23297	0.209751223	18569	2.873375E-03
Mfsd5	23298	0.095416905	11732	1.789454E-01
Mfsd6	23299	0.015508974	6095	8.274538E-01
Mfsd6l	23300	-0.028448896	3982	6.892395E-01
Mfsd7a	23301	0.03350801	7268	6.376144E-01
Mfsd7b	23302	0.094288318	11660	1.841647E-01
Mfsd7c	23303	-0.045149205	2323	5.255407E-01
Mfsd8	23304	0.006490059	5614	9.273266E-01
Mfsd9	23305	0.020546631	6373	7.727479E-01

Spearman Rank correlation analysis performed between Prdm1 and all-expressed genes within the Meredith RNA-seq dataset. Robust Prdm1–associated genes were identified using a cut-off of $p < 0.0005$.

Table S1, Related to Supplemental Figure 3C. Prdm1 associated genes

Mga	23306	0.124429373	13936	7.917451E-02
Mgam	23307	-0.029363927	3954	6.797863E-01
Mgarp	23308	-0.110568513	165.5	1.190777E-01
Mgat1	23309	0.368529395	20015	7.903831E-08
Mgat2	23310	-0.058287694	1428	4.123056E-01
Mgat3	23311	-0.03046631	3916	6.684637E-01
Mgat4a	23312	0.180587596	17434	1.049857E-02
Mgat4b	23313	0.050289756	8326	4.794457E-01
Mgat4c	23314	0.062710023	9373	3.776884E-01
Mgat5	23315	0.098298694	11937	1.661149E-01
Mgat5b	23316	0.127773369	14174	7.137884E-02
Mgea5	23317	0.152735467	15807	3.083845E-02
Mgl2	23318	0.192643736	17936	6.276715E-03
Mgll	23319	0.197557899	18142	5.046223E-03
Mgme1	23320	0.018948579	6278	7.899946E-01
Mgmt	23321	-0.071600782	933	3.136778E-01
Mgp	23322		#N/A	1.000000E+00
Mgrn1	23323	0.131243038	14364	6.396213E-02
Mgst1	23324	-0.000513136	5240	9.942462E-01
Mgst2	23325	-0.024625472	4111.5	7.292477E-01
Mgst3	23326	0.089807369	11330	2.059917E-01
Mia	23327		#N/A	1.000000E+00
Mia2	23328	0.059501093	9186	4.026263E-01
Mia3	23329	0.200966705	18285	4.324676E-03
Miat	23330	-0.001606767	5097.5	9.819847E-01
Mib1	23331	0.117452682	13489	9.764928E-02
Mib2	23332	0.050882593	8374	4.742743E-01
Mical1	23333	0.189523876	17823	7.190787E-03
Mical2	23334	0.090106719	11357	2.044778E-01
Mical3	23335	0.026141534	6878	7.132889E-01
Micalcl	23336	0.238906112	19347	6.569688E-04
Micall1	23337	0.128872186	14231	6.895789E-02
Micall2	23338	0.070503356	9873	3.211715E-01
Micu1	23339	0.198539643	18185	4.828077E-03
Micu2	23340	0.135680472	14670	5.540936E-02
Micu3	23341	0.09689776	11827	1.722637E-01
Mid1	23342	0.242838044	19411	5.307624E-04
Mid1ip1	23343	0.115635611	13366	1.029835E-01
Mid2	23344	0.180873649	17443	1.037476E-02
Midn	23345	0.190834028	17875	6.793398E-03
Mien1	23346	0.202387744	18338	4.052352E-03
Mier1	23347	0.196574518	18105	5.273558E-03
Mier2	23348	0.121339104	13746	8.697632E-02

Spearman Rank correlation analysis performed between Prdm1 and all-expressed genes within the Meredith RNA-seq dataset. Robust Prdm1–associated genes were identified using a cut-off of $p < 0.0005$.

Table S1, Related to Supplemental Figure 3C. Prdm1 associated genes

Mier3	23349	0.27166103	19731	9.974177E-05
Mif	23350	0.171995026	17021	1.487917E-02
Mif4gd	23351	0.116651784	13444	9.997275E-02
Miip	23352	0.159577153	16255	2.400185E-02
Mill1	23353	0.022942092	6690	7.471050E-01
Mill2	23354	0.000271077	5304	9.969604E-01
Milr1	23355	0.072353418	10018	3.086051E-01
Mina	23356	-0.025679687	4079	7.181377E-01
Mink1	23357	0.068235125	9719	3.370244E-01
Minos1	23358	0.15509603	15987	2.831259E-02
Minpp1	23359	0.054676555	8906	4.419099E-01
Mios	23360	0.006484818	5613	9.273851E-01
Miox	23361		#N/A	1.000000E+00
Mip	23362	-0.038406171	3065	5.892384E-01
Mipep	23363	0.142456589	15107	4.418967E-02
Mipol1	23364	0.202622597	18342	4.008863E-03
Mir100	23365		#N/A	1.000000E+00
Mir101a	23366		#N/A	1.000000E+00
Mir101b	23367		#N/A	1.000000E+00
Mir103-1	23368		#N/A	1.000000E+00
Mir103-2	23369		#N/A	1.000000E+00
Mir105	23370		#N/A	1.000000E+00
Mir106a	23371		#N/A	1.000000E+00
Mir106b	23372		#N/A	1.000000E+00
Mir107	23373		#N/A	1.000000E+00
Mir10a	23374		#N/A	1.000000E+00
Mir10b	23375		#N/A	1.000000E+00
Mir1186	23376	0.02421538	6767	7.335850E-01
Mir1186b	23377		#N/A	1.000000E+00
Mir1187	23378	-0.054451513	1703	4.437940E-01
Mir1188	23379		#N/A	1.000000E+00
Mir1190	23380		#N/A	1.000000E+00
Mir1191	23381		#N/A	1.000000E+00
Mir1192	23382		#N/A	1.000000E+00
Mir1193	23383		#N/A	1.000000E+00
Mir1195	23384		#N/A	1.000000E+00
Mir1196	23385		#N/A	1.000000E+00
Mir1197	23386		#N/A	1.000000E+00
Mir1198	23387		#N/A	1.000000E+00
Mir1199	23388		#N/A	1.000000E+00
Mir1224	23389		#N/A	1.000000E+00
Mir122a	23390		#N/A	1.000000E+00
Mir1247	23391		#N/A	1.000000E+00

Spearman Rank correlation analysis performed between Prdm1 and all-expressed genes within the Meredith RNA-seq dataset. Robust Prdm1–associated genes were identified using a cut-off of $p < 0.0005$.

Table S1, Related to Supplemental Figure 3C. Prdm1 associated genes

Mir1249	23392	#N/A	1.000000E+00
Mir124a-1	23393	#N/A	1.000000E+00
Mir124a-2	23394	#N/A	1.000000E+00
Mir124a-3	23395	#N/A	1.000000E+00
Mir1251	23396	#N/A	1.000000E+00
Mir125a	23397	#N/A	1.000000E+00
Mir125b-1	23398	#N/A	1.000000E+00
Mir125b-2	23399	#N/A	1.000000E+00
Mir126	23400	#N/A	1.000000E+00
Mir1264	23401	#N/A	1.000000E+00
Mir127	23402	#N/A	1.000000E+00
Mir128-1	23403	#N/A	1.000000E+00
Mir128-2	23404	#N/A	1.000000E+00
Mir129-1	23405	#N/A	1.000000E+00
Mir129-2	23406	#N/A	1.000000E+00
Mir1298	23407	#N/A	1.000000E+00
Mir1306	23408	#N/A	1.000000E+00
Mir130a	23409	#N/A	1.000000E+00
Mir130b	23410	#N/A	1.000000E+00
Mir132	23411	#N/A	1.000000E+00
Mir133a-1	23412	#N/A	1.000000E+00
Mir133a-2	23413	#N/A	1.000000E+00
Mir133b	23414	#N/A	1.000000E+00
Mir134	23415	#N/A	1.000000E+00
Mir135a-1	23416	#N/A	1.000000E+00
Mir135a-2	23417	#N/A	1.000000E+00
Mir135b	23418	#N/A	1.000000E+00
Mir136	23419	#N/A	1.000000E+00
Mir137	23420	#N/A	1.000000E+00
Mir138-1	23421	#N/A	1.000000E+00
Mir138-2	23422	#N/A	1.000000E+00
Mir139	23423	#N/A	1.000000E+00
Mir140	23424	#N/A	1.000000E+00
Mir141	23425	#N/A	1.000000E+00
Mir142	23426	#N/A	1.000000E+00
Mir143	23427	#N/A	1.000000E+00
Mir144	23428	#N/A	1.000000E+00
Mir145	23429	#N/A	1.000000E+00
Mir146	23430	#N/A	1.000000E+00
Mir146b	23431	#N/A	1.000000E+00
Mir147	23432	#N/A	1.000000E+00
Mir148a	23433	#N/A	1.000000E+00
Mir148b	23434	#N/A	1.000000E+00

Spearman Rank correlation analysis performed between Prdm1 and all-expressed genes within the Meredith RNA-seq dataset. Robust Prdm1–associated genes were identified using a cut-off of $p < 0.0005$.

Table S1, Related to Supplemental Figure 3C. Prdm1 associated genes

Mir149	23435		#N/A	1.000000E+00
Mir150	23436		#N/A	1.000000E+00
Mir151	23437		#N/A	1.000000E+00
Mir152	23438		#N/A	1.000000E+00
Mir153	23439		#N/A	1.000000E+00
Mir154	23440		#N/A	1.000000E+00
Mir155	23441		#N/A	1.000000E+00
Mir15a	23442		#N/A	1.000000E+00
Mir15b	23443		#N/A	1.000000E+00
Mir16-1	23444		#N/A	1.000000E+00
Mir16-2	23445		#N/A	1.000000E+00
Mir17	23446		#N/A	1.000000E+00
Mir17hg	23447	0.001261726	5376	9.858529E-01
Mir18	23448		#N/A	1.000000E+00
Mir181a-1	23449		#N/A	1.000000E+00
Mir181a-2	23450		#N/A	1.000000E+00
Mir181b-1	23451		#N/A	1.000000E+00
Mir181b-2	23452		#N/A	1.000000E+00
Mir181c	23453		#N/A	1.000000E+00
Mir181d	23454		#N/A	1.000000E+00
Mir182	23455		#N/A	1.000000E+00
Mir183	23456		#N/A	1.000000E+00
Mir1839	23457		#N/A	1.000000E+00
Mir184	23458		#N/A	1.000000E+00
Mir185	23459		#N/A	1.000000E+00
Mir186	23460		#N/A	1.000000E+00
Mir187	23461		#N/A	1.000000E+00
Mir188	23462		#N/A	1.000000E+00
Mir1893	23463		#N/A	1.000000E+00
Mir1894	23464		#N/A	1.000000E+00
Mir1895	23465	-0.038406171	3065	5.892384E-01
Mir1896	23466		#N/A	1.000000E+00
Mir1897	23467		#N/A	1.000000E+00
Mir1898	23468		#N/A	1.000000E+00
Mir1899	23469		#N/A	1.000000E+00
Mir18b	23470		#N/A	1.000000E+00
Mir190	23471		#N/A	1.000000E+00
Mir1900	23472		#N/A	1.000000E+00
Mir1901	23473		#N/A	1.000000E+00
Mir1902	23474		#N/A	1.000000E+00
Mir1903	23475		#N/A	1.000000E+00
Mir1904	23476		#N/A	1.000000E+00
Mir1905	23477		#N/A	1.000000E+00

Spearman Rank correlation analysis performed between Prdm1 and all-expressed genes within the Meredith RNA-seq dataset. Robust Prdm1–associated genes were identified using a cut-off of $p < 0.0005$.

Table S1, Related to Supplemental Figure 3C. Prdm1 associated genes

Mir1906-1	23478		#N/A	1.000000E+00
Mir1906-2	23479		#N/A	1.000000E+00
Mir1907	23480		#N/A	1.000000E+00
Mir190b	23481		#N/A	1.000000E+00
Mir1912	23482		#N/A	1.000000E+00
Mir192	23483		#N/A	1.000000E+00
Mir1927	23484		#N/A	1.000000E+00
Mir1928	23485	0.113548679	13094	1.093908E-01
Mir1929	23486		#N/A	1.000000E+00
Mir193	23487		#N/A	1.000000E+00
Mir1930	23488		#N/A	1.000000E+00
Mir1931	23489	-0.054450825	1984	4.437997E-01
Mir1932	23490		#N/A	1.000000E+00
Mir1933	23491		#N/A	1.000000E+00
Mir1934	23492		#N/A	1.000000E+00
Mir1935	23493		#N/A	1.000000E+00
Mir1936	23494		#N/A	1.000000E+00
Mir1938	23495		#N/A	1.000000E+00
Mir193b	23496		#N/A	1.000000E+00
Mir194-1	23497		#N/A	1.000000E+00
Mir194-2	23498		#N/A	1.000000E+00
Mir1941	23499		#N/A	1.000000E+00
Mir1942	23500		#N/A	1.000000E+00
Mir1943	23501		#N/A	1.000000E+00
Mir1945	23502		#N/A	1.000000E+00
Mir1946a	23503	0.074945048	10235	2.915517E-01
Mir1946b	23504		#N/A	1.000000E+00
Mir1947	23505		#N/A	1.000000E+00
Mir1948	23506		#N/A	1.000000E+00
Mir1949	23507		#N/A	1.000000E+00
Mir195	23508		#N/A	1.000000E+00
Mir1950	23509		#N/A	1.000000E+00
Mir1951	23510		#N/A	1.000000E+00
Mir1952	23511		#N/A	1.000000E+00
Mir1953	23512		#N/A	1.000000E+00
Mir1954	23513		#N/A	1.000000E+00
Mir1955	23514		#N/A	1.000000E+00
Mir1956	23515		#N/A	1.000000E+00
Mir1957	23516		#N/A	1.000000E+00
Mir1958	23517		#N/A	1.000000E+00
Mir1960	23518		#N/A	1.000000E+00
Mir1961	23519		#N/A	1.000000E+00
Mir1962	23520		#N/A	1.000000E+00

Spearman Rank correlation analysis performed between Prdm1 and all-expressed genes within the Meredith RNA-seq dataset. Robust Prdm1–associated genes were identified using a cut-off of $p < 0.0005$.

Table S1, Related to Supplemental Figure 3C. Prdm1 associated genes

Mir1963	23521	#N/A	1.000000E+00
Mir1964	23522	#N/A	1.000000E+00
Mir1965	23523	#N/A	1.000000E+00
Mir1966	23524	#N/A	1.000000E+00
Mir1967	23525	#N/A	1.000000E+00
Mir1968	23526	#N/A	1.000000E+00
Mir1969	23527	#N/A	1.000000E+00
Mir196a-1	23528	#N/A	1.000000E+00
Mir196a-2	23529	#N/A	1.000000E+00
Mir196b	23530	#N/A	1.000000E+00
Mir1970	23531	#N/A	1.000000E+00
Mir1971	23532	#N/A	1.000000E+00
Mir1981	23533	#N/A	1.000000E+00
Mir1982	23534	#N/A	1.000000E+00
Mir1983	23535	#N/A	1.000000E+00
Mir199a-1	23536	#N/A	1.000000E+00
Mir199a-2	23537	#N/A	1.000000E+00
Mir199b	23538	#N/A	1.000000E+00
Mir19a	23539	#N/A	1.000000E+00
Mir19b-1	23540	#N/A	1.000000E+00
Mir19b-2	23541	#N/A	1.000000E+00
Mir1a-1	23542	#N/A	1.000000E+00
Mir1b	23543	#N/A	1.000000E+00
Mir200a	23544	#N/A	1.000000E+00
Mir200b	23545	#N/A	1.000000E+00
Mir200c	23546	#N/A	1.000000E+00
Mir201	23547	#N/A	1.000000E+00
Mir202	23548	#N/A	1.000000E+00
Mir203	23549	#N/A	1.000000E+00
Mir204	23550	#N/A	1.000000E+00
Mir205	23551	#N/A	1.000000E+00
Mir206	23552	#N/A	1.000000E+00
Mir207	23553	#N/A	1.000000E+00
Mir208a	23554	#N/A	1.000000E+00
Mir208b	23555	#N/A	1.000000E+00
Mir20a	23556	#N/A	1.000000E+00
Mir20b	23557	#N/A	1.000000E+00
Mir21	23558	#N/A	1.000000E+00
Mir210	23559	#N/A	1.000000E+00
Mir211	23560	#N/A	1.000000E+00
Mir212	23561	#N/A	1.000000E+00
Mir2136	23562	#N/A	1.000000E+00
Mir2137	23563	#N/A	1.000000E+00

Spearman Rank correlation analysis performed between Prdm1 and all-expressed genes within the Meredith RNA-seq dataset. Robust Prdm1–associated genes were identified using a cut-off of $p < 0.0005$.

Table S1, Related to Supplemental Figure 3C. Prdm1 associated genes

Mir2139	23564	#N/A	1.000000E+00
Mir214	23565	#N/A	1.000000E+00
Mir215	23566	#N/A	1.000000E+00
Mir216a	23567	#N/A	1.000000E+00
Mir216b	23568	#N/A	1.000000E+00
Mir217	23569	#N/A	1.000000E+00
Mir218-1	23570	#N/A	1.000000E+00
Mir218-2	23571	#N/A	1.000000E+00
Mir219-1	23572	#N/A	1.000000E+00
Mir219-2	23573	#N/A	1.000000E+00
Mir22	23574	#N/A	1.000000E+00
Mir221	23575	#N/A	1.000000E+00
Mir222	23576	#N/A	1.000000E+00
Mir223	23577	#N/A	1.000000E+00
Mir224	23578	#N/A	1.000000E+00
Mir22hg	23579	#N/A	1.000000E+00
Mir23a	23580	#N/A	1.000000E+00
Mir23b	23581	#N/A	1.000000E+00
Mir24-2	23582	#N/A	1.000000E+00
Mir25	23583	#N/A	1.000000E+00
Mir26a-1	23584	#N/A	1.000000E+00
Mir26a-2	23585	#N/A	1.000000E+00
Mir26b	23586	#N/A	1.000000E+00
Mir27a	23587	#N/A	1.000000E+00
Mir27b	23588	#N/A	1.000000E+00
Mir28	23589	#N/A	1.000000E+00
Mir2861	23590	#N/A	1.000000E+00
Mir28b	23591	#N/A	1.000000E+00
Mir290	23592	#N/A	1.000000E+00
Mir291a	23593	#N/A	1.000000E+00
Mir291b	23594	#N/A	1.000000E+00
Mir292	23595	#N/A	1.000000E+00
Mir293	23596	#N/A	1.000000E+00
Mir294	23597	#N/A	1.000000E+00
Mir295	23598	#N/A	1.000000E+00
Mir296	23599	#N/A	1.000000E+00
Mir297-1	23600	#N/A	1.000000E+00
Mir297a-3	23601	#N/A	1.000000E+00
Mir297a-4	23602	#N/A	1.000000E+00
Mir297b	23603	#N/A	1.000000E+00
Mir297c	23604	#N/A	1.000000E+00
Mir298	23605	#N/A	1.000000E+00
Mir29a	23606	#N/A	1.000000E+00

Spearman Rank correlation analysis performed between Prdm1 and all-expressed genes within the Meredith RNA-seq dataset. Robust Prdm1–associated genes were identified using a cut-off of $p < 0.0005$.

Table S1, Related to Supplemental Figure 3C. Prdm1 associated genes

Mir29b-1	23607		#N/A	1.000000E+00
Mir29b-2	23608		#N/A	1.000000E+00
Mir29c	23609		#N/A	1.000000E+00
Mir300	23610		#N/A	1.000000E+00
Mir301	23611		#N/A	1.000000E+00
Mir301b	23612		#N/A	1.000000E+00
Mir302a	23613		#N/A	1.000000E+00
Mir302b	23614		#N/A	1.000000E+00
Mir302c	23615		#N/A	1.000000E+00
Mir302d	23616		#N/A	1.000000E+00
Mir3057	23617		#N/A	1.000000E+00
Mir3058	23618		#N/A	1.000000E+00
Mir3059	23619		#N/A	1.000000E+00
Mir3060	23620		#N/A	1.000000E+00
Mir3061	23621		#N/A	1.000000E+00
Mir3062	23622		#N/A	1.000000E+00
Mir3063	23623		#N/A	1.000000E+00
Mir3064	23624		#N/A	1.000000E+00
Mir3066	23625		#N/A	1.000000E+00
Mir3067	23626		#N/A	1.000000E+00
Mir3068	23627		#N/A	1.000000E+00
Mir3070a	23628		#N/A	1.000000E+00
Mir3070b	23629		#N/A	1.000000E+00
Mir3072	23630		#N/A	1.000000E+00
Mir3073	23631		#N/A	1.000000E+00
Mir3074-1	23632		#N/A	1.000000E+00
Mir3075	23633		#N/A	1.000000E+00
Mir3076	23634		#N/A	1.000000E+00
Mir3077	23635		#N/A	1.000000E+00
Mir3078	23636		#N/A	1.000000E+00
Mir3079	23637		#N/A	1.000000E+00
Mir3080	23638		#N/A	1.000000E+00
Mir3081	23639		#N/A	1.000000E+00
Mir3082	23640		#N/A	1.000000E+00
Mir3083	23641		#N/A	1.000000E+00
Mir3085	23642		#N/A	1.000000E+00
Mir3086	23643		#N/A	1.000000E+00
Mir3088	23644		#N/A	1.000000E+00
Mir3089	23645		#N/A	1.000000E+00
Mir3090	23646	0.164478601	16623.5	1.994547E-02
Mir3091	23647		#N/A	1.000000E+00
Mir3092	23648		#N/A	1.000000E+00
Mir3093	23649		#N/A	1.000000E+00

Spearman Rank correlation analysis performed between Prdm1 and all-expressed genes within the Meredith RNA-seq dataset. Robust Prdm1–associated genes were identified using a cut-off of $p < 0.0005$.

Table S1, Related to Supplemental Figure 3C. Prdm1 associated genes

Mir3094	23650	#N/A	1.000000E+00
Mir3095	23651	#N/A	1.000000E+00
Mir3096	23652	#N/A	1.000000E+00
Mir3097	23653	#N/A	1.000000E+00
Mir3098	23654	#N/A	1.000000E+00
Mir3099	23655	#N/A	1.000000E+00
Mir30a	23656	#N/A	1.000000E+00
Mir30b	23657	#N/A	1.000000E+00
Mir30c-1	23658	#N/A	1.000000E+00
Mir30c-2	23659	#N/A	1.000000E+00
Mir30d	23660	#N/A	1.000000E+00
Mir30e	23661	#N/A	1.000000E+00
Mir31	23662	#N/A	1.000000E+00
Mir3100	23663	#N/A	1.000000E+00
Mir3101	23664	#N/A	1.000000E+00
Mir3102	23665	#N/A	1.000000E+00
Mir3103	23666	#N/A	1.000000E+00
Mir3104	23667	#N/A	1.000000E+00
Mir3105	23668	#N/A	1.000000E+00
Mir3106	23669	#N/A	1.000000E+00
Mir3108	23670	#N/A	1.000000E+00
Mir3109	23671	#N/A	1.000000E+00
Mir3110	23672	#N/A	1.000000E+00
Mir3112	23673	#N/A	1.000000E+00
Mir3113	23674	#N/A	1.000000E+00
Mir32	23675	#N/A	1.000000E+00
Mir320	23676	#N/A	1.000000E+00
Mir322	23677	#N/A	1.000000E+00
Mir323	23678	#N/A	1.000000E+00
Mir324	23679	#N/A	1.000000E+00
Mir325	23680	#N/A	1.000000E+00
Mir326	23681	#N/A	1.000000E+00
Mir328	23682	#N/A	1.000000E+00
Mir329	23683	#N/A	1.000000E+00
Mir33	23684	#N/A	1.000000E+00
Mir330	23685	#N/A	1.000000E+00
Mir331	23686	#N/A	1.000000E+00
Mir335	23687	#N/A	1.000000E+00
Mir337	23688	#N/A	1.000000E+00
Mir338	23689	#N/A	1.000000E+00
Mir339	23690	#N/A	1.000000E+00
Mir340	23691	#N/A	1.000000E+00
Mir341	23692	#N/A	1.000000E+00

Spearman Rank correlation analysis performed between Prdm1 and all-expressed genes within the Meredith RNA-seq dataset. Robust Prdm1–associated genes were identified using a cut-off of $p < 0.0005$.

Table S1, Related to Supplemental Figure 3C. Prdm1 associated genes

Mir342	23693		#N/A	1.000000E+00
Mir343	23694		#N/A	1.000000E+00
Mir344	23695		#N/A	1.000000E+00
Mir344-2	23696		#N/A	1.000000E+00
Mir344b	23697		#N/A	1.000000E+00
Mir344c	23698		#N/A	1.000000E+00
Mir344d-1	23699		#N/A	1.000000E+00
Mir344d-2	23700		#N/A	1.000000E+00
Mir344d-3	23701		#N/A	1.000000E+00
Mir344e	23702		#N/A	1.000000E+00
Mir344f	23703		#N/A	1.000000E+00
Mir344g	23704		#N/A	1.000000E+00
Mir345	23705		#N/A	1.000000E+00
Mir346	23706		#N/A	1.000000E+00
Mir3470a	23707		#N/A	1.000000E+00
Mir3470b	23708		#N/A	1.000000E+00
Mir3471-1	23709		#N/A	1.000000E+00
Mir3471-2	23710		#N/A	1.000000E+00
Mir3472	23711		#N/A	1.000000E+00
Mir3473	23712		#N/A	1.000000E+00
Mir3473b	23713		#N/A	1.000000E+00
Mir3473c	23714		#N/A	1.000000E+00
Mir3473d	23715		#N/A	1.000000E+00
Mir3474	23716		#N/A	1.000000E+00
Mir3475	23717		#N/A	1.000000E+00
Mir34a	23718		#N/A	1.000000E+00
Mir34b	23719		#N/A	1.000000E+00
Mir34c	23720		#N/A	1.000000E+00
Mir350	23721		#N/A	1.000000E+00
Mir351	23722	0.063087476	9394	3.748185E-01
Mir3572	23723		#N/A	1.000000E+00
Mir361	23724		#N/A	1.000000E+00
Mir362	23725		#N/A	1.000000E+00
Mir363	23726		#N/A	1.000000E+00
Mir365-1	23727		#N/A	1.000000E+00
Mir365-2	23728		#N/A	1.000000E+00
Mir367	23729		#N/A	1.000000E+00
Mir369	23730		#N/A	1.000000E+00
Mir370	23731		#N/A	1.000000E+00
Mir374	23732		#N/A	1.000000E+00
Mir375	23733		#N/A	1.000000E+00
Mir376b	23734		#N/A	1.000000E+00
Mir377	23735		#N/A	1.000000E+00

Spearman Rank correlation analysis performed between Prdm1 and all-expressed genes within the Meredith RNA-seq dataset. Robust Prdm1–associated genes were identified using a cut-off of $p < 0.0005$.

Table S1, Related to Supplemental Figure 3C. Prdm1 associated genes

Mir378b	23736	#N/A	1.000000E+00
Mir379	23737	#N/A	1.000000E+00
Mir381	23738	#N/A	1.000000E+00
Mir382	23739	#N/A	1.000000E+00
Mir383	23740	#N/A	1.000000E+00
Mir384	23741	#N/A	1.000000E+00
Mir3960	23742	#N/A	1.000000E+00
Mir3962	23743	#N/A	1.000000E+00
Mir3963	23744	#N/A	1.000000E+00
Mir3964	23745	#N/A	1.000000E+00
Mir3965	23746	#N/A	1.000000E+00
Mir3968	23747	#N/A	1.000000E+00
Mir3971	23748	#N/A	1.000000E+00
Mir410	23749	#N/A	1.000000E+00
Mir411	23750	#N/A	1.000000E+00
Mir412	23751	#N/A	1.000000E+00
Mir421	23752	#N/A	1.000000E+00
Mir423	23753	#N/A	1.000000E+00
Mir425	23754	#N/A	1.000000E+00
Mir429	23755	#N/A	1.000000E+00
Mir431	23756	#N/A	1.000000E+00
Mir432	23757	#N/A	1.000000E+00
Mir433	23758	#N/A	1.000000E+00
Mir434	23759	#N/A	1.000000E+00
Mir446q	23760	#N/A	1.000000E+00
Mir448	23761	#N/A	1.000000E+00
Mir449a	23762	#N/A	1.000000E+00
Mir449b	23763	#N/A	1.000000E+00
Mir449c	23764	#N/A	1.000000E+00
Mir450-1	23765	#N/A	1.000000E+00
Mir450-2	23766	#N/A	1.000000E+00
Mir450b	23767	#N/A	1.000000E+00
Mir451	23768	#N/A	1.000000E+00
Mir452	23769	#N/A	1.000000E+00
Mir453	23770	#N/A	1.000000E+00
Mir455	23771	#N/A	1.000000E+00
Mir463	23772	#N/A	1.000000E+00
Mir465	23773	#N/A	1.000000E+00
Mir465b-1	23774	#N/A	1.000000E+00
Mir465b-2	23775	#N/A	1.000000E+00
Mir465c-1	23776	#N/A	1.000000E+00
Mir465c-2	23777	#N/A	1.000000E+00
Mir466	23778	#N/A	1.000000E+00

Spearman Rank correlation analysis performed between Prdm1 and all-expressed genes within the Meredith RNA-seq dataset. Robust Prdm1–associated genes were identified using a cut-off of $p < 0.0005$.

Table S1, Related to Supplemental Figure 3C. Prdm1 associated genes

Mir4660	23779		#N/A	1.000000E+00
Mir466b-1	23780		#N/A	1.000000E+00
Mir466b-2	23781		#N/A	1.000000E+00
Mir466b-3	23782		#N/A	1.000000E+00
Mir466b-4	23783		#N/A	1.000000E+00
Mir466b-5	23784		#N/A	1.000000E+00
Mir466b-6	23785		#N/A	1.000000E+00
Mir466b-7	23786		#N/A	1.000000E+00
Mir466b-8	23787		#N/A	1.000000E+00
Mir466c-1	23788		#N/A	1.000000E+00
Mir466c-2	23789		#N/A	1.000000E+00
Mir466d	23790		#N/A	1.000000E+00
Mir466e	23791		#N/A	1.000000E+00
Mir466f-1	23792		#N/A	1.000000E+00
Mir466f-2	23793		#N/A	1.000000E+00
Mir466f-3	23794		#N/A	1.000000E+00
Mir466f-4	23795		#N/A	1.000000E+00
Mir466g	23796		#N/A	1.000000E+00
Mir466h	23797		#N/A	1.000000E+00
Mir466i	23798	0.113548679	13094	1.093908E-01
Mir466j	23799		#N/A	1.000000E+00
Mir466k	23800		#N/A	1.000000E+00
Mir466l	23801		#N/A	1.000000E+00
Mir466m	23802		#N/A	1.000000E+00
Mir466n	23803		#N/A	1.000000E+00
Mir466p	23804		#N/A	1.000000E+00
Mir467a-1	23805		#N/A	1.000000E+00
Mir467a-10	23806		#N/A	1.000000E+00
Mir467a-2	23807		#N/A	1.000000E+00
Mir467a-3	23808		#N/A	1.000000E+00
Mir467a-4	23809		#N/A	1.000000E+00
Mir467a-5	23810		#N/A	1.000000E+00
Mir467a-6	23811		#N/A	1.000000E+00
Mir467a-7	23812		#N/A	1.000000E+00
Mir467a-8	23813		#N/A	1.000000E+00
Mir467a-9	23814		#N/A	1.000000E+00
Mir467b	23815		#N/A	1.000000E+00
Mir467c	23816		#N/A	1.000000E+00
Mir467d	23817		#N/A	1.000000E+00
Mir467e	23818		#N/A	1.000000E+00
Mir467f	23819		#N/A	1.000000E+00
Mir467g	23820		#N/A	1.000000E+00
Mir467h	23821		#N/A	1.000000E+00

Spearman Rank correlation analysis performed between Prdm1 and all-expressed genes within the Meredith RNA-seq dataset. Robust Prdm1–associated genes were identified using a cut-off of $p < 0.0005$.

Table S1, Related to Supplemental Figure 3C. Prdm1 associated genes

Mir470	23822		#N/A	1.000000E+00
Mir471	23823		#N/A	1.000000E+00
Mir483	23824		#N/A	1.000000E+00
Mir484	23825		#N/A	1.000000E+00
Mir485	23826		#N/A	1.000000E+00
Mir486	23827		#N/A	1.000000E+00
Mir487b	23828		#N/A	1.000000E+00
Mir488	23829		#N/A	1.000000E+00
Mir489	23830		#N/A	1.000000E+00
Mir490	23831		#N/A	1.000000E+00
Mir491	23832		#N/A	1.000000E+00
Mir493	23833		#N/A	1.000000E+00
Mir494	23834		#N/A	1.000000E+00
Mir495	23835		#N/A	1.000000E+00
Mir496	23836		#N/A	1.000000E+00
Mir497	23837		#N/A	1.000000E+00
Mir499	23838		#N/A	1.000000E+00
Mir500	23839		#N/A	1.000000E+00
Mir501	23840		#N/A	1.000000E+00
Mir503	23841		#N/A	1.000000E+00
Mir504	23842		#N/A	1.000000E+00
Mir505	23843		#N/A	1.000000E+00
Mir5097	23844		#N/A	1.000000E+00
Mir5098	23845		#N/A	1.000000E+00
Mir5099	23846		#N/A	1.000000E+00
Mir5100	23847		#N/A	1.000000E+00
Mir5101	23848		#N/A	1.000000E+00
Mir5103	23849		#N/A	1.000000E+00
Mir5104	23850		#N/A	1.000000E+00
Mir5105	23851	0.051505688	8427	4.688719E-01
Mir5106	23852		#N/A	1.000000E+00
Mir5107	23853		#N/A	1.000000E+00
Mir5108	23854		#N/A	1.000000E+00
Mir5109	23855	0.111066005	12793	1.174158E-01
Mir511	23856		#N/A	1.000000E+00
Mir5110	23857		#N/A	1.000000E+00
Mir5112	23858		#N/A	1.000000E+00
Mir5113	23859		#N/A	1.000000E+00
Mir5114	23860		#N/A	1.000000E+00
Mir5115	23861		#N/A	1.000000E+00
Mir5116	23862		#N/A	1.000000E+00
Mir5117	23863	-0.066856562	1139	3.468991E-01
Mir5118	23864		#N/A	1.000000E+00

Spearman Rank correlation analysis performed between Prdm1 and all-expressed genes within the Meredith RNA-seq dataset. Robust Prdm1–associated genes were identified using a cut-off of $p < 0.0005$.

Table S1, Related to Supplemental Figure 3C. Prdm1 associated genes

Mir5119	23865	#N/A	1.000000E+00
Mir5120	23866	#N/A	1.000000E+00
Mir5122	23867	#N/A	1.000000E+00
Mir5123	23868	#N/A	1.000000E+00
Mir5124	23869	#N/A	1.000000E+00
Mir5125	23870	#N/A	1.000000E+00
Mir5126	23871	#N/A	1.000000E+00
Mir5127	23872	#N/A	1.000000E+00
Mir5128	23873	#N/A	1.000000E+00
Mir5129	23874	#N/A	1.000000E+00
Mir5130	23875	#N/A	1.000000E+00
Mir5131	23876	#N/A	1.000000E+00
Mir5132	23877	#N/A	1.000000E+00
Mir5133	23878	#N/A	1.000000E+00
Mir5134	23879	#N/A	1.000000E+00
Mir5135	23880	#N/A	1.000000E+00
Mir5136	23881	#N/A	1.000000E+00
Mir532	23882	#N/A	1.000000E+00
Mir539	23883	#N/A	1.000000E+00
Mir540	23884	#N/A	1.000000E+00
Mir541	23885	#N/A	1.000000E+00
Mir542	23886	#N/A	1.000000E+00
Mir543	23887	#N/A	1.000000E+00
Mir544	23888	#N/A	1.000000E+00
Mir546	23889	#N/A	1.000000E+00
Mir547	23890	#N/A	1.000000E+00
Mir551b	23891	#N/A	1.000000E+00
Mir568	23892	#N/A	1.000000E+00
Mir574	23893	#N/A	1.000000E+00
Mir582	23894	#N/A	1.000000E+00
Mir592	23895	#N/A	1.000000E+00
Mir598	23896	#N/A	1.000000E+00
Mir599	23897	#N/A	1.000000E+00
Mir615	23898	#N/A	1.000000E+00
Mir652	23899	#N/A	1.000000E+00
Mir653	23900	#N/A	1.000000E+00
Mir654	23901	#N/A	1.000000E+00
Mir665	23902	#N/A	1.000000E+00
Mir666	23903	#N/A	1.000000E+00
Mir667	23904	#N/A	1.000000E+00
Mir669a-1	23905	#N/A	1.000000E+00
Mir669a-10	23906	#N/A	1.000000E+00
Mir669a-11	23907	#N/A	1.000000E+00

Spearman Rank correlation analysis performed between Prdm1 and all-expressed genes within the Meredith RNA-seq dataset. Robust Prdm1–associated genes were identified using a cut-off of $p < 0.0005$.

Table S1, Related to Supplemental Figure 3C. Prdm1 associated genes

Mir669a-12	23908		#N/A	1.000000E+00
Mir669a-2	23909		#N/A	1.000000E+00
Mir669a-3	23910		#N/A	1.000000E+00
Mir669a-4	23911		#N/A	1.000000E+00
Mir669a-5	23912		#N/A	1.000000E+00
Mir669a-6	23913		#N/A	1.000000E+00
Mir669a-7	23914		#N/A	1.000000E+00
Mir669a-8	23915		#N/A	1.000000E+00
Mir669a-9	23916		#N/A	1.000000E+00
Mir669b	23917		#N/A	1.000000E+00
Mir669c	23918		#N/A	1.000000E+00
Mir669d	23919		#N/A	1.000000E+00
Mir669d-2	23920		#N/A	1.000000E+00
Mir669e	23921		#N/A	1.000000E+00
Mir669f	23922		#N/A	1.000000E+00
Mir669g	23923		#N/A	1.000000E+00
Mir669h	23924		#N/A	1.000000E+00
Mir669i	23925		#N/A	1.000000E+00
Mir669j	23926		#N/A	1.000000E+00
Mir669k	23927		#N/A	1.000000E+00
Mir669l	23928		#N/A	1.000000E+00
Mir669m-1	23929		#N/A	1.000000E+00
Mir669m-2	23930		#N/A	1.000000E+00
Mir669n	23931		#N/A	1.000000E+00
Mir669o	23932		#N/A	1.000000E+00
Mir669p-1	23933		#N/A	1.000000E+00
Mir669p-2	23934		#N/A	1.000000E+00
Mir670	23935	-0.054451513	1703	4.437940E-01
Mir671	23936		#N/A	1.000000E+00
Mir672	23937		#N/A	1.000000E+00
Mir673	23938		#N/A	1.000000E+00
Mir674	23939		#N/A	1.000000E+00
Mir675	23940		#N/A	1.000000E+00
Mir676	23941		#N/A	1.000000E+00
Mir677	23942		#N/A	1.000000E+00
Mir678	23943		#N/A	1.000000E+00
Mir679	23944		#N/A	1.000000E+00
Mir680-1	23945		#N/A	1.000000E+00
Mir680-2	23946		#N/A	1.000000E+00
Mir680-3	23947		#N/A	1.000000E+00
Mir681	23948		#N/A	1.000000E+00
Mir682	23949		#N/A	1.000000E+00
Mir683-1	23950		#N/A	1.000000E+00

Spearman Rank correlation analysis performed between Prdm1 and all-expressed genes within the Meredith RNA-seq dataset. Robust Prdm1–associated genes were identified using a cut-off of $p < 0.0005$.

Table S1, Related to Supplemental Figure 3C. Prdm1 associated genes

Mir683-2	23951		#N/A	1.000000E+00
Mir686	23952	0.07440756	10172	2.950358E-01
Mir687	23953		#N/A	1.000000E+00
Mir688	23954		#N/A	1.000000E+00
Mir690	23955	-0.076995145	841	2.785155E-01
Mir692-1	23956	0.050677899	8356	4.760565E-01
Mir692-2a	23957		#N/A	1.000000E+00
Mir692-2b	23958		#N/A	1.000000E+00
Mir693	23959		#N/A	1.000000E+00
Mir694	23960		#N/A	1.000000E+00
Mir695	23961		#N/A	1.000000E+00
Mir697	23962		#N/A	1.000000E+00
Mir698	23963		#N/A	1.000000E+00
Mir7-1	23964		#N/A	1.000000E+00
Mir7-2	23965		#N/A	1.000000E+00
Mir700	23966		#N/A	1.000000E+00
Mir701	23967		#N/A	1.000000E+00
Mir702	23968		#N/A	1.000000E+00
Mir704	23969		#N/A	1.000000E+00
Mir705	23970		#N/A	1.000000E+00
Mir706	23971		#N/A	1.000000E+00
Mir707	23972		#N/A	1.000000E+00
Mir708	23973		#N/A	1.000000E+00
Mir709	23974		#N/A	1.000000E+00
Mir710	23975		#N/A	1.000000E+00
Mir711	23976		#N/A	1.000000E+00
Mir713	23977		#N/A	1.000000E+00
Mir717	23978		#N/A	1.000000E+00
Mir718	23979		#N/A	1.000000E+00
Mir719	23980		#N/A	1.000000E+00
Mir741	23981		#N/A	1.000000E+00
Mir742	23982		#N/A	1.000000E+00
Mir743	23983		#N/A	1.000000E+00
Mir743b	23984		#N/A	1.000000E+00
Mir744	23985		#N/A	1.000000E+00
Mir758	23986		#N/A	1.000000E+00
Mir759	23987		#N/A	1.000000E+00
Mir760	23988		#N/A	1.000000E+00
Mir761	23989		#N/A	1.000000E+00
Mir762	23990		#N/A	1.000000E+00
Mir763	23991		#N/A	1.000000E+00
Mir764	23992		#N/A	1.000000E+00
Mir767	23993		#N/A	1.000000E+00

Spearman Rank correlation analysis performed between Prdm1 and all-expressed genes within the Meredith RNA-seq dataset. Robust Prdm1–associated genes were identified using a cut-off of $p < 0.0005$.

Table S1, Related to Supplemental Figure 3C. Prdm1 associated genes

Mir770	23994		#N/A	1.000000E+00
Mir7b	23995		#N/A	1.000000E+00
Mir802	23996		#N/A	1.000000E+00
Mir804	23997		#N/A	1.000000E+00
Mir871	23998		#N/A	1.000000E+00
Mir872	23999		#N/A	1.000000E+00
Mir873	24000		#N/A	1.000000E+00
Mir874	24001		#N/A	1.000000E+00
Mir875	24002		#N/A	1.000000E+00
Mir876	24003		#N/A	1.000000E+00
Mir877	24004		#N/A	1.000000E+00
Mir878	24005		#N/A	1.000000E+00
Mir879	24006		#N/A	1.000000E+00
Mir880	24007		#N/A	1.000000E+00
Mir881	24008		#N/A	1.000000E+00
Mir882	24009		#N/A	1.000000E+00
Mir883a	24010		#N/A	1.000000E+00
Mir883b	24011		#N/A	1.000000E+00
Mir9-2	24012		#N/A	1.000000E+00
Mir9-3	24013		#N/A	1.000000E+00
Mir92-1	24014		#N/A	1.000000E+00
Mir92-2	24015		#N/A	1.000000E+00
Mir92b	24016		#N/A	1.000000E+00
Mir93	24017		#N/A	1.000000E+00
Mir96	24018		#N/A	1.000000E+00
Mir98	24019		#N/A	1.000000E+00
Mir99a	24020		#N/A	1.000000E+00
Mir99b	24021		#N/A	1.000000E+00
Mirg	24022	0.011208627	5838	8.748309E-01
Mirlet7a-1	24023		#N/A	1.000000E+00
Mirlet7a-2	24024		#N/A	1.000000E+00
Mirlet7b	24025		#N/A	1.000000E+00
Mirlet7c-1	24026		#N/A	1.000000E+00
Mirlet7c-2	24027		#N/A	1.000000E+00
Mirlet7d	24028		#N/A	1.000000E+00
Mirlet7f-2	24029		#N/A	1.000000E+00
Mirlet7g	24030		#N/A	1.000000E+00
Mirlet7i	24031		#N/A	1.000000E+00
Mis12	24032	0.087926745	11198	2.156875E-01
Mis18a	24033	0.158533508	16204	2.495181E-02
Mis18bp1	24034	0.210290348	18585	2.800688E-03
Mitd1	24035	0.088252209	11226	2.139866E-01
Mitf	24036	0.166592171	16753	1.838848E-02

Spearman Rank correlation analysis performed between Prdm1 and all-expressed genes within the Meredith RNA-seq dataset. Robust Prdm1–associated genes were identified using a cut-off of $p < 0.0005$.

Table S1, Related to Supplemental Figure 3C. Prdm1 associated genes

Mixl1	24037	0.14908383	15596	3.512301E-02
Mki67	24038	0.168029746	16825	1.739080E-02
Mki67ip	24039	0.058635532	9137	4.095170E-01
Mkks	24040	0.05949453	9185	4.026783E-01
Mkl1	24041	0.105153165	12367	1.383721E-01
Mkl2	24042	0.198389384	18179	4.860906E-03
Mkln1	24043	0.168812476	16867	1.686765E-02
Mknk1	24044	0.061806877	9323	3.846098E-01
Mknk2	24045	0.198882104	18194	4.753997E-03
Mkrn1	24046	0.007530036	5658	9.157214E-01
Mkrn1-ps1	24047		#N/A	1.000000E+00
Mkrn2	24048	0.076513676	10382	2.815411E-01
Mkrn3	24049	0.074574898	10201.5	2.939481E-01
Mks1	24050	0.122890551	13835	8.298579E-02
Mkx	24051	0.15636436	16075	2.703003E-02
Mlana	24052	-0.038406171	3065	5.892384E-01
Mlc1	24053	-0.054450825	1984	4.437997E-01
Mlec	24054	0.146917479	15475	3.789561E-02
Mlf1	24055	-0.027401411	4020	7.001205E-01
Mlf1ip	24056	0.042119952	7747	5.537198E-01
Mlf2	24057	0.102117377	12187	1.501874E-01
Mlh1	24058	-0.016332858	4460	8.184422E-01
Mlh3	24059	0.078728032	10514	2.678080E-01
Mlip	24060	0.007752874	5669	9.132369E-01
Mlkl	24061	0.195701434	18072	5.483043E-03
Mll1	24062	-0.026865227	4035	7.057141E-01
Mll2	24063	0.112138575	12864	1.138943E-01
Mll3	24064	0.12630427	14064	7.472315E-02
Mll5	24065	0.238357414	19336	6.766337E-04
Mllt1	24066	0.154981492	15976	2.843093E-02
Mllt10	24067	0.152614351	15798	3.097307E-02
Mllt11	24068	-0.006495598	4868	9.272647E-01
Mllt3	24069	0.143721297	15290	4.232210E-02
Mllt4	24070	0.126770586	14090	7.364812E-02
Mllt6	24071	0.027218858	6940	7.020231E-01
Mlph	24072	0.200494944	18261	4.418659E-03
Mlst8	24073	0.086265016	11070	2.245224E-01
Mlx	24074	0.143980227	15312	4.194802E-02
Mlxip	24075	0.126230995	14059	7.489322E-02
Mlxipl	24076	0.041775741	7730	5.569676E-01
Mlycd	24077	-0.026278551	4056	7.118525E-01
Mmaa	24078	0.028359787	6986	6.901627E-01
Mmab	24079	0.0224558	6659	7.522893E-01

Spearman Rank correlation analysis performed between Prdm1 and all-expressed genes within the Meredith RNA-seq dataset. Robust Prdm1–associated genes were identified using a cut-off of $p < 0.0005$.

Table S1, Related to Supplemental Figure 3C. Prdm1 associated genes

Mmachc	24080	0.062361487	9347	3.803504E-01
Mmadhc	24081	0.178791406	17345	1.130627E-02
Mmd	24082	0.133742164	14531	5.902122E-02
Mmd2	24083	-0.006349394	4871	9.288975E-01
Mme	24084	0.065081882	9540	3.598773E-01
Mmel1	24085	-0.033702148	3752	6.356657E-01
Mmgt1	24086	0.202880534	18351	3.961585E-03
Mmgt2	24087	0.197272904	18133	5.111185E-03
Mmp10	24088		#N/A	1.000000E+00
Mmp11	24089	0.152789767	15822	3.077826E-02
Mmp12	24090	0.011635093	5858.5	8.701096E-01
Mmp13	24091	0.119819531	13655	9.103243E-02
Mmp14	24092	0.097787179	11891	1.683407E-01
Mmp15	24093	0.096919217	11831	1.721683E-01
Mmp16	24094	0.000873372	5353	9.902071E-01
Mmp17	24095	0.053805701	8820	4.492258E-01
Mmp19	24096	0.250461861	19511	3.474961E-04
Mmp1a	24097	-0.038406171	3065	5.892384E-01
Mmp1b	24098	-0.038406171	3065	5.892384E-01
Mmp2	24099	-0.030875624	3897.5	6.642784E-01
Mmp20	24100	0.130651242	14333	6.518048E-02
Mmp21	24101	-0.038406171	3065	5.892384E-01
Mmp23	24102	0.007386486	5651	9.173222E-01
Mmp24	24103	0.035918819	7375	6.135955E-01
Mmp25	24104	0.021508807	6555	7.624166E-01
Mmp27	24105		#N/A	1.000000E+00
Mmp28	24106	0.03847064	7526	5.886130E-01
Mmp3	24107		#N/A	1.000000E+00
Mmp7	24108	0.08393014	10915	2.373654E-01
Mmp8	24109	0.211648697	18639	2.624881E-03
Mmp9	24110	0.044789389	7919	5.288493E-01
Mmrn1	24111	0.014039659	5990.5	8.435803E-01
Mmrn2	24112	0.204234431	18398	3.721566E-03
Mms19	24113	0.139779045	14938	4.837199E-02
Mms22l	24114	0.040340462	7651	5.706087E-01
mmu-let-7d	24115	0.04457998	7906	5.307798E-01
mmu-mir-137	24116	-0.040476297	2464	5.693109E-01
mmu-mir-155	24117	0.020875719	6412.5	7.692098E-01
mmu-mir-181b	24118	-0.054450825	1984	4.437997E-01
mmu-mir-1839	24119	0.0723941	10020	3.083325E-01
mmu-mir-1900	24120	0.232507492	19219	9.227975E-04
mmu-mir-1906	24121	0.100414522	12086	1.571409E-01
mmu-mir-205	24122	0.044495949	7898.5	5.315554E-01

Spearman Rank correlation analysis performed between Prdm1 and all-expressed genes within the Meredith RNA-seq dataset. Robust Prdm1–associated genes were identified using a cut-off of $p < 0.0005$.

Table S1, Related to Supplemental Figure 3C. Prdm1 associated genes

mmu-mir-290	24123		#N/A	1.000000E+00
mmu-mir-291a	24124		#N/A	1.000000E+00
mmu-mir-291b	24125		#N/A	1.000000E+00
mmu-mir-292	24126		#N/A	1.000000E+00
mmu-mir-293	24127		#N/A	1.000000E+00
mmu-mir-294	24128		#N/A	1.000000E+00
mmu-mir-295	24129		#N/A	1.000000E+00
mmu-mir-29b-:	24130	-0.076532927	850	2.814197E-01
mmu-mir-3061	24131		#N/A	1.000000E+00
mmu-mir-3078	24132		#N/A	1.000000E+00
mmu-mir-30c-:	24133		#N/A	1.000000E+00
mmu-mir-706	24134		#N/A	1.000000E+00
Mn1	24135	0.342568871	19997	6.857601E-07
Mnat1	24136	-0.029692069	3942	6.764083E-01
Mnd1	24137	-0.033398428	3765	6.387155E-01
Mnda	24138	0.158487439	16202	2.499448E-02
Mndal	24139	0.284105503	19826	4.561953E-05
Mnf1	24140	0.117244255	13477	9.824978E-02
Mns1	24141	0.092186607	11530	1.941808E-01
Mnt	24142	-0.002950369	5026	9.669267E-01
Mnx1	24143	0.075711049	10308	2.866340E-01
Moap1	24144		#N/A	1.000000E+00
Mob1a	24145	0.058146126	9099	4.134438E-01
Mob1b	24146	0.037074874	7435	6.022200E-01
Mob2	24147	-0.005220127	4919	9.415184E-01
Mob3a	24148	0.073215449	10081	3.028616E-01
Mob3b	24149	0.222419591	18967	1.548066E-03
Mob3c	24150	0.10111107	12123	1.542679E-01
Mob4	24151	0.189591095	17824	7.169905E-03
Mobp	24152	0.152674964	15804	3.090564E-02
Mocos	24153	0.119072063	13594	9.308227E-02
Mocs1	24154	0.101156443	12127	1.540821E-01
Mocs2	24155	0.026285695	6886	7.117777E-01
Mocs3	24156	0.039052459	7582	5.829828E-01
Mog	24157	-0.038406171	3065	5.892384E-01
Mogat1	24158	0.307959932	19934	9.155791E-06
Mogat2	24159	0.031913193	7181	6.537158E-01
Mogs	24160	0.177307687	17283	1.201425E-02
Mon1a	24161	0.074839684	10227	2.922325E-01
Mon1b	24162	-0.038406171	3065	5.892384E-01
Mon2	24163	0.118311118	13548	9.520665E-02
Morc1	24164	0.087326583	11150	2.188492E-01
Morc2a	24165	0.160386607	16358	2.328664E-02

Spearman Rank correlation analysis performed between Prdm1 and all-expressed genes within the Meredith RNA-seq dataset. Robust Prdm1–associated genes were identified using a cut-off of $p < 0.0005$.

Table S1, Related to Supplemental Figure 3C. Prdm1 associated genes

Morc2b	24166	0.113548679	13094	1.093908E-01
Morc3	24167	0.137610813	14797	5.199599E-02
Morc4	24168	-0.001453505	5120	9.837029E-01
Morf4l1	24169	0.160799153	16380	2.292924E-02
Morf4l2	24170	0.246358174	19459	4.371962E-04
Morn1	24171	-0.146749886	32	3.811759E-02
Morn2	24172	0.054046179	8868	4.471988E-01
Morn3	24173		#N/A	1.000000E+00
Morn4	24174	-0.024464584	4117	7.309483E-01
Morn5	24175	0.143605682	15224	4.249002E-02
Mos	24176		#N/A	1.000000E+00
Mospd1	24177	-0.076132004	859	2.839553E-01
Mospd2	24178	0.08182781	10732	2.493625E-01
Mospd3	24179	0.194857639	18036	5.692552E-03
Mospd4	24180	-0.054450825	1984	4.437997E-01
Mov10	24181	0.204306093	18401	3.709236E-03
Mov10l1	24182	-0.095267847	349	1.796284E-01
Moxd1	24183	-0.066855996	1239.5	3.469032E-01
Moxd2	24184		#N/A	1.000000E+00
Mpc1	24185	0.081755403	10728	2.497830E-01
Mpc1-ps	24186	0.005385758	5577	9.396663E-01
Mpc2	24187	0.132066288	14415	6.229838E-02
Mpdu1	24188	-0.164920654	8	1.961070E-02
Mpdz	24189	0.173373292	17095	1.408369E-02
Mpeg1	24190	0.109608145	12684	1.223376E-01
Mpg	24191	0.174546154	17146	1.343636E-02
Mphosph10	24192	0.008328754	5693	9.068204E-01
Mphosph6	24193	0.047992291	8149	4.997722E-01
Mphosph8	24194	0.111790443	12836	1.150281E-01
Mphosph9	24195	-0.080820818	633	2.552554E-01
Mpi	24196	0.149599827	15623	3.448856E-02
Mpl	24197	0.160985049	16395	2.276975E-02
Mplkip	24198	0.191787132	17904	6.516737E-03
Mpnd	24199	0.05504249	8930	4.388561E-01
Mpo	24200	0.032755852	7224	6.451877E-01
Mpp1	24201	0.080062194	10595	2.597579E-01
Mpp2	24202	0.033396506	7261	6.387348E-01
Mpp3	24203	0.254894387	19564	2.699946E-04
Mpp4	24204	0.000650576	5347	9.927051E-01
Mpp5	24205	0.073181428	10078	3.030869E-01
Mpp6	24206	0.16759667	16802	1.768627E-02
Mpp7	24207	0.070137574	9849	3.236947E-01
Mppe1	24208	0.064892312	9532	3.612813E-01

Spearman Rank correlation analysis performed between Prdm1 and all-expressed genes within the Meredith RNA-seq dataset. Robust Prdm1–associated genes were identified using a cut-off of $p < 0.0005$.

Table S1, Related to Supplemental Figure 3C. Prdm1 associated genes

Mpped1	24209		#N/A	1.000000E+00
Mpped2	24210	0.041055826	7695	5.637901E-01
Mprip	24211	0.217979453	18844	1.930344E-03
Mpst	24212	-0.075054658	883	2.908446E-01
Mptx1	24213	-0.066856562	1139	3.468991E-01
Mptx2	24214	0.021316405	6469	7.644793E-01
Mpv17	24215	0.162479724	16497	2.152177E-02
Mpv17l	24216	0.039282025	7592	5.807683E-01
Mpv17l2	24217	0.1163406	13420	1.008872E-01
Mpz	24218	-0.057826016	1437	4.160241E-01
Mpzl1	24219	0.087483687	11166	2.180184E-01
Mpzl2	24220	0.115812595	13383	1.024540E-01
Mpzl3	24221	0.147553048	15507	3.706365E-02
Mr1	24222	-0.095269893	339	1.796190E-01
Mrap	24223	-0.038406171	3065	5.892384E-01
Mrap2	24224	-0.00174887	5083.5	9.803918E-01
Mras	24225	0.089486698	11309	2.076224E-01
Mrc1	24226	0.10873014	12603	1.253780E-01
Mrc2	24227	-0.071565062	934	3.139199E-01
Mre11a	24228	0.141131626	15025	4.621977E-02
Mreg	24229	0.24257438	19406	5.384679E-04
Mrfap1	24230	0.106019694	12427	1.351335E-01
Mrgbp	24231	0.033261442	7256	6.400930E-01
Mrgpra1	24232	-0.077393023	832.5	2.760318E-01
Mrgpra2a	24233		#N/A	1.000000E+00
Mrgpra2b	24234	0.119874377	13658.5	9.088346E-02
Mrgpra3	24235		#N/A	1.000000E+00
Mrgpra4	24236	-0.000458481	5247	9.948591E-01
Mrgpra6	24237		#N/A	1.000000E+00
Mrgpra9	24238	-0.038406171	3065	5.892384E-01
Mrgprb1	24239	0.046604408	8055.5	5.122676E-01
Mrgprb2	24240		#N/A	1.000000E+00
Mrgprb3	24241		#N/A	1.000000E+00
Mrgprb4	24242		#N/A	1.000000E+00
Mrgprb5	24243		#N/A	1.000000E+00
Mrgprb8	24244		#N/A	1.000000E+00
Mrgprd	24245		#N/A	1.000000E+00
Mrgpre	24246	0.016371193	6139	8.180235E-01
Mrgprf	24247		#N/A	1.000000E+00
Mrgprg	24248		#N/A	1.000000E+00
Mrgprh	24249		#N/A	1.000000E+00
Mrgprx1	24250		#N/A	1.000000E+00
Mrgprx2	24251	-0.004526355	4961.5	9.492796E-01

Spearman Rank correlation analysis performed between Prdm1 and all-expressed genes within the Meredith RNA-seq dataset. Robust Prdm1–associated genes were identified using a cut-off of $p < 0.0005$.

Table S1, Related to Supplemental Figure 3C. Prdm1 associated genes

Mrgprx3-ps	24252		#N/A	1.000000E+00
Mri1	24253	0.30684711	19928	9.899592E-06
Mrm1	24254	0.112936786	12920	1.113275E-01
Mro	24255	-0.022562523	4156	7.511506E-01
Mroh1	24256	0.118200003	13541	9.552006E-02
Mroh2a	24257	-0.022541563	4157	7.513742E-01
Mroh2b	24258	0.075436838	10296	2.883880E-01
Mroh3	24259	0.052728522	8550.5	4.583686E-01
Mroh4	24260	-0.034782061	3717	6.248718E-01
Mroh5	24261	-0.018105422	4347	7.991361E-01
Mroh7	24262		#N/A	1.000000E+00
Mroh8	24263	-0.041020017	2452	5.641304E-01
Mroh9	24264	-0.038406171	3065	5.892384E-01
Mrp63	24265	0.10673324	12477	1.325106E-01
Mrpl1	24266	0.088626179	11246	2.120440E-01
Mrpl10	24267	0.207059964	18495	3.262423E-03
Mrpl11	24268	0.07811517	10473	2.715623E-01
Mrpl12	24269	0.2203827	18911	1.713908E-03
Mrpl13	24270	0.034038553	7294	6.322949E-01
Mrpl14	24271	0.076703308	10391	2.803468E-01
Mrpl15	24272	0.165431079	16678	1.923021E-02
Mrpl16	24273	0.118164401	13538	9.562065E-02
Mrpl17	24274	-0.025693447	4075	7.179931E-01
Mrpl18	24275	0.194354332	18015	5.820904E-03
Mrpl19	24276	0.225985553	19071	1.292619E-03
Mrpl2	24277		#N/A	1.000000E+00
Mrpl20	24278	0.222930773	18986	1.508815E-03
Mrpl21	24279	0.142565855	15119	4.402564E-02
Mrpl22	24280	0.001074747	5362	9.879492E-01
Mrpl23	24281	0.147789581	15524	3.675800E-02
Mrpl23-ps1	24282	-0.07739401	763.5	2.760257E-01
Mrpl24	24283	0.122298471	13802	8.449097E-02
Mrpl27	24284	0.059974252	9211	3.988888E-01
Mrpl28	24285	0.049797074	8276	4.837666E-01
Mrpl3	24286	0.12037782	13685	8.952503E-02
Mrpl30	24287	0.065007773	9536	3.604258E-01
Mrpl32	24288	0.09562171	11744	1.780101E-01
Mrpl33	24289	0.149799233	15638	3.424600E-02
Mrpl34	24290	0.143251342	15153	4.300818E-02
Mrpl35	24291	0.20569423	18442	3.477541E-03
Mrpl36	24292	0.154842423	15970	2.857519E-02
Mrpl37	24293	0.13497549	14619	5.670128E-02
Mrpl38	24294	0.104987324	12354	1.389986E-01

Spearman Rank correlation analysis performed between Prdm1 and all-expressed genes within the Meredith RNA-seq dataset. Robust Prdm1–associated genes were identified using a cut-off of $p < 0.0005$.

Table S1, Related to Supplemental Figure 3C. Prdm1 associated genes

Mrpl39	24295	0.197972556	18166	4.953029E-03
Mrpl4	24296	0.153457505	15862	3.004623E-02
Mrpl40	24297	0.046511498	8044	5.131099E-01
Mrpl41	24298	0.080211696	10614	2.588663E-01
Mrpl42	24299	0.023522576	6718	7.409315E-01
Mrpl43	24300	0.171919761	17015	1.492372E-02
Mrpl44	24301	0.083500236	10877	2.397852E-01
Mrpl45	24302	0.073214803	10080	3.028659E-01
Mrpl46	24303	0.109503849	12680	1.226957E-01
Mrpl47	24304	0.068030886	9704	3.384759E-01
Mrpl48	24305	0.118631003	13563	9.430896E-02
Mrpl48-ps	24306	-0.038406171	3065	5.892384E-01
Mrpl49	24307	-0.006944953	4855	9.222483E-01
Mrpl50	24308	0.020667825	6381	7.714444E-01
Mrpl51	24309	0.25779175	19598	2.283790E-04
Mrpl52	24310	0.107252729	12512	1.306257E-01
Mrpl53	24311	0.113088743	12933	1.108441E-01
Mrpl54	24312	0.043856702	7856	5.374744E-01
Mrpl55	24313	0.113935303	13250	1.081807E-01
Mrpl9	24314	0.014474439	6040	8.388012E-01
Mrps10	24315	0.202883036	18352	3.961129E-03
Mrps11	24316	0.116403606	13426	1.007016E-01
Mrps12	24317	0.083799973	10905	2.380963E-01
Mrps14	24318	0.097847659	11898	1.680764E-01
Mrps15	24319	0.140105346	14962	4.784528E-02
Mrps16	24320	0.050851714	8364	4.745429E-01
Mrps17	24321	0.030238004	7074	6.708026E-01
Mrps18a	24322	0.085092505	10993	2.309089E-01
Mrps18b	24323	0.141148273	15026	4.619379E-02
Mrps18c	24324	0.240510011	19368	6.024699E-04
Mrps2	24325	0.18909095	17806	7.326579E-03
Mrps21	24326	0.061472922	9298	3.871884E-01
Mrps22	24327	0.060235856	9222	3.968314E-01
Mrps23	24328	0.035124011	7337	6.214705E-01
Mrps24	24329	0.148493056	15562	3.586151E-02
Mrps25	24330	0.063225629	9405	3.737714E-01
Mrps26	24331	0.15725798	16117	2.615657E-02
Mrps27	24332	0.012617733	5908	8.592490E-01
Mrps28	24333	0.028415213	6990	6.895884E-01
Mrps30	24334	0.158194887	16186	2.526690E-02
Mrps31	24335	-0.113970721	139	1.080704E-01
Mrps33	24336	0.191021417	17880	6.738191E-03
Mrps34	24337	0.054136798	8871	4.464363E-01

Spearman Rank correlation analysis performed between Prdm1 and all-expressed genes within the Meredith RNA-seq dataset. Robust Prdm1–associated genes were identified using a cut-off of $p < 0.0005$.

Table S1, Related to Supplemental Figure 3C. Prdm1 associated genes

Mrps35	24338	0.078479307	10495	2.693274E-01
Mrps36	24339	0.01695791	6170	8.116213E-01
Mrps36-ps1	24340		#N/A	1.000000E+00
Mrps36-ps2	24341		#N/A	1.000000E+00
Mrps5	24342	0.166339903	16736	1.856859E-02
Mrps6	24343	0.270559953	19722	1.066987E-04
Mrps7	24344	0.100726417	12105	1.558495E-01
Mrps9	24345	-0.008940583	4788	9.000098E-01
Mrrf	24346	0.03910655	7585	5.824607E-01
Mrs2	24347	0.167632675	16804	1.766154E-02
Mrto4	24348	0.219045506	18875	1.831434E-03
Mrvi1	24349	0.143994472	15314	4.192753E-02
Ms4a1	24350	0.101029665	12120	1.546016E-01
Ms4a10	24351	-0.077393517	797	2.760287E-01
Ms4a13	24352		#N/A	1.000000E+00
Ms4a15	24353	0.117347614	13481	9.795163E-02
Ms4a18	24354	-0.110564915	172	1.190898E-01
Ms4a2	24355	-0.11057427	157	1.190583E-01
Ms4a3	24356	-0.013423893	4599	8.503585E-01
Ms4a4b	24357	0.014710664	6054	8.362071E-01
Ms4a4c	24358	0.166683376	16757	1.832374E-02
Ms4a4d	24359		#N/A	1.000000E+00
Ms4a5	24360	-0.001224019	5151	9.862757E-01
Ms4a6b	24361	0.119176226	13596	9.279443E-02
Ms4a6c	24362	0.115223189	13336	1.042256E-01
Ms4a6d	24363	0.059891116	9203	3.995440E-01
Ms4a7	24364	0.073498585	10098.5	3.009906E-01
Ms4a8a	24365		#N/A	1.000000E+00
Msantd1	24366	0.271905275	19735	9.825725E-05
Msantd2	24367	0.112763303	12906	1.118815E-01
Msantd3	24368	0.002856355	5464	9.679800E-01
Msantd4	24369	-0.086936797	443	2.209203E-01
Msc	24370	0.124910154	13963	7.801313E-02
Msgn1	24371		#N/A	1.000000E+00
Msh2	24372	0.127065367	14121	7.297504E-02
Msh3	24373	0.153624477	15871	2.986552E-02
Msh4	24374	0.138302391	14846	5.081636E-02
Msh5	24375	0.017957376	6221	8.007441E-01
Msh6	24376	-0.005315365	4912	9.404534E-01
Msi1	24377	0.050016657	8300	4.818382E-01
Msi2	24378	0.058041212	9091	4.142884E-01
Msl1	24379	0.230917738	19186	1.002669E-03
Msl2	24380	0.059756077	9197	4.006096E-01

Spearman Rank correlation analysis performed between Prdm1 and all-expressed genes within the Meredith RNA-seq dataset. Robust Prdm1–associated genes were identified using a cut-off of $p < 0.0005$.

Table S1, Related to Supplemental Figure 3C. Prdm1 associated genes

Msl3	24381	0.229964282	19149	1.053570E-03
Msl3l2	24382	0.200391817	18257	4.439446E-03
Msln	24383	-0.103966381	215	1.429035E-01
Mslnl	24384	0.068523674	9741	3.349804E-01
Msmb	24385	0.07521908	10268	2.897860E-01
Msmp	24386		#N/A	1.000000E+00
Msn	24387	0.114069182	13255	1.077642E-01
Msr1	24388	-0.077393517	797	2.760287E-01
Msra	24389	-0.029064539	3964	6.828739E-01
Msrb1	24390	0.04589461	7997	5.187200E-01
Msrb2	24391	0.001570942	5395	9.823863E-01
Msrb3	24392	-0.070711677	940	3.197401E-01
Mss51	24393	0.145774979	15397	3.943078E-02
Mst1	24394	0.166899302	16768	1.817125E-02
Mst1r	24395	0.206228485	18466	3.391909E-03
Mstn	24396	0.023460478	6714	7.415911E-01
Msto1	24397	0.12459269	13944	7.877845E-02
Msx1	24398	0.238115888	19330	6.854608E-04
Msx2	24399		#N/A	1.000000E+00
Msx3	24400	0.078194185	10479	2.710763E-01
mt-Atp6	24401		#N/A	1.000000E+00
mt-Atp8	24402		#N/A	1.000000E+00
mt-Co1	24403		#N/A	1.000000E+00
mt-Co2	24404		#N/A	1.000000E+00
mt-Co3	24405		#N/A	1.000000E+00
mt-Cytb	24406		#N/A	1.000000E+00
mt-Nd1	24407		#N/A	1.000000E+00
mt-Nd2	24408		#N/A	1.000000E+00
mt-Nd3	24409		#N/A	1.000000E+00
mt-Nd4	24410		#N/A	1.000000E+00
mt-Nd4l	24411		#N/A	1.000000E+00
mt-Nd5	24412		#N/A	1.000000E+00
mt-Nd6	24413		#N/A	1.000000E+00
mt-Rnr1	24414		#N/A	1.000000E+00
mt-Rnr2	24415		#N/A	1.000000E+00
mt-Ta	24416		#N/A	1.000000E+00
mt-Tc	24417		#N/A	1.000000E+00
mt-Td	24418		#N/A	1.000000E+00
mt-Te	24419		#N/A	1.000000E+00
mt-Tf	24420		#N/A	1.000000E+00
mt-Tg	24421		#N/A	1.000000E+00
mt-Th	24422		#N/A	1.000000E+00
mt-Ti	24423		#N/A	1.000000E+00

Spearman Rank correlation analysis performed between Prdm1 and all-expressed genes within the Meredith RNA-seq dataset. Robust Prdm1–associated genes were identified using a cut-off of $p < 0.0005$.

Table S1, Related to Supplemental Figure 3C. Prdm1 associated genes

mt-Tk	24424		#N/A	1.000000E+00
mt-Tl1	24425		#N/A	1.000000E+00
mt-Tl2	24426		#N/A	1.000000E+00
mt-Tm	24427		#N/A	1.000000E+00
mt-Tn	24428		#N/A	1.000000E+00
mt-Tp	24429		#N/A	1.000000E+00
mt-Tq	24430		#N/A	1.000000E+00
mt-Tr	24431		#N/A	1.000000E+00
mt-Ts1	24432		#N/A	1.000000E+00
mt-Ts2	24433		#N/A	1.000000E+00
mt-Tt	24434		#N/A	1.000000E+00
mt-Tv	24435		#N/A	1.000000E+00
mt-Tw	24436		#N/A	1.000000E+00
mt-Ty	24437		#N/A	1.000000E+00
Mt1	24438	-0.088076374	434	2.149043E-01
Mt2	24439	0.047440279	8111	5.047228E-01
Mt3	24440	0.050815946	8362	4.748542E-01
Mt4	24441	0.079618499	10567	2.624164E-01
Mta1	24442	0.228072539	19099	1.161651E-03
Mta2	24443	0.177224463	17277	1.205509E-02
Mta3	24444	0.045660974	7978	5.208530E-01
Mtag2	24445		#N/A	1.000000E+00
Mtap	24446	0.323116224	19971	3.060732E-06
Mtap7d3	24447	-0.038406171	3065	5.892384E-01
Mtbp	24448	0.148534523	15563	3.580925E-02
Mtch1	24449	0.124035254	13911	8.013693E-02
Mtch2	24450	0.191749593	17902	6.527440E-03
Mtcp1	24451	0.024501208	6789	7.305611E-01
Mtdh	24452	0.122315905	13805	8.444634E-02
Mterf	24453	-0.072874406	923.5	3.051254E-01
Mterfd1	24454	0.059451522	9183	4.030191E-01
Mterfd2	24455	0.104559134	12328	1.406263E-01
Mterfd3	24456	-0.02700176	4029	7.042882E-01
Mtf1	24457	0.127276341	14134	7.249639E-02
Mtf2	24458	0.155104038	15988	2.830433E-02
Mtfmt	24459	0.017112232	6180	8.099394E-01
Mtfp1	24460	0.227774261	19096	1.179593E-03
Mtfr1	24461	0.029918115	7061	6.740851E-01
Mtfr1l	24462	0.139039824	14890	4.958312E-02
Mtfr2	24463	-0.014716616	4527	8.361418E-01
Mtg1	24464	0.287471845	19851	3.668167E-05
Mthfd1	24465	0.116508666	13433	1.003925E-01
Mthfd1l	24466	0.114010707	13253	1.079460E-01

Spearman Rank correlation analysis performed between Prdm1 and all-expressed genes within the Meredith RNA-seq dataset. Robust Prdm1–associated genes were identified using a cut-off of $p < 0.0005$.

Table S1, Related to Supplemental Figure 3C. Prdm1 associated genes

Mthfd2	24467	0.081609408	10712	2.506325E-01
Mthfd2l	24468	0.052662812	8519	4.589296E-01
Mthfr	24469	0.061108171	9268	3.900168E-01
Mthfr-ps1	24470		#N/A	1.000000E+00
Mthfs	24471	0.037583577	7464	5.972445E-01
Mthfsd	24472	0.063293667	9411	3.732564E-01
Mtif2	24473	0.082442527	10785	2.458119E-01
Mtif3	24474	-0.077617437	666	2.746376E-01
Mtl5	24475		#N/A	1.000000E+00
Mtm1	24476	-0.098208891	292	1.665040E-01
Mtmr1	24477	0.051877074	8461	4.656681E-01
Mtmr10	24478	-0.108959531	189	1.245781E-01
Mtmr11	24479	0.082620145	10796	2.447925E-01
Mtmr12	24480	0.174209351	17133	1.361952E-02
Mtmr14	24481	0.103594136	12275	1.443478E-01
Mtmr2	24482	0.10841907	12578	1.264692E-01
Mtmr3	24483	0.254266793	19555	2.798926E-04
Mtmr4	24484	0.109525059	12682	1.226228E-01
Mtmr6	24485	0.156658252	16088	2.674005E-02
Mtmr7	24486	0.077744065	10457	2.738530E-01
Mtmr9	24487	0.171627282	17001	1.509795E-02
Mtnr1a	24488	-0.066856562	1139	3.468991E-01
Mtnr1b	24489	-0.054451513	1703	4.437940E-01
Mto1	24490	-0.0022594	5062	9.746694E-01
Mtor	24491	0.008857616	5719	9.009330E-01
Mtpap	24492	0.150131459	15659	3.384508E-02
Mtpn	24493	0.081606493	10711	2.506495E-01
Mtr	24494	0.058441113	9121	4.110743E-01
Mtrf1	24495	-0.037261737	3653	6.003902E-01
Mtrf1l	24496	-0.055617258	1502	4.340839E-01
Mtrr	24497	0.123359616	13867	8.180872E-02
Mtss1	24498	0.183587991	17576	9.262572E-03
Mtss1l	24499	0.042192318	7751	5.530382E-01
Mttp	24500	0.029136962	7024	6.821265E-01
Mtus1	24501	0.319836172	19964	3.899628E-06
Mtus2	24502	-0.019686406	4249	7.820185E-01
Mtx1	24503	0.260533494	19631	1.945738E-04
Mtx2	24504	0.151199046	15726	3.258362E-02
Mtx3	24505	0.187307028	17731	7.910523E-03
Muc1	24506	0.231160877	19190	9.900555E-04
Muc13	24507	0.137750867	14805	5.175528E-02
Muc15	24508	0.247780089	19479	4.039334E-04
Muc16	24509	-0.124243499	87	7.962724E-02

Spearman Rank correlation analysis performed between Prdm1 and all-expressed genes within the Meredith RNA-seq dataset. Robust Prdm1–associated genes were identified using a cut-off of $p < 0.0005$.

Table S1, Related to Supplemental Figure 3C. Prdm1 associated genes

Muc19	24510	-0.045438257	2313.5	5.228904E-01
Muc2	24511		#N/A	1.000000E+00
Muc20	24512	-0.038406171	3065	5.892384E-01
Muc3	24513	0.00180563	5410	9.797555E-01
Muc4	24514	-0.091780178	405	1.961626E-01
Muc5ac	24515	-0.095267438	357.5	1.796303E-01
Muc5b	24516		#N/A	1.000000E+00
Muc6	24517		#N/A	1.000000E+00
Mucl1	24518	0.093511276	11615	1.878226E-01
Mug1	24519	-0.110568873	161	1.190765E-01
Mug2	24520	0.013842768	5982.5	8.457464E-01
Mul1	24521	0.028727436	7002	6.863567E-01
Mum1	24522	0.099880436	12054	1.593710E-01
Mum1l1	24523	-0.108278693	192	1.269640E-01
Mup-ps1	24524		#N/A	1.000000E+00
Mup-ps10	24525		#N/A	1.000000E+00
Mup-ps11	24526		#N/A	1.000000E+00
Mup-ps12	24527		#N/A	1.000000E+00
Mup-ps13	24528		#N/A	1.000000E+00
Mup-ps14	24529		#N/A	1.000000E+00
Mup-ps15	24530		#N/A	1.000000E+00
Mup-ps16	24531		#N/A	1.000000E+00
Mup-ps17	24532		#N/A	1.000000E+00
Mup-ps18	24533		#N/A	1.000000E+00
Mup-ps19	24534		#N/A	1.000000E+00
Mup-ps2	24535		#N/A	1.000000E+00
Mup-ps20	24536		#N/A	1.000000E+00
Mup-ps21	24537	-0.038406171	3065	5.892384E-01
Mup-ps22	24538		#N/A	1.000000E+00
Mup-ps3	24539		#N/A	1.000000E+00
Mup-ps4	24540		#N/A	1.000000E+00
Mup-ps5	24541		#N/A	1.000000E+00
Mup-ps6	24542		#N/A	1.000000E+00
Mup-ps7	24543		#N/A	1.000000E+00
Mup-ps8	24544		#N/A	1.000000E+00
Mup-ps9	24545		#N/A	1.000000E+00
Mup1	24546		#N/A	1.000000E+00
Mup10	24547		#N/A	1.000000E+00
Mup11	24548	-0.038406171	3065	5.892384E-01
Mup12	24549	-0.038406171	3065	5.892384E-01
Mup13	24550		#N/A	1.000000E+00
Mup14	24551		#N/A	1.000000E+00
Mup15	24552		#N/A	1.000000E+00

Spearman Rank correlation analysis performed between Prdm1 and all-expressed genes within the Meredith RNA-seq dataset. Robust Prdm1–associated genes were identified using a cut-off of $p < 0.0005$.

Table S1, Related to Supplemental Figure 3C. Prdm1 associated genes

Mup16	24553	-0.038406171	3065	5.892384E-01
Mup17	24554	0.01287414	5926	8.564193E-01
Mup18	24555	-0.038406171	3065	5.892384E-01
Mup19	24556		#N/A	1.000000E+00
Mup2	24557		#N/A	1.000000E+00
Mup20	24558	-0.038406171	3065	5.892384E-01
Mup21	24559	-0.038406171	3065	5.892384E-01
Mup3	24560	-0.054450825	1984	4.437997E-01
Mup4	24561	-0.054451513	1703	4.437940E-01
Mup5	24562	0.044495949	7898.5	5.315554E-01
Mup6	24563	-0.086747139	513	2.219331E-01
Mup7	24564		#N/A	1.000000E+00
Mup8	24565		#N/A	1.000000E+00
Mup9	24566		#N/A	1.000000E+00
Murc	24567		#N/A	1.000000E+00
Mus81	24568	0.02279817	6680	7.486382E-01
Musk	24569	0.272158242	19738	9.674157E-05
Mustn1	24570	0.020434681	6357	7.739526E-01
Mut	24571	0.094835413	11693	1.816208E-01
Mutyh	24572	0.053267784	8685	4.537786E-01
Mvb12a	24573	0.190732357	17871	6.823521E-03
Mvb12b	24574	0.034587204	7315	6.268137E-01
Mvd	24575	0.151818387	15755	3.187028E-02
Mvk	24576	0.124394	13932	7.926051E-02
Mvp	24577	0.175263331	17179	1.305351E-02
Mx1	24578	-0.009055537	4781	8.987310E-01
Mx2	24579	0.184867511	17628	8.775925E-03
Mxd1	24580	0.043756975	7849	5.384008E-01
Mxd3	24581	0.222795166	18977	1.519138E-03
Mxd4	24582	0.202247121	18335	4.078594E-03
Mxi1	24583	0.108787136	12608	1.251789E-01
Mxra7	24584	-0.066664255	1286.5	3.482910E-01
Mxra8	24585		#N/A	1.000000E+00
Myadm	24586	0.218624524	18862	1.869928E-03
MYADM	24587		#N/A	1.000000E+00
Myadml2	24588	0.113548679	13094	1.093908E-01
Myb	24589	0.130561929	14328	6.536599E-02
Mybbp1a	24590	-0.015238707	4500	8.304149E-01
Mybl1	24591	0.22134155	18940	1.633918E-03
Mybl2	24592	0.085682511	11030	2.276794E-01
Mybpc1	24593	0.141647056	15056	4.542096E-02
Mybpc2	24594	0.061994595	9331	3.831649E-01
Mybpc3	24595	0.075328288	10286.5	2.890843E-01

Spearman Rank correlation analysis performed between Prdm1 and all-expressed genes within the Meredith RNA-seq dataset. Robust Prdm1–associated genes were identified using a cut-off of $p < 0.0005$.

Table S1, Related to Supplemental Figure 3C. Prdm1 associated genes

Mybph	24596	0.067211716	9655	3.443378E-01
Mybphl	24597		#N/A	1.000000E+00
Myc	24598	0.134265423	14578	5.802757E-02
Mycbp	24599	0.213940046	18711	2.350875E-03
Mycbp2	24600	0.120355093	13683	8.958600E-02
Mycbpap	24601	-0.076317226	856.5	2.827820E-01
Mycl1	24602	0.031321219	7147	6.597339E-01
Mycn	24603	0.002988389	5472	9.665008E-01
Mycs	24604		#N/A	1.000000E+00
Myct1	24605		#N/A	1.000000E+00
Myd88	24606		#N/A	1.000000E+00
Myef2	24607	0.173230397	17091	1.416440E-02
Myeov2	24608	0.224222004	19024	1.413687E-03
Myf5	24609	0.092806124	11565	1.911880E-01
Myf6	24610		#N/A	1.000000E+00
Myg1	24611	0.132605944	14455	6.122716E-02
Myh1	24612	0.021317126	6513.5	7.644715E-01
Myh10	24613	-0.013679524	4579	8.475432E-01
Myh11	24614	-0.019331955	4259	7.858474E-01
Myh13	24615	-0.056155117	1483	4.296452E-01
Myh14	24616	0.036731652	7418	6.055874E-01
Myh15	24617	-0.073029081	920	3.040973E-01
Myh2	24618	0.113548679	13094	1.093908E-01
Myh3	24619	-0.0738593	906	2.986181E-01
Myh4	24620	0.018246336	6236	7.976064E-01
Myh6	24621		#N/A	1.000000E+00
Myh7	24622	-0.071610969	932	3.136088E-01
Myh7b	24623		#N/A	1.000000E+00
Myh8	24624	0.109930598	12709.5	1.212354E-01
Myh9	24625	0.203006485	18356	3.938682E-03
Myl1	24626	-0.038406171	3065	5.892384E-01
Myl10	24627	0.113548679	13094	1.093908E-01
Myl12a	24628	0.171851509	17010	1.496422E-02
Myl12b	24629	0.26241064	19650	1.741862E-04
Myl2	24630	-0.040014788	2471	5.737258E-01
Myl3	24631	-0.038406171	3065	5.892384E-01
Myl4	24632	0.005333883	5576	9.402464E-01
Myl6	24633	0.178286635	17319	1.154289E-02
Myl6b	24634	-0.064455064	1335	3.645327E-01
Myl7	24635	0.058849548	9148	4.078068E-01
Myl9	24636	0.113548679	13094	1.093908E-01
Mylip	24637	0.001510631	5389	9.830625E-01
Mylk	24638	0.072601058	10036	3.069479E-01

Spearman Rank correlation analysis performed between Prdm1 and all-expressed genes within the Meredith RNA-seq dataset. Robust Prdm1–associated genes were identified using a cut-off of $p < 0.0005$.

Table S1, Related to Supplemental Figure 3C. Prdm1 associated genes

Mylk2	24639	-0.028358924	3985	6.901717E-01
Mylk3	24640	-0.051858334	2168	4.658294E-01
Mylk4	24641	0.077483653	10442	2.754682E-01
Mylpf	24642	-0.061669098	1379	3.856724E-01
Mynn	24643	0.098787351	11979	1.640091E-01
Myo10	24644	0.075804338	10331	2.860389E-01
Myo15	24645	-0.086755146	452	2.218903E-01
Myo15b	24646	-0.014457634	4543	8.389859E-01
Myo16	24647	-0.075943141	866	2.851550E-01
Myo18a	24648	0.146016382	15414	3.910212E-02
Myo18b	24649	0.140317019	14976.5	4.750617E-02
Myo19	24650	-0.077512444	668	2.752893E-01
Myo1a	24651	-0.024447711	4119	7.311268E-01
Myo1b	24652	0.190275833	17851	6.960248E-03
Myo1c	24653	-0.021307335	4198	7.645765E-01
Myo1d	24654	0.048038771	8152	4.993565E-01
Myo1e	24655	0.04568956	7982	5.205918E-01
Myo1f	24656	0.266827391	19697	1.338119E-04
Myo1g	24657	-0.044996539	2333	5.269432E-01
Myo1h	24658	-0.050854125	2185	4.745220E-01
Myo3a	24659	0.085895021	11048	2.265240E-01
Myo3b	24660	-0.00201128	5072	9.774504E-01
Myo5a	24661	0.063876785	9462	3.688604E-01
Myo5b	24662	0.011271468	5844	8.741349E-01
Myo5c	24663	0.157839167	16168	2.560158E-02
Myo6	24664	0.074545415	10180	2.941395E-01
Myo7a	24665	-0.011327383	4691	8.735157E-01
Myo7b	24666	0.236205716	19293	7.591050E-04
Myo9a	24667	0.091003172	11436	1.999924E-01
Myo9b	24668	0.119978703	13661	9.060061E-02
Myoc	24669	-0.038406171	3065	5.892384E-01
Myocd	24670	0.337991707	19995	9.841390E-07
Myod1	24671		#N/A	1.000000E+00
Myof	24672	0.207326964	18506	3.221801E-03
Myog	24673	-0.058212489	1430	4.129100E-01
Myom1	24674	-0.077394997	736.5	2.760195E-01
Myom2	24675	-0.001224019	5151	9.862757E-01
Myom3	24676	-0.084120131	590	2.363015E-01
Myot	24677	-0.109298988	187	1.234016E-01
Myoz1	24678	0.058607704	9134	4.097397E-01
Myoz2	24679		#N/A	1.000000E+00
Myoz3	24680	0.014709635	6053	8.362184E-01
Mypn	24681	0.032091587	7187	6.519066E-01

Spearman Rank correlation analysis performed between Prdm1 and all-expressed genes within the Meredith RNA-seq dataset. Robust Prdm1–associated genes were identified using a cut-off of $p < 0.0005$.

Table S1, Related to Supplemental Figure 3C. Prdm1 associated genes

Mypop	24682	0.156444817	16077	2.695038E-02
Myrf	24683	-0.043485935	2392	5.409225E-01
Myrfl	24684	0.004866775	5554	9.454707E-01
Myrip	24685	0.231456884	19197	9.748962E-04
Mysm1	24686	0.217275518	18827	1.998299E-03
Myt1	24687	0.108356382	12573	1.266899E-01
Myt1l	24688	0.097740795	11886	1.685436E-01
Myzap	24689		#N/A	1.000000E+00
Mzb1	24690	0.206451549	18474	3.356723E-03
Mzf1	24691	0.166291058	16732	1.860364E-02
Mzt1	24692	0.15055709	15683	3.333727E-02
Mzt2	24693	0.063208444	9403	3.739016E-01
n-R5-8s1	24694	-0.038406171	3065	5.892384E-01
n-R5s1	24695		#N/A	1.000000E+00
n-R5s10	24696		#N/A	1.000000E+00
n-R5s100	24697		#N/A	1.000000E+00
n-R5s101	24698		#N/A	1.000000E+00
n-R5s102	24699		#N/A	1.000000E+00
n-R5s103	24700		#N/A	1.000000E+00
n-R5s104	24701		#N/A	1.000000E+00
n-R5s105	24702		#N/A	1.000000E+00
n-R5s106	24703		#N/A	1.000000E+00
n-R5s107	24704		#N/A	1.000000E+00
n-R5s108	24705		#N/A	1.000000E+00
n-R5s109	24706		#N/A	1.000000E+00
n-R5s110	24707		#N/A	1.000000E+00
n-R5s111	24708		#N/A	1.000000E+00
n-R5s112	24709		#N/A	1.000000E+00
n-R5s113	24710		#N/A	1.000000E+00
n-R5s114	24711		#N/A	1.000000E+00
n-R5s115	24712		#N/A	1.000000E+00
n-R5s117	24713		#N/A	1.000000E+00
n-R5s118	24714		#N/A	1.000000E+00
n-R5s119	24715		#N/A	1.000000E+00
n-R5s12	24716		#N/A	1.000000E+00
n-R5s120	24717		#N/A	1.000000E+00
n-R5s121	24718		#N/A	1.000000E+00
n-R5s122	24719		#N/A	1.000000E+00
n-R5s123	24720		#N/A	1.000000E+00
n-R5s124	24721		#N/A	1.000000E+00
n-R5s125	24722		#N/A	1.000000E+00
n-R5s127	24723		#N/A	1.000000E+00
n-R5s128	24724		#N/A	1.000000E+00

Spearman Rank correlation analysis performed between Prdm1 and all-expressed genes within the Meredith RNA-seq dataset. Robust Prdm1–associated genes were identified using a cut-off of $p < 0.0005$.

Table S1, Related to Supplemental Figure 3C. Prdm1 associated genes

n-R5s129	24725		#N/A	1.000000E+00
n-R5s13	24726	-0.038406171	3065	5.892384E-01
n-R5s130	24727		#N/A	1.000000E+00
n-R5s131	24728		#N/A	1.000000E+00
n-R5s133	24729		#N/A	1.000000E+00
n-R5s134	24730		#N/A	1.000000E+00
n-R5s136	24731		#N/A	1.000000E+00
n-R5s138	24732		#N/A	1.000000E+00
n-R5s139	24733		#N/A	1.000000E+00
n-R5s14	24734		#N/A	1.000000E+00
n-R5s141	24735		#N/A	1.000000E+00
n-R5s142	24736		#N/A	1.000000E+00
n-R5s143	24737		#N/A	1.000000E+00
n-R5s144	24738		#N/A	1.000000E+00
n-R5s146	24739		#N/A	1.000000E+00
n-R5s149	24740		#N/A	1.000000E+00
n-R5s15	24741		#N/A	1.000000E+00
n-R5s150	24742		#N/A	1.000000E+00
n-R5s151	24743		#N/A	1.000000E+00
n-R5s152	24744		#N/A	1.000000E+00
n-R5s153	24745		#N/A	1.000000E+00
n-R5s154	24746		#N/A	1.000000E+00
n-R5s155	24747		#N/A	1.000000E+00
n-R5s156	24748		#N/A	1.000000E+00
n-R5s157	24749		#N/A	1.000000E+00
n-R5s158	24750	-0.038406171	3065	5.892384E-01
n-R5s159	24751		#N/A	1.000000E+00
n-R5s16	24752		#N/A	1.000000E+00
n-R5s160	24753		#N/A	1.000000E+00
n-R5s161	24754		#N/A	1.000000E+00
n-R5s162	24755		#N/A	1.000000E+00
n-R5s164	24756		#N/A	1.000000E+00
n-R5s165	24757		#N/A	1.000000E+00
n-R5s166	24758		#N/A	1.000000E+00
n-R5s167	24759		#N/A	1.000000E+00
n-R5s168	24760		#N/A	1.000000E+00
n-R5s169	24761		#N/A	1.000000E+00
n-R5s170	24762		#N/A	1.000000E+00
n-R5s171	24763		#N/A	1.000000E+00
n-R5s172	24764		#N/A	1.000000E+00
n-R5s173	24765		#N/A	1.000000E+00
n-R5s174	24766		#N/A	1.000000E+00
n-R5s175	24767	-0.038406171	3065	5.892384E-01

Spearman Rank correlation analysis performed between Prdm1 and all-expressed genes within the Meredith RNA-seq dataset. Robust Prdm1–associated genes were identified using a cut-off of $p < 0.0005$.

Table S1, Related to Supplemental Figure 3C. Prdm1 associated genes

n-R5s176	24768		#N/A	1.000000E+00
n-R5s178	24769		#N/A	1.000000E+00
n-R5s179	24770		#N/A	1.000000E+00
n-R5s18	24771		#N/A	1.000000E+00
n-R5s180	24772		#N/A	1.000000E+00
n-R5s182	24773		#N/A	1.000000E+00
n-R5s183	24774		#N/A	1.000000E+00
n-R5s185	24775	-0.066856562	1139	3.468991E-01
n-R5s187	24776		#N/A	1.000000E+00
n-R5s188	24777		#N/A	1.000000E+00
n-R5s189	24778		#N/A	1.000000E+00
n-R5s19	24779		#N/A	1.000000E+00
n-R5s191	24780		#N/A	1.000000E+00
n-R5s192	24781		#N/A	1.000000E+00
n-R5s193	24782		#N/A	1.000000E+00
n-R5s194	24783		#N/A	1.000000E+00
n-R5s195	24784		#N/A	1.000000E+00
n-R5s196	24785		#N/A	1.000000E+00
n-R5s197	24786		#N/A	1.000000E+00
n-R5s198	24787		#N/A	1.000000E+00
n-R5s2	24788	-0.038406171	3065	5.892384E-01
n-R5s200	24789		#N/A	1.000000E+00
n-R5s201	24790		#N/A	1.000000E+00
n-R5s202	24791		#N/A	1.000000E+00
n-R5s204	24792		#N/A	1.000000E+00
n-R5s205	24793		#N/A	1.000000E+00
n-R5s207	24794		#N/A	1.000000E+00
n-R5s209	24795		#N/A	1.000000E+00
n-R5s21	24796		#N/A	1.000000E+00
n-R5s210	24797		#N/A	1.000000E+00
n-R5s211	24798		#N/A	1.000000E+00
n-R5s213	24799		#N/A	1.000000E+00
n-R5s214	24800		#N/A	1.000000E+00
n-R5s215	24801		#N/A	1.000000E+00
n-R5s216	24802		#N/A	1.000000E+00
n-R5s217	24803		#N/A	1.000000E+00
n-R5s218	24804		#N/A	1.000000E+00
n-R5s219	24805		#N/A	1.000000E+00
n-R5s220	24806		#N/A	1.000000E+00
n-R5s23	24807		#N/A	1.000000E+00
n-R5s24	24808		#N/A	1.000000E+00
n-R5s25	24809		#N/A	1.000000E+00
n-R5s26	24810		#N/A	1.000000E+00

Spearman Rank correlation analysis performed between Prdm1 and all-expressed genes within the Meredith RNA-seq dataset. Robust Prdm1–associated genes were identified using a cut-off of $p < 0.0005$.

Table S1, Related to Supplemental Figure 3C. Prdm1 associated genes

n-R5s27	24811	#N/A	1.000000E+00
n-R5s28	24812	#N/A	1.000000E+00
n-R5s29	24813	#N/A	1.000000E+00
n-R5s3	24814	#N/A	1.000000E+00
n-R5s30	24815	#N/A	1.000000E+00
n-R5s31	24816	#N/A	1.000000E+00
n-R5s32	24817	#N/A	1.000000E+00
n-R5s33	24818	#N/A	1.000000E+00
n-R5s34	24819	#N/A	1.000000E+00
n-R5s36	24820	#N/A	1.000000E+00
n-R5s37	24821	#N/A	1.000000E+00
n-R5s39	24822	#N/A	1.000000E+00
n-R5s40	24823	#N/A	1.000000E+00
n-R5s41	24824	#N/A	1.000000E+00
n-R5s42	24825	#N/A	1.000000E+00
n-R5s43	24826	#N/A	1.000000E+00
n-R5s45	24827	#N/A	1.000000E+00
n-R5s46	24828	#N/A	1.000000E+00
n-R5s47	24829	#N/A	1.000000E+00
n-R5s48	24830	#N/A	1.000000E+00
n-R5s5	24831	#N/A	1.000000E+00
n-R5s50	24832	#N/A	1.000000E+00
n-R5s51	24833	#N/A	1.000000E+00
n-R5s52	24834	#N/A	1.000000E+00
n-R5s54	24835	#N/A	1.000000E+00
n-R5s56	24836	#N/A	1.000000E+00
n-R5s58	24837	#N/A	1.000000E+00
n-R5s6	24838	#N/A	1.000000E+00
n-R5s60	24839	#N/A	1.000000E+00
n-R5s61	24840	#N/A	1.000000E+00
n-R5s62	24841	#N/A	1.000000E+00
n-R5s63	24842	#N/A	1.000000E+00
n-R5s64	24843	#N/A	1.000000E+00
n-R5s65	24844	#N/A	1.000000E+00
n-R5s67	24845	#N/A	1.000000E+00
n-R5s68	24846	#N/A	1.000000E+00
n-R5s69	24847	#N/A	1.000000E+00
n-R5s7	24848	#N/A	1.000000E+00
n-R5s70	24849	#N/A	1.000000E+00
n-R5s71	24850	#N/A	1.000000E+00
n-R5s72	24851	#N/A	1.000000E+00
n-R5s73	24852	#N/A	1.000000E+00
n-R5s74	24853	#N/A	1.000000E+00

Spearman Rank correlation analysis performed between Prdm1 and all-expressed genes within the Meredith RNA-seq dataset. Robust Prdm1–associated genes were identified using a cut-off of $p < 0.0005$.

Table S1, Related to Supplemental Figure 3C. Prdm1 associated genes

n-R5s76	24854		#N/A	1.000000E+00
n-R5s77	24855		#N/A	1.000000E+00
n-R5s79	24856		#N/A	1.000000E+00
n-R5s8	24857		#N/A	1.000000E+00
n-R5s80	24858		#N/A	1.000000E+00
n-R5s82	24859		#N/A	1.000000E+00
n-R5s84	24860		#N/A	1.000000E+00
n-R5s85	24861		#N/A	1.000000E+00
n-R5s86	24862		#N/A	1.000000E+00
n-R5s87	24863		#N/A	1.000000E+00
n-R5s88	24864		#N/A	1.000000E+00
n-R5s89	24865		#N/A	1.000000E+00
n-R5s9	24866		#N/A	1.000000E+00
n-R5s90	24867		#N/A	1.000000E+00
n-R5s92	24868		#N/A	1.000000E+00
n-R5s93	24869		#N/A	1.000000E+00
n-R5s94	24870		#N/A	1.000000E+00
n-R5s95	24871		#N/A	1.000000E+00
n-R5s96	24872	-0.066856562	1139	3.468991E-01
n-R5s97	24873		#N/A	1.000000E+00
n-R5s98	24874		#N/A	1.000000E+00
N28178	24875	0.005036707	5565	9.435699E-01
N4bp1	24876	0.152036437	15771	3.162233E-02
N4bp2	24877	0.166835249	16766	1.821637E-02
N4bp2l1	24878	0.06497976	9533	3.606333E-01
N4bp2l2	24879	0.084682374	10966	2.331727E-01
N4bp3	24880	0.239041445	19350	6.522003E-04
N6amt1	24881	0.199241522	18212	4.677348E-03
N6amt2	24882	0.123642134	13880	8.110627E-02
Naa10	24883		#N/A	1.000000E+00
Naa11	24884		#N/A	1.000000E+00
Naa15	24885	0.16612053	16714	1.872645E-02
Naa16	24886	0.121614239	13766	8.625763E-02
Naa20	24887	0.086438045	11100	2.235907E-01
Naa25	24888	0.138077906	14831	5.119680E-02
Naa30	24889	-0.01451416	4542	8.383649E-01
Naa35	24890	0.172690038	17061	1.447326E-02
Naa38	24891	0.192023081	17911	6.449820E-03
Naa40	24892	0.120435869	13689	8.936944E-02
Naa50	24893	0.103310319	12258	1.454565E-01
Naa60	24894		#N/A	1.000000E+00
Naaa	24895	0.108905788	12616	1.247651E-01
Naalad2	24896	-0.086750697	472.5	2.219141E-01

Spearman Rank correlation analysis performed between Prdm1 and all-expressed genes within the Meredith RNA-seq dataset. Robust Prdm1–associated genes were identified using a cut-off of $p < 0.0005$.

Table S1, Related to Supplemental Figure 3C. Prdm1 associated genes

Naaladl1	24897		#N/A	1.000000E+00
Nab1	24898	0.234689926	19266	8.226591E-04
Nab2	24899	0.085354147	11012	2.294728E-01
Nabp1	24900	0.097089906	11843	1.714105E-01
Nabp2	24901	0.14600444	15412	3.911832E-02
Naca	24902	0.129550099	14274	6.749797E-02
Nacad	24903	0.113548679	13094	1.093908E-01
Nacc1	24904	0.210077786	18574	2.829145E-03
Nacc2	24905	0.22198534	18958	1.582140E-03
Nadk	24906	0.132365573	14439	6.170241E-02
Nadkd1	24907	0.138025877	14824	5.128532E-02
Nadsyn1	24908	0.071038116	9914	3.175055E-01
Nae1	24909	-0.015318605	4497	8.295393E-01
Naf1	24910	0.11638597	13424	1.007535E-01
Naga	24911	0.082690114	10805	2.443918E-01
Nagk	24912	0.121846809	13779	8.565385E-02
Naglu	24913	-0.004579404	4955	9.486860E-01
Nagpa	24914	0.248546142	19490	3.870011E-04
Nags	24915		#N/A	1.000000E+00
Naif1	24916	-0.110563117	176	1.190958E-01
Naip1	24917	0.052933125	8590	4.566240E-01
Naip2	24918	0.173554469	17102	1.398195E-02
Naip3	24919	0.21898978	18874	1.836488E-03
Naip5	24920	-0.02822399	3989	6.915705E-01
Naip6	24921	-0.066855996	1239.5	3.469032E-01
Nalcn	24922	0.025004896	6820	7.252425E-01
Nampt	24923	0.074357064	10168	2.953645E-01
Nanog	24924	-0.001224019	5151	9.862757E-01
Nanos1	24925	0.172829685	17069	1.439288E-02
Nanos2	24926	0.053805701	8820	4.492258E-01
Nanos3	24927		#N/A	1.000000E+00
Nanp	24928	0.023212512	6704	7.442270E-01
Nans	24929	0.093399685	11607	1.883523E-01
Nap1l1	24930	0.146370921	15440	3.862360E-02
Nap1l2	24931	-0.066856562	1139	3.468991E-01
Nap1l3	24932	0.087115402	11136	2.199696E-01
Nap1l4	24933	0.129226338	14254	6.819203E-02
Nap1l5	24934	0.003239784	5490	9.636845E-01
Napa	24935	0.04343833	7832	5.413660E-01
Napb	24936	0.064709762	9519	3.626365E-01
Napepld	24937	0.119790688	13651	9.111086E-02
Napg	24938	0.051824596	8456	4.661200E-01
Naprt1	24939	-0.02829001	3987	6.908860E-01

Spearman Rank correlation analysis performed between Prdm1 and all-expressed genes within the Meredith RNA-seq dataset. Robust Prdm1–associated genes were identified using a cut-off of $p < 0.0005$.

Table S1, Related to Supplemental Figure 3C. Prdm1 associated genes

Napsa	24940	0.040492736	7664	5.691540E-01
Narf	24941	0.063174524	9400	3.741585E-01
Narfl	24942	0.157761699	16139	2.567496E-02
Narg2	24943	0.143916633	15308	4.203964E-02
Nars	24944	-0.009701244	4757	8.915529E-01
Nars2	24945	0.100599119	12096	1.563756E-01
Nasp	24946	0.1972442	18132	5.117769E-03
Nat1	24947	0.166148435	16721.5	1.870631E-02
Nat10	24948	-0.066856562	1139	3.468991E-01
Nat14	24949	0.146005822	15413	3.911644E-02
Nat2	24950	0.137968096	14819	5.138376E-02
Nat3	24951		#N/A	1.000000E+00
Nat6	24952	0.183223478	17559	9.405514E-03
Nat8	24953	0.229057459	19118.5	1.104172E-03
Nat8b	24954		#N/A	1.000000E+00
Nat8l	24955	0.05019642	8312	4.802627E-01
Nat9	24956	0.002772415	5458	9.689205E-01
Nav1	24957	0.219723121	18893	1.770990E-03
Nav2	24958	0.238615459	19339	6.673189E-04
Nav3	24959	0.003199032	5487	9.641410E-01
Nbas	24960	-0.044336495	2356	5.330288E-01
Nbea	24961	0.178569583	17330	1.140972E-02
Nbeal1	24962	0.052736628	8578	4.582994E-01
Nbeal2	24963	0.280526239	19803	5.734897E-05
Nbl1	24964	-0.037960269	3633	5.935721E-01
Nbn	24965	0.190443045	17860	6.909888E-03
Nbr1	24966	0.180082238	17406	1.072050E-02
Ncald	24967	0.014343891	6032	8.402356E-01
Ncam1	24968	0.011776616	5867	8.685439E-01
Ncam2	24969	-0.022459031	4160	7.522548E-01
Ncan	24970	0.260406245	19629	1.960334E-04
Ncapd2	24971	0.116470004	13429	1.005062E-01
Ncapd3	24972	0.254125534	19551	2.821665E-04
Ncapg	24973	0.352655627	20007	3.030435E-07
Ncapg2	24974	0.133635236	14520	5.922598E-02
Ncaph	24975	0.145165495	15362	4.027096E-02
Ncaph2	24976	0.214096773	18718	2.333118E-03
Ncbp1	24977	0.06262257	9367	3.783553E-01
Ncbp2	24978	0.167690489	16806	1.762190E-02
Nccrp1	24979		#N/A	1.000000E+00
Ncdn	24980	0.011477948	5849	8.718488E-01
Nceh1	24981	-0.031273561	3874	6.602194E-01
Ncf1	24982	0.016552687	6147	8.160417E-01

Spearman Rank correlation analysis performed between Prdm1 and all-expressed genes within the Meredith RNA-seq dataset. Robust Prdm1–associated genes were identified using a cut-off of $p < 0.0005$.

Table S1, Related to Supplemental Figure 3C. Prdm1 associated genes

Ncf2	24983	0.052995513	8594	4.560928E-01
Ncf4	24984	0.2016989	18315	4.182369E-03
Nck1	24985	0.09894466	11990	1.633355E-01
Nck2	24986	0.071891573	9989	3.117115E-01
Nckap1	24987	0.200652367	18272	4.387095E-03
Nckap1l	24988	0.243742773	19424	5.050906E-04
Nckap5	24989	0.153254233	15851	3.026748E-02
Nckap5l	24990	0.090567304	11389	2.021643E-01
Nckipsd	24991	-0.033702148	3752	6.356657E-01
Ncl	24992	0.146942903	15476	3.786203E-02
Ncln	24993	0.103494716	12269	1.447355E-01
Ncmap	24994	0.168170962	16832	1.729538E-02
Ncoa1	24995	0.173354305	17094	1.409439E-02
Ncoa2	24996	0.143627063	15285	4.245893E-02
Ncoa3	24997	0.098659891	11968	1.645564E-01
Ncoa4	24998	0.075624021	10300	2.871899E-01
Ncoa5	24999	0.111573788	12824	1.157382E-01
Ncoa6	25000	0.040637386	7672	5.677738E-01
Ncoa7	25001	0.154284307	15909	2.916043E-02
Ncor1	25002	0.165199542	16669	1.940201E-02
Ncor2	25003	0.137054551	14761	5.296122E-02
Ncr1	25004	0.039813567	7624	5.756557E-01
Ncr3-ps	25005		#N/A	1.000000E+00
Ncrna00085	25006	-0.038406171	3065	5.892384E-01
Ncrna00086	25007	-0.042453771	2423	5.505789E-01
Ncs1	25008	0.017696894	6212	8.035753E-01
Ncstn	25009	0.180681844	17436	1.045764E-02
Nctc1	25010	0.048448014	8175.5	4.957045E-01
Ndc80	25011	0.236110727	19291	7.629508E-04
Nde1	25012	0.16226371	16481	2.169840E-02
Ndel1	25013	0.108485506	12581	1.262355E-01
Ndfip1	25014	0.092969429	11582	1.904047E-01
Ndfip2	25015	0.047802578	8129	5.014707E-01
Ndn	25016	-0.095269893	339	1.796190E-01
Ndnf	25017	0.065944334	9586	3.535329E-01
Ndnl2	25018		#N/A	1.000000E+00
Ndor1	25019	0.202770488	18348	3.981694E-03
Ndp	25020	0.110450943	12752	1.194731E-01
Ndrg1	25021	0.143642322	15286	4.243674E-02
Ndrg2	25022	-0.083826534	594	2.379470E-01
Ndrg3	25023	0.195936953	18082	5.425811E-03
Ndrg4	25024	0.142541199	15117	4.406261E-02
Ndst1	25025	0.098445162	11951	1.654816E-01

Spearman Rank correlation analysis performed between Prdm1 and all-expressed genes within the Meredith RNA-seq dataset. Robust Prdm1–associated genes were identified using a cut-off of $p < 0.0005$.

Table S1, Related to Supplemental Figure 3C. Prdm1 associated genes

Ndst2	25026	0.089239633	11288	2.088850E-01
Ndst3	25027	0.092304139	11538	1.936104E-01
Ndst4	25028	0.053183539	8604	4.544940E-01
Ndufa1	25029	0.126689298	14083	7.383461E-02
Ndufa10	25030	0.256173874	19576	2.508157E-04
Ndufa11	25031	0.098370124	11942	1.658058E-01
Ndufa12	25032	0.156528719	16081	2.686753E-02
Ndufa13	25033		#N/A	1.000000E+00
Ndufa2	25034	0.119657398	13635	9.147397E-02
Ndufa3	25035	0.201033698	18289	4.311477E-03
Ndufa4	25036	0.079467662	10562	2.633244E-01
Ndufa4l2	25037	0.122900064	13837	8.296179E-02
Ndufa5	25038	0.11548419	13356	1.034382E-01
Ndufa6	25039	0.133189249	14489	6.008637E-02
Ndufa7	25040	0.264432166	19671	1.544705E-04
Ndufa8	25041	0.197723813	18151	5.008747E-03
Ndufa9	25042	0.075005977	10251	2.911585E-01
Ndufab1	25043	0.219789176	18896	1.765196E-03
Ndufaf1	25044	0.171022385	16975	1.546393E-02
Ndufaf2	25045	0.095929995	11766	1.766090E-01
Ndufaf3	25046	0.073520997	10100	3.008428E-01
Ndufaf4	25047	0.031744477	7173	6.554288E-01
Ndufaf5	25048	0.068118061	9710	3.378559E-01
Ndufaf6	25049	0.103757929	12282	1.437109E-01
Ndufaf7	25050	0.15449881	15955	2.893430E-02
Ndufb10	25051	0.180302616	17419	1.062322E-02
Ndufb11	25052	0.152661874	15802	3.092019E-02
Ndufb2	25053	0.144449188	15332	4.127760E-02
Ndufb3	25054	0.162659852	16504	2.137544E-02
Ndufb4	25055	0.185833555	17675	8.423664E-03
Ndufb5	25056	0.102807548	12233	1.474364E-01
Ndufb6	25057	-0.004797938	4948	9.462408E-01
Ndufb7	25058	-0.015260726	4498	8.301736E-01
Ndufb8	25059	0.173969249	17121	1.375143E-02
Ndufb9	25060	0.064379066	9497	3.650996E-01
Ndufc1	25061	0.178720081	17340	1.133944E-02
Ndufc2	25062	0.110586327	12765	1.190179E-01
Ndufs1	25063	0.068542312	9742	3.348487E-01
Ndufs2	25064	0.164159028	16602	2.019056E-02
Ndufs3	25065	0.136628905	14738	5.370980E-02
Ndufs4	25066	0.123382754	13870	8.175101E-02
Ndufs5	25067	0.252883536	19535	3.029118E-04
Ndufs6	25068	0.092379514	11540	1.932452E-01

Spearman Rank correlation analysis performed between Prdm1 and all-expressed genes within the Meredith RNA-seq dataset. Robust Prdm1–associated genes were identified using a cut-off of $p < 0.0005$.

Ndufs7	25069	0.173754807	17110	1.387019E-02
Ndufs8	25070	0.211305889	18626	2.668279E-03
Ndufv1	25071	0.148976124	15588	3.525668E-02
Ndufv2	25072	0.120673517	13701	8.873475E-02
Ndufv3	25073	0.173213352	17090	1.417405E-02
Neat1	25074	0.095790133	11752	1.772436E-01
Neb	25075	0.185420788	17656	8.572612E-03
Nebl	25076	-0.054246172	2105	4.455170E-01
Necab1	25077	-0.01982266	4245	7.805480E-01
Necab2	25078	-0.054450825	1984	4.437997E-01
Necab3	25079	0.047870267	8136	5.008643E-01
Necap1	25080	0.032534359	7206	6.474249E-01
Necap2	25081	0.052275869	8489	4.622412E-01
Nedd1	25082	-0.008941689	4787	8.999975E-01
Nedd4	25083	0.138278675	14842	5.085644E-02
Nedd4l	25084	0.176819315	17258	1.225567E-02
Nedd8	25085	0.095824012	11754	1.770897E-01
Nedd9	25086	0.117177354	13474	9.844315E-02
Nefh	25087	-0.036508142	3671	6.077848E-01
Nefl	25088	0.077422004	10437	2.758515E-01
Nefm	25089	-0.010777384	4712	8.796095E-01
Negr1	25090	0.177002116	17269	1.216481E-02
Neil1	25091	0.157799267	16153.5	2.563935E-02
Neil2	25092	-0.00045847	5257	9.948592E-01
Neil3	25093	0.223526974	19003	1.464187E-03
Nek1	25094	-0.007989554	4820	9.105991E-01
Nek10	25095	0.018469523	6245	7.951851E-01
Nek11	25096	-0.091597608	406	1.970577E-01
Nek2	25097	0.210430174	18591	2.782111E-03
Nek3	25098	0.068916246	9767	3.322123E-01
Nek4	25099	-0.095269893	339	1.796190E-01
Nek5	25100	0.109048866	12626	1.242676E-01
Nek6	25101	-0.01424247	4555	8.413503E-01
Nek7	25102	0.188178615	17768	7.620243E-03
Nek8	25103	-0.120182643	101	9.004973E-02
Nek9	25104	0.105974575	12419	1.353007E-01
Nelfa	25105	0.000361571	5333	9.959457E-01
Nelfb	25106	0.106914528	12491	1.318504E-01
Nelfcd	25107	0.095869846	11760	1.768817E-01
Nelfe	25108	0.205641284	18440	3.486132E-03
Nell1	25109	0.112815433	12908	1.117148E-01
Nell2	25110	0.047139869	8087	5.074277E-01
Nemf	25111	0.110330699	12742	1.198786E-01

Spearman Rank correlation analysis performed between Prdm1 and all-expressed genes within the Meredith RNA-seq dataset. Robust Prdm1–associated genes were identified using a cut-off of $p < 0.0005$.

Table S1, Related to Supplemental Figure 3C. Prdm1 associated genes

Nenf	25112	-0.075815823	870	2.859657E-01
Neo1	25113	0.123061825	13846	8.255443E-02
Nepn	25114	0.082483721	10788	2.455752E-01
Nes	25115	-0.05397389	2116	4.478076E-01
Nespas	25116	-0.038406171	3065	5.892384E-01
Net1	25117	0.127395013	14146	7.222828E-02
Neto1	25118	-0.086755146	452	2.218903E-01
Neto2	25119	0.001377867	5382	9.845508E-01
Neu1	25120	0.210257145	18583	2.805116E-03
Neu2	25121	0.083171863	10847	2.416450E-01
Neu3	25122	0.16794744	16818	1.744662E-02
Neu4	25123	-0.066858259	1029.5	3.468868E-01
Neurl1a	25124	-0.104054756	214	1.425622E-01
Neurl1b	25125	0.206466154	18475	3.354431E-03
Neurl2	25126		#N/A	1.000000E+00
Neurl3	25127	0.026214998	6882	7.125186E-01
Neurl4	25128	0.031838395	7179	6.544750E-01
Neurod1	25129	-0.038406171	3065	5.892384E-01
Neurod2	25130	-0.066858259	1029.5	3.468868E-01
Neurod4	25131	0.022198129	6607.5	7.550408E-01
Neurod6	25132		#N/A	1.000000E+00
Neurog1	25133	-0.038406171	3065	5.892384E-01
Neurog2	25134		#N/A	1.000000E+00
Neurog3	25135		#N/A	1.000000E+00
Nexn	25136		#N/A	1.000000E+00
Nf1	25137	0.319968787	19965	3.861836E-06
Nf2	25138	0.119639086	13634	9.152395E-02
Nfam1	25139	0.00016526	5296	9.981469E-01
Nfasc	25140	0.001181751	5373	9.867496E-01
Nfat5	25141	0.189631333	17827	7.157431E-03
Nfatc1	25142	-0.063148377	1358	3.743567E-01
Nfatc2	25143	0.009446871	5746	8.943796E-01
Nfatc2ip	25144	0.019820411	6325	7.805723E-01
Nfatc3	25145	0.205995486	18459	3.429018E-03
Nfatc4	25146	0.113548679	13094	1.093908E-01
Nfe2	25147		#N/A	1.000000E+00
Nfe2l1	25148	0.090611842	11394	2.019416E-01
Nfe2l2	25149	0.208976045	18548	2.980884E-03
Nfe2l3	25150	0.111516974	12821	1.159249E-01
Nfia	25151	0.180098306	17408	1.071338E-02
Nfib	25152	0.159616294	16257	2.396683E-02
Nfic	25153	-0.032542948	3810	6.473381E-01
Nfil3	25154	-0.103745115	216	1.437607E-01

Spearman Rank correlation analysis performed between Prdm1 and all-expressed genes within the Meredith RNA-seq dataset. Robust Prdm1–associated genes were identified using a cut-off of $p < 0.0005$.

Table S1, Related to Supplemental Figure 3C. Prdm1 associated genes

Nfix	25155	0.088922271	11265	2.105151E-01
Nfkb1	25156	0.174231242	17135	1.360755E-02
Nfkb2	25157	-0.002567286	5047	9.712191E-01
Nfkbia	25158	0.175363104	17181	1.300102E-02
Nfkbib	25159	0.155135164	15989	2.827225E-02
Nfkbid	25160	0.1683812	16842	1.715418E-02
Nfkbie	25161	0.120801269	13711	8.839505E-02
Nfkbil1	25162	0.063826635	9458	3.692372E-01
Nfkbiz	25163	0.077611648	10449	2.746735E-01
Nfrkb	25164	-0.013165851	4609.5	8.532022E-01
Nfs1	25165	0.071100078	9918	3.170825E-01
Nfu1	25166	-0.022819764	4151	7.484081E-01
Nfx1	25167	0.186287674	17694	8.262453E-03
Nfxl1	25168	0.064684597	9518	3.628236E-01
Nfya	25169	0.35607073	20008	2.283482E-07
Nfyb	25170	0.07248455	10031	3.077268E-01
Nfyc	25171	0.254377719	19557	2.781190E-04
Ngb	25172	0.022198129	6607.5	7.550408E-01
Ngdn	25173	0.134687407	14600	5.723632E-02
Ngef	25174	0.103800425	12285	1.435460E-01
Ngf	25175	-0.101041068	269	1.545548E-01
Ngfr	25176	-0.055648288	1501	4.338271E-01
Ngfrap1	25177	0.145083395	15357	4.038527E-02
Ngly1	25178	0.220162581	18903	1.732769E-03
Ngp	25179	-0.077393517	797	2.760287E-01
Ngrn	25180	0.253463468	19544	2.930548E-04
Nhej1	25181	0.013739451	5978	8.468835E-01
Nhlh1	25182		#N/A	1.000000E+00
Nhlh2	25183	-0.086750697	472.5	2.219141E-01
Nhlrc1	25184	0.183444947	17566	9.318435E-03
Nhlrc2	25185	0.074276229	10161	2.958913E-01
Nhlrc3	25186	0.058384012	9116	4.115323E-01
Nhlrc4	25187	-0.038406171	3065	5.892384E-01
Nhp2	25188	0.119718631	13639	9.130702E-02
Nhp2l1	25189	0.084369913	10948	2.349079E-01
Nhs	25190	-0.043507898	2391	5.407180E-01
Nhsl1	25191	0.149222337	15604	3.495174E-02
Nhsl2	25192	0.046760892	8062	5.108507E-01
Niacr1	25193	0.144844251	15345	4.071982E-02
Nicn1	25194	0.010816298	5814	8.791781E-01
Nid1	25195	0.001361001	5381	9.847399E-01
Nid2	25196	0.138070125	14827	5.121003E-02
Nif3l1	25197	0.203525793	18372	3.845506E-03

Spearman Rank correlation analysis performed between Prdm1 and all-expressed genes within the Meredith RNA-seq dataset. Robust Prdm1–associated genes were identified using a cut-off of $p < 0.0005$.

Table S1, Related to Supplemental Figure 3C. Prdm1 associated genes

Nim1	25198	0.023880557	6740	7.371325E-01
Nin	25199	0.002978957	5471	9.666064E-01
Ninj1	25200	0.033545438	7270	6.372385E-01
Ninj2	25201	0.016286999	6135	8.189432E-01
Ninl	25202	-0.04877916	2222	4.927598E-01
Nip7	25203	0.151434059	15741	3.231136E-02
Nipa1	25204	-0.014348857	4551	8.401810E-01
Nipa2	25205	0.128468185	14209	6.984010E-02
Nipal1	25206	0.280083129	19798	5.898399E-05
Nipal2	25207	0.125058824	13972	7.765681E-02
Nipal3	25208	0.039850171	7630	5.753044E-01
Nipal4	25209	0.081465186	10698	2.514736E-01
Nipbl	25210	0.091471135	11481	1.976794E-01
Nipsnap1	25211	0.187448686	17741	7.862685E-03
Nipsnap3a	25212	-0.066856562	1139	3.468991E-01
Nipsnap3b	25213	0.069210407	9795	3.301478E-01
Nisch	25214	0.1759731	17220	1.268409E-02
Nit1	25215	0.250234499	19510	3.519806E-04
Nit2	25216	0.031558867	7162	6.573153E-01
Nkain1	25217	-0.001606767	5097.5	9.819847E-01
Nkain2	25218	-0.095269893	339	1.796190E-01
Nkain3	25219	0.210849571	18604	2.727057E-03
Nkain4	25220	0.080135012	10604	2.593234E-01
Nkap	25221	-0.011097897	4699	8.760574E-01
Nkapl	25222	-0.000841282	5208.5	9.905669E-01
Nkd1	25223	0.224201696	19023	1.415140E-03
Nkd2	25224	0.037189906	7444	6.010933E-01
Nkg7	25225	0.143605682	15224	4.249002E-02
Nkiras1	25226	0.140475442	14986	4.725368E-02
Nkiras2	25227	0.237643255	19318	7.030421E-04
Nkpd1	25228	0.053805701	8820	4.492258E-01
Nkrf	25229	0.023977264	6753	7.361073E-01
Nktr	25230	0.072448783	10028	3.079662E-01
Nkx1-1	25231		#N/A	1.000000E+00
Nkx1-2	25232	-0.066855996	1239.5	3.469032E-01
Nkx2-1	25233	0.113548679	13094	1.093908E-01
Nkx2-2	25234	-0.054450825	1984	4.437997E-01
Nkx2-2as	25235	0.03037917	7087.5	6.693560E-01
Nkx2-3	25236	-0.066858259	1029.5	3.468868E-01
Nkx2-4	25237	0.021316405	6469	7.644793E-01
Nkx2-5	25238		#N/A	1.000000E+00
Nkx2-6	25239	-0.026647474	4043	7.079903E-01
Nkx2-9	25240	0.143605682	15224	4.249002E-02

Spearman Rank correlation analysis performed between Prdm1 and all-expressed genes within the Meredith RNA-seq dataset. Robust Prdm1–associated genes were identified using a cut-off of $p < 0.0005$.

Table S1, Related to Supplemental Figure 3C. Prdm1 associated genes

Nkx3-1	25241	0.08165753	10714	2.503523E-01
Nkx3-2	25242		#N/A	1.000000E+00
Nkx6-1	25243		#N/A	1.000000E+00
Nkx6-2	25244	-0.073494512	914	3.010175E-01
Nkx6-3	25245		#N/A	1.000000E+00
Nle1	25246	-0.039509404	2481	5.785787E-01
Nlgn1	25247	0.253244081	19542	2.967481E-04
Nlgn2	25248	0.047153255	8091.5	5.073070E-01
Nlgn3	25249	0.1962304	18093	5.355252E-03
Nlk	25250	-0.00754645	4839	9.155383E-01
Nlk-ps1	25251	0.143605682	15224	4.249002E-02
Nln	25252	0.069120592	9788	3.307773E-01
Nlrc3	25253	0.064488247	9504	3.642853E-01
Nlrc4	25254	0.073951143	10133	2.980160E-01
Nlrc5	25255	0.172123086	17029	1.480364E-02
Nlrp10	25256	-0.059767536	1400	4.005191E-01
NLRP12	25257		#N/A	1.000000E+00
Nlrp12	25258	0.033526957	7269	6.374241E-01
Nlrp14	25259	0.113548679	13094	1.093908E-01
Nlrp1a	25260	0.106510101	12461	1.333266E-01
Nlrp1b	25261	-0.018790377	4285	7.917077E-01
Nlrp1c-ps	25262	-0.038406171	3065	5.892384E-01
Nlrp2	25263	0.021712385	6565.5	7.602361E-01
Nlrp3	25264	0.150808544	15701	3.304032E-02
Nlrp4a	25265	-0.066856562	1139	3.468991E-01
Nlrp4b	25266	-0.038406171	3065	5.892384E-01
Nlrp4c	25267	-0.054451513	1703	4.437940E-01
Nlrp4d	25268		#N/A	1.000000E+00
Nlrp4e	25269	0.06131197	9286	3.884350E-01
Nlrp4f	25270	-0.004937246	4940	9.446824E-01
Nlrp4g	25271		#N/A	1.000000E+00
Nlrp5	25272	0.218835436	18868	1.850551E-03
Nlrp5-ps	25273	-0.038406171	3065	5.892384E-01
Nlrp6	25274	0.090319075	11370.5	2.034088E-01
Nlrp9a	25275	0.094140343	11651	1.848572E-01
Nlrp9b	25276	0.013067932	5938	8.542818E-01
Nlrp9c	25277	-0.066856562	1139	3.468991E-01
Nlrx1	25278	0.091855237	11511	1.957955E-01
Nmb	25279	0.230247091	19167.5	1.038230E-03
Nmbr	25280		#N/A	1.000000E+00
Nmd3	25281	0.114110653	13260	1.076354E-01
Nme1	25282	0.129116387	14246	6.842905E-02
Nme2	25283	0.13891426	14877	4.979133E-02

Spearman Rank correlation analysis performed between Prdm1 and all-expressed genes within the Meredith RNA-seq dataset. Robust Prdm1–associated genes were identified using a cut-off of $p < 0.0005$.

Table S1, Related to Supplemental Figure 3C. Prdm1 associated genes

Nme3	25284	0.222486503	18970	1.542876E-03
Nme4	25285	0.151879602	15757.5	3.180051E-02
Nme5	25286	0.246579263	19464	4.318629E-04
Nme6	25287	0.061673502	9313	3.856384E-01
Nme7	25288	0.207317194	18505	3.223279E-03
Nme8	25289	0.086412184	11087.5	2.237298E-01
Nmi	25290	0.224864805	19040	1.368406E-03
Nmnat1	25291	0.042787587	7789	5.474467E-01
Nmnat2	25292	0.171690168	17004	1.506034E-02
Nmnat3	25293	0.185652896	17665	8.488571E-03
Nmral1	25294	0.212485749	18676	2.521593E-03
Nmrk1	25295	0.024033599	6757	7.355103E-01
Nmrk2	25296	0.087512912	11168	2.178641E-01
Nms	25297	-0.077393517	797	2.760287E-01
Nmt1	25298	0.039499237	7603	5.786765E-01
Nmt2	25299	0.000149394	5295	9.983248E-01
Nmu	25300	-0.038406171	3065	5.892384E-01
Nmur1	25301		#N/A	1.000000E+00
Nmur2	25302	-0.013118278	4616	8.537267E-01
Nnat	25303	0.026516957	6904	7.093558E-01
Nnmt	25304	-0.038406171	3065	5.892384E-01
Nnt	25305	0.134403247	14585	5.776816E-02
Noa1	25306	-0.000918299	5189	9.897033E-01
Nob1	25307	0.12626409	14062	7.481637E-02
Nobox	25308		#N/A	1.000000E+00
Noc2l	25309	0.102344606	12203	1.492774E-01
Noc3l	25310	0.07842109	10492	2.696838E-01
Noc4l	25311	0.066221328	9604	3.515102E-01
Nod1	25312	-0.038406171	3065	5.892384E-01
Nod2	25313	0.005094137	5568	9.429275E-01
Nodal	25314		#N/A	1.000000E+00
Nog	25315	0.043074077	7812	5.447655E-01
Nol10	25316	0.220961488	18931	1.665207E-03
Nol11	25317	0.195226217	18053	5.600170E-03
Nol12	25318	0.174806837	17156	1.329608E-02
Nol3	25319	0.125556225	14006	7.647420E-02
Nol4	25320	-0.164925328	7	1.960718E-02
Nol6	25321	0.095153449	11707	1.801539E-01
Nol7	25322	-0.019990899	4241	7.787334E-01
Nol8	25323	-0.046450871	2274	5.136598E-01
Nol9	25324	0.028073836	6977	6.931283E-01
Nolc1	25325	0.118013362	13528	9.604834E-02
Nom1	25326	0.164972868	16655	1.957148E-02

Spearman Rank correlation analysis performed between Prdm1 and all-expressed genes within the Meredith RNA-seq dataset. Robust Prdm1–associated genes were identified using a cut-off of $p < 0.0005$.

Table S1, Related to Supplemental Figure 3C. Prdm1 associated genes

Nomo1	25327	0.054220401	8877	4.457335E-01
Nono	25328	0.207450246	18513	3.203199E-03
Nop10	25329	0.07832634	10489	2.702647E-01
Nop14	25330	0.219230676	18882	1.814733E-03
Nop16	25331	0.159335769	16243	2.421875E-02
Nop2	25332	0.01902388	6281	7.891796E-01
Nop56	25333	0.021711264	6563	7.602481E-01
Nop58	25334	0.106209832	12439	1.344307E-01
Nop9	25335	0.218060417	18845	1.922665E-03
Nos1	25336	0.221045549	18934	1.658240E-03
Nos1ap	25337	0.017059182	6172	8.105175E-01
Nos2	25338	0.007265829	5643	9.186681E-01
Nos3	25339	-0.124244152	86	7.962564E-02
Nosip	25340	0.203638158	18376	3.825608E-03
Nostrin	25341	-0.090141725	414	2.043013E-01
Notch1	25342	0.057202505	9045	4.210771E-01
Notch2	25343	0.178986094	17354	1.121615E-02
Notch3	25344	-0.01420371	4557	8.417764E-01
Notch4	25345	-0.038406171	3065	5.892384E-01
Noto	25346	0.010792064	5810	8.794467E-01
Notum	25347		#N/A	1.000000E+00
Nov	25348	0.072136643	10006	3.100606E-01
Nova1	25349	-0.077336512	839	2.763837E-01
Nova2	25350	0.203154381	18366	3.911941E-03
Nox1	25351	0.019221858	6293	7.870378E-01
Nox3	25352	0.015218018	6080	8.306417E-01
Nox4	25353	0.133652944	14524	5.919203E-02
Noxa1	25354	-0.066858259	1029.5	3.468868E-01
Noxo1	25355	0.256524699	19587	2.457830E-04
Noxred1	25356	-0.077394997	736.5	2.760195E-01
Npas1	25357	-0.11057391	159	1.190596E-01
Npas2	25358	-0.054195968	2108	4.459389E-01
Npas3	25359	0.095090356	11703	1.804442E-01
Npas4	25360		#N/A	1.000000E+00
Npat	25361	0.238746111	19344	6.626479E-04
Npb	25362	0.146505133	15448	3.844376E-02
Npbwr1	25363	0.011123458	5833	8.757743E-01
Npc1	25364	0.158574339	16206	2.491405E-02
Npc1l1	25365	-0.033075221	3785	6.419675E-01
Npc2	25366	0.2284536	19104	1.139095E-03
Npcd	25367		#N/A	1.000000E+00
Npdc1	25368	0.133184724	14488	6.009515E-02
Npepl1	25369	-0.001828262	5082	9.795018E-01

Spearman Rank correlation analysis performed between Prdm1 and all-expressed genes within the Meredith RNA-seq dataset. Robust Prdm1–associated genes were identified using a cut-off of $p < 0.0005$.

Table S1, Related to Supplemental Figure 3C. Prdm1 associated genes

Npepps	25370	0.092972089	11583	1.903920E-01
Npff	25371	0.136303874	14716	5.428731E-02
Npffr1	25372	-0.077394997	736.5	2.760195E-01
Npffr2	25373	-0.100297238	274	1.576286E-01
Nphp1	25374	0.069657678	9827	3.270245E-01
Nphp3	25375	0.063451646	9429	3.720623E-01
Nphp4	25376	-0.038406171	3065	5.892384E-01
Nphs1	25377	0.040981791	7693	5.644939E-01
Nphs1as	25378		#N/A	1.000000E+00
Nphs2	25379	0.127545848	14155	7.188866E-02
Npl	25380	-0.051502448	2172	4.688999E-01
Nploc4	25381	0.138841733	14872	4.991192E-02
Npm1	25382	0.141819678	15070	4.515602E-02
Npm2	25383	-0.054450825	1984	4.437997E-01
Npm3	25384	0.017425353	6199	8.065295E-01
Npm3-ps1	25385		#N/A	1.000000E+00
Npn2	25386		#N/A	1.000000E+00
Npnt	25387	-0.022529462	4158	7.515033E-01
Nppa	25388	0.112540185	12893	1.125971E-01
Nppb	25389	0.143605682	15224	4.249002E-02
Nppc	25390	-0.086750697	472.5	2.219141E-01
Npr1	25391	0.196522408	18103	5.285856E-03
Npr2	25392	0.053267784	8685	4.537786E-01
Npr3	25393	0.108309397	12570	1.268556E-01
Nprl2	25394	0.110376448	12746	1.197242E-01
Nprl3	25395	-0.040782941	2456	5.663865E-01
Nps	25396		#N/A	1.000000E+00
Npsr1	25397	0.126176002	14057	7.502107E-02
Nptn	25398	0.097938055	11911	1.676819E-01
Nptx1	25399	-0.051901522	2167	4.654576E-01
Nptx2	25400	-0.033628159	3761	6.364081E-01
Nptxr	25401	0.067066721	9651	3.453820E-01
Npvf	25402	0.030375125	7082	6.693974E-01
Npw	25403	0.082935497	10824	2.429900E-01
Npy	25404	0.174964841	17164	1.321168E-02
Npy1r	25405	0.205821629	18453	3.456945E-03
Npy2r	25406	-0.038406171	3065	5.892384E-01
Npy5r	25407		#N/A	1.000000E+00
Npy6r	25408		#N/A	1.000000E+00
Nqo1	25409	0.091497552	11486	1.975494E-01
Nqo2	25410	-0.007203744	4846	9.193607E-01
Nr0b1	25411		#N/A	1.000000E+00
Nr0b2	25412		#N/A	1.000000E+00

Spearman Rank correlation analysis performed between Prdm1 and all-expressed genes within the Meredith RNA-seq dataset. Robust Prdm1–associated genes were identified using a cut-off of $p < 0.0005$.

Table S1, Related to Supplemental Figure 3C. Prdm1 associated genes

Nr1d1	25413	-0.099615394	280	1.604865E-01
Nr1d2	25414	-0.003714559	4994	9.583672E-01
Nr1h2	25415	0.114749547	13308	1.056667E-01
Nr1h3	25416	0.051225546	8398	4.712966E-01
Nr1h4	25417	0.241470033	19384	5.718792E-04
Nr1h5	25418	-0.016731569	4428.5	8.140896E-01
Nr1i2	25419		#N/A	1.000000E+00
Nr1i3	25420	0.020875719	6412.5	7.692098E-01
Nr2c1	25421	0.034299095	7304	6.296895E-01
Nr2c2	25422	0.160916505	16385	2.282845E-02
Nr2c2ap	25423	0.085168214	11000	2.304926E-01
Nr2e1	25424	0.080867205	10654	2.549819E-01
Nr2e3	25425	0.046062578	8016	5.171894E-01
Nr2f1	25426	0.299061029	19904	1.694822E-05
Nr2f2	25427	0.194474941	18023	5.789913E-03
Nr2f6	25428	0.179398156	17377	1.102751E-02
Nr3c1	25429	0.072926664	10058	3.047778E-01
Nr3c2	25430	0.107862783	12545	1.284387E-01
Nr4a1	25431	0.117657588	13506	9.706176E-02
Nr4a2	25432	0.029324307	7035	6.801946E-01
Nr4a3	25433	0.063779293	9456	3.695932E-01
Nr5a1	25434		#N/A	1.000000E+00
Nr5a2	25435	0.242839091	19412	5.307320E-04
Nr6a1	25436	0.150531619	15682	3.336748E-02
Nradd	25437	-0.001223987	5172.5	9.862760E-01
Nrap	25438	0.016286902	6134	8.189443E-01
Nrarp	25439	-0.058901303	1419	4.073938E-01
Nras	25440	0.241794558	19394	5.618673E-04
Nrbf2	25441	0.007370491	5650	9.175006E-01
Nrbp1	25442	-0.065779977	1310	3.547365E-01
Nrbp2	25443	0.037485366	7457	5.982036E-01
Nrcam	25444	0.105757753	12405	1.361063E-01
Nrd1	25445	0.117898623	13519	9.637425E-02
Nrde2	25446	-0.018766046	4295	7.919713E-01
Nrep	25447	0.03306841	7244	6.420362E-01
Nrf1	25448	0.15629137	16070	2.710247E-02
Nrg1	25449	0.014332903	6031	8.403563E-01
Nrg2	25450	0.07678706	10398	2.798204E-01
Nrg3	25451	0.10286012	12238	1.472284E-01
Nrg4	25452	-0.00589481	4884	9.339761E-01
Nrgn	25453	0.025691528	6857	7.180133E-01
Nrip1	25454	0.021359341	6545	7.640188E-01
Nrip2	25455	-0.041139327	2448	5.629967E-01

Spearman Rank correlation analysis performed between Prdm1 and all-expressed genes within the Meredith RNA-seq dataset. Robust Prdm1–associated genes were identified using a cut-off of $p < 0.0005$.

Table S1, Related to Supplemental Figure 3C. Prdm1 associated genes

Nrip3	25456	-0.056165168	1479.5	4.295625E-01
Nrk	25457	-0.017641277	4383	8.041802E-01
Nrl	25458	0.13398767	14550	5.855328E-02
Nrm	25459	0.16501107	16658	1.954283E-02
Nrn1	25460	-0.016731055	4437	8.140953E-01
Nrn1l	25461		#N/A	1.000000E+00
Nron	25462		#N/A	1.000000E+00
Nrp1	25463	0.177881047	17306	1.173617E-02
Nrp2	25464	0.035519664	7350	6.175449E-01
Nrsn1	25465	0.197090802	18124	5.153085E-03
Nrsn2	25466	-0.054450825	1984	4.437997E-01
Nrtn	25467	-0.063818778	1343.5	3.692963E-01
Nrxn1	25468	0.091102047	11447	1.995021E-01
Nrxn2	25469	0.115667869	13370	1.028868E-01
Nrxn3	25470	0.186186858	17689	8.298004E-03
Nsa2	25471	0.183157264	17555	9.431688E-03
Nsa2-ps2	25472	-0.038406171	3065	5.892384E-01
Nsd1	25473	0.131804178	14394	6.282419E-02
Nsdhl	25474	0.090684434	11400	2.015790E-01
Nsf	25475	0.304924808	19920	1.132112E-05
Nsfl1c	25476	0.099171274	12008	1.623687E-01
Nsg1	25477	0.013384031	5957	8.507976E-01
Nsg2	25478	0.161195058	16434	2.259072E-02
Nsl1	25479	0.139983821	14951	4.804088E-02
Nsmaf	25480	0.16127666	16439	2.252148E-02
Nsmce1	25481	-0.022278894	4166	7.541780E-01
Nsmce2	25482	0.126800888	14095	7.357870E-02
Nsmce4a	25483		#N/A	1.000000E+00
Nsmf	25484	-0.028242737	3988	6.913761E-01
Nsun2	25485	0.046393722	8041	5.141785E-01
Nsun3	25486	0.139958613	14947	4.808154E-02
Nsun4	25487	0.06073401	9249	3.929311E-01
Nsun5	25488	0.053267784	8685	4.537786E-01
Nsun6	25489	-0.022438132	4162	7.524778E-01
Nsun7	25490	-0.064482049	1332	3.643315E-01
Nt5c	25491	0.116591592	13440	1.001491E-01
Nt5c1a	25492		#N/A	1.000000E+00
Nt5c1b	25493		#N/A	1.000000E+00
Nt5c2	25494	0.087861783	11193	2.160281E-01
Nt5c3	25495	0.111173273	12800	1.170598E-01
Nt5c3b	25496	0.143420928	15159	4.275954E-02
Nt5dc1	25497	0.07119533	9927	3.164330E-01
Nt5dc2	25498	0.028628701	6999	6.873781E-01

Spearman Rank correlation analysis performed between Prdm1 and all-expressed genes within the Meredith RNA-seq dataset. Robust Prdm1–associated genes were identified using a cut-off of $p < 0.0005$.

Table S1, Related to Supplemental Figure 3C. Prdm1 associated genes

Nt5dc3	25499	0.134036003	14555	5.846152E-02
Nt5e	25500	0.064123369	9476.5	3.670112E-01
Nt5m	25501	0.024456805	6786	7.310306E-01
Ntan1	25502	0.046296852	8035	5.150584E-01
Ntf3	25503	0.098163581	11926	1.667006E-01
Ntf5	25504	-0.051977048	2164	4.648077E-01
Nthl1	25505	-0.054515251	1535	4.432599E-01
Ntm	25506	0.278956233	19786	6.334167E-05
Ntmt1	25507	0.118736414	13569	9.401462E-02
Ntn1	25508	0.236393784	19296	7.515433E-04
Ntn3	25509		#N/A	1.000000E+00
Ntn4	25510	0.063885692	9463	3.687935E-01
Ntn5	25511	-0.083249794	598	2.412027E-01
Ntng1	25512	0.129956295	14297	6.663537E-02
Ntng2	25513	0.032658185	7215	6.461738E-01
Ntpcr	25514	-0.056965126	1462	4.230102E-01
Ntrk1	25515		#N/A	1.000000E+00
Ntrk2	25516	0.140280053	14973	4.756524E-02
Ntrk3	25517	0.026992586	6931	7.043840E-01
Nts	25518	0.002605603	5451	9.707897E-01
Ntsr1	25519	0.00309841	5477	9.652682E-01
Ntsr2	25520		#N/A	1.000000E+00
Nuak1	25521	0.105377644	12383	1.375275E-01
Nuak2	25522	0.16145171	16449	2.237358E-02
Nub1	25523	0.17558439	17194	1.288525E-02
Nubp1	25524	0.127666545	14167	7.161784E-02
Nubp2	25525	0.007950137	5677	9.110383E-01
Nubpl	25526	0.05175227	8453	4.667434E-01
Nucb1	25527	0.088064255	11209	2.149676E-01
Nucb2	25528	0.115682136	13373	1.028441E-01
Nucks1	25529	0.237432834	19315	7.110026E-04
Nudc	25530	0.108915983	12617	1.247296E-01
Nudcd1	25531	0.155974771	16035	2.741859E-02
Nudcd2	25532	0.096029336	11776	1.761593E-01
Nudcd3	25533	0.158002712	16174	2.544724E-02
Nudt1	25534	-0.018873369	4276	7.908089E-01
Nudt10	25535	0.037149308	7440	6.014908E-01
Nudt11	25536	-0.05453613	1532	4.430850E-01
Nudt12	25537	0.233683405	19251	8.675291E-04
Nudt13	25538	0.046575156	8050	5.125327E-01
Nudt14	25539	0.164269073	16607	2.010587E-02
Nudt15	25540	0.07508192	10252	2.906689E-01
Nudt16	25541	-0.00518336	4922	9.419296E-01

Spearman Rank correlation analysis performed between Prdm1 and all-expressed genes within the Meredith RNA-seq dataset. Robust Prdm1–associated genes were identified using a cut-off of $p < 0.0005$.

Table S1, Related to Supplemental Figure 3C. Prdm1 associated genes

Nudt16l1	25542	-0.00793511	4824	9.112058E-01
Nudt17	25543	-0.075315317	880	2.891676E-01
Nudt18	25544	0.166286796	16731	1.860670E-02
Nudt19	25545	-0.002158755	5067	9.757974E-01
Nudt2	25546	0.18015059	17412	1.069025E-02
Nudt21	25547	0.21550951	18777	2.178477E-03
Nudt22	25548	0.023176109	6702	7.446142E-01
Nudt3	25549	0.076482519	10380	2.817377E-01
Nudt4	25550	0.157788509	16142	2.564955E-02
Nudt5	25551	0.194797388	18035	5.707782E-03
Nudt6	25552	0.135015019	14620	5.662819E-02
Nudt7	25553	-0.015225271	4502	8.305622E-01
Nudt8	25554	-0.021952439	4173	7.576672E-01
Nudt9	25555	0.019760357	6317	7.812203E-01
Nuf2	25556	0.069885368	9836	3.254419E-01
Nufip1	25557	0.169575366	16906	1.637100E-02
Nufip2	25558	0.18028401	17418	1.063140E-02
Nuggc	25559	0.104446629	12321	1.410564E-01
Numa1	25560	0.088292967	11229	2.137742E-01
Numb	25561	0.112344832	12882	1.132266E-01
Numbl	25562	0.029456652	7042	6.788311E-01
Nup107	25563	0.161126671	16430	2.264888E-02
Nup133	25564	0.081708167	10720	2.500576E-01
Nup153	25565	0.26333702	19660	1.648765E-04
Nup155	25566	0.148423803	15555	3.594894E-02
Nup160	25567	0.091120134	11451	1.994125E-01
Nup188	25568	0.13572246	14673	5.533319E-02
Nup205	25569	0.065142076	9541	3.594322E-01
Nup210	25570	0.189839018	17834	7.093354E-03
Nup210l	25571	0.189031743	17803	7.345326E-03
Nup214	25572	-0.011097356	4700	8.760634E-01
Nup35	25573	0.080439397	10629	2.575124E-01
Nup37	25574	0.048699462	8194	4.934676E-01
Nup43	25575	0.046844617	8067	5.100935E-01
Nup50	25576	0.135916835	14693	5.498170E-02
Nup54	25577	0.155075286	15985	2.833399E-02
Nup62	25578	0.195084777	18050	5.635461E-03
Nup62cl	25579	0.140000464	14953	4.801405E-02
Nup85	25580	0.11584595	13384	1.023545E-01
Nup88	25581	0.169185371	16883	1.662328E-02
Nup93	25582	0.170914176	16971	1.553020E-02
Nup98	25583	0.059292729	9174	4.042788E-01
Nupl1	25584	0.078990991	10530	2.662080E-01

Spearman Rank correlation analysis performed between Prdm1 and all-expressed genes within the Meredith RNA-seq dataset. Robust Prdm1–associated genes were identified using a cut-off of $p < 0.0005$.

Table S1, Related to Supplemental Figure 3C. Prdm1 associated genes

Nupl2	25585	-0.007304169	4842	9.182404E-01
Nupr1	25586	0.005447604	5579	9.389748E-01
Nupr1l	25587	0.046130979	8020	5.165667E-01
Nus1	25588	0.189599009	17825	7.167450E-03
Nusap1	25589	0.078998095	10531	2.661649E-01
Nutf2	25590	0.135151508	14632	5.637641E-02
Nutf2-ps1	25591		#N/A	1.000000E+00
Nutm1	25592		#N/A	1.000000E+00
Nvl	25593	0.029886049	7060	6.744144E-01
Nwd1	25594	0.162755758	16510	2.129788E-02
Nxf1	25595	0.214353493	18725	2.304296E-03
Nxf2	25596	-0.017760443	4381	8.028844E-01
Nxf3	25597	0.113548679	13094	1.093908E-01
Nxf7	25598	0.151119933	15719	3.267570E-02
Nxn	25599	0.162142925	16475	2.179770E-02
Nxnl1	25600	-0.002881632	5031	9.676968E-01
Nxnl2	25601	-0.038743724	2495	5.859673E-01
Nxpe2	25602	-0.091888749	404	1.956318E-01
Nxpe3	25603	0.080244528	10616	2.586708E-01
Nxpe4	25604	0.167970606	16820	1.743089E-02
Nxpe5	25605	-0.038406171	3065	5.892384E-01
Nxph1	25606	0.130530898	14325	6.543054E-02
Nxph2	25607	-0.083183554	600.5	2.415786E-01
Nxph3	25608		#N/A	1.000000E+00
Nxph4	25609	-0.054450825	1984	4.437997E-01
Nxt1	25610	0.276800594	19772	7.253258E-05
Nxt2	25611	0.084987412	10987	2.314875E-01
Nyap1	25612		#N/A	1.000000E+00
Nyap2	25613	0.030367779	7079	6.694727E-01
Nynrin	25614	-0.082020616	619	2.482450E-01
Nyx	25615	-0.054789901	1526	4.409628E-01
Oacyl	25616	-0.077397959	691.5	2.760011E-01
Oaf	25617	0.046086651	8017	5.169702E-01
Oard1	25618	0.044659826	7911	5.300433E-01
Oas1a	25619	-0.072876322	922	3.051126E-01
Oas1b	25620	0.177687534	17296	1.182938E-02
Oas1c	25621	0.189633988	17828	7.156609E-03
Oas1d	25622	-0.015749716	4486	8.248182E-01
Oas1e	25623	0.080122579	10602	2.593975E-01
Oas1f	25624	-0.054450825	1984	4.437997E-01
Oas1g	25625	-0.013540297	4588.5	8.490763E-01
Oas1h	25626	0.021211712	6450	7.656023E-01
Oas2	25627	0.219781289	18894.5	1.765887E-03

Spearman Rank correlation analysis performed between Prdm1 and all-expressed genes within the Meredith RNA-seq dataset. Robust Prdm1–associated genes were identified using a cut-off of $p < 0.0005$.

Table S1, Related to Supplemental Figure 3C. Prdm1 associated genes

Oas3	25628	0.181783924	17477	9.989294E-03
Oasl1	25629	-0.013134462	4613	8.535483E-01
Oasl2	25630	0.05605315	8988	4.304847E-01
Oat	25631		#N/A	1.000000E+00
Oat-rs1	25632		#N/A	1.000000E+00
Oaz1	25633	0.231905633	19211	9.523210E-04
Oaz1-ps	25634		#N/A	1.000000E+00
Oaz2	25635	0.101392852	12143	1.531169E-01
Oaz2-ps	25636		#N/A	1.000000E+00
Oaz3	25637		#N/A	1.000000E+00
Obfc1	25638	0.177159351	17273	1.208713E-02
Obox1	25639	-0.016695423	4452	8.144840E-01
Obox2	25640		#N/A	1.000000E+00
Obox3	25641	0.022198316	6629.5	7.550388E-01
Obox5	25642		#N/A	1.000000E+00
Obox6	25643		#N/A	1.000000E+00
Obp1a	25644	-0.043103239	2406	5.444930E-01
Obp1b	25645		#N/A	1.000000E+00
Obp2a	25646	0.004708517	5544	9.472413E-01
Obp2b	25647		#N/A	1.000000E+00
Obscn	25648	-0.15292573	23	3.062799E-02
Obsl1	25649	-0.002215249	5066	9.751643E-01
Oc90	25650	-0.086746694	532	2.219355E-01
Oca2	25651	0.011185874	5837	8.750829E-01
Ocel1	25652	0.082286835	10775	2.467078E-01
Ociad1	25653	0.179080055	17359	1.117289E-02
Ociad2	25654	-0.031314777	3872	6.597995E-01
Ocln	25655	0.210165928	18578	2.817314E-03
Ocm	25656		#N/A	1.000000E+00
Ocrl	25657	0.145583763	15384	3.969277E-02
Ocstamp	25658		#N/A	1.000000E+00
Odam	25659	0.053805701	8820	4.492258E-01
Odc1	25660	0.084885145	10980	2.320515E-01
Odf1	25661		#N/A	1.000000E+00
Odf2	25662	0.203136613	18365	3.915145E-03
Odf2l	25663	-0.006969154	4854	9.219782E-01
Odf3	25664	-0.066856562	1139	3.468991E-01
Odf3b	25665		#N/A	1.000000E+00
Odf3l1	25666	0.118865818	13576	9.365429E-02
Odf3l2	25667	-0.018447724	4312.5	7.954215E-01
Odf4	25668	0.126933796	14110.5	7.327484E-02
Ofcc1	25669	0.07924479	10548	2.646700E-01
Ofd1	25670	0.12391775	13901	8.042568E-02

Spearman Rank correlation analysis performed between Prdm1 and all-expressed genes within the Meredith RNA-seq dataset. Robust Prdm1–associated genes were identified using a cut-off of $p < 0.0005$.

Table S1, Related to Supplemental Figure 3C. Prdm1 associated genes

Ogdh	25671	0.256460338	19586	2.466992E-04
Ogdhl	25672	0.053267784	8685	4.537786E-01
Ogfod1	25673	0.026370915	6892	7.108849E-01
Ogfod2	25674	-0.029576633	3948	6.775959E-01
Ogfod3	25675	0.160314923	16353	2.334922E-02
Ogfr	25676	0.229639091	19142	1.071466E-03
Ogfrl1	25677	0.131799216	14393	6.283418E-02
Ogg1	25678	0.075849504	10335	2.857511E-01
Ogn	25679	-0.038406171	3065	5.892384E-01
Ogt	25680	0.182060499	17484	9.874711E-03
Oip5	25681	0.043818705	7854	5.378273E-01
Oit1	25682	0.086831193	11119	2.214838E-01
Oit3	25683	0.07494696	10242	2.915394E-01
Ola1	25684	0.276652497	19769	7.320791E-05
Olah	25685	0.050883637	8377	4.742652E-01
Olfm1	25686	0.097863762	11902	1.680060E-01
Olfm2	25687	-0.054451513	1703	4.437940E-01
Olfm3	25688	-0.038166547	3628	5.915655E-01
Olfm4	25689		#N/A	1.000000E+00
Olfml1	25690	-0.038406171	3065	5.892384E-01
Olfml2a	25691	0.193256873	17957	6.109795E-03
Olfml2b	25692	0.132586736	14453	6.126502E-02
Olfml3	25693	0.07494696	10242	2.915394E-01
Olfr1	25694		#N/A	1.000000E+00
Olfr10	25695		#N/A	1.000000E+00
Olfr1000	25696		#N/A	1.000000E+00
Olfr1001-ps1	25697		#N/A	1.000000E+00
Olfr1002	25698		#N/A	1.000000E+00
Olfr1003-ps1	25699		#N/A	1.000000E+00
Olfr1004-ps1	25700		#N/A	1.000000E+00
Olfr1006	25701	-0.038406171	3065	5.892384E-01
Olfr1008	25702	-0.038406171	3065	5.892384E-01
Olfr1009	25703		#N/A	1.000000E+00
Olfr101	25704	-0.038406171	3065	5.892384E-01
Olfr1010	25705		#N/A	1.000000E+00
Olfr1012	25706		#N/A	1.000000E+00
Olfr1013	25707		#N/A	1.000000E+00
Olfr1014	25708		#N/A	1.000000E+00
Olfr1015	25709		#N/A	1.000000E+00
Olfr1016	25710		#N/A	1.000000E+00
Olfr1017-ps1	25711		#N/A	1.000000E+00
Olfr1018	25712		#N/A	1.000000E+00
Olfr1019	25713		#N/A	1.000000E+00

Spearman Rank correlation analysis performed between Prdm1 and all-expressed genes within the Meredith RNA-seq dataset. Robust Prdm1–associated genes were identified using a cut-off of $p < 0.0005$.

Table S1, Related to Supplemental Figure 3C. Prdm1 associated genes

Olfr102	25714	-0.038406171	3065	5.892384E-01
Olfr1020	25715		#N/A	1.000000E+00
Olfr1021-ps1	25716		#N/A	1.000000E+00
Olfr1022	25717		#N/A	1.000000E+00
Olfr1023	25718		#N/A	1.000000E+00
Olfr1024	25719		#N/A	1.000000E+00
Olfr1025-ps1	25720		#N/A	1.000000E+00
Olfr1026	25721		#N/A	1.000000E+00
Olfr1027-ps1	25722		#N/A	1.000000E+00
Olfr1028	25723		#N/A	1.000000E+00
Olfr1029	25724		#N/A	1.000000E+00
Olfr103	25725	-0.038406171	3065	5.892384E-01
Olfr1030	25726		#N/A	1.000000E+00
Olfr1031	25727		#N/A	1.000000E+00
Olfr1032	25728		#N/A	1.000000E+00
Olfr1033	25729	0.063273093	9409	3.734121E-01
Olfr1034	25730		#N/A	1.000000E+00
Olfr1035-ps1	25731		#N/A	1.000000E+00
Olfr1036	25732		#N/A	1.000000E+00
Olfr1037	25733		#N/A	1.000000E+00
Olfr1038-ps	25734		#N/A	1.000000E+00
Olfr1039	25735		#N/A	1.000000E+00
Olfr104-ps	25736		#N/A	1.000000E+00
Olfr1040	25737		#N/A	1.000000E+00
Olfr1042	25738		#N/A	1.000000E+00
Olfr1043	25739		#N/A	1.000000E+00
Olfr1044	25740		#N/A	1.000000E+00
Olfr1045	25741		#N/A	1.000000E+00
Olfr1046	25742		#N/A	1.000000E+00
Olfr1047	25743		#N/A	1.000000E+00
Olfr1048	25744		#N/A	1.000000E+00
Olfr1049	25745		#N/A	1.000000E+00
Olfr105-ps	25746		#N/A	1.000000E+00
Olfr1050-ps1	25747		#N/A	1.000000E+00
Olfr1051	25748		#N/A	1.000000E+00
Olfr1052	25749		#N/A	1.000000E+00
Olfr1053	25750		#N/A	1.000000E+00
Olfr1054	25751		#N/A	1.000000E+00
Olfr1055	25752		#N/A	1.000000E+00
Olfr1056	25753		#N/A	1.000000E+00
Olfr1057	25754		#N/A	1.000000E+00
Olfr1058	25755		#N/A	1.000000E+00
Olfr106-ps	25756		#N/A	1.000000E+00

Spearman Rank correlation analysis performed between Prdm1 and all-expressed genes within the Meredith RNA-seq dataset. Robust Prdm1–associated genes were identified using a cut-off of $p < 0.0005$.

Table S1, Related to Supplemental Figure 3C. Prdm1 associated genes

Olfr1061	25757		#N/A	1.000000E+00
Olfr1062	25758		#N/A	1.000000E+00
Olfr1063-ps1	25759		#N/A	1.000000E+00
Olfr1065	25760		#N/A	1.000000E+00
Olfr1066	25761		#N/A	1.000000E+00
Olfr1069-ps1	25762		#N/A	1.000000E+00
Olfr107	25763		#N/A	1.000000E+00
Olfr1073-ps1	25764		#N/A	1.000000E+00
Olfr1076	25765		#N/A	1.000000E+00
Olfr1077-ps1	25766		#N/A	1.000000E+00
Olfr1078-ps1	25767		#N/A	1.000000E+00
Olfr1079	25768		#N/A	1.000000E+00
Olfr108	25769		#N/A	1.000000E+00
Olfr1080	25770	-0.054450825	1984	4.437997E-01
Olfr1081-ps1	25771		#N/A	1.000000E+00
Olfr1082	25772		#N/A	1.000000E+00
Olfr1083-ps	25773		#N/A	1.000000E+00
Olfr1084	25774		#N/A	1.000000E+00
Olfr1085	25775		#N/A	1.000000E+00
Olfr1086	25776		#N/A	1.000000E+00
Olfr1087	25777		#N/A	1.000000E+00
Olfr1089	25778		#N/A	1.000000E+00
Olfr109	25779		#N/A	1.000000E+00
Olfr1090	25780		#N/A	1.000000E+00
Olfr1093	25781		#N/A	1.000000E+00
Olfr1094	25782		#N/A	1.000000E+00
Olfr1095	25783		#N/A	1.000000E+00
Olfr1096-ps1	25784		#N/A	1.000000E+00
Olfr1097	25785		#N/A	1.000000E+00
Olfr1098	25786		#N/A	1.000000E+00
Olfr1099	25787		#N/A	1.000000E+00
Olfr11	25788		#N/A	1.000000E+00
Olfr110	25789		#N/A	1.000000E+00
Olfr1100	25790		#N/A	1.000000E+00
Olfr1101	25791		#N/A	1.000000E+00
Olfr1102	25792		#N/A	1.000000E+00
Olfr1103-ps1	25793		#N/A	1.000000E+00
Olfr1104	25794		#N/A	1.000000E+00
Olfr1105	25795		#N/A	1.000000E+00
Olfr1106	25796		#N/A	1.000000E+00
Olfr1107	25797		#N/A	1.000000E+00
Olfr1109	25798		#N/A	1.000000E+00
Olfr111	25799		#N/A	1.000000E+00

Spearman Rank correlation analysis performed between Prdm1 and all-expressed genes within the Meredith RNA-seq dataset. Robust Prdm1–associated genes were identified using a cut-off of $p < 0.0005$.

Table S1, Related to Supplemental Figure 3C. Prdm1 associated genes

Olfr1110	25800		#N/A	1.000000E+00
Olfr1111	25801		#N/A	1.000000E+00
Olfr1112	25802		#N/A	1.000000E+00
Olfr1113	25803		#N/A	1.000000E+00
Olfr1115	25804		#N/A	1.000000E+00
Olfr1116-ps	25805		#N/A	1.000000E+00
Olfr1117-ps1	25806		#N/A	1.000000E+00
Olfr1118	25807		#N/A	1.000000E+00
Olfr112	25808	0.053805701	8820	4.492258E-01
Olfr1120	25809		#N/A	1.000000E+00
Olfr1121	25810		#N/A	1.000000E+00
Olfr1122	25811		#N/A	1.000000E+00
Olfr1123	25812		#N/A	1.000000E+00
Olfr1124	25813		#N/A	1.000000E+00
Olfr1126	25814		#N/A	1.000000E+00
Olfr1127-ps1	25815		#N/A	1.000000E+00
Olfr1128	25816		#N/A	1.000000E+00
Olfr1129	25817		#N/A	1.000000E+00
Olfr113	25818		#N/A	1.000000E+00
Olfr1130	25819		#N/A	1.000000E+00
Olfr1131	25820		#N/A	1.000000E+00
Olfr1132	25821		#N/A	1.000000E+00
Olfr1133	25822		#N/A	1.000000E+00
Olfr1134	25823		#N/A	1.000000E+00
Olfr1135	25824		#N/A	1.000000E+00
Olfr1136	25825	0.110532946	12761	1.191972E-01
Olfr1137	25826		#N/A	1.000000E+00
Olfr1138	25827		#N/A	1.000000E+00
Olfr114	25828		#N/A	1.000000E+00
Olfr1140	25829		#N/A	1.000000E+00
Olfr1141	25830		#N/A	1.000000E+00
Olfr1143	25831		#N/A	1.000000E+00
Olfr1144-ps1	25832		#N/A	1.000000E+00
Olfr1145	25833		#N/A	1.000000E+00
Olfr1146-ps1	25834		#N/A	1.000000E+00
Olfr1147-ps1	25835		#N/A	1.000000E+00
Olfr1148	25836		#N/A	1.000000E+00
Olfr1149-ps1	25837		#N/A	1.000000E+00
Olfr115	25838		#N/A	1.000000E+00
Olfr1150-ps1	25839		#N/A	1.000000E+00
Olfr1151	25840		#N/A	1.000000E+00
Olfr1152	25841		#N/A	1.000000E+00
Olfr1153	25842		#N/A	1.000000E+00

Spearman Rank correlation analysis performed between Prdm1 and all-expressed genes within the Meredith RNA-seq dataset. Robust Prdm1–associated genes were identified using a cut-off of $p < 0.0005$.

Table S1, Related to Supplemental Figure 3C. Prdm1 associated genes

Olfr1154	25843		#N/A	1.000000E+00
Olfr1155	25844		#N/A	1.000000E+00
Olfr1156	25845		#N/A	1.000000E+00
Olfr1157	25846		#N/A	1.000000E+00
Olfr1158	25847		#N/A	1.000000E+00
Olfr1159-ps1	25848		#N/A	1.000000E+00
Olfr116	25849		#N/A	1.000000E+00
Olfr1160	25850		#N/A	1.000000E+00
Olfr1161	25851		#N/A	1.000000E+00
Olfr1162	25852		#N/A	1.000000E+00
Olfr1163	25853		#N/A	1.000000E+00
Olfr1164	25854		#N/A	1.000000E+00
Olfr1165-ps	25855		#N/A	1.000000E+00
Olfr1166	25856	-0.038406171	3065	5.892384E-01
Olfr1167	25857		#N/A	1.000000E+00
Olfr1168	25858		#N/A	1.000000E+00
Olfr1169-ps1	25859		#N/A	1.000000E+00
Olfr117	25860		#N/A	1.000000E+00
Olfr1170	25861		#N/A	1.000000E+00
Olfr1171-ps1	25862		#N/A	1.000000E+00
Olfr1172-ps1	25863		#N/A	1.000000E+00
Olfr1173	25864		#N/A	1.000000E+00
Olfr1174-ps	25865		#N/A	1.000000E+00
Olfr1175-ps	25866		#N/A	1.000000E+00
Olfr1176	25867		#N/A	1.000000E+00
Olfr1177-ps	25868	-0.038406171	3065	5.892384E-01
Olfr1178	25869		#N/A	1.000000E+00
Olfr1179	25870		#N/A	1.000000E+00
Olfr118	25871		#N/A	1.000000E+00
Olfr1180	25872		#N/A	1.000000E+00
Olfr1181	25873		#N/A	1.000000E+00
Olfr1182	25874		#N/A	1.000000E+00
Olfr1183	25875		#N/A	1.000000E+00
Olfr1184	25876		#N/A	1.000000E+00
Olfr1185-ps1	25877		#N/A	1.000000E+00
Olfr1186	25878		#N/A	1.000000E+00
Olfr1187-ps1	25879		#N/A	1.000000E+00
Olfr1188	25880		#N/A	1.000000E+00
Olfr1189	25881		#N/A	1.000000E+00
Olfr119	25882		#N/A	1.000000E+00
Olfr1191-ps1	25883		#N/A	1.000000E+00
Olfr1192-ps1	25884		#N/A	1.000000E+00
Olfr1193	25885		#N/A	1.000000E+00

Spearman Rank correlation analysis performed between Prdm1 and all-expressed genes within the Meredith RNA-seq dataset. Robust Prdm1–associated genes were identified using a cut-off of $p < 0.0005$.

Table S1, Related to Supplemental Figure 3C. Prdm1 associated genes

Olfr1195	25886		#N/A	1.000000E+00
Olfr1196	25887		#N/A	1.000000E+00
Olfr1197	25888		#N/A	1.000000E+00
Olfr1198	25889		#N/A	1.000000E+00
Olfr1199	25890		#N/A	1.000000E+00
Olfr12	25891		#N/A	1.000000E+00
Olfr120	25892		#N/A	1.000000E+00
Olfr1200	25893		#N/A	1.000000E+00
Olfr1201	25894		#N/A	1.000000E+00
Olfr1202	25895		#N/A	1.000000E+00
Olfr1204	25896		#N/A	1.000000E+00
Olfr1205	25897		#N/A	1.000000E+00
Olfr1206	25898		#N/A	1.000000E+00
Olfr1208	25899		#N/A	1.000000E+00
Olfr1209	25900		#N/A	1.000000E+00
Olfr121	25901	0.162808768	16526.5	2.125512E-02
Olfr1211	25902		#N/A	1.000000E+00
Olfr1212	25903		#N/A	1.000000E+00
Olfr1213	25904	0.053267784	8685	4.537786E-01
Olfr1214	25905		#N/A	1.000000E+00
Olfr1215	25906		#N/A	1.000000E+00
Olfr1216	25907		#N/A	1.000000E+00
Olfr1217	25908		#N/A	1.000000E+00
Olfr1218	25909		#N/A	1.000000E+00
Olfr1219	25910		#N/A	1.000000E+00
Olfr122	25911	-0.054451513	1703	4.437940E-01
Olfr1220	25912	-0.038406171	3065	5.892384E-01
Olfr1221	25913		#N/A	1.000000E+00
Olfr1222	25914		#N/A	1.000000E+00
Olfr1223	25915		#N/A	1.000000E+00
Olfr1224-ps1	25916		#N/A	1.000000E+00
Olfr1225	25917		#N/A	1.000000E+00
Olfr1226	25918		#N/A	1.000000E+00
Olfr1228	25919		#N/A	1.000000E+00
Olfr1229	25920		#N/A	1.000000E+00
Olfr123	25921		#N/A	1.000000E+00
Olfr1230	25922		#N/A	1.000000E+00
Olfr1231	25923		#N/A	1.000000E+00
Olfr1232	25924		#N/A	1.000000E+00
Olfr1233	25925		#N/A	1.000000E+00
Olfr1234	25926		#N/A	1.000000E+00
Olfr1235-ps1	25927		#N/A	1.000000E+00
Olfr1238	25928		#N/A	1.000000E+00

Spearman Rank correlation analysis performed between Prdm1 and all-expressed genes within the Meredith RNA-seq dataset. Robust Prdm1–associated genes were identified using a cut-off of $p < 0.0005$.

Table S1, Related to Supplemental Figure 3C. Prdm1 associated genes

Olfr1239	25929		#N/A	1.000000E+00
Olfr124	25930		#N/A	1.000000E+00
Olfr1240	25931		#N/A	1.000000E+00
Olfr1241	25932		#N/A	1.000000E+00
Olfr1242	25933	-0.054450825	1984	4.437997E-01
Olfr1243	25934	-0.038406171	3065	5.892384E-01
Olfr1245	25935		#N/A	1.000000E+00
Olfr1246	25936	0.143605682	15224	4.249002E-02
Olfr1247	25937		#N/A	1.000000E+00
Olfr1248	25938		#N/A	1.000000E+00
Olfr1249	25939		#N/A	1.000000E+00
Olfr125	25940		#N/A	1.000000E+00
Olfr1250	25941		#N/A	1.000000E+00
Olfr1251	25942		#N/A	1.000000E+00
Olfr1252	25943		#N/A	1.000000E+00
Olfr1253	25944		#N/A	1.000000E+00
Olfr1254	25945		#N/A	1.000000E+00
Olfr1255	25946		#N/A	1.000000E+00
Olfr1256	25947	-0.038406171	3065	5.892384E-01
Olfr1257	25948	-0.038406171	3065	5.892384E-01
Olfr1258	25949		#N/A	1.000000E+00
Olfr1259	25950		#N/A	1.000000E+00
Olfr126	25951		#N/A	1.000000E+00
Olfr1260	25952		#N/A	1.000000E+00
Olfr1261	25953		#N/A	1.000000E+00
Olfr1262	25954		#N/A	1.000000E+00
Olfr1263	25955		#N/A	1.000000E+00
Olfr1264	25956		#N/A	1.000000E+00
Olfr1265	25957		#N/A	1.000000E+00
Olfr1267-ps1	25958		#N/A	1.000000E+00
Olfr1268-ps1	25959		#N/A	1.000000E+00
Olfr1269	25960		#N/A	1.000000E+00
Olfr127	25961		#N/A	1.000000E+00
Olfr1270	25962		#N/A	1.000000E+00
Olfr1271	25963		#N/A	1.000000E+00
Olfr1272	25964		#N/A	1.000000E+00
Olfr1273-ps	25965		#N/A	1.000000E+00
Olfr1274-ps	25966		#N/A	1.000000E+00
Olfr1275	25967		#N/A	1.000000E+00
Olfr1276	25968		#N/A	1.000000E+00
Olfr1277	25969		#N/A	1.000000E+00
Olfr1278	25970	0.07521908	10268	2.897860E-01
Olfr1279	25971		#N/A	1.000000E+00

Spearman Rank correlation analysis performed between Prdm1 and all-expressed genes within the Meredith RNA-seq dataset. Robust Prdm1–associated genes were identified using a cut-off of $p < 0.0005$.

Table S1, Related to Supplemental Figure 3C. Prdm1 associated genes

Olfr128	25972		#N/A	1.000000E+00
Olfr1280	25973		#N/A	1.000000E+00
Olfr1281	25974		#N/A	1.000000E+00
Olfr1282	25975		#N/A	1.000000E+00
Olfr1283	25976		#N/A	1.000000E+00
Olfr1284	25977		#N/A	1.000000E+00
Olfr1285	25978		#N/A	1.000000E+00
Olfr1286	25979		#N/A	1.000000E+00
Olfr1287	25980		#N/A	1.000000E+00
Olfr1288	25981		#N/A	1.000000E+00
Olfr1289	25982		#N/A	1.000000E+00
Olfr129	25983		#N/A	1.000000E+00
Olfr1290	25984		#N/A	1.000000E+00
Olfr1291-ps1	25985		#N/A	1.000000E+00
Olfr1292-ps1	25986		#N/A	1.000000E+00
Olfr1293-ps	25987		#N/A	1.000000E+00
Olfr1294	25988		#N/A	1.000000E+00
Olfr1295	25989		#N/A	1.000000E+00
Olfr1296-ps1	25990		#N/A	1.000000E+00
Olfr1297	25991		#N/A	1.000000E+00
Olfr1298	25992		#N/A	1.000000E+00
Olfr1299	25993	0.047870267	8136	5.008643E-01
Olfr13	25994		#N/A	1.000000E+00
Olfr130	25995		#N/A	1.000000E+00
Olfr1300-ps1	25996		#N/A	1.000000E+00
Olfr1301	25997	-0.038406171	3065	5.892384E-01
Olfr1302	25998		#N/A	1.000000E+00
Olfr1303	25999		#N/A	1.000000E+00
Olfr1305	26000		#N/A	1.000000E+00
Olfr1306	26001		#N/A	1.000000E+00
Olfr1307	26002		#N/A	1.000000E+00
Olfr1308	26003		#N/A	1.000000E+00
Olfr1309	26004		#N/A	1.000000E+00
Olfr131	26005	0.152789767	15822	3.077826E-02
Olfr1310	26006		#N/A	1.000000E+00
Olfr1311	26007		#N/A	1.000000E+00
Olfr1312	26008		#N/A	1.000000E+00
Olfr1313	26009		#N/A	1.000000E+00
Olfr1314	26010		#N/A	1.000000E+00
Olfr1315-ps1	26011		#N/A	1.000000E+00
Olfr1316	26012		#N/A	1.000000E+00
Olfr1317	26013		#N/A	1.000000E+00
Olfr1318	26014	-0.038406171	3065	5.892384E-01

Spearman Rank correlation analysis performed between Prdm1 and all-expressed genes within the Meredith RNA-seq dataset. Robust Prdm1–associated genes were identified using a cut-off of $p < 0.0005$.

Table S1, Related to Supplemental Figure 3C. Prdm1 associated genes

Olfr132	26015		#N/A	1.000000E+00
Olfr1320	26016		#N/A	1.000000E+00
Olfr1321	26017		#N/A	1.000000E+00
Olfr1322	26018		#N/A	1.000000E+00
Olfr1323	26019		#N/A	1.000000E+00
Olfr1324	26020		#N/A	1.000000E+00
Olfr1325	26021		#N/A	1.000000E+00
Olfr1326-ps1	26022		#N/A	1.000000E+00
Olfr1327-ps1	26023		#N/A	1.000000E+00
Olfr1328	26024		#N/A	1.000000E+00
Olfr1329	26025		#N/A	1.000000E+00
Olfr133	26026		#N/A	1.000000E+00
Olfr1330	26027		#N/A	1.000000E+00
Olfr1331	26028	-0.038406171	3065	5.892384E-01
Olfr1332-ps1	26029		#N/A	1.000000E+00
Olfr1333	26030		#N/A	1.000000E+00
Olfr1335	26031		#N/A	1.000000E+00
Olfr1336	26032		#N/A	1.000000E+00
Olfr1337	26033		#N/A	1.000000E+00
Olfr1338	26034		#N/A	1.000000E+00
Olfr1339	26035		#N/A	1.000000E+00
Olfr134	26036		#N/A	1.000000E+00
Olfr1340	26037		#N/A	1.000000E+00
Olfr1341	26038		#N/A	1.000000E+00
Olfr1342	26039	-0.054451513	1703	4.437940E-01
Olfr1343-ps1	26040		#N/A	1.000000E+00
Olfr1344	26041		#N/A	1.000000E+00
Olfr1346	26042		#N/A	1.000000E+00
Olfr1347	26043		#N/A	1.000000E+00
Olfr1348	26044		#N/A	1.000000E+00
Olfr1349	26045		#N/A	1.000000E+00
Olfr135	26046		#N/A	1.000000E+00
Olfr1350	26047		#N/A	1.000000E+00
Olfr1351	26048		#N/A	1.000000E+00
Olfr1352	26049		#N/A	1.000000E+00
Olfr1353	26050		#N/A	1.000000E+00
Olfr1354	26051		#N/A	1.000000E+00
Olfr1355	26052		#N/A	1.000000E+00
Olfr1356	26053		#N/A	1.000000E+00
Olfr1357	26054		#N/A	1.000000E+00
Olfr1359	26055		#N/A	1.000000E+00
Olfr136	26056		#N/A	1.000000E+00
Olfr1360	26057		#N/A	1.000000E+00

Spearman Rank correlation analysis performed between Prdm1 and all-expressed genes within the Meredith RNA-seq dataset. Robust Prdm1–associated genes were identified using a cut-off of $p < 0.0005$.

Table S1, Related to Supplemental Figure 3C. Prdm1 associated genes

Olfr1361	26058		#N/A	1.000000E+00
Olfr1362	26059		#N/A	1.000000E+00
Olfr1364	26060		#N/A	1.000000E+00
Olfr1366	26061		#N/A	1.000000E+00
Olfr1367	26062		#N/A	1.000000E+00
Olfr1368	26063		#N/A	1.000000E+00
Olfr1369-ps1	26064		#N/A	1.000000E+00
Olfr137	26065		#N/A	1.000000E+00
Olfr1370	26066	-0.054450825	1984	4.437997E-01
Olfr1371	26067		#N/A	1.000000E+00
Olfr1372-ps1	26068		#N/A	1.000000E+00
Olfr1373	26069		#N/A	1.000000E+00
Olfr1374-ps1	26070		#N/A	1.000000E+00
Olfr1375-ps1	26071		#N/A	1.000000E+00
Olfr1376-ps1	26072		#N/A	1.000000E+00
Olfr1377	26073		#N/A	1.000000E+00
Olfr1378	26074		#N/A	1.000000E+00
Olfr1379-ps1	26075		#N/A	1.000000E+00
Olfr138	26076	-0.038406171	3065	5.892384E-01
Olfr1380	26077		#N/A	1.000000E+00
Olfr1381	26078		#N/A	1.000000E+00
Olfr1382	26079		#N/A	1.000000E+00
Olfr1383	26080		#N/A	1.000000E+00
Olfr1384	26081		#N/A	1.000000E+00
Olfr1385	26082		#N/A	1.000000E+00
Olfr1386	26083		#N/A	1.000000E+00
Olfr1387	26084		#N/A	1.000000E+00
Olfr1388	26085		#N/A	1.000000E+00
Olfr1389	26086		#N/A	1.000000E+00
Olfr139	26087		#N/A	1.000000E+00
Olfr1390	26088		#N/A	1.000000E+00
Olfr1391	26089		#N/A	1.000000E+00
Olfr1392	26090		#N/A	1.000000E+00
Olfr1393	26091		#N/A	1.000000E+00
Olfr1394	26092		#N/A	1.000000E+00
Olfr1395	26093		#N/A	1.000000E+00
Olfr1396	26094		#N/A	1.000000E+00
Olfr1397-ps1	26095		#N/A	1.000000E+00
Olfr140	26096		#N/A	1.000000E+00
Olfr1402	26097		#N/A	1.000000E+00
Olfr1404	26098		#N/A	1.000000E+00
Olfr1406	26099		#N/A	1.000000E+00
Olfr1407-ps1	26100		#N/A	1.000000E+00

Spearman Rank correlation analysis performed between Prdm1 and all-expressed genes within the Meredith RNA-seq dataset. Robust Prdm1–associated genes were identified using a cut-off of $p < 0.0005$.

Table S1, Related to Supplemental Figure 3C. Prdm1 associated genes

Olfr1408	26101		#N/A	1.000000E+00
Olfr141	26102		#N/A	1.000000E+00
Olfr1410	26103		#N/A	1.000000E+00
Olfr1411	26104		#N/A	1.000000E+00
Olfr1412	26105		#N/A	1.000000E+00
Olfr1413	26106		#N/A	1.000000E+00
Olfr1414	26107		#N/A	1.000000E+00
Olfr1415	26108		#N/A	1.000000E+00
Olfr1416	26109		#N/A	1.000000E+00
Olfr1417	26110		#N/A	1.000000E+00
Olfr1418	26111		#N/A	1.000000E+00
Olfr1419	26112		#N/A	1.000000E+00
Olfr142	26113		#N/A	1.000000E+00
Olfr1420	26114		#N/A	1.000000E+00
Olfr1423	26115		#N/A	1.000000E+00
Olfr1424	26116		#N/A	1.000000E+00
Olfr1425	26117		#N/A	1.000000E+00
Olfr1426	26118		#N/A	1.000000E+00
Olfr1427	26119		#N/A	1.000000E+00
Olfr1428	26120		#N/A	1.000000E+00
Olfr143	26121		#N/A	1.000000E+00
Olfr1431	26122		#N/A	1.000000E+00
Olfr1432	26123		#N/A	1.000000E+00
Olfr1433	26124		#N/A	1.000000E+00
Olfr1436	26125		#N/A	1.000000E+00
Olfr1437	26126		#N/A	1.000000E+00
Olfr1440	26127		#N/A	1.000000E+00
Olfr1441	26128		#N/A	1.000000E+00
Olfr1442	26129		#N/A	1.000000E+00
Olfr1443	26130	0.007505821	5656	9.159914E-01
Olfr1444	26131		#N/A	1.000000E+00
Olfr1445	26132		#N/A	1.000000E+00
Olfr1446	26133		#N/A	1.000000E+00
Olfr1447	26134		#N/A	1.000000E+00
Olfr1448	26135		#N/A	1.000000E+00
Olfr1449	26136		#N/A	1.000000E+00
Olfr145	26137		#N/A	1.000000E+00
Olfr1450	26138		#N/A	1.000000E+00
Olfr1451	26139		#N/A	1.000000E+00
Olfr1452-ps1	26140		#N/A	1.000000E+00
Olfr1453	26141		#N/A	1.000000E+00
Olfr1454	26142		#N/A	1.000000E+00
Olfr1456-ps1	26143		#N/A	1.000000E+00

Spearman Rank correlation analysis performed between Prdm1 and all-expressed genes within the Meredith RNA-seq dataset. Robust Prdm1–associated genes were identified using a cut-off of $p < 0.0005$.

Table S1, Related to Supplemental Figure 3C. Prdm1 associated genes

Olfr1457	26144		#N/A	1.000000E+00
Olfr1458	26145		#N/A	1.000000E+00
Olfr1459	26146		#N/A	1.000000E+00
Olfr146	26147	0.113548679	13094	1.093908E-01
Olfr1461	26148		#N/A	1.000000E+00
Olfr1462	26149		#N/A	1.000000E+00
Olfr1463	26150		#N/A	1.000000E+00
Olfr1465	26151		#N/A	1.000000E+00
Olfr1466	26152		#N/A	1.000000E+00
Olfr1467	26153		#N/A	1.000000E+00
Olfr1469	26154		#N/A	1.000000E+00
Olfr147	26155		#N/A	1.000000E+00
Olfr1471	26156		#N/A	1.000000E+00
Olfr1472	26157		#N/A	1.000000E+00
Olfr1474	26158		#N/A	1.000000E+00
Olfr1475	26159		#N/A	1.000000E+00
Olfr1477	26160		#N/A	1.000000E+00
Olfr148	26161		#N/A	1.000000E+00
Olfr1480	26162		#N/A	1.000000E+00
Olfr1484	26163		#N/A	1.000000E+00
Olfr1487	26164		#N/A	1.000000E+00
Olfr1489	26165		#N/A	1.000000E+00
Olfr149	26166		#N/A	1.000000E+00
Olfr1490	26167		#N/A	1.000000E+00
Olfr1491	26168		#N/A	1.000000E+00
Olfr1494	26169		#N/A	1.000000E+00
Olfr1495	26170		#N/A	1.000000E+00
Olfr1496	26171		#N/A	1.000000E+00
Olfr1497	26172	0.160304018	16323	2.335876E-02
Olfr1499	26173		#N/A	1.000000E+00
Olfr15	26174		#N/A	1.000000E+00
Olfr150	26175		#N/A	1.000000E+00
Olfr1500	26176		#N/A	1.000000E+00
Olfr1501	26177	-0.086746694	532	2.219355E-01
Olfr1502	26178		#N/A	1.000000E+00
Olfr1503-ps1	26179		#N/A	1.000000E+00
Olfr1504	26180	-0.07739401	763.5	2.760257E-01
Olfr1505	26181		#N/A	1.000000E+00
Olfr1506	26182		#N/A	1.000000E+00
Olfr1507	26183		#N/A	1.000000E+00
Olfr1508	26184		#N/A	1.000000E+00
Olfr1509	26185		#N/A	1.000000E+00
Olfr1510	26186		#N/A	1.000000E+00

Spearman Rank correlation analysis performed between Prdm1 and all-expressed genes within the Meredith RNA-seq dataset. Robust Prdm1–associated genes were identified using a cut-off of $p < 0.0005$.

Table S1, Related to Supplemental Figure 3C. Prdm1 associated genes

Olfr1511	26187		#N/A	1.000000E+00
Olfr1512	26188		#N/A	1.000000E+00
Olfr1513	26189		#N/A	1.000000E+00
Olfr152	26190		#N/A	1.000000E+00
Olfr153	26191		#N/A	1.000000E+00
Olfr1532-ps1	26192		#N/A	1.000000E+00
Olfr1535	26193	0.143605682	15224	4.249002E-02
Olfr1537	26194		#N/A	1.000000E+00
Olfr154	26195		#N/A	1.000000E+00
Olfr155	26196		#N/A	1.000000E+00
Olfr156	26197	-0.002881499	5034.5	9.676983E-01
Olfr157	26198	0.063982328	9467.5	3.680682E-01
Olfr159	26199		#N/A	1.000000E+00
Olfr16	26200		#N/A	1.000000E+00
Olfr160	26201		#N/A	1.000000E+00
Olfr161	26202		#N/A	1.000000E+00
Olfr164	26203		#N/A	1.000000E+00
Olfr165	26204		#N/A	1.000000E+00
Olfr166	26205		#N/A	1.000000E+00
Olfr167	26206		#N/A	1.000000E+00
Olfr168	26207		#N/A	1.000000E+00
Olfr169	26208		#N/A	1.000000E+00
Olfr17	26209		#N/A	1.000000E+00
Olfr170	26210		#N/A	1.000000E+00
Olfr171	26211		#N/A	1.000000E+00
Olfr172	26212		#N/A	1.000000E+00
Olfr173	26213		#N/A	1.000000E+00
Olfr175-ps1	26214	-0.066858259	1029.5	3.468868E-01
Olfr177	26215		#N/A	1.000000E+00
Olfr178	26216		#N/A	1.000000E+00
Olfr179	26217		#N/A	1.000000E+00
Olfr18	26218	0.091279115	11463.5	1.986261E-01
Olfr180	26219		#N/A	1.000000E+00
Olfr181	26220		#N/A	1.000000E+00
Olfr183	26221	-0.054451513	1703	4.437940E-01
Olfr186	26222		#N/A	1.000000E+00
Olfr187	26223		#N/A	1.000000E+00
Olfr19	26224		#N/A	1.000000E+00
Olfr190	26225		#N/A	1.000000E+00
Olfr191	26226		#N/A	1.000000E+00
Olfr192	26227		#N/A	1.000000E+00
Olfr193	26228		#N/A	1.000000E+00
Olfr194	26229		#N/A	1.000000E+00

Spearman Rank correlation analysis performed between Prdm1 and all-expressed genes within the Meredith RNA-seq dataset. Robust Prdm1–associated genes were identified using a cut-off of $p < 0.0005$.

Table S1, Related to Supplemental Figure 3C. Prdm1 associated genes

Olfr195	26230		#N/A	1.000000E+00
Olfr196	26231		#N/A	1.000000E+00
Olfr197	26232		#N/A	1.000000E+00
Olfr198	26233		#N/A	1.000000E+00
Olfr199	26234		#N/A	1.000000E+00
Olfr2	26235		#N/A	1.000000E+00
Olfr20	26236		#N/A	1.000000E+00
Olfr201	26237		#N/A	1.000000E+00
Olfr202	26238		#N/A	1.000000E+00
Olfr203	26239		#N/A	1.000000E+00
Olfr204	26240		#N/A	1.000000E+00
Olfr205	26241		#N/A	1.000000E+00
Olfr206	26242		#N/A	1.000000E+00
Olfr209	26243		#N/A	1.000000E+00
Olfr211	26244	-0.038406171	3065	5.892384E-01
Olfr212	26245	-0.001606767	5097.5	9.819847E-01
Olfr213	26246		#N/A	1.000000E+00
Olfr214	26247		#N/A	1.000000E+00
Olfr215	26248		#N/A	1.000000E+00
Olfr218	26249		#N/A	1.000000E+00
Olfr22-ps1	26250		#N/A	1.000000E+00
Olfr220	26251		#N/A	1.000000E+00
Olfr221	26252		#N/A	1.000000E+00
Olfr222	26253		#N/A	1.000000E+00
Olfr223	26254		#N/A	1.000000E+00
Olfr224	26255		#N/A	1.000000E+00
Olfr225	26256		#N/A	1.000000E+00
Olfr228	26257		#N/A	1.000000E+00
Olfr229	26258		#N/A	1.000000E+00
Olfr23	26259		#N/A	1.000000E+00
Olfr231	26260		#N/A	1.000000E+00
Olfr235	26261		#N/A	1.000000E+00
Olfr237-ps1	26262		#N/A	1.000000E+00
Olfr239	26263		#N/A	1.000000E+00
Olfr24	26264		#N/A	1.000000E+00
Olfr243	26265		#N/A	1.000000E+00
Olfr247	26266		#N/A	1.000000E+00
Olfr248	26267		#N/A	1.000000E+00
Olfr25	26268		#N/A	1.000000E+00
Olfr250	26269		#N/A	1.000000E+00
Olfr251	26270		#N/A	1.000000E+00
Olfr259	26271		#N/A	1.000000E+00
Olfr26	26272		#N/A	1.000000E+00

Spearman Rank correlation analysis performed between Prdm1 and all-expressed genes within the Meredith RNA-seq dataset. Robust Prdm1–associated genes were identified using a cut-off of $p < 0.0005$.

Table S1, Related to Supplemental Figure 3C. Prdm1 associated genes

Olfr260-ps1	26273		#N/A	1.000000E+00
Olfr262	26274		#N/A	1.000000E+00
Olfr263	26275		#N/A	1.000000E+00
Olfr266	26276		#N/A	1.000000E+00
Olfr267	26277		#N/A	1.000000E+00
Olfr268-ps1	26278		#N/A	1.000000E+00
Olfr269-ps1	26279		#N/A	1.000000E+00
Olfr27	26280	-0.054450825	1984	4.437997E-01
Olfr270	26281		#N/A	1.000000E+00
Olfr271-ps1	26282		#N/A	1.000000E+00
Olfr272	26283	-0.038406171	3065	5.892384E-01
Olfr273	26284		#N/A	1.000000E+00
Olfr275	26285		#N/A	1.000000E+00
Olfr279	26286		#N/A	1.000000E+00
Olfr281	26287		#N/A	1.000000E+00
Olfr282	26288		#N/A	1.000000E+00
Olfr283	26289		#N/A	1.000000E+00
Olfr284	26290		#N/A	1.000000E+00
Olfr285	26291		#N/A	1.000000E+00
Olfr286	26292		#N/A	1.000000E+00
Olfr287	26293	-0.000359426	5269	9.959698E-01
Olfr288	26294		#N/A	1.000000E+00
Olfr29-ps1	26295		#N/A	1.000000E+00
Olfr290	26296		#N/A	1.000000E+00
Olfr291	26297		#N/A	1.000000E+00
Olfr292	26298	-0.054451513	1703	4.437940E-01
Olfr293	26299		#N/A	1.000000E+00
Olfr294	26300		#N/A	1.000000E+00
Olfr295	26301		#N/A	1.000000E+00
Olfr296-ps1	26302		#N/A	1.000000E+00
Olfr297	26303		#N/A	1.000000E+00
Olfr298	26304		#N/A	1.000000E+00
Olfr299	26305		#N/A	1.000000E+00
Olfr3	26306		#N/A	1.000000E+00
Olfr30	26307		#N/A	1.000000E+00
Olfr300-ps1	26308		#N/A	1.000000E+00
Olfr301	26309	0.013842768	5982.5	8.457464E-01
Olfr303	26310		#N/A	1.000000E+00
Olfr304	26311		#N/A	1.000000E+00
Olfr305	26312		#N/A	1.000000E+00
Olfr306-ps1	26313		#N/A	1.000000E+00
Olfr307	26314		#N/A	1.000000E+00
Olfr308	26315		#N/A	1.000000E+00

Spearman Rank correlation analysis performed between Prdm1 and all-expressed genes within the Meredith RNA-seq dataset. Robust Prdm1–associated genes were identified using a cut-off of $p < 0.0005$.

Table S1, Related to Supplemental Figure 3C. Prdm1 associated genes

Olfr309	26316		#N/A	1.000000E+00
Olfr31	26317		#N/A	1.000000E+00
Olfr310	26318		#N/A	1.000000E+00
Olfr311	26319		#N/A	1.000000E+00
Olfr312	26320		#N/A	1.000000E+00
Olfr313	26321		#N/A	1.000000E+00
Olfr314	26322		#N/A	1.000000E+00
Olfr315	26323		#N/A	1.000000E+00
Olfr316	26324		#N/A	1.000000E+00
Olfr317	26325		#N/A	1.000000E+00
Olfr318	26326		#N/A	1.000000E+00
Olfr319	26327		#N/A	1.000000E+00
Olfr32	26328		#N/A	1.000000E+00
Olfr320	26329		#N/A	1.000000E+00
Olfr323	26330		#N/A	1.000000E+00
Olfr324	26331		#N/A	1.000000E+00
Olfr325	26332		#N/A	1.000000E+00
Olfr328	26333		#N/A	1.000000E+00
Olfr329-ps	26334		#N/A	1.000000E+00
Olfr33	26335		#N/A	1.000000E+00
Olfr330	26336	0.144375081	15329	4.138294E-02
Olfr331	26337		#N/A	1.000000E+00
Olfr332	26338		#N/A	1.000000E+00
Olfr333-ps1	26339		#N/A	1.000000E+00
Olfr334-ps1	26340		#N/A	1.000000E+00
Olfr335-ps	26341		#N/A	1.000000E+00
Olfr336-ps1	26342		#N/A	1.000000E+00
Olfr337-ps1	26343		#N/A	1.000000E+00
Olfr338	26344		#N/A	1.000000E+00
Olfr339	26345		#N/A	1.000000E+00
Olfr340	26346		#N/A	1.000000E+00
Olfr341	26347		#N/A	1.000000E+00
Olfr342	26348		#N/A	1.000000E+00
Olfr343-ps1	26349		#N/A	1.000000E+00
Olfr344	26350		#N/A	1.000000E+00
Olfr345	26351		#N/A	1.000000E+00
Olfr346	26352		#N/A	1.000000E+00
Olfr347	26353		#N/A	1.000000E+00
Olfr348	26354		#N/A	1.000000E+00
Olfr349-ps1	26355		#N/A	1.000000E+00
Olfr350	26356		#N/A	1.000000E+00
Olfr351	26357		#N/A	1.000000E+00
Olfr352	26358		#N/A	1.000000E+00

Spearman Rank correlation analysis performed between Prdm1 and all-expressed genes within the Meredith RNA-seq dataset. Robust Prdm1–associated genes were identified using a cut-off of $p < 0.0005$.

Table S1, Related to Supplemental Figure 3C. Prdm1 associated genes

Olfr353	26359		#N/A	1.000000E+00
Olfr354	26360		#N/A	1.000000E+00
Olfr355	26361		#N/A	1.000000E+00
Olfr356	26362		#N/A	1.000000E+00
Olfr357	26363		#N/A	1.000000E+00
Olfr358	26364		#N/A	1.000000E+00
Olfr359-ps1	26365		#N/A	1.000000E+00
Olfr360	26366		#N/A	1.000000E+00
Olfr361	26367		#N/A	1.000000E+00
Olfr362	26368		#N/A	1.000000E+00
Olfr363-ps	26369		#N/A	1.000000E+00
Olfr364-ps1	26370		#N/A	1.000000E+00
Olfr365	26371		#N/A	1.000000E+00
Olfr366	26372		#N/A	1.000000E+00
Olfr367-ps	26373		#N/A	1.000000E+00
Olfr368	26374		#N/A	1.000000E+00
Olfr370	26375		#N/A	1.000000E+00
Olfr371	26376		#N/A	1.000000E+00
Olfr372	26377		#N/A	1.000000E+00
Olfr373	26378		#N/A	1.000000E+00
Olfr374	26379	-0.038406171	3065	5.892384E-01
Olfr376	26380		#N/A	1.000000E+00
Olfr377-ps1	26381		#N/A	1.000000E+00
Olfr378	26382		#N/A	1.000000E+00
Olfr379-ps1	26383		#N/A	1.000000E+00
Olfr38	26384		#N/A	1.000000E+00
Olfr380	26385		#N/A	1.000000E+00
Olfr381	26386		#N/A	1.000000E+00
Olfr382	26387		#N/A	1.000000E+00
Olfr383-ps1	26388		#N/A	1.000000E+00
Olfr384	26389		#N/A	1.000000E+00
Olfr385	26390		#N/A	1.000000E+00
Olfr387-ps1	26391		#N/A	1.000000E+00
Olfr388-ps1	26392		#N/A	1.000000E+00
Olfr389	26393		#N/A	1.000000E+00
Olfr39	26394	0.154685737	15965	2.873847E-02
Olfr390	26395	-0.038406171	3065	5.892384E-01
Olfr391-ps	26396		#N/A	1.000000E+00
Olfr392	26397		#N/A	1.000000E+00
Olfr393	26398		#N/A	1.000000E+00
Olfr394	26399		#N/A	1.000000E+00
Olfr395	26400		#N/A	1.000000E+00
Olfr396-ps1	26401		#N/A	1.000000E+00

Spearman Rank correlation analysis performed between Prdm1 and all-expressed genes within the Meredith RNA-seq dataset. Robust Prdm1–associated genes were identified using a cut-off of $p < 0.0005$.

Table S1, Related to Supplemental Figure 3C. Prdm1 associated genes

Olfr397	26402	#N/A	1.000000E+00
Olfr398	26403	#N/A	1.000000E+00
Olfr399	26404	#N/A	1.000000E+00
Olfr400-ps1	26405	#N/A	1.000000E+00
Olfr401	26406	#N/A	1.000000E+00
Olfr402	26407	#N/A	1.000000E+00
Olfr403	26408	#N/A	1.000000E+00
Olfr404-ps1	26409	#N/A	1.000000E+00
Olfr405-ps1	26410	#N/A	1.000000E+00
Olfr406	26411	#N/A	1.000000E+00
Olfr407-ps1	26412	#N/A	1.000000E+00
Olfr408-ps1	26413	#N/A	1.000000E+00
Olfr409-ps1	26414	#N/A	1.000000E+00
Olfr410	26415	#N/A	1.000000E+00
Olfr411	26416	#N/A	1.000000E+00
Olfr412	26417	#N/A	1.000000E+00
Olfr414	26418	#N/A	1.000000E+00
Olfr417	26419	#N/A	1.000000E+00
Olfr418-ps1	26420	#N/A	1.000000E+00
Olfr419	26421	#N/A	1.000000E+00
Olfr420	26422	#N/A	1.000000E+00
Olfr421-ps1	26423	#N/A	1.000000E+00
Olfr424	26424	#N/A	1.000000E+00
Olfr427	26425	#N/A	1.000000E+00
Olfr429	26426	#N/A	1.000000E+00
Olfr43	26427	#N/A	1.000000E+00
Olfr430	26428	#N/A	1.000000E+00
Olfr432	26429	#N/A	1.000000E+00
Olfr433	26430	#N/A	1.000000E+00
Olfr434	26431	#N/A	1.000000E+00
Olfr435	26432	#N/A	1.000000E+00
Olfr437	26433	#N/A	1.000000E+00
Olfr44	26434	#N/A	1.000000E+00
Olfr441	26435	#N/A	1.000000E+00
Olfr443-ps1	26436	#N/A	1.000000E+00
Olfr444	26437	#N/A	1.000000E+00
Olfr446	26438	#N/A	1.000000E+00
Olfr447	26439	#N/A	1.000000E+00
Olfr448	26440	#N/A	1.000000E+00
Olfr449	26441	#N/A	1.000000E+00
Olfr45	26442	#N/A	1.000000E+00
Olfr450	26443	#N/A	1.000000E+00
Olfr452	26444	#N/A	1.000000E+00

Spearman Rank correlation analysis performed between Prdm1 and all-expressed genes within the Meredith RNA-seq dataset. Robust Prdm1–associated genes were identified using a cut-off of $p < 0.0005$.

Table S1, Related to Supplemental Figure 3C. Prdm1 associated genes

Olfr453	26445	#N/A	1.000000E+00
Olfr455	26446	#N/A	1.000000E+00
Olfr456	26447	#N/A	1.000000E+00
Olfr457	26448	#N/A	1.000000E+00
Olfr458	26449	#N/A	1.000000E+00
Olfr459	26450	#N/A	1.000000E+00
Olfr46	26451	#N/A	1.000000E+00
Olfr460	26452	#N/A	1.000000E+00
Olfr461	26453	#N/A	1.000000E+00
Olfr462	26454	#N/A	1.000000E+00
Olfr463	26455	#N/A	1.000000E+00
Olfr464	26456	#N/A	1.000000E+00
Olfr466	26457	#N/A	1.000000E+00
Olfr467	26458	#N/A	1.000000E+00
Olfr469	26459	#N/A	1.000000E+00
Olfr47	26460	#N/A	1.000000E+00
Olfr470	26461	#N/A	1.000000E+00
Olfr472	26462	#N/A	1.000000E+00
Olfr473	26463	#N/A	1.000000E+00
Olfr474	26464	#N/A	1.000000E+00
Olfr475-ps1	26465	#N/A	1.000000E+00
Olfr476	26466	#N/A	1.000000E+00
Olfr477	26467	#N/A	1.000000E+00
Olfr478	26468	#N/A	1.000000E+00
Olfr479	26469	#N/A	1.000000E+00
Olfr48	26470	#N/A	1.000000E+00
Olfr480	26471	#N/A	1.000000E+00
Olfr481	26472	#N/A	1.000000E+00
Olfr482	26473	#N/A	1.000000E+00
Olfr483	26474	#N/A	1.000000E+00
Olfr484	26475	#N/A	1.000000E+00
Olfr485	26476	#N/A	1.000000E+00
Olfr486	26477	#N/A	1.000000E+00
Olfr487	26478	#N/A	1.000000E+00
Olfr488	26479	#N/A	1.000000E+00
Olfr49	26480	#N/A	1.000000E+00
Olfr490	26481	#N/A	1.000000E+00
Olfr491	26482	#N/A	1.000000E+00
Olfr492	26483	#N/A	1.000000E+00
Olfr493	26484	#N/A	1.000000E+00
Olfr494	26485	#N/A	1.000000E+00
Olfr495	26486	#N/A	1.000000E+00
Olfr497	26487	#N/A	1.000000E+00

Spearman Rank correlation analysis performed between Prdm1 and all-expressed genes within the Meredith RNA-seq dataset. Robust Prdm1–associated genes were identified using a cut-off of $p < 0.0005$.

Table S1, Related to Supplemental Figure 3C. Prdm1 associated genes

Olfr498	26488		#N/A	1.000000E+00
Olfr5	26489		#N/A	1.000000E+00
Olfr50	26490		#N/A	1.000000E+00
Olfr502	26491		#N/A	1.000000E+00
Olfr503	26492		#N/A	1.000000E+00
Olfr504	26493		#N/A	1.000000E+00
Olfr506	26494		#N/A	1.000000E+00
Olfr507	26495		#N/A	1.000000E+00
Olfr508	26496		#N/A	1.000000E+00
Olfr509	26497		#N/A	1.000000E+00
Olfr51	26498		#N/A	1.000000E+00
Olfr510	26499	-0.038406171	3065	5.892384E-01
Olfr512	26500		#N/A	1.000000E+00
Olfr513	26501		#N/A	1.000000E+00
Olfr514	26502		#N/A	1.000000E+00
Olfr516	26503		#N/A	1.000000E+00
Olfr517	26504		#N/A	1.000000E+00
Olfr518	26505		#N/A	1.000000E+00
Olfr519	26506		#N/A	1.000000E+00
Olfr52	26507		#N/A	1.000000E+00
Olfr520	26508		#N/A	1.000000E+00
Olfr521	26509		#N/A	1.000000E+00
Olfr522	26510		#N/A	1.000000E+00
Olfr523	26511		#N/A	1.000000E+00
Olfr524	26512		#N/A	1.000000E+00
Olfr525	26513		#N/A	1.000000E+00
Olfr527	26514		#N/A	1.000000E+00
Olfr53	26515		#N/A	1.000000E+00
Olfr530	26516		#N/A	1.000000E+00
Olfr531	26517		#N/A	1.000000E+00
Olfr532	26518		#N/A	1.000000E+00
Olfr533	26519		#N/A	1.000000E+00
Olfr535	26520		#N/A	1.000000E+00
Olfr536	26521	-0.016731569	4428.5	8.140896E-01
Olfr538	26522		#N/A	1.000000E+00
Olfr539	26523		#N/A	1.000000E+00
Olfr54	26524		#N/A	1.000000E+00
Olfr541	26525		#N/A	1.000000E+00
Olfr543	26526		#N/A	1.000000E+00
Olfr544	26527		#N/A	1.000000E+00
Olfr545	26528		#N/A	1.000000E+00
Olfr547	26529		#N/A	1.000000E+00
Olfr548-ps1	26530		#N/A	1.000000E+00

Spearman Rank correlation analysis performed between Prdm1 and all-expressed genes within the Meredith RNA-seq dataset. Robust Prdm1–associated genes were identified using a cut-off of $p < 0.0005$.

Table S1, Related to Supplemental Figure 3C. Prdm1 associated genes

Olfr549	26531		#N/A	1.000000E+00
Olfr55	26532		#N/A	1.000000E+00
Olfr550	26533		#N/A	1.000000E+00
Olfr551	26534		#N/A	1.000000E+00
Olfr552	26535		#N/A	1.000000E+00
Olfr553	26536		#N/A	1.000000E+00
Olfr554	26537		#N/A	1.000000E+00
Olfr555	26538		#N/A	1.000000E+00
Olfr556	26539		#N/A	1.000000E+00
Olfr557	26540		#N/A	1.000000E+00
Olfr558	26541		#N/A	1.000000E+00
Olfr559	26542		#N/A	1.000000E+00
Olfr56	26543	-0.010998656	4703	8.771570E-01
Olfr561	26544		#N/A	1.000000E+00
Olfr564	26545		#N/A	1.000000E+00
Olfr566	26546		#N/A	1.000000E+00
Olfr568	26547		#N/A	1.000000E+00
Olfr569	26548		#N/A	1.000000E+00
Olfr57	26549		#N/A	1.000000E+00
Olfr570	26550		#N/A	1.000000E+00
Olfr571	26551		#N/A	1.000000E+00
Olfr572	26552		#N/A	1.000000E+00
Olfr573-ps1	26553		#N/A	1.000000E+00
Olfr574	26554		#N/A	1.000000E+00
Olfr575	26555		#N/A	1.000000E+00
Olfr576	26556		#N/A	1.000000E+00
Olfr577	26557		#N/A	1.000000E+00
Olfr578	26558		#N/A	1.000000E+00
Olfr58	26559		#N/A	1.000000E+00
Olfr582	26560		#N/A	1.000000E+00
Olfr583	26561		#N/A	1.000000E+00
Olfr584	26562		#N/A	1.000000E+00
Olfr585	26563		#N/A	1.000000E+00
Olfr586	26564		#N/A	1.000000E+00
Olfr587-ps1	26565		#N/A	1.000000E+00
Olfr588-ps1	26566		#N/A	1.000000E+00
Olfr589	26567		#N/A	1.000000E+00
Olfr59	26568		#N/A	1.000000E+00
Olfr590-ps1	26569		#N/A	1.000000E+00
Olfr591	26570		#N/A	1.000000E+00
Olfr592	26571		#N/A	1.000000E+00
Olfr593	26572		#N/A	1.000000E+00
Olfr594	26573		#N/A	1.000000E+00

Spearman Rank correlation analysis performed between Prdm1 and all-expressed genes within the Meredith RNA-seq dataset. Robust Prdm1–associated genes were identified using a cut-off of $p < 0.0005$.

Table S1, Related to Supplemental Figure 3C. Prdm1 associated genes

Olfr595-ps1	26574		#N/A	1.000000E+00
Olfr596	26575		#N/A	1.000000E+00
Olfr597	26576		#N/A	1.000000E+00
Olfr598	26577		#N/A	1.000000E+00
Olfr599	26578		#N/A	1.000000E+00
Olfr6	26579		#N/A	1.000000E+00
Olfr60	26580		#N/A	1.000000E+00
Olfr600	26581		#N/A	1.000000E+00
Olfr601	26582		#N/A	1.000000E+00
Olfr602-ps1	26583		#N/A	1.000000E+00
Olfr603	26584		#N/A	1.000000E+00
Olfr605	26585		#N/A	1.000000E+00
Olfr606	26586		#N/A	1.000000E+00
Olfr607	26587		#N/A	1.000000E+00
Olfr608	26588		#N/A	1.000000E+00
Olfr609	26589		#N/A	1.000000E+00
Olfr61	26590		#N/A	1.000000E+00
Olfr610	26591		#N/A	1.000000E+00
Olfr611	26592		#N/A	1.000000E+00
Olfr612	26593		#N/A	1.000000E+00
Olfr613	26594	0.01926319	6295	7.865908E-01
Olfr615	26595		#N/A	1.000000E+00
Olfr616	26596		#N/A	1.000000E+00
Olfr617	26597		#N/A	1.000000E+00
Olfr618	26598		#N/A	1.000000E+00
Olfr619	26599		#N/A	1.000000E+00
Olfr62	26600	-0.038406171	3065	5.892384E-01
Olfr620	26601		#N/A	1.000000E+00
Olfr621-ps1	26602		#N/A	1.000000E+00
Olfr622	26603		#N/A	1.000000E+00
Olfr623	26604		#N/A	1.000000E+00
Olfr624	26605		#N/A	1.000000E+00
Olfr625-ps1	26606		#N/A	1.000000E+00
Olfr626-ps1	26607		#N/A	1.000000E+00
Olfr628	26608		#N/A	1.000000E+00
Olfr629	26609	0.157799267	16153.5	2.563935E-02
Olfr63	26610		#N/A	1.000000E+00
Olfr630	26611	0.143605682	15224	4.249002E-02
Olfr631	26612	0.075244915	10282	2.896199E-01
Olfr632	26613		#N/A	1.000000E+00
Olfr633	26614		#N/A	1.000000E+00
Olfr634-ps1	26615		#N/A	1.000000E+00
Olfr635	26616		#N/A	1.000000E+00

Spearman Rank correlation analysis performed between Prdm1 and all-expressed genes within the Meredith RNA-seq dataset. Robust Prdm1–associated genes were identified using a cut-off of $p < 0.0005$.

Table S1, Related to Supplemental Figure 3C. Prdm1 associated genes

Olfr636-ps1	26617		#N/A	1.000000E+00
Olfr637-ps1	26618		#N/A	1.000000E+00
Olfr638	26619		#N/A	1.000000E+00
Olfr639	26620		#N/A	1.000000E+00
Olfr64	26621		#N/A	1.000000E+00
Olfr640	26622		#N/A	1.000000E+00
Olfr641	26623		#N/A	1.000000E+00
Olfr642	26624		#N/A	1.000000E+00
Olfr643	26625		#N/A	1.000000E+00
Olfr644	26626		#N/A	1.000000E+00
Olfr645	26627		#N/A	1.000000E+00
Olfr646	26628		#N/A	1.000000E+00
Olfr647-ps1	26629		#N/A	1.000000E+00
Olfr648	26630		#N/A	1.000000E+00
Olfr649	26631		#N/A	1.000000E+00
Olfr65	26632		#N/A	1.000000E+00
Olfr650-ps1	26633		#N/A	1.000000E+00
Olfr651	26634		#N/A	1.000000E+00
Olfr652	26635		#N/A	1.000000E+00
Olfr653	26636		#N/A	1.000000E+00
Olfr654	26637		#N/A	1.000000E+00
Olfr655	26638		#N/A	1.000000E+00
Olfr656	26639		#N/A	1.000000E+00
Olfr657	26640		#N/A	1.000000E+00
Olfr658	26641		#N/A	1.000000E+00
Olfr659	26642		#N/A	1.000000E+00
Olfr66	26643		#N/A	1.000000E+00
Olfr660-ps1	26644		#N/A	1.000000E+00
Olfr661	26645		#N/A	1.000000E+00
Olfr663	26646		#N/A	1.000000E+00
Olfr664	26647		#N/A	1.000000E+00
Olfr665	26648		#N/A	1.000000E+00
Olfr666	26649		#N/A	1.000000E+00
Olfr667	26650		#N/A	1.000000E+00
Olfr668	26651		#N/A	1.000000E+00
Olfr669	26652	0.160304018	16323	2.335876E-02
Olfr67	26653		#N/A	1.000000E+00
Olfr670	26654		#N/A	1.000000E+00
Olfr671	26655		#N/A	1.000000E+00
Olfr672	26656		#N/A	1.000000E+00
Olfr675	26657		#N/A	1.000000E+00
Olfr676	26658		#N/A	1.000000E+00
Olfr677	26659		#N/A	1.000000E+00

Spearman Rank correlation analysis performed between Prdm1 and all-expressed genes within the Meredith RNA-seq dataset. Robust Prdm1–associated genes were identified using a cut-off of $p < 0.0005$.

Olfr678	26660		#N/A	1.000000E+00
Olfr679	26661		#N/A	1.000000E+00
Olfr68	26662		#N/A	1.000000E+00
Olfr680-ps1	26663		#N/A	1.000000E+00
Olfr681	26664		#N/A	1.000000E+00
Olfr682-ps1	26665		#N/A	1.000000E+00
Olfr683	26666		#N/A	1.000000E+00
Olfr684	26667		#N/A	1.000000E+00
Olfr685	26668		#N/A	1.000000E+00
Olfr686	26669		#N/A	1.000000E+00
Olfr687	26670		#N/A	1.000000E+00
Olfr688	26671		#N/A	1.000000E+00
Olfr689	26672		#N/A	1.000000E+00
Olfr69	26673		#N/A	1.000000E+00
Olfr690	26674		#N/A	1.000000E+00
Olfr691	26675		#N/A	1.000000E+00
Olfr692	26676		#N/A	1.000000E+00
Olfr693	26677		#N/A	1.000000E+00
Olfr694	26678		#N/A	1.000000E+00
Olfr695	26679		#N/A	1.000000E+00
Olfr697	26680		#N/A	1.000000E+00
Olfr698	26681		#N/A	1.000000E+00
Olfr699	26682		#N/A	1.000000E+00
Olfr70	26683		#N/A	1.000000E+00
Olfr700	26684		#N/A	1.000000E+00
Olfr701	26685	0.021317126	6513.5	7.644715E-01
Olfr702	26686		#N/A	1.000000E+00
Olfr703	26687		#N/A	1.000000E+00
Olfr704	26688		#N/A	1.000000E+00
Olfr705	26689		#N/A	1.000000E+00
Olfr706	26690		#N/A	1.000000E+00
Olfr707	26691		#N/A	1.000000E+00
Olfr709-ps1	26692		#N/A	1.000000E+00
Olfr71	26693		#N/A	1.000000E+00
Olfr710	26694		#N/A	1.000000E+00
Olfr711	26695		#N/A	1.000000E+00
Olfr713	26696		#N/A	1.000000E+00
Olfr714	26697		#N/A	1.000000E+00
Olfr715	26698		#N/A	1.000000E+00
Olfr716	26699		#N/A	1.000000E+00
Olfr718-ps1	26700		#N/A	1.000000E+00
Olfr719-ps	26701		#N/A	1.000000E+00
Olfr720	26702		#N/A	1.000000E+00

Spearman Rank correlation analysis performed between Prdm1 and all-expressed genes within the Meredith RNA-seq dataset. Robust Prdm1–associated genes were identified using a cut-off of $p < 0.0005$.

Table S1, Related to Supplemental Figure 3C. Prdm1 associated genes

Olfr721-ps1	26703		#N/A	1.000000E+00
Olfr722	26704		#N/A	1.000000E+00
Olfr723	26705		#N/A	1.000000E+00
Olfr724	26706		#N/A	1.000000E+00
Olfr725	26707		#N/A	1.000000E+00
Olfr726	26708		#N/A	1.000000E+00
Olfr727	26709		#N/A	1.000000E+00
Olfr728	26710		#N/A	1.000000E+00
Olfr729	26711		#N/A	1.000000E+00
Olfr73	26712		#N/A	1.000000E+00
Olfr730	26713		#N/A	1.000000E+00
Olfr731	26714		#N/A	1.000000E+00
Olfr732	26715		#N/A	1.000000E+00
Olfr733	26716		#N/A	1.000000E+00
Olfr734	26717		#N/A	1.000000E+00
Olfr735	26718		#N/A	1.000000E+00
Olfr736	26719		#N/A	1.000000E+00
Olfr738	26720	-0.038406171	3065	5.892384E-01
Olfr739	26721		#N/A	1.000000E+00
Olfr74	26722		#N/A	1.000000E+00
Olfr740	26723		#N/A	1.000000E+00
Olfr741	26724		#N/A	1.000000E+00
Olfr742	26725		#N/A	1.000000E+00
Olfr743	26726		#N/A	1.000000E+00
Olfr744	26727		#N/A	1.000000E+00
Olfr745	26728		#N/A	1.000000E+00
Olfr746	26729		#N/A	1.000000E+00
Olfr747	26730		#N/A	1.000000E+00
Olfr748	26731		#N/A	1.000000E+00
Olfr749	26732	0.053267784	8685	4.537786E-01
Olfr75-ps1	26733		#N/A	1.000000E+00
Olfr750	26734		#N/A	1.000000E+00
Olfr753-ps1	26735		#N/A	1.000000E+00
Olfr754-ps1	26736		#N/A	1.000000E+00
Olfr755-ps1	26737		#N/A	1.000000E+00
Olfr756-ps1	26738		#N/A	1.000000E+00
Olfr757-ps1	26739		#N/A	1.000000E+00
Olfr758-ps1	26740		#N/A	1.000000E+00
Olfr759-ps1	26741		#N/A	1.000000E+00
Olfr76	26742		#N/A	1.000000E+00
Olfr760-ps1	26743		#N/A	1.000000E+00
Olfr761	26744		#N/A	1.000000E+00
Olfr762-ps1	26745		#N/A	1.000000E+00

Spearman Rank correlation analysis performed between Prdm1 and all-expressed genes within the Meredith RNA-seq dataset. Robust Prdm1–associated genes were identified using a cut-off of $p < 0.0005$.

Table S1, Related to Supplemental Figure 3C. Prdm1 associated genes

Olfr763	26746		#N/A	1.000000E+00
Olfr765	26747		#N/A	1.000000E+00
Olfr767	26748		#N/A	1.000000E+00
Olfr768	26749		#N/A	1.000000E+00
Olfr769	26750		#N/A	1.000000E+00
Olfr77	26751	-0.038406171	3065	5.892384E-01
Olfr770	26752		#N/A	1.000000E+00
Olfr771	26753		#N/A	1.000000E+00
Olfr772	26754		#N/A	1.000000E+00
Olfr773	26755		#N/A	1.000000E+00
Olfr774	26756		#N/A	1.000000E+00
Olfr775	26757		#N/A	1.000000E+00
Olfr776	26758	-0.054450825	1984	4.437997E-01
Olfr777	26759		#N/A	1.000000E+00
Olfr779	26760		#N/A	1.000000E+00
Olfr78	26761		#N/A	1.000000E+00
Olfr780	26762		#N/A	1.000000E+00
Olfr781	26763		#N/A	1.000000E+00
Olfr782	26764		#N/A	1.000000E+00
Olfr784	26765		#N/A	1.000000E+00
Olfr786	26766		#N/A	1.000000E+00
Olfr787	26767		#N/A	1.000000E+00
Olfr788	26768		#N/A	1.000000E+00
Olfr790	26769		#N/A	1.000000E+00
Olfr791	26770		#N/A	1.000000E+00
Olfr792	26771		#N/A	1.000000E+00
Olfr794	26772		#N/A	1.000000E+00
Olfr796	26773		#N/A	1.000000E+00
Olfr798	26774		#N/A	1.000000E+00
Olfr799	26775		#N/A	1.000000E+00
Olfr8	26776		#N/A	1.000000E+00
Olfr800	26777		#N/A	1.000000E+00
Olfr801	26778		#N/A	1.000000E+00
Olfr802	26779		#N/A	1.000000E+00
Olfr803	26780		#N/A	1.000000E+00
Olfr804	26781		#N/A	1.000000E+00
Olfr805	26782		#N/A	1.000000E+00
Olfr806	26783		#N/A	1.000000E+00
Olfr807	26784		#N/A	1.000000E+00
Olfr808	26785		#N/A	1.000000E+00
Olfr809	26786		#N/A	1.000000E+00
Olfr810	26787		#N/A	1.000000E+00
Olfr811	26788		#N/A	1.000000E+00

Spearman Rank correlation analysis performed between Prdm1 and all-expressed genes within the Meredith RNA-seq dataset. Robust Prdm1–associated genes were identified using a cut-off of $p < 0.0005$.

Table S1, Related to Supplemental Figure 3C. Prdm1 associated genes

Olfr812	26789		#N/A	1.000000E+00
Olfr813	26790		#N/A	1.000000E+00
Olfr814	26791		#N/A	1.000000E+00
Olfr815	26792		#N/A	1.000000E+00
Olfr816	26793		#N/A	1.000000E+00
Olfr818	26794		#N/A	1.000000E+00
Olfr819	26795		#N/A	1.000000E+00
Olfr820	26796		#N/A	1.000000E+00
Olfr821	26797		#N/A	1.000000E+00
Olfr822	26798		#N/A	1.000000E+00
Olfr823	26799		#N/A	1.000000E+00
Olfr824	26800		#N/A	1.000000E+00
Olfr825	26801		#N/A	1.000000E+00
Olfr826	26802		#N/A	1.000000E+00
Olfr827	26803		#N/A	1.000000E+00
Olfr828	26804		#N/A	1.000000E+00
Olfr829	26805		#N/A	1.000000E+00
Olfr830	26806		#N/A	1.000000E+00
Olfr831-ps1	26807	0.053267784	8685	4.537786E-01
Olfr832	26808		#N/A	1.000000E+00
Olfr833-ps1	26809		#N/A	1.000000E+00
Olfr834	26810		#N/A	1.000000E+00
Olfr835	26811		#N/A	1.000000E+00
Olfr836	26812		#N/A	1.000000E+00
Olfr837	26813		#N/A	1.000000E+00
Olfr843	26814		#N/A	1.000000E+00
Olfr844	26815		#N/A	1.000000E+00
Olfr845	26816		#N/A	1.000000E+00
Olfr846	26817		#N/A	1.000000E+00
Olfr847	26818		#N/A	1.000000E+00
Olfr849	26819		#N/A	1.000000E+00
Olfr850	26820		#N/A	1.000000E+00
Olfr851	26821		#N/A	1.000000E+00
Olfr853	26822		#N/A	1.000000E+00
Olfr854	26823		#N/A	1.000000E+00
Olfr855	26824		#N/A	1.000000E+00
Olfr857	26825		#N/A	1.000000E+00
Olfr859	26826		#N/A	1.000000E+00
Olfr860	26827	0.143605682	15224	4.249002E-02
Olfr862	26828		#N/A	1.000000E+00
Olfr866	26829		#N/A	1.000000E+00
Olfr867	26830		#N/A	1.000000E+00
Olfr868	26831		#N/A	1.000000E+00

Spearman Rank correlation analysis performed between Prdm1 and all-expressed genes within the Meredith RNA-seq dataset. Robust Prdm1–associated genes were identified using a cut-off of $p < 0.0005$.

Table S1, Related to Supplemental Figure 3C. Prdm1 associated genes

Olfr869	26832		#N/A	1.000000E+00
Olfr870	26833		#N/A	1.000000E+00
Olfr871	26834		#N/A	1.000000E+00
Olfr872	26835	-0.054451513	1703	4.437940E-01
Olfr873	26836		#N/A	1.000000E+00
Olfr874	26837		#N/A	1.000000E+00
Olfr875	26838		#N/A	1.000000E+00
Olfr876	26839		#N/A	1.000000E+00
Olfr877	26840		#N/A	1.000000E+00
Olfr878	26841		#N/A	1.000000E+00
Olfr881	26842		#N/A	1.000000E+00
Olfr883	26843		#N/A	1.000000E+00
Olfr884	26844		#N/A	1.000000E+00
Olfr885	26845		#N/A	1.000000E+00
Olfr887	26846		#N/A	1.000000E+00
Olfr888	26847		#N/A	1.000000E+00
Olfr889	26848		#N/A	1.000000E+00
Olfr890	26849		#N/A	1.000000E+00
Olfr891	26850		#N/A	1.000000E+00
Olfr892-ps1	26851		#N/A	1.000000E+00
Olfr893	26852		#N/A	1.000000E+00
Olfr894	26853		#N/A	1.000000E+00
Olfr895	26854		#N/A	1.000000E+00
Olfr898	26855		#N/A	1.000000E+00
Olfr9	26856		#N/A	1.000000E+00
Olfr90	26857		#N/A	1.000000E+00
Olfr901	26858		#N/A	1.000000E+00
Olfr902	26859		#N/A	1.000000E+00
Olfr904	26860		#N/A	1.000000E+00
Olfr905	26861		#N/A	1.000000E+00
Olfr906	26862		#N/A	1.000000E+00
Olfr907	26863		#N/A	1.000000E+00
Olfr908	26864		#N/A	1.000000E+00
Olfr91	26865		#N/A	1.000000E+00
Olfr910	26866		#N/A	1.000000E+00
Olfr911-ps1	26867		#N/A	1.000000E+00
Olfr912	26868		#N/A	1.000000E+00
Olfr913	26869		#N/A	1.000000E+00
Olfr914	26870		#N/A	1.000000E+00
Olfr916	26871		#N/A	1.000000E+00
Olfr917	26872		#N/A	1.000000E+00
Olfr918	26873		#N/A	1.000000E+00
Olfr919	26874		#N/A	1.000000E+00

Spearman Rank correlation analysis performed between Prdm1 and all-expressed genes within the Meredith RNA-seq dataset. Robust Prdm1–associated genes were identified using a cut-off of $p < 0.0005$.

Table S1, Related to Supplemental Figure 3C. Prdm1 associated genes

Olfr92	26875		#N/A	1.000000E+00
Olfr920	26876	-0.000458472	5251.5	9.948592E-01
Olfr921	26877		#N/A	1.000000E+00
Olfr922	26878		#N/A	1.000000E+00
Olfr923	26879		#N/A	1.000000E+00
Olfr924	26880		#N/A	1.000000E+00
Olfr926	26881		#N/A	1.000000E+00
Olfr93	26882		#N/A	1.000000E+00
Olfr930	26883		#N/A	1.000000E+00
Olfr933	26884	-0.038406171	3065	5.892384E-01
Olfr934	26885		#N/A	1.000000E+00
Olfr935	26886		#N/A	1.000000E+00
Olfr936	26887		#N/A	1.000000E+00
Olfr937	26888		#N/A	1.000000E+00
Olfr938	26889		#N/A	1.000000E+00
Olfr94	26890		#N/A	1.000000E+00
Olfr943	26891		#N/A	1.000000E+00
Olfr944	26892		#N/A	1.000000E+00
Olfr945	26893		#N/A	1.000000E+00
Olfr948	26894		#N/A	1.000000E+00
Olfr95	26895		#N/A	1.000000E+00
Olfr951	26896		#N/A	1.000000E+00
Olfr952	26897		#N/A	1.000000E+00
Olfr954	26898		#N/A	1.000000E+00
Olfr955	26899		#N/A	1.000000E+00
Olfr957	26900		#N/A	1.000000E+00
Olfr958	26901		#N/A	1.000000E+00
Olfr959	26902		#N/A	1.000000E+00
Olfr96	26903		#N/A	1.000000E+00
Olfr960	26904		#N/A	1.000000E+00
Olfr961	26905		#N/A	1.000000E+00
Olfr963	26906		#N/A	1.000000E+00
Olfr964-ps1	26907		#N/A	1.000000E+00
Olfr965	26908		#N/A	1.000000E+00
Olfr967	26909		#N/A	1.000000E+00
Olfr968	26910		#N/A	1.000000E+00
Olfr969	26911		#N/A	1.000000E+00
Olfr97	26912		#N/A	1.000000E+00
Olfr970	26913		#N/A	1.000000E+00
Olfr971	26914		#N/A	1.000000E+00
Olfr972	26915		#N/A	1.000000E+00
Olfr974	26916		#N/A	1.000000E+00
Olfr975	26917		#N/A	1.000000E+00

Spearman Rank correlation analysis performed between Prdm1 and all-expressed genes within the Meredith RNA-seq dataset. Robust Prdm1–associated genes were identified using a cut-off of $p < 0.0005$.

Table S1, Related to Supplemental Figure 3C. Prdm1 associated genes

Olfr976	26918		#N/A	1.000000E+00
Olfr978	26919		#N/A	1.000000E+00
Olfr979	26920		#N/A	1.000000E+00
Olfr98	26921		#N/A	1.000000E+00
Olfr980	26922		#N/A	1.000000E+00
Olfr981	26923		#N/A	1.000000E+00
Olfr982	26924		#N/A	1.000000E+00
Olfr983	26925		#N/A	1.000000E+00
Olfr984	26926		#N/A	1.000000E+00
Olfr985	26927	-0.077393517	797	2.760287E-01
Olfr986	26928		#N/A	1.000000E+00
Olfr987	26929	-0.038406171	3065	5.892384E-01
Olfr988	26930	0.053267784	8685	4.537786E-01
Olfr99	26931		#N/A	1.000000E+00
Olfr990-ps1	26932		#N/A	1.000000E+00
Olfr992	26933		#N/A	1.000000E+00
Olfr993	26934		#N/A	1.000000E+00
Olfr994	26935		#N/A	1.000000E+00
Olfr995	26936		#N/A	1.000000E+00
Olfr996	26937		#N/A	1.000000E+00
Olfr997-ps1	26938		#N/A	1.000000E+00
Olfr998	26939		#N/A	1.000000E+00
Olfr999-ps1	26940		#N/A	1.000000E+00
Olig1	26941	-0.095267438	357.5	1.796303E-01
Olig2	26942	0.134631789	14596	5.734010E-02
Olig3	26943	0.003124816	5482	9.649724E-01
Olr1	26944	0.150270221	15666	3.367881E-02
Oma1	26945	0.137760481	14807	5.173879E-02
Omd	26946	0.231147818	19189	9.907293E-04
Omg	26947		#N/A	1.000000E+00
Omp	26948	0.045967926	8003	5.180516E-01
Omt2a	26949	0.108758338	12607	1.252795E-01
Omt2b	26950	0.075710566	10305	2.866371E-01
Onecut1	26951		#N/A	1.000000E+00
Onecut2	26952	0.107208334	12510	1.307860E-01
Onecut3	26953	-0.013952569	4568	8.445383E-01
Ooep	26954	0.162965591	16543	2.112903E-02
Oog1	26955	-0.054450825	1984	4.437997E-01
Oog2	26956		#N/A	1.000000E+00
Oog3	26957		#N/A	1.000000E+00
OOG4	26958		#N/A	1.000000E+00
Oog4	26959	-0.095273984	329	1.796002E-01
Oosp1	26960	-0.01649706	4459	8.166490E-01

Spearman Rank correlation analysis performed between Prdm1 and all-expressed genes within the Meredith RNA-seq dataset. Robust Prdm1–associated genes were identified using a cut-off of $p < 0.0005$.

Table S1, Related to Supplemental Figure 3C. Prdm1 associated genes

Opa1	26961	0.082626195	10798	2.447578E-01
Opa3	26962	0.124541884	13941	7.890149E-02
Opalin	26963	0.143605682	15224	4.249002E-02
Opcml	26964	0.23627303	19294	7.563904E-04
Ophn1	26965	0.179088262	17362	1.116912E-02
Oplah	26966	0.085477239	11019	2.287993E-01
Opn1mw	26967	0.037182848	7442	6.011624E-01
Opn1sw	26968	-0.018447724	4312.5	7.954215E-01
Opn3	26969	0.070583869	9879	3.206178E-01
Opn4	26970	0.182294195	17509.5	9.778799E-03
Opn5	26971	0.038261076	7509	5.906470E-01
Oprd1	26972	-0.071777888	930	3.124793E-01
Oprk1	26973	-0.077397959	691.5	2.760011E-01
Oprl1	26974		#N/A	1.000000E+00
Oprm1	26975	0.138723351	14868	5.010929E-02
Optc	26976	0.005032777	5564	9.436138E-01
Optn	26977	0.246085162	19456	4.438662E-04
Orai1	26978	0.083964972	10919	2.371701E-01
Orai2	26979	0.093365908	11606	1.885128E-01
Orai3	26980	0.059782599	9199	4.004002E-01
Oraov1	26981	0.120052076	13667	9.040211E-02
Orc1	26982	-0.066858259	1029.5	3.468868E-01
Orc2	26983	0.060236963	9223	3.968227E-01
Orc3	26984	0.007367201	5649	9.175373E-01
Orc4	26985	0.087143875	11140	2.198183E-01
Orc5	26986	0.125711203	14016	7.610872E-02
Orc6	26987	0.167528475	16799	1.773320E-02
Orm1	26988	-0.036885706	3665	6.040749E-01
Orm2	26989	-0.077394997	736.5	2.760195E-01
Orm3	26990		#N/A	1.000000E+00
Ormdl1	26991	0.17273789	17065	1.444567E-02
Ormdl2	26992	-0.039554386	2479	5.781460E-01
Ormdl3	26993	0.085461409	11018	2.288858E-01
Os9	26994	0.202929139	18354	3.952732E-03
Osbp	26995	0.03505269	7331	6.221792E-01
Osbp2	26996	0.12384755	13893	8.059859E-02
Osbpl10	26997	0.15630356	16072	2.709036E-02
Osbpl11	26998	0.031511265	7160	6.577995E-01
Osbpl1a	26999	0.026170943	6879	7.129805E-01
Osbpl2	27000	-0.139197083	40	4.932337E-02
Osbpl3	27001	0.121556784	13758	8.640731E-02
Osbpl5	27002	0.098154585	11925	1.667397E-01
Osbpl6	27003	-0.055791667	1497	4.326417E-01

Spearman Rank correlation analysis performed between Prdm1 and all-expressed genes within the Meredith RNA-seq dataset. Robust Prdm1–associated genes were identified using a cut-off of $p < 0.0005$.

Table S1, Related to Supplemental Figure 3C. Prdm1 associated genes

Osbpl7	27004	0.233579222	19245	8.722998E-04
Osbpl8	27005	0.192514747	17933	6.312346E-03
Osbpl9	27006	0.132534821	14449	6.136746E-02
Oscar	27007	-0.054450825	1984	4.437997E-01
Oscp1	27008	0.202862701	18350	3.964837E-03
Osgep	27009	0.068998036	9776	3.316375E-01
Osgepl1	27010	-0.021898177	4175	7.582476E-01
Osgin1	27011	0.122897414	13836	8.296847E-02
Osgin2	27012	0.124264277	13923	7.957653E-02
Osm	27013	0.075328288	10286.5	2.890843E-01
Osmr	27014	0.16959654	16908	1.635740E-02
Osr1	27015	-0.066856562	1139	3.468991E-01
Osr2	27016	0.110504365	12758	1.192933E-01
Ost4	27017	0.2581563	19602	2.235885E-04
Ostc	27018	0.241968078	19396	5.565808E-04
Ostf1	27019	0.159815826	16265	2.378902E-02
Ostm1	27020	0.172862581	17071	1.437400E-02
Ostn	27021	-0.082422326	613	2.459280E-01
Otc	27022	0.074574898	10201.5	2.939481E-01
Otoa	27023	-0.100614262	271	1.563130E-01
Otof	27024		#N/A	1.000000E+00
Otog	27025	0.039546669	7605	5.782202E-01
Otogl	27026	0.163783431	16582	2.048194E-02
Otol1	27027	0.176086238	17227.5	1.262605E-02
Otop1	27028		#N/A	1.000000E+00
Otop2	27029	0.071450202	9947	3.146992E-01
Otop3	27030		#N/A	1.000000E+00
Otor	27031	-0.038406171	3065	5.892384E-01
Otos	27032	0.038757099	7545	5.858379E-01
Otp	27033	0.162808768	16526.5	2.125512E-02
Ott	27034		#N/A	1.000000E+00
OTTMUSG0000(	27035		#N/A	1.000000E+00
Otub1	27036	0.189393371	17817	7.231483E-03
Otub2	27037	-0.025387293	4089	7.212134E-01
Otud1	27038	-0.103159572	257	1.460480E-01
Otud3	27039	0.104362091	12315	1.413802E-01
Otud4	27040	0.050254309	8322	4.797559E-01
Otud5	27041	0.156260027	16068	2.713363E-02
Otud6a	27042		#N/A	1.000000E+00
Otud6b	27043	0.249198837	19498	3.730962E-04
Otud7a	27044	0.1196911	13637	9.138205E-02
Otud7b	27045	0.263526725	19664	1.630283E-04
Otx1	27046	0.260759097	19632	1.920109E-04

Spearman Rank correlation analysis performed between Prdm1 and all-expressed genes within the Meredith RNA-seq dataset. Robust Prdm1–associated genes were identified using a cut-off of $p < 0.0005$.

Table S1, Related to Supplemental Figure 3C. Prdm1 associated genes

Otx2	27047	0.113548679	13094	1.093908E-01
Ovca2	27048	0.022237233	6646	7.546230E-01
Ovch2	27049		#N/A	1.000000E+00
Ovgp1	27050	-0.018447724	4312.5	7.954215E-01
Ovol1	27051	-0.048651397	2225	4.938948E-01
Ovol2	27052	0.053267784	8685	4.537786E-01
Oxa1l	27053	0.13269156	14459	6.105861E-02
Oxct1	27054	0.133105659	14485	6.024877E-02
Oxct2a	27055		#N/A	1.000000E+00
Oxct2b	27056		#N/A	1.000000E+00
Oxgr1	27057	0.042893115	7804	5.464583E-01
Oxld1	27058	-0.017417138	4396	8.066189E-01
Oxnad1	27059	0.099121589	12007	1.625803E-01
Oxr1	27060	0.100770475	12108	1.556678E-01
Oxsm	27061	0.066567987	9627	3.489891E-01
Oxsr1	27062		#N/A	1.000000E+00
Oxt	27063	-0.086746249	548	2.219378E-01
Oxtr	27064	0.126047776	14050.5	7.531986E-02
P2rx1	27065	-0.030875359	3900	6.642811E-01
P2rx2	27066	0.071180894	9923	3.165314E-01
P2rx3	27067	0.152675221	15805	3.090535E-02
P2rx4	27068	0.034895105	7324	6.237465E-01
P2rx5	27069	0.050883637	8377	4.742652E-01
P2rx6	27070		#N/A	1.000000E+00
P2rx7	27071	0.058475706	9124	4.107970E-01
P2ry1	27072	0.070871548	9903	3.186445E-01
P2ry10	27073	0.09990813	12059	1.592548E-01
P2ry12	27074	-0.094666809	378	1.824020E-01
P2ry13	27075	0.021316405	6469	7.644793E-01
P2ry14	27076	0.183789218	17583	9.184489E-03
P2ry2	27077	0.063365727	9418	3.727114E-01
P2ry4	27078	0.031004514	7131	6.629626E-01
P2ry6	27079	-0.020397608	4226	7.743516E-01
P4ha1	27080	0.29904694	19903	1.696448E-05
P4ha2	27081	0.090988401	11434	2.000657E-01
P4ha3	27082	0.046514852	8045	5.130794E-01
P4hb	27083	0.12800564	14190	7.086141E-02
P4htm	27084	0.076993209	10412	2.785276E-01
Pa2g4	27085	0.198079118	18169	4.929330E-03
Pabpc1	27086	0.144857037	15348	4.070188E-02
Pabpc1l	27087		#N/A	1.000000E+00
Pabpc1l2a-ps	27088		#N/A	1.000000E+00
Pabpc1l2b-ps	27089		#N/A	1.000000E+00

Spearman Rank correlation analysis performed between Prdm1 and all-expressed genes within the Meredith RNA-seq dataset. Robust Prdm1–associated genes were identified using a cut-off of $p < 0.0005$.

Table S1, Related to Supplemental Figure 3C. Prdm1 associated genes

Pabpc2	27090	-0.018845765	4277	7.911078E-01
Pabpc4	27091	0.078182886	10477	2.711457E-01
Pabpc4l	27092	0.196953899	18122	5.184788E-03
Pabpc5	27093		#N/A	1.000000E+00
Pabpc6	27094		#N/A	1.000000E+00
Pabpn1	27095	0.09838033	11946	1.657617E-01
Pabpn1l	27096	-0.054450825	1984	4.437997E-01
Pacrg	27097	0.074025548	10137	2.975288E-01
Pacrgl	27098	0.196335746	18097	5.330123E-03
Pacs1	27099	0.122278931	13801	8.454101E-02
Pacs2	27100	0.010125286	5777	8.868436E-01
Pacsin1	27101	0.242593932	19407	5.378930E-04
Pacsin2	27102	0.097702649	11882	1.687106E-01
Pacsin3	27103	-0.014936007	4513	8.337342E-01
Padi1	27104	-0.053756716	2126	4.496393E-01
Padi2	27105	0.066380419	9612	3.503518E-01
Padi3	27106	0.08428891	10941	2.353592E-01
Padi4	27107	0.192355888	17926	6.356475E-03
Padi6	27108		#N/A	1.000000E+00
Paf1	27109	0.000304498	5307	9.965857E-01
Pafah1b1	27110	0.155539149	16009	2.785871E-02
Pafah1b2	27111	0.087922739	11197	2.157085E-01
Pafah1b3	27112	0.170167885	16938	1.599412E-02
Pafah2	27113	0.056346464	9001	4.280724E-01
Pag1	27114	0.072732713	10041	3.060692E-01
Pagr1a	27115	-0.038406171	3065	5.892384E-01
Pagr1b	27116	-0.048520129	2227	4.950624E-01
Pah	27117	-0.048496005	2229	4.952771E-01
Paics	27118	0.108844767	12613	1.249778E-01
Paip1	27119	0.010685107	5804	8.806326E-01
Paip2	27120	0.039782993	7615	5.759492E-01
Paip2b	27121	0.064621594	9512	3.632922E-01
Pak1	27122	0.088786469	11256	2.112153E-01
Pak1ip1	27123	-0.031031478	3886	6.626875E-01
Pak2	27124	0.187089255	17720	7.984569E-03
Pak3	27125	0.052283593	8490	4.621750E-01
Pak4	27126	0.005074581	5567	9.431462E-01
Pak6	27127	0.052844823	8584	4.573765E-01
Pak7	27128	0.026212444	6881	7.125454E-01
Palb2	27129	0.074551089	10181	2.941027E-01
Pald1	27130	0.141632953	15055	4.544266E-02
Palld	27131	0.312609762	19945	6.583583E-06
Palm	27132	0.03981323	7621.5	5.756589E-01

Spearman Rank correlation analysis performed between Prdm1 and all-expressed genes within the Meredith RNA-seq dataset. Robust Prdm1–associated genes were identified using a cut-off of $p < 0.0005$.

Table S1, Related to Supplemental Figure 3C. Prdm1 associated genes

Palm2	27133	0.143676612	15288	4.238693E-02
Palm3	27134		#N/A	1.000000E+00
Palmd	27135	0.193383162	17963	6.075911E-03
Pam	27136	0.168705031	16862	1.693864E-02
Pam16	27137	0.152249259	15782	3.138190E-02
Pamr1	27138	0.035858775	7370	6.141889E-01
Pan2	27139	0.274840721	19758	8.196184E-05
Pan3	27140	0.164867797	16647	1.965047E-02
Pank1	27141	0.067869621	9694	3.396249E-01
Pank2	27142	0.104804222	12346	1.396929E-01
Pank3	27143	0.012137126	5882	8.645577E-01
Pank4	27144	0.033051422	7242	6.422073E-01
Panx1	27145	0.148943899	15587	3.529676E-02
Panx2	27146	-0.038406171	3065	5.892384E-01
Panx3	27147		#N/A	1.000000E+00
Paox	27148	0.142628789	15126	4.393139E-02
Papd4	27149	0.130708983	14335	6.506077E-02
Papd5	27150	0.208510462	18537	3.047193E-03
Papd7	27151	0.016367958	6138	8.180588E-01
Papln	27152	0.175085151	17170	1.314773E-02
Papola	27153	0.23150976	19199	9.722109E-04
Papolb	27154		#N/A	1.000000E+00
Papolg	27155	0.204889415	18421	3.610227E-03
Pappa	27156	0.090105593	11356	2.044835E-01
Pappa2	27157	0.059213021	9168	4.049119E-01
Papss1	27158	0.084732768	10967	2.328937E-01
Papss2	27159	-0.014683548	4533	8.365048E-01
Paqr3	27160	0.110304258	12740	1.199679E-01
Paqr4	27161	-0.038406171	3065	5.892384E-01
Paqr5	27162	0.037962797	7480	5.935475E-01
Paqr6	27163	-0.038406171	3065	5.892384E-01
Paqr7	27164	0.122737791	13828	8.337206E-02
Paqr8	27165	0.200875996	18280	4.342606E-03
Paqr9	27166	0.211295545	18624	2.669599E-03
Pard3	27167	0.225293227	19055	1.338968E-03
Pard3b	27168	0.09853701	11959	1.650854E-01
Pard6a	27169	0.02654196	6906	7.090941E-01
Pard6b	27170	0.050600118	8351	4.767346E-01
Pard6g	27171	0.118236484	13543	9.541707E-02
Parg	27172	0.207891715	18519	3.137382E-03
Park2	27173	0.097839521	11897	1.681119E-01
Park7	27174	0.182922731	17549	9.524917E-03
Parl	27175	0.017439317	6201	8.063775E-01

Spearman Rank correlation analysis performed between Prdm1 and all-expressed genes within the Meredith RNA-seq dataset. Robust Prdm1–associated genes were identified using a cut-off of $p < 0.0005$.

Table S1, Related to Supplemental Figure 3C. Prdm1 associated genes

Parm1	27176	0.161699838	16457	2.216536E-02
Parn	27177	-0.00956807	4763	8.930326E-01
Parp1	27178	0.243233585	19419	5.193939E-04
Parp10	27179	0.002802966	5461	9.685782E-01
Parp11	27180	0.040351369	7652	5.705044E-01
Parp12	27181	0.010589429	5794	8.816936E-01
Parp14	27182	0.165319986	16676	1.931247E-02
Parp16	27183	0.046244381	8030	5.155352E-01
Parp2	27184	0.005253075	5573	9.411500E-01
Parp3	27185	0.07175	9974	3.126678E-01
Parp4	27186	0.055279416	8944	4.368853E-01
Parp6	27187	0.118967687	13584	9.337140E-02
Parp8	27188	0.015676682	6103	8.256175E-01
Parp9	27189	0.169046338	16873	1.671403E-02
Parpbp	27190	0.092742272	11563	1.914949E-01
Pars2	27191	0.041822555	7731	5.565254E-01
Parva	27192	-0.03287462	3790	6.439894E-01
Parvb	27193	0.015599065	6099	8.264672E-01
Parvg	27194	0.095849592	11759	1.769736E-01
Pask	27195	-0.026995062	4030	7.043581E-01
Pate2	27196	0.095549134	11739	1.783411E-01
Pate4	27197	0.08346028	10874	2.400109E-01
Patl1	27198	0.219267016	18883	1.811471E-03
Patl2	27199	0.116938212	13459	9.913683E-02
Patz1	27200	0.069407762	9809	3.287673E-01
Pawr	27201	0.016861168	6164	8.126761E-01
Pax1	27202	0.091479537	11484	1.976381E-01
Pax2	27203	0.081284693	10683	2.525290E-01
Pax3	27204	0.037442236	7452	5.986250E-01
Pax4	27205	0.023709233	6735.5	7.389498E-01
Pax5	27206	0.084297444	10942	2.353116E-01
Pax6	27207	0.099789225	12049	1.597542E-01
Pax6os1	27208	0.223935666	19015	1.434296E-03
Pax7	27209		#N/A	1.000000E+00
Pax8	27210	0.279600508	19791	6.081460E-05
Pax9	27211	-0.001563228	5115	9.824728E-01
Paxbp1	27212	0.110072398	12720	1.207532E-01
Paxip1	27213	0.218760902	18866	1.857378E-03
Pbdc1	27214	0.207368252	18509	3.215560E-03
Pbk	27215	0.211453916	18631	2.649461E-03
Pbld1	27216	-0.02995588	3928	6.736972E-01
Pbld2	27217	-0.107145315	199	1.310137E-01
Pbp2	27218		#N/A	1.000000E+00

Spearman Rank correlation analysis performed between Prdm1 and all-expressed genes within the Meredith RNA-seq dataset. Robust Prdm1–associated genes were identified using a cut-off of $p < 0.0005$.

Table S1, Related to Supplemental Figure 3C. Prdm1 associated genes

Pbrm1	27219	0.150375016	15671	3.355370E-02
Pbsn	27220		#N/A	1.000000E+00
Pbx1	27221	0.094629448	11680	1.825754E-01
Pbx2	27222	-0.085553561	560	2.283825E-01
Pbx3	27223	0.111331861	12812	1.165351E-01
Pbx4	27224	0.081983299	10743	2.484610E-01
Pbxip1	27225	0.120167621	13673	9.009022E-02
Pcbd1	27226	0.059255321	9171	4.045758E-01
Pcbd2	27227	0.215195036	18751	2.212070E-03
Pcbp1	27228	0.196866347	18116	5.205154E-03
Pcbp2	27229	0.183621325	17577	9.249597E-03
Pcbp3	27230	0.058903463	9151	4.073766E-01
Pcbp4	27231	0.025400228	6845	7.210772E-01
Pcca	27232	0.083654371	10892	2.389156E-01
Pccb	27233	0.207235798	18501	3.235619E-03
Pcdh1	27234	0.030104186	7070	6.721750E-01
Pcdh10	27235	0.034979855	7329	6.229034E-01
Pcdh11x	27236	0.040957824	7692	5.647219E-01
Pcdh12	27237	0.076062641	10353	2.843955E-01
Pcdh15	27238	0.079065856	10534	2.657537E-01
Pcdh17	27239	0.03440387	7308	6.286430E-01
Pcdh18	27240	0.018804082	6269	7.915592E-01
Pcdh19	27241	0.145352952	15375	4.001096E-02
Pcdh20	27242	-0.017074363	4409	8.103520E-01
Pcdh7	27243	-0.047988458	2233	4.998064E-01
Pcdh8	27244	-0.019871831	4244	7.800175E-01
Pcdh9	27245	0.067828758	9692	3.399164E-01
Pcdhac1	27246	0.086709084	11113	2.221367E-01
Pcdhb1	27247		#N/A	1.000000E+00
Pcdhb10	27248	0.052728522	8550.5	4.583686E-01
Pcdhb11	27249		#N/A	1.000000E+00
Pcdhb12	27250		#N/A	1.000000E+00
Pcdhb13	27251	0.132586736	14453	6.126502E-02
Pcdhb14	27252	0.047153255	8091.5	5.073070E-01
Pcdhb15	27253	-0.066855996	1239.5	3.469032E-01
Pcdhb16	27254	0.110804467	12778	1.182872E-01
Pcdhb17	27255	0.151956144	15764	3.171344E-02
Pcdhb18	27256	0.022934577	6689	7.471851E-01
Pcdhb19	27257	-0.001606767	5097.5	9.819847E-01
Pcdhb2	27258		#N/A	1.000000E+00
Pcdhb20	27259		#N/A	1.000000E+00
Pcdhb21	27260		#N/A	1.000000E+00
Pcdhb22	27261	0.160985049	16395	2.276975E-02

Spearman Rank correlation analysis performed between Prdm1 and all-expressed genes within the Meredith RNA-seq dataset. Robust Prdm1–associated genes were identified using a cut-off of $p < 0.0005$.

Table S1, Related to Supplemental Figure 3C. Prdm1 associated genes

Pcdhb3	27262		#N/A	1.000000E+00
Pcdhb4	27263	0.113548679	13094	1.093908E-01
Pcdhb5	27264	-0.0429256	2418	5.461543E-01
Pcdhb6	27265		#N/A	1.000000E+00
Pcdhb7	27266	0.031174451	7138	6.612294E-01
Pcdhb8	27267		#N/A	1.000000E+00
Pcdhb9	27268		#N/A	1.000000E+00
Pcdhgc4	27269	0.166094571	16711	1.874521E-02
Pced1a	27270	-0.05867829	1422	4.091750E-01
Pced1b	27271	0.117294936	13480	9.810350E-02
Pcf11	27272	0.098374399	11944	1.657873E-01
Pcgf1	27273	0.11362581	13233	1.091485E-01
Pcgf2	27274	-0.101283855	268	1.535614E-01
Pcgf3	27275	0.13188073	14400	6.267024E-02
Pcgf5	27276	0.080598601	10639	2.565687E-01
Pcgf6	27277	0.068767502	9758	3.332594E-01
Pcid2	27278	-0.00356255	5004	9.600695E-01
Pcif1	27279	0.182053177	17483	9.877729E-03
Pck1	27280	-0.038541788	2501	5.879232E-01
Pck2	27281	0.168100498	16829	1.734294E-02
Pclo	27282	0.047092132	8085	5.078582E-01
Pcm1	27283	0.051566924	8434	4.683428E-01
Pcmt1	27284	0.193031314	17951	6.170734E-03
Pcmtd1	27285	0.204567941	18408	3.664492E-03
Pcmtd2	27286	-0.009728072	4756	8.912548E-01
Pcna	27287	0.141805235	15069	4.517814E-02
Pcna-ps1	27288		#N/A	1.000000E+00
Pcna-ps2	27289	0.117000607	13466	9.895547E-02
Pcnp	27290	0.131645698	14378	6.314388E-02
Pcnt	27291	-0.04375184	2379	5.384485E-01
Pcnx	27292	0.260889106	19635	1.905483E-04
Pcnxl2	27293	0.081322498	10687	2.523077E-01
Pcnxl3	27294	0.076780917	10396	2.798590E-01
Pcnxl4	27295	-0.061048697	1384	3.904792E-01
Pcolce	27296	0.074945048	10235	2.915517E-01
Pcolce2	27297	0.129412947	14266	6.779128E-02
Pcp2	27298	0.098146131	11923	1.667764E-01
Pcp4	27299	-0.111096628	150	1.173141E-01
Pcp4l1	27300	-0.085419739	566	2.291137E-01
Pcsk1	27301	-0.038406171	3065	5.892384E-01
Pcsk1n	27302	-0.038406171	3065	5.892384E-01
Pcsk2	27303	0.245743783	19452	4.523391E-04
Pcsk4	27304		#N/A	1.000000E+00

Spearman Rank correlation analysis performed between Prdm1 and all-expressed genes within the Meredith RNA-seq dataset. Robust Prdm1–associated genes were identified using a cut-off of $p < 0.0005$.

Table S1, Related to Supplemental Figure 3C. Prdm1 associated genes

Pcsk5	27305	0.118821675	13574	9.377709E-02
Pcsk6	27306	-0.031057437	3884	6.624227E-01
Pcsk7	27307	0.143767344	15293	4.225537E-02
Pcsk9	27308	-0.052541134	2151	4.599696E-01
Pctp	27309	-0.022814463	4152	7.484646E-01
Pcx	27310	0.23529091	19274	7.968967E-04
Pcyox1	27311	0.100287056	12078	1.576710E-01
Pcyox1l	27312	0.035263786	7340	6.200824E-01
Pcyt1a	27313	-0.000132956	5278	9.985092E-01
Pcyt1b	27314	0.008898688	5724	9.004760E-01
Pcyt2	27315	0.143930814	15309	4.201920E-02
Pdap1	27316	0.152779286	15811	3.078987E-02
Pdc	27317	-0.054451513	1703	4.437940E-01
Pdcd1	27318	-0.038406171	3065	5.892384E-01
Pdcd10	27319	0.092576883	11556	1.922915E-01
Pdcd11	27320	0.158884092	16218	2.462916E-02
Pdcd1lg2	27321	0.158374179	16193	2.509965E-02
Pdcd2	27322	0.146566686	15450	3.836151E-02
Pdcd2l	27323	0.195737136	18074	5.474332E-03
Pdcd4	27324	0.087235807	11145	2.193303E-01
Pdcd5	27325	0.056961253	9036	4.230418E-01
Pdcd6	27326	0.023468815	6716	7.415025E-01
Pdcd6ip	27327	0.184822204	17625	8.792762E-03
Pdcd7	27328	0.053996158	8865	4.476200E-01
Pdcl	27329	-0.107365479	197	1.302194E-01
Pdcl2	27330	0.070321798	9864	3.224223E-01
Pdcl3	27331	0.106411226	12454	1.336894E-01
Pddc1	27332	0.279522786	19790	6.111435E-05
Pde10a	27333	-0.037429239	3648	5.987521E-01
Pde11a	27334	0.160480447	16363	2.320492E-02
Pde12	27335	0.032727443	7221	6.454745E-01
Pde1a	27336	0.113756032	13242	1.087405E-01
Pde1b	27337	-0.085522094	562	2.285543E-01
Pde1c	27338	0.038574283	7532	5.876082E-01
Pde2a	27339	-0.026173873	4059	7.129498E-01
Pde3a	27340	0.022553861	6661	7.512430E-01
Pde3b	27341	0.126761675	14089	7.366854E-02
Pde4a	27342	0.14874038	15577	3.555076E-02
Pde4b	27343	0.167514837	16798	1.774259E-02
Pde4c	27344	0.08893117	11266	2.104692E-01
Pde4d	27345	0.051263722	8409	4.709658E-01
Pde4dip	27346	0.11434136	13276	1.069212E-01
Pde5a	27347	0.168807658	16866	1.687083E-02

Spearman Rank correlation analysis performed between Prdm1 and all-expressed genes within the Meredith RNA-seq dataset. Robust Prdm1–associated genes were identified using a cut-off of $p < 0.0005$.

Table S1, Related to Supplemental Figure 3C. Prdm1 associated genes

Pde6a	27348	0.021316405	6469	7.644793E-01
Pde6b	27349	-0.066856562	1139	3.468991E-01
Pde6c	27350	0.095282963	11718	1.795591E-01
Pde6d	27351	0.071998862	9998	3.109881E-01
Pde6g	27352		#N/A	1.000000E+00
Pde6h	27353	-0.032132188	3838	6.514951E-01
Pde7a	27354	0.089661977	11318	2.067299E-01
Pde7b	27355	0.053443936	8769	4.522849E-01
Pde8a	27356	0.0880103	11205	2.152499E-01
Pde8b	27357	0.021589227	6559	7.615550E-01
Pde9a	27358	0.225787787	19067	1.305707E-03
Pdf	27359	0.052728522	8550.5	4.583686E-01
Pdgfa	27360	0.111361048	12813	1.164387E-01
Pdgfb	27361	0.100191373	12075	1.580698E-01
Pdgfc	27362	-0.099621258	279	1.604617E-01
Pdgfd	27363	0.0902585	11367	2.037133E-01
Pdgfra	27364	0.142282797	15097	4.445162E-02
Pdgfrb	27365	-0.078921589	655	2.666297E-01
Pdgfrl	27366	-0.025547742	4084	7.195251E-01
Pdha1	27367	0.13108558	14358	6.428445E-02
Pdha2	27368		#N/A	1.000000E+00
Pdhb	27369	0.147854057	15533	3.667505E-02
Pdhx	27370	0.010829602	5815	8.790306E-01
Pdia2	27371		#N/A	1.000000E+00
Pdia3	27372	0.146458809	15443	3.850575E-02
Pdia4	27373	0.20386201	18383	3.786245E-03
Pdia5	27374	-0.043011533	2415	5.453503E-01
Pdia6	27375	0.20210135	18330	4.105959E-03
Pdik1l	27376	0.165992485	16704	1.881914E-02
Pdilt	27377	-0.038406171	3065	5.892384E-01
Pdk1	27378	0.130514873	14324	6.546390E-02
Pdk2	27379	0.123642707	13881	8.110485E-02
Pdk3	27380	0.029965926	7064	6.735941E-01
Pdk4	27381	0.119874377	13658.5	9.088346E-02
Pdlim1	27382	0.077293709	10430	2.766504E-01
Pdlim2	27383	0.105186936	12372.5	1.382448E-01
Pdlim3	27384	0.042955466	7807	5.458748E-01
Pdlim4	27385	0.044712349	7914	5.295591E-01
Pdlim5	27386	0.220438994	18914	1.709115E-03
Pdlim7	27387	0.051248698	8407	4.710960E-01
Pdp1	27388	0.07119186	9926	3.164566E-01
Pdp2	27389	0.07583409	10332	2.858493E-01
Pdpk1	27390	0.188903896	17798	7.385954E-03

Spearman Rank correlation analysis performed between Prdm1 and all-expressed genes within the Meredith RNA-seq dataset. Robust Prdm1–associated genes were identified using a cut-off of $p < 0.0005$.

Table S1, Related to Supplemental Figure 3C. Prdm1 associated genes

Pdpn	27391	-0.017830579	4376	8.021220E-01
PDPN	27392		#N/A	1.000000E+00
Pdpr	27393	0.046025613	8011	5.175260E-01
Pdrg1	27394	0.090592235	11392	2.020396E-01
Pds5a	27395	-0.024792869	4104	7.274798E-01
Pds5b	27396	0.186692654	17705	8.121003E-03
Pdss1	27397	0.091311096	11470	1.984682E-01
Pdss2	27398	0.136748093	14746	5.349930E-02
Pdx1	27399	0.18218536	17494	9.823364E-03
Pdxdc1	27400	0.149836384	15641	3.420097E-02
Pdxk	27401	0.054705359	8908	4.416691E-01
Pdxk-ps	27402	0.056108186	8991	4.300314E-01
Pdxp	27403		#N/A	1.000000E+00
Pdyn	27404	0.064568701	9509	3.636859E-01
Pdzd11	27405	0.057071455	9040	4.221437E-01
Pdzd2	27406	0.085114001	10995	2.307906E-01
Pdzd3	27407	0.019630193	6310.5	7.826254E-01
Pdzd4	27408	0.162153703	16477	2.178882E-02
Pdzd7	27409	-0.050329836	2191	4.790951E-01
Pdzd8	27410	0.345377995	20000	5.478245E-07
Pdzd9	27411		#N/A	1.000000E+00
Pdzk1	27412	-0.066856562	1139	3.468991E-01
Pdzk1ip1	27413	-0.048043883	2232	4.993108E-01
Pdzrn3	27414	0.09922591	12011	1.621362E-01
Pdzrn4	27415	0.152326625	15788	3.129488E-02
Pea15a	27416	-0.012363649	4655	8.620548E-01
Pea15b	27417		#N/A	1.000000E+00
Pear1	27418	0.123668478	13883.5	8.104102E-02
Pebp1	27419	0.126895836	14102	7.336152E-02
Pebp4	27420	0.152634541	15799	3.095060E-02
Pecam1	27421	0.027996803	6973	6.939281E-01
Pecr	27422	0.154075017	15896	2.938252E-02
Pef1	27423	0.106656726	12472	1.327899E-01
Peg10	27424	0.153722066	15875	2.976033E-02
Peg12	27425	0.151630203	15748	3.208561E-02
Peg3	27426	-0.058883132	1420	4.075388E-01
Peli1	27427	0.183015636	17552	9.487889E-03
Peli2	27428	-0.037186713	3654	6.011245E-01
Peli3	27429		#N/A	1.000000E+00
Pelo	27430		#N/A	1.000000E+00
Pelp1	27431	0.032690805	7218	6.458444E-01
Pemt	27432	0.073668441	10109	2.998719E-01
Penk	27433	0.042866343	7801.5	5.467090E-01

Spearman Rank correlation analysis performed between Prdm1 and all-expressed genes within the Meredith RNA-seq dataset. Robust Prdm1–associated genes were identified using a cut-off of $p < 0.0005$.

Table S1, Related to Supplemental Figure 3C. Prdm1 associated genes

Peo1	27434	0.048612492	8189	4.942407E-01
Pepd	27435	0.158116014	16181	2.534078E-02
Per1	27436	-0.009674673	4759	8.918481E-01
Per2	27437	-0.07998511	643	2.602185E-01
Per3	27438		#N/A	1.000000E+00
Perp	27439	0.171423953	16993	1.522011E-02
Pes1	27440	0.065902832	9583	3.538365E-01
Pet100	27441	-0.021378165	4196	7.638170E-01
Pet112	27442	0.119743859	13645	9.123830E-02
Pet2	27443	-0.054451513	1703	4.437940E-01
Pex1	27444	0.116993221	13465	9.897692E-02
Pex10	27445	-0.010461915	4730	8.831080E-01
Pex11a	27446	0.003168145	5486	9.644870E-01
Pex11b	27447	0.174930144	17161	1.323017E-02
Pex11c	27448	0.033695764	7280	6.357298E-01
Pex12	27449	0.024792694	6807	7.274816E-01
Pex13	27450	0.248073782	19482	3.973622E-04
Pex14	27451	0.060651688	9242	3.935740E-01
Pex16	27452	0.018471444	6246	7.951643E-01
Pex19	27453	0.183354334	17563	9.353976E-03
Pex2	27454	-0.009073732	4780	8.985286E-01
Pex26	27455	0.125387764	13997	7.687308E-02
Pex3	27456	0.133042346	14478	6.037201E-02
Pex5	27457	-0.020456351	4222	7.737194E-01
Pex5l	27458	-0.076214541	858	2.834320E-01
Pex6	27459		#N/A	1.000000E+00
Pex7	27460	0.023887132	6741	7.370628E-01
Pf4	27461	-0.10591213	203	1.355323E-01
Pfas	27462	0.079203071	10542	2.649224E-01
Pfdn1	27463	0.070588311	9880	3.205872E-01
Pfdn2	27464	0.208773825	18543	3.009523E-03
Pfdn4	27465	0.090599597	11393	2.020028E-01
Pfdn5	27466	0.176879412	17262	1.222573E-02
Pfkfb1	27467	0.226092151	19074	1.285615E-03
Pfkfb2	27468	0.119267644	13605	9.254239E-02
Pfkfb3	27469	0.114423113	13286	1.066690E-01
Pfkfb4	27470	0.130288736	14310	6.593612E-02
Pfkl	27471	0.13677073	14747	5.345940E-02
Pfkm	27472	0.075776249	10328	2.862180E-01
Pfkp	27473	0.186809393	17712	8.080630E-03
Pfn1	27474	0.116633797	13443	1.000254E-01
Pfn2	27475	0.142664416	15131	4.387812E-02
Pfn3	27476		#N/A	1.000000E+00

Spearman Rank correlation analysis performed between Prdm1 and all-expressed genes within the Meredith RNA-seq dataset. Robust Prdm1–associated genes were identified using a cut-off of $p < 0.0005$.

Pfn4	27477	0.132773093	14462.5	6.089845E-02
Pfpl	27478		#N/A	1.000000E+00
Pga5	27479	-0.045438257	2313.5	5.228904E-01
Pgam1	27480	0.150875013	15708	3.296220E-02
Pgam1-ps1	27481		#N/A	1.000000E+00
Pgam1-ps2	27482	0.098991981	11994	1.631332E-01
Pgam2	27483	-0.033667949	3760	6.360088E-01
Pgam5	27484	0.046198091	8027	5.159562E-01
Pgap1	27485	0.000779905	5350	9.912550E-01
Pgap2	27486	0.116287506	13415	1.010439E-01
Pgap3	27487	0.022053982	6592	7.565814E-01
Pgbd1	27488	-0.011444739	4690	8.722164E-01
Pgbd5	27489	-0.004937246	4940	9.446824E-01
Pgc	27490	-0.02966754	3943	6.766606E-01
Pgd	27491	0.009460477	5748	8.942284E-01
Pgf	27492		#N/A	1.000000E+00
Pggt1b	27493	-0.159587542	14	2.399255E-02
Pgk1	27494	0.214223981	18723	2.318796E-03
Pgk1-rs7	27495	0.138976867	14886	4.968742E-02
Pgk2	27496	-0.038406171	3065	5.892384E-01
Pgls	27497	0.010928252	5822	8.779372E-01
Pglyrp1	27498	0.085483388	11020	2.287657E-01
Pglyrp2	27499	0.142675325	15132	4.386182E-02
Pglyrp3	27500	0.096074797	11780	1.759537E-01
Pglyrp4	27501		#N/A	1.000000E+00
Pgm1	27502	-0.060823433	1388	3.922334E-01
Pgm2	27503	0.14336275	15157	4.284470E-02
Pgm2l1	27504	0.089360309	11295	2.082676E-01
Pgm3	27505	0.014147879	5998	8.423902E-01
Pgm5	27506	-0.116049802	131	1.017478E-01
Pgp	27507	0.122875139	13834	8.302470E-02
Pgpep1	27508	0.119750823	13646	9.121934E-02
Pgpep1l	27509	-0.038406171	3065	5.892384E-01
Pgr	27510	-0.033626273	3762	6.364270E-01
Pgr15l	27511	-0.066855996	1239.5	3.469032E-01
Pgrmc1	27512	0.106137743	12434	1.346968E-01
Pgrmc2	27513	0.026077149	6876	7.139641E-01
Pgs1	27514	-0.009840396	4752	8.900071E-01
Phactr1	27515	0.055290829	8945	4.367905E-01
Phactr2	27516	0.138310406	14848	5.080282E-02
Phactr3	27517	0.050243059	8321	4.798544E-01
Phactr4	27518	0.053270298	8762	4.537573E-01
Phax	27519	-0.040370973	2466	5.703171E-01

Spearman Rank correlation analysis performed between Prdm1 and all-expressed genes within the Meredith RNA-seq dataset. Robust Prdm1–associated genes were identified using a cut-off of $p < 0.0005$.

Table S1, Related to Supplemental Figure 3C. Prdm1 associated genes

Phb	27520	0.089144332	11283	2.093736E-01
Phb2	27521	0.145515549	15379	3.978658E-02
Phc1	27522	-0.021733121	4182	7.600141E-01
Phc2	27523	0.2467173	19468	4.285637E-04
Phc3	27524	0.073067552	10069	3.038420E-01
Phex	27525	0.294261493	19882	2.342911E-05
Phf1	27526	0.206901986	18487	3.286675E-03
Phf10	27527	0.161073966	16429	2.269380E-02
Phf11a	27528	0.157879924	16169	2.556304E-02
Phf11b	27529	0.002881322	5465	9.677003E-01
Phf11c	27530	0.079642816	10570	2.622702E-01
Phf11d	27531	0.055233465	8939	4.372672E-01
Phf12	27532	0.062185813	9336	3.816964E-01
Phf13	27533	0.149876311	15643	3.415263E-02
Phf14	27534	0.146301278	15434	3.871721E-02
Phf15	27535	-0.009394627	4770	8.949604E-01
Phf16	27536	0.241784346	19393	5.621799E-04
Phf17	27537	0.189464293	17821	7.209342E-03
Phf19	27538	0.096503632	11808	1.740237E-01
Phf2	27539	0.084804423	10972	2.324974E-01
Phf20	27540	0.06131026	9284	3.884482E-01
Phf20l1	27541	0.042837937	7798	5.469750E-01
Phf21a	27542	0.058277796	9107	4.123852E-01
Phf21b	27543	0.148277991	15545	3.613360E-02
Phf23	27544	0.091285615	11465	1.985940E-01
Phf3	27545	0.218769605	18867	1.856579E-03
Phf5a	27546	0.008358556	5697	9.064884E-01
Phf6	27547	0.114784021	13312	1.055613E-01
Phf7	27548	-0.080310383	638	2.582789E-01
Phf8	27549	0.052715494	8523	4.584798E-01
Phgdh	27550	0.083714566	10895	2.385766E-01
Phgr1	27551		#N/A	1.000000E+00
Phip	27552	0.227267362	19084	1.210666E-03
Phka1	27553	-0.077927033	663	2.727220E-01
Phka2	27554	0.13767719	14801	5.188179E-02
Phkb	27555	0.098954896	11992	1.632917E-01
Phkg1	27556	0.052192227	8486	4.629588E-01
Phkg2	27557	0.220923085	18930	1.668399E-03
Phlda1	27558	0.051630985	8441	4.677897E-01
Phlda2	27559	0.020434681	6357	7.739526E-01
Phlda3	27560	-0.002420902	5052	9.728595E-01
Phldb1	27561	0.068766502	9757	3.332665E-01
Phldb2	27562	0.111607815	12826	1.156264E-01

Spearman Rank correlation analysis performed between Prdm1 and all-expressed genes within the Meredith RNA-seq dataset. Robust Prdm1–associated genes were identified using a cut-off of $p < 0.0005$.

Table S1, Related to Supplemental Figure 3C. Prdm1 associated genes

Phldb3	27563	-0.016731569	4428.5	8.140896E-01
Phlpp1	27564	0.203791152	18381	3.798666E-03
Phlpp2	27565	0.157593641	16131	2.583479E-02
Phospho1	27566		#N/A	1.000000E+00
Phospho2	27567	0.029027862	7017	6.832525E-01
Phox2a	27568	-0.054451513	1703	4.437940E-01
Phox2b	27569	0.020875719	6412.5	7.692098E-01
Phpt1	27570	0.100792659	12109	1.555763E-01
Phrf1	27571	0.00493206	5562	9.447404E-01
Phtf1	27572	0.058355486	9112	4.117613E-01
Phtf2	27573	0.195292912	18057	5.583598E-03
Phxr2	27574		#N/A	1.000000E+00
Phxr4	27575	-0.038289346	3626	5.903724E-01
Phyh	27576	0.136812093	14752	5.338656E-02
Phyhd1	27577	-0.045915975	2294	5.185252E-01
Phyhip	27578	-0.095280123	319	1.795721E-01
Phyhipl	27579	0.238228518	19333	6.813313E-04
Pi15	27580	0.107390077	12518	1.301308E-01
Pi16	27581	-0.086746694	532	2.219355E-01
Pi4k2a	27582	0.033045184	7241	6.422701E-01
Pi4k2b	27583	0.017700986	6213	8.035308E-01
Pi4ka	27584	0.20911764	18552	2.960979E-03
Pi4kb	27585	0.099495072	12030	1.609948E-01
Pianp	27586	0.03553939	7354	6.173494E-01
Pias1	27587	0.004113202	5522	9.539039E-01
Pias2	27588	-0.009075511	4778	8.985089E-01
Pias3	27589	-0.001912287	5079	9.785600E-01
Pias4	27590	0.00962912	5756	8.923542E-01
Pibf1	27591	0.164896211	16649	1.962908E-02
Picalm	27592	0.282955087	19816	4.911776E-05
Pick1	27593	-0.076925121	843	2.789542E-01
Pid1	27594	0.112981753	12928	1.111843E-01
Piezo1	27595	0.210991948	18611	2.708592E-03
Piezo2	27596	0.233490221	19240	8.763945E-04
Pif1	27597	0.122214285	13798	8.470675E-02
Pifo	27598	0.029182643	7028	6.816553E-01
Piga	27599	0.21220837	18659	2.555404E-03
Pigb	27600	0.129415128	14268	6.778661E-02
Pigc	27601	0.006933234	5627	9.223791E-01
Pigf	27602	0.149353263	15611	3.479050E-02
Pigg	27603	0.138029285	14825	5.127951E-02
Pigh	27604	0.16072698	16377	2.299142E-02
Pigk	27605	0.212261845	18663	2.548854E-03

Spearman Rank correlation analysis performed between Prdm1 and all-expressed genes within the Meredith RNA-seq dataset. Robust Prdm1–associated genes were identified using a cut-off of $p < 0.0005$.

Table S1, Related to Supplemental Figure 3C. Prdm1 associated genes

Pigl	27606	0.099014893	11995.5	1.630354E-01
Pigm	27607	0.091474941	11483	1.976607E-01
Pign	27608	0.228204186	19101	1.153813E-03
Pigo	27609	0.067541095	9678	3.419731E-01
Pigp	27610	-0.05389331	2123	4.484868E-01
Pigq	27611	0.04077528	7683	5.664595E-01
Pigr	27612	0.115926205	13390	1.021153E-01
Pigs	27613	0.062798329	9380	3.770158E-01
Pigt	27614	0.135297055	14646	5.610894E-02
Pigu	27615	0.23265108	19223	9.158794E-04
Pigv	27616	0.045817326	7991	5.194251E-01
Pigw	27617	0.097984923	11913	1.674776E-01
Pigx	27618	0.124952935	13966	7.791046E-02
Pigyl	27619	0.166348113	16738	1.856270E-02
Pigz	27620	0.021189106	6447	7.658448E-01
Pih1d1	27621	0.232021386	19213	9.465763E-04
Pih1d2	27622	0.214906769	18735	2.243277E-03
Pih1d3	27623		#N/A	1.000000E+00
Pik3ap1	27624	0.039874832	7634	5.750678E-01
Pik3c2a	27625	0.13106682	14356	6.432295E-02
Pik3c2b	27626	0.135339986	14649	5.603025E-02
Pik3c2g	27627	0.039795988	7617	5.758244E-01
Pik3c3	27628	0.07631545	10370	2.827932E-01
Pik3ca	27629	0.069389219	9805	3.288969E-01
Pik3cb	27630	0.147644825	15515	3.694480E-02
Pik3cd	27631	0.017389687	6196	8.069177E-01
Pik3cg	27632	0.09698353	11835	1.718825E-01
Pik3ip1	27633	0.015172357	6079	8.311422E-01
Pik3r1	27634	0.125029427	13970	7.772716E-02
Pik3r2	27635	-0.073416768	915	3.015305E-01
Pik3r3	27636	0.008265624	5690	9.075235E-01
Pik3r4	27637	0.115019218	13320	1.048443E-01
Pik3r5	27638	0.054567933	8896	4.428187E-01
Pik3r6	27639	-0.046386076	2276	5.142479E-01
Pikfyve	27640	0.116904464	13457	9.923503E-02
Pilra	27641	0.135188292	14640	5.630872E-02
Pilrb1	27642	0.18218536	17494	9.823364E-03
Pilrb2	27643	0.053805701	8820	4.492258E-01
Pim1	27644	0.072406353	10022	3.082504E-01
Pim2	27645	0.080678119	10646	2.560982E-01
Pim3	27646	0.048580179	8188	4.945281E-01
Pin1	27647	0.258412876	19605	2.202731E-04
Pin1rt1	27648		#N/A	1.000000E+00

Spearman Rank correlation analysis performed between Prdm1 and all-expressed genes within the Meredith RNA-seq dataset. Robust Prdm1–associated genes were identified using a cut-off of $p < 0.0005$.

Table S1, Related to Supplemental Figure 3C. Prdm1 associated genes

Pin4	27649	0.180975359	17447	1.033105E-02
Pink1	27650	0.076785101	10397	2.798327E-01
Pinlyp	27651	0.104886868	12349	1.393792E-01
Pinx1	27652	0.088889726	11263	2.106827E-01
Pip	27653		#N/A	1.000000E+00
Pip4k2a	27654	0.024576452	6792	7.297657E-01
Pip4k2b	27655	0.180702437	17437	1.044871E-02
Pip4k2c	27656	0.184016466	17592	9.097010E-03
Pip5k1a	27657	0.070294942	9858	3.226076E-01
Pip5k1b	27658	0.132554562	14450	6.132849E-02
Pip5k1c	27659	0.086254829	11069	2.245774E-01
Pip5kl1	27660	0.230217498	19157	1.039825E-03
Pipox	27661	-0.066856562	1139	3.468991E-01
Pir	27662	0.2251149	19045	1.351150E-03
Pira2	27663	-0.07385697	908	2.986333E-01
Pirt	27664	0.092419183	11542	1.930533E-01
Pisd	27665	0.081722187	10721	2.499761E-01
Pisd-ps1	27666	-0.003004465	5025	9.663207E-01
Pisd-ps2	27667	0.052728522	8550.5	4.583686E-01
Pithd1	27668	0.267858062	19705	1.257431E-04
Pitpna	27669	0.115806675	13381	1.024717E-01
Pitpnb	27670	0.0269219	6927	7.051221E-01
Pitpnc1	27671	0.169759604	16914	1.625299E-02
PITPNM1	27672		#N/A	1.000000E+00
Pitpnm1	27673	0.077498219	10443	2.753777E-01
Pitpnm2	27674	0.32041548	19968	3.737087E-06
Pitpnm3	27675	-0.021524641	4190.5	7.622470E-01
Pitrm1	27676	0.140730529	15006	4.684948E-02
Pitx1	27677	0.051686392	8449	4.673115E-01
Pitx2	27678	0.068998677	9777	3.316330E-01
Pitx3	27679	0.152649014	15800	3.093450E-02
Piwil1	27680	0.083810603	10907.5	2.380365E-01
Piwil2	27681	0.012716044	5914	8.581638E-01
Piwil4	27682	0.16703704	16772	1.807456E-02
Pja1	27683	0.091223567	11459	1.989006E-01
Pja2	27684	0.182234961	17501	9.803031E-03
Pkd1	27685	0.344136151	19999	6.051666E-07
Pkd1l1	27686	0.03146971	7158	6.582223E-01
Pkd1l2	27687	0.011659269	5861	8.698421E-01
Pkd1l3	27688	0.118010621	13527	9.605611E-02
Pkd2	27689	0.060875867	9255	3.918246E-01
Pkd2l1	27690	0.008917786	5726	9.002635E-01
Pkd2l2	27691	0.058942688	9156	4.070638E-01

Spearman Rank correlation analysis performed between Prdm1 and all-expressed genes within the Meredith RNA-seq dataset. Robust Prdm1–associated genes were identified using a cut-off of $p < 0.0005$.

Table S1, Related to Supplemental Figure 3C. Prdm1 associated genes

Pkdcc	27692	0.046391636	8040	5.141975E-01
Pkdrej	27693		#N/A	1.000000E+00
Pkhd1	27694	0.019413932	6300	7.849614E-01
Pkhd1l1	27695	0.143899803	15306	4.206391E-02
Pkia	27696	0.042770897	7787	5.476031E-01
Pkib	27697	0.131583614	14375	6.326947E-02
Pkig	27698	0.031522528	7161	6.576849E-01
Pklr	27699	0.162102339	16472	2.183116E-02
Pkm	27700	0.128014606	14191	7.084150E-02
Pkmyt1	27701	0.20328713	18367	3.888078E-03
Pkn1	27702	0.137483302	14787	5.221595E-02
Pkn2	27703	0.084522841	10955	2.340575E-01
Pkn3	27704	0.001131473	5367	9.873133E-01
Pknox1	27705	0.170390717	16952	1.585435E-02
Pknox2	27706	0.142513264	15114	4.410453E-02
Pkp1	27707	0.0581391	9097	4.135003E-01
Pkp2	27708	0.059824789	9201	4.000672E-01
Pkp3	27709	0.223812212	19012	1.443266E-03
Pkp4	27710	0.102592526	12217	1.482893E-01
Pla1a	27711	-0.007805855	4828	9.126464E-01
Pla2g10	27712	0.083770179	10902	2.382638E-01
Pla2g12a	27713	0.197098731	18126	5.151254E-03
Pla2g12b	27714	0.136925146	14760	5.318788E-02
Pla2g15	27715	-0.014369456	4550	8.399547E-01
Pla2g16	27716	0.032552244	7208	6.472441E-01
Pla2g1b	27717	-0.117569104	125	9.731512E-02
Pla2g2a	27718		#N/A	1.000000E+00
Pla2g2c	27719		#N/A	1.000000E+00
Pla2g2d	27720	0.163196967	16553	2.094420E-02
Pla2g2e	27721	-0.054450825	1984	4.437997E-01
Pla2g2f	27722	0.114359632	13278	1.068648E-01
Pla2g3	27723	-0.065408072	1318	3.574695E-01
Pla2g4a	27724	0.091291896	11469	1.985630E-01
Pla2g4b	27725	0.093627918	11623	1.872702E-01
Pla2g4c	27726	0.036642966	7411	6.064589E-01
Pla2g4d	27727	0.097663348	11879	1.688829E-01
Pla2g4e	27728	0.115122569	13331	1.045305E-01
Pla2g4f	27729	0.155976	16036	2.741735E-02
Pla2g5	27730	-0.033388476	3771	6.388155E-01
Pla2g6	27731	0.206969697	18492	3.276261E-03
Pla2g7	27732	-0.047281121	2254	5.061549E-01
Pla2r1	27733	0.107833633	12543	1.285426E-01
Plaa	27734	0.087669175	11177	2.170404E-01

Spearman Rank correlation analysis performed between Prdm1 and all-expressed genes within the Meredith RNA-seq dataset. Robust Prdm1–associated genes were identified using a cut-off of $p < 0.0005$.

Table S1, Related to Supplemental Figure 3C. Prdm1 associated genes

Plac1	27735	0.087452563	11160	2.181828E-01
Plac1l	27736	0.132006377	14407	6.241824E-02
Plac8	27737	0.108198376	12563	1.272477E-01
Plac8l1	27738	0.023067505	6695.5	7.457699E-01
Plac9a	27739	-0.038406171	3065	5.892384E-01
Plac9b	27740		#N/A	1.000000E+00
Plag1	27741	-0.055797911	1496	4.325901E-01
Plagl1	27742	0.173717288	17107	1.389106E-02
Plagl2	27743	0.172974591	17080	1.430989E-02
Plat	27744	0.036157999	7389	6.112343E-01
Plau	27745	0.138712189	14867	5.012793E-02
Plaur	27746	0.045086937	7947	5.261125E-01
Plb1	27747	0.095360157	11727	1.792052E-01
Plbd1	27748	0.022112252	6594	7.559585E-01
Plbd2	27749	-0.000444962	5260	9.950106E-01
Plcb1	27750	0.020457677	6368	7.737051E-01
Plcb2	27751	0.135386777	14654	5.594458E-02
Plcb3	27752	0.113211327	12938	1.104552E-01
Plcb4	27753	-0.006076276	4876	9.319484E-01
Plcd1	27754	-0.032167478	3837	6.511375E-01
Plcd3	27755	0.050540156	8340	4.772577E-01
Plcd4	27756	0.111613846	12828	1.156066E-01
Plce1	27757	0.218441959	18854	1.886849E-03
Plcg1	27758	0.147012738	15479	3.776992E-02
Plcg2	27759	0.134956054	14617	5.673725E-02
Plch1	27760	0.035088544	7334	6.218229E-01
Plch2	27761	0.026330867	6889	7.113044E-01
Plcl1	27762	0.282995013	19818	4.899224E-05
Plcl2	27763	0.200228149	18250	4.472618E-03
Plcxd1	27764	0.024065122	6758	7.351763E-01
Plcxd2	27765	0.066476825	9619	3.496509E-01
Plcxd3	27766	0.028655562	7001	6.871002E-01
Plcz1	27767	-0.010686107	4717	8.806215E-01
Pld1	27768	0.097909021	11909	1.678085E-01
Pld2	27769	0.068556473	9744	3.347486E-01
Pld3	27770	0.314045779	19949	5.939320E-06
Pld4	27771	0.132339401	14435	6.175434E-02
Pld5	27772	0.124078559	13914	8.003072E-02
Pld6	27773	0.036086209	7386	6.119426E-01
Pldi	27774		#N/A	1.000000E+00
Plec	27775	0.162797518	16516	2.126419E-02
Plek	27776	0.016738412	6160	8.140150E-01
Plek2	27777	0.097884258	11905	1.679166E-01

Spearman Rank correlation analysis performed between Prdm1 and all-expressed genes within the Meredith RNA-seq dataset. Robust Prdm1–associated genes were identified using a cut-off of $p < 0.0005$.

Table S1, Related to Supplemental Figure 3C. Prdm1 associated genes

Plekha1	27778	0.065186774	9544	3.591020E-01
Plekha2	27779	0.105178926	12370	1.382750E-01
Plekha3	27780	0.249258625	19499	3.718459E-04
Plekha4	27781	-0.097029663	301	1.716777E-01
Plekha5	27782	-0.030601149	3908	6.670838E-01
Plekha6	27783	-0.065109051	1324	3.596764E-01
Plekha7	27784	0.062554335	9362	3.788761E-01
Plekha8	27785	-0.036487208	3672	6.079908E-01
Plekhb1	27786	0.051571404	8436.5	4.683041E-01
Plekhb2	27787	0.011966959	5872	8.664389E-01
Plekhd1	27788	-0.117606715	112	9.720737E-02
Plekhf1	27789	-0.031503567	3863.5	6.578778E-01
Plekhf2	27790	0.200746561	18275	4.368306E-03
Plekhg1	27791	0.114486595	13289	1.064735E-01
Plekhg2	27792	0.058532287	9128	4.103436E-01
Plekhg3	27793	0.302183694	19912	1.368599E-05
Plekhg4	27794	0.053805701	8820	4.492258E-01
Plekhg5	27795	0.035522141	7351	6.175203E-01
Plekhg6	27796	0.020875719	6412.5	7.692098E-01
Plekhh1	27797	-0.044731287	2350	5.293846E-01
Plekhh2	27798	0.088441898	11236	2.129997E-01
Plekhh3	27799	0.075863285	10336	2.856633E-01
Plekhj1	27800	0.049725493	8272	4.843961E-01
Plekhm1	27801	-0.031074696	3883	6.622466E-01
Plekhm2	27802	-0.065496336	1316	3.568197E-01
Plekhm3	27803	0.12268151	13827	8.351473E-02
Plekhn1	27804	-0.008866063	4791	9.008390E-01
Plekho1	27805	0.007430066	5653	9.168362E-01
Plekho2	27806	0.012872663	5922	8.564356E-01
Plekhs1	27807	0.07920454	10543	2.649135E-01
Plg	27808	-0.066856562	1139	3.468991E-01
Plgrkt	27809	0.291352513	19869	2.843004E-05
Plin1	27810	0.053267784	8685	4.537786E-01
Plin2	27811	0.119367147	13614	9.226869E-02
Plin3	27812	0.105996742	12423	1.352185E-01
Plin4	27813	-0.066855996	1239.5	3.469032E-01
Plin5	27814	0.049855249	8285.5	4.832553E-01
Plk-ps1	27815		#N/A	1.000000E+00
Plk1	27816	0.11898765	13585	9.331605E-02
Plk1s1	27817	0.110084701	12721	1.207114E-01
Plk2	27818	-0.01787551	4374	8.016337E-01
Plk3	27819	0.098525555	11957	1.651347E-01
Plk4	27820	0.122492246	13818	8.399596E-02

Spearman Rank correlation analysis performed between Prdm1 and all-expressed genes within the Meredith RNA-seq dataset. Robust Prdm1–associated genes were identified using a cut-off of $p < 0.0005$.

Table S1, Related to Supplemental Figure 3C. Prdm1 associated genes

Plk5	27821		#N/A	1.000000E+00
Pllp	27822		#N/A	1.000000E+00
Pln	27823	0.170247144	16942	1.594428E-02
Plod1	27824	0.140480215	14987	4.724609E-02
Plod2	27825	0.149895787	15646	3.412907E-02
Plod3	27826	0.067850192	9693	3.397634E-01
Plp1	27827	-0.014716102	4528	8.361474E-01
Plp2	27828	0.165467746	16679	1.920313E-02
Plrg1	27829	0.108604266	12590	1.258187E-01
Pls1	27830	0.015240582	6082	8.303943E-01
Pls3	27831	0.205991146	18458	3.429712E-03
Plscr1	27832	-0.004753109	4950	9.467424E-01
Plscr2	27833	0.010600707	5795.5	8.815685E-01
Plscr3	27834	0.131972618	14405	6.248587E-02
Plscr4	27835	-0.054451513	1703	4.437940E-01
Plscr5	27836	-0.001697596	5090	9.809665E-01
Pltp	27837	0.16294058	16542	2.114910E-02
Plvap	27838	0.201310137	18303	4.257394E-03
Plxdc1	27839	0.061251404	9277	3.889047E-01
Plxdc2	27840	0.265842401	19682	1.419731E-04
Plxna1	27841	0.002535693	5443	9.715731E-01
Plxna2	27842	0.05471743	8909	4.415682E-01
Plxna3	27843	0.122047226	13789	8.513627E-02
Plxna4	27844	0.09093809	11428	2.003156E-01
Plxnb1	27845	0.038898629	7574	5.844690E-01
Plxnb2	27846	0.095944251	11767	1.765444E-01
Plxnb3	27847		#N/A	1.000000E+00
Plxnc1	27848	0.07071152	9889	3.197412E-01
Plxnd1	27849	0.025982367	6870	7.149586E-01
Pm20d1	27850	-0.012497807	4647	8.605731E-01
Pm20d2	27851		#N/A	1.000000E+00
Pmaip1	27852	0.107784083	12539	1.287192E-01
Pmch	27853	0.020875719	6412.5	7.692098E-01
Pmel	27854	0.149237896	15605	3.493255E-02
Pmepa1	27855	0.083753993	10901	2.383548E-01
Pmf1	27856	0.121542795	13756	8.644379E-02
Pmfbp1	27857	0.163832537	16586	2.044364E-02
Pml	27858	0.139431045	14917	4.893904E-02
Pmm1	27859	0.230216116	19154	1.039900E-03
Pmm2	27860	0.140634932	14995	4.700062E-02
Pmp2	27861	-0.077393517	797	2.760287E-01
Pmp22	27862	0.020524702	6372	7.729838E-01
Pmpca	27863	0.174399454	17140	1.351587E-02

Spearman Rank correlation analysis performed between Prdm1 and all-expressed genes within the Meredith RNA-seq dataset. Robust Prdm1–associated genes were identified using a cut-off of $p < 0.0005$.

Table S1, Related to Supplemental Figure 3C. Prdm1 associated genes

Pmpcb	27864	0.153526478	15867	2.997147E-02
Pms1	27865	0.087308281	11149	2.189462E-01
Pms2	27866	0.170544918	16957	1.575825E-02
Pmvk	27867	0.250486036	19512	3.470224E-04
Pnck	27868	0.000307056	5327	9.965570E-01
Pnkd	27869	-0.046984452	2260	5.088300E-01
Pnkp	27870	0.029847904	7059	6.748063E-01
Pnldc1	27871	0.021317126	6513.5	7.644715E-01
Pnlip	27872	-0.000841282	5208.5	9.905669E-01
Pnliprp1	27873	-0.032762514	3799	6.451205E-01
Pnliprp2	27874		#N/A	1.000000E+00
Pnma1	27875	-0.054450825	1984	4.437997E-01
Pnma2	27876	0.074945048	10235	2.915517E-01
Pnma3	27877		#N/A	1.000000E+00
Pnma5	27878		#N/A	1.000000E+00
Pnmal1	27879		#N/A	1.000000E+00
Pnmal2	27880	0.065463945	9559	3.570580E-01
Pnmt	27881	-0.054450825	1984	4.437997E-01
Pnn	27882	0.230353573	19174	1.032507E-03
Pno1	27883	0.130493991	14321	6.550739E-02
Pnoc	27884	0.104507593	12326	1.408232E-01
Pnp	27885	0.077772142	10458	2.736792E-01
Pnp2	27886		#N/A	1.000000E+00
Pnpla1	27887	0.034640314	7318	6.262842E-01
Pnpla2	27888	0.076732501	10395	2.801633E-01
Pnpla3	27889	0.062475138	9353	3.794811E-01
Pnpla5	27890		#N/A	1.000000E+00
Pnpla6	27891	0.045981209	8004	5.179306E-01
Pnpla7	27892	-0.054776933	1528	4.410711E-01
Pnpla8	27893	0.130316738	14314	6.587749E-02
Pnpo	27894	-0.083183554	600.5	2.415786E-01
Pnpt1	27895	0.313413979	19946	6.215029E-06
Pnrc1	27896	0.251835985	19526	3.215020E-04
Pnrc2	27897	0.027061352	6934	7.036662E-01
Poc1a	27898	0.117400197	13484	9.780022E-02
Poc1b	27899	0.044826125	7923	5.285111E-01
Poc5	27900	0.156696184	16092	2.670282E-02
Podn	27901	0.087479576	11165	2.180401E-01
Podnl1	27902	-0.095267438	357.5	1.796303E-01
Podxl	27903	0.049167274	8230	4.893204E-01
Podxl2	27904	-0.004526355	4961.5	9.492796E-01
Pof1b	27905	0.235728134	19282	7.786234E-04
Pofut1	27906	0.024584271	6793	7.296831E-01

Spearman Rank correlation analysis performed between Prdm1 and all-expressed genes within the Meredith RNA-seq dataset. Robust Prdm1–associated genes were identified using a cut-off of $p < 0.0005$.

Table S1, Related to Supplemental Figure 3C. Prdm1 associated genes

Pofut2	27907	0.21186487	18648	2.597845E-03
Pogk	27908	2.68564E-06	5291	9.999699E-01
Poglut1	27909	0.128670298	14224	6.939761E-02
Pogz	27910	0.110755243	12777	1.184518E-01
Pola1	27911	-0.08634773	553	2.240767E-01
Pola2	27912	0.101014705	12119	1.546630E-01
Polb	27913	0.158171429	16184	2.528886E-02
Pold1	27914	0.14248561	15111	4.414605E-02
Pold2	27915	0.116718638	13451	9.977715E-02
Pold3	27916	-0.001411797	5124	9.841705E-01
Pold4	27917	0.058472089	9123	4.108259E-01
POLD4	27918		#N/A	1.000000E+00
Poldip2	27919	0.211384368	18628	2.658287E-03
Poldip3	27920	0.08984439	11334	2.058041E-01
Pole	27921	0.102332746	12201	1.493248E-01
Pole2	27922	0.085644805	11028	2.278848E-01
Pole3	27923	0.15257252	15797	3.101968E-02
Pole4	27924	0.209599656	18566	2.894115E-03
Polg	27925	0.017934237	6219	8.009955E-01
Polg2	27926	0.011843567	5870	8.678034E-01
Polh	27927	-0.012884965	4628	8.562999E-01
Poli	27928	0.022198316	6629.5	7.550388E-01
Polk	27929	0.108505474	12582	1.261654E-01
Poll	27930	0.199024034	18198	4.723595E-03
Polm	27931	0.032849187	7227	6.442460E-01
Poln	27932	0.246161793	19457	4.419845E-04
Polq	27933	0.029161313	7025	6.818753E-01
Polr1a	27934	0.027761149	6963	6.963766E-01
Polr1b	27935	0.222873035	18985	1.513203E-03
Polr1c	27936	-0.03309535	3781	6.417648E-01
Polr1d	27937	0.115897462	13386	1.022009E-01
Polr1e	27938	0.165640652	16684	1.907586E-02
Polr2a	27939	0.178677256	17334	1.135940E-02
Polr2b	27940	0.148379004	15553	3.600559E-02
Polr2c	27941	-0.013104641	4620	8.538770E-01
Polr2d	27942	0.239004183	19349	6.535100E-04
Polr2e	27943	0.023944157	6745	7.364582E-01
Polr2f	27944	0.075903709	10342	2.854059E-01
Polr2g	27945	0.210273052	18584	2.802994E-03
Polr2h	27946	0.181745073	17474	1.000548E-02
Polr2i	27947	0.164992325	16657	1.955688E-02
Polr2j	27948	0.067497817	9673	3.422832E-01
Polr2k	27949	0.13917037	14900	4.936742E-02

Spearman Rank correlation analysis performed between Prdm1 and all-expressed genes within the Meredith RNA-seq dataset. Robust Prdm1–associated genes were identified using a cut-off of $p < 0.0005$.

Table S1, Related to Supplemental Figure 3C. Prdm1 associated genes

Polr2l	27950	0.19524355	18054	5.595859E-03
Polr2m	27951	0.130964999	14350	6.453219E-02
Polr3a	27952	0.158771935	16215	2.473199E-02
Polr3b	27953	0.188493473	17780	7.517733E-03
Polr3c	27954	0.151995471	15769	3.166879E-02
Polr3d	27955	0.315417991	19956	5.379984E-06
Polr3e	27956	-0.014161907	4563	8.422360E-01
Polr3f	27957	0.156107413	16045	2.728577E-02
Polr3g	27958	0.073449007	10091	3.013177E-01
Polr3gl	27959	0.009348363	5739	8.954747E-01
Polr3h	27960	0.023763613	6737	7.383728E-01
Polr3k	27961	0.093474638	11612	1.879964E-01
Polrmt	27962	0.057425648	9057	4.192646E-01
Pom121	27963	0.141149417	15027	4.619200E-02
Pom121l12	27964		#N/A	1.000000E+00
Pom121l2	27965		#N/A	1.000000E+00
Pomc	27966	0.164701303	16637	1.977620E-02
Pomgnt1	27967	0.022216115	6645	7.548486E-01
Pomp	27968	0.136791427	14751	5.342294E-02
Pomt1	27969	0.022198129	6607.5	7.550408E-01
Pomt2	27970	0.037989222	7482	5.932903E-01
Pon1	27971	0.134200998	14569.5	5.814916E-02
Pon2	27972	0.130293602	14311	6.592593E-02
Pon3	27973	0.132170572	14422	6.209018E-02
Pop1	27974	0.159054884	16227	2.447327E-02
Pop4	27975	0.219669069	18891	1.775744E-03
Pop5	27976	0.175212747	17176	1.308020E-02
Pop7	27977	0.172925187	17073	1.433814E-02
Popdc2	27978	0.127284236	14137	7.247853E-02
Popdc3	27979	0.018544421	6253	7.943730E-01
Por	27980	0.088868316	11260	2.107931E-01
Porcn	27981	0.166105836	16713	1.873707E-02
Postn	27982	0.092254087	11533	1.938531E-01
Pot1a	27983	0.168331474	16840	1.718748E-02
Pot1b	27984	0.047644811	8120	5.028855E-01
Poteg	27985	0.157940525	16171	2.550583E-02
Pou1f1	27986	-0.038406171	3065	5.892384E-01
Pou2af1	27987	0.149128613	15599	3.506756E-02
Pou2f1	27988	0.237018725	19308	7.269119E-04
Pou2f2	27989	0.132251012	14428	6.192998E-02
Pou2f3	27990	0.206495256	18478	3.349868E-03
Pou2f3-rs1	27991		#N/A	1.000000E+00
Pou3f1	27992	0.273018819	19744	9.174773E-05

Spearman Rank correlation analysis performed between Prdm1 and all-expressed genes within the Meredith RNA-seq dataset. Robust Prdm1–associated genes were identified using a cut-off of $p < 0.0005$.

Table S1, Related to Supplemental Figure 3C. Prdm1 associated genes

Pou3f2	27993	0.168499494	16853	1.707517E-02
Pou3f3	27994		#N/A	1.000000E+00
Pou3f4	27995		#N/A	1.000000E+00
Pou4f1	27996	0.156671421	16091	2.672712E-02
Pou4f2	27997	0.053267784	8685	4.537786E-01
Pou4f3	27998		#N/A	1.000000E+00
Pou5f1	27999	-0.038406171	3065	5.892384E-01
Pou5f2	28000		#N/A	1.000000E+00
Pou6f1	28001	0.05447529	8892	4.435947E-01
Pou6f2	28002	0.064486117	9503	3.643011E-01
Pp2d1	28003	0.056422391	9005	4.274493E-01
Ppa1	28004	0.076725822	10394	2.802053E-01
Ppa2	28005	0.006012611	5599	9.326597E-01
Ppan	28006	-0.024472418	4116	7.308655E-01
Ppap2a	28007	0.041895088	7735	5.558405E-01
Ppap2b	28008	0.070612674	9882	3.204198E-01
Ppap2c	28009	0.033680754	7278	6.358803E-01
Ppapdc1a	28010	0.07189585	9990	3.116826E-01
Ppapdc1b	28011	0.178456124	17324	1.146296E-02
Ppapdc2	28012	0.171416337	16992	1.522470E-02
Ppapdc3	28013	-0.054451513	1703	4.437940E-01
Ppara	28014	0.129738207	14285	6.709738E-02
Ppard	28015	0.042006616	7739	5.547882E-01
Pparg	28016	0.122529945	13819	8.389993E-02
Ppargc1a	28017	0.098748249	11975	1.641768E-01
Ppargc1b	28018	0.256436077	19585	2.470454E-04
Ppat	28019	-0.037675463	3642	5.963477E-01
Ppbp	28020		#N/A	1.000000E+00
Ppcdc	28021	-0.070485378	942	3.212952E-01
Ppcs	28022	-0.094597175	379	1.827253E-01
Ppdpf	28023	0.201402047	18304	4.239548E-03
Ppef1	28024	-0.011968835	4670	8.664181E-01
Ppef2	28025	0.08557083	11025	2.282882E-01
Ppfia1	28026	0.172122019	17028	1.480427E-02
Ppfia2	28027	0.255505605	19572	2.606687E-04
Ppfia3	28028	-0.037846511	3637	5.946801E-01
Ppfia4	28029	0.051230127	8404	4.712569E-01
Ppfibp1	28030	0.217788682	18841	1.948549E-03
Ppfibp2	28031	0.147487694	15503	3.714848E-02
Pphln1	28032	0.077552327	10444	2.750416E-01
Ppia	28033	0.205794743	18451	3.461283E-03
Ppib	28034	0.12057099	13697	8.900813E-02
Ppic	28035	0.083902727	10912	2.375192E-01

Spearman Rank correlation analysis performed between Prdm1 and all-expressed genes within the Meredith RNA-seq dataset. Robust Prdm1–associated genes were identified using a cut-off of $p < 0.0005$.

Table S1, Related to Supplemental Figure 3C. Prdm1 associated genes

Ppid	28036	0.090703915	11402	2.014817E-01
Ppie	28037	0.047152856	8089	5.073106E-01
Ppif	28038	-0.006540841	4865	9.267595E-01
Ppig	28039	0.132679423	14458	6.108248E-02
Ppih	28040	0.104408665	12319	1.412017E-01
Ppil1	28041	0.200632979	18271	4.390971E-03
Ppil2	28042	0.068605319	9745	3.344035E-01
Ppil3	28043	0.17515122	17174	1.311272E-02
Ppil4	28044	-0.015038985	4507	8.326046E-01
Ppil6	28045	0.054906936	8917	4.399859E-01
Ppip5k1	28046	0.037149707	7441	6.014869E-01
Ppip5k2	28047	0.14398691	15313	4.193841E-02
Ppl	28048	-0.018290012	4328	7.971324E-01
Ppm1a	28049	0.088699918	11251	2.116625E-01
Ppm1b	28050	0.128473624	14211	6.982816E-02
Ppm1d	28051	0.17028084	16947	1.592313E-02
Ppm1e	28052	0.020466928	6370	7.736055E-01
Ppm1f	28053	-0.012064404	4667	8.653615E-01
Ppm1g	28054	0.133382391	14502	5.971251E-02
Ppm1h	28055	0.039508821	7604	5.785843E-01
Ppm1j	28056	0.09963975	12036	1.603837E-01
Ppm1k	28057	0.057130176	9043	4.216656E-01
Ppm1l	28058	0.125795655	14023	7.591015E-02
Ppm1m	28059	0.150796806	15698	3.305413E-02
Ppm1n	28060		#N/A	1.000000E+00
Ppme1	28061	0.130889917	14348	6.468684E-02
Ppox	28062	-0.076317426	854.5	2.827807E-01
Ppp1ca	28063	0.11805406	13530	9.593295E-02
PPP1CA	28064		#N/A	1.000000E+00
Ppp1cb	28065	0.154096065	15899	2.936012E-02
Ppp1cc	28066	0.176978932	17268	1.217630E-02
Ppp1r10	28067	0.248288924	19486	3.926116E-04
Ppp1r11	28068	0.169151225	16880	1.664552E-02
Ppp1r12a	28069	0.213207735	18690	2.435481E-03
Ppp1r12b	28070	0.234041849	19257	8.512979E-04
Ppp1r12c	28071	0.126385447	14072	7.453509E-02
Ppp1r13b	28072	-0.108031704	194	1.278382E-01
Ppp1r13l	28073	-0.05029439	2192	4.794052E-01
Ppp1r14a	28074	-0.001224019	5151	9.862757E-01
Ppp1r14b	28075	0.200689278	18273	4.379724E-03
Ppp1r14c	28076	0.036697396	7413	6.059240E-01
Ppp1r14d	28077	0.105954047	12418	1.353768E-01
Ppp1r15a	28078	0.085343564	11010	2.295307E-01

Spearman Rank correlation analysis performed between Prdm1 and all-expressed genes within the Meredith RNA-seq dataset. Robust Prdm1–associated genes were identified using a cut-off of $p < 0.0005$.

Table S1, Related to Supplemental Figure 3C. Prdm1 associated genes

Ppp1r15b	28079	0.244648295	19434	4.805508E-04
Ppp1r16a	28080	-0.101549721	265	1.524790E-01
Ppp1r16b	28081	0.105282549	12378	1.378848E-01
Ppp1r17	28082	0.139985211	14952	4.803864E-02
Ppp1r18	28083	0.19487129	18037	5.689106E-03
Ppp1r1a	28084	0.075260396	10283	2.895204E-01
Ppp1r1b	28085	0.118381122	13550	9.500962E-02
Ppp1r1c	28086	-0.003354087	5013	9.624042E-01
Ppp1r2	28087	0.146309572	15436	3.870605E-02
Ppp1r2-ps1	28088		#N/A	1.000000E+00
Ppp1r2-ps2	28089		#N/A	1.000000E+00
Ppp1r2-ps3	28090		#N/A	1.000000E+00
Ppp1r2-ps4	28091		#N/A	1.000000E+00
Ppp1r2-ps6	28092		#N/A	1.000000E+00
Ppp1r2-ps7	28093		#N/A	1.000000E+00
Ppp1r2-ps9	28094		#N/A	1.000000E+00
Ppp1r21	28095	0.118827352	13575	9.376129E-02
Ppp1r26	28096	0.09069342	11401	2.015341E-01
Ppp1r27	28097		#N/A	1.000000E+00
Ppp1r32	28098	-0.038406171	3065	5.892384E-01
Ppp1r35	28099	0.026398849	6896	7.105923E-01
Ppp1r36	28100	-0.038406171	3065	5.892384E-01
Ppp1r37	28101	0.083360379	10857	2.405761E-01
Ppp1r3a	28102	0.024351285	6778	7.321467E-01
Ppp1r3b	28103	0.12021206	13676	8.997049E-02
Ppp1r3c	28104	0.063750846	9454	3.698071E-01
Ppp1r3d	28105	-0.015036437	4509	8.326326E-01
Ppp1r3e	28106	0.062517695	9358	3.791559E-01
Ppp1r3f	28107	0.007930402	5676	9.112582E-01
Ppp1r3g	28108	0.160985049	16395	2.276975E-02
Ppp1r42	28109	0.143605682	15224	4.249002E-02
Ppp1r7	28110	0.142325807	15101	4.438667E-02
Ppp1r8	28111	0.025073986	6825	7.245139E-01
Ppp1r9a	28112	0.158472986	16200	2.500788E-02
Ppp1r9b	28113	0.064232708	9488	3.661930E-01
Ppp2ca	28114	0.096405546	11800	1.744637E-01
Ppp2cb	28115	0.197563413	18143	5.044973E-03
Ppp2r1a	28116	0.276734247	19771	7.283440E-05
Ppp2r1b	28117	0.184307691	17607	8.985983E-03
Ppp2r2a	28118	0.03178703	7175	6.549966E-01
Ppp2r2b	28119	0.069141018	9791	3.306341E-01
Ppp2r2c	28120	0.16291165	16538	2.117233E-02
Ppp2r2d	28121	0.019368657	6297	7.854507E-01

Spearman Rank correlation analysis performed between Prdm1 and all-expressed genes within the Meredith RNA-seq dataset. Robust Prdm1–associated genes were identified using a cut-off of $p < 0.0005$.

Table S1, Related to Supplemental Figure 3C. Prdm1 associated genes

Ppp2r3a	28122	0.149400413	15614	3.473259E-02
Ppp2r3c	28123	0.066988947	9643	3.459430E-01
Ppp2r3d	28124		#N/A	1.000000E+00
Ppp2r4	28125	0.182896682	17546	9.535322E-03
Ppp2r5a	28126	0.085644752	11027	2.278851E-01
Ppp2r5b	28127	0.110654487	12768	1.187892E-01
Ppp2r5c	28128	0.103285175	12257	1.455550E-01
Ppp2r5d	28129	-0.010235207	4736	8.856235E-01
Ppp2r5e	28130	0.04291391	7805	5.462637E-01
Ppp3ca	28131	0.110430583	12751	1.195417E-01
Ppp3cb	28132	0.087646142	11176	2.171616E-01
Ppp3cc	28133	0.280264324	19800	5.831018E-05
Ppp3r1	28134	0.004259328	5528	9.522682E-01
Ppp3r2	28135		#N/A	1.000000E+00
Ppp4c	28136	0.202211615	18334	4.085244E-03
Ppp4r1	28137	0.138159378	14836	5.105846E-02
Ppp4r1l-ps	28138	0.087147593	11141	2.197986E-01
Ppp4r2	28139	0.1514828	15744	3.225513E-02
PPP4R2	28140		#N/A	1.000000E+00
Ppp4r4	28141	0.061854482	9326	3.842430E-01
Ppp5c	28142	0.072000706	9999	3.109756E-01
Ppp6c	28143	-0.024903155	4102	7.263157E-01
Ppp6r1	28144	0.229126012	19123	1.100270E-03
Ppp6r2	28145	-0.068679224	961	3.338818E-01
Ppp6r3	28146	0.233783225	19252	8.629806E-04
Pprc1	28147	0.128473299	14210	6.982887E-02
Ppt1	28148	0.112507075	12890	1.127036E-01
Ppt2	28149		#N/A	1.000000E+00
Pptc7	28150	0.248330573	19487	3.916981E-04
Ppwd1	28151	0.0805606	10637	2.567937E-01
Ppy	28152	-0.028850973	3969	6.850796E-01
Ppyr1	28153		#N/A	1.000000E+00
Pqbp1	28154	0.138071425	14828	5.120782E-02
Pqlc1	28155	0.048922743	8214	4.914859E-01
Pqlc2	28156	-0.098244829	291	1.663482E-01
Pqlc3	28157	0.201855739	18318	4.152440E-03
Pradc1	28158	0.147545406	15506	3.707356E-02
Praf2	28159	0.116747423	13453	9.969302E-02
Pram1	28160	0.101799123	12168	1.514690E-01
Prame	28161	0.111128611	12797	1.172079E-01
Pramef12	28162	-0.045728124	2303	5.202395E-01
Pramef17	28163		#N/A	1.000000E+00
Pramef6	28164		#N/A	1.000000E+00

Spearman Rank correlation analysis performed between Prdm1 and all-expressed genes within the Meredith RNA-seq dataset. Robust Prdm1–associated genes were identified using a cut-off of $p < 0.0005$.

Table S1, Related to Supplemental Figure 3C. Prdm1 associated genes

Pramef8	28165	0.088164319	11211	2.144449E-01
PRAMEF8	28166		#N/A	1.000000E+00
Pramel	28167		#N/A	1.000000E+00
PRAMEL1	28168		#N/A	1.000000E+00
Pramel1	28169	-0.038406171	3065	5.892384E-01
Pramel3	28170		#N/A	1.000000E+00
Pramel4	28171	-0.038406171	3065	5.892384E-01
Pramel5	28172		#N/A	1.000000E+00
Pramel6	28173		#N/A	1.000000E+00
Pramel7	28174		#N/A	1.000000E+00
Prap1	28175	-0.117572864	119.5	9.730435E-02
Prb1	28176	-0.038406171	3065	5.892384E-01
Prc1	28177	0.131653981	14379	6.312713E-02
Prcc	28178	0.032984087	7236	6.428858E-01
Prcp	28179	0.140377737	14980	4.740926E-02
Prdm1	28180	1	20031.5	#DIV/0!
Prdm10	28181	-0.005402072	4904	9.394839E-01
Prdm11	28182	0.165027012	16660	1.953088E-02
Prdm12	28183	0.052728522	8550.5	4.583686E-01
Prdm13	28184	-0.038406171	3065	5.892384E-01
Prdm14	28185	-0.030877746	3889	6.642567E-01
Prdm15	28186	0.167424267	16794	1.780511E-02
Prdm16	28187	0.080483638	10632	2.572499E-01
Prdm2	28188	0.108116061	12559	1.275391E-01
Prdm4	28189	0.008405207	5699	9.059689E-01
Prdm5	28190	0.001638603	5401	9.816279E-01
Prdm6	28191	0.035764941	7364	6.151167E-01
Prdm8	28192	0.168700079	16861	1.694192E-02
Prdm9	28193	0.234451559	19261	8.330879E-04
Prdx1	28194	0.11319571	12937	1.105047E-01
Prdx2	28195	0.114273657	13269	1.071304E-01
Prdx2-rs3	28196		#N/A	1.000000E+00
Prdx3	28197	0.096087149	11781	1.758979E-01
Prdx4	28198	0.235744152	19283	7.779613E-04
Prdx5	28199	0.18736103	17733	7.892256E-03
Prdx6	28200	0.230808973	19185	1.008359E-03
Prdx6-ps2	28201		#N/A	1.000000E+00
Prdx6b	28202		#N/A	1.000000E+00
Preb	28203	0.112965428	12925	1.112363E-01
Prelid1	28204	0.081484824	10699	2.513589E-01
Prelid2	28205	0.142269889	15096	4.447113E-02
Prelp	28206	0.020875719	6412.5	7.692098E-01
Prep	28207	0.108023922	12556	1.278658E-01

Spearman Rank correlation analysis performed between Prdm1 and all-expressed genes within the Meredith RNA-seq dataset. Robust Prdm1–associated genes were identified using a cut-off of $p < 0.0005$.

Table S1, Related to Supplemental Figure 3C. Prdm1 associated genes

Prepl	28208	-0.069382962	955	3.289406E-01
Prex1	28209	-0.014860625	4516	8.345613E-01
Prex2	28210	0.136517652	14727	5.390690E-02
Prf1	28211	-0.079168354	652	2.651326E-01
Prg2	28212	0.024232279	6770	7.334061E-01
Prg3	28213	-0.032791095	3791	6.448320E-01
Prg4	28214	-0.046359218	2278	5.144918E-01
Prh1	28215		#N/A	1.000000E+00
Prhoxnb	28216	-0.054451513	1703	4.437940E-01
Prickle1	28217	0.202068763	18329	4.112099E-03
Prickle2	28218	0.188222715	17771	7.605810E-03
Prickle3	28219	0.401867094	20028	3.675337E-09
Prickle4	28220		#N/A	1.000000E+00
Prim1	28221	0.1790216	17356	1.119979E-02
Prim2	28222	0.003374971	5494	9.621703E-01
Prima1	28223	0.019630193	6310.5	7.826254E-01
Prkaa1	28224	0.099855561	12051	1.594754E-01
Prkaa2	28225	0.085619926	11026	2.280204E-01
Prkab1	28226	0.106883062	12489	1.319648E-01
Prkab2	28227	0.087213962	11143	2.194462E-01
Prkaca	28228	0.01275436	5916	8.577409E-01
Prkaca-ps1	28229		#N/A	1.000000E+00
Prkacb	28230	-0.067882546	968	3.395327E-01
Prkag1	28231	-0.020177013	4232	7.767274E-01
Prkag2	28232	-0.053477719	2132	4.519987E-01
Prkag3	28233		#N/A	1.000000E+00
Prkar1a	28234	0.075895853	10340	2.854559E-01
Prkar1b	28235	0.07142286	9943	3.148849E-01
Prkar2a	28236	0.274806269	19756	8.213744E-05
Prkar2b	28237	-0.026945018	4031	7.048807E-01
Prkca	28238	0.119592089	13632	9.165231E-02
Prkcb	28239	0.129079061	14244	6.850967E-02
PRKCC	28240		#N/A	1.000000E+00
Prkcd	28241	0.077583894	10448	2.748457E-01
Prkcdbp	28242	0.137614417	14798	5.198978E-02
Prkce	28243	0.112943793	12921	1.113052E-01
Prkcg	28244	0.053267784	8685	4.537786E-01
Prkch	28245	0.116508853	13434	1.003920E-01
Prkci	28246	0.278162935	19780	6.658879E-05
Prkcq	28247	0.14596069	15408	3.917773E-02
Prkcsh	28248	0.188872904	17797	7.395833E-03
Prkcz	28249	-0.194206339	1	5.859135E-03
Prkd1	28250	-0.129054692	67	6.856234E-02

Spearman Rank correlation analysis performed between Prdm1 and all-expressed genes within the Meredith RNA-seq dataset. Robust Prdm1–associated genes were identified using a cut-off of $p < 0.0005$.

Table S1, Related to Supplemental Figure 3C. Prdm1 associated genes

Prkd2	28251	0.125571043	14010	7.643919E-02
Prkd3	28252	0.105300022	12381	1.378191E-01
Prkdc	28253	0.054656244	8905	4.420798E-01
Prkg1	28254	0.11365257	13237	1.090646E-01
Prkg2	28255	0.078594461	10503	2.686232E-01
Prkra	28256	0.115047295	13323	1.047590E-01
Prkrip1	28257	-0.02820374	3990	6.917805E-01
Prkrir	28258	0.180166824	17413	1.068307E-02
Prkx	28259	0.069036836	9782	3.313650E-01
Prl	28260		#N/A	1.000000E+00
Prl2a1	28261		#N/A	1.000000E+00
Prl2b1	28262	-0.038406171	3065	5.892384E-01
Prl2c1	28263	-0.038406171	3065	5.892384E-01
Prl2c2	28264	0.162808768	16526.5	2.125512E-02
Prl2c3	28265		#N/A	1.000000E+00
Prl2c5	28266	0.023976542	6751.5	7.361149E-01
Prl3a1	28267	-0.038406171	3065	5.892384E-01
Prl3b1	28268	-0.038406171	3065	5.892384E-01
Prl3c1	28269		#N/A	1.000000E+00
Prl3d1	28270	0.160304018	16323	2.335876E-02
Prl3d2	28271		#N/A	1.000000E+00
Prl3d3	28272		#N/A	1.000000E+00
Prl4a1	28273	0.113548679	13094	1.093908E-01
Prl5a1	28274	0.143605682	15224	4.249002E-02
Prl6a1	28275		#N/A	1.000000E+00
Prl7a1	28276		#N/A	1.000000E+00
Prl7a2	28277		#N/A	1.000000E+00
Prl7b1	28278		#N/A	1.000000E+00
Prl7c1	28279		#N/A	1.000000E+00
Prl7d1	28280		#N/A	1.000000E+00
Prl8a1	28281		#N/A	1.000000E+00
Prl8a2	28282	-0.038406171	3065	5.892384E-01
Prl8a6	28283	0.151119933	15719	3.267570E-02
Prl8a8	28284	-0.09526621	368.5	1.796359E-01
Prl8a9	28285		#N/A	1.000000E+00
Prlh	28286		#N/A	1.000000E+00
Prlhr	28287		#N/A	1.000000E+00
Prlr	28288	0.211538935	18634	2.638707E-03
Prm1	28289	0.113548679	13094	1.093908E-01
Prm2	28290		#N/A	1.000000E+00
Prm3	28291	0.053805701	8820	4.492258E-01
Prmt1	28292	0.032178561	7190	6.510253E-01
Prmt10	28293	0.147575082	15511	3.703509E-02

Spearman Rank correlation analysis performed between Prdm1 and all-expressed genes within the Meredith RNA-seq dataset. Robust Prdm1–associated genes were identified using a cut-off of $p < 0.0005$.

Table S1, Related to Supplemental Figure 3C. Prdm1 associated genes

Prmt2	28294	-0.119558223	104	9.174490E-02
Prmt3	28295	0.245385063	19447	4.614036E-04
Prmt5	28296	0.048752842	8200	4.929935E-01
Prmt6	28297	0.184158641	17597	9.042657E-03
Prmt7	28298	-0.141088318	37	4.628742E-02
Prmt8	28299	0.022198316	6629.5	7.550388E-01
Prnd	28300	0.059193062	9166	4.050706E-01
Prnp	28301	0.155821592	16028	2.757267E-02
Prob1	28302		#N/A	1.000000E+00
Proc	28303	-0.038406171	3065	5.892384E-01
Proca1	28304	0.057837556	9076	4.159309E-01
Procr	28305	0.050312663	8328	4.792453E-01
Prodh	28306	-0.092249085	398	1.938774E-01
Prodh2	28307	0.176145386	17230	1.259581E-02
Prok1	28308		#N/A	1.000000E+00
Prok2	28309	0.329422519	19985	1.905821E-06
Prokr1	28310	0.164224282	16604	2.014030E-02
Prokr2	28311	-0.030867646	3904	6.643599E-01
Prol1	28312		#N/A	1.000000E+00
Prom1	28313	0.054529735	8895	4.431386E-01
Prom2	28314	0.104965588	12353	1.390809E-01
Prop1	28315	0.087115402	11136	2.199696E-01
Prorsd1	28316	0.171383563	16989	1.524448E-02
Pros1	28317	0.007225586	5641	9.191170E-01
Prosc	28318	0.136144627	14707	5.457213E-02
Proser1	28319	-0.004483786	4969	9.497560E-01
Proser2	28320	0.237018887	19309	7.269056E-04
Prox1	28321	0.009014794	5732	8.991843E-01
Prox2	28322	0.048654955	8191.5	4.938632E-01
Proz	28323	-0.136799745	46	5.340830E-02
Prp2	28324		#N/A	1.000000E+00
Prpf18	28325	0.095144016	11706	1.801973E-01
Prpf19	28326	0.194048643	17993	5.900119E-03
Prpf3	28327	0.121495101	13753	8.656825E-02
Prpf31	28328	-0.014548015	4538	8.379931E-01
Prpf38a	28329	0.274913717	19759	8.159094E-05
Prpf38b	28330	0.117031322	13468	9.886628E-02
Prpf39	28331	0.169207188	16887	1.660907E-02
Prpf4	28332	0.106198126	12438	1.344739E-01
Prpf40a	28333	0.286458007	19846	3.918295E-05
Prpf40b	28334	0.06632709	9610	3.507398E-01
Prpf4b	28335	0.14376848	15294	4.225372E-02
Prpf6	28336	0.047220005	8099	5.067054E-01

Spearman Rank correlation analysis performed between Prdm1 and all-expressed genes within the Meredith RNA-seq dataset. Robust Prdm1–associated genes were identified using a cut-off of $p < 0.0005$.

Table S1, Related to Supplemental Figure 3C. Prdm1 associated genes

Prpf8	28337	0.154173639	15903	2.927769E-02
Prph	28338	-0.014336468	4554	8.403172E-01
Prph2	28339	-0.015134061	4504	8.315621E-01
Prpmp5	28340	0.109930598	12709.5	1.212354E-01
Prps1	28341	0.025744785	6858	7.174536E-01
Prps1l1	28342		#N/A	1.000000E+00
Prps1l3	28343		#N/A	1.000000E+00
Prps2	28344	0.181204261	17453	1.023326E-02
Prpsap1	28345	0.055382812	8949	4.360269E-01
Prpsap2	28346	0.164209157	16603	2.015194E-02
Prr11	28347	0.111892099	12844	1.146961E-01
Prr12	28348	0.072129932	10005	3.101057E-01
Prr13	28349	0.126450466	14074	7.438475E-02
Prr14	28350	0.082625468	10797	2.447620E-01
Prr14l	28351	0.245835466	19453	4.500489E-04
Prr15	28352	-0.025113345	4096	7.240990E-01
Prr15l	28353		#N/A	1.000000E+00
Prr16	28354	0.144274992	15322	4.152557E-02
Prr18	28355		#N/A	1.000000E+00
Prr19	28356		#N/A	1.000000E+00
Prr22	28357	-0.038406171	3065	5.892384E-01
Prr23a	28358		#N/A	1.000000E+00
Prr24	28359	0.04823945	8154	4.975639E-01
Prr3	28360	-0.10261912	261	1.481836E-01
Prr5	28361	0.240378124	19366	6.067885E-04
Prr5l	28362	0.12411037	13916	7.995277E-02
Prr7	28363		#N/A	1.000000E+00
Prr9	28364	-0.063723578	1352	3.700123E-01
Prrc1	28365	0.205396663	18433	3.526077E-03
Prrc2a	28366	-0.006218666	4874	9.303577E-01
Prrc2b	28367	0.199059794	18202	4.715963E-03
Prrc2c	28368	0.178353844	17322	1.151113E-02
Prrg1	28369	0.047952411	8144	5.001290E-01
Prrg2	28370	-0.031208333	3879	6.608840E-01
Prrg3	28371	-0.029715454	3941	6.761678E-01
Prrg4	28372	-0.040158779	2468	5.723466E-01
Prrt1	28373	0.143605682	15224	4.249002E-02
Prrt2	28374	0.076893672	10408	2.791513E-01
Prrt3	28375		#N/A	1.000000E+00
Prrt4	28376	-0.017417495	4389.5	8.066150E-01
Prrx1	28377	0.084819371	10974.5	2.324148E-01
Prrx2	28378	-0.024374922	4124	7.318966E-01
Prrxl1	28379	-0.039782961	2473	5.759495E-01

Spearman Rank correlation analysis performed between Prdm1 and all-expressed genes within the Meredith RNA-seq dataset. Robust Prdm1–associated genes were identified using a cut-off of $p < 0.0005$.

Table S1, Related to Supplemental Figure 3C. Prdm1 associated genes

Prss1	28380		#N/A	1.000000E+00
Prss12	28381	-0.056287346	1476	4.285580E-01
Prss16	28382	0.048799051	8204	4.925832E-01
Prss2	28383		#N/A	1.000000E+00
Prss21	28384		#N/A	1.000000E+00
Prss22	28385	0.053805701	8820	4.492258E-01
Prss23	28386	-0.077394997	736.5	2.760195E-01
Prss27	28387	0.092991868	11585	1.902973E-01
Prss28	28388	-0.054450825	1984	4.437997E-01
Prss29	28389	-0.067422642	974	3.428223E-01
Prss3	28390		#N/A	1.000000E+00
Prss30	28391	0.054140154	8872	4.464081E-01
Prss32	28392	-0.062315832	1375	3.806999E-01
Prss33	28393	-0.038406171	3065	5.892384E-01
Prss34	28394		#N/A	1.000000E+00
Prss35	28395	0.12104201	13731	8.775774E-02
Prss36	28396	-0.033702148	3752	6.356657E-01
Prss37	28397		#N/A	1.000000E+00
Prss38	28398	-0.066858259	1029.5	3.468868E-01
Prss39	28399		#N/A	1.000000E+00
Prss40	28400	-0.038406171	3065	5.892384E-01
Prss41	28401	0.242418018	19402.5	5.430862E-04
Prss42	28402		#N/A	1.000000E+00
Prss43	28403	0.121628602	13767	8.622024E-02
Prss44	28404		#N/A	1.000000E+00
Prss45	28405		#N/A	1.000000E+00
Prss46	28406	0.113548679	13094	1.093908E-01
Prss47	28407		#N/A	1.000000E+00
Prss48	28408		#N/A	1.000000E+00
Prss50	28409	-0.103163006	242.5	1.460345E-01
Prss51	28410		#N/A	1.000000E+00
Prss52	28411		#N/A	1.000000E+00
Prss53	28412	0.013384031	5957	8.507976E-01
Prss54	28413		#N/A	1.000000E+00
Prss55	28414	0.113548679	13094	1.093908E-01
Prss56	28415		#N/A	1.000000E+00
Prss57	28416	0.127452468	14148	7.209877E-02
Prss58	28417		#N/A	1.000000E+00
Prss8	28418	0.127130152	14125	7.282779E-02
Prtg	28419	0.24846134	19489	3.888425E-04
Prtn3	28420	-0.038406171	3065	5.892384E-01
Prune	28421	0.132324159	14433	6.178460E-02
Prune2	28422	0.114293417	13272	1.070693E-01

Spearman Rank correlation analysis performed between Prdm1 and all-expressed genes within the Meredith RNA-seq dataset. Robust Prdm1–associated genes were identified using a cut-off of $p < 0.0005$.

Table S1, Related to Supplemental Figure 3C. Prdm1 associated genes

Prx	28423	0.052728522	8550.5	4.583686E-01
Psap	28424	0.141934803	15078	4.498005E-02
Psapl1	28425	0.210070494	18573	2.830126E-03
Psat1	28426	0.175416953	17184	1.297276E-02
Psca	28427	0.102660193	12223	1.480205E-01
Psd	28428	0.030385835	7091	6.692877E-01
Psd2	28429	0.101170101	12129	1.540262E-01
Psd3	28430	0.028804123	7005	6.855639E-01
Psd4	28431	0.19208669	17915	6.431885E-03
Psen1	28432	0.360596637	20013	1.561268E-07
Psen2	28433	0.020733399	6383	7.707393E-01
Psenen	28434	0.124244854	13922	7.962393E-02
Psg16	28435	0.047269832	8103	5.062565E-01
Psg17	28436	-0.066856562	1139	3.468991E-01
Psg18	28437	-0.066855996	1239.5	3.469032E-01
Psg19	28438		#N/A	1.000000E+00
Psg20	28439	-0.09526662	364	1.796340E-01
Psg21	28440	-0.054514896	1537.5	4.432629E-01
Psg22	28441	-0.038406171	3065	5.892384E-01
Psg23	28442	0.086531034	11107	2.230911E-01
Psg25	28443	0.189003976	17802	7.354133E-03
Psg26	28444	-0.086746694	532	2.219355E-01
Psg27	28445	-0.148516936	28	3.583141E-02
Psg28	28446	-0.066858259	1029.5	3.468868E-01
Psg29	28447	0.005994321	5596	9.328641E-01
Psip1	28448	0.180492871	17428	1.053986E-02
Pskh1	28449	-0.034750029	3719	6.251909E-01
Psma1	28450	0.159366254	16245	2.419126E-02
Psma2	28451	0.205791085	18449	3.461873E-03
Psma3	28452	0.183423357	17564	9.326893E-03
Psma4	28453	0.121274334	13743	8.714620E-02
Psma5	28454	0.142471786	15109	4.416682E-02
Psma6	28455	0.146359866	15439	3.863845E-02
Psma7	28456	0.137986281	14823	5.135276E-02
Psma8	28457	0.137252128	14770	5.261669E-02
Psmb1	28458	0.187312663	17732	7.908615E-03
Psmb10	28459	0.213216335	18691	2.434472E-03
Psmb11	28460	0.106935293	12492	1.317750E-01
Psmb2	28461	0.00943047	5745	8.945620E-01
Psmb3	28462	0.119544882	13629	9.178139E-02
Psmb4	28463	0.081079039	10666	2.537352E-01
Psmb5	28464	0.150834884	15704	3.300934E-02
Psmb5-ps	28465	0.05148026	8426	4.690917E-01

Spearman Rank correlation analysis performed between Prdm1 and all-expressed genes within the Meredith RNA-seq dataset. Robust Prdm1–associated genes were identified using a cut-off of $p < 0.0005$.

Table S1, Related to Supplemental Figure 3C. Prdm1 associated genes

Psmb6	28466	0.178151883	17314	1.160679E-02
Psmb6-ps	28467	0.156129434	16056.5	2.726377E-02
Psmb7	28468	0.156613804	16084	2.678374E-02
Psmb7-ps2	28469		#N/A	1.000000E+00
Psmb8	28470	0.141761999	15065	4.524440E-02
Psmb9	28471	0.213586352	18701	2.391399E-03
Psmc1	28472	0.145607583	15385	3.966005E-02
Psmc2	28473	0.033361573	7260	6.390859E-01
Psmc3	28474	0.110974781	12786	1.177191E-01
Psmc3ip	28475	0.153347201	15858	3.016612E-02
Psmc4	28476	0.182465615	17525	9.708972E-03
Psmc5	28477	0.173703187	17106	1.389891E-02
Psmc6	28478	0.199485808	18227	4.625888E-03
Psmd1	28479	0.091903476	11513	1.955599E-01
Psmd10	28480	0.14134929	15038	4.588101E-02
Psmd11	28481	0.231852677	19206	9.549599E-04
Psmd12	28482	0.182319202	17517	9.768586E-03
Psmd13	28483	0.133328083	14500	5.981744E-02
Psmd14	28484	0.179839081	17394	1.082875E-02
Psmd2	28485	0.158681015	16211	2.481562E-02
Psmd3	28486	0.110857944	12781	1.181086E-01
Psmd4	28487	0.153517865	15866	2.998079E-02
Psmd5	28488	0.189866448	17835	7.084929E-03
Psmd6	28489	0.14078485	15008	4.676378E-02
Psmd7	28490	0.036415459	7403	6.086971E-01
Psmd8	28491	0.256242404	19579	2.498252E-04
Psmd9	28492	0.113678133	13239	1.089844E-01
Psme1	28493	0.123323218	13866	8.189957E-02
Psme2	28494	0.170020549	16932	1.608712E-02
Psme2b-ps	28495		#N/A	1.000000E+00
Psme3	28496	0.152813164	15832	3.075236E-02
Psme4	28497	0.055583785	8959	4.343610E-01
Psmf1	28498	0.051576333	8439	4.682616E-01
Psmg1	28499	0.036785097	7421	6.050625E-01
Psmg2	28500	0.133460693	14504	5.956149E-02
Psmg3	28501	0.050014977	8299	4.818530E-01
Psmg4	28502	0.063485575	9433	3.718061E-01
Psors1c2	28503	0.002230815	5428	9.749898E-01
Pspc1	28504	0.043472271	7833	5.410498E-01
Psph	28505	0.258582961	19606	2.181007E-04
Pspn	28506		#N/A	1.000000E+00
Psrc1	28507	-0.026515694	4047	7.093690E-01
Pstk	28508	0.081888854	10736	2.490083E-01

Spearman Rank correlation analysis performed between Prdm1 and all-expressed genes within the Meredith RNA-seq dataset. Robust Prdm1–associated genes were identified using a cut-off of $p < 0.0005$.

Table S1, Related to Supplemental Figure 3C. Prdm1 associated genes

Pstpip1	28509	-0.054451513	1703	4.437940E-01
Pstpip2	28510	0.145806278	15400	3.938804E-02
Ptafr	28511	0.242240068	19399.5	5.483867E-04
Ptar1	28512	0.109492511	12666.5	1.227347E-01
Ptbp1	28513	-0.024675236	4109	7.287220E-01
Ptbp2	28514	0.223601099	19008	1.458724E-03
Ptbp3	28515	0.282455865	19813	5.071302E-05
Ptcd1	28516	-0.049637564	2205	4.851700E-01
Ptcd2	28517	-0.058093518	1433	4.138672E-01
Ptcd3	28518	0.203515241	18371	3.847379E-03
Ptch1	28519	0.074574898	10201.5	2.939481E-01
Ptch2	28520	0.174802091	17155	1.329862E-02
Ptchd1	28521	0.179492249	17383	1.098483E-02
Ptchd2	28522	-0.066855996	1239.5	3.469032E-01
Ptchd3	28523		#N/A	1.000000E+00
Ptchd4	28524	0.171677675	17003	1.506780E-02
Ptcra	28525		#N/A	1.000000E+00
Ptdss1	28526	-0.065730252	1313	3.551011E-01
Ptdss2	28527	0.040955418	7691	5.647448E-01
Pten	28528	0.261287556	19641	1.861303E-04
Pter	28529	0.142815966	15136	4.365209E-02
Ptf1a	28530		#N/A	1.000000E+00
Ptgdr	28531	-0.029527535	3950	6.781013E-01
Ptgds	28532	-0.012706962	4637	8.582640E-01
Ptger1	28533		#N/A	1.000000E+00
Ptger2	28534	0.211130072	18616	2.690788E-03
Ptger3	28535	0.147492628	15504	3.714207E-02
Ptger4	28536	0.165617129	16683	1.909313E-02
Ptges	28537	-0.028064573	3993	6.932245E-01
Ptges2	28538	0.078672719	10510	2.681454E-01
Ptges3	28539	0.151995928	15770	3.166827E-02
Ptges3l	28540		#N/A	1.000000E+00
Ptgfr	28541	-0.038406171	3065	5.892384E-01
Ptgfrn	28542	0.008540763	5707	9.044596E-01
Ptgir	28543	0.05770139	9069	4.170312E-01
Ptgis	28544	0.052545383	8505	4.599332E-01
Ptgr1	28545	0.062456901	9351	3.796205E-01
Ptgr2	28546	0.070052577	9843	3.242829E-01
Ptgs1	28547	0.074080182	10141	2.971714E-01
Ptgs2	28548	0.0717795	9981	3.124684E-01
Pth	28549	0.022198129	6607.5	7.550408E-01
Pth1r	28550	0.102596146	12219	1.482749E-01
Pth2	28551		#N/A	1.000000E+00

Spearman Rank correlation analysis performed between Prdm1 and all-expressed genes within the Meredith RNA-seq dataset. Robust Prdm1–associated genes were identified using a cut-off of $p < 0.0005$.

Table S1, Related to Supplemental Figure 3C. Prdm1 associated genes

Pth2r	28552	0.065718574	9573	3.551868E-01
Pthlh	28553	-0.044983679	2334	5.270615E-01
Ptk2	28554	0.124106774	13915	7.996158E-02
Ptk2b	28555	0.209162696	18556	2.954670E-03
Ptk6	28556	0.201727202	18316	4.176954E-03
Ptk7	28557	0.167187602	16781	1.796937E-02
Ptma	28558	0.187283117	17730	7.918623E-03
Ptma-ps1	28559	0.143605682	15224	4.249002E-02
Ptms	28560	0.165173183	16667	1.942165E-02
Ptn	28561	0.059150515	9163	4.054089E-01
Ptov1	28562	-0.027512215	4013	6.989665E-01
Ptp4a1	28563	0.200491155	18260	4.419421E-03
Ptp4a2	28564	0.153331119	15857	3.018363E-02
Ptp4a3	28565	0.112096958	12859	1.140293E-01
Ptpdc1	28566	0.023453646	6713	7.416637E-01
Ptpla	28567	-0.022097769	4168	7.561133E-01
Ptplad1	28568	0.035096265	7336	6.217461E-01
Ptplad2	28569	0.220786408	18927	1.679804E-03
Ptplb	28570	0.162090138	16471	2.184122E-02
Ptpmt1	28571	0.048629782	8190	4.940870E-01
Ptpn1	28572	0.193987087	17991	5.916186E-03
Ptpn11	28573	0.161533176	16451	2.230503E-02
Ptpn12	28574	0.003101189	5478	9.652371E-01
Ptpn13	28575	0.138588417	14857	5.033503E-02
Ptpn14	28576	0.150356994	15670	3.357519E-02
Ptpn18	28577	0.129396439	14264	6.782666E-02
Ptpn2	28578	0.237336862	19314	7.146608E-04
Ptpn20	28579	0.101562166	12151	1.524285E-01
Ptpn21	28580	0.014576992	6047	8.376748E-01
Ptpn22	28581	0.111602969	12825	1.156423E-01
Ptpn23	28582	0.025445931	6847	7.205962E-01
Ptpn3	28583	-0.095390217	311	1.790675E-01
Ptpn4	28584	0.19811355	18170	4.921694E-03
Ptpn5	28585	0.061789752	9322	3.847417E-01
Ptpn6	28586	0.208835403	18544	3.000776E-03
Ptpn7	28587	0.12128169	13745	8.712689E-02
Ptpn9	28588	0.090540984	11381	2.022960E-01
Ptpra	28589	0.113424088	12952	1.097830E-01
Ptprb	28590	0.005947077	5595	9.333920E-01
Ptprc	28591	0.215127463	18748	2.219350E-03
Ptprcap	28592	0.288223689	19856	3.492475E-05
PTPRCAP	28593		#N/A	1.000000E+00
Ptprd	28594	0.200742994	18274	4.369016E-03

Spearman Rank correlation analysis performed between Prdm1 and all-expressed genes within the Meredith RNA-seq dataset. Robust Prdm1–associated genes were identified using a cut-off of $p < 0.0005$.

Table S1, Related to Supplemental Figure 3C. Prdm1 associated genes

Ptpre	28595	0.063239086	9406	3.736695E-01
Ptprf	28596	0.191308205	17888	6.654474E-03
Ptprg	28597	0.114240903	13268	1.072317E-01
Ptprh	28598	0.014622776	6049	8.371721E-01
Ptprj	28599	-0.063799266	1347	3.694430E-01
Ptprk	28600	0.013574646	5974	8.486980E-01
Ptprm	28601	0.093352425	11603	1.885769E-01
Ptprn	28602	-0.095265801	372	1.796378E-01
Ptprn2	28603	0.032013787	7185	6.526954E-01
Ptpro	28604	0.091335701	11475	1.983468E-01
Ptprq	28605	0.022794806	6679	7.486740E-01
Ptprr	28606	0.12611088	14053	7.517269E-02
Ptprs	28607	0.047186843	8094	5.070042E-01
Ptprt	28608	0.005946457	5594	9.333989E-01
Ptpru	28609	-0.073857552	907	2.986295E-01
Ptprv	28610	0.109801726	12702	1.216750E-01
Ptprz1	28611	0.012583787	5906	8.596237E-01
Ptrf	28612	-0.052058312	2160	4.641090E-01
Ptrh1	28613	0.158207054	16187	2.525552E-02
Ptrh2	28614	0.214185778	18720	2.323089E-03
Ptrhd1	28615	0.175440082	17185	1.296064E-02
Pts	28616	0.044434667	7895	5.321214E-01
Pttg1	28617	-0.000503416	5242	9.943552E-01
Pttg1ip	28618	0.082332867	10777	2.464427E-01
Ptx3	28619	0.204298306	18400	3.710573E-03
Ptx4	28620		#N/A	1.000000E+00
Puf60	28621	0.165882963	16695	1.889873E-02
Pum1	28622	0.126598544	14079	7.404327E-02
Pum2	28623	0.162455736	16495	2.154132E-02
Pura	28624	0.175563094	17192	1.289635E-02
Purb	28625	0.137345451	14782	5.245461E-02
Purg	28626	0.126829376	14097	7.351348E-02
Pus1	28627	0.015346843	6088	8.292298E-01
Pus10	28628	0.035321883	7344	6.195059E-01
Pus3	28629	0.314188962	19951	5.878473E-06
Pus7	28630	0.023288978	6708	7.434139E-01
Pus7l	28631	-0.001405935	5125	9.842362E-01
Pusl1	28632	0.044562227	7905	5.309436E-01
Pvalb	28633	0.037494943	7458	5.981101E-01
Pvr	28634	-0.002137462	5068	9.760361E-01
Pvrl1	28635	0.262386913	19649	1.744309E-04
Pvrl2	28636	0.118897297	13580	9.356680E-02
Pvrl3	28637	0.03679069	7422	6.050076E-01

Spearman Rank correlation analysis performed between Prdm1 and all-expressed genes within the Meredith RNA-seq dataset. Robust Prdm1–associated genes were identified using a cut-off of $p < 0.0005$.

Table S1, Related to Supplemental Figure 3C. Prdm1 associated genes

Pvrl4	28638	0.002748404	5456	9.691896E-01
Pvt1	28639	0.117593582	13498	9.724498E-02
Pwp1	28640	0.194251479	18006	5.847450E-03
Pwp2	28641	0.139740859	14934	4.843394E-02
Pwwp2a	28642	0.044648085	7910	5.301516E-01
Pwwp2b	28643	0.081079579	10667	2.537321E-01
Pxdc1	28644	0.166969929	16769	1.812161E-02
Pxdn	28645	0.150048402	15654	3.394494E-02
Pxk	28646	0.045891186	7995	5.187513E-01
Pxmp2	28647	0.219840979	18898	1.760665E-03
Pxmp4	28648	0.158808619	16216	2.469831E-02
Pxn	28649	0.024362408	6781	7.320290E-01
Pxt1	28650		#N/A	1.000000E+00
Pycard	28651	0.224520289	19031	1.392507E-03
Pycr1	28652	0.224523478	19032	1.392283E-03
Pycr2	28653	-0.040777053	2457	5.664426E-01
Pycrl	28654	0.036297314	7396	6.098608E-01
Pydc3	28655	0.165033582	16661	1.952596E-02
Pydc4	28656	0.234574967	19263	8.276735E-04
Pygb	28657	0.112274279	12873	1.134547E-01
Pygl	28658	0.054320005	8884	4.448970E-01
Pygm	28659	-0.019133402	4263	7.879945E-01
Pygo1	28660	-0.142749903	34.5	4.375050E-02
Pygo2	28661	0.14690572	15474	3.791115E-02
Pyhin1	28662	0.187239027	17728	7.933579E-03
Pyroxd1	28663	0.188845777	17795	7.404489E-03
Pyroxd2	28664	0.168579952	16856	1.702161E-02
Pyurf	28665	0.06777198	9687	3.403217E-01
Pyy	28666	-0.111027523	151	1.175437E-01
Pzp	28667	-0.021139362	4202	7.663787E-01
Qars	28668	0.217342206	18830	1.991769E-03
Qdpr	28669	0.104746868	12338	1.399109E-01
Qk	28670	0.179080713	17360	1.117259E-02
Qpct	28671	-0.110568513	165.5	1.190777E-01
Qpctl	28672	0.195420993	18060	5.551895E-03
Qprt	28673	0.036747428	7420	6.054325E-01
Qrfp	28674	0.109492511	12666.5	1.227347E-01
Qrfpr	28675	0.078722329	10513	2.678428E-01
Qrich1	28676	0.168674372	16860	1.695894E-02
Qrich2	28677		#N/A	1.000000E+00
Qrsl1	28678	0.20115544	18296	4.287583E-03
Qser1	28679	0.206702304	18484	3.317563E-03
Qsox1	28680	0.113778693	13244	1.086696E-01

Spearman Rank correlation analysis performed between Prdm1 and all-expressed genes within the Meredith RNA-seq dataset. Robust Prdm1–associated genes were identified using a cut-off of $p < 0.0005$.

Table S1, Related to Supplemental Figure 3C. Prdm1 associated genes

Qsox2	28681	0.109619129	12685	1.222999E-01
Qtrt1	28682	0.02556914	6853	7.193000E-01
Qtrtd1	28683	0.237442061	19316	7.106518E-04
R3hcc1	28684	0.090409579	11375	2.029544E-01
R3hcc1l	28685	0.130315988	14313	6.587906E-02
R3hdm1	28686	-0.040946593	2454	5.648287E-01
R3hdm2	28687	0.177659945	17294	1.184272E-02
R3hdm4	28688	0.111266402	12806	1.167514E-01
R3hdml	28689	-0.054451513	1703	4.437940E-01
R74862	28690	0.01909219	6285	7.884404E-01
Rab1	28691	0.229578433	19140	1.074835E-03
Rab10	28692	0.07569875	10303	2.867125E-01
Rab11a	28693	0.112694534	12901	1.121017E-01
Rab11b	28694	0.066659151	9629	3.483280E-01
Rab11b-ps2	28695		#N/A	1.000000E+00
Rab11fip1	28696	0.188154023	17766	7.628301E-03
Rab11fip2	28697	0.171133569	16981	1.539608E-02
Rab11fip3	28698	0.085157584	10998	2.305511E-01
Rab11fip4	28699	0.083520784	10881	2.396691E-01
Rab11fip5	28700	0.066292569	9608	3.509911E-01
Rab12	28701	0.221387028	18943	1.630210E-03
Rab13	28702	-0.014755363	4522	8.357165E-01
Rab14	28703	0.218914887	18871	1.843300E-03
Rab15	28704	0.060404801	9232	3.955060E-01
Rab17	28705	0.129972413	14299	6.660133E-02
Rab18	28706	0.125947519	14038	7.555414E-02
Rab19	28707	0.088201388	11223	2.142515E-01
Rab1b	28708	0.02183646	6582	7.589080E-01
Rab20	28709	0.039249716	7591	5.810797E-01
Rab21	28710	0.113526985	12957	1.094590E-01
Rab22a	28711	0.265758858	19681	1.426863E-04
Rab23	28712	-0.053977579	2113.5	4.477765E-01
Rab24	28713	-0.018567169	4298	7.941264E-01
Rab25	28714	0.255903714	19574	2.547565E-04
Rab26	28715	-0.021243686	4200	7.652592E-01
Rab27a	28716	0.09651126	11810	1.739895E-01
Rab27b	28717	0.060708997	9246	3.931264E-01
Rab28	28718	0.005302063	5574	9.406022E-01
Rab2a	28719	0.188599908	17783	7.483359E-03
Rab2b	28720	-0.022432305	4163	7.525400E-01
Rab30	28721	0.077785199	10460	2.735984E-01
Rab31	28722	0.053380227	8766	4.528248E-01
Rab32	28723	0.169810979	16918	1.622022E-02

Spearman Rank correlation analysis performed between Prdm1 and all-expressed genes within the Meredith RNA-seq dataset. Robust Prdm1–associated genes were identified using a cut-off of $p < 0.0005$.

Table S1, Related to Supplemental Figure 3C. Prdm1 associated genes

Rab33a	28724	-0.044852651	2344	5.282669E-01
Rab33b	28725	0.031807735	7178	6.547863E-01
Rab34	28726	-0.071384728	938	3.151440E-01
Rab35	28727	-0.026403393	4051	7.105447E-01
Rab36	28728	-0.038406171	3065	5.892384E-01
Rab37	28729	0.128576835	14214	6.960194E-02
Rab38	28730	0.073022636	10066	3.041401E-01
Rab39	28731	0.108742298	12604.5	1.253355E-01
Rab39b	28732	-0.004320904	4974	9.515790E-01
Rab3a	28733	0.154891623	15972	2.852408E-02
Rab3b	28734	0.135177967	14639	5.632771E-02
Rab3c	28735	0.065325849	9550	3.580755E-01
Rab3d	28736	0.194326801	18013	5.827999E-03
Rab3gap1	28737	0.053598639	8778	4.509753E-01
Rab3gap2	28738	0.111836891	12839	1.148763E-01
Rab3il1	28739	-0.018447724	4312.5	7.954215E-01
Rab3ip	28740	0.176204838	17234	1.256547E-02
Rab40b	28741	0.090840211	11414	2.008024E-01
Rab40c	28742	0.053481683	8770	4.519651E-01
Rab42	28743	0.105231689	12374	1.380762E-01
Rab43	28744	0.113224233	12939	1.104144E-01
Rab44	28745	0.022198129	6607.5	7.550408E-01
Rab4a	28746		#N/A	1.000000E+00
Rab4b	28747	0.31724377	19959	4.712987E-06
Rab5a	28748	0.18131916	17459	1.018449E-02
Rab5b	28749	0.002345177	5435	9.737081E-01
Rab5c	28750	0.134127247	14561	5.828861E-02
Rab6a	28751	0.248648978	19491	3.847789E-04
Rab6b	28752	0.152766881	15809	3.080362E-02
Rab7	28753	0.14364621	15287	4.243109E-02
Rab7l1	28754	0.000877463	5354	9.901612E-01
Rab8a	28755	0.27603881	19765	7.606972E-05
Rab8b	28756	0.063708308	9452	3.701272E-01
Rab9	28757	0.071131461	9921	3.168684E-01
Rab9b	28758	0.057830394	9075	4.159888E-01
Rab9b-ps1	28759		#N/A	1.000000E+00
Rabac1	28760	0.069077543	9785	3.310793E-01
Rabep1	28761	0.07742757	10439	2.758169E-01
Rabep2	28762	0.132029872	14413	6.237121E-02
Rabepk	28763	0.106712633	12476	1.325858E-01
Rabgap1	28764	0.141884238	15075	4.505727E-02
Rabgap1l	28765	0.159097433	16230	2.443457E-02
Rabgef1	28766	0.063550221	9438	3.713184E-01

Spearman Rank correlation analysis performed between Prdm1 and all-expressed genes within the Meredith RNA-seq dataset. Robust Prdm1–associated genes were identified using a cut-off of $p < 0.0005$.

Table S1, Related to Supplemental Figure 3C. Prdm1 associated genes

Rabggta	28767	0.095322386	11724	1.793783E-01
Rabggtb	28768	0.212142218	18656	2.563528E-03
Rabif	28769	0.157159912	16116	2.625123E-02
Rabl2	28770	0.093055038	11592	1.899950E-01
Rabl3	28771	0.151833422	15756	3.185313E-02
Rabl5	28772	0.055732387	8968	4.331316E-01
Rabl6	28773	-0.018160945	4332	7.985333E-01
Rac1	28774	0.245868695	19454	4.492216E-04
Rac2	28775	0.169410743	16899	1.647708E-02
Rac3	28776	0.127811225	14175	7.129430E-02
Racgap1	28777	0.239437549	19356	6.384265E-04
Rad1	28778	-0.028346363	3986	6.903018E-01
Rad17	28779	-0.009346936	4771	8.954906E-01
Rad18	28780	-0.054115816	2109	4.466128E-01
Rad21	28781	0.21090043	18608	2.720448E-03
Rad21l	28782	-0.054451513	1703	4.437940E-01
Rad23a	28783	0.249510264	19503	3.666263E-04
Rad23b	28784	0.197894757	18162	4.970395E-03
Rad50	28785	0.03315552	7249	6.411589E-01
Rad51	28786	0.052447517	8496	4.607706E-01
Rad51ap1	28787	0.029746912	7051	6.758444E-01
Rad51ap2	28788	-0.038406171	3065	5.892384E-01
Rad51b	28789	0.278874365	19785	6.366975E-05
Rad51c	28790	0.09618851	11787	1.754404E-01
Rad51d	28791	0.147843196	15532	3.668901E-02
Rad52	28792	-0.001434793	5121	9.839127E-01
Rad54b	28793	-0.040659931	2459	5.675588E-01
Rad54l	28794	0.21117776	18617	2.684666E-03
Rad54l2	28795	0.147516014	15505	3.711170E-02
Rad9a	28796	0.166069901	16708	1.876305E-02
RAD9A	28797		#N/A	1.000000E+00
Rad9b	28798	0.091498163	11487	1.975464E-01
Radil	28799	0.068210352	9713.5	3.372002E-01
Rae1	28800	0.111679899	12831	1.153900E-01
Raet1d	28801	-0.045728293	2300	5.202379E-01
Raet1e	28802	0.228912619	19112	1.112458E-03
Raf1	28803	0.20201433	18327	4.122374E-03
Rag1	28804	-0.03213205	3839	6.514965E-01
Rag2	28805	0.122030667	13787	8.517894E-02
Rai1	28806	0.137093019	14764	5.289399E-02
Rai14	28807	0.194450191	18021	5.796261E-03
Rai2	28808	-0.016906124	4417	8.121859E-01
Rala	28809	0.000528584	5342	9.940730E-01

Spearman Rank correlation analysis performed between Prdm1 and all-expressed genes within the Meredith RNA-seq dataset. Robust Prdm1–associated genes were identified using a cut-off of $p < 0.0005$.

Table S1, Related to Supplemental Figure 3C. Prdm1 associated genes

Ralb	28810	0.117499207	13493	9.751564E-02
Ralbp1	28811	0.158388752	16195	2.508609E-02
Ralgapa1	28812	0.200923584	18282	4.333191E-03
Ralgapa2	28813	0.091561256	11495	1.972362E-01
Ralgapb	28814	0.263606926	19666	1.622528E-04
Ralgds	28815	0.09443026	11670	1.835022E-01
Ralgps1	28816	0.114373118	13279	1.068232E-01
Ralgps2	28817	0.208591964	18538	3.035490E-03
Raly	28818	0.18533377	17650	8.604309E-03
Ralyl	28819	0.039804719	7618	5.757406E-01
Ramp1	28820	0.125079745	13974	7.760677E-02
Ramp2	28821	0.113548679	13094	1.093908E-01
Ramp3	28822	0.042517718	7776	5.499782E-01
Ran	28823	0.16589924	16697	1.888688E-02
Ranbp1	28824	0.076152361	10357	2.838262E-01
Ranbp10	28825	0.050378901	8330	4.786662E-01
Ranbp17	28826	0.159643117	16258	2.394286E-02
Ranbp2	28827	0.010274251	5784	8.851902E-01
Ranbp3	28828	0.193895885	17988	5.940064E-03
Ranbp3l	28829	0.038758411	7558.5	5.858252E-01
Ranbp6	28830	0.184322908	17608	8.980215E-03
Ranbp9	28831	0.239394255	19355	6.399188E-04
Rangap1	28832	0.192141131	17917	6.416570E-03
Rangrf	28833	0.153004602	15840	3.054110E-02
Rap1a	28834	0.177537167	17288	1.190226E-02
Rap1a-ps2	28835	0.152789767	15822	3.077826E-02
Rap1b	28836	0.106842626	12485	1.321120E-01
Rap1gap	28837	0.020093892	6333	7.776231E-01
Rap1gap2	28838	0.178878018	17349	1.126610E-02
Rap1gds1	28839	0.107783141	12538	1.287226E-01
Rap2a	28840	0.00608625	5601	9.318370E-01
Rap2b	28841	0.03367323	7276	6.359558E-01
Rap2c	28842	0.083806311	10906	2.380606E-01
Rapgef1	28843	0.112956258	12924	1.112655E-01
Rapgef2	28844	0.184102471	17595	9.064096E-03
Rapgef3	28845	0.160987082	16412.5	2.276801E-02
Rapgef4	28846	0.27483019	19757	8.201548E-05
Rapgef5	28847	-0.110801969	152	1.182955E-01
Rapgef6	28848	0.268529708	19709	1.207325E-04
Rapgefl1	28849	-0.095327838	312	1.793533E-01
Raph1	28850	0.235414856	19275	7.916768E-04
Rapsn	28851	0.020434854	6365.5	7.739507E-01
Rara	28852	0.019665523	6314	7.822439E-01

Spearman Rank correlation analysis performed between Prdm1 and all-expressed genes within the Meredith RNA-seq dataset. Robust Prdm1–associated genes were identified using a cut-off of $p < 0.0005$.

Table S1, Related to Supplemental Figure 3C. Prdm1 associated genes

Rarb	28853	0.077210567	10421	2.771689E-01
Rarg	28854	0.067250898	9657	3.440560E-01
Rarres1	28855	0.0259637	6868	7.151546E-01
Rarres2	28856	-0.018954438	4274	7.899312E-01
Rars	28857	0.068732775	9755	3.335042E-01
Rars2	28858	0.039733054	7614	5.764287E-01
Rasa1	28859	-0.006506696	4867	9.271408E-01
Rasa2	28860	-0.085909952	556	2.264430E-01
Rasa3	28861	0.20396667	18389	3.767967E-03
Rasa4	28862	0.048705248	8195	4.934162E-01
Rasal1	28863	-0.031503431	3866.5	6.578792E-01
Rasal2	28864	0.052230782	8487	4.626280E-01
Rasal3	28865	0.028017662	6976	6.937115E-01
Rasd1	28866	0.050507291	8335	4.775446E-01
Rasd2	28867	0.002253895	5430	9.747311E-01
Rasef	28868	0.009371251	5740	8.952202E-01
Rasgef1a	28869	-0.112449558	145	1.128888E-01
Rasgef1b	28870	0.160166985	16285	2.347885E-02
Rasgef1c	28871	0.067617113	9680	3.414288E-01
Rasgrf1	28872	0.246671751	19466	4.296497E-04
Rasgrf2	28873	0.12669978	14084	7.381054E-02
Rasgrp1	28874	-0.087090543	441	2.201018E-01
Rasgrp2	28875	-0.027393757	4021	7.002002E-01
Rasgrp3	28876	0.203863522	18384	3.785981E-03
Rasgrp4	28877	0.272591323	19740	9.419744E-05
Rasip1	28878	0.091061462	11442	1.997032E-01
Rasl10a	28879	0.000193643	5301	9.978287E-01
Rasl10b	28880		#N/A	1.000000E+00
Rasl11a	28881	-0.082661296	610	2.445568E-01
Rasl11b	28882	0.059957099	9208	3.990240E-01
Rasl12	28883	-0.077397959	691.5	2.760011E-01
Rasl2-9	28884	0.194460899	18022	5.793514E-03
Rassf1	28885	0.091555748	11494	1.972633E-01
RASSF10	28886	0.154341066	15916	2.910045E-02
Rassf10	28887		#N/A	1.000000E+00
Rassf2	28888	0.139084107	14895	4.950986E-02
Rassf3	28889	0.212596993	18680	2.508147E-03
Rassf4	28890	0.058726285	9143	4.087913E-01
Rassf5	28891	0.134718387	14603	5.717859E-02
Rassf6	28892	0.098787349	11978	1.640091E-01
Rassf7	28893	0.160985049	16395	2.276975E-02
Rassf8	28894	0.084463549	10952	2.343869E-01
Rassf9	28895	0.276111802	19767	7.572392E-05

Spearman Rank correlation analysis performed between Prdm1 and all-expressed genes within the Meredith RNA-seq dataset. Robust Prdm1–associated genes were identified using a cut-off of $p < 0.0005$.

Table S1, Related to Supplemental Figure 3C. Prdm1 associated genes

Raver1	28896	0.082524129	10790	2.453432E-01
Raver2	28897	0.168609793	16857	1.700178E-02
Rax	28898	-0.038406171	3065	5.892384E-01
Rb1	28899	0.213287566	18693	2.426126E-03
Rb1cc1	28900	0.104784381	12344	1.397683E-01
Rbak	28901	0.126676114	14081	7.386489E-02
Rbbp4	28902	0.206429556	18473	3.360177E-03
Rbbp5	28903	0.056796349	9027	4.243878E-01
Rbbp6	28904	0.027369415	6947	7.004538E-01
Rbbp7	28905	0.171251871	16983	1.532418E-02
Rbbp8	28906	-0.039577001	2477	5.779285E-01
Rbbp8nl	28907		#N/A	1.000000E+00
Rbbp9	28908	-0.007577566	4837	9.151914E-01
Rbck1	28909	0.217596053	18837	1.967090E-03
Rbfa	28910	-0.007301424	4843	9.182710E-01
Rbfox1	28911	0.068315066	9726	3.364573E-01
Rbfox2	28912	0.019773885	6321	7.810743E-01
Rbfox3	28913	0.063666223	9449	3.704441E-01
Rbks	28914	-0.051947165	2165	4.650648E-01
Rbl1	28915	0.132340574	14436	6.175201E-02
Rbl2	28916	-0.023736254	4130	7.386631E-01
Rbm10	28917	0.033249111	7254	6.402170E-01
Rbm11	28918	-0.02926168	3958	6.808402E-01
Rbm12	28919		#N/A	1.000000E+00
Rbm12b1	28920		#N/A	1.000000E+00
Rbm12b2	28921	0.106227153	12442	1.343668E-01
Rbm14	28922	0.080220517	10615	2.588138E-01
Rbm15	28923	0.285492639	19838	4.171324E-05
Rbm15b	28924	-0.030554282	3910	6.675633E-01
Rbm17	28925	0.066050862	9593	3.527541E-01
Rbm18	28926	0.055789004	8972	4.326637E-01
Rbm19	28927	0.092856549	11569	1.909459E-01
Rbm20	28928	-0.075251226	881	2.895793E-01
Rbm22	28929	0.157575363	16130	2.585223E-02
Rbm24	28930	0.111173801	12801	1.170581E-01
Rbm25	28931	0.086568431	11109	2.228904E-01
Rbm26	28932	0.099549027	12031	1.607667E-01
Rbm27	28933	0.249949852	19507	3.576708E-04
Rbm28	28934	0.141792618	15067	4.519747E-02
Rbm3	28935	0.14606996	15418	3.902948E-02
Rbm31y	28936		#N/A	1.000000E+00
Rbm33	28937	0.20519096	18431	3.559987E-03
Rbm34	28938	0.237876049	19325	6.943312E-04

Spearman Rank correlation analysis performed between Prdm1 and all-expressed genes within the Meredith RNA-seq dataset. Robust Prdm1–associated genes were identified using a cut-off of $p < 0.0005$.

Table S1, Related to Supplemental Figure 3C. Prdm1 associated genes

Rbm38	28939	0.041515196	7713	5.594321E-01
Rbm39	28940	0.183206014	17558	9.412411E-03
Rbm4	28941	0.147868875	15534	3.665601E-02
Rbm41	28942	-0.031516186	3857	6.577494E-01
Rbm42	28943	0.171302481	16984	1.529351E-02
Rbm43	28944	-0.094864612	376	1.814857E-01
Rbm44	28945	-0.066856562	1139	3.468991E-01
Rbm45	28946	0.096427022	11803	1.743673E-01
Rbm46	28947	-0.103162243	244	1.460375E-01
Rbm47	28948	0.245702732	19450	4.533680E-04
Rbm48	28949	-0.013068246	4621	8.542784E-01
Rbm4b	28950	0.221904011	18955	1.588597E-03
Rbm5	28951	0.179978928	17400	1.076638E-02
Rbm6	28952	0.053607678	8779	4.508988E-01
Rbm7	28953	0.128379098	14204	7.003587E-02
Rbm8a	28954	0.161897108	16463	2.200102E-02
Rbms1	28955	0.143872281	15303	4.210363E-02
Rbms2	28956	0.070466967	9871	3.214219E-01
Rbms3	28957	0.210965746	18610	2.711982E-03
Rbmx	28958	0.050392737	8332	4.785452E-01
Rbmx2	28959	0.150568873	15684	3.332331E-02
Rbmxl1	28960	0.200499979	18262	4.417646E-03
Rbmxl2	28961		#N/A	1.000000E+00
Rbmy	28962		#N/A	1.000000E+00
Rbp1	28963	0.081931489	10740	2.487611E-01
Rbp2	28964		#N/A	1.000000E+00
Rbp3	28965		#N/A	1.000000E+00
Rbp4	28966	-0.00045847	5257	9.948592E-01
Rbp7	28967	-0.01368196	4578	8.475164E-01
Rbpj	28968	0.148382837	15554	3.600074E-02
Rbpjl	28969		#N/A	1.000000E+00
Rbpms	28970	0.103229512	12255	1.457734E-01
Rbpms2	28971	-0.001224019	5151	9.862757E-01
Rbpsuh-ps1	28972		#N/A	1.000000E+00
Rbpsuh-rs3	28973		#N/A	1.000000E+00
Rbx1	28974	0.078873787	10523	2.669203E-01
Rc3h1	28975	0.184333639	17610	8.976149E-03
Rc3h2	28976	0.233510121	19242	8.754774E-04
Rcan1	28977	-0.017125814	4407	8.097914E-01
Rcan2	28978	0.124590792	13943	7.878304E-02
Rcan3	28979	0.048759165	8201	4.929373E-01
Rcbtb1	28980	0.035595292	7356	6.167957E-01
Rcbtb2	28981	0.125929743	14037	7.559575E-02

Spearman Rank correlation analysis performed between Prdm1 and all-expressed genes within the Meredith RNA-seq dataset. Robust Prdm1–associated genes were identified using a cut-off of $p < 0.0005$.

Table S1, Related to Supplemental Figure 3C. Prdm1 associated genes

Rcc1	28982	-0.003769879	4992	9.577477E-01
Rcc2	28983	0.155590234	16011	2.780678E-02
Rccd1	28984	0.04546004	7966	5.226910E-01
Rce1	28985	-0.012329011	4658	8.624374E-01
Rchy1	28986	0.16776344	16810	1.757198E-02
Rcl1	28987	-0.049567531	2207	4.857868E-01
Rcn1	28988	0.218317977	18850	1.898420E-03
Rcn2	28989	0.056847281	9030	4.239718E-01
Rcn3	28990	-0.033075221	3785	6.419675E-01
Rcor1	28991	0.224035975	19018	1.427046E-03
Rcor2	28992		#N/A	1.000000E+00
Rcor3	28993	0.067337641	9661	3.434325E-01
Rcsd1	28994	-0.000358214	5270	9.959833E-01
Rcvrn	28995	0.140345061	14979	4.746139E-02
Rd3	28996	0.040534432	7668	5.687560E-01
Rdh1	28997	0.095838552	11758	1.770237E-01
Rdh10	28998	0.10906394	12628	1.242153E-01
Rdh11	28999	0.061624343	9309	3.860179E-01
Rdh12	29000	0.04566693	7980	5.207985E-01
Rdh13	29001	0.133527997	14510	5.943193E-02
Rdh14	29002	0.052962566	8593	4.563733E-01
Rdh16	29003	0.161014234	16426	2.274480E-02
Rdh18-ps	29004		#N/A	1.000000E+00
Rdh19	29005	0.004547181	5540	9.490466E-01
Rdh5	29006	0.079852992	10580	2.610091E-01
Rdh7	29007	0.144848021	15346	4.071453E-02
Rdh8	29008		#N/A	1.000000E+00
Rdh9	29009	-0.019516529	4252	7.838529E-01
Rdm1	29010	0.082400829	10781	2.460516E-01
Rdx	29011	0.233452397	19237	8.781400E-04
Rec8	29012		#N/A	1.000000E+00
Reck	29013	-0.062453238	1373	3.796485E-01
Recql	29014	0.045641962	7975	5.210267E-01
Recql4	29015	0.188941405	17800.5	7.374013E-03
Recql5	29016	0.032985952	7237	6.428670E-01
Recql5as1	29017	0.086412184	11087.5	2.237298E-01
Reep1	29018	0.051983707	8469	4.647504E-01
Reep2	29019	-0.038406171	3065	5.892384E-01
Reep3	29020	0.186317066	17695	8.252114E-03
Reep4	29021	0.198407881	18180	4.856854E-03
Reep5	29022	0.07663875	10389	2.807530E-01
Reep6	29023	-0.008118756	4818	9.091595E-01
Reg1	29024	-0.001606757	5107.5	9.819849E-01

Spearman Rank correlation analysis performed between Prdm1 and all-expressed genes within the Meredith RNA-seq dataset. Robust Prdm1–associated genes were identified using a cut-off of $p < 0.0005$.

Table S1, Related to Supplemental Figure 3C. Prdm1 associated genes

Reg2	29025	-0.038406171	3065	5.892384E-01
Reg3a	29026	0.025194487	6836	7.232439E-01
Reg3b	29027	-0.022979343	4149	7.467084E-01
Reg3d	29028	-0.043687291	2385	5.390486E-01
Reg3g	29029	0.083592346	10885	2.392653E-01
Reg4	29030	0.135460048	14661	5.581066E-02
Rel	29031	0.121034947	13730	8.777638E-02
Rela	29032	0.16310656	16551	2.101625E-02
Relb	29033	0.024963974	6815	7.256741E-01
Rell1	29034	0.141337502	15035	4.589930E-02
Rell2	29035	0.134104035	14560	5.833256E-02
Reln	29036	0.107183824	12508	1.308745E-01
Relt	29037	0.159288478	16242	2.426144E-02
Rem1	29038	0.203079187	18362	3.925516E-03
Rem2	29039		#N/A	1.000000E+00
Ren1	29040	-0.066855996	1239.5	3.469032E-01
Renbp	29041	0.094509216	11672	1.831344E-01
Rep15	29042		#N/A	1.000000E+00
Repin1	29043	0.204747156	18416	3.634151E-03
Reps1	29044	0.086692816	11112	2.222238E-01
Reps2	29045	0.127698953	14168	7.154527E-02
Rer1	29046	0.199069701	18203	4.713851E-03
Rere	29047	0.295306455	19886	2.184492E-05
Rerg	29048	0.103513425	12271	1.446625E-01
Rergl	29049	0.020434681	6357	7.739526E-01
Resp18	29050	-0.092072357	401	1.947364E-01
Rest	29051	0.010355512	5789	8.842885E-01
Ret	29052	-0.104288309	212	1.416633E-01
Retn	29053	-0.00045847	5257	9.948592E-01
Retnla	29054	-0.071629044	931	3.134864E-01
Retnlb	29055		#N/A	1.000000E+00
Retnlg	29056	-0.049747415	2200	4.842033E-01
Retsat	29057	0.153468095	15864	3.003474E-02
Rev1	29058	0.062855049	9384	3.765841E-01
Rev3l	29059	0.063717252	9453	3.700599E-01
Rex2	29060	0.020611413	6377.5	7.720510E-01
Rexo1	29061	0.191516476	17895	6.594260E-03
Rexo2	29062	0.115612222	13364	1.030536E-01
Rexo4	29063	0.12402218	13910	8.016901E-02
Rfc1	29064	0.134391497	14584	5.779023E-02
Rfc2	29065	0.21533514	18757	2.197046E-03
Rfc3	29066	0.123112688	13849	8.242667E-02
Rfc4	29067	0.145185798	15364	4.024273E-02

Spearman Rank correlation analysis performed between Prdm1 and all-expressed genes within the Meredith RNA-seq dataset. Robust Prdm1–associated genes were identified using a cut-off of $p < 0.0005$.

Table S1, Related to Supplemental Figure 3C. Prdm1 associated genes

Rfc5	29068	0.117148444	13470	9.852680E-02
Rfesd	29069	-0.010002589	4745	8.882058E-01
Rffl	29070	0.014964346	6070	8.334233E-01
Rfk	29071	0.078295756	10484	2.704524E-01
Rfng	29072	0.009984249	5769	8.884095E-01
Rfpl3s	29073	0.074574898	10201.5	2.939481E-01
Rfpl4	29074	-0.054450825	1984	4.437997E-01
Rfpl4b	29075		#N/A	1.000000E+00
Rft1	29076	0.149502351	15618	3.460767E-02
Rftn1	29077	0.175541312	17189	1.290772E-02
Rftn2	29078	-0.051796035	2169	4.663661E-01
Rfwd2	29079	0.159141537	16232	2.439451E-02
Rfwd3	29080	0.14565338	15388	3.959722E-02
Rfx1	29081	0.010513723	5791	8.825333E-01
Rfx2	29082	0.22976242	19143	1.064646E-03
Rfx3	29083	0.120136168	13672	9.017503E-02
Rfx4	29084	0.193494897	17973	6.046072E-03
Rfx5	29085	0.25733865	19596	2.344664E-04
Rfx6	29086	0.160277746	16292	2.338174E-02
Rfx7	29087	0.150440498	15677	3.347573E-02
Rfx8	29088	0.192070519	17913	6.436440E-03
Rfxank	29089	0.038164848	7489	5.915820E-01
Rfxap	29090	0.158057399	16177	2.539581E-02
Rgag1	29091	0.126933796	14110.5	7.327484E-02
Rgag4	29092	-0.066858259	1029.5	3.468868E-01
Rgcc	29093	0.154317601	15911	2.912524E-02
Rgl1	29094	0.008861005	5720	9.008952E-01
Rgl2	29095	-0.013046132	4622	8.545222E-01
Rgl3	29096	0.245050905	19439	4.699985E-04
Rgma	29097	0.001532567	5391	9.828165E-01
Rgmb	29098	0.149593338	15622	3.449648E-02
Rgn	29099	-0.054450825	1984	4.437997E-01
Rgp1	29100	0.218859128	18870	1.848386E-03
Rgr	29101	0.11917647	13597	9.279375E-02
Rgs1	29102	0.16914068	16879	1.665240E-02
Rgs10	29103	0.112263897	12871	1.134882E-01
Rgs11	29104	0.009014794	5732	8.991843E-01
Rgs12	29105	0.225704053	19066	1.311284E-03
Rgs13	29106	0.031318251	7145	6.597641E-01
Rgs14	29107	-0.035651813	3698	6.162361E-01
Rgs16	29108	0.1066525	12470	1.328054E-01
Rgs17	29109	0.003012316	5473	9.662327E-01
Rgs18	29110	-0.003389066	5010	9.620124E-01

Spearman Rank correlation analysis performed between Prdm1 and all-expressed genes within the Meredith RNA-seq dataset. Robust Prdm1–associated genes were identified using a cut-off of $p < 0.0005$.

Table S1, Related to Supplemental Figure 3C. Prdm1 associated genes

Rgs19	29111	0.19674877	18111	5.232617E-03
Rgs2	29112	0.133665157	14526	5.916862E-02
Rgs20	29113	-0.101846905	263	1.512760E-01
Rgs22	29114	-0.042352727	2427	5.515287E-01
Rgs3	29115	0.080543669	10636	2.568940E-01
Rgs4	29116	0.000513269	5341	9.942447E-01
Rgs5	29117	0.128740839	14230	6.924371E-02
Rgs6	29118	0.173147539	17086	1.421138E-02
Rgs7	29119	0.018993293	6279	7.895106E-01
Rgs7bp	29120	0.099220696	12010	1.621584E-01
Rgs8	29121	0.062439749	9349	3.797517E-01
Rgs9	29122	-0.114980288	135	1.049627E-01
Rgs9bp	29123	0.001083536	5364	9.878507E-01
Rgsl1	29124	-0.034599978	3724	6.266863E-01
Rhag	29125	-0.044260762	2365	5.337293E-01
Rhbdd1	29126	0.147554314	15508	3.706201E-02
Rhbdd2	29127	0.087521836	11169	2.178170E-01
Rhbdd3	29128	0.283945212	19825	4.609248E-05
Rhbdf1	29129	0.06310322	9396	3.746991E-01
Rhbdf2	29130	0.15335513	15859	3.015749E-02
Rhbdl1	29131	0.152871165	15836	3.068822E-02
Rhbdl2	29132	0.097863244	11900	1.680083E-01
Rhbdl3	29133	0.186009208	17681	8.360980E-03
Rhbg	29134	0.105076931	12361	1.386598E-01
Rhcg	29135		#N/A	1.000000E+00
Rhd	29136	-0.000108165	5279.5	9.987871E-01
Rheb	29137	0.11167184	12830	1.154164E-01
Rhebl1	29138	0.09686807	11824	1.723958E-01
Rhno1	29139	0.140278027	14972	4.756848E-02
Rho	29140	-0.038406171	3065	5.892384E-01
Rhoa	29141	0.119956904	13660	9.065965E-02
Rhob	29142	0.164958446	16654	1.958230E-02
Rhobtb1	29143	0.054492998	8893	4.434463E-01
Rhobtb2	29144	0.136177288	14711	5.451362E-02
Rhobtb3	29145	0.002377909	5436	9.733413E-01
Rhoc	29146	-0.009490531	4765	8.938944E-01
Rhod	29147	-0.06445518	1334	3.645318E-01
Rhof	29148		#N/A	1.000000E+00
Rhog	29149	0.206202875	18464	3.395970E-03
Rhoh	29150	-0.077472724	669	2.755361E-01
Rhoj	29151	0.073905264	10118	2.983166E-01
Rhoq	29152	0.052599478	8509.5	4.594707E-01
Rhot1	29153	0.176564621	17247	1.238325E-02

Spearman Rank correlation analysis performed between Prdm1 and all-expressed genes within the Meredith RNA-seq dataset. Robust Prdm1–associated genes were identified using a cut-off of $p < 0.0005$.

Table S1, Related to Supplemental Figure 3C. Prdm1 associated genes

Rhot2	29154	0.04546004	7966	5.226910E-01
Rhou	29155		#N/A	1.000000E+00
Rhov	29156	0.21225291	18662	2.549947E-03
Rhox1	29157	-0.038406171	3065	5.892384E-01
Rhox10	29158		#N/A	1.000000E+00
Rhox11	29159		#N/A	1.000000E+00
Rhox12	29160	0.168018651	16822.5	1.739831E-02
Rhox13	29161	-0.038406171	3065	5.892384E-01
Rhox2a	29162		#N/A	1.000000E+00
Rhox2b	29163	-0.054450825	1984	4.437997E-01
Rhox2c	29164		#N/A	1.000000E+00
Rhox2d	29165	-0.054450825	1984	4.437997E-01
Rhox2e	29166		#N/A	1.000000E+00
Rhox2f	29167		#N/A	1.000000E+00
Rhox2g	29168		#N/A	1.000000E+00
Rhox2h	29169		#N/A	1.000000E+00
Rhox3-ps	29170	-0.077397959	691.5	2.760011E-01
Rhox3a	29171		#N/A	1.000000E+00
Rhox3b	29172		#N/A	1.000000E+00
Rhox3c	29173		#N/A	1.000000E+00
Rhox3e	29174		#N/A	1.000000E+00
Rhox3f	29175		#N/A	1.000000E+00
Rhox3g	29176	-0.066856562	1139	3.468991E-01
Rhox3h	29177		#N/A	1.000000E+00
Rhox4a	29178		#N/A	1.000000E+00
Rhox4b	29179		#N/A	1.000000E+00
Rhox4c	29180		#N/A	1.000000E+00
Rhox4d	29181		#N/A	1.000000E+00
Rhox4e	29182		#N/A	1.000000E+00
Rhox4f	29183		#N/A	1.000000E+00
Rhox4g	29184	0.053267784	8685	4.537786E-01
RHOX5	29185		#N/A	1.000000E+00
Rhox5	29186	0.022198316	6629.5	7.550388E-01
RHOX6	29187		#N/A	1.000000E+00
Rhox6	29188		#N/A	1.000000E+00
RHOX7	29189		#N/A	1.000000E+00
Rhox7	29190	-0.038406171	3065	5.892384E-01
RHOX8	29191		#N/A	1.000000E+00
Rhox8	29192	0.039316116	7594	5.804397E-01
RHOX9	29193		#N/A	1.000000E+00
Rhox9	29194		#N/A	1.000000E+00
Rhpn1	29195		#N/A	1.000000E+00
Rhpn2	29196	0.135263742	14645	5.617007E-02

Spearman Rank correlation analysis performed between Prdm1 and all-expressed genes within the Meredith RNA-seq dataset. Robust Prdm1–associated genes were identified using a cut-off of $p < 0.0005$.

Table S1, Related to Supplemental Figure 3C. Prdm1 associated genes

Rian	29197	-0.05424326	2106.5	4.455415E-01
Ribc1	29198	0.025108338	6827.5	7.241518E-01
Ribc2	29199		#N/A	1.000000E+00
Ric3	29200	0.080937509	10657	2.545677E-01
Ric8	29201	0.119022481	13591	9.321953E-02
Ric8b	29202	0.049822257	8277	4.835452E-01
Rictor	29203	0.101573037	12153	1.523844E-01
Rif1	29204	0.169842734	16921	1.619999E-02
Riiad1	29205	-0.086746694	532	2.219355E-01
Rilp	29206	-0.066855996	1239.5	3.469032E-01
Rilpl1	29207	0.303224721	19914	1.273752E-05
Rilpl2	29208	0.181220116	17455	1.022652E-02
Rimbp2	29209	0.068775667	9759	3.332019E-01
Rimbp3	29210		#N/A	1.000000E+00
Rimkla	29211	0.071977496	9995	3.111320E-01
Rimklb	29212	0.012837329	5919	8.568254E-01
Rims1	29213	0.171835425	17008	1.497378E-02
Rims2	29214	0.032837055	7226	6.443683E-01
Rims3	29215	0.126933796	14110.5	7.327484E-02
Rims4	29216	0.053267784	8685	4.537786E-01
Rin1	29217	0.132566574	14451	6.130479E-02
Rin2	29218	0.155463489	16005	2.793576E-02
Rin3	29219	0.069635016	9824	3.271823E-01
Ring1	29220	-0.008797123	4794	9.016061E-01
Rinl	29221	0.148434275	15557	3.593571E-02
Rint1	29222	0.099911781	12060	1.592395E-01
Riok1	29223	0.135660038	14669	5.544646E-02
Riok2	29224	-0.020987773	4205	7.680061E-01
Riok3	29225	0.161413878	16447	2.240547E-02
Ripk1	29226	0.037860329	7477	5.945454E-01
Ripk2	29227	0.00827124	5691	9.074609E-01
Ripk3	29228	0.101924836	12177	1.509617E-01
Ripk4	29229	0.147346881	15496	3.733182E-02
Ripply1	29230	0.167405542	16791	1.781806E-02
Ripply2	29231	0.007358174	5648	9.176380E-01
Ripply3	29232	0.223803166	19011	1.443925E-03
Rit1	29233	0.099329197	12023	1.616975E-01
Rit2	29234	0.211206407	18618	2.680994E-03
Rlbp1	29235		#N/A	1.000000E+00
Rlf	29236	0.135300878	14647	5.610193E-02
Rlim	29237	0.160668838	16373	2.304162E-02
Rln1	29238		#N/A	1.000000E+00
Rln3	29239		#N/A	1.000000E+00

Spearman Rank correlation analysis performed between Prdm1 and all-expressed genes within the Meredith RNA-seq dataset. Robust Prdm1–associated genes were identified using a cut-off of $p < 0.0005$.

Table S1, Related to Supplemental Figure 3C. Prdm1 associated genes

Rltpr	29240	0.048330759	8162	4.967494E-01
Rmdn1	29241	-0.063692778	1353	3.702441E-01
Rmdn2	29242	0.1980644	18168	4.932597E-03
Rmdn3	29243	-0.019037266	4271	7.890347E-01
Rmi1	29244	-0.053526428	2130	4.515863E-01
Rmi2	29245	0.0967034	11819	1.731300E-01
Rmnd1	29246	0.104713866	12336	1.400364E-01
Rmnd5a	29247	0.231663353	19204	9.644490E-04
Rmnd5b	29248	0.053438209	8768	4.523334E-01
Rmrp	29249	-0.038406171	3065	5.892384E-01
Rmst	29250	-0.005925588	4880	9.336321E-01
Rn5s	29251		#N/A	1.000000E+00
Rn7sk	29252	-0.001606767	5097.5	9.819847E-01
Rnase1	29253	-0.015454864	4495	8.280464E-01
Rnase10	29254	-0.030877746	3889	6.642567E-01
Rnase11	29255		#N/A	1.000000E+00
Rnase12	29256		#N/A	1.000000E+00
Rnase13	29257		#N/A	1.000000E+00
Rnase4	29258	0.047969658	8147	4.999746E-01
Rnase6	29259	0.130755637	14338	6.496419E-02
Rnase9	29260		#N/A	1.000000E+00
Rnaseh1	29261	0.124792061	13954	7.829712E-02
Rnaseh2a	29262	-0.026895348	4034	7.053994E-01
Rnaseh2b	29263	0.091290705	11468	1.985689E-01
Rnaseh2c	29264	0.071449582	9946	3.147034E-01
Rnasek	29265	0.172723451	17064	1.445399E-02
Rnasel	29266	0.188710991	17786	7.447634E-03
Rnaset2a	29267	0.224525454	19033	1.392143E-03
Rnaset2b	29268	-0.089346878	425	2.083363E-01
Rnd1	29269	0.125813891	14025	7.586733E-02
Rnd2	29270	-0.066855996	1239.5	3.469032E-01
Rnd3	29271	0.223436061	19000	1.470914E-03
Rnf10	29272	0.079629004	10568	2.623533E-01
Rnf103	29273	0.162722742	16506	2.132455E-02
Rnf11	29274	0.199427621	18224	4.638099E-03
Rnf111	29275	0.161363851	16444	2.244771E-02
Rnf112	29276	-0.066855996	1239.5	3.469032E-01
Rnf113a1	29277	0.23698807	19307	7.281026E-04
RNF113A2	29278		#N/A	1.000000E+00
Rnf113a2	29279		#N/A	1.000000E+00
Rnf114	29280	0.14414258	15317	4.171490E-02
Rnf115	29281	0.106061109	12430	1.349802E-01
Rnf121	29282	0.147884514	15535	3.663592E-02

Spearman Rank correlation analysis performed between Prdm1 and all-expressed genes within the Meredith RNA-seq dataset. Robust Prdm1–associated genes were identified using a cut-off of $p < 0.0005$.

Table S1, Related to Supplemental Figure 3C. Prdm1 associated genes

Rnf122	29283	0.193185889	17956	6.128915E-03
Rnf123	29284	0.079757119	10577	2.615839E-01
Rnf125	29285	0.162801375	16518	2.126108E-02
Rnf126	29286	0.19679463	18112	5.221890E-03
Rnf128	29287	0.134707126	14601	5.719957E-02
Rnf13	29288	0.068446353	9736	3.355273E-01
Rnf130	29289	0.061278868	9280	3.886916E-01
Rnf133	29290		#N/A	1.000000E+00
Rnf135	29291	0.088578198	11241	2.122926E-01
Rnf138	29292	0.115656113	13369	1.029220E-01
Rnf138rt1	29293		#N/A	1.000000E+00
Rnf139	29294	0.21583543	18784	2.144152E-03
Rnf14	29295	0.154252237	15908	2.919437E-02
Rnf141	29296	0.038533795	7528	5.880006E-01
Rnf144a	29297	0.208069691	18524	3.111196E-03
Rnf144b	29298	0.158464649	16198	2.501561E-02
Rnf145	29299	0.314898802	19953	5.585457E-06
Rnf146	29300	0.096327453	11794	1.748147E-01
Rnf148	29301		#N/A	1.000000E+00
Rnf149	29302	0.144637638	15336	4.101073E-02
Rnf150	29303	0.0841732	10933	2.360049E-01
Rnf151	29304		#N/A	1.000000E+00
Rnf152	29305	0.228363223	19103	1.144408E-03
Rnf157	29306	0.039850224	7631	5.753039E-01
Rnf165	29307	-0.065168912	1322	3.592339E-01
Rnf166	29308	0.003670491	5503	9.588607E-01
Rnf167	29309	0.095228887	11711	1.798072E-01
Rnf168	29310	0.04226501	7756	5.523539E-01
Rnf169	29311	0.1924462	17929	6.331354E-03
Rnf17	29312	0.057823808	9073	4.160419E-01
Rnf170	29313	0.056536261	9009	4.265157E-01
Rnf180	29314	0.140142608	14965	4.778544E-02
Rnf181	29315	0.046009972	8005	5.176685E-01
Rnf182	29316	0.013164497	5942.5	8.532171E-01
Rnf183	29317	0.141056311	15022	4.633747E-02
Rnf185	29318	0.091083491	11445	1.995940E-01
Rnf186	29319	0.201053988	18291	4.307486E-03
Rnf187	29320	0.211031128	18612	2.703531E-03
Rnf19a	29321	0.038779611	7571	5.856200E-01
Rnf19b	29322	0.149969531	15648	3.403999E-02
Rnf2	29323	-0.09494768	374	1.811020E-01
Rnf20	29324	0.016175768	6132	8.201587E-01
Rnf207	29325	0.038757099	7545	5.858379E-01

Spearman Rank correlation analysis performed between Prdm1 and all-expressed genes within the Meredith RNA-seq dataset. Robust Prdm1–associated genes were identified using a cut-off of $p < 0.0005$.

Rnf208	29326	0.07521908	10268	2.897860E-01
Rnf212	29327	-0.077394997	736.5	2.760195E-01
Rnf213	29328	0.221179608	18936	1.647184E-03
Rnf214	29329	-0.085492182	564	2.287177E-01
Rnf215	29330	0.063603101	9444	3.709197E-01
Rnf216	29331	0.106417093	12455	1.336678E-01
Rnf217	29332	0.096881468	11825	1.723362E-01
Rnf219	29333	-0.087613624	436	2.173329E-01
Rnf220	29334	0.139571968	14927	4.870875E-02
Rnf222	29335	-0.066858259	1029.5	3.468868E-01
Rnf223	29336	-0.038406171	3065	5.892384E-01
Rnf24	29337	-0.000242114	5276	9.972852E-01
Rnf25	29338	0.212141721	18655	2.563589E-03
Rnf26	29339	0.112268977	12872	1.134718E-01
Rnf31	29340	0.10764473	12532	1.292171E-01
Rnf32	29341	0.053164998	8603	4.546515E-01
Rnf34	29342	0.307236573	19932	9.632992E-06
Rnf38	29343	0.160499025	16365	2.318877E-02
Rnf39	29344		#N/A	1.000000E+00
Rnf4	29345	0.102331277	12200	1.493307E-01
Rnf40	29346	0.063628839	9445	3.707257E-01
Rnf41	29347	0.073492102	10097	3.010334E-01
Rnf43	29348	0.082958008	10830	2.428617E-01
Rnf44	29349	0.136840508	14755	5.333657E-02
Rnf5	29350	0.087540971	11171	2.177160E-01
Rnf6	29351	0.146615173	15454	3.829682E-02
Rnf7	29352	0.011582434	5857	8.706924E-01
Rnf8	29353	0.257963548	19600	2.261096E-04
Rnft1	29354	0.195945688	18085	5.423699E-03
Rnft2	29355	0.042235231	7754	5.526342E-01
Rngtt	29356	0.106222184	12441	1.343851E-01
Rnh1	29357	0.131559234	14373	6.331885E-02
Rnls	29358	0.171986145	17020	1.488442E-02
Rnmt	29359	0.169050553	16874	1.671127E-02
Rnmtl1	29360	-0.120911944	98	8.810160E-02
Rnpc3	29361	0.173445286	17097	1.404319E-02
Rnpep	29362	0.067687538	9685	3.409251E-01
Rnpepl1	29363	0.053814405	8853	4.491524E-01
Rnps1	29364	0.264270897	19669	1.559634E-04
Rnu11	29365	0.113548679	13094	1.093908E-01
Rnu12	29366	-0.054450825	1984	4.437997E-01
Rnu1a1	29367		#N/A	1.000000E+00
Rnu1b1	29368		#N/A	1.000000E+00

Spearman Rank correlation analysis performed between Prdm1 and all-expressed genes within the Meredith RNA-seq dataset. Robust Prdm1–associated genes were identified using a cut-off of $p < 0.0005$.

Table S1, Related to Supplemental Figure 3C. Prdm1 associated genes

Rnu1b2	29369		#N/A	1.000000E+00
Rnu1b6	29370		#N/A	1.000000E+00
Rnu2-10	29371	-0.054451513	1703	4.437940E-01
Rnu3a	29372		#N/A	1.000000E+00
Rnu3b1	29373		#N/A	1.000000E+00
Rnu3b2	29374		#N/A	1.000000E+00
Rnu3b3	29375		#N/A	1.000000E+00
Rnu3b4	29376		#N/A	1.000000E+00
Rnu5g	29377		#N/A	1.000000E+00
Rnu6	29378		#N/A	1.000000E+00
Rnu7	29379		#N/A	1.000000E+00
Rnu73b	29380		#N/A	1.000000E+00
Rny1	29381		#N/A	1.000000E+00
Rny3	29382	-0.038406171	3065	5.892384E-01
Robo1	29383	0.0165193	6145	8.164062E-01
Robo2	29384	0.085821522	11044	2.269232E-01
Robo3	29385	0.015534368	6096	8.271757E-01
Robo4	29386	0.021757451	6575	7.597536E-01
Rock1	29387	0.114237193	13267	1.072432E-01
Rock2	29388	0.030624443	7104	6.668455E-01
Rogdi	29389	0.104602734	12331	1.404599E-01
Rom1	29390	-0.170135402	4	1.601458E-02
Romo1	29391	0.175544775	17190	1.290591E-02
Ropn1	29392	-0.038406171	3065	5.892384E-01
Ropn1l	29393	0.086425786	11099	2.236566E-01
Ror1	29394	0.057587373	9064	4.179539E-01
Ror2	29395	-0.103161098	248	1.460420E-01
Rora	29396	0.169957523	16926	1.612705E-02
Rorb	29397	0.055094154	8932	4.384259E-01
Rorc	29398	0.023340567	6709	7.428654E-01
Ros1	29399	-0.037611332	3643.5	5.969735E-01
Rp1	29400	-0.038406171	3065	5.892384E-01
Rp1l1	29401	-0.103166823	231.5	1.460195E-01
RP23-103N6.1	29402		#N/A	1.000000E+00
RP23-106C4.4	29403		#N/A	1.000000E+00
RP23-106E9.1	29404		#N/A	1.000000E+00
RP23-111M12.	29405		#N/A	1.000000E+00
RP23-113F14.4	29406	-0.086755146	452	2.218903E-01
RP23-114E15.1	29407		#N/A	1.000000E+00
RP23-11P22.14	29408		#N/A	1.000000E+00
RP23-11P22.15	29409	0.021317126	6513.5	7.644715E-01
RP23-122L19.2	29410		#N/A	1.000000E+00
RP23-124D10.1	29411		#N/A	1.000000E+00

Spearman Rank correlation analysis performed between Prdm1 and all-expressed genes within the Meredith RNA-seq dataset. Robust Prdm1–associated genes were identified using a cut-off of $p < 0.0005$.

Table S1, Related to Supplemental Figure 3C. Prdm1 associated genes

RP23-124N11.3	29412	0.171485828	16997	1.518284E-02
RP23-124N16.2	29413		#N/A	1.000000E+00
RP23-128F3.1	29414		#N/A	1.000000E+00
RP23-129E9.4	29415		#N/A	1.000000E+00
RP23-131O22.2	29416		#N/A	1.000000E+00
RP23-134A10.1	29417	-0.038406171	3065	5.892384E-01
RP23-134A10.2	29418		#N/A	1.000000E+00
RP23-134A10.3	29419		#N/A	1.000000E+00
RP23-134A10.4	29420		#N/A	1.000000E+00
RP23-136M3.8	29421	0.050001765	8296	4.819689E-01
RP23-136O17.1	29422	-0.054451513	1703	4.437940E-01
RP23-137I3.1	29423		#N/A	1.000000E+00
RP23-138D5.1	29424		#N/A	1.000000E+00
RP23-138N1.1	29425	0.126349728	14071	7.461779E-02
RP23-140F24.1	29426		#N/A	1.000000E+00
RP23-142J2.1	29427	-0.000841234	5228	9.905674E-01
RP23-145J9.2	29428		#N/A	1.000000E+00
RP23-145L9.8	29429		#N/A	1.000000E+00
RP23-146P1.1	29430		#N/A	1.000000E+00
RP23-146P1.2	29431		#N/A	1.000000E+00
RP23-14K19.1	29432	0.053805701	8820	4.492258E-01
RP23-14N12.5	29433		#N/A	1.000000E+00
RP23-151I7.1	29434	0.073928833	10125	2.981622E-01
RP23-152L21.1	29435		#N/A	1.000000E+00
RP23-152P5.2	29436	-0.038406171	3065	5.892384E-01
RP23-155H5.2	29437	0.037700968	7469	5.960989E-01
RP23-155H5.3	29438		#N/A	1.000000E+00
RP23-156K23.1	29439		#N/A	1.000000E+00
RP23-156K23.2	29440		#N/A	1.000000E+00
RP23-157G22.1	29441		#N/A	1.000000E+00
RP23-159J2.2	29442		#N/A	1.000000E+00
RP23-15M14.1	29443		#N/A	1.000000E+00
RP23-161F5.4	29444	0.048460979	8182	4.955890E-01
RP23-161N17.1	29445	-0.054451513	1703	4.437940E-01
RP23-168E14.4	29446		#N/A	1.000000E+00
RP23-168E14.5	29447	-0.054451513	1703	4.437940E-01
RP23-168E14.7	29448	0.053267784	8685	4.537786E-01
RP23-169M5.1	29449	-0.038406171	3065	5.892384E-01
RP23-16G10.2	29450		#N/A	1.000000E+00
RP23-174B18.1	29451		#N/A	1.000000E+00
RP23-174N23.1	29452		#N/A	1.000000E+00
RP23-175C13.2	29453		#N/A	1.000000E+00
RP23-175C13.3	29454		#N/A	1.000000E+00

Spearman Rank correlation analysis performed between Prdm1 and all-expressed genes within the Meredith RNA-seq dataset. Robust Prdm1–associated genes were identified using a cut-off of $p < 0.0005$.

Table S1, Related to Supplemental Figure 3C. Prdm1 associated genes

RP23-175C13.4	29455		#N/A	1.000000E+00
RP23-175C13.5	29456		#N/A	1.000000E+00
RP23-175C13.6	29457		#N/A	1.000000E+00
RP23-177B9.1	29458	-0.038406171	3065	5.892384E-01
RP23-180D16.5	29459	-0.054451513	1703	4.437940E-01
RP23-180L12.4	29460	-0.038406171	3065	5.892384E-01
RP23-183E20.1	29461		#N/A	1.000000E+00
RP23-186N8.1	29462		#N/A	1.000000E+00
RP23-188F24.7	29463		#N/A	1.000000E+00
RP23-18L24.1	29464	0.050870414	8368.5	4.743803E-01
RP23-190H11.4	29465		#N/A	1.000000E+00
RP23-190N21.1	29466		#N/A	1.000000E+00
RP23-192H11.1	29467	-0.066858259	1029.5	3.468868E-01
RP23-193J20.5	29468		#N/A	1.000000E+00
RP23-197G3.3	29469		#N/A	1.000000E+00
RP23-197G3.5	29470		#N/A	1.000000E+00
RP23-197K11.3	29471		#N/A	1.000000E+00
RP23-197K11.4	29472		#N/A	1.000000E+00
RP23-197K11.5	29473		#N/A	1.000000E+00
RP23-197P10.1	29474		#N/A	1.000000E+00
RP23-1B19.3	29475		#N/A	1.000000E+00
RP23-202F23.6	29476		#N/A	1.000000E+00
RP23-202F23.7	29477		#N/A	1.000000E+00
RP23-202I21.1	29478	0.028644364	7000	6.872160E-01
RP23-202I21.2	29479	-0.054451513	1703	4.437940E-01
RP23-203H20.2	29480		#N/A	1.000000E+00
RP23-204I16.2	29481		#N/A	1.000000E+00
RP23-205C7.1	29482	0.00025276	5303	9.971658E-01
RP23-206J9.3	29483	-0.07739401	763.5	2.760257E-01
RP23-206J9.5	29484	0.151879602	15757.5	3.180051E-02
RP23-207N5.1	29485	-0.001606767	5097.5	9.819847E-01
RP23-210I20.4	29486	-0.004526355	4961.5	9.492796E-01
RP23-214I13.2	29487	0.152272071	15784	3.135622E-02
RP23-218F7.1	29488	-0.038406171	3065	5.892384E-01
RP23-21C15.1	29489	0.113548679	13094	1.093908E-01
RP23-220F4.1	29490		#N/A	1.000000E+00
RP23-220P13.1	29491		#N/A	1.000000E+00
RP23-222I2.1	29492	0.160304018	16323	2.335876E-02
RP23-222K15.2	29493	-0.011206622	4697	8.748531E-01
RP23-223E24.1	29494		#N/A	1.000000E+00
RP23-223E24.2	29495		#N/A	1.000000E+00
RP23-227N23.1	29496	-0.066858259	1029.5	3.468868E-01
RP23-22I7.1	29497	0.048448014	8175.5	4.957045E-01

Spearman Rank correlation analysis performed between Prdm1 and all-expressed genes within the Meredith RNA-seq dataset. Robust Prdm1–associated genes were identified using a cut-off of $p < 0.0005$.

Table S1, Related to Supplemental Figure 3C. Prdm1 associated genes

RP23-235P13.1	29498		#N/A	1.000000E+00
RP23-237H8.2	29499	0.179790704	17393	1.085040E-02
RP23-239A11.1	29500		#N/A	1.000000E+00
RP23-239F10.2	29501		#N/A	1.000000E+00
RP23-239F10.4	29502		#N/A	1.000000E+00
RP23-239L21.4	29503	0.082269134	10768	2.468098E-01
RP23-23B10.3	29504		#N/A	1.000000E+00
RP23-23I2.1	29505		#N/A	1.000000E+00
RP23-244I2.2	29506		#N/A	1.000000E+00
RP23-245F10.1	29507	-0.054451513	1703	4.437940E-01
RP23-246H16.2	29508		#N/A	1.000000E+00
RP23-247A1.2	29509		#N/A	1.000000E+00
RP23-247H23.2	29510		#N/A	1.000000E+00
RP23-251E2.4	29511	0.17058565	16960	1.573296E-02
RP23-251I6.1	29512	0.046025613	8011	5.175260E-01
RP23-251N10.1	29513	0.146027145	15415	3.908752E-02
RP23-253C24.2	29514		#N/A	1.000000E+00
RP23-254F23.1	29515		#N/A	1.000000E+00
RP23-255J24.1	29516		#N/A	1.000000E+00
RP23-258B18.1	29517		#N/A	1.000000E+00
RP23-262I3.2	29518		#N/A	1.000000E+00
RP23-262J12.1	29519		#N/A	1.000000E+00
RP23-262O4.8	29520	-0.038406171	3065	5.892384E-01
RP23-263M10.	29521	-0.038406171	3065	5.892384E-01
RP23-267D22.1	29522	-0.029824287	3931	6.750490E-01
RP23-268A5.1	29523		#N/A	1.000000E+00
RP23-268C9.3	29524		#N/A	1.000000E+00
RP23-269P8.1	29525	0.113548679	13094	1.093908E-01
RP23-269P8.2	29526	0.04230569	7760	5.519711E-01
RP23-271G7.2	29527		#N/A	1.000000E+00
RP23-271G7.3	29528	0.160304018	16323	2.335876E-02
RP23-272A7.2	29529		#N/A	1.000000E+00
RP23-273K11.1	29530	0.083452862	10867	2.400529E-01
RP23-274E2.5	29531		#N/A	1.000000E+00
RP23-274E2.6	29532		#N/A	1.000000E+00
RP23-274E2.7	29533	-0.038406171	3065	5.892384E-01
RP23-274E2.8	29534		#N/A	1.000000E+00
RP23-275E20.1	29535		#N/A	1.000000E+00
RP23-281M6.1	29536		#N/A	1.000000E+00
RP23-282M20.	29537		#N/A	1.000000E+00
RP23-285C18.2	29538	-0.04965826	2204	4.849878E-01
RP23-285C18.3	29539	0.160987082	16412.5	2.276801E-02
RP23-285H4.1	29540		#N/A	1.000000E+00

Spearman Rank correlation analysis performed between Prdm1 and all-expressed genes within the Meredith RNA-seq dataset. Robust Prdm1–associated genes were identified using a cut-off of $p < 0.0005$.

Table S1, Related to Supplemental Figure 3C. Prdm1 associated genes

RP23-287H13.1	29541		#N/A	1.000000E+00
RP23-287O2.2	29542		#N/A	1.000000E+00
RP23-288L22.3	29543		#N/A	1.000000E+00
RP23-289F7.2	29544	0.050544302	8344.5	4.772215E-01
RP23-291E6.5	29545		#N/A	1.000000E+00
RP23-292P21.1	29546		#N/A	1.000000E+00
RP23-296J16.1	29547		#N/A	1.000000E+00
RP23-296L9.1	29548		#N/A	1.000000E+00
RP23-299M19.	29549	-0.103163388	239	1.460330E-01
RP23-2B18.1	29550		#N/A	1.000000E+00
RP23-2H9.1	29551		#N/A	1.000000E+00
RP23-301L2.2	29552		#N/A	1.000000E+00
RP23-301L2.3	29553		#N/A	1.000000E+00
RP23-301L2.4	29554		#N/A	1.000000E+00
RP23-302C16.2	29555	0.025108338	6827.5	7.241518E-01
RP23-302C16.3	29556	-0.000108165	5279.5	9.987871E-01
RP23-304D1.1	29557		#N/A	1.000000E+00
RP23-30H7.1	29558	0.021317126	6513.5	7.644715E-01
RP23-311B24.7	29559		#N/A	1.000000E+00
RP23-311B8.2	29560		#N/A	1.000000E+00
RP23-317M11.	29561		#N/A	1.000000E+00
RP23-320B10.2	29562		#N/A	1.000000E+00
RP23-324J8.6	29563		#N/A	1.000000E+00
RP23-326B21.7	29564	-0.038406171	3065	5.892384E-01
RP23-32L5.4	29565		#N/A	1.000000E+00
RP23-32L5.5	29566		#N/A	1.000000E+00
RP23-331E4.1	29567		#N/A	1.000000E+00
RP23-335E18.1	29568		#N/A	1.000000E+00
RP23-337B16.1	29569		#N/A	1.000000E+00
RP23-337F6.1	29570		#N/A	1.000000E+00
RP23-337J16.5	29571		#N/A	1.000000E+00
RP23-342M4.1	29572	-0.054450825	1984	4.437997E-01
RP23-345E17.1	29573		#N/A	1.000000E+00
RP23-345J21.2	29574		#N/A	1.000000E+00
RP23-346H4.1	29575		#N/A	1.000000E+00
RP23-347H20.1	29576		#N/A	1.000000E+00
RP23-348F1.1	29577		#N/A	1.000000E+00
RP23-348L9.1	29578		#N/A	1.000000E+00
RP23-34E15.1	29579	-0.051369758	2175	4.700476E-01
RP23-34F5.1	29580		#N/A	1.000000E+00
RP23-356G2.1	29581	0.021317126	6513.5	7.644715E-01
RP23-358B17.1	29582		#N/A	1.000000E+00
RP23-359A23.1	29583		#N/A	1.000000E+00

Spearman Rank correlation analysis performed between Prdm1 and all-expressed genes within the Meredith RNA-seq dataset. Robust Prdm1–associated genes were identified using a cut-off of $p < 0.0005$.

Table S1, Related to Supplemental Figure 3C. Prdm1 associated genes

RP23-359C15.1	29584		#N/A	1.000000E+00
RP23-359M14.	29585		#N/A	1.000000E+00
RP23-360N5.2	29586	0.168466304	16849.5	1.709730E-02
RP23-360N5.3	29587		#N/A	1.000000E+00
RP23-360N5.4	29588	-0.038406171	3065	5.892384E-01
RP23-361G19.1	29589		#N/A	1.000000E+00
RP23-368D4.4	29590		#N/A	1.000000E+00
RP23-368D4.5	29591	-0.038406171	3065	5.892384E-01
RP23-369P2.1	29592		#N/A	1.000000E+00
RP23-375N21.5	29593	0.053805701	8820	4.492258E-01
RP23-376O10.1	29594		#N/A	1.000000E+00
RP23-377H14.1	29595		#N/A	1.000000E+00
RP23-378G4.2	29596	-0.038406171	3065	5.892384E-01
RP23-37C8.1	29597		#N/A	1.000000E+00
RP23-380I13.1	29598	-0.038406171	3065	5.892384E-01
RP23-380O16.4	29599		#N/A	1.000000E+00
RP23-387H3.1	29600		#N/A	1.000000E+00
RP23-390C13.1	29601		#N/A	1.000000E+00
RP23-390K15.1	29602		#N/A	1.000000E+00
RP23-392K24.4	29603	0.196649669	18107	5.255866E-03
RP23-393I12.2	29604	0.004555424	5541	9.489543E-01
RP23-393I12.5	29605		#N/A	1.000000E+00
RP23-393I12.6	29606		#N/A	1.000000E+00
RP23-396N6.8	29607		#N/A	1.000000E+00
RP23-397L11.2	29608	-0.004937044	4944	9.446847E-01
RP23-398A2.1	29609		#N/A	1.000000E+00
RP23-399J8.10	29610		#N/A	1.000000E+00
RP23-3D20.1	29611		#N/A	1.000000E+00
RP23-3D20.2	29612	0.143605682	15224	4.249002E-02
RP23-3F1.5	29613	0.021317126	6513.5	7.644715E-01
RP23-3N21.2	29614		#N/A	1.000000E+00
RP23-3N21.3	29615		#N/A	1.000000E+00
RP23-3N21.4	29616		#N/A	1.000000E+00
RP23-3N21.5	29617		#N/A	1.000000E+00
RP23-404J7.2	29618		#N/A	1.000000E+00
RP23-404J7.4	29619		#N/A	1.000000E+00
RP23-404J7.5	29620		#N/A	1.000000E+00
RP23-407D18.1	29621	-0.038406171	3065	5.892384E-01
RP23-412B2.6	29622	-0.025194834	4093	7.232402E-01
RP23-416A5.1	29623		#N/A	1.000000E+00
RP23-416A5.2	29624	0.048446374	8169.5	4.957191E-01
RP23-416A5.3	29625	0.063354619	9414	3.727954E-01
RP23-416E12.1	29626	0.16741515	16792	1.781141E-02

Spearman Rank correlation analysis performed between Prdm1 and all-expressed genes within the Meredith RNA-seq dataset. Robust Prdm1–associated genes were identified using a cut-off of $p < 0.0005$.

Table S1, Related to Supplemental Figure 3C. Prdm1 associated genes

RP23-418G1.1	29627	0.020875719	6412.5	7.692098E-01
RP23-419L21.1	29628		#N/A	1.000000E+00
RP23-421G2.1	29629		#N/A	1.000000E+00
RP23-426G16.1	29630		#N/A	1.000000E+00
RP23-426K20.1	29631		#N/A	1.000000E+00
RP23-428C21.3	29632		#N/A	1.000000E+00
RP23-428C21.5	29633	-0.038406171	3065	5.892384E-01
RP23-429A10.1	29634		#N/A	1.000000E+00
RP23-429E19.2	29635		#N/A	1.000000E+00
RP23-429I18.1	29636		#N/A	1.000000E+00
RP23-42D10.1	29637		#N/A	1.000000E+00
RP23-42E3.1	29638		#N/A	1.000000E+00
RP23-433F5.1	29639	-0.077393517	797	2.760287E-01
RP23-433F5.2	29640		#N/A	1.000000E+00
RP23-433F5.4	29641		#N/A	1.000000E+00
RP23-437I5.1	29642		#N/A	1.000000E+00
RP23-438L8.1	29643		#N/A	1.000000E+00
RP23-43F3.4	29644	0.076984127	10411	2.785845E-01
RP23-441L19.1	29645	0.169402057	16896	1.648270E-02
RP23-442M18.	29646		#N/A	1.000000E+00
RP23-44N5.2	29647	0.135169958	14636.5	5.634245E-02
RP23-44O19.1	29648		#N/A	1.000000E+00
RP23-452I19.1	29649		#N/A	1.000000E+00
RP23-454K24.2	29650		#N/A	1.000000E+00
RP23-455H16.2	29651		#N/A	1.000000E+00
RP23-458N20.1	29652		#N/A	1.000000E+00
RP23-459L15.5	29653		#N/A	1.000000E+00
RP23-460B2.1	29654		#N/A	1.000000E+00
RP23-463F7.10	29655	0.1639619	16592	2.034304E-02
RP23-463F7.5	29656		#N/A	1.000000E+00
RP23-463F7.6	29657		#N/A	1.000000E+00
RP23-463F7.8	29658	0.091471625	11482	1.976770E-01
RP23-468P13.2	29659	-0.038406171	3065	5.892384E-01
RP23-469H5.2	29660	0.027738102	6962	6.966162E-01
RP23-469N6.2	29661		#N/A	1.000000E+00
RP23-474B13.3	29662	0.036029921	7382	6.124982E-01
RP23-474B13.4	29663		#N/A	1.000000E+00
RP23-476C23.4	29664		#N/A	1.000000E+00
RP23-479D21.2	29665		#N/A	1.000000E+00
RP23-4F1.1	29666		#N/A	1.000000E+00
RP23-4F1.5	29667	-0.077397959	691.5	2.760011E-01
RP23-50H13.5	29668	-0.038406171	3065	5.892384E-01
RP23-51D4.2	29669		#N/A	1.000000E+00

Spearman Rank correlation analysis performed between Prdm1 and all-expressed genes within the Meredith RNA-seq dataset. Robust Prdm1–associated genes were identified using a cut-off of $p < 0.0005$.

Table S1, Related to Supplemental Figure 3C. Prdm1 associated genes

RP23-51D4.3	29670	0.059117609	9162	4.056707E-01
RP23-52J20.3	29671		#N/A	1.000000E+00
RP23-55O17.1	29672	0.053267784	8685	4.537786E-01
RP23-58H5.2	29673		#N/A	1.000000E+00
RP23-58H5.5	29674		#N/A	1.000000E+00
RP23-58H5.6	29675		#N/A	1.000000E+00
RP23-58H5.7	29676		#N/A	1.000000E+00
RP23-58H5.8	29677		#N/A	1.000000E+00
RP23-58L22.2	29678	0.104290365	12311	1.416554E-01
RP23-58L22.3	29679	-0.077394997	736.5	2.760195E-01
RP23-58L22.6	29680		#N/A	1.000000E+00
RP23-58L22.7	29681		#N/A	1.000000E+00
RP23-58L22.8	29682		#N/A	1.000000E+00
RP23-59P17.1	29683		#N/A	1.000000E+00
RP23-59P17.2	29684	0.053267784	8685	4.537786E-01
RP23-61D6.4	29685		#N/A	1.000000E+00
RP23-62M19.1	29686		#N/A	1.000000E+00
RP23-67I3.1	29687		#N/A	1.000000E+00
RP23-70A15.5	29688		#N/A	1.000000E+00
RP23-70F22.1	29689	0.106314226	12445	1.340460E-01
RP23-71K9.4	29690		#N/A	1.000000E+00
RP23-71L2.6	29691		#N/A	1.000000E+00
RP23-72M20.5	29692	-0.077393517	797	2.760287E-01
RP23-72M20.6	29693	-0.077393023	832.5	2.760318E-01
RP23-73F23.2	29694		#N/A	1.000000E+00
RP23-74O12.3	29695	-0.001224019	5151	9.862757E-01
RP23-76C13.2	29696		#N/A	1.000000E+00
RP23-76C13.3	29697		#N/A	1.000000E+00
RP23-76C13.4	29698		#N/A	1.000000E+00
RP23-76C13.7	29699	-0.038406171	3065	5.892384E-01
RP23-77B14.1	29700		#N/A	1.000000E+00
RP23-79G18.1	29701		#N/A	1.000000E+00
RP23-80D9.1	29702		#N/A	1.000000E+00
RP23-81C12.1	29703	0.088437354	11235	2.130233E-01
RP23-81C12.3	29704	0.183654484	17579	9.236706E-03
RP23-88A13.3	29705		#N/A	1.000000E+00
RP23-91L18.2	29706	0.098384184	11948.5	1.657450E-01
RP23-91L18.3	29707		#N/A	1.000000E+00
RP23-91L18.4	29708		#N/A	1.000000E+00
RP23-91L18.5	29709		#N/A	1.000000E+00
RP23-94E5.1	29710		#N/A	1.000000E+00
RP23-94F11.7	29711		#N/A	1.000000E+00
RP23-95K12.12	29712		#N/A	1.000000E+00

Spearman Rank correlation analysis performed between Prdm1 and all-expressed genes within the Meredith RNA-seq dataset. Robust Prdm1–associated genes were identified using a cut-off of $p < 0.0005$.

Table S1, Related to Supplemental Figure 3C. Prdm1 associated genes

RP23-95K12.9	29713		#N/A	1.000000E+00
RP23-96D5.2	29714		#N/A	1.000000E+00
RP23-97D14.8	29715		#N/A	1.000000E+00
RP24-107B13.1	29716		#N/A	1.000000E+00
RP24-114L16.1	29717		#N/A	1.000000E+00
RP24-117M7.1	29718		#N/A	1.000000E+00
RP24-118H13.2	29719		#N/A	1.000000E+00
RP24-123I12.1	29720		#N/A	1.000000E+00
RP24-131C5.1	29721		#N/A	1.000000E+00
RP24-134G9.1	29722		#N/A	1.000000E+00
RP24-144M14.	29723	0.1544596	15936.5	2.897553E-02
RP24-149G24.1	29724	0.019770131	6319	7.811148E-01
RP24-150D18.2	29725		#N/A	1.000000E+00
RP24-152K24.7	29726		#N/A	1.000000E+00
RP24-158L2.1	29727	-0.032464329	3811	6.481329E-01
RP24-160J12.1	29728	0.01173719	5863	8.689800E-01
RP24-167K8.1	29729		#N/A	1.000000E+00
RP24-173K24.1	29730	-0.038406171	3065	5.892384E-01
RP24-175I3.1	29731		#N/A	1.000000E+00
RP24-179I13.1	29732		#N/A	1.000000E+00
RP24-189G6.1	29733	0.038758411	7558.5	5.858252E-01
RP24-189K8.2	29734		#N/A	1.000000E+00
RP24-192J9.1	29735	0.143605682	15224	4.249002E-02
RP24-196P19.1	29736		#N/A	1.000000E+00
RP24-204J10.1	29737		#N/A	1.000000E+00
RP24-205N24.3	29738	-0.066856562	1139	3.468991E-01
RP24-210G2.1	29739		#N/A	1.000000E+00
RP24-210J6.2	29740		#N/A	1.000000E+00
RP24-211P18.4	29741		#N/A	1.000000E+00
RP24-211P18.5	29742		#N/A	1.000000E+00
RP24-213L12.1	29743	-0.038406171	3065	5.892384E-01
RP24-215A14.2	29744		#N/A	1.000000E+00
RP24-218M19.	29745	0.050870414	8368.5	4.743803E-01
RP24-219I3.1	29746		#N/A	1.000000E+00
RP24-226I7.1	29747		#N/A	1.000000E+00
RP24-232F23.1	29748		#N/A	1.000000E+00
RP24-232F23.2	29749		#N/A	1.000000E+00
RP24-240G4.1	29750		#N/A	1.000000E+00
RP24-242N1.1	29751	0.033325736	7259	6.394463E-01
RP24-243P7.6	29752		#N/A	1.000000E+00
RP24-243P7.7	29753		#N/A	1.000000E+00
RP24-245K18.1	29754		#N/A	1.000000E+00
RP24-246F8.1	29755	0.113548679	13094	1.093908E-01

Spearman Rank correlation analysis performed between Prdm1 and all-expressed genes within the Meredith RNA-seq dataset. Robust Prdm1–associated genes were identified using a cut-off of $p < 0.0005$.

Table S1, Related to Supplemental Figure 3C. Prdm1 associated genes

RP24-251F16.3	29756		#N/A	1.000000E+00
RP24-252H17.1	29757		#N/A	1.000000E+00
RP24-255F16.1	29758		#N/A	1.000000E+00
RP24-257G18.1	29759		#N/A	1.000000E+00
RP24-258M24.	29760	0.082762295	10811.5	2.439788E-01
RP24-263I14.7	29761		#N/A	1.000000E+00
RP24-267K12.1	29762		#N/A	1.000000E+00
RP24-271M20.	29763	0.193715787	17982	5.987469E-03
RP24-271M20.	29764		#N/A	1.000000E+00
RP24-271M20.	29765		#N/A	1.000000E+00
RP24-273O7.1	29766		#N/A	1.000000E+00
RP24-278M16.	29767		#N/A	1.000000E+00
RP24-285L8.1	29768		#N/A	1.000000E+00
RP24-286J21.4	29769		#N/A	1.000000E+00
RP24-286J21.5	29770		#N/A	1.000000E+00
RP24-289G15.1	29771		#N/A	1.000000E+00
RP24-290G2.1	29772		#N/A	1.000000E+00
RP24-294G11.2	29773		#N/A	1.000000E+00
RP24-310O9.1	29774		#N/A	1.000000E+00
RP24-313O6.1	29775		#N/A	1.000000E+00
RP24-317K11.2	29776		#N/A	1.000000E+00
RP24-317K15.1	29777		#N/A	1.000000E+00
RP24-323K23.2	29778	-0.038406171	3065	5.892384E-01
RP24-326F11.1	29779		#N/A	1.000000E+00
RP24-331K22.1	29780		#N/A	1.000000E+00
RP24-333N14.1	29781		#N/A	1.000000E+00
RP24-335D17.1	29782	-0.038406171	3065	5.892384E-01
RP24-335D17.2	29783	-0.038406171	3065	5.892384E-01
RP24-339K18.1	29784		#N/A	1.000000E+00
RP24-340J12.1	29785	-0.054451513	1703	4.437940E-01
RP24-341L14.1	29786	0.022198316	6629.5	7.550388E-01
RP24-344E5.1	29787		#N/A	1.000000E+00
RP24-347J6.1	29788	-0.038406171	3065	5.892384E-01
RP24-350A15.2	29789		#N/A	1.000000E+00
RP24-352J7.1	29790	-0.038406171	3065	5.892384E-01
RP24-357L20.1	29791		#N/A	1.000000E+00
RP24-361E14.1	29792		#N/A	1.000000E+00
RP24-363L9.4	29793	-0.066856562	1139	3.468991E-01
RP24-363O21.2	29794		#N/A	1.000000E+00
RP24-364D22.1	29795		#N/A	1.000000E+00
RP24-365B9.1	29796		#N/A	1.000000E+00
RP24-372H7.1	29797		#N/A	1.000000E+00
RP24-372J15.1	29798	0.073928833	10125	2.981622E-01

Spearman Rank correlation analysis performed between Prdm1 and all-expressed genes within the Meredith RNA-seq dataset. Robust Prdm1–associated genes were identified using a cut-off of $p < 0.0005$.

Table S1, Related to Supplemental Figure 3C. Prdm1 associated genes

RP24-376E16.2	29799		#N/A	1.000000E+00
RP24-378P20.1	29800		#N/A	1.000000E+00
RP24-391C9.1	29801		#N/A	1.000000E+00
RP24-409D4.2	29802		#N/A	1.000000E+00
RP24-410M8.1	29803		#N/A	1.000000E+00
RP24-413G2.4	29804	0.052728522	8550.5	4.583686E-01
RP24-414A22.2	29805		#N/A	1.000000E+00
RP24-414A22.4	29806		#N/A	1.000000E+00
RP24-414A22.6	29807	-0.038406171	3065	5.892384E-01
RP24-414A22.8	29808		#N/A	1.000000E+00
RP24-422C24.2	29809		#N/A	1.000000E+00
RP24-422F20.2	29810		#N/A	1.000000E+00
RP24-426K19.1	29811		#N/A	1.000000E+00
RP24-426O7.1	29812		#N/A	1.000000E+00
RP24-435G18.1	29813		#N/A	1.000000E+00
RP24-448C16.1	29814		#N/A	1.000000E+00
RP24-448C16.2	29815	0.016300249	6136	8.187985E-01
RP24-456B4.1	29816		#N/A	1.000000E+00
RP24-456G16.3	29817		#N/A	1.000000E+00
RP24-465N15.1	29818	0.1189881	13586	9.331480E-02
RP24-465N15.2	29819	0.081085405	10671.5	2.536978E-01
RP24-469I17.1	29820		#N/A	1.000000E+00
RP24-469I17.2	29821		#N/A	1.000000E+00
RP24-481H2.2	29822		#N/A	1.000000E+00
RP24-484D12.1	29823		#N/A	1.000000E+00
RP24-488N15.1	29824		#N/A	1.000000E+00
RP24-493B12.1	29825	0.120129743	13671	9.019236E-02
RP24-496O18.1	29826		#N/A	1.000000E+00
RP24-499C1.1	29827	0.156129434	16056.5	2.726377E-02
RP24-500L4.2	29828		#N/A	1.000000E+00
RP24-510G5.4	29829		#N/A	1.000000E+00
RP24-539G2.1	29830	0.073115751	10073	3.035222E-01
RP24-541A1.2	29831		#N/A	1.000000E+00
RP24-542I6.1	29832	0.143605682	15224	4.249002E-02
RP24-546F2.1	29833	-0.018105422	4347	7.991361E-01
RP24-547D11.2	29834	0.112205135	12867	1.136785E-01
RP24-547D11.3	29835	0.093022561	11588	1.901504E-01
RP24-547D11.4	29836		#N/A	1.000000E+00
RP24-547D11.5	29837		#N/A	1.000000E+00
RP24-547D11.6	29838		#N/A	1.000000E+00
RP24-548J11.1	29839		#N/A	1.000000E+00
RP24-576J4.1	29840		#N/A	1.000000E+00
RP24-62G16.1	29841		#N/A	1.000000E+00

Spearman Rank correlation analysis performed between Prdm1 and all-expressed genes within the Meredith RNA-seq dataset. Robust Prdm1–associated genes were identified using a cut-off of $p < 0.0005$.

Table S1, Related to Supplemental Figure 3C. Prdm1 associated genes

RP24-64D24.2	29842	0.215376689	18759	2.192608E-03
RP24-64D24.4	29843	0.246487079	19462	4.340792E-04
RP24-67K5.1	29844	-0.077397959	691.5	2.760011E-01
RP24-72E12.1	29845	0.157047659	16110	2.635994E-02
RP24-76E2.5	29846		#N/A	1.000000E+00
RP24-78M9.1	29847		#N/A	1.000000E+00
RP24-78O14.3	29848		#N/A	1.000000E+00
RP24-80K11.4	29849	-0.038406171	3065	5.892384E-01
RP24-80O2.1	29850		#N/A	1.000000E+00
RP24-81A22.1	29851		#N/A	1.000000E+00
RP24-82B23.2	29852		#N/A	1.000000E+00
RP24-90K8.1	29853		#N/A	1.000000E+00
RP24-94D24.1	29854		#N/A	1.000000E+00
RP24-96N12.1	29855	-0.054451513	1703	4.437940E-01
RP24-98O8.1	29856		#N/A	1.000000E+00
Rp2h	29857	0.17150133	16999	1.517352E-02
Rp9	29858	0.159890383	16269	2.372287E-02
Rpa1	29859	0.258641597	19607	2.173564E-04
Rpa2	29860	0.10555446	12397	1.368650E-01
Rpa3	29861	0.207577428	18515	3.184111E-03
Rpain	29862	0.073037221	10067	3.040433E-01
Rpap1	29863	0.087642716	11175	2.171797E-01
Rpap2	29864	-0.051277969	2176	4.708424E-01
Rpap3	29865	0.16822593	16836	1.725837E-02
Rpe	29866	0.120840163	13715	8.829184E-02
Rpe65	29867	-0.038406171	3065	5.892384E-01
Rpf1	29868	0.088623707	11244	2.120568E-01
Rpf2	29869	0.23461405	19265	8.259656E-04
Rpgr	29870	0.200814634	18278	4.354773E-03
Rpgrip1	29871	0.106568168	12464	1.331138E-01
Rpgrip1l	29872	-0.001723524	5089	9.806759E-01
Rph3a	29873	0.016618366	6152	8.153249E-01
Rph3al	29874	0.14204943	15082	4.480541E-02
Rpia	29875	0.214823778	18733	2.252335E-03
Rpl10	29876	0.206604469	18481	3.332793E-03
Rpl10-ps1	29877		#N/A	1.000000E+00
Rpl10-ps2	29878	-0.030877746	3889	6.642567E-01
Rpl10-ps3	29879	0.14447869	15333	4.123572E-02
Rpl10-ps4	29880	0.068272528	9723	3.367590E-01
Rpl10-ps6	29881	0.053267784	8685	4.537786E-01
Rpl10a	29882	0.198191809	18175	4.904378E-03
Rpl10a-ps1	29883	0.150111982	15658	3.386847E-02
Rpl10a-ps2	29884	0.122773898	13831	8.328063E-02

Spearman Rank correlation analysis performed between Prdm1 and all-expressed genes within the Meredith RNA-seq dataset. Robust Prdm1–associated genes were identified using a cut-off of $p < 0.0005$.

Table S1, Related to Supplemental Figure 3C. Prdm1 associated genes

Rpl10l	29885	-0.017005976	4413	8.110973E-01
Rpl11	29886	0.230277084	19172	1.036615E-03
Rpl12	29887	0.161311351	16441	2.249210E-02
Rpl12-ps1	29888	0.082269134	10768	2.468098E-01
Rpl12-ps2	29889	0.115284896	13343	1.040390E-01
Rpl13	29890		#N/A	1.000000E+00
Rpl13-ps1	29891		#N/A	1.000000E+00
Rpl13-ps3	29892	0.228459609	19105	1.138743E-03
Rpl13-ps5	29893	-0.018105422	4347	7.991361E-01
Rpl13a	29894	0.241039055	19377	5.854301E-04
Rpl13a-ps1	29895	0.077321138	10432	2.764794E-01
Rpl14	29896	0.185078248	17641	8.697987E-03
Rpl14-ps1	29897	0.136509632	14726	5.392113E-02
Rpl15	29898	0.113409632	12949	1.098285E-01
Rpl15-ps1	29899	0.020875719	6412.5	7.692098E-01
Rpl15-ps3	29900		#N/A	1.000000E+00
Rpl17	29901	0.102592637	12218	1.482889E-01
Rpl17-ps1	29902	0.081085405	10671.5	2.536978E-01
Rpl17-ps4	29903	0.099924314	12062	1.591869E-01
Rpl17-ps5	29904	0.082029217	10750	2.481952E-01
Rpl18	29905	0.123371151	13868	8.177994E-02
Rpl18-ps1	29906		#N/A	1.000000E+00
Rpl18-ps2	29907	-0.017074363	4409	8.103520E-01
Rpl18a	29908	0.155747018	16021	2.764794E-02
Rpl18a-ps1	29909	0.09093801	11427	2.003160E-01
Rpl19	29910	0.162784315	16513	2.127483E-02
Rpl19-ps1	29911	-0.054451513	1703	4.437940E-01
Rpl19-ps11	29912	0.053267784	8685	4.537786E-01
Rpl19-ps12	29913	-0.054451513	1703	4.437940E-01
Rpl19-ps4	29914	0.113548679	13094	1.093908E-01
Rpl21	29915	0.196678742	18108	5.249036E-03
Rpl21-ps10	29916	-0.066858259	1029.5	3.468868E-01
Rpl21-ps11	29917	-0.054451513	1703	4.437940E-01
Rpl21-ps12	29918		#N/A	1.000000E+00
Rpl21-ps13	29919		#N/A	1.000000E+00
Rpl21-ps14	29920	-0.000841282	5208.5	9.905669E-01
Rpl21-ps15	29921		#N/A	1.000000E+00
Rpl21-ps3	29922	-0.018105422	4347	7.991361E-01
Rpl21-ps4	29923	-0.038406171	3065	5.892384E-01
Rpl21-ps5	29924	0.090555233	11385.5	2.022247E-01
Rpl21-ps6	29925	0.12709556	14123	7.290639E-02
Rpl21-ps7	29926	0.153226271	15850	3.029803E-02
Rpl21-ps8	29927		#N/A	1.000000E+00

Spearman Rank correlation analysis performed between Prdm1 and all-expressed genes within the Meredith RNA-seq dataset. Robust Prdm1–associated genes were identified using a cut-off of $p < 0.0005$.

Table S1, Related to Supplemental Figure 3C. Prdm1 associated genes

Rpl21-ps9	29928	-0.038406171	3065	5.892384E-01
Rpl22	29929	0.178274044	17317	1.154885E-02
Rpl22-ps1	29930	-0.000810573	5230	9.909112E-01
Rpl22l1	29931	0.155202876	15990	2.820257E-02
Rpl23	29932	0.174356382	17139	1.353930E-02
Rpl23a	29933	0.163203596	16554	2.093892E-02
Rpl23a-ps1	29934	-0.053977579	2113.5	4.477765E-01
Rpl23a-ps12	29935	0.113548679	13094	1.093908E-01
Rpl23a-ps13	29936	-0.038406171	3065	5.892384E-01
Rpl23a-ps2	29937		#N/A	1.000000E+00
Rpl23a-ps3	29938	0.182398428	17520.5	9.736288E-03
Rpl23a-ps4	29939	0.01287414	5926	8.564193E-01
Rpl23a-ps6	29940	0.1544596	15936.5	2.897553E-02
Rpl24	29941	0.232670809	19224	9.149326E-04
Rpl26	29942	0.168624904	16858	1.699175E-02
Rpl26-ps2	29943	-0.054450825	1984	4.437997E-01
Rpl26-ps4	29944	-0.095280123	319	1.795721E-01
Rpl26-ps5	29945	-0.038406171	3065	5.892384E-01
Rpl27	29946	0.177854541	17304	1.174889E-02
Rpl27-ps1	29947	-0.038406171	3065	5.892384E-01
Rpl27-ps2	29948	0.015676693	6106.5	8.256174E-01
Rpl27-ps3	29949	0.110513727	12760	1.192618E-01
Rpl27a	29950	0.210694616	18599	2.747281E-03
Rpl27a-ps1	29951	0.025831533	6863	7.165423E-01
Rpl27a-ps2	29952	-0.025939652	4068	7.154070E-01
Rpl28	29953	0.206252253	18467	3.388144E-03
Rpl28-ps1	29954	0.11201494	12855	1.142959E-01
Rpl28-ps3	29955	0.164478601	16623.5	1.994547E-02
Rpl28-ps4	29956	-0.000841282	5208.5	9.905669E-01
Rpl29	29957	0.203501532	18370	3.849814E-03
Rpl29-ps1	29958		#N/A	1.000000E+00
Rpl29-ps2	29959		#N/A	1.000000E+00
Rpl29-ps5	29960	0.157799267	16153.5	2.563935E-02
Rpl3	29961	0.196279547	18095	5.343515E-03
Rpl3-ps1	29962	0.133747073	14532	5.901183E-02
Rpl3-ps2	29963	0.089371505	11299	2.082104E-01
Rpl30	29964	0.207235435	18500	3.235675E-03
Rpl30-ps1	29965		#N/A	1.000000E+00
Rpl30-ps10	29966		#N/A	1.000000E+00
Rpl30-ps11	29967	0.046994573	8076	5.087386E-01
Rpl30-ps2	29968	0.083452862	10867	2.400529E-01
Rpl30-ps3	29969	-0.001224019	5151	9.862757E-01
Rpl30-ps5	29970	-0.038406171	3065	5.892384E-01

Spearman Rank correlation analysis performed between Prdm1 and all-expressed genes within the Meredith RNA-seq dataset. Robust Prdm1–associated genes were identified using a cut-off of $p < 0.0005$.

Table S1, Related to Supplemental Figure 3C. Prdm1 associated genes

Rpl30-ps8	29971		#N/A	1.000000E+00
Rpl30-ps9	29972	0.000377196	5334	9.957705E-01
Rpl31	29973	0.207202427	18498	3.240691E-03
Rpl31-ps1	29974	0.235570475	19280	7.851676E-04
Rpl31-ps10	29975	0.111705112	12833	1.153074E-01
Rpl31-ps11	29976	0.04046805	7660	5.693897E-01
Rpl31-ps12	29977	0.137437524	14785	5.229510E-02
Rpl31-ps13	29978	-0.045401505	2316	5.232270E-01
Rpl31-ps14	29979	0.022198316	6629.5	7.550388E-01
Rpl31-ps15	29980	0.03100427	7130	6.629651E-01
Rpl31-ps16	29981	0.111469887	12817.5	1.160799E-01
Rpl31-ps17	29982	0.084085758	10927	2.364937E-01
Rpl31-ps18	29983	-0.038406171	3065	5.892384E-01
Rpl31-ps19	29984	-0.032450477	3820	6.482730E-01
Rpl31-ps20	29985	-0.032762514	3799	6.451205E-01
Rpl31-ps21	29986	-0.054451513	1703	4.437940E-01
Rpl31-ps22	29987	-0.066858259	1029.5	3.468868E-01
Rpl31-ps23	29988	-0.066156793	1302	3.519808E-01
Rpl31-ps24	29989	0.099271295	12017.5	1.619434E-01
Rpl31-ps25	29990	0.080367521	10622	2.579393E-01
Rpl31-ps4	29991	0.113548679	13094	1.093908E-01
Rpl31-ps5	29992	-0.054451513	1703	4.437940E-01
Rpl31-ps6	29993	0.060437612	9235	3.952489E-01
Rpl31-ps7	29994	0.053944466	8861	4.480555E-01
Rpl31-ps8	29995	-0.068429773	964	3.356447E-01
Rpl31-ps9	29996	0.048654955	8191.5	4.938632E-01
Rpl32	29997	0.188916019	17799	7.382093E-03
Rpl32-ps	29998	0.132773093	14462.5	6.089845E-02
Rpl34	29999	0.177659187	17293	1.184309E-02
Rpl34-ps1	30000		#N/A	1.000000E+00
Rpl35	30001	0.196422535	18101	5.309500E-03
Rpl35-ps1	30002	0.065421341	9556	3.573717E-01
Rpl35a	30003	0.164354907	16612	2.004003E-02
Rpl35a-ps2	30004		#N/A	1.000000E+00
Rpl35a-ps3	30005		#N/A	1.000000E+00
Rpl35a-ps4	30006		#N/A	1.000000E+00
Rpl35a-ps5	30007	0.115453146	13353	1.035316E-01
Rpl35a-ps6	30008		#N/A	1.000000E+00
Rpl35a-ps7	30009	0.140317019	14976.5	4.750617E-02
Rpl36	30010	0.270124781	19720	1.095715E-04
Rpl36-ps1	30011		#N/A	1.000000E+00
Rpl36-ps10	30012	-0.013819265	4574	8.460050E-01
Rpl36-ps2	30013	-0.038406171	3065	5.892384E-01

Spearman Rank correlation analysis performed between Prdm1 and all-expressed genes within the Meredith RNA-seq dataset. Robust Prdm1–associated genes were identified using a cut-off of $p < 0.0005$.

Table S1, Related to Supplemental Figure 3C. Prdm1 associated genes

Rpl36-ps3	30014	0.095293951	11720	1.795087E-01
Rpl36-ps4	30015	-0.038406171	3065	5.892384E-01
Rpl36-ps7	30016	-0.054451513	1703	4.437940E-01
Rpl36-ps8	30017		#N/A	1.000000E+00
Rpl36a	30018	0.184420977	17614	8.943119E-03
Rpl36a-ps1	30019	0.120835043	13714	8.830542E-02
Rpl36a-ps3	30020	-0.077397959	691.5	2.760011E-01
Rpl36al	30021	0.141125296	15024	4.622965E-02
Rpl37	30022	0.214674733	18730	2.268686E-03
Rpl37a	30023	0.209161304	18555	2.954865E-03
Rpl38	30024	0.188863908	17796	7.398702E-03
Rpl38-ps1	30025	-0.077394997	736.5	2.760195E-01
Rpl38-ps2	30026	0.161269753	16437	2.252734E-02
Rpl39	30027	0.224334487	19027	1.405666E-03
Rpl39-ps	30028	0.075715395	10317.5	2.866063E-01
Rpl39l	30029	0.10879498	12610	1.251515E-01
Rpl3l	30030		#N/A	1.000000E+00
Rpl4	30031	0.162211281	16480	2.174145E-02
Rpl41	30032	0.192927533	17947	6.198955E-03
Rpl48-ps1	30033		#N/A	1.000000E+00
Rpl5	30034	0.172497072	17049	1.458497E-02
Rpl5-ps1	30035	0.002568575	5447.5	9.712046E-01
Rpl6	30036	0.221761079	18952	1.600004E-03
Rpl7	30037	0.215463132	18769	2.183402E-03
Rpl7a	30038	0.131280314	14366	6.388602E-02
Rpl7a-ps10	30039	-0.054451513	1703	4.437940E-01
Rpl7a-ps11	30040		#N/A	1.000000E+00
Rpl7a-ps12	30041	0.068620304	9746	3.342977E-01
Rpl7a-ps13	30042	0.021317126	6513.5	7.644715E-01
Rpl7a-ps3	30043	-0.038406171	3065	5.892384E-01
Rpl7a-ps5	30044		#N/A	1.000000E+00
Rpl7a-ps8	30045		#N/A	1.000000E+00
Rpl7a-ps9	30046	0.143605682	15224	4.249002E-02
Rpl7l1	30047	0.14989316	15644	3.413225E-02
Rpl7l1-ps1	30048		#N/A	1.000000E+00
Rpl8	30049	0.180325024	17420	1.061337E-02
Rpl9	30050	0.200328256	18254	4.452302E-03
Rpl9-ps1	30051	0.081140536	10678	2.533741E-01
Rpl9-ps2	30052	0.046994573	8076	5.087386E-01
Rpl9-ps3	30053	0.106334743	12447	1.339705E-01
Rpl9-ps4	30054		#N/A	1.000000E+00
Rpl9-ps5	30055	0.166176687	16727	1.868593E-02
Rpl9-ps6	30056	-0.038406171	3065	5.892384E-01

Spearman Rank correlation analysis performed between Prdm1 and all-expressed genes within the Meredith RNA-seq dataset. Robust Prdm1–associated genes were identified using a cut-off of $p < 0.0005$.

Table S1, Related to Supplemental Figure 3C. Prdm1 associated genes

Rpl9-ps7	30057	-0.038406171	3065	5.892384E-01
Rplp0	30058	0.155767873	16022	2.762687E-02
Rplp0-ps1	30059	0.189988431	17842.5	7.047572E-03
Rplp1	30060	0.167221767	16783	1.794558E-02
Rplp1-ps1	30061	0.205115223	18430	3.572546E-03
Rplp2	30062	0.133716461	14529	5.907038E-02
Rpn1	30063	0.217573137	18835	1.969306E-03
Rpn2	30064	0.087937939	11201	2.156288E-01
Rpp14	30065	0.079669798	10573	2.621081E-01
Rpp21	30066	0.101228385	12133	1.537879E-01
Rpp25	30067		#N/A	1.000000E+00
Rpp25l	30068	0.025554386	6851	7.194552E-01
Rpp30	30069	0.189070892	17805	7.332925E-03
Rpp38	30070	-0.007793521	4829	9.127838E-01
Rpp40	30071	-0.060071133	1395	3.981261E-01
Rpph1	30072		#N/A	1.000000E+00
Rprd1a	30073	0.185685838	17666	8.476702E-03
Rprd1b	30074	0.205738842	18445	3.470316E-03
Rprd2	30075	0.130101402	14303	6.632941E-02
Rprl1	30076		#N/A	1.000000E+00
Rprl2	30077		#N/A	1.000000E+00
Rprl3	30078		#N/A	1.000000E+00
Rprm	30079	0.033504894	7267	6.376457E-01
Rprml	30080		#N/A	1.000000E+00
Rps10	30081	0.188372873	17777	7.556851E-03
Rps10-ps1	30082		#N/A	1.000000E+00
Rps10-ps2	30083	0.06687767	9638	3.467465E-01
Rps10-ps3	30084	0.140002704	14954	4.801044E-02
Rps10-ps4	30085	0.085764067	11042	2.272355E-01
Rps11	30086	0.173092137	17083	1.424287E-02
Rps11-ps1	30087	0.125958255	14040.5	7.552903E-02
Rps11-ps2	30088	-0.050949714	2182	4.736907E-01
Rps11-ps3	30089	0.17803054	17309	1.166460E-02
Rps11-ps4	30090	0.252384649	19528	3.116370E-04
Rps11-ps5	30091	-0.054451513	1703	4.437940E-01
Rps12	30092	0.190005555	17845	7.042342E-03
Rps12-ps1	30093	0.168220295	16835	1.726216E-02
Rps12-ps10	30094	0.089711012	11326	2.064807E-01
Rps12-ps11	30095		#N/A	1.000000E+00
Rps12-ps12	30096		#N/A	1.000000E+00
Rps12-ps13	30097		#N/A	1.000000E+00
Rps12-ps14	30098		#N/A	1.000000E+00
Rps12-ps15	30099		#N/A	1.000000E+00

Spearman Rank correlation analysis performed between Prdm1 and all-expressed genes within the Meredith RNA-seq dataset. Robust Prdm1–associated genes were identified using a cut-off of $p < 0.0005$.

Table S1, Related to Supplemental Figure 3C. Prdm1 associated genes

Rps12-ps16	30100		#N/A	1.000000E+00
Rps12-ps17	30101		#N/A	1.000000E+00
Rps12-ps18	30102		#N/A	1.000000E+00
Rps12-ps19	30103	0.252400019	19529	3.113647E-04
Rps12-ps20	30104		#N/A	1.000000E+00
Rps12-ps21	30105	-0.038406171	3065	5.892384E-01
Rps12-ps22	30106		#N/A	1.000000E+00
Rps12-ps23	30107	-0.004937246	4940	9.446824E-01
Rps12-ps26	30108		#N/A	1.000000E+00
Rps12-ps3	30109		#N/A	1.000000E+00
Rps12-ps5	30110	0.091818189	11509	1.959767E-01
Rps12-ps6	30111		#N/A	1.000000E+00
Rps12-ps7	30112	0.120831424	13713	8.831502E-02
Rps12-ps9	30113	-0.066858259	1029.5	3.468868E-01
Rps12l1	30114	0.113548679	13094	1.093908E-01
Rps12l2	30115	0.179443661	17379	1.100685E-02
Rps13	30116	0.232589217	19222	9.188541E-04
Rps13-ps1	30117		#N/A	1.000000E+00
Rps13-ps2	30118	0.113548679	13094	1.093908E-01
Rps13-ps4	30119		#N/A	1.000000E+00
Rps13-ps5	30120	0.197667896	18147	5.021349E-03
Rps13-ps6	30121		#N/A	1.000000E+00
Rps14	30122	0.176759713	17254	1.228542E-02
Rps15	30123	0.23776398	19320	6.985122E-04
Rps15-ps2	30124	0.160987082	16412.5	2.276801E-02
Rps15a	30125	0.157049022	16111	2.635862E-02
Rps15a-ps1	30126	0.065421341	9556	3.573717E-01
Rps15a-ps2	30127	-0.066856562	1139	3.468991E-01
Rps15a-ps3	30128	0.101898822	12175	1.510666E-01
Rps15a-ps4	30129	0.119727097	13641	9.128395E-02
Rps15a-ps5	30130	0.11012449	12726.5	1.205764E-01
Rps15a-ps6	30131	0.100724442	12104	1.558577E-01
Rps15a-ps7	30132		#N/A	1.000000E+00
Rps15a-ps8	30133	-0.054451513	1703	4.437940E-01
Rps16	30134	0.23380816	19253	8.618478E-04
Rps16-ps2	30135	0.088537719	11239	2.125024E-01
Rps16-ps3	30136	0.084819371	10974.5	2.324148E-01
Rps17	30137	0.169776571	16915	1.624216E-02
Rps18	30138	0.166677704	16756	1.832776E-02
Rps18-ps1	30139	-0.095280123	319	1.795721E-01
Rps18-ps2	30140	-0.054451513	1703	4.437940E-01
Rps18-ps3	30141		#N/A	1.000000E+00
Rps19	30142	0.157703854	16136	2.572988E-02

Spearman Rank correlation analysis performed between Prdm1 and all-expressed genes within the Meredith RNA-seq dataset. Robust Prdm1–associated genes were identified using a cut-off of $p < 0.0005$.

Table S1, Related to Supplemental Figure 3C. Prdm1 associated genes

Rps19-ps1	30143		#N/A	1.000000E+00
Rps19-ps10	30144	0.1544596	15936.5	2.897553E-02
Rps19-ps11	30145	0.015627066	6100	8.261607E-01
Rps19-ps12	30146	0.138239432	14838	5.092282E-02
Rps19-ps13	30147	0.139781044	14939	4.836875E-02
Rps19-ps2	30148		#N/A	1.000000E+00
Rps19-ps3	30149	0.159368427	16246	2.418931E-02
Rps19-ps4	30150	0.012177115	5885	8.641158E-01
Rps19-ps5	30151	-0.066858259	1029.5	3.468868E-01
Rps19-ps6	30152		#N/A	1.000000E+00
Rps19-ps7	30153	0.05927809	9173	4.043950E-01
Rps19-ps8	30154	-0.052176377	2158	4.630948E-01
Rps19-ps9	30155	0.010732176	5807	8.801107E-01
Rps19bp1	30156	0.157623255	16134	2.580657E-02
Rps2	30157	0.222980074	18988	1.505078E-03
Rps2-ps10	30158	-0.038406171	3065	5.892384E-01
Rps2-ps13	30159	0.044635397	7909	5.302686E-01
Rps2-ps4	30160		#N/A	1.000000E+00
Rps2-ps6	30161	0.1544596	15936.5	2.897553E-02
Rps2-ps9	30162		#N/A	1.000000E+00
Rps20	30163	0.18134889	17460	1.017191E-02
Rps21	30164	0.216258258	18794	2.100354E-03
Rps23	30165	0.188025714	17763	7.670470E-03
Rps23-ps	30166	0.004847219	5553	9.456895E-01
Rps24	30167	0.18843184	17779	7.537702E-03
Rps24-ps2	30168	-0.03367206	3757.5	6.359676E-01
Rps24-ps3	30169	0.078670469	10508	2.681591E-01
Rps25	30170	0.201024407	18287	4.313305E-03
Rps25-ps1	30171	0.22141422	18945	1.627997E-03
Rps26	30172	0.174407426	17141	1.351154E-02
Rps26-ps1	30173		#N/A	1.000000E+00
Rps27	30174	0.255378727	19569	2.625796E-04
Rps27a	30175	0.11834371	13549	9.511488E-02
Rps27a-ps2	30176		#N/A	1.000000E+00
Rps27l	30177	0.210385501	18588	2.788034E-03
Rps28	30178	0.176834499	17259	1.224810E-02
Rps29	30179	0.227681302	19095	1.185236E-03
Rps3	30180	0.175203997	17175	1.308482E-02
Rps3a1	30181	0.135032179	14622	5.659649E-02
Rps3a2	30182	0.21549506	18774.5	2.180011E-03
Rps3a3	30183		#N/A	1.000000E+00
Rps4x	30184	0.153749146	15877	2.973119E-02
Rps4y2	30185	0.016035577	6127	8.216912E-01

Spearman Rank correlation analysis performed between Prdm1 and all-expressed genes within the Meredith RNA-seq dataset. Robust Prdm1–associated genes were identified using a cut-off of $p < 0.0005$.

Table S1, Related to Supplemental Figure 3C. Prdm1 associated genes

Rps5	30186	0.195302854	18058	5.581131E-03
Rps6	30187	0.177566452	17290	1.188804E-02
Rps6-ps1	30188	0.113548679	13094	1.093908E-01
Rps6-ps2	30189	0.091347262	11476	1.982898E-01
Rps6-ps3	30190	-0.038406171	3065	5.892384E-01
Rps6-ps4	30191		#N/A	1.000000E+00
Rps6ka1	30192	0.294778845	19884	2.263170E-05
Rps6ka2	30193	0.078224046	10480	2.708927E-01
Rps6ka3	30194	0.094136307	11650	1.848761E-01
Rps6ka4	30195	0.135349077	14650	5.601360E-02
Rps6ka5	30196	0.201569631	18313	4.207183E-03
Rps6ka6	30197	0.143772807	15295	4.224746E-02
Rps6kb1	30198	0.374932591	20021	4.501193E-08
RPS6KB2	30199		#N/A	1.000000E+00
Rps6kb2	30200	-0.034154638	3732	6.311335E-01
Rps6kc1	30201	-0.038097486	3630	5.922369E-01
Rps6kl1	30202	-0.081961917	621	2.485848E-01
Rps7	30203	0.155480938	16006	2.791797E-02
Rps8	30204	0.210326122	18586	2.795925E-03
Rps8-ps1	30205	-0.038406171	3065	5.892384E-01
Rps8-ps2	30206		#N/A	1.000000E+00
Rps9	30207	0.172060953	17024	1.484024E-02
Rpsa	30208	0.146075134	15419	3.902248E-02
Rpsa-ps10	30209	-0.041514627	2444	5.594375E-01
Rpsa-ps11	30210	0.038071936	7486	5.924855E-01
Rpsa-ps12	30211	0.068244511	9720	3.369578E-01
Rpsa-ps2	30212		#N/A	1.000000E+00
Rpsa-ps3	30213	-0.066858259	1029.5	3.468868E-01
Rpsa-ps4	30214	-0.024553063	4114	7.300129E-01
Rpsa-ps5	30215	0.156159263	16067	2.723399E-02
Rpsa-ps9	30216	-0.066856562	1139	3.468991E-01
Rptn	30217	0.000176606	5299	9.980197E-01
Rptor	30218	0.037056262	7433	6.024024E-01
Rpusd1	30219	-0.089437298	424	2.078744E-01
Rpusd2	30220	0.043247263	7819	5.431479E-01
Rpusd3	30221	-0.018790377	4285	7.917077E-01
Rpusd4	30222	0.162905054	16537	2.117763E-02
Rqcd1	30223	0.315233421	19955	5.452192E-06
Rrad	30224	0.12215124	13795	8.486864E-02
Rraga	30225	0.070665558	9886	3.200566E-01
Rragb	30226	0.218976822	18873	1.837665E-03
Rragc	30227	0.185018309	17639	8.720092E-03
Rragd	30228	0.048296398	8159	4.970558E-01

Spearman Rank correlation analysis performed between Prdm1 and all-expressed genes within the Meredith RNA-seq dataset. Robust Prdm1–associated genes were identified using a cut-off of $p < 0.0005$.

Table S1, Related to Supplemental Figure 3C. Prdm1 associated genes

Rras	30229	0.119123727	13595	9.293941E-02
Rras2	30230	0.086471351	11104	2.234116E-01
Rrbp1	30231	0.202101367	18331	4.105956E-03
Rreb1	30232	0.114680932	13304	1.058768E-01
Rrh	30233	-0.038406171	3065	5.892384E-01
Rrm1	30234	0.247648643	19477	4.069069E-04
Rrm2	30235	0.191945408	17907	6.471780E-03
Rrm2b	30236	0.148345921	15552	3.604747E-02
Rrn3	30237	0.007706345	5667	9.137556E-01
Rrnad1	30238	-0.015709047	4490	8.252633E-01
Rrp1	30239	0.060430847	9234	3.953019E-01
Rrp12	30240	0.040934736	7690	5.649415E-01
Rrp15	30241	0.088762333	11254	2.113400E-01
Rrp1b	30242	-0.018414892	4324	7.957776E-01
Rrp36	30243	-0.024984407	4100	7.254586E-01
Rrp7a	30244	-0.080604765	634	2.565322E-01
Rrp8	30245	0.309314786	19937	8.321671E-06
Rrp9	30246	0.290594313	19866	2.989009E-05
Rrs1	30247	0.067924585	9699	3.392330E-01
Rs1	30248	0.127303387	14140	7.243522E-02
Rsad1	30249	0.141356459	15039	4.586989E-02
Rsad2	30250	0.117735701	13510	9.683853E-02
Rsbn1	30251	0.217468423	18833	1.979463E-03
Rsbn1l	30252	0.140083981	14960	4.787962E-02
Rsc1a1	30253		#N/A	1.000000E+00
Rsf1	30254	0.112445218	12884	1.129028E-01
Rsg1	30255	-0.017074363	4409	8.103520E-01
Rsl1	30256	-0.054451513	1703	4.437940E-01
Rsl1d1	30257	0.073433856	10089	3.014177E-01
Rsl24d1	30258	0.213452813	18697	2.406864E-03
Rslcan18	30259	0.084636591	10962	2.334264E-01
Rsph1	30260	-0.051006447	2181	4.731978E-01
Rsph10b2	30261	-0.077394997	736.5	2.760195E-01
Rsph3a	30262	0.164926793	16653	1.960608E-02
Rsph3b	30263	0.118241086	13544	9.540409E-02
Rsph4a	30264	0.125181758	13980	7.736315E-02
Rsph6a	30265	0.160304018	16323	2.335876E-02
Rsph9	30266	0.053267784	8685	4.537786E-01
Rspo1	30267	0.053267784	8685	4.537786E-01
Rspo2	30268	-0.012506924	4644	8.604724E-01
Rspo3	30269	-0.095267847	349	1.796284E-01
Rspo4	30270		#N/A	1.000000E+00
Rspry1	30271	0.158365173	16192	2.510803E-02

Spearman Rank correlation analysis performed between Prdm1 and all-expressed genes within the Meredith RNA-seq dataset. Robust Prdm1–associated genes were identified using a cut-off of $p < 0.0005$.

Table S1, Related to Supplemental Figure 3C. Prdm1 associated genes

Rsrc1	30272	0.103929968	12293	1.430443E-01
Rsrc2	30273	0.211723834	18644	2.615455E-03
Rsu1	30274	-0.035677815	3696	6.159787E-01
Rtbdn	30275		#N/A	1.000000E+00
Rtca	30276	-0.000991138	5185	9.888867E-01
Rtdr1	30277	-0.077397959	691.5	2.760011E-01
Rtel1	30278	-0.031995957	3846	6.528762E-01
Rtf1	30279	-0.050040246	2196	4.816313E-01
Rtfdc1	30280	0.085415865	11016	2.291349E-01
Rtkn	30281	-0.090350803	413	2.032494E-01
Rtkn2	30282	0.170006207	16930	1.609620E-02
Rtl1	30283	0.031870553	7180	6.541486E-01
Rtn1	30284	0.014735263	6055	8.359371E-01
Rtn2	30285	0.050882593	8374	4.742743E-01
Rtn3	30286	0.172150959	17031	1.478724E-02
Rtn4	30287	0.096779553	11822	1.727902E-01
Rtn4ip1	30288	0.014144083	5996	8.424319E-01
Rtn4r	30289	-0.049697631	2203	4.846412E-01
Rtn4rl1	30290	0.048289833	8157	4.971144E-01
Rtn4rl2	30291		#N/A	1.000000E+00
Rtp1	30292	-5.84624E-05	5288	9.993445E-01
Rtp2	30293		#N/A	1.000000E+00
Rtp3	30294		#N/A	1.000000E+00
Rtp4	30295	0.117709574	13508	9.691315E-02
Rttn	30296	0.04641467	8042	5.139884E-01
Rufy1	30297	0.027261062	6943	7.015831E-01
Rufy2	30298	0.153290177	15853	3.022826E-02
Rufy3	30299	0.179164924	17366	1.113394E-02
Rufy4	30300	-0.038406171	3065	5.892384E-01
Rundc1	30301	-0.054521472	1533	4.432078E-01
Rundc3a	30302	0.053805701	8820	4.492258E-01
Rundc3b	30303	0.195580598	18068	5.512615E-03
Runx1	30304	0.117152788	13472	9.851423E-02
Runx1t1	30305	0.193423137	17966	6.065221E-03
Runx2	30306	0.242612752	19408	5.373401E-04
Runx3	30307	0.127204819	14128	7.265837E-02
Rusc1	30308	0.222814716	18978	1.517646E-03
Rusc2	30309	0.032321525	7197	6.495776E-01
Ruvbl1	30310	0.062259017	9341	3.811352E-01
Ruvbl2	30311	0.316567426	19958	4.950340E-06
Rwdd1	30312	0.171963877	17017	1.489759E-02
Rwdd2a	30313	-0.04614375	2283	5.164505E-01
Rwdd2b	30314	0.21740319	18832	1.985814E-03

Spearman Rank correlation analysis performed between Prdm1 and all-expressed genes within the Meredith RNA-seq dataset. Robust Prdm1–associated genes were identified using a cut-off of $p < 0.0005$.

Table S1, Related to Supplemental Figure 3C. Prdm1 associated genes

Rwdd3	30315	0.065484021	9561	3.569103E-01
Rwdd4a	30316	0.061929585	9328	3.836649E-01
Rxfp1	30317	-0.066856562	1139	3.468991E-01
Rxfp2	30318	0.006282487	5610	9.296448E-01
Rxfp3	30319	-0.077397959	691.5	2.760011E-01
Rxfp4	30320		#N/A	1.000000E+00
Rxra	30321	0.011972544	5873	8.663771E-01
Rxrb	30322	0.088592571	11242	2.122181E-01
Rxrg	30323	0.008665491	5711	9.030711E-01
Rybp	30324	-0.078332759	661	2.702253E-01
Ryk	30325	0.21873529	18865	1.859729E-03
Ryr1	30326	0.011505005	5851	8.715493E-01
Ryr2	30327	-0.004640357	4953	9.480040E-01
Ryr3	30328	-0.030819205	3906	6.648547E-01
S100a1	30329	0.223941504	19016	1.433873E-03
S100a10	30330	0.184299434	17605	8.989115E-03
S100a11	30331	0.15112508	15721	3.266971E-02
S100a13	30332	0.048890921	8212	4.917680E-01
S100a14	30333	0.08154291	10703	2.510201E-01
S100a16	30334	0.146054095	15416	3.905098E-02
S100a2	30335	0.031800255	7177	6.548623E-01
S100a3	30336	0.045782301	7989	5.197448E-01
S100a4	30337	0.146468906	15445	3.849223E-02
S100a5	30338	0.135808742	14682	5.517694E-02
S100a6	30339	0.134336763	14582	5.789317E-02
S100a7a	30340		#N/A	1.000000E+00
S100a8	30341	-0.037096353	3658	6.020096E-01
S100a9	30342	0.03037285	7080	6.694207E-01
S100b	30343	0.090778871	11410	2.011079E-01
S100g	30344	0.040868942	7686	5.655676E-01
S100pbp	30345	0.126702235	14085	7.380490E-02
S100z	30346	-0.049876322	2199	4.830702E-01
S1pr1	30347	-0.029773507	3936	6.755709E-01
S1pr2	30348	0.241079952	19379	5.841316E-04
S1pr3	30349	-0.028669519	3972	6.869558E-01
S1pr4	30350	0.079902447	10585	2.607129E-01
S1pr5	30351	0.133764286	14536	5.897893E-02
Saa1	30352	-0.002462319	5051	9.723953E-01
Saa2	30353	0.009617857	5753	8.924794E-01
Saa3	30354	0.001614269	5398	9.819006E-01
Saa4	30355	0.129314385	14262	6.800271E-02
Saal1	30356	0.074593675	10222	2.938262E-01
Sac3d1	30357	-0.006986758	4853	9.217818E-01

Spearman Rank correlation analysis performed between Prdm1 and all-expressed genes within the Meredith RNA-seq dataset. Robust Prdm1–associated genes were identified using a cut-off of $p < 0.0005$.

Table S1, Related to Supplemental Figure 3C. Prdm1 associated genes

Sacm1l	30358	0.115702375	13376	1.027835E-01
Sacs	30359	0.108395262	12576	1.265530E-01
Sae1	30360	0.148601502	15566	3.572497E-02
Safb	30361	0.057620061	9066	4.176892E-01
Safb2	30362	0.021964979	6588	7.575331E-01
Sag	30363	0.117218072	13476	9.832542E-02
Sall1	30364	-0.000841282	5208.5	9.905669E-01
Sall2	30365	0.053267784	8685	4.537786E-01
Sall3	30366	0.0734655	10094	3.012088E-01
Sall4	30367	0.128389381	14206	7.001325E-02
Samd1	30368	0.115726298	13378	1.027119E-01
Samd10	30369	0.022554025	6662	7.512412E-01
Samd11	30370	0.219481786	18887	1.792306E-03
Samd12	30371	0.196190522	18091	5.364792E-03
Samd13	30372	0.143605682	15224	4.249002E-02
Samd14	30373	0.055251024	8942	4.371212E-01
Samd15	30374	0.064426334	9501	3.647469E-01
Samd3	30375	-0.004691384	4951	9.474330E-01
Samd4	30376	0.031080888	7135	6.621834E-01
Samd4b	30377	-0.003493995	5007	9.608372E-01
Samd5	30378	0.048448014	8175.5	4.957045E-01
Samd7	30379	0.094676268	11684	1.823581E-01
Samd8	30380	-0.012263359	4662	8.631628E-01
Samd9l	30381	-0.041615614	2442	5.584817E-01
Samhd1	30382	0.200052122	18244	4.508543E-03
Samm50	30383	0.19905104	18200	4.717831E-03
Samsn1	30384	0.05892287	9153	4.072218E-01
Samt1	30385		#N/A	1.000000E+00
Samt2	30386		#N/A	1.000000E+00
Samt3	30387		#N/A	1.000000E+00
Samt4	30388		#N/A	1.000000E+00
Sap130	30389	0.041771407	7729	5.570086E-01
Sap18	30390	0.177127729	17272	1.210272E-02
Sap25	30391	0.080203479	10613	2.589153E-01
Sap30	30392	0.054901355	8915	4.400325E-01
Sap30bp	30393	0.116701325	13449	9.982777E-02
Sap30l	30394	0.06176262	9321	3.849509E-01
Sapcd1	30395	0.044980678	7932	5.270891E-01
Sapcd2	30396	0.134079138	14556	5.837972E-02
Sar1a	30397	0.146680336	15458	3.821004E-02
Sar1b	30398	0.184857937	17627	8.779480E-03
Sardh	30399	0.147104693	15483	3.764892E-02
Sarm1	30400	0.126319722	14067	7.468732E-02

Spearman Rank correlation analysis performed between Prdm1 and all-expressed genes within the Meredith RNA-seq dataset. Robust Prdm1–associated genes were identified using a cut-off of $p < 0.0005$.

Table S1, Related to Supplemental Figure 3C. Prdm1 associated genes

Sarnp	30401	0.128727324	14227	6.927317E-02
Sars	30402	0.085214454	11004	2.302387E-01
Sars2	30403	-0.062190798	1378	3.816582E-01
Sart1	30404	0.172065388	17025	1.483763E-02
Sart3	30405	0.216495713	18802	2.076116E-03
Sash1	30406	0.149037994	15593	3.517984E-02
Sash3	30407	0.11807377	13533	9.587710E-02
Sass6	30408	-0.036130471	3683	6.115058E-01
Sat1	30409	0.132879984	14469	6.068901E-02
Sat2	30410		#N/A	1.000000E+00
Satb1	30411	0.139419555	14916	4.895786E-02
Satb2	30412	0.077368975	10434	2.761815E-01
Satl1	30413	-0.077394997	736.5	2.760195E-01
Sav1	30414	-0.006567255	4863	9.264646E-01
Saysd1	30415	-0.10971239	184	1.219804E-01
Sbds	30416	0.236424977	19297	7.502958E-04
Sbf1	30417	0.06086837	9254	3.918831E-01
Sbf2	30418	0.115072324	13327	1.046830E-01
Sbk1	30419	-0.021685065	4184	7.605286E-01
Sbk2	30420	-0.001989556	5073	9.776939E-01
Sbno1	30421	0.066546175	9624	3.491474E-01
Sbno2	30422	0.004778855	5546	9.464543E-01
Sbp	30423	-0.110568513	165.5	1.190777E-01
Sbpl	30424	0.144904304	15349	4.063559E-02
Sbsn	30425	0.120990175	13723	8.789465E-02
Sbspon	30426	0.075711532	10311	2.866309E-01
Sc4mol	30427	0.097104492	11844	1.713459E-01
Sc5d	30428	0.123138292	13855	8.236243E-02
Scaf1	30429	0.24568502	19449	4.538126E-04
Scaf11	30430	0.130402468	14318	6.569828E-02
Scaf4	30431	0.181639235	17468	1.004971E-02
Scaf8	30432	0.205650781	18441	3.484590E-03
Scai	30433	0.10856732	12588	1.259482E-01
Scamp1	30434	0.08219685	10760	2.472266E-01
Scamp2	30435	0.117256545	13478	9.821429E-02
Scamp3	30436	0.063092835	9395	3.747778E-01
Scamp4	30437	0.17226496	17035	1.472035E-02
Scamp5	30438	-0.042203958	2431	5.529286E-01
Scand1	30439	0.128348243	14202	7.010378E-02
Scap	30440	0.21357944	18699	2.392197E-03
Scaper	30441	0.050585787	8350	4.768596E-01
Scara3	30442	-0.010581066	4723	8.817863E-01
Scara5	30443	0.108985454	12621	1.244879E-01

Spearman Rank correlation analysis performed between Prdm1 and all-expressed genes within the Meredith RNA-seq dataset. Robust Prdm1–associated genes were identified using a cut-off of $p < 0.0005$.

Table S1, Related to Supplemental Figure 3C. Prdm1 associated genes

Scarb1	30444	0.161300564	16440	2.250123E-02
Scarb2	30445	0.058937508	9154	4.071051E-01
Scarf1	30446	-0.038406171	3065	5.892384E-01
Scarf2	30447		#N/A	1.000000E+00
Scarna10	30448		#N/A	1.000000E+00
Scarna13	30449		#N/A	1.000000E+00
Scarna17	30450		#N/A	1.000000E+00
Scarna2	30451	0.216415283	18799	2.084297E-03
Scarna3a	30452	-0.077393517	797	2.760287E-01
Scarna3b	30453		#N/A	1.000000E+00
Scarna6	30454		#N/A	1.000000E+00
Scarna8	30455		#N/A	1.000000E+00
Scarna9	30456		#N/A	1.000000E+00
Sccpdh	30457	0.055407169	8951	4.358248E-01
Scd1	30458	0.12153411	13754	8.646644E-02
Scd2	30459	0.114002234	13252	1.079723E-01
Scd3	30460	0.030961859	7128	6.633979E-01
Scd4	30461	-0.023227019	4144	7.440727E-01
Scel	30462	0.140600085	14992	4.705581E-02
Scfd1	30463	0.03636181	7399	6.092254E-01
Scfd2	30464	0.113642456	13236	1.090963E-01
Scg2	30465	-0.077393023	832.5	2.760318E-01
Scg3	30466	0.074574898	10201.5	2.939481E-01
Scg5	30467	0.117588458	13497	9.725966E-02
Scgb1a1	30468	0.164478601	16623.5	1.994547E-02
Scgb1b11	30469		#N/A	1.000000E+00
Scgb1b19	30470		#N/A	1.000000E+00
Scgb1b2	30471		#N/A	1.000000E+00
Scgb1b21	30472		#N/A	1.000000E+00
Scgb1b22-ps	30473		#N/A	1.000000E+00
Scgb1b23-ps	30474		#N/A	1.000000E+00
Scgb1b24	30475		#N/A	1.000000E+00
Scgb1b26-ps	30476		#N/A	1.000000E+00
Scgb1b27	30477		#N/A	1.000000E+00
Scgb1b29	30478		#N/A	1.000000E+00
Scgb1b3	30479		#N/A	1.000000E+00
Scgb1b30	30480		#N/A	1.000000E+00
Scgb1b7	30481		#N/A	1.000000E+00
Scgb1c1	30482	-0.065730248	1314	3.551012E-01
Scgb2a2	30483		#N/A	1.000000E+00
Scgb2b12	30484		#N/A	1.000000E+00
Scgb2b15	30485		#N/A	1.000000E+00
Scgb2b17	30486		#N/A	1.000000E+00

Spearman Rank correlation analysis performed between Prdm1 and all-expressed genes within the Meredith RNA-seq dataset. Robust Prdm1–associated genes were identified using a cut-off of $p < 0.0005$.

Table S1, Related to Supplemental Figure 3C. Prdm1 associated genes

Scgb2b19	30487		#N/A	1.000000E+00
Scgb2b2	30488		#N/A	1.000000E+00
Scgb2b20	30489		#N/A	1.000000E+00
Scgb2b22-ps	30490		#N/A	1.000000E+00
Scgb2b23-ps	30491		#N/A	1.000000E+00
Scgb2b24	30492		#N/A	1.000000E+00
Scgb2b25-ps	30493		#N/A	1.000000E+00
Scgb2b26	30494	-0.038406171	3065	5.892384E-01
Scgb2b27	30495		#N/A	1.000000E+00
Scgb2b3	30496		#N/A	1.000000E+00
Scgb2b7	30497		#N/A	1.000000E+00
Scgb3a1	30498		#N/A	1.000000E+00
Scgb3a2	30499		#N/A	1.000000E+00
Scgn	30500	0.124890888	13962	7.805941E-02
Schip1	30501	-0.00566754	4895	9.365161E-01
Scimp	30502	0.08087306	10655	2.549474E-01
Scin	30503	-0.054809083	1525	4.408026E-01
Sclt1	30504	0.134550137	14593	5.749273E-02
Scly	30505	0.147147518	15486	3.759269E-02
Scmh1	30506	0.053137855	8601	4.548821E-01
Scml1	30507	-0.038406171	3065	5.892384E-01
Scml2	30508	-0.014719183	4525.5	8.361136E-01
Scml4	30509	0.055001257	8926	4.391996E-01
Scn10a	30510	0.090791844	11412	2.010433E-01
Scn11a	30511	0.171312888	16985	1.528721E-02
Scn1a	30512	0.179904787	17397	1.079941E-02
Scn1b	30513	0.14616368	15423	3.890271E-02
Scn2a1	30514	0.063695142	9451	3.702264E-01
Scn2b	30515	0.141181995	15028	4.614119E-02
Scn3a	30516	0.255046662	19566	2.676426E-04
Scn3b	30517	0.022726631	6675	7.494006E-01
Scn4a	30518		#N/A	1.000000E+00
Scn4b	30519	0.01029389	5785	8.849723E-01
Scn5a	30520	-0.093310688	384	1.887755E-01
Scn7a	30521	0.160902334	16383	2.284060E-02
Scn8a	30522	0.111792071	12837	1.150228E-01
Scn9a	30523	0.031442446	7155	6.584997E-01
Scnm1	30524	-0.105646432	204	1.365213E-01
Scnn1a	30525	-0.068244475	966	3.369580E-01
Scnn1b	30526	0.094951925	11696	1.810824E-01
Scnn1g	30527	-0.092281486	397	1.937202E-01
Sco1	30528	0.173798805	17112	1.384575E-02
Sco2	30529	0.001266544	5377	9.857989E-01

Spearman Rank correlation analysis performed between Prdm1 and all-expressed genes within the Meredith RNA-seq dataset. Robust Prdm1–associated genes were identified using a cut-off of $p < 0.0005$.

Table S1, Related to Supplemental Figure 3C. Prdm1 associated genes

Scoc	30530	0.08192072	10739	2.488235E-01
Scp2	30531	-0.077397959	691.5	2.760011E-01
Scp2-ps2	30532		#N/A	1.000000E+00
Scp2d1	30533		#N/A	1.000000E+00
Scpep1	30534	0.304420533	19918	1.172488E-05
Scrg1	30535	-0.066856562	1139	3.468991E-01
Scrib	30536	0.068937076	9770	3.320659E-01
Scrn1	30537	0.132344604	14437	6.174401E-02
Scrn2	30538	0.189416939	17818	7.224119E-03
Scrn3	30539	0.059106382	9161	4.057600E-01
Scrt1	30540	-0.001606757	5107.5	9.819849E-01
Scrt2	30541	-0.018907216	4275	7.904424E-01
Sct	30542	-0.012836372	4631	8.568360E-01
Sctr	30543	-0.000841282	5208.5	9.905669E-01
Scube1	30544	0.109811643	12703	1.216411E-01
Scube2	30545	0.200978512	18286	4.322347E-03
Scube3	30546	0.026411408	6898	7.104608E-01
Scx	30547	0.098632569	11964	1.646739E-01
Scyl1	30548	-0.002256025	5063	9.747073E-01
Scyl2	30549	0.029444338	7041	6.789579E-01
Scyl3	30550	0.171821518	17007	1.498204E-02
Sdad1	30551	-0.026284397	4055	7.117913E-01
Sdc1	30552	0.053997375	8866	4.476098E-01
Sdc2	30553	-0.082884048	607	2.432834E-01
Sdc3	30554	0.145284952	15369	4.010511E-02
Sdc4	30555	0.293327632	19876	2.493610E-05
Sdcbp	30556	0.228787692	19108	1.119650E-03
Sdcbp2	30557	0.022213206	6644	7.548797E-01
Sdccag3	30558	0.140441039	14983	4.730841E-02
Sdccag8	30559	-0.084823508	575	2.323919E-01
Sde2	30560	0.222553935	18971	1.537661E-03
Sdf2	30561	0.012652126	5911	8.588693E-01
Sdf2l1	30562	0.254566804	19560	2.751197E-04
Sdf4	30563	0.250859581	19518	3.397786E-04
Sdha	30564	0.182209471	17499	9.813475E-03
Sdhaf1	30565	0.127363858	14143	7.229859E-02
Sdhaf2	30566	0.13881009	14871	4.996461E-02
Sdhb	30567	0.020278208	6342	7.756373E-01
Sdhc	30568	0.11623931	13411	1.011863E-01
Sdhd	30569	0.093049738	11590	1.900204E-01
Sdk1	30570	0.137978507	14820	5.136601E-02
Sdk2	30571	0.095464977	11736	1.787255E-01
Sdpr	30572	-0.054451513	1703	4.437940E-01

Spearman Rank correlation analysis performed between Prdm1 and all-expressed genes within the Meredith RNA-seq dataset. Robust Prdm1–associated genes were identified using a cut-off of $p < 0.0005$.

Table S1, Related to Supplemental Figure 3C. Prdm1 associated genes

Sdr16c5	30573		#N/A	1.000000E+00
Sdr16c6	30574	0.069926195	9840	3.251587E-01
Sdr39u1	30575	0.139464323	14920	4.888457E-02
Sdr42e1	30576	0.150308195	15667	3.363343E-02
Sdr9c7	30577	0.12010172	13669	9.026800E-02
Sds	30578	0.00030705	5316.5	9.965570E-01
Sdsl	30579	-0.113070261	143	1.109028E-01
Sebox	30580		#N/A	1.000000E+00
Sec1	30581	0.125775899	14021	7.595657E-02
Sec11a	30582	0.120425286	13688	8.939779E-02
Sec11c	30583	0.136016182	14703	5.480276E-02
Sec13	30584	0.208183805	18529	3.094510E-03
Sec14l1	30585	0.08734131	11153	2.187713E-01
Sec14l2	30586	0.125288721	13990	7.710838E-02
Sec14l3	30587	0.098949964	11991	1.633128E-01
Sec14l4	30588	0.038758411	7558.5	5.858252E-01
Sec14l5	30589		#N/A	1.000000E+00
Sec16a	30590	0.175719132	17203	1.281521E-02
Sec16b	30591	0.011837391	5869	8.678717E-01
Sec22a	30592	0.154142324	15901	2.931094E-02
Sec22b	30593	0.122745021	13829	8.335374E-02
Sec22c	30594	0.218175292	18847	1.911817E-03
Sec23a	30595	0.224653875	19036	1.383116E-03
Sec23b	30596	0.184663176	17623	8.852086E-03
Sec23ip	30597	0.264562912	19672	1.532700E-04
Sec24a	30598	0.151323667	15735	3.243900E-02
Sec24b	30599	0.001183439	5374	9.867306E-01
Sec24c	30600	0.209463099	18563	2.912918E-03
Sec24d	30601	0.198831922	18190	4.764788E-03
Sec31a	30602	0.111224017	12804	1.168917E-01
Sec31b	30603		#N/A	1.000000E+00
Sec61a1	30604	0.14770406	15517	3.686826E-02
Sec61a2	30605	0.007837715	5673	9.122912E-01
Sec61b	30606	0.124221454	13921	7.968107E-02
Sec61g	30607	0.181525086	17465	1.009759E-02
Sec62	30608	0.142318006	15100	4.439845E-02
Sec63	30609	0.180104839	17409	1.071049E-02
Secisbp2	30610	0.155069521	15984	2.833994E-02
Secisbp2l	30611	0.010534134	5792	8.823069E-01
Sectm1a	30612	0.103366448	12262	1.452367E-01
Sectm1b	30613	-0.038406171	3065	5.892384E-01
Seh1l	30614	0.086962061	11124	2.207857E-01
Sel1l	30615	0.112918855	12917	1.113847E-01

Spearman Rank correlation analysis performed between Prdm1 and all-expressed genes within the Meredith RNA-seq dataset. Robust Prdm1–associated genes were identified using a cut-off of $p < 0.0005$.

Table S1, Related to Supplemental Figure 3C. Prdm1 associated genes

Sel1l2	30616	0.114668812	13303	1.059139E-01
Sel1l3	30617	0.114646668	13301	1.059818E-01
Sele	30618		#N/A	1.000000E+00
Selenbp1	30619	0.012280314	5891	8.629754E-01
Selenbp2	30620	0.053267784	8685	4.537786E-01
Selk	30621	0.210553075	18596	2.765874E-03
Sell	30622	0.024351285	6778	7.321467E-01
Selm	30623	0.078575576	10500	2.687386E-01
Selp	30624	0.051839375	8458	4.659927E-01
Selplg	30625	0.18122962	17456	1.022248E-02
Selrc1	30626	0.176325828	17238	1.250393E-02
Sema3a	30627	-0.093185474	386	1.893721E-01
Sema3b	30628	0.012802463	5918	8.572101E-01
Sema3c	30629	0.075619451	10299	2.872191E-01
Sema3d	30630	0.185882885	17677	8.406018E-03
Sema3e	30631	0.169264287	16892	1.657196E-02
Sema3f	30632	0.087549039	11173	2.176735E-01
Sema3g	30633		#N/A	1.000000E+00
Sema4a	30634	-0.037741655	3639	5.957021E-01
Sema4b	30635	-0.020434916	4223	7.739501E-01
Sema4c	30636	0.063534419	9437	3.714375E-01
Sema4d	30637	0.082698432	10806	2.443442E-01
Sema4f	30638	-0.081420946	626	2.517320E-01
Sema4g	30639	0.084863425	10979	2.321714E-01
Sema5a	30640	0.072012681	10000	3.108950E-01
Sema5b	30641	0.124983137	13969	7.783804E-02
Sema6a	30642	0.056557803	9013	4.263392E-01
Sema6b	30643	0.068781858	9760	3.331583E-01
Sema6c	30644		#N/A	1.000000E+00
Sema6d	30645	0.214015542	18714	2.342306E-03
Sema7a	30646	0.106287268	12444	1.341453E-01
Senp1	30647	0.129410817	14265	6.779584E-02
Senp2	30648	0.200959204	18284	4.326156E-03
Senp3	30649	0.100005737	12067	1.588457E-01
Senp5	30650	-0.027287457	4024	7.013079E-01
Senp6	30651	0.21842834	18853	1.888117E-03
Senp7	30652	0.136583478	14733	5.379021E-02
Senp8	30653	0.105513502	12392	1.370182E-01
Sephs1	30654	0.04910684	8223	4.898551E-01
Sephs2	30655	-0.018138548	4336	7.987765E-01
Sepn1	30656	-0.044358462	2355	5.328257E-01
Sepp1	30657	0.10752525	12524	1.296452E-01
Sepsecs	30658	0.090387539	11374	2.030650E-01

Spearman Rank correlation analysis performed between Prdm1 and all-expressed genes within the Meredith RNA-seq dataset. Robust Prdm1–associated genes were identified using a cut-off of $p < 0.0005$.

Table S1, Related to Supplemental Figure 3C. Prdm1 associated genes

Sepw1	30659	0.122679024	13826	8.352104E-02
Serac1	30660	0.138109217	14833	5.114360E-02
Serbp1	30661	0.231893719	19209	9.529141E-04
Serf1	30662	0.117806268	13515	9.663722E-02
Serf2	30663	0.087822183	11188	2.162359E-01
Sergef	30664	0.160935984	16386	2.281175E-02
Serhl	30665	0.095244357	11714	1.797362E-01
Serinc1	30666	0.208593468	18539	3.035275E-03
Serinc2	30667	0.210663803	18597	2.751319E-03
Serinc3	30668	0.210247707	18582	2.806376E-03
Serinc4	30669	0.006263827	5607	9.298532E-01
Serinc5	30670	0.182533977	17532	9.681248E-03
Serp1	30671	0.192107659	17916	6.425982E-03
Serp2	30672	-0.016730711	4448.5	8.140990E-01
Serpina10	30673	0.018928849	6275	7.902082E-01
Serpina11	30674	-0.033388619	3766.5	6.388141E-01
Serpina12	30675	-0.06446002	1333	3.644957E-01
Serpina1a	30676	0.113548679	13094	1.093908E-01
Serpina1b	30677	-0.066858259	1029.5	3.468868E-01
Serpina1c	30678		#N/A	1.000000E+00
Serpina1d	30679		#N/A	1.000000E+00
Serpina1e	30680		#N/A	1.000000E+00
Serpina1f	30681	0.031633724	7168.5	6.565542E-01
Serpina3a	30682	-0.054451513	1703	4.437940E-01
Serpina3b	30683		#N/A	1.000000E+00
Serpina3c	30684	-0.095269893	339	1.796190E-01
Serpina3d-ps	30685		#N/A	1.000000E+00
Serpina3e-ps	30686	-0.054450825	1984	4.437997E-01
Serpina3f	30687	0.004370324	5534	9.510258E-01
Serpina3g	30688		#N/A	1.000000E+00
Serpina3h	30689	0.099884831	12056	1.593525E-01
Serpina3i	30690	0.021262466	6454	7.650578E-01
Serpina3j	30691	-0.089493765	423	2.075863E-01
Serpina3k	30692		#N/A	1.000000E+00
Serpina3l-ps	30693		#N/A	1.000000E+00
Serpina3m	30694	-0.095273984	329	1.796002E-01
Serpina3n	30695	0.001078371	5363	9.879086E-01
Serpina4-ps1	30696		#N/A	1.000000E+00
Serpina5	30697		#N/A	1.000000E+00
Serpina6	30698		#N/A	1.000000E+00
Serpina7	30699	0.051678616	8446	4.673786E-01
Serpina9	30700	0.030819098	7119	6.648558E-01
Serpinb1-ps1	30701		#N/A	1.000000E+00

Spearman Rank correlation analysis performed between Prdm1 and all-expressed genes within the Meredith RNA-seq dataset. Robust Prdm1–associated genes were identified using a cut-off of $p < 0.0005$.

Table S1, Related to Supplemental Figure 3C. Prdm1 associated genes

Serpinb10	30702	0.034698681	7322	6.257024E-01
SERPINB10	30703		#N/A	1.000000E+00
Serpinb11	30704	0.0552275	8938	4.373167E-01
Serpinb12	30705	-0.153792059	19	2.968508E-02
Serpinb13	30706	0.000252701	5302	9.971665E-01
Serpinb1a	30707	0.107633043	12531	1.292590E-01
Serpinb1b	30708	0.086045722	11056	2.257072E-01
Serpinb1c	30709	-0.086748028	498.5	2.219283E-01
Serpinb2	30710	-0.028032305	3996	6.935595E-01
Serpinb3a	30711	0.113548679	13094	1.093908E-01
Serpinb3b	30712	0.118173678	13539	9.559443E-02
Serpinb3c	30713		#N/A	1.000000E+00
Serpinb3d	30714		#N/A	1.000000E+00
Serpinb5	30715	0.099739099	12044	1.599651E-01
Serpinb6a	30716	0.038441853	7522	5.888922E-01
Serpinb6b	30717	0.046158174	8023	5.163193E-01
Serpinb6c	30718	-0.055896814	1492	4.317736E-01
Serpinb6d	30719	0.020875719	6412.5	7.692098E-01
Serpinb6e	30720	-0.054514896	1537.5	4.432629E-01
Serpinb7	30721	0.054051416	8869	4.471547E-01
Serpinb8	30722	0.059634089	9194	4.015737E-01
Serpinb9	30723	0.05332693	8764	4.532767E-01
Serpinb9b	30724	0.181110924	17451	1.027304E-02
Serpinb9c	30725	0.07521908	10268	2.897860E-01
Serpinb9d	30726	0.052728522	8550.5	4.583686E-01
Serpinb9e	30727		#N/A	1.000000E+00
Serpinb9f	30728	-0.066856562	1139	3.468991E-01
Serpinb9g	30729	-0.000494087	5244	9.944598E-01
Serpinc1	30730	0.079612182	10564	2.624544E-01
Serpind1	30731	0.166369694	16740.5	1.854724E-02
Serpine1	30732	0.014376261	6034	8.398799E-01
Serpine2	30733	0.06961278	9821	3.273372E-01
Serpine3	30734	0.225027364	19041	1.357167E-03
Serpinf1	30735	0.054948097	8922	4.396427E-01
Serpinf2	30736	-0.066856562	1139	3.468991E-01
Serping1	30737	0.04374018	7846	5.385569E-01
Serpinh1	30738	0.034384983	7307	6.288316E-01
Serpini1	30739	0.090660573	11398	2.016981E-01
Serpini2	30740		#N/A	1.000000E+00
Sertad1	30741	0.102655893	12222	1.480376E-01
Sertad2	30742	0.100566637	12095	1.565101E-01
Sertad3	30743	0.10194974	12181	1.508614E-01
Sertad4	30744	0.044007627	7868	5.360740E-01

Spearman Rank correlation analysis performed between Prdm1 and all-expressed genes within the Meredith RNA-seq dataset. Robust Prdm1–associated genes were identified using a cut-off of $p < 0.0005$.

Table S1, Related to Supplemental Figure 3C. Prdm1 associated genes

Sertm1	30745	0.189988431	17842.5	7.047572E-03
Sesn1	30746	0.078537393	10497	2.689720E-01
Sesn2	30747	0.016665435	6156	8.148112E-01
Sesn3	30748	0.089522525	11311	2.074397E-01
Sestd1	30749	0.090180904	11363	2.041039E-01
Set	30750	0.206274546	18468	3.384616E-03
Setbp1	30751	0.151315143	15734	3.244888E-02
Setd1a	30752	-0.050732648	2188	4.755794E-01
Setd1b	30753	0.162344715	16487	2.163202E-02
Setd2	30754	0.064998861	9534	3.604918E-01
Setd3	30755	0.0900441	11348	2.047939E-01
Setd4	30756		#N/A	1.000000E+00
Setd5	30757	0.022914499	6687	7.473989E-01
Setd6	30758	0.262756128	19654	1.706583E-04
Setd7	30759	-0.026845133	4036	7.059240E-01
Setd8	30760	0.099047581	12001	1.628958E-01
Setdb1	30761	0.201128767	18295	4.292808E-03
Setdb2	30762	0.102742752	12228	1.476930E-01
Setmar	30763	0.081437712	10695.5	2.516340E-01
Setx	30764	0.087528441	11170	2.177821E-01
Sez6	30765	0.083439169	10861	2.401303E-01
Sez6l	30766	-0.049424926	2209	4.870442E-01
Sez6l2	30767	-0.036637488	3668	6.065128E-01
Sf1	30768	0.197169527	18129	5.134933E-03
Sf3a1	30769	0.006208688	5606	9.304692E-01
Sf3a2	30770	0.071048989	9916	3.174313E-01
Sf3a3	30771	0.151966795	15765	3.170134E-02
Sf3b1	30772	0.267529693	19704	1.282630E-04
Sf3b2	30773	0.233186798	19230	8.904874E-04
Sf3b3	30774	0.147283933	15492	3.741402E-02
Sf3b4	30775	0.091159572	11452	1.992172E-01
Sf3b5	30776	0.136559654	14732	5.383241E-02
Sfi1	30777	0.12155134	13757	8.642151E-02
Sfmbt1	30778	0.099106487	12005	1.626446E-01
Sfmbt2	30779	0.081407349	10692	2.518114E-01
Sfn	30780	0.040656891	7675	5.675878E-01
Sfpi1	30781	0.046972777	8072	5.089354E-01
Sfpq	30782	0.212307928	18665	2.543221E-03
Sfr1	30783	0.238278002	19335	6.795243E-04
Sfrp1	30784	0.010883286	5821	8.784356E-01
Sfrp2	30785	0.145438713	15377	3.989248E-02
Sfrp4	30786	-0.001223987	5172.5	9.862760E-01
Sfrp5	30787	0.262693624	19652	1.712915E-04

Spearman Rank correlation analysis performed between Prdm1 and all-expressed genes within the Meredith RNA-seq dataset. Robust Prdm1–associated genes were identified using a cut-off of $p < 0.0005$.

Sfrs18	30788	0.188803476	17789	7.418006E-03
Sfswap	30789	0.196319086	18096	5.334090E-03
Sft2d1	30790	0.091630124	11497	1.968980E-01
Sft2d2	30791	0.155951833	16034	2.744161E-02
Sft2d3	30792	0.018521986	6249	7.946162E-01
Sfta2	30793	-0.077394997	736.5	2.760195E-01
Sftpa1	30794	-0.054451513	1703	4.437940E-01
Sftpb	30795	0.179553377	17384	1.095718E-02
Sftpc	30796		#N/A	1.000000E+00
Sftpd	30797	0.094768377	11689	1.819311E-01
Sfxn1	30798	0.162383488	16492	2.160031E-02
Sfxn2	30799	0.068264485	9721	3.368160E-01
Sfxn3	30800	0.110102414	12725	1.206513E-01
Sfxn4	30801	-0.011633857	4681	8.701233E-01
Sfxn5	30802	0.016583993	6150	8.157000E-01
Sgca	30803	0.020875719	6412.5	7.692098E-01
Sgcb	30804	0.115107301	13330	1.045768E-01
Sgcd	30805	0.08648518	11105	2.233373E-01
Sgce	30806	0.102661913	12224.5	1.480137E-01
Sgcg	30807	-0.064342191	1336	3.653749E-01
Sgcz	30808	0.087692812	11178	2.169160E-01
Sgip1	30809	0.014346114	6033	8.402112E-01
Sgk1	30810	0.040615101	7670	5.679863E-01
Sgk2	30811	-0.038406171	3065	5.892384E-01
Sgk3	30812	0.104807816	12347	1.396792E-01
Sgms1	30813	0.149533671	15619	3.456936E-02
Sgms2	30814	0.077885333	10462	2.729794E-01
Sgol1	30815	0.359845307	20012	1.663665E-07
Sgol2	30816	0.071364477	9941	3.152817E-01
Sgpl1	30817	0.053089619	8600	4.552922E-01
Sgpp1	30818	0.184359027	17612	8.966537E-03
Sgpp2	30819	0.043181579	7815	5.437612E-01
Sgsh	30820	0.105418454	12385	1.373743E-01
Sgsm1	30821	0.044812634	7921	5.286353E-01
Sgsm2	30822	0.096227821	11789	1.752632E-01
Sgsm3	30823	0.139533588	14923	4.877138E-02
Sgta	30824	0.024311694	6775	7.325656E-01
Sgtb	30825	0.221516049	18947	1.619733E-03
Sh2b1	30826	0.015780809	6111	8.244779E-01
Sh2b2	30827	-0.054450825	1984	4.437997E-01
Sh2b3	30828	0.064500157	9505	3.641965E-01
Sh2d1a	30829	-0.000841282	5208.5	9.905669E-01
Sh2d1b1	30830	0.074574898	10201.5	2.939481E-01

Spearman Rank correlation analysis performed between Prdm1 and all-expressed genes within the Meredith RNA-seq dataset. Robust Prdm1–associated genes were identified using a cut-off of $p < 0.0005$.

Table S1, Related to Supplemental Figure 3C. Prdm1 associated genes

Sh2d1b2	30831		#N/A	1.000000E+00
Sh2d2a	30832	0.008194312	5685	9.083178E-01
Sh2d3c	30833	0.137726287	14803	5.179746E-02
Sh2d4a	30834	0.012963934	5936	8.554288E-01
Sh2d4b	30835	0.175624288	17196	1.286448E-02
Sh2d5	30836	0.050628615	8353	4.764861E-01
Sh2d6	30837	0.00140357	5385	9.842627E-01
Sh2d7	30838	0.113548679	13094	1.093908E-01
Sh3bgr	30839	-0.038406171	3065	5.892384E-01
Sh3bgrl	30840	0.11420934	13265	1.073294E-01
Sh3bgrl2	30841	0.065824453	9578	3.544105E-01
Sh3bgrl3	30842	0.172751302	17067	1.443795E-02
Sh3bp1	30843	0.287281341	19850	3.713988E-05
Sh3bp2	30844	0.05055379	8348	4.771388E-01
Sh3bp4	30845	-0.023143106	4145	7.449654E-01
Sh3bp5	30846	0.146757082	15464	3.810804E-02
Sh3bp5l	30847	-0.003760439	4993	9.578534E-01
Sh3d19	30848	0.258104298	19601	2.242661E-04
Sh3d21	30849	-0.002533205	5048	9.716010E-01
Sh3d2c-ps1	30850		#N/A	1.000000E+00
Sh3gl1	30851	0.005618989	5582	9.370588E-01
Sh3gl2	30852	-0.09428313	380	1.841889E-01
Sh3gl3	30853	0.10474908	12339	1.399025E-01
Sh3glb1	30854	0.035290491	7341	6.198174E-01
Sh3glb2	30855	0.134269522	14579	5.801984E-02
Sh3kbp1	30856	-0.041911105	2435	5.556893E-01
Sh3pxd2a	30857	0.219382492	18884	1.801143E-03
Sh3pxd2b	30858	-0.044253983	2366	5.337920E-01
Sh3rf1	30859	0.180375411	17424	1.059126E-02
Sh3rf2	30860	0.210201585	18580	2.812540E-03
Sh3rf3	30861	0.058890183	9150	4.074826E-01
Sh3tc1	30862	-0.086748473	486.5	2.219259E-01
Sh3tc2	30863	0.088857483	11259	2.108489E-01
Sh3yl1	30864	0.029002675	7015	6.835126E-01
Shank1	30865	0.232484186	19218	9.239249E-04
Shank2	30866	0.156663612	16089	2.673479E-02
Shank3	30867	0.085435582	11017	2.290271E-01
Sharpin	30868	0.221382364	18942	1.630590E-03
Shb	30869	0.039704396	7613	5.767040E-01
Shbg	30870	-0.038406171	3065	5.892384E-01
Shc1	30871	0.135399268	14656	5.592174E-02
Shc2	30872	0.095649375	11745	1.778840E-01
Shc3	30873	0.039972955	7639	5.741267E-01

Spearman Rank correlation analysis performed between Prdm1 and all-expressed genes within the Meredith RNA-seq dataset. Robust Prdm1–associated genes were identified using a cut-off of $p < 0.0005$.

Shc4	30874	-0.003795613	4991	9.574596E-01
Shcbp1	30875	0.067958622	9700	3.389905E-01
Shcbp1l	30876	-0.001224019	5151	9.862757E-01
Shd	30877	-0.077393517	797	2.760287E-01
She	30878	0.24598422	19455	4.463561E-04
Shf	30879	0.018592105	6257	7.938561E-01
Shfm1	30880	0.100139102	12072	1.582880E-01
Shh	30881		#N/A	1.000000E+00
Shisa2	30882	-0.113291927	142	1.102002E-01
Shisa3	30883	0.044096573	7876	5.352495E-01
Shisa4	30884	0.01726268	6189	8.083006E-01
Shisa5	30885	0.204387923	18404	3.695200E-03
Shisa6	30886	-0.015862797	4475	8.235809E-01
Shisa7	30887	0.014124464	5995	8.426477E-01
Shisa9	30888	0.211238292	18619	2.676913E-03
Shkbp1	30889	0.165884123	16696	1.889788E-02
Shmt1	30890	0.020328569	6343	7.750950E-01
Shmt2	30891	0.110992311	12788	1.176608E-01
Shoc2	30892	-0.019461327	4258	7.844493E-01
Shox2	30893	-0.000841234	5228	9.905674E-01
Shpk	30894	0.030661712	7106	6.664644E-01
Shprh	30895	0.14862826	15569	3.569135E-02
Shq1	30896	0.031363896	7149	6.592993E-01
Shroom1	30897	-0.124248073	82	7.961607E-02
Shroom2	30898	0.101868271	12172	1.511898E-01
Shroom3	30899	0.082671871	10801	2.444962E-01
Shroom4	30900	-0.026129541	4063	7.134146E-01
Siae	30901	0.101929616	12178	1.509425E-01
Siah1a	30902	0.088212731	11224	2.141924E-01
Siah1b	30903	0.258725175	19610	2.162995E-04
Siah2	30904	0.182487299	17529	9.700170E-03
Siah3	30905	0.154064684	15894.5	2.939352E-02
Sidt1	30906	-0.09283028	387	1.910720E-01
Sidt2	30907	0.274238924	19750	8.508044E-05
Sigirr	30908	0.066040431	9591	3.528303E-01
Siglec1	30909	-0.054450825	1984	4.437997E-01
Siglec15	30910	0.038229357	7499.5	5.909551E-01
Siglec5	30911	0.086813913	11118	2.215761E-01
Siglece	30912	0.13362092	14519	5.925344E-02
Siglecg	30913	0.042288114	7758	5.521365E-01
Siglech	30914	0.16986028	16922	1.618882E-02
Sigmar1	30915	0.126325741	14069	7.467337E-02
Sik1	30916	0.207011574	18494	3.269834E-03

Table S1, Related to Supplemental Figure 3C. Prdm1 associated genes

Sik2	30917	0.265427375	19680	1.455493E-04
Sik3	30918	0.072509149	10032	3.075623E-01
Sike1	30919	0.051041956	8389	4.728894E-01
Sil1	30920	-0.030286274	3920	6.703078E-01
Sim1	30921	0.092095859	11524	1.946220E-01
Sim2	30922	-0.005348205	4909.5	9.400862E-01
Simc1	30923	0.089257687	11289	2.087926E-01
Sin3a	30924	0.314090977	19950	5.920048E-06
Sin3b	30925	0.069078034	9786	3.310758E-01
Sipa1	30926	0.105718308	12401	1.362532E-01
Sipa1l1	30927	0.112822773	12909	1.116913E-01
Sipa1l2	30928	0.022420488	6657	7.526662E-01
Sipa1l3	30929	0.037604222	7465	5.970429E-01
Sirpa	30930	0.002409528	5439	9.729869E-01
Sirpb1a	30931	0.090089482	11353.5	2.045648E-01
Sirpb1b	30932	0.088625848	11245	2.120458E-01
Sirpb1c	30933	0.182294195	17509.5	9.778799E-03
Sirt1	30934	0.123163401	13856	8.229946E-02
Sirt2	30935	0.068040032	9706	3.384108E-01
Sirt3	30936	0.021741135	6569	7.599283E-01
Sirt4	30937	0.143743528	15291	4.228987E-02
Sirt5	30938	0.052798006	8582	4.577757E-01
Sirt6	30939	0.033949623	7292	6.331853E-01
Sirt7	30940	0.18741458	17739	7.874179E-03
Sis	30941	0.032895251	7232	6.437814E-01
Sit1	30942	-0.032450477	3820	6.482730E-01
Siva1	30943	0.112168107	12865	1.137985E-01
Six1	30944	0.203733753	18378	3.808754E-03
Six2	30945	0.199412882	18222	4.641197E-03
Six3	30946	0.055991787	8985	4.309903E-01
Six3os1	30947	-0.054450825	1984	4.437997E-01
Six4	30948	0.080098991	10600	2.595383E-01
Six5	30949	-0.025820186	4072	7.166614E-01
Six6	30950		#N/A	1.000000E+00
Ska1	30951	0.216252905	18793	2.100903E-03
Ska2	30952	0.129236748	14257	6.816962E-02
Ska3	30953	0.223230855	18992	1.486200E-03
Skap1	30954	0.230560783	19179	1.021455E-03
Skap2	30955	0.01265347	5912	8.588545E-01
Ski	30956	0.155026945	15982	2.838392E-02
Skida1	30957	0.123131705	13853	8.237895E-02
Skil	30958	0.283184734	19819	4.839989E-05
Skint1	30959	-0.017974668	4370	8.005563E-01

Spearman Rank correlation analysis performed between Prdm1 and all-expressed genes within the Meredith RNA-seq dataset. Robust Prdm1–associated genes were identified using a cut-off of $p < 0.0005$.

Table S1, Related to Supplemental Figure 3C. Prdm1 associated genes

Skint10	30960	0.090130942	11360	2.043557E-01
Skint11	30961	-0.038406171	3065	5.892384E-01
Skint2	30962	0.072188704	10009	3.097107E-01
Skint3	30963	0.036614441	7408	6.067393E-01
Skint4	30964	0.106896079	12490	1.319175E-01
Skint5	30965	0.136712371	14742	5.356232E-02
Skint6	30966	0.07196929	9994	3.111874E-01
Skint7	30967	0.020336297	6344	7.750117E-01
Skint8	30968	0.00576685	5585	9.354061E-01
Skint9	30969	0.04834322	8165	4.966383E-01
Skiv2l	30970	0.208313245	18532	3.075682E-03
Skiv2l-ps1	30971		#N/A	1.000000E+00
Skiv2l2	30972	0.201293134	18302	4.260703E-03
Skor1	30973		#N/A	1.000000E+00
Skor2	30974	-0.018447724	4312.5	7.954215E-01
Skp1a	30975	0.20059843	18267	4.397886E-03
Skp2	30976	0.194073539	17994	5.893632E-03
Sla	30977	0.051460734	8424	4.692606E-01
Sla2	30978	0.084636591	10962	2.334264E-01
Slain1	30979	0.010677255	5802	8.807197E-01
Slain2	30980	0.054570887	8898	4.427940E-01
Slamf1	30981	0.089653943	11317	2.067707E-01
Slamf6	30982	0.248905018	19496	3.792973E-04
Slamf7	30983	0.208976926	18549	2.980760E-03
Slamf8	30984	0.083271848	10853	2.410777E-01
Slamf9	30985	0.180873586	17442	1.037479E-02
Slbp	30986	0.205758044	18448	3.467211E-03
Slc10a1	30987	-0.056323315	1475	4.282625E-01
Slc10a2	30988		#N/A	1.000000E+00
Slc10a3	30989	0.133162181	14487	6.013891E-02
Slc10a4	30990	-0.026175139	4057	7.129365E-01
Slc10a5	30991	0.051020925	8387	4.730720E-01
Slc10a6	30992		#N/A	1.000000E+00
Slc10a7	30993	0.115783678	13379	1.025404E-01
Slc11a1	30994	0.068633323	9749	3.342058E-01
Slc11a2	30995	0.000296745	5306	9.966726E-01
Slc12a1	30996	0.18173028	17471	1.001165E-02
Slc12a2	30997	0.25358289	19547	2.910623E-04
Slc12a3	30998	0.105461443	12388	1.372132E-01
Slc12a4	30999	0.085913224	11050	2.264253E-01
Slc12a5	31000	0.001683992	5405	9.811190E-01
Slc12a6	31001	0.305195459	19921	1.110989E-05
Slc12a7	31002	-0.040999165	2453	5.643287E-01

Spearman Rank correlation analysis performed between Prdm1 and all-expressed genes within the Meredith RNA-seq dataset. Robust Prdm1–associated genes were identified using a cut-off of $p < 0.0005$.

Table S1, Related to Supplemental Figure 3C. Prdm1 associated genes

Slc12a8	31003	-0.043995046	2371	5.361907E-01
Slc12a9	31004	0.172664445	17060	1.448803E-02
Slc13a1	31005	0.179449166	17381	1.100435E-02
Slc13a2	31006	0.079997682	10590	2.601433E-01
Slc13a3	31007	0.065828721	9579	3.543792E-01
Slc13a4	31008	0.108742298	12604.5	1.253355E-01
Slc13a5	31009		#N/A	1.000000E+00
Slc14a1	31010	0.105889771	12414	1.356153E-01
Slc14a2	31011	-0.089678098	420	2.066480E-01
Slc15a1	31012	-0.011691366	4678	8.694870E-01
Slc15a2	31013	0.067150385	9654	3.447792E-01
Slc15a3	31014	0.095913484	11764	1.766838E-01
Slc15a4	31015	0.218080879	18846	1.920728E-03
Slc15a5	31016	0.004038581	5519	9.547392E-01
Slc16a1	31017	0.073798704	10114	2.990158E-01
Slc16a10	31018	0.038169327	7490	5.915385E-01
Slc16a11	31019	-0.018103658	4360	7.991553E-01
Slc16a12	31020	0.130393582	14317	6.571683E-02
Slc16a13	31021	-0.029261571	3959.5	6.808413E-01
Slc16a14	31022	0.145640339	15387	3.961510E-02
Slc16a2	31023	0.066947107	9641	3.462450E-01
Slc16a3	31024	-0.09253448	393	1.924961E-01
Slc16a4	31025	0.179556139	17385	1.095593E-02
Slc16a5	31026	-0.054451513	1703	4.437940E-01
Slc16a6	31027	0.309396147	19938	8.273948E-06
Slc16a7	31028	0.017564876	6206	8.050112E-01
Slc16a8	31029		#N/A	1.000000E+00
Slc16a9	31030	-0.002318126	5055	9.740113E-01
Slc17a1	31031	-0.055064922	1516	4.386693E-01
Slc17a2	31032		#N/A	1.000000E+00
Slc17a3	31033	0.156656665	16087	2.674161E-02
Slc17a4	31034	0.212144329	18657	2.563268E-03
Slc17a5	31035	0.011648214	5860	8.699645E-01
Slc17a6	31036	-0.101853834	262	1.512481E-01
Slc17a7	31037		#N/A	1.000000E+00
Slc17a8	31038	0.105163132	12368	1.383345E-01
Slc17a9	31039	0.29344364	19877	2.474404E-05
Slc18a1	31040	0.145660505	15389	3.958745E-02
Slc18a2	31041	-0.022022927	4172	7.569134E-01
Slc18b1	31042	0.110051044	12718	1.208257E-01
Slc19a1	31043	0.115323159	13348	1.039235E-01
Slc19a2	31044	0.138620195	14860	5.028179E-02
Slc19a3	31045	0.061556544	9304	3.865418E-01

Spearman Rank correlation analysis performed between Prdm1 and all-expressed genes within the Meredith RNA-seq dataset. Robust Prdm1–associated genes were identified using a cut-off of $p < 0.0005$.

Table S1, Related to Supplemental Figure 3C. Prdm1 associated genes

Slc1a1	31046	0.033001594	7239	6.427093E-01
Slc1a2	31047	0.029088053	7018	6.826312E-01
Slc1a3	31048	0.193145158	17953	6.139910E-03
Slc1a4	31049	0.145905618	15406	3.925263E-02
Slc1a5	31050	0.266162009	19686	1.392753E-04
Slc1a6	31051	-0.032450477	3820	6.482730E-01
Slc1a7	31052	-0.086746694	532	2.219355E-01
Slc20a1	31053	-0.06055476	1390	3.943319E-01
Slc20a2	31054	-0.00585894	4885	9.343769E-01
Slc22a1	31055	-0.054450825	1984	4.437997E-01
Slc22a12	31056	-0.066254282	1296	3.512700E-01
Slc22a13	31057		#N/A	1.000000E+00
Slc22a13b-ps	31058		#N/A	1.000000E+00
Slc22a14	31059	0.123683759	13885	8.100319E-02
Slc22a15	31060	-0.024394898	4122	7.316853E-01
Slc22a16	31061	0.152789767	15822	3.077826E-02
Slc22a17	31062	0.086233722	11066	2.246912E-01
Slc22a18	31063	0.027801149	6964	6.959608E-01
Slc22a19	31064	-0.054451513	1703	4.437940E-01
Slc22a2	31065	0.028302023	6984	6.907614E-01
Slc22a20	31066	-0.070670908	941	3.200199E-01
Slc22a21	31067	0.022198129	6607.5	7.550408E-01
Slc22a22	31068	-0.045267039	2318	5.244595E-01
Slc22a23	31069	0.066280128	9606	3.510817E-01
Slc22a26	31070	-0.038406171	3065	5.892384E-01
Slc22a27	31071	0.156820091	16099	2.658151E-02
Slc22a28	31072		#N/A	1.000000E+00
Slc22a29	31073	0.160987082	16412.5	2.276801E-02
Slc22a3	31074	0.070103468	9846	3.239306E-01
Slc22a30	31075	0.014300976	6004	8.407072E-01
Slc22a4	31076	-0.080036461	641	2.599116E-01
Slc22a5	31077	-0.045144853	2327	5.255807E-01
Slc22a6	31078	-0.038406171	3065	5.892384E-01
Slc22a7	31079	-0.096510233	304	1.739941E-01
Slc22a8	31080	0.014913507	6064.5	8.339810E-01
Slc23a1	31081	-0.013385467	4601	8.507818E-01
Slc23a2	31082	0.088876388	11261	2.107515E-01
Slc23a3	31083	-0.077393517	797	2.760287E-01
Slc24a1	31084	-0.035143135	3707	6.212805E-01
Slc24a2	31085	0.007388843	5652	9.172960E-01
Slc24a3	31086	0.115034241	13322	1.047987E-01
Slc24a4	31087	0.067675468	9684	3.410114E-01
Slc24a5	31088	0.215187417	18750	2.212890E-03

Spearman Rank correlation analysis performed between Prdm1 and all-expressed genes within the Meredith RNA-seq dataset. Robust Prdm1–associated genes were identified using a cut-off of $p < 0.0005$.

Slc24a6	31089	0.114488168	13290	1.064687E-01
Slc25a1	31090	-0.028895983	3967	6.846145E-01
Slc25a10	31091	0.121008625	13726	8.784589E-02
Slc25a11	31092	0.076669209	10390	2.805613E-01
Slc25a12	31093	0.115251247	13339	1.041408E-01
Slc25a13	31094	0.015347886	6090	8.292184E-01
Slc25a14	31095	0.041826455	7732	5.564885E-01
Slc25a15	31096	0.030851587	7120	6.645239E-01
Slc25a16	31097	0.133725208	14530	5.905365E-02
Slc25a17	31098	0.188323844	17774	7.572806E-03
Slc25a18	31099	-0.016731569	4428.5	8.140896E-01
Slc25a19	31100	0.104653615	12332	1.402659E-01
Slc25a2	31101		#N/A	1.000000E+00
Slc25a20	31102	0.012450029	5901	8.611007E-01
Slc25a21	31103	0.144784307	15342	4.080404E-02
Slc25a22	31104	0.05487988	8913	4.402117E-01
Slc25a23	31105	0.064175701	9483	3.666194E-01
Slc25a24	31106	0.042599069	7782	5.492145E-01
Slc25a25	31107	0.036378852	7400	6.090575E-01
Slc25a26	31108	0.091986292	11515	1.951557E-01
Slc25a27	31109	0.269789802	19716	1.118321E-04
Slc25a28	31110	-0.086294655	554	2.243626E-01
Slc25a29	31111	-0.044084038	2369.5	5.353657E-01
Slc25a3	31112	0.137345228	14781	5.245500E-02
Slc25a30	31113	-0.001224019	5151	9.862757E-01
Slc25a31	31114	-0.045149205	2323	5.255407E-01
Slc25a32	31115	0.067763429	9686	3.403828E-01
Slc25a33	31116	0.038605577	7535	5.873050E-01
Slc25a34	31117	0.14090065	15015	4.658153E-02
Slc25a35	31118	-0.01592114	4472	8.229427E-01
Slc25a36	31119	0.127483965	14149	7.202784E-02
Slc25a37	31120	0.096903235	11828	1.722394E-01
Slc25a38	31121	0.078270734	10483	2.706060E-01
Slc25a39	31122	0.15315268	15848	3.037854E-02
Slc25a4	31123	0.059920358	9206	3.993135E-01
Slc25a40	31124	0.108599215	12589	1.258364E-01
Slc25a41	31125	0.113192287	12936	1.105156E-01
Slc25a42	31126	-0.074632632	892	2.935735E-01
Slc25a43	31127	-0.07739401	763.5	2.760257E-01
Slc25a44	31128	0.123189763	13858	8.223339E-02
Slc25a45	31129	0.065553238	9562	3.564011E-01
Slc25a46	31130	0.13921043	14904	4.930138E-02
Slc25a47	31131	0.242753195	19409	5.332309E-04

Spearman Rank correlation analysis performed between Prdm1 and all-expressed genes within the Meredith RNA-seq dataset. Robust Prdm1–associated genes were identified using a cut-off of $p < 0.0005$.

Table S1, Related to Supplemental Figure 3C. Prdm1 associated genes

Slc25a48	31132	0.060109662	9219	3.978231E-01
Slc25a5	31133	0.222668904	18974	1.528808E-03
Slc25a51	31134	0.10677144	12480	1.323713E-01
Slc25a53	31135	0.091059892	11441	1.997110E-01
Slc25a54	31136	-0.066855996	1239.5	3.469032E-01
Slc26a1	31137		#N/A	1.000000E+00
Slc26a10	31138	-0.035722696	3692.5	6.155346E-01
Slc26a11	31139	-0.095273984	329	1.796002E-01
Slc26a2	31140	0.014242217	6002	8.413531E-01
Slc26a3	31141	0.082605987	10794.5	2.448737E-01
Slc26a4	31142	-0.095267438	357.5	1.796303E-01
Slc26a5	31143	0.071667294	9964.5	3.132274E-01
Slc26a6	31144		#N/A	1.000000E+00
Slc26a7	31145	-0.003517417	5006	9.605749E-01
Slc26a8	31146	0.063632619	9446	3.706972E-01
Slc26a9	31147	-0.043687291	2385	5.390486E-01
Slc27a1	31148	0.04051549	7665	5.689368E-01
Slc27a2	31149	-0.054492041	1541	4.434543E-01
Slc27a3	31150	0.05419691	8876	4.459309E-01
Slc27a4	31151	0.156017688	16040	2.737555E-02
Slc27a5	31152	-0.001224019	5151	9.862757E-01
Slc27a6	31153	-0.043108183	2399.5	5.444468E-01
Slc28a1	31154	0.151341089	15737	3.241883E-02
Slc28a2	31155	0.060736391	9250	3.929125E-01
Slc28a3	31156	0.279670547	19793	6.054567E-05
Slc29a1	31157	0.077967257	10467	2.724737E-01
Slc29a2	31158	-7.57115E-05	5285	9.991510E-01
Slc29a3	31159	0.057661838	9068	4.173512E-01
Slc29a4	31160	-0.066855996	1239.5	3.469032E-01
Slc2a1	31161	0.167850071	16813	1.751286E-02
Slc2a10	31162	0.096894574	11826	1.722779E-01
Slc2a12	31163	0.125115292	13976	7.752181E-02
Slc2a13	31164	0.231576008	19202	9.688560E-04
Slc2a2	31165		#N/A	1.000000E+00
Slc2a3	31166	0.058482235	9125	4.107446E-01
Slc2a4	31167	-0.05462106	1531	4.423741E-01
Slc2a4rg-ps	31168	-0.047538025	2247	5.038443E-01
Slc2a5	31169	0.014330687	6029	8.403807E-01
Slc2a6	31170	0.058676926	9139	4.091859E-01
Slc2a7	31171	-0.038406171	3065	5.892384E-01
Slc2a8	31172	0.159031135	16226	2.449490E-02
Slc2a9	31173	-0.039689666	2474	5.768455E-01
Slc30a1	31174	0.08319796	10848	2.414968E-01

Spearman Rank correlation analysis performed between Prdm1 and all-expressed genes within the Meredith RNA-seq dataset. Robust Prdm1–associated genes were identified using a cut-off of $p < 0.0005$.

Table S1, Related to Supplemental Figure 3C. Prdm1 associated genes

Slc30a10	31175	0.185582422	17662	8.514010E-03
Slc30a2	31176	-0.079272749	650	2.645010E-01
Slc30a3	31177	-0.038406171	3065	5.892384E-01
Slc30a4	31178	-0.000584856	5238	9.934421E-01
Slc30a5	31179	0.204009149	18393	3.760571E-03
Slc30a6	31180	-0.032901401	3789	6.437193E-01
Slc30a7	31181	0.12513631	13977	7.747161E-02
Slc30a8	31182	0.053805701	8820	4.492258E-01
Slc30a9	31183	0.22343837	19001	1.470743E-03
Slc31a1	31184	0.035685223	7360	6.159054E-01
Slc31a2	31185	0.023897859	6742	7.369490E-01
Slc32a1	31186		#N/A	1.000000E+00
Slc33a1	31187	0.002608054	5452	9.707622E-01
Slc34a1	31188	0.143605682	15224	4.249002E-02
Slc34a2	31189	0.102327588	12199	1.493454E-01
Slc34a3	31190		#N/A	1.000000E+00
Slc35a1	31191	0.105729705	12402	1.362108E-01
Slc35a2	31192	0.201875506	18320	4.148682E-03
Slc35a3	31193	0.14783778	15531	3.669598E-02
Slc35a4	31194	0.202119001	18333	4.102637E-03
Slc35a5	31195	0.008739716	5715	9.022450E-01
Slc35b1	31196	0.050097693	8304	4.811276E-01
Slc35b2	31197	0.151620766	15747	3.209644E-02
Slc35b3	31198	0.058778252	9146	4.083761E-01
Slc35b4	31199	0.064622449	9513	3.632859E-01
Slc35c1	31200	0.177365455	17284	1.198597E-02
Slc35c2	31201	0.124982537	13968	7.783948E-02
Slc35d1	31202	0.052182956	8484	4.630384E-01
Slc35d2	31203	0.137816541	14812	5.164272E-02
Slc35d3	31204	-0.015718001	4489	8.251653E-01
Slc35e1	31205	0.087851203	11192	2.160836E-01
Slc35e2	31206	0.079492924	10563	2.631722E-01
Slc35e3	31207	0.144686757	15337	4.094142E-02
Slc35e4	31208		#N/A	1.000000E+00
Slc35f1	31209	0.084891783	10982	2.320149E-01
Slc35f2	31210	0.043401749	7830	5.417070E-01
Slc35f3	31211	0.059049949	9159	4.062092E-01
Slc35f4	31212	0.015465102	6094	8.279343E-01
Slc35f5	31213	0.22469204	19037	1.380444E-03
Slc35f6	31214	0.174815649	17157	1.329136E-02
Slc35g1	31215	0.100606891	12097	1.563435E-01
Slc35g2	31216	-0.038406171	3065	5.892384E-01
Slc35g3	31217		#N/A	1.000000E+00

Spearman Rank correlation analysis performed between Prdm1 and all-expressed genes within the Meredith RNA-seq dataset. Robust Prdm1–associated genes were identified using a cut-off of $p < 0.0005$.

Table S1, Related to Supplemental Figure 3C. Prdm1 associated genes

Slc36a1	31218	0.068422381	9735	3.356970E-01
Slc36a2	31219	0.051565914	8432	4.683516E-01
Slc36a3	31220	0.080173596	10608	2.590933E-01
Slc36a4	31221	0.118662275	13565	9.422156E-02
Slc37a1	31222	-0.005144698	4924	9.423620E-01
Slc37a2	31223	0.110883901	12782	1.180220E-01
Slc37a3	31224	0.158132244	16183	2.532557E-02
Slc37a4	31225	0.0815845	10706	2.507776E-01
Slc38a1	31226	0.069452674	9811	3.284537E-01
Slc38a10	31227	0.075904101	10343	2.854034E-01
Slc38a11	31228	-0.066856562	1139	3.468991E-01
Slc38a2	31229	0.134912364	14615	5.681817E-02
Slc38a3	31230	-0.124241866	88	7.963123E-02
Slc38a4	31231	0.059086314	9160	4.059197E-01
Slc38a5	31232	-0.066855996	1239.5	3.469032E-01
Slc38a6	31233	0.055661669	8965	4.337164E-01
Slc38a7	31234	-0.075334452	879	2.890448E-01
Slc38a8	31235	0.001096955	5365	9.877003E-01
Slc38a9	31236	0.256199012	19577	2.504519E-04
Slc39a1	31237	0.117454238	13490	9.764481E-02
Slc39a1-ps	31238		#N/A	1.000000E+00
Slc39a10	31239	-0.075847188	867	2.857658E-01
Slc39a11	31240	0.132383344	14443	6.166717E-02
Slc39a12	31241	-0.082250661	614	2.469163E-01
Slc39a13	31242	0.187140568	17723	7.967067E-03
Slc39a14	31243	0.156975503	16105	2.643002E-02
Slc39a2	31244	0.069202065	9793	3.302062E-01
Slc39a3	31245	0.137779751	14809	5.170575E-02
Slc39a4	31246	0.284338868	19828	4.493913E-05
Slc39a5	31247	-0.086746249	548	2.219378E-01
Slc39a6	31248	0.174903228	17160	1.324453E-02
Slc39a7	31249	0.20812637	18527	3.102898E-03
Slc39a8	31250	0.024187263	6762	7.338827E-01
Slc39a9	31251	0.179307878	17374	1.106860E-02
Slc3a1	31252	0.214090471	18716	2.333830E-03
Slc3a2	31253	0.181427232	17463	1.013880E-02
Slc40a1	31254	-0.038406171	3065	5.892384E-01
Slc41a1	31255	0.01692085	6166	8.120253E-01
Slc41a2	31256	0.068454084	9738	3.354726E-01
Slc41a3	31257	-0.083949924	592	2.372545E-01
Slc43a1	31258	0.112981682	12927	1.111845E-01
Slc43a2	31259	0.042215923	7753	5.528159E-01
Slc43a3	31260	0.094108833	11649	1.850049E-01

Spearman Rank correlation analysis performed between Prdm1 and all-expressed genes within the Meredith RNA-seq dataset. Robust Prdm1–associated genes were identified using a cut-off of $p < 0.0005$.

Table S1, Related to Supplemental Figure 3C. Prdm1 associated genes

Slc44a1	31261	0.275929415	19764	7.659077E-05
Slc44a2	31262	0.007979238	5679	9.107140E-01
Slc44a3	31263	0.065712462	9572	3.552316E-01
Slc44a4	31264	0.096948617	11832	1.720376E-01
Slc44a5	31265	0.097146765	11846	1.711586E-01
Slc45a1	31266	-0.086755146	452	2.218903E-01
Slc45a2	31267	-0.054451513	1703	4.437940E-01
Slc45a3	31268	0.046553617	8048	5.127280E-01
Slc45a4	31269	0.135440537	14659	5.584630E-02
Slc46a1	31270	0.147232759	15491	3.748096E-02
Slc46a2	31271	0.129126473	14248	6.840728E-02
Slc46a3	31272	-0.085466977	565	2.288554E-01
Slc47a1	31273	-0.014440265	4546	8.391767E-01
Slc47a2	31274	-0.066855996	1239.5	3.469032E-01
Slc48a1	31275	0.135094948	14625	5.648063E-02
Slc4a1	31276	0.143605682	15224	4.249002E-02
Slc4a10	31277	0.119431749	13621	9.209133E-02
Slc4a11	31278	0.092399396	11541	1.931490E-01
Slc4a1ap	31279	0.105439455	12387	1.372956E-01
Slc4a2	31280	0.081310682	10685	2.523768E-01
Slc4a3	31281	-0.077394997	736.5	2.760195E-01
Slc4a4	31282	0.284483439	19829	4.452242E-05
Slc4a5	31283	0.074574898	10201.5	2.939481E-01
Slc4a7	31284	0.179305908	17373	1.106950E-02
Slc4a8	31285	0.010143876	5778	8.866373E-01
Slc4a9	31286	0.216180282	18792	2.108369E-03
Slc50a1	31287	0.100920759	12115	1.550489E-01
Slc51a	31288	0.082739909	10808	2.441069E-01
Slc51b	31289	0.266037022	19685	1.403246E-04
Slc52a2	31290	0.104118483	12300	1.423165E-01
Slc52a3	31291	-0.110562037	181.5	1.190994E-01
Slc5a1	31292	0.085872682	11046	2.266453E-01
Slc5a10	31293	0.108618218	12600	1.257698E-01
Slc5a11	31294	0.074180003	10149	2.965191E-01
Slc5a12	31295	-0.066856562	1139	3.468991E-01
Slc5a2	31296	-0.038406171	3065	5.892384E-01
Slc5a3	31297		#N/A	1.000000E+00
Slc5a4a	31298	-0.038406171	3065	5.892384E-01
Slc5a4b	31299	0.081754989	10727	2.497854E-01
Slc5a5	31300	0.042567653	7779	5.495093E-01
Slc5a6	31301	-0.042064613	2434	5.542414E-01
Slc5a7	31302	-0.054451513	1703	4.437940E-01
Slc5a8	31303	0.013232239	5948	8.524704E-01

Spearman Rank correlation analysis performed between Prdm1 and all-expressed genes within the Meredith RNA-seq dataset. Robust Prdm1–associated genes were identified using a cut-off of $p < 0.0005$.

Table S1, Related to Supplemental Figure 3C. Prdm1 associated genes

Slc5a9	31304	0.024594892	6794	7.295709E-01
Slc6a1	31305	-0.045149205	2323	5.255407E-01
Slc6a11	31306	0.162172763	16479	2.177313E-02
Slc6a12	31307		#N/A	1.000000E+00
Slc6a13	31308	-0.009446023	4766	8.943891E-01
Slc6a14	31309	-0.035485624	3700	6.178822E-01
Slc6a15	31310	0.100438829	12087	1.570400E-01
Slc6a16	31311	-0.038406171	3065	5.892384E-01
Slc6a17	31312	-0.054451513	1703	4.437940E-01
Slc6a18	31313	0.078155799	10475	2.713123E-01
Slc6a19	31314	-0.110568513	165.5	1.190777E-01
Slc6a2	31315	0.081988533	10744	2.484307E-01
Slc6a20a	31316	0.123581851	13877	8.125575E-02
Slc6a20b	31317		#N/A	1.000000E+00
Slc6a3	31318	-0.077394997	736.5	2.760195E-01
Slc6a4	31319	0.041554421	7715	5.590608E-01
Slc6a5	31320	0.084415652	10950	2.346533E-01
Slc6a6	31321	0.125764729	14020	7.598282E-02
Slc6a7	31322	-0.038738669	2496	5.860162E-01
Slc6a8	31323	0.106088049	12432	1.348805E-01
Slc6a9	31324	-0.00527424	4916	9.409133E-01
Slc7a1	31325	0.066256492	9605	3.512539E-01
Slc7a10	31326	0.071768225	9977	3.125446E-01
Slc7a11	31327	-0.041089781	2449	5.634674E-01
Slc7a12	31328	-0.038406171	3065	5.892384E-01
Slc7a13	31329	0.146690283	15460	3.819681E-02
Slc7a14	31330	0.079912948	10587	2.606501E-01
Slc7a15	31331	0.001382891	5384	9.844945E-01
Slc7a2	31332	0.003139914	5483	9.648033E-01
Slc7a3	31333	-0.018496204	4300	7.948958E-01
Slc7a4	31334	-0.086746694	532	2.219355E-01
Slc7a5	31335	0.028010342	6974	6.937875E-01
Slc7a6	31336	0.097075886	11842	1.714727E-01
Slc7a6os	31337	0.148642793	15571	3.567310E-02
Slc7a7	31338	0.211880101	18649	2.595950E-03
Slc7a8	31339	-0.01652847	4458	8.163061E-01
Slc7a9	31340	0.185468231	17660	8.555374E-03
Slc8a1	31341	0.164127915	16599	2.021456E-02
Slc8a2	31342	-0.001989518	5074	9.776943E-01
Slc8a3	31343	0.108561242	12587	1.259696E-01
Slc9a1	31344	0.093310384	11601	1.887769E-01
Slc9a2	31345	-0.050191301	2194	4.803075E-01
Slc9a3	31346	-0.038406171	3065	5.892384E-01

Spearman Rank correlation analysis performed between Prdm1 and all-expressed genes within the Meredith RNA-seq dataset. Robust Prdm1–associated genes were identified using a cut-off of $p < 0.0005$.

Table S1, Related to Supplemental Figure 3C. Prdm1 associated genes

Slc9a3r1	31347	0.134244328	14575	5.806736E-02
Slc9a3r2	31348	-0.018447724	4312.5	7.954215E-01
Slc9a4	31349	-0.032445878	3830.5	6.483195E-01
Slc9a5	31350	-0.046051185	2286	5.172931E-01
Slc9a6	31351	-0.016707059	4451	8.143571E-01
Slc9a7	31352	-0.009276233	4773	8.962766E-01
Slc9a8	31353	-0.037408577	3649	5.989540E-01
Slc9a9	31354	-0.029167658	3961	6.818098E-01
Slc9b1	31355	0.090938232	11429	2.003149E-01
Slc9b2	31356	-0.038406171	3065	5.892384E-01
Slc9c1	31357	-0.034769424	3718	6.249977E-01
Slco1a1	31358	-0.032450477	3820	6.482730E-01
Slco1a4	31359	0.063365727	9418	3.727114E-01
Slco1a5	31360	0.098707923	11973	1.643500E-01
Slco1a6	31361	-0.0187867	4293	7.917475E-01
Slco1b2	31362		#N/A	1.000000E+00
Slco1c1	31363	-0.003543874	5005	9.602786E-01
Slco2a1	31364	0.045116673	7949	5.258394E-01
Slco2b1	31365	-0.054450825	1984	4.437997E-01
Slco3a1	31366	0.13863007	14861	5.026526E-02
Slco4a1	31367	0.026850208	6922	7.058710E-01
Slco4c1	31368	0.108607082	12591.5	1.258088E-01
Slco5a1	31369	-0.009429813	4767	8.945692E-01
Slco6b1	31370	0.109492511	12666.5	1.227347E-01
Slco6c1	31371	0.095309088	11722	1.794392E-01
Slco6d1	31372	-0.05643476	1471.5	4.273478E-01
Slfn1	31373	0.087820071	11186	2.162470E-01
Slfn10-ps	31374	-0.055662515	1500	4.337094E-01
Slfn14	31375	-0.054451513	1703	4.437940E-01
Slfn2	31376	0.049899036	8290	4.828707E-01
Slfn3	31377	0.020120083	6336	7.773408E-01
Slfn4	31378	0.09092839	11426	2.003638E-01
Slfn5	31379	-0.06666503	1285	3.482854E-01
Slfn8	31380	0.062701453	9372	3.777537E-01
Slfn9	31381	0.08133953	10690	2.522080E-01
Slfnl1	31382		#N/A	1.000000E+00
Slirp	31383	0.197850158	18159	4.980376E-03
Slit1	31384	0.104201783	12307	1.419958E-01
Slit2	31385	-0.012346688	4657	8.622422E-01
Slit3	31386	0.041270677	7704	5.617498E-01
Slitrk1	31387	0.047153255	8091.5	5.073070E-01
Slitrk2	31388	-0.039951293	2472	5.743344E-01
Slitrk3	31389		#N/A	1.000000E+00

Spearman Rank correlation analysis performed between Prdm1 and all-expressed genes within the Meredith RNA-seq dataset. Robust Prdm1–associated genes were identified using a cut-off of $p < 0.0005$.

Table S1, Related to Supplemental Figure 3C. Prdm1 associated genes

Slitrk4	31390	0.205816029	18452	3.457848E-03
Slitrk5	31391	0.298518994	19899	1.758458E-05
Slitrk6	31392	0.051225809	8399.5	4.712944E-01
Slk	31393	0.054248928	8879	4.454939E-01
Slmap	31394	0.118988795	13588	9.331288E-02
Slmo1	31395	0.049150675	8226	4.894672E-01
Slmo2	31396	0.184366851	17613	8.963577E-03
Sln	31397		#N/A	1.000000E+00
Slpi	31398	0.229102371	19122	1.101614E-03
Sltm	31399	0.199730177	18237	4.574920E-03
Slu7	31400	0.119547418	13630	9.177446E-02
Slurp1	31401	0.075328288	10286.5	2.890843E-01
Slx	31402	0.045056653	7941.5	5.263908E-01
Slx1b	31403	0.23300307	19229	8.991218E-04
Slx4	31404	0.077649918	10450	2.744362E-01
Slx4ip	31405	0.063334118	9412	3.729504E-01
Slxl1	31406		#N/A	1.000000E+00
Smad1	31407	0.144316756	15326	4.146600E-02
Smad2	31408	0.088987073	11269	2.101815E-01
Smad3	31409	0.145946736	15407	3.919670E-02
Smad4	31410	0.132009692	14408	6.241161E-02
Smad5	31411	0.004354781	5533	9.511998E-01
Smad6	31412	0.03137771	7151	6.591587E-01
Smad7	31413	0.151163935	15723	3.262446E-02
Smad9	31414	0.212381963	18671	2.534196E-03
Smagp	31415	-0.032195328	3836	6.508554E-01
Smap1	31416	0.070126645	9848	3.237703E-01
Smap2	31417	0.193418396	17965	6.066488E-03
Smarca1	31418	0.054595506	8901	4.425880E-01
Smarca2	31419	0.199704174	18236	4.580320E-03
Smarca4	31420	0.109208044	12638	1.237159E-01
Smarca5	31421	0.088717249	11253	2.115729E-01
Smarca5-ps	31422	0.021317126	6513.5	7.644715E-01
Smarcad1	31423	0.050750395	8359	4.754249E-01
Smarcal1	31424	0.13932704	14911	4.910958E-02
Smarcb1	31425	0.037385168	7449	5.991829E-01
Smarcc1	31426	0.128608613	14217	6.953241E-02
Smarcc2	31427	0.22515819	19050	1.348184E-03
Smarcd1	31428	0.096331579	11795	1.747961E-01
Smarcd2	31429	0.190334984	17854	6.942396E-03
Smarcd3	31430	0.203601309	18374.5	3.832123E-03
Smarce1	31431	0.154759394	15968	2.866161E-02
Smc1a	31432	0.028754877	7004	6.860730E-01

Spearman Rank correlation analysis performed between Prdm1 and all-expressed genes within the Meredith RNA-seq dataset. Robust Prdm1–associated genes were identified using a cut-off of $p < 0.0005$.

Table S1, Related to Supplemental Figure 3C. Prdm1 associated genes

Smc1b	31433	-0.124244806	84	7.962405E-02
Smc2	31434	0.206469411	18476	3.353920E-03
Smc3	31435	0.199292416	18216	4.666585E-03
Smc4	31436	0.29850938	19898	1.759607E-05
Smc5	31437	0.069546609	9818	3.277984E-01
Smc6	31438	0.099633887	12034	1.604084E-01
Smchd1	31439	0.242435129	19404	5.425790E-04
Smco1	31440	-0.040023176	2470	5.736454E-01
Smco2	31441		#N/A	1.000000E+00
Smco3	31442		#N/A	1.000000E+00
Smco4	31443	0.030944387	7125	6.635763E-01
Smcp	31444	0.229057459	19118.5	1.104172E-03
Smcr7	31445	0.084657207	10964	2.333121E-01
Smcr7l	31446	0.16339032	16569	2.079081E-02
Smcr8	31447	-0.011232471	4694	8.745668E-01
Smdt1	31448	0.079209815	10544	2.648816E-01
Smek1	31449	0.159205641	16237	2.433638E-02
Smek2	31450	0.203114941	18363	3.919056E-03
Smek3-ps	31451		#N/A	1.000000E+00
Smg1	31452	0.14872864	15576	3.556546E-02
Smg5	31453	0.089425812	11305	2.079330E-01
Smg6	31454	0.03286026	7228	6.441343E-01
Smg7	31455	0.108983942	12620	1.244932E-01
Smg8	31456	-0.025688152	4076	7.180487E-01
Smg9	31457	-0.077393517	797	2.760287E-01
Smgc	31458		#N/A	1.000000E+00
Smim1	31459	-0.001606757	5107.5	9.819849E-01
Smim11	31460	0.041638622	7720	5.582640E-01
Smim12	31461	0.056516733	9008	4.266757E-01
Smim13	31462	0.075876742	10338	2.855776E-01
Smim14	31463	0.066220393	9603	3.515170E-01
Smim15	31464	0.238498968	19338	6.715092E-04
Smim17	31465		#N/A	1.000000E+00
Smim18	31466		#N/A	1.000000E+00
Smim19	31467	0.02366033	6730	7.394688E-01
Smim20	31468	-0.049384669	2211	4.873995E-01
Smim3	31469	0.003456358	5496	9.612588E-01
Smim4	31470	0.090490874	11379	2.025469E-01
Smim5	31471	0.093208816	11597	1.892608E-01
Smim6	31472	-0.038406171	3065	5.892384E-01
Smim7	31473	0.056217834	8995	4.291293E-01
Smim8	31474	0.255235959	19568	2.647453E-04
Smim9	31475		#N/A	1.000000E+00

Spearman Rank correlation analysis performed between Prdm1 and all-expressed genes within the Meredith RNA-seq dataset. Robust Prdm1–associated genes were identified using a cut-off of $p < 0.0005$.

Table S1, Related to Supplemental Figure 3C. Prdm1 associated genes

Smlr1	31476		#N/A	1.000000E+00
Smn1	31477	0.010611799	5798	8.814455E-01
Smndc1	31478	0.253001867	19537	3.008759E-04
Smo	31479	0.163300283	16559	2.086211E-02
Smoc1	31480	0.047864772	8132	5.009135E-01
Smoc2	31481	0.293724105	19879	2.428546E-05
Smok2a	31482		#N/A	1.000000E+00
Smok2b	31483	-0.054451513	1703	4.437940E-01
Smok3a	31484	0.113548679	13094	1.093908E-01
Smok3b	31485	-0.038406171	3065	5.892384E-01
Smok3c	31486		#N/A	1.000000E+00
Smok4a	31487	-0.054450825	1984	4.437997E-01
Smox	31488	0.060692045	9244	3.932588E-01
Smpd1	31489	0.278812439	19784	6.391898E-05
Smpd2	31490	0.094830515	11692	1.816434E-01
Smpd3	31491		#N/A	1.000000E+00
Smpd4	31492	0.077672447	10453	2.742965E-01
Smpd5	31493		#N/A	1.000000E+00
Smpdl3a	31494	0.083334035	10855	2.407252E-01
Smpdl3b	31495	0.007792416	5670	9.127962E-01
Smpx	31496	0.025142859	6832.5	7.237880E-01
Smr2	31497	-0.038406171	3065	5.892384E-01
Smr3a	31498		#N/A	1.000000E+00
Sms	31499	0.148208375	15544	3.622205E-02
Smt3h1-ps	31500		#N/A	1.000000E+00
Smt3h2-ps	31501	-0.054451513	1703	4.437940E-01
Smt3h2-ps2	31502		#N/A	1.000000E+00
Smt3h2-ps4	31503		#N/A	1.000000E+00
Smtn	31504		#N/A	1.000000E+00
Smtnl1	31505	0.054917786	8919	4.398955E-01
Smtnl2	31506	0.030721573	7109	6.658524E-01
Smu1	31507	0.113902139	13249	1.082841E-01
Smug1	31508	-0.005112384	4928	9.427234E-01
Smurf1	31509	0.050209884	8318	4.801448E-01
Smurf2	31510	-0.10516674	206	1.383209E-01
Smyd1	31511	0.206902089	18488	3.286659E-03
Smyd2	31512	0.007650981	5664	9.143728E-01
Smyd3	31513	0.108874637	12615	1.248737E-01
Smyd4	31514	0.22522293	19053	1.343758E-03
Smyd5	31515	-0.087074	442	2.201897E-01
Snai1	31516		#N/A	1.000000E+00
Snai2	31517	0.125608834	14011	7.634997E-02
Snai3	31518	0.113548679	13094	1.093908E-01

Spearman Rank correlation analysis performed between Prdm1 and all-expressed genes within the Meredith RNA-seq dataset. Robust Prdm1–associated genes were identified using a cut-off of $p < 0.0005$.

Table S1, Related to Supplemental Figure 3C. Prdm1 associated genes

Snap23	31519	0.047796652	8128	5.015238E-01
Snap25	31520	0.131318243	14368	6.380865E-02
Snap29	31521	-0.074385774	896	2.951776E-01
Snap47	31522	0.101168846	12128	1.540314E-01
Snap91	31523	-0.005257605	4918	9.410993E-01
Snapc1	31524	0.220251536	18905	1.725124E-03
Snapc2	31525	0.095592044	11742	1.781453E-01
Snapc3	31526	0.189931632	17837	7.064945E-03
Snapc4	31527	0.062129491	9333.5	3.821286E-01
Snapc5	31528	0.081157102	10679	2.532769E-01
Snapin	31529	0.039839206	7629	5.754096E-01
Snca	31530	0.083657414	10893	2.388985E-01
Sncaip	31531	0.208674366	18541	3.023699E-03
Sncb	31532	0.073162306	10077	3.032136E-01
Sncg	31533	-0.054066211	2110	4.470302E-01
Snd1	31534	0.166585562	16752	1.839318E-02
Sned1	31535	0.031707269	7171	6.558068E-01
Snf8	31536	0.172074609	17026	1.483219E-02
Snhg11	31537	0.093291836	11599	1.888652E-01
Snhg12	31538	0.18601742	17682	8.358060E-03
Snhg3	31539	0.125084994	13975	7.759422E-02
Snhg5	31540	0.229159963	19125	1.098342E-03
Snhg6	31541	0.049183897	8233	4.891733E-01
Snhg9	31542	0.073959456	10134	2.979615E-01
Snip1	31543	0.113870773	13246	1.083819E-01
Snn	31544	0.074044339	10139	2.974058E-01
Snora15	31545		#N/A	1.000000E+00
Snora16a	31546		#N/A	1.000000E+00
Snora17	31547		#N/A	1.000000E+00
Snora19	31548		#N/A	1.000000E+00
Snora20	31549		#N/A	1.000000E+00
Snora21	31550		#N/A	1.000000E+00
Snora23	31551		#N/A	1.000000E+00
Snora24	31552	0.154064684	15894.5	2.939352E-02
Snora26	31553		#N/A	1.000000E+00
Snora28	31554		#N/A	1.000000E+00
Snora2b	31555		#N/A	1.000000E+00
Snora3	31556		#N/A	1.000000E+00
Snora30	31557		#N/A	1.000000E+00
Snora31	31558		#N/A	1.000000E+00
Snora33	31559		#N/A	1.000000E+00
Snora34	31560		#N/A	1.000000E+00
Snora35	31561		#N/A	1.000000E+00

Spearman Rank correlation analysis performed between Prdm1 and all-expressed genes within the Meredith RNA-seq dataset. Robust Prdm1–associated genes were identified using a cut-off of $p < 0.0005$.

Table S1, Related to Supplemental Figure 3C. Prdm1 associated genes

Snora36b	31562		#N/A	1.000000E+00
Snora41	31563		#N/A	1.000000E+00
Snora43	31564		#N/A	1.000000E+00
Snora44	31565		#N/A	1.000000E+00
Snora47	31566		#N/A	1.000000E+00
Snora52	31567		#N/A	1.000000E+00
Snora5c	31568		#N/A	1.000000E+00
Snora61	31569		#N/A	1.000000E+00
Snora62	31570		#N/A	1.000000E+00
Snora64	31571		#N/A	1.000000E+00
Snora65	31572		#N/A	1.000000E+00
Snora68	31573		#N/A	1.000000E+00
Snora69	31574		#N/A	1.000000E+00
Snora70	31575		#N/A	1.000000E+00
Snora73a	31576		#N/A	1.000000E+00
Snora73b	31577		#N/A	1.000000E+00
Snora74a	31578		#N/A	1.000000E+00
Snora75	31579		#N/A	1.000000E+00
Snora78	31580		#N/A	1.000000E+00
Snora7a	31581		#N/A	1.000000E+00
Snora81	31582		#N/A	1.000000E+00
Snord100	31583		#N/A	1.000000E+00
Snord104	31584	0.048375215	8166	4.963531E-01
Snord107	31585		#N/A	1.000000E+00
Snord11	31586		#N/A	1.000000E+00
Snord110	31587		#N/A	1.000000E+00
Snord111	31588		#N/A	1.000000E+00
Snord116l1	31589		#N/A	1.000000E+00
Snord116l2	31590		#N/A	1.000000E+00
Snord118	31591	0.049751717	8273	4.841654E-01
Snord12	31592		#N/A	1.000000E+00
Snord123	31593		#N/A	1.000000E+00
Snord13	31594	0.160999982	16425	2.275698E-02
Snord14a	31595		#N/A	1.000000E+00
Snord14c	31596		#N/A	1.000000E+00
Snord14d	31597		#N/A	1.000000E+00
Snord14e	31598		#N/A	1.000000E+00
Snord15a	31599		#N/A	1.000000E+00
Snord15b	31600		#N/A	1.000000E+00
Snord16a	31601		#N/A	1.000000E+00
Snord17	31602		#N/A	1.000000E+00
Snord19	31603		#N/A	1.000000E+00
Snord1a	31604		#N/A	1.000000E+00

Spearman Rank correlation analysis performed between Prdm1 and all-expressed genes within the Meredith RNA-seq dataset. Robust Prdm1–associated genes were identified using a cut-off of $p < 0.0005$.

Table S1, Related to Supplemental Figure 3C. Prdm1 associated genes

Snord1b	31605		#N/A	1.000000E+00
Snord1c	31606		#N/A	1.000000E+00
Snord2	31607		#N/A	1.000000E+00
Snord21	31608		#N/A	1.000000E+00
Snord22	31609	-0.066855996	1239.5	3.469032E-01
Snord23	31610		#N/A	1.000000E+00
Snord32a	31611		#N/A	1.000000E+00
Snord33	31612		#N/A	1.000000E+00
Snord34	31613		#N/A	1.000000E+00
Snord35a	31614		#N/A	1.000000E+00
Snord35b	31615		#N/A	1.000000E+00
Snord37	31616		#N/A	1.000000E+00
Snord38a	31617		#N/A	1.000000E+00
Snord42a	31618		#N/A	1.000000E+00
Snord42b	31619		#N/A	1.000000E+00
Snord45b	31620		#N/A	1.000000E+00
Snord45c	31621		#N/A	1.000000E+00
Snord47	31622		#N/A	1.000000E+00
Snord49a	31623		#N/A	1.000000E+00
Snord49b	31624		#N/A	1.000000E+00
Snord4a	31625		#N/A	1.000000E+00
Snord52	31626		#N/A	1.000000E+00
Snord53	31627		#N/A	1.000000E+00
Snord55	31628		#N/A	1.000000E+00
Snord57	31629		#N/A	1.000000E+00
Snord58b	31630		#N/A	1.000000E+00
Snord61	31631		#N/A	1.000000E+00
Snord64	31632		#N/A	1.000000E+00
Snord65	31633	0.083452862	10867	2.400529E-01
Snord66	31634		#N/A	1.000000E+00
Snord67	31635		#N/A	1.000000E+00
Snord68	31636		#N/A	1.000000E+00
Snord69	31637		#N/A	1.000000E+00
Snord7	31638		#N/A	1.000000E+00
Snord70	31639		#N/A	1.000000E+00
Snord71	31640		#N/A	1.000000E+00
Snord72	31641		#N/A	1.000000E+00
Snord73a	31642		#N/A	1.000000E+00
Snord8	31643		#N/A	1.000000E+00
Snord82	31644		#N/A	1.000000E+00
Snord83b	31645	-0.038406171	3065	5.892384E-01
Snord85	31646		#N/A	1.000000E+00
Snord87	31647	0.164478601	16623.5	1.994547E-02

Spearman Rank correlation analysis performed between Prdm1 and all-expressed genes within the Meredith RNA-seq dataset. Robust Prdm1–associated genes were identified using a cut-off of $p < 0.0005$.

Table S1, Related to Supplemental Figure 3C. Prdm1 associated genes

Snord88a	31648		#N/A	1.000000E+00
Snord88c	31649		#N/A	1.000000E+00
Snord89	31650		#N/A	1.000000E+00
Snord90	31651		#N/A	1.000000E+00
Snord91a	31652		#N/A	1.000000E+00
Snord92	31653		#N/A	1.000000E+00
Snord93	31654		#N/A	1.000000E+00
Snord95	31655		#N/A	1.000000E+00
Snord96a	31656	0.082118947	10755	2.476764E-01
Snord98	31657		#N/A	1.000000E+00
Snord99	31658		#N/A	1.000000E+00
Snph	31659	-0.066856562	1139	3.468991E-01
Snrk	31660	0.210743724	18603	2.740857E-03
Snrnp200	31661	0.223352334	18996	1.477133E-03
Snrnp25	31662	0.048000826	8151	4.996958E-01
Snrnp27	31663	0.12919737	14252	6.825441E-02
Snrnp35	31664	-0.076981672	842	2.785999E-01
Snrnp40	31665	0.130537009	14326	6.541783E-02
Snrnp48	31666	0.152789424	15812	3.077864E-02
Snrnp70	31667	0.078305891	10485	2.703902E-01
Snrp1c-ps2	31668		#N/A	1.000000E+00
Snrpa	31669	0.162765609	16511	2.128993E-02
Snrpa1	31670	0.086080554	11059	2.255188E-01
Snrpb	31671	0.159612704	16256	2.397004E-02
Snrpb2	31672	0.215600048	18782	2.168892E-03
Snrpc	31673	0.213263972	18692	2.428887E-03
Snrpd1	31674	0.174940241	17162	1.322479E-02
Snrpd2	31675	0.205873435	18455	3.448602E-03
Snrpd3	31676	0.040642423	7673	5.677257E-01
Snrpe	31677	0.138299755	14845	5.082082E-02
Snrpf	31678	0.211741365	18645	2.613261E-03
Snrpg	31679	0.151928992	15761	3.174430E-02
Snrpn	31680	0.02500697	6821	7.252206E-01
Snta1	31681	0.015254993	6085	8.302364E-01
Sntb1	31682	0.10125951	12135	1.536608E-01
Sntb2	31683	0.030734408	7113	6.657212E-01
Sntg1	31684	0.135400049	14657	5.592031E-02
Sntg2	31685	0.044177355	7879	5.345013E-01
Sntn	31686	0.166148435	16721.5	1.870631E-02
Snupn	31687	0.113668967	13238	1.090132E-01
Snurf	31688		#N/A	1.000000E+00
Snw1	31689	0.162999933	16544	2.110151E-02
Snx1	31690	0.077693044	10454	2.741689E-01

Spearman Rank correlation analysis performed between Prdm1 and all-expressed genes within the Meredith RNA-seq dataset. Robust Prdm1–associated genes were identified using a cut-off of $p < 0.0005$.

Table S1, Related to Supplemental Figure 3C. Prdm1 associated genes

Snx10	31691	0.084076267	10926	2.365468E-01
Snx11	31692	0.193266289	17958	6.107263E-03
Snx12	31693	0.026788715	6915	7.065136E-01
Snx13	31694	0.144335439	15327	4.143938E-02
Snx14	31695	-0.027482356	4015	6.992774E-01
Snx15	31696	0.050310963	8327	4.792602E-01
Snx16	31697	0.14000531	14955	4.800624E-02
Snx17	31698	0.108530283	12585	1.260782E-01
Snx18	31699	0.148843104	15583	3.542236E-02
Snx19	31700	0.010246566	5782	8.854975E-01
Snx2	31701	0.115142132	13332	1.044712E-01
Snx20	31702	0.038223605	7492	5.910110E-01
Snx21	31703	0.020875719	6412.5	7.692098E-01
Snx22	31704	0.223350989	18995	1.477233E-03
Snx24	31705	0.083463466	10875	2.399929E-01
Snx25	31706	0.046621859	8057	5.121095E-01
Snx27	31707	0.229070652	19121	1.103420E-03
Snx29	31708	0.016606851	6151	8.154505E-01
Snx3	31709	0.184243536	17600	9.010338E-03
Snx30	31710	-0.047371657	2253	5.053400E-01
Snx31	31711	0.116603816	13441	1.001133E-01
Snx32	31712	0.150849887	15705	3.299171E-02
Snx33	31713	0.114526246	13296	1.063515E-01
Snx4	31714	0.118490694	13555	9.470187E-02
Snx5	31715	0.172957866	17077	1.431944E-02
Snx6	31716	0.001650877	5402	9.814903E-01
Snx7	31717	0.221938043	18956	1.585892E-03
Snx8	31718	0.117200845	13475	9.837522E-02
Snx9	31719	0.258910095	19614	2.139783E-04
Soat1	31720	0.178685363	17335	1.135562E-02
Soat2	31721	0.044217104	7880	5.341333E-01
Sobp	31722	0.168457218	16847	1.710337E-02
Socs1	31723	-0.064610467	1331	3.633750E-01
Socs2	31724	0.24879668	19493	3.816078E-04
Socs3	31725	-0.045648198	2308	5.209697E-01
Socs4	31726	0.172335658	17042	1.467900E-02
Socs5	31727	0.047377769	8108	5.052850E-01
Socs6	31728	0.018993853	6280	7.895045E-01
Socs7	31729	0.118054352	13531	9.593212E-02
Sod1	31730	0.225173411	19051	1.347142E-03
Sod2	31731	0.087229515	11144	2.193637E-01
Sod3	31732	0.013382325	5954	8.508164E-01
Soga1	31733	-0.023969724	4126	7.361872E-01

Spearman Rank correlation analysis performed between Prdm1 and all-expressed genes within the Meredith RNA-seq dataset. Robust Prdm1–associated genes were identified using a cut-off of $p < 0.0005$.

Soga2	31734	0.025659106	6856	7.183541E-01
Soga3	31735	0.289323167	19861	3.249773E-05
Sohlh1	31736	-0.124240559	90	7.963442E-02
Sohlh2	31737		#N/A	1.000000E+00
Solh	31738	-0.018790377	4285	7.917077E-01
Son	31739	0.160712491	16376	2.300392E-02
Sorbs1	31740	0.138912012	14876	4.979506E-02
Sorbs2	31741	0.160444111	16362	2.323653E-02
Sorbs3	31742	-0.054451513	1703	4.437940E-01
Sorcs1	31743	0.093804533	11631	1.864359E-01
Sorcs2	31744	0.12174226	13776	8.592485E-02
Sorcs3	31745	0.214247208	18724	2.316189E-03
Sord	31746	0.155395216	15998	2.800545E-02
Sorl1	31747	0.100611488	12098	1.563245E-01
Sort1	31748	-0.152923162	24	3.063082E-02
Sos1	31749	0.046541533	8047	5.128375E-01
Sos2	31750	0.202307956	18337	4.067223E-03
Sost	31751	0.041660817	7722	5.580541E-01
Sostdc1	31752	0.012338853	5894	8.623287E-01
Sowaha	31753	-0.018105422	4347	7.991361E-01
Sowahb	31754	0.07459229	10221	2.938352E-01
SOWAHC	31755	0.159905172	16272	2.370977E-02
Sowahc	31756		#N/A	1.000000E+00
Sowahd	31757	0.021316405	6469	7.644793E-01
Sox1	31758		#N/A	1.000000E+00
Sox10	31759	0.113548679	13094	1.093908E-01
Sox11	31760	-0.038406171	3065	5.892384E-01
Sox12	31761	0.037577761	7463	5.973013E-01
Sox13	31762	0.220431003	18913	1.709795E-03
Sox14	31763		#N/A	1.000000E+00
Sox15	31764	0.137863847	14813	5.156178E-02
Sox17	31765	-0.038406171	3065	5.892384E-01
Sox18	31766	-0.038406171	3065	5.892384E-01
Sox2	31767	0.021316405	6469	7.644793E-01
Sox21	31768	0.094344639	11663	1.839016E-01
Sox2ot	31769	0.038276817	7514	5.904941E-01
Sox3	31770		#N/A	1.000000E+00
Sox30	31771	0.075715395	10317.5	2.866063E-01
Sox4	31772	0.095905714	11763	1.767190E-01
Sox5	31773	0.148635267	15570	3.568255E-02
Sox6	31774	-0.003819257	4990	9.571948E-01
Sox7	31775	0.003506328	5498	9.606991E-01
Sox8	31776	-0.017780336	4378	8.026681E-01

Spearman Rank correlation analysis performed between Prdm1 and all-expressed genes within the Meredith RNA-seq dataset. Robust Prdm1–associated genes were identified using a cut-off of $p < 0.0005$.

Table S1, Related to Supplemental Figure 3C. Prdm1 associated genes

Sox9	31777	0.19880294	18189	4.771030E-03
Sp1	31778	0.13397604	14549	5.857538E-02
Sp100	31779	0.140533423	14990	4.716155E-02
Sp110	31780	0.011397544	5847	8.727389E-01
Sp140	31781	0.14526764	15367	4.012911E-02
Sp2	31782	0.117547335	13495	9.737754E-02
Sp3	31783	0.172571004	17052	1.454208E-02
Sp4	31784	-0.013597452	4586	8.484468E-01
Sp5	31785		#N/A	1.000000E+00
Sp6	31786	-0.020151631	4233	7.770009E-01
Sp7	31787	-0.038406171	3065	5.892384E-01
Sp8	31788	0.024792811	6808	7.274804E-01
Sp9	31789	0.139943292	14946	4.810626E-02
Spa17	31790	0.04556997	7972	5.216850E-01
Spaca1	31791	-0.038406171	3065	5.892384E-01
Spaca3	31792	0.132899707	14471	6.065043E-02
Spaca4	31793		#N/A	1.000000E+00
Spaca5	31794		#N/A	1.000000E+00
Spaca7	31795		#N/A	1.000000E+00
Spag1	31796	-0.008753314	4798	9.020936E-01
Spag11a	31797		#N/A	1.000000E+00
Spag11b	31798		#N/A	1.000000E+00
Spag16	31799	0.020510341	6371	7.731384E-01
Spag17	31800	0.225815093	19069	1.303893E-03
Spag4	31801		#N/A	1.000000E+00
Spag5	31802	0.198138557	18172	4.916155E-03
Spag6	31803	0.024273369	6772	7.329712E-01
Spag7	31804	0.179212061	17368	1.111235E-02
Spag8	31805		#N/A	1.000000E+00
Spag9	31806	0.087939415	11202	2.156211E-01
Spam1	31807		#N/A	1.000000E+00
Spanxn4	31808		#N/A	1.000000E+00
Sparc	31809	0.078602395	10504	2.685747E-01
Sparcl1	31810	-0.054450825	1984	4.437997E-01
Spast	31811	0.227423913	19092	1.200990E-03
Spata1	31812	0.093709459	11628	1.868847E-01
Spata13	31813	0.098839221	11984	1.637867E-01
Spata16	31814	0.073949455	10132	2.980270E-01
Spata17	31815	-0.009006825	4784	8.992729E-01
Spata18	31816	0.031631551	7167	6.565763E-01
Spata19	31817		#N/A	1.000000E+00
Spata2	31818	-0.057444808	1445	4.191092E-01
Spata20	31819		#N/A	1.000000E+00

Spearman Rank correlation analysis performed between Prdm1 and all-expressed genes within the Meredith RNA-seq dataset. Robust Prdm1–associated genes were identified using a cut-off of $p < 0.0005$.

Table S1, Related to Supplemental Figure 3C. Prdm1 associated genes

Spata21	31820	-0.038406171	3065	5.892384E-01
Spata22	31821	0.112464606	12887	1.128403E-01
Spata24	31822	0.12673398	14087	7.373205E-02
Spata25	31823		#N/A	1.000000E+00
Spata2l	31824	0.083452862	10867	2.400529E-01
Spata3	31825		#N/A	1.000000E+00
Spata31	31826		#N/A	1.000000E+00
Spata31d1a	31827		#N/A	1.000000E+00
Spata31d1b	31828		#N/A	1.000000E+00
Spata31d1c	31829		#N/A	1.000000E+00
Spata31d1d	31830		#N/A	1.000000E+00
Spata32	31831	-0.038406171	3065	5.892384E-01
Spata4	31832		#N/A	1.000000E+00
Spata5	31833	0.092883493	11573	1.908166E-01
Spata5l1	31834		#N/A	1.000000E+00
Spata6	31835	0.046999521	8079	5.086939E-01
Spata7	31836	-0.065884327	1308	3.539720E-01
Spata9	31837	-0.054451513	1703	4.437940E-01
Spatc1	31838	0.053267784	8685	4.537786E-01
Spatc1l	31839		#N/A	1.000000E+00
Spats1	31840		#N/A	1.000000E+00
Spats2	31841	-0.098665272	289	1.645333E-01
Spats2l	31842	0.058555138	9131	4.101605E-01
Spc24	31843	0.405043902	20029	2.694154E-09
Spc25	31844	0.026300382	6887	7.116238E-01
Spcs1	31845	0.185584159	17664	8.513382E-03
Spcs2	31846	0.224052961	19020	1.425821E-03
Spcs2-ps	31847	0.113548679	13094	1.093908E-01
Spcs3	31848	0.164034824	16596	2.028651E-02
Spdef	31849	-0.038771606	2493	5.856975E-01
Spdl1	31850	0.114574943	13298	1.062019E-01
Spdya	31851	-0.066858259	1029.5	3.468868E-01
Spdyb	31852		#N/A	1.000000E+00
Specc1	31853	0.094653308	11682	1.824646E-01
Specc1l	31854	0.06886529	9764	3.325708E-01
Speer1-ps1	31855	-0.038406171	3065	5.892384E-01
Speer2	31856		#N/A	1.000000E+00
Speer3	31857	-0.054451513	1703	4.437940E-01
Speer4a	31858		#N/A	1.000000E+00
Speer4b	31859	-0.038406171	3065	5.892384E-01
Speer4c	31860		#N/A	1.000000E+00
SPEER4D	31861		#N/A	1.000000E+00
Speer4d	31862		#N/A	1.000000E+00

Spearman Rank correlation analysis performed between Prdm1 and all-expressed genes within the Meredith RNA-seq dataset. Robust Prdm1–associated genes were identified using a cut-off of $p < 0.0005$.

Table S1, Related to Supplemental Figure 3C. Prdm1 associated genes

Speer4e	31863	0.022198129	6607.5	7.550408E-01
Speer4f	31864	0.053267784	8685	4.537786E-01
Speer5-ps1	31865	-0.037868917	3636	5.944618E-01
Speer6-ps1	31866		#N/A	1.000000E+00
Speer7-ps1	31867	0.053805701	8820	4.492258E-01
Spef1	31868	0.100340899	12081	1.574469E-01
Spef2	31869	0.222982619	18989	1.504886E-03
Speg	31870	-0.092585059	391	1.922520E-01
Spem1	31871		#N/A	1.000000E+00
Spen	31872	0.124416025	13934	7.920696E-02
Spert	31873		#N/A	1.000000E+00
Spesp1	31874	-0.054451513	1703	4.437940E-01
Spg11	31875	0.016324098	6137	8.185379E-01
Spg20	31876	0.00835679	5695	9.065081E-01
Spg21	31877	0.157802694	16165	2.563611E-02
Spg7	31878		#N/A	1.000000E+00
Sphk1	31879	0.01813582	6230	7.988061E-01
Sphk2	31880	0.182648098	17536	9.635122E-03
Sphkap	31881	0.151314638	15733	3.244946E-02
Spib	31882	0.169208985	16888	1.660791E-02
Spic	31883	-0.014801778	4519	8.352071E-01
Spice1	31884	0.083916611	10913	2.374413E-01
Spin1	31885	0.185237421	17648	8.639526E-03
Spin2	31886	0.246887369	19472.5	4.245310E-04
Spin4	31887	-0.038406171	3065	5.892384E-01
Spink10	31888	0.294707369	19883	2.274032E-05
Spink11	31889	0.084141322	10930.5	2.361831E-01
Spink12	31890	-0.0248247	4103	7.271437E-01
Spink13	31891	0.20976607	18570	2.871351E-03
Spink14	31892	0.022198129	6607.5	7.550408E-01
Spink2	31893	-0.066855996	1239.5	3.469032E-01
Spink3	31894	-0.012482271	4649	8.607447E-01
Spink4	31895	-0.054450825	1984	4.437997E-01
Spink5	31896	-0.092555965	392	1.923924E-01
Spink6	31897	0.113548679	13094	1.093908E-01
Spink7	31898		#N/A	1.000000E+00
Spink8	31899	0.022198316	6629.5	7.550388E-01
Spinkl	31900	-0.012908019	4626	8.560455E-01
Spint1	31901	0.051743174	8452	4.668218E-01
Spint2	31902	0.146124916	15422	3.895510E-02
Spint3	31903	0.113548679	13094	1.093908E-01
Spint4	31904		#N/A	1.000000E+00
Spint5	31905	0.053805701	8820	4.492258E-01

Spearman Rank correlation analysis performed between Prdm1 and all-expressed genes within the Meredith RNA-seq dataset. Robust Prdm1–associated genes were identified using a cut-off of $p < 0.0005$.

Table S1, Related to Supplemental Figure 3C. Prdm1 associated genes

Spire1	31906	0.127254216	14132	7.254647E-02
Spire2	31907		#N/A	1.000000E+00
Spn	31908	0.351965132	20006	3.207602E-07
Spns1	31909	0.148758893	15579	3.552759E-02
Spns2	31910	0.01287414	5926	8.564193E-01
Spns3	31911	0.06832006	9727	3.364219E-01
Spo11	31912	-0.081068745	631	2.537957E-01
Spocd1	31913		#N/A	1.000000E+00
Spock1	31914	0.222735975	18975	1.523664E-03
Spock2	31915	0.08294186	10825	2.429537E-01
Spock3	31916	0.0398935	7637	5.748887E-01
Spon1	31917	0.18229625	17516	9.777960E-03
Spon2	31918	-0.038406171	3065	5.892384E-01
Spop	31919	0.154562097	15959	2.886787E-02
Spopl	31920	0.075222767	10278	2.897623E-01
Spp1	31921	0.234597385	19264	8.266935E-04
Spp2	31922		#N/A	1.000000E+00
Sppl2a	31923	0.163246774	16555	2.090459E-02
Sppl2b	31924	0.118896449	13579	9.356916E-02
Sppl2c	31925	-0.038406171	3065	5.892384E-01
Sppl3	31926	0.123189399	13857	8.223430E-02
Spr	31927	0.173994558	17126	1.373748E-02
Spr-ps1	31928	-0.038406171	3065	5.892384E-01
Spred1	31929	-0.002226351	5064	9.750398E-01
Spred2	31930	0.079430328	10556	2.635495E-01
Spred3	31931	0.160304018	16323	2.335876E-02
Sprn	31932	0.021316405	6469	7.644793E-01
Sprr1a	31933	-0.078821171	657	2.672405E-01
Sprr1b	31934	-0.008464472	4802	9.053090E-01
Sprr2a1	31935		#N/A	1.000000E+00
Sprr2a2	31936		#N/A	1.000000E+00
Sprr2a3	31937	-0.013940904	4569	8.446666E-01
Sprr2b	31938	-0.084354669	585	2.349928E-01
Sprr2d	31939	-0.055608318	1503	4.341579E-01
Sprr2e	31940	-0.086748028	498.5	2.219283E-01
Sprr2f	31941	-0.038406171	3065	5.892384E-01
Sprr2g	31942		#N/A	1.000000E+00
Sprr2h	31943	0.053267784	8685	4.537786E-01
Sprr2i	31944	-0.055337258	1508	4.364050E-01
Sprr2j-ps	31945	0.028489997	6992	6.888139E-01
Sprr2k	31946	-0.054451513	1703	4.437940E-01
Sprr3	31947	-0.090114438	415	2.044389E-01
Sprr4	31948		#N/A	1.000000E+00

Spearman Rank correlation analysis performed between Prdm1 and all-expressed genes within the Meredith RNA-seq dataset. Robust Prdm1–associated genes were identified using a cut-off of $p < 0.0005$.

Table S1, Related to Supplemental Figure 3C. Prdm1 associated genes

Sprtn	31949	-0.077397959	691.5	2.760011E-01
Spry1	31950	0.092848801	11567	1.909831E-01
Spry2	31951	0.215379174	18760	2.192343E-03
Spry3	31952		#N/A	1.000000E+00
Spry4	31953	0.124268634	13925	7.956590E-02
Spryd3	31954	0.106939397	12493	1.317601E-01
Spryd4	31955	0.087542366	11172	2.177087E-01
Spryd7	31956	0.054227029	8878	4.456778E-01
Spsb1	31957	0.009488351	5749	8.939186E-01
Spsb2	31958	-0.006075519	4877	9.319569E-01
Spsb3	31959	-0.044731474	2349	5.293829E-01
Spsb4	31960	-0.010530057	4726	8.823521E-01
Spt1	31961	0.08712614	11139	2.199125E-01
Spta1	31962	0.068925207	9769	3.321493E-01
Sptan1	31963	0.005853281	5589	9.344402E-01
Sptb	31964	-0.009075138	4779	8.985130E-01
Sptbn1	31965	0.050928143	8379	4.738782E-01
Sptbn2	31966	0.075710566	10305	2.866371E-01
Sptbn4	31967	-0.067964302	967	3.389500E-01
Sptbn5	31968	0.049855249	8285.5	4.832553E-01
Sptlc1	31969	0.14668152	15459	3.820846E-02
Sptlc2	31970	-0.036159074	3682	6.112237E-01
Sptlc3	31971	0.03305682	7243	6.421529E-01
Sptssa	31972	0.180044363	17403	1.073730E-02
Sptssb	31973	0.139778661	14937	4.837261E-02
Spty2d1	31974	0.022849099	6683	7.480955E-01
Spz1	31975	-0.038406171	3065	5.892384E-01
Sqle	31976	0.058156734	9102	4.133584E-01
Sqrdl	31977	0.063477802	9431	3.718648E-01
Sqstm1	31978	0.093409987	11609	1.883033E-01
Sra1	31979	0.064840227	9528	3.616677E-01
Srbd1	31980	-0.030516634	3914	6.679485E-01
Src	31981	0.053396017	8767	4.526909E-01
Srcap	31982	0.036385581	7401	6.089913E-01
Srcin1	31983	-0.028547518	3977	6.882183E-01
Srcrb4d	31984	-0.099301017	283	1.618171E-01
Srd5a1	31985	-0.00625426	4873	9.299601E-01
Srd5a2	31986	-0.038406171	3065	5.892384E-01
Srd5a3	31987	0.279642941	19792	6.065153E-05
Srebf1	31988	0.138634452	14862	5.025792E-02
Srebf2	31989	0.186519429	17700	8.181241E-03
Srek1	31990	0.076048378	10350	2.844861E-01
Srek1ip1	31991	0.192786443	17944	6.237505E-03

Spearman Rank correlation analysis performed between Prdm1 and all-expressed genes within the Meredith RNA-seq dataset. Robust Prdm1–associated genes were identified using a cut-off of $p < 0.0005$.

Table S1, Related to Supplemental Figure 3C. Prdm1 associated genes

Srf	31992	0.237875946	19324	6.943350E-04
Srfbp1	31993	-0.025928942	4069	7.155194E-01
Srgap1	31994	-0.010645826	4719	8.810682E-01
Srgap2	31995	0.12100481	13725	8.785597E-02
Srgap3	31996	0.121716743	13774	8.599110E-02
Srgn	31997	0.11091734	12784	1.179105E-01
Sri	31998	0.109517889	12681	1.226474E-01
Srl	31999	-0.053025641	2138	4.558364E-01
Srm	32000	0.187552959	17747	7.827637E-03
Srms	32001	0.037700968	7469	5.960989E-01
Srp14	32002	0.040357966	7653	5.704414E-01
Srp19	32003	0.083517188	10879	2.396894E-01
Srp54a	32004		#N/A	1.000000E+00
Srp54b	32005		#N/A	1.000000E+00
Srp54c	32006		#N/A	1.000000E+00
Srp68	32007	0.107689547	12534	1.290569E-01
Srp72	32008	0.177541682	17289	1.190007E-02
Srp9	32009	0.19852229	18183	4.831858E-03
Srpk1	32010	0.050670104	8355	4.761244E-01
Srpk2	32011	0.112687876	12900	1.121230E-01
Srpk3	32012	0.044878115	7926	5.280325E-01
Srpr	32013	0.237796121	19322	6.973107E-04
Srprb	32014	0.165985233	16702	1.882440E-02
Srpx	32015	-0.012907981	4627	8.560460E-01
Srpx2	32016	0.149746926	15634	3.430949E-02
Srr	32017	0.062754563	9375	3.773491E-01
Srrd	32018	0.105177208	12369	1.382814E-01
Srrm1	32019	0.128582284	14215	6.959002E-02
Srrm2	32020	0.291990337	19870	2.725417E-05
Srrm3	32021	0.016516967	6143	8.164317E-01
Srrm4	32022	0.18388674	17589	9.146857E-03
Srrt	32023	0.058546809	9130	4.102273E-01
Srsf1	32024	0.131317387	14367	6.381039E-02
Srsf10	32025	0.078319822	10487	2.703047E-01
Srsf11	32026	0.08695697	11123	2.208128E-01
Srsf12	32027	0.083045154	10838	2.423654E-01
Srsf2	32028	0.073740273	10112	2.993996E-01
Srsf3	32029	0.15169926	15750	3.200645E-02
Srsf4	32030	0.12440715	13933	7.922853E-02
Srsf5	32031	0.135031661	14621	5.659744E-02
Srsf6	32032	0.170825248	16967	1.558486E-02
Srsf7	32033	0.056830449	9029	4.241092E-01
Srsf9	32034	0.19775091	18154	5.002650E-03

Spearman Rank correlation analysis performed between Prdm1 and all-expressed genes within the Meredith RNA-seq dataset. Robust Prdm1–associated genes were identified using a cut-off of $p < 0.0005$.

Table S1, Related to Supplemental Figure 3C. Prdm1 associated genes

Srsx	32035		#N/A	1.000000E+00
Srsy	32036		#N/A	1.000000E+00
Srxn1	32037	-0.044824587	2346	5.285252E-01
Sry	32038		#N/A	1.000000E+00
Ss18	32039	0.018918533	6273	7.903199E-01
Ss18l1	32040	0.12486703	13960	7.811674E-02
Ssb	32041	0.205792275	18450	3.461681E-03
Ssbp1	32042	0.145829229	15403	3.935672E-02
Ssbp2	32043	0.146594562	15453	3.832431E-02
Ssbp3	32044	0.074505388	10178	2.943996E-01
Ssbp4	32045	0.195688161	18071	5.486284E-03
Ssc5d	32046	-0.054450825	1984	4.437997E-01
Ssfa2	32047	-0.007560635	4838	9.153802E-01
Ssh1	32048	-0.04236961	2426	5.513699E-01
Ssh2	32049	0.134669609	14598	5.726952E-02
Ssh3	32050	0.088775354	11255	2.112727E-01
Ssmem1	32051	0.021316405	6469	7.644793E-01
Ssna1	32052	0.146208012	15426	3.884286E-02
Sspn	32053	0.121462195	13749	8.665420E-02
Sspo	32054	0.057535247	9062	4.183761E-01
Ssr1	32055	0.232731519	19226	9.120247E-04
Ssr2	32056	0.024755241	6802	7.278770E-01
Ssr3	32057	0.190466417	17863	6.902875E-03
Ssr4	32058	0.114779345	13309	1.055756E-01
Ssrp1	32059	0.077084016	10416	2.779594E-01
Sssca1	32060	0.119363858	13612	9.227772E-02
Sst	32061		#N/A	1.000000E+00
Sstr1	32062	0.141011971	15020	4.640688E-02
Sstr2	32063	-0.038406171	3065	5.892384E-01
Sstr3	32064	-0.009424485	4768	8.946285E-01
Sstr4	32065	-0.038406171	3065	5.892384E-01
Sstr5	32066	0.1544596	15936.5	2.897553E-02
Ssty1	32067		#N/A	1.000000E+00
SSTY2	32068		#N/A	1.000000E+00
Ssty2	32069		#N/A	1.000000E+00
Ssu2	32070		#N/A	1.000000E+00
Ssu72	32071	0.178495608	17327	1.144441E-02
Ssx2ip	32072	0.184996695	17636	8.728075E-03
Ssx9	32073		#N/A	1.000000E+00
Ssxa1	32074	0.143605682	15224	4.249002E-02
Ssxb1	32075		#N/A	1.000000E+00
Ssxb10	32076	0.109492511	12666.5	1.227347E-01
Ssxb2	32077		#N/A	1.000000E+00

Spearman Rank correlation analysis performed between Prdm1 and all-expressed genes within the Meredith RNA-seq dataset. Robust Prdm1–associated genes were identified using a cut-off of $p < 0.0005$.

Table S1, Related to Supplemental Figure 3C. Prdm1 associated genes

Ssxb3	32078		#N/A	1.000000E+00
Ssxb5	32079		#N/A	1.000000E+00
Ssxb6	32080		#N/A	1.000000E+00
Ssxb8	32081	0.052728522	8550.5	4.583686E-01
Ssxb9	32082		#N/A	1.000000E+00
St13	32083	0.196706959	18109	5.242415E-03
St14	32084	0.231263983	19191	9.847508E-04
St18	32085	0.180140736	17411	1.069460E-02
St3gal1	32086	0.05109929	8393	4.723917E-01
St3gal2	32087	0.169623823	16911	1.633989E-02
St3gal3	32088	0.079932416	10589	2.605336E-01
St3gal4	32089	-0.05380802	2124	4.492062E-01
St3gal5	32090	0.074994322	10249	2.912337E-01
St3gal6	32091	0.069238811	9796	3.299489E-01
St5	32092	0.126901196	14103	7.334928E-02
St6gal1	32093	0.208348157	18534	3.070621E-03
St6gal2	32094	-0.011656546	4679	8.698723E-01
St6galnac1	32095	0.099108572	12006	1.626358E-01
St6galnac2	32096	0.052562201	8506	4.597894E-01
St6galnac3	32097	0.040529228	7667	5.688056E-01
St6galnac4	32098	0.211124309	18615	2.691529E-03
St6galnac5	32099	-0.004369969	4971	9.510298E-01
St6galnac6	32100	0.194521161	18026	5.778076E-03
St7	32101	0.005704861	5584	9.360990E-01
St7l	32102	0.082806106	10816	2.437284E-01
St8sia1	32103	0.014420386	6036	8.393951E-01
St8sia2	32104	0.036502031	7406	6.078450E-01
St8sia3	32105	0.046892898	8068	5.096570E-01
St8sia4	32106	0.301733315	19911	1.411672E-05
St8sia5	32107	-0.054451513	1703	4.437940E-01
St8sia6	32108	0.165936503	16700	1.885978E-02
Stab1	32109	-0.038406171	3065	5.892384E-01
Stab2	32110	0.108670897	12601	1.255853E-01
Stac	32111	0.07432613	10166	2.955660E-01
Stac2	32112	0.223594551	19007	1.459206E-03
Stac3	32113	0.143605682	15224	4.249002E-02
Stag1	32114	0.184585473	17621	8.881202E-03
Stag2	32115	0.212577414	18679	2.510509E-03
Stag3	32116	0.113548679	13094	1.093908E-01
Stam	32117	0.212341562	18669	2.539117E-03
Stam2	32118	0.203771613	18380	3.802097E-03
Stambp	32119	0.067273138	9658	3.438960E-01
Stambpl1	32120	0.171470627	16996	1.519199E-02

Spearman Rank correlation analysis performed between Prdm1 and all-expressed genes within the Meredith RNA-seq dataset. Robust Prdm1–associated genes were identified using a cut-off of $p < 0.0005$.

Table S1, Related to Supplemental Figure 3C. Prdm1 associated genes

Stap1	32121	-0.00764107	4833	9.144833E-01
Stap2	32122	0.110826511	12780	1.182135E-01
Star	32123	0.296190126	19889	2.058475E-05
Stard10	32124	0.081761826	10729	2.497457E-01
Stard13	32125	-0.096452939	305	1.742510E-01
Stard3	32126	0.144540904	15335	4.114754E-02
Stard3nl	32127	0.046311353	8036	5.149266E-01
Stard4	32128	0.029718362	7047	6.761379E-01
Stard5	32129	-0.125227318	76	7.725455E-02
Stard6	32130	-0.013805826	4575	8.461529E-01
Stard7	32131	0.00752446	5657	9.157835E-01
Stard8	32132	-0.011480672	4688	8.718187E-01
Stard9	32133	0.130648583	14332	6.518600E-02
Stat1	32134	0.138095995	14832	5.116606E-02
Stat2	32135	-0.062507935	1371	3.792305E-01
Stat3	32136	0.117994023	13526	9.610321E-02
Stat4	32137	0.245361687	19445	4.620001E-04
Stat5a	32138	0.074519868	10179	2.943055E-01
Stat5b	32139	0.056591565	9014	4.260627E-01
Stat6	32140	-0.041025177	2451	5.640814E-01
Stau1	32141	0.127111413	14124	7.287036E-02
Stau2	32142	0.204645813	18412	3.651281E-03
Stbd1	32143	-0.054451513	1703	4.437940E-01
Stc1	32144	0.017549891	6204	8.051743E-01
Stc2	32145	0.023540464	6721	7.407415E-01
Steap1	32146		#N/A	1.000000E+00
Steap2	32147	0.106959382	12494	1.316875E-01
Steap3	32148	0.096985606	11836.5	1.718733E-01
Steap4	32149	-0.046700734	2269	5.113952E-01
Stfa1	32150	0.160304018	16323	2.335876E-02
Stfa2	32151	-0.038406171	3065	5.892384E-01
Stfa2l1	32152	-0.086746694	532	2.219355E-01
Stfa3	32153	0.015913971	6118	8.230211E-01
Stil	32154	0.115499768	13357	1.033913E-01
Stim1	32155	0.166342202	16737	1.856694E-02
Stim2	32156	0.356252129	20009	2.249203E-07
Stip1	32157	0.309892042	19939	7.988641E-06
Stk10	32158	0.170496981	16956	1.578807E-02
Stk11	32159	0.016773019	6162	8.136375E-01
Stk11ip	32160	0.156466362	16078	2.692909E-02
Stk16	32161	0.13902312	14889	4.961077E-02
Stk17b	32162	0.133365315	14501	5.974549E-02
Stk19	32163	0.149839988	15642	3.419660E-02

Spearman Rank correlation analysis performed between Prdm1 and all-expressed genes within the Meredith RNA-seq dataset. Robust Prdm1–associated genes were identified using a cut-off of $p < 0.0005$.

Table S1, Related to Supplemental Figure 3C. Prdm1 associated genes

Stk19-ps1	32164		#N/A	1.000000E+00
Stk24	32165	0.151582396	15746	3.214051E-02
Stk25	32166	0.062930265	9388	3.760122E-01
Stk3	32167	0.136659581	14740	5.365556E-02
Stk30	32168	-0.00329262	5016	9.630927E-01
Stk31	32169	0.045056653	7941.5	5.263908E-01
Stk32a	32170	0.231456677	19196	9.749068E-04
Stk32b	32171	-0.042176176	2433	5.531902E-01
Stk32c	32172	0.140673734	14998	4.693922E-02
Stk33	32173	-0.012880275	4629	8.563516E-01
Stk35	32174	0.15252188	15792	3.107619E-02
Stk36	32175	0.113548679	13094	1.093908E-01
Stk38	32176	0.131781651	14392	6.286955E-02
Stk38l	32177	0.169904435	16924	1.616075E-02
Stk39	32178	-0.007138264	4849	9.200912E-01
Stk4	32179	0.211412302	18629	2.654739E-03
Stk40	32180	0.233202781	19231	8.897398E-04
Stmn1	32181	0.125233075	13985	7.724084E-02
Stmn2	32182	-0.077772753	664	2.736754E-01
Stmn3	32183	-0.086748028	498.5	2.219283E-01
Stmn4	32184	-0.038406171	3065	5.892384E-01
Stmnd1	32185	-0.047267781	2256	5.062750E-01
Stom	32186	0.087284938	11148	2.190698E-01
Stoml1	32187	0.008703397	5713	9.026492E-01
Stoml2	32188	0.16300542	16545	2.109712E-02
Stoml3	32189	-0.002295611	5059.5	9.742636E-01
Ston1	32190	-0.054450825	1984	4.437997E-01
Ston2	32191	0.157039901	16109	2.636746E-02
Stox1	32192	0.163913281	16588	2.038080E-02
Stox2	32193	0.093955949	11640	1.857228E-01
Stpg1	32194	0.014301797	6015.5	8.406982E-01
Stpg2	32195	-0.00964749	4761	8.921501E-01
Stra13	32196	0.201890427	18322	4.145847E-03
Stra6	32197	0.148570697	15565	3.576371E-02
Stra8	32198	0.065148313	9542	3.593861E-01
Strada	32199	0.225653029	19064	1.314694E-03
Stradb	32200	0.230471558	19176	1.026201E-03
Strap	32201	0.163340681	16562	2.083009E-02
Strbp	32202	0.239356454	19353	6.412244E-04
Strc	32203	0.078822826	10520	2.672305E-01
Strip1	32204	0.04719818	8095	5.069020E-01
Strip2	32205	0.078881517	10524	2.668733E-01
Strn	32206	-0.021766895	4180	7.596525E-01

Spearman Rank correlation analysis performed between Prdm1 and all-expressed genes within the Meredith RNA-seq dataset. Robust Prdm1–associated genes were identified using a cut-off of $p < 0.0005$.

Table S1, Related to Supplemental Figure 3C. Prdm1 associated genes

Strn3	32207	0.018557249	6254	7.942339E-01
Strn4	32208	0.195158371	18052	5.617074E-03
Stt3a	32209	0.087244372	11146	2.192849E-01
Stt3b	32210	0.162356866	16489	2.162207E-02
Stub1	32211	0.091212209	11456	1.989568E-01
Stx11	32212	-0.021816528	4178	7.591213E-01
Stx12	32213	0.173377669	17096	1.408123E-02
Stx16	32214	0.217124897	18821	2.013119E-03
Stx17	32215	0.028234904	6982	6.914573E-01
Stx18	32216	-0.074913231	886	2.917572E-01
Stx19	32217	0.018123933	6228	7.989351E-01
Stx1a	32218	0.106381914	12451	1.337971E-01
Stx1b	32219	-0.026518762	4046	7.093369E-01
Stx2	32220	-0.012832646	4632	8.568771E-01
Stx3	32221	0.146857867	15472	3.797444E-02
Stx4a	32222	-0.059384639	1409	4.035494E-01
Stx5a	32223	0.094084091	11648	1.851210E-01
Stx6	32224	0.052904179	8588	4.568706E-01
Stx7	32225	0.066572056	9628	3.489595E-01
Stx8	32226	-0.001039426	5181	9.883453E-01
Stxbp1	32227	-0.034548946	3725	6.271952E-01
Stxbp2	32228	0.130633542	14331	6.521721E-02
Stxbp3a	32229	0.155824137	16029	2.757010E-02
Stxbp3b	32230	0.053805701	8820	4.492258E-01
Stxbp4	32231	-0.011947905	4672	8.666495E-01
Stxbp5	32232	0.006204886	5605	9.305116E-01
Stxbp5l	32233	0.087065282	11131	2.202361E-01
Stxbp6	32234	0.052083528	8476	4.638923E-01
Styk1	32235	0.0491587	8228	4.893962E-01
Styx	32236	-0.020103581	4235	7.775187E-01
Styxl1	32237	-0.038406171	3065	5.892384E-01
Sub1	32238	0.196103839	18088	5.385582E-03
Sucla2	32239	0.048924493	8215	4.914703E-01
Suclg1	32240	0.074108174	10143	2.969884E-01
Suclg2	32241	0.073419219	10088	3.015143E-01
Sucnr1	32242	0.019630193	6310.5	7.826254E-01
Suco	32243	0.133464686	14505	5.955380E-02
Suds3	32244	0.115293252	13345	1.040138E-01
Sufu	32245	0.032993707	7238	6.427888E-01
Sugp1	32246	0.072105437	10004	3.102705E-01
Sugp2	32247	0.07046537	9870	3.214329E-01
Sugt1	32248	0.071426501	9944	3.148602E-01
Sulf1	32249	-0.027383413	4022	7.003080E-01

Spearman Rank correlation analysis performed between Prdm1 and all-expressed genes within the Meredith RNA-seq dataset. Robust Prdm1–associated genes were identified using a cut-off of $p < 0.0005$.

Table S1, Related to Supplemental Figure 3C. Prdm1 associated genes

Sulf2	32250	0.012643021	5909	8.589698E-01
Sult1a1	32251	0.042566467	7778	5.495205E-01
Sult1b1	32252	0.132183524	14424	6.206436E-02
Sult1c1	32253	-0.077393023	832.5	2.760318E-01
Sult1c2	32254	-0.089541137	422	2.073449E-01
Sult1d1	32255	0.096627779	11816	1.734679E-01
Sult1e1	32256		#N/A	1.000000E+00
Sult2a1	32257	0.113548679	13094	1.093908E-01
Sult2a2	32258	0.086412184	11087.5	2.237298E-01
Sult2a3	32259	0.000307056	5327	9.965570E-01
Sult2a4	32260	-0.038406171	3065	5.892384E-01
Sult2a5	32261	-0.016731569	4428.5	8.140896E-01
Sult2a6	32262	0.143605682	15224	4.249002E-02
Sult2a7	32263	0.074180476	10151	2.965160E-01
Sult2b1	32264	-0.023016602	4146	7.463117E-01
Sult3a1	32265	-0.054450825	1984	4.437997E-01
Sult4a1	32266	-0.043103239	2406	5.444930E-01
Sult5a1	32267	-0.04601882	2290	5.175879E-01
Sult6b1	32268		#N/A	1.000000E+00
Sumf1	32269	0.109123689	12633	1.240080E-01
Sumf2	32270	0.063431092	9426.5	3.722175E-01
Sumo1	32271	0.124811469	13956	7.825039E-02
Sumo2	32272	0.161568846	16452	2.227507E-02
Sumo3	32273	0.174561429	17148	1.342810E-02
Sun1	32274	0.231370792	19194	9.792831E-04
Sun2	32275	0.191247264	17887	6.672186E-03
Sun3	32276	0.160987082	16412.5	2.276801E-02
Sun5	32277		#N/A	1.000000E+00
Suox	32278	-0.063818592	1345	3.692977E-01
Supt16	32279	0.06411174	9471	3.670982E-01
Supt20	32280	0.139411905	14915	4.897039E-02
Supt20-ps	32281		#N/A	1.000000E+00
Supt3	32282	0.030433783	7092	6.687967E-01
Supt4a	32283	0.112114673	12860	1.139718E-01
Supt5	32284	0.122772315	13830	8.328464E-02
Supt6	32285	0.146466788	15444	3.849507E-02
Supt7l	32286	-0.053576955	2128	4.511587E-01
Supv3l1	32287	0.012238041	5888	8.634425E-01
Surf1	32288	-0.051984244	2163	4.647458E-01
Surf2	32289	0.088577642	11240	2.122954E-01
Surf4	32290	0.158552369	16205	2.493436E-02
Surf6	32291	0.250214041	19509	3.523868E-04
Susd1	32292	0.012058408	5878	8.654278E-01

Spearman Rank correlation analysis performed between Prdm1 and all-expressed genes within the Meredith RNA-seq dataset. Robust Prdm1–associated genes were identified using a cut-off of $p < 0.0005$.

Table S1, Related to Supplemental Figure 3C. Prdm1 associated genes

Susd2	32293	0.073262529	10084	3.025500E-01
Susd3	32294	0.001350293	5379	9.848600E-01
Susd4	32295	0.028933048	7012	6.842316E-01
Susd5	32296	-0.038406171	3065	5.892384E-01
Suv39h1	32297	-0.036169564	3681	6.111202E-01
Suv39h2	32298	0.03027559	7075	6.704173E-01
Suv420h1	32299	0.113104275	12934	1.107947E-01
Suv420h2	32300	0.072569693	10033	3.071575E-01
Suz12	32301	0.175656829	17198	1.284755E-02
Sv2a	32302	0.126956914	14118	7.322209E-02
Sv2b	32303	0.101911768	12176	1.510144E-01
Sv2c	32304	0.138939067	14880	4.975013E-02
Sva	32305		#N/A	1.000000E+00
Sval1	32306	-0.09526621	368.5	1.796359E-01
Sval2	32307		#N/A	1.000000E+00
Sval3	32308		#N/A	1.000000E+00
Svep1	32309	-0.033099177	3780	6.417263E-01
Svil	32310	0.11292956	12919	1.113506E-01
Svip	32311	0.098761238	11977	1.641211E-01
Svop	32312	-0.103167205	227	1.460180E-01
Svopl	32313	0.021569003	6558	7.617717E-01
Svs1	32314		#N/A	1.000000E+00
Svs2	32315		#N/A	1.000000E+00
Svs3a	32316	-0.066855996	1239.5	3.469032E-01
Svs3b	32317	-0.066855996	1239.5	3.469032E-01
Svs4	32318	-0.054450825	1984	4.437997E-01
Svs5	32319		#N/A	1.000000E+00
Svs6	32320		#N/A	1.000000E+00
Swap70	32321	0.10751526	12523	1.296811E-01
Swi5	32322	0.160596836	16371	2.310392E-02
Swsap1	32323	0.138669728	14864	5.019890E-02
Swt1	32324	0.141503804	15052	4.564180E-02
Syap1	32325	0.142519709	15115	4.409485E-02
Sybu	32326	0.097616408	11878	1.690887E-01
Syce1	32327	-0.003703707	4996.5	9.584887E-01
Syce1l	32328	-0.111789593	146	1.150309E-01
Syce2	32329	0.156671181	16090	2.672736E-02
Syce3	32330		#N/A	1.000000E+00
Sycn	32331	-0.110562757	179.5	1.190970E-01
Sycp1	32332	0.082482218	10787	2.455838E-01
Sycp2	32333	0.150104196	15657	3.387783E-02
Sycp2l	32334	0.179998737	17401	1.075757E-02
Sycp3	32335	0.191018822	17879	6.738953E-03

Spearman Rank correlation analysis performed between Prdm1 and all-expressed genes within the Meredith RNA-seq dataset. Robust Prdm1–associated genes were identified using a cut-off of $p < 0.0005$.

Table S1, Related to Supplemental Figure 3C. Prdm1 associated genes

Syde1	32336	-0.086746249	548	2.219378E-01
Syde2	32337	0.040903973	7689	5.652342E-01
Syf2	32338	0.007058319	5629	9.209832E-01
Syk	32339	0.098244767	11935	1.663485E-01
Sympk	32340	-0.100614056	272	1.563138E-01
Syn1	32341	0.053028039	8595	4.558160E-01
Syn2	32342	0.026742294	6914	7.069988E-01
Syn3	32343	0.0848625	10978	2.321765E-01
Syna	32344	-0.018103565	4364	7.991563E-01
Synb	32345		#N/A	1.000000E+00
Sync	32346	0.014664182	6051	8.367175E-01
Syncrip	32347	0.255435944	19571	2.617162E-04
Syndig1	32348	0.047776796	8126	5.017017E-01
Syndig1l	32349	0.254267429	19556	2.798824E-04
Syne1	32350	-0.026172773	4060	7.129613E-01
Syne2	32351	0.232311012	19215	9.323417E-04
Syne3	32352	-0.000377196	5267	9.957705E-01
Syne4	32353	0.094407311	11668	1.836091E-01
Syngap1	32354		#N/A	1.000000E+00
Syngr1	32355	-0.010048733	4742	8.876935E-01
Syngr2	32356	0.135130128	14631	5.641579E-02
Syngr3	32357	0.086412184	11087.5	2.237298E-01
Syngr4	32358	-0.038406171	3065	5.892384E-01
Synj1	32359	0.058049517	9092	4.142215E-01
Synj2	32360	-0.063078309	1360	3.748880E-01
Synj2bp	32361	0.359371776	20010	1.731479E-07
Synm	32362	-0.103539051	217	1.445625E-01
Synpo	32363	0.049610996	8263	4.854039E-01
Synpo2	32364	0.337422912	19993	1.028910E-06
Synpo2l	32365	0.133567615	14515	5.935578E-02
Synpr	32366	0.040873746	7687	5.655219E-01
Synrg	32367	0.080713926	10647	2.558866E-01
Syp	32368	-0.011471895	4689	8.719158E-01
Sypl	32369	0.096288072	11793	1.749919E-01
Sypl2	32370	0.097999559	11915	1.674138E-01
Sys1	32371	-0.010354435	4731	8.843004E-01
Syt1	32372	0.001985662	5418	9.777375E-01
Syt10	32373	0.061044227	9263	3.905139E-01
Syt11	32374	0.220636761	18919	1.692373E-03
Syt12	32375	0.226057126	19072	1.287913E-03
Syt13	32376	0.02887643	7011	6.848166E-01
Syt14	32377	-0.097361427	298	1.702102E-01
Syt15	32378	-0.002309098	5057	9.741124E-01

Spearman Rank correlation analysis performed between Prdm1 and all-expressed genes within the Meredith RNA-seq dataset. Robust Prdm1–associated genes were identified using a cut-off of $p < 0.0005$.

Syt16	32379	-0.045725917	2305	5.202596E-01
Syt17	32380	0.13543866	14658	5.584973E-02
Syt2	32381	0.073881432	10117	2.984729E-01
Syt3	32382	0.098529518	11958	1.651177E-01
Syt4	32383	0.092260427	11534.5	1.938224E-01
Syt5	32384		#N/A	1.000000E+00
Syt6	32385	0.152272071	15784	3.135622E-02
Syt7	32386	0.25001006	19508	3.564602E-04
Syt8	32387	0.153482631	15865	3.001897E-02
Syt9	32388	0.206723043	18485	3.314343E-03
Sytl1	32389	0.148820212	15582	3.545094E-02
Sytl2	32390	-0.054451513	1703	4.437940E-01
Sytl3	32391	0.157799267	16153.5	2.563935E-02
Sytl4	32392	0.062682404	9371	3.778989E-01
Sytl5	32393	0.053267784	8685	4.537786E-01
Syvn1	32394	0.202654311	18345	4.003023E-03
Szrd1	32395	0.176339105	17240	1.249720E-02
Szt2	32396	0.016551519	6146	8.160545E-01
T	32397	0.022198129	6607.5	7.550408E-01
T2	32398	0.019806784	6322	7.807193E-01
Taar1	32399		#N/A	1.000000E+00
Taar2	32400		#N/A	1.000000E+00
Taar3	32401		#N/A	1.000000E+00
Taar4	32402		#N/A	1.000000E+00
Taar5	32403		#N/A	1.000000E+00
Taar6	32404		#N/A	1.000000E+00
Taar7a	32405		#N/A	1.000000E+00
Taar7b	32406		#N/A	1.000000E+00
Taar7c-ps	32407		#N/A	1.000000E+00
Taar7d	32408		#N/A	1.000000E+00
Taar7e	32409		#N/A	1.000000E+00
Taar7f	32410		#N/A	1.000000E+00
Taar8a	32411		#N/A	1.000000E+00
Taar8b	32412		#N/A	1.000000E+00
Taar8c	32413		#N/A	1.000000E+00
Taar9	32414		#N/A	1.000000E+00
Tab1	32415	0.121538974	13755	8.645376E-02
Tab2	32416	-0.0192937	4260	7.862610E-01
Tab3	32417	0.076790871	10399	2.797965E-01
Tac1	32418	0.011038465	5826	8.767159E-01
Tac2	32419	-0.005034268	4932	9.435971E-01
Tac4	32420	0.094341695	11662	1.839153E-01
Tacc1	32421	0.118453394	13554	9.480655E-02

Spearman Rank correlation analysis performed between Prdm1 and all-expressed genes within the Meredith RNA-seq dataset. Robust Prdm1–associated genes were identified using a cut-off of $p < 0.0005$.

Table S1, Related to Supplemental Figure 3C. Prdm1 associated genes

Tacc2	32422	0.051235834	8405	4.712075E-01
Tacc3	32423	0.171636784	17002	1.509226E-02
Taco1	32424	0.284916815	19833	4.329494E-05
Tacr1	32425	0.12652711	14077	7.420784E-02
Tacr2	32426	-0.001606767	5097.5	9.819847E-01
Tacr3	32427	0.207242881	18502	3.234544E-03
Tacstd2	32428	0.10851636	12584	1.261271E-01
Tada1	32429	0.182918545	17548	9.526588E-03
Tada2a	32430	-0.015798723	4482	8.242819E-01
Tada2b	32431	0.133748556	14533	5.900899E-02
Tada3	32432	0.080829255	10651	2.552057E-01
Taf1	32433	0.236968517	19305	7.288629E-04
Taf10	32434	0.175712846	17202	1.281847E-02
Taf11	32435	0.105746424	12403	1.361485E-01
Taf12	32436	0.212863693	18688	2.476176E-03
Taf13	32437	0.189985185	17840	7.048564E-03
Taf15	32438	0.27827619	19782	6.611583E-05
Taf1a	32439	0.19396331	17990	5.922403E-03
Taf1b	32440	0.062229406	9338	3.813621E-01
Taf1c	32441	0.098684735	11971	1.644496E-01
Taf1d	32442	0.133198229	14491	6.006894E-02
Taf2	32443	-0.08821412	433	2.141851E-01
Taf3	32444	0.07234554	10014	3.086579E-01
Taf4a	32445	0.140635096	14996	4.700036E-02
Taf4b	32446	0.051539674	8430	4.685782E-01
Taf5	32447	0.118813078	13573	9.380101E-02
Taf5l	32448	0.056302092	9000	4.284368E-01
Taf6	32449	0.150190495	15662	3.377426E-02
Taf6l	32450	0.071197501	9928	3.164182E-01
Taf7	32451	0.014315993	6028	8.405422E-01
Taf7l	32452	0.001442702	5387.5	9.838240E-01
Taf8	32453	0.209535377	18564	2.902952E-03
Taf9	32454	0.094693048	11686	1.822802E-01
Taf9b	32455	0.145662916	15390	3.958414E-02
Tagap	32456	0.061756086	9319	3.850013E-01
Tagap1	32457	0.128978211	14236	6.872787E-02
Tagln	32458	0.044262848	7882	5.337100E-01
Tagln2	32459	0.246489694	19463	4.340162E-04
Tagln3	32460	-0.000841282	5208.5	9.905669E-01
Tal1	32461	-0.038406171	3065	5.892384E-01
Tal2	32462	-0.066855996	1239.5	3.469032E-01
Taldo1	32463	0.160954398	16388	2.279598E-02
Tamm41	32464	0.032182262	7191	6.509878E-01

Spearman Rank correlation analysis performed between Prdm1 and all-expressed genes within the Meredith RNA-seq dataset. Robust Prdm1–associated genes were identified using a cut-off of $p < 0.0005$.

Table S1, Related to Supplemental Figure 3C. Prdm1 associated genes

Tanc1	32465	0.01111231	5832	8.758978E-01
Tanc2	32466	-0.0437857	2377	5.381339E-01
Tango2	32467	0.005831581	5587	9.346827E-01
Tango6	32468	0.070775984	9893	3.192991E-01
Tank	32469	0.11219484	12866	1.137118E-01
Taok1	32470	0.163949854	16590	2.035239E-02
Taok2	32471	0.067288592	9659	3.437850E-01
Taok3	32472	0.184278558	17604	8.997036E-03
Tap1	32473	0.187930587	17760	7.701868E-03
Tap2	32474	0.141321339	15033	4.592440E-02
Tapbp	32475	0.130898152	14349	6.466987E-02
Tapbpl	32476	0.098496793	11954	1.652588E-01
Tapt1	32477	0.180097136	17407	1.071390E-02
Tarbp2	32478	0.157530319	16129	2.589523E-02
Tardbp	32479	0.174259464	17136	1.359213E-02
Tarm1	32480	0.143605682	15224	4.249002E-02
Tars	32481	0.110578307	12764	1.190448E-01
Tars2	32482	0.061426914	9296	3.875445E-01
Tarsl2	32483	0.002119637	5421	9.762359E-01
Tas1r1	32484	0.053267784	8685	4.537786E-01
Tas1r2	32485		#N/A	1.000000E+00
Tas1r3	32486	-0.038406171	3065	5.892384E-01
Tas2r102	32487		#N/A	1.000000E+00
Tas2r103	32488		#N/A	1.000000E+00
Tas2r104	32489		#N/A	1.000000E+00
Tas2r105	32490		#N/A	1.000000E+00
Tas2r106	32491		#N/A	1.000000E+00
Tas2r107	32492		#N/A	1.000000E+00
Tas2r108	32493		#N/A	1.000000E+00
Tas2r109	32494	-0.054450825	1984	4.437997E-01
Tas2r110	32495		#N/A	1.000000E+00
Tas2r113	32496		#N/A	1.000000E+00
Tas2r114	32497		#N/A	1.000000E+00
Tas2r115	32498		#N/A	1.000000E+00
Tas2r116	32499		#N/A	1.000000E+00
Tas2r117	32500		#N/A	1.000000E+00
Tas2r118	32501		#N/A	1.000000E+00
Tas2r119	32502		#N/A	1.000000E+00
Tas2r120	32503		#N/A	1.000000E+00
Tas2r121	32504		#N/A	1.000000E+00
Tas2r122	32505		#N/A	1.000000E+00
Tas2r123	32506		#N/A	1.000000E+00
Tas2r124	32507		#N/A	1.000000E+00

Spearman Rank correlation analysis performed between Prdm1 and all-expressed genes within the Meredith RNA-seq dataset. Robust Prdm1–associated genes were identified using a cut-off of $p < 0.0005$.

Table S1, Related to Supplemental Figure 3C. Prdm1 associated genes

Tas2r125	32508		#N/A	1.000000E+00
Tas2r126	32509		#N/A	1.000000E+00
Tas2r129	32510		#N/A	1.000000E+00
Tas2r130	32511		#N/A	1.000000E+00
Tas2r131	32512		#N/A	1.000000E+00
Tas2r134	32513		#N/A	1.000000E+00
Tas2r135	32514		#N/A	1.000000E+00
Tas2r136	32515		#N/A	1.000000E+00
Tas2r137	32516		#N/A	1.000000E+00
Tas2r138	32517		#N/A	1.000000E+00
Tas2r139	32518		#N/A	1.000000E+00
Tas2r140	32519		#N/A	1.000000E+00
Tas2r143	32520		#N/A	1.000000E+00
Tas2r144	32521		#N/A	1.000000E+00
Tasp1	32522	0.181013779	17449	1.031458E-02
Tat	32523		#N/A	1.000000E+00
Tatdn1	32524	0.086231644	11065	2.247024E-01
Tatdn2	32525	0.133490859	14506	5.950339E-02
Tatdn3	32526	0.068813407	9762	3.329360E-01
Tax1bp1	32527	0.129028752	14239	6.861845E-02
Tax1bp3	32528	0.204610873	18411	3.657203E-03
Taz	32529	0.026061517	6875	7.141281E-01
Tbata	32530	0.033177155	7250	6.409412E-01
Tbc1d1	32531	0.093602285	11621	1.873915E-01
Tbc1d10a	32532	0.051412257	8421	4.696798E-01
Tbc1d10b	32533	0.133221194	14495	6.002440E-02
Tbc1d10c	32534	0.196450687	18102	5.302826E-03
TBC1D10C	32535		#N/A	1.000000E+00
Tbc1d12	32536	0.060853188	9253	3.920014E-01
Tbc1d13	32537	0.204881401	18420	3.611571E-03
Tbc1d14	32538	-0.031732522	3855	6.555502E-01
Tbc1d15	32539	0.049469946	8252	4.866471E-01
Tbc1d16	32540	0.11679572	13454	9.955199E-02
Tbc1d17	32541	-0.09196828	403	1.952436E-01
Tbc1d19	32542	0.179620129	17388	1.092705E-02
Tbc1d2	32543	-0.001224019	5151	9.862757E-01
Tbc1d20	32544	0.091170702	11453	1.991621E-01
Tbc1d21	32545		#N/A	1.000000E+00
Tbc1d22a	32546	0.11685592	13456	9.937642E-02
Tbc1d22b	32547		#N/A	1.000000E+00
Tbc1d23	32548	0.171399988	16990	1.523457E-02
Tbc1d24	32549	0.149664874	15627	3.440928E-02
Tbc1d25	32550	-0.080465687	636	2.573564E-01

Spearman Rank correlation analysis performed between Prdm1 and all-expressed genes within the Meredith RNA-seq dataset. Robust Prdm1–associated genes were identified using a cut-off of $p < 0.0005$.

Table S1, Related to Supplemental Figure 3C. Prdm1 associated genes

Tbc1d2b	32551	0.25221177	19527	3.147146E-04
Tbc1d30	32552	0.117483405	13491	9.756101E-02
Tbc1d4	32553	0.126847748	14100	7.347145E-02
Tbc1d5	32554	0.006010064	5598	9.326882E-01
Tbc1d7	32555	0.199124781	18207	4.702122E-03
Tbc1d8	32556	0.105942878	12417	1.354182E-01
Tbc1d8b	32557	0.005915365	5591	9.337464E-01
Tbc1d9	32558	0.021400909	6547	7.635731E-01
Tbc1d9b	32559	0.095828081	11756	1.770713E-01
Tbca	32560	0.13577933	14678	5.523016E-02
Tbcb	32561	0.197213041	18130	5.124925E-03
Tbcc	32562	0.054438237	8890	4.439052E-01
Tbccd1	32563	-0.039343123	2487	5.801795E-01
Tbcd	32564	0.06385548	9461	3.690205E-01
Tbce	32565	0.060694018	9245	3.932433E-01
Tbcel	32566	-0.01313818	4612	8.535073E-01
Tbck	32567	0.129311466	14261	6.800897E-02
Tbk1	32568	0.159950024	16277	2.367007E-02
Tbkbp1	32569	-0.018790377	4285	7.917077E-01
Tbl1x	32570	0.225124088	19047	1.350520E-03
Tbl1xr1	32571	0.103086117	12246	1.463369E-01
Tbl2	32572	0.225581885	19063	1.319461E-03
Tbl3	32573	0.226059511	19073	1.287756E-03
Tbp	32574	0.018292461	6238	7.971059E-01
Tbpl1	32575	0.152762565	15808	3.080841E-02
Tbpl2	32576	-0.038406171	3065	5.892384E-01
Tbr1	32577	-0.054451513	1703	4.437940E-01
Tbrg1	32578	0.146090272	15421	3.900198E-02
Tbrg4	32579	0.312351751	19944	6.706155E-06
Tbx1	32580	-0.054451513	1703	4.437940E-01
Tbx10	32581		#N/A	1.000000E+00
Tbx15	32582	-0.019119691	4268	7.881429E-01
Tbx18	32583	0.046016196	8006	5.176118E-01
Tbx19	32584	-0.077394997	736.5	2.760195E-01
Tbx2	32585	-0.032450477	3820	6.482730E-01
Tbx20	32586	-0.036196075	3679	6.108587E-01
Tbx21	32587	-0.025521893	4086	7.197970E-01
Tbx22	32588	0.045056653	7941.5	5.263908E-01
Tbx3	32589	0.14240018	15105	4.427455E-02
Tbx4	32590	-0.005759226	4891	9.354913E-01
Tbx5	32591	0.120630006	13700	8.885068E-02
Tbx6	32592	0.201877203	18321	4.148360E-03
Tbxa2r	32593	-0.038406171	3065	5.892384E-01

Spearman Rank correlation analysis performed between Prdm1 and all-expressed genes within the Meredith RNA-seq dataset. Robust Prdm1–associated genes were identified using a cut-off of $p < 0.0005$.

Table S1, Related to Supplemental Figure 3C. Prdm1 associated genes

Tbxas1	32594	0.1584832	16201	2.499841E-02
Tc2n	32595	0.067518529	9676	3.421348E-01
Tcaim	32596	0.056640792	9017	4.256597E-01
Tcam1	32597	0.1033749	12264	1.452037E-01
Tcap	32598		#N/A	1.000000E+00
Tcea1	32599	0.10918405	12636	1.237990E-01
Tcea1-ps1	32600		#N/A	1.000000E+00
Tcea2	32601	0.152059748	15774	3.159592E-02
Tcea3	32602	0.035947558	7380	6.133116E-01
Tceal1	32603	0.081762057	10730.5	2.497444E-01
Tceal3	32604	0.062129491	9333.5	3.821286E-01
Tceal5	32605		#N/A	1.000000E+00
Tceal6	32606		#N/A	1.000000E+00
Tceal7	32607	0.053805701	8820	4.492258E-01
Tceal8	32608	-0.063120175	1359	3.745705E-01
Tceanc	32609	0.164917123	16652	1.961335E-02
Tceanc2	32610	0.335794705	19992	1.168078E-06
Tceb1	32611	0.090531441	11380	2.023438E-01
Tceb2	32612	0.212327601	18667	2.540820E-03
Tceb3	32613	0.17198524	17019	1.488496E-02
Tcerg1	32614	0.096740157	11820	1.729659E-01
Tcerg1l	32615	-0.025073661	4098	7.245174E-01
Tcf12	32616	0.174115788	17129	1.367079E-02
Tcf15	32617		#N/A	1.000000E+00
Tcf19	32618	0.084891402	10981	2.320170E-01
Tcf20	32619	0.217891965	18843	1.938674E-03
Tcf21	32620		#N/A	1.000000E+00
Tcf23	32621	-0.10317255	224	1.459970E-01
Tcf25	32622	0.20640969	18472	3.363301E-03
Tcf3	32623	0.206193441	18463	3.397467E-03
Tcf4	32624	0.17231051	17038	1.469370E-02
Tcf7	32625	0.15030834	15668	3.363326E-02
Tcf7l1	32626	0.260364713	19628	1.965120E-04
Tcf7l2	32627	0.066283641	9607	3.510562E-01
Tcfl5	32628	0.009622637	5754	8.924262E-01
Tchh	32629	-0.004180162	4980	9.531543E-01
Tchhl1	32630		#N/A	1.000000E+00
Tchp	32631	0.167894259	16815	1.748277E-02
Tcirg1	32632	0.140152255	14967	4.776995E-02
Tcl1	32633		#N/A	1.000000E+00
Tcl1b1	32634		#N/A	1.000000E+00
Tcl1b2	32635	0.053805701	8820	4.492258E-01
Tcl1b3	32636		#N/A	1.000000E+00

Spearman Rank correlation analysis performed between Prdm1 and all-expressed genes within the Meredith RNA-seq dataset. Robust Prdm1–associated genes were identified using a cut-off of $p < 0.0005$.

Table S1, Related to Supplemental Figure 3C. Prdm1 associated genes

Tcl1b4	32637		#N/A	1.000000E+00
Tcl1b5	32638		#N/A	1.000000E+00
Tcn2	32639	0.016954004	6168	8.116639E-01
Tcof1	32640	0.162471737	16496	2.152828E-02
Tcp1	32641	0.151981694	15766	3.168442E-02
Tcp1-ps1	32642	0.053267784	8685	4.537786E-01
Tcp10a	32643		#N/A	1.000000E+00
Tcp10b	32644	-0.066856562	1139	3.468991E-01
Tcp10c	32645	0.137540885	14792	5.211652E-02
Tcp11	32646	-0.054451513	1703	4.437940E-01
Tcp11l1	32647	0.173917176	17118	1.378019E-02
Tcp11l2	32648	0.04503989	7937	5.265448E-01
Tcrg-C1	32649		#N/A	1.000000E+00
Tcrg-C2	32650		#N/A	1.000000E+00
Tcrg-C3	32651		#N/A	1.000000E+00
Tcrg-C4	32652	-0.038406171	3065	5.892384E-01
Tcrg-V3	32653		#N/A	1.000000E+00
Tcrg-V4	32654		#N/A	1.000000E+00
Tcrg-V5	32655		#N/A	1.000000E+00
Tcrg-V6	32656		#N/A	1.000000E+00
Tcrg-V7	32657		#N/A	1.000000E+00
Tcstv1	32658		#N/A	1.000000E+00
Tcstv3	32659		#N/A	1.000000E+00
Tcta	32660	0.020561865	6374	7.725840E-01
Tcte1	32661	-0.059855751	1399	3.998229E-01
Tcte2	32662	0.114497838	13291	1.064389E-01
Tcte3	32663		#N/A	1.000000E+00
Tctex1d1	32664	0.112498373	12889	1.127316E-01
Tctex1d2	32665	0.063854062	9460	3.690311E-01
Tctex1d4	32666	0.113548679	13094	1.093908E-01
Tctn1	32667	0.045196005	7953	5.251111E-01
Tctn2	32668	-0.055336718	1509.5	4.364094E-01
Tctn3	32669	0.096772881	11821	1.728199E-01
Tdg	32670	0.090054268	11349	2.047425E-01
Tdgf1	32671		#N/A	1.000000E+00
Tdgf1-ps1	32672		#N/A	1.000000E+00
Tdgf1-ps2	32673		#N/A	1.000000E+00
Tdh	32674	0.088985891	11268	2.101876E-01
Tdo2	32675	0.033934235	7291	6.333394E-01
Tdp1	32676	0.116715038	13450	9.978767E-02
Tdp2	32677	0.213412175	18696	2.411588E-03
Tdpoz1	32678	0.052728522	8550.5	4.583686E-01
Tdpoz2	32679		#N/A	1.000000E+00

Spearman Rank correlation analysis performed between Prdm1 and all-expressed genes within the Meredith RNA-seq dataset. Robust Prdm1–associated genes were identified using a cut-off of $p < 0.0005$.

Table S1, Related to Supplemental Figure 3C. Prdm1 associated genes

Tdpoz3	32680		#N/A	1.000000E+00
Tdpoz4	32681		#N/A	1.000000E+00
Tdpoz5	32682		#N/A	1.000000E+00
Tdrd1	32683	0.021787669	6581	7.594301E-01
Tdrd12	32684	-0.054450825	1984	4.437997E-01
Tdrd3	32685	0.206913972	18489	3.284830E-03
Tdrd5	32686	0.022198316	6629.5	7.550388E-01
Tdrd6	32687		#N/A	1.000000E+00
Tdrd7	32688	0.124940146	13965	7.794114E-02
Tdrd9	32689	0.109271317	12642	1.234971E-01
Tdrkh	32690	-0.077393517	797	2.760287E-01
Tead1	32691	0.176175496	17232	1.258044E-02
Tead2	32692	0.070992254	9910	3.178189E-01
Tead3	32693	0.03463874	7317	6.262998E-01
Tead4	32694	0.160304018	16323	2.335876E-02
Tec	32695	0.10939609	12647	1.230666E-01
Tecpr1	32696	0.064832706	9527	3.617235E-01
Tecpr2	32697	0.063122932	9397	3.745496E-01
Tecr	32698	0.071640886	9959	3.134062E-01
Tecrl	32699	0.014301797	6015.5	8.406982E-01
Tecta	32700		#N/A	1.000000E+00
Tectb	32701	-0.054451513	1703	4.437940E-01
Teddm1	32702		#N/A	1.000000E+00
Tef	32703	0.081222836	10682	2.528914E-01
Tefm	32704	0.056135831	8993	4.298039E-01
Tek	32705	0.253503546	19546	2.923847E-04
Tekt1	32706	0.143605682	15224	4.249002E-02
Tekt2	32707	0.040730281	7679	5.668882E-01
Tekt3	32708		#N/A	1.000000E+00
Tekt4	32709		#N/A	1.000000E+00
Tekt5	32710	0.04165608	7721	5.580989E-01
Telo2	32711	0.17616216	17231	1.258724E-02
Ten1	32712	0.018372029	6240	7.962426E-01
Tenc1	32713	-0.066856562	1139	3.468991E-01
Tenm1	32714	0.108931317	12618	1.246763E-01
Tenm2	32715	0.096233286	11790	1.752386E-01
Tenm3	32716	0.12155815	13760	8.640375E-02
Tenm4	32717	0.128730812	14229	6.926557E-02
Tep1	32718	0.165270543	16674	1.934918E-02
Tepp	32719	-0.077393517	797	2.760287E-01
Terc	32720		#N/A	1.000000E+00
Terf1	32721	0.112330421	12881	1.132732E-01
Terf2	32722	0.09117877	11454	1.991222E-01

Spearman Rank correlation analysis performed between Prdm1 and all-expressed genes within the Meredith RNA-seq dataset. Robust Prdm1–associated genes were identified using a cut-off of $p < 0.0005$.

Table S1, Related to Supplemental Figure 3C. Prdm1 associated genes

Terf2ip	32723	0.26635856	19688	1.376401E-04
Tert	32724	-0.038406171	3065	5.892384E-01
Tes	32725	0.118508684	13557	9.465142E-02
Tes3-ps	32726		#N/A	1.000000E+00
Tesc	32727	-0.046701236	2268	5.113906E-01
Tescl	32728		#N/A	1.000000E+00
Tesk1	32729	0.158762881	16213	2.474030E-02
Tesk2	32730	0.116083548	13399	1.016476E-01
Tespa1	32731	0.06064071	9240	3.936598E-01
Tet1	32732	0.178956925	17353	1.122961E-02
Tet2	32733	0.140702682	15001	4.689347E-02
Tet3	32734	0.19384488	17986	5.953455E-03
Tex10	32735	0.17463924	17149	1.338612E-02
Tex101	32736		#N/A	1.000000E+00
Tex11	32737	-0.008200529	4814	9.082485E-01
Tex12	32738		#N/A	1.000000E+00
Tex13	32739		#N/A	1.000000E+00
Tex13a	32740		#N/A	1.000000E+00
Tex14	32741	0.028412074	6989	6.896210E-01
Tex15	32742	0.03037917	7087.5	6.693560E-01
Tex16	32743	0.039560733	7607	5.780849E-01
Tex19.1	32744	0.113548679	13094	1.093908E-01
Tex19.2	32745		#N/A	1.000000E+00
Tex2	32746	0.180076516	17405	1.072304E-02
Tex21	32747	-0.012392859	4651	8.617321E-01
Tex22	32748		#N/A	1.000000E+00
Tex24	32749		#N/A	1.000000E+00
Tex26	32750	0.176404734	17241	1.246394E-02
Tex261	32751	0.133051998	14480	6.035321E-02
Tex264	32752	0.070794059	9895	3.191752E-01
Tex28	32753		#N/A	1.000000E+00
Tex29	32754	-0.038406171	3065	5.892384E-01
Tex30	32755	0.266585959	19693	1.357707E-04
Tex33	32756	-0.038406171	3065	5.892384E-01
Tex35	32757	-0.060369708	1391	3.957811E-01
Tex36	32758	-0.038406171	3065	5.892384E-01
Tex37	32759		#N/A	1.000000E+00
Tex38	32760		#N/A	1.000000E+00
Tex40	32761		#N/A	1.000000E+00
Tex9	32762	0.14622347	15430	3.882201E-02
Tfam	32763	-0.008852258	4792	9.009926E-01
Tfap2a	32764	0.108151147	12560	1.274148E-01
Tfap2b	32765	-0.045749568	2297	5.200436E-01

Spearman Rank correlation analysis performed between Prdm1 and all-expressed genes within the Meredith RNA-seq dataset. Robust Prdm1–associated genes were identified using a cut-off of $p < 0.0005$.

Table S1, Related to Supplemental Figure 3C. Prdm1 associated genes

Tfap2c	32766	0.040562192	7669	5.684911E-01
Tfap2d	32767	0.065429868	9558	3.573090E-01
Tfap2e	32768	0.164478601	16623.5	1.994547E-02
Tfap4	32769	0.018069753	6225	7.995235E-01
Tfb1m	32770	0.129960763	14298	6.662593E-02
Tfb2m	32771	0.083157042	10845	2.417292E-01
Tfcp2	32772	0.26658922	19694	1.357441E-04
Tfcp2l1	32773	0.12957785	14278	6.743875E-02
Tfdp1	32774	0.287516043	19852	3.657612E-05
Tfdp2	32775	0.089857287	11335	2.057387E-01
Tfe3	32776	0.121177318	13739	8.740115E-02
Tfeb	32777	-0.013226251	4606	8.525364E-01
Tfec	32778	0.040730281	7679	5.668882E-01
Tff1	32779	-0.031503567	3863.5	6.578778E-01
Tff2	32780	-0.097562648	297	1.693247E-01
Tff3	32781	0.012925476	5932.5	8.558530E-01
Tfg	32782	0.120517954	13694	8.914981E-02
Tfip11	32783	-0.130880138	58	6.470701E-02
Tfpi	32784	-0.008138736	4816	9.089369E-01
Tfpi2	32785		#N/A	1.000000E+00
Tfpt	32786	0.071353012	9939	3.153596E-01
Tfr2	32787	0.001107773	5366	9.875790E-01
Tfrc	32788	0.03929952	7593	5.805997E-01
Tg	32789	0.174166234	17131	1.364313E-02
Tgds	32790	0.002177936	5426	9.755824E-01
Tgfa	32791	0.056683598	9021	4.253095E-01
Tgfb1	32792	0.122466921	13816	8.406052E-02
Tgfb1i1	32793	-0.086748028	498.5	2.219283E-01
Tgfb2	32794	0.055938417	8979	4.314304E-01
Tgfb3	32795	0.02440169	6784	7.316135E-01
Tgfbi	32796	0.005923798	5592	9.336521E-01
Tgfbr1	32797	0.166804713	16764	1.823791E-02
Tgfbr2	32798	0.117011167	13467	9.892480E-02
Tgfbr3	32799	0.095192395	11710	1.799749E-01
Tgfbrap1	32800	-0.037394813	3650	5.990886E-01
Tgif1	32801	0.090548869	11383	2.022565E-01
Tgif2	32802	0.031789057	7176	6.549760E-01
Tgif2lx1	32803		#N/A	1.000000E+00
Tgif2lx2	32804		#N/A	1.000000E+00
Tgm1	32805	-0.066858259	1029.5	3.468868E-01
Tgm2	32806	0.103869031	12288	1.432802E-01
Tgm3	32807	0.10778938	12540	1.287003E-01
Tgm4	32808	-0.045728124	2303	5.202395E-01

Spearman Rank correlation analysis performed between Prdm1 and all-expressed genes within the Meredith RNA-seq dataset. Robust Prdm1–associated genes were identified using a cut-off of $p < 0.0005$.

Table S1, Related to Supplemental Figure 3C. Prdm1 associated genes

Tgm5	32809	-0.042973211	2417	5.457088E-01
Tgm6	32810	0.091725708	11505	1.964294E-01
Tgm7	32811	-0.038406171	3065	5.892384E-01
Tgoln1	32812	0.261390241	19642	1.850073E-04
Tgs1	32813	0.157049632	16112	2.635802E-02
Tgtp1	32814	0.134885536	14611.5	5.686790E-02
Tgtp2	32815	0.108608001	12596	1.258056E-01
Th	32816	0.076093811	10354	2.841976E-01
Tha1	32817	-0.117572522	121	9.730533E-02
Thada	32818	0.169944872	16925	1.613507E-02
Thap1	32819	-0.004989208	4935	9.441011E-01
Thap11	32820	0.1436233	15284	4.246440E-02
Thap2	32821	-0.065743368	1312	3.550049E-01
Thap3	32822	0.061229027	9274	3.890783E-01
Thap4	32823	-0.017310055	4402	8.077847E-01
Thap7	32824	0.106524225	12462	1.332748E-01
Thap8	32825		#N/A	1.000000E+00
Thbd	32826	-0.020772979	4215	7.703138E-01
Thbs1	32827	0.037431891	7451	5.987261E-01
Thbs2	32828	-0.110564915	172	1.190898E-01
Thbs3	32829	0.051291672	8412	4.707237E-01
Thbs4	32830	-0.000841282	5208.5	9.905669E-01
Theg	32831		#N/A	1.000000E+00
Them4	32832	0.093709829	11629	1.868829E-01
Them5	32833	-0.054451513	1703	4.437940E-01
Them6	32834	0.225062476	19042	1.354751E-03
Themis	32835	-0.024919933	4101	7.261387E-01
Themis2	32836	0.230920425	19187	1.002529E-03
Themis3	32837	0.160987082	16412.5	2.276801E-02
Thg1l	32838	0.049249774	8240	4.885909E-01
Thnsl1	32839	-0.012297868	4661	8.627815E-01
Thnsl2	32840	0.147732522	15520	3.683153E-02
Thoc1	32841	0.207310872	18504	3.224236E-03
Thoc2	32842	0.131156904	14361	6.413828E-02
Thoc3	32843	0.156697333	16093	2.670169E-02
Thoc5	32844	0.086454589	11102	2.235017E-01
Thoc6	32845	0.17211004	17027	1.481132E-02
Thoc7	32846	0.167763528	16811	1.757192E-02
Thop1	32847	0.021317126	6513.5	7.644715E-01
Thpo	32848		#N/A	1.000000E+00
Thra	32849	0.010050054	5773	8.876788E-01
Thrap3	32850	0.141185202	15029	4.613619E-02
Thrb	32851	0.19434943	18014	5.822167E-03

Spearman Rank correlation analysis performed between Prdm1 and all-expressed genes within the Meredith RNA-seq dataset. Robust Prdm1–associated genes were identified using a cut-off of $p < 0.0005$.

Table S1, Related to Supplemental Figure 3C. Prdm1 associated genes

Thrsp	32852	0.032053981	7186	6.522878E-01
Thsd1	32853	0.139045799	14892.5	4.957323E-02
Thsd4	32854	0.022309345	6649	7.538528E-01
Thsd7a	32855	0.02798156	6971	6.940863E-01
Thsd7b	32856	0.098148259	11924	1.667672E-01
Thtpa	32857	0.114522441	13295	1.063632E-01
Thumpd1	32858	0.081945059	10742	2.486825E-01
Thumpd2	32859	-0.046166569	2282	5.162429E-01
Thumpd3	32860	0.167268223	16786	1.791327E-02
Thy1	32861	0.011769095	5866	8.686271E-01
Thyn1	32862	0.135984554	14699	5.485968E-02
Tia1	32863	0.159839568	16266	2.376794E-02
Tial1	32864	0.135866395	14690	5.507273E-02
Tiam1	32865	0.116164197	13406	1.014086E-01
Tiam2	32866	-0.02153883	4189	7.620949E-01
Ticam1	32867	0.063268281	9408	3.734485E-01
Ticam2	32868	-0.01057139	4724	8.818937E-01
Ticrr	32869	0.155702305	16018	2.769316E-02
Tie1	32870	0.00030705	5316.5	9.965570E-01
Tifa	32871	0.025556904	6852	7.194287E-01
Tifab	32872	0.13382888	14540	5.885558E-02
Tigd2	32873	0.028973943	7014	6.838092E-01
Tigd3	32874	0.10244607	12209	1.488724E-01
Tigd4	32875	0.101583206	12154	1.523431E-01
Tigd5	32876	0.038462056	7525	5.886962E-01
Tigit	32877	-0.066858259	1029.5	3.468868E-01
Timd2	32878	0.066564628	9626	3.490134E-01
Timd4	32879	0.073965883	10136	2.979194E-01
Timeless	32880	-0.000996327	5184	9.888285E-01
Timm10	32881	-0.058250544	1429	4.126041E-01
Timm10b	32882	0.056550789	9012	4.263967E-01
Timm13	32883	-0.005625591	4897	9.369850E-01
Timm17a	32884	0.160336327	16354	2.333052E-02
Timm17b	32885	0.236791556	19302	7.357781E-04
Timm21	32886	0.011125709	5834	8.757493E-01
Timm22	32887	0.142317906	15099	4.439860E-02
Timm23	32888	0.143303316	15156	4.293185E-02
Timm44	32889	0.139748509	14935	4.842152E-02
Timm50	32890	0.177197718	17276	1.206824E-02
Timm8a1	32891	0.036420623	7404	6.086462E-01
Timm8a2	32892	-0.056434944	1469.5	4.273463E-01
Timm8b	32893	0.092911487	11576	1.906823E-01
Timm9	32894	-0.04312197	2398	5.443180E-01

Spearman Rank correlation analysis performed between Prdm1 and all-expressed genes within the Meredith RNA-seq dataset. Robust Prdm1–associated genes were identified using a cut-off of $p < 0.0005$.

Table S1, Related to Supplemental Figure 3C. Prdm1 associated genes

Timmdc1	32895	0.21616688	18791	2.109749E-03
Timp1	32896	0.091373289	11478	1.981614E-01
Timp2	32897	0.06568714	9571	3.554175E-01
Timp3	32898	0.154304297	15910	2.913930E-02
Timp4	32899	-0.066856562	1139	3.468991E-01
Tinag	32900	-0.013651674	4582	8.478498E-01
Tinagl1	32901	0.167141782	16778	1.800132E-02
Tinf2	32902	0.136555356	14731	5.384003E-02
Tiparp	32903	0.021609128	6560	7.613418E-01
Tipin	32904	0.164516209	16632	1.991680E-02
Tiprl	32905	0.050218842	8320	4.800664E-01
Tirap	32906	0.264570518	19673	1.532005E-04
Tjap1	32907	0.093879094	11635	1.860845E-01
Tjp1	32908	0.123093492	13847	8.247487E-02
Tjp2	32909	0.12451161	13940	7.897488E-02
Tjp3	32910	0.070285127	9857	3.226753E-01
Tk1	32911	0.125892459	14032	7.568306E-02
Tk2	32912	0.147145032	15485	3.759595E-02
Tkt	32913	-0.004159157	4981	9.533894E-01
Tktl1	32914	0.071415159	9942	3.149372E-01
Tktl2	32915		#N/A	1.000000E+00
Tlcd1	32916	0.067057016	9649	3.454520E-01
Tlcd2	32917	0.156115341	16046	2.727784E-02
Tldc1	32918	0.020434681	6357	7.739526E-01
Tldc2	32919		#N/A	1.000000E+00
Tle1	32920	0.048847913	8206	4.921496E-01
Tle2	32921	0.078825977	10521	2.672113E-01
Tle3	32922	0.129735287	14284	6.710358E-02
Tle4	32923	0.081496733	10701	2.512894E-01
Tle6	32924	-0.026420372	4050	7.103669E-01
Tlk1	32925	0.095951843	11770	1.765100E-01
Tlk2	32926	0.191361743	17890	6.638949E-03
Tll1	32927	0.182231451	17500	9.804469E-03
Tll2	32928	-0.048916715	2217	4.915393E-01
Tln1	32929	0.138409143	14851	5.063627E-02
Tln2	32930	0.162634338	16502	2.139612E-02
Tlr1	32931	0.168179222	16834	1.728982E-02
Tlr11	32932	0.080873698	10656	2.549436E-01
Tlr12	32933	0.031462735	7157	6.582932E-01
Tlr13	32934	0.15104399	15714	3.276431E-02
Tlr2	32935	0.038585125	7533	5.875032E-01
Tlr3	32936	0.052132829	8481	4.634688E-01
Tlr4	32937	0.295310942	19887	2.183834E-05

Spearman Rank correlation analysis performed between Prdm1 and all-expressed genes within the Meredith RNA-seq dataset. Robust Prdm1–associated genes were identified using a cut-off of $p < 0.0005$.

Table S1, Related to Supplemental Figure 3C. Prdm1 associated genes

Tlr5	32938	-0.066858259	1029.5	3.468868E-01
Tlr6	32939		#N/A	1.000000E+00
Tlr7	32940	0.159178806	16235	2.436070E-02
Tlr8	32941		#N/A	1.000000E+00
Tlr9	32942		#N/A	1.000000E+00
Tlx1	32943	-0.054450825	1984	4.437997E-01
Tlx2	32944	0.156129434	16056.5	2.726377E-02
Tlx3	32945		#N/A	1.000000E+00
Tm2d1	32946	0.057128431	9042	4.216798E-01
Tm2d2	32947	0.142568195	15121	4.402213E-02
Tm2d3	32948	0.029925224	7062	6.740120E-01
Tm4sf1	32949	0.038758411	7558.5	5.858252E-01
Tm4sf19	32950		#N/A	1.000000E+00
Tm4sf20	32951	-0.03602617	3684	6.125352E-01
Tm4sf4	32952	0.045936564	7999	5.183375E-01
Tm4sf5	32953	0.140289504	14974	4.755013E-02
Tm6sf1	32954	0.138670169	14865	5.019816E-02
Tm6sf2	32955	-0.038406171	3065	5.892384E-01
Tm7sf2	32956	0.088050917	11207	2.150374E-01
Tm7sf3	32957	0.02438546	6783	7.317852E-01
Tm9sf1	32958	0.105012438	12357	1.389036E-01
Tm9sf2	32959	0.190673459	17867	6.841024E-03
Tm9sf3	32960	0.061253645	9278	3.888873E-01
Tm9sf4	32961	0.173550456	17101	1.398419E-02
Tma16	32962	0.057018231	9039	4.225773E-01
Tma7	32963	0.146003992	15411	3.911893E-02
Tma7-ps	32964	0.038229357	7499.5	5.909551E-01
Tmbim1	32965	-0.013148971	4611	8.533883E-01
Tmbim4	32966	0.199558174	18229	4.610742E-03
Tmbim6	32967	0.175146963	17172	1.311498E-02
Tmc1	32968	0.157524433	16128	2.590086E-02
Tmc2	32969	0.103869256	12289	1.432793E-01
Tmc3	32970	-0.038406171	3065	5.892384E-01
Tmc4	32971	0.09172217	11504	1.964467E-01
Tmc5	32972	0.128728812	14228	6.926993E-02
Tmc6	32973	0.153466927	15863	3.003600E-02
Tmc7	32974	-0.024715924	4107	7.282922E-01
Tmc8	32975	0.064191806	9485	3.664989E-01
Tmcc1	32976	0.12948637	14272	6.763413E-02
Tmcc2	32977	-0.010348411	4732	8.843673E-01
Tmcc3	32978	0.109010617	12625	1.244005E-01
Tmco1	32979	0.177306213	17282	1.201497E-02
Tmco2	32980		#N/A	1.000000E+00

Spearman Rank correlation analysis performed between Prdm1 and all-expressed genes within the Meredith RNA-seq dataset. Robust Prdm1–associated genes were identified using a cut-off of $p < 0.0005$.

Table S1, Related to Supplemental Figure 3C. Prdm1 associated genes

Tmco3	32981	0.039397716	7597	5.796537E-01
Tmco4	32982	0.000589045	5346	9.933951E-01
Tmco5	32983		#N/A	1.000000E+00
Tmco5b	32984	-0.038406171	3065	5.892384E-01
Tmco6	32985	0.126843487	14099	7.348120E-02
Tmed1	32986	-0.113574362	140	1.093101E-01
Tmed10	32987	0.197919714	18164	4.964818E-03
Tmed11	32988	-0.086747139	513	2.219331E-01
Tmed2	32989	0.226667573	19078	1.248402E-03
Tmed3	32990	0.192629883	17935	6.280533E-03
Tmed4	32991	0.046258664	8031	5.154054E-01
Tmed5	32992	0.306859885	19929	9.890737E-06
Tmed6	32993	-0.038406171	3065	5.892384E-01
Tmed7	32994	0.215218364	18753	2.209562E-03
Tmed8	32995	0.134333776	14581	5.789879E-02
Tmed9	32996	0.124279541	13926	7.953929E-02
Tmeff1	32997	0.148301654	15547	3.610358E-02
Tmeff2	32998	0.118275742	13547	9.530634E-02
Tmem100	32999	-0.117577649	117	9.729063E-02
Tmem101	33000	-0.00173561	5088	9.805404E-01
Tmem102	33001	0.033204119	7252	6.406698E-01
Tmem104	33002	0.111892306	12845	1.146954E-01
Tmem106a	33003	-0.030362391	3917	6.695279E-01
Tmem106b	33004	0.103402466	12266	1.450959E-01
Tmem106c	33005	0.127981604	14189	7.091482E-02
Tmem107	33006	0.092625823	11558	1.920555E-01
Tmem108	33007	0.03056662	7100	6.674370E-01
Tmem109	33008	0.206337488	18470	3.374673E-03
Tmem11	33009	0.141458124	15048	4.571241E-02
Tmem110	33010	0.151243424	15730	3.253206E-02
Tmem115	33011	0.078308187	10486	2.703761E-01
Tmem116	33012	-0.005089004	4930	9.429849E-01
Tmem117	33013	0.064884749	9531	3.613374E-01
Tmem119	33014	0.143605682	15224	4.249002E-02
Tmem120a	33015	-0.092379766	395	1.932440E-01
Tmem120b	33016	0.170007428	16931	1.609542E-02
Tmem121	33017	0.015676393	6102	8.256207E-01
Tmem123	33018	0.233272411	19233	8.864900E-04
Tmem125	33019	-0.038406171	3065	5.892384E-01
Tmem126a	33020	-0.004221822	4977	9.526880E-01
Tmem126b	33021	0.286097384	19842	4.011076E-05
Tmem127	33022	0.06351059	9436	3.716173E-01
Tmem128	33023	0.170479039	16955	1.579925E-02

Spearman Rank correlation analysis performed between Prdm1 and all-expressed genes within the Meredith RNA-seq dataset. Robust Prdm1–associated genes were identified using a cut-off of $p < 0.0005$.

Table S1, Related to Supplemental Figure 3C. Prdm1 associated genes

Tmem129	33024	0.112876163	12911	1.115209E-01
Tmem130	33025	0.025544243	6849.5	7.195619E-01
Tmem131	33026	0.102303169	12198	1.494430E-01
Tmem132a	33027	-0.08164349	624	2.504340E-01
Tmem132b	33028	-0.098157464	294	1.667272E-01
Tmem132c	33029	0.008708016	5714	9.025978E-01
Tmem132d	33030	-0.050750976	2186	4.754198E-01
Tmem132e	33031	0.113548679	13094	1.093908E-01
Tmem134	33032	0.126100866	14052	7.519603E-02
TMEM134	33033		#N/A	1.000000E+00
Tmem135	33034	0.121093972	13732	8.762066E-02
Tmem136	33035	0.013458335	5965.5	8.499790E-01
Tmem138	33036	0.039442407	7601	5.792234E-01
Tmem139	33037		#N/A	1.000000E+00
Tmem140	33038	0.118758674	13570	9.395256E-02
Tmem141	33039	0.045123932	7950	5.257728E-01
Tmem143	33040	0.166710466	16759	1.830454E-02
Tmem144	33041	0.022198316	6629.5	7.550388E-01
Tmem145	33042		#N/A	1.000000E+00
Tmem147	33043	0.007328025	5647	9.179743E-01
Tmem14a	33044	0.043324218	7827	5.424299E-01
Tmem14c	33045	0.044503888	7902	5.314821E-01
Tmem150a	33046	0.166148435	16721.5	1.870631E-02
Tmem150b	33047	-0.044861116	2341	5.281889E-01
Tmem150c	33048	0.082020958	10748	2.482430E-01
Tmem151a	33049	-0.038406171	3065	5.892384E-01
Tmem151b	33050	0.007287809	5645	9.184229E-01
Tmem154	33051	0.212849592	18686	2.477857E-03
Tmem156	33052	-0.086750697	472.5	2.219141E-01
Tmem158	33053	0.129441714	14270	6.772968E-02
Tmem159	33054	0.122386376	13810	8.426612E-02
Tmem160	33055	0.320127874	19966	3.816960E-06
Tmem161a	33056	0.074451208	10175	2.947518E-01
Tmem161b	33057	-0.002026528	5071	9.772795E-01
Tmem163	33058	0.203073815	18361	3.926488E-03
Tmem164	33059	0.294188529	19881	2.354369E-05
Tmem165	33060	0.123042159	13844	8.260387E-02
Tmem167	33061	0.233544158	19244	8.739109E-04
Tmem167b	33062	0.052480876	8498	4.604851E-01
Tmem168	33063	0.087914152	11196	2.157535E-01
Tmem169	33064	-0.038406171	3065	5.892384E-01
Tmem17	33065	0.100712333	12103	1.559077E-01
Tmem170	33066	-0.06379035	1348	3.695100E-01

Spearman Rank correlation analysis performed between Prdm1 and all-expressed genes within the Meredith RNA-seq dataset. Robust Prdm1–associated genes were identified using a cut-off of $p < 0.0005$.

Table S1, Related to Supplemental Figure 3C. Prdm1 associated genes

Tmem170b	33067	0.118711725	13568	9.408349E-02
Tmem171	33068	-0.066856562	1139	3.468991E-01
Tmem173	33069	0.110951781	12785	1.177957E-01
Tmem174	33070		#N/A	1.000000E+00
Tmem175	33071	0.151806638	15752	3.188369E-02
Tmem176a	33072	0.17511631	17171	1.313121E-02
Tmem176b	33073	0.086455828	11103	2.234951E-01
Tmem177	33074	0.113548679	13094	1.093908E-01
Tmem178	33075	-0.038406171	3065	5.892384E-01
Tmem178b	33076	0.169571218	16905	1.637367E-02
Tmem179	33077	-0.038406171	3065	5.892384E-01
Tmem179b	33078	0.267077249	19699	1.318125E-04
Tmem18	33079	0.019815744	6323	7.806226E-01
Tmem180	33080	0.010319984	5786	8.846827E-01
Tmem181a	33081	0.053267784	8685	4.537786E-01
Tmem181b-ps	33082		#N/A	1.000000E+00
Tmem181c-ps	33083		#N/A	1.000000E+00
Tmem182	33084	-0.038406171	3065	5.892384E-01
Tmem183a	33085	0.305647943	19924	1.076507E-05
TMEM184A	33086	0.143605682	15224	4.249002E-02
Tmem184a	33087	0.147175265	15487	3.755629E-02
Tmem184b	33088	0.143241768	15150	4.302226E-02
Tmem184c	33089	0.116491506	13431	1.004429E-01
Tmem185b	33090	0.058675469	9138	4.091976E-01
Tmem186	33091	0.211743307	18646	2.613018E-03
Tmem189	33092	-0.063818778	1343.5	3.692963E-01
Tmem19	33093	0.033719126	7281	6.354954E-01
Tmem190	33094		#N/A	1.000000E+00
Tmem191c	33095	0.270449875	19721	1.074187E-04
Tmem192	33096	0.034082105	7298	6.318591E-01
Tmem194	33097	0.027896127	6970	6.949737E-01
Tmem194b	33098	0.133053876	14481	6.034955E-02
Tmem196	33099	-0.086746694	532	2.219355E-01
Tmem198	33100		#N/A	1.000000E+00
Tmem198b	33101	0.114736704	13307	1.057060E-01
Tmem199	33102	0.029216186	7029	6.813093E-01
Tmem2	33103	0.115534541	13360	1.032868E-01
Tmem200a	33104	0.049855249	8285.5	4.832553E-01
Tmem200b	33105		#N/A	1.000000E+00
Tmem200c	33106		#N/A	1.000000E+00
Tmem201	33107	-0.047508344	2248	5.041109E-01
Tmem202	33108		#N/A	1.000000E+00
Tmem203	33109	0.071695104	9970	3.130391E-01

Spearman Rank correlation analysis performed between Prdm1 and all-expressed genes within the Meredith RNA-seq dataset. Robust Prdm1–associated genes were identified using a cut-off of $p < 0.0005$.

Table S1, Related to Supplemental Figure 3C. Prdm1 associated genes

Tmem204	33110	0.156129434	16056.5	2.726377E-02
Tmem205	33111	0.13679082	14750	5.342401E-02
Tmem206	33112	0.166789992	16763	1.824831E-02
Tmem207	33113	0.198845474	18193	4.761871E-03
Tmem208	33114	0.22938599	19134	1.085587E-03
Tmem209	33115	0.206205296	18465	3.395586E-03
Tmem210	33116		#N/A	1.000000E+00
Tmem211	33117		#N/A	1.000000E+00
Tmem212	33118	-0.038406171	3065	5.892384E-01
Tmem213	33119		#N/A	1.000000E+00
Tmem214	33120	0.171028854	16976	1.545997E-02
Tmem215	33121		#N/A	1.000000E+00
Tmem216	33122	0.004908042	5561	9.450091E-01
Tmem217	33123	-0.029345499	3955	6.799762E-01
Tmem218	33124	0.0958278	11755	1.770725E-01
Tmem219	33125	0.168342183	16841	1.718031E-02
Tmem220	33126	0.039879318	7635	5.750247E-01
Tmem221	33127		#N/A	1.000000E+00
Tmem222	33128	0.014424248	6037	8.393526E-01
Tmem223	33129	0.132263661	14432	6.190482E-02
Tmem225	33130		#N/A	1.000000E+00
Tmem229a	33131	0.195579992	18067	5.512764E-03
Tmem229b	33132	-0.076074634	863	2.843194E-01
Tmem230	33133	0.051379169	8419	4.699661E-01
Tmem231	33134	0.271521795	19728	1.005974E-04
Tmem232	33135	0.162050338	16470	2.187409E-02
Tmem233	33136	-0.000841282	5208.5	9.905669E-01
Tmem234	33137	0.134725936	14605	5.716453E-02
Tmem235	33138		#N/A	1.000000E+00
Tmem236	33139	0.141801558	15068	4.518377E-02
Tmem237	33140	0.175256564	17178	1.305708E-02
Tmem238	33141	0.099780794	12048	1.597897E-01
Tmem239	33142		#N/A	1.000000E+00
Tmem240	33143	-0.038406171	3065	5.892384E-01
Tmem241	33144	0.17217392	17033	1.477375E-02
Tmem242	33145	0.161462817	16450	2.236422E-02
Tmem243	33146	0.09008166	11351	2.046043E-01
Tmem245	33147	0.141022052	15021	4.639109E-02
Tmem246	33148	0.12156993	13762	8.637305E-02
Tmem247	33149	0.160304018	16323	2.335876E-02
Tmem248	33150	0.113421423	12951	1.097914E-01
Tmem25	33151	-0.038406171	3065	5.892384E-01
Tmem251	33152	0.229500453	19137	1.079180E-03

Spearman Rank correlation analysis performed between Prdm1 and all-expressed genes within the Meredith RNA-seq dataset. Robust Prdm1–associated genes were identified using a cut-off of $p < 0.0005$.

Table S1, Related to Supplemental Figure 3C. Prdm1 associated genes

Tmem252	33153	0.098009372	11916.5	1.673711E-01
Tmem253	33154	-0.054450825	1984	4.437997E-01
Tmem254a	33155		#N/A	1.000000E+00
Tmem254b	33156		#N/A	1.000000E+00
Tmem254c	33157		#N/A	1.000000E+00
Tmem255a	33158	0.165154635	16666	1.943548E-02
Tmem255b	33159	0.020434681	6357	7.739526E-01
Tmem256	33160	-0.036895506	3664	6.039788E-01
Tmem258	33161	0.129846019	14292	6.686866E-02
Tmem259	33162	0.070284159	9856	3.226820E-01
Tmem26	33163	0.119493598	13625	9.192179E-02
Tmem260	33164	0.168085993	16827	1.735274E-02
Tmem27	33165	0.042945725	7806	5.459659E-01
Tmem28	33166	0.220659828	18923	1.690430E-03
Tmem29	33167	0.005843763	5588	9.345465E-01
Tmem30a	33168	0.199624066	18232	4.596989E-03
Tmem30b	33169	0.117150646	13471	9.852043E-02
Tmem30c	33170	0.053805701	8820	4.492258E-01
Tmem33	33171	0.195943566	18084	5.424212E-03
Tmem35	33172	0.142100681	15089	4.472751E-02
Tmem37	33173	0.070307574	9861	3.225204E-01
Tmem38a	33174	0.022573858	6663	7.510297E-01
Tmem38b	33175	0.080089201	10598	2.595967E-01
Tmem39a	33176	0.08082042	10650	2.552578E-01
Tmem39b	33177	0.049988961	8295	4.820812E-01
Tmem40	33178	-0.0112492	4692	8.743815E-01
Tmem41a	33179	0.166062197	16707	1.876863E-02
Tmem41b	33180	0.175712703	17201	1.281854E-02
Tmem42	33181	0.016643264	6155	8.150532E-01
Tmem43	33182	0.087489669	11167	2.179868E-01
Tmem44	33183	0.113529326	12958	1.094516E-01
Tmem45a	33184	0.10002561	12069	1.587625E-01
Tmem45b	33185	0.096485706	11806	1.741040E-01
Tmem47	33186	0.193842084	17985	5.954190E-03
Tmem48	33187	0.079459756	10560	2.633721E-01
Tmem5	33188	-0.00332759	5015	9.627010E-01
Tmem50a	33189	0.205497686	18435	3.509531E-03
Tmem50b	33190	0.188017016	17762	7.673336E-03
Tmem51	33191	0.093880315	11636	1.860788E-01
Tmem51as1	33192	0.104439931	12320	1.410820E-01
Tmem52	33193	-0.030875227	3902	6.642825E-01
Tmem52b	33194	0.078518281	10496	2.690889E-01
Tmem53	33195	0.110269174	12733	1.200864E-01

Spearman Rank correlation analysis performed between Prdm1 and all-expressed genes within the Meredith RNA-seq dataset. Robust Prdm1–associated genes were identified using a cut-off of $p < 0.0005$.

Table S1, Related to Supplemental Figure 3C. Prdm1 associated genes

Tmem54	33196	0.013164107	5940.5	8.532214E-01
Tmem55a	33197	0.093684897	11625	1.870007E-01
Tmem55b	33198	0.036461908	7405	6.082398E-01
Tmem56	33199	0.14679402	15467	3.805903E-02
Tmem57	33200	0.194482232	18024	5.788044E-03
Tmem59	33201	0.241675262	19387	5.655287E-04
Tmem59l	33202	0.113548679	13094	1.093908E-01
Tmem60	33203	0.150728462	15696	3.313465E-02
Tmem61	33204	0.047203372	8096	5.068552E-01
Tmem62	33205	0.156984191	16106	2.642157E-02
Tmem63a	33206	0.058038635	9090	4.143092E-01
Tmem63b	33207	0.054852374	8912	4.404412E-01
Tmem63c	33208	0.165944699	16701	1.885383E-02
Tmem64	33209	0.185828983	17674	8.425302E-03
Tmem65	33210	-0.07974242	648	2.616720E-01
Tmem66	33211	0.096026871	11775	1.761704E-01
Tmem67	33212	0.168758486	16864	1.690329E-02
Tmem68	33213	0.092088179	11523	1.946594E-01
Tmem69	33214	0.229466886	19136	1.081055E-03
Tmem70	33215	0.181733743	17472	1.001021E-02
Tmem71	33216	0.139541	14925	4.875928E-02
Tmem72	33217	0.07521908	10268	2.897860E-01
Tmem74	33218		#N/A	1.000000E+00
Tmem74b	33219		#N/A	1.000000E+00
Tmem79	33220	-0.038070731	3631	5.924972E-01
Tmem8	33221	0.161163758	16432	2.261732E-02
Tmem80	33222	0.087741726	11180	2.166587E-01
Tmem81	33223	0.090778033	11409	2.011121E-01
Tmem82	33224	0.088830265	11257	2.109893E-01
Tmem86a	33225	-0.01009861	4741	8.871398E-01
Tmem86b	33226	-0.095267847	349	1.796284E-01
Tmem87a	33227	0.180811683	17440	1.040147E-02
Tmem87b	33228	0.039829044	7627	5.755071E-01
Tmem88	33229	0.142996312	15141	4.338439E-02
Tmem88b	33230	0.182189154	17498	9.821807E-03
Tmem89	33231		#N/A	1.000000E+00
Tmem8b	33232	0.006616784	5620	9.259116E-01
Tmem8c	33233	-0.00324393	5017	9.636381E-01
Tmem9	33234	0.152195571	15780	3.144240E-02
Tmem91	33235	0.143605682	15224	4.249002E-02
Tmem92	33236		#N/A	1.000000E+00
Tmem95	33237		#N/A	1.000000E+00
Tmem97	33238	0.101892226	12174	1.510932E-01

Spearman Rank correlation analysis performed between Prdm1 and all-expressed genes within the Meredith RNA-seq dataset. Robust Prdm1–associated genes were identified using a cut-off of $p < 0.0005$.

Table S1, Related to Supplemental Figure 3C. Prdm1 associated genes

Tmem98	33239	0.05815215	9101	4.133953E-01
Tmem9b	33240	0.14248877	15112	4.414131E-02
Tmf1	33241	0.089269641	11291	2.087314E-01
Tmie	33242	0.040198287	7647	5.719685E-01
Tmigd1	33243	-0.097781073	295	1.683674E-01
Tmlhe	33244		#N/A	1.000000E+00
Tmod1	33245	-0.010305687	4734	8.848414E-01
Tmod2	33246	-0.057017337	1456	4.225846E-01
Tmod3	33247	0.030926335	7123	6.637606E-01
Tmod4	33248		#N/A	1.000000E+00
Tmpo	33249	0.114627166	13300	1.060416E-01
Tmppe	33250	0.128605535	14216	6.953914E-02
Tmprss11a	33251	0.036711701	7414	6.057834E-01
Tmprss11bnl	33252	0.143605682	15224	4.249002E-02
Tmprss11c	33253	0.091314121	11471	1.984533E-01
Tmprss11d	33254	-0.000607765	5235	9.931852E-01
Tmprss11e	33255	-0.038406171	3065	5.892384E-01
Tmprss11f	33256	0.094676268	11684	1.823581E-01
Tmprss11g	33257	0.070422707	9867.5	3.217267E-01
Tmprss12	33258	0.015676693	6106.5	8.256174E-01
Tmprss13	33259	0.055994019	8986	4.309719E-01
Tmprss15	33260	0.021523625	6556	7.622579E-01
Tmprss2	33261	0.101829205	12169	1.513475E-01
Tmprss3	33262	-0.001224019	5151	9.862757E-01
Tmprss4	33263	0.054466277	8891	4.436702E-01
Tmprss5	33264	-0.077393517	797	2.760287E-01
Tmprss6	33265	-0.054450825	1984	4.437997E-01
Tmprss7	33266	0.201199528	18299	4.278960E-03
Tmprss9	33267	-0.066705033	1283	3.479955E-01
Tmsb10	33268	0.147338512	15495	3.734274E-02
Tmsb15a	33269	-0.110563117	176	1.190958E-01
Tmsb15b1	33270	0.045360957	7960	5.235985E-01
Tmsb15b2	33271	-0.01263616	4639	8.590456E-01
Tmsb15l	33272		#N/A	1.000000E+00
Tmsb4x	33273	0.194212837	18005	5.857452E-03
Tmtc1	33274	0.053767897	8787	4.495449E-01
Tmtc2	33275	0.055589218	8960	4.343160E-01
Tmtc3	33276	0.263627819	19667	1.620513E-04
Tmtc4	33277	0.022675663	6665	7.499440E-01
Tmub1	33278	0.036722257	7417	6.056797E-01
Tmub2	33279	0.130321826	14315	6.586685E-02
Tmx1	33280	0.173990775	17124	1.373956E-02
Tmx2	33281	0.117718101	13509	9.688880E-02

Spearman Rank correlation analysis performed between Prdm1 and all-expressed genes within the Meredith RNA-seq dataset. Robust Prdm1–associated genes were identified using a cut-off of $p < 0.0005$.

Table S1, Related to Supplemental Figure 3C. Prdm1 associated genes

Tmx3	33282	0.188187597	17769	7.617301E-03
Tmx4	33283	0.084304753	10943	2.352709E-01
Tnc	33284	0.18445569	17617	8.930022E-03
Tnf	33285	0.070626281	9883	3.203263E-01
Tnfaip1	33286	0.22528659	19054	1.339419E-03
Tnfaip2	33287	0.033127904	7248	6.414370E-01
Tnfaip3	33288	0.166664003	16755	1.833747E-02
Tnfaip6	33289	0.075084218	10253	2.906541E-01
Tnfaip8	33290	-0.000886648	5190	9.900582E-01
Tnfaip8l1	33291	0.182899254	17547	9.534294E-03
Tnfaip8l2	33292	-0.031190668	3881.5	6.610641E-01
Tnfaip8l3	33293	0.005539074	5581	9.379522E-01
Tnfrsf10b	33294	0.039637218	7609	5.773495E-01
Tnfrsf11a	33295	0.104315547	12312	1.415587E-01
Tnfrsf11b	33296	-0.003834083	4988	9.570288E-01
Tnfrsf12a	33297	0.053805701	8820	4.492258E-01
Tnfrsf13b	33298	0.380955903	20025	2.620907E-08
Tnfrsf13c	33299	0.097727459	11885	1.686020E-01
Tnfrsf14	33300	0.053267784	8685	4.537786E-01
Tnfrsf17	33301	0.015170343	6078	8.311643E-01
Tnfrsf18	33302	0.064740379	9524	3.624090E-01
Tnfrsf19	33303	0.010633758	5800	8.812020E-01
Tnfrsf1a	33304	0.182511476	17531	9.690365E-03
Tnfrsf1b	33305	0.010833306	5816	8.789896E-01
Tnfrsf21	33306	-0.015780228	4483	8.244843E-01
Tnfrsf22	33307	0.057750351	9070	4.166354E-01
Tnfrsf23	33308	0.196232307	18094	5.354796E-03
Tnfrsf25	33309		#N/A	1.000000E+00
Tnfrsf26	33310	0.04577129	7987	5.198453E-01
Tnfrsf4	33311	0.113346979	12945	1.100262E-01
Tnfrsf8	33312	0.055313892	8947	4.365990E-01
Tnfrsf9	33313	-0.030059262	3923	6.726359E-01
Tnfsf10	33314	0.10446533	12322	1.409848E-01
Tnfsf11	33315	-0.066156793	1302	3.519808E-01
Tnfsf12	33316	-0.009252819	4774	8.965370E-01
Tnfsf13	33317		#N/A	1.000000E+00
Tnfsf13b	33318	0.021757451	6575	7.597536E-01
Tnfsf14	33319		#N/A	1.000000E+00
Tnfsf15	33320	0.142399692	15104	4.427529E-02
Tnfsf18	33321	0.160847405	16381	2.288775E-02
Tnfsf4	33322	-0.024566572	4113	7.298702E-01
Tnfsf8	33323	0.273444872	19746	8.936580E-05
Tnfsf9	33324	0.001330412	5378	9.850829E-01

Spearman Rank correlation analysis performed between Prdm1 and all-expressed genes within the Meredith RNA-seq dataset. Robust Prdm1–associated genes were identified using a cut-off of $p < 0.0005$.

Table S1, Related to Supplemental Figure 3C. Prdm1 associated genes

Tnik	33325	0.083565548	10884	2.394164E-01
Tnip1	33326	0.05871804	9141	4.088572E-01
Tnip2	33327	0.145527954	15380	3.976951E-02
Tnip3	33328	0.054067254	8870	4.470214E-01
Tnk1	33329	0.123061013	13845	8.255647E-02
Tnk2	33330	0.064287846	9492	3.657808E-01
Tnks	33331	0.14172543	15062	4.530051E-02
Tnks1bp1	33332	0.06558575	9564	3.561621E-01
Tnks2	33333	0.146288449	15433	3.873447E-02
Tnmd	33334	-0.163471243	9	2.072690E-02
Tnn	33335	0.194042058	17992	5.901836E-03
Tnnc1	33336	0.073928833	10125	2.981622E-01
Tnnc2	33337	-0.05506313	1518.5	4.386842E-01
Tnni1	33338	-0.05506313	1518.5	4.386842E-01
Tnni2	33339	0.168314069	16839	1.719916E-02
Tnni3	33340	0.200166111	18247	4.485250E-03
Tnni3k	33341	0.01294526	5934	8.556347E-01
Tnnt1	33342	0.035841544	7368	6.143592E-01
Tnnt2	33343	0.044499877	7901	5.315191E-01
Tnnt3	33344	-0.007293557	4844	9.183588E-01
Tnp1	33345		#N/A	1.000000E+00
Tnp2	33346	-0.056961277	1463	4.230416E-01
Tnpo1	33347	0.066484891	9620	3.495923E-01
Tnpo2	33348	0.065961037	9587	3.534107E-01
Tnpo3	33349	0.049961161	8293	4.823252E-01
Tnr	33350	0.189953544	17839	7.058238E-03
Tnrc18	33351	0.036907161	7425	6.038644E-01
Tnrc6a	33352	0.158179009	16185	2.528176E-02
Tnrc6b	33353	0.067964124	9701	3.389513E-01
Tnrc6c	33354	0.168046027	16826	1.737977E-02
Tns1	33355	-0.002124238	5070	9.761843E-01
Tns3	33356	0.053504095	8772	4.517753E-01
Tns4	33357	-0.010481583	4728	8.828898E-01
Tnxa	33358	-0.038406171	3065	5.892384E-01
Tnxb	33359	0.114782157	13310	1.055670E-01
Tob1	33360	0.149197485	15603	3.498242E-02
Tob2	33361	0.178555822	17329	1.141617E-02
Toe1	33362	-0.065120317	1323	3.595931E-01
Tollip	33363	0.170658352	16963	1.568789E-02
Tom1	33364	0.075336593	10290	2.890310E-01
Tom1l1	33365	0.060636132	9239	3.936956E-01
Tom1l2	33366	0.027717838	6960	6.968270E-01
Tomm20	33367	0.118668211	13566	9.420498E-02

Spearman Rank correlation analysis performed between Prdm1 and all-expressed genes within the Meredith RNA-seq dataset. Robust Prdm1–associated genes were identified using a cut-off of $p < 0.0005$.

Table S1, Related to Supplemental Figure 3C. Prdm1 associated genes

Tomm20l	33368	-0.038406171	3065	5.892384E-01
Tomm22	33369	0.061617169	9307	3.860733E-01
Tomm34	33370	0.169230038	16890	1.659421E-02
Tomm40	33371	0.069447329	9810	3.284910E-01
Tomm40l	33372	-0.051148801	2178	4.719621E-01
Tomm5	33373	0.108165763	12561	1.273631E-01
Tomm6	33374	0.031412638	7154	6.588031E-01
Tomm7	33375	0.077896149	10463	2.729126E-01
Tomm70a	33376	0.190044798	17846	7.030369E-03
Tomt	33377	0.243622084	19423	5.084475E-04
Tonsl	33378	0.119780437	13650	9.113874E-02
Top1	33379	0.267419161	19703	1.291218E-04
Top1mt	33380	0.053043478	8599	4.556847E-01
Top2a	33381	0.123839683	13891	8.061799E-02
Top2b	33382	0.133773765	14537	5.896081E-02
Top3a	33383	0.206727528	18486	3.313647E-03
Top3b	33384	0.050153125	8308	4.806419E-01
Topaz1	33385	0.01287525	5929	8.564070E-01
Topbp1	33386	0.166199938	16730	1.866918E-02
Topors	33387	0.069625311	9823	3.272499E-01
Tor1a	33388	0.059788011	9200	4.003575E-01
Tor1aip1	33389	0.116034199	13397	1.017941E-01
Tor1aip2	33390	0.102560928	12214	1.484150E-01
Tor1b	33391	0.022943289	6692	7.470923E-01
Tor2a	33392	0.162570568	16498	2.144786E-02
Tor3a	33393	0.054290395	8883	4.451456E-01
Tor4a	33394	-0.036016652	3685	6.126292E-01
Tox	33395	0.268944818	19711	1.177297E-04
Tox2	33396	-0.103163388	239	1.460330E-01
Tox3	33397	0.072751028	10044	3.059471E-01
Tox4	33398	0.220342361	18909	1.717351E-03
Tpbg	33399	0.12370716	13888	8.094529E-02
Tpbpa	33400	-0.025168883	4095	7.235137E-01
Tpbpb	33401		#N/A	1.000000E+00
Tpcn1	33402	0.157420178	16125	2.600065E-02
Tpcn2	33403		#N/A	1.000000E+00
Tpd52	33404	-0.036467213	3673	6.081876E-01
Tpd52-ps	33405		#N/A	1.000000E+00
Tpd52l1	33406	0.176543784	17246	1.239374E-02
Tpd52l2	33407	0.151543468	15745	3.218527E-02
Tpgs1	33408	0.04380211	7852	5.379814E-01
Tpgs2	33409	0.236708738	19299	7.390351E-04
Tph1	33410	-0.085634162	559	2.279428E-01

Spearman Rank correlation analysis performed between Prdm1 and all-expressed genes within the Meredith RNA-seq dataset. Robust Prdm1–associated genes were identified using a cut-off of $p < 0.0005$.

Table S1, Related to Supplemental Figure 3C. Prdm1 associated genes

Tph2	33411	-0.016099701	4469	8.209901E-01
Tpi-rs8	33412		#N/A	1.000000E+00
Tpi1	33413	0.089539279	11313	2.073543E-01
Tpk1	33414	0.119266227	13604	9.254630E-02
Tpm1	33415	0.08588752	11047	2.265647E-01
Tpm2	33416	0.13111961	14360	6.421468E-02
Tpm3	33417	0.164516521	16633	1.991656E-02
Tpm3-rs2	33418		#N/A	1.000000E+00
Tpm3-rs7	33419	-0.06786264	969	3.396746E-01
Tpm4	33420	0.09955084	12032	1.607590E-01
Tpmt	33421	-0.014719183	4525.5	8.361136E-01
Tpo	33422	0.125386915	13996	7.687510E-02
Tpp1	33423	0.196865403	18115	5.205374E-03
Tpp2	33424	0.126454605	14075	7.437519E-02
Tppp	33425	-0.107307688	198	1.304275E-01
Tppp2	33426		#N/A	1.000000E+00
Tppp3	33427	0.114951958	13318	1.050490E-01
Tpr	33428	0.21361708	18703	2.387853E-03
Tpra1	33429	0.004038184	5518	9.547437E-01
Tprg	33430	0.093338807	11602	1.886417E-01
Tprgl	33431	0.184939603	17631	8.749193E-03
Tprkb	33432	0.136771552	14748	5.345795E-02
Tprn	33433	0.006456266	5612	9.277039E-01
Tpsab1	33434	-0.038406171	3065	5.892384E-01
Tpsb2	33435	-0.038406171	3065	5.892384E-01
Tpsg1	33436	-0.054451513	1703	4.437940E-01
Tpst1	33437	0.235234516	19273	7.992821E-04
Tpst2	33438	0.2033647	18368	3.874195E-03
Tpt1	33439	0.214142061	18719	2.328010E-03
Tpt1-ps1	33440	0.092687102	11562	1.917603E-01
Tpt1-ps2	33441	0.163743075	16580	2.051347E-02
Tpt1-ps3	33442		#N/A	1.000000E+00
Tpt1-ps5	33443	-0.095280123	319	1.795721E-01
Tpt1-ps6	33444	0.123933203	13907	8.038766E-02
Tpte	33445	0.049170356	8231	4.892931E-01
Tpx2	33446	0.097403503	11867	1.700248E-01
Tra2a	33447	0.076563984	10384	2.812240E-01
Tra2b	33448	0.150922349	15709	3.290666E-02
Trabd	33449	0.218182234	18848	1.911163E-03
Trabd2b	33450	0.251270511	19523	3.319722E-04
Trac	33451	-0.066855996	1239.5	3.469032E-01
Tradd	33452	-0.072369465	925	3.084975E-01
Traf1	33453	0.123039603	13843	8.261029E-02

Spearman Rank correlation analysis performed between Prdm1 and all-expressed genes within the Meredith RNA-seq dataset. Robust Prdm1–associated genes were identified using a cut-off of $p < 0.0005$.

Table S1, Related to Supplemental Figure 3C. Prdm1 associated genes

Traf2	33454	0.050004508	8297	4.819448E-01
Traf3	33455	0.063853007	9459	3.690391E-01
Traf3ip1	33456	0.042664787	7783	5.485979E-01
Traf3ip2	33457	-0.033429616	3764	6.384020E-01
Traf3ip3	33458	0.190270469	17850	6.961869E-03
Traf4	33459	-0.138103092	42	5.115400E-02
Traf5	33460	0.075998463	10347	2.848032E-01
Traf6	33461	0.154203262	15907	2.924626E-02
Traf7	33462	0.058011851	9086	4.145250E-01
Trafd1	33463	0.138280904	14843	5.085267E-02
Traip	33464	0.285784019	19840	4.093375E-05
Traj1	33465		#N/A	1.000000E+00
Traj11	33466		#N/A	1.000000E+00
Traj12	33467		#N/A	1.000000E+00
Traj13	33468		#N/A	1.000000E+00
Traj14	33469		#N/A	1.000000E+00
Traj15	33470		#N/A	1.000000E+00
Traj16	33471		#N/A	1.000000E+00
Traj17	33472		#N/A	1.000000E+00
Traj18	33473		#N/A	1.000000E+00
Traj19	33474		#N/A	1.000000E+00
Traj2	33475		#N/A	1.000000E+00
Traj20	33476		#N/A	1.000000E+00
Traj21	33477		#N/A	1.000000E+00
Traj22	33478		#N/A	1.000000E+00
Traj23	33479		#N/A	1.000000E+00
Traj24	33480		#N/A	1.000000E+00
Traj25	33481		#N/A	1.000000E+00
Traj26	33482		#N/A	1.000000E+00
Traj27	33483		#N/A	1.000000E+00
Traj28	33484		#N/A	1.000000E+00
Traj29	33485		#N/A	1.000000E+00
Traj3	33486		#N/A	1.000000E+00
Traj30	33487		#N/A	1.000000E+00
Traj31	33488		#N/A	1.000000E+00
Traj32	33489		#N/A	1.000000E+00
Traj33	33490		#N/A	1.000000E+00
Traj34	33491		#N/A	1.000000E+00
Traj35	33492		#N/A	1.000000E+00
Traj36	33493		#N/A	1.000000E+00
Traj37	33494		#N/A	1.000000E+00
Traj38	33495		#N/A	1.000000E+00
Traj39	33496		#N/A	1.000000E+00

Spearman Rank correlation analysis performed between Prdm1 and all-expressed genes within the Meredith RNA-seq dataset. Robust Prdm1–associated genes were identified using a cut-off of $p < 0.0005$.

Table S1, Related to Supplemental Figure 3C. Prdm1 associated genes

Traj4	33497		#N/A	1.000000E+00
Traj40	33498		#N/A	1.000000E+00
Traj41	33499		#N/A	1.000000E+00
Traj42	33500		#N/A	1.000000E+00
Traj43	33501		#N/A	1.000000E+00
Traj44	33502		#N/A	1.000000E+00
Traj45	33503		#N/A	1.000000E+00
Traj46	33504		#N/A	1.000000E+00
Traj48	33505		#N/A	1.000000E+00
Traj49	33506	-0.038406171	3065	5.892384E-01
Traj5	33507		#N/A	1.000000E+00
Traj50	33508		#N/A	1.000000E+00
Traj51	33509		#N/A	1.000000E+00
Traj52	33510		#N/A	1.000000E+00
Traj53	33511		#N/A	1.000000E+00
Traj54	33512		#N/A	1.000000E+00
Traj55	33513		#N/A	1.000000E+00
Traj56	33514		#N/A	1.000000E+00
Traj57	33515		#N/A	1.000000E+00
Traj58	33516		#N/A	1.000000E+00
Traj59	33517		#N/A	1.000000E+00
Traj6	33518		#N/A	1.000000E+00
Traj60	33519		#N/A	1.000000E+00
Traj61	33520		#N/A	1.000000E+00
Traj7	33521		#N/A	1.000000E+00
Traj8	33522		#N/A	1.000000E+00
Traj9	33523		#N/A	1.000000E+00
Trak1	33524	0.11250973	12891	1.126951E-01
Trak2	33525	0.159435586	16251	2.412885E-02
Tram1	33526	0.199520878	18228	4.618542E-03
Tram1l1	33527	0.021317126	6513.5	7.644715E-01
Tram2	33528	0.129685989	14282	6.720839E-02
Trank1	33529	0.051839491	8459	4.659917E-01
Trap1	33530	0.095382472	11729	1.791030E-01
Trap1a	33531	0.143605682	15224	4.249002E-02
Trappc1	33532	0.072054903	10002	3.106106E-01
Trappc10	33533	0.011283938	5845	8.739968E-01
Trappc11	33534	0.009648699	5758	8.921367E-01
Trappc12	33535	0.100727143	12106	1.558465E-01
Trappc13	33536	0.123595571	13878	8.122171E-02
Trappc2	33537	0.106733854	12478	1.325083E-01
Trappc2l	33538	0.107330793	12514	1.303443E-01
Trappc3	33539	-0.002847662	5036	9.680774E-01

Spearman Rank correlation analysis performed between Prdm1 and all-expressed genes within the Meredith RNA-seq dataset. Robust Prdm1–associated genes were identified using a cut-off of $p < 0.0005$.

Table S1, Related to Supplemental Figure 3C. Prdm1 associated genes

Trappc4	33540	0.09143747	11480	1.978452E-01
Trappc5	33541	-0.034814295	3715	6.245509E-01
Trappc6a	33542	0.145297507	15371	4.008771E-02
Trappc6b	33543	0.198666193	18187	4.800582E-03
Trappc8	33544	0.276105356	19766	7.575440E-05
Trappc9	33545	0.126480391	14076	7.431564E-02
Trat1	33546	0.020875719	6412.5	7.692098E-01
Trav1	33547		#N/A	1.000000E+00
Trav10	33548		#N/A	1.000000E+00
Trav10d	33549		#N/A	1.000000E+00
Trav10n	33550		#N/A	1.000000E+00
Trav11	33551		#N/A	1.000000E+00
Trav11d	33552		#N/A	1.000000E+00
Trav12-1	33553		#N/A	1.000000E+00
Trav12-2	33554		#N/A	1.000000E+00
Trav12-3	33555		#N/A	1.000000E+00
Trav12d-1	33556		#N/A	1.000000E+00
Trav12d-2	33557		#N/A	1.000000E+00
Trav12d-3	33558		#N/A	1.000000E+00
Trav12n-1	33559		#N/A	1.000000E+00
Trav12n-2	33560		#N/A	1.000000E+00
Trav12n-3	33561		#N/A	1.000000E+00
Trav13-1	33562		#N/A	1.000000E+00
Trav13-2	33563		#N/A	1.000000E+00
Trav13-3	33564		#N/A	1.000000E+00
Trav13-4-dv7	33565		#N/A	1.000000E+00
Trav13d-1	33566		#N/A	1.000000E+00
Trav13d-2	33567		#N/A	1.000000E+00
Trav13d-3	33568		#N/A	1.000000E+00
Trav13d-4	33569		#N/A	1.000000E+00
Trav13n-2	33570		#N/A	1.000000E+00
Trav13n-3	33571		#N/A	1.000000E+00
Trav13n-4	33572		#N/A	1.000000E+00
Trav14-1	33573		#N/A	1.000000E+00
Trav14-2	33574		#N/A	1.000000E+00
Trav14-3	33575		#N/A	1.000000E+00
Trav14d-1	33576		#N/A	1.000000E+00
Trav14d-2	33577		#N/A	1.000000E+00
Trav14d-3-dv8	33578		#N/A	1.000000E+00
Trav14n-1	33579		#N/A	1.000000E+00
Trav14n-2	33580		#N/A	1.000000E+00
Trav14n-3	33581		#N/A	1.000000E+00
Trav15-1-dv6-1	33582		#N/A	1.000000E+00

Spearman Rank correlation analysis performed between Prdm1 and all-expressed genes within the Meredith RNA-seq dataset. Robust Prdm1–associated genes were identified using a cut-off of $p < 0.0005$.

Table S1, Related to Supplemental Figure 3C. Prdm1 associated genes

Trav15-2-dv6-2	33583	#N/A	1.000000E+00
Trav15d-1-dv6d	33584	#N/A	1.000000E+00
Trav15d-2-dv6d	33585	#N/A	1.000000E+00
Trav15n-1	33586	#N/A	1.000000E+00
Trav15n-2	33587	#N/A	1.000000E+00
Trav16	33588	#N/A	1.000000E+00
Trav16d-dv11	33589	#N/A	1.000000E+00
Trav16n	33590	#N/A	1.000000E+00
Trav17	33591	#N/A	1.000000E+00
Trav18	33592	#N/A	1.000000E+00
Trav19	33593	#N/A	1.000000E+00
Trav2	33594	#N/A	1.000000E+00
Trav20	33595	#N/A	1.000000E+00
Trav21-dv12	33596	#N/A	1.000000E+00
Trav3-1	33597	#N/A	1.000000E+00
Trav3-3	33598	#N/A	1.000000E+00
Trav3-4	33599	#N/A	1.000000E+00
Trav3d-3	33600	#N/A	1.000000E+00
Trav3n-3	33601	#N/A	1.000000E+00
Trav4-2	33602	#N/A	1.000000E+00
Trav4-3	33603	#N/A	1.000000E+00
Trav4-4-dv10	33604	#N/A	1.000000E+00
Trav4d-2	33605	#N/A	1.000000E+00
Trav4d-3	33606	#N/A	1.000000E+00
Trav4d-4	33607	#N/A	1.000000E+00
Trav4n-3	33608	#N/A	1.000000E+00
Trav4n-4	33609	#N/A	1.000000E+00
Trav5-1	33610	#N/A	1.000000E+00
Trav5-4	33611	#N/A	1.000000E+00
Trav5d-3	33612	#N/A	1.000000E+00
Trav5d-4	33613	#N/A	1.000000E+00
Trav5n-3	33614	#N/A	1.000000E+00
Trav5n-4	33615	#N/A	1.000000E+00
Trav6-1	33616	#N/A	1.000000E+00
Trav6-2	33617	#N/A	1.000000E+00
Trav6-3	33618	#N/A	1.000000E+00
Trav6-4	33619	#N/A	1.000000E+00
Trav6-5	33620	#N/A	1.000000E+00
Trav6-6	33621	#N/A	1.000000E+00
Trav6-7-dv9	33622	#N/A	1.000000E+00
Trav6d-3	33623	#N/A	1.000000E+00
Trav6d-4	33624	#N/A	1.000000E+00
Trav6d-5	33625	#N/A	1.000000E+00

Spearman Rank correlation analysis performed between Prdm1 and all-expressed genes within the Meredith RNA-seq dataset. Robust Prdm1–associated genes were identified using a cut-off of $p < 0.0005$.

Table S1, Related to Supplemental Figure 3C. Prdm1 associated genes

Trav6d-6	33626		#N/A	1.000000E+00
Trav6d-7	33627		#N/A	1.000000E+00
Trav6n-5	33628		#N/A	1.000000E+00
Trav6n-6	33629		#N/A	1.000000E+00
Trav6n-7	33630		#N/A	1.000000E+00
Trav7-2	33631		#N/A	1.000000E+00
Trav7-3	33632		#N/A	1.000000E+00
Trav7-4	33633		#N/A	1.000000E+00
Trav7-6	33634		#N/A	1.000000E+00
Trav7d-3	33635		#N/A	1.000000E+00
Trav7d-4	33636		#N/A	1.000000E+00
Trav7d-5	33637		#N/A	1.000000E+00
Trav7n-4	33638		#N/A	1.000000E+00
Trav7n-6	33639		#N/A	1.000000E+00
Trav8-1	33640		#N/A	1.000000E+00
Trav8-2	33641		#N/A	1.000000E+00
Trav8d-1	33642		#N/A	1.000000E+00
Trav8d-2	33643		#N/A	1.000000E+00
Trav8n-2	33644		#N/A	1.000000E+00
Trav9-1	33645		#N/A	1.000000E+00
Trav9-2	33646		#N/A	1.000000E+00
Trav9-4	33647		#N/A	1.000000E+00
Trav9d-1	33648		#N/A	1.000000E+00
Trav9d-2	33649		#N/A	1.000000E+00
Trav9d-3	33650		#N/A	1.000000E+00
Trav9d-4	33651		#N/A	1.000000E+00
Trav9n-1	33652		#N/A	1.000000E+00
Trav9n-2	33653		#N/A	1.000000E+00
Trav9n-3	33654		#N/A	1.000000E+00
Trav9n-4	33655		#N/A	1.000000E+00
Trbc1	33656	0.071667294	9964.5	3.132274E-01
Trbc2	33657	-0.000841282	5208.5	9.905669E-01
Trbd1	33658		#N/A	1.000000E+00
Trbd2	33659		#N/A	1.000000E+00
Trbj1-1	33660		#N/A	1.000000E+00
Trbj1-2	33661		#N/A	1.000000E+00
Trbj1-3	33662		#N/A	1.000000E+00
Trbj1-4	33663		#N/A	1.000000E+00
Trbj1-5	33664		#N/A	1.000000E+00
Trbj1-6	33665		#N/A	1.000000E+00
Trbj1-7	33666		#N/A	1.000000E+00
Trbj2-1	33667		#N/A	1.000000E+00
Trbj2-2	33668		#N/A	1.000000E+00

Spearman Rank correlation analysis performed between Prdm1 and all-expressed genes within the Meredith RNA-seq dataset. Robust Prdm1–associated genes were identified using a cut-off of $p < 0.0005$.

Table S1, Related to Supplemental Figure 3C. Prdm1 associated genes

Trbj2-3	33669		#N/A	1.000000E+00
Trbj2-4	33670		#N/A	1.000000E+00
Trbj2-5	33671		#N/A	1.000000E+00
Trbj2-6	33672		#N/A	1.000000E+00
Trbj2-7	33673		#N/A	1.000000E+00
Trbv1	33674		#N/A	1.000000E+00
Trbv10	33675		#N/A	1.000000E+00
Trbv12-1	33676		#N/A	1.000000E+00
Trbv12-2	33677		#N/A	1.000000E+00
Trbv13-1	33678		#N/A	1.000000E+00
Trbv13-2	33679		#N/A	1.000000E+00
Trbv13-3	33680		#N/A	1.000000E+00
Trbv14	33681		#N/A	1.000000E+00
Trbv15	33682		#N/A	1.000000E+00
Trbv16	33683		#N/A	1.000000E+00
Trbv17	33684		#N/A	1.000000E+00
Trbv19	33685		#N/A	1.000000E+00
Trbv2	33686		#N/A	1.000000E+00
Trbv20	33687		#N/A	1.000000E+00
Trbv21	33688		#N/A	1.000000E+00
Trbv23	33689		#N/A	1.000000E+00
Trbv26	33690		#N/A	1.000000E+00
Trbv29	33691		#N/A	1.000000E+00
Trbv3	33692		#N/A	1.000000E+00
Trbv30	33693		#N/A	1.000000E+00
Trbv31	33694		#N/A	1.000000E+00
Trbv4	33695		#N/A	1.000000E+00
Trbv5	33696		#N/A	1.000000E+00
Trcg1	33697	-0.038406171	3065	5.892384E-01
Trdd1	33698		#N/A	1.000000E+00
Trdd2	33699		#N/A	1.000000E+00
Trdj1	33700		#N/A	1.000000E+00
Trdj2	33701		#N/A	1.000000E+00
Trdmt1	33702	-0.065715118	1315	3.552122E-01
Trdn	33703	0.046567959	8049	5.125979E-01
Trdv1	33704		#N/A	1.000000E+00
Trdv2-1	33705		#N/A	1.000000E+00
Trdv2-2	33706	-0.054450825	1984	4.437997E-01
Trdv4	33707		#N/A	1.000000E+00
Trdv5	33708		#N/A	1.000000E+00
Treh	33709	-0.066855996	1239.5	3.469032E-01
Trem1	33710	0.021317126	6513.5	7.644715E-01
Trem2	33711	0.044692002	7912	5.297467E-01

Spearman Rank correlation analysis performed between Prdm1 and all-expressed genes within the Meredith RNA-seq dataset. Robust Prdm1–associated genes were identified using a cut-off of $p < 0.0005$.

Table S1, Related to Supplemental Figure 3C. Prdm1 associated genes

Trem3	33712	0.194426528	18020	5.802336E-03
Treml1	33713	-0.038406171	3065	5.892384E-01
Treml2	33714	0.089688848	11322	2.065933E-01
Treml4	33715	0.209140397	18554	2.957791E-03
Trerf1	33716	-0.008460622	4803	9.053519E-01
Trex1	33717		#N/A	1.000000E+00
Trex2	33718	0.070306791	9860	3.225258E-01
Trf	33719	0.025899292	6865	7.158307E-01
Trgj1	33720		#N/A	1.000000E+00
Trgj2	33721		#N/A	1.000000E+00
Trgj3	33722		#N/A	1.000000E+00
Trgv2	33723		#N/A	1.000000E+00
Trh	33724		#N/A	1.000000E+00
Trhde	33725	0.115937514	13392	1.020816E-01
Trhr	33726	0.196335906	18098	5.330085E-03
Trhr2	33727	-0.054450825	1984	4.437997E-01
Triap1	33728	0.057846394	9078	4.158596E-01
Trib1	33729	0.223605487	19009	1.458401E-03
Trib2	33730	0.1307391	14337	6.499841E-02
Trib3	33731	-0.013874983	4571	8.453919E-01
Tril	33732	0.113548679	13094	1.093908E-01
Trim10	33733	0.160304018	16323	2.335876E-02
Trim11	33734	0.02442273	6785	7.313909E-01
Trim12a	33735	0.223564175	19006	1.461443E-03
Trim12c	33736	0.236580067	19298	7.441218E-04
Trim13	33737	-0.105077274	207	1.386585E-01
Trim14	33738	0.305687029	19925	1.073576E-05
Trim15	33739	0.13754922	14793	5.210214E-02
Trim16	33740	0.07494696	10242	2.915394E-01
Trim17	33741	-0.059404652	1408	4.033906E-01
Trim2	33742	0.141659794	15057	4.540136E-02
Trim21	33743	0.115673741	13371	1.028692E-01
Trim23	33744	0.02653784	6905	7.091373E-01
Trim24	33745	0.155435134	16002	2.796468E-02
Trim25	33746	0.229853314	19147	1.059646E-03
Trim26	33747	0.292073662	19871	2.710399E-05
Trim27	33748	0.192676634	17940	6.267657E-03
Trim28	33749	-0.003077815	5021	9.654989E-01
Trim29	33750	0.054366898	8887	4.445035E-01
Trim3	33751	0.286244715	19843	3.972924E-05
Trim30a	33752	0.245319682	19444	4.630737E-04
Trim30b	33753	0.138142562	14835	5.108699E-02
Trim30c	33754		#N/A	1.000000E+00

Spearman Rank correlation analysis performed between Prdm1 and all-expressed genes within the Meredith RNA-seq dataset. Robust Prdm1–associated genes were identified using a cut-off of $p < 0.0005$.

Table S1, Related to Supplemental Figure 3C. Prdm1 associated genes

Trim30d	33755	0.082128853	10757	2.476192E-01
Trim30e-ps1	33756	0.013842768	5982.5	8.457464E-01
Trim31	33757	0.299963136	19907	1.593717E-05
Trim32	33758	0.167956382	16819	1.744055E-02
Trim33	33759	0.166481025	16747	1.846764E-02
Trim34a	33760	0.189207366	17810	7.289841E-03
Trim34b	33761	0.132586736	14453	6.126502E-02
Trim35	33762	0.061415847	9293	3.876302E-01
Trim36	33763	-0.001748614	5085	9.803946E-01
Trim37	33764	0.148301748	15548	3.610346E-02
Trim38	33765	0.160987082	16412.5	2.276801E-02
Trim39	33766	0.031302163	7143	6.599280E-01
Trim40	33767	0.120256463	13677	8.985099E-02
Trim41	33768	0.015808708	6113	8.241726E-01
Trim42	33769	0.020611413	6377.5	7.720510E-01
Trim43a	33770	-0.034791257	3716	6.247803E-01
Trim43b	33771	0.009455661	5747	8.942819E-01
Trim43c	33772	0.197775826	18156	4.997050E-03
Trim44	33773	0.149263698	15607	3.490074E-02
Trim45	33774	-0.044852651	2344	5.282669E-01
Trim46	33775	-0.016940955	4416	8.118061E-01
Trim47	33776	0.147091782	15481	3.766589E-02
Trim5	33777	0.123913566	13899	8.043598E-02
Trim50	33778	-0.038406171	3065	5.892384E-01
Trim52	33779		#N/A	1.000000E+00
Trim54	33780	0.093053905	11591	1.900005E-01
Trim55	33781	0.122142283	13794	8.489166E-02
Trim56	33782	0.182787224	17542	9.579153E-03
Trim58	33783	-0.095280123	319	1.795721E-01
Trim59	33784	0.085154974	10997	2.305654E-01
Trim6	33785	0.009559301	5751	8.931301E-01
Trim60	33786	-0.044861116	2341	5.281889E-01
Trim61	33787		#N/A	1.000000E+00
Trim62	33788	-0.104642649	210	1.403077E-01
Trim63	33789	0.120696541	13704	8.867345E-02
Trim65	33790		#N/A	1.000000E+00
Trim66	33791	5.11172E-05	5294	9.994268E-01
Trim67	33792	0.055132697	8934	4.381052E-01
Trim68	33793	-0.010203794	4738	8.859722E-01
Trim69	33794	0.176966165	17267	1.218263E-02
Trim7	33795	0.032292243	7196	6.498740E-01
Trim71	33796	0.038031883	7485	5.928751E-01
Trim72	33797	0.000879918	5355	9.901337E-01

Spearman Rank correlation analysis performed between Prdm1 and all-expressed genes within the Meredith RNA-seq dataset. Robust Prdm1–associated genes were identified using a cut-off of $p < 0.0005$.

Trim75	33798	0.053267784	8685	4.537786E-01
Trim8	33799	0.218259312	18849	1.903918E-03
Trim9	33800	0.051008732	8385	4.731779E-01
Triml1	33801	-0.054451513	1703	4.437940E-01
Triml2	33802	-0.038406171	3065	5.892384E-01
Trio	33803	0.109214257	12639	1.236944E-01
Triobp	33804	0.043962049	7862	5.364967E-01
Trip10	33805	0.178943104	17351	1.123600E-02
Trip11	33806	0.141850031	15073	4.510957E-02
Trip12	33807	0.262832777	19656	1.698847E-04
Trip13	33808	-0.001640569	5092	9.816058E-01
Trip4	33809	0.158963769	16223	2.455633E-02
Trip6	33810	-0.141082832	38	4.629600E-02
Triqk	33811	0.070199409	9853	3.232673E-01
Trit1	33812	-0.066664255	1286.5	3.482910E-01
Trmt1	33813	0.194540607	18028	5.773102E-03
Trmt10a	33814	0.017261427	6188	8.083142E-01
Trmt10b	33815	-0.089293765	426	2.086079E-01
Trmt10c	33816	0.002568741	5449	9.712027E-01
Trmt11	33817	-0.02246521	4159	7.521889E-01
Trmt112	33818	0.215172975	18749	2.214444E-03
Trmt112-ps2	33819	0.142475201	15110	4.416169E-02
Trmt12	33820	0.059933207	9207	3.992122E-01
Trmt13	33821	0.08268099	10802	2.444440E-01
Trmt1l	33822	0.125216602	13984	7.728009E-02
Trmt2a	33823	0.182070981	17486	9.870391E-03
Trmt2b	33824	-0.037487561	3647	5.981822E-01
Trmt44	33825	-0.038406171	3065	5.892384E-01
Trmt5	33826	-0.110562037	181.5	1.190994E-01
Trmt6	33827	0.198755114	18188	4.781347E-03
Trmt61a	33828	0.174690538	17151	1.335850E-02
Trmt61b	33829	0.066135135	9600	3.521388E-01
Trmu	33830	0.058721481	9142	4.088297E-01
Trnau1ap	33831	0.22940408	19135	1.084572E-03
Trnp1	33832	0.090471093	11377	2.026460E-01
Trnt1	33833	0.084244162	10938	2.356088E-01
Tro	33834		#N/A	1.000000E+00
Troap	33835	-0.006988697	4852	9.217601E-01
Trove2	33836	0.271359396	19727	1.016041E-04
Trp53	33837	0.269385561	19714	1.146181E-04
Trp53-ps	33838		#N/A	1.000000E+00
Trp53bp1	33839	0.080795162	10649	2.554068E-01
Trp53bp2	33840	-0.004836138	4945	9.458135E-01

Spearman Rank correlation analysis performed between Prdm1 and all-expressed genes within the Meredith RNA-seq dataset. Robust Prdm1–associated genes were identified using a cut-off of $p < 0.0005$.

Table S1, Related to Supplemental Figure 3C. Prdm1 associated genes

Trp53cor1	33841	0.092465541	11548	1.928291E-01
Trp53i11	33842	0.190458924	17862	6.905122E-03
Trp53i13	33843	0.015098436	6074	8.319527E-01
Trp53inp1	33844	0.11004996	12717	1.208294E-01
Trp53inp2	33845	0.159269081	16241	2.427897E-02
Trp53rk	33846	-0.016012169	4471	8.219471E-01
Trp53tg5	33847		#N/A	1.000000E+00
Trp63	33848	0.076456996	10376	2.818988E-01
Trp73	33849	0.105857939	12411	1.357336E-01
Trpa1	33850	0.085706783	11033	2.275472E-01
Trpc1	33851	0.062624908	9368	3.783374E-01
Trpc2	33852	0.113438955	12954	1.097361E-01
Trpc3	33853	0.037855817	7476	5.945894E-01
Trpc4	33854	0.03005205	7066	6.727100E-01
Trpc4ap	33855	0.190730543	17870	6.824059E-03
Trpc5	33856	0.03845615	7523	5.887535E-01
Trpc6	33857	-0.029632741	3947	6.770186E-01
Trpc7	33858	0.199468723	18225	4.629471E-03
Trpd52l3	33859		#N/A	1.000000E+00
Trpm1	33860	0.043257178	7820	5.430554E-01
Trpm2	33861	0.067921528	9698	3.392548E-01
Trpm3	33862	0.162272445	16482	2.169123E-02
Trpm4	33863	0.009629162	5757	8.923537E-01
Trpm5	33864	-0.044852651	2344	5.282669E-01
Trpm6	33865	0.152532964	15794	3.106381E-02
Trpm7	33866	0.104686574	12333	1.401403E-01
Trpm8	33867	-0.084599978	578	2.336294E-01
Trps1	33868	0.092310526	11539	1.935794E-01
Trpt1	33869	0.068518974	9740	3.350136E-01
Trpv1	33870		#N/A	1.000000E+00
Trpv2	33871	0.337502846	19994	1.022503E-06
Trpv3	33872	0.127545848	14155	7.188866E-02
Trpv4	33873	0.109311817	12644	1.233573E-01
Trpv5	33874	-0.086755146	452	2.218903E-01
Trpv6	33875		#N/A	1.000000E+00
Trrap	33876	-0.048786765	2221	4.926923E-01
Trub1	33877	0.137931249	14816	5.144662E-02
Trub2	33878	0.103552567	12273	1.445098E-01
Try10	33879		#N/A	1.000000E+00
Try4	33880		#N/A	1.000000E+00
Try5	33881		#N/A	1.000000E+00
Tsacc	33882	0.07494696	10242	2.915394E-01
Tsc1	33883	0.127970197	14188	7.094017E-02

Spearman Rank correlation analysis performed between Prdm1 and all-expressed genes within the Meredith RNA-seq dataset. Robust Prdm1–associated genes were identified using a cut-off of $p < 0.0005$.

Table S1, Related to Supplemental Figure 3C. Prdm1 associated genes

Tsc2	33884	0.171496993	16998	1.517613E-02
Tsc22d1	33885	0.176040642	17225	1.264941E-02
Tsc22d2	33886	0.066961151	9642	3.461436E-01
Tsc22d3	33887	0.04957471	8259	4.857236E-01
Tsc22d4	33888	0.208323931	18533	3.074132E-03
Tsen15	33889	0.026815553	6920	7.062331E-01
Tsen2	33890	0.056375729	9003	4.278322E-01
Tsen34	33891	0.094163511	11653	1.847486E-01
Tsen54	33892	0.007112963	5630	9.203735E-01
Tsfm	33893	0.106839946	12484	1.321217E-01
Tsg101	33894	0.189245948	17812	7.277702E-03
Tsga10	33895	0.239945389	19363	6.211601E-04
Tsga10ip	33896	0.18218536	17494	9.823364E-03
Tsga13	33897	0.074574898	10201.5	2.939481E-01
Tsga8	33898		#N/A	1.000000E+00
Tshb	33899	0.090319075	11370.5	2.034088E-01
Tshr	33900	0.124643983	13948	7.865439E-02
Tshz1	33901	0.003849323	5510	9.568582E-01
Tshz2	33902	0.168427198	16846	1.712342E-02
Tshz3	33903	-0.036963362	3661	6.033132E-01
Tsix	33904	0.225661595	19065	1.314121E-03
Tsks	33905		#N/A	1.000000E+00
Tsku	33906	0.102276118	12196	1.495512E-01
Tslp	33907	0.026186067	6880	7.128219E-01
Tsn	33908	0.26737002	19702	1.295054E-04
Tsnax	33909	-0.010960534	4704	8.775794E-01
Tsnaxip1	33910		#N/A	1.000000E+00
Tspan1	33911	0.071187574	9925	3.164859E-01
Tspan10	33912	-0.038406171	3065	5.892384E-01
Tspan11	33913	-0.038406171	3065	5.892384E-01
Tspan12	33914	-0.030015393	3925	6.730862E-01
Tspan13	33915	0.204431555	18405	3.687736E-03
Tspan14	33916	-0.054492278	1540	4.434523E-01
Tspan15	33917	0.195021543	18045	5.651303E-03
Tspan17	33918	-0.077393517	797	2.760287E-01
Tspan18	33919	0.062912168	9386	3.761498E-01
Tspan2	33920	0.122308083	13804	8.446636E-02
Tspan3	33921	0.111395541	12815	1.163249E-01
Tspan31	33922	0.089005814	11273	2.100851E-01
Tspan32	33923	0.12098834	13722	8.789950E-02
Tspan33	33924	0.076298597	10368	2.828998E-01
Tspan4	33925	0.115962102	13393	1.020084E-01
Tspan5	33926	0.057146011	9044	4.215367E-01

Spearman Rank correlation analysis performed between Prdm1 and all-expressed genes within the Meredith RNA-seq dataset. Robust Prdm1–associated genes were identified using a cut-off of $p < 0.0005$.

Table S1, Related to Supplemental Figure 3C. Prdm1 associated genes

Tspan6	33927	0.110273259	12735	1.200726E-01
Tspan7	33928	-0.03526127	3704	6.201074E-01
Tspan8	33929	0.081375994	10691	2.519947E-01
Tspan9	33930	0.134216965	14574	5.811901E-02
Tspear	33931	-0.117568763	126	9.731610E-02
Tspo	33932	0.134501818	14589	5.758321E-02
Tspo2	33933		#N/A	1.000000E+00
Tspy-ps	33934		#N/A	1.000000E+00
Tspyl1	33935	0.189460373	17820	7.210564E-03
Tspyl2	33936	0.102503542	12211	1.486434E-01
Tspyl3	33937	-0.03968831	2475	5.768585E-01
Tspyl4	33938	0.092299984	11537	1.936305E-01
Tspyl5	33939	0.030788867	7116	6.651646E-01
Tsr1	33940	0.070761828	9892	3.193962E-01
Tsr2	33941	0.012986637	5937	8.551784E-01
Tsr3	33942	0.027986005	6972	6.940402E-01
Tssc1	33943	-0.013840742	4572	8.457687E-01
Tssc4	33944	0.23962263	19359	6.320831E-04
Tssk1	33945		#N/A	1.000000E+00
Tssk2	33946		#N/A	1.000000E+00
Tssk3	33947		#N/A	1.000000E+00
Tssk4	33948	0.012510416	5904	8.604339E-01
Tssk5	33949		#N/A	1.000000E+00
Tssk6	33950	-0.054451513	1703	4.437940E-01
Tst	33951	0.030114934	7071	6.720647E-01
Tsta3	33952	0.074031534	10138	2.974896E-01
Tstd1	33953	0.058008057	9085	4.145556E-01
Tstd2	33954	0.026628126	6911	7.081926E-01
Tstd3	33955	0.200861472	18279	4.345483E-03
Tsx	33956	-0.033842756	3743	6.342559E-01
Ttbk1	33957	0.110142006	12728	1.205170E-01
Ttbk2	33958		#N/A	1.000000E+00
Ttc1	33959	0.311284769	19942	7.236427E-06
Ttc12	33960	0.125556558	14007	7.647341E-02
Ttc13	33961	0.178468466	17325	1.145716E-02
Ttc14	33962	0.206547626	18479	3.341670E-03
Ttc16	33963	-0.045728124	2303	5.202395E-01
Ttc17	33964	0.158330229	16191	2.514056E-02
Ttc18	33965	-0.001606757	5107.5	9.819849E-01
Ttc19	33966	0.349413808	20003	3.952265E-07
Ttc21a	33967	0.137979339	14822	5.136459E-02
Ttc21b	33968	0.147323024	15494	3.736295E-02
Ttc22	33969	0.146652217	15457	3.824747E-02

Spearman Rank correlation analysis performed between Prdm1 and all-expressed genes within the Meredith RNA-seq dataset. Robust Prdm1–associated genes were identified using a cut-off of $p < 0.0005$.

Ttc23	33970	-0.009685131	4758	8.917319E-01
Ttc23l	33971	0.008042291	5681	9.100114E-01
Ttc24	33972	-0.038406171	3065	5.892384E-01
Ttc25	33973	-0.083865975	593	2.377255E-01
Ttc26	33974	0.142056868	15083	4.479410E-02
Ttc27	33975	0.150319765	15669	3.361961E-02
Ttc28	33976	0.182467818	17526	9.708077E-03
Ttc29	33977	-0.059044693	1415	4.062511E-01
Ttc3	33978	0.052017436	8473	4.644603E-01
Ttc30a1	33979	-0.018103658	4360	7.991553E-01
Ttc30a2	33980		#N/A	1.000000E+00
Ttc30b	33981	-0.033685727	3755	6.358305E-01
Ttc32	33982	0.19716862	18128	5.135142E-03
Ttc33	33983	0.041210645	7699	5.623195E-01
Ttc34	33984		#N/A	1.000000E+00
Ttc36	33985	-0.086746694	532	2.219355E-01
Ttc37	33986	0.040099158	7644	5.729175E-01
Ttc38	33987	0.067343449	9662	3.433908E-01
Ttc39a	33988	-0.000515622	5239	9.942184E-01
Ttc39b	33989	0.051566252	8433	4.683486E-01
Ttc39c	33990	0.149600429	15624	3.448783E-02
Ttc39d	33991	-0.095269893	339	1.796190E-01
Ttc4	33992	-0.047400416	2252	5.050812E-01
Ttc5	33993	0.089370338	11296	2.082164E-01
Ttc6	33994	0.042444137	7769	5.506694E-01
Ttc7	33995	0.260861406	19634	1.908591E-04
Ttc7b	33996	0.155577199	16010	2.782002E-02
Ttc8	33997	0.01953327	6305	7.836721E-01
Ttc9	33998	0.185164643	17645	8.666212E-03
Ttc9b	33999		#N/A	1.000000E+00
Ttc9c	34000	0.125559665	14008	7.646607E-02
Ttf1	34001	0.070798031	9896	3.191480E-01
Ttf2	34002	0.148345392	15551	3.604814E-02
Tti1	34003	-0.013692461	4577	8.474008E-01
Tti2	34004	0.023524318	6719	7.409130E-01
Ttk	34005	0.101751519	12165	1.516614E-01
Ttl	34006	-0.039511718	2480	5.785564E-01
Ttll1	34007	-0.013834702	4573	8.458351E-01
Ttll10	34008	-0.018790377	4285	7.917077E-01
Ttll11	34009	0.099323611	12022	1.617212E-01
Ttll12	34010	-0.000353424	5271	9.960371E-01
Ttll13	34011	0.187620974	17748.5	7.804850E-03
Ttll2	34012	-0.072957106	921	3.045754E-01

Spearman Rank correlation analysis performed between Prdm1 and all-expressed genes within the Meredith RNA-seq dataset. Robust Prdm1–associated genes were identified using a cut-off of $p < 0.0005$.

Table S1, Related to Supplemental Figure 3C. Prdm1 associated genes

Ttll3	34013	-0.009748154	4755	8.910317E-01
Ttll4	34014	0.059604494	9189	4.018078E-01
Ttll5	34015	-0.029649566	3945	6.768455E-01
Ttll6	34016	-0.054451513	1703	4.437940E-01
Ttll7	34017	-0.086755146	452	2.218903E-01
Ttll8	34018	-0.000841282	5208.5	9.905669E-01
Ttll9	34019	0.054942821	8920	4.396867E-01
Ttn	34020	-0.005304417	4913	9.405759E-01
Ttpa	34021	0.050544302	8344.5	4.772215E-01
Ttpal	34022	0.211699481	18641	2.618507E-03
Ttr	34023	0.156129434	16056.5	2.726377E-02
Ttyh1	34024	-0.023499877	4139	7.411726E-01
Ttyh2	34025	0.114950418	13317	1.050537E-01
Ttyh3	34026	0.073108459	10071	3.035706E-01
Tub	34027	0.05027093	8324	4.796104E-01
Tuba1a	34028	0.150802059	15700	3.304795E-02
Tuba1b	34029	0.218501143	18855	1.881348E-03
Tuba1c	34030	0.108990295	12622	1.244711E-01
Tuba3a	34031	0.049711368	8269.5	4.845204E-01
Tuba3b	34032	0.134506409	14590	5.757461E-02
Tuba4a	34033	0.0761393	10356	2.839090E-01
Tuba8	34034	0.000945354	5358	9.894000E-01
Tubal3	34035	-0.013118278	4616	8.537267E-01
Tubb1	34036	0.03859329	7534	5.874240E-01
Tubb2a	34037	0.181295314	17457	1.019460E-02
Tubb2b	34038	0.116965247	13461	9.905821E-02
Tubb3	34039		#N/A	1.000000E+00
Tubb4a	34040	-0.028627192	3974	6.873937E-01
Tubb4b	34041	0.194274935	18007	5.841387E-03
Tubb4b-ps1	34042		#N/A	1.000000E+00
Tubb5	34043	0.173856073	17115	1.381400E-02
Tubb6	34044	0.182731499	17539	9.601536E-03
Tubd1	34045	0.281454771	19809	5.405995E-05
Tube1	34046	0.125851286	14028	7.577958E-02
Tubg1	34047	0.00232725	5433	9.739090E-01
Tubg2	34048	-0.013305152	4604	8.516668E-01
Tubgcp2	34049	0.195029	18047	5.649433E-03
Tubgcp3	34050	0.289482435	19862	3.215963E-05
Tubgcp4	34051	-0.05488755	1523	4.401477E-01
Tubgcp5	34052	-0.015812735	4479	8.241286E-01
Tubgcp6	34053	0.108065908	12558	1.277168E-01
Tufm	34054	0.039423056	7599	5.794097E-01
Tuft1	34055	0.182033494	17482	9.885847E-03

Spearman Rank correlation analysis performed between Prdm1 and all-expressed genes within the Meredith RNA-seq dataset. Robust Prdm1–associated genes were identified using a cut-off of $p < 0.0005$.

Table S1, Related to Supplemental Figure 3C. Prdm1 associated genes

Tug1	34056	0.15761061	16132	2.581862E-02
Tulp1	34057	-0.076075371	861	2.843147E-01
Tulp2	34058	0.194525649	18027	5.776928E-03
Tulp3	34059	0.06499914	9535	3.604897E-01
Tulp4	34060	0.168757399	16863	1.690401E-02
Tusc1	34061	0.180113177	17410	1.070680E-02
Tusc2	34062	0.013723932	5976	8.470543E-01
Tusc3	34063	0.056954193	9034	4.230994E-01
Tusc5	34064	-0.004369004	4972	9.510406E-01
Tut1	34065	-0.006377937	4870	9.285787E-01
Tvp23a	34066	0.006281801	5609	9.296525E-01
Tvp23b	34067	0.217377484	18831	1.988322E-03
Twf1	34068	0.205725067	18444	3.472545E-03
Twf2	34069	0.008824486	5717	9.013016E-01
Twist1	34070		#N/A	1.000000E+00
Twist2	34071	0.16003052	16279	2.359897E-02
Twistnb	34072	0.050122705	8305	4.809084E-01
Twsg1	34073	0.03771497	7472	5.959624E-01
Txk	34074	0.086918145	11121	2.210198E-01
Txlna	34075	0.074931311	10232	2.916404E-01
Txlnb	34076	0.073115751	10073	3.035222E-01
Txlng	34077	0.152396331	15790	3.121666E-02
Txn1	34078	0.22067162	18925	1.689437E-03
Txn2	34079	0.055242149	8941	4.371950E-01
Txndc11	34080	0.118219775	13542	9.546423E-02
Txndc12	34081	0.092288193	11536	1.936877E-01
Txndc15	34082	0.211585145	18635	2.632878E-03
Txndc16	34083	0.258852661	19612	2.146967E-04
Txndc17	34084	-0.005417184	4902	9.393149E-01
Txndc2	34085	0.143605682	15224	4.249002E-02
Txndc5	34086	0.203394926	18369	3.868797E-03
Txndc8	34087	0.081762057	10730.5	2.497444E-01
Txndc9	34088	0.219534867	18888	1.787597E-03
Txnip	34089	0.19134941	17889	6.642523E-03
Txnl1	34090	0.136004521	14701	5.482374E-02
Txnl4a	34091	0.046787385	8066	5.106110E-01
Txnl4b	34092	0.078014589	10469	2.721819E-01
Txnrd1	34093	0.074118692	10144	2.969196E-01
Txnrd2	34094	0.127253485	14131	7.254813E-02
Txnrd3	34095	-0.024757321	4105	7.278551E-01
Tyk2	34096	0.095898044	11762	1.767538E-01
Tymp	34097	-0.019133696	4262	7.879914E-01
Tyms	34098	0.110506846	12759	1.192849E-01

Spearman Rank correlation analysis performed between Prdm1 and all-expressed genes within the Meredith RNA-seq dataset. Robust Prdm1–associated genes were identified using a cut-off of $p < 0.0005$.

Table S1, Related to Supplemental Figure 3C. Prdm1 associated genes

Tyr	34099	0.010226762	5781	8.857173E-01
Tyro3	34100	0.113548679	13094	1.093908E-01
Tyrobp	34101	0.113376088	12946	1.099343E-01
Tyrp1	34102	-0.038406171	3065	5.892384E-01
Tysnd1	34103	0.146278378	15432	3.874803E-02
Tyw1	34104	-0.012546795	4643	8.600322E-01
Tyw3	34105	0.002280552	5431	9.744324E-01
Tyw5	34106	0.025117923	6830	7.240508E-01
U2	34107		#N/A	1.000000E+00
U2af1	34108	0.173878492	17117	1.380158E-02
U2af1l4	34109	0.190160779	17849	6.995090E-03
U2af2	34110	0.059958162	9209	3.990156E-01
U2surp	34111	0.271580645	19729	1.002349E-04
U3	34112		#N/A	1.000000E+00
U6	34113		#N/A	1.000000E+00
U90926	34114	-0.062529552	1370	3.790654E-01
Uaca	34115	0.022198129	6607.5	7.550408E-01
Uap1	34116	0.130198244	14307	6.612586E-02
Uap1l1	34117	0.098508986	11956	1.652062E-01
Uba1	34118	0.259586783	19620	2.056804E-04
Uba1y	34119	-0.036935945	3662	6.035821E-01
Uba2	34120	0.118938803	13581	9.345155E-02
Uba3	34121	0.096129032	11782	1.757088E-01
Uba5	34122	0.115708627	13377	1.027648E-01
Uba52	34123	0.155607877	16012	2.778887E-02
Uba6	34124	-0.018092582	4366	7.992756E-01
Uba7	34125	0.014012526	5988	8.438787E-01
Ubac1	34126	-0.056470589	1468	4.270540E-01
Ubac2	34127	-0.009816411	4753	8.902735E-01
Ubald1	34128	0.057459695	9059	4.189885E-01
Ubald2	34129	0.177248687	17278	1.204319E-02
Ubap1	34130	-0.022226271	4167	7.547401E-01
Ubap2	34131	0.166138959	16717	1.871315E-02
Ubap2l	34132	0.119309439	13608	9.242735E-02
Ubash3a	34133	0.071667294	9964.5	3.132274E-01
Ubash3b	34134	-0.014127712	4564	8.426120E-01
Ubb	34135	0.188368494	17775	7.558275E-03
Ubc	34136	0.220263496	18906	1.724099E-03
Ubd	34137	0.090125599	11359	2.043826E-01
Ube2a	34138	0.183892696	17590	9.144563E-03
Ube2b	34139		#N/A	1.000000E+00
Ube2c	34140	0.082037147	10751	2.481494E-01
Ube2cbp	34141	0.154035147	15890	2.942499E-02

Spearman Rank correlation analysis performed between Prdm1 and all-expressed genes within the Meredith RNA-seq dataset. Robust Prdm1–associated genes were identified using a cut-off of $p < 0.0005$.

Table S1, Related to Supplemental Figure 3C. Prdm1 associated genes

Ube2d1	34142	0.092918146	11578	1.906504E-01
Ube2d2a	34143	0.130028982	14301	6.648197E-02
Ube2d2b	34144	0.182398428	17520.5	9.736288E-03
Ube2d3	34145	0.230714418	19182	1.013330E-03
Ube2d4	34146		#N/A	1.000000E+00
Ube2dnl1	34147		#N/A	1.000000E+00
Ube2dnl2	34148		#N/A	1.000000E+00
Ube2e1	34149	0.141493046	15050	4.565842E-02
Ube2e2	34150	0.041827818	7733	5.564757E-01
Ube2e3	34151	0.190921674	17877	6.767527E-03
Ube2f	34152	0.08914235	11282	2.093837E-01
Ube2g1	34153	0.136537454	14730	5.387177E-02
Ube2g2	34154	0.070547273	9876	3.208694E-01
Ube2h	34155	0.290849348	19868	2.939126E-05
Ube2i	34156	0.145353324	15376	4.001044E-02
Ube2j1	34157	0.14918769	15602	3.499452E-02
Ube2j2	34158	-0.011564055	4685	8.708958E-01
Ube2k	34159	0.108350837	12572	1.267095E-01
Ube2l3	34160	0.116172947	13407	1.013827E-01
Ube2l6	34161	0.119725468	13640	9.128839E-02
Ube2m	34162	0.23870527	19342	6.641048E-04
Ube2n	34163	0.161048926	16428	2.271516E-02
Ube2nl	34164		#N/A	1.000000E+00
Ube2o	34165	-0.099754341	277	1.599009E-01
Ube2q1	34166	0.113317789	12942	1.101184E-01
Ube2q2	34167	0.215646644	18783	2.163974E-03
Ube2ql1	34168	0.117906595	13520	9.635158E-02
Ube2r2	34169	0.080769585	10648	2.555578E-01
Ube2s	34170	0.111970592	12852	1.144403E-01
Ube2t	34171	0.292499409	19872	2.634875E-05
Ube2u	34172	0.262829376	19655	1.699189E-04
Ube2v1	34173	0.021444718	6548	7.631035E-01
Ube2v2	34174	0.136746665	14745	5.350182E-02
Ube2w	34175	0.094206704	11657	1.845464E-01
Ube2z	34176	0.233892864	19256	8.580100E-04
Ube3a	34177	0.152275501	15787	3.135236E-02
Ube3b	34178	0.179444115	17380	1.100664E-02
Ube3c	34179	0.217736844	18840	1.953523E-03
Ube4a	34180	0.070381281	9866	3.220121E-01
Ube4b	34181	0.101351047	12140	1.532873E-01
Ubfd1	34182	0.051739619	8451	4.668524E-01
Ubiad1	34183	-0.103166823	231.5	1.460195E-01
Ubl3	34184	0.034650812	7319	6.261795E-01

Spearman Rank correlation analysis performed between Prdm1 and all-expressed genes within the Meredith RNA-seq dataset. Robust Prdm1–associated genes were identified using a cut-off of $p < 0.0005$.

Table S1, Related to Supplemental Figure 3C. Prdm1 associated genes

Ubl4	34185	0.11108819	12794	1.173421E-01
Ubl4b	34186	-0.038406171	3065	5.892384E-01
Ubl5	34187	0.182399627	17523	9.735799E-03
Ubl7	34188	0.137532759	14791	5.213054E-02
Ublcp1	34189	0.130882954	14347	6.470120E-02
Ubn1	34190	0.155233681	15991	2.817092E-02
Ubn2	34191	0.075223074	10279	2.897603E-01
Ubox5	34192	0.055574745	8958	4.344358E-01
Ubp1	34193	0.057905672	9080	4.153811E-01
Ubqln1	34194	0.193701008	17981	5.991374E-03
Ubqln2	34195	0.069480943	9815	3.282564E-01
Ubqln3	34196		#N/A	1.000000E+00
Ubqln4	34197	0.135832729	14685	5.513356E-02
Ubqlnl	34198		#N/A	1.000000E+00
Ubr1	34199	0.029740947	7048	6.759057E-01
Ubr2	34200	0.02308345	6698	7.456002E-01
Ubr3	34201	0.265988964	19683	1.407300E-04
Ubr4	34202	0.175737885	17204	1.280548E-02
Ubr5	34203	0.096360719	11798	1.746651E-01
Ubr7	34204	0.192072313	17914	6.435935E-03
Ubtd1	34205	0.20473632	18414	3.635979E-03
Ubtd2	34206	0.186068217	17685	8.340015E-03
Ubtf	34207	0.175069919	17169	1.315581E-02
Ubtfl1	34208	0.091084598	11446	1.995885E-01
Ubxn1	34209	0.105039984	12359	1.387994E-01
Ubxn10	34210		#N/A	1.000000E+00
Ubxn11	34211	0.128883336	14232	6.893367E-02
Ubxn2a	34212	0.157394327	16122	2.602545E-02
Ubxn2b	34213	0.093619431	11622	1.873103E-01
Ubxn4	34214	0.142635296	15127	4.392166E-02
Ubxn6	34215	0.029275306	7033	6.806997E-01
Ubxn7	34216	0.18545698	17659	8.559459E-03
Ubxn8	34217	0.149285964	15608	3.487331E-02
Uchl1	34218	-0.038754609	2494	5.858620E-01
Uchl3	34219	0.244027984	19427	4.972390E-04
Uchl4	34220	0.225925218	19070	1.296599E-03
Uchl5	34221	0.093519445	11616	1.877839E-01
Uck1	34222	0.056654764	9019	4.255454E-01
Uck2	34223	0.158381258	16194	2.509306E-02
Uckl1	34224	0.148343241	15550	3.605086E-02
Ucma	34225	0.04259543	7781	5.492486E-01
Ucn	34226		#N/A	1.000000E+00
Ucn2	34227	0.035015175	7330	6.225522E-01

Spearman Rank correlation analysis performed between Prdm1 and all-expressed genes within the Meredith RNA-seq dataset. Robust Prdm1–associated genes were identified using a cut-off of $p < 0.0005$.

Table S1, Related to Supplemental Figure 3C. Prdm1 associated genes

Ucn3	34228		#N/A	1.000000E+00
Ucp1	34229	0.04658885	8053	5.124086E-01
Ucp2	34230	0.199103983	18205	4.706547E-03
Ucp3	34231	0.083452862	10867	2.400529E-01
Uevld	34232	0.018035599	6223	7.998944E-01
Ufc1	34233	0.024020709	6755	7.356469E-01
Ufd1l	34234	0.131873791	14399	6.268419E-02
Ufl1	34235	0.075227708	10280	2.897305E-01
Ufm1	34236	0.252467554	19532	3.101711E-04
Ufsp1	34237	-0.049310495	2213	4.880544E-01
Ufsp2	34238	0.20115713	18297	4.287252E-03
Ugcg	34239	0.18723403	17726	7.935275E-03
Ugdh	34240	-0.014706085	4529	8.362574E-01
Uggt1	34241	0.033031425	7240	6.424087E-01
Uggt2	34242	0.220832156	18928	1.675978E-03
Ugp2	34243	0.165035103	16662	1.952482E-02
Ugt1a1	34244		#N/A	1.000000E+00
Ugt1a10	34245	-0.038406171	3065	5.892384E-01
Ugt1a2	34246		#N/A	1.000000E+00
Ugt1a5	34247		#N/A	1.000000E+00
Ugt1a6a	34248		#N/A	1.000000E+00
Ugt1a6b	34249		#N/A	1.000000E+00
Ugt1a7c	34250	0.07521908	10268	2.897860E-01
Ugt1a8	34251		#N/A	1.000000E+00
Ugt1a9	34252	0.113548679	13094	1.093908E-01
Ugt2a1	34253	-0.038406171	3065	5.892384E-01
Ugt2a3	34254	-0.000458472	5251.5	9.948592E-01
Ugt2b1	34255		#N/A	1.000000E+00
Ugt2b34	34256	0.038229357	7499.5	5.909551E-01
Ugt2b35	34257		#N/A	1.000000E+00
Ugt2b36	34258	-0.054451513	1703	4.437940E-01
Ugt2b37	34259	-0.038406171	3065	5.892384E-01
Ugt2b38	34260	0.046992983	8073	5.087530E-01
Ugt2b5	34261	-0.077393517	797	2.760287E-01
Ugt3a1	34262	-0.038406171	3065	5.892384E-01
Ugt3a2	34263	0.204921954	18424	3.604775E-03
Ugt8a	34264	-0.099778912	276	1.597976E-01
Uhmk1	34265	0.046261295	8032	5.153815E-01
Uhrf1	34266	0.098543221	11960	1.650586E-01
Uhrf1bp1	34267	0.23697579	19306	7.285801E-04
Uhrf1bp1l	34268	0.159773095	16263	2.382700E-02
Uhrf2	34269	0.184358036	17611	8.966912E-03
Uimc1	34270	0.106846531	12486	1.320977E-01

Spearman Rank correlation analysis performed between Prdm1 and all-expressed genes within the Meredith RNA-seq dataset. Robust Prdm1–associated genes were identified using a cut-off of $p < 0.0005$.

Table S1, Related to Supplemental Figure 3C. Prdm1 associated genes

Ulbp1	34271	0.113548679	13094	1.093908E-01
Ulk1	34272	0.034905545	7325	6.236426E-01
Ulk2	34273	0.089988729	11341	2.050736E-01
Ulk3	34274	0.039790759	7616	5.758746E-01
Ulk4	34275	0.076925156	10410	2.789540E-01
Umod	34276	-0.019986735	4242	7.787783E-01
Umodl1	34277	-0.066856562	1139	3.468991E-01
Umps	34278	0.290508942	19865	3.005885E-05
Unc119	34279	0.025141067	6831	7.238068E-01
Unc119b	34280	0.045516683	7969	5.221725E-01
Unc13a	34281	0.166339454	16735	1.856891E-02
Unc13b	34282	0.060016859	9213	3.985533E-01
Unc13c	34283	0.101507398	12148	1.526509E-01
Unc13d	34284	0.069369657	9804	3.290336E-01
Unc45a	34285	0.151000912	15713	3.281467E-02
Unc45b	34286	-0.010780472	4711	8.795753E-01
Unc50	34287	0.162742095	16507	2.130892E-02
Unc5a	34288	-0.053004144	2139	4.560194E-01
Unc5b	34289	0.083013583	10834	2.425451E-01
Unc5c	34290	0.1809679	17446	1.033425E-02
Unc5cl	34291	-0.10317255	224	1.459970E-01
Unc5d	34292	-0.054782882	1527	4.410214E-01
Unc79	34293	0.108270962	12567	1.269912E-01
Unc80	34294	0.050559564	8349	4.770884E-01
Unc93a	34295	-0.074050317	902	2.973667E-01
Unc93b1	34296	0.227417257	19091	1.201400E-03
Uncx	34297	-0.038406171	3065	5.892384E-01
Ung	34298	0.05565547	8964	4.337677E-01
Unk	34299	0.09455854	11677	1.829049E-01
Unkl	34300	0.122645561	13824	8.360597E-02
Uox	34301	0.113548679	13094	1.093908E-01
Upb1	34302	0.134098265	14559	5.834348E-02
Upf1	34303	0.152563419	15796	3.102983E-02
Upf2	34304	-0.045387123	2317	5.233588E-01
Upf3a	34305	0.079783519	10578	2.614255E-01
Upf3b	34306	0.101502015	12147	1.526728E-01
Upk1a	34307	0.005051706	5566	9.434021E-01
Upk1b	34308	-0.054450825	1984	4.437997E-01
Upk2	34309	0.014913507	6064.5	8.339810E-01
Upk3a	34310	-0.005265066	4917	9.410159E-01
Upk3b	34311		#N/A	1.000000E+00
Upk3bl	34312	-0.054451513	1703	4.437940E-01
Upp1	34313	-0.066856562	1139	3.468991E-01

Spearman Rank correlation analysis performed between Prdm1 and all-expressed genes within the Meredith RNA-seq dataset. Robust Prdm1–associated genes were identified using a cut-off of $p < 0.0005$.

Table S1, Related to Supplemental Figure 3C. Prdm1 associated genes

Upp2	34314	0.048953243	8217	4.912155E-01
Uprt	34315	0.071035763	9913	3.175216E-01
Uqcc	34316	0.001236911	5375	9.861311E-01
Uqcr10	34317	0.152168113	15778	3.147339E-02
Uqcr11	34318	0.135925374	14694	5.496630E-02
Uqcrb	34319	0.182602485	17535	9.653535E-03
Uqcrc1	34320	0.096235239	11791	1.752298E-01
Uqcrc2	34321	0.103499212	12270	1.447179E-01
Uqcrfs1	34322	0.145220967	15365	4.019387E-02
Uqcrh	34323	0.198286035	18177	4.883602E-03
Uqcrq	34324	0.215364276	18758	2.193933E-03
Urb1	34325	0.016561928	6148	8.159409E-01
Urb2	34326		#N/A	1.000000E+00
Urgcp	34327	0.284518247	19830	4.442263E-05
Uri1	34328	0.023477826	6717	7.414068E-01
Urm1	34329	-0.035722696	3692.5	6.155346E-01
Uroc1	34330	0.142827778	15137	4.363452E-02
Urod	34331	0.218617903	18861	1.870539E-03
Uros	34332	0.069404944	9808	3.287870E-01
Usb1	34333	0.147561249	15510	3.705302E-02
Use1	34334	0.235554704	19278	7.858250E-04
Usf1	34335	0.150156466	15660	3.381507E-02
Usf2	34336	0.160117193	16282	2.352262E-02
Ush1c	34337	-0.022095106	4169	7.561417E-01
Ush1g	34338		#N/A	1.000000E+00
Ush2a	34339	0.193458869	17970	6.055679E-03
Ushbp1	34340	0.004458738	5537	9.500363E-01
Usmg5	34341	0.177409282	17285	1.196455E-02
Uso1	34342	0.060053512	9215	3.982648E-01
Usp-ps	34343		#N/A	1.000000E+00
Usp1	34344	0.293753357	19880	2.423809E-05
Usp10	34345	0.223988744	19017	1.430455E-03
Usp11	34346	0.222331269	18963	1.554941E-03
Usp12	34347	0.069907125	9838	3.252910E-01
Usp13	34348	0.085387945	11015	2.292877E-01
Usp14	34349	0.226676771	19079	1.247815E-03
Usp15	34350	0.052088306	8477	4.638512E-01
Usp16	34351	0.131560916	14374	6.331544E-02
Usp17la	34352	0.109799969	12701	1.216810E-01
Usp17lb	34353		#N/A	1.000000E+00
Usp17lc	34354		#N/A	1.000000E+00
Usp17ld	34355		#N/A	1.000000E+00
Usp17le	34356		#N/A	1.000000E+00

Spearman Rank correlation analysis performed between Prdm1 and all-expressed genes within the Meredith RNA-seq dataset. Robust Prdm1–associated genes were identified using a cut-off of $p < 0.0005$.

Table S1, Related to Supplemental Figure 3C. Prdm1 associated genes

Usp18	34357	0.023677284	6731	7.392889E-01
Usp19	34358	0.203945204	18387	3.771710E-03
Usp2	34359	0.190046677	17847	7.029796E-03
Usp20	34360	-0.020605514	4218	7.721145E-01
Usp21	34361	0.149707275	15632	3.435768E-02
Usp22	34362	0.212337293	18668	2.539638E-03
Usp24	34363	0.164810075	16644	1.969398E-02
Usp25	34364	0.162575428	16499	2.144392E-02
Usp26	34365	0.105881191	12412	1.356472E-01
Usp27x	34366	-0.038406171	3065	5.892384E-01
Usp28	34367	0.142095269	15087	4.473573E-02
Usp29	34368		#N/A	1.000000E+00
Usp3	34369	0.099695852	12041	1.601472E-01
Usp30	34370	-0.056067499	1486	4.303665E-01
Usp31	34371	0.186998764	17716	8.015518E-03
Usp32	34372	0.072946246	10062	3.046476E-01
Usp33	34373	0.11922419	13603	9.266212E-02
Usp34	34374	0.132027253	14412	6.237645E-02
Usp35	34375	-0.101495574	266	1.526990E-01
Usp36	34376	0.208481217	18536	3.051403E-03
Usp37	34377	0.143261257	15154	4.299361E-02
Usp38	34378	0.027562577	6953	6.984423E-01
Usp39	34379	0.215549118	18779	2.174279E-03
Usp4	34380	0.125329666	13992	7.701104E-02
Usp40	34381	0.010625542	5799	8.812931E-01
Usp42	34382	-0.002483279	5050	9.721604E-01
Usp43	34383	-0.069982191	950	3.247705E-01
Usp44	34384	-0.054451513	1703	4.437940E-01
Usp45	34385	0.131966516	14404	6.249810E-02
Usp46	34386	0.136398453	14722	5.411874E-02
Usp47	34387	0.043792131	7850	5.380742E-01
Usp48	34388	0.015695002	6110	8.254170E-01
Usp49	34389	0.100692421	12102	1.559899E-01
Usp5	34390	0.089994991	11342	2.050419E-01
Usp50	34391	0.20400102	18390	3.761986E-03
Usp51	34392		#N/A	1.000000E+00
Usp53	34393	0.166122877	16715	1.872476E-02
Usp54	34394	0.019502727	6302	7.840020E-01
Usp6nl	34395	0.3061068	19926	1.042575E-05
Usp7	34396	0.079864248	10582	2.609417E-01
Usp8	34397	-0.0280208	3997	6.936789E-01
Usp9x	34398	0.205540268	18438	3.502578E-03
Usp9y	34399	0.099271295	12017.5	1.619434E-01

Spearman Rank correlation analysis performed between Prdm1 and all-expressed genes within the Meredith RNA-seq dataset. Robust Prdm1–associated genes were identified using a cut-off of $p < 0.0005$.

Table S1, Related to Supplemental Figure 3C. Prdm1 associated genes

Uspl1	34400	0.064738309	9523	3.624244E-01
Ust	34401	0.183476901	17569	9.305930E-03
Utf1	34402	0.077813641	10461	2.734225E-01
Utp11l	34403	0.124810616	13955	7.825244E-02
Utp14a	34404	0.003944347	5514	9.557943E-01
Utp14b	34405		#N/A	1.000000E+00
Utp15	34406	0.07965863	10572	2.621752E-01
Utp18	34407	0.107090666	12501	1.312115E-01
Utp20	34408	0.267200813	19700	1.308341E-04
Utp23	34409	0.012649747	5910	8.588956E-01
Utp3	34410	0.18351267	17572	9.291950E-03
Utp6	34411	0.055595424	8961	4.342646E-01
Utrn	34412	0.093629945	11624	1.872606E-01
Uts2	34413		#N/A	1.000000E+00
Uts2b	34414	-0.128738567	70	6.924866E-02
Uts2r	34415	0.014759448	6059.5	8.356716E-01
Uty	34416	0.203053835	18360	3.930103E-03
Uvrag	34417	0.109842229	12704	1.215367E-01
Uvssa	34418	0.166784514	16762	1.825217E-02
Uxs1	34419	0.116142821	13404	1.014719E-01
Uxt	34420	0.005157238	5572	9.422218E-01
V1ra8	34421		#N/A	1.000000E+00
V1rd19	34422		#N/A	1.000000E+00
Vac14	34423	0.048339111	8163	4.966749E-01
Vamp1	34424	0.062786773	9378	3.771038E-01
Vamp2	34425	0.021968321	6589	7.574973E-01
Vamp3	34426		#N/A	1.000000E+00
Vamp4	34427	0.115693737	13374	1.028094E-01
Vamp5	34428	0.309219452	19936	8.377923E-06
Vamp7	34429		#N/A	1.000000E+00
Vamp7-ps	34430		#N/A	1.000000E+00
Vamp8	34431	0.125918844	14036	7.562126E-02
Vangl1	34432	0.109063641	12627	1.242163E-01
Vangl2	34433	0.043598039	7839	5.398788E-01
Vapa	34434	0.113751812	13241	1.087537E-01
Vapb	34435	-0.017947184	4371	8.008548E-01
Vars	34436	0.119382138	13616	9.222751E-02
Vars2	34437	0.057753131	9071	4.166129E-01
Vash1	34438	0.053267784	8685	4.537786E-01
Vash2	34439	-0.06355058	1354	3.713157E-01
Vasn	34440		#N/A	1.000000E+00
Vasp	34441	0.216686976	18810	2.056779E-03
Vat1	34442	0.077419089	10436	2.758696E-01

Spearman Rank correlation analysis performed between Prdm1 and all-expressed genes within the Meredith RNA-seq dataset. Robust Prdm1–associated genes were identified using a cut-off of $p < 0.0005$.

Table S1, Related to Supplemental Figure 3C. Prdm1 associated genes

Vat1l	34443	0.091030718	11440	1.998557E-01
Vaultrc5	34444		#N/A	1.000000E+00
Vav1	34445	0.044049353	7869	5.356872E-01
Vav2	34446	0.179677541	17390	1.090120E-02
Vav3	34447	0.245251054	19442	4.648328E-04
Vax1	34448		#N/A	1.000000E+00
Vax2	34449	-0.054450825	1984	4.437997E-01
Vax2os	34450	-0.054450825	1984	4.437997E-01
Vax2os1	34451		#N/A	1.000000E+00
Vbp1	34452	0.101619906	12159	1.521943E-01
Vcam1	34453	0.140682718	14999	4.692502E-02
Vcan	34454	0.116145126	13405	1.014651E-01
Vcl	34455	0.112115296	12861	1.139698E-01
Vcp	34456	0.106093429	12433	1.348606E-01
Vcp-rs	34457	-0.086750697	472.5	2.219141E-01
Vcpip1	34458	0.215075887	18742	2.224921E-03
Vdac1	34459	0.156105678	16044	2.728750E-02
Vdac2	34460	0.210865404	18607	2.724998E-03
Vdac3	34461	0.064150387	9482	3.668089E-01
Vdac3-ps1	34462	0.101615529	12157	1.522120E-01
Vdr	34463	-0.027662926	4010	6.973981E-01
Vegfa	34464	0.022006511	6591	7.570889E-01
Vegfb	34465	0.127174127	14126	7.272797E-02
Vegfc	34466	0.04829958	8160	4.970274E-01
Veph1	34467	0.286257857	19844	3.969537E-05
Vezf1	34468	0.311186884	19941	7.287018E-06
Vezt	34469	0.033897258	7289	6.337098E-01
Vgf	34470	0.043308592	7825	5.425756E-01
Vgll1	34471	0.086412184	11087.5	2.237298E-01
Vgll2	34472	-0.054451513	1703	4.437940E-01
Vgll3	34473	0.189680829	17832	7.142114E-03
Vgll4	34474	-0.089751571	417	2.062748E-01
Vhl	34475	0.046772714	8064	5.107438E-01
Vhl-ps1	34476		#N/A	1.000000E+00
Vil1	34477	0.099265369	12014	1.619685E-01
Vill	34478	-0.038406171	3065	5.892384E-01
Vim	34479	0.06869976	9752	3.337370E-01
Vimp	34480	0.160278087	16293	2.338144E-02
Vip	34481	0.030375125	7082	6.693974E-01
Vipas39	34482	0.156813897	16098	2.658756E-02
Vipr1	34483	0.039036124	7580	5.831406E-01
Vipr2	34484	-0.055336718	1509.5	4.364094E-01
Vit	34485	-0.079880088	645	2.608468E-01

Spearman Rank correlation analysis performed between Prdm1 and all-expressed genes within the Meredith RNA-seq dataset. Robust Prdm1–associated genes were identified using a cut-off of $p < 0.0005$.

Table S1, Related to Supplemental Figure 3C. Prdm1 associated genes

Vkorc1	34486		#N/A	1.000000E+00
Vkorc1l1	34487	0.052523367	8503	4.601215E-01
Vldlr	34488	0.006043031	5600	9.323199E-01
Vma21	34489	0.142920215	15138	4.349718E-02
Vma21-ps	34490		#N/A	1.000000E+00
Vmac	34491	0.032394289	7198	6.488413E-01
Vmn1r-ps1	34492		#N/A	1.000000E+00
Vmn1r-ps10	34493		#N/A	1.000000E+00
Vmn1r-ps100	34494		#N/A	1.000000E+00
Vmn1r-ps102	34495		#N/A	1.000000E+00
Vmn1r-ps103	34496		#N/A	1.000000E+00
Vmn1r-ps104	34497		#N/A	1.000000E+00
Vmn1r-ps106	34498		#N/A	1.000000E+00
Vmn1r-ps107	34499		#N/A	1.000000E+00
Vmn1r-ps108	34500		#N/A	1.000000E+00
Vmn1r-ps109	34501		#N/A	1.000000E+00
Vmn1r-ps11	34502		#N/A	1.000000E+00
Vmn1r-ps110	34503		#N/A	1.000000E+00
Vmn1r-ps111	34504		#N/A	1.000000E+00
Vmn1r-ps112	34505		#N/A	1.000000E+00
Vmn1r-ps113	34506		#N/A	1.000000E+00
Vmn1r-ps114	34507		#N/A	1.000000E+00
Vmn1r-ps116	34508		#N/A	1.000000E+00
Vmn1r-ps12	34509		#N/A	1.000000E+00
Vmn1r-ps120	34510		#N/A	1.000000E+00
Vmn1r-ps123	34511		#N/A	1.000000E+00
Vmn1r-ps125	34512		#N/A	1.000000E+00
Vmn1r-ps126	34513		#N/A	1.000000E+00
Vmn1r-ps127	34514		#N/A	1.000000E+00
Vmn1r-ps128	34515		#N/A	1.000000E+00
Vmn1r-ps132	34516		#N/A	1.000000E+00
Vmn1r-ps135	34517	-0.038406171	3065	5.892384E-01
Vmn1r-ps14	34518		#N/A	1.000000E+00
Vmn1r-ps141	34519		#N/A	1.000000E+00
Vmn1r-ps142	34520		#N/A	1.000000E+00
Vmn1r-ps143	34521		#N/A	1.000000E+00
Vmn1r-ps144	34522		#N/A	1.000000E+00
Vmn1r-ps145	34523		#N/A	1.000000E+00
Vmn1r-ps146	34524		#N/A	1.000000E+00
Vmn1r-ps147	34525		#N/A	1.000000E+00
Vmn1r-ps148	34526		#N/A	1.000000E+00
Vmn1r-ps149	34527		#N/A	1.000000E+00
Vmn1r-ps15	34528		#N/A	1.000000E+00

Spearman Rank correlation analysis performed between Prdm1 and all-expressed genes within the Meredith RNA-seq dataset. Robust Prdm1–associated genes were identified using a cut-off of $p < 0.0005$.

Vmn1r-ps150	34529	#N/A	1.000000E+00
Vmn1r-ps151	34530	#N/A	1.000000E+00
Vmn1r-ps152	34531	#N/A	1.000000E+00
Vmn1r-ps17	34532	#N/A	1.000000E+00
Vmn1r-ps18	34533	#N/A	1.000000E+00
Vmn1r-ps19	34534	#N/A	1.000000E+00
Vmn1r-ps2	34535	#N/A	1.000000E+00
Vmn1r-ps20	34536	#N/A	1.000000E+00
Vmn1r-ps21	34537	#N/A	1.000000E+00
Vmn1r-ps22	34538	#N/A	1.000000E+00
Vmn1r-ps23	34539	#N/A	1.000000E+00
Vmn1r-ps24	34540	#N/A	1.000000E+00
Vmn1r-ps25	34541	#N/A	1.000000E+00
Vmn1r-ps26	34542	#N/A	1.000000E+00
Vmn1r-ps27	34543	#N/A	1.000000E+00
Vmn1r-ps28	34544	#N/A	1.000000E+00
Vmn1r-ps29	34545	#N/A	1.000000E+00
Vmn1r-ps31	34546	#N/A	1.000000E+00
Vmn1r-ps32	34547	#N/A	1.000000E+00
Vmn1r-ps34	34548	#N/A	1.000000E+00
Vmn1r-ps40	34549	#N/A	1.000000E+00
Vmn1r-ps45	34550	#N/A	1.000000E+00
Vmn1r-ps46	34551	#N/A	1.000000E+00
Vmn1r-ps47	34552	#N/A	1.000000E+00
Vmn1r-ps48	34553	#N/A	1.000000E+00
Vmn1r-ps49	34554	#N/A	1.000000E+00
Vmn1r-ps50	34555	#N/A	1.000000E+00
Vmn1r-ps51	34556	#N/A	1.000000E+00
Vmn1r-ps52	34557	#N/A	1.000000E+00
Vmn1r-ps53	34558	#N/A	1.000000E+00
Vmn1r-ps54	34559	#N/A	1.000000E+00
Vmn1r-ps55	34560	#N/A	1.000000E+00
Vmn1r-ps56	34561	#N/A	1.000000E+00
Vmn1r-ps57	34562	#N/A	1.000000E+00
Vmn1r-ps58	34563	#N/A	1.000000E+00
Vmn1r-ps59	34564	#N/A	1.000000E+00
Vmn1r-ps60	34565	#N/A	1.000000E+00
Vmn1r-ps61	34566	#N/A	1.000000E+00
Vmn1r-ps62	34567	#N/A	1.000000E+00
Vmn1r-ps63	34568	#N/A	1.000000E+00
Vmn1r-ps64	34569	#N/A	1.000000E+00
Vmn1r-ps65	34570	#N/A	1.000000E+00
Vmn1r-ps66	34571	#N/A	1.000000E+00

Spearman Rank correlation analysis performed between Prdm1 and all-expressed genes within the Meredith RNA-seq dataset. Robust Prdm1–associated genes were identified using a cut-off of $p < 0.0005$.

Table S1, Related to Supplemental Figure 3C. Prdm1 associated genes

Vmn1r-ps67	34572		#N/A	1.000000E+00
Vmn1r-ps68	34573		#N/A	1.000000E+00
Vmn1r-ps69	34574		#N/A	1.000000E+00
Vmn1r-ps70	34575	0.182294195	17509.5	9.778799E-03
Vmn1r-ps71	34576		#N/A	1.000000E+00
Vmn1r-ps73	34577		#N/A	1.000000E+00
Vmn1r-ps74	34578		#N/A	1.000000E+00
Vmn1r-ps75	34579		#N/A	1.000000E+00
Vmn1r-ps76	34580		#N/A	1.000000E+00
Vmn1r-ps77	34581		#N/A	1.000000E+00
Vmn1r-ps78	34582		#N/A	1.000000E+00
Vmn1r-ps79	34583		#N/A	1.000000E+00
Vmn1r-ps8	34584		#N/A	1.000000E+00
Vmn1r-ps80	34585		#N/A	1.000000E+00
Vmn1r-ps83	34586		#N/A	1.000000E+00
Vmn1r-ps84	34587		#N/A	1.000000E+00
Vmn1r-ps85	34588		#N/A	1.000000E+00
Vmn1r-ps86	34589		#N/A	1.000000E+00
Vmn1r-ps9	34590		#N/A	1.000000E+00
Vmn1r-ps92	34591		#N/A	1.000000E+00
Vmn1r-ps93	34592		#N/A	1.000000E+00
Vmn1r-ps94	34593		#N/A	1.000000E+00
Vmn1r-ps95	34594		#N/A	1.000000E+00
Vmn1r-ps97	34595		#N/A	1.000000E+00
Vmn1r-ps99	34596		#N/A	1.000000E+00
Vmn1r1	34597		#N/A	1.000000E+00
Vmn1r10	34598		#N/A	1.000000E+00
Vmn1r100	34599		#N/A	1.000000E+00
Vmn1r101	34600		#N/A	1.000000E+00
Vmn1r103	34601		#N/A	1.000000E+00
Vmn1r104	34602		#N/A	1.000000E+00
Vmn1r107	34603		#N/A	1.000000E+00
Vmn1r11	34604		#N/A	1.000000E+00
Vmn1r111	34605		#N/A	1.000000E+00
Vmn1r112	34606		#N/A	1.000000E+00
Vmn1r113	34607		#N/A	1.000000E+00
Vmn1r114	34608		#N/A	1.000000E+00
Vmn1r115	34609		#N/A	1.000000E+00
Vmn1r116	34610		#N/A	1.000000E+00
Vmn1r117	34611		#N/A	1.000000E+00
Vmn1r118	34612		#N/A	1.000000E+00
Vmn1r119	34613		#N/A	1.000000E+00
Vmn1r12	34614		#N/A	1.000000E+00

Spearman Rank correlation analysis performed between Prdm1 and all-expressed genes within the Meredith RNA-seq dataset. Robust Prdm1–associated genes were identified using a cut-off of $p < 0.0005$.

Table S1, Related to Supplemental Figure 3C. Prdm1 associated genes

Vmn1r120	34615		#N/A	1.000000E+00
Vmn1r121	34616		#N/A	1.000000E+00
Vmn1r122	34617		#N/A	1.000000E+00
Vmn1r123	34618		#N/A	1.000000E+00
Vmn1r124	34619		#N/A	1.000000E+00
Vmn1r125	34620		#N/A	1.000000E+00
Vmn1r126	34621		#N/A	1.000000E+00
Vmn1r127	34622		#N/A	1.000000E+00
Vmn1r128	34623		#N/A	1.000000E+00
Vmn1r129	34624		#N/A	1.000000E+00
Vmn1r13	34625		#N/A	1.000000E+00
Vmn1r130	34626		#N/A	1.000000E+00
Vmn1r132	34627		#N/A	1.000000E+00
Vmn1r135	34628		#N/A	1.000000E+00
Vmn1r137	34629		#N/A	1.000000E+00
Vmn1r139	34630		#N/A	1.000000E+00
Vmn1r14	34631		#N/A	1.000000E+00
Vmn1r142	34632		#N/A	1.000000E+00
Vmn1r143	34633		#N/A	1.000000E+00
Vmn1r148	34634		#N/A	1.000000E+00
Vmn1r149	34635		#N/A	1.000000E+00
Vmn1r15	34636		#N/A	1.000000E+00
Vmn1r151	34637		#N/A	1.000000E+00
Vmn1r152	34638		#N/A	1.000000E+00
Vmn1r155	34639		#N/A	1.000000E+00
Vmn1r157	34640		#N/A	1.000000E+00
Vmn1r158	34641		#N/A	1.000000E+00
Vmn1r159	34642		#N/A	1.000000E+00
Vmn1r16	34643		#N/A	1.000000E+00
Vmn1r160	34644		#N/A	1.000000E+00
Vmn1r163	34645		#N/A	1.000000E+00
Vmn1r165	34646		#N/A	1.000000E+00
Vmn1r167	34647	0.160304018	16323	2.335876E-02
Vmn1r168	34648		#N/A	1.000000E+00
Vmn1r169	34649		#N/A	1.000000E+00
Vmn1r17	34650	-0.038406171	3065	5.892384E-01
Vmn1r170	34651		#N/A	1.000000E+00
Vmn1r171	34652	0.21549506	18774.5	2.180011E-03
Vmn1r172	34653		#N/A	1.000000E+00
Vmn1r173	34654		#N/A	1.000000E+00
Vmn1r174	34655		#N/A	1.000000E+00
Vmn1r175	34656		#N/A	1.000000E+00
Vmn1r176	34657		#N/A	1.000000E+00

Spearman Rank correlation analysis performed between Prdm1 and all-expressed genes within the Meredith RNA-seq dataset. Robust Prdm1–associated genes were identified using a cut-off of $p < 0.0005$.

Table S1, Related to Supplemental Figure 3C. Prdm1 associated genes

Vmn1r177	34658		#N/A	1.000000E+00
Vmn1r178	34659		#N/A	1.000000E+00
Vmn1r179	34660		#N/A	1.000000E+00
Vmn1r18	34661		#N/A	1.000000E+00
Vmn1r180	34662		#N/A	1.000000E+00
Vmn1r181	34663		#N/A	1.000000E+00
Vmn1r183	34664		#N/A	1.000000E+00
Vmn1r184	34665		#N/A	1.000000E+00
Vmn1r185	34666		#N/A	1.000000E+00
Vmn1r188	34667		#N/A	1.000000E+00
Vmn1r189	34668		#N/A	1.000000E+00
Vmn1r19	34669		#N/A	1.000000E+00
Vmn1r190-ps	34670		#N/A	1.000000E+00
Vmn1r191	34671		#N/A	1.000000E+00
Vmn1r192	34672		#N/A	1.000000E+00
Vmn1r193	34673		#N/A	1.000000E+00
Vmn1r194	34674		#N/A	1.000000E+00
Vmn1r195	34675		#N/A	1.000000E+00
Vmn1r196	34676		#N/A	1.000000E+00
Vmn1r197	34677		#N/A	1.000000E+00
Vmn1r198	34678		#N/A	1.000000E+00
Vmn1r199	34679		#N/A	1.000000E+00
Vmn1r2	34680		#N/A	1.000000E+00
Vmn1r20	34681		#N/A	1.000000E+00
Vmn1r200	34682		#N/A	1.000000E+00
Vmn1r201	34683		#N/A	1.000000E+00
Vmn1r202	34684		#N/A	1.000000E+00
Vmn1r203	34685		#N/A	1.000000E+00
Vmn1r204	34686		#N/A	1.000000E+00
Vmn1r205	34687		#N/A	1.000000E+00
Vmn1r206	34688		#N/A	1.000000E+00
Vmn1r207-ps	34689		#N/A	1.000000E+00
Vmn1r208	34690		#N/A	1.000000E+00
Vmn1r209	34691		#N/A	1.000000E+00
Vmn1r21	34692	0.113548679	13094	1.093908E-01
Vmn1r210	34693		#N/A	1.000000E+00
Vmn1r211	34694		#N/A	1.000000E+00
Vmn1r212	34695		#N/A	1.000000E+00
Vmn1r213	34696	-0.077397959	691.5	2.760011E-01
Vmn1r214	34697		#N/A	1.000000E+00
Vmn1r215	34698		#N/A	1.000000E+00
Vmn1r216	34699		#N/A	1.000000E+00
Vmn1r217	34700		#N/A	1.000000E+00

Spearman Rank correlation analysis performed between Prdm1 and all-expressed genes within the Meredith RNA-seq dataset. Robust Prdm1–associated genes were identified using a cut-off of $p < 0.0005$.

Table S1, Related to Supplemental Figure 3C. Prdm1 associated genes

Vmn1r218	34701		#N/A	1.000000E+00
Vmn1r219	34702		#N/A	1.000000E+00
Vmn1r22	34703		#N/A	1.000000E+00
Vmn1r220	34704		#N/A	1.000000E+00
Vmn1r221	34705		#N/A	1.000000E+00
Vmn1r222	34706		#N/A	1.000000E+00
Vmn1r223	34707		#N/A	1.000000E+00
Vmn1r224	34708		#N/A	1.000000E+00
Vmn1r225	34709		#N/A	1.000000E+00
Vmn1r226	34710		#N/A	1.000000E+00
Vmn1r227	34711		#N/A	1.000000E+00
Vmn1r228	34712	-0.066856562	1139	3.468991E-01
Vmn1r229	34713		#N/A	1.000000E+00
Vmn1r23	34714		#N/A	1.000000E+00
Vmn1r230	34715		#N/A	1.000000E+00
Vmn1r231	34716		#N/A	1.000000E+00
Vmn1r232	34717		#N/A	1.000000E+00
Vmn1r233	34718		#N/A	1.000000E+00
Vmn1r234	34719		#N/A	1.000000E+00
Vmn1r235	34720		#N/A	1.000000E+00
Vmn1r236	34721		#N/A	1.000000E+00
Vmn1r237	34722		#N/A	1.000000E+00
Vmn1r238	34723		#N/A	1.000000E+00
Vmn1r239-ps	34724		#N/A	1.000000E+00
Vmn1r24	34725		#N/A	1.000000E+00
Vmn1r25	34726		#N/A	1.000000E+00
Vmn1r26	34727		#N/A	1.000000E+00
Vmn1r27	34728		#N/A	1.000000E+00
Vmn1r28	34729		#N/A	1.000000E+00
Vmn1r29	34730		#N/A	1.000000E+00
Vmn1r3	34731		#N/A	1.000000E+00
Vmn1r30	34732		#N/A	1.000000E+00
Vmn1r31	34733		#N/A	1.000000E+00
Vmn1r32	34734	-0.054450825	1984	4.437997E-01
Vmn1r33	34735	-0.038406171	3065	5.892384E-01
Vmn1r34	34736		#N/A	1.000000E+00
Vmn1r35	34737		#N/A	1.000000E+00
Vmn1r36	34738		#N/A	1.000000E+00
Vmn1r37	34739		#N/A	1.000000E+00
Vmn1r38	34740		#N/A	1.000000E+00
Vmn1r39	34741		#N/A	1.000000E+00
Vmn1r4	34742	0.000985463	5360	9.889503E-01
Vmn1r40	34743		#N/A	1.000000E+00

Spearman Rank correlation analysis performed between Prdm1 and all-expressed genes within the Meredith RNA-seq dataset. Robust Prdm1–associated genes were identified using a cut-off of $p < 0.0005$.

Table S1, Related to Supplemental Figure 3C. Prdm1 associated genes

Vmn1r41	34744	-0.054451513	1703	4.437940E-01
Vmn1r42	34745		#N/A	1.000000E+00
Vmn1r43	34746		#N/A	1.000000E+00
Vmn1r44	34747		#N/A	1.000000E+00
Vmn1r45	34748		#N/A	1.000000E+00
Vmn1r46	34749		#N/A	1.000000E+00
Vmn1r47	34750		#N/A	1.000000E+00
Vmn1r48	34751		#N/A	1.000000E+00
Vmn1r49	34752		#N/A	1.000000E+00
Vmn1r5	34753		#N/A	1.000000E+00
Vmn1r50	34754		#N/A	1.000000E+00
Vmn1r51	34755	-0.066856562	1139	3.468991E-01
Vmn1r52	34756		#N/A	1.000000E+00
Vmn1r53	34757		#N/A	1.000000E+00
Vmn1r54	34758		#N/A	1.000000E+00
Vmn1r55	34759		#N/A	1.000000E+00
Vmn1r56	34760		#N/A	1.000000E+00
Vmn1r57	34761		#N/A	1.000000E+00
Vmn1r58	34762	0.009789796	5763	8.905691E-01
Vmn1r59	34763		#N/A	1.000000E+00
Vmn1r6	34764		#N/A	1.000000E+00
Vmn1r60	34765		#N/A	1.000000E+00
Vmn1r61	34766		#N/A	1.000000E+00
VMN1R62	34767		#N/A	1.000000E+00
Vmn1r62	34768		#N/A	1.000000E+00
VMN1R63	34769		#N/A	1.000000E+00
Vmn1r63	34770	-0.054450825	1984	4.437997E-01
Vmn1r64	34771		#N/A	1.000000E+00
Vmn1r65	34772	0.030465235	7095	6.684747E-01
Vmn1r66	34773		#N/A	1.000000E+00
Vmn1r67	34774		#N/A	1.000000E+00
Vmn1r68	34775		#N/A	1.000000E+00
Vmn1r69	34776	-0.018105422	4347	7.991361E-01
Vmn1r7	34777		#N/A	1.000000E+00
Vmn1r70	34778		#N/A	1.000000E+00
Vmn1r71	34779		#N/A	1.000000E+00
Vmn1r72	34780		#N/A	1.000000E+00
Vmn1r73	34781		#N/A	1.000000E+00
Vmn1r74	34782		#N/A	1.000000E+00
Vmn1r75	34783		#N/A	1.000000E+00
Vmn1r76	34784		#N/A	1.000000E+00
Vmn1r77	34785	-0.038406171	3065	5.892384E-01
Vmn1r78	34786		#N/A	1.000000E+00

Spearman Rank correlation analysis performed between Prdm1 and all-expressed genes within the Meredith RNA-seq dataset. Robust Prdm1–associated genes were identified using a cut-off of $p < 0.0005$.

Table S1, Related to Supplemental Figure 3C. Prdm1 associated genes

Vmn1r79	34787		#N/A	1.000000E+00
Vmn1r8	34788		#N/A	1.000000E+00
Vmn1r80	34789		#N/A	1.000000E+00
Vmn1r81	34790		#N/A	1.000000E+00
Vmn1r82	34791		#N/A	1.000000E+00
Vmn1r83	34792		#N/A	1.000000E+00
Vmn1r84	34793		#N/A	1.000000E+00
Vmn1r85	34794		#N/A	1.000000E+00
Vmn1r86	34795		#N/A	1.000000E+00
Vmn1r87	34796		#N/A	1.000000E+00
Vmn1r88	34797		#N/A	1.000000E+00
Vmn1r89	34798		#N/A	1.000000E+00
Vmn1r9	34799	0.113548679	13094	1.093908E-01
Vmn1r90	34800		#N/A	1.000000E+00
Vmn1r91	34801		#N/A	1.000000E+00
Vmn1r93	34802		#N/A	1.000000E+00
Vmn1r94	34803		#N/A	1.000000E+00
Vmn1r95	34804		#N/A	1.000000E+00
Vmn2r-ps10	34805		#N/A	1.000000E+00
Vmn2r-ps104	34806		#N/A	1.000000E+00
Vmn2r-ps11	34807		#N/A	1.000000E+00
Vmn2r-ps110	34808		#N/A	1.000000E+00
Vmn2r-ps111	34809		#N/A	1.000000E+00
Vmn2r-ps114	34810		#N/A	1.000000E+00
Vmn2r-ps115	34811		#N/A	1.000000E+00
Vmn2r-ps116	34812		#N/A	1.000000E+00
Vmn2r-ps117	34813		#N/A	1.000000E+00
Vmn2r-ps118	34814		#N/A	1.000000E+00
Vmn2r-ps119	34815	-0.038406171	3065	5.892384E-01
Vmn2r-ps12	34816		#N/A	1.000000E+00
Vmn2r-ps120	34817		#N/A	1.000000E+00
Vmn2r-ps121	34818		#N/A	1.000000E+00
Vmn2r-ps122	34819		#N/A	1.000000E+00
Vmn2r-ps123	34820	-0.022315432	4165	7.537878E-01
Vmn2r-ps124	34821		#N/A	1.000000E+00
Vmn2r-ps125	34822		#N/A	1.000000E+00
Vmn2r-ps126	34823	-0.018447724	4312.5	7.954215E-01
Vmn2r-ps127	34824		#N/A	1.000000E+00
Vmn2r-ps128	34825		#N/A	1.000000E+00
Vmn2r-ps129	34826	0.031596895	7164	6.569286E-01
Vmn2r-ps13	34827		#N/A	1.000000E+00
Vmn2r-ps130	34828		#N/A	1.000000E+00
Vmn2r-ps131	34829		#N/A	1.000000E+00

Spearman Rank correlation analysis performed between Prdm1 and all-expressed genes within the Meredith RNA-seq dataset. Robust Prdm1–associated genes were identified using a cut-off of $p < 0.0005$.

Table S1, Related to Supplemental Figure 3C. Prdm1 associated genes

Vmn2r-ps132	34830		#N/A	1.000000E+00
Vmn2r-ps133	34831	-0.066856562	1139	3.468991E-01
Vmn2r-ps134	34832		#N/A	1.000000E+00
Vmn2r-ps15	34833		#N/A	1.000000E+00
Vmn2r-ps159	34834	-0.054450825	1984	4.437997E-01
Vmn2r-ps16	34835		#N/A	1.000000E+00
Vmn2r-ps17	34836		#N/A	1.000000E+00
Vmn2r-ps18	34837		#N/A	1.000000E+00
Vmn2r-ps19	34838		#N/A	1.000000E+00
Vmn2r-ps2	34839		#N/A	1.000000E+00
Vmn2r-ps20	34840		#N/A	1.000000E+00
Vmn2r-ps21	34841		#N/A	1.000000E+00
Vmn2r-ps23	34842		#N/A	1.000000E+00
Vmn2r-ps3	34843		#N/A	1.000000E+00
Vmn2r-ps31	34844		#N/A	1.000000E+00
Vmn2r-ps32	34845		#N/A	1.000000E+00
Vmn2r-ps33	34846		#N/A	1.000000E+00
Vmn2r-ps34	34847		#N/A	1.000000E+00
Vmn2r-ps36	34848	-0.054451513	1703	4.437940E-01
Vmn2r-ps37	34849		#N/A	1.000000E+00
Vmn2r-ps38	34850		#N/A	1.000000E+00
Vmn2r-ps4	34851		#N/A	1.000000E+00
Vmn2r-ps40	34852		#N/A	1.000000E+00
Vmn2r-ps41	34853		#N/A	1.000000E+00
Vmn2r-ps42	34854		#N/A	1.000000E+00
Vmn2r-ps43	34855		#N/A	1.000000E+00
Vmn2r-ps44	34856		#N/A	1.000000E+00
Vmn2r-ps45	34857		#N/A	1.000000E+00
Vmn2r-ps46	34858		#N/A	1.000000E+00
Vmn2r-ps47	34859		#N/A	1.000000E+00
Vmn2r-ps49	34860		#N/A	1.000000E+00
Vmn2r-ps5	34861		#N/A	1.000000E+00
Vmn2r-ps51	34862		#N/A	1.000000E+00
Vmn2r-ps52	34863		#N/A	1.000000E+00
Vmn2r-ps53	34864	-0.038406171	3065	5.892384E-01
Vmn2r-ps54	34865	0.10850609	12583	1.261632E-01
Vmn2r-ps55	34866		#N/A	1.000000E+00
Vmn2r-ps56	34867		#N/A	1.000000E+00
Vmn2r-ps57	34868	-0.054450825	1984	4.437997E-01
Vmn2r-ps58	34869		#N/A	1.000000E+00
Vmn2r-ps59	34870		#N/A	1.000000E+00
Vmn2r-ps6	34871		#N/A	1.000000E+00
Vmn2r-ps60	34872		#N/A	1.000000E+00

Spearman Rank correlation analysis performed between Prdm1 and all-expressed genes within the Meredith RNA-seq dataset. Robust Prdm1–associated genes were identified using a cut-off of $p < 0.0005$.

Table S1, Related to Supplemental Figure 3C. Prdm1 associated genes

Vmn2r-ps62	34873		#N/A	1.000000E+00
Vmn2r-ps64	34874		#N/A	1.000000E+00
Vmn2r-ps65	34875		#N/A	1.000000E+00
Vmn2r-ps66	34876		#N/A	1.000000E+00
Vmn2r-ps67	34877		#N/A	1.000000E+00
Vmn2r-ps68	34878		#N/A	1.000000E+00
Vmn2r-ps69	34879	-0.038406171	3065	5.892384E-01
Vmn2r-ps7	34880		#N/A	1.000000E+00
Vmn2r-ps70	34881		#N/A	1.000000E+00
Vmn2r-ps71	34882		#N/A	1.000000E+00
Vmn2r-ps72	34883		#N/A	1.000000E+00
Vmn2r-ps73	34884		#N/A	1.000000E+00
Vmn2r-ps74	34885		#N/A	1.000000E+00
Vmn2r-ps75	34886		#N/A	1.000000E+00
Vmn2r-ps76	34887		#N/A	1.000000E+00
Vmn2r-ps77	34888		#N/A	1.000000E+00
Vmn2r-ps78	34889		#N/A	1.000000E+00
Vmn2r-ps79	34890		#N/A	1.000000E+00
Vmn2r-ps8	34891		#N/A	1.000000E+00
Vmn2r-ps80	34892		#N/A	1.000000E+00
Vmn2r-ps81	34893		#N/A	1.000000E+00
Vmn2r-ps82	34894	-0.038406171	3065	5.892384E-01
Vmn2r-ps83	34895		#N/A	1.000000E+00
Vmn2r-ps84	34896		#N/A	1.000000E+00
Vmn2r-ps85	34897		#N/A	1.000000E+00
Vmn2r-ps86	34898		#N/A	1.000000E+00
Vmn2r-ps87	34899		#N/A	1.000000E+00
Vmn2r-ps9	34900		#N/A	1.000000E+00
Vmn2r-ps94	34901		#N/A	1.000000E+00
Vmn2r1	34902		#N/A	1.000000E+00
Vmn2r10	34903	-0.038406171	3065	5.892384E-01
Vmn2r100	34904		#N/A	1.000000E+00
Vmn2r101	34905	0.046025613	8011	5.175260E-01
Vmn2r102	34906	0.113548679	13094	1.093908E-01
Vmn2r103	34907	0.160304018	16323	2.335876E-02
Vmn2r104	34908		#N/A	1.000000E+00
Vmn2r105	34909		#N/A	1.000000E+00
Vmn2r106	34910	-0.038406171	3065	5.892384E-01
Vmn2r107	34911	-0.045728801	2298	5.202333E-01
Vmn2r108	34912	0.160985049	16395	2.276975E-02
Vmn2r109	34913	-0.038406171	3065	5.892384E-01
Vmn2r11	34914	0.1544596	15936.5	2.897553E-02
Vmn2r110	34915	-0.038406171	3065	5.892384E-01

Spearman Rank correlation analysis performed between Prdm1 and all-expressed genes within the Meredith RNA-seq dataset. Robust Prdm1–associated genes were identified using a cut-off of $p < 0.0005$.

Table S1, Related to Supplemental Figure 3C. Prdm1 associated genes

Vmn2r111	34916	0.182294195	17509.5	9.778799E-03
Vmn2r112	34917	0.143605682	15224	4.249002E-02
Vmn2r113	34918	0.113548679	13094	1.093908E-01
Vmn2r114	34919	-0.038406171	3065	5.892384E-01
Vmn2r115	34920	0.188804702	17792.5	7.417613E-03
Vmn2r116	34921		#N/A	1.000000E+00
Vmn2r117	34922	0.113548679	13094	1.093908E-01
Vmn2r118	34923	-0.042549604	2420	5.496788E-01
Vmn2r12	34924	0.095574113	11741	1.782271E-01
Vmn2r120	34925	-0.110581467	154.5	1.190342E-01
Vmn2r121	34926		#N/A	1.000000E+00
Vmn2r122	34927		#N/A	1.000000E+00
Vmn2r124	34928	-0.038406171	3065	5.892384E-01
Vmn2r13	34929		#N/A	1.000000E+00
Vmn2r14	34930	-0.038406171	3065	5.892384E-01
Vmn2r15	34931	0.053267784	8685	4.537786E-01
Vmn2r16	34932	-0.038406171	3065	5.892384E-01
Vmn2r17	34933	-0.005546546	4898	9.378687E-01
Vmn2r18	34934	-0.038406171	3065	5.892384E-01
Vmn2r19	34935	-0.038406171	3065	5.892384E-01
Vmn2r2	34936		#N/A	1.000000E+00
Vmn2r20	34937		#N/A	1.000000E+00
Vmn2r21	34938		#N/A	1.000000E+00
Vmn2r22	34939	0.162808768	16526.5	2.125512E-02
Vmn2r23	34940		#N/A	1.000000E+00
Vmn2r24	34941	0.051920795	8465	4.652917E-01
Vmn2r25	34942	0.113548679	13094	1.093908E-01
Vmn2r26	34943	-0.001224019	5151	9.862757E-01
Vmn2r27	34944	0.076478035	10378.5	2.817660E-01
Vmn2r28	34945		#N/A	1.000000E+00
Vmn2r29	34946	0.038229357	7499.5	5.909551E-01
Vmn2r3	34947		#N/A	1.000000E+00
Vmn2r30	34948	-0.066858259	1029.5	3.468868E-01
Vmn2r31	34949		#N/A	1.000000E+00
Vmn2r32	34950		#N/A	1.000000E+00
Vmn2r33	34951		#N/A	1.000000E+00
Vmn2r34	34952		#N/A	1.000000E+00
Vmn2r35	34953	-0.038406171	3065	5.892384E-01
Vmn2r36	34954	-0.038406171	3065	5.892384E-01
Vmn2r37	34955		#N/A	1.000000E+00
Vmn2r38	34956	0.143605682	15224	4.249002E-02
Vmn2r39	34957		#N/A	1.000000E+00
Vmn2r4	34958	0.192970125	17950	6.187359E-03

Spearman Rank correlation analysis performed between Prdm1 and all-expressed genes within the Meredith RNA-seq dataset. Robust Prdm1–associated genes were identified using a cut-off of $p < 0.0005$.

Vmn2r40	34959		#N/A	1.000000E+00
Vmn2r41	34960	-0.038406171	3065	5.892384E-01
Vmn2r42	34961	-0.077394997	736.5	2.760195E-01
Vmn2r43	34962	-0.038406171	3065	5.892384E-01
Vmn2r44	34963	0.217279508	18828	1.997907E-03
Vmn2r45	34964		#N/A	1.000000E+00
Vmn2r46	34965	-0.038406171	3065	5.892384E-01
Vmn2r47	34966		#N/A	1.000000E+00
Vmn2r48	34967	0.113548679	13094	1.093908E-01
Vmn2r49	34968		#N/A	1.000000E+00
Vmn2r5	34969		#N/A	1.000000E+00
Vmn2r50	34970		#N/A	1.000000E+00
Vmn2r51	34971	0.113548679	13094	1.093908E-01
Vmn2r52	34972	-0.054451513	1703	4.437940E-01
Vmn2r53	34973		#N/A	1.000000E+00
Vmn2r54	34974	0.143605682	15224	4.249002E-02
Vmn2r55	34975	0.101346245	12139	1.533069E-01
Vmn2r56	34976	0.045056653	7941.5	5.263908E-01
Vmn2r57	34977		#N/A	1.000000E+00
Vmn2r58	34978	0.086412184	11087.5	2.237298E-01
Vmn2r59	34979	-0.038406171	3065	5.892384E-01
Vmn2r6	34980		#N/A	1.000000E+00
Vmn2r60	34981	0.097128619	11845	1.712390E-01
Vmn2r61	34982		#N/A	1.000000E+00
Vmn2r62	34983		#N/A	1.000000E+00
Vmn2r63	34984	0.021316405	6469	7.644793E-01
Vmn2r65	34985		#N/A	1.000000E+00
Vmn2r66	34986	0.117748257	13511	9.680269E-02
Vmn2r67	34987		#N/A	1.000000E+00
Vmn2r68	34988	-0.103159953	253.5	1.460465E-01
Vmn2r69	34989		#N/A	1.000000E+00
Vmn2r7	34990		#N/A	1.000000E+00
Vmn2r70	34991		#N/A	1.000000E+00
Vmn2r71	34992		#N/A	1.000000E+00
Vmn2r72	34993	0.143605682	15224	4.249002E-02
Vmn2r73	34994	-0.038406171	3065	5.892384E-01
Vmn2r74	34995	-0.038406171	3065	5.892384E-01
Vmn2r75	34996	-0.043687937	2382.5	5.390426E-01
Vmn2r76	34997		#N/A	1.000000E+00
Vmn2r77	34998		#N/A	1.000000E+00
Vmn2r78	34999		#N/A	1.000000E+00
Vmn2r79	35000	0.160987082	16412.5	2.276801E-02
Vmn2r8	35001	-0.038406171	3065	5.892384E-01

Table S1, Related to Supplemental Figure 3C. Prdm1 associated genes

Vmn2r80	35002	-0.032450477	3820	6.482730E-01
Vmn2r81	35003	-0.077397959	691.5	2.760011E-01
Vmn2r82	35004	0.166148435	16721.5	1.870631E-02
Vmn2r83	35005	-0.054450825	1984	4.437997E-01
Vmn2r84	35006	0.113548679	13094	1.093908E-01
Vmn2r85	35007		#N/A	1.000000E+00
Vmn2r86	35008	-0.054451513	1703	4.437940E-01
Vmn2r87	35009		#N/A	1.000000E+00
Vmn2r88	35010		#N/A	1.000000E+00
Vmn2r89	35011		#N/A	1.000000E+00
Vmn2r9	35012		#N/A	1.000000E+00
Vmn2r90	35013	-0.095280123	319	1.795721E-01
Vmn2r91	35014	-0.054451513	1703	4.437940E-01
Vmn2r92	35015		#N/A	1.000000E+00
Vmn2r93	35016	-0.066858259	1029.5	3.468868E-01
Vmn2r94	35017		#N/A	1.000000E+00
Vmn2r95	35018	0.14001476	14956.5	4.799102E-02
Vmn2r96	35019	-0.038406171	3065	5.892384E-01
Vmn2r97	35020	0.014301797	6015.5	8.406982E-01
Vmn2r98	35021	-0.066858259	1029.5	3.468868E-01
Vmn2r99	35022	-0.086748028	498.5	2.219283E-01
Vmo1	35023	0.05152521	8429	4.687032E-01
Vmp1	35024	0.067811872	9691	3.400369E-01
Vnn1	35025	0.052135744	8482	4.634437E-01
Vnn3	35026	0.071182474	9924	3.165206E-01
Vopp1	35027	0.164913353	16651	1.961618E-02
Vprbp	35028	0.083033952	10836	2.424291E-01
Vpreb1	35029	0.0815845	10706	2.507776E-01
Vpreb2	35030		#N/A	1.000000E+00
Vpreb3	35031	0.145570187	15383	3.971143E-02
Vps11	35032	0.163564093	16572	2.065378E-02
Vps13a	35033	0.073214106	10079	3.028705E-01
Vps13b	35034	0.225090053	19044	1.352856E-03
Vps13c	35035	0.063167222	9399	3.742139E-01
Vps13d	35036	0.160161987	16284	2.348324E-02
Vps16	35037	0.083731177	10897	2.384832E-01
Vps18	35038	0.009421081	5742	8.946663E-01
Vps25	35039	0.160791703	16379	2.293565E-02
Vps26a	35040	0.122001052	13785	8.525529E-02
Vps26b	35041	0.142136266	15091	4.467349E-02
Vps28	35042	0.08111587	10677	2.535189E-01
Vps29	35043	-0.014765972	4521	8.356000E-01
Vps33a	35044	-0.029402613	3952	6.793877E-01

Spearman Rank correlation analysis performed between Prdm1 and all-expressed genes within the Meredith RNA-seq dataset. Robust Prdm1–associated genes were identified using a cut-off of $p < 0.0005$.

Table S1, Related to Supplemental Figure 3C. Prdm1 associated genes

Vps33b	35045	0.058377407	9115	4.115853E-01
Vps35	35046	0.07217766	10007	3.097849E-01
Vps36	35047	-0.107913194	196	1.282593E-01
Vps37a	35048	0.16049795	16364	2.318971E-02
Vps37b	35049	-0.026084527	4065	7.138868E-01
Vps37c	35050	0.127645505	14164	7.166499E-02
Vps37d	35051	-0.054450825	1984	4.437997E-01
Vps39	35052	0.063567399	9440	3.711888E-01
Vps41	35053	0.190806509	17873	6.801540E-03
Vps45	35054	0.145637601	15386	3.961886E-02
Vps4a	35055	-0.114205289	137	1.073420E-01
Vps4b	35056	0.087746063	11181	2.166359E-01
Vps51	35057	-0.030348899	3918	6.696661E-01
Vps52	35058	0.037090191	7436	6.020699E-01
Vps53	35059	-0.049955863	2198	4.823717E-01
Vps54	35060	0.069950044	9841	3.249933E-01
Vps72	35061	0.180200788	17414	1.066807E-02
Vps8	35062	0.266631439	19695	1.353997E-04
Vps9d1	35063	0.021347836	6544	7.641422E-01
Vrk1	35064	0.028871415	7009	6.848684E-01
Vrk2	35065	0.121974113	13784	8.532479E-02
Vrk3	35066	0.150797125	15699	3.305376E-02
Vrtn	35067	0.075711049	10308	2.866340E-01
Vsig1	35068	0.143799711	15297	4.220852E-02
Vsig10	35069	0.042157974	7749	5.533616E-01
Vsig10l	35070	0.113548679	13094	1.093908E-01
Vsig2	35071	0.032618092	7213	6.465788E-01
Vsig4	35072	-0.038406171	3065	5.892384E-01
Vsig8	35073	-0.054450825	1984	4.437997E-01
Vsnl1	35074	0.07494696	10242	2.915394E-01
Vstm2a	35075	-0.016873604	4418	8.125405E-01
Vstm2b	35076	0.041609934	7717	5.585354E-01
Vstm2l	35077	0.113548679	13094	1.093908E-01
Vstm4	35078	-0.00126578	5128	9.858075E-01
Vstm5	35079	0.131962791	14403	6.250557E-02
Vsx1	35080	0.142209151	15094	4.456302E-02
Vsx2	35081	0.261545617	19643	1.833200E-04
Vta1	35082	0.08455677	10957	2.338691E-01
Vtcn1	35083		#N/A	1.000000E+00
Vti1a	35084	-0.096096744	309	1.758546E-01
Vti1b	35085	0.141347506	15037	4.588378E-02
Vtn	35086		#N/A	1.000000E+00
Vwa1	35087	0.005419453	5578	9.392896E-01

Spearman Rank correlation analysis performed between Prdm1 and all-expressed genes within the Meredith RNA-seq dataset. Robust Prdm1–associated genes were identified using a cut-off of $p < 0.0005$.

Table S1, Related to Supplemental Figure 3C. Prdm1 associated genes

Vwa2	35088	0.030502237	7098	6.680959E-01
Vwa3a	35089	-0.056165168	1479.5	4.295625E-01
Vwa3b	35090	0.042327955	7762	5.517617E-01
Vwa5a	35091	0.085483755	11021	2.287637E-01
Vwa5b1	35092	-0.086750697	472.5	2.219141E-01
Vwa5b2	35093	0.081085405	10671.5	2.536978E-01
Vwa7	35094	0.042434111	7768	5.507636E-01
Vwa8	35095	-0.018027218	4368	7.999854E-01
Vwa9	35096	0.027872623	6967	6.952179E-01
Vwc2	35097	0.021369849	6546	7.639062E-01
Vwc2l	35098	0.021734438	6568	7.600000E-01
Vwce	35099	-0.001224019	5151	9.862757E-01
Vwde	35100	0.027595803	6957	6.980965E-01
Vwf	35101	0.056248977	8997	4.288733E-01
Wac	35102	0.259094623	19618	2.116851E-04
Wap	35103		#N/A	1.000000E+00
Wapal	35104	0.102662616	12226	1.480109E-01
Wars	35105	0.233479415	19239	8.768929E-04
Wars2	35106	0.195922715	18081	5.429256E-03
[truncated: 55,792 more chars]
